# Safety and immunogenicity of an AS03-adjuvanted plant-based SARS-CoV-2 vaccine in Adults with and without Comorbidities

**DOI:** 10.1038/s41541-022-00561-2

**Published:** 2022-11-09

**Authors:** Nathalie Charland, Philipe Gobeil, Stéphane Pillet, Iohann Boulay, Annie Séguin, Alexander Makarkov, Gretchen Heizer, Kapil Bhutada, Asif Mahmood, Sonia Trépanier, Karen Hager, Julia Jiang-Wright, Judith Atkins, Pooja Saxena, Matthew P. Cheng, Donald C. Vinh, Philippe Boutet, François Roman, Robbert Van Der Most, Maria Angeles Ceregido, Marc Dionne, Guy Tellier, Jean-Sébastien Gauthier, Brandon Essink, Michael Libman, Jason Haffizulla, André Fréchette, Marc-André D’Aoust, Nathalie Landry, Brian J. Ward

**Affiliations:** 1 Medicago Inc., 1020 route de l’Église, Bureau 600, Québec, QC Canada; 2 Research Institute of the McGill University Health Centre, 1001 Decarie St, Montréal, QC Canada; 3 GSK, Wavre, Belgium; 4 CHU de Québec-Université Laval, 2400 d’Estimauville, Québec City, QC Canada; 5 Manna Research, 101-13714 Boul Du Curé-Labelle, Suite 101, Mirabel, QC Canada; 6 Q&T Research Sherbrooke Inc., 2185 King Street West, Suite 101, Sherbrooke, QC Canada; 7 Meridian Clinical Research, 3319 N 107th St, Omaha, NE USA; 8 Precision Clinical Research, 8399 West Oakland Park Blvd., Suite B & C, Sunrise, FL USA; 9 Diex Research Québec Inc., 205 rue Montmagny, Suite 103, Québec City, QC Canada; 10 Present Address: Vertex Pharmaceticals, 50 Northern Avenue, Boston, MA USA; 11 Present Address: BioNTech SE, An der Goldgrube 12, Mainz, Germany

**Keywords:** Protein vaccines, SARS-CoV-2, Viral infection, Recombinant vaccine, Adjuvants

## Abstract

The rapid spread of SARS-CoV-2 continues to impact humanity on a global scale with rising total morbidity and mortality. Despite the development of several effective vaccines, new products are needed to supply ongoing demand and to fight variants. We report herein a pre-specified interim analysis of the phase 2 portion of a Phase 2/3, randomized, placebo-controlled trial of a coronavirus virus-like particle (CoVLP) vaccine candidate, produced in plants that displays the SARS-CoV-2 spike glycoprotein, adjuvanted with AS03 (NCT04636697). A total of 753 participants were recruited between 25th November 2020 and 24th March 2021 into three groups: Healthy Adults (18–64 years: *N* = 306), Older Adults (≥65 years: *N* = 282) and Adults with Comorbidities (≥18 years: *N* = 165) and randomized 5:1 to receive two intramuscular doses of either vaccine (3.75 µg CoVLP/dose+AS03) or placebo, 21 days apart. This report presents safety, tolerability and immunogenicity data up to 6 months post-vaccination. The immune outcomes presented include neutralizing antibody (NAb) titres as measured by pseudovirion assay at days 21 and 42 as well as neutralizing antibody cross-reactivity to several variants of concern (VOCs): Alpha, Beta, Gamma, Delta, and Omicron (BA.1), up to 201 days post-immunization. Cellular (IFN-γ and IL-4 ELISpot) response data in day 21 and 42 peripheral blood are also presented. In this study, CoVLP+AS03 was well-tolerated and adverse events (AE) after each dose were generally mild to moderate and transient. Solicited AEs in Older Adults and Adults with Comorbidities were generally less frequent than in Healthy Adults and the reactogenicity was higher after the second dose. CoVLP+AS03 induced seroconversion in >35% of participants in each group after the first dose and in ~98% of participants, 21 days after the second dose. In all cohorts, 21-days after the second dose, NAb levels in sera against the vaccine strain were ~10-times those in a panel of convalescent sera. Cross-reactivity to Alpha, Beta and Delta variants was generally retained to day 201 (>80%) while cross-reactivity to the Gamma variant was reduced but still substantial at day 201 (73%). Cross-reactivity to the Omicron variant fell from 72% at day 42 to 20% at day 201. Almost all participants in all groups (>88%) had detectable cellular responses (IFN-γ, IL-4 or both) at 21 days after the second dose. A Th1-biased response was most evident after the first dose and was still present after the second dose. These data demonstrated that CoVLP+AS03 is well-tolerated and highly immunogenic, generating a durable (at least 6 months) immune response against different VOCs, in adults ≥18 years of age, with and without comorbidities.

## Introduction

Following a cluster of pneumonia cases in the city of Wuhan in Hubei province of China in December 2019^1^, a novel coronavirus (Severe Acute Respiratory Syndrome Coronavirus 2 [SARS-CoV-2]) was identified as the causative agent. The disease was subsequently named ‘coronavirus disease 2019’, or COVID-19^1,2^. The rapid global spread of COVID-19 prompted the World Health Organization (WHO) to declare a pandemic in March 2020^3^. As of November 2022, there have been more than 628 million cases of COVID-19 and ~6.6 million deaths^4^. This public health emergency sparked a remarkable global effort to develop vaccines using a wide range of traditional and novel platforms including messenger ribonucleic acid (mRNA), deoxyribonucleic acid (DNA), inactivated virus, live viral vectors, recombinant proteins, peptides, or virus-like particles (VLPs)^5,6^. As of November 2022, 234 of these vaccines have entered clinical testing and at least 49 of them have been authorized for use in at least one country^7^. Despite these successes, there remains an urgent global need to identify additional safe and effective vaccines, particularly candidates with the potential to provide some level of protection against a broad range of variants.

While it is now clear that humoral immunity is highly correlated with protection^8^, both innate and cellular immunity^9–12^ likely also contribute to protection against SARS-CoV-2 infection. Passive antibody transfer has proven protective in both non-human primate animal models and in the early treatment of some patients^13–15^. Correlates of protection have been proposed for both binding and neutralizing antibodies^8,16,17^. In parallel, a role for cell-mediated immunity which is intrinsically more cross-protective^18^, has been suggested for viral clearance and prevention of serious disease, as well as for long-term immunity^14,19–21^. Optimally, SARS-CoV-2 vaccines provide a well-coordinated response engaging multiple elements of the immune system.

The vaccine candidate developed by Medicago, hereafter referred to as CoVLP+AS03, consists of a Coronavirus-like particle (CoVLP) which is a self-assembling VLP that displays trimers of recombinant modified Spike (S) protein of SARS-CoV-2 (ancestral variant) embedded in a lipid envelope. The VLPs are produced in a plant host (*Nicotiana benthamiana*)^22^ and closely resemble the native structure of SARS-CoV-2 (see Supplementary Figure 1). The VLPs are administered with an oil-in-water adjuvant: Adjuvant System 03 (AS03) manufactured by GlaxoSmithKline (GSK)^23^. AS03 initiates a transient innate immune response at the injection site and draining lymph node in animal models^24,25^ and in human peripheral blood^10,26,27^ that can potentiate and shape both antibody and T-cell responses^28–30^. AS03 has been used in the licensed pandemic A/H1N1pdm09 influenza vaccines Arepanrix^TM^ H1N1 (in Canada) and Pandemrix (in Europe), of which over 90 million doses have been administered worldwide, as well as in other licensed (Q-Pan H5N1 in the USA) or candidate vaccines^31^. In Medicago’s Phase 1 study, AS03 significantly enhanced both cellular and humoral responses to CoVLP and the vaccine showed an acceptable safety profile^32^.

Herein we report safety and immunogenicity results of the Phase 2 portion of a Phase 2/3 randomized, placebo-controlled study conducted at multiple sites in Canada and the USA in three study populations: healthy adults 18–64 years of age (“Healthy Adults”), healthy adults ≥65 years of age (“Older Adults”) and adults ≥18 years of age with significant comorbidities (“Adults with Comorbidities”). The primary vaccine efficacy results from the Phase 3 portion of this study have recently been reported^33^ in which CoVLP+AS03 provided substantial protection (~70% vaccine efficacy) in an environment dominated by multiple variants including proportions of Delta (34%) and Gamma (32%) cases with lesser contributions from Alpha, Lambda and Mu strains (8%). However, the timing of the Phase 3 study during active global vaccine roll-out meant that relatively few “Adults with Comorbidities” (9.3%) and very few “Older Adults” (≥65 years of age; 0.5%) could be recruited. As a result, the Phase 2 portion of this study serves to confirm the immunogenicity of the CoVLP (3.75 µg)+AS03 formulation observed in the Phase 1 dose-ranging study^32^ across the adult age range and in those with and without high-risk comorbid conditions. In light of the ongoing emergence of new variants of interest/concern (VOI/VOC) and observed waning of vaccine efficacy^34,35^, the earlier start of the Phase 2 portion of the study permitted us to report here not only the NAb and cellular responses to the ancestral strain at 21-days after the second dose (D42) and at 6 months (D201), but also cross-reactive NAb responses to VOCs: Alpha, Beta, Gamma, Delta and Omicron (BA.1), at these same time points.

## Results

### Demographic and baseline clinical characteristics

Participants were screened for SARS-CoV-2 antibodies using a commercial ELISA (see Methods) that targets the nucleocapsid protein (although both seronegative and seropositive participants were enrolled) and randomized 5:1 to receive CoVLP+AS03 or placebo.

**Fig. 1:**
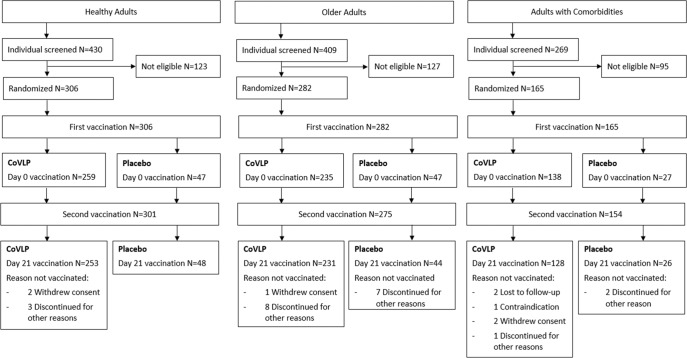
Trial profile – subject disposition. Enrollment and follow-up of study participants vaccinated with CoVLP with AS03 or placebo after the first and second dose administration. For both Healthy Adults and Older Adults, one subject was mis-dosed for the second dose administration and received a placebo in error. One individual in the Adults with Comorbidities group that was initially seropositive at D0 did not receive an injection. For more details of subject disposition, see Table 1.

Participant demographics are presented in Table 1 and subject disposition is presented in Fig. 1. The mean ages of Healthy Adults were 43.7 years and 42.4 years in the vaccinated and placebo groups, respectively. The mean ages of Older Adults were 71.1 years and 71.7 years in the vaccinated and placebo groups, respectively. Finally in Adults with Comorbidities, the mean ages were 56.6 and 57.1 in the vaccinated and placebo groups, respectively. In all groups, participants were predominantly White or Caucasian (96.7% in Healthy Adults, 98.6% in Older Adults, and 81.8% in Adults with Comorbidities). A minority of participants in the three populations self-identified as Hispanic or Latino (2.9% in Healthy Adults, 1.8% in Older Adults, and 14.5% in Adults with Comorbidities), Asian (2.0%, 1.1%, and 0%) or Black or African American (1.0%, 0.0%, and 17.6%). The Phase 3 portion of the Phase 2/3 study included a more diverse population than that recruited in Phase 2^33^.

**Table 1:** Summary of demographics and baseline characteristics (NCT04636697).

	Healthy Adults		Older Adults		Adults with Comorbidities	
	CoVLP 3.75 µg +AS03	Placebo	CoVLP 3.75 µg +AS03	Placebo	CoVLP 3.75 µg +AS03	Placebo
Participants (*N*)	259	47	235	47	138	27
Sex, *n* (%)						
Male	112 (43.2)	23 (48.9)	110 (46.8)	19 (40.4)	77 (55.8)	16 (59.3)
Female	147 (56.8)	24 (51.1)	125 (53.2)	28 (59.6)	61 (44.2)	11 (40.7)
Race/Ethnicity, n (%)						
American Indian or Alaska Native	0	0	1 (0.4)	0	1 (0.7)	0
Asian	5 (1.9)	1 (2.1)	3 (1.3)	0	0	0
Black or African American	2 (0.8)	1 (2.1)	0	0	23 (16.7)	6 (22.2)
Hispanic or Latino	8 (3.1)	1 (2.1)	5 (2.1)	0	17 (12.3)	7 (25.9)
White or Caucasian	251 (96.9)	45 (95.7)	231 (98.3)	47 (100)	114 (82.6)	21 (77.8)
Not reported	1 (0.4)	0	0	0	0	0
Age at the time of informed consent (years)						
Mean (SD)	43.7 (13.00)	42.4 (13.29)	71.1 (5.14)	71.7 (4.87)	56.6 (13.75)	57.1 (12.69)
Median	45.0	45.0	70.0	72.0	58.0	58.0
Min, Max	18, 64	18, 64	65, 88	65, 87	23, 82	24, 81
SARS-CoV-2 at Baseline, *n* (%)						
Positive	8 (3.1)	0	5 (2.1)	1 (2.1)	13 (9.4)	4 (14.8)
Negative	247 (95.4)	46 (97.9)	225 (95.7)	46 (97.9)	122 (88.4)	23 (85.2)

*Max* maximum, *Min* minimum, *n* number of participants in categories, *SD* standard deviation.

Three hundred and six Healthy Adults, 282 Older Adults, and 165 Adults with Comorbidities were enrolled in the study between 25th November 2020 and 24th March 2021. Of the 753 participants who received a first injection (placebo or vaccine), 730 (96.7%) also received a second injection. Details of the comorbidities in the Adults with Comorbidities cohort are presented in Supplementary Table 1.

### Safety

Safety and tolerability data after the first and second doses are provided for 306 and 301 participants in the Healthy Adults, 282 and 275 participants in the Older Adults, and 165 and 154 participants in the Adults with Comorbidities cohort, respectively, up to the safety cut-off date of April 28th, 2021. Overall, the vaccine was well-tolerated in all populations, with a slightly milder reactogenicity profile in the Older Adults (except for erythema) and Adults with Comorbidities.

**Fig. 2:**
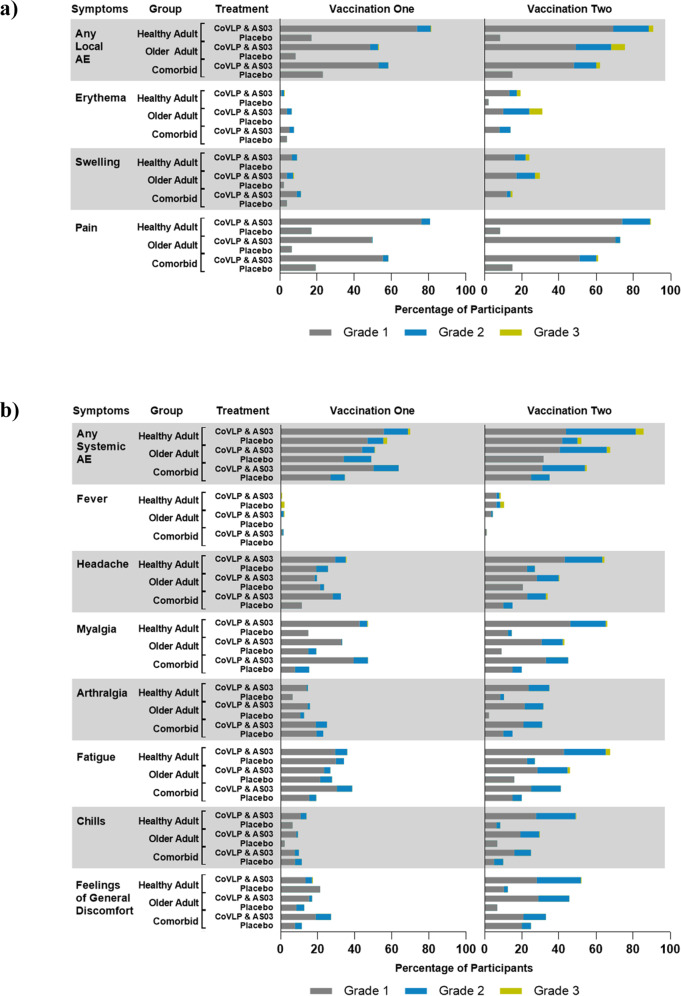
Solicited local and systemic adverse events 7 days after the first or second vaccine candidate dose. Participants were monitored for solicited local (panel **a**) and systemic (panel **b**) AEs from the time of vaccination through 7 days after administration of each dose. No Grade 4 (potentially life-threatening) events were reported. Participants who did not report AEs make up the remainder of the 100% calculation (not shown). If any of the solicited AEs persisted beyond 7 days after each vaccination (when applicable), it was recorded as an unsolicited AE. Fever was defined as oral temperature ≥38.0 °C.

Reactogenicity is illustrated in Fig. 2 for solicited local adverse events (AEs) (panel a) and systemic AEs (panel b). The frequency of at least one solicited AE in vaccinated individuals increased after the second dose relative to the first dose, in Healthy Adults and in Older Adults. However, Older Adults generally reported fewer solicited AEs (at least one AE after the first and second dose): 66.8% and 83.5% in the Older Adults compared to 88.4% and 94.5% of Healthy Adults reported solicited AEs respectively. This pattern of increased reactivity after the second dose was not observed in Adults with Comorbidities in whom the frequency of solicited AEs was 71.0% and 64.8% after first and second doses, respectively.

After the first dose, the most frequently reported solicited local AE was injection site pain (80.7% of Healthy Adults, 49.8% of Older Adults, 57.2% of Adults with Comorbidities) while the most frequently reported solicited systemic AEs were fatigue (35.9% of Healthy Adults, 26.8% of Older Adults, 38.4% of Adults with Comorbidities), myalgia (46.7% of Healthy Adults, 33.2% of Older Adults, 46.4% of Adults with Comorbidities) and headache (35.5% of Healthy Adults, 19.6% of Older Adults, 32.6% of Adults with Comorbidities). After the second dose, pain at the injection site was the most frequently reported solicited local AE (89.3% of Healthy Adults, 73.6% of Older Adults, 60.9% of Adults with Comorbidities), while fatigue (67.6% of Healthy Adults, 46.8% of Older Adults, 43.8% of Adults with Comorbidities), myalgia (66.0% of Healthy Adults, 43.3% of Older Adults, 48.4% of Adults with Comorbidities), and headache (64.0% of Healthy Adults, 40.7% of Older Adults, 33.6% of Adults with Comorbidities) were the most frequently reported systemic AEs.

In all populations, the large majority of solicited local or systemic AEs were Grade 1 (mild) or Grade 2 (moderate) in severity; and transient, typically resolving within 24 h to 3 days. Grade 3 (severe) solicited AEs were reported by 1.5% and 6.3% of Healthy Adults after the first and second doses respectively, while in Older Adults 0.9% and 8.7%. In Adults with Comorbidities, 0% and 3.1% of participants experienced Grade 3 AEs, after the first and second doses respectively. A greater frequency of Grade 3 erythema was observed post-second dose in Older Adults (6.9%) relative to Healthy Adults (2.0%). No Grade 4 AE was reported by any participant. No related Serious AEs (SAEs) have been reported to date. Adverse Events of Special Interest (AESI) monitored in this study were Vaccine-Associated Enhanced Disease (VAED), anaphylaxis, and potential immune-mediated disorders. At the data cut-off date, no cases of VAED, anaphylaxis or potential immune-mediated disorders were reported meeting the case definitions of these as per Protocol (see Supplementary Materials). As of July 2022, 3 pregnancies have been reported between 4 and 10 months after the second dose. The exposure to vaccine was pre-conception in these cases. The outcome of pregnancy is unknown in two subjects, while one subject had a spontaneous abortion after 8 weeks of pregnancy. Unsolicited AEs were monitored until Day 42, while SAEs and Medically Attended AEs (MAAEs) were monitored up to the end of the study. Incidences of unsolicited AEs, SAEs and MAAEs reported until day 42 were similar in Healthy Adults and Older Adults: 28.6% and 26.1% after first and second dose respectively in Healthy Adults, 30.2% and 22.5% after first and second dose respectively in Older Adults. The unsolicited events were reported at much lower incidence rates in Adults with Comorbidities: at 6.5% and 9.4% after first and second dose respectively (Supplementary Table 2).

### Immunogenicity: antibody response

**Fig. 3:**
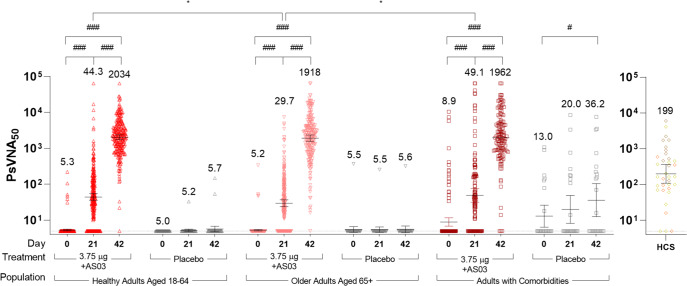
Humoral response in Healthy Adults, Older Adults, and Adults with Comorbidities. Pseudovirion neutralization titers at baseline (Day 0) and 21 days after the first dose (Day 21) or the second dose (Day 42) of either CoVLP+AS03 or placebo. Bars and numerical values indicate geometric means and error bars indicate 95CI. Significant differences between Days 0, 21 and 42 are indicated by # (^###^*p* < 0.001; paired T-test of log-transformed values, SAS). Significant differences across study populations at D21 or D42 are indicated by * (*p < 0.05; one-way ANOVA on log-transformed data for GMT, SAS). HCS: Human convalescent sera.

Ancestral strain-specific pseudovirion neutralizing antibody (NAb) responses in sera are illustrated in Fig. 3 and shown in Supplementary Table 3. Relative to both pre-vaccine sera (Baseline) and placebo controls, significant increases were observed in geometric mean titers (GMTs) in all three cohorts at 21 days (D21) after the first dose with further significant increases 21 days after the second dose (D42).

A single dose of CoVLP+AS03 induced a four-fold rise in NAb (seroconversion) in a slightly larger proportion of the Healthy Adults (51.3%) relative to Older Adults (38.4%, one-way ANOVA on log-transformed data for GMT, *p* < 0.01), an effect also reflected in the D21 GMTs (44.3 in Healthy Adults and 29.7 in Older Adults, *p* < 0.05). There was no significant difference in seroconversion between Healthy Adults and Adults with Comorbidities (44.2% in Adults with Comorbidities) or in D21 GMTs (49.1 in Adults with Comorbidities). The differences in seroconversion rates and GMTs between the Healthy Adults and Older Adults disappeared with the second dose of CoVLP+AS03. The GMTs at D42 were 2034 for Healthy Adults and 1918 in Older Adults. The seroconversion rates were 99.2% in Healthy Adults and 97.7% in Older Adults. Between Healthy Adults and Adults with Comorbidities at D42, there were no statistical differences in GMT (1962 in Adults with Comorbidities) although a difference in seroconversion (95.8% in Adults with Comorbidities, *p* < 0.05) was observed.

Consistent with observations from the Phase 1 clinical trial^32^, the NAb titers elicited by CoVLP+AS03 at D42 were approximately 10-times higher than those observed in a panel of human convalescent sera (HCS) at 2–3 months after recovery from natural infection (10.2x in Healthy Adults, 9.6x in Older Adults, and 9.9x in Adults with Comorbidities).

To assist in standardizing the NAb results, the WHO reference standard 20/136, pooled plasma from individuals with particularly high titers^36^, was included in the pseudovirion NAb assay yielding a reference titer of 1872. Expressing the GMT results in International Units (IU/mL; by dividing by 1.872) after the first and second doses respectively, the Healthy Adults in our Phase 2 study had NAb values of 23.7 and 1087 IU/mL (at D21 and D42), the Older Adults had values of 15.9 and 1025 IU/mL, and the Adults with Comorbidities had values of 26.2 and 1048 IU/mL. Using this methodology, HCS had a value of 106 IU/ml.

Prior to vaccination, 31 (4.12%) of the participants the Phase 2 portion of the study were seropositive including 24 vaccinated subjects for which NAbs were measured: 7 Healthy Adults (GMT at D0 32.3, 95% confidence interval (95CI): 8.6, 121.9), 5 Older Adults (GMT 46.0, 95CI: 7.2, 294.1) and 12 Adults with Comorbidities (GMT 141.6, 95CI: 39.4, 509.7). The NAb response was robust in those baseline seropositive CoVLP+AS03 recipients at both D21 with GMTs of 3078 (95CI: 397.8, 23810), 1762 (95CI: 174.4, 17787), and 1466 (95CI: 181.5, 11842) and at D42 with GMTs of 7426 (95CI: 2620, 21048), 6918 (95CI: 1189, 40259), and 4032 (95CI: 890, 18257), in Healthy Adults, Older Adults and Adults with Comorbidities, respectively.

Overall, two doses of CoVLP+AS03 induced strong and comparable NAb responses in all groups, and a single dose of CoVLP+AS03 was sufficient to generate a robust response in seropositive participants. Although small differences between Healthy Adults and Older Adults were observed at D21 after the first dose, these differences disappeared with the second dose.

### Immunogenicity: neutralizing antibody cross-reactivity and durability

**Fig. 4:**
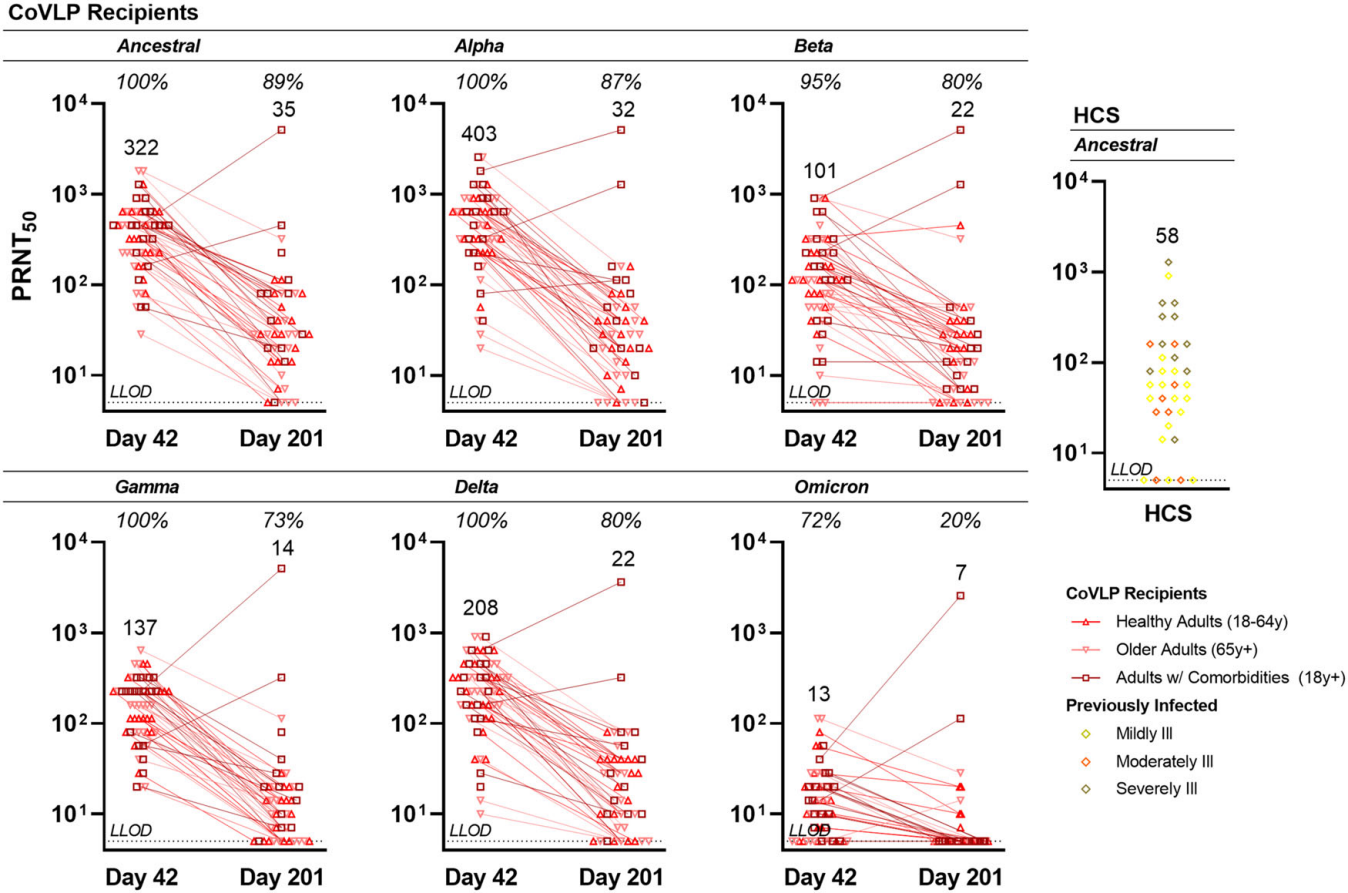
Neutralizing antibody cross-reactivity to SARS-CoV-2 variants. To evaluate neutralizing antibody cross-reactivity, NAb of participants vaccinated with 3.75 µg CoVLP adjuvanted with AS03 (*n* = 19, 20, and 18 for Healthy Adults aged 18–64, Older Adults aged 65 or more, and Adults with Comorbidities, respectively at D42 and *n* = 17, 15, and 13 for Healthy Adults aged 18–64, Older Adults aged 65 or more, and Adults with Comorbidities, respectively at D201) were quantified in a live virus neutralization assay with the ancestral strain and the Alpha, Beta, Gamma, Delta and Omicron variants. Convalescent sera or plasma samples were collected at least 14 days after a positive diagnosis of COVID-19 (RT-PCR) from individuals whose illness was classified as mild, moderate, or severe/critical (*n* = 35). Individual values are indicated with red lines; geometric means are indicated above each series of data points. Percentage seropositivity relative to the variant being tested are shown at the top of each dataset. The lower limit of detection (LLOD) is shown with a dotted black line. HCS: Human convalescent sera.

NAb cross-reactivity to VOC at both 21-days (D42) and six-months (D201) post-vaccination were examined using a live virus neutralization assay (Fig. 4, Supplementary Table 4). NAb were readily detected at D42 in 57 of 57 (100%) participants to the ancestral strain as well as Alpha, Gamma and Delta variants, and in 54 of 57 (94.7%) participants to the Beta variant (generally 10–100x higher than the lower limit of detection (LLOD)). Cross-reactivity to the Omicron (BA.1) variant was reduced and observed in 41 of 57 (71.9%) participants. The D42 GMTs are reported as follows: 322 (95CI: 254, 408) to the ancestral strain, 403 (95CI: 305, 533) to Alpha, 101 (95CI: 72.3, 141) to Beta, 137 (95CI: 109, 172) to Gamma, 208 (95CI: 158, 273) to Delta, and 13.2 (95CI: 10.5, 16.7) to Omicron VOC.

At D201, persistent reactivity was observed in 40 of 45 (88.9%) participants to the ancestral strain, and persistent cross-reactivity was observed in 39 of 45 (86.7%) participants to the Alpha variant, 36 of 45 (80.0%) participants to the Beta and Delta variants, 33 of 45 (73.3%) participants to the Gamma variant, and 9 of 45 (20.0%) participants to the Omicron variant. The D201 GMTs to the ancestral strain and Alpha, Beta, Gamma, Delta, and Omicron variants were 34.6 (95CI: 23.1, 51.8), 31.8 (95CI: 20.9, 48.3), 21.9 (95CI: 14.2, 33.8), 14.5 (95CI: 9.9, 21.2), 21.6 (95CI: 14.7, 31.9), and 7.2 (95CI: 5.2, 10.0), respectively. Using the live virus neutralization assay for the panel of HCS, 30/35 (85.7%) had a detectable NAb response to the ancestral strain (GMT 58.3; 95CI: 35.1, 96.8).

Overall, cross-reactivity to Alpha and Delta variants was comparable to the ancestral strain while a modest decrease was observed for the more antigenically distant Beta and Gamma strains. Cross-reactivity to the antigenically distinct Omicron (BA.1) variant was reduced.

### Immunogenicity: cell mediated response

**Fig. 5:**
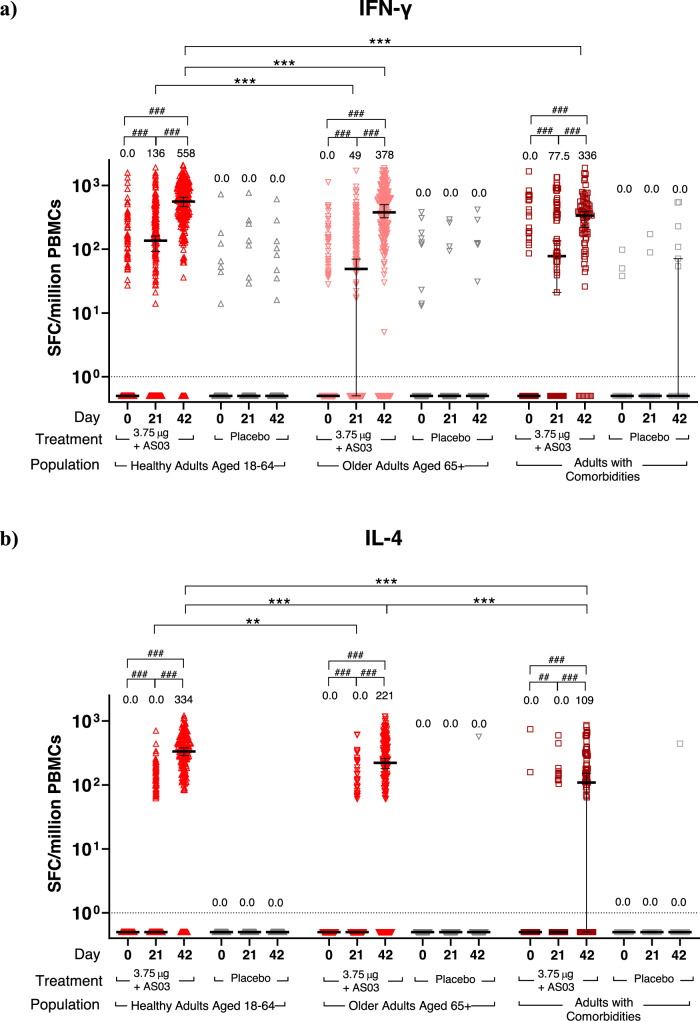
Cellular immune response in Healthy Adults, Older Adults, and Adults with comorbidities. IFN-γ and IL-4 ELISpot Responses. IFN-γ (panel **a**) and IL-4 (panel **b**) spot forming cell (SFC) counts at baseline (Day 0), 21 days after the first immunization (Day 21) and 21 days after the second immunization (Day 42) with CoVLP (3.75 µg) adjuvanted with AS03 or placebo are represented. Bars indicate median (±95CI). Results of statistical analysis are represented for relevant comparisons. Significant differences between time points for each vaccine regimen are indicated by # (^##^*p* < 0.01, ^###^*p* < 0.001; Wilcoxon signed rank test, SAS). Significant differences between study populations at D21 and D42 are indicated by * (*p < 0.05, ***p < 0.001; Kruskal–Wallis test, GraphPad Prism v9.0.1). Both IFN-γ and IL-4 responses in participants injected with placebo were significantly lower (not represented, Wilcoxon rank sum test, SAS) than participants who received the adjuvanted CoVLP at both Day 21 and Day 42 regardless of the population cohort. An arbitrary value of 0.5 SFC was assigned to samples with undetectable values.

To assess the antigen-specific Th1 response in peripheral blood mononuclear cells (PBMC), IFN-γ ELISpots were performed. As observed during the Phase 1 CoVLP clinical trial (NCT0445004)^32^, a substantial minority of individuals (21%, 17% and 27% in Healthy Adults, Older Adults and Adults with Comorbidities, respectively) had pre-existing (D0) IFN-γ ELISpot responses to the S protein. Vaccination with CoVLP+AS03 induced a significant (Wilcoxon signed rank test, *p* < 0.001) increase of the IFN-γ response at D21 in all groups and this response was further increased (*p* < 0.001) after the second dose at D42 in the three populations, while no effect was observed in placebo recipients (Fig. 5a). Healthy Adults vaccinated with CoVLP+AS03 had a higher IFN-γ response compared to Adults with Comorbidities at D42 (*p* < 0.001) and to Older Adults at both D21 and D42 (both *p* < 0.001). The IFN-γ response at D0 significantly correlated (Spearman correlation test, *p* < 0.001) with detection of NAb at baseline and this positive correlation was maintained after both the first and the second doses in vaccinated participants. The proportion of participants with a detectable IFN-γ response was 69% of the Healthy Adults, 54% of Older Adults and 63% of the Adults with Comorbidities after one dose (D21) and reached 96% (Healthy Adults) and 88% (Older Adults and Adults with Comorbidities) after the second dose.

To assess the antigen-specific Th2 response induced by the vaccine candidate, IL-4 ELISpots were performed. Also consistent with the results from the CoVLP Phase 1 clinical trial^32^, with the exception of two Adults with Comorbidities, no subject had a measurable IL-4 response pre-vaccination. Vaccination with CoVLP+AS03 significantly (Kruskal–Wallis test, *p* < 0.01) increased the IL-4 responses after one dose and this was further significantly (*p* < 0.001) increased after a second dose in the three populations, while no effect was observed in placebo groups (Fig. 5b). After two doses, Adults with Comorbidities had a significantly (*p* < 0.001) lower IL-4 responses than either Healthy Adults or Older adults. The IL-4 response was also significantly lower in Older Adults relative to Healthy Adults after one (*p* < 0.01) or two (*p* < 0.001) doses. While a small portion of participants responding to CoVLP+AS03 elicited an IL-4 response after one dose (33%, 17% and 18% in Healthy Adults, Older Adults and Adults with Comorbidities respectively), the proportion of responders increased to 92% (Healthy Adults), 79% (Older Adults) and 62% (Adults with Comorbidities) after the second dose. The number of Th1-type (IFN-γ) responding cells was consistently 1.7 to 3.0-fold greater than the Th2-type (IL-4) cells in all groups 21 days after the second dose.

## Discussion

The Phase 2 portion of the Phase 2/3 study of CoVLP+AS03 described herein was designed to confirm the selection of CoVLP dose and adjuvant identified in the Phase 1 trial^32^ and to assess the performance of the chosen formulation in three populations: Healthy Adults, Older Adults, and Adults with Comorbidities. The primary outcomes for the Phase 2 portion of this study focused on short-term (up to D42) safety and tolerability of CoVLP+AS03 and the ability of this novel vaccine candidate to induce both NAb and cellular responses to the SARS-CoV-2 spike protein. The Phase 3 portion of the study was designed to confirm the tolerability and safety of the vaccine formulation and revealed an overall vaccine efficacy (VE) of 71.0% against any symptomatic disease, 75.3% against the Delta variant and 88.6% against the Gamma variant (and 100% for a smaller number of Alpha, Lambda, and Mu variant infections) (all per Protocol set)^33^. Among the COVID-19 cases adjudicated to be included in the primary vaccine efficacy analysis for the Phase 3, none was found to be caused by either the ancestral strain or the Omicron strain.

Reactogenicity in Healthy Adults in the Phase 2 study was similar to the profiles observed in both the small Phase 1 study in 18–55-year-old adults who received the CoVLP (3.75 µg) + AS03 formulation (*n* = 20)^32^ and in the much larger Phase 3 portion of the study (21,778 Healthy Adults and 2361 Adults with Comorbidities in the Intention-to-treat set)^33^. Local reactogenicity was primarily characterized by injection site pain in most participants while systemic reactogenicity was primarily characterized by myalgia, fatigue, and/or headache in approximately 70% of the study participants; solicited AEs were mostly mild-to-moderate and transient in nature. It is noteworthy that the Phase 1 trial included both unadjuvanted and AS03-adjuvanted groups at three CoVLP dose levels (3.75, 7.5 and 15 µg/dose)^32^, and higher reactogenicity was noted in adjuvanted formulations, as expected. Both local and systemic reactogenicity observed in this study were in line with what would be expected from AS03-adjuvanted vaccines^26,37,38^. The Phase 2 safety data in the Older Adults were also consistent with observations in the small number of participants ≥65 years of age in the Phase 3 portion of the study (127 Older Adults in the Intention-to-treat set)^33^. As expected^27^ and has been observed for mRNA, adenovirus vector, inactivated virus, and recombinant protein-based COVID-19 vaccines^39–46^, slightly reduced reactogenicity was observed in the Older Adult population, as compared to the Healthy Adult cohort^47^. Overall, these Phase 2 data contribute to the overall safety profile of CoVLP+AS03 and suggest that this vaccine candidate is well-tolerated in adults ≥18 years of age, with and without comorbidities.

Both the humoral and cellular immune responses seen in the Phase 2 study confirmed the robust immunogenicity results documented in the 18–55 year-old Healthy Adults during the Phase 1 study^32^ and extended these observations to Older Adults and Adults with Comorbidities. After two doses of CoVLP+AS03, seroconversion was observed in ~98% of the participants and the overall NAb titers were ~10x higher than those found in convalescent sera. The NAb responses in the Adults with Comorbidities were generally very similar to those seen in the Healthy Adults after both the first and second doses. Although the NAb response after the first dose of CoVLP+AS03 was slightly reduced in the Older Adults compared to the Healthy Adults, the difference between these two cohorts disappeared after the second dose. This observation of reduced immunogenicity in Older Adults after the first dose is consistent with the generally reduced capacity of older individuals to respond to vaccination and with findings with other SARS-CoV-2 vaccines^46,48,49^. The decreased ability of even healthy older individuals to mount strong immune responses after vaccination is likely multi-factorial including a general decline in immune function (i.e.: immunosenescence) and chronic low-level inflammation (so-called ‘inflammaging’)^50^. Consistent with the observations that adjuvants can enhance vaccine-induced responses in older individuals^51^, these results suggest that two-doses of CoVLP+AS03 can overcome the age-associated limitations relating to NAb production.

The total number of participants in the current study across all populations with pre-existing NAb titers to SARS-CoV-2 was low (*n* = 31, 4.12%) but vaccination of these individuals with CoVLP+AS03 nonetheless induced a substantial increase in titers, suggesting that CoVLP+AS03 can significantly boost a pre-existing memory response. This observation, consistent with other SARS-CoV-2 vaccines^52–54^ provides strong support for vaccinating both infection naïve and previously infected individuals.

Overall, the NAb titers induced by CoVLP+AS03 in all populations compared favourably with a panel of convalescent serum/plasma. While this method can be used to draw broad comparisons between studies^8,16^, this approach has limitations for comparing responses between groups and between trials with different vaccines. For this reason, we included the WHO reference standard 20/136, pooled antibodies from recovered COVID-19 patients with very high NAb response in multiple laboratory studies^55^ in our serological analysis so that the performance of CoVLP+AS03 could be assessed relative to other vaccines. This analysis confirmed the induction of very high NAb titres (>1000 IU/mL) by CoVLP+AS03 in all three groups studied. In this study, the mean standardized value for the HCS was 106.3 IU/mL, a value noticeably lower than the 1000 IU/mL of the 20/136 WHO reference pool, a difference attributable to the selection of high titer individuals in the pooled WHO reference standard relative to the more diverse titers observed across the mild, moderate and severely ill individuals from which the convalescent sera in this study are drawn.

The emergence and dominance of SARS-CoV-2 variants has resulted in reduced vaccine efficacy at least in part due to reduced cross-reactivity of immune responses mounted to the ancestral spike protein present in all deployed vaccines relative to the antigenically differentiated VOI and VOC in circulation^34,56^. Investigation of CoVLP+AS03’s capacity to generate NAbs to VOI/VOC is relevant to understanding its position in the evolving viral landscape. The robust and durable cross-reactivity to the Alpha, Delta, and Gamma variants observed correlate well with the efficacy observed in the efficacy portion of the Phase 3 trial wherein efficacies of 75.3% (Delta), 88.6% (Gamma) and 100% (Alpha, albeit with a reduced number of participants) were observed (all per Protocol set)^33^. Efficacy to the Omicron variants remains unknown at the time of writing but based on the observed NAb cross-reactivity observed herein, and consistent with the results published for other vaccines^57,58^, VE to Omicron is likely to be reduced relative to the predominant Gamma and Delta variants encountered during the initial portion of the Phase 3 study.

Although attention to vaccine-induced immune responses for SARS-CoV-2 has focused primarily on antibody production, there is compelling evidence that cellular responses contribute to both recovery from infection and long-term immunity^14,59^. Despite the growing consensus that T cell responses are important, neither the optimal vaccine-induced cellular response^60^ nor the influence of pre-existing, cross-reactive T cell immunity on vaccine-induced responses are yet fully understood^61^. In the current study, the Th1 (IFN-γ) and Th2 (IL-4) cellular response induced by CoVLP+AS03 was, like the NAb response, consistent with the Phase 1 results^32^. Although an IFN-γ dominated response was observed after the first dose in the three populations, the strong Th1 bias evolved to include a substantial IL-4 induction after the second dose. Even though the IL-4 response increased significantly after the second dose, spot forming unit (SFU) counts for IFN-γ remained approximately 1.7 to 3-fold higher than those for IL-4. The limitation of using only these two ELISpot assays to assess the pattern of cellular immunity generated by CoVLP+AS03 is acknowledged. In the current work, a substantial proportion of participants (21%, 17% and 27% for Healthy Adults, Older Adults and Adults with Comorbidities respectively) had evidence of a pre-existing IFN-γ response to the SARS-CoV-2 spike peptide pool at D0 (Fig. 4). Interestingly, there was no accompanying pre-existing IL-4 response. Very similar pre-existing T cell responses have been reported in 26–60% of SARS-CoV-2 naïve individuals^59,61–63^. Since T cell responses are intrinsically cross-reactive, such pre-existing immunity is widely considered to be the result of prior exposure to circulating human ‘common cold’ coronaviruses^64^. As has been previously reported^61^, participants with evidence of pre-existing cross-reactive memory in our study mounted both antibody and cellular responses to CoVLP+AS03 vaccination that were at least as strong as those seen in those who appeared to be antigen-naïve. Whether or not the boosting of such pre-existing immunity contributes to the protection provided by vaccination is unknown.

While the possibility of VAED was initially a point of concern in COVID-19 vaccine development^65,66^, there has since been no evidence of disease enhancement in animal models, clinical trials or in the real-world data reported to date. Specifically, there has been no suggestion that vaccine-induced Th2 responses are associated with VAED^60^. On the contrary, it is possible that the Th2-type response induced by CoVLP+AS03 that included strong IL-4 production, contributed to the high vaccine-induced NAb titers by supporting T helper follicular cell expansion, germinal center formation and B-cell maturation^20,67–69^. Although speculative, such Th2-driven effects could also contribute to long-term memory B-cell responses^70^.

Although a robust cellular immune response was induced in most of the Older Adults included in this study, the slightly lower IFN-γ and IL-4 responses observed in this population suggest that at least some aspects of the aged immune system cannot be entirely overcome with CoVLP+AS03. Given the clear age-related differences in both the clinical manifestations of COVID-19 and the immune response generated by SARS-CoV-2 infection^71^, it is not surprising that vaccine-induced responses might also differ between younger and older individuals^72^. Indeed, similar age-related differences in immune responses have been reported for several of the SARS-CoV-2 vaccines in development or in use^42,73^ and age-related differences in vaccine efficacy with some of the deployed vaccines have been reported^74–76^. We also observed a small but significant difference in the cellular immune responses in Adults with Comorbidities relative to Healthy Adults. The spectrum of comorbidities exhibited by the participants in this study (detailed in Supplementary Table 1) does not allow speculation regarding specific mechanisms of action to explain this reduction as some of the implicated conditions can potentially promote pro-inflammatory responses while others or their associated treatments can be immunosuppressive. Given the high NAb titers induced by CoVLP+AS03 and the generally strong cellular response across all populations after two doses, it is unclear whether the in vitro differences in cellular responses will result in clinically relevant differences in protection. Indeed, in the Phase 3 portion of this study, overall efficacy of CoVLP+AS03 in Adults with Comorbidities was actually slightly higher than that observed in Healthy Adults (76.8%: 95CI 21.5, 94.8 and 70.9%: 95CI 57.7, 80.4 respectively; all per Protocol set)^33^.

In conclusion, this report of the Phase 2 portion of the Phase 2/3 study of CoVLP+AS03 provides evidence that this vaccine candidate is well-tolerated and highly immunogenic in adults ≥18 years of age with and without comorbidities. Compared to either a panel of convalescent serum/plasma or the WHO standard serum reagent (20/136), the NAb response induced by CoVLP+AS03 was among the highest reported for any SARS-CoV-2 vaccine^8^. Across the broad age range of study participants (18–88 years of age), almost all mounted either a strong NAb response, a Th1/Th2-pattern cellular response, or both, following two doses of CoVLP+AS03. Cross-reactive NAb responses to the Alpha, Beta, Gamma, Delta and even the Omicron BA.1 variants were substantial at 21 days after the second dose. The NAb responses were well-maintained for at least 6 months after vaccination in most participants for all but the Omicron variant.

## Methods

### CoVLP vaccine candidate and adjuvant

The CoVLP vaccine candidate consists of the full-length spike protein from SARS-CoV-2 (strain hCoV-19/USA/CA2/2020) incorporating the modifications R667G, R668S, R670S, K971P, and V972P. The modified spike protein was expressed in *Nicotiana benthamiana* by transient transfection, resulting in spontaneous trimer formation and CoVLP assembly and budding^32^. The CoVLPs were purified and formulated in phosphate-buffered saline (PBS) with polysorbate 80. The AS03 adjuvant is an oil-in-water emulsion containing DL-α-tocopherol and squalene, supplied by GSK. The placebo was PBS with polysorbate 80.

### Vaccine preparation and injection

CoVLP was available in multi-dose vials (10 doses/vial) at 15 µg/mL and stored at 2–8 °C until shortly before use. The AS03 adjuvant was supplied in multi-dose vials (10 doses/vial). Prior to injection, 2.5 mL of CoVLP and 2.5 mL of AS03 were mixed to obtain 10 vaccine doses of 0.5 mL each. Each dose of the vaccine contained 3.75 µg of CoVLP formulated in PBS with polysorbate 80, 11.86 mg of DL-α-tocopherol and 10.69 mg of squalene. Once mixed, CoVLP+AS03 was stored at room temperature protected from light and had to be used within 6 h. All injections were administered intramuscularly in the deltoid using a 23-gauge needle of appropriate length based on body mass index (BMI). The first and second doses were administered contralaterally when possible.

### Study design

The phase 2 portion of the study was a randomized, observer-blinded, placebo-controlled study with male and female participants. The study was approved by Advarra Central Institutional Review Boards (USA and Canada), IWK Health Centre Research Ethics Board, McGill University Health Centre Research Ethics Board, Comité d’Éthique de la Recherche du CHU de Québec – Université Laval as well as the Center for Biologics Evaluation and Research (CBER) of the U.S. Food and Drug Administration (FDA) and Biologic and Radiopharmaceutical Drugs Directorate (BRDD) of Health Canada and was carried out in accordance with the Declaration of Helsinki and the principles of Good Clinical Practices. Written informed consent was obtained from all study participants prior to any study procedure. Participants were offered modest compensation for their participation in this study (i.e.: time off work, displacement costs, etc.).

Participants were screened up to 14 days in advance of the first vaccine administration and must have demonstrated a satisfactory baseline medical assessment by history, general physical examination, hematologic, biochemic, and serologic analysis. Although a test for SARS-CoV-2 antibodies was performed at screening using a commercial ELISA that targets the nucleocapsid (N) protein (ElecSys: Roche Diagnostics), both seronegative and seropositive participants were enrolled.

For Healthy Adults, participants had to be 18–64 years of age. For Older Adults, participants had to be 65 years of age or older and to be non-institutionalized (e.g., not living in rehabilitation centers or old-age homes; living in an elderly community was acceptable). For Adults with Comorbidities, most frequent comorbidities were appetite and general nutritional disorders (obesity), allergic conditions, vascular hypertensive disorders, lipid metabolism disorders, glucose metabolism disorders (diabetes mellitus) and joint disorders (details in Supplementary Table 1). In this study, the Adults with Comorbidities group was older than the Healthy Adults group, had a different ethnic composition, and had a higher rate of baseline seropositivity (details in Table 1).

For both Healthy Adults and Older Adults, participants must have been in good general health with no clinically relevant abnormalities that could jeopardize subject safety or interfere with study assessments, as determined by medical history, physical examination, and vital signs, and have had a body mass index less than 30 kg/m^2^. Adults with Comorbidities included participants with one or more comorbid conditions that puts them at higher risk for severe COVID-19 such as obesity, hypertension, type 1 or type 2 diabetes, chronic obstructive pulmonary disease (COPD), cardiovascular diseases, chronic kidney diseases, or a compromised immune system (e.g., treatment-controlled HIV infection, organ transplant recipients, or patients receiving cancer chemotherapy). Female participants of childbearing potential must have had a negative pregnancy test result at screening and vaccination and used a highly effective method of contraception for one month prior to vaccination and at least one month after the last study vaccination. Exclusion criteria for Healthy Adults and Older Adults included i) any significant acute or chronic, uncontrolled medical or neuropsychiatric illness, ii) any chronic medical condition associated with elevated risk of severe outcome of COVID-19, iii) any confirmed or suspected current immunosuppressive condition or immunodeficiency, including cancer, HIV, hepatitis B or C infection, iv) current autoimmune disease, v) administration of any medication or treatment that could alter the vaccine immune response. In all three study populations, exclusion criteria also included vi) administration of any vaccine within 14 days prior to vaccination or planned administration of any vaccine up to Day 28 of the study, vii) administration of any other SARS-CoV-2 / COVID-19, or other experimental coronavirus vaccine at any time prior to or during the study, viii) history of virologically-confirmed COVID-19, ix) rash, dermatological condition, tattoos, muscle mass, or any other abnormalities at injection site that could interfere with injection site reaction rating, x) use of prophylactic medications (e.g., antihistamines [H1 receptor antagonists], nonsteroidal anti-inflammatory drugs [NSAIDs], systemic and topical glucocorticoids, non-opioid and opioid analgesics) within 24 h prior to the vaccination to prevent or pre-empt symptoms due to vaccination, xi) history of a serious allergic response to any of the constituents of CoVLP, including AS03, xii) history of documented anaphylactic reaction to plants or plant components (including tobacco, fruits and nuts), xiii) personal or family (first-degree relatives) history of narcolepsy, xiv) history of Guillain-Barré Syndrome. Sentinel participants (10 in each group) were first enrolled in Older Adults and Adults with Comorbidities groups, and unblinded safety data after each dose were reviewed by the Independent Data Monitoring Committee (IDMC). Enrollment into the Phase 2 portion of the study was closed on 25th March 2021.

The participants and the personnel collecting the safety information and working in testing laboratories remained blinded to treatment allocation. On D0, D21 and D42, serum and PBMC were processed for immune outcomes. All safety information was collected, and all laboratory procedures were carried out by study staff blinded to treatment allocation.

### Primary and secondary objectives

The primary objectives of the Phase 2 portion of the study were to assess safety and tolerability and immunogenicity to CoVLP+AS03 at Study Day 0, Day 21 and Day 42, compared to placebo in Healthy Adults, Older Adults, and Adults with Comorbidities.

Primary safety outcomes were the occurrence(s) of i) immediate AEs within 30 min after each vaccination; ii) solicited local and systemic AEs up to 7 days after each vaccination; iii) unsolicited AEs, serious AEs (SAEs), AEs leading to withdrawal, AESIs, and deaths up to 21 days after each vaccination; iv) normal and abnormal urine, and hematological and biochemical values.

Primary immunogenicity outcomes were i) NAb titers measured using a pseudovirion neutralization assays and ii) IFN-γ and IL-4 ELISpot responses at 21 days after each dose of vaccine.

A secondary safety outcome was the occurrence(s) of SAEs, AEs leading to withdrawal, AESIs, and deaths from 22 days after the last vaccination up to the end of the study. Secondary immunogenicity outcomes were immune responses measured on study days 128, 201 and 386 The data collected up to the last time point of study day 386 will be released once study follow-up has been completed.

### Safety assessments

In this manuscript, safety assessments are reported up to the cut-off date of April 28th, 2021. Both passive (diary) and active monitoring of safety signals were performed for the first 42 days of the study and continued throughout the study. Active monitoring included telephone contacts with participants one and eight days after each vaccination as well as a site visit on Day 3 after vaccination. Participants were required to return to the Investigator site on Days 128, 201, and 386 for safety follow-ups and immunogenicity assessments. In addition, study participants were contacted weekly to detect any symptoms that may be associated with COVID-19 and were instructed to report any changes in their health to the Investigator site.

Solicited AEs were assessed by the participants as Grade 1 to 4 (mild, moderate, severe, or potentially life-threatening) according to criteria described in the Protocol (see Supplementary Material)^32^. Per Protocol, all solicited AEs (local, systemic) were considered related events. Unsolicited adverse events were monitored for 21 days after each dose. SAEs, MAAEs, AEs leading to withdrawal and AESIs were collected throughout the study. Throughout the trial, an unblinded medical monitor (Syneos Health, Canada) was reviewing stopping rules from the trial and the Pharmacovigilance team at Medicago were reviewing AESIs (including VAED, anaphylaxis or severe allergic reactions, and potential immune-mediated disorders) and SAEs (including death). Methods for grading unsolicited AEs and trial-stopping rules are detailed in the Protocol. Unsolicited AEs were coded according to the terms used in the Medical Dictionary for Regulatory Activities (MedDRA), version 24.0.

Based on IDMC recommendations, the following event(s) could pause or halt the study for further review and assessment of the event(s): i) If any subject experienced an SAE after administration of the vaccine that was considered related to vaccine; ii) If 5% or more subjects who received the CoVLP formulation experienced the same or similar Grade 3 or higher solicited local AE or systemic AE, which began within 7 days after administration of the vaccine; or experienced the same or similar Grade 3 or higher unsolicited AE (including symptoms, signs or laboratory safety AEs) that was judged anything but unrelated to the vaccine; iii) If an important imbalance in unusual manifestations of COVID-19 or severity of COVID-19 symptoms was observed between the CoVLP and placebo groups.

### SARS-CoV-2 pseudovirion and live virus neutralization assays, convalescent sera/plasma and WHO reference standard

The pseudovirion neutralization assay (Nexelis, Quebec, Canada) was based on a genetically modified Vesicular Stomatitis Virus (VSV) from which the glycoprotein G was removed, and a luciferase reporter introduced^32^. The modified VSV vector expresses full length SARS-CoV-2 S glycoprotein (NXL137-1 in POG2 containing 2019-nCOV Wuhan-Hu-1; Genebank: MN908947) from which the last nineteen amino acids of the cytoplasmic tail were removed (rVSVΔG-Luc-Spike ΔCT). Pseudovirions are mixed with sera of vaccinated individuals and the degree of neutralization is quantified using human ACE-2 expressing VERO cells and reduction in luciferase-based luminescence. For each sample, the neutralizing titer was defined as the reciprocal dilution corresponding to the 50% neutralization titer (NT_50_), when compared to the pseudovirion control without sera. Samples below cut-off were attributed a value of 5 (half the minimum required dilution).

Neutralizing antibody analysis was performed using a cell-based cytopathic effect assay (VisMederi, Sienna, Italy) based on ancestral SARS-CoV-2 virus (2019 nCOV ITALY/INMI1, provided by EVAg; Genebank: MT066156). For each sample, the neutralizing titer was defined as the reciprocal dilution corresponding to the NT_50_. Samples below cut-off were attributed a value of 5 (half the minimum required dilution). Full details of this assay are provided in Supplementary Methods.

For assessment of cross-reactivity against variants, the assay was conducted with live virus: Alpha (swab isolate 14484; mutations: N501Y, A570D, D614G, P678H, T716I, S982A, T572I, S735L, D69/70, D144Y), Beta (hCoV-19/Netherlands/NoordHolland_10159/2021), Gamma (human isolate PG_253 Clade Nexstrain 20 J/501Y.V3; Mutations: L18F, T20N, P26S, D138Y, R190T, K417T, E484K, N501Y, D614G, H655Y), Delta (sab isolate 31944, mutations: G142D, E156–158del, R158G, L452R, T478K, D614G, P681R, R582Q, D950N, V1061V), and Omicron (VMR_SARSCOV2_Omicron_C1, BA.1, Mutations: A67V, H69del, T95I, G142D, V143–145del, L212I, K417N, N440K, G446S, S477N, E484A, Q493R, G496S, Q498R, N501Y, Y505H, T547K, D614G, H655Y, N679K, P681H, N764K, D796Y, N856K, Q954H, N969K) variants.

Results were compared to sera/plasma from COVID-19 convalescent patients. These were collected from a total of 35 individuals with confirmed diagnosis. Time between the onset of the symptoms and sample collection varied between 27 and 105 days. Four sera samples were collected by Solomon Park (Burien, WA, USA) and 20 sera samples by Sanguine BioSciences (Sherman Oaks, CA, USA); all were from non-hospitalized individuals. Eleven plasma samples were collected from previously hospitalized patients at McGill University Health Centre. Disease severity was ranked as mild (COVID-19 symptoms without shortness of breath), moderate (shortness of breath reported), or severe (hospitalized). These samples were analyzed in parallel to clinical study samples, using the assays described above. Demographic characteristics have been previously described^32^.

To facilitate the comparability of results across different trials, the WHO International Standard for anti-SARS-CoV-2 immunoglobulin (human; NIBSC code: 20/136) was established to allow conversion of neutralization assay titers into international units (IU/mL). This standard consists of pooled plasma obtained from eleven individuals recovered from SARS-CoV-2 infection and with high NAb titers. Upon multiple assessments using the validated PNA assay, a conversion factor of 1.872 was established. Hence, the antibody titers presented throughout this manuscript could be expressed as IU/mL by dividing the NT_50_ by this factor. Similarly, a conversion factor of 0.91 could be applied to convert the live virus Wuhan strain titers to IU/mL.

### Immunogenicity- interferon-γ and interleukin-4 ELISpot

PBMC samples from study participants were analyzed by IFN-γ or IL-4 ELISpots (Caprion, Quebec, Canada) using a pool of 15-mer peptides with 11 amino-acid overlaps from SARS-CoV-2 S protein (USA-CA2/2020, Genbank: MN994468.1, Genscript, purity >90%). Full details of the methodology are in Supplementary Methods.

### Analysis populations and statistical analysis plan

Randomization was managed by Syneos Health (Canada) with Medicago oversight using Medidata Rave RTSM interactive randomization tool (2021.2.0, Medidata, USA). Statistical analysis and data presentation was conducted using SAS (SAS Institute, North Carolina) and Prism (GraphPad Software, San Diego).

The sample size of 753 participants made it possible to perform the initial evaluation of the vaccine immunogenicity and detect major differences in rates of AEs between groups. The sample size was not large enough to detect all types of, including less frequent or rare, AEs. The analyses of all immunogenicity endpoints were performed using randomized participants who received CoVLP+AS03 or placebo from the Intention-to-Treat population set. Immunogenicity was evaluated by humoral immune response (NAb assays) and cell-mediated immune response (ELISpot) induced in participants on D0, 21 and 42. To assess the humoral immune response, the GMT was calculated and compared between CoVLP+AS03 and placebo groups using an ANOVA on the log-transformed titers. The log transformation was used to meet the normal assumption for the ANOVA. At each time point, the GMT and corresponding 95CI of each treatment were obtained by exponential back-transformation of the least square mean. GMT were compared between study populations at D21 and D42 using an ANOVA. Comparison of D0 seronegative and seropositive values at D21 was conducted by unpaired *t* test of log-transformed values. Fisher’s exact test was used to compare seroconversion rate between the treatment groups. The 95CI for seroconversion was calculated using the exact Clopper-Pearson method. The specific Th1and Th2 responses along with the corresponding 95CI for the median induced on D0, D21 and D42 were measured by the number of cells expressing IFN-γ and IL-4 respectively, using ELISpot. The difference in IFN-γ and IL-4 response between treatment groups at each time point was compared using a non-parametric Wilcoxon Rank Sum Test. The difference in IFN-γ and IL-4 were also compared between study populations at D21 and D42 using a Kruskal-Wallis Test. Safety assessment are based on the Safety Analysis Set, i.e., all participants who received at least one dose of either the CoVLP+AS03 or placebo. Occurrence and incidence of safety events were reported for each treatment group. No formal hypothesis-testing analysis of AE incidence rates was performed and results were not corrected for multiple comparisons.

### Reporting summary

Further information on research design is available in the Nature Research Reporting Summary linked to this article.

## Supplementary information

Supplementary Information

REPORTING SUMMARY

## Data Availability

Medicago Inc. is committed to providing access to anonymized data collected during the trial that underlie the results reported in this article, at the end of the clinical trial, which is currently scheduled to be 1 year after the last participant is enrolled, unless granted an extension. Medicago Inc. will collaborate with its partners (GSK, Wavre, Belgium) on such requests before disclosure. Proposals should be directed to wardb@medicago.com or daoustma@medicago.com. To gain access, data requestors will need to sign a data access agreement and access will be granted for non-commercial research purposes only. The following publicly available databases were accessed to complete this work: GISAID database (https://www.gisaid.org/) and Genbank (https://www.ncbi.nlm.nih.gov/genbank/).
