# Supplementary material for: Safety and immunogenicity of an AS03-adjuvanted plant-based SARS-CoV-2 vaccine in Adults with and without Comorbidities
Source: NPJ Vaccines. 2022 Nov 9;7:142. doi: 10.1038/s41541-022-00561-2 (PMC9646261; doi:10.1038/s41541-022-00561-2)
Supplement: Supplementary file 1 — Supplementary Information [file 41541_2022_561_MOESM1_ESM.pdf]

SUPPLEMENTARY MATERIAL

For Manuscript "Safety and Immunogenicity of an AS03-Adjuvanted Plant-Based SARS-CoV-2 Vaccine in Adults with and without Comorbidities" by Charland *et al.*

This supplement contains the following items:

- **Supplementary Methods** **Pg 2**
- **Supplementary Table 1**: Details of Comorbidities in the 'Adults with Comorbidities' cohort **Pg 6**
- **Supplementary Table 2:** Summary of Unsolicited Adverse Events up to 21-days after first and second dose in Healthy Adults, Older Adults, and Adults with Comorbidities **Pg 7**
- **Supplementary Table 3:** Summary of Pseudovirion Antibody Neutralization Responses in Healthy Adults, Older Adults, and Adults with Comorbidities **Pg 8**
- **Supplementary Table 4:** Summary of Live Virus Antibody Neutralization Responses in CoVLP+AS03 Vaccinated Healthy Adults, Older Adults, and Adults with Comorbidities Relative to Ancestral Strain and Alpha, Beta, Gamma, Delta, and Omicron (BA.1) Variants **Pg 9**
- **Supplementary Figure 1**: Representative Electron Microscopy Image of CoVLP **Pg 10**
- **Protocol:** Original Protocol, Final Protocol and Summary of Amendments **Pg 11**
- **Statistical Analysis Plan (SAP)**: Original SAP and Final SAP including list of amendments **Pg 604**

Supplementary Methods

SARS-CoV-2 Pseudovirus Neutralization Assay

Neutralizing antibody (NAb) analysis was performed using a cell-based pseudotyped virus neutralization assay (Nexelis). Pseudotyped virus particles were generated using a genetically modified VSV backbone from which the glycoprotein G was removed and luciferase reporter introduced (rVSVΔG-luciferase, Kerafast) to allow quantification using relative luminescence units (RLU). This rVSVΔG-luciferase virus was transduced into HEK293T cells that had previously been transduced with the full-length SARS-CoV-2 S glycoprotein (NXL137-1 in POG2 containing 2019-nCOV Wuhan-Hu-1; GenBank: MN908947) from which the last 19 amino acids of the cytoplasmic tail were removed (rVSVΔG-luciferase-spike ΔCT). Serial dilutions (starting dilution of 1/10, twofold, eight dilutions, in complete growth media) of the heat-inactivated human sera (56 °C for 30 min) were prepared in a 96-well plate in duplicates. The SARS-CoV-2 pseudovirus (in complete growth media) was added to the sera dilutions to reach a target concentration equivalent to approximately 150,000 RLU per well, and the mixture was incubated at 37 °C with 5% CO2 supplementation for 1 h. Serum–pseudovirus complexes were then transferred onto plates previously seeded overnight with Vero E6 cells (ATCC CRL-1586), expressing ACE-2 receptor, and incubated at 37 °C with 5% CO2 supplementation for 20–24 h. Once incubation was completed, cells were lysed, and samples were equilibrated using the ONE-Glo EX Luciferase Assay System (Promega), incubated for 3 min at room temperature, and the luminescence level was read using a luminescence plate reader (i3× plate reader, Molecular Devices). The resulting RLU was inversely proportional to the level of NAbs present in the serum. For each sample, the neutralizing titer was defined as the reciprocal dilution corresponding to the 50% neutralization (NT50) when compared to the pseudoparticle control. The NT50 was interpolated from a linear regression using the two dilutions flanking the NT50. Samples below the cutoff were attributed a value of 5 (half the minimum required dilution). This is a qualified assay (for example, precision, reproducibility and robustness), but its sensitivity for the full spectrum of COVID-19 disease is not currently known. Its precise specificity is also unknown, although it has been evaluated using a panel of well-defined sera (that is, COVID-negative and COVID-positive controls as well as samples from individuals infected with other common human coronaviruses).

SARS-CoV-2 microneutralization cytopathic effect-based assay

NAb analysis was also performed using a cell-based cytopathic effect (CPE) assay as previously described (VisMederi)65. Sera samples were first heat inactivated (56 °C for 30 min) and then serially diluted (starting dilution of 1/10, twofold, eight dilutions, in complete growth media). Wild-type SARS-Co-2 virus (2019 nCOV ITALY/INMI1, provided by EVAg; GenBank: MT066156) was then added at a final concentration of 25 TCID50 per ml (in complete growth media), and plates were incubated for 1 h at 37 °C with 5% CO2 supplementation. At the end of the incubation, the mixture was transferred onto duplicate 96-well microtiter plates pre-seeded overnight with Vero E6 cells (ATCC CRL-1586) expressing ACE-2 receptor to form a uniform monolayer. Plates were then incubated for 3 d at 37 °C with 5% CO2 supplementation. After incubation, each well was read under an inverted optical microscope and evaluated for the presence of CPE in at least 50% of the cells contained in the well. In this assay, there is typically an abrupt 'on–off' transition between no CPE and destruction of virtually the entire monolayer at one higher dilution. The neutralization titer was defined as the reciprocal of the highest sample dilution that protects at least 50% of the cells from CPE (NT50). If no neutralization was observed, samples were attributed a titer value of 5 (half the minimum required dilution). This is a qualified assay (for example, precision, reproducibility and robustness), but its sensitivity is not known for the full spectrum of COVID-19 disease. Its precise specificity is also not known at the current time, although it has been evaluated using a panel of well-defined sera (that is, COVID-negative and COVID-positive controls as well as samples from individuals infected with other common human coronaviruses).

IFN-γ ELISpot

Cell-mediated immune response was evaluated using an IFN-γ ELISpot assay (human IFN-γ ELISpot assay, Cellular Technology Limited). Cryopreserved PBMCs were rapidly thawed and allowed to rest between 2 and 3 h at 37 °C with 5% CO2 supplementation, in CTL-Test media supplemented with 1% glutamine and 1% penicillin–streptomycin. Cells were enumerated and dispensed at 0.5 × 106 cells per well, in duplicates, onto PVDF filter plates pre-coated with an IFN-γ-specific capture antibody. Cells were stimulated using a pool of peptides (15-mer peptides with 11-amino acid overlaps) covering the full sequence of SARS-CoV-2 S protein (USA-CA2/2020, GenBank: MN994468.1, GenScript, purity >90%) at a concentration of 2.19 μg ml−1 for 18–24 h at 37 °C with 5% CO2 supplementation. After washes (PBS-Tween), biotinylated

anti-IFN-γ detection antibody was added to the plates and incubated for 2 h at room temperature, after which, after another round of washes, a streptavidin–alkaline phosphatase conjugate was added and incubated for 30 min at room temperature. After washing steps, substrate solution was added and incubated at room temperature for 15 min, after which the plate was rinsed and left to air dry pending spot enumeration, using an ELISpot reader (ImmunoSpot S6 Universal Analyzer, Cellular Technology Limited). The mean of peptide pool stimulation duplicates was calculated and normalized using the mean of the negative control replicates (control media) and multiplied by a factor of 2 to express cell counts per million cells. Because PBMCs were not available from all participants at Day 0, and because the median value for Day 0 IFN-γ responses across all groups was less than the lower limit of detection of the assay used, the pooled Day 0 results were used to assess vaccine-attributable S protein-specific responses at Day 21 and Day 42.

IL-4 ELISpot

Cell-mediated immune response was evaluated using an IL-4 ELISpot assay (human IL-4 ELISpot assay, Cellular Technology Limited). Cryopreserved PBMCs were rapidly thawed and allowed to rest between 2 and 3 h at 37 °C with 5% CO2 supplementation, in CTL-Test media supplemented with 1% glutamine and 1% penicillin–streptomycin. Cells were enumerated and dispensed at 0.5 × 106 cells per well, in duplicates, onto PVDF filter plates pre-coated with an IL-4-specific capture antibody. Cells were stimulated using a pool of peptides (15-mer peptides) overlapping the full sequence of SARS-CoV-2 S protein (USA-CA2/2020, GenBank: MN994468.1) at a concentration of 2.19 μg ml−1 for 32–48 h at 37 °C with 5% CO2 supplementation. After washes (PBS-Tween), biotinylated anti-IL-4 detection antibody was added to the plates and incubated for 2 h at room temperature, after which, after another round of washes, a streptavidin–alkaline phosphatase conjugate was added and incubated for 30 min at room temperature. After washing steps, substrate solution was added and incubated at room temperature for 15 min, after which the plate was rinsed and left to air dry pending spot enumeration, using an ELISpot reader (ImmunoSpot S6 Universal Analyzer, Cellular Technology Limited). The mean of peptide pool stimulation duplicates was calculated and normalized using the mean of the negative control replicates (control media) and multiplied by a factor of 2 to express cell counts per million cells. Because PBMCs were not available from all participants at Day 0, and because the median value for Day 0 IL-4 responses across all groups was less than the lower limit of detection of the assay used, the pooled Day 0 results were used to assess vaccine-attributable S protein-specific responses at Day 21 and Day 42.

Convalescent serum and plasma samples

Sera from COVID-19 convalescent patients were collected from a total of 35 individuals with confirmed disease diagnosis. Time between the onset of the symptoms and sample collection varied between 27 and 105 d. Three samples were collected by Solomon Park, and 20 samples were collected by Sanguine BioSciences; all were from non-hospitalized individuals. Eleven plasma samples were collected from previously hospitalized patients at McGill University Health Centre. Disease severity was ranked as mild (COVID-19 symptoms without shortness of breath), moderate (shortness of breath reported) and severe (hospitalized). These samples were analyzed in parallel of clinical study samples, using the assays described above.

Supplementary Table 1 | Details of Comorbidities in the 'Adults with Comorbidities' cohort

	Adults with Comorbidities	
	CoVLP 3.75 µg +AS03	Placebo
Participants (N)	138	27
Obesity, n (%)	85 (61.6)	23 (85.2)
Documented Hypertension, n (%)	66 (47.8)	14 (51.9)
Type 1 or Type 2 Diabetes, n (%)	33 (23.9)	8 (29.6)
Cardiovascular Disease, n (%)	28 (20.3)	3 (11.1)
Chronic Obstructive Lung Disease (COPD), n (%)	8 (5.8)	1 (3.7)
Chronic Kidney Disease, n (%)	4 (2.9)	0 (0.0)
Immunocompromised, n (%)	11 (8.0)	1 (3.7)
Other chronic diseases, n (%)	17 (12.3)	1 (3.7)

The 'Immunocompromised' category includes HIV-infected patients, organ transplant recipients and those recovering from cancer chemotherapy. The category of 'other chronic diseases' includes participants with Asthma, Chronic Bronchitis, Crohn's disease, Dyslipidemia, Hyperlipidemia, Hypothyroidism, Osteoporosis, Psoriasis, Pulmonary Fibrosis, Sleep apnea, Smokers, and Ulcerative Colitis.

Supplementary Table 2 | Summary of Unsolicited Adverse Events up to 21-days after first and second dose in Healthy Adults, Older Adults, and Adults with Comorbidities

	**Up to 21-days after First dose**		**Up-to 21-days after Second dose**		**Overall**	
	CoVLP+AS03	Placebo	CoVLP+AS03	Placebo	CoVLP+AS03	Placebo
**Healthy Adults**						
Participants (N)	259	47	253	48	259	47
Unsolicited AEs, n (%)	74 (28.6)	6 (12.8)	66 (26.1)	8 (16.7)	110 (42.5)	12 (25.5)
≥ Grade 3, n (%)	1 (0.4)	0	3 (1.2)	0	4 (1.5)	0
Serious AEs, n (%)	0	0	0	0	0	0
Related Serious AEs, n (%)	0	0	0	0	0	0
Medically Attended AEs, n (%)	7 (2.7)	2 (4.3)	10 (4.0)	2 (4.2)	17 (6.6)	3 (6.4)
Related Medically Attended AEs, n (%)	2 (0.8)	1 (2.1)	1 (0.4)	0	3 (1.2)	1 (2.1)
**Older Adults**						
Participants (N)	235	47	231	44	235	47
Unsolicited AEs, n (%)	71 (30.2)	16 (34.0)	52 (22.5)	11 (25.0)	96 (40.9)	23 (48.9)
≥ Grade 3, n (%)	2 (0.9)	0	1 (0.4)	1 (2.3)	3 (1.3)	1 (2.1)
Serious AEs, n (%)	0	0	0	0	0	0
Related Serious AEs, n (%)	0	0	0	0	0	0
Medically Attended AEs, n (%)	7 (3.0)	1 (2.1)	7 (3.0)	2 (4.5)	14 (6.0)	2 (4.3)
Related Medically Attended AEs, n (%)	0	0	1 (0.4)	0	1 (0.4)	0
**Adults with Comorbidities**						
Participants (N)	138	27	128	26	138	27
Unsolicited AEs, n (%)	9 (6.5)	2 (7.4)	12 (9.4)	2 (7.7)	19 (13.8)	4 (14.8)
≥ Grade 3, n (%)	0	0	0	0	0	0
Serious AEs, n (%)	1 (0.7)	0	0	0	1 (0.7)	0
Related Serious AEs, n (%)	0	0	0	0	0	0
Medically Attended AEs, n (%)	3 (2.2)	0	5 (3.9)	1 (3.8)	8 (5.8)	1 (3.7)
Related Medically Attended AEs, n (%)	0	0	0	0	0	0

N: total number of participants, n: number of participants reporting at least 1 occurrence of the specified event category, AE: adverse event, Grade 3: Severe

Supplementary Table 3 | Summary of Pseudovirion Antibody Neutralization Responses in Healthy Adults, Older Adults, and Adults with Comorbidities

	**Healthy Adults**		**Older Adults**		**Adults with Comorbidities**	
	CoVLP+AS03	Placebo	CoVLP+AS03	Placebo	CoVLP+AS03	Placebo
**NAb Titer**						
Day 0						
GMT (95CI)	5.3 (5.1, 5.6)	5.0 (NE, NE)	5.2 (5.0, 5.5)	5.5 (4.6, 6.6)	8.9 (6.8, 11.6)	13.0 (6.4, 26.2)
Day 21						
GMT (95CI)	44.3 (35.8, 54.8)	5.2 (4.8, 5.6)	29.7 (23.7, 37.1)	5.5 (4.6, 6.5)	49.1 (31.8, 75.7)	20.0 (8.2, 49.0)
Day 42						
GMT (95CI)	2034 (1754, 2358)	5.7 (4.7, 6.8)	1918 (1571, 2342)	5.6 (4.5, 6.9)	1962 (1442, 2667)	36.2 (12.5, 104.7)
**Seroconversion Rate**						
Day 21						
SCR, % (95CI)	51.3 (44.7, 57.8)	0 (0.0, 8.2)	38.4 (31.9, 45.1)	0 (0.0, 7.9)	44.2 (35.1, 53.5)	7.7 (0.9, 25.1)
Day 42						
SCR, % (95CI)	99.2 (97.0, 99.9)	2.4 (0.1, 12.6)	97.7 (94.7, 99.2)	0 (0.0, 9.0)	95.8 (90.4, 98.6)	17.4 (5.0, 38.8)

NAb: neutralizing antibody, GMT: geometric mean titer, 95CI: 95% confidence interval, SCR: seroconversion rate, NE: not estimable

Supplementary Table 4 | Summary of Live Virus Antibody Neutralization Responses in CoVLP+AS03 Vaccinated Healthy Adults, Older Adults, and Adults with Comorbidities Relative to Ancestral Strain and Alpha, Beta, Gamma, Delta, and Omicron (BA.1) Variants

	**Healthy Adults**		**Older Adults**		**Adults with Comorbidities**		**All Vaccinated Participants**	
	GMT (95CI)	% Seropositive	GMT (95CI)	% Seropositive	GMT (95CI)	% Seropositive	GMT (95CI)	% Seropositive
**Day 42**	n=19		n=20		n=18		n=57	
Ancestral	338 (241, 475)	100%	288 (174, 478)	100%	346 (223, 537)	100%	322 (254, 408)	100%
Alpha	413 (292, 585)	100%	343 (188, 627)	100%	470 (276, 803)	100%	403 (305, 533)	100%
Beta	129 (87.7, 189)	100%	62.8 (31.0, 127)	85.0%	132 (71.0, 246)	100%	101 (72.3, 141)	94.7%
Gamma	141 (99.8, 199)	100%	130 (82.5, 205)	100%	140 (88.4, 221)	100%	137 (109, 172)	100%
Delta	214 (142, 323)	100%	197 (113, 345)	100%	214 (128, 358)	100%	208 (158, 273)	100%
Omicron	12.9 (8.4, 19.8)	73.7%	13.0 (8.1, 20.8)	60.0%	13.9 (9.7, 19.9)	83.3%	13.2 (10.5, 16.7)	71.9%
**Day 201**	n=17		n=15		n=13		n=45	
Ancestral	29.5 (18.5, 47.0)	94.1%	22.5 (11.7, 43.2)	80.0%	70.0 (24.1, 203)	92.3%	34.6 (23.1, 51.8)	88.9%
Alpha	28.2 (17.8, 44.9)	94.1%	18.2 (10.1, 32.9)	73.3%	70.0 (22.1, 222)	92.3%	31.8 (20.9, 48.3)	86.7%
Beta	22.6 (12.8, 39.8)	82.4%	13.8 (7.0, 27.3)	60.0%	35.0 (10.3, 120)	100%	21.9 (14.2, 33.8)	80.0%
Gamma	10.0 (7.3, 13.7)	64.7%	11.0 (6.8, 18.8)	66.7%	32.3 (10.3, 101)	92.3%	14.5 (9.9, 21.2)	73.3%
Delta	18.8 (11.7, 30.3)	76.5%	14.8 (8.4, 26.2)	73.3%	40.0 (13.8, 116)	92.3%	21.6 (14.7, 31.9)	80.0%
Omicron	6.5 (5.1, 8.4)	29.4%	6.0 (4.5, 8.0)	13.3%	10.3 (3.3, 31.8)	15.4%	7.2 (5.2, 10.0)	20.0%

n: number of individuals included in analysis, GMT: geometric mean titer, 95CI: 95% confidence interval

Supplementary Figure 1 | Representative Electron Microscopy Image of CoVLP

A negatively stained CoVLP imaged using a Transmission Electron Microscope (JEOL JEM 1010). Scale bar: 100 nm.

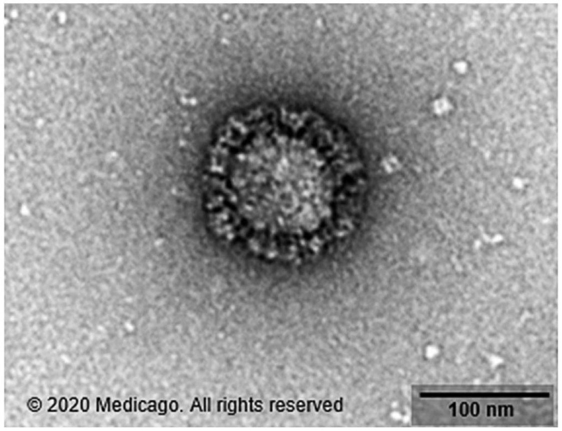

Clinical Protocol

A Randomized, Observer-Blind, Placebo-Controlled, Phase 2/3 Study to Assess the Safety, Efficacy, and Immunogenicity of a Recombinant Coronavirus-Like Particle COVID-19 Vaccine in Adults 18 Years of Age or Older and Children and Adolescents 5-17 Years of Age

CP-PRO-CoVLP-021; Phase 2/3

Coronavirus-Like Particle COVID-19 Vaccine

Name of Sponsor:	Medicago R&D Inc.<br>1020 route de l'Église, bureau 600<br>Québec (Qc), Canada G1V 3V9

Status:	Final version 1.0
Date:	25 September 2020

Confidential Information

Information in this protocol is confidential and should not be disclosed, other than to those directly involved in the execution or the ethical review of the study, without written authorization from Medicago R&D Inc. (herein after known as "Medicago"), and its affiliates. This study will be conducted in accordance with applicable Good Clinical Practice (GCP) guidelines, the United States Code of Federal Regulations (CFR), and International Conference on Harmonization (ICH) guidelines.

SIGNATURES

Study Title:	A Randomized, Observer-Blind, Placebo-Controlled, Phase 2/3 Study to Assess the Safety, Efficacy, and Immunogenicity of a Recombinant Coronavirus-Like Particle COVID-19 Vaccine in Adults 18 Years of Age or Older and Children and Adolescents 5-17 Years of Age
Study Author(s):	[Redacted]

I confirm that Medicago R&D Inc. has approved the version 1.0 (dated 25-Sep-2020) of the protocol CP-PRO-CoVLP-021 and agree that it may be issued to the relevant authorized study personnel, Independent Ethics Committees and Regulatory Authorities.

[Redacted]	Date (YYYY-MM-DD)
[Redacted]	Date (YYYY-MM-DD)
[Redacted]	Date (YYYY-MM-DD)

SIGNATURES (continued)

[Redacted]	Date (YYYY-MM-DD)
[Redacted]	Date (YYYY-MM-DD)
[Redacted]	Date (YYYY-MM-DD)

INVESTIGATOR AGREEMENT

I have read the version 1.0 (dated 25-Sep-2020) Protocol No. CP-PRO-CoVLP-021 titled, " ".

I have fully discussed the objectives of this study and the contents of this protocol with the Sponsor's representative.

I understand that the information in this protocol is confidential and should not be disclosed, other than to those directly involved in the execution or the ethical review of the study, without prior written authorization from Medicago. It is, however, permissible to discuss information contained in this protocol with a subject in order to obtain consent once Institutional Review Board (IRB) approval is obtained.

I agree to conduct this study according to this protocol and to comply with its requirements, subject to ethical and safety considerations and guidelines, and to conduct the study in accordance with International Conference on Harmonisation (ICH) guidelines, Good Clinical Practice (GCP), Investigational New Drug (IND) regulations, and other applicable regulatory requirements.

I understand that the Sponsor may decide to suspend or prematurely terminate this study at any time for whatever reason and that such a decision will be communicated to me in writing. Conversely, should I decide to withdraw from execution of the study, I will communicate my intention immediately in writing to the Sponsor.

Principal Investigator:

Name (typed or printed): ____________________

Institution and Address: ____________________

____

Telephone Number: ____________________

____________________ ____________________
Signature Date (YYYY-MM-DD)

Note: If the address or the telephone number of the Investigator changes during the course of the study, written notification will be provided by the Investigator to the Sponsor, and a protocol amendment will not be required.

TABLE OF CONTENTS

SIGNATURES ... 2
INVESTIGATOR AGREEMENT ... 4
TABLE OF CONTENTS ... 5
LIST OF TABLES ... 10
SYNOPSIS ... 11
ABBREVIATIONS ... 36
1 INTRODUCTION ... 38
1.1 Background ... 38
1.1.1 Elderly Population ... 39
1.1.2 Adults with Comorbidities ... 41
1.1.3 Pediatric Population ... 41
1.2 Background of the Investigational Product ... 42
1.2.1 Background of the Adjuvant AS03 ... 43
1.3 Pre-clinical Studies ... 44
1.4 Clinical Studies ... 44
1.5 Overall Rationale for the Study ... 44
2 OBJECTIVES ... 45
2.1 Primary Objectives ... 45
2.2 Secondary Objectives ... 45
2.3 Exploratory Objectives ... 47
3 STUDY ADMINISTRATIVE STRUCTURE ... 49
4 STUDY DESIGN AND RATIONALE ... 49
4.1 Overview of Study Design ... 49
4.2 Rationale of Study Design ... 51
4.2.1 Randomization ... 51
4.2.2 Blinding ... 51
4.2.3 Dose Selection and Dosage Regimen ... 51
4.2.4 Route of Administration ... 51
4.2.5 Study Duration ... 51
5 STUDY POPULATION ... 52
5.1 Inclusion Criteria ... 52

5.2 Exclusion Criteria 54
5.3 Prior and Concomitant Therapy 56
5.4 Prohibited Therapy 57
6 TREATMENT ALLOCATION AND BLINDING 58
6.1 Randomization 58
6.2 Blinding 58
7 DOSAGE AND ADMINISTRATION 59
8 SPECIMENS AND CLINICAL SUPPLIES 59
8.1 Management of Samples 59
8.2 Collection of Samples 60
8.3 Clinical Supplies 62
9 TREATMENT COMPLIANCE 62
10 STUDY EVALUATIONS 62
10.1 Study Procedures 62
10.1.1 Overview 62
10.1.2 General COVID-19 Precautions at Clinical Sites 62
10.1.3 Screening (Visits 1) 63
10.1.4 Dose 1 Vaccination (Day 0) (Visit 2) 64
10.1.4.1 Prior to Vaccination 64
10.1.4.2 Vaccination 65
10.1.4.3 Thirty Minutes Post-vaccination 65
10.1.5 Day 1 and Day 8 (Telephone Contact) 68
10.1.6 Day 3 Follow-up (Visit 3 for Phase 2 portion only) 68
10.1.7 Day 21 Dose 2 Vaccination (Visit 4 for Phase 2 portion and Visit 3 for Phase 3 portion) 69
10.1.7.1 Pre-vaccination 69
10.1.7.2 Dose 2 Vaccination 70
10.1.7.3 Post-Dose 2 Vaccination 70
10.1.8 Day 22 and Day 29 (Telephone Contact) 71
10.1.9 Day 24 (Visit 5 for Phase 2 portion only) 71
10.1.10 Surveillance for COVID-19 Cases 71
10.1.11 Day 42 (Visit 6 for Phase 2 portion and Visit 4 for Phase 3 portion) 72

10.1.12 Day 201 (Visit 7 for Phase 2 portion and Visit 5 for Phase 3 portion) 72
10.1.13 Final Visit – Day 386 (Visit 8 for Phase 2 portion and Visit 5 for Phase 3 portion).. 73
10.2 Safety 74
10.2.1 Safety Evaluations 74
10.2.1.1 Solicited Local and Systemic Adverse Events 74
10.2.1.2 Adverse Events and SARS-CoV-2 Positive Reports...................................... 75
10.2.1.3 Clinical Laboratory Tests 76
10.2.1.4 Vital Signs 77
10.2.1.5 Physical Examinations... 78
10.2.2 Pregnancy.. 78
10.2.3 Safety Endpoints 78
10.2.3.1 Primary Endpoints 78
10.2.3.2 Secondary Endpoints 79
10.2.3.3 Exploratory Endpoints 79
10.3 Immunogenicity 79
10.3.1 Immunogenicity Evaluations 79
10.3.2 Immunogenicity Endpoints.. 80
10.3.2.1 Primary Endpoints 80
10.3.2.2 Secondary Endpoints 80
10.3.2.3 Exploratory Endpoints 80
10.4 Efficacy 81
10.4.1 Efficacy Evaluations 81
10.4.2 Efficacy Endpoints.. 82
10.4.2.1 Primary Endpoints 82
10.4.2.2 Secondary Endpoints 82
10.4.2.3 Exploratory Endpoint 83
11 SUBJECT COMPLETION/WITHDRAWAL 83
11.1 Temporary Contraindications 83
11.2 Screening Failures.. 83
11.3 Contraindications for Subsequent Vaccination.. 83
11.4 Removal of Subjects from Treatment or Assessment.. 84

11.4.1 Follow-up of Discontinuations 85
11.4.2 Lost to Follow-up Procedures 85
11.5 Interruption of the Study 85
12 STATISTICAL METHODS 86
12.1 Analysis Populations 86
12.1.1 Safety Analysis Set 86
12.1.2 Intention-To-Treat Set 86
12.1.3 Per Protocol Set 86
12.1.4 Immunogenicity Per Protocol Set 86
12.2 Sample Size Determination 87
12.3 Day 21 and Possibly Day 42 Immunogenicity Data Analysis and Interim Efficacy Analyses 88
12.4 Initiation of the Phase 3 Portion 88
12.5 Safety Analyses 89
12.5.1 Analysis of Primary Endpoints 89
12.5.2 Analysis of Secondary Endpoints 89
12.5.3 Analysis of Exploratory Endpoint 90
12.6 Immunogenicity Analyses 90
12.6.1 Analysis of Primary Endpoints 90
12.6.2 Analysis of Secondary Endpoints 91
12.6.3 Analysis of Exploratory Endpoints 92
12.7 Efficacy Analyses 92
12.7.1 Analysis of Primary Endpoint 92
12.7.2 Analysis of Secondary Endpoints 93
12.7.3 Analysis of Exploratory Endpoint 93
12.8 Baseline and Subject Disposition 94
13 ADVERSE EVENT REPORTING 94
13.1 Definitions 94
13.1.1 Serious Adverse Events 94
13.1.2 Adverse Events 95
13.1.2.1 Medically Attended Adverse Events 96
13.1.3 Adverse Events of Special Interest 96

13.1.3.1 Adverse Events of Special Interest for the Coronavirus-Like Particle COVID-19 Vaccine 96
13.1.3.1.1 Vaccine Enhanced Disease 96
13.1.3.1.2 Hypersensitivity Reactions 97
13.1.3.2 Adverse Events of Special Interest for the Adjuvants 97
13.1.3.2.1 Potential Immune-Mediated Diseases and Other AESI as Listed in Section 19.4 97
13.1.4 Expectedness of an Adverse Drug Reaction 98
13.1.5 Initial SAE, AESI, and Pregnancy Reports Reporting by the Investigator 98
13.1.6 Follow-up Reporting by the Investigator 98
13.1.7 Reporting of SAEs Occurring after Study Termination 98
13.1.8 Causal Relationship 99
13.1.9 Reporting of SAEs to Health Authorities and IRB 99
13.1.10 Independent Data Monitoring Committee 100
13.1.11 Safety Review and Stopping Rules 100
14 INVESTIGATIONAL PRODUCT INFORMATION 102
14.1 Identity of Investigational Product 102
14.1.1 Study Vaccine Composition 102
14.1.2 Placebo 102
14.1.3 Preparation and Administration of Study Vaccine 102
14.1.4 Preparation, Handling, Storage, and Precautions for Use 103
14.2 Packaging 103
14.3 Labeling 103
14.4 Drug Accountability 103
15 STUDY-SPECIFIC MATERIALS 104
16 ETHICAL ASPECTS 104
16.1 Study-Specific Design Considerations 104
16.2 Regulatory Ethics Compliance 104
16.2.1 Investigator Responsibilities 104
16.2.2 Independent Ethics Committee or Institutional Review Board 105
16.2.3 Informed Consent 106
16.2.4 Privacy of Personal Data 106

17 ADMINISTRATIVE REQUIREMENTS 106
17.1 Protocol Amendments 106
17.2 Regulatory Documentation 107
17.2.1 Regulatory Approval/Notification 107
17.3 Source Documentation 107
17.3.1 Diary and Memory Aid 108
17.4 Case Report Form Completion 108
17.5 Quality Control (Study Monitoring) 108
17.6 Quality Assurance 109
17.7 Record Retention 109
17.8 Study Completion/Termination 110
17.8.1 Study Completion 110
17.8.2 Study Termination 110
17.9 Registration of Clinical Studies and Disclosure of Results 110
17.10 Publication Policy 110
18 REFERENCES 111
19 APPENDICES 120
19.1 Appendix 1 – Sample Ruler to Measure Local Adverse Events 120
19.2 Appendix 2 – Subject Diary Sample Pages 121
19.3 Appendix 3 – Subject Memory Aid Sample Pages 127
19.4 Appendix 4 – List of Potential Immune-Mediated Diseases 134

LIST OF TABLES

Table 1 Time and Events Schedule: General Information for the Phase 2 Portion 31
Table 2 Time and Events Schedule: General Information for the Phase 3 Portion 33
Table 3 Time and Events Schedule: Onset of COVID-19 35
Table 4 Study Populations 44
Table 5 Study Administrative Structure 49
Table 6 Estimated Blood Volume Drawn: Phase 2 Portion 60
Table 7 Estimated Blood Volume Drawn: Phase 3 Portion 61
Table 8 Severity Grades for Solicited Local and Systemic Adverse Events 75
Table 9 Clinical Laboratory Tests for the Phase 2 Portion of the Study 76

SYNOPSIS

**Sponsor:**	Medicago R&D Inc.
**Investigational Product:**	Coronavirus-Like Particle (CoVLP) COVID-19 Vaccine
**Active Substance(s):**	The Coronavirus-Like Particle COVID-19 Vaccine is composed of recombinant spike (S) glycoprotein expressed as virus-like particles (VLPs). The 3.75 µg dose of CoVLP and the number of doses to be administered in this study (i.e. two doses 21 days apart) as well as the adjuvant (i.e. AS03 manufactured by GlaxoSmithKline) have been determined based on the available Phase 1 study data.
**Protocol Title:**	A Randomized, Observer-Blind, Placebo-Controlled, Phase 2/3 Study to Assess the Safety, Efficacy, and Immunogenicity of a Recombinant Coronavirus-Like Particle COVID-19 Vaccine in Adults 18 Years of Age or Older and Children and Adolescents 5-17 Years of Age
**Protocol Number:**	CP-PRO-CoVLP-021
**Development Phase:**	Phase 2/3
**Study Center(s):**	The study will be conducted at multiple sites in the United States (US) and Canada for the Phase 2 portion while the Phase 3 portion may also incorporate sites in Europe and/or Latin America.
**Study Rationale:**	This Phase 2/3 study is a multi-portion design to confirm that the chosen formulation and dosing regimen of CoVLP (i.e. two doses of 3.75 µg CoVLP adjuvanted with AS03 [hereafter referred to as "CoVLP formulation"] given 21 days apart) has an acceptable immunogenicity and safety profile in adults 18 years of age or older and children and adolescents 5 to 17 years of age (Phase 2 portion). The efficacy of this CoVLP formulation in these same Study Populations will be assessed in the Phase 3 portion.<br><br>The Phase 2 portion is a randomized, observer-blinded, placebo-controlled study to evaluate the CoVLP formulation, compared to placebo, in subjects from the following four Study Populations:

Study Population	Age and/or Health Category	Age Range (years)
1	Healthy adults	18-64

2	Healthy children and adolescents	5-17
3	Elderly adults	≥ 65
4	Adults with significant co-morbidities	≥ 18

In each Study Population, 120 subjects will be randomized 4:1 to receive the CoVLP formulation:placebo. All subjects will be followed for a period of 12 months after the last vaccination for durability of the immune responses, safety, and efficacy.

The Phase 3 portion is an event-driven, randomized, observer-blinded, placebo-controlled design that will evaluate the efficacy and safety of the CoVLP formulation, compared to placebo, in the four Study Populations presented above. Up to 30 000 subjects will be enrolled in a 1:1 ratio to receive the CoVLP formulation:placebo. Immunogenicity will only be evaluated in a subset of 384 subjects.

All subjects will be followed for efficacy until approximately 160 COVID-19 cases have been accumulated in order to evaluate the primary efficacy endpoint of the Phase 3 portion. Subjects will be followed for safety for a period of 12 months after the last vaccination. Persistence of the vaccine-induced immune responses up to 12 months after the last vaccination will be assessed in a subset of subjects.

**Planned Study Period:**	The planned duration of the Phase 2 portion is approximately 14 to 16 months and the Phase 3 portion is approximately 14 months, from screening procedures up to the end of each portion.

Study Objectives

Primary Objectives:

Phase 2 portion

The primary objectives of the Phase 2 portion of the study are:

Safety:

- To assess the safety and tolerability of the CoVLP formulation, compared to placebo, up to 21 days after the last vaccination;

Immunogenicity:

- To assess the immunogenicity of the CoVLP formulation, compared to placebo, up to 21 days after the last vaccination, as determined by:
  - Neutralizing antibody (Nab) titers induced by the vaccine against the SARS-CoV-2 virus;

	• IFN-γ enzyme-linked immuno spot assay (ELISpot) response induced by the vaccine against the SARS-CoV-2 virus to assess the T helper 1 (Th1) type response.<br><br>**Phase 3 portion**<br>The primary objective of the Phase 3 portion of the study is:<br>**Efficacy:**<br>• To evaluate the absolute efficacy of the CoVLP formulation, compared to placebo, in the prevention of laboratory-confirmed symptomatic SARS-CoV-2 infection (virologic method) starting 14 days after the last vaccination.
**Secondary Objectives:**	**Phase 2 portion**<br>The secondary objectives of the Phase 2 portion of the study are:<br>**Immunogenicity:**<br>• To assess the immunogenicity of the CoVLP formulation, compared to placebo, as determined by the:<br>• Neutralizing antibody titers induced by the vaccine against the SARS-CoV-2 virus at Days 201 and Day 386;<br>• IgG (enzyme-linked immunosorbent assay [ELISA]) titers induced by the vaccine against the SARS-CoV-2 virus up to 21 days after the last vaccination and at Day 201 and Day 386;<br>• The ratio of neutralizing antibody titers:IgG (ELISA) antibody titers at Day 21, Day 42, Day 201, and Day 386;<br>• IFN-γ ELISpot response induced by the vaccine against the SARS-CoV-2 virus at Day 201 and Day 386;<br>• Interleukin-4 (IL-4) ELISpot response induced by the vaccine against the SARS-CoV-2 virus up to 21 days after the last vaccination and at Day 201 and Day 386;<br>**Safety:**<br>• To assess the safety and tolerability of the CoVLP formulation, compared to placebo, as determined by the occurrence(s) of:<br>• Medically attended adverse events (MAAEs), serious adverse events (SAEs), AEs leading to

<table>
<tr>
<td></td>
<td>

withdrawal, AEs of special interest (AESIs; including vaccine-enhanced disease [VED]), and deaths from 22 days after the last vaccination up to the end of the study;

Efficacy:

- To evaluate the absolute efficacy of the CoVLP formulation, compared to placebo, in the prevention of laboratory-confirmed symptomatic SARS-CoV-2 infection (virologic method) starting 14 days after the last vaccination;
- To evaluate the absolute efficacy of the CoVLP formulation, compared to placebo, in the prevention of severe COVID-19 disease starting 14 days after the last vaccination. Severe COVID-19 disease will be defined as virologically-confirmed SARS-CoV-2 infection with the occurrence of a new onset of any of the following:
  - Clinical signs at rest indicative of severe systemic illness (respiratory rate ≥ 30 per minute, heart rate ≥ 125 per minute, SpO2 ≤ 93% on room air at sea level or PaO2/FiO2 < 300 mmHg);
  - Respiratory failure (defined as needing high-flow oxygen, non-invasive ventilation, mechanical ventilation or ECMO);
  - Evidence of shock (SBP < 90 mmHg, DBP < 60 mmHg, or requiring vasopressors);
  - Significant acute renal, hepatic, or neurologic dysfunction;
  - Hospitalization;
  - Death.

  The list above applies to the adult populations; for children and adolescents, the following list of occurrences is applicable:

  - Severe pneumonia:
    - Polypnea: ≥ 30 times/min (>5 years) (after ruling out the effects of fever and crying);
    - Oxygen saturation < 92% under a resting state;
    - Dyspnea: assisted breathing (moans, nasal flaring, and three concave sign), cyanosis, intermittent apnea;
    - Disturbance of consciousness: somnolence, coma, or convulsion;

</td>
</tr>
</table>

	- Pulmonary high-resolution CT (HRCT) examination showing bilateral or multi-lobe infiltrates, rapid progression of disease in a short period or with pleural effusion;<br>- Critical cases: those who meet any of the following criteria and require hospitalization:<br>- Respiratory failure requiring mechanical ventilation;<br>- Shock;<br>- Combined with other organs failure;<br>- Death.<br><br>**Phase 3 portion**<br>The secondary objectives of the Phase 3 portion of the study are:<br>**Efficacy:**<br>- To evaluate the absolute efficacy of the CoVLP formulation, compared to placebo, in the prevention of severe COVID-19 disease starting 14 days after the last vaccination (see definition of severe COVID-19 disease above);<br>- To assess COVID-19-related symptoms, and the severity of these symptoms, up until resolution of subjects administered the CoVLP formulation compared to subjects administered the placebo;<br>**Immunogenicity:**<br>- To assess the immunogenicity of the CoVLP formulation, compared to placebo, up to 21 days after the last vaccination, as determined by:<br>- Neutralizing antibody titers induced by the vaccine against the SARS-CoV-2 virus;<br>- IFN-γ ELISpot response induced by the vaccine against the SARS-CoV-2 virus to assess the Th1 type response;<br>**Safety:**<br>- To assess the safety and tolerability of the CoVLP formulation, compared to placebo, up to the end of the study.
**Exploratory Objectives:**	**Phase 2 portion**<br>The exploratory objectives of the Phase 2 portion of the study are:

	**Efficacy:**<br>• To assess COVID-19-related symptoms, and the severity of these symptoms, up until resolution of subjects administered the CoVLP formulation compared to subjects administered the placebo;<br>**Immunogenicity:**<br>• To further characterize the immune response of the CoVLP formulation, if it is likely to be informative;<br>**Safety:**<br>• To further characterize the safety profile of the CoVLP formulation, if it is likely to be informative.<br><br>**Phase 3 portion**<br>The exploratory objectives of the Phase 3 portion of the study are:<br>**Immunogenicity:**<br>• To assess the immunogenicity of the CoVLP formulation, compared to placebo, in a subset of subjects, as determined by the:<br>• Neutralizing antibody titers induced by the vaccine against the SARS-CoV-2 virus at Day 201 and Day 386;<br>• IgG (ELISA) titers induced by the vaccine against the SARS-CoV-2 virus up to 21 days after the last vaccination and at Day 201 and Day 386;<br>• The ratio of neutralizing antibody titers:IgG (ELISA) antibody titers at Day 21, Day 42, Day 201, and Day 386;<br>• IFN-γ ELISpot response induced by the vaccine against the SARS-CoV-2 virus at Day 201 and Day 386;<br>• IL-4 ELISpot response induced by the vaccine against the SARS-CoV-2 virus up to 21 days after the last vaccination and at Day 201 and Day 386;<br>• Specific cell-mediated immune (CMI) response induced by the vaccine against the SARS-CoV-2 virus up to 21 days after the last vaccination and at Day 201 and Day 386, as measured by the percentage of CD4+ T cells expressing functional markers;

	• To further characterize the immune response of the CoVLP formulation in a subset of subjects, if it is likely to be informative.<br>**Safety:**<br>• To further characterize the safety profile of the CoVLP formulation, if it is likely to be informative.
**Number of Planned Subjects:**	Overall, up to a maximum of (are planned for randomization in the Phase 2 and Phase 3 portions in total.<br>**Phase 2 portion**:<br>In the Phase 2 portion of the study, up to 480 subjects will be enrolled in a 4:1 ratio to receive CoVLP formulation:placebo in each Study Population.<br>**Phase 3 portion**:<br>In the Phase 3 portion of the study, up to 30 000 subjects will be enrolled in a 1:1 ratio to receive CoVLP formulation:placebo.<br>Only a subset of approximately 384 subjects (96 subjects from each Study Population) will be included in the immunogenicity evaluation. These subjects will be enrolled from selected sites in Canada and/or the US that will be defined prior to the start of the Phase 3 portion.
**Sample Size Determination:**	**Phase 2 portion:**<br>The sample size of up to 480 subjects will serve to confirm vaccine immunogenicity in the four Study Populations and detect sizable differences in rates of adverse events. The sample size is not large enough to detect less frequent or rare adverse reactions. With 384 subjects receiving the CoVLP formulation, there will be at least 95 % probability of observing an adverse event that occurs as frequently as 1 in 128 vaccinees.<br>**Phase 3 portion:**<br>The sample size of up to 30 000 subjects has been calculated using an exact conditional Poisson approach based on the following assumptions:<br>• A 1:1 ratio for CoVLP formulation:placebo;<br>• At least 90 % statistical power to obtain an observed absolute vaccine efficacy of the CoVLP formulation, compared to placebo, of at least 50 % with an appropriately alpha-adjusted 95 % confidence interval lower limit greater than 30 %;

	• A SARS-CoV-2 attack rate in unvaccinated subjects of 0.8 %;<br>• An attrition of 5 %;<br>• An interim analysis for an early efficacy assessment will be performed when 80 laboratory-confirmed COVID-19 cases have been collected in the Phase 3 portion;<br>• A statistical correction for the interim analysis will be applied that uses the O'Brien-Fleming alpha-spending function. With interim and final assessments at 80 and 160 cases, for example, the two-sided test p-value thresholds will be 0.0027 and 0.0478, respectively.<br>With 15 000 subjects receiving the CoVLP formulation, there will be at least 95 % probability of observing an adverse event that occurs as frequently as 1 in 5000 vaccinees.<br>An immunogenicity subset sample size of 384 subjects is deemed sufficient to provide meaningful statistical assessment of immunologic outcomes.
**Study Population:**	Male and female subjects aged 5 years of age and older from ethnically and racially diverse populations will be included in each of the four Study Populations in the study.
**Dosage and Administration:**	In the Phase 2 and Phase 3 portions of the study, the CoVLP formulation has been selected based on the Phase 1 study data, including acceptable safety and immunogenicity profiles. Subjects in each of the four Study Populations will receive the same CoVLP formulation or placebo.<br>Subjects will receive two intramuscular (IM) injections 21 days apart on Days 0 and 21, into the deltoid region of the alternating arm (each arm will be injected once), of one of the following:<br>• CoVLP formulation;<br>• Placebo.<br>The volume of each injection will be 0.5 mL.
**Study Design:**	**<u>Phase 2 portion</u>:**<br>The Phase 2 portion is a randomized, observer-blinded, placebo-controlled, design with male and female subjects. Subjects will be enrolled from four Study Populations:

Study Population	Age and/or Health Category	Age Range (years)
1	Healthy adults	18-64
2	Healthy children and adolescents	5-17
3	Elderly adults	≥ 65

4	Adults with significant co-morbidities	≥ 18

In each of the four Study Populations, 120 subjects will be randomized 4:1 and will receive the same CoVLP formulation or placebo. Subjects will receive two IM injections 21 days apart on Days 0 and 21, into the deltoid region of the alternating arm (each arm will be injected once), of one of the following:

- CoVLP formulation;
- Placebo.

Adults 18 years of age and older (i.e. Study Populations #1, #3, and #4) will begin enrollment at the same time. Enrollment of elderly adult subjects (Study Population #3) will be stratified by age in a 2:1 ratio of 65 to 74 years of age and 75 years of age and older, respectively. Enrollment of healthy children and adolescents aged 5 to 17 years (i.e. Study Population #2) will begin only after the safety data following the second vaccination for the Healthy Adult population (i.e. Day 28 for all subjects in Study Population #1) have been reviewed by the Independent Data Monitoring Committee (IDMC). Study Population #2 will be stratified by age in a 1:1 ratio of adolescents 12-17 years of age followed by children 5-11 years of age. Enrollment of children will only begin after the IDMC review of safety data following the second vaccination for all adolescent subjects by the IDMC (i.e. Day 28).

The IDMC will review unblinded safety data for the first 10 sentinel subjects after each dose in all Study Populations, with the exception of Study Population #1, according to the details outlined in the IDMC Charter. All sentinel subjects will be dosed at least 30 minutes apart.

Subjects will be screened up to 14 days in advance of the first vaccine administration and will demonstrate a satisfactory baseline medical assessment by history, general physical examination, haematological, biochemical, and serological analysis. Although a test for SARS-CoV-2 antibodies will be performed at screening, both seronegative and seropositive subjects will be enrolled. On Day 0 and Day 21, vaccine administration will occur. Phone contacts will be made one day and eight days after each vaccine administration, specifically for review of the subject's safety and concomitant medication data. Visits to the Investigator site will occur 3 days after each vaccine administration for key safety assessments, and 21 days after each vaccine administration for key safety and immunogenicity assessments. Subjects will return to the

	Investigator site on Day 201 and Day 386 (6-month and 12-month safety follow-ups and immunogenicity assessments after the last vaccine administration).<br><br>Active and passive surveillance will be conducted in the study from 14 days after the last vaccination until 386 days. For passive surveillance, subjects will be instructed to report symptoms meeting the definition of new COVID-19 disease. For active surveillance, subjects will be contacted once per week via the subject's preferred method (e.g. phone, text, email, electronic diary), and asked about symptoms of COVID-19 disease.<br><br>**Phase 3 portion:**<br><br>The Phase 3 portion is a randomized, observer-blinded, placebo-controlled, efficacy, safety, immunogenicity (in a subset of subjects), multicenter design with male and female subjects. Similar to the Phase 2 portion, subjects will be enrolled from four Study Populations (listed above in the Study Rationale section) and each population will be roughly stratified by age in the same manner. The overall goal of the Phase 3 portion of this study is to enroll approximately 30 000 subjects who are generally representative of the overall target population in terms of racial diversity with enrichment for those at highest risk for serious COVID-19 disease (i.e. the elderly and those with co-morbidities) and relatively smaller numbers of the subjects at lowest risk of serious COVID-19 (i.e. children and adolescents).<br><br>In each Study Population, subjects will be randomized into one of two treatment groups:<br>• CoVLP formulation;<br>• Placebo.<br><br>Subjects will be screened up to 14 days in advance of the first vaccine administration and will demonstrate a satisfactory baseline medical assessment by history and general physical examination. Although a test for SARS-CoV-2 antibodies will be performed at screening, both seronegative and seropositive subjects will be enrolled. On Day 0 and Day 21, vaccine administration will occur. Phone contacts will be made one day and eight days after each vaccine administration, specifically for review of the subject's safety and concomitant medication data. Visit to the Investigator site will occur 21 days after each vaccine administration for immunogenicity assessments in the immunogenicity subset only. Subjects will return to the Investigator site on Day 201 and Day 386 (6-month and 12-

	month safety follow-ups and immunogenicity assessments [immunogenicity subset only] after the last vaccine administration).<br><br>Active and passive surveillance will be conducted in the study from 14 days after the last vaccination until 160 COVID-19 cases have been accumulated. For passive surveillance, subjects will be instructed to report symptoms meeting the definition of new COVID-19 disease. For active surveillance, subjects will be contacted once per week via the subject's preferred method (e.g. phone, text, email, electronic diary), and asked about symptoms of COVID-19 disease.
**Blinding:**	Both the Phase 2 and Phase 3 portions of this study will be observer-blind.<br><br>During the entire duration of the study, the following individuals will not have access to treatment allocation (i.e. remain "blind"): the subjects, the Investigators and all personnel involved in the clinical conduct of the study (except the staff involved in the preparation and administration of the study vaccine, the quality assurance auditor, and quality control reviewers), Medicago clinical staff and medical staff involved in safety evaluations (e.g. causality assessments), and all personnel involved in sample analysis at the central and testing (Nab, ELISA, ELISpot, ICS [flow cytometry], serological, and virologic method SARS-CoV-2 diagnostics) laboratories.<br><br>During the study, the IDMC and an unblinded team, compromised of the unblinded medical monitor, unblinded biostatistician, unblinded senior reviewer, and unblinded programmer involved in the preparation of the safety data summary for the IDMC reviews, will have access to or be aware of treatment allocation (i.e. be "unblinded"). The IDMC will review safety data and will make recommendations for the entire duration of the study. The same unblinded statistical team will be involved in the Day 21 and Day 42 (if needed) immunogenicity data analyses in the Phase 2 portion and in the interim efficacy analyses in the Phase 3 portion.<br><br>During the study, the Sponsor's study team will remain blinded.
**Safety Evaluations:**	Safety and tolerability endpoints will include immediate AEs (30 minutes after each vaccination), solicited local and systemic AEs (up to seven days after each vaccination), unsolicited AEs up to 21 days after each vaccination, SAEs, MAAEs, AEs leading to withdrawal, AESIs (including VED), and deaths up to the end of each phase of the study.

**Immunogenicity Evaluations:**	Immunogenicity will be evaluated in all subjects in the Phase 2 portion and in a subset of 384 subjects in the Phase 3 portion of the study across the four Study Populations with age distribution similar to the Phase 2 portion. Immunologic outcomes will include the humoral immune response (neutralization assay and IgG ELISA) and the CMI response induced in subjects on Day 0, Day 21, Day 42, Day 201, and Day 386.
**Efficacy Evaluations:**	Following the first vaccination, subjects will be asked to report symptoms associated with COVID-19 from 14 days after the last vaccination until the end of the Phase 2 portion or until the required number of COVID-19 cases have been collected in order to evaluate the primary efficacy endpoint in the Phase 3 portion of the study (through active or passive surveillance). COVID-19 will be defined as the presence of a laboratory-confirmed (virologic method) SARS-CoV-2 infection with the occurrence of a new onset of one or more of the following symptoms that persist(s) for or reoccur(s) after a period of at least 12 hours:<br>• Fever or chills;<br>• Cough;<br>• Shortness of breath or difficulty breathing;<br>• Fatigue;<br>• Muscle or body aches;<br>• Headache;<br>• New loss of taste or smell;<br>• Sore throat;<br>• Congestion or runny nose;<br>• Nausea or vomiting;<br>• Diarrhea.<br>The symptoms listed above apply to the adult populations, and will also apply to children and adolescents with the following additions:<br>Additional symptoms:<br>• Abdominal pain;<br>• Poor appetite.<br>Subjects who report any symptoms associated with COVID-19 after the start of active and passive surveillance will be requested to visit the Investigator site to provide nasal or nasopharyngeal (NP) swabs (2 per subject per event) to test for COVID-19.

	The swabs will be collected within 72 hours (preferably within 48 hours) of the reporting of a COVID-19-associated symptom. If swabs cannot be collected within 72 hours after reporting of the COVID-19-associated symptom, nasal or NP swabs should be collected at the next available opportunity for the subject to visit the Investigator site. One of the swabs will be submitted for analysis using a virologic method while the other swab will be kept as a back-up. All subjects with virologically-confirmed SARS-COV-2 infection will be asked to keep a detailed daily record of their symptoms and provide serial (every other day) self-administered nasal/NP/buccal swabs for testing, using a virologic method, until symptoms resolution.
**Primary Endpoint:**	**Phase 2 portion**<br>In the Phase 2 portion, the primary endpoints are:<br>**Safety:**<br>• Occurrence, intensity, and relationship to vaccination of immediate AEs (30 minutes after each vaccination);<br>• Occurrence, intensity, and relationship to vaccination of solicited local and systemic AEs (for seven days following each vaccine administration);<br>• Occurrence, intensity, and relationship of unsolicited AEs for 21 days following each vaccine administration;<br>• Number and percentage of subjects with normal and abnormal clinically significant urine, haematological and biochemical values prior to and three days following each vaccination;<br>• Occurrences of SAEs, MAAEs, AEs leading to withdrawal, AESIs (including VED), and deaths up to 21 days following each vaccine administration;<br>**Immunogenicity:**<br>• Nab response induced in each Study Population against the SARS-CoV-2 virus on Days 0, 21, and 42 will be analyzed using the following parameter: geometric mean titers (GMT), seroconversion (SC) rate, and geometric mean fold rise (GMFR);<br>• Specific Th1 CMI response induced in each Study Population against the SARS-CoV-2 virus on Days 0, 21, and 42, as measured by IFN-γ ELISpot.

	**Phase 3 portion**<br>In the Phase 3 portion, the primary endpoints are:<br>**Efficacy:**<br>• First occurrence, in a subject, of laboratory-confirmed (virologic method) symptomatic SARS-CoV-2 infection (≥ 14 days post-last vaccination).
**Secondary Endpoints:**	**Phase 2 portion**<br>In the Phase 2 portion, the secondary endpoints are:<br>**Immunogenicity:**<br>• Nab antibody response induced in each Study Population against the SARS-CoV-2 virus on Day 201 and Day 386 will be analyzed using the following parameters: GMT, SC rate, and GMFR;<br>• Specific antibody response induced in each Study Population against the SARS-CoV-2 virus will be measured by the total IgG levels on Days 0, 21, 42, 201, and 386 and analyzed using the following parameters: GMT, SC rate, and GMFR;<br>• The ratio of neutralizing antibody titers: IgG (ELISA) antibody titers at Day 21, Day 42, Day 201, and Day 386;<br>• Specific Th1 CMI response induced in each Study Population against the SARS-CoV-2 virus on Day 201 and Day 386, as measured by IFN-γ ELISpot;<br>• Specific T helper 2 (Th2) CMI response induced in each Study Population against the SARS-CoV-2 virus on Days 0, 21, 42, 201, and 386, as measured by IL-4 ELISpot;<br>**Safety:**<br>• Occurrences of SAEs, MAAEs, AEs leading to withdrawal, AESIs (including VED), and deaths from Day 43 to Day 201;<br>• Occurrences of SAEs, MAAEs, AEs leading to withdrawal, AESIs (including VED), and deaths from Day 202 to Day 386.<br>**Efficacy:**<br>• First occurrence, in a subject, of laboratory-confirmed (virologic method) symptomatic SARS-CoV-2 infection (≥ 14 days post-last vaccination);

	• Occurrences of severe COVID-19 disease (≥ 14 days post-last vaccination). Refer to the definition of severe COVID-19 disease in the Secondary Objectives section above.<br><br>**Phase 3 portion**<br>In the Phase 3 portion, the secondary endpoints are:<br>**Efficacy:**<br>• Occurrences of severe COVID-19 disease (≥ 14 days post-last vaccination). Refer to the definition of severe COVID-19 disease in the Secondary Objectives section above;<br>• Occurrence and intensity of COVID-19-related symptoms up to resolution of the symptoms;<br>**Immunogenicity:**<br>• Nab response induced in each Study Population against the SARS-CoV-2 virus on Days 0, 21, and 42 will be analyzed using the following parameter: GMT, SC rate, and GMFR;<br>• Specific Th1 CMI response induced in each Study Population against the SARS-CoV-2 virus on Days 0, 21, and 42, as measured by IFN-γ ELISpot.<br>**Safety:**<br>• Occurrence, intensity, and relationship to vaccination of immediate AEs (30 minutes after each vaccination);<br>• Occurrence, intensity, and relationship to vaccination of solicited local and systemic AEs (for seven days following each vaccine administration);<br>• Occurrence, intensity, and relationship of unsolicited AEs for 21 days following each vaccine administration;<br>• Occurrences of SAEs, MAAEs, AEs leading to withdrawal, AESIs (including VED), and deaths up to 21 days following each vaccine administration;<br>• Occurrences of SAEs, MAAEs, AEs leading to withdrawal, AESIs (including VED, and deaths from Day 43 to Day 201;<br>• Occurrences of SAEs, MAAEs, AEs leading to withdrawal, AESIs (including VED), and deaths from Day 202 to Day 386.

**Exploratory Endpoints:**	**Phase 2 portion**<br>In the Phase 2 portion, the exploratory endpoints are:<br>**Efficacy:**<br>• Occurrence and intensity of COVID-19-related symptoms up to resolution of the symptoms;<br>**Immunogenicity:**<br>• Further characterization of the immune response of the CoVLP formulation, if it is likely to be informative;<br>**Safety:**<br>• Further characterization of the safety profile of the CoVLP formulation, if it is likely to be informative.<br><br>**Phase 3 portion**<br>In the Phase 3 portion, the exploratory endpoints are:<br>**Immunogenicity:**<br>• Nab antibody response induced in each Study Population against the SARS-CoV-2 virus on Days 201 and 386 analyzed, in a subset of subjects, using the following parameters: GMT, SC rate, and GMFR;<br>• Specific antibody response induced in each Study Population against the SARS-CoV-2 virus measured by the total IgG levels on Days 0, 21, 42, 201, and 386, in a subset of subjects, and analyzed using the following parameters: GMT, SC rate, and GMFR;<br>• The ratio of neutralizing antibody titers:IgG (ELISA) antibody titers at Day 21, Day 42, Day 201, and Day 386 in a subset of subjects;<br>• Specific Th1 CMI response induced in each Study Population against the SARS-CoV-2 virus on Days 201 and 386, as measured by IFN-γ ELISpot in a subset of subjects;<br>• Specific Th2 CMI response induced in each Study Population against the SARS-CoV-2 virus on Days 0, 21, 42, 201, and 386, as measured by IL-4 ELISpot in a subset of subjects;<br>• Specific CMI response induced in each Study Population against the SARS-CoV-2 virus up to 21 days after the last vaccination and at Day 201 and Day 386,

	as measured by the percentage of CD4+ T cells expressing functional markers in a subset of subjects;<br>• Further characterization of the immune response of the CoVLP formulation in a subset of subjects, if it is likely to be informative;<br>**Safety:**<br>• Further characterization of the safety profile of the CoVLP formulation, if it is likely to be informative.
**Safety Review:**	During the study, the IDMC will review safety data to ensure the ongoing safety of all the subjects in the study. Information about the planned and ad hoc IDMC review meetings is available in the IDMC charter.<br>During the Phase 2 portion, the IDMC will review the safety data collected up to Day 28 (last subject) from all the subjects in each Study Population to confirm that each Study Population can be included in the Phase 3 portion of the study.
**Statistical Methods:**	**Populations:**<br>Descriptive statistical analyses will be performed on pre-defined population sets (the safety analysis set [SAS], the Intention-to-Treat [ITT], the per protocol [PP] set, and the immunogenicity per protocol [IPP] set) for each Study Population in the Phase 2 and Phase 3 portions, based on what will be provided in the Statistical Analysis Plan (SAP).<br>In each of the Study Populations for the Phase 2 and Phase 3 portions:<br>• All safety analyses will be performed using the SAS;<br>• All efficacy analyses will be performed using both the PP set and the ITT set;<br>• All immunogenicity analyses will be performed using both the IPP set and the immunogenicity ITT set;<br>• Analyses using the PP set and IPP set will be considered the primary analyses for these objectives.
	**Phase 2 portion: Day 21 and possibly Day 42 Immunogenicity Data Analysis:**<br>The unblinded statistical team will perform immunogenicity data analysis after Day 21 (last subject) in each of the Study Populations. The data analysis will involve selected individuals from the unblinded statistical team and Medicago who will be unblinded (as described in Blinding section above). This analysis will confirm that an acceptable immune response has

	been induced in each Study Population prior to the initiation of the Phase 3 portion of the study, without having to wait until after the end of the 386 day follow-up period for study completion. In the event that the Day 21 NAb response in any of the Study Populations is <50 % of that achieved by healthy adults, then a similar data analysis will be performed after Day 42 (last subject) for the Study Population(s) with the lower Nab response in order to initiate the Phase 3 portion of the study for the particular Study Population. However, if at Day 42 the Nab response for a Study Population is still <50 % of that achieved by healthy adults, an ad hoc IDMC meeting will be convened to consider changes to the Protocol to permit alternate vaccination strategies in the implicated Study Population(s).<br>**Phase 3 portion: Interim Efficacy Analyses:**<br>When 80 laboratory-confirmed COVID-19 cases have been collected, the unblinded statistical team will perform a first interim analysis for the Phase 3 portion of the efficacy data in order to test for early vaccine efficacy. A second interim analysis for the Phase 3 portion will be performed as soon as 160 cases are collected in order to perform the final analysis and produce final efficacy outputs.<br>**Phase 3 Portion Initiation:**<br>The start of the Phase 3 portion of the study will be determined independently for each Study Population and will be based on the outcomes of the following two assessments during the Phase 2 portion of the study:<br>• Day 21 Data Analysis of the immunogenicity data (described above);<br>• Day 28 Safety Review (described in the Safety Review section above).<br>If the Day 21 immunogenicity data and Day 28 safety data results are favorable and show acceptable safety and immune responses for Study Populations #3 and #4, assuming the results in Study Population #1 will be favorable, then the Phase 3 portion of the study can start for all three Study Populations.<br>As mentioned above, if the Day 21 immune response is not acceptable in one or both of Study Populations #3 and #4, then the Day 42 immunogenicity data will be assessed in the applicable Study Population(s) for the purposes of determining

	whether the Phase 3 portion may be started for that/those Study Population(s).<br>**Statistical Analyses:**<br>All descriptive and inferential statistical analyses will be performed using Statistical Analysis System® (SAS®) software (version 9.4 or higher).<br>In general, categorical data will be summarized using the number and percent of subjects in each category and continuous data will be summarized using descriptive statistics (mean or geometric mean, median, standard deviation, minimum, and maximum).<br>Analyses of primary, secondary, and exploratory endpoints will include each Study Population as a whole as well as comparisons by age strata.<br>**Safety Analyses:**<br>Safety and tolerability endpoints will be summarized by treatment using descriptive statistics.<br>**Immunogenicity Analyses:**<br>For the immunogenicity analyses, point estimates and two-sided 95 % confidence interval (CI) for all immunogenicity endpoints and responses for the treatment groups will be calculated. GMT will be compared among treatment groups by using Analysis of Variance (ANOVA) on log-transformed data. GMFR will be derived by using Analysis of Covariance (ANCOVA) to model the difference in the log of the titer values between Day 21/42/201/386 and Day 0, with treatment group as main effect and baseline titer as covariate. For GMT and GMFR comparisons, Tukey's range test will be performed to show p-values for the pairwise comparisons between treatment groups. Fisher's exact tests will be used to compare SC among treatment groups. The CMI response will be compared among treatment groups using appropriate non-parametric (Wilcoxon) models.<br>**Efficacy Analyses:**<br>Once approximately 160 COVID-19 cases have accumulated during the study, the primary efficacy endpoint of the Phase 3 portion of the study will be evaluated to determine if the vaccine efficacy success criterion has been met. For the efficacy endpoints, absolute vaccine efficacy (VE) will be evaluated. An interim VE analysis will be performed when 80 cases have been collected for an early efficacy assessment.

	Absolute VE will be evaluated as the relative risk of CoVLP vaccinated versus unvaccinated subjects to develop laboratory-confirmed symptomatic SARS-CoV-2 infection ≥ 14 days after the last vaccination, as per the following formula:<br>Absolute VE=(1-RR)=(1-ARV/ARU)*100 %<br>Where:<br>RR= relative risk<br>ARV = attack rate in subjects vaccinated with CoVLP; and<br>ARU = attack rate in unvaccinated subjects.<br>The absolute VE success criterion for the primary efficacy endpoint, in the Phase 3 portion, is defined as a ≥ 50 % point estimate and a > 30 % lower limit of the appropriately alpha-adjusted confidence interval that maintains overall Type I error at 5 %. Statistical significance of the primary efficacy endpoint can be achieved at the interim analysis or at the primary analysis.<br>Absolute VE will also be calculated for the secondary and exploratory efficacy endpoints. The absolute VE success criterion for the secondary efficacy endpoints, in the Phase 3 portion, is defined as > 0 % lower limit of the appropriately alpha-adjusted confidence interval that maintains overall Type I error at 5 %.<br>The CI around RR will be calculated based on score confidence limits.

Table 1 Time and Events Schedule: General Information for the Phase 2 Portion

**Visit Type**	**Screening**	**Vaccination**	**Post-vaccination Visits/Contacts**									
**Study Day**	**Day -14 to 0**	**Day 0**	**Day 1 (+ 1)**	**Day 3 (± 1)**	**Day 8 (± 1)**	**Day 21 (± 2) [8]**	**Day 22 (+ 1)**	**Day 24 (± 1)**	**Day 29 (± 1)**	**Day 42 (± 2)**	**Day 201 (± 14)**	**Day 386 (± 14)**
**Visit Number**	**1**	**2**	**Phone**	**3**	**Phone**	**4**	**Phone**	**5**	**Phone**	**6**	**7**	**8**
Informed consent	X											
Demographics	X											
Height, weight, and BMI	X	X										
Medical history/prior medication	X	X[1]										
History/symptom-directed physical examination	X[2]											
Vital Signs	X	X[3]		X		X[3]		X		X	X	X
Inclusion/exclusion criteria	X	X										
Urinalysis	X			X		X[9]		X				
Pregnancy test [4]	X	X[9]				X[9]				X		
Test for SARS-CoV-2 antibodies	X											
Serology tests for HIV, Hepatitis B, and Hepatitis C	X											
Blood chemistry And Haematology	X			X		X[9]		X				
Immunogenicity - Serology (Nab assay and ELISA)		X[9]				X[9]				X	X	X
Immunogenicity – CMI Response (ELISpot and ICS)		X[9]				X[9]				X	X	X
Randomization		X										
Vaccine admin		X				X						
Immediate surveillance (30 minutes)		X				X						
Provide and collect diary and memory aid instructions		X		X		X		X		X	X	X
Oral digital thermometer and instructions on reactions[5]		X				X						
Collection of solicited local/ systemic adverse events		X	X	X	X	X	X	X	X			
Concomitant medications[6]		At any time during the study period										

**Visit Type**	**Screening**	**Vaccination**	**Post-vaccination Visits/Contacts**									
**Study Day**	**Day -14 to 0**	**Day 0**	**Day 1 (+ 1)**	**Day 3 (± 1)**	**Day 8 (± 1)**	**Day 21 (± 2)[8]**	**Day 22 (+ 1)**	**Day 24 (± 1)**	**Day 29 (± 1)**	**Day 42 (± 2)**	**Day 201 (± 14)**	**Day 386 (± 14)**
**Visit Number**	**1**	**2**	**Phone**	**3**	**Phone**	**4**	**Phone**	**5**	**Phone**	**6**	**7**	**8**
Collection of COVID-19 symptoms through passive and active surveillance		Passive Surveillance: Subjects (and/or their parent/legally acceptable representative [LAR]) will be instructed to contact the study site if they experience symptoms of COVID-19 from 14 days after the last vaccination until the end of the Phase 2 or until 160 COVID-19 cases have been accumulated in the Phase 3 portion of the study.<br>Active Surveillance: Between 14 days after the last vaccination and the end of the Phase 2 or until 160 COVID-19 cases have been accumulated in the Phase 3 portion of the study, the subjects will be contacted once per week via the method most likely to be successful (e.g. subject's (and/or their parent/LAR's) preferred method [phone, text, email, electronic diary]), and asked about symptoms of COVID-19.										
Testing for laboratory confirmation of SARS-CoV-2 infection		Subjects who report any symptoms associated with COVID-19 after the start of the active and passive surveillance will be requested to visit the Investigator site to provide nasal or NP swabs (2 per subject per event) to test for COVID-19.The swabs will be collected within 72 hours (preferably within 48 hours) of the reporting of a COVID-19-associated symptom. If swabs cannot be collected within 72 hours after reporting of the COVID-19-associated symptom, nasal or NP swabs should be collected at the next available opportunity for the subject to visit the Investigator site.										
AEs, SAEs, MAAEs, and AESIs[7]		At any time during the study period.										
Termination record												X

[1] Record any changes in medical history and medications and confirmation that the subject continues to meet all inclusion and no exclusion criteria since screening.
[2] History/symptom-directed physical examinations will not be routinely performed at any other visits, unless new complaints or concerns are raised by either the study subject or study staff, and if deemed to be necessary by the Investigator.
[3] Record prior to study vaccine, after the observation period and as deemed necessary.
[4] In all females of childbearing potential in Study Populations #1, #2, and #4; it will be tested in serum at screening and in urine at Days 0, 21, and 42.
[5] After vaccination, subjects (and/or their parent/LAR) will be instructed on the diary and memory aid provided for their use for recording reactions, AEs, and concomitant medication use.
[6] After the Day 42 visit, concomitant medication collection will be limited to those used to treat an SAE, MAAE, AE leading to withdrawal, AESIs, or an AE that occurred before Day 42; any vaccine not foreseen in the study protocol; and prohibited medications.
[7] AEs will be collected up to Day 42; SAEs, MAAEs, AEs leading to withdrawal, and AESIs will be collected through to the end of the study. Investigators should report SAEs, MAAEs, AESIs, and pregnancy reports to Sponsor within 24 hours of the Investigator learning of the event. Specific contacts for the collection of information regarding all of these events will occur on Day 386 (during the Day 386 final visit) for SAEs, MAAEs, AEs leading to withdrawal, and AESI.
[8] All efforts will be done to have subjects returning on planned date for Day 21 activities. If for any reason the visit is done before or after this planned date, subsequent visits/procedures (Days 22, 24, 29, 42, 201, and 386) will be adjusted accordingly.
[9] Blood and urine samples are to be collected prior to vaccination on the respective day.

Table 2 Time and Events Schedule: General Information for the Phase 3 Portion

**Visit Type**	**Screening**	**Vaccination**	**Post-vaccination Visits/Contacts**							
**Study Day**	**Day -14 to 0**	**Day 0**	**Day 1 (+ 1)**	**Day 8 (± 1)**	**Day 21 (± 2)** [8]	**Day 22 (+ 1)**	**Day 29 (± 1)**	**Day 42 (± 2)**	**Day 201 (± 14)**	**Day 386 (± 14)**
**Visit Number**	**1**	**2**	**Phone**	**Phone**	**3**	**Phone**	**Phone**	**4**	**5**	**6**
Informed consent	X									
Demographics	X									
Height, weight, and BMI	X	X								
Medical history/prior medication	X	X[1]								
History/symptom-directed physical examination	X[2]									
Vital Signs	X	X[3]			X[3]			X	X	X
Inclusion/exclusion criteria	X	X								
Pregnancy test [4]	X	X[9]			X[9]			X		
Test for SARS-CoV-2 antibodies	X									
Immunogenicity - Serology (Nab assay and ELISA)[10]		X[9]			X[9]			X	X	X
Immunogenicity – CMI Response (ELISpot and ICS)[10]		X[9]			X[9]			X	X	X
Randomization		X								
Vaccine admin		X			X					
Immediate surveillance (30 minutes)		X			X					
Provide and collect diary and memory aid instructions		X			X			X	X	X
Oral digital thermometer and instructions on reactions[5]		X			X					
Collection of solicited local/ systemic adverse events		X	X	X	X	X	X			
Concomitant medications[6]		At any time during the study period								
Collection of COVID-19 symptoms through passive and active surveillance		Passive Surveillance: Subjects (and/or their parent/LAR) will be instructed to contact the study site if they experience symptoms of COVID-19 from 14 days after the last vaccination until the end of the Phase 2 or until 160 COVID-19 cases have been accumulated in the Phase 3 portion of the study.<br>Active Surveillance: Between 14 days after the last vaccination and the end of the Phase 2 or until 160 COVID-19 cases have been accumulated in the Phase 3 portion of the study, the subjects will be contacted once per week via the method most likely to be successful (e.g.								

Visit Type	Screening	Vaccination	Post-vaccination Visits/Contacts							
**Study Day**	**Day -14 to 0**	**Day 0**	**Day 1 (+ 1)**	**Day 8 (± 1)**	**Day 21 (± 2)** [8]	**Day 22 (+ 1)**	**Day 29 (± 1)**	**Day 42 (± 2)**	**Day 201 (± 14)**	**Day 386 (± 14)**
**Visit Number**	**1**	**2**	**Phone**	**Phone**	**3**	**Phone**	**Phone**	**4**	**5**	**6**
		subject's (and/or their parent/LAR's)preferred method [phone, text, email, electronic diary]), and asked about symptoms of COVID-19.								
Testing for laboratory confirmation of SARS-CoV-2 infection		Subjects who report any symptoms associated with COVID-19 after the start of the active and passive surveillance will be requested to visit the Investigator site to provide nasal or NP swabs (2 per subject per event) to test for COVID-19. The swabs will be collected within 72 hours (preferably within 48 hours) of the reporting of a COVID-19-associated symptom. If swabs cannot be collected within 72 hours after reporting of the COVID-19-associated symptom, nasal or NP swabs should be collected at the next available opportunity for the subject to visit the Investigator site.								
AEs, SAEs, MAAEs, and AESIs[7]		At any time during the study period.								
Termination record										X

[1] Record any changes in medical history and medications and confirmation that the subject continues to meet all inclusion and no exclusion criteria since screening.

[2] History/symptom-directed physical examinations will not be routinely performed at any other visits, unless new complaints or concerns are raised by either the study subject or study staff, and if deemed to be necessary by the Investigator.

[3] Record prior to study vaccine, after the observation period and as deemed necessary.

[4] In all females of childbearing potential in Study Populations #1, #2, and #4; it will be tested in urine at screening and at Days 0, 21, and 42. Study Population #1: in the Phase 3 portion of the study, any female subject who is in the 1st or 2nd trimester of her pregnancy cannot participate in the study. However, any female subject who is in the 3rd trimester of her low-risk pregnancy can participate in the study.

[5] After vaccination, subjects (and/or their parent/LAR) will be instructed on the diary and memory aid provided for their use for recording reactions, AEs, and concomitant medication use.

[6] After the Day 42 visit, concomitant medication collection will be limited to those used to treat an SAE, MAAE, AE leading to withdrawal, AESIs, or an AE that occurred before Day 42; any vaccine not foreseen in the study protocol; and prohibited medications.

[7] AEs will be collected up to Day 42; SAEs, MAAEs, AEs leading to withdrawal, and AESIs will be collected through to the end of the study. Investigators should report SAEs, MAAEs, AESIs, and pregnancy reports to Sponsor within 24 hours of the Investigator learning of the event. Specific contacts for the collection of information regarding all of these events will occur on Day 386 (during the Day 386 final visit) for SAEs, MAAEs, AEs leading to withdrawal, and AESI.

[8] All efforts will be done to have subjects returning on planned date for Day 21 activities. If for any reason the visit is done before or after this planned date, subsequent visits/procedures (Days 22, 29, 42, 201, and 386) will be adjusted accordingly.

[9] Blood and urine samples are to be collected prior to vaccination on the respective day.

[10] These samples will only be collected from a subset of subjects.

Table 3 Time and Events Schedule: Onset of COVID-19

Days after Reporting of COVID-19[3]:	0-3 Days	0-3 Days[1]	Monitoring[2]
**Contact Type**	**Phone**	**Visit**	**Phone / Text / Email Messaging / Electronic Diary**
Verify information on COVID-19 and confirm swab collection within 72 hours (preferably within 48 hours) of the reporting of a COVID-19 symptom	X		
Remind subject to continue to record data and in a timely manner	X		
Collection of information on COVID-19 symptoms	X	X	X

[1] Nasal or NP swab collection is to be done within 72 hours after reporting of the COVID-19 symptoms (preferably within 48 hours) and prior to the use of any antiviral treatment medication. If swab collection cannot be done within 72 hours after reporting of the COVID-19 symptoms, swabs should be collected at the next available opportunity for the subject to visit the Investigator site and date and time of collection recorded.
[2] After swab collection is done, the planned active surveillance (phone, text, email, electronic diary) will continue and will include questions on the progression of COVID-19.
[3] Swab collection will be done for any subject who reports any COVID-19-associated symptom after the start of the active and passive surveillance.

ABBREVIATIONS

AE	adverse event
AESI	adverse event of special interest
ANOVA	analysis of variance
BMI	body mass index
BP	blood pressure
CBER	Center for Biologics Evaluation and Research
CI	confidence interval
CMI	cell-mediated immune (response)
eCRF	electronic case report form
ELISA	enzyme-linked immunosorbent assay
ELISpot	enzyme-linked immuno spot assay
FDA	Food and Drug Administration
GCP	good clinical practice
GMFR	geometric mean fold rise or seroconversion factor
GMT	geometric mean titer
HR	heart rate
IB	Investigator's Brochure
ICF	informed consent form
ICH	International Council for Harmonisation
IDMC	Independent Data Monitoring Committee
IEC	independent ethics committee
IFN-γ	interferon gamma
IM	intramuscular
IRB	institutional review board
IRT	interactive response technology
ITT	intention-to-treat
MedDRA	Medical Dictionary for Regulatory Activities
Nab	neutralizing antibody
OR	odds ratio
OT	oral temperature
PBMC	peripheral blood mononuclear cell
pIMD	potential immune-mediated disease
PP	per protocol
RR	respiratory rate
SAE	serious adverse event
SAP	statistical analysis plan
SAS	safety analysis set
SAS®	Statistical Analysis System®

SC	seroconversion
VED	vaccine-enhanced disease
VLP	virus-like particle
WHO	World Health Organization
US	United States

1 INTRODUCTION

1.1 Background

A cluster of pneumonia cases of unknown aetiology was identified in the city of Wuhan in Hubei province of China in December 2019 [Zhu 2020]. The clinical manifestations included fever, cough, and fatigue, while other symptoms include sputum production, headache, haemoptysis, diarrhoea, dyspnoea, and lymphopenia [Lake 2020]. Clinical features revealed by a chest computed tomography (CT) scan presented as pneumonia, however, there were abnormal features such as acute respiratory distress syndrome, acute cardiac injury, and incidence of multiple organ failure that led to death in some cases [Chen 2020]. The symptoms of the disease were more severe in older age groups with comorbidities, while hypertension, type 2 diabetes, asthma and chronic obstructive pulmonary disease (COPD) were also identified as risk factors [Liu 2020a, Yang 2020]. A novel coronavirus, Severe Acute Respiratory Syndrome Coronavirus 2 (SARS-CoV-2), formerly known as the 2019 novel Coronavirus (2019-nCoV), was identified as the agent that caused the pneumonia outbreak, and the disease was subsequently named 'coronavirus disease 2019', or COVID-19 [Guan 2020, Zhu 2020]. The rapidly evolving situation with SARS-CoV-2 infection in China and spread of the disease across many countries prompted the World Health Organization (WHO) to declare a pandemic in March 2020 [WHO 2020].

Coronaviruses are frequent causes of respiratory infections where six major species are known to cause human infections besides the SARS-CoV-2. Previous outbreaks of coronavirus infections include the severe acute respiratory syndrome (SARS) and the Middle East respiratory syndrome (MERS), which have been characterized as a great public health threat [Liu 2020]. In 2002–2003, severe acute respiratory syndrome coronavirus (SARS-CoV) emerged from bat and palm civet and infected over 8 000 people and caused about 800 deaths [Cheng 2007]. In 2012, Middle East respiratory syndrome coronavirus (MERS-CoV) was discovered as the causative agent of a severe respiratory syndrome in Saudi Arabia, with nearly 2 500 confirmed cases and 858 deaths. It remains endemic in Middle East, and dromedary camel is thought to be the zoonotic reservoir host of MERS-CoV [Memish 2020]. Less virulent coronavirus species include NL63, 229E, OC43 and HKU14 that account for 10 to 30 % of common cold cases, with only occasional spreading to the lower respiratory tract [Paules 2020, Su 2016].

Coronaviruses are enveloped positive-sense RNA viruses ranging from 60 nm to 140 nm in diameter with spike-like projections on its surface giving it a crown-like appearance under the electron microscope. SARS-CoV-2 particles consist of a helical nucleocapsid structure, formed by the association between nucleocapsid (N) phosphoproteins and the viral genomic RNA, which is surrounded by a lipid bilayer where three types of structural proteins are inserted: the spike (S), the membrane (M), and the envelope (E) proteins. SARS-CoV-2 uses its S glycoprotein, a main target for the neutralizing antibody, to bind to its receptor angiotensin-converting enzyme 2 (ACE2), and mediate membrane fusion and virus genome release into the cytosol of an infected cell. Each monomer of trimeric S protein is about 180 kDa, and contains two subunits, S1 and S2, mediating attachment and membrane fusion, respectively [Chen 2020a, Ou 2020].

Since there is currently no effective treatment available for coronavirus infections, significant efforts have been made to the development of vaccines and therapeutic drugs. Most of therapeutic approaches that are currently being tested are based on repurposing the therapeutic agents previously designed for other applications. These agents can either directly target the virus replication cycle or aim boosting the innate antiviral immune responses or attenuating the damage induced by dysregulated inflammatory responses [Ahn 2020, Stebbing 2020].

Vaccination is the most effective strategy for preventing infectious diseases since it appears to be more cost-effective than therapeutic drugs and reduces morbidity and mortality without causing long-lasting undesirable effects. Although three human coronaviruses emerged worldwide over the past two decades, causing considerable threat to global health, there is still no approved vaccines for human coronaviruses. Research groups around the world are accelerating the development of COVID-19 vaccines using various approaches [Ahn 2020, Lu 2020].

A protective role of both humoral and cell-mediated immune responses against highly pathogenic coronaviruses has been suggested [Enjuanes 2016]. The SARS-CoV outbreak of 2002–2003 demonstrated that while antibody-mediated protection appeared to occur, antibody titers were found to be relatively short-lived in convalescent patients, dropping substantially by two years post-infection [Tang 2011]. In contrast, T cell responses remained detectable up to 11 years post-infection, and thus showed considerable promise for SARS-CoV vaccine development [Ng 2016]. Antibody responses against S protein, the most abundant protein of SARS-CoV-2, have been shown to protect from the infection [Du 2009, Ou 2020]. Current evidence strongly suggest that induction of a Th1 type response is important for successful control of highly pathogenic coronaviruses including SARS-CoV-2, as well as for reducing the theoretical risk of vaccine-enhanced disease [Honda-Okubo 2015]. These observations have been highly influential in the vaccine development efforts to prevent SARS-CoV-2 infection.

A large number of biopharmaceutical companies and academic laboratories are racing to develop prophylactic vaccine candidates against SARS-CoV-2 using a wide range of platforms including mRNA, DNA, inactivated virus, live viral vectors, recombinant proteins, peptides, and virus-like particles (VLPs) [Thanh Le 2020]. The vaccine candidate developed by Medicago R&D Inc. (Medicago) is a self-assembling virus-like particle (VLP) that displays trimers of recombinant S protein of SARS-CoV-2 integrated into the lipid bilayer of the nanoparticles. These VLPs are produced in a plant (*Nicotiana benthamiana*) and closely resemble the native structure of SARS-CoV-2 viruses, allowing them to be easily recognized by the immune system. In contrast to the native viruses, these plant-derived VLPs lack viral genetic material, and therefore are non-replicative and non-infections [Medicago 2020]. Based on positive results of pre-clinical testing in mice and non-human primates (Indian Rhesus macaques) and a successful Phase 1 study, Medicago plans to initiate this Phase 2/3 study.

1.1.1 Elderly Population

Although all age groups are at risk of contracting COVID-19, older persons are at a significantly higher risk of severe disease, hospitalization, intensive care unit (ICU) admissions, and death [Chen 2020b, Yang 2020a, Zhou 2020]. In the United States, population-based rates of

laboratory-confirmed COVID-19–associated hospitalization increased with age: 2.5 % in those 18–49 years of age, 7.4 % in those 50–64 years of age, and 13.8 % in those 65 years of age and older, ranging from 12.2 % in those 65–74 years of age to 17.2 % in those ≥85 years of age [Garg 2020]. The ICU admission rates are lowest among adults 20–44 years of age (2 %–4 %) and highest among adults 75–84 years of age (11%–31%) [CDC 2020]. Case fatality rates are as low as 0.05 % in individuals 20–29 years of age, increasing to 1.23 % in those 50–59 years of age, 5.80 % in those 60–69 years of age, and raising dramatically to 19.93 % in persons 70–79 years of age and 34.37 % in those 80 years of age and older in Canada [Health Canada 2020]. Although advanced age is an independent risk factor for severe COVID-19 [Chen 2020, Stokes 2020], the risk of poor clinical outcomes in older adults is strongly influenced to the prevalence of underlying medical conditions in the elderly [Du 2020, Shahid 2020].

Age adversely affects a wide variety of physiological functions, including the ability to mount robust and effective immune responses, which results in increased vulnerability to infectious diseases, diminished responses to vaccination, and a susceptibility to age-related inflammatory diseases [Pinti 2016]. The effect of aging on innate immunity has been observed in dendritic cells, macrophages, neutrophils, natural killer cells, and other cell types. The key innate immune functions such as chemotaxis and phagocytosis, antigen presentation, cytotoxicity, signals transduction, and secretion of and response to cytokines were found to be altered with advanced age [Shaw 2013]. Ageing is also associated with the increased production of pro-inflammatory cytokines. The continuous production of inflammatory mediators could potentially contribute to the onset of age-associated inflammatory diseases, frailty and mortality in older individuals [Furman 2019]. The impairment of adaptive immunity with aging is associated with decreased number of circulating B cells, lower antibody production, changed of antibody specificity repertoires and of B cell dynamics, and overall weakened humoral responses in older individuals [Frasca 2011]. Reduced numbers of circulating T cell and CD4+ and CD8+ subsets, a gradual shift from naïve CD45RA+ to activated or memory CD45RO+ cells, increased proportion of senescent or exhausted T cells that are functionally dormant have been correlated with frailty and dysregulation of adaptive immune responses in the elderly [Bektas 2017].

These age-related changes of immune system result in the elderly being less likely to benefit from vaccinations against a range of infectious diseases. Vaccine-induced immune responses are frequently lower in the elderly compared to younger adults [Crooke 2019, Pinti 2016]. The lower antibody responses observed in the elderly cannot be attributed solely to defects in B cell function. Age-related changes in antigen uptake, processing and presentation, as well as functional defects of T cells, can also contribute to reduced antibody responses [Clark 2012, Sasaki 2011]. Although less well-studied, cell-mediated immunity after vaccination can also be lower in older individuals [Weinberg 2019]. Various strategies have been pursued in order to improve vaccine-elicited immune responses and vaccine effectiveness in the elderly, including high-dose formulations and the use of immunity boosting adjuvants [Domnich 2017, Wilkinson 2017].

1.1.2 Adults with Comorbidities

People of any age with serious underlying medical conditions such as diabetes and cardiovascular or lung disease are at a greater risk of severe COVID-19. Those individuals with underlying uncontrolled medical conditions such as diabetes, hypertension, lung, liver, and kidney disease, cancer patients on chemotherapy, smokers, transplant recipients, and patients taking steroids represent a vulnerable population who are not only at a higher risk of developing severe illness but are also at an increased risk of death [Li 2020, Sanyaolu 2020]. Hypertension (odds ratio [OR]: 2.29), diabetes (OR: 2.47), chronic obstructive pulmonary disease (COPD; OR: 5.97), cardiovascular disease (OR: 2.93), and cerebrovascular disease (OR: 3.89) were independent risk factors associated with COVID-19 [Wang 2020]. A strong association of worse clinical outcomes in COVID-19 disease with obesity, even in the absence of any other comorbidity, has been reported [Korakas 2020]. The in-hospital death OR was found as high as 2.70 in patients with coronary artery disease, 2.48 in patients with congestive heart failure, 1.95 in those with cardiac arrhythmias, 2.96 in patients with COPD, and 1.79 for current smokers [Mehra 2020]. Overall, the risk of severe COVID-19 requiring admission to intensive care unit, invasive ventilation, or leading to death was found to be 1.79 fold greater among patients with at least one comorbidity and 2.59 fold greater among patients with two or more comorbidities compared to generally healthy subjects [Guan 2020a].

The need for an effective COVID-19 vaccine for people with underlying health conditions is obvious; however, vaccination strategies for these individuals may require some adjustment to achieve the desirable immune responses and vaccine effectiveness. Many comorbidities can adversely impact the response to vaccination, either due to general deterioration of the immune reactivity or as the result of pharmaceutical management of the underlying disease [Kwetkat 2020, Zimmermann 2019]. For example, patients with COPD generally respond poorly to influenza vaccination with significantly lower antibody titers and seroconversion rates: 43% in COPD compared to 90 % of healthy participants [Nath 2014]. Congestive heart failure patients develop a significantly lower T cell immune response to live-attenuated varicella-zoster vaccine and inactivated influenza vaccine than those without a cardiac impairment [Vardeny 2010, Verschoor 2017]. The effectiveness of 23-valent polysaccharide pneumococcal vaccine (PPV23) against invasive pneumococcal disease in individuals aged ≥65 years falls from 45 % in the healthy elderly to 25 % in high-risk immunocompetent patients with chronic respiratory/heart/liver/renal disease; diabetes mellitus; cerebrospinal fluid leaks; cochlear implants and is further reduced to 13 % in patients with underlying health conditions thought to be associated with an immune impairment (asplenia/splenic dysfunction, malignancy or an immunosuppressive drug treatment) [Djennad 2019].

1.1.3 Pediatric Population

People of all ages, including children, are at risk of contracting COVID-19 [Liu 2020b]. However, relatively fewer cases of symptomatic COVID-19 are observed among children compared to adult patients. In the US, 1.7 % of COVID-19 cases occurred in individuals aged <18 years, while this age group represents 22 % of the population [CDC 2020a]. Children

typically have milder disease and a better prognosis comparted to adults, and very few deaths in children have been reported to date [Ludvigsson 2020]. Nevertheless, children who are in contact with severe COVID-19 cases, those with underlying diseases, such as congenital heart, lung and airway diseases, chronic heart and kidney diseases, malnutrition, tumors, diabetes, immunodeficiency, hereditary metabolic diseases, those taking immunosuppressive medications, and infants under 3 months of age are considered to be at high risk of developing severe or critical illness [Shen 2020]. A severe, potentially life-threatening health condition called multisystem inflammatory syndrome is thought to be associated with SARS-CoV-2 infection in children. Its clinical manifestations such as acute illness accompanied by a hyperinflammatory syndrome leading to multiorgan failure and shock are to some extend similar to those of Kawasaki disease and toxic shock syndrome [Feldstein 2020]. Children may also play a role in SARS-CoV-2 community transmission. They often have symptoms of acute upper respiratory tract infection rather than lower respiratory tract involvement [Dong 2020]. Pediatric subjects were found to have detectable viral RNA in fecal samples 18 to 30 days from the disease onset [Cai 2020], which raised a concern about fecal-oral transmission of the infection. Prolonged shedding of the virus through nasal secretions and stool may have substantial implications for SARS-CoV-2 community spread at child-care facilities, schools and at home [Cruz 2020].

Children generally respond well to all vaccines including pandemic influenza vaccines and adjuvanted formulations, in terms of safety, immunogenicity and effectiveness [Dowling 2015, Garcia-Sicilia 2015, Manzoli 2012]. Antigen dose sparing has been achieved with adjuvanted vaccine formulations in children [Bravo 2020; Vesikari 2012].

1.2 Background of the Investigational Product

Medicago has developed a plant-based system (*Nicotiana benthamiana*) for transient expression of recombinant viral proteins, and this system has been used to produce VLPs bearing the SARS-CoV-2 S glycoprotein. Previously, Medicago used the same manufacturing platform to produce the Quadrivalent VLP Influenza Vaccine intended for active immunization for the prevention of influenza disease caused by influenza A subtype viruses and type B viruses contained in the vaccine. This plant-based platform has the following characteristics:

- The recombinant S protein is based on the genetic sequence of SARS-CoV-2 and is not subject to random mutation;
- Plant-based VLP manufacturing does not require the use of live viruses so no inactivation by chemicals or detergent-splitting is needed;
- The trimeric S proteins displayed in the surface of the VLPs are in a stabilized pre-fusion conformation that resembles the native structure of SARS-CoV-2 viruses, theoretically allowing them to be easily recognized by the immune system to induce a robust neutralizing antibody response and reduce the risk of vaccine enhanced disease [Lambert 2020].
- Very few microbial pathogens can infect both plants and humans so the risk of exposure to potentially pathogenic adventitious agents is greatly reduced;

- Medicago's previous clinical data suggests that plant-made VLP vaccines induce not only antibodies, but also strong CD4+ T cell immunity which may be important for both the persistence of immunity and the provision of better protection [Channappanavar 2014].

Nicotiana benthamiana is a non-transgenic plant that is a distant wild relative from Australia of the tobacco plant, *Nicotiana tabacum*. The transfer vector used to move the targeted S protein DNA construct into the plant cells is the bacterium *Agrobacterium tumefaciens*. This vector then directs the expression of the desired viral protein. The SARS-CoV-2 S protein is aligned at the plant plasma membrane via a transmembrane domain, and buds off the plant plasma membrane in the form of VLPs. Thus, the viral S protein trimers are anchored in a lipid bilayer of plant cell origin.

1.2.1 Background of the Adjuvant AS03

The adjuvant AS03 is an established effective adjuvant used in the formulations for Arepanrix™ and Pandemrix™ and is manufactured by GlaxoSmithKline. The AS03-adjuvanted pandemic influenza vaccines have been shown to be more immunogenic than non-adjuvanted vaccines, offering the potential of cross-clade immunity and feasibility of antigen-sparing. High efficacy and effectiveness have been demonstrated for AS03-adjuvanted H1N1 pandemic influenza vaccines in a wide range of populations [Garcon 2012]. Clinical data with AS03-adjuvanted antigen-sparing formulations have shown that immunization against influenza caused by the potential pandemic subtypes H5N1, H1N1, H7N1, H7N9, and H9N2 has demonstrated satisfactory immunogenic potency, as measured by haemagglutinin-inhibition titers, with reduced antigen doses in adults [Baz 2013, Jackson 2015, Lansbury 2017, Leroux-Roels 2007, Madan 2017, Madan 2017a, Madan 2017b, McElhaney 2013, Yang 2013, Yin 2011]. Also, AS03-adjuvanted H5N1 vaccines were shown to induce cross-clade neutralizing antibody responses [Leroux-Roels 2007] and antibody affinity maturation [Khurana 2018].

Data from clinical trials with over 55 000 vaccinated subjects showed that AS03-adjuvanted influenza vaccines exhibited an acceptable safety profile [Cohet 2019, Garcon 2012, Vaughn 2014]. Increased reactogenicity, both local and general, is consistently noted for AS03-adjuvanted vaccines compared with the corresponding unadjuvanted vaccines [Garcon 2012, Launay 2013, Nolan 2014, Waddington 2010]. Most symptoms were mild to moderate in intensity and of short duration. An increased risk of narcolepsy was observed in some individuals after the vaccination campaign with Pandemrix™ in 2009-2010. A similar risk of narcolepsy was not identified with other non-adjuvanted influenza vaccines or other AS03-adjuvanted vaccines, like Arepanrix™ [Montplaisir 2014, Cohet 2015]. Current data suggest that cases of narcolepsy seen immediately following the 2009-2010 pandemic were most likely the result of an immune cascade, triggered by CD4 T cell cross-reactivity to HA proteins from the H1N1 virus itself and hypocretin. Research is continuing to assess whether either of the main components of the 2009/2010 flu pandemic vaccine (e.g., the viral proteins in the form used in the vaccine or the AS03 adjuvant) may have contributed to the reaction.

1.3 Pre-clinical Studies

For comprehensive preclinical information regarding the safety and toxicity of CoVLP, refer to the current Investigator's Brochure (IB). No safety signal was identified in either the mouse or non-human primate (NHP) study. No suggestion of vaccine-enhanced disease was observed in the NHP upon challenge after either one or two doses of CoVLP alone (15 µg) or formulated with two different adjuvants (e.g. AS03, CpG 1018). Based upon the immunologic data collected to date, both adjuvants strongly enhanced antibody and T cell responses to CoVLP without evidence of a bias towards a Th2 response.

1.4 Clinical Studies

Currently, a Phase 1 clinical study (CP-PRO-CoVLP-019) testing one and two doses of CoVLP at three different dose levels (3.75 µg, 7.5 µg and 15 µg) alone and in combination with two different adjuvants (e.g. AS03, CpG1018) in 180 healthy adults is nearing completion with complete safety data to Day 42 and key Day 42 immunogenicity data available at the time of protocol preparation. Safety data collected to date do not reveal any safety concerns about either CoVLP alone or with either of the two adjuvants at any dose level. The data from this study clearly demonstrate the superiority of AS03 in the induction of anti-SARS-COV-2 antibodies at all dose levels. These data also suggest that two doses of AS03-adjuvanted CoVLP are needed to achieve consistently high antibody responses in healthy adults since robust boosting in the Day 42 antibody response after two doses of AS03-adjuvanted CoVLP was observed at all dose levels, Medicago has decided to carry the lowest dose, 3.75 µg, forward into the Phase 2/3 study.

1.5 Overall Rationale for the Study

This Phase 2/3 study is a multi-portion design to confirm that the chosen formulation and dosing regimen of CoVLP (i.e. two doses of 3.75 µg CoVLP adjuvanted with AS03 given 21 days apart) has an acceptable immunogenicity and safety profile in adults 18 years of age or older, including those with significant co-morbidities, and in children and adolescents 5 to 17 years of age (Phase 2 portion). The efficacy of this CoVLP formulation in these same Study Populations will be assessed in the Phase 3 portion.

The Phase 2 portion is a randomized, observer-blinded, placebo-controlled study to evaluate the CoVLP formulation, compared to placebo, in subjects from the four Study Populations presented in Table 4:

Table 4 Study Populations

Study Population	Age and/or Health Category	Age Range (years)
1	Healthy adults	18-64
2	Healthy children and adolescents	5-17
3	Elderly adults	≥ 65
4	Adults with significant co-morbidities	≥ 18

In each Study Population, 120 subjects will be randomized 4:1 to receive the CoVLP formulation:placebo. All subjects will be followed for a period of 12 months after the last vaccination for durability of the immune responses, safety, and efficacy.

The Phase 3 portion is an event-driven, randomized, observer-blinded, placebo-controlled design that will evaluate the efficacy and safety of the CoVLP formulation, compared to placebo, in each of the four Study Populations. Up to 30 000 subjects will be enrolled 1:1 to receive the CoVLP formlation:placebo. Immunogenicity will only be evaluated in a subset of 384 subjects.

All subjects will be followed for efficacy until approximately 160 COVID-19 cases have been accumulated in order to evaluate the primary efficacy endpoint of the Phase 3 portion. Subjects will be followed for safety for a period of 12 months after the last vaccination. Persistence of the vaccine-induced immune response up to 12 months after the last vaccination will be assessed in a subset of subjects.

2 OBJECTIVES

2.1 Primary Objectives

The primary objectives of the Phase 2 portion of the study are:

Safety:

- To assess the safety and tolerability of the CoVLP formulation, compared to placebo, up to 21 days after the last vaccination;

Immunogenicity:

- To assess the immunogenicity of the CoVLP formulation, compared to placebo, up to 21 days after the last vaccination, as determined by:
  - Neutralizing antibody titers induced by the vaccine against the SARS-CoV-2 virus;
  - IFN-γ ELISpot response induced by the vaccine against the SARS-CoV-2 virus to assess the Th1 type response.

The primary objective of the Phase 3 portion of the study is:

Efficacy:

- To evaluate the absolute efficacy of the CoVLP formulation, compared to placebo, in the prevention of laboratory-confirmed symptomatic SARS-CoV-2 infection (virologic method) starting 14 days after the last vaccination.

2.2 Secondary Objectives

The secondary objectives of the Phase 2 portion of the study are:

Immunogenicity:

- To assess the immunogenicity of the CoVLP formulation, compared to placebo, as determined by the:

- Neutralizing antibody titers induced by the vaccine against the SARS-CoV-2 virus at Days 201 and Day 386;
- IgG (ELISA) titers induced by the vaccine against the SARS-CoV-2 virus up to 21 days after the last vaccination and at Day 201 and Day 386;
- The ratio of neutralizing antibody titers:IgG (ELISA) antibody titers at Day 21, Day 42, Day 201, and Day 386;
- IFN-γ ELISpot response induced by the vaccine against the SARS-CoV-2 virus at Day 201 and Day 386;
- IL-4 ELISpot response induced by the vaccine against the SARS-CoV-2 virus up to 21 days after the last vaccination and at Day 201 and Day 386;

Safety:

- To assess the safety and tolerability of the CoVLP formulation, compared to placebo, as determined by the occurrence(s) of:
  - MAAEs, SAEs, AEs leading to withdrawal, AESIs (including VED), and deaths from 22 days after the last vaccination up to the end of the study;

Efficacy:

- To evaluate the absolute efficacy of the CoVLP formulation, compared to placebo, in the prevention of laboratory-confirmed symptomatic SARS-CoV-2 infection (virologic method) starting 14 days after the last vaccination;
- To evaluate the absolute efficacy of the CoVLP formulation, compared to placebo, in the prevention of severe COVID-19 disease starting 14 days after the last vaccination. Severe COVID-19 disease will be defined as virologically-confirmed SARS-CoV-2 infection with the occurrence of a new onset of any of the following:
  - Clinical signs at rest indicative of severe systemic illness (respiratory rate ≥ 30 per minute, heart rate ≥ 125 per minute, SpO2 ≤ 93% on room air at sea level or PaO2/FiO2 < 300 mmHg);
  - Respiratory failure (defined as needing high-flow oxygen, non-invasive ventilation, mechanical ventilation or ECMO);
  - Evidence of shock (SBP < 90 mmHg, DBP < 60 mmHg, or requiring vasopressors);
  - Significant acute renal, hepatic, or neurologic dysfunction;
  - Hospitalization;
  - Death.

  The list above applies to the adult populations; for children and adolescents, the following list of occurrences [Shen 2020] is applicable:

  - Severe pneumonia:
    - Polypnea: ≥ 30 times/min (>5 years) (after ruling out the effects of fever and crying);

    - Oxygen saturation < 92% under a resting state;
    - Dyspnea: assisted breathing (moans, nasal flaring, and three concave sign), cyanosis, intermittent apnea;
    - Disturbance of consciousness: somnolence, coma, or convulsion;
    - Pulmonary high-resolution CT (HRCT) examination showing bilateral or multi-lobe infiltrates, rapid progression of disease in a short period or with pleural effusion
- Critical cases: those who meet any of the following criteria and require hospitalization:
    - Respiratory failure requiring mechanical ventilation;
    - Shock;
    - Combined with other organs failure;
- Death.

The secondary objectives of the Phase 3 portion of the study are:

Efficacy:

- To evaluate the absolute efficacy of the CoVLP formulation, compared to placebo, in the prevention of severe COVID-19 disease starting 14 days after the last vaccination (see definition for severe COVID-19 disease above);
- To assess COVID-19-related symptoms, and the severity of these symptoms, up until resolution of subjects administered the CoVLP formulation compared to subjects administered the placebo;

Immunogenicity:

- To assess the immunogenicity of the CoVLP formulation, compared to placebo, up to 21 days after the last vaccination, as determined by:
  - Neutralizing antibody titers induced by the vaccine against the SARS-CoV-2 virus;
  - IFN-γ ELISpot response induced by the vaccine against the SARS-CoV-2 virus to assess the Th1 type response;

Safety:

- To assess the safety and tolerability of the CoVLP formulation, compared to placebo, up to the end of the study.

2.3 Exploratory Objectives

The exploratory objectives of the Phase 2 portion of the study are:

Efficacy:

- To assess COVID-19-related symptoms, and the severity of these symptoms, up until resolution of subjects administered the CoVLP formulation compared to subjects administered the placebo;

Immunogenicity:

- To further characterize the immune response of the CoVLP formulation, if it is likely to be informative;

Safety:

- To further characterize the safety profile of the CoVLP formulation, if it is likely to be informative.

The exploratory objectives of the Phase 3 portion of the study are:

Immunogenicity:

- To assess the immunogenicity of the CoVLP formulation, compared to placebo, in a subset of subjects, as determined by the:
  - Neutralizing antibody titers induced by the vaccine against the SARS-CoV-2 virus at Day 201 and Day 386;
  - IgG (ELISA) titers induced by the vaccine against the SARS-CoV-2 virus up to 21 days after the last vaccination and at Day 201 and Day 386;
  - The ratio of neutralizing antibody titers:IgG (ELISA) antibody titers at Day 21, Day 42, Day 201, and Day 386;
  - IFN-γ ELISpot response induced by the vaccine against the SARS-CoV-2 virus at Day 201 and Day 386;
  - IL-4 ELISpot response induced by the vaccine against the SARS-CoV-2 virus up to 21 days after the last vaccination and at Day 201 and Day 386;
  - Specific cell-mediated immune (CMI) response induced by the vaccine against the SARS-CoV-2 virus up to 21 days after the last vaccination and at Day 201 and Day 386, as measured by the percentage of CD4+ T cells expressing functional markers;
- To further characterize the immune response of the CoVLP formulation in a subset of subjects, if it is likely to be informative;

Safety:

- To further characterize the safety profile of the CoVLP formulation, if it is likely to be informative.

3 STUDY ADMINISTRATIVE STRUCTURE

Table 5 Study Administrative Structure

Role	Name and Address
**Sponsor**	Medicago R&D Inc.<br>1020, route de l'Église, bureau 600,<br>Sainte-Foy (Qc) Canada G1V 3V9
Medical Officer:	[Redacted]
Medical Monitor:	[Redacted]
Senior Director, Clinical Research:	[Redacted]
Medical Writer	[Redacted]

4 STUDY DESIGN AND RATIONALE

4.1 Overview of Study Design

The Phase 2 portion is a randomized, observer-blinded, placebo-controlled study with male and female subjects. Subjects will be enrolled from four Study Populations (refer to Table 4).

In each of the four Study Populations, 120 subjects will be randomized 4:1 and will receive the same CoVLP formulation or placebo. Subjects will receive two IM injections 21 days apart on Days 0 and 21, into the deltoid region of the alternating arm (each arm will be injected once), of one of the following:

- CoVLP formulation;
- Placebo.

Adults 18 years of age and older (i.e. Study Populations #1, #3, and #4) will begin enrollment at the same time. Enrollment of elderly adult subjects (Study Population #3) will be stratified by age in a 2:1 ratio of 65 to 74 years of age and 75 years of age and older, respectively. Enrollment of healthy children and adolescents aged 5 to 17 years (i.e. Study Population #2) will begin only after seven-day safety data following the second vaccination for the Healthy Adult population (i.e. Day 28 for all subjects in Study Population #1) have been reviewed by the IDMC. Study Population #2 will be stratified by age in a 1:1 ratio of adolescents 12-17 years of age followed by children 5-11 years of age. Enrollment of children will only begin after the seven-day safety data following the second vaccination for all adolescent subjects have been reviewed by the IDMC (i.e. Day 28).

The IDMC will review unblinded safety data for the first 10 sentinel subjects after each dose in all Study Populations, with the exception of Study Population #1, according to the details outlined in the IDMC Charter. All sentinel subjects will be dosed at least 30 minutes apart.

Subjects will be screened up to 14 days in advance of the first vaccine administration and will demonstrate a satisfactory baseline medical assessment by history, general physical examination, haematological, biochemical, and serological analysis. Although a test for SARS-CoV-2 antibodies will be performed at screening, both seronegative and seropositive subjects will be enrolled. On Day 0 and Day 21, vaccine administration will occur. Phone contacts will be made one day and eight days after each vaccine administration, specifically for review of the subject’s safety and concomitant medication data. Visits to the Investigator site will occur 3 days after each vaccine administration for key safety assessments, and 21 days after each vaccine administration for key safety and immunogenicity assessments. Subjects will return to the Investigator site on Day 201 and Day 386 (6-month and 12-month safety follow-ups and immunogenicity assessments after the last vaccine administration).

Active and passive surveillance will be conducted in the study from 14 days after the last vaccination until 386 days. For passive surveillance, subjects (and/or their parent/LAR) will be instructed to report symptoms meeting the definition of new COVID-19 disease. For active surveillance, subjects (and/or their parent/LAR) will be contacted once per week via the subject’s (and/or their parent’s/LAR’s) preferred method (e.g. phone, text, email, electronic diary), and asked about symptoms of COVID-19 disease.

The Phase 3 portion is a randomized, observer-blinded, placebo-controlled, efficacy, safety, immunogenicity (in a subset of subjects), multicenter design with male and female subjects. Similar to the Phase 2 portion, subjects will be enrolled from four Study Populations (refer to Table 3) and each population will be stratified by age in the same manner. The overall goal of the Phase 3 portion of this study is to enroll approximately 30 000 subjects who are generally representative of the overall target population in terms of racial diversity with some degree of enrichment for those at highest risk for serious COVID-19 disease (i.e. the elderly and those with co-morbidities) and relatively smaller numbers of the subjects at lowest risk of serious COVID-19 (i.e. children and adolescents).

In each Study Population, subjects will be randomized into one of two treatment groups:

- CoVLP formulation;
- Placebo.

Subjects will be screened up to 14 days in advance of the first vaccine administration and will demonstrate a satisfactory baseline medical assessment by history and general physical examination. Although a test for SARS-CoV-2 antibodies will be performed at screening, both seronegative and seropositive subjects will be enrolled. On Day 0 and Day 21, vaccine administration will occur. Phone contacts will be made one day and eight days after each vaccine administration, specifically for review of the subject’s safety and concomitant medication data. Visit to the Investigator site will occur 21 days after each vaccine administration for immunogenicity assessments in the immunogenicity subset only. Subjects will return to the Investigator site on Day 201 and Day 386 (6-month and 12-month safety follow-ups and immunogenicity assessments [immunogenicity subset only] after the last vaccine administration).

Active and passive surveillance will be conducted in the study from 14 days after the last vaccination until 160 COVID-19 cases have been accumulated in order to evaluate the primary efficacy endpoint of the Phase 3 portion of the study. For passive surveillance, subjects (and/or their parent/LAR) will be instructed to report symptoms meeting the definition of new COVID-19 disease. For active surveillance, subjects (or their parent/LAR) will be contacted once per week via the subject's (and/or their parent's/LAR's) preferred method (e.g. phone, text, email, electronic diary), and asked about symptoms of COVID-19 disease.

4.2 Rationale of Study Design

4.2.1 Randomization

Randomization will be used to minimize bias in the assignment of subjects to treatment groups, within each Study Population, to increase the likelihood that known and unknown subject attributes (e.g. demographic and baseline characteristics) are evenly balanced across treatment groups and to enhance the validity of statistical comparisons across treatment groups within each Study Population.

4.2.2 Blinding

Both portions of the study will be observer-blind to reduce potential bias during data collection and evaluation of the study endpoints. Details of who will remain blinded during the Phase 2 and Phase 3 portions of the study are presented in Section 6.2.

Selected individuals involved in the IDMC reviews and/or interim data analyses will be unblinded during the Phase 2 and Phase 3 portions of the study, with details presented in Section 6.2.

4.2.3 Dose Selection and Dosage Regimen

In both the Phase 2 and Phase 3 portions, a single dose level of the CoVLP formulation (i.e. 3.75 µg CoVLP adjuvanted with AS03) will be tested in a two-dose regimen on Day 0 and Day 21.

4.2.4 Route of Administration

The route of administration used for the CoVLP formulation is the IM route, specifically in the deltoid muscle of the arm, since it reliable route of administration with good absorption compared to the subcutaneous route. The IM route was used in the Phase 1 study.

4.2.5 Study Duration

The planned duration of the Phase 2 portion is approximately 14 to 16 months and the Phase 3 portion is approximately 14 months, from screening procedures up to the end of each portion.

5 STUDY POPULATION

5.1 Inclusion Criteria

Subjects must meet all of the following inclusion criteria at the Screening (Visit 1) and/or Vaccination (Visit 2) visits to be eligible for participation in this study; no protocol waivers are allowed. All Investigator assessment-based judgements must be carefully and fully documented in the source documents:

1. Subjects (and/or their parent/LAR) must have read, understood, and signed the informed consent form (ICF) prior to participating in the study; subjects must also complete study-related procedures and the subjects (and/or their parent/LAR) must communicate with the study staff at visits and by phone during the study;
2. At the Screening visit (Visit 1), male and female subjects must be
   - Study Population #1: 18 to 64 (has not yet had his/her 65th birthday) years of age, inclusive;
   - Study Population #2: 5 to 17 (has not yet had his/her 18th birthday) years of age, inclusive;
   - Study Population #3: 65 years of age or older;
   - Study Population #4: 18 years of age or older;
3. At Screening (Visit 1) and Vaccination (Visit 2), subject must have a body mass index (BMI) of:
   - Study Populations #1 and #3: $\geq$ 18.5 and < 30 kg/m^2;
   - Study Population #2: > 5th and < 85th percentile based on the CDC ‘BMI for ‘age’ scale (https;//www.cdc.gov/healthyweight/bmi/calculator.html);
4. Subjects (and/or their parent/LAR) are considered by the Investigator to be reliable and likely to cooperate with the assessment procedures and be available for the duration of the study;
5. Study Populations #1 and #2: Subjects must be in good general health prior to study participation, with no clinically relevant abnormalities that could jeopardize subject safety or interfere with study assessments, as determined by medical history, physical examination, and vital signs. Investigator discretion will be permitted with this inclusion criterion;

   Note: Subjects with a pre-existing chronic disease will be allowed to participate if the disease is stable and, according to the Investigator’s judgment that must be documented in the source documents, the condition is unlikely to confound the results of the study or pose additional risk to the subject by participating in the study. Stable disease is generally defined as no new onset or exacerbation of pre-existing chronic disease three months prior to vaccination. Based on the Investigator’s judgment, a subject with more recent stabilization of a disease could also be eligible.
6. Study Populations #1, #2, and #4: Female subjects of childbearing potential must have a negative serum pregnancy test result at Screening (Visit 1) and a negative urine pregnancy test result at Vaccination (Visit 2), with the following exception:

- Study Population #1 (for the Phase 3 portion of the study only): Female subjects who are in the 3rd trimester of a low-risk pregnancy at Screening (Visit 1) can be enrolled in the study;

Non-childbearing females are defined as:

- Surgically-sterile (defined as bilateral tubal ligation, hysterectomy or bilateral oophorectomy performed more than one month prior to the first study vaccination); or
- Post-menopausal (absence of menses for 12 consecutive months and age consistent with natural cessation of ovulation);
- Non-ovulating, pre-menstrual girls < 17 years of age;

7. Study Populations #1, #2, and #4: With the exception of low-risk pregnant women in their third trimester noted above in inclusion criterion no. 6 for the Phase 3 study only, female subjects of childbearing potential must use an effective method of contraception for one month prior to vaccination (Visit 2) and agree to continue employing highly effective birth control measures for at least one month after the last study vaccination (or in the case of early termination, she must not plan to become pregnant for at least one month after her last study vaccination), with the exception of the following subjects:

The following relationship or methods of contraception are considered to be highly effective:

- Combined (estrogen and progestogen containing) hormonal contraception associated with inhibition of ovulation:
  - Oral;
  - Intravaginal;
  - Transdermal;
- Progestogen-only hormonal contraception associated with inhibition of ovulation:
  - Oral;
  - Injectable;
  - Implantable;
- Intra-uterine device with or without hormonal release;
- Vasectomised partner, provided that this partner is the sole sexual partner of the study participant and that the vasectomised partner has received a medical assessment of the surgical success;
- Credible self-reported history of heterosexual vaginal intercourse abstinence prior to and for at least one month after the last study vaccination. Abstinent subjects who are ovulating should be asked what method(s) they would use should their circumstances change, and subjects without a well-defined plan should be excluded. Ideally, the parent/LAR should not be present when a female subject < 18 years of age is reporting her sexual activity level and method of contraception;
- Female partner.

8. Study Population #3: Subjects must be non-institutionalized (e.g. not living in rehabilitation centres or old-age homes; living in an elderly community is acceptable) and have no acute or evolving medical problems prior to study participation and no clinically relevant abnormalities that could jeopardize subject safety or interfere with study assessments, as assessed by the Principal Investigator or sub-Investigator (thereafter referred as Investigator) and determined by medical history, physical examination, serology (only for the Phase 2 portion), clinical chemistry and haematology tests (only for the Phase 2 portion), urinalysis (only for the Phase 2 portion), and vital signs. Investigator discretion will be permitted with this inclusion criterion.

   Note: Subjects with a pre-existing chronic disease will be allowed to participate if the disease is stable and, according to the Investigator's judgment that must be documented in the source documents, the condition is unlikely to confound the results of the study or pose additional risk to the subject by participating in the study. Stable disease is generally defined as no new onset or exacerbation of pre-existing chronic disease three months prior to vaccination. Based on the Investigator's judgment and documented in source documentation, a subject with more recent stabilization of a disease could also be eligible;
9. Study Population #4: Subjects must have one or more co-morbid conditions that puts them at higher risk for severe COVID-19 disease. These co-morbidities include but are not limited to obesity, documented hypertension, type-1 or type 2 diabetes, chronic obstructive lung diseases (COPD), cardiovascular disease, chronic kidney, or be immunocompromised persons (e.g., HIV-infected patients, organ transplant recipients, or patients receiving cancer chemotherapy). Investigator discretion will be permitted with this inclusion criterion.

5.2 Exclusion Criteria

Subjects who meet any of the following criteria at the Screening (Visit 1) and/or Vaccination (Visit 2) visits will not be eligible for participation in this study; no protocol waivers are allowed. All Investigator assessment-based judgements must be thoroughly documented in the source documents:

1. Study Populations #1, #2, and #3: According to the Investigator's opinion, significant acute or chronic, uncontrolled medical or neuropsychiatric illness.

   Acute disease is defined as presence of any moderate or severe acute illness with or without a fever within 48 hours prior to the Screening (Visit 1) and/or Vaccination visit (Visit 2). 'Uncontrolled' is defined as:

   - Requiring a new medical or surgical treatment during the three months prior to study vaccine administration;
   - Requiring any significant change in a chronic medication (i.e. drug, dose, frequency) during the three months prior to study vaccine administration due to uncontrolled symptoms or drug toxicity unless the innocuous nature of the medication change meets the criteria outlined in inclusion criterion no. 5 (Study Population 1) or no. 8 (Study Population 3) and is appropriately justified and documented by the Investigator.

   Investigator discretion is permitted with this exclusion criterion and must be carefully and fully documented in the source documents;

2. Study Populations #1, #2, and #3: Any confirmed or suspected current immunosuppressive condition or immunodeficiency, including cancer, human immunodeficiency virus (HIV), hepatitis B or C infection (subjects with a history of cured hepatitis B or C infection without any signs of immunodeficiency at present time are allowed). Investigator discretion is permitted with this exclusion criterion;
3. Study Populations #1, #2, and #3: Current autoimmune disease (such as rheumatoid arthritis, systemic lupus erythematosus or multiple sclerosis). Investigator discretion is permitted with this exclusion criterion, and subjects may be eligible to participate with appropriate written justification in the source document (i.e. subjects with a history of autoimmune disease who are disease-free without treatment for three years or more, or on stable thyroid replacement therapy, mild psoriasis [i.e. a small number of minor plaques requiring no systemic treatment], etc.);
4. Study Populations #1, #2, and #3: Administration of any medication or treatment that may alter the vaccine immune responses, such as:
   - Study Populations #1 and #3: Systemic glucocorticoids at a dose exceeding 10 mg of prednisone (or equivalent) per day for more than seven consecutive days or for 10 or more days in total, within one month prior to the Vaccination visit (Visit 2). Inhaled, nasal, ophthalmic, dermatological, and other topical glucocorticoids are permitted;
   - Study Population #2: Systemic glucocorticoids at a dose exceeding 0.28 mg / kg of prednisone (or equivalent) per day for more than seven consecutive days or for ten or more days in total, within one month prior to the Vaccination visit (Visit 2). Inhaled, nasal, ophthalmic, dermatological, and other topical glucocorticoids are permitted;
   - Cytotoxic, antineoplastic, or immunosuppressant drugs - within 36 months prior to Vaccination (Visit 2);
   - Any immunoglobulin preparations or blood products, blood transfusion – within 6 months prior to Vaccination (Visit 2);
5. Study Population #4: Acute disease defined as presence of any moderate or severe acute illness with or without a fever within 48 hours prior to the Screening (Visit 1) and/or Vaccination visit (Visit 2);
6. Administration of any vaccine within 30 days prior to Vaccination (Visit 2); planned administration of any vaccine during the study (up to blood sampling on Day 42 of the study). Immunization on an emergency basis during the study will be evaluated on case-by-case basis by the Investigator;
7. Administration of any other SARS-CoV-2 / COVID-19, or other experimental coronavirus vaccine at any time prior to or during the study;
8. Use of any investigational or non-registered product within 30 days or 5 half-lives, whichever is longer, prior to Vaccination (Visit 2) or planned use during the study period. Subjects who are in a prolonged post-administration observation period of another investigational or marketed drug clinical study, for which there is no ongoing exposure to the investigational or marketed product and all scheduled on-site visits are completed, will be allowed to take part in this study, if all other eligibility criteria are met;

9. Have a rash, dermatological condition, tattoos, muscle mass, or any other abnormalities at injection site that may interfere with injection site reaction rating. Investigator discretion will be permitted with this exclusion criterion;
10. Use of any prescription antiviral drugs with the intention of COVID-19 prophylaxis, including those that are thought to be effective for prevention of COVID-19 but have not been licensed for this indication, within one month prior to Vaccination (Visit 2);
11. For the Phase 2 portion of the study only: Use of prophylactic medications (e.g. antihistamines [H1 receptor antagonists], nonsteroidal anti-inflammatory drugs [NSAIDs], systemic and topical glucocorticoids, non-opioid and opioid analgesics) within 24 hours prior to the Vaccination (Visit 2) to prevent or pre-empt symptoms due to vaccination;
12. History of a serious allergic response to any of the constituents of CoVLP including AS03;
13. History of a documented anaphylactic reactions to plants or plant components (including tobacco, fruits and nuts);
14. Subjects with a history of Guillain-Barré Syndrome;
15. Study Populations #1 (for the Phase 2 portion of the study), #2, and #4: Any female subject who has a positive or doubtful pregnancy test result prior to vaccination or who is lactating;
16. Any female subject who is in the 1st or 2nd trimester of her pregnancy or whose pregnancy is considered to be high-risk by her obstetrician;
17. Subjects identified as an Investigator or employee of the Investigator or clinical site with direct involvement in the proposed study, or identified as an immediate family member (i.e. parent, spouse, natural or adopted child) of the Investigator or employee with direct involvement in the proposed study, or any employees of Medicago.

5.3 Prior and Concomitant Therapy

New or changed medications reported by the subject after the first vaccination and through to the end of the study will be recorded in the source documents as a concomitant medication as per the conditions outlined in the next paragraph. Since AEs may be secondary to new medications, the Investigator will explore the reasons for the change or for the new medication intake and document these AEs, if any.

Concomitant medications must be reported (reason for use, dates of administration, dosage, and route) if the use meets the following conditions:

- Within 30 days preceding vaccination: any treatments and/or medications specifically contraindicated (e.g. vaccines, any immunoglobulins or other blood products, or any immune modifying drugs, etc.);
- From randomization to Day 42, inclusive: any medication (including, but not limited to, over-the-counter medicines such as aspirin or antacids), vitamins, and mineral supplements;
- From Day 43 to the end of the study, inclusive: any concomitant medication(s) administered to treat an AESI, MAAE, SAE, or AE leading to withdrawal; any concomitant medication used to treat an AE that occurred before Day 42 and that is still being used afterwards (i.e. on-going use);

- Any concomitant medication used to treat conditions reported as medical history;
- Any investigational medication or vaccine; any vaccine not foreseen in the study protocol.

5.4 Prohibited Therapy

The following medications or therapies are prohibited during the conduct of this study:

1. Administration of any vaccine (other than the study vaccine) up to blood sampling on Day 42 of the study as well as administration of any investigational or approved coronavirus vaccine (other than the study vaccine) up to end of the study. Immunization on an emergency basis during the study will be evaluated on case-by-case basis by the Investigator;
2. Use of any investigational or non-registered product during the study period. Subjects may not participate in any other investigational or marketed drug study while participating in this study until after the study;
3. Study Populations #1, #2, and #3: Administration of any medication or treatment that may alter the vaccine immune responses, such as:
   - Systemic glucocorticoids;
   - Cytotoxic, antineoplastic, or immunosuppressant drugs;
   - Any immunoglobulin preparations or blood products, or blood transfusion.

   Administration of such medications should be specifically avoided up to blood sampling on Day 42 of the study. Use of any medication or treatment that may alter the vaccine immune responses due to a medical need during the study will be evaluated on case-by-case basis by the Investigator;
4. In the Phase 2 portion of the study, use of prophylactic medications (e.g. antihistamines [H1 receptor antagonists], nonsteroidal anti-inflammatory drugs [NSAIDs], systemic and topical glucocorticoids, non-opioid and opioid analgesics) within 24 hours prior to administration of the second dose of the vaccine to prevent or pre-empt symptoms due to vaccination
5. Use of any prescription antiviral drugs with the intention of COVID-19 prophylaxis, including those that are thought to be effective for prevention of COVID-19 but have not been licensed for this indication, during the study.

Given that an important objective of this study is to evaluate the tolerability of the study vaccine, the use of prophylactic medications to prevent or pre-empt symptoms due to vaccination is specifically prohibited up to seven days after each vaccination in the Phase 2 portion of the study (end of collection of solicited symptoms). A prophylactic medication is a medication administered in the absence of ANY symptom and in anticipation of a reaction to the vaccination (e.g. an antipyretic is considered to be prophylactic when it is given in the absence of fever or any other symptoms, to prevent fever from occurring, vitamins used to boost immune system, etc.).

If any of the Prohibited Therapy criteria are met by a subject, the subject may remain in the study however the inclusion of the subject's data within the PP set, IPP set, ITT set, or SAS may be impacted.

6 TREATMENT ALLOCATION AND BLINDING

6.1 Randomization

In the Phase 2 portion, subjects will be randomized 4:1 to receive the CoVLP formulation and placebo in each Study Population. In the Phase 3 portion, subjects will be randomized 1:1 to receive the CoVLP formulation and placebo in each Study Population. Randomization of elderly adult subjects (Study Population #3) will be stratified by age in a 2:1 ratio of 65 to 74 years of age and 75 years of age and older, respectively. Randomization of healthy children and adolescents (Study Population #2) will be stratified by age in a 1:1 ratio of adolescents 12-17 years of age followed by children 5-11 years of age. During Phase 2 and Phase 3 portions of the study, eligible subjects will be randomized at the Day 0 visit using the interactive response technology (IRT) system.

Once a randomization number has been assigned, it will not be re-used for any reason. No subjects will be randomized into the study more than once. If a randomization number is allocated incorrectly, no attempt will be made to remedy the error once the study vaccine has been dispensed: the subject will continue on the study with the assigned randomization number and associated treatment. The study staff will notify the Sponsor Contact as soon as the error is discovered without disclosing the study vaccine administered. Admission of subsequent eligible subjects will continue using the next unallocated number in the sequence.

Subjects will be enrolled into treatment groups in each phase of the study based on a computer-generated randomization schedule prepared by or under the supervision of Medicago before the study. The randomization number and treatment will be recorded along with a unique subject number for each subject in the investigational product accountability log.

6.2 Blinding

Both portions of the study will be observer-blinded.

During the entire duration of the study, the following individuals will not have access to treatment allocation (i.e. remain “blind”): the subjects (and/or their parent/LAR), the Investigators and all personnel involved in the clinical conduct of the study (except the staff involved in the preparation and administration of the study vaccine, the quality assurance auditor, and quality control reviewers), Medicago clinical staff and medical staff involved in safety evaluations (e.g. causality assessments), and all personnel involved in sample analysis at the central and testing (Nab, ELISA, ELISpot, ICS [flow cytometry], serological, and virologic method SARS-CoV-2 diagnostics) laboratories.

During the study, the IDMC and unblinded team, compromised of the unblinded medical monitor, unblinded biostatistician, unblinded senior reviewer, and unblinded programmer involved in the preparation of the safety data summary for the IDMC reviews, will have access to or be aware of treatment allocation (i.e. be “unblinded”). The IDMC will review safety data and will make recommendations for the entire duration of the study. The same unblinded

statistical team will be involved in the Day 21 and Day 42 (if needed) immunogenicity data analyses in the Phase 2 portion and in the interim efficacy analyses in the Phase 3 portion.

During the study, the Sponsor's Study Team will remain blinded. A small number of Medicago personnel (not included in Study Team) will be involved in the planned analyses during the Phase 2 and Phase 3 portions. The selected individuals and the process and level of unblinding will be documented.

Since the CoVLP formulation will have a different physical appearance and viscosity from the placebo, the site staff involved in the preparation and administration of the treatments will not be involved in any activity that could introduce a bias, such as the evaluations of reactogenicity or AEs experienced by the subjects following vaccination.

Any code break will be documented and reported to Medicago (or its designee) in a timely manner. In a medical emergency, the Investigator may unblind the treatment for that subject without prior consultation with the Sponsor. In such an event, the Investigator will need to contact the responsible Medical Monitor as soon as possible after the unblinding to discuss the case. The treatment allocation should not be disclosed by the Investigator to the responsible Medical Monitor.

7 DOSAGE AND ADMINISTRATION

In the Phase 2 and Phase 3 portions of the study, the CoVLP formulation has been selected based on the Phase 1 study data, including acceptable safety and immunogenicity profiles. Subjects in each of the four Study Populations will receive the same CoVLP formulation or placebo.

Subjects will receive two IM injections 21 days apart on Days 0 and 21, into the deltoid region of the alternating arm (each arm will be injected once), of one of the following:

- CoVLP formulation;
- Placebo.

The volume of each injection will be 0.5 mL.

8 SPECIMENS AND CLINICAL SUPPLIES

8.1 Management of Samples

Blood samples for biochemistry, haematology, and serology (for HIV, Hepatitis B, and Hepatitis C) and urine samples for urinalysis will be collected at screening and/or during the study for the Phase 2 portion of the study. Serum (Phase 2 portion only) and urine (dipstick or similar) pregnancy testing will be performed for females of childbearing potential. Blood samples will also be collected for the testing of SARS-CoV-2 antibodies at screening.

Blood samples for immunogenicity analysis will be collected from all subjects in the Phase 2 portion of the study and from a subset of subjects in the Phase 3 portion of the study. Should a subject be discontinued from the study, a sample will be drawn at the time of the final visit and sent to Medicago for analysis. Bioanalysis of the immunogenicity blood samples will be conducted at central laboratories.

Between vaccination and the end of the surveillance period, nasal or NP swabs will be collected from any subject who reports any symptoms associated with COVID-19 after the start of active and passive surveillance; these swabs will be collected each time a subject reports such an event within the specified timeframe, so multiple collections may be required from some subjects. Two swabs will be collected per subject per event (one from each nare). Bioanalysis of the nasal or NP swabs will be conducted at central laboratories.

Complete information on the handling, storage, and shipment of all laboratory samples will be described in the study-specific documentation.

8.2 Collection of Samples

During the Phase 2 and Phase 3 portions of the study, the Time and Events Schedules: General Information for each portion (see Table 1 and Table 2) summarizes the frequency and timing of the scheduled samples to be collected.

All subjects in the Phase 2 portion of the study will have blood sampled. Subjects will have blood volumes drawn of up to approximately 287 mL over a period of 386 days (Table 6).

Table 6 Estimated Blood Volume Drawn: Phase 2 Portion

Type of Sample	Volume per Sample (mL)	Number of Samples per Subject									Total Volume of Blood per Subject (mL)
		Visit 1 (Day -14 to 0)	Visit 2 (Day 0)	Visit 3 (Day 3)	Visit 4 (Day 21)	Visit 5 (Day 24)	Visit 6 (Day 42)	Visit 7 (Day 201)	Visit 8 (Day 386)	Total	
Test for SARS-CoV-2 antibodies	5	1								1	5
Biochemistry, haematology, serology (HIV, hepatitis B & C, pregnancy test)*	8	1		1	1	1				4	32
Serology for immunogenicity (Nab assay, ELISA)	10		1		1		1	1	1	5	50
CMI response (PBMC)†	20-40		1		1		1	1	1	5	100-200
Total volume of blood per subject (mL)†		13	30-50	8	38-58	8	30-50	30-50	30-50		187-287

* Samples collected during the screening visit should be performed under fasting conditions (approximately 12 hours) for cholesterol and triglyceride analysis. Note that cholesterol and triglyceride analysis will be performed only at screening.

† Only a volume of 20 mL will be drawn at each applicable time-point from children < 12 years of age or weigh less than 40 kg (90 lbs).

In the Phase 3 portion of the study, all subjects will have 5 mL of blood drawn at screening and only a subset of subjects included in the immunogenicity analyses will have an additional blood volume of up to approximately 250 mL drawn up to the end of the study (Day 386) (Table 7).

Table 7 Estimated Blood Volume Drawn: Phase 3 Portion

Type of Sample	Volume per Sample (mL)	Number of Samples per Subject							Total Volume of Blood per Subject (mL)
		Visit 1 (Day - 14 to 0)	Visit 2 (Day 0)	Visit 3 (Day 21)	Visit 4 (Day 42)	Visit 5 (Day 201)	Visit 6 (Day 386)	Total	
Test for SARS-CoV-2 antibodies	5	1						1	5
Serology for immunogenicity (Nab assay, ELISA)*	10		1	1	1	1	1	5	50
CMI response (PBMC)*†	20-40		1	1	1	1	1	5	100-200
Total volume of blood per subject (mL)†		5	30-50	30-50	30-50	30-50	30-50		155-255

* These samples will be collected for a pre-defined subset of 384 subjects.

† Only a volume of 20 mL will be drawn at each applicable time-point from children < 12 years of age or weigh less than 40 kg (90 lbs).

Urine samples will be collected at screening and Days 3, 21, and 24 during the Phase 2 portion of the study.

In the Phase 2 portion of the study, pregnancy testing will be performed for all females of childbearing potential (in Study Populations #1, #2, and #4). A serum pregnancy test will be performed during screening. A urine (dipstick or similar) pregnancy test will be performed at Day 0 prior to randomization and at Days 21 and 42. In Study Populations #1, #2, and #4, females of child bearing potential must have negative serum pregnancy test result from screening and urine pregnancy test result from Day 0 available prior to vaccination.

In the Phase 3 portion of the study, pregnancy testing will be performed for all females of childbearing potential (in Study Populations #1, #2, and #4). A urine (dipstick or similar) pregnancy test will be performed at screening and at Day 0 prior to randomization and at Days 21 and 42. In Study Populations #2 and #4, females of child bearing potential must have negative urine pregnancy test results from screening and Day 0 available prior to vaccination. In Study Population #1, any female subject who is in the 1st or 2nd trimester of her pregnancy cannot participate in the study. However, any female subject who is in the 3rd trimester of a low-risk pregnancy can participate in the study.

Nasal or NP swabs will be collected from any subject who reports any symptoms associated with COVID-19 after the start of active and passive surveillance. Two swabs will be collected per subject per event (one from each nare). The swabs will be collected within 72 hours (preferably within 48 hours) of the reporting of a COVID-19-associated symptom. If swabs cannot be collected within 72 hours after reporting of the COVID-19-associated symptom, nasal or NP swabs should be collected at the next available opportunity for the subject to visit the Investigator site.

Additional serial nasal/NP/buccal swabs (self-collected) and blood sampling may be requested of subjects with virologically-confirmed COVID-19 infection.

8.3 Clinical Supplies

The study center will be provided with or be responsible for the provision of supplies for nasal or NP swabs and blood collection and shipment supplies (e.g. aliquot tubes, aliquot labels, aliquot storage, shipping boxes, and accompanying manifests) as described in the study-specific documentation. Sites will be authorized to use their own materials if agreed to by Medicago (or its designee).

9 TREATMENT COMPLIANCE

Study treatments will be prepared and administered by trained unblinded site staff via IM injection to each subject at each Investigator site. In addition, the Investigator or designated study center personnel will maintain a log of all study treatments dispensed and administered to the subject. Investigational product inventory and accountability will be managed throughout the Phase 2 and Phase 3 portions of the study by unblinded site staff (details will be provided in the study-specific documentation). If an eligible subject refuses vaccination post-randomization, the reason will be documented in the source and electronic case report form (eCRF).

10 STUDY EVALUATIONS

10.1 Study Procedures

10.1.1 Overview

During the Phase 2 and Phase 3 portions of the study, the Time and Events Schedules: General Information for each portion (see Table 1 and Table 2) summarizes the frequency and timing of scheduled assessments applicable to each portion of the study.

10.1.2 General COVID-19 Precautions at Clinical Sites

Sites participating in this study will have processes in place locally for following recommendations of the local Public Health authorities for the management of COVID-19. Subjects (and/or their parent/LAR) will be asked to follow good hygiene and safe physical distancing measures such as wearing a face mask on site, frequent hand hygiene, and maintaining a physical distance of 2 meters (or 6 feet) from others, except during procedures where staff must come into close proximity of subjects (e.g. vaccination, blood draws, collecting vital sign measurements).

Staff at the clinical sites will also follow the same good hygiene and safe physical distancing measures as the subjects at the site. In addition, the staff will be responsible for disinfecting materials and/or areas between each use by subjects and staff. All staff involved with on-site procedures will have a back-up member who is qualified to perform the same duties / responsibilities in the event that a member of the staff is infected with SARS-CoV-2 or comes

into contact with an individual known to have COVID-19 and is restricted to self-isolation and following the recommendations of the local Public Health authorities for the management of COVID-19.

10.1.3 Screening (Visits 1)

The following procedures will be performed at the initial screening visit (Visit 1) for the Phase 2 and Phase 3 portions of the study:

- Review and proceed with the signature of the ICF. The Investigator or his/her designee, will fully inform the subject (and/or their parent/LAR) of the nature and scope of the study, potential risks and benefits of participation, and the study procedures involved and will answer all questions prior to requesting the subject's (and/or their parent/LAR) signature on the ICF. The subject's (and/or their parent/LAR's) consent must be obtained prior to performing any study-related procedures; the consent process must be clearly recorded and a copy of the signed ICF retained in the source documents. A copy of the ICF must be provided to the subject (and/or their parent/LAR);
- Review the inclusion and exclusion criteria and determine the subject's eligibility to participate in this study. The eligibility must be confirmed by the Investigator after review of all procedures and findings and prior to randomization;
- Collect and review demographics (gender, date of birth, age, race, and ethnicity) and body measurements (BMI, weight [kg or lbs], and height [cm or inches]) data. BMI is to be calculated as body weight (kg) divided by the square of height (m); the BMI result will be rounded to one decimal place using the standard convention. For the measurement of body weight, subjects will be lightly clothed, without shoes;
- Collect and review medical history, including the grade of any medical conditions (medical conditions are to be graded using the same scale as for AEs; see Section 13.1.2). The medical history should record significant problems active at the time of screening and present within the preceding six months. Problems that have been clinically inactive for more than six months preceding screening, but which might alter the subject's current or future medical management, should also be noted (e.g. cancer, autoimmune disease, known mitral valve prolapse or a remote history of a seizure disorder);
- Review and record current and previous medication use (up to 30 days prior to study vaccine administration), with the following exception (refer to exclusion criterion no. 5):
  - For subjects who have been administered the following medication or treatment, review and record current and previous medication use up to the time period specified for the medication or treatment:
    - Study Populations #1, #2, and #3: cytotoxic, antineoplastic, or immunosuppressant drugs – within 36 months prior to vaccination;
    - Study Populations #1, #2, and #3: any immunoglobulin preparations or blood products, blood transfusions – within 6 months prior to vaccination;

- Perform a history- or symptom-directed physical examination. The physical examination will be performed by the Investigator or sub-Investigator;
- Perform a vital signs measurement, including resting blood pressure (BP), heart rate (HR), respiratory rate (RR), and oral temperature (OT). OT will be collected in degrees Celsius or Fahrenheit using a digital thermometer. The OT measurement should not be collected immediately following consumption of a hot or cold beverage or after smoking. The BP will be taken after the subject has been in a seated position as per the site's standard procedure. The BP should be taken by cuff (manual or automated are both acceptable). BP, HR, and RR may be repeated once if judged necessary. All measurements (including any repeats) will be recorded in the source documents. Inclusion of subjects with an out-of-range BP or HR or RR measurement will be based on the Investigator's judgment;
- For the Phase 2 portion only: collect screening blood samples for biochemistry, haematology, and serology (HIV, Hepatitis B, and Hepatitis C screening) for analysis as well as to test for SARS-CoV-2 antibodies. Sample blood collection at the screening visit should be performed under fasting conditions (approximately 12 hours) for cholesterol and triglyceride analysis;
- For the Phase 2 portion only: perform urinalysis on all subjects;
- Perform serum (Phase 2 portion) or urine (Phase 3 portion) pregnancy testing for female subjects of childbearing potential at screening.

10.1.4 Dose 1 Vaccination (Day 0) (Visit 2)

Emergency equipment must be available on site and appropriate treatment must be instituted as soon as possible in the event of anaphylaxis or any other immediate hypersensitivity reaction. The Investigator will be on-site on vaccine administration days and for the duration of the observation period (minimum of 30 minutes after vaccination) for the last subject dosed on that day. The Investigator will be available on call for the remainder of the study. A physician should be immediately available at the clinical site to administer treatment or to apply procedures for any immediate AEs/SAEs.

10.1.4.1 Prior to Vaccination

The following procedures will be performed on Day 0 (Visit 2) prior to vaccination for the Phase 2 and Phase 3 portions of the study:

- Record changes in medical history and medications and confirm that the subject continues to meet all inclusion and no exclusion criteria;
- Perform a vital signs measurement, including resting BP, HR, RR, and OT. OT will be collected in degrees Celsius or Fahrenheit using a digital thermometer. The OT measurement should not be collected immediately following consumption of a hot or cold beverage or after smoking. The BP will be taken after the subject has been in a seated position as per the site's standard procedure. The BP should be taken by cuff (manual or automated are both acceptable). BP, HR, and RR may be repeated once if judged necessary.

All measurements (including any repeats) will be recorded in the source documents. Inclusion of subjects with an out-of-range BP or HR or RR measurement will be based on the Investigator's judgment;

- Measure the BMI; for this visit, only weight will be measured while the height will be obtained from that measured at the initial screening visit. The BMI result will be rounded to one decimal place using the standard convention. For the measurement of body weight, subjects will be lightly clothed, without shoes;
- Collect a urine sample for female subjects of childbearing potential and perform a urine dipstick (or similar) pregnancy test. No study vaccine must be administered to a childbearing potential female until a negative result is obtained and documented, with the following exception:
    - Study Population #1: in the Phase 3 portion of the study, any female subject who is in the 1st or 2nd trimester of her pregnancy cannot participate in the study. However, any female subject who is in the 3rd trimester of her low-risk pregnancy can participate in the study;
- If the subject is judged eligible for the study and is still willing to participate in the study, randomize the subject into the study;
- After confirmation of eligibility and randomization, collect baseline blood samples for immunogenicity (serology [Nab assay and ELISA] and CMI) assessments (Phase 2 portion - all subjects; Phase 3 portion - immunogenicity subset); prepare and store these samples until shipment to the analytical laboratory.

10.1.4.2 Vaccination

Once all pre-vaccination procedures have been completed and subject eligibility determined, the subject will be randomized and the study vaccine will be administered IM into the deltoid muscle of the non-dominant (if possible) arm, as described in the Investigational Product Management Manual. In order to prevent possible confounding of vaccination site reactions, whenever possible, blood samples will not be collected from the same arm as the one used for vaccination. The arms used, both for blood collection and vaccination, will be documented in the source documents. For subjects < 18 years of age and adult subjects with BMI < 30 kg/m^2, a 23 gauge needle of at least one inch or 2.5 cm in length can be used for vaccination. For subjects with BMI ≥ 30 kg/m^2, a needle of at least 1.5 inches or 3.5 cm in length must be used to ensure intramuscular delivery. The preparation and administration of the study vaccine will be performed by an unblinded site staff member.

10.1.4.3 Thirty Minutes Post-vaccination

The post-vaccination observations will be performed by a blinded site staff member. The following safety observation procedures will be performed for all subjects immediately following study vaccine administration:

- Subjects will remain in the clinic for at least 30 minutes post-vaccination for observation. The observation period will include an assessment of immediate solicited local and

systemic AEs. Solicited local and systemic AEs occurring within 30 minutes post-vaccination will be recorded in the subject diary and corresponding eCRF. All unsolicited AEs occurring within 30 minutes post-vaccination will be recorded in the subject source and AE eCRF. Any unusual signs or symptoms reported during the initial 30 minutes post-vaccination will result in continued close monitoring. Based on their condition, subjects may be asked to remain in the clinic for their safety for more than 30 minutes after vaccination (the reason will be recorded in the source documents). All data (including the assessment of solicited local and systemic AEs) will be recorded in the source documents during and after the observation period. Refer to Section 10.2.1 for details regarding the assessment of AEs and/or solicited local and systemic AEs;

- During the observation period, subjects (and/or their parent/LAR) will be provided with a measurement device template (Section 19.1) for measuring (in mm) solicited local AEs of erythema (redness) and swelling and an oral digital thermometer for recording daily temperature (in degrees Celsius or Fahrenheit). Subjects (and/or their parent/LAR) will also be provided with an electronic diary (Section 19.2) and will be shown how to enter their data in the diary. Each subject (and/or their parent/LAR) will be provided with the following instructions on the measurements they are to make:
  - How to collect his/her OT in degrees Celsius or Fahrenheit with the provided digital thermometer:
    - From the evening of Day 0 to the evening of Day 7, OT will be measured at approximately the same time each evening and the results recorded in the electronic diary;
    - The OT should not be collected immediately following consumption of a hot or cold beverage or after smoking;
    - The subject is to also take his/her OT if he/she feels feverish and to record the highest temperature of the day. In the event that a temperature ≥ 38.0 °C or ≥ 100.4 °F is obtained, the subject will be allowed to take over-the-counter antipyretics (e.g. acetaminophen/paracetamol, aspirin, naproxen, or ibuprofen) and will be advised to increase the frequency of OT measurements to approximately every four hours, until he/she is no longer febrile (fever is defined as a temperature of ≥ 38.0 ºC or ≥ 100.4 °F). The subject (and/or their parent/LAR) is to document medication intake, which will be reviewed by the site personnel;
  - How to measure any solicited local AEs, including erythema (redness) and swelling diameter at the injection site using the measurement template supplied for this purpose; subjects will also be requested to evaluate pain at the injection site. Local AEs will be assessed every day starting in the evening of Day 0 and up to the evening of Day 7 and the results will be recorded. The severity of solicited local AEs will be graded according to the Food and Drug Administration (FDA) Guidance for Industry: Toxicity Grading Scale for Healthy Adult and Adolescent Volunteers Enrolled in Preventive Vaccine Clinical Trials [FDA 2007], as presented in Table 8.

    - How to grade, on a daily basis from the evening of Day 0 through to the evening of Day 7, each of the solicited systemic AEs and their severity (as per the same guidance used for solicited local AEs; see Table 8) [FDA 2007] and to record the worst grade of the day for each of these solicited systemic AE. The instructions will include how to examine and grade swelling in the neck and axilla and to record any unusual feeling and/or swelling;
    - Subjects (and/or their parent/LAR) will be advised that they will be asked about the occurrence of any symptoms or events requiring medical attention and the use of any concomitant medication during the 21-day post-vaccination period, after each vaccination, and until the end of the study. Subjects will also be provided with a memory aid (Section 19.3) to record unsolicited AEs and any concomitant medication use and will be shown how to enter their data in the memory aid;
    - Subjects (and/or their parent/LAR) will be instructed to contact the clinical site for any unsolicited AEs and/or solicited local and systemic AEs of greater than Grade 2 (moderate). Based on their condition, the Investigator may request that the subject return to the clinic for evaluation;
    - Subjects (and/or their parent/LAR) will be advised on emergency contact information and instructions for contacting study personnel. Subjects will be advised to immediately contact the Investigator (or his/her designee) in the event of an SAE or a medical emergency. Subjects (and/or their parent/LAR) will be provided with a phone contact number and be instructed to call if any suspected reaction to the vaccination is felt to be significant or of concern;
    - Subjects (and/or their parent/LAR) will be advised to notify their health care professional(s) (e.g. primary care physician) that they are participating in a clinical research study of a SARS-CoV-2 / COVID-19 vaccine;

- After the 30-minute observation period (allowed window of + 15 minutes) is completed, measure vital signs (BP, HR, RR, and OT) as described in Section 10.2.1.4. Any out-of-range measurements will be assessed by the Investigator (or his/her designee) and any further action will be determined upon his/her medical decision;
- Instruct the subjects (and/or their parent/LAR) to perform measurements of local and systemic AEs at approximately the same time each day from Day 0 to Day 7 (preferably in the evening);
- Advise each subject (and/or their parent/LAR) to report to the clinical site if he/she/their child has tested positive for COVID-19;
- Provide appointments (date and time) for the next planned visit to the clinical site (Day 3 for the Phase 2 portion or Day 21 for the Phase 3 portion) and for the Days 1 and 8 phone contacts;
- The subject will be released from the clinical site once all Day 0 post-vaccination procedures have been completed and the subject is in stable condition.

10.1.5 Day 1 and Day 8 (Telephone Contact)

The post-vaccination phone contacts will be performed by blinded site staff members. The following procedures will be performed during the phone contact (acceptable interval of + 1 day for Day 1, and ± 1 day for Day 8) for the Phase 2 and Phase 3 portions of the study:

- Ask the subjects (and/or their parent/LAR) about any difficulties in recording their data, any change in health, any visits to health care facilities and/or medical practitioners, and any use of concomitant medications. Record the information in the source documents;
- For any unsolicited AEs and/or solicited local and systemic AEs of greater than Grade 2 (moderate), the Investigator should be informed within 24 hours of the time the clinical site is made aware of the event. The Investigator may request that the subject return to the clinic for evaluation;
- Advise subjects (and/or their parent/LAR) to immediately contact the Investigator (or his/her designee) in the event of any AE that requires medical attention. Subjects (and/or their parent/LAR) will be provided with a phone contact number and be instructed to call if any suspected reaction to the vaccination is felt to be significant or of concern;
- Remind the subjects (and/or their parent/LAR) how to measure and record any solicited local and systemic AEs. Subjects (and/or their parent/LAR) should also be reminded to record any changes in health, including changes in AEs and changes in medications;
- Remind each subject (and/or their parent/LAR) to immediately report to the clinical site any symptoms associated with COVID-19 or if he/she/their child has tested positive for COVID-19, as defined in Section 10.4.1. Ensure that subjects (and/or their parent/LAR) have the memory aid listing the COVID-19 symptoms and contact information for the study site;
- Remind subjects (and/or their parent/LAR) of their next appointment (date and time) for the clinical visit and/or the next telephone contact. Subjects (and/or their parent/LAR) will also be reminded to record their data in the diary and memory aid in a timely manner.

In the event that a subject (and/or their parent/LAR) cannot be reached via phone, he/she may be contacted by text message or via email (if these contacts are available). However, the phone should be the initial and preferred means of communication. At least three attempts within allowed time window should be made in order to reach the subject (and/or their parent/LAR).

10.1.6 Day 3 Follow-up (Visit 3 for Phase 2 portion only)

The post-vaccination follow-up visit procedures will be performed by blinded site staff members at the clinical site. For subjects in quarantine, these procedures will be performed by blinded trained medical staff and may be at the subject's home (or place of quarantine). The following procedures will be performed during the Day 3 visit (- 1 day) for the Phase 2 portion of the study:

- Perform a vital signs measurement, including resting BP, HR, RR, and OT;
- Perform urinalysis on all subjects;

- Collect blood samples for biochemistry and haematology;
- Review the diary and memory aid content with the subject (and/or their parent/LAR) to ensure appropriate completion. Corrections must be made by the subject (and/or their parent/LAR) him/herself and all corrections must be initialed and dated;
- Review the subject's safety data and ensure all updates on concomitant medication usage and any changes in the subject's health (AEs, SAEs, AESIs, or MAAEs) are recorded appropriately;
- Advise the subjects (and/or their parent/LAR) to immediately contact the Investigator or his/her designee, in the event of any AE that requires a visit to an emergency room and/or hospitalization;
- Remind each subject (and/or their parent/LAR) to immediately report to the clinical site any symptoms associated with COVID-19 or if he/she/their child has tested positive for COVID-19, as defined in Section 10.4.1. Ensure that subjects (and/or their parent/LAR) have the memory aid listing the COVID-19 symptoms and contact information for the study site;
- Remind the subjects (and/or their parent/LAR) of their next telephone contact (date and time).

10.1.7 Day 21 Dose 2 Vaccination (Visit 4 for Phase 2 portion and Visit 3 for Phase 3 portion)

If for any reason the Day 21 visit is done before or after the planned date, then subsequent visits/procedures (Days 22, 24 (Phase 2 portion only), 29, 42, 201, and 386) will be adjusted accordingly.

10.1.7.1 Pre-vaccination

The pre-vaccination procedures will be performed by blinded site staff members. The following procedures will be performed during the Day 21 (± 2 days) visit prior to vaccination for the Phase 2 and Phase 3 portions of the study:

- Perform a vital signs measurement, including resting BP, HR, RR, and OT;
- Perform urinalysis on all subjects;
- Perform urine (dipstick or similar) pregnancy testing for female subjects of childbearing potential at screening. No study vaccine must be administered to a childbearing potential female until a negative result is obtained and documented, with the following exception:
  - Study Population #1: in the Phase 3 portion of the study, any female subject who is in the 1st or 2nd trimester of her pregnancy cannot participate in the study. However, any female subject who is in the 3rd trimester of her pregnancy can participate in the study;
- Phase 2 portion only: collect blood samples for biochemistry and haematology;

- Phase 2 portion (all subjects) and Phase 3 portion (immunogenicity subset): collect blood samples for immunogenicity (serology [Nab assay and ELISA] and CMI) assessments; prepare and store these samples until shipment to the analytical laboratories;
- Review the diary content with the subject (and/or their parent/LAR) to ensure appropriate completion. Review the memory aid with the subject (and/or their parent/LAR) as well. Corrections must be made by the subject (and/or their parent/LAR) him/herself and all corrections must be initialed and dated;
- Review the subject's safety data and ensure all updates on concomitant medication usage and any changes in the subject's health (AEs, SAEs, or AESIs) are recorded appropriately;
- Advise the subjects (and/or their parent/LAR) to immediately contact the Investigator or his/her designee, in the event of any AE that requires a visit to an emergency room and/or hospitalization;
- Assess the subject for contraindications to the second vaccination according to Section 11.3. If the subject has no contraindications to the second vaccination, proceed to register the visit in the interactive response technology (IRT) system;
- Register the visit in the IRT system. The IRT system will assign the same treatment assignment at Day 21 as Day 0. If the subject has a contraindication for the second vaccination, record the status according to the IRT manual.

10.1.7.2 Dose 2 Vaccination

The same procedures will be performed during the administration of the vaccine on Day 21 as will be performed during the administration of the vaccine on Day 0 (see Section 10.1.4.2 for detailed procedures) for the Phase 2 and Phase 3 portions of the study, with the following exception regarding which arm to use for IM administration:

- The study vaccine should be administered IM into the deltoid muscle of the alternate arm (i.e. the arm not used for the previous dose administration). Record the administration, left or right arm, in the subject source documents.

10.1.7.3 Post-Dose 2 Vaccination

The same procedures will be performed following the administration of the vaccine on Day 21 as will be performed following the administration of the vaccine on Day 0 (see Section 10.1.4.3 for detailed procedures) for the Phase 2 and Phase 3 portions of the study, with the following exceptions:

- Provide appointments (date and time) for the next planned visit to the clinical site (Day 24 for the Phase 2 portion or Day 42 for the Phase 3 portion) and for Days 22 and 29 telephone contacts;
- The subject will be released from the clinical site once all post-vaccination procedures have been completed and the subject is in stable condition.

10.1.8 Day 22 and Day 29 (Telephone Contact)

The same procedures will be performed during the phone contacts on Day 22 and Day 29 as were performed during phone contacts on Day 1 and Day 8 (see Section 10.1.5 for detailed procedures) for the Phase 2 and Phase 3 portions of the study.

10.1.9 Day 24 (Visit 5 for Phase 2 portion only)

The same procedure will be performed during the Day 24 visit as were performed during the follow-up visit on Day 3 (see Section 10.1.6 for detail procedures) for the Phase 2 portion of the study.

10.1.10 Surveillance for COVID-19 Cases

From 14 days following the last vaccination, passive and active surveillance will be performed until the end of the Phase 2 or until approximately 160 COVID-19 cases have been accumulated in the Phase 3 portion of the study:

- Passive surveillance: subjects (and/or their parent/LAR) will be instructed to contact the study site within 72 hours in case they experience symptoms of COVID-19, as defined in Section 10.4.1;
- Active surveillance: the subjects (and/or their parent/LAR) will be contacted once per week via the method most likely to be successful (e.g. subject's preferred method [phone, text, email, electronic diary]), and asked about symptoms of COVID-19;
- If a subject reports any symptom(s) of COVID-19:
  - Collect information regarding COVID-19, including all applicable symptoms and the onset date:
    - The onset date is defined as the first date when one or more of the symptoms occurred and persisted for at least 12 hours;
    - Symptoms collected should also include the presence or absence, severity, and duration of concurrent symptoms (i.e. fever, feverishness [feeling of warmth], chills [shivering], tiredness [fatigue], headache, myalgia [muscle aches], nausea, vomiting, or diarrhea);
  - Collect information on any associated concomitant medication use;
  - Schedule a time when the subject can return to the clinical site for the collection of nasal or NP swabs. The nasal or NP swabs are to be collected within 72 hours (preferably within 48 hours) after the reporting of a COVID-19 symptom;

    Note: If swabs cannot be collected within 72 hours after reporting of the COVID-19-associated symptom, nasal or NP swabs should be collected at the next available opportunity for the subject to visit the Investigator site and date and time of collection recorded.
  - COVID-19 cases that start on or after 14 days after the last vaccination will be followed up. This follow-up will be conducted via the planned active surveillance contacts. All

follow-up information should be collected, regardless of whether nasal or NP swabs could be obtained. As part of this follow up, the following information will be needed:

- Collect any new information regarding the evolution of the COVID-19 case, including symptoms and any associated concomitant medication use;
- Collect data regarding the end date (if resolved). The end date is defined as the date on which the last symptom resolved;

- Ensure subjects (and/or their parent/LAR) have the memory aid listing the symptoms of COVID-19 and contact information for the study site; ensure subjects use the diary or memory aid, as applicable, to record reportable information.

In the event that a subject (and/or their parent/LAR) cannot be reached via telephone, he/she may be contacted by text message or via email (if these contacts are available). However, the telephone should be the initial and preferred means of communication. At least three attempts within allowed time window should be made in order to reach the subject (and/or their parent/LAR).

10.1.11 Day 42 (Visit 6 for Phase 2 portion and Visit 4 for Phase 3 portion)

The same procedure will be performed during the Day 42 visit as were performed during the pre-vaccination portion of the visit on Day 21 (see Section 10.1.7.1 for detail procedures) for the Phase 2 and Phase 3 portions of the study, with the following exceptions:

- Review the diary and memory aid content with the subject (and/or their parent/LAR) to ensure appropriate completion. Corrections must be made by the subject (and/or their parent/LAR) him/herself and all corrections must be initialed and dated. Collect the memory aid and provide subjects (and/or their parent/LAR) with another memory aid for the collection of safety data from Day 43 to Day 201;
- Do not collect blood samples for biochemistry and haematology;
- Phase 2 portion (all subjects) and Phase 3 portion (immunogenicity subset): collect blood samples for immunogenicity (serology [Nab assay and ELISA] and CMI) assessments; prepare and store these samples until shipment to the analytical laboratory;
- Remind each subject (and/or their parent/LAR) to immediately report to the clinical site any symptoms associated with COVID-19 or if he/she/their child has tested positive for COVID-19, as defined in Section 10.4.1. Ensure that subjects (and/or their parent/LAR) have the memory aid listing the COVID-19 symptoms and contact information for the study site;
- Provide appointments (date and time) for the next telephone contacts and the next planned visit to the clinical site (Day 201).

10.1.12 Day 201 (Visit 7 for Phase 2 portion and Visit 5 for Phase 3 portion)

The post-vaccination visit procedures will be performed by blinded site staff members at the clinical site. For subjects in quarantine, these procedures will be performed by blinded trained

medical staff and may be at the subject's home (or place of quarantine). The following procedures will be performed during the Day 201 visit (± 14 days) for the Phase 2 and Phase 3 portions of the study:

- Review the memory aid content with the subject (and/or their parent/LAR) to ensure appropriate completion. Corrections must be made by the subject him/herself and all corrections must be initialed and dated. Collect manual memory aid and provide subjects (and/or their parent/LAR) with another memory aid for the collection of safety data from Day 202 to Day 386;
- Review the subject's safety data and ensure all updates on concomitant medication usage and any changes in the subject's health (AEs ongoing from Day 42, SAEs, AESIs, or MAAEs) are recorded appropriately;
- Perform a vital signs measurement, including resting BP, HR, RR, and OT;
- Phase 2 portion (all subjects) and Phase 3 portion (immunogenicity subset): collect blood samples for immunogenicity (serology [Nab assay and ELISA] and CMI) assessments; prepare and store these samples until shipment to the analytical laboratories;
- Remind each subject (and/or their parent/LAR) to immediately report to the clinical site any symptoms associated with COVID-19 or if he/she/their child has tested positive for COVID-19, as defined in Section 10.4.1. Ensure that subjects (and/or their parent/LAR) have the memory aid listing the COVID-19 symptoms and contact information for the study site;
- Provide appointments (date and time) for the next telephone contacts and the next planned visit to the clinical site (Day 386).

10.1.13 Final Visit – Day 386 (Visit 8 for Phase 2 portion and Visit 5 for Phase 3 portion)

The post-vaccination final visit procedures will be performed by blinded site staff members at the clinical site. For subjects in quarantine, these procedures will be performed by blinded trained medical staff and may be at the subject's home (or place of quarantine). The following procedures will be performed during the Day 386 visit (± 14 days) for the Phase 2 and Phase 3 portions of the study:

- Review the subject's safety data and ensure all updates on concomitant medication usage and any changes in the subject's health (AEs ongoing from Day 42, SAEs, AESIs, or MAAEs) are recorded appropriately. Collect the subject's memory aid;
- Confirm that the subject has continued to comply with protocol requirements (e.g. no use of prohibited concomitant medications, reporting of any COVID-19 symptoms);
- Perform a vital signs measurement, including resting BP, HR, RR, and OT;
- Phase 2 portion (all subjects) and Phase 3 portion (immunogenicity subset): collect blood samples for immunogenicity (serology [Nab assay and ELISA] and CMI) assessments; prepare and store these samples until shipment to the analytical laboratories.

Any subject who withdraws consent from the study will be asked to undergo Day 386 visit procedures within two weeks of withdrawal, if the subject (and/or their parent/LAR) agrees.

10.2 Safety

10.2.1 Safety Evaluations

Safety and tolerability will be evaluated by solicited local and systemic adverse events (immediate solicited adverse events within 30 minutes post-vaccination and solicited adverse events up to seven days after each vaccination), unsolicited AEs within 30 minutes post-vaccination and up to 21 days after each vaccination, SAEs, AESIs, MAAEs, and AEs leading to withdrawal up to the end of the study. In addition, events will be monitored for possible VED, hypersensitivity components, and potential immune-mediated diseases, from all reported events during the study (collected AEs, SAEs, AESIs, MAAEs, and AEs leading to withdrawal). Clinical safety methods will include repeated urine, blood chemistry, and haematology testing.

10.2.1.1 Solicited Local and Systemic Adverse Events

Subjects will be monitored for both solicited local AEs (erythema, swelling, and pain at the injection site) and solicited systemic AEs (fever, headache, fatigue, muscle aches, joint aches, chills, a feeling of general discomfort, swelling in the axilla, and swelling in the neck) from the time of each vaccination through seven days. While the subjects remain in the clinic following vaccine administration (at least 30 minutes), staff will monitor them for solicited local and systemic AEs; after release from the clinic facility, from the evening of each vaccination to the evening of the seventh day after each vaccination, the subject (and/or their parent/LAR) will measure and record the subject’s local and systemic AEs in the diary.

The intensity of the solicited local and systemic AEs will be assessed by the subject, documented in the diary, and graded as: mild (1), moderate (2), severe (3) or potentially life threatening (4) (please refer to Table 8). The causal relationship with the study vaccine of the solicited local and systemic AEs will be assessed by the Investigator (definitely not related, probably not related, possibly related, probably related or definitely related) and documented in the Investigator section of the diary; see Section 13.1.8 for a definition of these causal relationships.

The Investigator should assess solicited AEs and determine if any meet the criteria for SAE. Any solicited local or systemic AEs that meet the criteria for SAE should be reported to the Sponsor within 24 hours (Section 13.1.5) and entered as an SAE in the eCRF.

Note: Fever is also a symptom of COVID-19. Subjects (and/or their parent/LAR) should be encouraged to contact the clinical site if the subject experience fever alone or associated with other symptoms such as chills, muscle aches, or malaise within the first three days after vaccination.

Table 8 Severity Grades for Solicited Local and Systemic Adverse Events

**Symptoms**	**Severity**				
	**None**	**Grade 1 (Mild)**	**Grade 2 (Moderate)**	**Grade 3 (Severe)**	**Grade 4 (Potentially life-threatening)**
**Injection Site Adverse Events (Local Adverse Events)**					
**Erythema (redness)**	< 25 mm	25 - 50 mm	51 - 100 mm	> 100 mm	Necrosis or exfoliative dermatitis
**Swelling**	< 25 mm	25 - 50 mm and does not interfere with activity	51 – 100 mm or interferes with activity	> 100 mm or prevents daily activity	Necrosis
**Pain**	None	Does not interfere with activity	Repeated use of non-narcotic pain reliever for more than 24 hours or interferes with activity	Any use of narcotic pain reliever or prevents daily activity	Results in a visit to emergency room (ER) or hospitalization
**Solicited Systemic Adverse Events**					
**Fever (ºC or ºF)**	< 38.0 ºC<br>< 100.4 ºF	38.0 - 38.4 ºC<br>100.4 - 101.1 ºF	38.5 - 38.9 ºC<br>101.2 - 102.0 ºF	39.0 - 40.0 ºC<br>102.1 - 104.0 ºF	> 40.0 ºC<br>> 104.0 ºF
**Headache**	None	No interference with activity	Repeated use of non-narcotic pain reliever for more than 24 hours or some interference with activity	Significant; any use of narcotic pain reliever or prevents daily activity	Results in a visit to emergency room (ER) or hospitalization
**Fatigue**	None	No interference with activity	Some interference with activity	Significant; prevents daily activity	Results in a visit to emergency room (ER) or hospitalization
**Muscle aches**	None	No interference with activity	Some interference with activity	Significant; prevents daily activity	Results in a visit to emergency room (ER) or hospitalization
**Joint aches, chills, feeling of general discomfort or uneasiness (malaise), swelling in the axilla, swelling in the neck**	None	No interference with activity	Some interference with activity not requiring medical intervention	Prevents daily activity and requires medical intervention	Results in a visit to emergency room (ER) or hospitalization

10.2.1.2 Adverse Events and SARS-CoV-2 Positive Reports

All spontaneous unsolicited AEs occurring within 21 days after each vaccination will be reported in the “Adverse Event” screen in the subject’s eCRF, irrespective of intensity or whether or not they are considered to be vaccination-related. Thereafter, from the 43rd day to the end of the study, SAEs, AEs leading to withdrawal, MAAEs and AESIs will be monitored and reported in the eCRF.

The intensity of unsolicited AEs will be graded as: mild (1), moderate (2), severe (3) or potentially life threatening (4), according to the FDA Guidance for Industry [FDA 2007]. Their causal relationship with the study vaccine will be assessed by the Investigator (definitely not related, probably not related, possibly related, probably related or definitely related); see Section 13.1.8 for a definition of these causal relationships.

COVID-19 cases will be continuously monitored by an unblinded team, including an unblinded Medical Monitor, to identify an adverse imbalance of COVID-19 and/or severe COVID-19 cases between subjects who receive the vaccine or placebo. Such imbalance would trigger either the stopping or alert rules (see Section 13.1.11). The unblinded team will contact the IDMC in the event either the stopping or alert rule is met. The IDMC will review the available study data and make a recommendation to the Sponsor. Full details of the VED surveillance process can be found in the Medical Management Plan and IDMC Charter.

10.2.1.3 Clinical Laboratory Tests

Blood samples for biochemistry and hematology, HIV, Hepatitis B and Hepatitis C markers in serum and urine samples for urinalysis will be collected according to the Time and Events Schedule (see Table 1) for the Phase 2 portion of the study. Blood sample for testing for SARS-CoV-2 antibodies will be collected according to the Time and Events Schedules (see Table 2) for the Phase 2 and Phase 3 portions of the study. In addition, serum or urine samples from all females of childbearing potential in Study Populations #1, #2, and #4 for pregnancy testing will be collected. Any laboratory result outside of the testing laboratory's normal range will be classified as 'clinically significant' (CS) or 'not clinically significant' (NCS) by the site Investigator, with appropriate documentation. Any laboratory test performed at Screening with a value outside the normal range may be repeated (prior to vaccination) at the discretion of the Investigator. Repeated tests will be considered the Baseline results.

The Investigator must review the laboratory reports, document this review, and record any clinically relevant changes occurring during the study in the source documents. The tests to be performed by the laboratory are presented in Table 9.

Table 9 Clinical Laboratory Tests for the Phase 2 Portion of the Study

**Biochemistry (serum):**	
Sodium	Alkaline phosphatase
Potassium	Alaninetransferase (ALT)
Urea	Aspartatetransferase (AST)
Creatinine	Gamma glutamyltransferase (GGT)
Glucose	Cholesterol (total, HDL, LDL)
Bilirubin (total)	Triglyceride
Albumin	Chloride
Total protein	Calcium
	Phosphorus

**Haematology:**	
Haemoglobin<br>Hematocrit<br>Red blood cells<br>Platelets<br>Mean platelet volume (MPV)<br>White cell count (total, WBC)<br>Neutrophils	Mean cell haemoglobin (MCH)<br>Mean cell concentration (MCHC)<br>Mean cell volume (MCV)<br>Lymphocytes<br>Monocytes<br>Eosinophils<br>Basophils
**Serology:**	
HIV<br>Hepatitis C	Hepatitis B
**Urinalysis:**	
Macroscopic examination (color, aspect)<br>pH<br>Specific gravity	Glucose<br>Protein<br>Blood

Other clinical laboratory tests include a test to measure SARS-CoV-2 antibodies at screening for all subjects in all Study Populations, and a serum (Phase 2 portion) or urine (Phase 3 portion) pregnancy test at screening and a urine (dipstick or similar) pregnancy test prior to randomization on Day 0 and on Days 21 and 42 for all female subjects of childbearing potential in Study Populations #1, #2, and #4.

All protocol required safety laboratory parameters are defined in study-specific documentation.

10.2.1.4 Vital Signs

Vital signs measurements (resting BP, HR, RR, and OT) will be performed as part of screening procedures, prior to vaccination (on Day 0 and Day 21), and after the post-vaccination 30-minute surveillance period (and repeated if deemed necessary by the Investigator). In addition, vital signs measurements will be performed at the clinic site visits on Day 3 (Phase 2 portion only), Day 24 (Phase 2 portions only), Day 42, Day 201, and Day 386 for the Phase 2 and Phase 3 portions of the study.

OT will be collected in degrees Celsius or Fahrenheit using a digital thermometer by the Sponsor. The OT measurement should not be collected immediately following consumption of a hot or cold beverage or after smoking.

BP will be taken after the subject has been in a seated position as per the site's standard procedure. BP should be taken by cuff (manual or automated are both acceptable). BP, HR and RR may be repeated once if judged necessary. All measurements (including any repeats) will be recorded in the source documents.

10.2.1.5 Physical Examinations

A history- or symptom-directed physical examination will be performed by the Investigator (or sub-Investigator) as part of screening procedures.

10.2.2 Pregnancy

Female subjects who become pregnant during the Phase 2 or Phase 3 portions of the study or are in the 3rd trimester of their pregnancy (Study Population #1 only) during the Phase 3 portion of the study will be followed for safety. The Investigator, or his/her designee, will collect pregnancy information on any subject who becomes pregnant or is pregnant while participating in this study. The Investigator or his/her designee will record pregnancy information on the Pregnancy Report Form (for the template of the form, refer to study documentation) and submit it to the Sponsor Safety Contact (see Section 13.1.5) within 24 hours of learning of a subject's pregnancy post-first vaccination. The subject will be followed to determine the outcome of the pregnancy. At the end of the pregnancy, whether full-term or premature, information on the status of the mother and child will be forwarded to the Sponsor Safety Contact, if available. Generally, follow-up will be no longer than eight weeks following the estimated delivery date.

While pregnancy itself and elective termination of a pregnancy for non-medical reasons are not considered to be an AE/SAE, any pregnancy complication or elective termination of a pregnancy for medical reasons will be recorded in the Pregnancy Report Form or as an SAE and will be followed. A spontaneous abortion is always considered to be an SAE and will be reported as such. Furthermore, any SAE occurring as a result of a post-study pregnancy and considered reasonably related in time to receipt of the investigational product by the Investigator, will be reported to the Sponsor Safety Contact. While the Investigator is not obligated to actively seek this information from former study subjects, he/she may learn of a pregnancy through spontaneous reporting. Information on pregnancies identified during the screening phase/prior to vaccine administration need to be collected for female subjects in the 3rd trimester of their pregnancy in Study Population #1 for the Phase 3 portion of the study.

10.2.3 Safety Endpoints

10.2.3.1 Primary Endpoints

In the Phase 2 portion, the primary safety endpoints are:

- Occurrence, intensity, and relationship to vaccination of immediate AEs (30 minutes after each vaccination);
- Occurrence, intensity, and relationship to vaccination of solicited local and systemic AEs (for seven days following each vaccine administration);
- Occurrence, intensity, and relationship of unsolicited AEs for 21 days following each vaccine administration;
- Number and percentage of subjects with normal and abnormal clinically significant urine, haematological and biochemical values prior to and three days following each vaccination;

- Occurrences of SAEs, MAAEs, AEs leading to withdrawal, AESIs (including VED), and deaths up to 21 days following each vaccine administration.

10.2.3.2 Secondary Endpoints

In the Phase 2 portion, the secondary safety endpoints are:

- Occurrences of SAEs, MAAEs, AEs leading to withdrawal, AESIs (including VED), and deaths from Day 43 to Day 201;
- Occurrences of SAEs, MAAEs, AEs leading to withdrawal, AESIs (including VED), and deaths from Day 202 to Day 386.

In the Phase 3 portion, the secondary safety endpoints are:

- Occurrence, intensity, and relationship to vaccination of immediate AEs (30 minutes after each vaccination);
- Occurrence, intensity, and relationship to vaccination of solicited local and systemic AEs (for seven days following each vaccine administration);
- Occurrence, intensity, and relationship of unsolicited AEs for 21 days following each vaccine administration;
- Occurrences of SAEs, MAAEs, AEs leading to withdrawal, AESIs (including VED), and deaths up to 21 days following each vaccine administration;
- Occurrences of SAEs, MAAEs, AEs leading to withdrawal, AESIs (including VED, and deaths from Day 43 to Day 201;
- Occurrences of SAEs, MAAEs, AEs leading to withdrawal, AESIs (including VED), and deaths from Day 202 to Day 386.

10.2.3.3 Exploratory Endpoints

In the Phase 2 and Phase 3 portions, the exploratory safety endpoint is:

- Further characterization of the safety profile of the CoVLP formulation, if it is likely to be informative.

10.3 Immunogenicity

10.3.1 Immunogenicity Evaluations

Immunogenicity will be evaluated by the humoral immune response (Nab assay and IgG ELISA) and the CMI response induced in subjects on Days 0, 21, 42, 201, and 386 in all subjects in the Phase 2 portion and only in a subset of subjects in the Phase 3 portion of the study.

The blood samples for immunogenicity will be analyzed in one or more central laboratories; information on processing and the central laboratories will be provided in the study-specific documentation.

10.3.2 Immunogenicity Endpoints

Point estimates and 95 % CI will be calculated for all immunogenicity endpoints and responses for the treatment groups will be compared using descriptive statistics.

10.3.2.1 Primary Endpoints

In the Phase 2 portion, the primary immunogenicity endpoints are:

- Nab response induced in each Study Population against the SARS-CoV-2 virus on Days 0, 21, and 42 will be analyzed using the following parameter: GMT, SC rate, and GMFR;
- Specific Th1 CMI response induced in each Study Population against the SARS-CoV-2 virus on Days 0, 21, and 42, as measured by IFN-γ ELISpot.

10.3.2.2 Secondary Endpoints

In the Phase 2 portion, the secondary immunogenicity endpoints are:

- Nab antibody response induced in each Study Population against the SARS-CoV-2 virus on Day 201 and Day 386 will be analyzed using the following parameters: GMT, SC rate, and GMFR;
- Specific antibody response induced in each Study Population against the SARS-CoV-2 virus will be measured by the total IgG levels on Days 0, 21, 42, 201, and 386 and analyzed using the following parameters: GMT, SC rate, and GMFR;
- The ratio of neutralizing antibody titers:IgG ELISA antibody titers at Day 21, Day 42, Day 201, and Day 386;
- Specific Th1 CMI response induced in each Study Population against the SARS-CoV-2 virus on Day 201 and Day 386, as measured by IFN-γ ELISpot;
- Specific Th2 CMI response induced in each Study Population against the SARS-CoV-2 virus on Days 0, 21, 42, 201, and 386, as measured by IL-4 (ELISpot).

In the Phase 3 portion, the secondary immunogenicity endpoints are:

- Nab response induced in each Study Population against the SARS-CoV-2 virus on Days 0, 21, and 42 will be analyzed using the following parameter: GMT, SC rate, and GMFR;
- Specific Th1 CMI response induced in each Study Population against the SARS-CoV-2 virus on Days 0, 21, and 42, as measured by IFN-γ ELISpot.

10.3.2.3 Exploratory Endpoints

In the Phase 2 portion, the exploratory immunogenicity endpoint is:

- Further characterization of the immune response of the CoVLP formulation, if it is likely to be informative.

In the Phase 3 portion, the exploratory immunogenicity endpoints are:

- Nab antibody response induced in each Study Population against the SARS-CoV-2 virus on Days 201, and 386 analyzed, in a subset of subjects, using the following parameters: GMT, SC rate, and GMFR;
- Specific antibody response induced in each Study Population against the SARS-CoV-2 virus measured by the total IgG levels on Days 0, 21, 42, 201, and 386, in a subset of subjects, and analyzed using the following parameters: GMT, SC rate, and GMFR;
- The ratio of neutralizing antibody titers:IgG ELISA antibody titers at Day 21, Day 42, Day 201, and Day 386 in a subset of subjects;
- Specific Th1 CMI response induced in each Study Population against the SARS-CoV-2 virus on Days 201, and 386, as measured by IFN-γ ELISpot in a subset of subjects;
- Specific Th2 CMI response induced in each Study Population against the SARS-CoV-2 virus on Days 0, 21, 42, 201, and 386, as measured by IL-4 (ELISpot) in a subset of subjects;
- Specific CMI response induced in each Study Population against the SARS-CoV-2 virus up to 21 days after the last vaccination and at Day 201 and Day 386, as measured by the percentage of CD4+ T cells expressing functional markers in a subset of subjects;
- Further characterization of the immune response of the CoVLP formulation in a subset of subjects, if it is likely to be informative.

10.4 Efficacy

10.4.1 Efficacy Evaluations

Following the first vaccination, each subject (and/or their parent/LAR) will be asked to report symptoms associated with COVID-19 from 14 days after the last vaccination until the end of the Phase 2 portion or until the required number of COVID-19 cases have been collected in order to evaluate the primary efficacy endpoint in the Phase 3 portion of the study (through active or passive surveillance). COVID-19 will be defined as the presence of a laboratory-confirmed (virologic method) SARS-CoV-2 infection with the occurrence of a new onset of one or more of the following symptoms that persist(s) for or reoccur(s) after a period of at least 12 hours:

- Fever or chills;
- Cough;
- Shortness of breath or difficulty breathing;
- Fatigue;
- Muscle or body aches;
- Headache;
- New loss of taste or smell;
- Sore throat;
- Congestion or runny nose;
- Nausea or vomiting;
- Diarrhea.

The symptoms listed above apply to the adult populations, and will also apply to children and adolescents with the following addition:

Additional symptoms:

- Abdominal pain;
- Poor appetite.

Subjects who report any symptoms associated with COVID-19 after the start of active and passive surveillance will be requested to visit the Investigator site to provide nasal or NP swabs (2 per subject per event) to test for COVID-19. At the visit, subjects will be trained on daily recording of their symptoms using a COVID-19 diary and collecting self-administered nasal/NP/buccal swabs for testing using a virologic method.

The swabs will be collected within 72 hours (preferably within 48 hours) of the reporting of a COVID-19-associated symptom. If swabs cannot be collected within 72 hours after reporting of the COVID-19-associated symptom, nasal or NP swabs should be collected at the next available opportunity for the subject to visit the Investigator site. One of the swabs will be submitted for analysis using a virologic method while the other swab will be kept as a back-up. All subjects with virologically-confirmed SARS-COV-2 infection will be asked to keep a detailed daily record of their symptoms and provide serial (every other day) self-administered nasal/NP/buccal swabs for testing, using a virologic method, until symptoms resolution.

10.4.2 Efficacy Endpoints

10.4.2.1 Primary Endpoints

In the Phase 3 portion, the primary efficacy endpoint is:

- First occurrence, in a subject, of laboratory-confirmed (virologic method) symptomatic SARS-CoV-2 infection (≥ 14 days post-last vaccination).

10.4.2.2 Secondary Endpoints

In the Phase 2 portion, the secondary efficacy endpoints are:

- First occurrence, in a subject, of laboratory-confirmed (virologic method) symptomatic SARS-CoV-2 infection (≥ 14 days post-last vaccination);
- Occurrences of severe COVID-19 disease (≥ 14 days post-last vaccination). Refer to the definition of severe COVID-19 disease in Section 2.2.

In the Phase 3 portion, the secondary efficacy endpoints are:

- Occurrences of severe COVID-19 disease (≥ 14 days post-last vaccination). Refer to the definition of severe COVID-19 disease in Section 2.2;
- Occurrence and intensity of COVID-19-related symptoms up to resolution of the symptoms.

10.4.2.3 Exploratory Endpoint

In the Phase 2 portion, the exploratory efficacy endpoint is:

- Occurrence and intensity of COVID-19-related symptoms up to resolution of the symptoms.

11 SUBJECT COMPLETION/WITHDRAWAL

11.1 Temporary Contraindications

An exclusion criterion that renders subjects ineligible for Phase 2 or Phase 3 portions of the study may be temporary in nature:

- Acute disease defined as presence of any moderate or severe acute illness with or without a fever within 48 hours prior to vaccination.

If, on the day of randomization, a subject is considered ineligible due to this "temporary contraindication", the subject should be considered as a screening failure. Following the resolution of such conditions, a subject may be rescreened (including the informed consent process) under a new number and, if considered eligible by the Investigator, be enrolled in the study.

11.2 Screening Failures

Screening failures are subjects who have signed (or whose parent/LAR has signed) the study-specific ICF but are not eligible for enrollment (subjects who were not randomized) in the Phase 2 or Phase 3 portions of the study, due to failure on one or more of the inclusion or exclusion criteria or because the subject (and/or their parent/LAR) withdrew consent prior to randomization.

Recording of screening failures documented in the study records maintained at the participating clinical sites and recorded in the IRT system. Screen failures will not receive a safety follow-up. Any subjects who are considered as a screening failure should be indicated as such. A screening failure subject can be rescreened (under a new number). If the subject is rescreened (including the informed consent process), a new subject number will be allocated.

11.3 Contraindications for Subsequent Vaccination

The following events constitute absolute contraindication to the further administration of the study treatments at Day 21; if any of these events occur during the study, the subject will not receive an additional dose of vaccine but will continue with the Day 201 scheduled visit (see Section 10.1.12) and the Day 386 scheduled visit (see Section 10.1.13) at the discretion of the Investigator:

- Pregnancy, with the exception of female subjects in the 3rd trimester of a low-risk pregnancy in Study Population #1 for the Phase 3 portion (see Section 10.2.2);
- Anaphylaxis in response to first vaccine administration;

- Have a laboratory-confirmed SARS-CoV-2 infection;
- Acute disease within 48 hours prior to vaccination (acute disease is defined as presence of any moderate or severe acute illness with or without a fever);
- Suspected or confirmed pIMD;
- Experience a possibly related, probably related, or definitely related SAE or Grade 3 or higher related AE that cannot be clearly attributed to another cause. The Investigator's opinion to administer the second dose of the vaccine will be presented to the IDMC for advice;
- Discovery of any health condition which, in the Investigator's opinion, may place the subject at increased risk from receipt of further vaccination; or discovery of a change in the subject's status which may render him/her unable to comply with protocol-mandated safety follow-up. If an AE occurred prior to or at the time of the Day 21 scheduled visit, the subject may be discontinued at the discretion of the Investigator or the subject may be vaccinated at a later date, however, within the time window specified in the protocol (see Section 10.1.7.1). The subject must be followed until resolution of the event as with any AE (see Section 13.1.2).

11.4 Removal of Subjects from Treatment or Assessment

Subjects will be advised that they are free to withdraw from the Phase 2 or Phase 3 portion of the study at any time without prejudice to their future medical care by the physician or the institution. Subjects who withdraw or are withdrawn from the Phase 2 or Phase 3 portion of the study after vaccination will not be replaced.

Every reasonable effort will be made to ensure that each subject complies with the protocol and completes all study visits. However, a subject may withdraw or be withdrawn from participation in the Phase 2 or Phase 3 portion of the study if:

- The subject (and/or their parent/LAR) withdraws consent;
- The subject is lost to follow-up;
- The subject is incarcerated or incapacitated during the conduct of the clinical study;
- The subject has moved away from the study area and can no longer fulfill the terms of their participation in the clinical study;
- The subject displays non-compliance to the terms of their participation in the clinical study (based on Investigator's or Medicago's [or its designee's] opinion);
- The Investigator has lost confidence in the subject's ability to adhere to the terms of their participation in the clinical study (based on Investigator's opinion);
- Safety reasons as judged by the Investigator and/or Medicago (or its designee);
- Medicago or the regulatory authority(ies) terminates the clinical study.

Withdrawal subjects will be those who leave the Phase 2 or Phase 3 portion of the study before Day 386, for whatever reason; withdrawal subjects will not be replaced. Any subject who

withdraws consent (or whose parent/LAR has withdrawn consent) from the Phase 2 or Phase 3 portion of the study will be asked to visit the clinical site within two weeks of withdrawal, if the subject (and/or their parent/LAR) agrees. The procedures performed for the final assessment will comprise of those for the Day 386 visit, if permitted by the subject (and/or their parent/LAR). All withdrawal subjects must be reported to Medicago (and/or its designee). The reason for withdrawal should be documented in the subjects' records and in the appropriate section of the eCRF. The eCRF must be completed up to and including the time of the drop-out/final assessment.

A subject may be considered discontinued from treatment if the subject does not receive both vaccine administrations (i.e. second vaccine administration is not completed; see Section 11.3 for details). A subject discontinuation from the treatment may not necessarily be discontinued from the Phase 2 or Phase 3 portion of the study as further study procedures and follow-up may be performed (safety and immunogenicity), if permitted by the subject (and/or their parent/LAR). All discontinued from treatment subjects must be reported to Medicago (and/or its designee). The reason for discontinuation from treatment should be documented in the subjects' records and in the appropriate section of the eCRF. The eCRF must be completed up to and including the time of the drop-out/final assessment.

11.4.1 Follow-up of Discontinuations

All subjects who receive a study vaccine will be followed for safety until the end of the Phase 2 or Phase 3 portion of the study, if permitted by the subject (and/or their parent/LAR).

11.4.2 Lost to Follow-up Procedures

Every attempt will be made to contact study subjects (and/or their parent/LAR) who are lost to follow-up. At least three contacts will be attempted and recorded in the source documents. As a last resort, one registered letter requesting contact with the site will be sent to any subject (and/or their parent/LAR) with whom the clinic staff no longer has contact. All attempts at contact will be documented in the subject's source documents.

11.5 Interruption of the Study

The Investigator (in consultation with Medicago [or its designee]), Medicago, or Regulatory Authorities may interrupt or terminate the Phase 2 or Phase 3 portions of the study for any reason. In addition, the IDMC may interrupt or halt the Phase 2 or Phase 3 portions of the study for safety reasons (refer to Section 13.1.11). The Investigator will immediately, on discontinuance of the Phase 2 or Phase 3 portions of the clinical study at the clinical site, inform both the study subjects (and/or their parent/LAR) and the Institutional Review Board (IRB) responsible for the study of the discontinuance, provide them with reasons for the discontinuance and advise them in writing of any potential health risks to the subjects themselves or to other persons. It is Medicago's (or its designee's) responsibility to report discontinuance of the Phase 2 or Phase 3 portions of the study to the local regulatory agencies within the appropriate timeframe, providing them with the reasons for the discontinuance and advising them in writing

of any potential health risks to the study subjects or to other persons. Medicago (or its designee) must then inform the Investigator that the appropriate notifications were done.

12 STATISTICAL METHODS

A general description of the statistical methods used to analyze the safety and immunogenicity data is outlined below. Complete details will be provided in the SAP, which will be finalized prior to database lock.

All descriptive and inferential statistical analyses will be performed using Statistical Analysis System® (SAS®) software (version 9.4 or higher).

12.1 Analysis Populations

12.1.1 Safety Analysis Set

The SAS is defined as all subjects who received at least one dose of either the CoVLP formulation or placebo. All safety analyses will be performed using the SAS and according to actual vaccination received. More particularly, safety data for subjects will be analyzed after each vaccination (Day 0-Day 21 period) according to the last vaccine they actually received, and after any vaccination (Day 0-Day 21 after last vaccination period/Day 0-End of study period) according to the actual vaccine received at the first dose.

12.1.2 Intention-To-Treat Set

The ITT set will consist of all subjects who were randomized in the study. Subjects who received the wrong treatment will be analyzed as randomized.

12.1.3 Per Protocol Set

The PP set will consist of a subset of the ITT set who participated in the study until the end of the surveillance period in the Phase 2 or Phase 3 portions of the study; had no major protocol deviations related to subject eligibility, the ability to develop a valid immune response, prohibited medication use, or the efficacy analyses; and who received either the CoVLP formulation or placebo. Major protocol deviations will be identified and documented during a blinded data review prior to database lock and confirmed at the time of database lock. The PP set will be the primary analysis population for the efficacy endpoints. Subjects who received the wrong treatment, but for whom the treatment received can be unequivocally confirmed, will be analyzed as treated, provided they have no other deviations that compromise their data.

The analyses of all efficacy endpoints will be performed using the PP set and the ITT set.

12.1.4 Immunogenicity Per Protocol Set

The IPP set will consist of a subset of the ITT set who participated in the immunogenicity portion of the Phase 2 or Phase 3 portions of the study and completed the study with no major deviations related to subject eligibility, the ability to develop a valid immune response, prohibited medication use, or the immunogenicity analyses; and who received either the CoVLP

formulation or placebo. For the Day 21 analysis, this should include the subjects who received the first vaccine dose and had Day 0 and Day 21 immunogenicity sample collections. For the Day 42 analysis, this should include the subjects who received both vaccine doses and had Day 0 and Day 42 immunogenicity sample collections. For the Day 201 analysis, this should include the subjects who received both vaccine doses and had Day 0 and Day 201 immunogenicity sample collections. For the Day 386 analysis, this should include the subjects who received both vaccine doses and had Day 0 and Day 386 immunogenicity sample collections. Subjects who had blood samples for immunogenicity taken outside of the time window for blood sample collection are to be excluded from the IPP set for the specific visit. Subjects who received the wrong treatment at the first vaccination, but for whom the treatment received can be unequivocally confirmed, will be analyzed as treated, provided they have no other deviations that compromise their data. Subjects who receive a treatment at the second vaccination that is not the same treatment received at the first vaccination will not be included in the IPP set at Day 42, Day 201, and Day 386; the subjects will remain in the Day 21 analysis and analyzed as treated.

The analyses of all immunogenicity endpoints will be performed using the IPP set, as the primary analysis population, and the immunogenicity ITT set, as a secondary analysis population.

12.2 Sample Size Determination

Overall, up to a maximum of 30 480 subjects are planned for randomization in the Phase 2 and Phase 3 portion of the study in total.

For the Phase 2 portion, the sample size of up to 480 subjects will make it possible to evaluate vaccine immunogenicity and detect sizable differences in rates of adverse events. The sample size is not large enough to detect less frequent or rare adverse reactions. With 384 subjects receiving the CoVLP formulation, there will be at least 95 % probability of observing an AE that occurs as frequently as 1 in 128 vaccinees.

For the Phase 3 portion, the sample size of up to 30 000 subjects has been calculated using an exact conditional Poisson approach based on the following assumptions:

- A 1:1 ratio for CoVLP formulation:placebo;
- Assumed vaccine efficacy of the CoVLP formulation, compared to placebo, of 60 %;
- At least 90 % statistical power to obtain an observed absolute vaccine efficacy of the CoVLP formulation, compared to placebo, of at least 50 % with an appropriately alpha-adjusted 95 % confidence interval lower limit greater than 30 %;
- A SARS-CoV-2 attack rate of 0.8 %;
- An attrition of 5 %;
- An interim analysis for an early efficacy assessment will be performed when 80 laboratory-confirmed COVID-19 cases have been collected in the Phase 3 portion;
- A statistical correction for the interim analysis will be applied that uses the O'Brien-Fleming alpha-spending function. With interim and final assessments at 80 and 160 cases, for example, the two-sided test p-value thresholds will be 0.0027 and 0.0478, respectively.

With 15 000 subjects receiving the CoVLP formulation, there will be at least 95 % probability of observing an AE that occurs as frequently as 1 in 5000 vaccinees.

An immunogenicity subset sample size of 384 subjects is deemed sufficient to provide meaningful statistical assessment of immunologic outcomes.

12.3 Day 21 and Possibly Day 42 Immunogenicity Data Analysis and Interim Efficacy Analyses

For the Phase 2 portion, the unblinded statistical team will perform immunogenicity data analysis after Day 21 (last subject) in each of the Study Populations. The data analysis will involve selected individuals from the unblinded statistical team and Medicago who will be unblinded (see Section 6.2). This analysis will confirm that an acceptable immune response has been induced in each Study Population prior to the initiation of the Phase 3 portion of the study, without having to wait until after the end of the 386 day follow-up period for study completion. In the event that the Day 21 NAb response in any of the Study Populations is <50% of that achieved by healthy adults, then a similar data analysis will be performed after Day 42 (last subject) for the Study Populations with the lower Nab response in order to initiate the Phase 3 portion of the study for the particular Study Population. However, if at Day 42 the Nab response for a Study Population is still <50 % of that achieved by healthy adults, an ad hoc IDMC meeting will be convened to consider changes to the Protocol to permit alternate vaccination strategies in the implicated Study Population(s).

For the Phase 3 portion, when 80 laboratory-confirmed COVID-19 cases have been collected, the unblinded statistical team will perform a first interim analysis of the efficacy data in order to test for early vaccine efficacy. A second interim analysis for the Phase 3 portion will be performed as soon as 160 cases are collected in order to perform the final analysis and produce final efficacy outputs.

12.4 Initiation of the Phase 3 Portion

The start of the Phase 3 portion of the study will be determined independently for each Study Population and will be based on the outcomes of the following two assessments during the Phase 2 portion of the study:

- Day 21 Data Analysis of the immunogenicity data (refer to Section 12.3);
- Day 28 Safety Review (refer to Section 13.1.11).

If the Day 21 immunogenicity data and Day 28 safety data results are favorable and show acceptable safety and immune responses for Study Populations #3 and #4, assuming the results in Study Population #1 will be favorable, then the Phase 3 portion of the study can start for all three Study Populations.

As mentioned above, if the Day 21 immune response is not acceptable in one or both of Study Populations #3 and #4, then the Day 42 immunogenicity data will be assessed in the applicable Study Population(s) for the purposes of determining whether the Phase 3 portion may be started for that/those Study Population(s).

12.5 Safety Analyses

The safety endpoints are defined in Section 10.2.3.

The original terms used in the eCRFs by Investigators to identify AEs will be coded using the Medical Dictionary for Regulatory Activities (MedDRA®). All eCRF reported AEs with onset post-vaccination will be included in the safety analyses.

Special attention will be given to those subjects who die, who discontinue from the study due to an AE, who experience an SAE (e.g. summaries, listings, and narrative preparation may be provided, as appropriate), or who experience an AESI (see Section 13.1.3).

Safety summary tables will present data for the overall population and for each age stratum according to each Study Population, as applicable.

12.5.1 Analysis of Primary Endpoints

The primary safety endpoints are defined in Section 10.2.3.1.

For the Phase 2 portion, immediate AEs (30 minutes after each vaccination) and solicited local and systemic AEs reported up to seven days after each vaccination will be summarized by treatment using descriptive statistics. In addition, all unsolicited AEs, SAEs, AEs leading to subject withdrawal, AESIs, MAAEs, and deaths reported up to 21 days after each vaccination will be summarized by treatment using descriptive statistics.

Laboratory (biochemical, haematological, and urine) data will be summarized by type of laboratory test. Reference ranges and markedly abnormal results will be used in the summary of laboratory data. Descriptive statistics will be calculated for each laboratory test at Baseline and at each scheduled timepoint (Day 3, Day 21, and Day 24). Changes from Baseline results will be presented in pre- versus post-treatment cross tabulations (with classes for below, within, and above normal ranges).

12.5.2 Analysis of Secondary Endpoints

The secondary safety endpoints are defined in Section 10.2.3.2.

For the Phase 3 portion, immediate AEs (30 minutes after each vaccination) and solicited local and systemic AEs reported up to seven days after each vaccination will be summarized by treatment using descriptive statistics. In addition, all unsolicited AEs, SAEs, AEs leading to subject withdrawal, AESIs, MAAEs, and deaths reported up to 21 days after each vaccination will be summarized by treatment using descriptive statistics.

For the Phase 2 and Phase 3 portions, SAEs, AEs leading to subject withdrawal, AESIs, MAAEs, and deaths reported from Day 43 to the end of the study will be summarized by treatment using descriptive statistics.

For the following categories, AE information will also be presented by gender (male and female) and race (Caucasian or White; Black or African American; Asian):

- Immediate solicited and unsolicited AEs up to 30 minutes after each vaccination;

- Solicited local and systemic AEs within first seven days after each vaccination;
- Most frequent unsolicited AEs within 21 days after vaccination;
- AESIs;
- SAEs;
- MAAEs;
- AEs leading to death;
- AEs leading to withdrawal.

12.5.3 Analysis of Exploratory Endpoint

The exploratory safety endpoint is defined in Section 10.2.3.3.

For the Phase 2 and Phase 3 portions, further characterization of the safety profile of the CoVLP formulation will be done and the results presented, if it is likely to be informative.

12.6 Immunogenicity Analyses

Immunogenicity endpoints are described in Section 10.3.2.

All descriptive and inferential statistical analyses will be performed using SAS® software (version 9.4 or higher). Details of the statistical analyses are provided in the SAP.

Immunogenicity summary tables will present data for:

- All subjects according to Study Population;
- Subjects who are seropositive at baseline for SARS-CoV-2 antibodies according to Study Population;
- Subjects who are seronegative at baseline for SARS-CoV-2 antibodies according to Study Population;
- Each of the different age strata according to Study Population, as applicable.

12.6.1 Analysis of Primary Endpoints

The primary immunogenicity endpoints are defined in Section 10.3.2.1.

For the Phase 2 portion, the following analyses for the NAb assay will be performed on the IPP set:

- GMT (Day 0, Day 21, and Day 42): The point estimates and the corresponding two-sided 95 % CI by treatment group will be calculated as the antilog of the mean and 95 % CI of log transformed titer values;
- SC rate (Day 0, Day 21, and Day 42): The point estimates and the corresponding two-sided 95 % CI for subjects achieving SC by treatment group will be calculated and reported;
- GMFR: the geometric mean of the ratio of GMTs (Day 21/Day 0 and Day 42/Day 0).

The GMFR will be derived by using ANCOVA to model the difference in the log of the titer values between Day 21 and Day 0 and between Day 42 and Day 0, with treatment group as main effect and baseline titer as covariate.

GMT will be compared between treatment groups using the analysis of variance (ANOVA) model. GMFR will be compared using the ANCOVA model. For SC rate, Fisher's exact tests will be used to compare between the treatment groups.

The specific Th1 CMI response induced on Day 0, Day 21, and Day 42 will be measured by the number of T cells expressing IFN-γ, using ELISpot, for each treatment group. The specific Th2 CMI response induced on Day 0, Day 21, and Day 42 will be measured by the number of T cells expressing IL-4, using ELISpot, for each treatment group. The response will be compared between treatment groups and timepoints using appropriate non-parametric (Wilcoxon) models. Additional details will be provided in the SAP.

12.6.2 Analysis of Secondary Endpoints

The secondary immunogenicity endpoints are defined in Section 10.3.2.2.

For the Phase 2 portion: the following analyses for the NAb assay will be performed on the immunogenicity PP set:

- GMT (Days 201 and 386): The point estimates and the corresponding two-sided 95 % CI by treatment group will be calculated as the antilog of the mean and 95 % CI of log transformed titer values;
- SC rate (Days 201 and 386): The point estimates and the corresponding two-sided 95 % CI for subjects achieving SC by treatment group will be calculated and reported;
- GMFR: the geometric mean of the ratio of GMTs (Day 201/Day 0 and Day 386/Day 0).

For the analyses of the total IgG antibody response, the GMT and SC rate will be calculated on Day 0, Day 21, Day 42, Day 201, and Day 386 as well as the GMFR (Day 21/Day 0, Day 42/Day 0, Day 201/Day 0, and Day 386/Day 0).

The GMFR will be derived by using ANCOVA to model the difference in the log of the titer values between Day 21 and Day 0, between Day 42 and Day 0, Day 201 and Day 0, and Day 386 and Day 0 with treatment group as main effect and baseline titer as covariate.

GMT will be compared between treatment groups using the analysis of variance (ANOVA) model. GMFR will be compared using the ANCOVA model. For SC rate, Fisher's exact tests will be used to compare between the treatment groups.

The Nab/IgG ratio will be calculated as the antilog of the Nab/IgG ratio and 95 % CI of log transformed titer values. The ratio will be compared between treatment groups using ANOVA.

The specific Th1 CMI response induced on Day 201 and Day 386 will be measured by the number of T cells expressing IFN-γ, using ELISpot, for each treatment group. The specific Th2 CMI response induced on Day 201 and Day 386 will be measured by the number of T cells expressing IL-4, using ELISpot, for each treatment group. The responses will be compared

between treatment groups and timepoints using appropriate non-parametric (Wilcoxon) models. Additional details will be provided in the SAP.

For the Phase 3 portion, the same immunogenicity analyses will be performed as described for the primary immunogenicity endpoints for the Phase 2 portion of the study.

12.6.3 Analysis of Exploratory Endpoints

The exploratory immunogenicity endpoints are defined in Section 10.3.2.3.

For the Phase 2 portion, if deemed necessary, further characterization of the immune response of the CoVLP formulation will be done and the results presented.

For the Phase 3 portion, the same immunogenicity analyses will be performed as described for the secondary immunogenicity endpoints for the Phase 2 portion of the study.

In addition, the CMI response induced on Day 0, Day 21, Day 42, Day 201, and Day 386 will be measured by the percentage of T cells expressing functional markers, using flow cytometry, for each treatment group. The responses will be compared between treatment groups and timepoints using appropriate non-parametric (Wilcoxon) models. Additional details will be provided in the SAP. If deemed necessary, further characterization of the immune response of the CoVLP formulation will be done and the results presented.

12.7 Efficacy Analyses

Efficacy endpoints are described in Section 10.4.2.

All descriptive and inferential statistical analyses will be performed using Statistical Analysis System® (SAS®) software (version 9.4 or higher). Details of the statistical analyses are provided in the SAP.

Efficacy summary tables will present data for the overall population and for each age stratum according to each Study Population, as applicable.

12.7.1 Analysis of Primary Endpoint

The primary efficacy endpoint is defined in Section 10.4.2.1.

For the Phase 3 portion of the study, once approximately 160 COVID-19 cases have accumulated during the study, the primary efficacy endpoint of the Phase 3 portion of the study will be evaluated to determine if the vaccine efficacy success criterion has been met. For the efficacy endpoints, absolute VE will be evaluated. An interim analysis will be performed when 80 cases have been collected for an early efficacy assessment.

Absolute VE will be evaluated as the relative risk of CoVLP formulation vaccinated versus unvaccinated subjects to develop laboratory-confirmed symptomatic SARS-CoV-2 infection ≥ 14 days after the last vaccination, as per the following formula:

$$\text{Absolute VE}=(1-RR)=(1-ARV/ARU)*100\ \%$$

Where:

RR= relative risk

ARV = attack rate in subjects vaccinated with the CoVLP formulation; and

ARU = attack rate in unvaccinated subjects.

The absolute VE success criterion for the primary efficacy endpoint, in the Phase 3 portion, is defined as a ≥ 50 % point estimate and a > 30 % lower limit of the appropriately alpha-adjusted confidence interval that maintains overall Type I error at 5%. Statistical significance of the primary efficacy endpoint can be achieved at the interim analysis or at the primary analysis.

The 95% CI around RR will be calculated based on score confidence limits [Miettinen 1985, Farrington 1990].

12.7.2 Analysis of Secondary Endpoints

The secondary efficacy endpoints are defined in Section 10.4.2.2.

For the Phase 2 portion of the study, the absolute VE will be evaluated for the secondary efficacy endpoints.

Absolute VE will be evaluated as the relative risk of CoVLP formulation vaccinated versus unvaccinated subjects to develop laboratory-confirmed symptomatic SARS-CoV-2 infection ≥ 14 days after the last vaccination, as per the formula used for the analysis of the primary efficacy endpoint. In addition, the same formula will be used to evaluate the relative risk of CoVLP formulation vaccinated versus unvaccinated to develop severe COVID-19 disease ≥ 14 days after the last vaccination.

For the Phase 3 portion of the study, the absolute VE will also be evaluated for the secondary efficacy endpoints. Absolute VE will be evaluated as the relative risk of CoVLP formulation vaccinated versus unvaccinated subjects to develop severe COVID-19 disease ≥ 14 days after the last vaccination

The absolute VE success criterion for the secondary efficacy endpoints, in the Phase 3 portion, is defined as > 0 % lower limit of the appropriately alpha-adjusted confidence interval that maintains overall Type I error at 5%.

The 95% CI around RR will be calculated based on score confidence limits.

For the Phase 3 portion, the occurrences of COVID-19-related symptoms and their severity will be summarized by treatment using descriptive statistics. The occurrence of unusual manifestations of COVID-19 as well as the severity of COVID-19 symptoms will also be used to assess imbalance between the CoVLP and placebo groups as a possible indication of enhanced disease (full details provided in the Medical Monitoring Plan and IDMC Charter).

12.7.3 Analysis of Exploratory Endpoint

The exploratory efficacy endpoint is defined in Section 10.4.2.3.

For the Phase 2 portion, the occurrences of COVID-19-related symptoms and their severity will be summarized by treatment using descriptive statistics. The occurrence of unusual manifestations of COVID-19 as well as the severity of COVID-19 symptoms will also be used to assess imbalance between the CoVLP and placebo groups as a possible indication of enhanced disease (full details provided in the Medical Monitoring Plan and IDMC Charter).

12.8 Baseline and Subject Disposition

Demographic data will be presented in listings and summarized by treatment. Continuous variables including age, weight, height, and BMI will be summarized with mean, median, standard deviation, minimum, and maximum. Count and percentage of subjects will be presented for categorical variables such as sex at birth, race, ethnicity, and baseline SARS-CoV-2 testing results.

The number of subjects in different study disposition statuses will be summarized by number and, when applicable, percentage (based on vaccinated subjects). Subjects' completion/discontinuation status will be listed (including subject identifier, date of completion/early discontinuation and, for those who discontinued early, the specific reason(s) for discontinuation).

13 ADVERSE EVENT REPORTING

Timely, accurate, and complete reporting and analysis of safety information from clinical studies are crucial for the protection of subjects, Investigators, and the Sponsor, and are mandated by regulatory agencies worldwide.

13.1 Definitions

13.1.1 Serious Adverse Events

An SAE is any untoward medical occurrence (whether or not considered to be related to the study vaccine) that, at any dose:

- Results in death;
- Is life-threatening (at the time of the event);

  Note: the term "life-threatening" in the definition of an SAE refers to an event that put the subject at risk of death at the time of the event; it does not refer to an event that hypothetically might have caused death if it were more severe;
- Requires inpatient hospitalization (≥ 24 hours) or prolongation of existing hospitalization (elective hospitalizations/procedures for pre-existing conditions that have not worsened are excluded);
- Results in persistent or significant disability/incapacity;
- Is a congenital abnormality/birth defect;
- Is another medically important event.

Medical and scientific judgment should be exercised in deciding whether expedited reporting is appropriate in other situations, such as important medical events that may not be immediately life-threatening or result in death or hospitalization but that may jeopardize the subject's health or may require an intervention to prevent one of the other outcomes listed in the definition above. These events should be considered serious. Examples of such events are intensive treatment in an emergency room for allergic bronchospasm, blood dyscrasias, or convulsions that do not result in hospitalization or the development of drug dependency or drug abuse. See Section 13.1.5 for initial SAE reporting by the Investigator.

13.1.2 Adverse Events

An AE or adverse experience is defined as any untoward medical occurrence in a subject or clinical investigation subject who was administered a pharmaceutical product, with or without a causal relationship with the treatment. An AE can be any unfavorable and unintended sign, symptom, or disease temporally associated with the use of a medicinal product, whether or not related to a medicinal product.

Information such as the date and time of onset and resolution (duration), intensity (defined below), seriousness, any required treatment or action taken, outcome, relationship to the investigational vaccine, and whether the AE caused withdrawal from the study will be collected.

The intensity of all AEs will be graded as mild (1), moderate (2) severe (3), or potentially life threatening (4), according to the FDA Guidance for Industry: Toxicity Grading Scale for Healthy Adult and Adolescent Volunteers Enrolled in Preventive Vaccine Clinical Trials [FDA 2007] for the events covered in the guidance and the following definitions for all other events:

Mild (Grade 1):	The AE is easily tolerated and does not interfere with usual activity;
Moderate (Grade 2):	The AE interferes with daily activity, but the subject is still able to function;
Severe (Grade 3):	The AE is incapacitating and the subject is unable to work or complete usual activity;
Potentially life-threatening (Grade 4):	The AE is likely to be life-threatening if not treated in a timely manner.

The Investigator will be instructed to closely monitor each subject who experiences an AE (whether ascribed to the investigational product or not) until the outcome of the AE has been determined.

If any of the solicited local or systemic AEs persist beyond Day 7 after each vaccination (when applicable), these will also be recorded as unsolicited AEs. In this case, the AE start will be set as eight days post-vaccination. The subject will be requested to note when the AE resolves and to report this information to the Investigator or clinic staff at the next visit at the clinical site or contact.

The clinical importance of AEs will be determined based upon the Investigator's judgment. The Investigator must ensure that any sample obtained to follow-up on an AE is properly labeled and

stored. The Investigator and others responsible for care of the subjects should institute any supplementary investigations of significant AEs based on the clinical assessment of the likely causative factor. This may include seeking the opinion of a specialist in the field of the AE.

All unsolicited AEs occurring within 21 days after each vaccination must be reported in the "Adverse Event" screen in the subject's eCRF, irrespective of intensity or whether or not they are considered to be vaccination-related. Thereafter, from Day 43 through to Day 386, SAEs, AEs leading to withdrawal, and AESIs will be monitored and reported in the eCRF. Additional details on eCRF entries can be found in the eCRF completion guidelines.

13.1.2.1 Medically Attended Adverse Events

Medically attended adverse events are defined as symptoms or illnesses requiring hospitalization, or emergency room visit, or visit to/by a health care provider.

13.1.3 Adverse Events of Special Interest

13.1.3.1 Adverse Events of Special Interest for the Coronavirus-Like Particle COVID-19 Vaccine

13.1.3.1.1 Vaccine Enhanced Disease

Safety signal of VED after exposure to the CoVLP formulation will be closely monitored and assessed by retrieving data for this AESI as follows: AEs within the system organ class (SOC): immune system disorders and high level group term (HLGT): lower respiratory tract disorders (excluding obstruction and infection), cardiac disorders, signs and symptoms not elsewhere classified (NEC), vascular disorders, heart failures NEC, arteriosclerosis, stenosis, vascular insufficiency and necrosis, cardiac arrhythmias, myocardial disorders, and vascular hemorrhagic disorders. High level term (HLT): renal failure and impairment and preferred term (PT): pericarditis, coagulopathy, deep vein thrombosis, pulmonary embolism, cerebrovascular accidents, peripheral ischemia, liver injury, Guillain-Barre syndrome, anosmia, ageusia, encephalitis, chilblains, vasculitis, erythema multiforme (based on standardized MedDRA® classification) [Law 2020, Law 2020a] that require inpatient hospitalization (≥ 24 hours) and have laboratory confirmed SARS-Cov-2 infection will be monitored for assessment of any potential case of VED. As this list can be updated during the study, any further changes to the AESI terms will be updated in the Signal Management Plan (SMP).

The occurrence of unusual manifestations of COVID-19 as well as the severity of COVID-19 symptoms will be continuously reviewed by an independent and unblinded programmer and senior medical monitor for possible imbalance between the CoVLP and placebo groups as a possible indication of enhanced disease (full details provided in the Medical Monitoring Plan and IDMC Charter).

A sub-optimal immune response after vaccination may increase the risk of VED in subjects who receive a vaccine formulation retrospectively considered to be "sub-optimal" (i.e. subjects who received a lower dose level/regimen and/or non-adjuvanted formulation compared to the final

formulation determined to be safe and effective). These subjects (and/or their parent/LAR) will be informed that they/their child may be eligible to participate in a potential extension study of the CoVLP formulation and in this study, they/their child will be administered the final vaccine formulation.

13.1.3.1.2 Hypersensitivity Reactions

All reported events will also be monitored for hypersensitivity reactions after exposure to the CoVLP formulation. In eight clinical studies conducted to date with the Quadrivalent VLP Influenza Vaccine (QVLP) produced using similar plant-based technology, all reported events were monitored for a possible hypersensitivity component (events were searched using both narrow and broad standardized MedDRA® queries). Based on these data, there was a single case of possible early anaphylactic reaction associated with use of QVLP in humans. A small number of subjects had potential hypersensitivity reactions judged to be related to vaccine administration (no more than 0.3 % of subjects in any given QVLP treatment group experienced one of these events) and the events were distributed fairly evenly among treatment groups, including the placebo and the active comparator groups. However, since severe reactions are considered to be an important potential risk (based on the theoretical risk that using plants for the production of biotherapeutics may induce hypersensitivity), Medicago will require that appropriate medical treatment and supervision are available to manage any possible anaphylactic reactions in this study. To collect data on these events, Medicago will closely monitor and assess allergic or allergic-like reactions as AESIs.

13.1.3.2 Adverse Events of Special Interest for the Adjuvants

13.1.3.2.1 Potential Immune-Mediated Diseases and Other AESI as Listed in Section 19.4

Potential immune-mediated diseases (pIMDs) are a subset of AEs that include autoimmune diseases and other inflammatory and/or neurologic disorders of interest which may or may not have an autoimmune aetiology. Adverse events that need to be recorded and reported as pIMDs include those listed in Section 19.4.

However, the Investigator will exercise their medical and scientific judgement in deciding whether other diseases have an autoimmune origin (that is pathophysiology involving systemic or organ-specific pathogenic autoantibodies) and should also be recorded as a pIMD.

When there is enough evidence to make any of the diagnoses mentioned in Section 19.4, the AE must be reported as a pIMD. Symptoms, signs or conditions which might (or might not) represent the above diagnoses, should be recorded and reported as AEs but not as pIMDs until the final or definitive diagnosis has been determined, and alternative diagnoses have been eliminated or shown to be less likely.

In order to facilitate the documentation of pIMDs in the eCRF, a pIMD standard questionnaire and a list of preferred terms (PTs) and PT codes corresponding to the above diagnoses will be available to Investigators at study start.

Once a pIMD is diagnosed (serious or non-serious) in a study subject, the Investigator (or designate) must complete, date and sign an electronic Expedited Adverse Events Report.

13.1.4 Expectedness of an Adverse Drug Reaction

An "unexpected" adverse reaction is one in which the nature or severity of the event is not consistent with information in the current version of the IB. Expedited reporting to the regulatory authorities is required if an SAE occurs that is both unexpected and considered possibly, probably, or definitely related to the study vaccine.

13.1.5 Initial SAE, AESI, and Pregnancy Reports Reporting by the Investigator

Details on safety reporting are defined in study-specific documentation, including SAE processing, country-specific reporting requirements, regulatory unblinding, roles and responsibilities of different stake holders, and contact details of personnel responsible for safety reporting. A brief summary of salient information is presented below.

All post-vaccination SAEs, AESIs, and pregnancy reports will be reported from the time of receiving the study vaccine on Day 0 through to the end of the study (final scheduled visit). The Investigator (or designee) must report, by e-mail, all SAEs, AESIs, and pregnancy reports whether considered related to the study vaccine or not to Medicago within 24 hours of the Investigator learning of the event. The Investigator must also complete, sign, and date the paper SAE report form, and send, via e-mail, a copy to the Medicago safety e-mail address and the appropriate regional e-mail address within 24 hours of awareness of event:

Medicago Safety e-mail address:	Medicago-ClinicalSafety@medicago.com
Sponsor Safety Contact:	[Redacted]

Serious AEs will be reported to the local (or contract) IRB by the Investigator according to the IRB's policy and procedures.

13.1.6 Follow-up Reporting by the Investigator

All SAEs, regardless of causality, will be followed as described in Section 13.1.2. When appropriate, documentation of any medical tests or examinations performed will be provided to document resolution/stabilization of the event.

13.1.7 Reporting of SAEs Occurring after Study Termination

All SAEs occurring during the safety follow-up period will be followed until resolution or for a period of 30 days from the final subject's visit (which ever occurs first), regardless of conclusion of the study. However, all related SAEs occurring during the safety follow-up period will be followed until resolution or stabilization.

A post-study AE/SAE is defined as any event that occurs outside of the AE/SAE detection period (post Day 386). Active follow-up for AEs or SAEs will continue until Day 386 for all subjects. However, after Day 386, if the Investigator learns of any SAE, including death, at any time after a subject has been discharged from the study, and he/she considers the event to be reasonably related to the investigational product, the Investigator will promptly notify the Sponsor Safety Contacts for Reporting SAEs. These related SAEs will be followed until resolution or stabilization.

13.1.8 Causal Relationship

The Investigator must make the determination of relationship to the study vaccine for each AE. The Investigator should decide whether, in his/her medical judgment, there is a reasonable possibility that the event may have been caused by the investigational vaccine. If there is any valid reason, even if undetermined or untested, for suspecting a possible cause-and-effect relationship between the investigational vaccine and the occurrence of the AE, then the AE should be considered “definitely related”, “probably related”, or “possibly related”. Otherwise, if no valid reason exists for suggesting a possible relationship, then the AE should be classified as “probably not related” or “definitely not related”. The following guidance should be followed:

Definitely Not Related:	The AE is clearly not related to the administration of the study vaccine. Another cause of the event is most plausible; and/or a clinically plausible temporal sequence is inconsistent with the onset of the event and the study vaccine administration; and/or a causal relationship is considered biologically implausible.
Probably Not Related:	There is no medical evidence to suggest that the AE is related to the study vaccine. The event can be readily explained by the subject’s underlying medical condition or concomitant therapy or lacks a plausible temporal relationship to the study vaccine.
Possibly Related:	A direct cause and effect relationship between the study vaccine and the AE has not been demonstrated but there is a reasonable possibility that the event was caused by the study vaccine.
Probably Related:	There probably is a direct cause and effect relationship between the AE and the study vaccine. A plausible biologic mechanism and temporal relationship exist and there is no more likely explanation.
Definitely Related:	There is a direct cause and effect relationship between the AE and the study vaccine.

AE outcomes will be classified as recovered/resolved, not recovered/not resolved, recovered/resolved with sequelae, recovering/resolving, or death.

13.1.9 Reporting of SAEs to Health Authorities and IRB

Medicago or its designee will be responsible for reporting SAEs that are deemed both possibly related to the study vaccine and considered to be unexpected (‘unexpected’ refers to events that do not appear in the package labeling or in the study vaccine IB) to the regulatory authorities in an expedited manner.

The Investigator will be responsible for reporting the SAEs that meet IRB reporting requirements directly to the relevant ethical review board as soon as possible, and will also provide the ethical review board with any safety reports prepared by Medicago or its designee.

All SAEs that are suspected, unexpected serious adverse reactions (SUSARs) should be reported to regulatory authorities by phone or by facsimile transmission as soon as possible but, in no event, later than seven calendar days for deaths and life-threatening events, and 15 calendar days for other SAEs after Medicago's (or its designee's) initial receipt of the information. These events should be followed to resolution, stabilization, or return to baseline, regardless of conclusion of the study.

13.1.10 Independent Data Monitoring Committee

The IDMC will consist of a multidisciplinary group of three clinicians and/or physicians and one biostatistician who will, collectively, have extensive experience in the use of vaccines for patient treatment and in the conduct and safety monitoring of randomized clinical trials.

The IDMC will monitor the study conduct and review blinded and unblinded safety data at pre-defined times during the study for the purpose of providing recommendations to the Sponsor about whether:

- subject vaccination in different Study Populations will occur;
- subject vaccination in each Study Population will continue;
- the study portion will continue in the event of a pre-defined safety signal.

The IDMC review will ensure the ongoing safety of the subjects in the study and the scientific integrity of the study. Additional information about the planned and ad hoc IDMC review meetings is available in the IDMC Charter.

13.1.11 Safety Review and Stopping Rules

The IDMC will review safety data for the four Study Populations in the Phase 2 portion of the study. The unblinded safety data for the first 10 sentinel subjects after each dose in all Study Populations, with the exception of Study Population #1, will be reviewed by the IDMC before permitting enrollment of the remaining subjects, according to the details outlined in the IDMC Charter.

These IDMC reviews will detect any early negative trends in the safety data from a subset of subjects vaccinated within each Study Population and may necessitate a decision to not administer the study vaccine to the remaining study subjects.

The IDMC Charter outlines the actions to be considered if any of the IDMC reviews suggest a concern about the safety of the vaccine (see Stopping Rules below) in a Study Population according to the safety review parameters.

During the Phase 2 portion, the IDMC will review the safety data collected up to Day 28 (last subject) from all the subjects in each Study Population to confirm that each Study Population can be included in the Phase 3 portion of the study.

Stopping Rules

Safety monitoring of safety signals will be performed for each of the Study Populations throughout the Phase 2 and Phase 3 portions of the study. Stopping rules or conditions for stopping each portion of the study would occur if there was clear evidence of harm or harmful effects such as SAEs related to the treatment (study vaccine). An SAE which is assessed as unrelated to the study vaccine would not warrant stopping the trial.

For sentinel subjects in Study Populations #2, #3, and #4 during the Phase 2 portion of the study:

If any of the following events occur to the sentinel subjects, the study enrollment and vaccinations may be paused based on IDMC recommendations:

- Any SAE in a subject for which causality cannot be attributed to another cause;
- Two (2) or more subjects who receive the CoVLP formulation experience a similar AE (i.e. the same Preferred Terms based on MedDRA] coding):
  - Experience the same Grade 3 or higher solicited local AE or systemic AE within 7 days after administration of the vaccine;
  - Experience the same Grade 3 AE or higher unsolicited AE, vital sign, and/or clinical laboratory abnormality, in the same Preferred terms based on the MedDRA coding, and is considered related to the vaccine.

After vaccination of all sentinel subjects in Phase 2, the stopping rules for the remainder of the Phase 2 and the Phase 3 portions of the study:

The study may be paused based on IDMC recommendations if any of the following events occur:

- Any subject experiences an SAE after administration of the vaccine that is considered related to vaccine;
- If 5 % or more subjects who receive the CoVLP formulation experience a similar AE (i.e. the same Preferred Terms based on MedDRA coding):
  - Experience the same Grade 3 or higher solicited local AE or systemic AE, within 7 days after administration of the vaccine;
  - Experience the same Grade 3 AE or higher unsolicited AE, vital sign, and/or clinical laboratory abnormality, in the same Preferred terms based on the MedDRA coding, after administration of the vaccine and is considered related to the vaccine;
- If an important imbalance in unusual manifestations of COVID-19 or severity of COVID-19 symptoms is observed between the CoVLP and placebo groups (full details provided in the Medical Monitoring Plan and IDMC Charter).

If any of the stopping rules are met,, the Sponsor will to seek the guidance of the IDMC according to the process outlined in the IDMC Charter.

14 INVESTIGATIONAL PRODUCT INFORMATION

14.1 Identity of Investigational Product

The CoVLP formulation is the CoVLP vaccine composed of recombinant spike (S) glycoproteins expressed as VLPs and administered with the adjuvant AS03.

For more information regarding the CoVLP formulation, refer to the current IB.

14.1.1 Study Vaccine Composition

The CoVLP vaccine is a sterile transparent to opalescent, colorless to yellowish or greenish liquid suspension. The CoVLP vaccine will be supplied in 0.35 mL single dose vials. The S glycoprotein concentration in the vaccine will be 15 µg/mL for a final dosage of 3.75 µg.

AS03 is an established effective adjuvant licensed for Arepanrix™ H1N1 and Arepanrix™ H5N1 (in Canada). The adjuvant is a whitish to yellowish homogeneous milky liquid emulsion and it will be provided in the original sterile vial container received from the manufacturer (GlaxoSmithKline). A 0.25 mL dose of AS03 represents one human dose.

Further specific information relating to investigational product storage, shipment and treatment preparation is provided in the Investigational Product Management Manual (IPMM).

14.1.2 Placebo

The control product (placebo) will be composed of a phosphate-buffered saline (PBS) solution (100 mM $NaKPO_4$, 150 mM NaCl) and 0.01 % polysorbate 80 at pH 7.4. The control product will be provided in vials.

Further specific information relating to the investigational product storage and shipment is provided in the IPMM.

14.1.3 Preparation and Administration of Study Vaccine

The study treatments will be prepared by unblinded staff members at the clinical site as described in the Investigational Product Management Manual. The prepared study treatment will subsequently be administered to subjects by an unblinded staff member. The unblinded staff members must not be involved in the evaluation of any AEs or reactogenicity evaluations following vaccination; a blinded staff member must be present for post-vaccination observation.

The products to be used for study treatment administration will be handled in an aseptic manner.

The CoVLP formulation will be administered on study Day 0 and Day 21 as an IM injection of 0.5 mL, into the deltoid muscle. For subjects < 18 years of age and adult subjects with BMI < 30 kg/m^2, a 23 gauge needle of at least one inch or 2.5 cm in length can be used for vaccination. For subjects with BMI ≥ 30 kg/m^2, a needle of at least 1.5 inches or 3.5 cm in length must be used to ensure intramuscular delivery.

The product administered will be recorded in study-specific documentation (refer to IPMM) by the clinical site, which will be separate from the study medication record for drug preparation.

After drug accountability monitoring and reconciliation has been completed by the site and unblinded monitor, all study treatments (used and unused vials) will be destroyed locally upon Medicago's approval or returned to Medicago (or its designee) in accordance with instructions provided in the IPMM.

Further specific information relating to investigational product storage, shipment and treatment preparation is provided in the IPMM.

14.1.4 Preparation, Handling, Storage, and Precautions for Use

The investigational products should be stored in an access-restricted area between 2 ºC and 8 ºC and protected from light; the vaccine should, however, be at room temperature before administration (i.e. the vaccine should not be administered cold and should be taken out of the refrigerator at least five minutes prior to administration).

The IPMM provides additional details on treatment preparation, handling and storage of the investigational product.

14.2 Packaging

The CoVLP vaccine, AS03, and placebo will be packaged in separate boxes containing multiple vials of each investigational product.

14.3 Labeling

The investigational product vials will have a product and study-specific label containing information that meets the applicable regulatory requirements. It is not possible to have a matching placebo in this study, therefore the vial labels will not be blinded. The study treatment will be prepared and administered by trained unblinded staff. Blinding measures will be applied to maintain the observer-blindness of the blinded staff.

14.4 Drug Accountability

The Investigator is responsible for ensuring that all study treatments received at the site are inventoried and accounted for throughout the study. The dispensing of study treatment to each subject must be documented on study-specific documentation.

The investigational products must be handled in strict accordance with the IPMM and the vial label. The investigational products will be stored in a limited access area or in a locked cabinet under appropriate environmental conditions. The unblinded site staff will complete accountability for all investigational products (CoVLP, AS03, and placebo). Refer to the IPMM for details on the disposition of the used and unused vials.

The study drug should be dispensed under the supervision of the Investigator (or a qualified member of the investigational staff), who remains blinded as to which treatment is administered. The study drug will be supplied only to subjects participating in the study. Returned study drugs may not be relabeled or reassigned for use for other subjects. The Investigator agrees neither to

dispense the study drug from, nor store it at, any site other than the study centers agreed upon with Medicago (or its designee).

15 STUDY-SPECIFIC MATERIALS

The Investigator will be provided with the following:

- eCRF;
- Forms and questionnaires for special assessments;
- Thermometers and measurements template;
- Study manuals.

16 ETHICAL ASPECTS

16.1 Study-Specific Design Considerations

On-going blinded medical review will be performed by Medicago (or its designee) throughout the duration of the Phase 2 and Phase 3 portions of the study; subjects (and/or their parent/LAR) will be given any new information that may affect their decision to continue participation in the study.

Blood samples will be collected from subjects for immunogenicity analysis, although not all will be analysed immediately. Subject (and/or their parent/LAR) consent will be obtained for the use of any leftover samples. Since blood sample collection is a minimally invasive procedure and the amount of blood collected will be a relatively small amount (Table 6 and Table 7), the benefits of obtaining this data outweigh the small level of risk associated with the collection of samples that may not require analysis.

In the event a safe and effective treatment or vaccine becomes available for COVID-19 during the Phase 3 portion of the study, consideration will be given to offering the treatment/vaccine to the subjects who were administered placebo after all the COVID-19 cases required for the study have been collected. The consideration will take into account the local regulatory stipulations for the availability of such treatment/vaccine to the local population.

16.2 Regulatory Ethics Compliance

16.2.1 Investigator Responsibilities

The Investigator is responsible for ensuring that the study is performed in accordance with the protocol, current International Conference on Harmonisation (ICH) guidelines on Good Clinical Practice (GCP), and applicable regulatory and country-specific requirements.

GCP is an international ethical and scientific quality standard for designing, conducting, recording, and reporting studies that involve the participation of human subjects. Compliance with this standard provides public assurance that the rights, safety, and well-being of study subjects are protected, consistent with the principles that originated in the Declaration of Helsinki, and that the study data are reliable.

16.2.2 Independent Ethics Committee or Institutional Review Board

Before the start of the study, the Investigator (or Medicago, where required) will provide the Independent Ethics Committee (IEC)/IRB with current and complete copies of the following documents (as required by local regulations):

- Final protocol and, if applicable, amendments to the protocol;
- Sponsor-approved ICF (and any other written materials to be provided to the subjects);
- IB (or equivalent information) and amendments/addenda;
- Information on compensation for study-related injuries or payments to subjects for participation in the study, if applicable;
- Investigator's *curriculum vitae* or equivalent information (unless not required, as documented by the IEC/IRB);
- If applicable, any Sponsor-approved subject recruiting materials;
- Information regarding funding, name of the sponsor, institutional affiliations, other potential conflicts of interest, and incentives for subjects;
- Any other documents that the IEC/IRB requests to fulfill its obligation.

This study will be undertaken only after the IEC/IRB has given full approval of the final protocol, amendments (if any, excluding the ones that are purely administrative in nature, with no consequences for subjects, data, or study conduct), the ICF, applicable recruiting materials, and subject compensation programs, and Medicago (or its designee) has received a copy of this approval. This approval letter must be dated and must clearly identify the IEC/IRB and the documents being approved. In addition, the Investigator must wait for written confirmation from Medicago (or its designee) that the study can be started.

During the study, the Investigator (or Medicago [or its designee], where required) will send the following documents and updates to the IEC/IRB for their review and approval, where appropriate:

- Protocol amendments;
- Revision(s) to ICF and any other written materials to be provided to subjects;
- If applicable, new or revised subject recruiting materials approved by Medicago;
- Revisions to compensation for study-related injuries or payment to subjects for participation in the study, if applicable;
- New edition(s) of the IB and amendments/addenda;
- Summaries of the study at intervals stipulated in guidelines of the IEC/IRB (at a minimum, annually);
- Reports of AEs that are serious, unlisted/unexpected, and associated with the study drug (when applicable);
- New information that may adversely affect the safety of the subjects or the conduct of the study;

- Deviations from or changes to the protocol to eliminate immediate hazards to subjects;
- Reports of deaths of subjects under the Investigator's care;
- Notification if a new Investigator is responsible for the study at any of the sites;
- Development Safety Update Report and Line Listings, where applicable;
- Any other requirements of the IEC/IRB.

At least once a year, the IEC/IRB will be asked to review and reapprove this study. The re-approval should be documented in writing. At the end of the study, the Investigator (or Medicago [or its designee], where required) will notify the IEC/IRB of study completion.

16.2.3 Informed Consent

Each subject (and/or their parent/LAR) who participates in the study must first give written consent according to local requirements after the nature of the study has been fully explained, including the risks and requirements of the study. The consent form must be signed before performing any study-related activity. The consent form that is used must have been approved by regulatory authorities, Medicago, and the reviewing IEC/IRB. The informed consent should be in accordance with principles that originated in the Declaration of Helsinki, current ICH and GCP guidelines, applicable regulatory requirements, and Medicago policy. During the study, subjects (and/or their parent/LAR) will be given any new information that may affect their decision to continue their participation in the study. They will be told that their consent to participate is voluntary and that it may be withdrawn at any time, with no reason given, and without penalty or loss of benefits to which they would otherwise be entitled. Only subjects (and/or their parent/LAR) who are fully able to understand the risks, benefits, and potential AEs of the study and who provide their consent voluntarily will be enrolled.

16.2.4 Privacy of Personal Data

The collection and processing of personal data from subjects enrolled in this study will be limited to those data that are necessary to support the development, registration, and future marketing of the investigational product. The data must be collected and processed with adequate precautions to ensure confidentiality and compliance with applicable data privacy protection laws and regulations. Appropriate technical and organizational measures to protect the personal data against unauthorized disclosures or access, accidental or unlawful destruction, or accidental loss or alteration must be in place. Medicago (or its designee) personnel whose responsibilities require access to personal data agree to keep the identity of study subjects confidential.

17 ADMINISTRATIVE REQUIREMENTS

17.1 Protocol Amendments

Neither the Investigator nor Medicago will modify this protocol without a formal amendment. All protocol amendments must be issued and approved by Medicago, and signed and dated by the Investigator. Protocol amendments must not be implemented without prior IEC/IRB approval

or when the relevant competent authority has raised any grounds for non-acceptance, except when necessary in order to eliminate an immediate hazard to the subjects, in which case the amendment must be promptly submitted to the IEC/IRB and relevant competent authority. Documentation of amendment approval by the Investigator and IEC/IRB must be provided to Medicago or its designee.

> Note that administrative changes may be implemented without prior IRB approval; however, the Investigator or Medicago, as applicable, must notify the IRB of any administrative change and ensure that IRB acknowledges receipt of the administrative changes.

The Investigator is responsible for notifying the IRB of all protocol amendments and ensuring that the IRB has approved any amendments when local IRBs are used. When a central IRB is used, Medicago or its designee will inform the IRB on behalf of the sites.

During the course of the study, in situations where a departure from the protocol is unavoidable, the Investigator or other physician in attendance will contact the appropriate Medicago representative or its designee (see Contact Information pages provided separately). Except in emergency situations, this contact should be made before implementing any departure from the protocol. In all cases, contact with Medicago or its designee must be made as soon as possible to discuss the situation and to agree on an appropriate course of action. The data recorded in the eCRF and source documents will reflect any departure from the protocol, and the source documents will describe this departure and the circumstances that made a deviation necessary.

17.2 Regulatory Documentation

17.2.1 Regulatory Approval/Notification

This protocol and any amendment(s) must be submitted to the appropriate regulatory authorities in each respective country, as applicable. A study may not be initiated until all local regulatory requirements are met.

17.3 Source Documentation

At a minimum, source documentation must be available for the following information:

- Subject identification, eligibility, and study identification;
- ICF discussion and date of informed consent;
- Dates of visits;
- Results of safety and study procedures as required by the protocol;
- Record of all reactions, AEs, and associated follow-ups;
- Concomitant medication;
- Drug receipt, dispensing, and return records;
- Study drug preparation and administration information;

- Date of study completion and reason for early discontinuation of study drug or withdrawal from the study, if applicable.

It is recommended that the author of an entry in the source documents be identifiable.

At a minimum, the type and level of detail of source data available for a study subject should be consistent with that commonly recorded at the site as a basis for standard medical care. If Investigator judgement was used in the determination of eligibility, an explanation for inclusion of the subject in the study must be provided in the source documents. Specific details required as source data for the study will be reviewed with the Investigator before the study and will be described in the monitoring guidelines (or other equivalent document).

17.3.1 Diary and Memory Aid

Subjects (and/or their parent/LAR) will be provided with a paper or electronic diary or application in which to record solicited AEs, COVID-19 symptoms, and other safety information. For example, the electronic diary may be configured to transmit images of the memory aid to sites. The electronic diary may also be used for the active weekly surveillance for COVID-19 symptoms and daily symptoms for cases of COVID-19. Subject diaries are considered source documents.

Subjects (and/or their parent/LAR) will be provided with memory aids to record information on unsolicited AEs, SAEs, AEs leading to withdrawal, AESIs, and reportable concomitant medications from Day 0 to Day 42, Day 43 to Day 201, and Day 202 to the end of the study (Day 386). These memory aids will be collected by the site and are intended to be used by the subjects to help them in reporting this information to site staff during phone contacts and clinical site visits.

17.4 Case Report Form Completion

An eCRF will be provided for each subject who is randomized and receives a dose of study drug. Screening failures will also be entered in an eCRF; data entered should include date of birth and reason for screening failure.

Data must be entered into the eCRFs in English. The Investigator must verify that all data entries in the eCRFs are accurate and correct. All eCRF entries, corrections, and alterations must be made by the Investigator or other authorized study-site personnel.

Please refer to the eCRF completion guidelines for details of data entry requirements.

17.5 Quality Control (Study Monitoring)

In accordance with applicable regulations, GCP, and Medicago (or its designee) procedures, the site will be contacted prior to the study start to review the protocol and study requirements with the clinical study staff. Medicago (or its designee) will review their responsibilities to satisfy regulatory, ethical, and company (Medicago) requirements.

The Investigator or institution, if applicable, will authorize and provide direct access to all relevant documents to allow review of the appropriate conduct of the study by Medicago's (or its designee's) monitor. The Investigator(s) must allocate their time and the time of their staff to the monitors, discuss any findings or issues, and take appropriate actions to maintain the quality and integrity of the study data and conduct.

Protocol deviations will be reviewed by Medicago (or its designee) to identify any non-compliances likely to have a significant effect on the safety and rights of a subject or the reliability and robustness of the data generated. These deviations will be included in the clinical study report.

17.6 Quality Assurance

To ensure compliance with GCP and all other applicable regulatory requirements, Medicago (or its designee) may conduct quality assurance assessments and/or an audit of the site. Regulatory agencies may conduct regulatory inspections at any time during or after completion of the study. If an audit or an inspection is conducted, the Investigator and/or institution (if applicable) must agree to grant auditors or inspectors direct access to all relevant documents and to allocate their time and the time of their staff to discuss the conduct of the study and any findings or issues, and to implement corrective and/or preventative actions to address any findings or issues identified.

17.7 Record Retention

Following closure of the study, the Investigator must maintain all site study records in a safe and secure location. The records must be easily accessible when needed (e.g. for a Medicago [or its designee] audit or regulatory inspection) and must be available for review in conjunction with assessment of the facility, supporting systems, and relevant site staff.

When permitted by local laws and regulations or institutional policy, some or all of the records may be maintained in a format other than a hard copy (e.g. scanned electronic). The Investigator must ensure that all reproductions are legible and are a true and accurate copy of the original.

The Investigator must follow the time period for retaining the site records in order to comply with all applicable regulatory requirements. The minimum retention time will meet the strictest standard applicable to a particular site, as dictated by local laws and regulations, Medicago (or its designee) standard operating procedures (SOPs), and/or institutional requirements. The Investigator must contact Medicago (or its designee) prior to the disposal of any study documents at the end of the retention period.

If the responsible Investigator retires, relocates, or, for any other reason, withdraws from the responsibility of keeping the study records, custody must be transferred to another qualified person who will accept the responsibility and is approved by Medicago. Medicago (or its designee) must be notified in writing of the name and address of the new custodian. Under no circumstance shall the Investigator relocate or dispose of any study documents without having obtained prior written approval from Medicago (or its designee).

If it becomes necessary for Medicago or the appropriate regulatory authority to review any documentation relating to this study, the Investigator (or new custodian) must permit access to such reports.

17.8 Study Completion/Termination

17.8.1 Study Completion

Each portion of the study will be considered to be completed with the last contact with the last subject participating in the particular portion of the study. The final data from the investigational site will be sent to Medicago (or designee) after completion of the final subject visit at that site. Investigational sites will be closed after the portion completion. An investigational site is considered closed when all required documents and study supplies have been collected, all data have been entered, monitored, locked, and signed and a site closure visit has been performed.

17.8.2 Study Termination

Medicago (or its designee) reserves the right to close the investigational site or terminate the study at any time for any reason at the sole discretion of Medicago. Investigational sites will be closed after study termination once Medicago (or its designee) has completed all study related tasks. Reasons for the early closure of an investigational site by Medicago (or its designee) or the Investigator may include, but are not limited to:

- Failure of the Investigator to comply with the protocol, Medicago's (or its designee's) procedures, or GCP guidelines;
- Inadequate recruitment of subjects by the Investigator;
- Discontinuation of further drug development.

The Investigator may initiate site closure at any time, provided there is reasonable cause and sufficient notice is given in advance of the intended closure.

Site closure following an Investigator request can only be performed when all active study subjects have completed the study period, Medicago (or its designee) has collected all required documents and study supplies, and all data have been entered, monitored and locked.

17.9 Registration of Clinical Studies and Disclosure of Results

Study information from this study will be posted on publicly available clinical trial registries in countries where applicable and will include information required by law. In addition, the results summary will be posted to the same clinical trial registries, to the extent specified by law, and will include information required by regulatory authorities.

17.10 Publication Policy

The data derived from this study are the property of Medicago and cannot be published without prior authorization from Medicago. Any publication activities (i.e. preparation and submission of abstracts and manuscripts) will be at the discretion of Medicago.

Any proposed publication regarding this study, not prepared by Medicago personnel, must be provided to Medicago for comments and review at least 45 days prior to its intended publication. The proposed publication shall not include any confidential information or protected information to preserve Intellectual Property rights; any such information must be removed from the proposed publication.

18 REFERENCES

Ahn DG, Shin HJ, Kim MH, Lee S, Kim HS, Myoung J, Kim BT, Kim SJ. Current status of epidemiology, diagnosis, therapeutics, and vaccines for novel coronavirus disease 2019 (COVID-19). J Microbiol Biotechnol. 2020 Mar 28;30(3):313-324. doi: 10.4014/jmb.2003.03011.

Baz M, Luke CJ, Cheng X, Jin H, Subbarao K. H5N1 vaccines in humans. Virus Res 2013;178:78-98. doi: 10.1016/j.virusres.2013.05.006.

Bektas A, Schurman SH, Sen R, Ferrucci L. Human T cell immunosenescence and inflammation in aging. J Leukoc Biol. 2017 Oct;102(4):977-988. doi: 10.1189/jlb.3RI0716-335R.

Bravo LC, Carlos JC, Gatchalian SR, Montellano MEB, Tabora CFCB, Thierry-Carstensen B, Tingskov PN, Sørensen C, Wachmann H, Bandyopadhyay AS, Nielsen PI, Kusk MV. Immunogenicity and safety of an adjuvanted inactivated polio vaccine, IPV-Al, compared to standard IPV: A phase 3 observer-blinded, randomised, controlled trial in infants vaccinated at 6, 10, 14 weeks and 9 months of age. Vaccine. 2020 Jan 16;38(3):530-538. doi: 10.1016/j.vaccine.2019.10.064.

Cai J, Xu J, Lin D, Yang Z, Xu L, Qu Z, Zhang Y, Zhang H, Jia R, Liu P, Wang X, Ge Y, Xia A, Tian H, Chang H, Wang C, Li J, Wang J, Zeng M. A Case Series of children with 2019 novel coronavirus infection: clinical and epidemiological features. Clin Infect Dis. 2020 Feb 28:ciaa198. doi: 10.1093/cid/ciaa198.

CDC COVID-19 Response Team. Severe Outcomes Among Patients with Coronavirus Disease 2019 (COVID-19) - United States, February 12-March 16, 2020. MMWR Morb Mortal Wkly Rep. 2020 Mar 27;69(12):343-346. doi: 10.15585/mmwr.mm6912e2.

CDC COVID-19 Response Team. Coronavirus Disease 2019 in Children - United States, February 12-April 2, 2020. MMWR Morb Mortal Wkly Rep. 2020a Apr 10;69(14):422-426. doi: 10.15585/mmwr.mm6914e4.

Channappanavar R, Zhao J, Perlman S. T cell-mediated immune response to respiratory coronaviruses. Immunol Res. 2014 Aug;59(1-3):118-28. doi: 10.1007/s12026-014-8534-z.

Chen N, Zhou M, Dong X, Qu J, Gong F, Han Y, Qiu Y, Wang J, Liu Y, Wei Y, Xia J, Yu T, Zhang X, Zhang L. Epidemiological and clinical characteristics of 99 cases of 2019 novel coronavirus pneumonia in Wuhan, China: a descriptive study. Lancet. 2020 Feb 15;395(10223):507-513. doi: 10.1016/S0140-6736(20)30211-7.

Chen Y, Liu Q, Guo D. Emerging coronaviruses: Genome structure, replication, and pathogenesis. J Med Virol. 2020a Apr;92(4):418-423. doi: 10.1002/jmv.25681.

Chen L, Yu J, He W, Chen L, Yuan G, Dong F, Chen W, Cao Y, Yang J, Cai L, Wu D, Ran Q, Li L, Liu Q, Ren W, Gao F, Wang H, Chen Z, Gale RP, Li Q, Hu Y. Risk factors for death in 1859 subjects with COVID-19. Leukemia. 2020b Jun 16:1-11. doi: 10.1038/s41375-020-0911-0.

Cheng VC, Lau SK, Woo PC, Yuen KY. Severe acute respiratory syndrome coronavirus as an agent of emerging and reemerging infection. Clin Microbiol Rev. 2007 Oct;20(4):660-94.

Clark HL, Banks R, Jones L, Hornick TR, Higgins PA, Burant CJ, Canaday DH. Characterization of MHC-II antigen presentation by B cells and monocytes from older individuals. Clin Immunol. 2012 Aug;144(2):172-7. doi: 10.1016/j.clim.2012.06.005.

Cohet C, Montplaisir J, Petit D, Quinn MJ, Ouakki M, Deceuninck G, et al. Applying the test-negative design to vaccine safety studies: risk of narcolepsy associated with A/H1N1 (2009) pandemic influenza vaccination in Quebec [abstract 713]. Pharmacoepidemiol Drug Saf 2015;24:406-407. doi: 10.1002/pds.

Cohet C, van der Most R, Bauchau V, Bekkat-Berkani R, Doherty TM, Schuind A, Tavares Da Silva F, Rappuoli R, Carcon N, Innis BL. Safety of AS03-adjuvanted influenza vaccines: A review of the evidence. Vaccine 2019;37(23):3006-3021. doi: 10.1016/j.vaccine.2019.04.048.

COVID-19: Medicago’s development programs. Available online: https://www.medicago.com/en/covid-19-programs (accessed on April 23, 2020).

Crooke SN, Ovsyannikova IG, Poland GA, Kennedy RB. Immunosenescence and human vaccine immune responses. Immun Ageing. 2019 Sep 13;16:25. doi: 10.1186/s12979-019-0164-9.

Cruz AT, Zeichner SL. COVID-19 in Children: Initial Characterization of the Pediatric Disease. Pediatrics. 2020 Jun;145(6):e20200834. doi: 10.1542/peds.2020-0834.

Djennad A, Ramsay ME, Pebody R, Fry NK, Sheppard C, Ladhani SN, Andrews NJ. Effectiveness of 23-Valent Polysaccharide Pneumococcal Vaccine and Changes in Invasive Pneumococcal Disease Incidence from 2000 to 2017 in Those Aged 65 and Over in England and Wales. EClinicalMedicine. 2019 Jan 2;6:42-50. doi: 10.1016/j.eclinm.2018.12.007.

Domnich A, Arata L, Amicizia D, Puig-Barberà J, Gasparini R, Panatto D. Effectiveness of MF59-adjuvanted seasonal influenza vaccine in the elderly: A systematic review and meta-analysis. Vaccine. 2017 Jan 23;35(4):513-520. doi: 10.1016/j.vaccine.2016.12.011.

Dong Y, Mo X, Hu Y, Qi X, Jiang F, Jiang Z, Tong S. Epidemiology of COVID-19 Among Children in China. Pediatrics. 2020 Jun;145(6):e20200702. doi: 10.1542/peds.2020-0702.

Dowling DJ, Levy O. Pediatric Vaccine Adjuvants: Components of the Modern Vaccinologist's Toolbox. Pediatr Infect Dis J. 2015 Dec;34(12):1395-8. doi: 10.1097/INF.0000000000000893.

Du L, He Y, Zhou Y, Liu S, Zheng BJ, Jiang S. The spike protein of SARS-CoV - a target for vaccine and therapeutic development. Nat Rev Microbiol. 2009 Mar;7(3):226-36. doi: 10.1038/nrmicro2090.

Du RH, Liang LR, Yang CQ, Wang W, Cao TZ, Li M, Guo GY, Du J, Zheng CL, Zhu Q, Hu M, Li XY, Peng P, Shi HZ. Predictors of mortality for patients with COVID-19 pneumonia caused by SARS-CoV-2: a prospective cohort study. Eur Respir J. 2020 May 7;55(5):2000524. doi: 10.1183/13993003.00524-2020.

Enjuanes L, Zuñiga S, Castaño-Rodriguez C, Gutierrez-Alvarez J, Canton J, Sola I. Molecular basis of coronavirus virulence and vaccine development. Adv Virus Res. 2016;96:245-286. doi: 10.1016/bs.aivir.2016.08.003.

Farrington CP, Manning G. “Test Statistics and Sample Size Formulae for Comparative Binomial Trials with Null Hypothesis of Non-zero Risk Difference or Non-unity Relative Risk.” Statistics in Medicine. 1990;9:1447–1454.

FDA. Guidance for Industry: Toxicity grading scale for healthy adult and adolescent volunteers enrolled in preventive vaccine clinical trials. 2007.

Feldstein LR, Rose EB, Horwitz SM, Collins JP, Newhams MM, Son MBF, Newburger JW, Kleinman LC, Heidemann SM, Martin AA, Singh AR, Li S, Tarquinio KM, Jaggi P, Oster ME, Zackai SP, Gillen J, Ratner AJ, Walsh RF, Fitzgerald JC, Keenaghan MA, Alharash H, Doymaz S, Clouser KN, Giuliano JS Jr, Gupta A, Parker RM, Maddux AB, Havalad V, Ramsingh S, Bukulmez H, Bradford TT, Smith LS, Tenforde MW, Carroll CL, Riggs BJ, Gertz SJ, Daube A, Lansell A, Coronado Munoz A, Hobbs CV, Marohn KL, Halasa NB, Patel MM, Randolph AG; Overcoming COVID-19 Investigators and the CDC COVID-19 Response Team. Multisystem Inflammatory Syndrome in U.S. Children and Adolescents. N Engl J Med. 2020 Jul 23;383(4):334-346. doi: 10.1056/NEJMoa2021680.

Frasca D, Diaz A, Romero M, Landin AM, Blomberg BB. Age effects on B cells and humoral immunity in humans. Ageing Res Rev. 2011 Jul;10(3):330-5. doi: 10.1016/j.arr.2010.08.004.

Furman D, Campisi J, Verdin E, Carrera-Bastos P, Targ S, Franceschi C, Ferrucci L, Gilroy DW, Fasano A, Miller GW, Miller AH, Mantovani A, Weyand CM, Barzilai N, Goronzy JJ, Rando TA, Effros RB, Lucia A, Kleinstreuer N, Slavich GM. Chronic inflammation in the etiology of disease across the life span. Nat Med. 2019 Dec;25(12):1822-1832. doi: 10.1038/s41591-019-0675-0.

Garcia-Sicilia J, Arístegui J, Omeñaca F, Carmona A, Tejedor JC, Merino JM, García-Corbeira P, Walravens K, Bambure V, Moris P, Caplanusi A, Gillard P, Dieussaert I. Safety and persistence of the humoral and cellular immune responses induced by 2 doses of an AS03-adjuvanted A(H1N1)pdm09 pandemic influenza vaccine administered to infants, children and adolescents: Two open, uncontrolled studies. Hum Vaccin Immunother. 2015;11(10):2359-69. doi: 10.1080/21645515.2015.1063754.

Garcon N, Vaughn DW, Didierlaurent AM. Development and evaluation of AS03, an Adjuvant System containing alpha-tocopherol and squalene in an oil-in-water emulsion. Expert Rev Vaccines 2012;11:349-366. doi: 10.1586/erv.11.192.

Garg S, Kim L, Whitaker M, O'Halloran A, Cummings C, Holstein R, Prill M, Chai SJ, Kirley PD, Alden NB, Kawasaki B, Yousey-Hindes K, Niccolai L, Anderson EJ, Openo KP, Weigel A, Monroe ML, Ryan P, Henderson J, Kim S, Como-Sabetti K, Lynfield R, Sosin D, Torres S, Muse A, Bennett NM, Billing L, Sutton M, West N, Schaffner W, Talbot HK, Aquino C, George A, Budd A, Brammer L, Langley G, Hall AJ, Fry A. Hospitalization Rates and Characteristics of Patients Hospitalized with Laboratory-Confirmed Coronavirus Disease 2019 - COVID-NET, 14 States, March 1-30, 2020. MMWR Morb Mortal Wkly Rep. 2020 Apr 17;69(15):458-464. doi: 10.15585/mmwr.mm6915e3.

Guan WJ, Ni ZY, Hu Y, Liang WH, Ou CQ, He JX, Liu L, Shan H, Lei CL, Hui DSC, Du B, Li LJ, Zeng G, Yuen KY, Chen RC, Tang CL, Wang T, Chen PY, Xiang J, Li SY, Wang JL, Liang ZJ, Peng YX, Wei L, Liu Y, Hu YH, Peng P, Wang JM, Liu JY, Chen Z, Li G, Zheng ZJ, Qiu SQ, Luo J, Ye CJ, Zhu SY, Zhong NS; China medical treatment expert group for COVID-19. Clinical characteristics of coronavirus disease 2019 in China. N Engl J Med. 2020 Feb 28. doi: 10.1056/NEJMoa2002032.

Guan WJ, Liang WH, Zhao Y, Liang HR, Chen ZS, Li YM, Liu XQ, Chen RC, Tang CL, Wang T, Ou CQ, Li L, Chen PY, Sang L, Wang W, Li JF, Li CC, Ou LM, Cheng B, Xiong S,

Ni ZY, Xiang J, Hu Y, Liu L, Shan H, Lei CL, Peng YX, Wei L, Liu Y, Hu YH, Peng P, Wang JM, Liu JY, Chen Z, Li G, Zheng ZJ, Qiu SQ, Luo J, Ye CJ, Zhu SY, Cheng LL, Ye F, Li SY, Zheng JP, Zhang NF, Zhong NS, He JX; China Medical Treatment Expert Group for COVID-19. Comorbidity and its impact on 1590 patients with COVID-19 in China: a nationwide analysis. Eur Respir J. 2020a May 14;55(5):2000547. doi: 10.1183/13993003.00547-2020.

Health Canada (HC). Coronavirus disease 2019 (COVID-19): Epidemiology update. Updated: July 23, 2020, 7 pm EDT. https://health-infobase.canada.ca/covid-19/epidemiological-summary-covid-19-cases.html#a7 (accessed July 24, 2020).

Heplisav-B® (Hepatitis B Vaccine [Recombinant], Adjuvanted) solution for intramuscular injection [package insert]. Emeryville (CA): Dynavax Technologies Corporation, 2020 [revised May 2020; cited 9 June 2020].

Honda-Okubo Y, Barnard D, Ong CH, Peng BH, Tseng CT, Petrovsky N. Severe acute respiratory syndrome-associated coronavirus vaccines formulated with delta inulin adjuvants provide enhanced protection while ameliorating lung eosinophilic immunopathology. J Virol. 2015 Mar;89(6):2995-3007. doi: 10.1128/JVI.02980-14.

Jackson LA, Campbell JD, Frey SE, Edwards KM, Keitel QA, Kotloff KL, Berry AA, Graham I, Atmar RL, Creech CB, Thomsen IP, Patel SM, Gutierrez AF, Anderson EL, Sahly HM, Hill H, Noah DL, Bellamy AR. Effect of varying doses of a monovalent H7N9 influenza vaccine with and without AS03 and MF59 adjuvants on immune response: a randomised clinical trial. JAMA 2015;314:237-46. doi: 10.1001/jama.2015.7916.

Khurana S, Coyle EM, Manischewitz J, King LR, Gao J, Germain RN, Schwartzberg PL, Tsang JS, Golding H, CHI Consortium. AS03-adjuvanted H5N1 vaccine promotes antibody diversity and affinity maturation, NAI titers, cross-clade H5N1 neutralization, but not H1N1 cross-subtype neutralization. NPJ Vaccines 2018;3:40. doi: 10.1038/s41541-018-0076-2.

Korakas E, Ikonomidis I, Kousathana F, Balampanis K, Kountouri A, Raptis A, Palaiodimou L, Kokkinos A, Lambadiari V. Obesity and COVID-19: immune and metabolic derangement as a possible link to adverse clinical outcomes. Am J Physiol Endocrinol Metab. 2020 Jul 1;319(1):E105-E109.

Kwetkat A, Heppner HJ. Comorbidities in the Elderly and Their Possible Influence on Vaccine Response. Interdiscip Top Gerontol Geriatr. 2020;43:73-85. doi: 10.1159/000504491.

Lake MA. What we know so far: COVID-19 current clinical knowledge and research. Clin Med (Lond). 2020 Mar;20(2):124-127. doi: 10.7861/clinmed.2019-coron.

Lambert P-H, Ambrosino DM, Andersen SR, Baric RS, Black SB, Chen RT, Dekker CL, Didierlaurent AM, Graham BS, Martin SD, Molrine DC, Perlman S, Picard-Fraser PA, Pollard AJ, Qin C, Subbarao K, Cramer JP. Consensus summary report for CEPI/BC March 12-13, 2020 meeting: assessment of risk of disease enhancement with COVID-19 vaccines. Vaccine, 2020 May 25. doi: https://doi.org/10.1016/j.vaccine.2020.05.064.

Lansbury L, Smith S, Beyer W, Karamehic E, Pasic-Juhas E, Sikira H, Mateus A, Oshitani H, Zhao H, Beck CR, Nguyen-Van-Tam JS. Effectiveness of 2009 pandemic influenza A(H1N1) vaccines: a systematic review and meta-analysis. Vaccine 2017;35:1996–2006. doi: 10.1016/j.vaccine.2017.02.059.

Launay O, Duval X, Fitoussi S, Jilg W, Kerdpanich A, Montellano M, Schwarz TF, Watanveerade V, Wenzel JJ, Zalcman G, Bambure V, Li P, Caplanusi A, Madan A, Gillard P, Vaughn DW. Extended antigen sparing potential of AS03-adjuvanted

pandemic H1N1 vaccines in children, and immunologic equivalence of two formulations of AS03-adjuvanted H1N1 vaccines: results from two randomised trials. BMC Infect Dis 2013;13:435. doi: 10.1186/1471-2334-13-435.

Law B, Sturkenboom M. Safety platform for emergency vaccines (SPEAC). D2.3 Priority list of adverse events of special interest: COVID-19. Report v1.1. 2020 Mar 5.

Law B, Sturkenboom M. Safety platform for emergency vaccines (SPEAC). D2.3 Priority list of adverse events of special interest: COVID-19. Report v2.0. 2020a May 25.

Leroux-Roels I, Borkowski A, Vanwolleghem T, Drame M, Clement F, Hons E, Devaster J-M, Leroux-Roels G. Antigen sparing and cross-reactive immunity with an adjuvanted rH5N1 prototype pandemic influenza vaccine: a randomised controlled trial. Lancet 2007;370:580–9. doi: 10.1016/s0140-6736(07)61297-5.

Li H, Liu Z, Ge J. Scientific research progress of COVID-19/SARS-CoV-2 in the first five months. J Cell Mol Med. 2020 Jun;24(12):6558-6570. doi: 10.1111/jcmm.15364.

Liu J, Zheng X, Tong Q, Li W, Wang B, Sutter K, Trilling M, Lu M, Dittmer U, Yang D. Overlapping and discrete aspects of the pathology and pathogenesis of the emerging human pathogenic coronaviruses SARS-CoV, MERS-CoV, and 2019-nCoV. J Med Virol. 2020 May;92(5):491-494. doi: 10.1002/jmv.25709.

Liu K, Chen Y, Lin R, Han K. Clinical features of COVID-19 in elderly patients: A comparison with young and middle-aged patients. J Infect. 2020a Mar 27. pii: S0163-4453(20)30116-X. doi: 10.1016/j.jinf.2020.03.005.

Liu W, Zhang Q, Chen J, Xiang R, Song H, Shu S, Chen L, Liang L, Zhou J, You L, Wu P, Zhang B, Lu Y, Xia L, Huang L, Yang Y, Liu F, Semple MG, Cowling BJ, Lan K, Sun Z, Yu H, Liu Y. Detection of Covid-19 in Children in Early January 2020 in Wuhan, China. N Engl J Med. 2020b Apr 2;382(14):1370-1371. doi: 10.1056/NEJMc2003717.

Lu S. Timely development of vaccines against SARS-CoV-2. Emerg Microbes Infect. 2020 Mar 8;9(1):542-544. doi: 10.1080/22221751.2020.1737580.

Ludvigsson JF. Systematic review of COVID-19 in children shows milder cases and a better prognosis than adults. Acta Paediatr. 2020 Jun;109(6):1088-1095. doi: 10.1111/apa.15270.

Madan A, Collins H, Sheldon E, Frenette L, Chu L, Friel D, Drame M, Vaughn DW, Innis BL, Schuind A. Evaluation of a primary course of H9N2 vaccine with or without AS03 adjuvant in adults: a phase I/II randomized trial. Vaccine 2017;35:4621–8. doi: 10.1016/j.vaccine.2017.07.013.

Madan A, Ferguson M, Rheault P, Seiden D, Toma A, Friel D, Soni J, Li P, Innis BL, Schuind A. Immunogenicity and safety of an AS03-adjuvanted H7N1 vaccine in adults 65 years of age and older: a phase II, observer-blind, randomized, controlled trial. Vaccine 2017a;35:1865–72. doi: 10.1016/j.vaccine.2017.02.057.

Madan A, Ferguson M, Sheldon E, Segall N, Chu L, Toma A, Rheault P, Friel D, Soni J, Li P, Innis BL, Schuind A. Immunogenicity and safety of an AS03-adjuvanted H7N1 vaccine in healthy adults: a phase I/II, observer-blind, randomized, controlled trial. Vaccine 2017b;35:1431–9. doi: 10.1016/j.vaccine.2017.01.054.

Manzoli L, Ioannidis JP, Flacco ME, De Vito C, Villari P. Effectiveness and harms of seasonal and pandemic influenza vaccines in children, adults and elderly: a critical review and re-analysis of 15 meta-analyses. Hum Vaccin Immunother. 2012 Jul;8(7):851-62. doi: 10.4161/hv.19917.

McElhaney JE, Beran J, Devaster JM, Esen M, Launay O, Leroux-Roels G, Ruiz-Palacios GM, van Essen GA, Caplanusi A, Claeys C, Durand C, Duval X, Idrissi ME, Falsey AR, Feldman G, Frey SE, Galtier F, Hwang S-J, Innis BL, Kovac M, Kremsner P, McNeil S, Nowakowski A, Richardus JH, Trofa A, Oostvogels L, Influence65 study group. AS03-adjuvanted versus non-adjuvanted inactivated trivalent influenza vaccine against seasonal influenza in elderly people: a phase 3 randomised trial. Lancet Infect Dis 2013;13:485-496. doi: 10.1016/S1473-3099(13)70046-X.

Mehra MR, Desai SS, Kuy S, Henry TD, Patel AN. Cardiovascular Disease, Drug Therapy, and Mortality in Covid-19. N Engl J Med. 2020 Jun 18;382(25):e102. doi: 10.1056/NEJMoa2007621.

Memish ZA, Perlman S, Van Kerkhove MD, Zumla A. Middle East respiratory syndrome. Lancet. 2020 Mar 28;395(10229):1063-1077. doi: 10.1016/S0140-6736(19)33221-0.

Miettinen OS, Nurminen MM. “Comparative Analysis of Two Rates.” Statistics in Medicine. 1985;4:213–226.

Montplaisir J, Petit D, Quinn MJ, Ouakki M, Deceuninck G, Desautels A, Mignot E, De Wals P. Risk of narcolepsy associated with inactivated adjuvanted AS03A/H1N1 (2009) pandemic influenza vaccine in Québec. PLoS One 2014;9(9):e108489. doi: 10.1371/journal.pone.0108489.

Nath KD, Burel JG, Shankar V, Pritchard AL, Towers M, Looke D, Davies JM, Upham JW. Clinical factors associated with the humoral immune response to influenza vaccination in chronic obstructive pulmonary disease. Int J Chron Obstruct Pulmon Dis. 2014;9:51-6. doi: 10.2147/COPD.S53590.

Ng OW, Chia A, Tan AT, Jadi RS, Leong HN, Bertoletti A, Tan YJ. Memory T cell responses targeting the SARS coronavirus persist up to 11 years post-infection. Vaccine. 2016 Apr 12;34(17):2008-14. doi: 10.1016/j.vaccine.2016.02.063.

Nolan T, Izurieta P, Lee BW, Chan PC, Marshall H, Booy R, Drame M, Vaughn DW. Heterologous prime-boost vaccination using an AS03B-adjuvanted influenza A(H5N1) vaccine in infants and children < 3 years of age. J Infect Dis 2014;210(11):1800-10. doi: 10.1093/infdis/jiu359.

Ou X, Liu Y, Lei X, Li P, Mi D, Ren L, Guo L, Guo R, Chen T, Hu J, Xiang Z, Mu Z, Chen X, Chen J, Hu K, Jin Q, Wang J, Qian Z. Characterization of spike glycoprotein of SARS-CoV-2 on virus entry and its immune cross-reactivity with SARS-CoV. Nat Commun. 2020 Mar 27;11(1):1620. doi: 10.1038/s41467-020-15562-9.

Paules CI, Marston HD, Fauci AS. Coronavirus infections-more than just the common cold. JAMA. 2020 Jan 23. doi: 10.1001/jama.2020.0757.

Pinti M, Appay V, Campisi J, Frasca D, Fülöp T, Sauce D, Larbi A, Weinberger B, Cossarizza A. Aging of the immune system – focus on inflammation and vaccination. Eur J Immunol. 2016 Oct;46(10):2286-2301. doi: 10.1002/eji.201546178.

Sanyaolu A, Okorie C, Marinkovic A, Patidar R, Younis K, Desai P, Hosein Z, Padda I, Mangat J, Altaf M. Comorbidity and its Impact on Patients with COVID-19. SN Compr. Clin. Med. 2020. doi.org/10.1007/s42399-020-00363-4

Sasaki S, Sullivan M, Narvaez CF, Holmes TH, Furman D, Zheng NY, Nishtala M, Wrammert J, Smith K, James JA, Dekker CL, Davis MM, Wilson PC, Greenberg HB, He XS. Limited efficacy of inactivated influenza vaccine in elderly individuals is associated with decreased production of vaccine-specific antibodies. J Clin Invest. 2011 Aug;121(8):3109-19. doi: 10.1172/JCI57834.

Shahid Z, Kalayanamitra R, McClafferty B, Kepko D, Ramgobin D, Patel R, Aggarwal CS, Vunnam R, Sahu N, Bhatt D, Jones K, Golamari R, Jain R. COVID-19 and Older Adults: What We Know. J Am Geriatr Soc. 2020 May;68(5):926-929. doi: 10.1111/jgs.16472.

Shaw AC, Goldstein DR, Montgomery RR. Age-dependent dysregulation of innate immunity. Nat Rev Immunol. 2013 Dec;13(12):875-87. doi: 10.1038/nri3547.

Shen KL, Yang YH, Jiang RM, Wang TY, Zhao DC, Jiang Y, Lu XX, Jin RM, Zheng YJ, Xu BP, Xie ZD, Liu ZS, Li XW, Lin LK, Shang YX, Shu SN, Bai Y, Lu M, Lu G, Deng JK, Luo WJ, Xiong LJ, Liu M, Cui YX, Ye LP, Li JF, Shao JB, Gao LW, Wang YY, Wang XF; China National Clinical Research Center for Respiratory Diseases; National Center for Children's Health, Beijing, China; Group of Respirology, Chinese Pediatric Society, Chinese Medical Association; Chinese Medical Doctor Association Committee on Respirology Pediatrics; China Medicine Education Association Committee on Pediatrics; Chinese Research Hospital Association Committee on Pediatrics; China Non-government Medical Institutions Association Committee on Pediatrics; China Association of Traditional Chinese Medicine, Committee on Children's Health and Medicine Research; China News of Drug Information Association, Committee on Children's Safety Medication; Global Pediatric Pulmonology Alliance. Updated diagnosis, treatment and prevention of COVID-19 in children: experts' consensus statement (condensed version of the second edition). World J Pediatr. 2020 Jun;16(3):232-239. doi: 10.1007/s12519-020-00362-4.

Stebbing J, Phelan A, Griffin I, Tucker C, Oechsle O, Smith D, Richardson P. COVID-19: combining antiviral and anti-inflammatory treatments. Lancet Infect Dis. 2020 Apr;20(4):400-402. doi: 10.1016/S1473-3099(20)30132-8.

Stokes EK, Zambrano LD, Anderson KN, Marder EP, Raz KM, El Burai Felix S, Tie Y, Fullerton KE. Coronavirus Disease 2019 Case Surveillance — United States, January 22–May 30, 2020. MMWR Morb Mortal Wkly Rep. 2020 Jun 19;69(24):759-765. doi: 10.15585/mmwr.mm6924e2.

Su S, Wong G, Shi W, Liu J, Lai ACK, Zhou J, Liu W, Bi Y, Gao GF. Epidemiology, genetic recombination, and pathogenesis of coronaviruses. Trends Microbiol. 2016 Jun;24(6):490-502. doi: 10.1016/j.tim.2016.03.003.

Tang F, Quan Y, Xin ZT, Wrammert J, Ma MJ, Lv H, Wang TB, Yang H, Richardus JH, Liu W, Cao WC. Lack of peripheral memory B cell responses in recovered patients with severe acute respiratory syndrome: a six-year follow-up study. J Immunol. 2011 Jun 15;186(12):7264-8. doi: 10.4049/jimmunol.0903490.

Thanh Le T, Andreadakis Z, Kumar A, Gómez Román R, Tollefsen S, Saville M, Mayhew S. The COVID-19 vaccine development landscape. Nat Rev Drug Discov. 2020 Apr 9. doi: 10.1038/d41573-020-00073-5.

Vaughn DW, Seifert H, Hepburn A, Dewe W, Li P, Drame M, Cohet C, Innis BL, Fries LF. Safety of AS03-adjuvanted inactivated split virion A(H1N1)pdm09 and H5N1 influenza virus vaccines administered to adults: pooled analysis of 28 clinical trials. Hum Vaccin Immunother 2014;10(10):2942-57. doi: 10.4161/21645515.2014.972149.

Vardeny O, Moran JJ, Sweitzer NK, Johnson MR, Hayney MS. Decreased T-cell responses to influenza vaccination in patients with heart failure. Pharmacotherapy. 2010 Jan;30(1):10-6. doi: 10.1592/phco.30.1.10.

Verschoor CP, Lelic A, Parsons R, Evelegh C, Bramson JL, Johnstone J, Loeb MB, Bowdish DME. Serum C-Reactive Protein and Congestive Heart Failure as Significant Predictors

of Herpes Zoster Vaccine Response in Elderly Nursing Home Residents. J Infect Dis. 2017 Jul 15;216(2):191-197. doi: 10.1093/infdis/jix257.

Vesikari T, Pepin S, Kusters I, Hoffenbach A, Denis M. Assessment of squalene adjuvanted and non-adjuvanted vaccines against pandemic H1N1 influenza in children 6 months to 17 years of age. Hum Vaccin Immunother. 2012 Sep;8(9):1283-92. doi: 10.4161/hv.21265.

Waddington CS, Walker WT, Oeser C, Reiner A, John T, Wilkins S, Casey M, Eccleston PE, Allen RJ, Okike I, Ladhani S, Sheasby E, Hoschler K, Andrews N, Waight P, Collinson AC, Heath PT, Finn A, Faust SN, Snape MD, Miller E, Pollard AJ. Safety and immunogenicity of AS03B adjuvanted split virion versus non-adjuvanted whole virion H1N1 influenza vaccine in UK children aged 6 months-12 years: open label, randomised, parallel group, multicentre study. BMJ 2010;340:c2649. doi: 10.1136/bmj.c2649.

Wang B, Li R, Lu Z, Huang Y. Does comorbidity increase the risk of patients with COVID-19: evidence from meta-analysis. Aging (Albany NY). 2020 Apr 8;12(7):6049-6057. doi: 10.18632/aging.103000.

Weinberg A, Pang L, Johnson MJ, Caldas Y, Cho A, Tovar-Salazar A, Canniff J, Schmader KE, Popmihajlov Z, Levin MJ. The Effect of Age on the Immunogenicity of the Live Attenuated Zoster Vaccine Is Predicted by Baseline Regulatory T Cells and Varicella-Zoster Virus-Specific T Cell Immunity. J Virol. 2019 Jul 17;93(15):e00305-19. doi: 10.1128/JVI.00305-19.

Wilkinson K, Wei Y, Szwajcer A, Rabbani R, Zarychanski R, Abou-Setta AM, Mahmud SM. Efficacy and safety of high-dose influenza vaccine in elderly adults: A systematic review and meta-analysis. Vaccine. 2017 May 15;35(21):2775-2780. doi: 10.1016/j.vaccine.2017.03.092.

World Health Organization. WHO Director-General's opening remarks at the media briefing on COVID-19 – 11 March 2020. https://www.who.int/dg/speeches/detail/who-director-general-s-opening-remarks-at-the-media-briefing-on-covid-19---11-march-2020. Published 11 March 2020. Accessed April 23, 2020.

Yang J, Zheng Y, Gou X, Pu K, Chen Z, Guo Q, Ji R, Wang H, Wang Y, Zhou Y. Prevalence of comorbidities in the novel Wuhan coronavirus (COVID-19) infection: a systematic review and meta-analysis. Int J Infect Dis. 2020 Mar 12. pii: S1201-9712(20)30136-3. doi: 10.1016/j.ijid.2020.03.017.

Yang WH, Dionne M, Kyle M, Aggarwal N, Li P, Madariaga M, Godeaux O, Vaughn DW. Long term immunogenicity of an AS03-adjuvanted influenza A(H1N1)pdm09 vaccine in young and elderly adults: an observer-blind, randomised trial. Vaccine 2013;31:4389-4397. doi: 10.1016/j.vaccine.2013.07.007.

Yang X, Yu Y, Xu J, Shu H, Xia J, Liu H, Wu Y, Zhang L, Yu Z, Fang M, Yu T, Wang Y, Pan S, Zou X, Yuan S, Shang Y. Clinical course and outcomes of critically ill patients with SARS-CoV-2 pneumonia in Wuhan, China: a single-centered, retrospective, observational study. Lancet Respir Med. 2020a May;8(5):475-481. doi: 10.1016/S2213-2600(20)30079-5.

Yin JK, Khandaker G, Rashid H, Heron L, Ridda I, Booy R. Immunogenicity and safety of pandemic influenza A (H1N1) 2009 vaccine: systematic review and meta-analysis. Influenza Other Respir Viruses 2011;5(5):299-305. doi: 10.1111/j.1750-2659.2011.00229.x.

Zhou F, Yu T, Du R, Fan G, Liu Y, Liu Z, Xiang J, Wang Y, Song B, Gu X, Guan L, Wei Y, Li H, Wu X, Xu J, Tu S, Zhang Y, Chen H, Cao B. Clinical course and risk factors for

mortality of adult inpatients with COVID-19 in Wuhan, China: a retrospective cohort study. Lancet. 2020 Mar 28;395(10229):1054-1062. doi: 10.1016/S0140-6736(20)30566-3.

Zhu N, Zhang D, Wang W, Li X, Yang B, Song J, Zhao X, Huang B, Shi W, Lu R, Niu P, Zhan F, Ma X, Wang D, Xu W, Wu G, Gao GF, Tan W; China novel coronavirus investigating and research team. A novel coronavirus from patients with pneumonia in China, 2019. N Engl J Med. 2020 Feb 20;382(8):727-733. doi: 10.1056/NEJMoa2001017.

Zimmermann P, Curtis N. Factors That Influence the Immune Response to Vaccination. Clin Microbiol Rev. 2019 Mar 13;32(2):e00084-18. doi: 10.1128/CMR.00084-18.

19 APPENDICES

The clinical documents included in the appendices are meant as examples only; the actual documents used during the trial may differ slightly.

19.1 Appendix 1 – Sample Ruler to Measure Local Adverse Events

Site Adverse Event Measuring Ruler

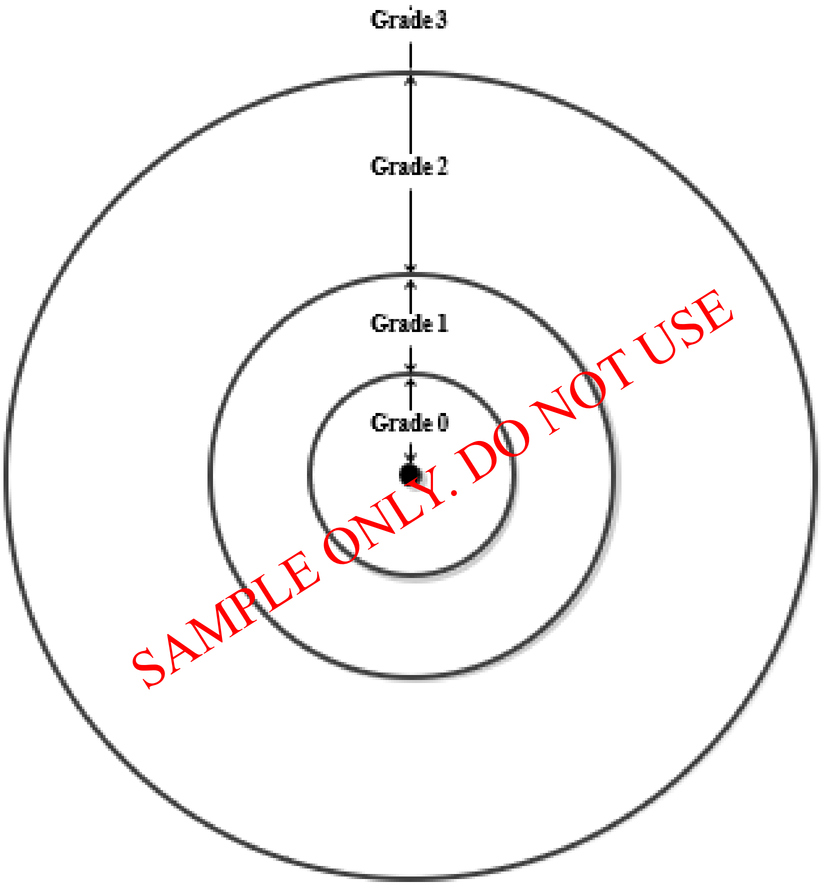

Grading for redness and swelling where the vaccine was given	
Grade 0	None or less than 25 mm
Grade 1	Between 25 mm and 50 mm
Grade 2	Between 51 and 100 mm
Grade 3	More than 100 mm
Grade 4	skin/tissue loss at the injection site (blister or ulcer formation) of any size

19.2 Appendix 2 – Subject Diary Sample Pages

DIARY (DAY 0 to DAY 7)	
**Study Name**	A Randomized, Observer-Blind, Placebo-Controlled, Phase 2/3 Study to Assess the Safety, Efficacy, and Immunogenicity of a Recombinant Coronavirus-Like Particle COVID-19 Vaccine in Adults 18 Years of Age or Older and Children and Adolescents 5-17 Years of Age
**Protocol Number**	CP-PRO-CoVLP-021
**Sponsor**	Medicago R&D Inc.<br>1020, route de l'Église, bureau 600<br>Québec (Qc), Canada G1V 3V9
**Clinical site Address**	
**Principal Investigator Name**	
**Clinical Research Coordinator**	
**24-hour Emergency Number**	

SAMPLE ONLY. DO NOT USE

DIARY (DAY 0 to DAY 7) INSTRUCTIONS

- You should fill in your Diary at approximately the same time, preferably in the evening.
- You will need to refer to this Diary during the Telephone Calls (on Day 1 and Day 8).
- You need to bring this Diary with you at the Day 3 and Day 21 visits.

- Fill in the Day 0 to Day 7 tables EVERY DAY for the first 7 days after you receive your vaccine dose.
- Fill in each day's column by entering the WORST grade for each symptom that you had during the period.
- Grayed out sections are to be left blank and will be completed by the site personnel.
- To complete this document, use blue or black ink. No pencil or liquid paper is allowed.
- If you made a mistake, cross out the incorrect information, initial and date, and write the correct answer beside.
- Date should be recorded with 2 digits for day, 3 letters for month and 4 digits for year

January	JAN	April	APR	July	JUL	October	OCT
February	FEB	May	MAY	August	AUG	November	NOV
March	MAR	June	JUN	September	SEP	December	DEC

SAMPLE ONLY. DO NOT USE

TEMPERATURE:

- To record the temperature, use the thermometer you were given at the clinic. Do not drink, eat food or smoke prior to taking your temperature.
- You should take your temperature for the first 7 days following vaccination at approximately the same time each evening and at any other time if you feel feverish.
- You will need to record the highest temperature of the day on page 4.
- If your temperature is 38°C or 100.4°F or higher, you are allowed to take over-the-counter antipyretics (e.g., acetaminophen/paracetamol, aspirin, naproxen, or ibuprofen) and you should increase the frequency of temperature measurements to approximately every 4 hours, until you don't have a fever anymore.
- Medication intake needs to be documented on the Memory Aid and will be reviewed by the site personnel.

DIARY (DAY 0 to DAY 7) INSTRUCTIONS

SYMPTOMS:

- omplete the table referring to the following periods:
  - For the 1st column: the period between the vaccination and the 30 minutes post-vaccination;
  - For the 2nd column: the period between the last evaluation (30 minutes post-vaccination) and the evening;
  - For the remaining columns: the period since the previous evaluation of the symptoms.
- If the symptom is not present, indicate 0.
- If the symptom is present, a grade should be indicated.
- Evaluate the grade of all other symptoms according to the definitions:
  - **0= No symptom**
  - **1= Does not interfere with activity**
  - **2= Repeated use of non-narcotic pain reliever (e.g. Advil, Tylenol) >24 hours or interferes with activity but does not require medical intervention**
  - **3= Any use of narcotic pain reliever (e.g. codeine, morphine) or prevents daily activity and require medical intervention**
  - **4= Visit to Emergency room or hospitalization**
- List any other problems (not listed on pages 4 to 6) on the Memory Aid (Day 0 to Day 21) page "side effects (symptoms)".
- Symptoms listed on pages 4 to 6 which persist longer than 7 days after study vaccine administration must also be listed on the Memory Aid (Day 0 to Day 21) page "side effects (symptoms)".

You received the study vaccine on: |__|__| |__|__|__| |__|__|__|__| at: |__|__| : |__|__|
dd mmm yyyy hh mm

SAMPLE ONLY. DO NOT USE

DIARY (DAY 0 to DAY 7)

**Day 0 to Day 7**		**30 min, Post-dose**	**Evening, day of dose**	**1st Day after dose**	**2nd Day after dose**	**3rd Day after dose**	**4th Day after dose**	**5th Day after dose**	**6th Day after dose**	**7th Day after dose**
**Date (dd-mmm-yyyy)**										
Oral Temperature	°C / °F									
	Causality									
Redness where the injection was given	Grade									
	Causality									
	Use the measuring tool given to you at the site to estimate the size of the red area around the injection site and indicate the grade above.									
Swelling where the injection was given	Grade									
	Causality									
	Use the measuring tool given to you at the site to estimate the size of the swollen area around the injection site and indicate the grade above.									
Pain at vaccine injection site	Grade									
	Causality									
	**0**= No symptom; **1**= Does not interfere with activity; **2**= Repeated use of non-narcotic pain reliever >24 hours or interferes with activity; **3**= Any use of narcotic pain reliever or prevents daily activity; **4**= Visit to Emergency room or hospitalization.									

SAMPLE ONLY. DO NOT USE

Comments:

MD initials and date: |__|__|__|__|__|__|__|__|__|
dd mmm yyyy

DIARY (DAY 0 to DAY 7)

**Day 0 to Day 7**		**30 min, Post-dose**	**Evening, day of dose**	**1st Day after dose**	**2nd Day after dose**	**3rd Day after dose**	**4th Day after dose**	**5th Day after dose**	**6th Day after dose**	**7th Day after dose**
Headache	Grade									
	Causality									
**GRADES**	**0**= No symptom; **1**= Does not interfere with activity; **2**= Repeated use of non-narcotic pain reliever >24 hours or interferes with activity; **3**= Any use of narcotic pain reliever or prevents daily activity; **4**= Visit to Emergency room or hospitalization.									
Muscle aches	Grade									
	Causality									
**GRADES**	**0**= No symptom; **1**= Does not interfere with activity; **2**= Interferes with activity; **3**= Prevents daily activity; **4**= Visit to Emergency room or hospitalization.									
Fatigue	Grade									
	Causality									
**GRADES**	**0**= No symptom; **1**= Does not interfere with activity; **2**= Interferes with activity; **3**= Prevents daily activity; **4**= Visit to Emergency room or hospitalization.									
Joint aches	Grade									
	Causality									
**GRADES**	**0**= No symptom; **1**= Does not interfere with activity; **2**= Interferes with activity but does not require medical intervention; **3**= Prevents daily activity and requires medical intervention; **4**= Visit to Emergency room or hospitalization.									

SAMPLE ONLY. DO NOT USE

Comments:

MD initials and date: |__|__|__|__|__|__|__|__|__|
dd mmm yyyy

DIARY (DAY 0 to DAY 7)										
**Day 0 to Day 7**		**30 min, Post-dose**	**Evening, day of dose**	**1st Day after dose**	**2nd Day after dose**	**3rd Day after dose**	**4th Day after dose**	**5th Day after dose**	**6th Day after dose**	**7th Day after dose**
Chills	Grade									
	Causality									
Feelings of general discomfort or uneasiness	Grade									
	Causality									
Feeling of swelling in the neck	Grade									
	Causality									
Feeling of swelling in the axilla (armpit)	Grade									
	Causality									

SAMPLE ONLY. DO NOT USE

GRADES **0**= No symptom; **1**= Does not interfere with activity; **2**= Interferes with activity but does not require medical intervention; **3**= Prevents daily activity and requires medical intervention; **4**= Visit to Emergency room or hospitalization.

Comments:

I confirm I have reviewed the 3 pages of solicited symptoms and evaluated the causalities when needed. I confirm I have reviewed all entries in the Comments section on this page.

MD initials and date: |_|_|_|_|_|_|_|_|_|
dd mmm yyyy

19.3 Appendix 3 – Subject Memory Aid Sample Pages

MEMORY AID (DAY 0 to DAY 42)	
**Study Name**	A Randomized, Observer-Blind, Placebo-Controlled, Phase 2/3 Study to Assess the Safety, Efficacy, and Immunogenicity of a Recombinant Coronavirus-Like Particle COVID-19 Vaccine in Adults 18 Years of Age or Older and Children and Adolescents 5-17 Years of Age
**Protocol Number**	CP-PRO-CoVLP-021
**Sponsor**	Medicago R&D Inc.<br>1020, route de l'Église, suite 600<br>Québec, QC, Canada G1V 3V9
**Clinical site Address**	
**Principal Investigator Name**	
**Clinical Research Coordinator**	
**24-hour Emergency Number**	

SAMPLE ONLY. DO NOT USE

MEMORY AID (DAY 0 to DAY 42) INSTRUCTIONS

- You should fill in your Memory aid at approximately the same time, preferably in the evening.
- You will need to refer to this Memory Aid during the Telephone Calls (on Day 1, Day 8, Day 22, and Day 29).
- You need to bring this Memory Aid with you at the Day 3, Day 21, Day 24, and Day 42 visits.

- Grayed out sections are to be left blank and will be completed by the site personnel.
- To complete this document, use blue or black ink. No pencil or liquid paper is allowed.
- If you made a mistake, cross out the incorrect information, initial and date, and write the correct answer beside.
- Date should be recorded with 2 digits for day, 3 letters for month and 4 digits for year

January	JAN	April	APR	July	JUL	October	OCT
February	FEB	May	MAY	August	AUG	November	NOV
March	MAR	June	JUN	September	SEP	December	DEC

SIDE EFFECTS (Symptoms) and MEDICATION:

- Use the space provided to record important information that you want to tell us about changes in your health. Note any health problems you experience, worsening of previous problems, or new medicines you are taking. Examples may include prescription drugs (including vaccines), over-the-counter drugs, herbal supplements and/or vitamins. Indicate each intake of medication you are usually taking as needed.
- It is especially important that you make a note of problems that required a visit to your doctor, a hospital stay, or a visit to an emergency room.
- It is also important that you make a note of changes in your medications.
- Each medication must be associated to a problem, but each problem does not require a medication associated with it.
- Indicate the start and stop date (when available) for each symptom and medication. However, if it is still ongoing at the Day 21 or Day 42 visits, you will be required to tick the ongoing checkbox.
- Please contact the study site if you experience any severe effects or if you have any concerns at any time by using the emergency contact number provided on the first page of this Memory Aid.

SAMPLE ONLY. DO NOT USE

You received the first study vaccine on: |__|__|__|__|__|__|__|__|__| at: |__|__| : |__|__|
dd mmm yyyy hh mm

You received the second study vaccine on: |__|__|__|__|__|__|__|__|__| at: |__|__| : |__|__|
dd mmm yyyy hh mm

COVID-19 INFORMATION

SYMPTOMS FOR COVID-19:

- The symptoms associated with COVID-19 are similar to the flu. These symptoms include:
  - Fever or chills;
  - Cough;
  - Shortness of breath or difficulty breathing;
  - Fatigue;
  - Muscle or body aches;
  - Headache;
  - New loss of taste or smell;
  - Sore throat;
  - Congestion or runny nose;
  - Nausea or vomiting;
  - Diarrhea.
- The symptoms listed above apply to the adult populations and also apply to children and adolescents with the following additional symptoms:
  - Abdominal pain;
  - Poor appetite.

- Individuals who have COVID-19 may show some symptoms or none at all.
- Symptoms for COVID-19 can appear up to 14 days after exposure to the virus.

SAMPLE ONLY. DO NOT USE

MEMORY AID (DAY 0 to DAY 21)

Day 0 to Day 21 ❑ Nothing to report

#	Side Effects (symptoms)	**Grade** (See below)	**Date and time it started**	**Date and time it ended**	**Did you receive medical care?**	**Validated with subject**
			dd mmm yyyy<br>hh : mm	dd mmm yyyy<br>hh : mm<br>❑ Ongoing at Day 21	❑ Yes<br>❑ No	❑ Initial and date:<br>dd mmm yyyy
			dd mmm yyyy<br>hh : mm	dd mmm yyyy<br>hh : mm<br>❑ Ongoing at Day 21	❑ Yes<br>❑ No	❑ Initial and date:<br>dd mmm yyyy

SAMPLE ONLY. DO NOT USE

GRADES **0**= No symptom; **1**= Does not interfere with activity; **2**= Repeated use of non-narcotic pain reliever >24 hours or interferes with activity but does not require medical intervention; **3**= Any use of narcotic pain reliever or prevents daily activity and does require medical intervention; **4**= Visit to Emergency room or hospitalization.

Comments:

MD initials and date: dd mmm yyyy

MEMORY AID (DAY 0 to DAY 21)

Day 0 to Day 21 ❑ Nothing to report

#	**Medication** (Name, Dose, Route and Frequency)	**Start date**	**Stop date**	**Reason(s) why you are taking this medication?**	**Validated with subject**
		dd mmm yyyy	dd mmm yyyy<br>❑ Ongoing at Day 21		❑ Initial and date:<br>dd mmm yyyy
		dd mmm yyyy	dd mmm yyyy<br>❑ Ongoing at Day 21		❑ Initial and date:<br>dd mmm yyyy
		dd mmm yyyy	dd mmm yyyy<br>❑ Ongoing at Day 21		❑ Initial and date:<br>dd mmm yyyy

SAMPLE ONLY. DO NOT USE

**Dose**			**Route**			**Frequency**		
Capsule	Tablespoon	g	Oral	Intramuscular	Ophthalmic	Once a day	Every other day	Continuous
Tablet	Teaspoon	mcg	Topical	Nasal	Intra-articular	Twice a day	Every 3 days	Intermittent
Ring	Puff	mg	Inhalation	Intravenous	Rectal	Three times/day	Every 2 weeks	Once
Application	Drops	IU	Intrauterine	Transdermal	Sublingual	1x per week	Every 6 months	As needed
Lozenge	mL	Units	Vaginal	Subcutaneous	Unknown	2x per week	Every hour	Unknown
Syringe	%	Unknown				Once a month	Once a year	

Comments:

MD initials and date: dd mmm yyyy

MEMORY AID (DAY 22 to DAY 42)

Day 22 to Day 42					❑ Nothing to report	
#	Side Effects (symptoms)	Grade (See below)	Date and time it started	Date and time it ended	Did you receive medical care?	Validated with subject
			dd mmm yyyy<br>hh : mm	dd mmm yyyy<br>hh : mm<br>❑ Ongoing at Day 42	❑ Yes<br>❑ No	❑ Initial and date:<br>dd mmm yyyy
			dd mmm yyyy<br>hh : mm	dd mmm yyyy<br>hh : mm<br>❑ Ongoing at Day 42	❑ Yes<br>❑ No	❑ Initial and date:<br>dd mmm yyyy

SAMPLE ONLY. DO NOT USE

GRADES **0**= No symptom; **1**= Does not interfere with activity; **2**= Repeated use of non-narcotic pain reliever >24 hours or interferes with activity but does not require medical intervention; **3**= Any use of narcotic pain reliever or prevents daily activity and does require medical intervention; **4**= Visit to Emergency room or hospitalization.

Comments:

MD initials and date: dd mmm yyyy

MEMORY AID (DAY 22 to DAY 42)

Day 22 to Day 42 ❑ Nothing to report

#	**Medication** (Name, Dose, Route and Frequency)	**Start date**	**Stop date**	**Reason(s) why you are taking this medication?**	**Validated with subject**
		dd mmm yyyy	dd mmm yyyy<br>❑ Ongoing at Day 42		❑ Initial and date:<br>dd mmm yyyy
		dd mmm yyyy	dd mmm yyyy<br>❑ Ongoing at Day 42		❑ Initial and date:<br>dd mmm yyyy
		dd mmm yyyy	dd mmm yyyy<br>❑ Ongoing at Day 42		❑ Initial and date:<br>dd mmm yyyy

SAMPLE ONLY. DO NOT USE

**Dose**			**Route**			**Frequency**		
Capsule	Tablespoon	g	Oral	Intramuscular	Ophthalmic	Once a day	Every other day	Continuous
Tablet	Teaspoon	mcg	Topical	Nasal	Intra-articular	Twice a day	Every 3 days	Intermittent
Ring	Puff	mg	Inhalation	Intravenous	Rectal	Three times/day	Every 2 weeks	Once
Application	Drops	IU	Intrauterine	Transdermal	Sublingual	1x per week	Every 6 months	As needed
Lozenge	mL	Units	Vaginal	Subcutaneous	Unknown	2x per week	Every hour	Unknown
Syringe	%	Unknown				Once a month	Once a year	

Comments:

MD initials and date: dd mmm yyyy

19.4 Appendix 4 – List of Potential Immune-Mediated Diseases

Neuroinflammatory disorders	Musculoskeletal disorders	Skin disorders
• Cranial nerve neuropathy, including paralysis and paresis (eg, Bell's palsy).<br>• Optic neuritis.<br>• Multiple sclerosis.<br>• Transverse myelitis.<br>• Guillain-Barré syndrome, including Miller Fisher syndrome and other variants.<br>• Acute disseminated encephalomyelitis, including site specific variants, eg, noninfectious encephalitis, encephalomyelitis, myelitis, myeloradiculoneuritis.<br>• Myasthenia gravis, including Lambert-Eaton myasthenic syndrome.<br>• Demyelinating peripheral neuropathies and plexopathies including:<br>- Chronic inflammatory demyelinating polyneuropathy.<br>- Multifocal motor neuropathy.<br>- Polyneuropathies associated with monoclonal gammopathy.<br>• Narcolepsy.<br>• Tolosa Hunt syndrome.	• Systemic lupus erythematosus and associated conditions.<br>• Systemic scleroderma (systemic sclerosis), including:<br>- Diffuse scleroderma.<br>- CREST syndrome.<br>• Idiopathic inflammatory myopathies, including:<br>- Dermatomyositis.<br>- Polymyositis.<br>• Anti-synthetase syndrome.<br>• Rheumatoid arthritis and associated conditions including:<br>- Juvenile idiopathic arthritis.<br>- Still's disease.<br>• Polymyalgia rheumatica.<br>• Spondyloarthropathies, including:<br>- Ankylosing spondylitis.<br>- Reactive arthritis (Reiter's syndrome).<br>- Undifferentiated spondyloarthritis.<br>- Psoriatic arthritis.<br>- Enteropathic arthritis.<br>• Relapsing polychondritis.<br>• Mixed connective tissue disorder.<br>• Gout.	• Psoriasis.<br>• Vitiligo.<br>• Erythema nodosum.<br>• Autoimmune bullous skin diseases (including pemphigus, pemphigoid, and dermatitis herpetiformis).<br>• Lichen planus.<br>• Sweet's syndrome.<br>• Localized scleroderma (morphea).<br>• Cutaneous lupus erythematosus.<br>• Rosacea.
**Vasculitis**	**Blood disorders**	**Others**
• Large vessels vasculitis including:<br>- Giant cell arteritis (temporal arteritis).<br>- Takayasu's arteritis.<br>• Medium sized and/or small vessels vasculitis including:<br>- Polyarteritis nodosa.<br>- Kawasaki's disease.<br>- Microscopic polyangiitis.<br>- Wegener's granulomatosis (granulomatosis with polyangiitis).	• Autoimmune hemolytic anemia.<br>• Autoimmune thrombocytopenia.<br>• Antiphospholipid syndrome.<br>• Pernicious anemia.<br>• Autoimmune aplastic anemia.<br>• Autoimmune neutropenia.<br>• Autoimmune pancytopenia.	• Autoimmune glomerulonephritis including:<br>- IgA nephropathy.<br>- Glomerulonephritis rapidly progressive.<br>- Membranous glomerulonephritis.<br>- Membranoproliferative glomerulonephritis.<br>- Mesangioproliferative glomerulonephritis.<br>- Tubulointerstitial nephritis and uveitis syndrome.

Vasculitis (continued)	Blood disorders (continued)	Others (continued)
- Churg–Strauss syndrome (allergic granulomatous angiitis or eosinophilic granulomatosis with polyangiitis).<br>- Buerger's disease (thromboangiitis obliterans).<br>- Necrotizing vasculitis (cutaneous or systemic).<br>- Antineutrophil cytoplasmic antibody (ANCA) positive vasculitis (type unspecified).<br>- Henoch-Schonlein purpura (IgA vasculitis).<br>- Behcet's syndrome.<br>- Leukocytoclastic vasculitis.		• Ocular autoimmune diseases including:<br>- Autoimmune uveitis.<br>- Autoimmune retinitis.<br>• Autoimmune myocarditis/cardiomyopathy.<br>• Sarcoidosis.<br>• Stevens-Johnson syndrome.<br>• Sjögren's syndrome.<br>• Alopecia areata.<br>• Idiopathic pulmonary fibrosis.<br>• Goodpasture syndrome.<br>• Raynaud's phenomenon.
**Liver disorders**	**Gastrointestinal disorders**	**Endocrine disorders**
• Autoimmune hepatitis.<br>• Primary biliary cirrhosis.<br>• Primary sclerosing cholangitis.<br>• Autoimmune cholangitis.	• Inflammatory bowel disease, including:<br>- Crohn's disease.<br>- Ulcerative colitis.<br>- Microscopic colitis.<br>- Ulcerative proctitis.<br>• Celiac disease.<br>• Autoimmune pancreatitis.	• Autoimmune thyroiditis (including Hashimoto thyroiditis).<br>• Grave's or Basedow's disease.<br>• Diabetes mellitus type 1.<br>• Addison's disease.<br>• Polyglandular autoimmune syndrome.<br>• Autoimmune hypophysitis.

Clinical Protocol

A Randomized, Observer-Blind, Placebo-Controlled, Phase 2/3 Study to Assess the Safety, Efficacy, and Immunogenicity of a Recombinant Coronavirus-Like Particle COVID-19 Vaccine in Adults 18 Years of Age or Older

CP-PRO-CoVLP-021; Phase 2/3

Coronavirus-Like Particle COVID-19 Vaccine

Name of Sponsor:	Medicago R&D Inc.<br>1020 route de l'Église, bureau 600<br>Québec (Qc), Canada G1V 3V9
Status:	Final version 7.2
Date:	25 January 2022

Confidential Information

Information in this protocol is confidential and should not be disclosed, other than to those directly involved in the execution or the ethical review of the study, without written authorization from Medicago R&D Inc. (herein after known as "Medicago"), and its affiliates. This study will be conducted in accordance with applicable Good Clinical Practice (GCP) guidelines, the United States Code of Federal Regulations (CFR), and International Conference on Harmonization (ICH) guidelines.

SIGNATURES

Study Title:	A Randomized, Observer-Blind, Placebo-Controlled, Phase 2/3 Study to Assess the Safety, Efficacy, and Immunogenicity of a Recombinant Coronavirus-Like Particle COVID-19 Vaccine in Adults 18 Years of Age or Older	
Study Author(s):	[Redacted]	[Redacted]
	[Redacted]	[Redacted]
	[Redacted]	[Redacted]
	[Redacted]	[Redacted]

I confirm that Medicago R&D Inc. has approved the version 7.2 (dated 25-Jan-2022) of the protocol CP-PRO-CoVLP-021 and agree that it may be issued to the relevant authorized study personnel, Independent Ethics Committees and Regulatory Authorities.

[Redacted] Date (YYYY-MM-DD)

[Redacted] Date (YYYY-MM-DD)

[Redacted] Date (YYYY-MM-DD)

SIGNATURES (continued)

[Redacted]	Date (YYYY-MM-DD)
[Redacted]	Date (YYYY-MM-DD)

INVESTIGATOR AGREEMENT

I have read the version 7.2 (dated 25-Jan-2022) Protocol No. CP-PRO-CoVLP-021 titled, "A Randomized, Observer-Blind, Placebo-Controlled, Phase 2/3 Study to Assess the Safety, Efficacy, and Immunogenicity of a Recombinant Coronavirus-Like Particle COVID-19 Vaccine in Adults 18 Years of Age or Older".

I have fully discussed the objectives of this study and the contents of this protocol with the Sponsor's representative.

I understand that the information in this protocol is confidential and should not be disclosed, other than to those directly involved in the execution or the ethical review of the study, without prior written authorization from Medicago. It is, however, permissible to discuss information contained in this protocol with a subject in order to obtain consent once Institutional Review Board (IRB) approval is obtained.

I agree to conduct this study according to this protocol and to comply with its requirements, subject to ethical and safety considerations and guidelines, and to conduct the study in accordance with International Conference on Harmonisation (ICH) guidelines, Good Clinical Practice (GCP), Investigational New Drug (IND) regulations, and other applicable regulatory requirements.

I understand that the Sponsor may decide to suspend or prematurely terminate this study at any time for whatever reason and that such a decision will be communicated to me in writing. Conversely, should I decide to withdraw from execution of the study, I will communicate my intention immediately in writing to the Sponsor.

Principal Investigator:

Name (typed or printed): ______________________________

Institution and Address: ______________________________

____

Telephone Number: ______________________________

______________________________ ______________________________
Signature Date (YYYY-MM-DD)

Note: If the address or the telephone number of the Investigator changes during the course of the study, written notification will be provided by the Investigator to the Sponsor, and a protocol amendment will not be required.

TABLE OF CONTENTS

SIGNATURES 2
INVESTIGATOR AGREEMENT 4
TABLE OF CONTENTS 5
LIST OF TABLES 11
LIST OF FIGURES 11
SYNOPSIS 13
ABBREVIATIONS 51
1 INTRODUCTION 53
1.1 Background 53
1.1.1 Elderly Population 55
1.1.2 Adults with Comorbidities 56
1.1.3 Risk:Benefit in the Different Study Populations 57
1.2 Background of the Investigational Product 57
1.2.1 Background of the Adjuvant AS03 58
1.3 Pre-clinical Studies 59
1.4 Clinical Studies 59
1.5 Overall Rationale for the Study 59
2 OBJECTIVES 61
2.1 Primary Objectives 61
2.2 Secondary Objectives 62
2.3 Exploratory Objectives 64
3 STUDY ADMINISTRATIVE STRUCTURE 65
4 STUDY DESIGN AND RATIONALE 65
4.1 Overview of Study Design 65
4.2 Rationale of Study Design 68
4.2.1 Randomization 68
4.2.2 Blinding 68
4.2.3 Dose Selection and Dosage Regimen 69
4.2.4 Route of Administration 69
4.2.5 Study Duration 69
5 STUDY POPULATION 69

5.1 Inclusion Criteria ... 69
5.2 Exclusion Criteria ... 72
5.3 Prior and Concomitant Therapy ... 74
5.4 Prohibited Therapy ... 74
6 TREATMENT ALLOCATION AND BLINDING ... 75
6.1 Randomization ... 75
6.2 Blinding ... 76
7 DOSAGE AND ADMINISTRATION ... 78
8 SPECIMENS AND CLINICAL SUPPLIES ... 78
8.1 Management of Samples ... 78
8.2 Collection of Samples ... 79
8.3 Clinical Supplies ... 81
9 TREATMENT COMPLIANCE ... 81
10 STUDY EVALUATIONS ... 82
10.1 Study Procedures ... 82
10.1.1 Overview ... 82
10.1.2 General COVID-19 Precautions at Clinical Sites ... 82
10.1.3 Screening (Visit 1 for Phase 2 Portion and Phase 3 Portion) ... 82
10.1.4 Dose 1 Vaccination (Day 0) (Visit 2 for the Phase 2 Portion and Continuation of Visit 1 for Phase 3 Portion) ... 84
10.1.4.1 Prior to Vaccination ... 84
10.1.4.2 Vaccination ... 85
10.1.4.3 Thirty Minutes Post-vaccination ... 85
10.1.5 Day 1 and Day 8 (Telephone Contact for Phase 2 portion only) ... 87
10.1.6 Surveillance for COVID-19 Cases ... 88
10.1.7 Day 3 Follow-up (Visit 3 for Phase 2 portion only) ... 90
10.1.8 Day 21 Dose 2 Vaccination (Visit 4 for Phase 2 portion and Visit 2 for Phase 3 portion) ... 91
10.1.8.1 Pre-vaccination ... 91
10.1.8.2 Dose 2 Vaccination ... 91
10.1.8.3 Post-Dose 2 Vaccination ... 92
10.1.9 Day 22 and Day 29 (Telephone Contact for Phase 2 portion only) ... 92

10.1.10 Day 24 (Visit 5 for Phase 2 portion only) ... 92
10.1.11 Day 42 (Visit 6 for Phase 2 portion and Visit 3 for Phase 3 portion) ... 92
10.1.12 Day 128 (Visit 7 for Phase 2 portion only) ... 93
10.1.13 Day 201 (Visit 8 for Phase 2 portion and Visit 4 for Phase 3 portion) ... 93
10.1.14 Day 386 (Visit 8 for Phase 2 portion and Visit 5/Telephone Contact for Phase 3 portion) ... 94
10.1.14.1 Visit 8 for Phase 2 portion and Visit 5 for Phase 3 portion ... 95
10.1.14.2 Telephone Contact for Phase 3 portion ... 95
10.1.15 Placebo Subjects who Receive the CoVLP Formulation post-RA/A for Phase 2 and Phase 3 portions and Subjects who Received a Single Dose of the CoVLP Formulation prior to RA/A ... 96
10.1.15.1 Dose 1 and Dose 2 vaccinations with the CoVLP formulation ... 96
10.1.15.1.1 Thirty Minutes Post-vaccination ... 97
10.1.15.2 Monthly Calls Thereafter (Telephone Contact) ... 97
10.1.15.3 Final Telephone Contact ... 97
10.2 Safety ... 98
10.2.1 Safety Evaluations ... 98
10.2.1.1 Solicited Local and Systemic Adverse Events ... 98
10.2.1.2 Unsolicited Adverse Events ... 99
10.2.1.3 SARS-CoV-2 Positive Reports ... 100
10.2.1.4 Clinical Laboratory Tests ... 101
10.2.1.5 Vital Signs ... 102
10.2.1.6 Physical Examinations ... 102
10.2.2 Pregnancy ... 103
10.2.3 Safety Endpoints ... 103
10.2.3.1 Primary Endpoints ... 103
10.2.3.2 Secondary Endpoints ... 103
10.2.3.3 Exploratory Endpoint ... 104
10.3 Immunogenicity ... 104
10.3.1 Immunogenicity Evaluations ... 104
10.3.2 Immunogenicity Endpoints ... 105
10.3.2.1 Primary Endpoints ... 105
10.3.2.2 Secondary Endpoints ... 105

10.3.2.3 Exploratory Endpoint 106
10.4 Efficacy 106
10.4.1 Efficacy Evaluations 106
10.4.2 Efficacy Endpoints 108
10.4.2.1 Primary Endpoint 108
10.4.2.2 Secondary Endpoints 108
10.4.2.3 Exploratory Endpoint 109
11 SUBJECT COMPLETION/WITHDRAWAL 109
11.1 Temporary Contraindications 109
11.2 Screening Failures 110
11.3 Contraindications for Subsequent Vaccination 110
11.4 Removal of Subjects from Treatment or Assessment 113
11.4.1 Follow-up of Discontinuations 114
11.4.2 Lost to Follow-up Procedures 114
11.5 Interruption of the Study 114
11.6 Premature Unblinding Due to Availability of Approved or Authorized COVID-19 Vaccines Prior to RA/A 114
12 STATISTICAL METHODS 116
12.1 Analysis Populations 116
12.1.1 Safety Analysis Set 116
12.1.2 Intention-To-Treat Set 118
12.1.3 Per Protocol Set 118
12.1.4 Immunogenicity Per Protocol Set 120
12.2 Sample Size Determination 122
12.3 Day 21 and Day 42 Immunogenicity Data Analysis and Efficacy and Safety Analyses 122
12.4 Initiation of the Phase 3 Portion 124
12.5 Safety Analyses 125
12.5.1 Analysis of Primary Endpoints 125
12.5.2 Analysis of Secondary Endpoints 125
12.5.3 Analysis of Exploratory Endpoint 126
12.6 Immunogenicity Analyses 126

12.6.1 Analysis of Primary Endpoints 127
12.6.2 Analysis of Secondary Endpoints 127
12.6.3 Analysis of Exploratory Endpoints 128
12.7 Efficacy Analyses 129
12.7.1 Analysis of Primary Endpoint 129
12.7.2 Analysis of Secondary Endpoints 130
12.7.3 Analysis of Exploratory Endpoints 131
12.8 Baseline and Subject Disposition 132
13 ADVERSE EVENT REPORTING 132
13.1 Definitions 132
13.1.1 Serious Adverse Events 132
13.1.2 Adverse Events 133
13.1.2.1 Medically Attended Adverse Events 134
13.1.3 Protocol-Defined Adverse Events of Special Interest 134
13.1.3.1 Adverse Events of Special Interest for the Coronavirus-Like Particle COVID-19 Vaccine 134
13.1.3.1.1 Vaccine-Associated Enhanced Disease 134
13.1.3.1.2 Hypersensitivity Reactions (Anaphylaxis and Severe Allergic Reactions)........ 135
13.1.3.2 Adverse Events of Special Interest for the Adjuvants 135
13.1.3.2.1 Potential Immune-Mediated Diseases and Other AESI as Listed in Section 19.4 135
13.1.4 Expectedness of an Adverse Drug Reaction 136
13.1.5 Initial SAE, AESI, and Pregnancy Reports Reporting by the Investigator 136
13.1.6 Follow-up Reporting by the Investigator 137
13.1.7 Reporting of SAEs Occurring after Study Termination 137
13.1.8 Causal Relationship 137
13.1.9 Reporting of SAEs to Health Authorities and IRB 138
13.1.10 Independent Data Monitoring Committee 138
13.1.11 Safety Review and Stopping Rules 139
14 INVESTIGATIONAL PRODUCT INFORMATION 140
14.1 Identity of Investigational Product 140
14.1.1 Study Vaccine Composition 140

14.1.2 Placebo 141
14.1.3 Preparation and Administration of Study Vaccine 141
14.1.4 Preparation, Handling, Storage, and Precautions for Use 142
14.2 Packaging 142
14.3 Labeling 142
14.4 Drug Accountability 142
15 STUDY-SPECIFIC MATERIALS 142
16 ETHICAL ASPECTS 143
16.1 Study-Specific Design Considerations 143
16.2 Regulatory Ethics Compliance 143
16.2.1 Investigator Responsibilities 143
16.2.2 Independent Ethics Committee or Institutional Review Board 143
16.2.3 Informed Consent 145
16.2.4 Privacy of Personal Data 145
17 ADMINISTRATIVE REQUIREMENTS 145
17.1 Protocol Amendments 145
17.2 Regulatory Documentation 146
17.2.1 Regulatory Approval/Notification 146
17.3 Source Documentation 146
17.3.1 Diary and Memory Aid 147
17.4 Case Report Form Completion 147
17.5 Quality Control (Study Monitoring) 147
17.6 Quality Assurance 148
17.7 Record Retention 148
17.8 Study Completion/Termination 148
17.8.1 Study Completion 148
17.8.2 Study Termination 149
17.9 Registration of Clinical Studies and Disclosure of Results 149
17.10 Publication Policy 149
17.10.1 Publication Release of Results 149
18 REFERENCES 150
19 APPENDICES 158

19.1 Appendix 1 – Sample Ruler to Measure Local Adverse Events 158
19.2 Appendix 2 – Subject Diary Sample Pages 159
19.3 Appendix 3 – Subject Memory Aid Sample Pages 165
19.4 Appendix 4 – List of Potential Immune-Mediated Diseases 172
19.5 Appendix 5 – Symptoms, Risk Factors, and Disease Severity of COVID-19 174
19.6 Appendix 6 – COVID-19 Diary Sample Pages 176
19.7 Appendix 7 – IDMC Meetings and Safety Data Review 180

LIST OF TABLES

Table 1 Time and Events Schedule: General Information for the Phase 2 Portion 42
Table 2 Time and Events Schedule: General Information for the Phase 3 Portion 45
Table 3 Time and Events Schedule: General Information for Placebo Subjects who will Receive the CoVLP Formulation Post-RA/A in the Phase 2 and 3 Portions and Subjects who Received a Single Dose of the CoVLP Formulation prior to RA/A 48
Table 4 Time and Events Schedule: Onset of COVID-19 50
Table 5 Study Populations 60
Table 6 Study Administrative Structure 65
Table 7 Estimated Blood Volume Drawn: Phase 2 Portion 79
Table 8 Estimated Blood Volume Drawn: Phase 3 Portion 80
Table 9 Severity Grades for Solicited Local and Systemic Adverse Events 99
Table 10 Clinical Laboratory Tests for the Phase 2 Portion of the Study 101
Table 11 Symptoms and Risk Factors associated with COVID-19 174
Table 12 COVID-19 Disease Severity 175
Table 13 Schedule of IDMC Meetings and Safety Data Review for Phase 2 Portion 180
Table 14 Schedule of IDMC Meetings and Safety Data Review for Phase 3 Portion 185

LIST OF FIGURES

Figure 1 Study Enrollment Flow for Phase 2 Portion 67
Figure 2 Decision Tree for Possible Premature Unblinding in Phase 2 and Phase 3 Portions Prior to RA/A 115
Figure 3 Decision Tree for Handling of Safety Data from Subjects Who Request to Become Prematurely Unblinded to Receive an Approved or Authorized COVID-19 Vaccine in Phase 2 and Phase 3 Portions Prior to RA/A 117

Figure 4 Decision Tree for Handling of Efficacy Data from Subjects Who Request to Become Prematurely Unblinded to Receive an Approved or Authorized COVID-19 Vaccine in Phase 2 and Phase 3 Portions Prior to RA/A 119

Figure 5 Decision Tree for Handling of Immunogenicity Data from Subjects Who Request to Become Prematurely Unblinded to Receive an Approved or Authorized COVID-19 Vaccine in Phase 2 and Phase 3 Portions Prior to RA/A 120

SYNOPSIS

<table>
<tr><td>Sponsor:</td><td>Medicago R&D Inc.</td></tr>
<tr><td>Investigational Product:</td><td>Coronavirus-Like Particle (CoVLP) COVID-19 Vaccine</td></tr>
<tr><td>Active Substance(s):</td><td>The Coronavirus-Like Particle COVID-19 Vaccine is composed of recombinant spike (S) glycoprotein expressed as virus-like particles (VLPs). The 3.75 µg dose of CoVLP and the number of doses to be administered in this study (i.e. two doses 21 days apart) as well as the adjuvant (i.e. AS03 manufactured by GlaxoSmithKline) have been determined based on the available Phase 1 study data.</td></tr>
<tr><td>Protocol Title:</td><td>A Randomized, Observer-Blind, Placebo-Controlled, Phase 2/3 Study to Assess the Safety, Efficacy, and Immunogenicity of a Recombinant Coronavirus-Like Particle COVID-19 Vaccine in Adults 18 Years of Age or Older</td></tr>
<tr><td>Protocol Number:</td><td>CP-PRO-CoVLP-021</td></tr>
<tr><td>Development Phase:</td><td>Phase 2/3</td></tr>
<tr><td>Study Center(s):</td><td>The study will be conducted at multiple sites in the United States (US) and Canada for the Phase 2 portion while the Phase 3 portion will also incorporate sites in Europe and Latin America.</td></tr>
<tr><td>Study Rationale:</td><td>This Phase 2/3 study is a multi-portion design to confirm that the chosen formulation and dosing regimen of CoVLP (i.e. two doses of 3.75 µg CoVLP adjuvanted with AS03 [hereafter referred to as “CoVLP formulation”] given 21 days apart) has an acceptable immunogenicity and safety profile in adults 18 years of age or older, including those with significant comorbidities (Phase 2 portion). The efficacy of this CoVLP formulation in these same Study Populations will be assessed in the Phase 3 portion.

The Phase 2 portion is a randomized, observer-blinded, placebo-controlled study to evaluate the CoVLP formulation, compared to placebo, in subjects from the following three Study Populations:
<table>
<tr><th>Study Population</th><th>Age and/or Health Category</th><th>Age Range (years)</th></tr>
<tr><td>1</td><td>Healthy adults</td><td>18-64</td></tr>
<tr><td>2</td><td>Healthy elderly adults*</td><td>≥ 65</td></tr>
</table>
</td></tr>
</table>

3	Adults with significant comorbidities*	≥ 18

*Subjects to be enrolled in country(ies) that has/have provided authorization to enroll these subjects in the study.

In each Study Population, up to 306 subjects will be randomized 5:1 to receive the CoVLP formulation:placebo. The total number of subjects in each Study Population may vary depending on when sufficient data has been accumulated to make a decision on when each Study Population can procced to the Phase 3 portion. Unless full enrollment (i.e., enrolled up to 306 subjects) has already been achieved, further enrollment into each Study Population will be closed once sufficient data has been accumulated to make the decision to proceed to Phase 3. All subjects will be followed for a period of 12 months after the last vaccination for durability of the immune responses, safety, and efficacy.

The Phase 3 portion is an event-driven, randomized, observer-blinded, placebo-controlled design that will evaluate the efficacy and safety of the CoVLP formulation, compared to placebo, in the same Study Populations #1 - #3 presented above. Up to 30 000 subjects will be enrolled in a 1:1 ratio to receive the CoVLP formulation or placebo.

The primary efficacy endpoint will be evaluated once at least 160 laboratory-confirmed COVID-19 cases (≥ 7 days post-second vaccination) have been accumulated and the median safety follow-up duration of at least 2 months (post-administration of the second vaccination) of at least 3000 subjects in each of the CoVLP formulation and placebo groups has been achieved. After the success criterion has been met for the primary efficacy endpoint of the Phase 3 portion, Regulatory Authorization/Approval (RA/A) for the CoVLP formulation will be pursued.

Upon obtaining the first RA/A in any country for the CoVLP formulation, Period 2 (Table 3) may begin to support the following:

1. Subjects in the placebo group will be offered the CoVLP formulation and will only be followed passively to identify symptomatic COVID-19 cases and for safety until the end of the study. A blood sample will be obtained immediately prior to the administration of the first dose of the CoVLP formulation for serologic analyses but no further blood

	samples will be collected in this group. Placebo recipients who choose to receive an approved or authorized COVID-19 vaccine for which they are eligible instead of the CoVLP formulation will be withdrawn from the study;<br>2. Subjects in the CoVLP formulation group will complete the study as planned with a blood draw at Day 201 to assess protection against serologically-confirmed COVID-19, unless they decide to receive an approved or authorized COVID-19 vaccine for which they are eligible and thus will be withdrawn from the study.<br><br>Subjects in the placebo group who do not want to be administered the CoVLP formulation and still want to remain in the study will continue the study as planned with a blood draw at Day 201. Upon obtaining the RA/A, the CoVLP formulation will be offered to placebo subjects in the Phase 2 portion of the study. Once the placebo subject receives the CoVLP formulation, the subject will only be followed for safety until the end of the study and no further blood draws will be collected. Subjects in the placebo group who do not wish to receive the CoVLP formulation or subjects who are in the CoVLP formulation group will continue the study as planned.<br><br>Subjects in the CoVLP formulation group will be followed for efficacy (i.e. active and passive surveillance) and safety until RA/A and then for COVID-19 cases (i.e. passive surveillance only) and safety until the end of the study (Day 386). Subjects in the placebo group will be followed for efficacy (i.e. active and passive surveillance) and safety up until RA/A and followed for safety and COVID-19 cases (i.e. passive surveillance only) until the end of the study. The sero-status of all subjects will be assessed at the time of enrollment. Serum samples will be obtained from all subjects at Day 42 so that a possible serologic correlate of protection can be evaluated once the primary efficacy outcome has been met and at later timepoints in the study. Only the subjects who initially receive the CoVLP formulation or placebo subjects who do not want the CoVLP formulation among the 288 subjects (96 subjects from each Study Population) in the immunogenicity subset will be assessed for the persistence of the vaccine-induced cellular immune responses until Day 386.

**Planned Study Period:**	The planned duration of the Phase 2 portion is approximately 14 to 16 months, from screening procedures up to the end of the Phase 2 portion.<br>The planned duration of the Phase 3 portion is approximately 13 months, from screening procedures up to the end of the Phase 3 portion.
**Study Objectives**<br>**Primary Objectives:**	**Phase 2 portion**<br>The primary objectives of the Phase 2 portion of the study are:<br>**Safety:**<br>• To assess the safety and tolerability of the CoVLP formulation, compared to placebo in each of the Study Populations, up to 21 days after the second vaccination;<br>**Immunogenicity:**<br>• To assess the immunogenicity of the CoVLP formulation, compared to placebo, up to 21 days after the second vaccination in each of the Study Populations, as determined by:<br>• Neutralizing antibody (Nab) titers induced by the vaccine against the SARS-CoV-2 virus;<br>• IFN-γ enzyme-linked immuno spot assay (ELISpot) response induced by the vaccine against the SARS-CoV-2 virus to assess the T helper 1 (Th1) type response.<br>**Phase 3 portion**<br>The primary objective of the Phase 3 portion of the study is:<br>**Efficacy:**<br>• To evaluate the efficacy of the CoVLP formulation, compared to placebo, in the prevention of laboratory-confirmed symptomatic SARS-CoV-2 infection (virologic method) starting 7 days after the second vaccination at the time of the primary vaccine efficacy (PVE) analysis (i.e., once at least 160 COVID-19 cases [≥ 7 days post-second vaccination] have been collected and the median safety follow-up duration of at least 2 months (post-administration of the second vaccination) of at least 3000 subjects in each of the CoVLP formulation and placebo groups has been achieved).
**Secondary Objectives:**	**Phase 2 portion**<br>The secondary objectives of the Phase 2 portion of the study are:

	**Immunogenicity:**<br>• To assess the relative neutralizing antibody response between the healthy adults (Study Population #1) and the healthy elderly populations (Study Population #2; each age strata) at 21 days after the second vaccination;<br>• To assess the relative neutralizing antibody response between the healthy adults (Study Population #1) and the healthy elderly adults (Study Population #2) combined and the adults with significant comorbidities (Study Population #3) at 21 days after the second vaccination;<br>• To assess the immunogenicity of the CoVLP formulation, compared to placebo, when applicable, in each of the Study Populations, as determined by the:<br>◦ Persistence of Nab titers induced by the vaccine against the SARS-CoV-2 virus at Days 128, 201, and Day 386;<br>◦ IgG (enzyme-linked immunosorbent assay [ELISA]) titers induced by the vaccine against the SARS-CoV-2 virus up to 21 days after the second vaccination and persistence of these antibodies at Day 128, Day 201, and Day 386;<br>◦ The ratio of Nab titers:IgG (ELISA) antibody titers at Day 21, Day 42, Day 128, Day 201, and Day 386;<br>◦ IFN-γ ELISpot response induced by the vaccine against the SARS-CoV-2 virus at Day 201 and Day 386;<br>◦ Interleukin-4 (IL-4) ELISpot response induced by the vaccine against the SARS-CoV-2 virus up to 21 days after the second vaccination and at Day 201 and Day 386;<br>**Safety:**<br>• To assess the relative incidence of solicited local and systemic AEs up to 7 days after the first and second vaccinations between the healthy adults (Study Population #1) and each healthy elderly population (Study Population #2) in the CoVLP formulation group prior to RA/A only;<br>• To assess the relative incidence of solicited local and systemic AEs up to 7 days after the first and second vaccinations between the healthy adults (Study Population #1) and the healthy elderly adults (Study Population #2) combined and the adults with significant comorbidities (Study Population #3) in the CoVLP formulation group prior to RA/A only;

	• To assess safety and tolerability of the CoVLP formulation, compared to placebo in each of the Study Populations , as determined by the occurrence(s) of:<br>◦ Medically attended adverse events (MAAEs), serious adverse events (SAEs), AEs leading to withdrawal, AEs of special interest (AESIs; including vaccine-associated enhanced disease [VAED]), and deaths from 22 days after the second vaccination up to RA/A;<br>**Efficacy:**<br>• To evaluate the efficacy of the CoVLP formulation, compared to placebo, in the prevention of laboratory-confirmed symptomatic SARS-CoV-2 infection (virologic method) starting 7 days after the second vaccination;<br>• To evaluate the efficacy of the CoVLP formulation, compared to placebo, in the prevention of severe COVID-19 disease starting 7 days after the second vaccination. Severe COVID-19 disease will be defined as virologically-confirmed SARS-CoV-2 infection with the occurrence of a new onset of any of the following:<br>◦ Clinical signs at rest indicative of severe systemic illness (respiratory rate ≥ 30 per minute, heart rate ≥ 125 per minute, SpO2 ≤ 93% on room air at sea level or PaO2/FiO2 < 300 mmHg);<br>◦ Respiratory failure (defined as needing high-flow oxygen, non-invasive ventilation, mechanical ventilation or ECMO);<br>◦ Evidence of shock (SBP < 90 mmHg, DBP < 60 mmHg, or requiring vasopressors);<br>◦ Significant acute renal, hepatic, or neurologic dysfunction;<br>◦ Hospitalization;<br>◦ Death.<br>**Phase 3 portion**<br>The secondary objectives of the Phase 3 portion of the study are:<br>**Efficacy:**<br>◦ To evaluate the efficacy of the CoVLP formulation in the prevention of laboratory-confirmed asymptomatic SARS-CoV-2 infection at Day 201 in subjects who were seronegative at baseline (confirmed by serologic method);

	• To evaluate the efficacy of the CoVLP formulation, compared to placebo, in the prevention of severe COVID-19 disease starting 7 days after the second vaccination at the time of the PVE analysis (see definition of severe COVID-19 disease above);<br>• To assess COVID-19-related symptoms in virologically-confirmed cases, and the severity of these symptoms up until resolution in subjects administered the CoVLP formulation compared to subjects administered the placebo at the time of the PVE analysis;<br>• To evaluate the efficacy of the CoVLP formulation, compared to placebo, in the prevention of laboratory-confirmed symptomatic SARS-CoV-2 infection (virologic method) starting after the first vaccination at the time of the PVE analysis;<br>• To evaluate the efficacy of the CoVLP formulation, compared to placebo, in the prevention of laboratory-confirmed symptomatic SARS-CoV-2 infection (virologic method) after the first vaccination and prior to the second vaccination at the time of the PVE analysis;<br>• To evaluate the efficacy of the CoVLP formulation, compared to placebo, in the prevention of laboratory-confirmed symptomatic SARS-CoV-2 infection (virologic method) after the second vaccination and prior to 7 days post-second vaccination at the time of the PVE analysis;<br>• To assess the duration and intensity of viral shedding after virologically-confirmed SARS-CoV-2 infection in subjects administered the CoVLP formulation compared to subjects administered the placebo at the time of the PVE analysis;<br>• To evaluate the efficacy of the CoVLP formulation, compared to placebo, in the prevention of laboratory-confirmed symptomatic SARS-CoV-2 infection (virologic method) by strain starting 7 days after the second vaccination at the time of the PVE analysis;<br>**Immunogenicity:**<br>• To assess the immunogenicity of the CoVLP formulation, compared to placebo when applicable, in the immunogenicity subset, as determined by:<br>◦ Nab titers induced by the vaccine against the SARS-CoV-2 virus at Day 0 (prior to vaccination; all

	subjects), Day 21, Day 42 (all subjects), Day 201 and Day 386;<br>• IgG (ELISA) titers induced by the vaccine against the SARS-CoV-2 virus up to 21 days after the second vaccination and at Day 201 and Day 386;<br>• The ratio of Nab titers:IgG (ELISA) antibody titers at Day 21, Day 42, Day 201 and Day 386;<br>• IFN-γ ELISpot response induced by the vaccine against the SARS-CoV-2 virus up to 21 days after the second vaccination and at Day 201 and Day 386;<br>• IL-4 ELISpot response induced by the vaccine against the SARS-CoV-2 virus up to 21 days after the second vaccination and at Day 201 and Day 386;<br>**Safety:**<br>• To assess the safety and tolerability of the CoVLP formulation up to the end of the study.
**Exploratory Objectives:**	**Phase 2 portion**<br>The exploratory objectives of the Phase 2 portion of the study are:<br>**Efficacy:**<br>• To assess COVID-19-related symptoms in subjects with virologically-confirmed disease, and the severity of these symptoms, up until resolution in subjects administered the CoVLP formulation compared to subjects administered the placebo;<br>**Immunogenicity:**<br>• To further characterize the immune response of the CoVLP formulation, if it is likely to be informative;<br>**Safety:**<br>• To further characterize the safety profile of the CoVLP formulation, if it is likely to be informative.<br>**Phase 3 portion**<br>The exploratory objectives of the Phase 3 portion of the study are:<br>**Efficacy:**<br>• To evaluate the efficacy of the CoVLP formulation, compared to placebo, in the prevention of severe COVID-19 disease by strain starting 7 days after the

<table>
<tr><td></td><td>
second vaccination at the time of the PVE analysis (see definition of severe COVID-19 disease above);
Immunogenicity:
• To assess the immunogenicity of the CoVLP formulation, compared to placebo when applicable, in the immunogenicity subset, as determined by:
  • Specific cell-mediated immune (CMI) response induced by the vaccine against the SARS-CoV-2 virus up to 21 days after the second vaccination and at Day 201 and Day 386, as measured by the percentage of CD4+ T cells expressing functional markers;
• To further characterize the immune response of the CoVLP formulation, if it is likely to be informative;
Safety:
• To further characterize the safety profile of the CoVLP formulation, if it is likely to be informative.
</td></tr>
<tr><td>Number of Planned Subjects:</td><td>
Overall, up to a maximum of 30 918 subjects are planned for randomization in the Phase 2 and Phase 3 portions in total.
<u>Phase 2 portion</u>:
In the Phase 2 portion of the study, up to 306 subjects will be enrolled in a 5:1 ratio to receive CoVLP formulation:placebo in each Study Population (up to 918 subjects in total). The total number of subjects in each Study Population may vary depending on when sufficient data has been accumulated to make a decision on when each Study Population can procced to the Phase 3 portion. Unless full enrollment (i.e., enrolled up to 306 subjects) has already been achieved, further enrollment into each Study Population will be closed once sufficient data has been accumulated to make the decision to proceed to Phase 3.
<u>Phase 3 portion</u>:
In the Phase 3 portion of the study, up to 30 000 subjects will be enrolled in a 1:1 ratio to receive CoVLP formulation:placebo.
Only a subset of approximately 288 subjects (96 subjects from each Study Population) will be included in the immunogenicity evaluation. These subjects will be enrolled from selected sites. Subjects that have consented to participate in this subset will be selected after the Investigational Product randomization has been performed in the randomization and trial supply management (RTSM) system. Immunogenicity subset subjects will be randomized 5:1 CoVLP:placebo. Therefore, some
</td></tr>
</table>

	subjects that opt-in to the subset may not be selected after the secondary randomization.
**Sample Size Determination:**	**<u>Phase 2 portion</u>:**<br>The sample size of up to 918 subjects will serve to confirm vaccine immunogenicity in the three Study Populations and detect sizable differences in rates of adverse events. The sample size is not large enough to detect less frequent or rare adverse reactions. With 765 subjects receiving the CoVLP formulation, there will be at least 95 % probability of observing an adverse event that occurs as frequently as 1 in 255 vaccinees.<br>**<u>Phase 3 portion</u>:**<br>The sample size of up to 30 000 subjects has been calculated using an exact conditional Poisson approach based on the following assumptions:<br>• A 1:1 ratio for CoVLP formulation:placebo;<br>• At least 90 % statistical power to reject the null hypothesis of 1) a lower limit of the 95 % confidence interval (CI) for a vaccine efficacy of the CoVLP formulation compared to placebo ≤ 30 % and 2) true efficacy < 50 %, assuming a vaccine efficacy of 60 %;<br>• A SARS-CoV-2 attack rate (i.e. symptomatic disease) in subjects receiving placebo of 0.8 % across all study populations;<br>• An attrition of 5 %.<br>With 15 000 subjects receiving the CoVLP formulation, there will be at least 95 % probability of observing an adverse event that occurs as frequently as 1 in 5000 vaccinees.<br>An immunogenicity subset sample size of 288 subjects is deemed sufficient to provide meaningful statistical assessment of immunologic outcomes.
**Study Population:**	Male and female subjects aged 18 years of age and older from ethnically and racially diverse populations will be included in each of the three Study Populations in the study.
**Dosage and Administration:**	The CoVLP formulation for the Phase 2 and Phase 3 portions of the study has been selected based on the Phase 1 study data, including acceptable safety and immunogenicity profiles.<br>**<u>Phase 2 portion</u>:**<br>Subjects will receive two intramuscular (IM) injections 21 days apart on Days 0 and 21, into the deltoid region of the alternating

	(if possible) arm (i.e., each arm will be injected once), of one of the following:<br>• CoVLP formulation;<br>• Placebo.<br>The use of the same dose of CoVLP for all Study Populations may not be adequate. Subjects in Study Population #1 (healthy adults 18 to 64 years of age) will receive the standard dose of CoVLP (3.75 µg) adjuvanted with AS03 in an injection volume of 0.5 mL.<br>Study Populations #2 and #3 (elderly adults or adults with significant comorbidities, respectively) will receive the standard dose of CoVLP (3.75 µg) adjuvanted with AS03 in an injection volume of 0.5 mL. If an acceptable immune response is not achieved at either Day 21 or Day 42, the IDMC may recommend a dose escalation to 7.5 µg CoVLP adjuvanted with AS03 in a volume of 0.5 mL, and the same number of subjects (i.e. up to 306 subjects per Study Population) in the particular Study Population will be recruited, using the same enrollment approach (i.e. use of sentinel subjects and IDMC reviews). If the higher dose of the CoVLP formulation achieves an acceptable immune response in the Phase 2 portion, then the same higher dose will be used in that particular Study Population during the Phase 3 portion of the study.<br>**<u>Phase 3 portion</u>:**<br>Each Study Population will receive the same dose of CoVLP adjuvanted with AS03 and injection volume established for that particular Study Population during the Phase 2 portion of the study.<br>Subjects will receive two IM injections 21 days apart on Days 0 and 21, into the deltoid region of the alternating (if possible) arm (i.e., each arm will be injected once), of either the CoVLP formulation or the placebo.<br>Subjects in the placebo group will be offered the CoVLP formulation once RA/A is obtained.
**Study Design:**	**<u>Phase 2 portion</u>:**<br>The Phase 2 portion is a randomized, observer-blinded, placebo-controlled, design with male and female subjects. Subjects will be enrolled from three Study Populations:

Study Population	Age and/or Health Category	Age Range (years)
1	Healthy adults	18-64

medicago

2	Healthy elderly adults*	≥ 65
3	Adults with significant comorbidities*	≥ 18

*Subjects to be enrolled in country(ies) that has/have provided authorization to enroll these subjects in the study.

In each of the three Study Populations, up to 306 subjects will be randomized 5:1 and will receive the same CoVLP formulation or placebo. The total number of subjects in each Study Population may vary depending on when sufficient data has been accumulated to make a decision on when each Study Population can procced to the Phase 3 portion. Unless full enrollment (i.e., enrolled up to 306 subjects) has already been achieved, further enrollment into each Study Population will be closed once sufficient data has been accumulated to make the decision to proceed to Phase 3. Subjects will receive two IM injections 21 days apart on Days 0 and 21, into the deltoid region of the alternating (if possible) arm (i.e., each arm will be injected once), of one of the following:

- CoVLP formulation;
- Placebo.

All subjects will begin enrollment at the same time. To the greatest extent possible given local conditions (i.e. COVID-19 vaccine roll-out), enrollment of elderly adult subjects (Study Population #2) will be stratified by age in an approximately 2:1 ratio of 65 to 74 years of age and 75 years of age and older, respectively. Enrollment of subjects in Study Population #3 will include adults with co-existing conditions such as, but not limited to, obesity or being overweight, cardiovascular diseases, hyperlipidemia, chronic kidney disease or related disorders, chronic respiratory disease, pre-diabetes and diabetes (type 1 and type 2), and treatment-controlled HIV.

The IDMC will review unblinded safety data (i.e. solicited and unsolicited safety data including laboratory safety data collected up to 7 days after each vaccination) for the first 10 sentinel subjects after each dose in all Study Populations, with the exception of Study Population #1, according to the details outlined in the IDMC Charter. All sentinel subjects will be dosed at least 30 minutes apart.

Subjects will be screened up to 14 days in advance of the first vaccine administration and will demonstrate a satisfactory baseline medical assessment by history, general physical examination, haematological, biochemical, and serological analysis. Although a test for SARS-CoV-2 antibodies will be

	performed at screening, both seronegative and seropositive subjects will be enrolled. On Day 0 and Day 21, vaccine administration will occur. Phone contacts will be made one day and eight days after each vaccine administration, specifically for review of the subject's safety and concomitant medication data. Visits to the Investigator site will occur 3 days after each vaccine administration for key safety assessments, and 21 days after each vaccine administration for key safety and immunogenicity assessments. Subjects will return to the Investigator site on Day 128, Day 201, and Day 386 (3-month, 6-month, and 12-month safety follow-ups and immunogenicity assessments after the last vaccine administration).<br>Active and passive surveillance will be conducted in the study from the day of the first vaccination (Day 0, post-vaccination) until 386 days. For passive surveillance, subjects will be instructed to report symptoms meeting the definition of new COVID-19 disease. For active surveillance, subjects will be contacted once per week via phone, text, or email and asked about symptoms of COVID-19 disease.<br><br>**Phase 3 portion:**<br>The Phase 3 portion is a randomized, observer-blinded, placebo-controlled, efficacy, safety, immunogenicity (in a subset of subjects), multicenter design with male and female subjects. Similar to the Phase 2 portion, subjects will be enrolled from three Study Populations:

Study Population	Age and/or Health Category	Age Range (years)
1	Healthy adults	18-64
2	Healthy elderly adults*	≥ 65
3	Adults with significant comorbidities*	≥ 18

*Subjects to be enrolled in country(ies) that has/have provided authorization to enroll these subjects in the study.

Study Population #2 will be roughly stratified by age in the same manner to the greatest extent possible given local conditions (i.e. COVID-19 vaccine roll-out). Enrollment of subjects in Study Population #3 will include adults with co-existing conditions such as, but not limited to, obesity (moderate or greater), cardiovascular diseases, hyperlipidemia, chronic kidney disease or related disorders, chronic respiratory disease, pre-diabetes and diabetes (type 1 and type 2), and treatment-controlled HIV.

The overall goal of the Phase 3 portion of this study is to enroll approximately 30 000 subjects who are generally representative of the overall target population in terms of racial diversity with enrichment for those at highest risk for serious COVID-19 disease (i.e. the elderly and those with significant comorbidities).

In each Study Population, subjects will be randomized to receive either the CoVLP formulation or the placebo. Subjects in the placebo group will be offered the CoVLP formulation once RA/A is obtained.

Subjects will be screened and will demonstrate a satisfactory baseline medical assessment by history and general physical examination prior to the first vaccine administration. On Day 0 and Day 21, vaccine administration will occur. Subjects will be provided with a diary and memory aid to collect safety and concomitant medication data. A visit to the Investigator site will occur on Day 21 (immunogenicity subset only) and Day 42 (all subjects) for immunogenicity assessments. Subjects who initially receive the CoVLP formulation or subjects in the placebo group who do not want the CoVLP formulation will return to the Investigator site on Day 201 and Day 386 (6-month and 12-month safety follow-ups and immunogenicity assessments [immunogenicity subset only] after the last vaccine administration).

Active surveillance will be conducted in the study from the day of the first vaccination (Day 0, post-vaccination) until RA/A and

	passive surveillance will be conducted in the study from the day of the first vaccination (Day 0, post-vaccination) until the end of the study. For passive surveillance, subjects will be instructed to report symptoms meeting the definition of new COVID-19 disease. For active surveillance, subjects will be contacted once per week via the electronic diary and asked about symptoms of COVID-19 disease. Other methods of contact (phone, text, email) will only be used as a last resort to contact the subject. In addition, to monitor asymptomatic infection, subjects who initially receive the CoVLP formulation or subjects in the placebo group who do not want the CoVLP formulation will perform a test for SARS-CoV-2 N antibodies on Day 0 and Day 201. Subjects in the placebo group who receive the CoVLP formulation post-RA/A will perform the same test on Day 0 and just prior to receiving the first dose of the CoVLP formulation.<br><br>Once subjects in the placebo group receive the CoVLP formulation (post-RA/A), they will no longer be followed for efficacy (no more active surveillance) or immunogenicity (those included in the immunogenicity subset). Passive surveillance for possible COVID-19 cases will continue however. Subjects will provide a blood draw immediately prior to receiving the first dose of the CoVLP formulation and then will be followed for safety with monthly phone contacts until Day 386, when the final study telephone contact will occur.
**Safety Evaluations:**	Safety and tolerability endpoints will include immediate AEs (30 minutes after each vaccination), solicited local and systemic AEs (up to seven days after each vaccination), unsolicited AEs up to 21 days after each vaccination, SAEs, MAAEs, AEs leading to withdrawal, AESIs (including VAED), and deaths up to the end of each phase of the study. In the Phase 3 portion post-RA/A, the same safety endpoints will be collected (including immediate AEs) from subjects in the placebo group who receive the CoVLP formulation with one exception: solicited local and systemic AEs (up to seven days after each vaccination) will not be collected after the administration of the CoVLP formulation.
**Immunogenicity Evaluations:**	Immunogenicity will be evaluated in all subjects in the Phase 2 portion and in a subset of 288 subjects in the Phase 3 portion of the study across the Study Populations with age distribution similar to the Phase 2 portion.<br><br>Immunologic outcomes will include the humoral immune response (Nab assay and ELISA) and the CMI response induced

	in subjects on Day 0, Day 21, Day 42, Day 128 (Nab assay and ELISA only for Phase 2 portion only), Day 201, and Day 386.<br>In the Phase 2 and Phase 3 portion, immunologic outcomes will be determined for subjects who originally receive the CoVLP formulation and placebo subjects who do not want the CoVLP formulation post-RA/A until the end of the study. These outcomes will also be determined in placebo subjects before RA/A if these subjects receive the CoVLP formulation post-RA/A.
**Efficacy Evaluations:**	Following the first vaccination, subjects will be asked to report symptoms associated with COVID-19 from the day of the first vaccination (Day 0, post vaccination) until the end of the Phase 2 portion or the Phase 3 portion of the study (through active or passive surveillance; active surveillance in the Phase 3 portion will end once RA/A is obtained). COVID-19 will be defined as the presence of a laboratory-confirmed (virologic method) SARS-CoV-2 infection with the occurrence of a new onset of one or more of the following symptoms:<br>• Fever or chills;<br>• Cough;<br>• Shortness of breath or difficulty breathing;<br>• Fatigue;<br>• Muscle or body aches;<br>• Headache;<br>• New loss of taste or smell;<br>• Sore throat;<br>• Congestion or runny nose;<br>• Nausea or vomiting;<br>• Diarrhea.<br>Subjects will also be advised to inform the clinical site if they have tested positive for SARS-CoV-2 infection, with or without the presence of any symptoms associated with COVID-19, and will be asked to provide a documented test result. If/when the subject begins to experience symptoms associated with COVID-19, the process for the collection of symptoms and nasal or nasopharyngeal (NP) swabs (as noted below) will be followed.<br>Subjects who report any symptoms associated with COVID-19 after the start of active and passive surveillance will be requested to visit the Investigator site to provide nasal or NP swabs to test for SARS-CoV-2 infection. During the visit, the Investigator will confirm whether nasal or NP swabs should be

	collected. If the subject cannot visit the Investigator site, then the subject may receive a visit at home (or hospital, if applicable) to collect the nasal/NP swabs samples or be provided with self-administered nasal swabs for sample collection. During the 7 days following each vaccination, Investigator judgement will be used to determine whether swabs should be collected from subjects reporting symptoms similar to those associated with COVID-19 since such symptoms may also be due to the reactogenicity of the study treatment administered rather than SARS-CoV-2 infection.<br><br>The swabs will be collected within 72 hours (preferably within 48 hours) of the reporting of a COVID-19-associated symptom. If swabs cannot be collected within 72 hours after reporting of the COVID-19-associated symptom, nasal or NP swabs should be collected at the next available opportunity for the subject to visit the Investigator site or at home (or hospital, if applicable).<br><br>Two swabs per event per subject will be collected and submitted for analysis using a virologic method, at the central virology laboratory. To mitigate the risk of long delays in shipping samples to the central virology laboratory, an additional local virologic test will be performed. The purpose of this supplementary test is to provide a preliminary diagnosis and permit appropriate symptom-recording and sample collection as quickly as possible.<br><br>All subjects with virologically-confirmed SARS-CoV-2 infection, based on results from the locally performed testing, will be asked to provide serial (every other day) self-administered nasal swabs (2 swabs every other day for the central virology laboratory) for approximately 2 weeks. In the event, the COVID-19-associated symptoms persist for more than 2 weeks, then the Investigator's clinical judgement is required to extend the collection of swabs beyond the 2 weeks from the subject. If the COVID-19-associated symptoms have resolved in less than 2 weeks and the Investigator believes it is likely the subject has virologically recovered, then two consecutive negative results, obtained locally, are required prior to stopping the collection of the self-administered swabs for the purpose of testing at the central laboratory.<br><br>At the same visit where the Investigator will confirm whether nasal or NP swabs should be collected, subjects will be trained on daily recording of their COVID-19-associated symptoms using a COVID-19 diary and will be asked to immediately start recording their symptoms in the COVID-19 diary. Subjects will collect any COVID-19-associated symptoms in their COVID-19

	diary until a negative swab result for the subject is received from the central virology laboratory or until the subject has no more COVID-19-associated symptoms. The swab sample results from the central virology laboratory will be considered the primary results for vaccine efficacy evaluation. For subjects who are hospitalized with severe COVID-19 and cannot provide samples for testing at the central virology laboratory, SARS-CoV-2 testing results from the hospital may be considered for use in the evaluation of vaccine efficacy if documentation of the local testing procedures and results is available.
**Primary Endpoints:**	**Phase 2 portion**<br>In the Phase 2 portion, the primary endpoints are:<br>**Safety:**<br>• Occurrence, intensity, and relationship to vaccination of immediate AEs (30 minutes after each vaccination);<br>• Occurrence and intensity of solicited local and systemic AEs (for seven days following each vaccine administration);<br>• Occurrence, intensity, and relationship of unsolicited AEs for 21 days following each vaccine administration;<br>• Number and percentage of subjects with normal and abnormal clinically significant urine, haematological and biochemical values prior to and three days following each vaccination;<br>• Occurrences of SAEs, MAAEs, AEs leading to withdrawal, AESIs (including VAED), and deaths up to 21 days following each vaccine administration;<br>**Immunogenicity:**<br>• Nab response induced in each Study Population against the SARS-CoV-2 virus on Days 0, 21, and 42 will be analyzed using the following parameter: geometric mean titers (GMT), seroconversion (SC) rate, and geometric mean fold rise (GMFR);<br>• Specific Th1 CMI response induced in each Study Population against the SARS-CoV-2 virus on Days 0, 21, and 42, as measured by IFN-γ ELISpot.

	**Phase 3 portion**<br>In the Phase 3 portion, the primary endpoint is:<br>**Efficacy:**<br>• First occurrence, in a subject, of laboratory-confirmed (virologic method) symptomatic SARS-CoV-2 infection (≥ 7 days post-second vaccination) at the time of the PVE analysis.
**Secondary Endpoints:**	**Phase 2 portion**<br>In the Phase 2 portion, the secondary endpoints are:<br>**Immunogenicity:**<br>• Relative neutralizing antibody response between the healthy adults (Study Population #1) and the healthy elderly populations (Study Population #2; each age strata) induced at 21 days after the second vaccination will be analyzed using the following parameter: GMT;<br>• Relative neutralizing antibody response for the combination of healthy adults (Study Population #1) and healthy elderly adults (Study Population #2) compared to adults and elderly adults with significant comorbidities (Study Population #3) induced at 21 days after the second vaccination will be analyzed using the following parameter: GMT;<br>• Persistence of the Nab antibody response induced in each Study Population against the SARS-CoV-2 virus at Day 128, Day 201, and Day 386 will be analyzed using the following parameters: GMT, SC rate, and GMFR;<br>• Specific antibody response induced in each Study Population against the SARS-CoV-2 virus will be measured by the total IgG levels on Days 0, 21, and 42, and the persistence of these antibodies at Days 128, 201, and 386 and analyzed using the following parameters: GMT, SC rate, and GMFR;<br>• The ratio of neutralizing antibody titers: IgG (ELISA) antibody titers at Day 21, Day 42, Day 128, Day 201, and Day 386;<br>• Specific Th1 CMI response induced in each Study Population against the SARS-CoV-2 virus on Day 201 and Day 386, as measured by IFN-γ ELISpot;<br>• Specific T helper 2 (Th2) CMI response induced in each Study Population against the SARS-CoV-2 virus on

	Days 0, 21, 42, 201, and 386, as measured by IL-4 ELISpot;<br>**Safety:**<br>• Relative occurrence of solicited local and systemic AEs by intensity grades for seven days following each vaccine administration between the healthy adults (Study Population #1) and each healthy elderly population (Study Population #2);<br>• Relative occurrence of solicited local and systemic AEs by intensity grades for seven days following each vaccine administration for the combination of healthy adults (Study Population #1) and the healthy elderly adults (Study Population #2) compared to adults and elderly adults with significant comorbidities (Study Population #3);<br>• Occurrences of SAEs, MAAEs, AEs leading to withdrawal, AESIs (including VAED), and deaths from Day 43 to Day 201;<br>• Occurrences of SAEs, MAAEs, AEs leading to withdrawal, AESIs (including VAED), and deaths from Day 202 to Day 386.<br>**Efficacy:**<br>• First occurrence, in a subject, of laboratory-confirmed (virologic method) symptomatic SARS-CoV-2 infection (≥ 7 days post-second vaccination);<br>• Occurrences of severe COVID-19 disease (≥ 7 days post-second vaccination). Refer to the definition of severe COVID-19 disease in the Secondary Objectives section above.<br><br>**Phase 3 portion**<br>In the Phase 3 portion, the secondary endpoints are:<br>**Efficacy:**<br>• Occurrences of laboratory-confirmed asymptomatic SARS-CoV-2 infection at Day 201 in subjects who were seronegative at baseline (confirmed by the ELISA method for the N protein);<br>• Occurrences of severe COVID-19 disease (≥ 7 days post-second vaccination) at the time of the PVE analysis. Refer to the definition of severe COVID-19 disease in the Secondary Objectives section above;

	• Occurrence and intensity of COVID-19-related symptoms in virologically-confirmed cases up until resolution of the symptoms in subjects administered the CoVLP formulation compared to subjects administered the placebo at the time of the PVE analysis;<br>• First occurrence, in a subject, of laboratory-confirmed (virologic method) symptomatic SARS-CoV-2 infection (≥ first vaccination) at the time of the PVE analysis;<br>• First occurrence, in a subject, of laboratory-confirmed (virologic method) symptomatic SARS-CoV-2 infection (≥ first vaccination and < second vaccination) at the time of the PVE analysis;<br>• First occurrence, in a subject, of laboratory-confirmed (virologic method) symptomatic SARS-CoV-2 infection (≥ second vaccination and < 7 days post-second vaccination) at the time of the PVE analysis;<br>• Duration and intensity of viral shedding after virologically-confirmed SARS-CoV-2 infection at the time of the PVE analysis;<br>• First occurrence, in a subject, of laboratory-confirmed (virologic method) symptomatic SARS-CoV-2 infection (≥ 7 days post-second vaccination) by strain at the time of the PVE analysis;<br>**Immunogenicity:**<br>• In the immunogenicity subset, Nab antibody response induced in each Study Population against the SARS-CoV-2 virus on Days 0 (all subjects), 21, 42 (all subjects), 201, and 386 analyzed, using the following parameters: GMT, SC rate, and GMFR;<br>• In the immunogenicity subset, specific antibody response induced in each Study Population against the SARS-CoV-2 virus measured by the total IgG levels on Days 0, 21, 42, 201, and 386, and analyzed using the following parameters: GMT, SC rate, and GMFR;<br>• In the immunogenicity subset, the ratio of neutralizing antibody titers:IgG (ELISA) antibody titers at Day 21, Day 42, Day 201, and Day 386;<br>• In the immunogenicity subset, specific Th1 CMI response induced in each Study Population against the SARS-CoV-2 virus on Days 0, 21, 42, 201, and 386, as measured by IFN-γ ELISpot;<br>• In the immunogenicity subset, specific Th2 CMI response induced in each Study Population against the

	SARS-CoV-2 virus on Days 0, 21, 42, 201, and 386, as measured by IL-4 ELISpot;<br>**Safety:**<br>• Occurrence, intensity, and relationship to vaccination of immediate AEs (30 minutes after each vaccine administration);<br>• Occurrence and intensity of solicited local and systemic AEs (for seven days following each vaccine administration) for the initial set of vaccinations;<br>• Occurrence, intensity, and relationship of unsolicited AEs for 21 days following each vaccine administration;<br>• Occurrences of SAEs, MAAEs, AEs leading to withdrawal, AESIs (including VAED, anaphylaxis, and severe allergic reactions), and deaths up to 21 days following each vaccine administration;<br>• Occurrences of SAEs, MAAEs, AEs leading to withdrawal, AESIs (including VAED, anaphylaxis, and severe allergic reactions), and deaths from Day 43 to Day 201;<br>• Occurrences of SAEs, MAAEs, AEs leading to withdrawal, AESIs (including VAED, anaphylaxis, and severe allergic reactions), and deaths from Day 202 to Day 386.
**Exploratory Endpoints:**	**Phase 2 portion**<br>In the Phase 2 portion, the exploratory endpoints are:<br>**Efficacy:**<br>• Occurrence and intensity of COVID-19-related symptoms up to resolution of the symptoms;<br>**Immunogenicity:**<br>• Further characterization of the immune response of the CoVLP formulation, if it is likely to be informative;<br>**Safety:**<br>• Further characterization of the safety profile of the CoVLP formulation, if it is likely to be informative.<br><br>**Phase 3 portion**<br>In the Phase 3 portion, the exploratory endpoints are:<br>**Efficacy:**

	• Occurrences of severe COVID-19 disease (≥ 7 days post-second vaccination) by strain. Refer to the definition of severe COVID-19 disease in the Secondary Objectives section above;<br>**Immunogenicity:**<br>• In the immunogenicity subset, specific CMI response induced in each Study Population against the SARS-CoV-2 virus up to 21 days after the second vaccination and at Day 201 and Day 386, as measured by the percentage of CD4+ T cells expressing functional markers;<br>• Further characterization of the immune response of the CoVLP formulation, if it is likely to be informative;<br>**Safety:**<br>• Further characterization of the safety profile of the CoVLP formulation, if it is likely to be informative.
**Safety Review:**	During the study, the IDMC will review safety data to ensure the ongoing safety of all the subjects in the study. During the Phase 2 portion, the safety data (i.e. solicited and unsolicited safety data including laboratory safety data collected up to 7 days after each vaccination) for the first 10 sentinel subjects after each dose in all Study Populations, with the exception of Study Population #1, will be reviewed by the IDMC before permitting enrollment of the remaining subjects, according to the details outlined in the IDMC Charter. Additional information about the planned and ad hoc IDMC review meetings is also available in the IDMC Charter.<br>During the Phase 2 portion, the IDMC will review the safety data collected up to Day 28 from all the subjects in each Study Population to confirm that each Study Population can be included in the Phase 3 portion of the study. These safety data, as well as the Day 21 and Day 42 immunogenicity data, will be provided to Regulatory Authority(ies) as required for review prior to enrolling subjects from their jurisdictions in each Study Population into the Phase 3 portion of the study.
**Statistical Methods:**	**Populations:**<br>Descriptive statistical analyses will be performed on pre-defined population sets (the safety analysis set [SAS], the Intention-to-Treat [ITT] set, the per protocol [PP] set, and the immunogenicity per protocol [IPP] set) for each Study

medicago

	Population in the Phase 2 and Phase 3 portions, based on what will be provided in the Statistical Analysis Plan (SAP).<br><br>In each of the Study Populations for the Phase 2 and Phase 3 portions:<br><br>• All safety analyses will be performed using the SAS;<br>• All efficacy analyses will be performed using both the PP set and the ITT set;<br>• All immunogenicity analyses will be performed using both the IPP set and the immunogenicity ITT set;<br>• In the Phase 2 portion, analyses using the PP set and the IPP set will be considered the primary analyses for efficacy and immunogenicity, respectively;<br>• In the Phase 3 portion, analyses using the ITT set will be considered the primary analyses for efficacy. Analyses using the IPP set will be considered the primary analyses for immunogenicity.<br><br>As approved or authorized vaccines for COVID-19 become publicly available to subjects in some regions, subjects may request to become prematurely unblinded. The criteria to become prematurely unblinded in this circumstance is discussed in Section 6.2 and subject status upon premature unblinding is detailed in Section 11.6. Details of how the data for prematurely unblinded subjects will be handled is described in Section 12.1.
	**Phase 2 Portion: Day 21 and Day 42 Immunogenicity Data Analysis:**<br><br>The unblinded statistical team will analyze the immunogenicity data after Day 21 and Day 42 in each of the Study Populations. The data analysis will involve selected individuals from the unblinded statistical team and Medicago who will be unblinded (as described in Blinding section). The Day 21 analysis in Study Population #1 and the Day 21 and/or Day 42 analysis in Study Populations #2 and #3 will confirm whether an acceptable immune response has been induced in each Study Population prior to the initiation of the Phase 3 portion of the study (refer to the Phase 3 Portion Initiation section below for details of the criteria that will be used), without having to wait until after the end of the 386 day follow-up period for study completion. These immunogenicity data, as well as the Day 28 safety data, will be provided to Regulatory Authority(ies) as required for review prior to enrolling subjects from their jurisdictions in each Study Population into the Phase 3 portion of the study.

	If the Day 42 analysis in Study Population #2 or #3 confirms that an acceptable response has been induced (refer to Section 12.4 for details of the criteria that will be used at Day 42), the particular Study Population will be permitted to proceed into the Phase 3 portion of the study. However, if at Day 42, the immune response for Study Population #2 or #3 is still relatively weak compared to the healthy adult Day 42 responses, an ad hoc IDMC meeting will be convened to consider changes to the Protocol to permit alternate vaccination strategies in the particular Study Population.<br>**Phase 3 portion: Efficacy and Safety Analyses:**<br>When at least 160 laboratory-confirmed COVID-19 cases (≥ 7 days post-second vaccination) have been collected and the median safety follow-up duration of at least 2 months (post-administration of the second vaccination) of at least 3000 subjects in each of the CoVLP formulation and placebo groups has been achieved, the unblinded statistical team will perform the final analysis and produce final efficacy outputs for the Phase 3 portion of the efficacy data in order to test for vaccine efficacy. If the primary efficacy success criterion is met during this analysis, RA/A will be pursued.<br>Upon obtaining the first RA/A in any country for the CoVLP formulation, Period 2 (Table 3) may begin to support the following:<br>1. Subjects in the placebo group will be offered the CoVLP formulation and will only be followed passively to identify symptomatic COVID-19 cases and for safety until the end of the study. A blood sample will be obtained immediately prior to the administration of the first dose of the CoVLP formulation for serologic analyses but no further blood samples will be collected in this group. Placebo recipients who choose to receive an approved or authorized COVID-19 vaccine for which they are eligible instead of the CoVLP formulation will be withdrawn from the study;<br>2. Subjects in the CoVLP formulation group will complete the study as planned with a blood draw at Day 201 to assess protection against serologically-confirmed COVID-19, unless they decide to receive an approved or authorized COVID-19 vaccine for which they are eligible and thus will be withdrawn from the study.

	**Phase 3 Portion Initiation:**<br>The start of the Phase 3 portion of the study will be determined independently for each Study Population and will be based on the outcomes of the following two assessments during the Phase 2 portion of the study:<br>• Day 21 Data Analysis of the immunogenicity data in Study Population #1 (described above);<br>• Day 21 and/or Day 42 Data Analysis of the immunogenicity data in Study Population #2 and Study Population #3 (described above);<br>• Day 28 Safety Review in Study Population #1, Study Population #2, and Study Population #3 (described in the Safety Review section above).<br>Study Population #1: If the safety and immunogenicity profiles of the CoVLP formulation observed in the Phase 1 study are acceptable in the Phase 2 portion, this group will proceed into the Phase 3 portion. Another key outcome will be the absence of a significant safety signal as assessed by the IDMC.<br>Study Populations #2 and #3: If the safety and immunogenicity profiles of the CoVLP formulation in these populations are acceptable, entry of Study Population #2 or #3 into the Phase 3 portion will be based on both the absence of an important safety signal and demonstration of an acceptable immune response as assessed by the IDMC. The criteria for acceptability will be:<br>• No statistically significant difference in the Nab responses at Day 42 between Study Population #1 and Study Population #2;<br>• No statistically significant difference in the Nab responses at Day 42 between Study Population #1 and Study Population #2 combined and Study Populations #3;<br>• No significant higher incidence of solicited local and systemic AEs reported 7 days after each vaccine administration in Study Population #2 compared to Study Population #1;<br>• No significant higher incidence of solicited local and systemic AEs reported 7 days after each vaccine administration in Study Population #3 compared to Study Population #1 and Study Population #2 combined.<br>For Study Population #2, the same criteria will be applied to the comparisons of the two age strata for Study Population #2 versus Study Population #1. In the event of a significant

	difference between Study Population #1 and one of the age strata for Study Population #2, the next highest dose level (7.5 µg CoVLP) that can be administered to this population will be assessed in the same number of subjects (as planned for the age stratum in the Phase 2 portion).<br>**Publication/Release of Study Results for the Phase 2 and Phase 3 Portions**<br>After presenting to Regulatory Authority(ies), Medicago intends to release and/or publish the clinical data. Data that are released and/or published will only present group level results and will not involve any unblinding or the presentation of subject level data.<br>**Statistical Analyses:**<br>All descriptive and inferential statistical analyses will be performed using Statistical Analysis System® (SAS®) software (version 9.4 or higher).<br>In general, categorical data will be summarized using the number and percent of subjects in each category and continuous data will be summarized using descriptive statistics (mean or geometric mean, median, standard deviation, minimum, and maximum).<br>Analyses of primary, secondary, and exploratory endpoints will include each Study Population as a whole as well as comparisons by age strata, where applicable.<br>**Safety Analyses:**<br>Safety and tolerability endpoints will be summarized by treatment using descriptive statistics.<br>**Immunogenicity Analyses:**<br>For the immunogenicity analyses, point estimates and two-sided 95 % CI for all immunogenicity endpoints and responses for the treatment groups will be calculated. GMT will be compared among treatment groups by using Analysis of Variance (ANOVA) on log-transformed data. GMFR will be derived by using Analysis of Covariance (ANCOVA) to model the difference in the log of the titer values between Day 21/42/128/201/386 and Day 0, with treatment group as main effect and baseline titer as covariate. For GMT and GMFR comparisons, Tukey's range test will be performed to show p-values for the pairwise comparisons between treatment groups. Fisher's exact tests will be used to compare SC among treatment

	groups. The CMI response will be compared among treatment groups using appropriate non-parametric (Wilcoxon) models.<br>**Efficacy Analyses:**<br>Once at least 160 COVID-19 cases (≥ 7 days post-second vaccination) have accumulated during the study, the primary efficacy endpoint of the Phase 3 portion of the study will be evaluated to determine if the vaccine efficacy success criterion has been met. For the efficacy endpoints, vaccine efficacy (VE) will be evaluated.<br>The primary assessment of VE will be evaluated as the incidence rate ratio of CoVLP vaccinated versus placebo subjects to develop laboratory-confirmed symptomatic SARS-CoV-2 infection ≥ 7 days after the second vaccination, as per the following formula:<br>$$\text{VE}=(1\text{-IRR})=(1\text{-}(\frac{a}{N_1} \div \frac{b}{N_0}))*100\ \%$$<br>Where:<br>IRR= incidence rate ratio<br>a= number of subjects with at least one positive COVID-19 case; and<br>b= number of subjects with at least one positive COVID-19 cases; and<br>N_1= person-years for subjects vaccinated with CoVLP; and<br>N_0= person-years in subjects who received placebo.<br>Assuming the number of cases in each arm follows a Poisson distribution, then conditioning on the total number of cases and the ratio of person-time of follow up, exact 95 % CIs for the incidence rate ratio IRR, and hence for VE = 1-IRR, are obtained via binomial distribution calculations.<br>The VE success criterion for the primary efficacy endpoint, in the Phase 3 portion, is defined as a ≥ 50 % point estimate and a > 30 % lower limit of the 95 % CI.<br>VE will also be calculated for the secondary and exploratory efficacy endpoints. The VE success criterion for the secondary efficacy endpoints, in the Phase 3 portion, is defined as > 0 % lower limit of the 95 % CI.<br>To assess the duration and intensity of viral shedding after SARS-COV-2 infection, point estimates and two-sided 95 % CI will be calculated. The area under the curve of the viral titer

	curve, based on serial quantitative RT-PCR results, will be compared between treatment groups using a linear mixed model with testing dates considered as repeated measures. The peak viral shedding assessed by the lowest CT value and the number of days to negative test will be compared between treatment groups using an ANOVA.

Table 1 Time and Events Schedule: General Information for the Phase 2 Portion

Visit Type	Screening	Vaccination	Post-vaccination Visits/Contacts										
**Study Day**	**Day -14 to 0**	**Day 0**	**Day 1 (+ 1)**	**Day 3 (± 1)**	**Day 8 (± 1)**	**Day 21 (± 2)[8]**	**Day 22 (+ 1)**	**Day 24 (± 1)**	**Day 29 (± 1)**	**Day 42 (± 2)**	**Day 128 (± 14)**	**Day 201 (± 14)[10]**	**Day 386 (± 14)[11]**	**Unscheduled Contact for Unblinding[12]**
**Visit Number**	**1**	**2**	**Phone**	**3**	**Phone**	**4**	**Phone**	**5**	**Phone**	**6**	**7**	**8**	**9**	**Phone**
Informed consent	X													
Demographics	X													
Height, weight, and BMI	X	X												
Medical history/prior medication	X	X[1]												
Physical examination	X[2]													
Vital Signs	X	X[3]		X		X[3]		X		X	X	X	X	
Inclusion/exclusion criteria	X	X												
Urinalysis	X			X		X[9]		X						
Pregnancy test[4]	X	X[9]				X[9]				X				
Randomization		X												
Test for SARS-CoV-2 antibodies	X													
Serology tests for HIV, Hepatitis B, and Hepatitis C	X													
Blood chemistry And Haematology	X			X		X[9]		X						
Immunogenicity - Serology (Nab assay and ELISA)		X[9]				X[9]				X	X	X	X	

Visit Type	Screening	Vaccination	Post-vaccination Visits/Contacts											
**Study Day**	**Day -14 to 0**	**Day 0**	**Day 1 (+ 1)**	**Day 3 (± 1)**	**Day 8 (± 1)**	**Day 21 (± 2)[8]**	**Day 22 (+ 1)**	**Day 24 (± 1)**	**Day 29 (± 1)**	**Day 42 (± 2)**	**Day 128 (± 14)**	**Day 201 (± 14)[10]**	**Day 386 (± 14)[11]**	**Unscheduled Contact for Unblinding[12]**
**Visit Number**	**1**	**2**	**Phone**	**3**	**Phone**	**4**	**Phone**	**5**	**Phone**	**6**	**7**	**8**	**9**	**Phone**
Immunogenicity – CMI Response (ELISpot and ICS)		X[9]				X[9]				X		X	X	
Vaccine admin		X				X								
Immediate surveillance (30 minutes)		X				X								
Provide and collect diary and memory aid instructions		X		X		X		X		X	X	X	X	
Oral digital thermometer and instructions on reactions[5]		X				X								
Collection of solicited local/ systemic adverse events		X	X	X	X	X	X	X	X					
Concomitant medications[6]		At any time during the study period												
Collection of COVID-19 symptoms through passive and active surveillance		<u>Passive Surveillance</u>: Subjects will be instructed to contact the study site if they experience symptoms of COVID-19 from the day of the first vaccination (Day 0, post vaccination) until the end of the Phase 2 portion of the study.<br><u>Active Surveillance</u>: Between the day of the first vaccination (Day 0, post vaccination) and the end of the Phase 2 portion of the study, the subjects will be contacted once per week via phone, text, or email and asked about symptoms of COVID-19.												
Testing for laboratory confirmatio		Subjects who report any symptoms associated with COVID-19 after the start of the active and passive surveillance will be requested to visit the Investigator site to provide nasal or NP swabs (2 per subject per event) to test for COVID-19. If the subject cannot visit the Investigator site, then the subject may receive a												

Visit Type	Screening	Vaccination	Post-vaccination Visits/Contacts											
**Study Day**	**Day -14 to 0**	**Day 0**	**Day 1 (+ 1)**	**Day 3 (± 1)**	**Day 8 (± 1)**	**Day 21 (± 2)[8]**	**Day 22 (+ 1)**	**Day 24 (± 1)**	**Day 29 (± 1)**	**Day 42 (± 2)**	**Day 128 (± 14)**	**Day 201 (± 14)[10]**	**Day 386 (± 14)[11]**	**Unscheduled Contact for Unblinding[12]**
**Visit Number**	**1**	**2**	**Phone**	**3**	**Phone**	**4**	**Phone**	**5**	**Phone**	**6**	**7**	**8**	**9**	**Phone**
n of SARS-CoV-2 infection		visit at home to collect the nasal/NP swabs samples or be provided with self-administered nasal swabs for sample collection. The swabs will be collected within 72 hours (preferably within 48 hours) of the reporting of a COVID-19-associated symptom. If swabs cannot be collected within 72 hours after reporting of the COVID-19-associated symptom, nasal or NP swabs should be collected at the next available opportunity for the subject to visit the Investigator site.												
AEs, SAEs, MAAEs, and AESIs[7]		At any time during the study period.												
Termination record													X	

[1] Record any changes in medical history and medications and confirmation that the subject continues to meet all inclusion and no exclusion criteria since screening.

[2] A limited physical examination will occur at screening. History/symptom-directed physical examinations may be performed at other visits if 1) new complaints or concerns are raised by either the study subject or study staff and 2) deemed to be necessary by the Investigator.

[3] Record prior to study vaccine, after the observation period and as deemed necessary.

[4] In all females of childbearing potential in Study Populations #1 and #3; it will be tested in serum at screening and in urine at Days 0, 21, and 42.

[5] After vaccination, subjects will be instructed on the diary and memory aid provided for their use for recording reactions, AEs, and concomitant medication use.

[6] After the Day 42 visit, concomitant medication collection will be limited to those used to treat an SAE, MAAE, AE leading to withdrawal, AESIs, or an AE that occurred before Day 42; any vaccine not foreseen in the study protocol; and prohibited medications.

[7] AEs will be collected up to Day 42; SAEs, MAAEs, AEs leading to withdrawal, and AESIs will be collected through to the end of the study. Investigators should report SAEs, AESIs, and pregnancy reports to Sponsor within 24 hours of the Investigator learning of the event. Specific contacts for the collection of information regarding all of these events will occur on Day 386 (during the Day 386 final visit) for SAEs, MAAEs, AEs leading to withdrawal, and AESI.

[8] All efforts will be done to have subjects returning on planned date for Day 21 activities. If for any reason the visit is done before or after this planned date, subsequent visits/procedures (Days 22, 24, 29, 42, 201, and 386) will be adjusted accordingly.

[9] Blood and urine samples are to be collected prior to vaccination on the respective day.

[10] If RA/A occurs prior to Day 201, blood samples on Day 201 will not be collected from subjects in the placebo group who receive the CoVLP formulation post-RA/A. These subjects will follow the Table 3: Time and Events Schedule once they agree to receive the CoVLP formulation post-RA/A.

[11] If RA/A occurs prior to Day 386, blood samples on Day 386 will not be collected from subjects in the placebo group who receive the CoVLP formulation post-RA/A. These subjects will follow the Table 3: Time and Events Schedule once they agree to receive the CoVLP formulation post-RA/A.

[12] Once RA/A is obtained, subjects in the study will be contacted by phone (or any other method of communication such as the electronic diary, text, or email) and informed of the treatment they received in the study. Subjects in the placebo group will be offered the CoVLP formulation. Subjects in the placebo group who receive the CoVLP formulation (post-RA/A) will follow the Table 3: Time and Events Schedule. Subjects in the placebo group who do not want the CoVLP formulation and remain in the study will continue to follow the Table 1: Time and Events Schedule.

Table 2 Time and Events Schedule: General Information for the Phase 3 Portion

**Visit Type**	**Screening / Vaccination**	**Post-vaccination Visits/Contacts**				
**Study Day**	**Day 0**	**Day 21 (± 2) [8]**	**Day 42 (± 2) [10]**	**Day 201 (± 14)[12]**	**Day 386 (± 14)[13]**	**Unscheduled Contact for Unblinding[14]**
**Visit Number**	**1**	**2**	**3**	**4**	**5/Phone**	**Phone**
Informed consent	X					
Demographics	X					
Height, weight, and BMI	X					
Medical history/prior medication	X					
Physical examination	X[2]					
Vital Signs	X[3]	X[3]				
Inclusion/exclusion criteria	X					
Pregnancy test [4]	X[9]	X[9]	X			
Randomization	X					
Test for SARS-CoV-2 N antibodies	X[9]			X		
Immunogenicity - Serology (Nab assay only)	X[9,10]		X[10]			
Immunogenicity - Serology (Nab assay and ELISA)[1]	X[9]	X[9]	X	X	X	
Immunogenicity – CMI Response (ELISpot and ICS)[1]	X[9]	X[9]	X	X	X	
Vaccine admin	X	X				
Immediate surveillance (30 minutes)	X	X				
Provide and collect diary and memory aid instructions[13]	X	X	X	X	X	
Oral digital thermometer and instructions on reactions[5]	X	X				
Collection of solicited local/ systemic adverse events	X	X				
Concomitant medications[6]	At any time during the study					
Collection of COVID-19 symptoms through passive and active surveillance	Passive Surveillance: Subjects will be instructed to contact the study site if they experience symptoms of COVID-19 from the day of the first vaccination (Day 0, post vaccination) until the end of the Phase 3 portion of the study.<br>Subjects will also be advised to inform the clinical site if they have tested positive for SARS-CoV-2 infection, with or without the presence of any symptoms associated with COVID-19, and will be asked to provide a documented test result.<br>Active Surveillance: Between the day of the first vaccination (Day 0, post vaccination) and when RA/A is granted for the CoVLP formulation, the subjects will be contacted once per week via the electronic					

Visit Type	Screening / Vaccination	Post-vaccination Visits/Contacts				
**Study Day**	**Day 0**	**Day 21 (± 2)** [8]	**Day 42 (± 2)** [10]	**Day 201 (± 14)**[12]	**Day 386 (± 14)**[13]	**Unscheduled Contact for Unblinding**[14]
**Visit Number**	**1**	**2**	**3**	**4**	**5/Phone**	**Phone**
	diary and asked about symptoms of COVID-19. Other methods of contact (phone, text, email) will only be used as a last resort to contact the subject.					
Testing for laboratory confirmation of SARS-CoV-2 infection	Subjects who report any symptoms associated with COVID-19 after the start of the active and passive surveillance will be requested to visit the Investigator site to provide nasal or NP swabs to test for COVID-19. During the visit, the Investigator will confirm whether nasal or NP swabs should be collected. If the subject cannot visit the Investigator site, then the subject may receive a visit at home (or hospital, if applicable) to collect the nasal/NP swabs samples or be provided with self-administered nasal swabs for sample collection. During the 7 days following each vaccination, Investigator judgement will be used to determine whether swabs should be collected from subjects reporting symptoms similar to those associated with COVID-19 since such symptoms may also be due to the reactogenicity of the study treatment administered rather than SARS-CoV-2 infection.<br>The swabs will be collected within 72 hours (preferably within 48 hours) of the reporting of a COVID-19-associated symptom. If swabs cannot be collected within 72 hours after reporting of the COVID-19-associated symptom, nasal or NP swabs should be collected at the next available opportunity for the subject to visit the Investigator site or at home (or hospital, if applicable). Two swabs per event per subject will be collected and submitted for analysis using a virologic method, at the central virology laboratory. To mitigate the risk of long delays in shipping samples to the central virology laboratory, an additional local virologic test will be performed. The purpose of this supplementary test is to provide a preliminary diagnosis and permit appropriate symptom-recording and sample collection as quickly as possible.<br>All subjects with virologically-confirmed SARS-CoV-2 infection, based on results from the locally performed testing, will be asked to provide serial (every other day) self-administered nasal swabs (2 swabs every other day for the central virology laboratory) for approximately 2 weeks. In the event, the COVID-19-associated symptoms persist for more than 2 weeks, then the Investigator's clinical judgement is required to extend the collection of swabs beyond the 2 weeks from the subject. If the COVID-19-associated symptoms have resolved in less than 2 weeks and the Investigator believes it is likely the subject has virologically recovered, then two consecutive negative results, obtained locally, are required prior to stopping the collection of the self-administered swabs for the purpose of testing at the central laboratory.<br>At the same visit where the Investigator will confirm whether nasal or NP swabs should be collected, subjects will be trained on daily recording of their COVID-19-associated symptoms using a COVID-19 diary and will be asked to immediately start recording their symptoms in the COVID-19 diary. Subjects will collect any COVID-19-associated symptoms in their COVID-19 diary until a negative swab result for the subject is received from the central virology laboratory or until the subject has no more COVID-19-associated symptoms.					
AEs, SAEs, MAAEs, and AESIs[7]	At any time during the study.					
Termination record					X	

[1] These samples will only be collected from the subjects included in the immunogenicity subset.

[2] A limited physical examination will occur at screening. History/symptom-directed physical examinations may be performed at other visits if 1) new complaints or concerns are raised by either the study subject or study staff and 2) deemed to be necessary by the Investigator.

[3] Record prior to study vaccine, and as deemed necessary.

[4] In all females of childbearing potential in Study Populations #1 and #3; it will be tested in urine at Days 0, 21, and 42.

[5] After vaccination, subjects will be instructed on the diary and memory aid provided for their use for recording reactions, AEs, and concomitant medication use.

[6] After the Day 42 visit, concomitant medication collection will be limited to those used to treat an SAE, MAAE, AE leading to withdrawal, AESIs, or an AE that occurred before Day 42; any vaccine not foreseen in the study protocol; and prohibited medications.

[7] AEs will be collected up to Day 42; SAEs, MAAEs, AEs leading to withdrawal, and AESIs will be collected through to the end of the study. Investigators should report SAEs, AESIs, and pregnancy reports to Sponsor within 24 hours of the Investigator

Visit Type	Screening / Vaccination	Post-vaccination Visits/Contacts				
Study Day	Day 0	Day 21 (± 2) [8]	Day 42 (± 2) [10]	Day 201 (± 14)[12]	Day 386 (± 14)[13]	Unscheduled Contact for Unblinding[14]
Visit Number	1	2	3	4	5/Phone	Phone

learning of the event. Specific contacts for the collection of information regarding all of these events will occur on Day 386 for SAEs, MAAEs, AEs leading to withdrawal, and AESI.

[8] All efforts will be done to have subjects returning on planned date for Day 21 activities. If for any reason the visit is done before or after this planned date, subsequent visits/contacts (Days 42, 201, and 386) will be adjusted accordingly.

[9] Blood and urine (pregnancy test) samples are to be collected prior to vaccination on the respective day.

[10] An immunogenicity-serology (Nab assay only) sample at Day 0 and Day 42 will be collected from all subjects.

[11] During the first 8 days after each vaccination (prior to RA/A), if data entry in the electronic diary is missed for 2 consecutive days by a subject then the subject will receive a follow-up contact (phone, text, or email) to remind him/her to record his/her data in the electronic diary.

[12] If RA/A occurs prior to Day 201, blood samples on Day 201 will not be collected from subjects in the placebo group who receive the CoVLP formulation post-RA/A. These subjects will follow the Table 3: Time and Events Schedule once they agree to receive the CoVLP formulation post-RA/A.

[13] If RA/A occurs prior to Day 386, blood samples on Day 386 will not be collected from subjects in the placebo group who receive the CoVLP formulation post-RA/A. These subjects will follow the Table 3: Time and Events Schedule once they agree to receive the CoVLP formulation post-RA/A.

[14] Once RA/A is obtained, subjects in the study will be contacted by phone (or any other method of communication such as the electronic diary, text, or email) and informed of the treatment they received in the study. Subjects in the placebo group will be offered the CoVLP formulation. Subjects in the placebo group who receive the CoVLP formulation (post-RA/A) will follow the Table 3: Time and Events Schedule. Subjects in the placebo group who do not want the CoVLP formulation and remain in the study will continue to follow the Table 2: Time and Events Schedule.

Table 3 Time and Events Schedule for Period 2: General Information for Placebo Subjects who will Receive the CoVLP Formulation Post-RA/A in the Phase 2 and 3 Portions and Subjects who Received a Single Dose of the CoVLP Formulation prior to RA/A

Visit Type	Vaccination		Post-vaccination Contacts	
Study Day	Post-RA/A[8]	21 Days After First Vaccination (± 2)	Monthly Calls (± 14)[5]	Day 386 (± 14)
Visit Number	1	2	Phone	Phone
Contraindications assessment[1]	X	X		
Urine Pregnancy test[2]	X	X		
Test for SARS-CoV-2 N antibodies[6]	X[7]			
Vaccine admin	X	X		
Immediate surveillance (30 minutes)	X	X		
Provide and collect memory aid instructions	X	X		X
Concomitant medications[3]	At any time during the study period			
Collection of COVID-19 symptoms through passive surveillance	Passive Surveillance: Subjects will be instructed to contact the study site if they experience symptoms of COVID-19 from the day of the first vaccination of the CoVLP formulation (post vaccination) until the end of the Phase 3 portion of the study.<br>Subjects will also be advised to inform the clinical site if they have tested positive for SARS-CoV-2 infection, with or without the presence of any symptoms associated with COVID-19, and will be asked to provide a documented test result.			
Testing for laboratory confirmation of SARS-CoV-2 infection	Subjects who report any symptoms associated with COVID-19 after the start of the active and passive surveillance will be requested to visit the Investigator site to provide nasal or NP swabs to test for COVID-19. During the visit, the Investigator will confirm whether nasal or NP swabs should be collected. If the subject cannot visit the Investigator site, then the subject may receive a visit at home (or hospital, if applicable) to collect the nasal/NP swabs samples or be provided with self-administered nasal swabs for sample collection. During the 7 days following each vaccination, Investigator judgement will be used to determine whether swabs should be collected from subjects reporting symptoms similar to those associated with COVID-19 since such symptoms may also be due to the reactogenicity of the study treatment administered rather than SARS-CoV-2 infection.<br>The swabs will be collected within 72 hours (preferably within 48 hours) of the reporting of a COVID-19-associated symptom. If swabs cannot be collected within 72 hours after reporting of the COVID-19-associated symptom, nasal or NP swabs should be collected at the next available opportunity for the subject to visit the Investigator site or at home (or hospital, if applicable). Two swabs per event per subject will be collected and submitted for analysis using a virologic method, at the central virology laboratory. To mitigate the risk of long delays in shipping samples to the central virology laboratory, an additional local virologic test will be performed. The purpose of this supplementary test is to provide a preliminary diagnosis and permit appropriate symptom-recording and sample collection as quickly as possible.<br>All subjects with virologically-confirmed SARS-CoV-2 infection, based on results from the locally performed testing, will be asked to provide serial (every other day) self-administered nasal swabs (2 swabs every other day for the central virology laboratory) for approximately 2 weeks. In the event, the COVID-19-associated symptoms persist for more than 2 weeks, then the Investigator's clinical judgement is required to extend the collection of swabs beyond the 2 weeks from the subject. If the COVID-19-associated symptoms have resolved in less than 2 weeks and the Investigator believes it is likely the subject has virologically recovered, then two consecutive negative results, obtained locally, are required prior to stopping the collection of the self-administered swabs for the purpose of testing at the central laboratory.<br>At the same visit where the Investigator will confirm whether nasal or NP swabs should be collected, subjects will be trained on daily recording of their COVID-19-associated symptoms using a COVID-19 diary and will be asked to immediately start recording their symptoms in the COVID-19 diary. Subjects will collect any COVID-19-associated symptoms in their COVID-19 diary until a negative swab result for the subject is received			

Visit Type	Vaccination		Post-vaccination Contacts	
**Study Day**	**Post-RA/A[8]**	**21 Days After First Vaccination (± 2)**	**Monthly Calls (± 14)[5]**	**Day 386 (± 14)**
**Visit Number**	**1**	**2**	**Phone**	**Phone**
	from the central virology laboratory or until the subject has no more COVID-19-associated symptoms.			
AEs, SAEs, MAAEs, and AESIs[4]	At any time during the study			
Termination record				X

[1] These visits will consist of assessing subjects for contraindications to the first and second CoVLP formulation vaccinations.

[2] In all females of childbearing potential in Study Populations #1 and #3. Urine (pregnancy test) samples are to be collected prior to vaccination on the respective day.

[3] Forty-two (42) days after the first CoVLP formulation vaccination, concomitant medication collection will be limited to those used to treat an SAE, MAAE, AE leading to withdrawal, AESIs, or an AE that occurred before 42 days post-vaccination; any vaccine not foreseen in the study protocol; and prohibited medications.

[4] AEs will be collected up to 42 days after the first CoVLP formulation vaccination; SAEs, MAAEs, AEs leading to withdrawal, and AESIs will be collected through to the end of the study. Investigators should report SAEs, AESIs, and pregnancy reports to Sponsor within 24 hours of the Investigator learning of the event. Specific contacts for the collection of information regarding all of these events will occur on Day 386 for SAEs, MAAEs, AEs leading to withdrawal, and AESI.

[5] Subjects should be reached once a month with no more than 45 days between phone contacts (use the second vaccination date as starting reference or last day of contact with the clinic site or Investigator if a second vaccination did not occur).

[6] This blood sample will only be collected from placebo recipients who are going to receive two doses of the CoVLP formulation in the Phase 3 portion; this blood sample will not be collected from subjects who received a single dose of the CoVLP formulation prior to RA/A and are now going to receive a second dose of the CoVLP formulation post-RA/A. If RA/A occurs post-Day 201, then no blood sample will be collected since a blood sample to test for SARS-CoV-2 N antibodies will be collected on Day 201.

[7] Blood sample is to be collected prior to vaccination.

[8] Subjects who received a single dose of the CoVLP formulation prior to RA/A will only proceed with the Post-RA/A visit for dosing and do not need to complete the 21 Days After First Vaccination visit. However, these subjects will proceed with the Monthly Calls and the Day 386 Telephone Contact.

Table 4 Time and Events Schedule: Onset of COVID-19

**Days after Reporting of COVID-19[3]:**	**0-3 Days**	**0-3 Days[1]**	**Monitoring[2]**
**Contact Type**	**Phone**	**Visit**	**Phone / Text / Email Messaging / Electronic Diary**
Verify information on COVID-19 and confirm swab collection within 72 hours (preferably within 48 hours) of the reporting of a COVID-19 symptom. Swab collection will be done at the discretion of the site Investigator.	X		
Remind subject to continue to record data and in a timely manner	X		
Collection of information on COVID-19 symptoms	X	X	X

[1] Nasal or NP swab collection is to be done within 72 hours after reporting of the COVID-19 symptoms (preferably within 48 hours), at the discretion of the site Investigator, and prior to the use of any antiviral treatment medication. If swab collection cannot be done within 72 hours after reporting of the COVID-19 symptoms, swabs should be collected at the next available opportunity for the subject to visit the Investigator site or at home (or hospital, if applicable) and date and time of collection recorded.
[2] After swab collection is done, the planned active surveillance (phone, text, email, electronic diary) will continue and will include questions on the progression of COVID-19.
[3] Swab collection will be done for any subject who reports any COVID-19-associated symptom after the start of the active and passive surveillance, at the discretion of the site Investigator.

ABBREVIATIONS

AE	adverse event
AESI	adverse event of special interest
ANOVA	analysis of variance
BMI	body mass index
BP	blood pressure
CBER	Center for Biologics Evaluation and Research
CI	confidence interval
CMI	cell-mediated immune (response)
CMI	cell-mediated immunity
CSR	clinical study report
eCRF	electronic case report form
EDC	electronic data capture
ELISA	enzyme-linked immunosorbent assay
ELISpot	enzyme-linked immuno spot assay
FDA	Food and Drug Administration
GCP	good clinical practice
GMFR	geometric mean fold rise or seroconversion factor
GMT	geometric mean titer
HR	heart rate
IB	Investigator's Brochure
ICF	informed consent form
ICH	International Council for Harmonisation
IDMC	Independent Data Monitoring Committee
IEC	independent ethics committee
IFN-γ	interferon gamma
IM	intramuscular
IRB	institutional review board
IRT	interactive response technology
ITT	intention-to-treat
MedDRA	Medical Dictionary for Regulatory Activities
Nab	neutralizing antibody
OR	odds ratio
OT	oral temperature
PBMC	peripheral blood mononuclear cell
pIMD	potential immune-mediated disease
PP	per protocol
RA/A	Regulatory Authorization/Approval
RR	respiratory rate

RTSM	randomization and trial supply management system
SAE	serious adverse event
SAP	statistical analysis plan
SAS	safety analysis set
SAS®	Statistical Analysis System®
SC	seroconversion
VAED	vaccine-associated enhanced disease
VLP	virus-like particle
WHO	World Health Organization
UK	United Kingdom
US	United States

1 INTRODUCTION

1.1 Background

A cluster of pneumonia cases of unknown aetiology was identified in the city of Wuhan in Hubei province of China in December 2019 [Zhu 2020]. The clinical manifestations included fever, cough, and fatigue, while other symptoms include sputum production, headache, haemoptysis, diarrhoea, dyspnoea, and lymphopenia [Lake 2020]. Clinical features revealed by a chest computed tomography (CT) scan presented as pneumonia, however, there were abnormal features such as acute respiratory distress syndrome, acute cardiac injury, and incidence of multiple organ failure that led to death in some cases [Chen 2020]. The symptoms of the disease were more severe in older age groups with comorbidities, while hypertension, type 2 diabetes, asthma and chronic obstructive pulmonary disease (COPD) were also identified as risk factors [Liu 2020a, Yang 2020]. A novel coronavirus, Severe Acute Respiratory Syndrome Coronavirus 2 (SARS-CoV-2), formerly known as the 2019 novel Coronavirus (2019-nCoV), was identified as the agent that caused the pneumonia outbreak, and the disease was subsequently named 'coronavirus disease 2019', or COVID-19 [Guan 2020, Zhu 2020]. The rapidly evolving situation with SARS-CoV-2 infection in China and spread of the disease across many countries prompted the World Health Organization (WHO) to declare a pandemic in March 2020 [WHO 2020].

Coronaviruses are frequent causes of respiratory infections where six major species are known to cause human infections besides the SARS-CoV-2. Previous outbreaks of coronavirus infections include the severe acute respiratory syndrome (SARS) and the Middle East respiratory syndrome (MERS), which have been characterized as a great public health threat [Liu 2020]. In 2002–2003, severe acute respiratory syndrome coronavirus (SARS-CoV) emerged from bat and palm civet and infected over 8 000 people and caused about 800 deaths [Cheng 2007]. In 2012, Middle East respiratory syndrome coronavirus (MERS-CoV) was discovered as the causative agent of a severe respiratory syndrome in Saudi Arabia, with nearly 2 500 confirmed cases and 858 deaths. It remains endemic in Middle East, and dromedary camel is thought to be the zoonotic reservoir host of MERS-CoV [Memish 2020]. Less virulent coronavirus species include NL63, 229E, OC43 and HKU14 that account for 10 to 30 % of common cold cases, with only occasional spreading to the lower respiratory tract [Paules 2020, Su 2016].

Coronaviruses are enveloped positive-sense RNA viruses ranging from 60 nm to 140 nm in diameter with spike-like projections on its surface giving it a crown-like appearance under the electron microscope. SARS-CoV-2 particles consist of a helical nucleocapsid structure, formed by the association between nucleocapsid (N) phosphoproteins and the viral genomic RNA, which is surrounded by a lipid bilayer where three types of structural proteins are inserted: the spike (S), the membrane (M), and the envelope (E) proteins. SARS-CoV-2 uses its S glycoprotein, a main target for the neutralizing antibody, to bind to its receptor angiotensin-converting enzyme 2 (ACE2), and mediate membrane fusion and virus genome release into the cytosol of an infected cell. Each monomer of trimeric S protein is about 180 kDa, and contains two subunits, S1 and S2, mediating attachment and membrane fusion, respectively [Chen 2020a, Ou 2020].

Significant efforts have been made to the development of vaccines and therapeutic drugs. Currently, the therapy Remdesivir (Canada and the USA) and convalescent plasma (USA) have been approved to treat COVID-19, with specific conditions for use and for specific patients. Most therapeutic approaches that are currently being tested are based on repurposing the therapeutic agents previously designed for other applications. These agents can either directly target the virus replication cycle or aim boosting the innate antiviral immune responses or attenuating the damage induced by dysregulated inflammatory responses [Ahn 2020, Stebbing 2020].

Vaccination is the most effective strategy for preventing infectious diseases since it appears to be more cost-effective than therapeutic drugs and reduces morbidity and mortality without causing long-lasting undesirable effects. Although three human coronaviruses emerged worldwide over the past two decades, causing considerable threat to global health, only a few vaccines have been approved for the prevention of COVID-19 including the Pfizer-BioNTech COVID-19 vaccine (Canada and USA), the Moderna COVID-19 vaccine (Canada and USA), the AstraZeneca COVID-19 vaccine (Canada), and the Janssen COVID-19 vaccine (Canada and USA) with specific conditions, in a particular age range. Research groups around the world are accelerating the development of COVID-19 vaccines using various approaches [Ahn 2020, Lu 2020].

A protective role of both humoral and cell-mediated immune responses against highly pathogenic coronaviruses has been suggested [Enjuanes 2016]. The SARS-CoV outbreak of 2002–2003 demonstrated that while antibody-mediated protection appeared to occur, antibody titers were found to be relatively short-lived in convalescent patients, dropping substantially by two years post-infection [Tang 2011]. In contrast, T cell responses remained detectable up to 11 years post-infection, and thus showed considerable promise for SARS-CoV vaccine development [Ng 2016]. Antibody responses against S protein, the most abundant protein of SARS-CoV-2, have been shown to protect from the infection [Du 2009, Ou 2020]. Current evidence strongly suggest that induction of a Th1 type response is important for successful control of highly pathogenic coronaviruses including SARS-CoV-2, as well as for reducing the theoretical risk of vaccine-enhanced disease [Honda-Okubo 2015]. These observations have been highly influential in the vaccine development efforts to prevent SARS-CoV-2 infection.

A large number of biopharmaceutical companies and academic laboratories are racing to develop prophylactic vaccine candidates against SARS-CoV-2 using a wide range of platforms including mRNA, DNA, inactivated virus, live viral vectors, recombinant proteins, peptides, and virus-like particles (VLPs) [Thanh Le 2020]. The vaccine candidate developed by Medicago R&D Inc. (Medicago) is a self-assembling virus-like particle (VLP) that displays trimers of recombinant S protein of SARS-CoV-2 integrated into the lipid bilayer of the nanoparticles. These VLPs are produced in a plant (*Nicotiana benthamiana*) and closely resemble the native structure of SARS-CoV-2 viruses, allowing them to be easily recognized by the immune system. In contrast to the native viruses, these plant-derived VLPs lack viral genetic material, and therefore are non-replicative and non-infections [Medicago 2020]. Based on positive results of pre-clinical testing in mice and non-human primates (Indian Rhesus macaques) and a successful Phase 1 study, Medicago plans to initiate this Phase 2/3 study.

1.1.1 Elderly Population

Although all age groups are at risk of contracting COVID-19, older persons are at a significantly higher risk of severe disease, hospitalization, intensive care unit (ICU) admissions, and death [Chen 2020b, Yang 2020a, Zhou 2020]. In the United States, population-based rates of laboratory-confirmed COVID-19–associated hospitalization increased with age: 2.5 % in those 18–49 years of age, 7.4 % in those 50–64 years of age, and 13.8 % in those 65 years of age and older, ranging from 12.2 % in those 65–74 years of age to 17.2 % in those ≥85 years of age [Garg 2020]. The ICU admission rates are lowest among adults 20–44 years of age (2 %–4 %) and highest among adults 75–84 years of age (11%–31%) [CDC 2020]. Case fatality rates are as low as 0.05 % in individuals 20–29 years of age, increasing to 1.23 % in those 50–59 years of age, 5.80 % in those 60–69 years of age, and raising dramatically to 19.93 % in persons 70–79 years of age and 34.37 % in those 80 years of age and older in Canada [Health Canada 2020]. Although advanced age is an independent risk factor for severe COVID-19 [Chen 2020, Stokes 2020], the risk of poor clinical outcomes in older adults is strongly influenced to the prevalence of underlying medical conditions in the elderly [Du 2020, Shahid 2020].

Age adversely affects a wide variety of physiological functions, including the ability to mount robust and effective immune responses, which results in increased vulnerability to infectious diseases, diminished responses to vaccination, and a susceptibility to age-related inflammatory diseases [Pinti 2016]. The effect of aging on innate immunity has been observed in dendritic cells, macrophages, neutrophils, natural killer cells, and other cell types. The key innate immune functions such as chemotaxis and phagocytosis, antigen presentation, cytotoxicity, signals transduction, and secretion of and response to cytokines were found to be altered with advanced age [Shaw 2013]. Ageing is also associated with the increased production of pro-inflammatory cytokines. The continuous production of inflammatory mediators could potentially contribute to the onset of age-associated inflammatory diseases, frailty and mortality in older individuals [Furman 2019]. The impairment of adaptive immunity with aging is associated with decreased number of circulating B cells, lower antibody production, changed of antibody specificity repertoires and of B cell dynamics, and overall weakened humoral responses in older individuals [Frasca 2011]. Reduced numbers of circulating T cell and CD4+ and CD8+ subsets, a gradual shift from naïve CD45RA+ to activated or memory CD45RO+ cells, increased proportion of senescent or exhausted T cells that are functionally dormant have been correlated with frailty and dysregulation of adaptive immune responses in the elderly [Bektas 2017].

These age-related changes of immune system result in the elderly being less likely to benefit from vaccinations against a range of infectious diseases. Vaccine-induced immune responses are frequently lower in the elderly compared to younger adults [Crooke 2019, Pinti 2016]. The lower antibody responses observed in the elderly cannot be attributed solely to defects in B cell function. Age-related changes in antigen uptake, processing and presentation, as well as functional defects of T cells, can also contribute to reduced antibody responses [Clark 2012, Sasaki 2011]. Although less well-studied, cell-mediated immunity after vaccination can also be lower in older individuals [Weinberg 2019]. Various strategies have been pursued in order to improve vaccine-elicited immune responses and vaccine effectiveness in the elderly, including

high-dose formulations and the use of immunity boosting adjuvants [Domnich 2017, Wilkinson 2017].

1.1.2 Adults with Comorbidities

People of any age with serious underlying medical conditions such as diabetes and cardiovascular or lung disease are at a greater risk of severe COVID-19. Those individuals with underlying uncontrolled medical conditions such as diabetes, hypertension, lung, liver, and kidney disease, cancer patients on chemotherapy, smokers, transplant recipients, and patients taking steroids represent a vulnerable population who are not only at a higher risk of developing severe illness but are also at an increased risk of death [Li 2020, Sanyaolu 2020]. Hypertension (odds ratio [OR]: 2.29), diabetes (OR: 2.47), chronic obstructive pulmonary disease (COPD; OR: 5.97), cardiovascular disease (OR: 2.93), and cerebrovascular disease (OR: 3.89) were independent risk factors associated with COVID-19 [Wang 2020]. A strong association of worse clinical outcomes in COVID-19 disease with obesity, even in the absence of any other comorbidity, has been reported [Korakas 2020]. The in-hospital death OR was found as high as 2.70 in patients with coronary artery disease, 2.48 in patients with congestive heart failure, 1.95 in those with cardiac arrhythmias, 2.96 in patients with COPD, and 1.79 for current smokers [Mehra 2020]. Overall, the risk of severe COVID-19 requiring admission to intensive care unit, invasive ventilation, or leading to death was found to be 1.79 fold greater among patients with at least one comorbidity and 2.59 fold greater among patients with two or more comorbidities compared to generally healthy subjects [Guan 2020a].

The need for an effective COVID-19 vaccine for people with underlying health conditions is obvious; however, vaccination strategies for these individuals may require some adjustment to achieve the desirable immune responses and vaccine effectiveness. Many comorbidities can adversely impact the response to vaccination, either due to general deterioration of the immune reactivity or as the result of pharmaceutical management of the underlying disease [Kwetkat 2020, Zimmermann 2019]. For example, patients with COPD generally respond poorly to influenza vaccination with significantly lower antibody titers and seroconversion rates: 43% in COPD compared to 90 % of healthy participants [Nath 2014]. Congestive heart failure patients develop a significantly lower T cell immune response to live-attenuated varicella-zoster vaccine and inactivated influenza vaccine than those without a cardiac impairment [Vardeny 2010, Verschoor 2017]. The effectiveness of 23-valent polysaccharide pneumococcal vaccine (PPV23) against invasive pneumococcal disease in individuals aged ≥65 years falls from 45 % in the healthy elderly to 25 % in high-risk immunocompetent patients with chronic respiratory/heart/liver/renal disease; diabetes mellitus; cerebrospinal fluid leaks; cochlear implants and is further reduced to 13 % in patients with underlying health conditions thought to be associated with an immune impairment (asplenia/splenic dysfunction, malignancy or an immunosuppressive drug treatment) [Djennad 2019].

1.1.3 Risk:Benefit in the Different Study Populations

Although the focus of this protocol is on the administration of the CoVLP formulation in the adult and elderly populations, including those with comorbidities, the intent of Medicago's long-term clinical development program is to develop a CoVLP formulation that is safe and effective for use in a much larger range of the population, including children, adolescents, and pregnant women.

As outlined above, while most people appear to be equally susceptible to SARS-COV-2 infection, the risks from COVID-19 disease are not evenly distributed across all ages and all populations. Although the role of children in chains of transmission is still a matter of some debate [Li 2020, Zimmermann 2020], it is becoming clear that any infected individual including those who as pre-symptomatic, asymptomatic and symptomatic can contribute to transmission [Furukawa 2020]. Although the emergence of SARS-COV-2 has caused massive disruption, the proportion the world's population that has been infected remains relatively small despite its expanding global circulation for the past 10-12 months [Chen 2020c]. As a result, most authorities consider rapid immunization of the entire population will be needed to achieve herd immunity in a reasonable time-frame [Randolph 2020].

Because the risks of COVID-19 fall most heavily on the elderly and those with co-morbid conditions, the benefits in vaccinating young, healthy adults and children are primarily societal and indirect (i.e. public health, enhancing herd immunity, societal re-opening) while individuals in the higher risk groups also derive a clear and direct personal health benefit. The case of pregnant women at different stages of gestation as well as women of child-bearing potential (WOCBP) not actively avoiding pregnancy introduces unique risks at the same time as providing unique benefits for both the mother and the unborn child/neonate.

At the current time, there is no reason to suspect the CoVLP formulation that will be used in the proposed study poses an elevated risk specific to any of the populations discussed above. In particular, the adjuvant (AS03) has been used across the entire age range including pregnant women and WOCBP [Cohet 2015] (see Section 1.2.1). As a result, risk:benefit calculations for each of the study populations are driven primarily by the benefit denominator.

1.2 Background of the Investigational Product

Medicago has developed a plant-based system (*Nicotiana benthamiana*) for transient expression of recombinant viral proteins, and this system has been used to produce VLPs bearing the SARS-CoV-2 S glycoprotein. Previously, Medicago used the same manufacturing platform to produce the Quadrivalent VLP Influenza Vaccine intended for active immunization for the prevention of influenza disease caused by influenza A subtype viruses and type B viruses contained in the vaccine. This plant-based platform has the following characteristics:

- The recombinant S protein is based on the genetic sequence of SARS-CoV-2 and is not subject to random mutation;
- Plant-based VLP manufacturing does not require the use of live viruses so no inactivation by chemicals or detergent-splitting is needed;

- The trimeric S proteins displayed in the surface of the VLPs are in a stabilized pre-fusion conformation that resembles the native structure of SARS-CoV-2 viruses, theoretically allowing them to be easily recognized by the immune system to induce a robust neutralizing antibody response and reduce the risk of vaccine enhanced disease [Lambert 2020].
- Very few microbial pathogens can infect both plants and humans so the risk of exposure to potentially pathogenic adventitious agents is greatly reduced;
- Medicago's previous clinical data suggests that plant-made VLP vaccines induce not only antibodies, but also strong CD4+ T cell immunity which may be important for both the persistence of immunity and the provision of better protection [Channappanavar 2014].

Nicotiana benthamiana is a non-transgenic plant that is a distant wild relative from Australia of the tobacco plant, *Nicotiana tabacum*. The transfer vector used to move the targeted S protein DNA construct into the plant cells is the bacterium *Agrobacterium tumefaciens*. This vector then directs the expression of the desired viral protein. The SARS-CoV-2 S protein is aligned at the plant plasma membrane via a transmembrane domain, and buds off the plant plasma membrane in the form of VLPs. Thus, the viral S protein trimers are anchored in a lipid bilayer of plant cell origin.

1.2.1 Background of the Adjuvant AS03

The adjuvant AS03 is an established effective adjuvant used in the formulations for Arepanrix™ and Pandemrix™ and is manufactured by GlaxoSmithKline. The AS03-adjuvanted pandemic influenza vaccines have been shown to be more immunogenic than non-adjuvanted vaccines, offering the potential of cross-clade immunity and feasibility of antigen-sparing. High efficacy and effectiveness have been demonstrated for AS03-adjuvanted H1N1 pandemic influenza vaccines in a wide range of populations [Garcon 2012]. Clinical data with AS03-adjuvanted antigen-sparing formulations have shown that immunization against influenza caused by the potential pandemic subtypes H5N1, H1N1, H7N1, H7N9, and H9N2 has demonstrated satisfactory immunogenic potency, as measured by haemagglutinin-inhibition titers, with reduced antigen doses in adults [Baz 2013, Jackson 2015, Lansbury 2017, Leroux-Roels 2007, Madan 2017, Madan 2017a, Madan 2017b, McElhaney 2013, Yang 2013, Yin 2011]. Also, AS03-adjuvanted H5N1 vaccines were shown to induce cross-clade neutralizing antibody responses [Leroux-Roels 2007] and antibody affinity maturation [Khurana 2018].

Data from clinical trials with over 55 000 vaccinated subjects showed that AS03-adjuvanted influenza vaccines exhibited an acceptable safety profile [Cohet 2019, Garcon 2012, Vaughn 2014]. Increased reactogenicity, both local and general, is consistently noted for AS03-adjuvanted vaccines compared with the corresponding unadjuvanted vaccines [Garcon 2012, Launay 2013, Nolan 2014, Waddington 2010]. Most symptoms were mild to moderate in intensity and of short duration. An increased risk of narcolepsy was observed in some individuals after the vaccination campaign with Pandemrix™ in 2009-2010. A similar risk of narcolepsy was not identified with other non-adjuvanted influenza vaccines or other AS03-adjuvanted vaccines, like Arepanrix™ [Montplaisir 2014, Cohet 2015]. Current data suggest that cases of narcolepsy

seen immediately following the 2009-2010 pandemic were most likely the result of an immune cascade, triggered by CD4 T cell cross-reactivity to HA proteins from the H1N1 virus itself and hypocretin. Research is continuing to assess whether either of the main components of the 2009/2010 flu pandemic vaccine (e.g., the viral proteins in the form used in the vaccine or the AS03 adjuvant) may have contributed to the reaction.

1.3 Pre-clinical Studies

For comprehensive preclinical information regarding the safety and toxicity of CoVLP, refer to the current Investigator’s Brochure (IB). No safety signal was identified in either the mouse or non-human primate (NHP) study. No suggestion of vaccine-enhanced disease was observed in the NHP upon challenge after either one or two doses of CoVLP alone (15 µg) or formulated with two different adjuvants (e.g. AS03, CpG 1018). Based upon the immunologic data collected to date, both adjuvants strongly enhanced antibody and T cell responses to CoVLP without evidence of a bias towards a Th2 response.

1.4 Clinical Studies

Currently, a Phase 1 clinical study (CP-PRO-CoVLP-019) testing one and two doses of CoVLP at three different dose levels (3.75 µg, 7.5 µg and 15 µg) alone and in combination with two different adjuvants (e.g. AS03, CpG1018) in 180 healthy adults is nearing completion with complete safety data to Day 42 and key Day 42 immunogenicity data available at the time of protocol preparation. Safety data collected to date do not reveal any safety concerns about either CoVLP alone or with either of the two adjuvants at any dose level. The data from this study clearly demonstrate the superiority of AS03 in the induction of anti-SARS-COV-2 antibodies at all dose levels. These data also suggest that two doses of AS03-adjuvanted CoVLP are needed to achieve consistently high antibody responses in healthy adults since robust boosting in the Day 42 antibody response after two doses of AS03-adjuvanted CoVLP was observed at all dose levels, Medicago has decided to carry the lowest dose, 3.75 µg, forward into the Phase 2/3 study.

1.5 Overall Rationale for the Study

This Phase 2/3 study is a multi-portion design to confirm that the chosen formulation and dosing regimen of CoVLP (i.e. two doses of 3.75 µg CoVLP adjuvanted with AS03 given 21 days apart) has an acceptable immunogenicity and safety profile in adults 18 years of age or older, including those with significant comorbidities (Phase 2 portion). The efficacy of this CoVLP formulation in these same Study Populations will be assessed in the Phase 3 portion.

The Phase 2 portion is a randomized, observer-blinded, placebo-controlled study to evaluate the CoVLP formulation, compared to placebo, in subjects from the three Study Populations presented in Table 5:

Table 5 Study Populations

Study Population	Age and/or Health Category	Age Range (years)
1	Healthy adults	18-64
2	Healthy elderly adults*	≥ 65
3	Adults with significant comorbidities*	≥ 18

*Subjects to be enrolled in country(ies) that has/have provided authorization to enroll these subjects in the study.

In each Study Population, 306 subjects will be randomized 5:1 to receive the CoVLP formulation:placebo. The total number of subjects in each Study Population may vary depending on when sufficient data has been accumulated to make a decision on when each Study Population can procced to the Phase 3 portion. Unless full enrollment (i.e., enrolled up to 306 subjects) has already been achieved, further enrollment into each Study Population will be closed once sufficient data has been accumulated to make the decision to proceed to Phase 3. All subjects will be followed for a period of 12 months after the last vaccination for durability of the immune responses, safety, and efficacy.

The Phase 3 portion is an event-driven, randomized, observer-blinded, placebo-controlled design that will evaluate the efficacy and safety of the CoVLP formulation, compared to placebo, in each of the Study Populations #1 - #3. Up to 30 000 subjects will be enrolled 1:1 to receive the CoVLP formulation or placebo.

The primary efficacy endpoint will be evaluated once at least 160 laboratory-confirmed COVID-19 cases (≥ 7 days post-second vaccination) have been accumulated and the median safety follow-up duration of at least 2 months (post-administration of the second vaccination) of at least 3000 subjects in each of the CoVLP formulation and placebo groups has been achieved. After the success criterion has been met for the primary efficacy endpoint of the Phase 3 portion, Regulatory Authorization/Approval (RA/A) will be pursued.

Upon obtaining the first RA/A in any country for the CoVLP formulation, Period 2 (Table 3) may begin to support the following:

1. Subjects in the placebo group will be offered the CoVLP formulation and will only be followed passively to identify symptomatic COVID-19 cases and for safety until the end of the study. A blood sample will be obtained immediately prior to the administration of the first dose of the CoVLP formulation but no further blood samples will be collected in this group. Placebo recipients who choose to receive an approved or authorized COVID-19 vaccine for which they are eligible instead of the CoVLP formulation will be withdrawn from the study;
2. Subjects in the CoVLP formulation group will complete the study as planned with a blood draw at Day 201 to assess protection against serologically-confirmed COVID-19, unless they decide to receive an approved or authorized COVID-19 vaccine for which they are eligible and thus will be withdrawn from the study.

Subjects in the placebo group who do not want to be administered the CoVLP formulation and still want to remain in the study will continue the study as planned with a blood draw at Day 201.

Upon obtaining the RA/A, the CoVLP formulation will be offered to placebo subjects in the Phase 2 portion of the study. Once the placebo subject receives the CoVLP formulation, the subject will only be followed for safety until the end of the study and no further blood draws will be collected. Subjects in the placebo group who do not wish to receive the CoVLP formulation or subjects who are in the CoVLP formulation group will continue the study as planned.

Subjects in the CoVLP formulation group will be followed for efficacy (i.e. active and passive surveillance) and safety until RA/A and then for COVID-19 cases (i.e. passive surveillance only) and safety until the end of the study (Day 386). Subjects in the placebo group will be followed for efficacy (i.e. active and passive surveillance) and safety up until RA/A and followed for safety and COVID-19 cases (i.e. passive surveillance only) until the end of the study. The sero-status of all subjects will be assessed at the time of enrollment. Serum samples will be obtained from all subjects at Day 42 so that a possible serologic correlate of protection can be evaluated once the primary efficacy outcome has been met and at later timepoints in the study. Only the subjects who initially receive the CoVLP formulation or placebo subjects who do not want the CoVLP formulation among the 288 subjects (96 subjects from each Study Population) in the immunogenicity subset will be assessed for the persistence of the vaccine-induced cellular immune responses until Day 386.

2 OBJECTIVES

2.1 Primary Objectives

The primary objectives of the Phase 2 portion of the study are:

Safety:

- To assess the safety and tolerability of the CoVLP formulation, compared to placebo in each of the Study Populations, up to 21 days after the second vaccination;

Immunogenicity:

- To assess the immunogenicity of the CoVLP formulation, compared to placebo, up to 21 days after the second vaccination in each of the Study Populations, as determined by:
  - Neutralizing antibody titers induced by the vaccine against the SARS-CoV-2 virus;
  - IFN-γ ELISpot response induced by the vaccine against the SARS-CoV-2 virus to assess the Th1 type response.

The primary objective of the Phase 3 portion of the study is:

Efficacy:

- To evaluate the efficacy of the CoVLP formulation, compared to placebo, in the prevention of laboratory-confirmed symptomatic SARS-CoV-2 infection (virologic method) starting 7 days after the second vaccination at the time of the primary vaccine efficacy (PVE) analysis (i.e., once at least 160 COVID-19 cases [≥ 7 days post-second vaccination] have been collected and the median safety follow-up duration of at least 2 months (post-

administration of the second vaccination) of at least 3000 subjects in each of the CoVLP formulation and placebo groups has been achieved).

2.2 Secondary Objectives

The secondary objectives of the Phase 2 portion of the study are:

Immunogenicity:

- To assess the relative neutralizing antibody response between the healthy adults (Study Population #1) and the healthy elderly populations (Study Population #2; each age strata) at 21 days after the second vaccination;
- To assess the relative neutralizing antibody response between the healthy adults (Study Population #1) and the healthy elderly adults (Study Population #2) combined and the adults with significant comorbidities (Study Population #3) at 21 days after the second vaccination;
- To assess the immunogenicity of the CoVLP formulation, compared to placebo, when applicable, in each of the Study Populations, as determined by the:
  - Persistence of neutralizing antibody titers induced by the vaccine against the SARS-CoV-2 virus at Days 128, 201, and Day 386;
  - IgG (ELISA) titers induced by the vaccine against the SARS-CoV-2 virus up to 21 days after the second vaccination and persistence of these antibodies at Day 128, Day 201, and Day 386;
  - The ratio of neutralizing antibody titers:IgG (ELISA) antibody titers at Day 21, Day 42, Day 128, Day 201, and Day 386;
  - IFN-γ ELISpot response induced by the vaccine against the SARS-CoV-2 virus at Day 201 and Day 386;
  - IL-4 ELISpot response induced by the vaccine against the SARS-CoV-2 virus up to 21 days after the second vaccination and at Day 201 and Day 386;

Safety:

- To assess the relative incidence of solicited local and systemic AEs up to 7 days after the first and second vaccinations between the healthy adults (Study Population #1) and each healthy elderly population (Study Population #2) in the CoVLP formulation group prior to RA/A only;
- To assess the relative incidence of solicited local and systemic AEs up to 7 days after the first and second vaccinations between the healthy adults (Study Population #1) and the healthy elderly adults (Study Population #2) combined and the adults with significant comorbidities (Study Population #3) in the CoVLP formulation group prior to RA/A only;
- To assess safety and tolerability of the CoVLP formulation, compared to placebo in each of the Study Populations, as determined by the occurrence(s) of:

- MAAEs, SAEs, AEs leading to withdrawal, AESIs (including VAED), and deaths from 22 days after the second vaccination up to when RA/A is granted;

Efficacy:

- To evaluate the efficacy of the CoVLP formulation, compared to placebo, in the prevention of laboratory-confirmed symptomatic SARS-CoV-2 infection (virologic method) starting 7 days after the second vaccination;
- To evaluate the efficacy of the CoVLP formulation, compared to placebo, in the prevention of severe COVID-19 disease starting 7 days after the second vaccination. Severe COVID-19 disease will be defined as virologically-confirmed SARS-CoV-2 infection with the occurrence of a new onset of any of the following:
  - Clinical signs at rest indicative of severe systemic illness (respiratory rate ≥ 30 per minute, heart rate ≥ 125 per minute, SpO2 ≤ 93% on room air at sea level or PaO2/FiO2 < 300 mmHg);
  - Respiratory failure (defined as needing high-flow oxygen, non-invasive ventilation, mechanical ventilation or ECMO);
  - Evidence of shock (SBP < 90 mmHg, DBP < 60 mmHg, or requiring vasopressors);
  - Significant acute renal, hepatic, or neurologic dysfunction;
  - Hospitalization;
  - Death.

The secondary objectives of the Phase 3 portion of the study are:

Efficacy:

- To evaluate the efficacy of the CoVLP in the prevention of laboratory-confirmed asymptomatic SARS-CoV-2 infection at Day 201 in subjects who were seronegative at baseline (confirmed by serologic method);
- To evaluate the efficacy of the CoVLP formulation, compared to placebo, in the prevention of severe COVID-19 disease starting 7 days after the second vaccination at the time of the PVE analysis (see definition for severe COVID-19 disease above);
- To assess COVID-19-related symptoms in virologically-confirmed cases and the severity of these symptoms up until resolution in subjects administered the CoVLP formulation compared to subjects administered the placebo at the time of the PVE analysis;
- To evaluate the efficacy of the CoVLP formulation, compared to placebo, in the prevention of laboratory-confirmed symptomatic SARS-CoV-2 infection (virologic method) starting after the first vaccination at the time of the PVE analysis;
- To evaluate the efficacy of the CoVLP formulation, compared to placebo, in the prevention of laboratory-confirmed symptomatic SARS-CoV-2 infection (virologic method) after the first vaccination and prior to the second vaccination at the time of the PVE analysis;
- To evaluate the efficacy of the CoVLP formulation, compared to placebo, in the prevention of laboratory-confirmed symptomatic SARS-CoV-2 infection (virologic method) after the

second vaccination and prior to 7 days post-second vaccination at the time of the PVE analysis;

- To assess the duration and intensity of viral shedding after virologically-confirmed SARS-COV-2 infection in subjects administered the CoVLP formulation compared to subjects administered the placebo at the time of the PVE analysis;
- To evaluate the efficacy of the CoVLP formulation, compared to placebo, in the prevention of laboratory-confirmed symptomatic SARS-CoV-2 infection (virologic method) by strain starting 7 days after the second vaccination at the time of the PVE analysis;

Immunogenicity:

- To assess the immunogenicity of the CoVLP formulation, compared to placebo when applicable, in the immunogenicity subset, as determined by:
  - Nab titers induced by the vaccine against the SARS-CoV-2 virus at Day 0 (prior to vaccination; all subjects), Day 21, Day 42 (all subjects), Day 201, and Day 386;
  - IgG (ELISA) titers induced by the vaccine against the SARS-CoV-2 virus up to 21 days after the second vaccination and at Day 201, and Day 386;
  - The ratio of Nab titers:IgG (ELISA) antibody titers at Day 21, Day 42, Day 201, and Day 386;
  - IFN-γ ELISpot response induced by the vaccine against the SARS-CoV-2 virus up to 21 days after the second vaccination and at Day 201 and Day 386;
  - IL-4 ELISpot response induced by the vaccine against the SARS-CoV-2 virus up to 21 days after the second vaccination and at Day 201 and Day 386;

Safety:

- To assess the safety and tolerability of the CoVLP formulation up to the end of the study.

2.3 Exploratory Objectives

The exploratory objectives of the Phase 2 portion of the study are:

Efficacy:

- To assess COVID-19-related symptoms in subjects with virologically-confirmed disease and the severity of these symptoms, up until resolution in subjects administered the CoVLP formulation compared to subjects administered the placebo;

Immunogenicity:

- To further characterize the immune response of the CoVLP formulation, if it is likely to be informative;

Safety:

- To further characterize the safety profile of the CoVLP formulation, if it is likely to be informative.

The exploratory objectives of the Phase 3 portion of the study are:

Efficacy:

- To evaluate the efficacy of the CoVLP formulation, compared to placebo, in the prevention of severe COVID-19 disease by strain starting 7 days after the second vaccination at the time of the PVE analysis (see definition of severe COVID-19 disease above);

Immunogenicity:

- To assess the immunogenicity of the CoVLP formulation, compared to placebo when applicable, in the immunogenicity subset, as determined by:
  - Specific CMI response induced by the vaccine against the SARS-CoV-2 virus up to 21 days after the second vaccination and at Day 201 and Day 386, as measured by the percentage of CD4+ T cells expressing functional markers;
- To further characterize the immune response of the CoVLP formulation, if it is likely to be informative;

Safety:

- To further characterize the safety profile of the CoVLP formulation, if it is likely to be informative.

3 STUDY ADMINISTRATIVE STRUCTURE

Table 6 Study Administrative Structure

Role	Name and Address
**Sponsor**	Medicago R&D Inc.<br>1020, route de l'Église, bureau 600,<br>Sainte-Foy (Qc) Canada G1V 3V9
Medical Officer:	[Redacted]
Medical Monitor:	[Redacted]
Senior Director, Clinical Research:	[Redacted]
Medical Writer	[Redacted]

4 STUDY DESIGN AND RATIONALE

4.1 Overview of Study Design

The Phase 2 portion is a randomized, observer-blinded, placebo-controlled study with male and female subjects. Subjects will be enrolled from three Study Populations (refer to Table 5).

In each of the three Study Populations, 306 subjects will be randomized 5:1 and will receive the same CoVLP formulation or placebo. The total number of subjects in each Study Population may vary depending on when sufficient data has been accumulated to make a decision on when each Study Population can procced to the Phase 3 portion. Unless full enrollment (i.e., enrolled up to 306 subjects) has already been achieved, further enrollment into each Study Population will be closed once sufficient data has been accumulated to make the decision to proceed to Phase 3. Subjects will receive two IM injections 21 days apart on Days 0 and 21, into the deltoid region of the alternating (if possible) arm (i.e., each arm will be injected once), of one of the following:

- CoVLP formulation;
- Placebo.

All subjects will begin enrollment at the same time (refer to Figure 1). To the greatest extent possible given local conditions (i.e. COVID-19 vaccine roll-out), enrollment of elderly adult subjects (Study Population #2) will be stratified by age in an approximately 2:1 ratio of 65 to 74 years of age and 75 years of age and older, respectively. Enrollment of subjects in Study Population #3 will include adults with co-existing conditions such as, but not limited to, obesity or being overweight, documented hypertension, cardiovascular diseases, hyperlipidemia, chronic kidney disease or related disorders, chronic respiratory disease, pre-diabetes and diabetes (type 1 and type 2), and treatment-controlled HIV.

The IDMC will review unblinded safety data (i.e. solicited and unsolicited safety data including laboratory safety data collected up to 7 days after each vaccination; refer to Section 19.7) for the first 10 sentinel subjects after each dose in all Study Populations, with the exception of Study Population #1 prior to vaccinating the remaining subjects in each Study Population according to the details outlined in the IDMC Charter. All sentinel subjects will be dosed at least 30 minutes apart.

Subjects will be screened up to 14 days in advance of the first vaccine administration and will demonstrate a satisfactory baseline medical assessment by history, general physical examination, haematological, biochemical, and serological analysis. Although a test for SARS-CoV-2 antibodies will be performed at screening, both seronegative and seropositive subjects will be enrolled. On Day 0 and Day 21, vaccine administration will occur. Phone contacts will be made one day and eight days after each vaccine administration, specifically for review of the subject's safety and concomitant medication data. Visits to the Investigator site will occur 3 days after each vaccine administration for key safety assessments, and 21 days after each vaccine administration for key safety and immunogenicity assessments. Subjects will return to the Investigator site on Day 128, Day 201, and Day 386 (3-month, 6-month, and 12-month safety follow-ups and immunogenicity assessments after the last vaccine administration).

Active and passive surveillance will be conducted in the study from the day of the first vaccination (Day 0, post vaccination) until 386 days. For passive surveillance, subjects will be instructed to report symptoms meeting the definition of new COVID-19 disease. For active surveillance, subjects will be contacted once per week via phone, text, or email and asked about symptoms of COVID-19 disease.

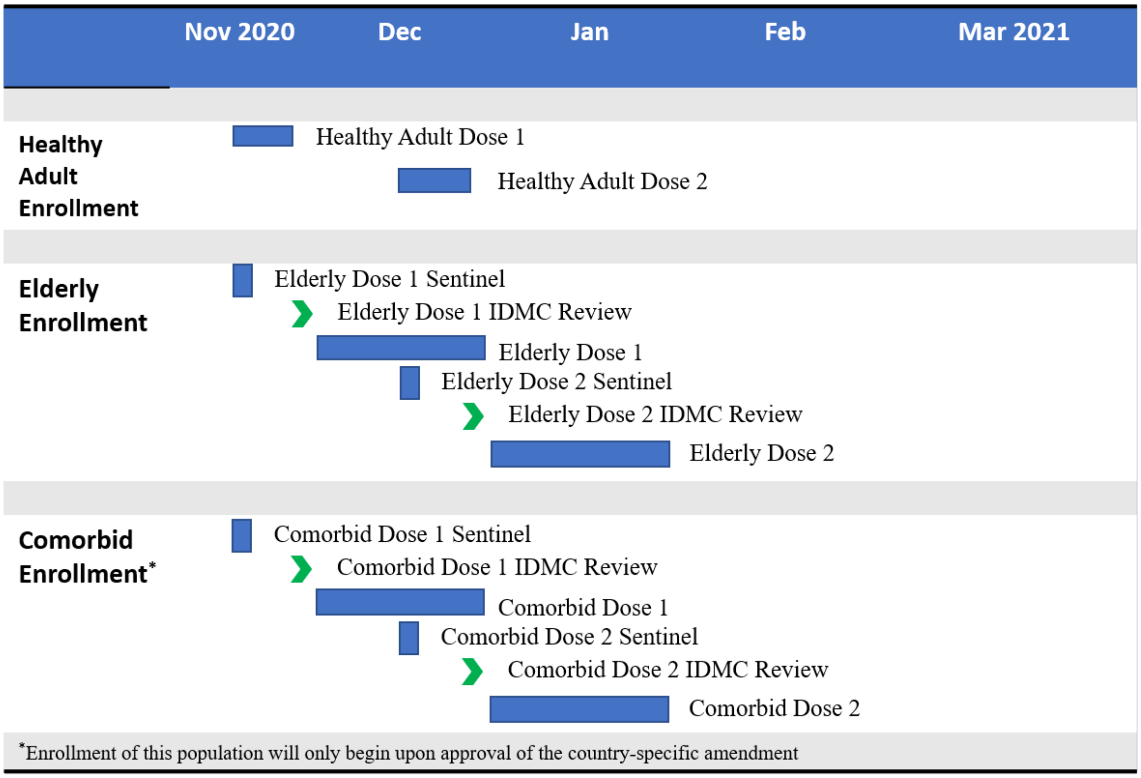

Figure 1 Study Enrollment Flow for Phase 2 Portion

The Phase 3 portion is a randomized, observer-blinded, placebo-controlled, efficacy, safety, immunogenicity (in a subset of subjects), multicenter design with male and female subjects. Similar to the Phase 2 portion, subjects will be enrolled from three Study Populations (refer to Table 5). Study Population #2 will be stratified by age in the same manner to the greatest extent possible given local conditions (i.e. COVID-19 vaccine roll-out). Enrollment of subjects in Study Population #3 will include adults with co-existing conditions such as, but not limited to, obesity (moderate or greater), cardiovascular diseases, hyperlipidemia, chronic kidney disease or related disorders, chronic respiratory disease, pre-diabetes and diabetes (type 1 and type 2), and treatment-controlled HIV.

The overall goal of the Phase 3 portion of this study is to enroll approximately 30 000 subjects who are generally representative of the overall target population in terms of racial diversity with some degree of enrichment for those at highest risk for serious COVID-19 disease (i.e. the elderly and adults with significant comorbidities).

Transition of each of the Study Populations from the Phase 2 to the Phase 3 portion of the study will be determined by the safety and immunogenicity profile unique to that Study Population in the Phase 2 portion (refer to Section 12.4 for details).

In each Study Population, subjects will be randomized to receive either the CoVLP formulation or the placebo. Subjects in the placebo group will be offered the CoVLP formulation once RA/A is obtained in any country.

Subjects will be screened and will demonstrate a satisfactory baseline medical assessment by history and general physical examination prior to the first vaccine administration. On Day 0 and Day 21, vaccine administration will occur. Subjects will be provided with a diary and memory aid to collect safety and concomitant medication data. A visit to the Investigator site will occur on Day 21 (immunogenicity subset only) and Day 42 (all subjects) for immunogenicity assessments. Subjects who initially receive the CoVLP formulation or subjects in the placebo group who do not want the CoVLP formulation will return to the Investigator site on Day 201, and Day 386 (6-month and 12-month safety follow-ups and immunogenicity assessments [immunogenicity subset only] after the last vaccine administration).

Active surveillance will be conducted in the study from the day of the first vaccination (Day 0, post-vaccination) until RA/A and passive surveillance will be conducted in the study from the day of the first vaccination (Day 0, post vaccination) until the end of the study. For passive surveillance, subjects will be instructed to report symptoms meeting the definition of new COVID-19 disease. For active surveillance, subjects will be contacted once per week via the electronic diary and asked about symptoms of COVID-19 disease. Other methods of contact (phone, text, email) will only be used as a last resort to contact the subject. In addition, to monitor asymptomatic infection, subjects who initially receive the CoVLP formulation or subjects in the placebo group who do not want the CoVLP formulation will perform a test for SARS-CoV-2 N antibodies on Day 0 and Day 201. Subjects in the placebo group who receive the CoVLP formulation post-RA/A will perform the same test on Day 0 and just prior to receiving the first dose of the CoVLP formulation.

Once subjects in the placebo group receive the CoVLP formulation (post-RA/A), they will no longer be followed for efficacy (no more active surveillance) or immunogenicity (those included in the immunogenicity subset). Passive surveillance for possible COVID-19 cases will continue however. Subjects will provide a blood draw immediately prior to receiving the first dose of the CoVLP formulation and then will be followed for safety with monthly phone contacts until Day 386, when the final study telephone contact will occur.

4.2 Rationale of Study Design

4.2.1 Randomization

Randomization will be used to minimize bias in the assignment of subjects to treatment groups, within each Study Population, to increase the likelihood that known and unknown subject attributes (e.g. demographic and baseline characteristics) are evenly balanced across treatment groups and to enhance the validity of statistical comparisons across treatment groups within each Study Population.

4.2.2 Blinding

Both portions of the study will be observer-blind to reduce potential bias during data collection and evaluation of the study endpoints. However, after the PVE analysis has been completed, the Phase 2 and Phase 3 portions will be unblinded in order to offer subjects in the placebo group the

CoVLP formulation or free them from their study obligations to receive a deployed vaccine once RA/A is obtained in any country and Period 2 (Table 3) is initiated. Details of who will remain blinded during the Phase 2 and Phase 3 portions of the study are presented in Section 6.2.

Selected individuals involved in the IDMC reviews and/or interim data analyses will be unblinded during the Phase 2 and Phase 3 portions of the study, with details presented in Section 6.2.

4.2.3 Dose Selection and Dosage Regimen

In both the Phase 2 and Phase 3 portions, a single dose level of the CoVLP formulation (i.e. 3.75 µg CoVLP adjuvanted with AS03) will be tested in a two-dose regimen on Day 0 and Day 21.

4.2.4 Route of Administration

The route of administration used for the CoVLP formulation is the IM route, specifically in the deltoid muscle of the arm, since it reliable route of administration with good absorption compared to the subcutaneous route. The IM route was used in the Phase 1 study.

4.2.5 Study Duration

The planned duration of the Phase 2 portion is approximately 14 to 16 months, from screening procedures up to the end of the Phase 2 portion (Day 386).

The planned duration of the Phase 3 portion is approximately 13 months, from screening procedures up to the end of the Phase 3 portion (Day 386).

5 STUDY POPULATION

5.1 Inclusion Criteria

Subjects must meet all of the following inclusion criteria at the Screening (Visit 1) and/or Vaccination (Visit 2) visits (these two visits are combined into one visit in the Phase 3 portion [Visit 1]) to be eligible for participation in this study; no protocol waivers are allowed. All Investigator assessment-based judgements must be carefully and fully documented in the source documents:

1. Subjects must have read, understood, and signed the informed consent form (ICF) prior to participating in the study; subjects must also complete study-related procedures and the subjects must communicate with the study staff at visits and by phone during the study;
2. At the Screening visit (Visit 1), male and female subjects must be:
   - Study Populations #1: 18 to 64 (has not yet had his/her 65th birthday) years of age, inclusive;
   - Study Population #2: 65 years of age or older;
   - Study Population #3: 18 years of age or older;

3. At Screening (Visit 1) and Vaccination (Visit 2), subject must have a body mass index (BMI) of:

   For the Phase 2 portion of the study only:
   - Study Populations #1 and #2: ≥ 18.5 and < 30 kg/m^2;
   - Study Population #3: no restrictions for BMI;

   For the Phase 3 portion of the study only:
   - Study Populations #1 and #2: ≥ 18.5 and < 35 kg/m^2;
   - Study Population #3: no restrictions for BMI;
4. Subjects are considered by the Investigator to be reliable and likely to cooperate with the assessment procedures and be available for the duration of the study;
5. Study Population #1: Subjects must be in good general health prior to study participation, with no clinically relevant abnormalities that could jeopardize subject safety or interfere with study assessments, as determined by medical history, physical examination, and vital signs. Investigator discretion will be permitted with this inclusion criterion;

   All regions except Canada: Note: Subjects with a pre-existing chronic disease will be allowed to participate if the disease is stable and, according to the Investigator's judgment that must be documented in the source documents, the condition is unlikely to confound the results of the study or pose additional risk to the subject by participating in the study. Stable disease is generally defined as no new onset or exacerbation of pre-existing chronic disease three months prior to vaccination. Based on the Investigator's judgment, a subject with more recent stabilization of a disease could also be eligible.
6. Study Populations #1 and #3: Female subjects of childbearing potential must have a negative serum pregnancy test result at Screening (Visit 1 for the Phase 2 portion) and/or a negative urine pregnancy test result at Vaccination (Visit 2 for the Phase 2 portion; Visit 1 for the Phase 3 portion):

   Non-childbearing females are defined as:
   - Surgically-sterile (defined as bilateral tubal ligation, hysterectomy or bilateral oophorectomy performed more than one month prior to the first study vaccination); or
   - Post-menopausal (absence of menses for 12 consecutive months and age consistent with natural cessation of ovulation);
7. Study Populations #1 and #3: Female subjects of childbearing potential must use an effective method of contraception for one month prior to vaccination (Visit 2 for the Phase 2 portion and Visit 1 for the Phase 3 portion) and agree to continue employing highly effective birth control measures for at least one month after the last study vaccination (or in the case of early termination, she must not plan to become pregnant for at least one month after her last study vaccination).

   The following relationship or methods of contraception are considered to be highly effective:
   - Combined (estrogen and progestogen containing) hormonal contraception associated with inhibition of ovulation:
     - Oral;

- Intravaginal;
- Transdermal;

- Progestogen-only hormonal contraception associated with inhibition of ovulation:
  - Oral;
  - Injectable;
  - Implantable;
- Intra-uterine device with or without hormonal release;
- Credible self-reported history of heterosexual vaginal intercourse abstinence prior to and for at least one month after the last study vaccination. Abstinent subjects who are ovulating should be asked what method(s) they would use should their circumstances change, and subjects without a well-defined plan should be excluded;
- Female partner;
- All regions except the US: Vasectomised partner, provided that this partner is the sole sexual partner of the study participant and that the vasectomised partner has received a medical assessment of the surgical success;
- Bilateral tubal occlusion.

8. Study Population #2: Subjects must be non-institutionalized (e.g. not living in rehabilitation centres or old-age homes; living in an elderly community is acceptable) and have no acute or evolving medical problems prior to study participation and no clinically relevant abnormalities that could jeopardize subject safety or interfere with study assessments, as assessed by the Principal Investigator or sub-Investigator (thereafter referred as Investigator) and determined by medical history, physical examination, serology (only for the Phase 2 portion), clinical chemistry and haematology tests (only for the Phase 2 portion), urinalysis (only for the Phase 2 portion), and vital signs. Investigator discretion will be permitted with this inclusion criterion.

   All regions except Canada: Note: Subjects with a pre-existing chronic disease will be allowed to participate if the disease is stable and, according to the Investigator's judgment that must be documented in the source documents, the condition is unlikely to confound the results of the study or pose additional risk to the subject by participating in the study. Stable disease is generally defined as no new onset or exacerbation of pre-existing chronic disease three months prior to vaccination. Based on the Investigator's judgment and documented in source documentation, a subject with more recent stabilization of a disease could also be eligible;
9. Study Population #3: Subjects must have one or more co-morbid conditions that puts them at higher risk for severe COVID-19 disease. These comorbidities include but are not limited to obesity (moderate or greater), hypertension, type 1 or type 2 diabetes, chronic obstructive pulmonary disease (COPD), cardiovascular diseases, chronic kidney diseases, or be immunocompromised persons (e.g., treatment-controlled HIV infection, organ transplant recipients, or patients receiving cancer chemotherapy). Investigator discretion will be permitted with this inclusion criterion.

5.2 Exclusion Criteria

Subjects who meet any of the following criteria at the Screening (Visit 1) and/or Vaccination (Visit 2) visits (these two visits are combined into one visit in the Phase 3 portion [Visit 1]) will not be eligible for participation in this study; no protocol waivers are allowed. All Investigator assessment-based judgements must be thoroughly documented in the source documents:

1. Study Populations #1 and #2: According to the Investigator's opinion, significant acute or chronic, uncontrolled medical or neuropsychiatric illness.

   Acute disease is defined as presence of any moderate or severe acute illness with or without a fever within 48 hours prior to the Screening (Visit 1) and/or Vaccination visit (Visit 2). 'Uncontrolled' is defined as:

   - Requiring a new medical or surgical treatment during the three months prior to study vaccine administration;
   - Requiring any significant change in a chronic medication (i.e. drug, dose, frequency) during the three months prior to study vaccine administration due to uncontrolled symptoms or drug toxicity unless the innocuous nature of the medication change meets the criteria outlined in inclusion criterion no. 5 (Study Population #1) or no. 8 (Study Population #2) and is appropriately justified and documented by the Investigator.

   Investigator discretion is permitted with this exclusion criterion and must be carefully and fully documented in the source documents;
2. Study Populations #1 and #2: Any chronic medical condition associated with elevated risk of severe outcomes of COVID-19, including obesity, diabetes (type 1 or type 2), significant cardiovascular or respiratory diseases including asthma, chronic renal failure, disorders of bleeding/coagulation, chronic inflammatory or autoimmune conditions, immunosuppressive conditions (including HIV), and hypertension;
3. Study Populations #1 and #2: Any confirmed or suspected current immunosuppressive condition or immunodeficiency, including cancer, human immunodeficiency virus (HIV), hepatitis B or C infection (subjects with a history of cured hepatitis B or C infection without any signs of immunodeficiency at present time are allowed). Investigator discretion is permitted with this exclusion criterion;
4. Study Populations #1 and #2: Current autoimmune disease (such as rheumatoid arthritis, systemic lupus erythematosus or multiple sclerosis). Investigator discretion is permitted with this exclusion criterion, and subjects may be eligible to participate with appropriate written justification in the source document (i.e. subjects with a history of autoimmune disease who are disease-free without treatment for three years or more, or on stable thyroid replacement therapy, mild psoriasis [i.e. a small number of minor plaques requiring no systemic treatment], etc.);
5. Study Populations #1 and #2: Administration of any medication or treatment that may alter the vaccine immune responses, such as:
   - Systemic glucocorticoids at a dose exceeding 10 mg of prednisone (or equivalent) per day for more than seven consecutive days or for 10 or more days in total, within one month prior to the Vaccination visit (Visit 2 for the Phase 2 portion and Visit 1 for the

Phase 3 portion). Inhaled, nasal, ophthalmic, dermatological, and other topical glucocorticoids are permitted;

- Cytotoxic, antineoplastic, or immunosuppressant drugs - within 36 months prior to Vaccination (Visit 2 for the Phase 2 portion and Visit 1 for the Phase 3 portion);
- Any immunoglobulin preparations or blood products, blood transfusion – within 6 months prior to Vaccination (Visit 2 for the Phase 2 portion and Visit 1 for the Phase 3 portion);

6. Study Population #3: Acute disease defined as presence of any moderate or severe acute illness with or without a fever within 48 hours prior to the Screening (Visit 1) and/or Vaccination visit (Visit 2);
7. Administration of any vaccine within 14 days prior to Vaccination (Visit 2 for the Phase 2 portion and Visit 1 for the Phase 3 portion); planned administration of any vaccine during the study (up to Day 28 of the study). Immunization on an emergency basis during the study will be evaluated on case-by-case basis by the Investigator;
8. Administration of any other SARS-CoV-2 / COVID-19, or other experimental coronavirus vaccine at any time prior to or during the study;
9. History of virologically-confirmed COVID-19;
10. Use of any investigational or non-registered product within 30 days or 5 half-lives, whichever is longer, prior to Vaccination (Visit 2 for the Phase 2 portion and Visit 1 for the Phase 3 portion) or planned use during the study period. Subjects who are in a prolonged post-administration observation period of another investigational or marketed drug clinical study, for which there is no ongoing exposure to the investigational or marketed product and all scheduled on-site visits are completed, will be allowed to take part in this study, if all other eligibility criteria are met;
11. Have a rash, dermatological condition, tattoos, muscle mass, or any other abnormalities at injection site that may interfere with injection site reaction rating. Investigator discretion will be permitted with this exclusion criterion;
12. Use of any prescription antiviral drugs with the intention of COVID-19 prophylaxis, including those that are thought to be effective for prevention of COVID-19 but have not been licensed for this indication, within one month prior to Vaccination (Visit 2 for the Phase 2 portion and Visit 1 for the Phase 3 portion);
13. For the Phase 2 portion of the study only: Use of prophylactic medications (e.g. antihistamines [H1 receptor antagonists], nonsteroidal anti-inflammatory drugs [NSAIDs], systemic and topical glucocorticoids, non-opioid and opioid analgesics) within 24 hours prior to the Vaccination (Visit 2) to prevent or pre-empt symptoms due to vaccination;
14. History of a serious allergic response to any of the constituents of CoVLP including AS03;
15. History of a documented anaphylactic reactions to plants or plant components (including tobacco, fruits and nuts);
16. Personal or family (first-degree relatives) history of narcolepsy;
17. Subjects with a history of Guillain-Barré Syndrome;
18. Study Populations #1 and #3: Any female subject who has a positive or doubtful pregnancy test result prior to vaccination or who is lactating;

19. Subjects identified as an Investigator or employee of the Investigator or clinical site with direct involvement in the proposed study, or identified as an immediate family member (i.e. parent, spouse, natural or adopted child) of the Investigator or employee with direct involvement in the proposed study, or any employees of Medicago.

5.3 Prior and Concomitant Therapy

New or changed medications reported by the subject after the first vaccination and through to the end of the study will be recorded in the source documents as a concomitant medication as per the conditions outlined in the next paragraph. Since AEs may be secondary to new medications, the Investigator will explore the reasons for the change or for the new medication intake and document these AEs, if any.

Concomitant medications must be reported (reason for use, dates of administration, dosage, and route) if the use meets the following conditions:

- Within 30 days preceding vaccination: any treatments and/or medications specifically contraindicated (e.g. vaccines, any immunoglobulins or other blood products, or any immune modifying drugs, etc.);
- From randomization to Day 42 (Phase 2 portion and Phase 3 portion), inclusive: any medication (including, but not limited to, over-the-counter medicines such as aspirin or antacids), vitamins, and mineral supplements;
- From Day 43 to the end of the study (Phase 2 portion or Phase 3 portion), inclusive: any concomitant medication(s) administered to treat an AESI, MAAE, SAE, or AE leading to withdrawal; any concomitant medication used to treat an AE that occurred before Day 42 and that is still being used afterwards (i.e. on-going use);
- Any concomitant medication used to treat COVID-19 symptoms;
- Any concomitant medication used to treat conditions reported as medical history;
- Any investigational medication or vaccine; any vaccine not foreseen in the study protocol.

5.4 Prohibited Therapy

The following medications or therapies are prohibited during the conduct of this study:

1. Administration of any vaccine (other than the study vaccine) up to Day 28 of the study (Phase 2 portion or Phase 3 portion) as well as administration of any investigational or approved coronavirus vaccine (other than the study vaccine) up to end of the study. Immunization on an emergency basis during the study will be evaluated on case-by-case basis by the Investigator;
2. Use of any investigational or non-registered product during the study period. Subjects may not participate in any other investigational or marketed drug study while participating in this study until after the study;
3. Study Populations #1 and #2: Administration of any medication or treatment that may alter the vaccine immune responses, such as:
    - Systemic glucocorticoids;

- Cytotoxic, antineoplastic, or immunosuppressant drugs;
- Any immunoglobulin preparations or blood products, or blood transfusion.

Administration of such medications should be specifically avoided up to Day 42 of the study (Phase 2 portion or Phase 3 portion). Use of any medication or treatment that may alter the vaccine immune responses due to a medical need during the study will be evaluated on case-by-case basis by the Investigator;

4. In the Phase 2 portion of the study, use of prophylactic medications (e.g. antihistamines [H1 receptor antagonists], nonsteroidal anti-inflammatory drugs [NSAIDs], systemic and topical glucocorticoids, non-opioid and opioid analgesics) within 24 hours prior to administration of the second dose of the vaccine to prevent or pre-empt symptoms due to vaccination
5. Use of any prescription antiviral drugs with the intention of COVID-19 prophylaxis, including those that are thought to be effective for prevention of COVID-19 but have not been licensed for this indication, during the study.

Given that an important objective of this study is to evaluate the tolerability of the study vaccine, the use of prophylactic medications to prevent or pre-empt symptoms due to vaccination is specifically prohibited up to seven days after each vaccination in the Phase 2 portion of the study (end of collection of solicited symptoms). A prophylactic medication is a medication administered in the absence of ANY symptom and in anticipation of a reaction to the vaccination (e.g. an antipyretic is considered to be prophylactic when it is given in the absence of fever or any other symptoms, to prevent fever from occurring, vitamins used to boost immune system, etc.).

If any of the Prohibited Therapy criteria are met by a subject, the subject may remain in the study however the inclusion of the subject's data within the PP set, IPP set, ITT set, or SAS may be impacted.

6 TREATMENT ALLOCATION AND BLINDING

6.1 Randomization

In the Phase 2 portion, subjects will be randomized 5:1 to receive the CoVLP formulation and placebo in each Study Population. In the Phase 3 portion, subjects will be randomized 1:1 to receive the CoVLP formulation and placebo in each Study Population. Randomization of elderly adult subjects (Study Population #2) will be stratified by age in an approximately 2:1 ratio of 65 to 74 years of age and 75 years of age and older, respectively. During Phase 2 and Phase 3 portions of the study, eligible subjects will be randomized at the Day 0 visit using the interactive response technology (IRT) system.

For the immunogenicity subset in the Phase 3 portion, subjects will be enrolled from select clinical sites based on the sites capability to perform either cryopreservation of peripheral blood mononuclear cell (PBMC) samples and / or the requirement to be located near the laboratory where whole blood samples could be delivered quickly for PBMC processing. At the clinical sites, selection of the subjects to be included in this subset will be performed by the unblinded

statistical team or the IRT system sequentially starting with the first subject of the day, based on age distribution (when applicable), treatment received, and the site's daily capacity to process PBMC samples until the targeted sample size of the site is obtained. The same process will be followed for enrolling subjects into each of the three Study Populations.

Once a randomization number has been assigned, it will not be re-used for any reason. No subjects will be randomized into the study more than once. If a randomization number is allocated incorrectly, no attempt will be made to remedy the error once the study vaccine has been dispensed: the subject will continue on the study with the assigned randomization number and associated treatment. The study staff will notify the Sponsor Contact as soon as the error is discovered without disclosing the study vaccine administered. Admission of subsequent eligible subjects will continue using the next unallocated number in the sequence.

Subjects will be enrolled into treatment groups in each phase of the study based on a computer-generated randomization schedule prepared by or under the supervision of Medicago before the study. The randomization number and treatment will be recorded along with a unique subject number for each subject in the investigational product accountability log.

6.2 Blinding

Both portions of the study will be observer-blinded.

From the start of the Phase 2 and Phase 3 portions up to completion of the PVE analysis, the following individuals will not have access to treatment allocation (i.e. remain "blind"): the subjects, the Investigators and all personnel involved in the clinical conduct of the study (except the staff involved in the preparation and administration of the study vaccine, the quality assurance auditor, and quality control reviewers), Medicago clinical staff and medical staff involved in safety evaluations (e.g. causality assessments), and all personnel involved in sample analysis at the central and testing (Nab, ELISA, ELISpot, ICS [flow cytometry], serological, and virologic method SARS-CoV-2 diagnostics) laboratories. Once RA/A is obtained, the Phase 2 and Phase 3 studies may initiate Period 2 (Table 3). After the PVE analysis is completed and in preparation for Period 2, the study may be unblinded so that subjects may decide whether to participate in Period 2 after first RA/A.

During the study, the IDMC and unblinded team, compromised of the unblinded medical monitor, unblinded biostatistician, unblinded senior reviewer, and unblinded programmer involved in the preparation of the safety data summary for the IDMC reviews, will have access to or be aware of treatment allocation (i.e. be "unblinded"). The IDMC will review safety data and will make recommendations for the entire duration of the study. The same unblinded statistical team will be involved in the Day 21 and Day 42 (if needed) immunogenicity data analyses in the Phase 2 portion and in the primary vaccine efficacy (PVE) analyses in the Phase 3 portion.

From the start of the Phase 2 and Phase 3 portions up to completion of the PVE analysis, the Sponsor's Study Team will remain blinded. A small number of Medicago personnel (not included in Study Team) will be involved in the planned analyses during the Phase 2 and Phase 3

portions. During the PVE analyses in the Phase 3 portion, some Medicago personnel will be unblinded at the group level to support the review of the analyses however these individuals will NOT be unblinded at the treatment allocation level. The selected individuals and the process and level of unblinding will be documented. After the PVE analysis is completed and in preparation for Period 2, the Sponsor may be unblinded in order for Period 2 to be initiated immediately after first RA/A.

In order to efficiently manage IP inventory at sites in the Phase 3 portion, a Medicago Unblinded Clinical Supply Manager may have access to site inventory and may therefore be exposed to subject level treatment allocation. The unblinded role of this individual will be documented and the individual will be trained to maintain the study blind. The Unblinded Clinical Supply Manager will not have access to study outcomes such as COVID-19 test results.

Since the CoVLP formulation will have a different physical appearance and viscosity from the placebo, the site staff involved in the preparation and administration of the treatments will not be involved in any activity that could introduce a bias, such as the evaluations of reactogenicity or AEs experienced by the subjects following vaccination.

Any code break will be documented and reported to Medicago (or its designee) in a timely manner. In a medical emergency, the Investigator may unblind the treatment for that subject without prior consultation with the Sponsor. In such an event, the Investigator will need to contact the responsible Medical Monitor as soon as possible after the unblinding to discuss the case. The treatment allocation should not be disclosed by the Investigator to the responsible Medical Monitor.

As approved or authorized vaccines for COVID-19 become publicly available to subjects in some regions, subjects may request to become prematurely unblinded. In order to proceed with any such requests for unblinding in the Phase 2 or Phase 3 portion, the following two criteria must be met:

1. Subject is eligible for an approved or authorized COVID-19 vaccine in their local region, and
2. Subject has been approached or received notification by a healthcare provider, or is eligible based on local rules and requirements, to receive a publicly available approved or authorized COVID-19 vaccine. The notification must be shared with the Investigator.

If the subject fulfils requirements of the two criteria, then the date of premature unblinding will be captured in the IRT system. If the subject does not meet both criteria, he/she will not be eligible for premature unblinding. Details regarding the subject's status in the study are included in Section 11.6.

Serological testing performed/requested by study subjects or performed as part of any medical procedures (e.g. blood/plasma donation) that may result in treatment unblinding is not permitted.

7 DOSAGE AND ADMINISTRATION

The CoVLP formulation selected for the Phase 2 and Phase 3 portions of the study has been selected based on the Phase 1 study data, including acceptable safety and immunogenicity profiles.

In the Phase 2 portion, subjects will receive two IM injections 21 days apart on Days 0 and 21, into the deltoid region of the alternating (if possible) arm (i.e., each arm will be injected once), of one of the following:

- CoVLP formulation;
- Placebo.

The use of the same dose of CoVLP for all Study Populations may not be adequate. Subjects in Study Population #1 (healthy adults 18 to 64 years of age) will receive the standard dose of CoVLP (3.75 µg) adjuvanted with AS03 in an injection volume of 0.5 mL.

Study Populations #2 and #3 (elderly adults or adults with significant comorbidities, respectively) will receive the standard dose of CoVLP (3.75 µg) adjuvanted with AS03 in an injection volume of 0.5 mL. If an acceptable immune response is not achieved at either Day 21 or Day 42, the IDMC may recommend a dose escalation to 7.5 µg CoVLP adjuvanted with AS03 in a volume of 0.5 mL, and the same number of subjects (i.e. 306 subjects per Study Population) in the particular Study Population will be recruited, using the same enrollment approach (i.e. use of sentinel subjects and IDMC reviews). If the higher dose of the CoVLP formulation achieves an acceptable immune response in the Phase 2 portion, then the same higher dose will be used in that particular Study Population during the Phase 3 portion of the study.

In the Phase 3 portion, each Study Population will receive the same dose of CoVLP adjuvanted with AS03 and injection volume established for that particular Study Population during the Phase 2 portion of the study.

Subjects will receive two IM injections 21 days apart on Days 0 and 21, into the deltoid region of the alternating (if possible) arm (i.e., each arm will be injected once), of either the CoVLP formulation or the placebo.

Subjects in the placebo group will be offered the CoVLP formulation once RA/A is obtained.

8 SPECIMENS AND CLINICAL SUPPLIES

8.1 Management of Samples

Blood samples for biochemistry, haematology, and serology (for HIV, Hepatitis B, and Hepatitis C) and urine samples for urinalysis will be collected at screening and/or during the study for the Phase 2 portion of the study. Serum (Phase 2 portion only) and urine (dipstick or similar) pregnancy testing will be performed for females of childbearing potential. Blood samples will also be collected for the testing of SARS-CoV-2 antibodies at screening for the Phase 2 portion and during the Phase 3 portion of the study.

Blood samples for immunogenicity analysis will be collected from all subjects in the Phase 2 portion of the study and from a subset of subjects in the Phase 3 portion of the study. In the Phase 3 portion, additional immunogenicity samples will be collected at Day 0 and Day 42 from all subjects. Bioanalysis of the immunogenicity blood samples will be conducted at central laboratories.

Between vaccination and the end of the surveillance period, nasal or NP swabs will be collected from any subject who reports any symptoms associated with COVID-19 after the start of active and passive surveillance at the discretion of the site Investigator; these swabs will be collected each time a subject reports such an event within the specified timeframe, so multiple collections may be required from some subjects. The site Investigator's reason for not collecting swabs from such a subject must be clearly documented. At least two swabs will be collected per subject per event (at least one from each nare). If the subject cannot visit the Investigator site, then the subject may receive a visit at home (or hospital, if applicable) to collect the nasal/NP swabs samples or be provided with self-administered nasal swabs for sample collection. Bioanalysis of the nasal or NP swabs will be conducted at the central laboratory as well as local laboratories, to mitigate the risk of long delays in shipping samples to the central virology laboratory (refer to Section 10.4.1 for more details).

Complete information on the supplies, handling, storage, and shipment of all laboratory samples will be described in the study-specific documentation.

8.2 Collection of Samples

During the Phase 2 and Phase 3 portions of the study, the Time and Events Schedules: General Information for each portion (see Table 1, Table 2, and Table 3) summarize the frequency and timing of the scheduled samples to be collected.

All subjects in the Phase 2 portion of the study will have blood sampled. Subjects will have blood volumes drawn of up to approximately 297 mL over a period of 386 days (Table 7).

Table 7 Estimated Blood Volume Drawn: Phase 2 Portion

Type of Sample	Volume per Sample (mL)	Number of Samples per Subject										Total Volume of Blood per Subject (mL)
		Visit 1 (Day -14 to 0)	Visit 2 (Day 0)	Visit 3 (Day 3)	Visit 4 (Day 21)	Visit 5 (Day 24)	Visit 6 (Day 42)	Visit 7 (Day 128)	Visit 8 (Day 201)	Visit 9 (Day 386)	Total	
Test for SARS-CoV-2 antibodies	5	1									1	5
Biochemistry, haematology, serology (HIV, hepatitis B & C, pregnancy test)*	8	1		1	1	1					4	32
Serology for immunogenicity (Nab assay, ELISA)	10		1		1		1	1	1	1	6	60

Type of Sample	Volume per Sample (mL)	Number of Samples per Subject										Total Volume of Blood per Subject (mL)
		Visit 1 (Day -14 to 0)	Visit 2 (Day 0)	Visit 3 (Day 3)	Visit 4 (Day 21)	Visit 5 (Day 24)	Visit 6 (Day 42)	Visit 7 (Day 128)	Visit 8 (Day 201)	Visit 9 (Day 386)	Total	
CMI response (PBMC)	40		1		1		1		1	1	5	200
Total volume of blood per subject (mL)		13	50	8	58	8	50	10	50	50		297

* Sample collected during the screening visit is recommended to be performed under fasting conditions (approximately 12 hours) for cholesterol and triglyceride analysis. Note that cholesterol and triglyceride analysis will be performed only at screening.

In the Phase 3 portion of the study, all subjects will have up to a total of 30 mL of blood drawn. Subjects included in the immunogenicity subset will have up to an additional blood volume of up to approximately 250 mL drawn up to the end of the study. In total, subjects will have up to 30 to 280 mL of blood drawn up to the end of the study (Table 8).

Once placebo subjects receive the CoVLP formulation post-RA/A, they will no longer provide any blood draws with the exception of the following:

- Subjects will provide a 5 mL blood sample immediately prior to the administration of the first dose of the CoVLP formulation to test for SARS-CoV-2 N antibodies. The total blood drawn up to the end of the study in all subjects remains the same.
  - If RA/A occurs post-Day 201, then no blood sample will be collected as a blood sample to test for SARS-CoV-2 N antibodies will be collected on Day 201.

Table 8 Estimated Blood Volume Drawn: Phase 3 Portion

Type of Sample	Volume per Sample (mL)	Number of Samples per Subject						Total Volume of Blood per Subject (mL)
		Visit 1 (Day 0)	Visit 2 (Day 21)	Visit 3 (Day 42)	Visit 4 (Day 201)	Visit 5 (Day 386)	Total	
Test for SARS-CoV-2 N antibodies	5	1			1†		2	10
Immunogenicity – serology (Nab assay only)	10	1		1			2	20
Immunogenicity-Serology (Nab assay, ELISA)*	10	1	1	1	1	1	5	50
Immunogenicity-CMI response (ELISpot and ICS)*	40	1	1	1	1	1	5	200
Total volume of blood per subject (mL)		15-65	50	10-60	5-55	50		30-280

* These samples will be collected for pre-defined subset of subjects.

Type of Sample	Volume per Sample (mL)	Number of Samples per Subject						Total Volume of Blood per Subject (mL)
		Visit 1 (Day 0)	Visit 2 (Day 21)	Visit 3 (Day 42)	Visit 4 (Day 201)	Visit 5 (Day 386)	Total	

† This blood sample will be collected from all subjects on Day 201. However, if RA/A occurs prior to Day 201, then only in placebo subjects receive the CoVLP formulation post-RA/A, this blood sample will be collected immediately prior to the administration of the first dose of the CoVLP formulation.

Urine samples will be collected at screening and Days 3, 21, and 24 during the Phase 2 portion of the study.

In the Phase 2 portion of the study, pregnancy testing will be performed for all females of childbearing potential (in Study Populations #1 and #3). A serum pregnancy test will be performed during screening. A urine (dipstick or similar) pregnancy test will be performed at Day 0 prior to randomization and at Days 21 and 42. In Study Populations #1 and #3, females of childbearing potential must have negative serum pregnancy test result from screening and urine pregnancy test results from Day 0 and Day 21 available prior to vaccination.

In the Phase 3 portion of the study, pregnancy testing will be performed for all females of childbearing potential (in Study Populations #1 and #3). A urine (dipstick or similar) pregnancy test will be performed at screening and at Day 0 prior to randomization and at Days 21 and 42. In Study Populations #1 and #3, females of childbearing potential must have negative urine pregnancy test results from screening and Day 0 and Day 21 available prior to vaccination.

8.3 Clinical Supplies

The study center will be provided with or be responsible for the provision of supplies for nasal or NP swabs and blood collection and shipment supplies (e.g. aliquot tubes, aliquot labels, aliquot storage, shipping boxes, and accompanying manifests) as described in the study-specific documentation. Sites will be authorized to use their own materials if agreed to by Medicago (or its designee).

9 TREATMENT COMPLIANCE

Study treatments will be prepared and administered by trained unblinded site staff via IM injection to each subject at each Investigator site. In addition, the Investigator or designated study center personnel will maintain a log of all study treatments dispensed and administered to the subject. Investigational product inventory and accountability will be managed throughout the Phase 2 and Phase 3 portions of the study by unblinded site staff (details will be provided in the study-specific documentation). If an eligible subject refuses vaccination post-randomization, the reason will be documented in the source and electronic case report form (eCRF).

10 STUDY EVALUATIONS

10.1 Study Procedures

10.1.1 Overview

During the Phase 2 and Phase 3 portions of the study, the Time and Events Schedules: General Information for each portion (see Table 1, Table 2, and Table 3) summarizes the frequency and timing of scheduled assessments applicable to each portion of the study.

For the Phase 3 portion, once placebo subjects receive the CoVLP formulation post-RA/A, these subjects will stop following the planned assessments (i.e. visits, contacts, surveillance) and will perform the following assessments in Section 10.1.15. Subjects in the placebo group who do not want the CoVLP formulation but want to remain in the study will continue the study as planned.

10.1.2 General COVID-19 Precautions at Clinical Sites

Sites participating in this study will have processes in place locally for following recommendations of the local Public Health authorities for the management of COVID-19. Subjects will be asked to follow good hygiene and safe physical distancing measures such as wearing a face mask on site, frequent hand hygiene, and maintaining a physical distance of 2 meters (or 6 feet) from others, except during procedures where staff must come into close proximity of subjects (e.g. vaccination, blood draws, collecting vital sign measurements).

Staff at the clinical sites will also follow the same good hygiene and safe physical distancing measures as the subjects at the site. In addition, the staff will be responsible for disinfecting materials and/or areas between each use by subjects and staff. All staff involved with on-site procedures will have a back-up member who is qualified to perform the same duties / responsibilities in the event that a member of the staff is infected with SARS-CoV-2 or comes into contact with an individual known to have COVID-19 and is restricted to self-isolation and following the recommendations of the local Public Health authorities for the management of COVID-19.

10.1.3 Screening (Visit 1 for Phase 2 Portion and Phase 3 Portion)

The following procedures will be performed at the initial screening visit (Visit 1) for the Phase 2 and Phase 3 portions of the study:

- Review and proceed with the signature of the ICF. The Investigator or his/her designee, will fully inform the subject of the nature and scope of the study, potential risks and benefits of participation, and the study procedures involved and will answer all questions prior to requesting the subject’s signature on the ICF. The subject’s consent must be obtained prior to performing any study-related procedures; the consent process must be clearly recorded and a copy of the signed ICF retained in the source documents. A copy of the ICF must be provided to the subject;

- Review the inclusion and exclusion criteria and determine the subject's eligibility to participate in this study. The eligibility must be confirmed by the Investigator after review of all procedures and findings and prior to randomization;
- Collect and review demographics (gender, date of birth, age, race, and ethnicity) and body measurements (BMI, weight [kg or lbs], and height [cm or inches]) data. BMI is to be calculated as body weight (kg) divided by the square of height (m); the BMI result will be rounded to one decimal place using the standard convention. For the measurement of body weight, subjects will be lightly clothed, without shoes;
- Collect and review medical history, including the grade of any medical conditions (medical conditions are to be graded using the same scale as for AEs; see Section 13.1.2). The medical history should record significant problems active at the time of screening and present within the preceding six months. Problems that have been clinically inactive for more than six months preceding screening, but which might alter the subject's current or future medical management, should also be noted (e.g. cancer, autoimmune disease, known mitral valve prolapse or a remote history of a seizure disorder);
- Review and record current and previous medication use (up to 30 days prior to study vaccine administration), with the following exception (refer to exclusion criterion no. 5):
  - For subjects who have been administered the following medication or treatment, review and record current and previous medication use up to the time period specified for the medication or treatment:
    - Study Populations #1 and #2: cytotoxic, antineoplastic, or immunosuppressant drugs – within 36 months prior to vaccination;
    - Study Populations #1 and #2: any immunoglobulin preparations or blood products, blood transfusions – within 6 months prior to vaccination;
- Perform a limited physical examination. The physical examination will be performed by the Investigator or sub-Investigator;
- Perform a vital signs measurement, including resting blood pressure (BP), heart rate (HR), respiratory rate (RR), and oral temperature (OT). OT will be collected in degrees Celsius or Fahrenheit using a digital thermometer. The OT measurement should not be collected immediately following consumption of a hot or cold beverage or after smoking. The BP will be taken after the subject has been in a seated position as per the site's standard procedure. The BP should be taken by cuff (manual or automated are both acceptable). BP, HR, and RR may be repeated once if judged necessary. All measurements (including any repeats) will be recorded in the source documents. Inclusion of subjects with an out-of-range BP or HR or RR measurement will be based on the Investigator's judgment;
- For the Phase 2 portion only: collect screening blood samples for biochemistry, haematology, and serology (HIV, Hepatitis B, and Hepatitis C screening) for analysis as well as to test for SARS-CoV-2 antibodies. Sample blood collection at the screening visit is recommended to be performed under fasting conditions (approximately 12 hours) for cholesterol and triglyceride analysis;

- For the Phase 2 portion only: perform urinalysis on all subjects;
- Perform serum (Phase 2 portion) or urine (Phase 3 portion) pregnancy testing for female subjects of childbearing potential at screening;
- For the Phase 3 portion only: proceed to the procedures for Dose 1 Vaccination (see Section 10.1.4) and perform only the procedures in Section 10.1.4.1 that have not been performed.

10.1.4 Dose 1 Vaccination (Day 0) (Visit 2 for the Phase 2 Portion and Continuation of Visit 1 for Phase 3 Portion)

Emergency equipment must be available on site and appropriate treatment must be instituted as soon as possible in the event of anaphylaxis or any other immediate hypersensitivity reaction. The Investigator will be on-site on vaccine administration days and for the duration of the observation period (minimum of 30 minutes after vaccination) for the last subject dosed on that day. The Investigator will be available on call for the remainder of the study. A physician should be immediately available at the clinical site to administer treatment or to apply procedures for any immediate AEs/SAEs.

10.1.4.1 Prior to Vaccination

The following procedures will be performed on Day 0 (Visit 2) prior to vaccination for the Phase 2 portion of the study. For the Phase 3 portion, the screening and vaccination visit will occur on the same day and the following procedures will be performed (as long as they have not been completed as per the Phase 3 specific procedures listed in Section 10.1.3):

- Record changes in medical history and medications and confirm that the subject continues to meet all inclusion and no exclusion criteria;
- Perform a vital signs measurement, including resting BP, HR, RR, and OT. OT will be collected in degrees Celsius or Fahrenheit using a digital thermometer. The OT measurement should not be collected immediately following consumption of a hot or cold beverage or after smoking. The BP will be taken after the subject has been in a seated position as per the site's standard procedure. The BP should be taken by cuff (manual or automated are both acceptable). BP, HR, and RR may be repeated once if judged necessary. All measurements (including any repeats) will be recorded in the source documents. Inclusion of subjects with an out-of-range BP or HR or RR measurement will be based on the Investigator's judgment;
- Measure the BMI; for this visit, only weight will be measured while the height will be obtained from that measured at the initial screening visit. The BMI result will be rounded to one decimal place using the standard convention. For the measurement of body weight, subjects will be lightly clothed, without shoes;
- Collect a urine sample for female subjects of childbearing potential and perform a urine dipstick (or similar) pregnancy test. No study vaccine must be administered to a childbearing potential female until a negative result is obtained and documented;

- If the subject is judged eligible for the study and is still willing to participate in the study, randomize the subject into the study;
- After confirmation of eligibility and randomization, collect baseline blood samples to test for:
  - SARS-CoV-2 antibodies (Phase 3 portion) assessment;
  - Immunogenicity (serology [Nab assay and ELISA] and CMI) assessments [Phase 2 portion - all subjects; Phase 3 portion - immunogenicity subset]);
  - Additional immunogenicity (serology) assessment (Phase 3 portion).

  Prepare and store these samples until shipment to the analytical laboratory.

10.1.4.2 Vaccination

Once all pre-vaccination procedures have been completed and subject eligibility determined, the subject will be randomized and the study vaccine will be administered IM into the deltoid muscle of the non-dominant (if possible) arm, as described in the Investigational Product Management Manual. In order to prevent possible confounding of vaccination site reactions, whenever possible, blood samples will not be collected from the same arm as the one used for vaccination. The arms used, both for blood collection and vaccination, will be documented in the source documents. For subjects with BMI < 30 kg/m^2, a 23 gauge needle of at least one inch or 2.5 cm in length can be used for vaccination. For subjects with BMI ≥ 30 kg/m^2, a needle of at least 1.5 inches or 3.5 cm in length must be used to ensure intramuscular delivery. The preparation and administration of the study vaccine will be performed by an unblinded site staff member.

10.1.4.3 Thirty Minutes Post-vaccination

The post-vaccination observations will be performed by a blinded site staff member. The following safety observation procedures will be performed for all subjects immediately following study vaccine administration:

- Subjects will remain in the clinic for at least 30 minutes post-vaccination for observation. The observation period will include an assessment of immediate solicited local and systemic AEs. Solicited local and systemic AEs occurring within 30 minutes post-vaccination will be recorded in the electronic diary by the subjects and corresponding eCRF. All unsolicited AEs occurring within 30 minutes post-vaccination will be recorded in the subject source and AE eCRF. Any unusual signs or symptoms reported during the initial 30 minutes post-vaccination will result in continued close monitoring. Based on their condition, subjects may be asked to remain in the clinic for their safety for more than 30 minutes after vaccination (the reason will be recorded in the source documents). All data (including the assessment of solicited local and systemic AEs) will be recorded in the source documents during and after the observation period. Refer to Section 10.2.1 for details regarding the assessment of AEs and/or solicited local and systemic AEs;
- During the observation period, subjects will be provided with a measurement device template (Section 19.1) for measuring (in mm) solicited local AEs of erythema (redness)

and swelling and an oral digital thermometer for recording daily temperature (in degrees Celsius or Fahrenheit). Subjects will also be provided with an electronic diary (Section 19.2) and will be shown how to enter their data in the diary. Each subject will be provided with the following instructions on the measurements they are to make:

- How to collect his/her OT in degrees Celsius or Fahrenheit with the provided digital thermometer:
  - From the evening of Day 0 to the evening of Day 7, OT will be measured at approximately the same time each evening and the results recorded in the electronic diary;
  - The OT should not be collected immediately following consumption of a hot or cold beverage or after smoking;
  - The subject is to also take his/her OT if he/she feels feverish and to record the highest temperature of the day. In the event that a temperature ≥ 38.0 °C or ≥ 100.4 °F is obtained, the subject will be allowed to take over-the-counter antipyretics (e.g. acetaminophen/paracetamol, aspirin, naproxen, or ibuprofen) and will be advised to increase the frequency of OT measurements to approximately every four hours, until he/she is no longer febrile (fever is defined as a temperature of ≥ 38.0 ºC or ≥ 100.4 °F). The subject is to document medication intake, which will be reviewed by the site personnel;
- How to measure any solicited local AEs, including erythema (redness) and swelling diameter at the injection site using the measurement template supplied for this purpose; subjects will also be requested to evaluate pain at the injection site. Local AEs will be assessed every day starting in the evening of Day 0 and up to the evening of Day 7 and the results will be recorded. The severity of solicited local AEs will be graded according to the Food and Drug Administration (FDA) Guidance for Industry: Toxicity Grading Scale for Healthy Adult and Adolescent Volunteers Enrolled in Preventive Vaccine Clinical Trials [FDA 2007], as presented in Table 9.
- How to grade, on a daily basis from the evening of Day 0 through to the evening of Day 7, each of the solicited systemic AEs and their severity (as per the same guidance used for solicited local AEs; see Table 9) [FDA 2007] and to record the worst grade of the day for each of these solicited systemic AE. The instructions will include how to examine and grade swelling in the neck and axilla and to record any unusual feeling and/or swelling;
- Subjects will be advised that they will be asked about the occurrence of any symptoms or events requiring medical attention and the use of any concomitant medication during the 21-day post-vaccination period, after each vaccination, and until the end of the study. Subjects will also be provided with a memory aid (Section 19.3) to record unsolicited AEs and any concomitant medication use and will be shown how to enter their data in the memory aid;

- Subjects will be instructed to contact the clinical site for any unsolicited AEs and/or solicited local and systemic AEs of greater than Grade 2 (moderate). Based on their condition, the Investigator may request that the subject return to the clinic for evaluation;
- Subjects will be advised on emergency contact information and instructions for contacting study personnel. Subjects will be advised to immediately contact the Investigator (or his/her designee) in the event of an SAE or a medical emergency. Subjects will be provided with a phone contact number and be instructed to call if any suspected reaction to the vaccination is felt to be significant or of concern;
- Subjects will be advised to notify their health care professional(s) (e.g. primary care physician) that they are participating in a clinical research study of a SARS-CoV-2 / COVID-19 vaccine;

- Phase 2 portion only: after the 30-minute observation period (allowed window of + 15 minutes) is completed, measure vital signs (BP, HR, RR, and OT) as described in Section 10.2.1.5. Any out-of-range measurements will be assessed by the Investigator (or his/her designee) and any further action will be determined upon his/her medical decision;
- Phase 2 portion and Phase 3 portion: Instruct the subjects to perform measurements of local and systemic AEs at approximately the same time each day from Day 0 to Day 7 (preferably in the evening);
- Post-RA/A: Subjects will not be required to collect solicited local and systemic AEs from Day 0 to Day 7 post-vaccination. Subjects will continue to use the memory aid to record unsolicited AEs and any concomitant medication use;
- Advise each subject to report to the clinical site if he/she has tested positive for COVID-19;
- Provide appointments (date and time) for the next planned visit to the clinical site (Day 3 for the Phase 2 portion or Day 21 for the Phase 3 portion) and for the Days 1 and 8 phone contacts (Phase 2 portion only);
- The subject will be released from the clinical site once all relevant Day 0 post-vaccination procedures have been completed and the subject is in stable condition.

10.1.5 Day 1 and Day 8 (Telephone Contact for Phase 2 portion only)

The post-vaccination phone contacts will be performed by blinded site staff members. The following procedures will be performed during the phone contact (acceptable interval of + 1 day for Day 1, and ± 1 day for Day 8) for the Phase 2 and Phase 3 portions of the study:

- Ask the subjects about any difficulties in recording their data, any change in health, any visits to health care facilities and/or medical practitioners, and any use of concomitant medications. Record the information in the source documents;
- For any unsolicited AEs and/or solicited local and systemic AEs of greater than Grade 2 (moderate), the Investigator should be informed within 24 hours of the time the clinical site is made aware of the event. The Investigator may request that the subject return to the clinic for evaluation;

- Advise subjects to immediately contact the Investigator (or his/her designee) in the event of any AE that requires medical attention. Subjects will be provided with a phone contact number and be instructed to call if any suspected reaction to the vaccination is felt to be significant or of concern;
- Remind the subjects how to measure and record any solicited local and systemic AEs. Subjects should also be reminded to record any changes in health, including changes in AEs and changes in medications;
- Remind each subject to immediately report to the clinical site any symptoms associated with COVID-19 or if he/she has tested positive for COVID-19, as defined in Section 10.4.1. Ensure that subjects have the memory aid listing the COVID-19 symptoms and contact information for the study site;
- Remind subjects of their next appointment (date and time) for the clinical visit and/or the next telephone contact. Subjects will also be reminded to record their data in the diary and memory aid in a timely manner.

In the event that a subject cannot be reached via phone, he/she may be contacted by text message or via email (if these contacts are available). However, the phone should be the initial and preferred means of communication. At least three attempts within allowed time window should be made in order to reach the subject.

10.1.6 Surveillance for COVID-19 Cases

From the day of the first vaccination (Day 0, post vaccination), passive and active surveillance will be performed until the end of the Phase 2 or the Phase 3 portion of the study, except active surveillance in the Phase 3 portion will end once RA/A is obtained:

- Passive surveillance: subjects will be instructed to contact the study site as soon as possible in case they experience symptoms of COVID-19, as defined in Section 10.4.1;
  - Subjects will also be advised to inform the clinical site if they have tested positive for SARS-CoV-2 infection, with or without the presence of any symptoms associated with COVID-19, and will be asked to provide a documented test result. If/when the subject begins to experience symptoms associated with COVID-19, the process for the collection of symptoms and nasal or NP swabs (as noted below) will be followed;
- Active surveillance: during the Phase 2 portion, subjects will be contacted once per week via phone, text, or email. During the Phase 3 portion, the subjects will be contacted once per week via the electronic diary and asked about symptoms of COVID-19. Other methods of contact (phone, text, email) will only be used as a last resort to contact the subject;
- If a subject reports any symptom(s) of COVID-19:
  - Collect information regarding COVID-19, including all applicable symptoms and the onset date:
    - The onset date is defined as the first date when one or more of the symptoms occurred;

  - Symptoms collected should also include the presence or absence, severity, and duration of concurrent symptoms (i.e. fever, feverishness [feeling of warmth], chills [shivering], tiredness [fatigue], headache, myalgia [muscle aches], nausea, vomiting, or diarrhea);
- Collect information on any associated concomitant medication use;
- Schedule a time when the subject can return to the clinical site for the collection of nasal or NP swabs. If the subject cannot visit the Investigator site, then schedule a home (or hospital, if applicable) visit to collect the nasal/NP swabs samples or provide the subject with self-administering nasal swabs for sample collection. Symptoms will be evaluated daily by the subject and reported in their memory aid until the subject visits the clinical site. The Investigator will confirm whether nasal or NP swabs should be collected. The nasal or NP swabs are to be collected within 72 hours (preferably within 48 hours) after the reporting of a COVID-19 symptom;

  Note: If swabs cannot be collected within 72 hours after reporting of the COVID-19-associated symptom, nasal or NP swabs should be collected at the next available opportunity for the subject to visit the Investigator site or at home (or hospital, if applicable) and date and time of collection recorded.
- At the same visit where the Investigator will confirm whether nasal or NP swabs should be collected, subjects will be trained on daily recording of their COVID-19-associated symptoms using a COVID-19 diary and will be asked to immediately start recording their symptoms in the COVID-19 diary. Subjects will collect any COVID-19-associated symptoms in their COVID-19 diary until a negative swab result for the subject is received from the central virology laboratory or until the subject has no more COVID-19-associated symptoms. The Investigator's clinical judgement may be applicable to determine when COVID-19 disease has resolved or COVID-19-associated symptoms have resolved with some sequelae for a subject and when the collection of COVID-19-associated symptoms can be stopped;
- COVID-19 cases that start on or after the day of the first vaccination (Day 0, post vaccination) will be followed up. This follow-up will be conducted via the planned active surveillance contacts. All follow-up information should be collected, regardless of whether nasal or NP swabs could be obtained. As part of this follow up, the following information will be needed (more details in Section 10.4.1):
  - Collect any new information regarding the evolution of the COVID-19 case, including symptoms and any associated concomitant medication use;
  - Collect the results of the subject's serial (every other day) self-administered nasal swab testing results. The swab testing is to be continued for approximately 2 weeks. However, if the COVID-19-associated symptoms:
    - Persist for more than 2 weeks, then the Investigator's clinical judgement is required to extend the collection of swabs beyond the 2 weeks;

    - Have resolved in less than 2 weeks and the Investigator believes the subject has virologically recovered, then two consecutive negative results, obtained locally, are required prior to stopping the collection of the self-administered swabs;
- If a subject has a positive COVID-19 case but does not present any COVID-19-associated symptoms, then there will be no collection of swabs from this subject nor the requirement to report daily COVID-19-associated symptoms. However, if this subject presents any COVID-19-associated symptoms, then the collection of swabs and reporting of symptoms can be started as described above;
- Ensure subjects have the memory aid listing the symptoms of COVID-19 and contact information for the study site; ensure subjects use the diary or memory aid, as applicable, to record reportable information.

10.1.7 Day 3 Follow-up (Visit 3 for Phase 2 portion only)

The post-vaccination follow-up visit procedures will be performed by blinded site staff members at the clinical site. For subjects in quarantine, these procedures will be performed by blinded trained medical staff and may be at the subject's home (or place of quarantine). The following procedures will be performed during the Day 3 visit (± 1 day) for the Phase 2 portion of the study:

- Perform a vital signs measurement, including resting BP, HR, RR, and OT;
- Perform urinalysis on all subjects;
- Collect blood samples for biochemistry and haematology;
- Review the diary and memory aid content with the subject to ensure appropriate completion. Corrections must be made by the subject him/herself and all corrections must be initialed and dated;
- Review the subject's safety data and ensure all updates on concomitant medication usage and any changes in the subject's health (AEs, SAEs, AESIs, or MAAEs) are recorded appropriately;
- Advise the subjects to immediately contact the Investigator or his/her designee, in the event of any AE that requires a visit to an emergency room and/or hospitalization;
- Remind each subject to immediately report to the clinical site any symptoms associated with COVID-19 or if he/she has tested positive for COVID-19, as defined in Section 10.4.1. Ensure that subjects have the memory aid listing the COVID-19 symptoms and contact information for the study site;
- Remind the subjects of their next telephone contact (date and time).

10.1.8 Day 21 Dose 2 Vaccination (Visit 4 for Phase 2 portion and Visit 2 for Phase 3 portion)

If for any reason the Day 21 visit is done before or after the planned date, then subsequent visits/procedures (Days 22 (Phase 2 portion only), 24 (Phase 2 portion only), 29 (Phase 2 portion only), 42, 128 (Phase 2 portion only), 201, and 386) will be adjusted accordingly.

10.1.8.1 Pre-vaccination

The pre-vaccination procedures will be performed by blinded site staff members. The following procedures will be performed during the Day 21 (± 2 days) visit prior to vaccination for the Phase 2 and Phase 3 portions of the study:

- Perform a vital signs measurement, including resting BP, HR, RR, and OT;
- Phase 2 portion only: perform urinalysis on all subjects;
- Perform urine (dipstick or similar) pregnancy testing for female subjects of childbearing potential. No study vaccine must be administered to a childbearing potential female until a negative result is obtained and documented;
- Phase 2 portion only: collect blood samples for biochemistry and haematology;
- Phase 2 portion (all subjects) and Phase 3 portion (immunogenicity subset): collect blood samples for immunogenicity (serology [Nab assay and ELISA] and CMI) assessments; prepare and store these samples until shipment to the analytical laboratories;
- Review the diary content with the subject to ensure appropriate completion. Review the memory aid with the subject as well. Corrections must be made by the subject him/herself and all corrections must be initialed and dated;
- Review the subject's safety data and ensure all updates on concomitant medication usage and any changes in the subject's health (AEs, SAEs, or AESIs) are recorded appropriately;
- Advise the subjects to immediately contact the Investigator or his/her designee, in the event of any AE that requires a visit to an emergency room and/or hospitalization;
- Assess the subject for contraindications to the second vaccination according to Section 11.3. If the subject has no contraindications to the second vaccination, proceed to register the visit in the subject's eCRF;
- Register the visit in the subject's eCRF. The IRT system will assign the same treatment assignment at Day 21 as Day 0. If the subject has a contraindication for the second vaccination, record the status in the subject's eCRF.

10.1.8.2 Dose 2 Vaccination

The same procedures will be performed during the administration of the vaccine on Day 21 as will be performed during the administration of the vaccine on Day 0 (see Section 10.1.4.2 for detailed procedures) for the Phase 2 and Phase 3 portions of the study, with the following exception regarding which arm to use for IM administration:

- The study vaccine will be administered IM into the deltoid muscle of the alternate (if possible) arm (i.e. the arm not used for the previous dose administration). Record the administration, left or right arm, in the subject source documents.

10.1.8.3 Post-Dose 2 Vaccination

The same procedures will be performed following the administration of the vaccine on Day 21 as will be performed following the administration of the vaccine on Day 0 (see Section 10.1.4.3 for detailed procedures) for the Phase 2 and Phase 3 portions of the study, with the following exceptions:

- Provide appointments (date and time) for the next planned visit to the clinical site (Day 24 for the Phase 2 portion or Day 42 for the Phase 3 portion) and for Days 22 and 29 telephone contacts (Phase 2 portion only);
- The subject will be released from the clinical site once all post-vaccination procedures have been completed and the subject is in stable condition.

10.1.9 Day 22 and Day 29 (Telephone Contact for Phase 2 portion only)

The same procedures will be performed during the phone contacts on Day 22 and Day 29 as were performed during phone contacts on Day 1 and Day 8 (see Section 10.1.5 for detailed procedures) for the Phase 2 and Phase 3 portions of the study.

10.1.10 Day 24 (Visit 5 for Phase 2 portion only)

The same procedure will be performed during the Day 24 visit as were performed during the follow-up visit on Day 3 (see Section 10.1.7 for detail procedures) for the Phase 2 portion of the study.

10.1.11 Day 42 (Visit 6 for Phase 2 portion and Visit 3 for Phase 3 portion)

For subjects in quarantine, these procedures will be performed by blinded trained medical staff and may be at the subject's home (or place of quarantine). The same procedure will be performed during the Day 42 visit as were performed during the pre-vaccination portion of the visit on Day 21 (see Section 10.1.8.1 for detail procedures) for the Phase 2 and Phase 3 portions of the study, with the following exceptions:

- Review the diary and memory aid content with the subject to ensure appropriate completion. Corrections must be made by the subject him/herself and all corrections must be initialed and dated. Collect the memory aid and provide subjects with another memory aid for the collection of safety data from Day 43 to Day 201;
- Do not collect blood samples for biochemistry and haematology;
- Phase 2 portion only: perform a vital signs measurement, including resting BP, HR, RR, and OT;
- Collect blood samples for:

- Immunogenicity (serology [Nab assay and ELISA] and CMI) assessments Phase 2 portion - all subjects; Phase 3 portion - immunogenicity subset;
- Additional immunogenicity (serology) assessment (Phase 3 portion).

Prepare and store these samples until shipment to the analytical laboratory;

- Remind each subject to immediately report to the clinical site any symptoms associated with COVID-19 or if he/she has tested positive for COVID-19, as defined in Section 10.4.1. Ensure that subjects have the memory aid listing the COVID-19 symptoms and contact information for the study site;
- Provide appointments (date and time) for the next telephone contacts and the next planned visit to the clinical site (Day 128 for the Phase 2 portion and Day 201 for the Phase 3 portion).

10.1.12 Day 128 (Visit 7 for Phase 2 portion only)

The post-vaccination visit procedures will be performed by blinded site staff members at the clinical site. For subjects in quarantine, these procedures will be performed by blinded trained medical staff and may be at the subject's home (or place of quarantine). The following procedures will be performed during the Day 201 visit (± 14 days) for the Phase 2 and Phase 3 portions of the study:

- Review the memory aid content with the subject to ensure appropriate completion. Corrections must be made by the subject him/herself and all corrections must be initialed and dated;
- Review the subject's safety data and ensure all updates on concomitant medication usage and any changes in the subject's health (AEs ongoing from Day 42, SAEs, AESIs, or MAAEs) are recorded appropriately;
- Perform a vital signs measurement, including resting BP, HR, RR, and OT;
- Collect blood samples for immunogenicity (serology [Nab assay and ELISA]) assessments; prepare and store these samples until shipment to the analytical laboratories;
- Remind each subject to immediately report to the clinical site any symptoms associated with COVID-19 or if he/she has tested positive for COVID-19, as defined in Section 10.4.1. Ensure that subjects have the memory aid listing the COVID-19 symptoms and contact information for the study site;
- Provide appointments (date and time) for the next telephone contacts and the next planned visit to the clinical site (Day 201).

10.1.13 Day 201 (Visit 8 for Phase 2 portion and Visit 4 for Phase 3 portion)

The post-vaccination visit procedures will be performed by blinded site staff members at the clinical site. For subjects in quarantine, these procedures will be performed by blinded trained medical staff and may be at the subject's home (or place of quarantine). The following procedures will be performed during the Day 201 visit (± 14 days):

- Review the memory aid content with the subject to ensure appropriate completion. Corrections must be made by the subject him/herself and all corrections must be initialed and dated. Collect manual memory aid and provide subjects with another memory aid for the collection of safety data from Day 202 to Day 386;
- Review the subject's safety data and ensure all updates on concomitant medication usage and any changes in the subject's health (AEs ongoing from Day 42, SAEs, AESIs, or MAAEs) are recorded appropriately;
- Phase 2 portion only: perform a vital signs measurement, including resting BP, HR, RR, and OT;
- Phase 3 portion only: collect blood sample to test for SARS-CoV-2 antibodies;
- Phase 2 portion (all subjects) and Phase 3 portion (immunogenicity subset): collect blood samples for immunogenicity (serology [Nab assay and ELISA] and CMI) assessments; prepare and store these samples until shipment to the analytical laboratories;
- Remind each subject to immediately report to the clinical site any symptoms associated with COVID-19 or if he/she has tested positive for COVID-19, as defined in Section 10.4.1. Ensure that subjects have the memory aid listing the COVID-19 symptoms and contact information for the study site;
- Provide appointments (date and time) for the next telephone contacts (including Day 386, if applicable) and the next planned visit to the clinical site (Day 386), if applicable.

10.1.14 Day 386 (Visit 8 for Phase 2 portion and Visit 5/Telephone Contact for Phase 3 portion)

In the Phase 2 or Phase 3 portion, subjects who are withdrawn/discontinued from the study due to receiving an approved or authorized COVID-19 vaccine prior to RA/A will be asked to complete the procedures for the final telephone contact (refer to Section 10.1.14.2) at the time of unblinding and prior to receipt of the approved or authorized COVID-19 vaccine, if the subject agrees. If the subject premature unblinding occurs prior to the Day 42 visit, then the subject will also be asked about any AEs and recorded appropriately. For subjects who receive the approved or authorized COVID-19 vaccine and then inform the Investigator, the final telephone contact will focus only on the period prior to receiving the approved or authorized COVID-19 vaccine and the subject may be informed retroactively about their treatment allocation.

For all other subjects who withdraw consent from the Phase 2 portion (subjects who initially receive the CoVLP formulation or subjects in the placebo group who do not want the CoVLP formulation post-RA/A) or Phase 3 portion (subjects in the immunogenicity subset who initially receive the CoVLP formulation or receive placebo and do not want the CoVLP formulation post-RA/A), they will be asked to undergo Day 386 visit (refer to Section 10.1.14.1) procedures within two weeks of withdrawal, if the subject agrees.

For all other subjects in the Phase 2 portion (subjects in the placebo group who receive the CoVLP formulation post-RA/A) or Phase 3 portion (all subjects who are not included in the immunogenicity subset and subjects in the placebo group who receive the CoVLP formulation

post-RA/A), they will be asked to participate in the final telephone contact (refer to Section 10.1.14.2) within two weeks of withdrawal, if the subject agrees.

10.1.14.1 Visit 8 for Phase 2 portion and Visit 5 for Phase 3 portion

For the Phase 2 portion (subjects who initially receive the CoVLP formulation or subjects in the placebo group who do not want the CoVLP formulation post-RA/A) and Phase 3 portion (subjects in the immunogenicity subset who initially receive the CoVLP formulation or receive placebo and do not want the CoVLP formulation post-RA/A): the post-vaccination final visit procedures will be performed by site staff members at the clinical site. For subjects in quarantine, these procedures will be performed by trained medical staff and may be at the subject's home (or place of quarantine). The following procedures will be performed during the Day 386 visit (± 14 days):

- Review the subject's safety data and ensure all updates on concomitant medication usage and any changes in the subject's health (AEs ongoing from Day 42, SAEs, AESIs, or MAAEs) are recorded appropriately. Collect the subject's memory aid;
- Confirm that the subject has continued to comply with protocol requirements (e.g. no use of prohibited concomitant medications, reporting of any COVID-19 symptoms);
- Phase 2 portion only: perform a vital signs measurement, including resting BP, HR, RR, and OT;
- Phase 2 portion (all subjects) and Phase 3 portion (immunogenicity subset): collect blood samples for immunogenicity (serology [Nab assay and ELISA] and CMI) assessments; prepare and store these samples until shipment to the analytical laboratories.

10.1.14.2 Telephone Contact for Phase 3 portion

For the Phase 2 portion (subjects in the placebo group who receive the CoVLP formulation post-RA/A) and the Phase 3 portion (all subjects who are not included in the immunogenicity subset and subjects in the placebo group who receive the CoVLP formulation post-RA/A): the post-vaccination final telephone contact will be performed by site staff members. The following procedures will be performed during the Day 386 phone contact (± 14 days):

- Review the subject's safety data and ensure all updates on concomitant medication usage and any changes in the subject's health (AEs ongoing from Day 42, SAEs, AESIs, or MAAEs) are recorded appropriately. Confirm the subject has sent images of the memory aid to the clinical site (using the electronic diary);
- Confirm that the subject has continued to comply with protocol requirements (e.g. no use of prohibited concomitant medications, reporting of any COVID-19 symptoms).

10.1.15 Placebo Subjects who Receive the CoVLP Formulation post-RA/A for Phase 2 and Phase 3 portions and Subjects who Received a Single Dose of the CoVLP Formulation prior to RA/A

For the Phase 2 and Phase 3 portions, placebo subjects who receive the CoVLP formulation post-RA/A will stop following the planned study assessments (i.e. visits, contacts, surveillance) and will perform the following assessments instead.

In addition, for subjects who only received a single CoVLP formulation dose prior to RA/A, the Investigator will use his/her discretion to determine whether these subjects should receive a second dose of the CoVLP formulation post-RA/A. Once a subject receives the second dose of the CoVLP formulation post-RA/A, then the subject will no longer follow the planned study assessments (i.e. visits, contacts, surveillance) and will perform the assessments detailed in Section 10.1.15.1, Section 10.1.15.1.1, Section 10.1.15.2, and Section 10.1.15.3 instead, with the following exceptions:

- Subjects will receive only one dose of CoVLP formulation;
- Subjects do not need to provide a blood sample prior to the administration of the CoVLP formulation.

10.1.15.1 Dose 1 and Dose 2 vaccinations with the CoVLP formulation

Phase 3 portion only: Prior to the administration of the first dose of the CoVLP formulation, a blood sample will be collected from subjects to test for SARS-CoV-2 antibodies. The collected blood sample will be prepared and stored until shipment to the analytical laboratory. The procedure to collect the blood sample will be performed by an unblinded site staff member.

Note: If RA/A occurs post-Day 201, then no blood sample will be collected as a blood sample to test for SARS-CoV-2 antibodies will be collected on Day 201.

Prior to the administration of dose 1 and dose 2 of the CoVLP formulation, subjects will be assessed for contraindications to the first and second CoVLP formulation vaccinations (refer to Section 11.3).

The first CoVLP formulation dose will be administered IM into the deltoid muscle of the non-dominant (if possible) arm, as described in the Investigational Product Management Manual. The arm used for vaccination will be documented in the source documents. For subjects with BMI < 30 kg/m^2, a 23 gauge needle of at least one inch or 2.5 cm in length can be used for vaccination. For subjects with BMI ≥ 30 kg/m^2, a needle of at least 1.5 inches or 3.5 cm in length must be used to ensure intramuscular delivery. The preparation and administration of the study vaccine will be performed by an unblinded site staff member.

The second CoVLP formulation dose will be administered 21 days after the first dose administration. The same procedures noted above will be followed. The second CoVLP formulation dose will be administered IM into the deltoid muscle of the alternate (if possible) arm (i.e. the arm not used for the previous dose administration). Record the administration, left or right arm, in the subject source documents.

10.1.15.1.1 Thirty Minutes Post-vaccination

The same procedures will be performed following each CoVLP formulation administration as will be performed following the administration of the study vaccine on Day 0 (see Section 10.1.4.3 for detailed procedures) for the Phase 2 and Phase 3 portions of the study, with the following exceptions:

- Non-immediate solicited local and systemic AEs (up to seven days after each vaccination) will not be collected;
- Vital signs measurements will not be collected;
- Provide appointments (date and time) for the monthly telephone calls and for the final study telephone call (Day 386);
- The subject will be released from the clinical site once all post-vaccination procedures have been completed and the subject is in stable condition.

10.1.15.2 Monthly Calls Thereafter (Telephone Contact)

Subjects should be contacted by telephone once every month (every 30 days ± 14 days; the second vaccination visit date as starting reference or last day of contact with the clinic site or Investigator if a second vaccination did not occur). The following procedures will be performed during the phone contacts:

- Ask the subjects about any change in health (AEs ongoing from 42 days after the first CoVLP formulation vaccination, SAEs, or AESIs), any visits to health care facilities, and/or medical practitioners and use of any concomitant medications. Record the information in the source documents;
- Advise the subjects to immediately contact the Investigator (or his/her designee), in the event of any AE which require a visit to the emergency and/or hospitalization;
- Remind the subjects of their next appointment (date and time) for the next telephone contact and/or the final telephone contact (Day 386);

In the event that a subject cannot be reached via telephone, he/she may be contacted by text message or via email (if these contacts are available). However, the telephone should be the initial and preferred means of communication. At least three attempts within allowed time window should be made in order to reach the subject.

10.1.15.3 Final Telephone Contact

The final telephone contact will be performed according to the procedures outlined in Section 10.1.14.2.

10.2 Safety

10.2.1 Safety Evaluations

Safety and tolerability will be evaluated by solicited local and systemic adverse events (immediate solicited adverse events within 30 minutes post-vaccination and solicited adverse events up to seven days after each vaccination), unsolicited AEs within 30 minutes post-vaccination and up to 21 days after each vaccination, SAEs, AESIs, MAAEs, and AEs leading to withdrawal up to the end of the study. In addition, events will be monitored for possible VAED, hypersensitivity components (anaphylaxis and severe allergic reactions), and potential immune-mediated diseases, from all reported events during the study (collected AEs, SAEs, AESIs, MAAEs, and AEs leading to withdrawal). In the Phase 3 portion post- RA/A, the same safety endpoints will be collected (including immediate AEs) from subjects in the placebo group who receive the CoVLP formulation with one exception: solicited local and systemic AEs (up to seven days after each vaccination) will not be collected after the administration of the CoVLP formulation.

Clinical safety methods will include repeated urine, blood chemistry, and haematology testing.

10.2.1.1 Solicited Local and Systemic Adverse Events

Subjects will be monitored for both solicited local AEs (erythema, swelling, and pain at the injection site) and solicited systemic AEs (fever, headache, fatigue, muscle aches, joint aches, chills, a feeling of general discomfort, swelling in the axilla, and swelling in the neck) from the time of each vaccination through seven days. While the subjects remain in the clinic following vaccine administration (at least 30 minutes), staff will monitor them for solicited local and systemic AEs; after release from the clinic facility, from the evening of each vaccination to the evening of the seventh day after each vaccination, the subject will measure and record the subject's local and systemic AEs in the diary. The only exception for the monitoring of solicited AEs is: solicited local and systemic AEs (up to seven days after each vaccination) will not be collected from subjects in the placebo group who receive the CoVLP formulation post-RA/A, however the immediate AEs (30 minutes after each vaccination) will still be collected.

The intensity of the solicited local and systemic AEs will be assessed by the subject, documented in the diary, and graded as: mild (1), moderate (2), severe (3) or potentially life threatening (4) (please refer to Table 9). The causal relationship of all solicited local and systemic AEs will be considered related.

The Investigator should assess solicited AEs and determine if any meet the criteria for SAE. Any solicited local or systemic AEs that meet the criteria for SAE should be reported to the Sponsor within 24 hours (Section 13.1.5) and entered as an SAE in the eCRF.

Note: Fever is also a symptom of COVID-19. Subjects should be encouraged to contact the clinical site if the subject experience fever alone or associated with other symptoms such as chills, muscle aches, or malaise within the first three days after vaccination.

Table 9 Severity Grades for Solicited Local and Systemic Adverse Events

**Symptoms**	**Severity**				
	**None**	**Grade 1 (Mild)**	**Grade 2 (Moderate)**	**Grade 3 (Severe)**	**Grade 4 (Potentially life-threatening)**
**Injection Site Adverse Events (Local Adverse Events)**					
**Erythema (redness)**	< 25 mm	25 - 50 mm	51 - 100 mm	> 100 mm	Necrosis or exfoliative dermatitis
**Swelling**	< 25 mm	25 - 50 mm and does not interfere with activity	51 – 100 mm or interferes with activity	> 100 mm or prevents daily activity	Necrosis
**Pain**	None	Does not interfere with activity	Repeated use of non-narcotic pain reliever for more than 24 hours or interferes with activity	Any use of narcotic pain reliever or prevents daily activity	Results in a visit to emergency room (ER) or hospitalization
**Solicited Systemic Adverse Events**					
**Fever (ºC or ºF)**	< 38.0 ºC<br>< 100.4 ºF	38.0 - 38.4 ºC<br>100.4 - 101.1 ºF	38.5 - 38.9 ºC<br>101.2 - 102.0 ºF	39.0 - 40.0 ºC<br>102.1 - 104.0 ºF	> 40.0 ºC<br>> 104.0 ºF
**Headache**	None	No interference with activity	Repeated use of non-narcotic pain reliever for more than 24 hours or some interference with activity	Significant; any use of narcotic pain reliever or prevents daily activity	Results in a visit to emergency room (ER) or hospitalization
**Fatigue**	None	No interference with activity	Some interference with activity	Significant; prevents daily activity	Results in a visit to emergency room (ER) or hospitalization
**Muscle aches**	None	No interference with activity	Some interference with activity	Significant; prevents daily activity	Results in a visit to emergency room (ER) or hospitalization
**Joint aches, chills, feeling of general discomfort or uneasiness (malaise), swelling in the axilla, swelling in the neck**	None	No interference with activity	Some interference with activity not requiring medical intervention	Prevents daily activity and requires medical intervention	Results in a visit to emergency room (ER) or hospitalization

10.2.1.2 Unsolicited Adverse Events

All spontaneous unsolicited AEs occurring within 21 days after each vaccination will be reported in the “Adverse Event” screen in the subject’s eCRF, irrespective of intensity or whether or not they are considered to be vaccination-related. Thereafter, from the 43rd day to the end of the

study, SAEs, AEs leading to withdrawal, MAAEs and AESIs will be monitored and reported in the eCRF.

The intensity of unsolicited AEs will be graded as: mild (1), moderate (2), severe (3) or potentially life threatening (4), according to the FDA Guidance for Industry [FDA 2007]. Their causal relationship with the study vaccine will be assessed by the Investigator (definitely not related, probably not related, possibly related, probably related or definitely related); see Section 13.1.8 for a definition of these causal relationships.

10.2.1.3 SARS-CoV-2 Positive Reports

COVID-19 cases will be continuously monitored by an unblinded team, including an unblinded Medical Monitor, to identify an adverse imbalance of COVID-19 and/or severe COVID-19 cases between subjects who receive the vaccine or placebo. Such imbalance would trigger either the stopping or alert rules (see Section 13.1.11). The unblinded team will review COVID-19 and/or severe COVID-19 cases on an ongoing basis to determine if the probability of observing the same or a more extreme case split is 5 % or less when the true incidence of severe disease is the same for the CoVLP and placebo recipients. If this criterion is met, then the unblinded team will advise the IDMC members who will review the information along with other available information and determine if the study should continue, be modified, be halted, or be stopped. The monitoring of potential imbalance of severe COVID-19 cases and pausing rule will start at a CoVLP-to-placebo case split of 5:0 for severe disease. The assessment will be performed from Study Day 0 to trial completion using a one-sided binomial test, which will provide probabilities of having an adverse imbalance between the CoVLP and placebo arms given an expected ratio of 5:1 in the Phase 2 portion and a ratio of 1:1 in the Phase 3 portion. In addition, an alert rule will be put in place and will be triggered when the probability of having imbalance in severe cases in the CoVLP arm versus placebo arm is less than 15 %. Full details of the VAED surveillance process can be found in the Medical Management Plan and IDMC Charter.

Potential COVID-19 cases will be reviewed by the unblinded medical monitor (part of the unblinded team) during the Phase 2 and Phase 3 portions of the study. Confirmed COVID-19 cases will not be recorded as AEs since these events will be collected for the planned efficacy evaluation. Also, confirmed COVID-19 cases will not be reported according to the process for reporting SAEs, even in the event such case may meet the definition of an SAE. However, any potential COVID-19 case that is not a confirmed event (i.e. does not meet the definition of the efficacy endpoints) and does meet the criteria for an SAE will be reported back to the clinical site of incidence. The unblinded medical monitor and the clinical site Investigator both must report the SAE to Medicago within 24 hours (see Section 13.1.5) of becoming or being made aware that the potential COVID-19 case was not a confirmed event. The Investigator's SAE awareness date will be the date on which the clinical site of incidence receives the SAE back from the unblinded medical monitor. Confirmed COVID-19 cases will be reported in the relevant screen (e.g. COVID-19 symptoms page) in the subject's eCRF within 1 day of reporting by the subject to the clinical site.

10.2.1.4 Clinical Laboratory Tests

Blood samples for biochemistry and hematology, HIV, Hepatitis B and Hepatitis C markers in serum and urine samples for urinalysis will be collected according to the Time and Events Schedule (see Table 1) for the Phase 2 portion of the study. Blood samples for testing for SARS-CoV-2 antibodies will be collected according to the Time and Events Schedules (see Table 1 and Table 2) for the Phase 2 and Phase 3 portions of the study. In addition, serum or urine samples from all females of childbearing potential in Study Populations #1 and #3 for pregnancy testing will be collected. Any laboratory result outside of the testing laboratory's normal range will be classified as 'clinically significant' (CS) or 'not clinically significant' (NCS) by the site Investigator, with appropriate documentation. Any laboratory test performed at Screening with a value outside the normal range may be repeated (prior to vaccination) at the discretion of the Investigator. Repeated tests will be considered the Baseline results.

The Investigator must review the laboratory reports, document this review, and record any clinically relevant changes occurring during the study in the source documents. The tests to be performed by the laboratory are presented in Table 10.

Table 10 Clinical Laboratory Tests for the Phase 2 Portion of the Study

**Biochemistry (serum):**	
Sodium	Alkaline phosphatase
Potassium	Alaninetransferase (ALT)
Urea	Aspartatetransferase (AST)
Creatinine	Gamma glutamyltransferase (GGT)
Glucose	Cholesterol (total, HDL, LDL)
Bilirubin (total)	Triglyceride
Albumin	Chloride
Total protein	Calcium
	Phosphorus
**Haematology:**	
Haemoglobin	Mean cell haemoglobin (MCH)
Hematocrit	Mean cell concentration (MCHC)
Red blood cells	Mean cell volume (MCV)
Platelets	Lymphocytes
Mean platelet volume (MPV)	Monocytes
White cell count (total, WBC)	Eosinophils
Neutrophils	Basophils

**Serology:**	
HIV<br>Hepatitis C	Hepatitis B
**Urinalysis:**	
Macroscopic examination (color, aspect)<br>pH<br>Specific gravity	Glucose<br>Protein<br>Blood

Other clinical laboratory tests include a test to measure SARS-CoV-2 antibodies at screening for all subjects in all Study Populations in the Phase 2 portion and at Day 0, Day 201 (in CoVLP formulation subjects and placebo subjects who do not want the CoVLP), and immediately prior to the administration of dose 1 of the CoVLP formulation in placebo subjects who receive the CoVLP formulation post-RA/A in the Phase 3 portion, and a serum (Phase 2 portion) or urine (Phase 3 portion; in each period) pregnancy test at screening and a urine (dipstick or similar) pregnancy test prior to randomization on Day 0 and on Days 21 and 42 for all female subjects of childbearing potential in Study Populations #1 and #3.

All protocol required safety laboratory parameters are defined in study-specific documentation.

10.2.1.5 Vital Signs

Vital signs measurements (resting BP, HR, RR, and OT) will be performed as part of screening procedures, prior to vaccination (on Day 0 and Day 21), and after the post-vaccination 30-minute surveillance period (and repeated if deemed necessary by the Investigator) during the Phase 2 portion. In addition, vital signs measurements will be performed at the clinic site visits on Day 3, Day 24, Day 42, Day 128, Day 201, and Day 386 for the Phase 2 portion of the study. Vital signs measurements will only be performed prior to each vaccination during the Phase 3 portion prior to RA/A.

OT will be collected in degrees Celsius or Fahrenheit using a digital thermometer. The OT measurement should not be collected immediately following consumption of a hot or cold beverage or after smoking.

BP will be taken after the subject has been in a seated position as per the site's standard procedure. BP should be taken by cuff (manual or automated are both acceptable). BP, HR and RR may be repeated once if judged necessary. All measurements (including any repeats) will be recorded in the source documents.

10.2.1.6 Physical Examinations

A limited physical examination will be performed by the Investigator (or sub-Investigator) as part of screening procedures. History/symptom-directed physical examinations may be performed at any other study visit if 1) new complaints or concerns are raised by either the study subject or study staff and 2) deemed to be necessary by the Investigator.

10.2.2 Pregnancy

Female subjects who become pregnant during the Phase 2 or Phase 3 portions of the study will be followed for safety. The Investigator, or his/her designee, will collect pregnancy information on any subject who becomes pregnant or is pregnant while participating in this study. The Investigator or his/her designee will record pregnancy information on the Pregnancy Report Form (for the template of the form, refer to study documentation) and submit it to the Sponsor Safety Contact (see Section 13.1.5) within 24 hours of learning of a subject's pregnancy post-first vaccination. The subject will be followed to determine the outcome of the pregnancy. At the end of the pregnancy, whether full-term or premature, information on the status of the mother and child will be forwarded to the Sponsor Safety Contact, if available. Generally, follow-up will be no longer than eight weeks following the estimated delivery date.

While pregnancy itself and elective termination of a pregnancy for non-medical reasons are not considered to be an AE/SAE, any pregnancy complication or elective termination of a pregnancy for medical reasons will be recorded in the Pregnancy Report Form or as an SAE and will be followed. A spontaneous abortion is always considered to be an SAE and will be reported as such. Furthermore, any SAE occurring as a result of a post-study pregnancy and considered reasonably related in time to receipt of the investigational product by the Investigator, will be reported to the Sponsor Safety Contact. While the Investigator is not obligated to actively seek this information from former study subjects, he/she may learn of a pregnancy through spontaneous reporting.

10.2.3 Safety Endpoints

10.2.3.1 Primary Endpoints

In the Phase 2 portion, the primary safety endpoints are:

- Occurrence, intensity, and relationship to vaccination of immediate AEs (30 minutes after each vaccination);
- Occurrence and intensity of solicited local and systemic AEs (for seven days following each vaccine administration);
- Occurrence, intensity, and relationship of unsolicited AEs for 21 days following each vaccine administration;
- Number and percentage of subjects with normal and abnormal clinically significant urine, haematological and biochemical values prior to and three days following each vaccination;
- Occurrences of SAEs, MAAEs, AEs leading to withdrawal, AESIs (including VAED), and deaths up to 21 days following each vaccine administration.

10.2.3.2 Secondary Endpoints

In the Phase 2 portion, the secondary safety endpoints are:

- Relative occurrence of solicited local and systemic AEs by intensity grades for seven days following each vaccine administration between the healthy adults (Study Population #1) and the healthy elderly population (Study Population #2, each age strata);
- Relative occurrence of solicited local and systemic AEs by intensity grades for seven days following each vaccine administration for the combination of healthy adults (Study Population #1) and the healthy elderly adults (Study Population #2) compared to adults and elderly adults with significant comorbidities (Study Population #3);
- Occurrences of SAEs, MAAEs, AEs leading to withdrawal, AESIs (including VAED), and deaths from Day 43 to Day 201;
- Occurrences of SAEs, MAAEs, AEs leading to withdrawal, AESIs (including VAED), and deaths from Day 202 to Day 386.

In the Phase 3 portion, the secondary safety endpoints are:

- Occurrence, intensity, and relationship to vaccination of immediate AEs (30 minutes after each vaccine administration);
- Occurrence and intensity of solicited local and systemic AEs (for seven days following each vaccine administration) for the initial set of vaccinations;
- Occurrence, intensity, and relationship of unsolicited AEs for 21 days following each vaccine administration;
- Occurrences of SAEs, MAAEs, AEs leading to withdrawal, AESIs (including VAED, anaphylaxis, and severe allergic reactions), and deaths up to 21 days following each vaccine administration;
- Occurrences of SAEs, MAAEs, AEs leading to withdrawal, AESIs (including VAED, anaphylaxis, and severe allergic reactions), and deaths from Day 43 to Day 201;
- Occurrences of SAEs, MAAEs, AEs leading to withdrawal, AESIs (including VAED, anaphylaxis, and severe allergic reactions), and deaths from Day 202 to Day 386.

10.2.3.3 Exploratory Endpoint

In the Phase 2 and Phase 3 portions, the exploratory safety endpoint is:

- Further characterization of the safety profile of the CoVLP formulation, if it is likely to be informative.

10.3 Immunogenicity

10.3.1 Immunogenicity Evaluations

Immunogenicity will be evaluated by the humoral immune response (Nab assay and ELISA) and the CMI response induced in subjects on Days 0, 21, 42, 128 (Nab assay and ELISA for Phase 2 only), 201, and 386 in all subjects in the Phase 2 portion and only in a subset of subjects in the Phase 3 portion (except Days 0 and 42 may be evaluated in all subjects for the Nab assay) of the study.

In the Phase 2 and Phase 3 portion, immunologic outcomes will be determined for subjects who originally receive the CoVLP formulation and placebo subjects who do not want the CoVLP formulation post-RA/A until the end of the study. These outcomes will also be determined in placebo subjects before RA/A if these subjects receive the CoVLP formulation post-RA/A.

The blood samples for immunogenicity will be analyzed in one or more central laboratories; information on processing and the central laboratories will be provided in the study-specific documentation.

10.3.2 Immunogenicity Endpoints

Point estimates and 95 % CI will be calculated for all immunogenicity endpoints and responses for the treatment groups will be compared using descriptive statistics.

10.3.2.1 Primary Endpoints

In the Phase 2 portion, the primary immunogenicity endpoints are:

- Nab response induced in each Study Population against the SARS-CoV-2 virus on Days 0, 21, and 42 will be analyzed using the following parameter: GMT, SC rate, and GMFR;
- Specific Th1 CMI response induced in each Study Population against the SARS-CoV-2 virus on Days 0, 21, and 42, as measured by IFN-γ ELISpot.

10.3.2.2 Secondary Endpoints

In the Phase 2 portion, the secondary immunogenicity endpoints are:

- Relative neutralizing antibody response between the healthy adults (Study Population #1) and the healthy elderly population (Study Population #2; each age strata) induced at 21 days after the second vaccination will be analyzed using the following parameter: GMT;
- Relative neutralizing antibody response for the combination of the healthy adults (Study Population #1) and the healthy elderly adults (Study Population #2) compared to adults and elderly adults with significant comorbidities (Study Population #3) induced at 21 days after the second vaccination will be analyzed using the following parameter: GMT;
- Persistence of the Nab antibody response induced in each Study Population against the SARS-CoV-2 virus on Day 128, Day 201, and Day 386 will be analyzed using the following parameters: GMT, SC rate, and GMFR;
- Specific antibody response induced in each Study Population against the SARS-CoV-2 virus will be measured by the total IgG levels on Days 0, 21, and 42, and the persistence of these antibodies on Days 128, 201, and 386 and analyzed using the following parameters: GMT, SC rate, and GMFR;
- The ratio of neutralizing antibody titers:IgG ELISA antibody titers at Day 21, Day 42, Day 128, Day 201, and Day 386;
- Specific Th1 CMI response induced in each Study Population against the SARS-CoV-2 virus on Day 201 and Day 386, as measured by IFN-γ ELISpot;

- Specific Th2 CMI response induced in each Study Population against the SARS-CoV-2 virus on Days 0, 21, 42, 201, and 386, as measured by IL-4 ELISpot.

In the Phase 3 portion, the secondary immunogenicity endpoints are:

- In the immunogenicity subset, Nab antibody response induced in each Study Population against the SARS-CoV-2 virus on Days 0, 21, 42, 201, and 386 analyzed, using the following parameters: GMT, SC rate, and GMFR;
- In the immunogenicity subset, specific antibody response induced in each Study Population against the SARS-CoV-2 virus measured by the total IgG levels on Days 0, 21, 42, 201, and 386, and analyzed using the following parameters: GMT, SC rate, and GMFR;
- In the immunogenicity subset, the ratio of neutralizing antibody titers:IgG ELISA antibody titers at Day 21, Day 42, Day 201, and Day 386;
- In the immunogenicity subset, specific Th1 CMI response induced in each Study Population against the SARS-CoV-2 virus on Days 0, 21, 42, 201, and 386, as measured by IFN-γ ELISpot;
- In the immunogenicity subset, specific Th2 CMI response induced in each Study Population against the SARS-CoV-2 virus on Days 0, 21, 42, 201, and 386, as measured by IL-4 ELISpot;

10.3.2.3 Exploratory Endpoint

In the Phase 2 portion, the exploratory immunogenicity endpoint is:

- Further characterization of the immune response of the CoVLP formulation, if it is likely to be informative.

In the Phase 3 portion, the exploratory immunogenicity endpoints are:

- In the immunogenicity subset, specific CMI response induced in each Study Population against the SARS-CoV-2 virus up to 21 days after the second vaccination and at Day 201 and Day 386, as measured by the percentage of CD4+ T cells expressing functional markers;
- Further characterization of the immune response of the CoVLP formulation, if it is likely to be informative.

10.4 Efficacy

10.4.1 Efficacy Evaluations

Following the first vaccination, each subject will be asked to report symptoms associated with COVID-19 from the day of the first vaccination (Day 0, post vaccination) until the end of the Phase 2 portion or the Phase 3 portion of the study (through active or passive surveillance; active surveillance in the Phase 3 portion will end once RA/A is obtained). COVID-19 will be defined as the presence of a laboratory-confirmed (virologic method) SARS-CoV-2 infection with the occurrence of a new onset of one or more of the following symptoms:

- Fever or chills;
- Cough;
- Shortness of breath or difficulty breathing;
- Fatigue;
- Muscle or body aches;
- Headache;
- New loss of taste or smell;
- Sore throat;
- Congestion or runny nose;
- Nausea or vomiting;
- Diarrhea.

Subjects will also be advised to inform the clinical site if they have tested positive for SARS-CoV-2 infection, with or without the presence of any symptoms associated with COVID-19, and will be asked to provide a documented test result. If/when the subject begins to experience symptoms associated with COVID-19, the process for the collection of symptoms and nasal or NP swabs (as noted below) will be followed.

Subjects who report any symptoms associated with COVID-19 after the start of active and passive surveillance will be requested to visit the Investigator site to provide nasal or NP swabs to test for COVID-19. During the visit, the site Investigator will confirm whether nasal or NP swabs should be collected. If the subject cannot visit the Investigator site, then the subject may receive a visit at home (or hospital, if applicable) to collect the nasal/NP swabs samples or be provided with self-administered nasal swabs for sample collection. At the visit, subjects will be trained on daily recording of their symptoms (refer to Section 19.5) using a COVID-19 diary (refer to Section 19.6) and collecting self-administered nasal swabs for testing using a virologic method. During the 7 days following each vaccination, Investigator judgement will be used to determine whether swabs should be collected from subjects reporting symptoms similar to those associated with COVID-19 since such symptoms may also be due to the reactogenicity of the study treatment administered rather than SARS-CoV-2 infection.

The swabs will be collected within 72 hours (preferably within 48 hours) of the reporting of a COVID-19-associated symptom. If swabs cannot be collected within 72 hours after reporting of the COVID-19-associated symptom, nasal or NP swabs should be collected at the next available opportunity for the subject to visit the Investigator site or at home (or hospital, if applicable).

Two swabs per event per subject will be collected and submitted for analysis using a virologic method, at the central virology laboratory. To mitigate the risk of long delays in shipping samples to the central virology laboratory, an additional local virologic test will be performed. The purpose of this supplementary test is to provide a preliminary diagnosis and permit appropriate symptom-recording and sample collection as quickly as possible.

All subjects with virologically-confirmed SARS-COV-2 infection, based on results from the locally performed testing, will be asked to provide serial (every other day) self-administered nasal swabs (2 swabs every other day for the central virology laboratory) for approximately 2

weeks. In the event, the COVID-19-associated symptoms persist for more than 2 weeks, then the Investigator's clinical judgement is required to extend the collection of swabs beyond the 2 weeks from the subject. If the COVID-19-associated symptoms have resolved in less than 2 weeks and the Investigator believes it is likely the subject has virologically recovered, then two consecutive negative results, obtained locally, are required prior to stopping the collection of the self-administered swabs for the purpose of testing at the central laboratory.

At the same visit where the Investigator will confirm whether nasal or NP swabs should be collected, subjects will be trained on daily recording of their COVID-19-associated symptoms (refer to Section 19.5) using a COVID-19 diary (refer to Section 19.6) and will be asked to immediately start recording their symptoms in the COVID-19 diary. Subjects will collect any COVID-19-associated symptoms in their COVID-19 diary until a negative swab result for the subject is received from the central virology laboratory or until the subject has no more COVID-19-associated symptoms.

The swab sample results from the central virology laboratory will be considered the primary results for vaccine efficacy evaluation. For subjects who are hospitalized with severe COVID-19 and cannot provide samples for testing at the central virology laboratory, SARS-CoV-2 testing results from the hospital may be considered for use in the evaluation of vaccine efficacy if documentation of the local testing procedures and results is available.

For the swabs sent to the central virology laboratory for testing using the virologic method, any swabs with a positive RT-PCR result (positive for the SARS-CoV-2 virus) will undergo additional testing. Direct sequencing will be used to identify the strain of the implicated virus. Information on processing and the method will be provided in the study-specific documentation.

10.4.2 Efficacy Endpoints

10.4.2.1 Primary Endpoint

In the Phase 3 portion, the primary efficacy endpoint is:

- First occurrence, in a subject, of laboratory-confirmed (virologic method) symptomatic SARS-CoV-2 infection (≥ 7 days post-second vaccination) at the time of the PVE analysis.

10.4.2.2 Secondary Endpoints

In the Phase 2 portion, the secondary efficacy endpoints are:

- First occurrence, in a subject, of laboratory-confirmed (virologic method) symptomatic SARS-CoV-2 infection (≥ 7 days post- second vaccination);
- Occurrences of severe COVID-19 disease (≥ 7 days post- second vaccination). Refer to the definition of severe COVID-19 disease in Section 2.2.

In the Phase 3 portion, the secondary efficacy endpoints are:

- Occurrences of laboratory-confirmed asymptomatic SARS-CoV-2 infection at Day 201 in subjects who were seronegative at baseline (confirmed by the ELISA method for the N protein);
- Occurrences of severe COVID-19 disease (≥ 7 days post- second vaccination) at the time of the PVE analysis. Refer to the definition of severe COVID-19 disease in Section 2.2;
- Occurrence and intensity of COVID-19-related symptoms in virologically-confirmed cases up until resolution of the symptoms in subjects administered the CoVLP formulation compared to subjects administered the placebo at the time of the PVE analysis;
- First occurrence, in a subject, of laboratory-confirmed (virologic method) symptomatic SARS-CoV-2 infection (≥ first vaccination) at the time of the PVE analysis;
- First occurrence, in a subject, of laboratory-confirmed (virologic method) symptomatic SARS-CoV-2 infection (≥ first vaccination and < second vaccination) at the time of the PVE analysis;
- First occurrence, in a subject, of laboratory-confirmed (virologic method) symptomatic SARS-CoV-2 infection (≥ second vaccination and < 7 days post-second vaccination) at the time of the PVE analysis;
- Duration and intensity of viral shedding after virologically-confirmed SARS-CoV-2 infection at the time of the PVE analysis;
- First occurrence, in a subject, of laboratory-confirmed (virologic method) symptomatic SARS-CoV-2 infection (≥ 7 days post-second vaccination) by strain at the time of the PVE analysis.

10.4.2.3 Exploratory Endpoint

In the Phase 2 portion, the exploratory efficacy endpoint is:

- Occurrence and intensity of COVID-19-related symptoms up to resolution of the symptoms.

In the Phase 3 portion, the exploratory efficacy endpoint is:

- Occurrences of severe COVID-19 disease (≥ 7 days post-second vaccination) by strain. Refer to the definition of severe COVID-19 disease in Section 2.2.

11 SUBJECT COMPLETION/WITHDRAWAL

11.1 Temporary Contraindications

An exclusion criterion that renders subjects ineligible for Phase 2 or Phase 3 portions of the study may be temporary in nature:

- Acute disease defined as presence of any moderate or severe acute illness with or without a fever within 48 hours prior to vaccination.

If, on the day of randomization, a subject is considered ineligible due to this "temporary contraindication", the subject should be considered as a screening failure. Following the resolution of such conditions, a subject may be rescreened (including the informed consent process) under a new number and, if considered eligible by the Investigator, be enrolled in the study.

11.2 Screening Failures

Screening failures are subjects who have signed the study-specific ICF but are not eligible for enrollment (subjects who were not randomized) in the Phase 2 or Phase 3 portions of the study, due to failure on one or more of the inclusion or exclusion criteria or because the subject withdrew consent prior to randomization.

Recording of screening failures documented in the study records maintained at the participating clinical sites and recorded in the IRT system. Screen failures will not receive a safety follow-up. Any subjects who are considered as a screening failure should be indicated as such. A screening failure subject can be rescreened (under a new number). If the subject is rescreened (including the informed consent process), a new subject number will be allocated.

11.3 Contraindications for Subsequent Vaccination

The following events constitute absolute contraindication to the further administration of the study treatments at Day 21 in the Phase 2 portion or Phase 3 portion:

- Pregnancy (see Section 10.2.2);
- Clinically-apparent hypersensitivity of any grade that is considered by the site Investigator to be related to the investigational product in response to first vaccine administration;
- Have a laboratory-confirmed SARS-CoV-2 infection. However, a subject may proceed with the further administration of the study treatments at least one month after the resolutions of COVID-19 symptoms;
- Acute disease within 48 hours prior to vaccination (acute disease is defined as presence of any moderate or severe acute illness with or without a fever). However, once the acute disease is resolved, then the subject may proceed with the further administration of the study treatments;
- Diagnosis of pIMD in the context of an AESI and defined according to Section 13.1.3.2.1;
- In the Phase 2 portion of the study: Experience a possibly related, probably related, or definitely related SAE or Grade 3 or higher related AE that cannot be clearly attributed to another cause. The Investigator's opinion to administer the second dose of the vaccine will be presented to the IDMC for advice;
- In the Phase 3 portion of the study: Experience a possibly related, probably related, or definitely related SAE that cannot be clearly attributed to another cause.

At the discretion of the Investigator, the following event constitutes contraindication to the further administration of the study treatments at Day 21 in the Phase 2 portion or Phase 3

portion, or to subjects in the placebo group who receive the CoVLP formulation post-RA/A in the Phase 2 portion or Phase 3 portion:

- In the Phase 2 portion of the study: Discovery of any health condition which, in the Investigator's opinion, may place the subject at increased risk from receipt of further vaccination; or discovery of a change in the subject's status which may render him/her unable to comply with protocol-mandated safety follow-up. If an AE occurred prior to or at the time of the Day 21 scheduled visit, the subject may be discontinued at the discretion of the Investigator or the subject may be vaccinated at a later date, however, within the time window specified in the protocol (see Section 10.1.8.1). The subject must be followed until resolution of the event as with any AE (see Section 13.1.2).
- In the Phase 3 portion of the study: Discovery of any health condition which, in the Investigator's opinion, may place the subject at increased risk from receipt of further vaccination; or discovery of a change in the subject's status which may render him/her unable to comply with protocol-mandated safety follow-up (e.g. if a subject experienced possibly related, probably related, or definitely related Grade 3 AE after first vaccination that lasted more than 72 hours, Investigator should carefully evaluate the risk/benefit balance prior to administering the subsequent dose). If an AE occurred prior to or at the time of the Day 21 scheduled visit, the subject may be discontinued at the discretion of the Investigator or the subject may be vaccinated at a later date, however, within the time window specified in the protocol (see Section 10.1.8.1). The subject must be followed until resolution of the event as with any AE (see Section 13.1.2).

If any of the events listed above (both lists) occur during the study, the subject will not receive an additional dose of vaccine but will continue with the Day 42 scheduled visit (see Section 10.1.11), Day 128 scheduled visit (see Section 10.1.12; Phase 2 portion only), the Day 201 scheduled visit (see Section 10.1.13), and the Day 386 scheduled visit/contact (see Section 10.1.14) of the respective portion at the discretion of the Investigator.

The following events constitute absolute contraindication to the administration of the CoVLP formulation to subjects in the placebo group who might otherwise receive the CoVLP formulation post-RA/A in the Phase 2 portion or Phase 3 portion:

- Pregnancy (see Section 10.2.2);
- Clinically-apparent hypersensitivity of any grade that is considered by the site Investigator to be related to the investigational product in response to first CoVLP formulation administration;
- Have a laboratory-confirmed SARS-CoV-2 infection. However, a subject may proceed with the further administration of the study treatments at least one month after the resolutions of COVID-19 symptoms;
- Acute disease within 48 hours prior to vaccination (acute disease is defined as presence of any moderate or severe acute illness with or without a fever). However, once the acute disease is resolved, then the subject may proceed with the further administration of the CoVLP formulation;

- Diagnosis of pIMD in the context of an AESI and defined according to Section 13.1.3.2.1;
- In the Phase 2 portion of the study: Experience a possibly related, probably related, or definitely related SAE or Grade 3 or higher related AE that cannot be clearly attributed to another cause. The Investigator's opinion to administer the second dose of the CoVLP formulation will be presented to the IDMC for advice. Note: This event will not apply to placebo recipients (prior to RA/A) who receive their first dose of the CoVLP formulation post-RA/A;
- In the Phase 3 portion of the study: Experience a possibly related, probably related, or definitely related SAE that cannot be clearly attributed to another cause. Note: This event will not apply to placebo recipients (prior to RA/A) who receive their first dose of the CoVLP formulation post-RA/A.

At the discretion of the Investigator, the following event constitutes contraindication to the administration of the CoVLP formulation to subjects in the placebo group who receive the CoVLP formulation post-RA/A in the Phase 2 portion or Phase 3 portion:

- In the Phase 2 portion of the study: Discovery of any health condition which, in the Investigator's opinion, may place the subject at increased risk from receipt of further vaccination; or discovery of a change in the subject's status which may render him/her unable to comply with protocol-mandated safety follow-up. If an AE occurred prior to or at the time of the first or second dose administration scheduled visits, the subject may be discontinued at the discretion of the Investigator or the subject may be vaccinated at a later date, however, within the time window specified in the protocol (see Section 10.1.15.1). The subject must be followed until resolution of the event as with any AE (see Section 13.1.2).
- In the Phase 3 portion of the study: Discovery of any health condition which, in the Investigator's opinion, may place the subject at increased risk from receipt of further vaccination; or discovery of a change in the subject's status which may render him/her unable to comply with protocol-mandated safety follow-up (e.g. if a subject experienced possibly related, probably related, or definitely related Grade 3 AE after first vaccination that lasted more than 72 hours, Investigator should carefully evaluate the risk/benefit balance prior to administering the subsequent dose). If an AE occurred prior to or at the time of the Day 21 scheduled visit, the subject may be discontinued at the discretion of the Investigator or the subject may be vaccinated at a later date, however, within the time window specified in the protocol (see Section 10.1.15.1). The subject must be followed until resolution of the event as with any AE (see Section 13.1.2).

If any of the events listed above (both lists) occur during the study, the subject may not receive the first and/or the second dose of the CoVLP formulation but will continue with the monthly calls (see Section 10.1.15.2) and the final telephone contact (see Section 10.1.15.3) of the respective portion at the discretion of the Investigator.

11.4 Removal of Subjects from Treatment or Assessment

Subjects will be advised that they are free to withdraw from the Phase 2 or Phase 3 portion of the study at any time without prejudice to their future medical care by the physician or the institution. Subjects who withdraw or are withdrawn from the Phase 2 or Phase 3 portion of the study after vaccination will not be replaced.

Every reasonable effort will be made to ensure that each subject complies with the protocol and completes all study visits. However, a subject may withdraw or be withdrawn from participation in the Phase 2 or Phase 3 portion of the study if:

- The subject withdraws consent;
- The subject is lost to follow-up;
- The subject is incarcerated or incapacitated during the conduct of the clinical study;
- The subject has moved away from the study area and can no longer fulfill the terms of their participation in the clinical study;
- The subject displays non-compliance to the terms of their participation in the clinical study (based on Investigator's or Medicago's [or its designee's] opinion);
- The Investigator has lost confidence in the subject's ability to adhere to the terms of their participation in the clinical study (based on Investigator's opinion);
- Safety reasons as judged by the Investigator and/or Medicago (or its designee);
- Medicago or the regulatory authority(ies) terminates the clinical study.

Withdrawal subjects will be those who leave the Phase 2 portion or Phase 3 portion of the study before Day 386, for whatever reason; withdrawal subjects will not be replaced. Any subject who withdraws consent from the study will be asked to visit the clinical site (Phase 2 portion or Phase 3 portion [immunogenicity subset]) or participate in the final telephone contact (Phase 3 portion [all subjects who are not included in the immunogenicity subset or placebo subjects in the immunogenicity subset who receive the CoVLP formulation post-RA/A]) within two weeks of withdrawal, if the subject agrees. The procedures performed for the final assessment or telephone contact will comprise of those for the Day 386 visit/telephone contact (as applicable), if permitted by the subject. In the Phase 2 or Phase 3 portion, subjects who are withdrawn from the study due to receiving an approved or authorized COVID-19 vaccine will be asked to complete the procedures for the final telephone contact at the time of unblinding and prior to receipt of the approved or authorized COVID-19 vaccine, if permitted by the subject.

All withdrawal subjects must be reported to Medicago (and/or its designee). The reason for withdrawal should be documented in the subjects' records and in the appropriate section of the eCRF. The eCRF must be completed up to and including the time of the drop-out/final assessment.

A subject may be considered discontinued from treatment if the subject does not receive both vaccine administrations (i.e. second vaccine administration is not completed; see Section 11.3 for details). A subject discontinuation from the treatment may not necessarily be discontinued from

the Phase 2 or Phase 3 portion of the study as further study procedures and follow-up may be performed (safety, immunogenicity, and efficacy), if permitted by the subject. All discontinued from treatment subjects must be reported to Medicago (and/or its designee). The reason for discontinuation from treatment should be documented in the subjects' records and in the appropriate section of the eCRF. The eCRF must be completed up to and including the time of the drop-out/final assessment.

11.4.1 Follow-up of Discontinuations

All subjects who receive a study vaccine will be followed for safety until the end of the Phase 2 or Phase 3 portion of the study, if permitted by the subject. However, subject who receives an approved or authorized COVID-19 vaccine will be asked to complete the final telephone contact at the time of unblinding and prior to receipt of the approved or authorized COVID-19 vaccine, if permitted by the subject.

11.4.2 Lost to Follow-up Procedures

Every attempt will be made to contact study subjects who are lost to follow-up. At least three contacts will be attempted and recorded in the source documents. As a last resort, one registered letter requesting contact with the site will be sent to any subject with whom the clinic staff no longer has contact. All attempts at contact will be documented in the subject's source documents.

11.5 Interruption of the Study

The Investigator (in consultation with Medicago [or its designee]), Medicago, or Regulatory Authorities may interrupt or terminate the Phase 2 or Phase 3 portions of the study for any reason. In addition, the IDMC may interrupt or halt the Phase 2 or Phase 3 portions of the study for safety reasons (refer to Section 13.1.11). The Investigator will immediately, on discontinuance of the Phase 2 or Phase 3 portions of the clinical study at the clinical site, inform both the study subjects and the Institutional Review Board (IRB) responsible for the study of the discontinuance, provide them with reasons for the discontinuance and advise them in writing of any potential health risks to the subjects themselves or to other persons. It is Medicago's (or its designee's) responsibility to report discontinuance of the Phase 2 or Phase 3 portions of the study to the local regulatory agencies within the appropriate timeframe, providing them with the reasons for the discontinuance and advising them in writing of any potential health risks to the study subjects or to other persons. Medicago (or its designee) must then inform the Investigator that the appropriate notifications were done.

11.6 Premature Unblinding Due to Availability of Approved or Authorized COVID-19 Vaccines Prior to RA/A

As discussed in Section 6.2, some subjects may request to become prematurely unblinded in order to receive an approved or authorized vaccine for COVID-19 when it becomes publicly available to them in their region. If subjects meet the criteria outlined in Section 6.2, they will be prematurely unblinded to the treatment they have received. The subject's status in the study will

be dependent on the treatment received and whether the subject actually receives the approved or authorized COVID-19 vaccine (refer to Figure 2).

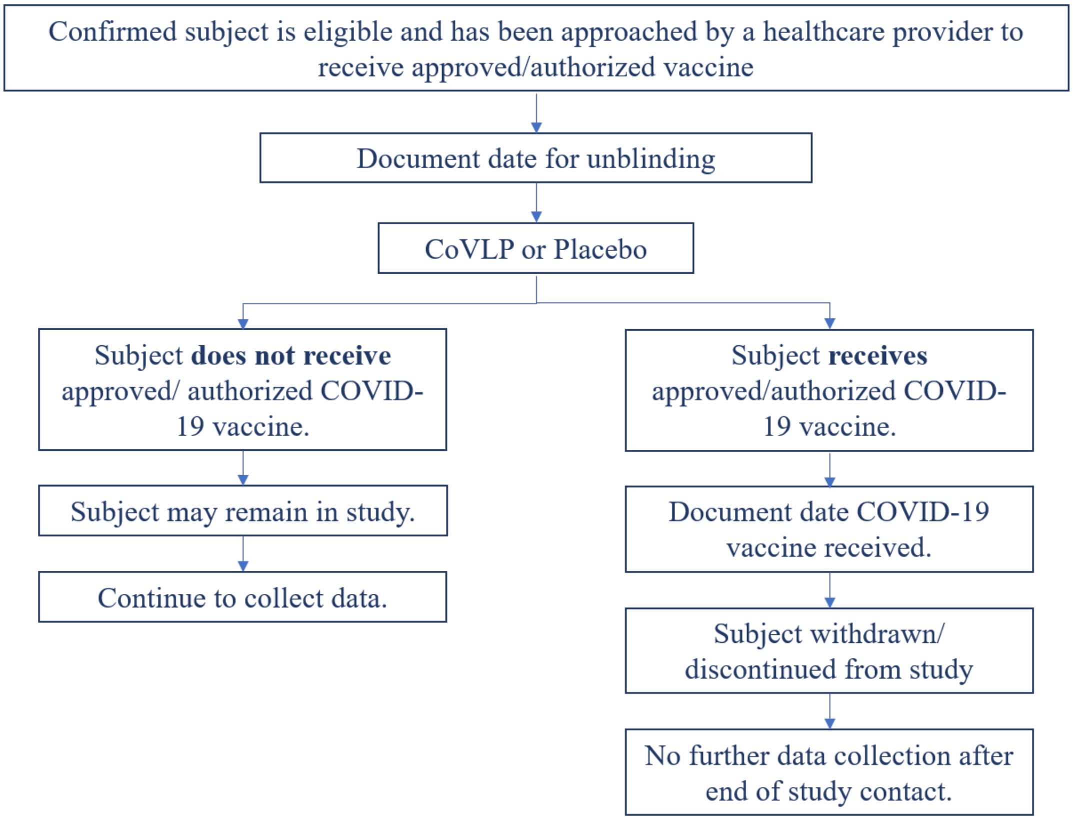

Figure 2 Decision Tree for Possible Premature Unblinding in Phase 2 and Phase 3 Portions Prior to RA/A

If the subject received the CoVLP formulation and did not receive the approved or authorized COVID-19 vaccine, then the subject may remain in the study. If the subject received placebo or received the CoVLP formulation and the approved or authorized COVID-19 vaccine, then the subject will be withdrawn/discontinued from the study. Subjects will be asked to complete the final telephone contact within two weeks of withdrawal/discontinuation, if permitted by the subject, after they have received the approved or authorized COVID-19 vaccine. The date of when the approved or authorized COVID-19 vaccine was received by the subject must be documented in the EDC.

In the Phase 3 portion, if a subject requests to be prematurely unblinded before the completion of their Day 21 visit and does not receive the approved or authorized COVID-19 vaccine, then the unblinded subject will be eligible to receive their second study vaccination at the Day 21 visit. Subjects will still be assessed for any contraindications for the second study vaccinations (as per Section 11.3).

Subject who receives the approved or authorized COVID-19 vaccine, prior to RA/A, will be asked to complete the final telephone contact at the time of unblinding and prior to receipt of the approved or authorized COVID-19 vaccine, if permitted by the subject.

12 STATISTICAL METHODS

A general description of the statistical methods used to analyze the safety and immunogenicity data is outlined below. Complete details will be provided in the SAP, which will be finalized prior to database lock.

All descriptive and inferential statistical analyses will be performed using Statistical Analysis System® (SAS®) software (version 9.4 or higher).

12.1 Analysis Populations

12.1.1 Safety Analysis Set

The SAS is defined as all subjects who received at least one dose of either the CoVLP formulation or placebo prior to RA/A. All safety analyses will be performed using the SAS and according to actual vaccination received. Safety data for subjects will be analyzed after each vaccination (Day 0-Day 21 period) according to the last vaccine they actually received, and after any vaccination (Day 0-Day 21 after last vaccination period/Day 0-End of study period) according to the actual vaccine received at the first dose.

After RA/A, safety data after each vaccination period for placebo subjects who receive the CoVLP formulation post-RA/A will be analyzed according to the last vaccine actually received and after any vaccination (Day 0-Day 21 after last vaccination period/Day 0 -End of study period) according to the actual vaccine received at first dose post-RA/A. Safety data collected after RA/A for all other subjects (i.e., those who did not receive CoVLP formulation post-RA/A and those receiving CoVLP formulation prior to RA/A) will be analyzed as described in the previous paragraph.

However, for subjects who become prematurely unblinded because they requested to be unblinded for the purposes of receiving an approved or authorized COVID-19 vaccine, their safety data will be handled according to Figure 3.

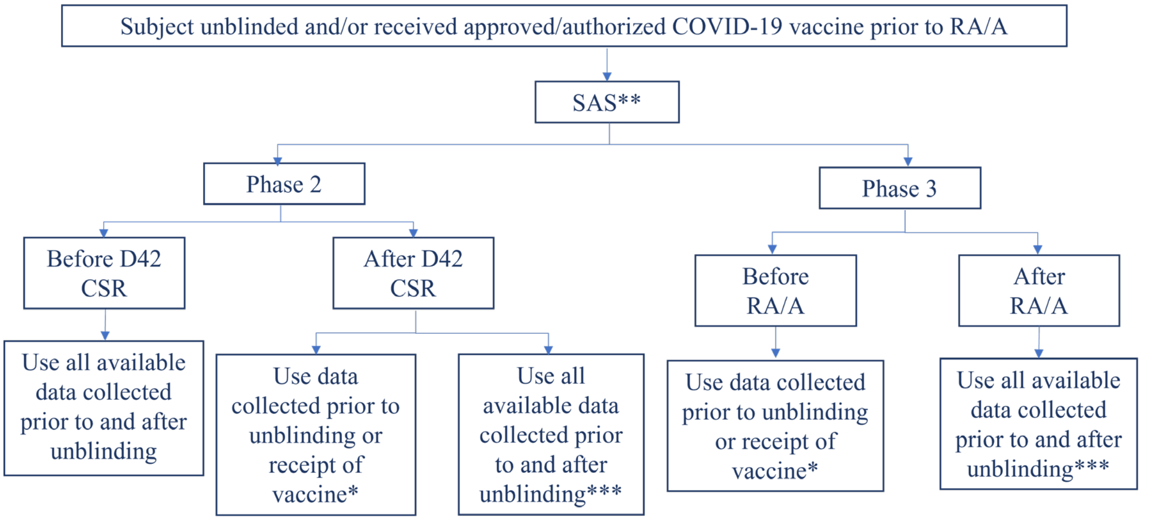

*whichever occurs first
**Subject who becomes unblinded or receives an approved or authorized COVID-19 vaccine will be included in the SAS however her/his data collected after unblinding or receipt of the vaccine, whichever comes first, will be excluded from some analyses, as noted in this flowchart.
***For subjects who receive the CoVLP formulation post-RA/A, analyses will be presented separately for data collected prior to and after RA/A.

Figure 3 Decision Tree for Handling of Safety Data from Subjects Who Request to Become Prematurely Unblinded to Receive an Approved or Authorized COVID-19 Vaccine in Phase 2 and Phase 3 Portions Prior to RA/A

For these particular subjects, the safety data collected will be analyzed in the following manner:

Phase 2 portion:

- Prior to Day 42 clinical study report (CSR):
  - Safety analyses performed using safety data collected from subjects who become unblinded because they requested to be unblinded for the purposes of receiving an approved or authorized COVID-19 vaccine will be analyzed using all available data collected prior and after unblinding;
- After Day 42 CSR:
  - Safety analyses performed using safety data collected prior to unblinding or receipt of an approved or authorized COVID-19 vaccine, which ever occurs first, from subjects in the SAS;
  - In addition, safety analyses will also be performed using safety data collected from subjects who become unblinded because they requested to be unblinded for the purposes of receiving an approved or authorized COVID-19 vaccine will be analyzed using all available data collected prior and after unblinding. For subjects who receive the CoVLP formulation post-RA/A, safety analyses will be presented separately for data collected prior to and after RA/A.

In the Phase 3 portion, the safety data collected from subjects who become unblinded because they requested to be unblinded for the purposes of receiving an approved or authorized COVID-19 vaccine will be analyzed in the following manner:

- Prior to RA/A:
  - Safety analyses performed using safety data collected prior to unblinding or receipt of an approved or authorized COVID-19 vaccine, which ever occurs first, from subjects in the SAS;
- After RA/A:
  - Safety analyses performed using safety data collected prior to and after unblinding from subjects in the SAS;
  - For subjects who receive the CoVLP formulation post-RA/A, safety analyses will be presented separately for data collected prior to and after RA/A.

12.1.2 Intention-To-Treat Set

The ITT set will consist of all subjects who were randomized in the study. Subjects who received the wrong treatment will be analyzed as randomized.

12.1.3 Per Protocol Set

The PP set will consist of a subset of the ITT set who participated in the study until the end of the surveillance period in the Phase 2 or Phase 3 portions of the study; had no major protocol deviations related to subject eligibility, the ability to develop a valid immune response, prohibited medication use, or the efficacy analyses; and who received both doses of either the CoVLP formulation or placebo. Major protocol deviations will be identified and documented during a blinded data review prior to database lock and confirmed at the time of database lock. The only exceptions for the PP set are the inclusion of subjects who become prematurely unblinded because they requested to be unblinded for the purposes of receiving an approved or authorized COVID-19 vaccine or subjects who received an approved or authorized COVID-19 vaccine (refer to Figure 4).

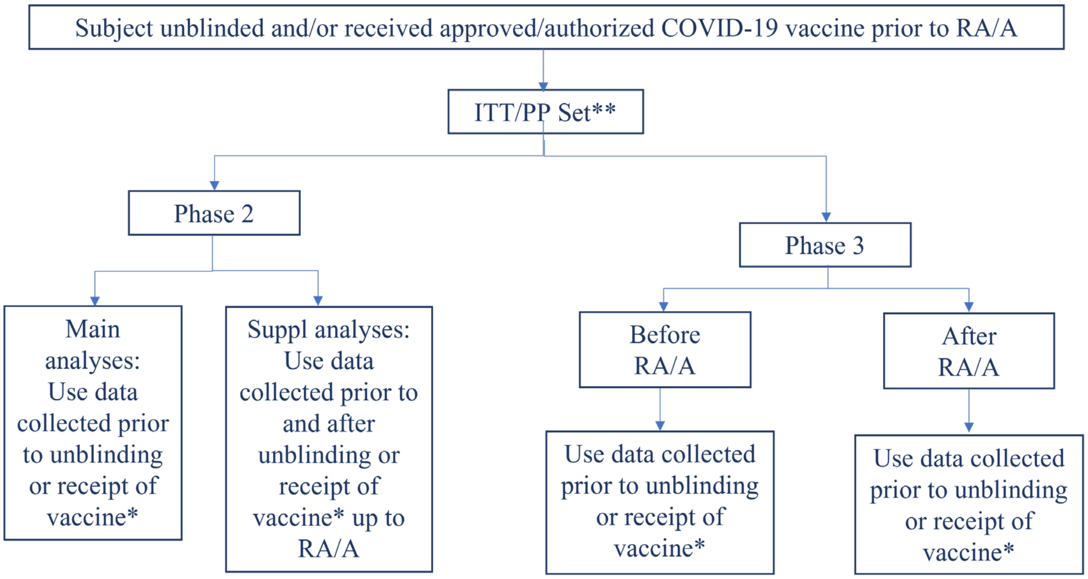

*whichever occurs first
**Subject who becomes unblinded or receives an approved or authorized COVID-19 vaccine will be included in the ITT/PP set however her/his data collected after unblinding or receipt of the vaccine, whichever comes first, will be excluded from some analyses, as noted in this flowchart.

Figure 4 Decision Tree for Handling of Efficacy Data from Subjects Who Request to Become Prematurely Unblinded to Receive an Approved or Authorized COVID-19 Vaccine in Phase 2 and Phase 3 Portions Prior to RA/A

For these particular subjects, the efficacy data collected will be analyzed in the following manner:

Phase 2 portion:

- Main efficacy analyses: efficacy analyses performed using efficacy data collected prior to unblinding or receipt of an approved or authorized COVID-19 vaccine, which ever occurs first, from subjects in the PP set and ITT set;
- Supplementary efficacy analyses: efficacy analyses performed using efficacy data collected prior to and after unblinding or receipt of an approved or authorized COVID-19 vaccine, which ever occurs first, from subjects in the PP set and ITT set up to RA/A.

Phase 3 portion:

- Prior to RA/A:
  - Efficacy analyses performed using efficacy data collected prior to unblinding or receipt of an approved or authorized COVID-19 vaccine, which ever occurs first, from subjects in the PP set and ITT set;
- After RA/A:
  - Efficacy analyses based on asymptomatic cases performed using efficacy data collected prior to unblinding or receipt of an approved or authorized COVID-19 vaccine, which ever occurs first, from subjects in the PP set and ITT set;

Subjects who received the wrong treatment, but for whom the treatment received can be unequivocally confirmed, will be analyzed as treated, provided they have no other deviations that compromise their data.

In Phase 2, the analyses of all efficacy endpoints will be performed using the PP set as primary analysis and the ITT set as a secondary analysis population.

In Phase 3, the analyses of all efficacy endpoints will be performed using the ITT set as primary analysis and the PP set as a secondary analysis population.

12.1.4 Immunogenicity Per Protocol Set

The IPP set will consist of a subset of the ITT set who participated in the immunogenicity portions of the Phase 2 or Phase 3 portions of the study and completed the study with no major deviations related to subject eligibility, the ability to develop a valid immune response, prohibited medication use, or the immunogenicity analyses; and who received either the CoVLP formulation or placebo. The only exceptions for the IPP set are the inclusion of subjects who become prematurely unblinded because they requested to be unblinded for the purposes of receiving an approved or authorized COVID-19 vaccine or subjects who received an approved or authorized COVID-19 vaccine (refer to Figure 5).

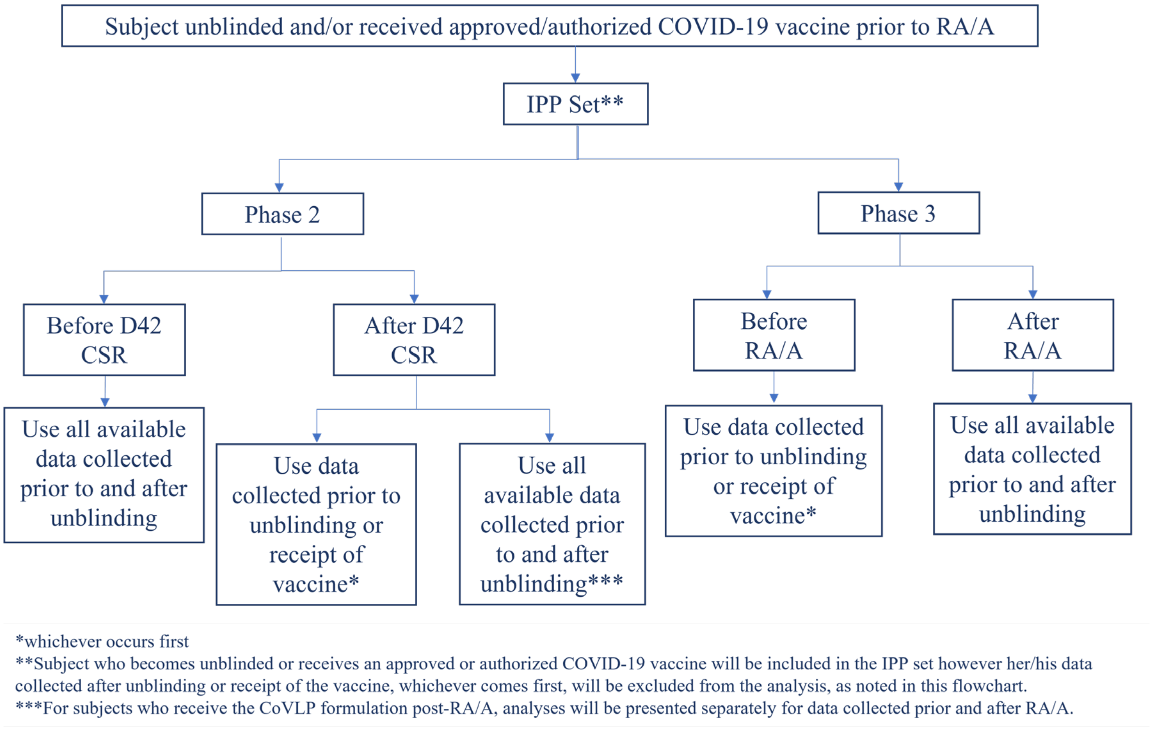

Figure 5 Decision Tree for Handling of Immunogenicity Data from Subjects Who Request to Become Prematurely Unblinded to Receive an Approved or Authorized COVID-19 Vaccine in Phase 2 and Phase 3 Portions Prior to RA/A

Phase 2 portion:

- Prior to Day 42 CSR:
  - Immunogenicity analyses performed using immunogenicity data collected from subjects who become unblinded because they requested to be unblinded for the purposes of receiving an approved or authorized COVID-19 vaccine will be analyzed using all available data collected prior and after unblinding from subjects in the ITT;
- After Day 42 CSR:
  - Immunogenicity analyses performed using immunogenicity data collected prior to unblinding or receipt of an approved or authorized COVID-19 vaccine, which ever occurs first, from subjects in the IPP/ITT;
  - In addition, immunogenicity analyses will also be performed using immunogenicity data collected from subjects who become unblinded because they requested to be unblinded for the purposes of receiving an approved or authorized COVID-19 vaccine will be analyzed using all available data collected prior and after unblinding, from subjects in the IPP/ITT. For subjects who receive the CoVLP formulation post-RA/A, immunogenicity analyses will be presented separately for data collected prior to and after RA/A.

Phase 3 portion:

For these particular subjects, the immunogenicity data collected will be analyzed in the following manner

- Prior to RA/A:
  - Immunogenicity analyses performed using immunogenicity data collected prior to unblinding or receipt of an approved or authorized COVID-19 vaccine, which ever occurs first, from subjects in the IPP/ITT;
- After RA/A:
  - Immunogenicity analyses performed using immunogenicity data collected prior to and after unblinding from subjects in the IPP/ITT.

For the Day 21 analysis, this should include the subjects who received the first vaccine dose and had Day 0 and Day 21 immunogenicity sample collections. For the Day 42 analysis, this should include the subjects who received both vaccine doses and had Day 0 and Day 42 immunogenicity sample collections. For the Day 128 analysis, this should include the subjects who received both vaccine doses and had Day 0 and Day 128 immunogenicity sample collections. For the Day 201 analysis, this should include the subjects who received both vaccine doses and had Day 0 and Day 201 immunogenicity sample collections. For the Day 386 analysis, this should include the subjects who received both vaccine doses and had Day 0 and Day 386 immunogenicity sample collections. Subjects who had blood samples for immunogenicity taken

outside of the time window for blood sample collection are to be excluded from the IPP set for the specific visit. Subjects who received the wrong treatment at the first vaccination, but for whom the treatment received can be unequivocally confirmed, will be analyzed as treated, provided they have no other deviations that compromise their data. Subjects who receive a treatment at the second vaccination that is not the same treatment received at the first vaccination will not be included in the IPP set at Day 42, Day 128, Day 201, and Day 386; the subjects will remain in the Day 21 analysis and analyzed as treated.

The analyses of all immunogenicity endpoints will be performed using the IPP set in both Phase 2 and Phase 3 portions, as the primary analysis population, and the ITT set, as a secondary analysis population.

12.2 Sample Size Determination

Overall, up to a maximum of 30 918 subjects are planned for randomization in the Phase 2 and Phase 3 portion of the study in total.

For the Phase 2 portion, the sample size of up to 918 subjects will make it possible to evaluate vaccine immunogenicity and detect sizable differences in rates of adverse events. The sample size is not large enough to detect less frequent or rare adverse reactions. With 765 subjects receiving the CoVLP formulation, there will be at least 95 % probability of observing an AE that occurs as frequently as 1 in 255 vaccinees.

For the Phase 3 portion, the sample size of up to 30 000 subjects has been calculated using an exact conditional Poisson approach based on the following assumptions:

- A 1:1 ratio for CoVLP formulation:placebo;
- Assumed vaccine efficacy of the CoVLP formulation, compared to placebo, of 60 %;
- At least 90 % statistical power to reject the null hypothesis of 1) a lower limit of the 95 % CI for a vaccine efficacy of the CoVLP formulation compared to placebo ≤ 30 % and 2) true efficacy < 50 %, assuming a vaccine efficacy of 60 %;
- A SARS-CoV-2 attack rate (i.e. symptomatic disease) in subjects who receive placebo of 0.8 % across all study populations;
- An attrition of 5 %.

With 15 000 subjects receiving the CoVLP formulation, there will be at least 95 % probability of observing an AE that occurs as frequently as 1 in 5000 vaccinees.

An immunogenicity subset sample size of 288 subjects is deemed sufficient to provide meaningful statistical assessment of immunologic outcomes.

12.3 Day 21 and Day 42 Immunogenicity Data Analysis and Efficacy and Safety Analyses

For the Phase 2 portion, the unblinded statistical team will analyze the immunogenicity data after Day 21 and Day 42 in each of the Study Populations. The data analysis will involve selected individuals from the unblinded statistical team and Medicago who will be unblinded (see Section 6.2). The Day 21 analysis in Study Population #1 and the Day 21 and/or Day 42 analysis

in Study Populations #2 and #3 will confirm whether an acceptable immune response has been induced in each Study Population prior to the initiation of the Phase 3 portion of the study (refer to Section 12.4 for details of the criteria that will be used), without having to wait until after the end of the 386 days follow-up period for study completion. These immunogenicity data, as well as the Day 28 safety data, will be provided to Regulatory Authority(ies) as required for review prior to enrolling subjects from their jurisdictions in each Study Population into the Phase 3 portion of the study.

If the Day 42 analysis in Study Population #2 or #3 confirms that an acceptable response has been induced (refer to Section 12.4 for details of the criteria that will be used at Day 42), the particular Study Population will be permitted to proceed into the Phase 3 portion of the study. However, if at Day 42, the immune response for Study Population #2 or #3 is still relatively weak compared to the healthy adult Day 42 responses, an ad hoc IDMC meeting will be convened to consider changes to the Protocol to permit alternate vaccination strategies in the particular Study Population.

The analyses performed up to Day 42 for available data for Study Populations #1, #2, and #3 will generate selected tables, listings, and figures (as applicable), by the unblinded statistical team. The results of these analyses will be used to prepare the CSR and an addendum to the CSR (up to Day 42) for the purpose of reporting to regulatory agencies the safety and immune response profile of the CoVLP formulation up to 42 days after the second vaccination. The Phase 2 results not included in the Phase 2 Day 42 CSR and addendum will be presented in a subsequent addendum to the CSR. In addition, the results included the Phase 2 Day 42 CSR and addendum will be used for publication.

For the Phase 3 portion, when at least 160 laboratory-confirmed COVID-19 cases (≥ 7 days post-second vaccination) have been collected and the median safety follow-up duration of at least 2 months (post-administration of the second vaccination) of at least 3000 subjects in each of the CoVLP formulation and placebo groups has been achieved, the unblinded statistical team will perform the final analysis and produce final efficacy outputs of the efficacy data in order to test for vaccine efficacy. If the primary efficacy success criterion is met during this analysis, RA/A will be pursued.

Upon obtaining the first RA/A in any country for the CoVLP formulation, Period 2 (Table 3) may begin to support the following:

1. Subjects in the placebo group will be offered the CoVLP formulation and will only be followed passively to identify symptomatic COVID-19 cases and for safety until the end of the study. A blood sample will be obtained immediately prior to the administration of the first dose of the CoVLP formulation for serologic analyses but no further blood samples will be collected in this group. Placebo recipients who choose to receive an approved or authorized COVID-19 vaccine for which they are eligible instead of the CoVLP formulation will be withdrawn from the study;
2. Subjects in the CoVLP formulation group will complete the study as planned with a blood draw at Day 201 to assess protection against serologically-confirmed COVID-19, unless

they decide to receive an approved or authorized COVID-19 vaccine for which they are eligible and thus will be withdrawn from the study.

The final analysis of the efficacy data to test for vaccine efficacy will produce final efficacy outputs that will be used, in addition to safety outputs, to prepare an addendum to the CSR for the purposes of reporting to regulatory agencies the efficacy and safety profile of the CoVLP formulation. The Phase 3 results that are not included in this addendum to the CSR will be presented in a subsequent addendum to the CSR.

12.4 Initiation of the Phase 3 Portion

The start of the Phase 3 portion of the study will be determined independently for each Study Population and will be based on the outcomes of the following two assessments during the Phase 2 portion of the study:

- Day 21 Data Analysis of the immunogenicity data in Study Population #1 (refer to Section 12.3);
- Day 21 and/or Day 42 Data Analysis of the immunogenicity data in Study Population #2 and Study Population #3 (refer to Section 12.3);
- Day 28 Safety Review in Study Population #1, Study Population #2, and Study Population #3 (refer to Section 13.1.11).

Study Population #1: If the safety and immunogenicity profiles of the CoVLP formulation observed in the Phase 1 study are acceptable in the Phase 2 portion, this group will proceed into the Phase 3 portion. Another key outcome will be the absence of a significant safety signal as assessed by the IDMC.

Study Populations #2 and #3: If the safety and immunogenicity profiles of the CoVLP formulation in these populations are acceptable, entry of Study Population #2 or #3 into the Phase 3 portion will be based on both the absence of an important safety signal and demonstration of an acceptable immune response as assessed by the IDMC. The criteria for acceptability will be:

- No statistically significant difference in the Nab responses at Day 42 between Study Population #1 and Study Population #2;
- No statistically significant difference in the Nab responses at Day 42 between Study Population #1 and Study Population #2 combined and Study Population #3;
- No significant higher incidence of solicited local and systemic AEs reported 7 days after each vaccine administration in Study Population #2 compared to Study Population #1;
- No significant higher incidence of solicited local and systemic AEs reported 7 days after each vaccine administration in Study Population #3 compared to Study Population #1 and Study Population #2 combined.

For Study Population #2, the same criteria will be applied to the comparisons of the two age strata for Study Population #2 versus Study Population #1. In the event of a significant difference

between Study Population #1 and one of the age strata for Study Population #2, the next highest dose level (7.5 µg CoVLP) that can be administered to this population will be assessed in the same number of subjects (as planned for the age stratum in the Phase 2 portion).

12.5 Safety Analyses

The safety endpoints are defined in Section 10.2.3.

The original terms used in the eCRFs by Investigators to identify AEs will be coded using the Medical Dictionary for Regulatory Activities (MedDRA®). All eCRF reported AEs with onset post-vaccination will be included in the safety analyses.

Special attention will be given to those subjects who die, who discontinue from the study due to an AE, who experience an SAE (e.g. summaries, listings, and narrative preparation may be provided, as appropriate), or who experience an AESI (see Section 13.1.3).

Safety summary tables will present data for the overall population and for each age stratum according to each Study Population, as applicable.

12.5.1 Analysis of Primary Endpoints

The primary safety endpoints are defined in Section 10.2.3.1.

For the Phase 2 portion, immediate AEs (30 minutes after each vaccination) and solicited local and systemic AEs reported up to seven days after each vaccination will be summarized by treatment using descriptive statistics. In addition, all unsolicited AEs, SAEs, AEs leading to subject withdrawal, AESIs, MAAEs, and deaths reported up to 21 days after each vaccination will be summarized by treatment using descriptive statistics.

Laboratory (biochemical, haematological, and urine) data will be summarized by type of laboratory test. Reference ranges and markedly abnormal results will be used in the summary of laboratory data. Descriptive statistics will be calculated for each laboratory test at Baseline and at each scheduled timepoint (Day 3, Day 21, and Day 24). Changes from Baseline results will be presented in pre- versus post-treatment cross tabulations (with classes for below, within, and above normal ranges).

12.5.2 Analysis of Secondary Endpoints

The secondary safety endpoints are defined in Section 10.2.3.2.

The incidence of solicited local and systemic AEs by intensity grades reported 7 days after each vaccine administration will be compared between Study Population #2 and Study Population #1 using Chi-square test. Each age strata in Study Population #2 will be compared separately to the Study Population #1.

The incidence of solicited local and systemic AEs by intensity grades reported 7 days after each vaccine administration will be compared between Study Population #3 and Study Population #1 and Study Population #2 combined using Chi-square test.

For the Phase 3 portion, immediate AEs (30 minutes after each vaccination) and solicited local and systemic AEs reported up to seven days after each vaccination (for the initial set of vaccinations) will be summarized by treatment using descriptive statistics. In addition, all unsolicited AEs, SAEs, AEs leading to subject withdrawal, AESIs, MAAEs, and deaths reported up to 21 days after each vaccination will be summarized by treatment using descriptive statistics.

For the Phase 2 and Phase 3 portions, SAEs, AEs leading to subject withdrawal, AESIs, MAAEs, and deaths reported from Day 43 to the end of the study will be summarized by treatment, when applicable, using descriptive statistics.

For the following categories, AE information will also be presented by gender (male and female) and race (Caucasian or White; Black or African American; Asian):

- Immediate solicited and unsolicited AEs up to 30 minutes after each vaccination;
- Solicited local and systemic AEs within first seven days after each vaccination;
- Most frequent unsolicited AEs within 21 days after vaccination;
- AESIs;
- SAEs;
- MAAEs;
- AEs leading to death;
- AEs leading to withdrawal.

12.5.3 Analysis of Exploratory Endpoint

The exploratory safety endpoint is defined in Section 10.2.3.3.

For the Phase 2 and Phase 3 portions, further characterization of the safety profile of the CoVLP formulation will be done and the results presented, if it is likely to be informative.

12.6 Immunogenicity Analyses

Immunogenicity endpoints are described in Section 10.3.2.

All descriptive and inferential statistical analyses will be performed using SAS® software (version 9.4 or higher). Details of the statistical analyses are provided in the SAP.

Immunogenicity summary tables will present data for:

- All subjects according to Study Population;
- Subjects who are seropositive at baseline for SARS-CoV-2 antibodies according to Study Population;
- Subjects who are seronegative at baseline for SARS-CoV-2 antibodies according to Study Population;
- Each of the different age strata according to Study Population, as applicable.

12.6.1 Analysis of Primary Endpoints

The primary immunogenicity endpoints are defined in Section 10.3.2.1.

For the Phase 2 portion, the following analyses for the Nab assay will be performed on the IPP set:

- GMT (Day 0, Day 21, and Day 42): The point estimates and the corresponding two-sided 95 % CI by treatment group will be calculated as the antilog of the mean and 95 % CI of log transformed titer values;
- SC rate (Day 0, Day 21, and Day 42): The point estimates and the corresponding two-sided 95 % CI for subjects achieving SC by treatment group will be calculated and reported. The SC rate is defined as the proportion of subjects achieving SC in the analysis set:
  - For subjects with detectable Nab titer at Day 0 (i.e. baseline Nab titer ≥10): a ≥ 4-fold increase in Nab titers between Day 0 and Day 21 and Day 42, respectively;
  - For subjects with undetectable Nab titer at Day 0 (i.e. baseline Nab titer < 10): Nab titer of ≥ 40 on Day 21 and Day 42, respectively;
- GMFR: the geometric mean of the ratio of GMTs (Day 21/Day 0 and Day 42/Day 0).

The GMFR will be derived by using ANCOVA to model the difference in the log of the titer values between Day 21 and Day 0 and between Day 42 and Day 0, with treatment group as main effect and baseline titer as covariate.

GMT will be compared between treatment groups using the analysis of variance (ANOVA) model. GMFR will be compared using the ANCOVA model. For SC rate, Fisher's exact tests will be used to compare between the treatment groups.

The specific Th1 CMI response induced on Day 0, Day 21, and Day 42 will be measured by the number of T cells expressing IFN-γ, using ELISpot, for each treatment group. The specific Th2 CMI response induced on Day 0, Day 21, and Day 42 will be measured by the number of T cells expressing IL-4, using ELISpot, for each treatment group. The response will be compared between treatment groups and timepoints using appropriate non-parametric (Wilcoxon) models. Additional details will be provided in the SAP.

12.6.2 Analysis of Secondary Endpoints

The secondary immunogenicity endpoints are defined in Section 10.3.2.2.

For the Phase 2 portion: the following analyses for the Nab assay will be performed on the immunogenicity PP set:

- GMT (Days 128, 201, and 386): The point estimates and the corresponding two-sided 95 % CI by treatment group will be calculated as the antilog of the mean and 95 % CI of log transformed titer values;
- SC rate (Days 128, 201, and 386): The point estimates and the corresponding two-sided 95 % CI for subjects achieving SC by treatment group will be calculated and reported;

- GMFR: the geometric mean of the ratio of GMTs (Day 128/Day 0, Day 201/Day 0, and Day 386/Day 0).

For the analyses of the total IgG antibody response, the GMT and SC rate will be calculated on Day 0, Day 21, Day 42, Day 128, Day 201, and Day 386 as well as the GMFR (Day 21/Day 0, Day 42/Day 0, Day 128/Day 0, Day 201/Day 0, and Day 386/Day 0).

The GMFR will be derived by using ANCOVA to model the difference in the log of the titer values between Day 21 and Day 0, between Day 42 and Day 0, Day 128 and Day 0, Day 201 and Day 0, and Day 386 and Day 0 with treatment group as main effect and baseline titer as covariate.

Nab GMT at Day 42 between Study Population #1 and Study Population #2 will be compared using the analysis of variance (ANOVA) model. Tukey's test will be performed to show p-values for the pairwise comparisons between Study Population #1 and each age strata in Study Population #2.

Nab GMT at Day 42 between Study Population #1 and Study Population #2 combined and Study Population #3 will be compared using the analysis of variance (ANOVA) model.

GMT will be compared between treatment groups, when applicable, using the analysis of variance (ANOVA) model. GMFR will be compared using the ANCOVA model. For SC rate, Fisher's exact tests will be used to compare between the treatment groups, when applicable.

The Nab/IgG ratio will be calculated as the antilog of the Nab/IgG ratio and 95 % CI of log transformed titer values. The ratio will be compared between treatment groups, when applicable, using ANOVA.

The specific Th1 CMI response induced on Day 201 and Day 386 will be measured by the number of T cells expressing IFN-γ, using ELISpot, for each treatment group. The specific Th2 CMI response induced on Day 201 and Day 386 will be measured by the number of T cells expressing IL-4, using ELISpot, for each treatment group. The responses will be compared between treatment groups, when applicable, and timepoints using appropriate non-parametric (Wilcoxon) models. Additional details will be provided in the SAP.

For the Phase 3 portion, the same immunogenicity analyses will be performed as described for the primary and secondary immunogenicity endpoints for the Phase 2 portion of the study (with the exception of the analysis on Day 128 and the Nab GMT comparisons between study populations). The responses will be compared between treatment groups, only when applicable.

12.6.3 Analysis of Exploratory Endpoints

The exploratory immunogenicity endpoints are defined in Section 10.3.2.3.

For the Phase 3 portion, the CMI response induced on Day 0, Day 21, Day 42, Day 201, and Day 386will be measured by the percentage of T cells expressing functional markers, using flow cytometry, for each treatment group. The responses will be compared between treatment groups,

when applicable, and timepoints using appropriate non-parametric (Wilcoxon) models. Additional details will be provided in the SAP.

For the Phase 2 and Phase 3 portions, if deemed necessary, further characterization of the immune response of the CoVLP formulation will be done and the results presented.

12.7 Efficacy Analyses

Efficacy endpoints are described in Section 10.4.2.

All descriptive and inferential statistical analyses will be performed using Statistical Analysis System® (SAS®) software (version 9.4 or higher). Details of the statistical analyses are provided in the SAP.

Efficacy summary tables will present data for the overall population and for each age stratum according to each Study Population, as applicable. In addition, efficacy summary tables will also be presented by gender, race, ethnicity and sero-status at baseline.

12.7.1 Analysis of Primary Endpoint

The primary efficacy endpoint is defined in Section 10.4.2.1.

For the Phase 3 portion of the study, once at least 160 COVID-19 cases (≥ 7 days post-second vaccination) have accumulated during the study, the primary efficacy endpoint of the Phase 3 portion of the study will be evaluated to determine if the vaccine efficacy success criterion has been met.

VE will be evaluated for any strain as the incidence rate ratio of CoVLP formulation vaccinated versus placebo subjects to develop laboratory-confirmed symptomatic SARS-CoV-2 infection ≥ 7 days after the second vaccination, as per the following formula:

$$VE=(1\text{-}IRR)=(1\text{-}(\frac{a}{N_1} \div \frac{b}{N_0}))*100\ \%$$

Where:

IRR= incidence rate ratio

a= number of subjects with at least one positive COVID-19 case; and

b= number of subjects with at least one positive COVID-19 case; and

N_1= person-years for subjects vaccinated with CoVLP; and

N_0= person-years in subjects who receive placebo.

For the ITT set analysis, the number of cases in the IRR calculation will include those occurring + 7 days after second vaccination per the definition of the primary endpoint. The follow-up time used in the calculation of person-years will be calculated from the day of randomization.

For the PP set analysis, the number of cases in the IRR calculation will also include those occurring + 7 days after second vaccination per the definition of the primary endpoint. The

follow-up time used in the calculation of person-years will be calculated from the date of second vaccination + 7 days.

For both analysis sets, censoring will be performed when subjects experience their first COVID-19 case, the date the database will be frozen for the primary analysis, the date subject is unblinded, the date subject receives an approved or authorized COVID-19 vaccine, or at the date of subject completion/withdrawal from the study, whichever occurs first. The VE success criterion for the primary efficacy endpoint, in the Phase 3 portion, is defined as a ≥ 50 % point estimate and a > 30 % lower limit of the 95 % CI.

Assuming the number of cases in each arm follows a Poisson distribution, then conditioning on the total number of cases and the ratio of person-time of follow up, the exact 95 % CIs for the incidence rate ratio IRR, and hence for VE = 1-IRR, are obtained via binomial distribution calculations [Rothman 2008].

12.7.2 Analysis of Secondary Endpoints

The secondary efficacy endpoints are defined in Section 10.4.2.2.

For the Phase 2 portion of the study, the VE will be evaluated for the secondary efficacy endpoints.

VE will be evaluated as the incidence rate ratio of CoVLP formulation vaccinated versus placebo subjects to develop laboratory-confirmed symptomatic SARS-CoV-2 infection ≥ 7 days after the second vaccination, as per the formula used for the analysis of the primary efficacy endpoint. In addition, the same formula will be used to evaluate the relative risk of CoVLP formulation vaccinated versus placebo to develop severe COVID-19 disease ≥ 7 days after the second vaccination.

For the Phase 3 portion of the study, secondary efficacy endpoints VE will be evaluated at the time of the PVE analysis. VE will be evaluated by strain as the incidence rate ratio of CoVLP formulation vaccinated versus placebo subjects to develop laboratory-confirmed symptomatic SARS-CoV-2 infection after the second vaccination. VE will also be evaluated for any strain as the incidence rate ratio of CoVLP formulation vaccinated versus placebo subjects to develop severe COVID-19 disease ≥ 7 days after the second vaccination. In addition, VE will be evaluated for any strain as the incidence rate ratio of CoVLP formulation vaccinated versus placebo subjects to develop laboratory-confirmed symptomatic SARS-CoV-2 infection after the first vaccination, after first vaccination and prior to the second vaccination, and after the second vaccination and prior to 7 days post-second vaccination. Post-RA/A, the number of symptomatic cases collected from passive surveillance will be listed and will not be summarized by treatment.

VE will be evaluated for any strain as the incidence rate ratio of CoVLP formulation vaccinated versus placebo subjects to develop laboratory-confirmed asymptomatic SARS-CoV-2 infection at Day 201 in subjects who were seronegative at baseline (confirmed by the ELISA method for the N protein). The number of cases in the CoVLP arm collected at Day 201 will be compared to the number of cases collected in the placebo arm prior to RA/A. Assuming the number of cases

in each arm follows a Poisson distribution, then conditioning on the total number of cases and the ratio of person-time of follow up, exact 95 % CIs for the incidence rate ratio IRR, and hence for VE = 1-IRR, are obtained via binomial distribution calculations [Rothman 2008].

The VE success criterion for the secondary efficacy endpoints, in the Phase 3 portion, is defined as > 0 % lower limit of the 95 % CI.

For the Phase 3 portion, at the time of the PVE analysis, the occurrences of COVID-19-related symptoms and their severity will be summarized by treatment using descriptive statistics. The occurrence of unusual manifestations of COVID-19 as well as the severity of COVID-19 symptoms will also be used to assess imbalance between the CoVLP and placebo groups as a possible indication of enhanced disease (full details provided in the Medical Management Plan and IDMC Charter).

The area under the curve of the viral titer curve, based on serial quantitative RT-PCR results, for each positive COVID-19 case will be calculated using the SARS-CoV-2 virus load (log 10 copies / mL) [Wathuo 2017]. The point estimates and the corresponding two-sided 95 % CI will be compared between treatment groups using a linear mixed model with testing dates considered as repeated measures.

The peak viral shedding will be assessed by retrieving the lowest CT value observed at any time at the laboratory for each positive COVID-19 case. Lowest CT values and the corresponding two-sided 95 % CI will be compared between treatment groups using an ANOVA.

For each positive COVID-19 case, the number of days to negative test from the start of the symptoms and the first positive test and the corresponding two-sided 95 % CI will be compared between treatment groups using an ANOVA.

Similarly to the analysis of the primary efficacy endpoint, for the ITT set analysis, the number of cases included will depend on the endpoint, and the follow-up time will be calculated from the day of randomization through censoring time. For the PP set analysis the number of cases will be adjusted according to the endpoint as well as the follow-up time.

12.7.3 Analysis of Exploratory Endpoints

The exploratory efficacy endpoints are defined in Section 10.4.2.3.

For the Phase 2 portion, the occurrences of COVID-19-related symptoms and their severity will be summarized by treatment using descriptive statistics. The occurrence of unusual manifestations of COVID-19 as well as the severity of COVID-19 symptoms will also be used to assess imbalance between the CoVLP and placebo groups as a possible indication of enhanced disease (full details provided in the Medical Management Plan and IDMC Charter).

VE will also be evaluated by strain as the incidence rate ratio of CoVLP formulation vaccinated versus placebo to develop severe COVID-19 disease ≥ 7 days after the second vaccination.

12.8 Baseline and Subject Disposition

Demographic data will be presented in listings and summarized by treatment. Continuous variables including age, weight, height, and BMI will be summarized with mean, median, standard deviation, minimum, and maximum. Count and percentage of subjects will be presented for categorical variables such as sex at birth, race, ethnicity, and baseline SARS-CoV-2 testing results.

The number of subjects in different study disposition statuses will be summarized by number and, when applicable, percentage (based on vaccinated subjects). Subjects' completion/discontinuation status will be listed (including subject identifier, date of completion/early discontinuation and, for those who discontinued early, the specific reason(s) for discontinuation).

13 ADVERSE EVENT REPORTING

Timely, accurate, and complete reporting and analysis of safety information from clinical studies are crucial for the protection of subjects, Investigators, and the Sponsor, and are mandated by regulatory agencies worldwide.

13.1 Definitions

13.1.1 Serious Adverse Events

An SAE is any untoward medical occurrence (whether or not considered to be related to the study vaccine) that, at any dose:

- Results in death;
- Is life-threatening (at the time of the event);

  Note: the term "life-threatening" in the definition of an SAE refers to an event that put the subject at risk of death at the time of the event; it does not refer to an event that hypothetically might have caused death if it were more severe;
- Requires inpatient hospitalization (≥ 24 hours) or prolongation of existing hospitalization (elective hospitalizations/procedures for pre-existing conditions that have not worsened are excluded);
- Results in persistent or significant disability/incapacity;
- Is a congenital abnormality/birth defect;
- Is another medically important event.

Medical and scientific judgment should be exercised in deciding whether expedited reporting is appropriate in other situations, such as important medical events that may not be immediately life-threatening or result in death or hospitalization but that may jeopardize the subject's health or may require an intervention to prevent one of the other outcomes listed in the definition above. These events should be considered serious. Examples of such events are intensive treatment in an emergency room for allergic bronchospasm, blood dyscrasias, or convulsions that

do not result in hospitalization or the development of drug dependency or drug abuse. See Section 13.1.5 for initial SAE reporting by the Investigator.

13.1.2 Adverse Events

An AE or adverse experience is defined as any untoward medical occurrence in a subject or clinical investigation subject who was administered a pharmaceutical product, with or without a causal relationship with the treatment. An AE can be any unfavorable and unintended sign, symptom, or disease temporally associated with the use of a medicinal product, whether or not related to a medicinal product.

Information such as the date and time of onset and resolution (duration), intensity (defined below), seriousness, any required treatment or action taken, outcome, relationship to the investigational vaccine, and whether the AE caused withdrawal from the study will be collected.

The intensity of all AEs will be graded as mild (1), moderate (2) severe (3), or potentially life threatening (4), according to the FDA Guidance for Industry: Toxicity Grading Scale for Healthy Adult and Adolescent Volunteers Enrolled in Preventive Vaccine Clinical Trials [FDA 2007] for the events covered in the guidance and the following definitions for all other events:

Mild (Grade 1):	The AE is easily tolerated and does not interfere with usual activity;
Moderate (Grade 2):	The AE interferes with daily activity, but the subject is still able to function;
Severe (Grade 3):	The AE is incapacitating and the subject is unable to work or complete usual activity;
Potentially life-threatening (Grade 4):	The AE is likely to be life-threatening if not treated in a timely manner.

Note: According to the FDA Guidance for Industry: Toxicity Grading Scale for Healthy Adult and Adolescent Volunteers Enrolled in Preventive Vaccine Clinical Trials [FDA 2007]: "*Nonetheless, we believe that categorization or grading of data as outlined in this document is supplementary to and should not replace full and complete data analysis. These guidelines for toxicity grading scales are primarily intended for healthy adult and adolescent volunteers.*" and "*Toxicity grading scales for laboratory abnormalities should consider the local laboratory reference values when the parameter limit values are defined. The characterization of laboratory parameters among some populations of healthy adults and adolescents may require the exercise of clinical judgment, for example, consideration of the potential for ethnic differences in white blood cell (WBC) counts or gender differences in creatine phosphokinase (CPK) values*."

The Investigator will be instructed to closely monitor each subject who experiences an AE (whether ascribed to the investigational product or not) until the outcome of the AE has been determined.

If any of the solicited local or systemic AEs persist beyond Day 7 after each vaccination (when applicable), these will also be recorded as unsolicited AEs. In this case, the AE start will be set as eight days post-vaccination. The subject will be requested to note when the AE resolves and to report this information to the Investigator or clinic staff at the next visit at the clinical site or contact.

The clinical importance of AEs will be determined based upon the Investigator's judgment. The Investigator must ensure that any sample obtained to follow-up on an AE is properly labeled and stored. The Investigator and others responsible for care of the subjects should institute any supplementary investigations of significant AEs based on the clinical assessment of the likely causative factor. This may include seeking the opinion of a specialist in the field of the AE.

All unsolicited AEs occurring within 21 days after each vaccination must be reported in the "Adverse Event" screen in the subject's eCRF, irrespective of intensity or whether or not they are considered to be vaccination-related. Thereafter, from Day 43 through to Day 386, SAEs, AEs leading to withdrawal, and AESIs will be monitored and reported in the eCRF. Additional details on eCRF entries can be found in the eCRF completion guidelines.

13.1.2.1 Medically Attended Adverse Events

Medically attended adverse events are defined as symptoms or illnesses requiring hospitalization, or emergency room visit, or visit to/by a health care provider.

13.1.3 Protocol-Defined Adverse Events of Special Interest

13.1.3.1 Adverse Events of Special Interest for the Coronavirus-Like Particle COVID-19 Vaccine

13.1.3.1.1 Vaccine-Associated Enhanced Disease

Safety signal of VAED after exposure to the CoVLP formulation will be closely monitored and assessed by retrieving data for this AESI as follows: AEs within the system organ class (SOC): immune system disorders and high level group term (HLGT): lower respiratory tract disorders (excluding obstruction and infection), cardiac disorders, signs and symptoms not elsewhere classified, vascular disorders, heart failures, arteriosclerosis, stenosis, vascular insufficiency and necrosis, cardiac arrhythmias, myocardial disorders, and vascular hemorrhagic disorders. High level term (HLT): renal failure and impairment and preferred term (PT): pericarditis, coagulopathy, deep vein thrombosis, pulmonary embolism, cerebrovascular accidents, peripheral ischemia, liver injury, Guillain-Barre syndrome, anosmia, ageusia, encephalitis, chilblains, vasculitis, erythema multiforme (based on standardized MedDRA® classification) [Law 2020, Law 2020a] that require inpatient hospitalization (≥ 24 hours) and have laboratory confirmed SARS-Cov-2 infection will be monitored for assessment of any potential case of VAED. As this list can be updated during the study, any further changes to the AESI terms will be updated in the Signal Management Plan (SMP).

The occurrence of unusual manifestations of COVID-19 as well as the severity of COVID-19 symptoms will be continuously reviewed by an independent and unblinded programmer and unblinded medical monitor for possible imbalance between the CoVLP and placebo groups as a possible indication of enhanced disease (full details provided in the Medical Management Plan and IDMC Charter).

13.1.3.1.2 Hypersensitivity Reactions (Anaphylaxis and Severe Allergic Reactions)

All reported events will also be monitored for hypersensitivity reactions after exposure to the CoVLP formulation. In eight clinical studies conducted to date with the Quadrivalent VLP Influenza Vaccine (QVLP) produced using similar plant-based technology, all reported events were monitored for a possible hypersensitivity component (events were searched using both narrow and broad standardized MedDRA® queries). Based on these data, there was a single case of possible early anaphylactic reaction associated with use of QVLP in humans. A small number of subjects had potential hypersensitivity reactions judged to be related to vaccine administration (no more than 0.3 % of subjects in any given QVLP treatment group experienced one of these events) and the events were distributed fairly evenly among treatment groups, including the placebo and the active comparator groups. However, since severe reactions are considered to be an important potential risk (based on the theoretical risk that using plants for the production of biotherapeutics may induce hypersensitivity), Medicago will require that appropriate medical treatment and supervision are available to manage any possible anaphylactic reactions in this study. To collect data on these events, Medicago will closely monitor and assess anaphylaxis and severe allergic reactions assessed as related to the investigational product as AESIs.

13.1.3.2 Adverse Events of Special Interest for the Adjuvants

13.1.3.2.1 Potential Immune-Mediated Diseases and Other AESI as Listed in Section 19.4

Potential immune-mediated diseases (pIMDs) are a subset of AEs that include autoimmune diseases and other inflammatory and/or neurologic disorders of interest which may or may not have an autoimmune aetiology. Adverse events that need to be recorded and reported as pIMDs include those listed in Section 19.4.

However, the Investigator will exercise their medical and scientific judgement in deciding whether other diseases have an autoimmune origin (that is pathophysiology involving systemic or organ-specific pathogenic autoantibodies) and should also be recorded as a pIMD.

When there is enough evidence to make any of the diagnoses mentioned in Section 19.4, the AE must be reported as a pIMD. Symptoms, signs or conditions which might (or might not) represent the above diagnoses, should be recorded and reported as AEs but not as pIMDs until the final or definitive diagnosis has been determined, and alternative diagnoses have been eliminated or shown to be less likely.

In order to facilitate the documentation of pIMDs in the eCRF, a pIMD standard questionnaire and a list of preferred terms (PTs) and PT codes corresponding to the above diagnoses will be available to Investigators at study start.

Once a pIMD is diagnosed (serious or non-serious) in a study subject, the Investigator (or designate) must complete, date and sign an electronic Expedited Adverse Events Report.

13.1.4 Expectedness of an Adverse Drug Reaction

An "unexpected" adverse reaction is one in which the nature or severity of the event is not consistent with information in the current version of the IB. Expedited reporting to the regulatory authorities is required if an SAE occurs that is both unexpected and considered possibly, probably, or definitely related to the study vaccine.

13.1.5 Initial SAE, AESI, and Pregnancy Reports Reporting by the Investigator

Details on safety reporting (refer to Sections 10.2.1.2 and 10.2.1.3) are defined in study-specific documentation, including SAE processing, country-specific reporting requirements, regulatory unblinding, roles and responsibilities of different stake holders, and contact details of personnel responsible for safety reporting. A brief summary of salient information is presented below.

All post-vaccination SAEs, AESIs, and pregnancy reports will be reported from the time of receiving the study vaccine on Day 0 through to the end of the study (final scheduled visit). The Investigator (or designee) must report all SAEs, AESIs (refer to Section 13.1.3), and pregnancy reports whether considered related to the study vaccine or not to Medicago within 24 hours of the Investigator learning of the event. For the Phase 2 portion of the study, the Investigator must complete, sign, and date the paper SAE/AESI report form and/or pregnancy report form, and send, via e-mail, a copy to the Medicago safety e-mail address (listed below) and the appropriate regional e-mail address, as required, within 24 hours of awareness of event.

For the Phase 3 portion of the study, the Investigator must complete the electronic SAE/AESI form in the electronic data capture (EDC) database within 24 hours of awareness of the event following the Electronic Case Report Form Completion Guidelines (eCCG). An automatic email notification will be sent to the Medicago Clinical Safety mailbox after the Investigator submits the electronic SAE/AESI form. If the EDC database is not available, the Investigator must complete, sign, and date the paper SAE/AESI report form, and send, via e-mail, a copy to the Medicago safety e-mail address (listed below) and the appropriate regional e-mail address, as required, within 24 hours of awareness of event. If the SAE/AESI paper reporting process is followed, the Investigator will need to enter the SAE /AESI information in the EDC system once available.

For the Phase 3 portion of the study, the Investigator must complete, sign, and date the paper pregnancy report form, and send, via e-mail, a copy to the Medicago clinical safety e-mail address (listed below) and the appropriate regional e-mail address within 24 hours of awareness of event.

Medicago Safety e-mail address:	Medicago-ClinicalSafety@medicago.com
Sponsor Safety Contact:	[Redacted]<br>[Redacted]

[Redacted]

Serious AEs will be reported to the local (or contract) IRB by the Investigator according to the IRB's policy and procedures.

13.1.6 Follow-up Reporting by the Investigator

All SAEs and AESIs, regardless of causality, will be followed as described in Section 13.1.2. When appropriate, documentation of any medical tests or examinations performed will be provided to document resolution/stabilization of the event.

13.1.7 Reporting of SAEs Occurring after Study Termination

All SAEs occurring during the safety follow-up period will be followed until resolution or for a period of 30 days from the final subject's visit (whichever occurs first), regardless of conclusion of the study. However, all related SAEs occurring during the safety follow-up period will be followed until resolution or stabilization.

A post-study AE/SAE is defined as any event that occurs outside of the AE/SAE detection period (post Day 386). Active follow-up for AEs or SAEs will continue until Day 386 for all subjects. However, after Day 386, if the Investigator learns of any SAE, including death, at any time after a subject has been discharged from the study, and he/she considers the event to be reasonably related to the investigational product, the Investigator will promptly notify the Sponsor Safety Contacts for Reporting SAEs. These related SAEs will be followed until resolution or stabilization.

13.1.8 Causal Relationship

The Investigator must make the determination of relationship to the study vaccine for each unsolicited AE. The causal relationship of all solicited local and systemic AEs will be considered related. The Investigator should decide whether, in his/her medical judgment, there is a reasonable possibility that the event may have been caused by the investigational vaccine. If there is any valid reason, even if undetermined or untested, for suspecting a possible cause-and-effect relationship between the investigational vaccine and the occurrence of the AE, then the AE should be considered "definitely related", "probably related", or "possibly related". Otherwise, if no valid reason exists for suggesting a possible relationship, then the AE should be classified as "probably not related" or "definitely not related". The following guidance should be followed:

Definitely Not Related:	The AE is clearly not related to the administration of the study vaccine. Another cause of the event is most plausible; and/or a clinically plausible temporal sequence is inconsistent with the onset of the event and the study vaccine administration; and/or a causal relationship is considered biologically implausible.
Probably Not Related:	There is no medical evidence to suggest that the AE is related to the study vaccine. The event can be readily explained by the subject's underlying medical condition or concomitant therapy or lacks a plausible temporal relationship to the study vaccine.

Possibly Related:	A direct cause and effect relationship between the study vaccine and the AE has not been demonstrated but there is a reasonable possibility that the event was caused by the study vaccine.
Probably Related:	There probably is a direct cause and effect relationship between the AE and the study vaccine. A plausible biologic mechanism and temporal relationship exist and there is no more likely explanation.
Definitely Related:	There is a direct cause and effect relationship between the AE and the study vaccine.

AE outcomes will be classified as recovered/resolved, not recovered/not resolved, recovered/resolved with sequelae, recovering/resolving, or death.

13.1.9 Reporting of SAEs to Health Authorities and IRB

Medicago or its designee will be responsible for reporting SAEs that are deemed both possibly related to the study vaccine and considered to be unexpected (‘unexpected’ refers to events that do not appear in the package labeling or in the study vaccine IB) to the regulatory authorities in an expedited manner.

The Investigator will be responsible for reporting the SAEs that meet IRB reporting requirements directly to the relevant ethical review board as soon as possible, and will also provide the ethical review board with any safety reports prepared by Medicago or its designee.

All SAEs that are suspected, unexpected serious adverse reactions (SUSARs) should be reported to regulatory authorities by phone or by facsimile transmission as soon as possible but, in no event, later than seven calendar days for deaths and life-threatening events, and 15 calendar days for other SAEs after Medicago's (or its designee’s) initial receipt of the information. These events should be followed to resolution, stabilization, or return to baseline, regardless of conclusion of the study.

13.1.10 Independent Data Monitoring Committee

The IDMC will consist of a multidisciplinary group of two clinicians and/or physicians and one biostatistician who will, collectively, have extensive experience in the use of vaccines for patient treatment and in the conduct and safety monitoring of randomized clinical trials.

The IDMC will monitor the study conduct and review blinded and unblinded safety data at pre-defined times during the study for the purpose of providing recommendations to the Sponsor about whether:

- subject vaccination in different Study Populations will occur;
- subject vaccination in each Study Population will continue;
- the study portion will continue in the event of a pre-defined safety signal.

The IDMC review will ensure the ongoing safety of the subjects in the study and the scientific integrity of the study. Additional information about the planned and ad hoc IDMC review meetings is available in the IDMC Charter.

13.1.11 Safety Review and Stopping Rules

The IDMC will review safety data (i.e. solicited and unsolicited safety data including laboratory safety data collected up to 7 days after each vaccination; refer to Section 19.7) for the three Study Populations in the Phase 2 portion of the study. The unblinded safety data for the first 10 sentinel subjects after each dose in all Study Populations, with the exception of Study Population #1, will be reviewed by the IDMC before permitting enrollment of the remaining subjects, according to the details outlined in the IDMC Charter.

These IDMC reviews will detect any early negative trends in the safety data from a subset of subjects vaccinated within each Study Population and may necessitate a decision to not administer the study vaccine to the remaining study subjects.

The IDMC Charter outlines the actions to be considered if any of the IDMC reviews suggest a concern about the safety of the vaccine (see Stopping Rules below) in a Study Population according to the safety review parameters.

During the Phase 2 portion, the IDMC will review the safety data collected up to Day 28 from all the subjects in each Study Population to confirm that each Study Population can be included in the Phase 3 portion of the study. These safety data, as well as the Day 21 and Day 42 immunogenicity data, will be provided to Regulatory Authority(ies) as required for review prior to enrolling subjects from their jurisdictions in each Study Population into the Phase 3 study.

Stopping Rules

Safety monitoring of safety signals will be performed for each of the Study Populations throughout the Phase 2 and Phase 3 portions of the study. Stopping rules or conditions for stopping each portion of the study would occur if there was clear evidence of harm or harmful effects such as SAEs related to the treatment (study vaccine). An SAE which is assessed as unrelated to the study vaccine would not warrant stopping the trial.

For sentinel subjects in Study Populations #2 and #3 during the Phase 2 portion of the study:

If any of the following events occur to the sentinel subjects, the study enrollment and vaccinations will be paused based on IDMC recommendations:

- Any SAE in a subject for which causality cannot be attributed to another cause;
- Two (2) or more subjects who receive the CoVLP formulation experience the same or similar AE:
  - Experience the same or similar Grade 3 or higher solicited local AE or systemic AE which began within 7 days after administration of the vaccine;
  - Experience the same or similar Grade 3 or higher unsolicited AE (including symptoms, signs or laboratory safety AEs) that is judged anything but unrelated to the vaccine.

After vaccination of all sentinel subjects in Phase 2, the stopping rules for the remainder of the Phase 2 and the Phase 3 portions of the study:

The study will be paused based on IDMC recommendations if any of the following events occur:

- Any subject experiences an SAE after administration of the vaccine that is considered related to vaccine;
- If 5 % or more subjects who receive the CoVLP formulation experience the same or similar AE:
  - Experience the same or similar Grade 3 or higher solicited local AE or systemic AE, which began within 7 days after administration of the vaccine;
  - Experience the same or similar Grade 3 or higher unsolicited AE (including symptoms, signs or laboratory safety AEs) that is judged anything but unrelated to the vaccine;
- If an important imbalance in unusual manifestations of COVID-19 or severity of COVID-19 symptoms is observed between the CoVLP and placebo groups. The unblinded team will review COVID-19 and/or severe COVID-19 cases on an ongoing basis to determine if the probability of observing the same or a more extreme case split is 5 % or less when the true incidence of severe disease is the same for the CoVLP and placebo recipients. If this criterion is met, then the unblinded team will advise the IDMC members who will review the information along with other available information and determine if the study should continue, be modified, be halted, or be stopped. The monitoring of potential imbalance of severe COVID-19 cases and pausing rule will start at a CoVLP-to-placebo case split of 5:0 for severe disease. The assessment will be performed from Study Day 0 to trial completion using a one-sided binomial test, which will provide probabilities of having an adverse imbalance between the CoVLP and placebo arms given an expected ratio of 5:1 in the Phase 2 portion and a ratio of 1:1 in the Phase 3 portion. In addition, an alert rule will be put in place and will be triggered when the probability of having imbalance in severe cases in the CoVLP arm versus placebo arm is less than 15 %.

If any of the stopping rules are met, the Sponsor will seek the guidance of the IDMC according to the process outlined in the IDMC Charter.

14 INVESTIGATIONAL PRODUCT INFORMATION

14.1 Identity of Investigational Product

The CoVLP formulation is the CoVLP vaccine composed of recombinant spike (S) glycoproteins expressed as VLPs and administered with the adjuvant AS03.

For more information regarding the CoVLP formulation, refer to the current IB.

14.1.1 Study Vaccine Composition

The CoVLP vaccine is a sterile transparent to opalescent, colorless to yellowish liquid suspension that may contain a small number of visible white particulates. The CoVLP vaccine

will be supplied in 0.35 mL single dose vials in the Phase 2 portion and in 3.1 ml multidose vials (10 doses/vial) in the Phase 3 portion. The S glycoprotein concentration in the vaccine will be 15 µg/mL for a final dosage of 3.75 µg.

AS03 is an established effective adjuvant licensed for Arepanrix™ H1N1 and Arepanrix™ H5N1 (in Canada). The adjuvant is a whitish to yellowish homogeneous milky liquid emulsion and it will be provided in the original sterile 3.0 ml multidose (10 doses/vial) vial container received from the manufacturer (GlaxoSmithKline). A 0.25 mL dose of AS03 represents one human dose.

Further specific information relating to investigational product storage, shipment and treatment preparation is provided in the Investigational Product Management Manual (IPMM).

14.1.2 Placebo

The control product (placebo) will be composed of a phosphate-buffered saline (PBS) solution (100 mM $NaKPO_4$, 150 mM NaCl) and 0.01 % polysorbate 80 at pH 7.4. The control product will be provided in 6.2 mL multidose vials.

Further specific information relating to the investigational product storage and shipment is provided in the IPMM.

14.1.3 Preparation and Administration of Study Vaccine

The study treatments will be prepared by unblinded staff members at the clinical site as described in the Investigational Product Management Manual. The prepared study treatment will subsequently be administered to subjects by an unblinded staff member. The unblinded staff members must not be involved in the evaluation of any AEs or reactogenicity evaluations following vaccination; a blinded staff member must be present for post-vaccination observation.

The products to be used for study treatment administration will be handled in an aseptic manner.

During the Phase 2 portion and the Phase 3 portion, the CoVLP formulation will be administered on study Day 0 and Day 21 as an IM injection of 0.5 mL, into the deltoid muscle. For subjects with BMI < 30 kg/m^2, a 23 gauge needle of at least one inch or 2.5 cm in length can be used for vaccination. For subjects with BMI ≥ 30 kg/m^2, a needle of at least 1.5 inches or 3.5 cm in length must be used to ensure intramuscular delivery. In addition, subjects in the placebo group will be offered the CoVLP formulation once RA/A for the CoVLP formulation is granted.

The product administered will be recorded in study-specific documentation (refer to IPMM) by the clinical site, which will be separate from the study medication record for drug preparation. After drug accountability monitoring and reconciliation has been completed by the site and unblinded monitor, all study treatments (used and unused vials) will be destroyed locally upon Medicago's approval or returned to Medicago (or its designee) in accordance with instructions provided in the IPMM.

Further specific information relating to investigational product storage, shipment and treatment preparation is provided in the IPMM.

14.1.4 Preparation, Handling, Storage, and Precautions for Use

The investigational products should be stored in an access-restricted area between 2 ºC and 8 ºC and protected from light; the vaccine should, however, be at room temperature before administration (i.e. the vaccine should not be administered cold and should be taken out of the refrigerator prior to administration [refer to IPMM for details regarding time allowance]).

The IPMM provides additional details on treatment preparation, handling and storage of the investigational product.

14.2 Packaging

The CoVLP vaccine, AS03, and placebo will be packaged in separate boxes containing multiple vials of each investigational product.

14.3 Labeling

The investigational product vials will have a product and study-specific label containing information that meets the applicable regulatory requirements. It is not possible to have a matching placebo in this study, therefore the vial labels will not be blinded. The study treatment will be prepared and administered by trained unblinded staff. Blinding measures will be applied to maintain the observer-blindness of the blinded staff.

14.4 Drug Accountability

The Investigator is responsible for ensuring that all study treatments received at the site are inventoried and accounted for throughout the study. The dispensing of study treatment to each subject must be documented on study-specific documentation.

The investigational products must be handled in strict accordance with the IPMM and the vial label. The investigational products will be stored in a limited access area or in a locked cabinet under appropriate environmental conditions. The unblinded site staff will complete accountability for all investigational products (CoVLP, AS03, and placebo). Refer to the IPMM for details on the disposition of the used and unused vials.

The study drug should be dispensed under the supervision of the Investigator (or a qualified member of the investigational staff), who remains blinded as to which treatment is administered. The study drug will be supplied only to subjects participating in the study. Returned study drugs may not be relabeled or reassigned for use for other subjects. The Investigator agrees neither to dispense the study drug from, nor store it at, any site other than the study centers agreed upon with Medicago (or its designee).

15 STUDY-SPECIFIC MATERIALS

The Investigator will be provided with the following:

- eCRF;
- Forms and questionnaires for special assessments;

- Thermometers and measurements template;
- Study manuals.

16 ETHICAL ASPECTS

16.1 Study-Specific Design Considerations

On-going blinded medical review will be performed by Medicago (or its designee) throughout the duration of the Phase 2 and Phase 3 portions of the study; subjects will be given any new information that may affect their decision to continue participation in the study.

Blood samples will be collected from subjects for immunogenicity analysis, although not all will be analysed immediately. Subject consent will be obtained for the use of any leftover samples. Since blood sample collection is a minimally invasive procedure and the amount of blood collected will be a relatively small amount (Table 7 and Table 8), the benefits of obtaining this data outweigh the small level of risk associated with the collection of samples that may not require analysis.

16.2 Regulatory Ethics Compliance

16.2.1 Investigator Responsibilities

The Investigator is responsible for ensuring that the study is performed in accordance with the protocol, current International Conference on Harmonisation (ICH) guidelines on Good Clinical Practice (GCP), and applicable regulatory and country-specific requirements.

GCP is an international ethical and scientific quality standard for designing, conducting, recording, and reporting studies that involve the participation of human subjects. Compliance with this standard provides public assurance that the rights, safety, and well-being of study subjects are protected, consistent with the principles that originated in the Declaration of Helsinki, and that the study data are reliable.

16.2.2 Independent Ethics Committee or Institutional Review Board

Before the start of the study, the Investigator (or Medicago, where required) will provide the Independent Ethics Committee (IEC)/IRB with current and complete copies of the following documents (as required by local regulations):

- Final protocol and, if applicable, amendments to the protocol;
- Sponsor-approved ICF (and any other written materials to be provided to the subjects);
- IB (or equivalent information) and amendments/addenda;
- Information on compensation for study-related injuries or payments to subjects for participation in the study, if applicable;
- Investigator's *curriculum vitae* or equivalent information (unless not required, as documented by the IEC/IRB);

- If applicable, any Sponsor-approved subject recruiting materials;
- Information regarding funding, name of the sponsor, institutional affiliations, other potential conflicts of interest, and incentives for subjects;
- Any other documents that the IEC/IRB requests to fulfill its obligation.

This study will be undertaken only after the IEC/IRB has given full approval of the final protocol, amendments (if any, excluding the ones that are purely administrative in nature, with no consequences for subjects, data, or study conduct), the ICF, applicable recruiting materials, and subject compensation programs, and Medicago (or its designee) has received a copy of this approval. This approval letter must be dated and must clearly identify the IEC/IRB and the documents being approved. In addition, the Investigator must wait for written confirmation from Medicago (or its designee) that the study can be started.

During the study, the Investigator (or Medicago [or its designee], where required) will send the following documents and updates to the IEC/IRB for their review and approval, where appropriate:

- Protocol amendments;
- Revision(s) to ICF and any other written materials to be provided to subjects;
- If applicable, new or revised subject recruiting materials approved by Medicago;
- Revisions to compensation for study-related injuries or payment to subjects for participation in the study, if applicable;
- New edition(s) of the IB and amendments/addenda;
- Summaries of the study at intervals stipulated in guidelines of the IEC/IRB (at a minimum, annually);
- Reports of AEs that are serious, unlisted/unexpected, and associated with the study drug (when applicable);
- New information that may adversely affect the safety of the subjects or the conduct of the study;
- Deviations from or changes to the protocol to eliminate immediate hazards to subjects;
- Reports of deaths of subjects under the Investigator’s care;
- Notification if a new Investigator is responsible for the study at any of the sites;
- Development Safety Update Report and Line Listings, where applicable;
- Any other requirements of the IEC/IRB.

At least once a year, the IEC/IRB will be asked to review and reapprove this study. The re-approval should be documented in writing. At the end of the study, the Investigator (or Medicago [or its designee], where required) will notify the IEC/IRB of study completion.

16.2.3 Informed Consent

Each subject who participates in the study must first give written consent according to local requirements after the nature of the study has been fully explained, including the risks and requirements of the study. The consent form must be signed before performing any study-related activity. The consent form that is used must have been approved by regulatory authorities, Medicago, and the reviewing IEC/IRB. The informed consent should be in accordance with principles that originated in the Declaration of Helsinki, current ICH and GCP guidelines, applicable regulatory requirements, and Medicago policy. During the study, subjects will be given any new information that may affect their decision to continue their participation in the study. They will be told that their consent to participate is voluntary and that it may be withdrawn at any time, with no reason given, and without penalty or loss of benefits to which they would otherwise be entitled. Only subjects who are fully able to understand the risks, benefits, and potential AEs of the study and who provide their consent voluntarily will be enrolled.

16.2.4 Privacy of Personal Data

The collection and processing of personal data from subjects enrolled in this study will be limited to those data that are necessary to support the development, registration, and future marketing of the investigational product. The data must be collected and processed with adequate precautions to ensure confidentiality and compliance with applicable data privacy protection laws and regulations. Appropriate technical and organizational measures to protect the personal data against unauthorized disclosures or access, accidental or unlawful destruction, or accidental loss or alteration must be in place. Medicago (or its designee) personnel whose responsibilities require access to personal data agree to keep the identity of study subjects confidential.

17 ADMINISTRATIVE REQUIREMENTS

17.1 Protocol Amendments

Neither the Investigator nor Medicago will modify this protocol without a formal amendment. All protocol amendments must be issued and approved by Medicago, and signed and dated by the Investigator. Protocol amendments must not be implemented without prior IEC/IRB approval or when the relevant competent authority has raised any grounds for non-acceptance, except when necessary in order to eliminate an immediate hazard to the subjects, in which case the amendment must be promptly submitted to the IEC/IRB and relevant competent authority. Documentation of amendment approval by the Investigator and IEC/IRB must be provided to Medicago or its designee.

Note that administrative changes may be implemented without prior IRB approval; however, the Investigator or Medicago, as applicable, must notify the IRB of any administrative change and ensure that IRB acknowledges receipt of the administrative changes.

The Investigator is responsible for notifying the IRB of all protocol amendments and ensuring that the IRB has approved any amendments when local IRBs are used. When a central IRB is used, Medicago or its designee will inform the IRB on behalf of the sites.

During the course of the study, in situations where a departure from the protocol is unavoidable, the Investigator or other physician in attendance will contact the appropriate Medicago representative or its designee (see Contact Information pages provided separately). Except in emergency situations, this contact should be made before implementing any departure from the protocol. In all cases, contact with Medicago or its designee must be made as soon as possible to discuss the situation and to agree on an appropriate course of action. The data recorded in the eCRF and source documents will reflect any departure from the protocol, and the source documents will describe this departure and the circumstances that made a deviation necessary.

17.2 Regulatory Documentation

17.2.1 Regulatory Approval/Notification

This protocol and any amendment(s) must be submitted to the appropriate regulatory authorities in each respective country, as applicable. A study may not be initiated until all local regulatory requirements are met.

17.3 Source Documentation

At a minimum, source documentation must be available for the following information:

- Subject identification, eligibility, and study identification;
- ICF discussion and date of informed consent;
- Dates of visits;
- Results of safety and study procedures as required by the protocol;
- Record of all reactions, AEs, and associated follow-ups;
- Concomitant medication;
- Drug receipt, dispensing, and return records;
- Study drug preparation and administration information;
- Date of study completion and reason for early discontinuation of study drug or withdrawal from the study, if applicable.

It is recommended that the author of an entry in the source documents be identifiable.

At a minimum, the type and level of detail of source data available for a study subject should be consistent with that commonly recorded at the site as a basis for standard medical care. If Investigator judgement was used in the determination of eligibility, an explanation for inclusion of the subject in the study must be provided in the source documents. Specific details required as source data for the study will be reviewed with the Investigator before the study and will be described in the monitoring guidelines (or other equivalent document).

17.3.1 Diary and Memory Aid

Subjects will be provided with a paper or electronic diary or application in which to record solicited AEs, COVID-19 symptoms, and other safety information. For example, the electronic diary may be configured to transmit images of the memory aid to sites. The electronic diary may also be used for the active weekly surveillance for COVID-19 symptoms and daily symptoms for cases of COVID-19. Subject diaries are considered source documents. During the first 8 days after each vaccination in the Phase 3 portion, if data entry in the electronic diary is missed for 2 consecutive days by a subject then the subject will receive a follow-up contact (phone, text, or email) to remind him/her to record his/her data in the electronic diary.

Subjects will be provided with memory aids to record information on unsolicited AEs, SAEs, AEs leading to withdrawal, AESIs, and reportable concomitant medications from Day 0 to Day 42, Day 43 to Day 201, and Day 202 to the end of the study (Day 386) in the Phase 2 portion and the Phase 3 portion. These memory aids will be collected by the site and are intended to be used by the subjects to help them in reporting this information to site staff during phone contacts and clinical site visits.

17.4 Case Report Form Completion

An eCRF will be provided for each subject who is randomized and receives a dose of study drug. Screening failures will also be entered in an eCRF; data entered should include date of birth and reason for screening failure.

Data must be entered into the eCRFs in English. The Investigator must verify that all data entries in the eCRFs are accurate and correct. All eCRF entries, corrections, and alterations must be made by the Investigator or other authorized study-site personnel.

Please refer to the eCRF completion guidelines for details of data entry requirements.

17.5 Quality Control (Study Monitoring)

In accordance with applicable regulations, GCP, and Medicago (or its designee) procedures, the site will be contacted prior to the study start to review the protocol and study requirements with the clinical study staff. Medicago (or its designee) will review their responsibilities to satisfy regulatory, ethical, and company (Medicago) requirements.

The Investigator or institution, if applicable, will authorize and provide direct access to all relevant documents to allow review of the appropriate conduct of the study by Medicago’s (or its designee’s) monitor. The Investigator(s) must allocate their time and the time of their staff to the monitors, discuss any findings or issues, and take appropriate actions to maintain the quality and integrity of the study data and conduct.

Protocol deviations will be reviewed by Medicago (or its designee) to identify any non-compliances likely to have a significant effect on the safety and rights of a subject or the reliability and robustness of the data generated. These deviations will be included in the clinical study report.

17.6 Quality Assurance

To ensure compliance with GCP and all other applicable regulatory requirements, Medicago (or its designee) may conduct quality assurance assessments and/or an audit of the site. Regulatory agencies may conduct regulatory inspections at any time during or after completion of the study. If an audit or an inspection is conducted, the Investigator and/or institution (if applicable) must agree to grant auditors or inspectors direct access to all relevant documents and to allocate their time and the time of their staff to discuss the conduct of the study and any findings or issues, and to implement corrective and/or preventative actions to address any findings or issues identified.

17.7 Record Retention

Following closure of the study, the Investigator must maintain all site study records in a safe and secure location. The records must be easily accessible when needed (e.g. for a Medicago [or its designee] audit or regulatory inspection) and must be available for review in conjunction with assessment of the facility, supporting systems, and relevant site staff.

When permitted by local laws and regulations or institutional policy, some or all of the records may be maintained in a format other than a hard copy (e.g. scanned electronic). The Investigator must ensure that all reproductions are legible and are a true and accurate copy of the original.

The Investigator must follow the time period for retaining the site records in order to comply with all applicable regulatory requirements. The minimum retention time will meet the strictest standard applicable to a particular site, as dictated by local laws and regulations, Medicago (or its designee) standard operating procedures (SOPs), and/or institutional requirements. The Investigator must contact Medicago (or its designee) prior to the disposal of any study documents at the end of the retention period.

If the responsible Investigator retires, relocates, or, for any other reason, withdraws from the responsibility of keeping the study records, custody must be transferred to another qualified person who will accept the responsibility and is approved by Medicago. Medicago (or its designee) must be notified in writing of the name and address of the new custodian. Under no circumstance shall the Investigator relocate or dispose of any study documents without having obtained prior written approval from Medicago (or its designee).

If it becomes necessary for Medicago or the appropriate regulatory authority to review any documentation relating to this study, the Investigator (or new custodian) must permit access to such reports.

17.8 Study Completion/Termination

17.8.1 Study Completion

Each portion of the study will be considered to be completed with the last contact with the last subject participating in the particular portion of the study. The final data from the investigational site will be sent to Medicago (or designee) after completion of the final subject visit at that site. Investigational sites will be closed after the portion completion. An investigational site is

considered closed when all required documents and study supplies have been collected, all data have been entered, monitored, locked, and signed and a site closure visit has been performed.

17.8.2 Study Termination

Medicago (or its designee) reserves the right to close the investigational site or terminate the study at any time for any reason at the sole discretion of Medicago. Investigational sites will be closed after study termination once Medicago (or its designee) has completed all study related tasks. Reasons for the early closure of an investigational site by Medicago (or its designee) or the Investigator may include, but are not limited to:

- Failure of the Investigator to comply with the protocol, Medicago's (or its designee's) procedures, or GCP guidelines;
- Inadequate recruitment of subjects by the Investigator;
- Discontinuation of further drug development.

The Investigator may initiate site closure at any time, provided there is reasonable cause and sufficient notice is given in advance of the intended closure.

Site closure following an Investigator request can only be performed when all active study subjects have completed the study period, Medicago (or its designee) has collected all required documents and study supplies, and all data have been entered, monitored and locked.

17.9 Registration of Clinical Studies and Disclosure of Results

Study information from this study will be posted on publicly available clinical trial registries in countries where applicable and will include information required by law. In addition, the results summary will be posted to the same clinical trial registries, to the extent specified by law, and will include information required by regulatory authorities.

17.10 Publication Policy

The data derived from this study are the property of Medicago and cannot be published without prior authorization from Medicago. Any publication activities (i.e. preparation and submission of abstracts and manuscripts) will be at the discretion of Medicago.

Any proposed publication regarding this study, not prepared by Medicago personnel, must be provided to Medicago for comments and review at least 45 days prior to its intended publication. The proposed publication shall not include any confidential information or protected information to preserve Intellectual Property rights; any such information must be removed from the proposed publication.

17.10.1 Publication Release of Results

As clinical data becomes available in the Phase 2 and Phase 3 portions, Medicago's intent is to release and/or publish this data after the clinical data have been presented to Regulatory Authority(ies). There may be multiple clinical data releases and/or publications generated over

the course of the Phase 2 and Phase 3 portions as the clinical data are presented to Regulatory Authority(ies). Moreover, the clinical data that are released and/or published will only present group level results and will not involve any unblinding or the presentation of subject level data.

18 REFERENCES

Ahn DG, Shin HJ, Kim MH, Lee S, Kim HS, Myoung J, Kim BT, Kim SJ. Current status of epidemiology, diagnosis, therapeutics, and vaccines for novel coronavirus disease 2019 (COVID-19). J Microbiol Biotechnol. 2020 Mar 28;30(3):313-324. doi: 10.4014/jmb.2003.03011.

Baz M, Luke CJ, Cheng X, Jin H, Subbarao K. H5N1 vaccines in humans. Virus Res 2013;178:78-98. doi: 10.1016/j.virusres.2013.05.006.

Bektas A, Schurman SH, Sen R, Ferrucci L. Human T cell immunosenescence and inflammation in aging. J Leukoc Biol. 2017 Oct;102(4):977-988. doi: 10.1189/jlb.3RI0716-335R.

CDC COVID-19 Response Team. Severe Outcomes Among Patients with Coronavirus Disease 2019 (COVID-19) - United States, February 12-March 16, 2020. MMWR Morb Mortal Wkly Rep. 2020 Mar 27;69(12):343-346. doi: 10.15585/mmwr.mm6912e2.

Channappanavar R, Zhao J, Perlman S. T cell-mediated immune response to respiratory coronaviruses. Immunol Res. 2014 Aug;59(1-3):118-28. doi: 10.1007/s12026-014-8534-z.

Chen N, Zhou M, Dong X, Qu J, Gong F, Han Y, Qiu Y, Wang J, Liu Y, Wei Y, Xia J, Yu T, Zhang X, Zhang L. Epidemiological and clinical characteristics of 99 cases of 2019 novel coronavirus pneumonia in Wuhan, China: a descriptive study. Lancet. 2020 Feb 15;395(10223):507-513. doi: 10.1016/S0140-6736(20)30211-7.

Chen Y, Liu Q, Guo D. Emerging coronaviruses: Genome structure, replication, and pathogenesis. J Med Virol. 2020a Apr;92(4):418-423. doi: 10.1002/jmv.25681.

Chen L, Yu J, He W, Chen L, Yuan G, Dong F, Chen W, Cao Y, Yang J, Cai L, Wu D, Ran Q, Li L, Liu Q, Ren W, Gao F, Wang H, Chen Z, Gale RP, Li Q, Hu Y. Risk factors for death in 1859 subjects with COVID-19. Leukemia. 2020b Jun 16:1-11. doi: 10.1038/s41375-020-0911-0.

Cheng VC, Lau SK, Woo PC, Yuen KY. Severe acute respiratory syndrome coronavirus as an agent of emerging and reemerging infection. Clin Microbiol Rev. 2007 Oct;20(4):660-94.

Chen X, Chen Z, Azman AS, Deng X, Chen X, Lu W, Zhao Z, Yang J, Viboud C, Ajelli M, Leung DT, Yu H. Serological evidence of human infection with SARS-CoV-2: a systematic review and meta-analysis. medRxiv. 2020c Sep 13:2020.09.11.20192773.

Clark HL, Banks R, Jones L, Hornick TR, Higgins PA, Burant CJ, Canaday DH. Characterization of MHC-II antigen presentation by B cells and monocytes from older individuals. Clin Immunol. 2012 Aug;144(2):172-7. doi: 10.1016/j.clim.2012.06.005.

Cohet C, Montplaisir J, Petit D, Quinn MJ, Ouakki M, Deceuninck G, et al. Applying the test-negative design to vaccine safety studies: risk of narcolepsy associated with A/H1N1 (2009) pandemic influenza vaccination in Quebec [abstract 713]. Pharmacoepidemiol Drug Saf 2015;24:406-407. doi: 10.1002/pds.

Cohet C, van der Most R, Bauchau V, Bekkat-Berkani R, Doherty TM, Schuind A, Tavares Da Silva F, Rappuoli R, Carcon N, Innis BL. Safety of AS03-adjuvanted influenza vaccines: A review of the evidence. Vaccine 2019;37(23):3006-3021. doi: 10.1016/j.vaccine.2019.04.048.

COVID-19: Medicago's development programs. Available online: https://www.medicago.com/en/covid-19-programs (accessed on April 23, 2020).

Crooke SN, Ovsyannikova IG, Poland GA, Kennedy RB. Immunosenescence and human vaccine immune responses. Immun Ageing. 2019 Sep 13;16:25. doi: 10.1186/s12979-019-0164-9.

Djennad A, Ramsay ME, Pebody R, Fry NK, Sheppard C, Ladhani SN, Andrews NJ. Effectiveness of 23-Valent Polysaccharide Pneumococcal Vaccine and Changes in Invasive Pneumococcal Disease Incidence from 2000 to 2017 in Those Aged 65 and Over in England and Wales. EClinicalMedicine. 2019 Jan 2;6:42-50. doi: 10.1016/j.eclinm.2018.12.007.

Domnich A, Arata L, Amicizia D, Puig-Barberà J, Gasparini R, Panatto D. Effectiveness of MF59-adjuvanted seasonal influenza vaccine in the elderly: A systematic review and meta-analysis. Vaccine. 2017 Jan 23;35(4):513-520. doi: 10.1016/j.vaccine.2016.12.011.

Du L, He Y, Zhou Y, Liu S, Zheng BJ, Jiang S. The spike protein of SARS-CoV - a target for vaccine and therapeutic development. Nat Rev Microbiol. 2009 Mar;7(3):226-36. doi: 10.1038/nrmicro2090.

Du RH, Liang LR, Yang CQ, Wang W, Cao TZ, Li M, Guo GY, Du J, Zheng CL, Zhu Q, Hu M, Li XY, Peng P, Shi HZ. Predictors of mortality for patients with COVID-19 pneumonia caused by SARS-CoV-2: a prospective cohort study. Eur Respir J. 2020 May 7;55(5):2000524. doi: 10.1183/13993003.00524-2020.

Enjuanes L, Zuñiga S, Castaño-Rodriguez C, Gutierrez-Alvarez J, Canton J, Sola I. Molecular basis of coronavirus virulence and vaccine development. Adv Virus Res. 2016;96:245-286. doi: 10.1016/bs.aivir.2016.08.003.

FDA. Guidance for Industry: Toxicity grading scale for healthy adult and adolescent volunteers enrolled in preventive vaccine clinical trials. 2007.

Frasca D, Diaz A, Romero M, Landin AM, Blomberg BB. Age effects on B cells and humoral immunity in humans. Ageing Res Rev. 2011 Jul;10(3):330-5. doi: 10.1016/j.arr.2010.08.004.

Furman D, Campisi J, Verdin E, Carrera-Bastos P, Targ S, Franceschi C, Ferrucci L, Gilroy DW, Fasano A, Miller GW, Miller AH, Mantovani A, Weyand CM, Barzilai N, Goronzy JJ, Rando TA, Effros RB, Lucia A, Kleinstreuer N, Slavich GM. Chronic inflammation in the etiology of disease across the life span. Nat Med. 2019 Dec;25(12):1822-1832. doi: 10.1038/s41591-019-0675-0.

Furukawa NW, Brooks JT, Sobel J. Evidence Supporting Transmission of Severe Acute Respiratory Syndrome Coronavirus 2 While Presymptomatic or Asymptomatic. Emerg Infect Dis. 2020 Jul;26(7):e201595.

Garcon N, Vaughn DW, Didierlaurent AM. Development and evaluation of AS03, an Adjuvant System containing alpha-tocopherol and squalene in an oil-in-water emulsion. Expert Rev Vaccines 2012;11:349-366. doi: 10.1586/erv.11.192.

Garg S, Kim L, Whitaker M, O'Halloran A, Cummings C, Holstein R, Prill M, Chai SJ, Kirley PD, Alden NB, Kawasaki B, Yousey-Hindes K, Niccolai L, Anderson EJ, Openo KP, Weigel A, Monroe ML, Ryan P, Henderson J, Kim S, Como-Sabetti K, Lynfield R, Sosin D, Torres S, Muse A, Bennett NM, Billing L, Sutton M, West N, Schaffner W, Talbot HK, Aquino C, George A, Budd A, Brammer L, Langley G, Hall AJ, Fry A. Hospitalization Rates and Characteristics of Patients Hospitalized with Laboratory-Confirmed Coronavirus Disease 2019 - COVID-NET, 14 States, March 1-30, 2020.

MMWR Morb Mortal Wkly Rep. 2020 Apr 17;69(15):458-464. doi: 10.15585/mmwr.mm6915e3.

Guan WJ, Ni ZY, Hu Y, Liang WH, Ou CQ, He JX, Liu L, Shan H, Lei CL, Hui DSC, Du B, Li LJ, Zeng G, Yuen KY, Chen RC, Tang CL, Wang T, Chen PY, Xiang J, Li SY, Wang JL, Liang ZJ, Peng YX, Wei L, Liu Y, Hu YH, Peng P, Wang JM, Liu JY, Chen Z, Li G, Zheng ZJ, Qiu SQ, Luo J, Ye CJ, Zhu SY, Zhong NS; China medical treatment expert group for COVID-19. Clinical characteristics of coronavirus disease 2019 in China. N Engl J Med. 2020 Feb 28. doi: 10.1056/NEJMoa2002032.

Guan WJ, Liang WH, Zhao Y, Liang HR, Chen ZS, Li YM, Liu XQ, Chen RC, Tang CL, Wang T, Ou CQ, Li L, Chen PY, Sang L, Wang W, Li JF, Li CC, Ou LM, Cheng B, Xiong S, Ni ZY, Xiang J, Hu Y, Liu L, Shan H, Lei CL, Peng YX, Wei L, Liu Y, Hu YH, Peng P, Wang JM, Liu JY, Chen Z, Li G, Zheng ZJ, Qiu SQ, Luo J, Ye CJ, Zhu SY, Cheng LL, Ye F, Li SY, Zheng JP, Zhang NF, Zhong NS, He JX; China Medical Treatment Expert Group for COVID-19. Comorbidity and its impact on 1590 patients with COVID-19 in China: a nationwide analysis. Eur Respir J. 2020a May 14;55(5):2000547. doi: 10.1183/13993003.00547-2020.

Health Canada (HC). Coronavirus disease 2019 (COVID-19): Epidemiology update. Updated: July 23, 2020, 7 pm EDT. https://health-infobase.canada.ca/covid-19/epidemiological-summary-covid-19-cases.html#a7 (accessed July 24, 2020).

Heplisav-B® (Hepatitis B Vaccine [Recombinant], Adjuvanted) solution for intramuscular injection [package insert]. Emeryville (CA): Dynavax Technologies Corporation, 2020 [revised May 2020; cited 9 June 2020].

Honda-Okubo Y, Barnard D, Ong CH, Peng BH, Tseng CT, Petrovsky N. Severe acute respiratory syndrome-associated coronavirus vaccines formulated with delta inulin adjuvants provide enhanced protection while ameliorating lung eosinophilic immunopathology. J Virol. 2015 Mar;89(6):2995-3007. doi: 10.1128/JVI.02980-14.

Jackson LA, Campbell JD, Frey SE, Edwards KM, Keitel QA, Kotloff KL, Berry AA, Graham I, Atmar RL, Creech CB, Thomsen IP, Patel SM, Gutierrez AF, Anderson EL, Sahly HM, Hill H, Noah DL, Bellamy AR. Effect of varying doses of a monovalent H7N9 influenza vaccine with and without AS03 and MF59 adjuvants on immune response: a randomised clinical trial. JAMA 2015;314:237-46. doi: 10.1001/jama.2015.7916.

Khurana S, Coyle EM, Manischewitz J, King LR, Gao J, Germain RN, Schwartzberg PL, Tsang JS, Golding H, CHI Consortium. AS03-adjuvanted H5N1 vaccine promotes antibody diversity and affinity maturation, NAI titers, cross-clade H5N1 neutralization, but not H1N1 cross-subtype neutralization. NPJ Vaccines 2018;3:40. doi: 10.1038/s41541-018-0076-2.

Korakas E, Ikonomidis I, Kousathana F, Balampanis K, Kountouri A, Raptis A, Palaiodimou L, Kokkinos A, Lambadiari V. Obesity and COVID-19: immune and metabolic derangement as a possible link to adverse clinical outcomes. Am J Physiol Endocrinol Metab. 2020 Jul 1;319(1):E105-E109.

Kwetkat A, Heppner HJ. Comorbidities in the Elderly and Their Possible Influence on Vaccine Response. Interdiscip Top Gerontol Geriatr. 2020;43:73-85. doi: 10.1159/000504491.

Lake MA. What we know so far: COVID-19 current clinical knowledge and research. Clin Med (Lond). 2020 Mar;20(2):124-127. doi: 10.7861/clinmed.2019-coron.

Lambert P-H, Ambrosino DM, Andersen SR, Baric RS, Black SB, Chen RT, Dekker CL, Didierlaurent AM, Graham BS, Martin SD, Molrine DC, Perlman S, Picard-Fraser PA,

Pollard AJ, Qin C, Subbarao K, Cramer JP. Consensus summary report for CEPI/BC March 12-13, 2020 meeting: assessment of risk of disease enhancement with COVID-19 vaccines. Vaccine, 2020 May 25. doi: https://doi.org/10.1016/j.vaccine.2020.05.064.

Lansbury L, Smith S, Beyer W, Karamehic E, Pasic-Juhas E, Sikira H, Mateus A, Oshitani H, Zhao H, Beck CR, Nguyen-Van-Tam JS. Effectiveness of 2009 pandemic influenza A(H1N1) vaccines: a systematic review and meta-analysis. Vaccine 2017;35:1996–2006. doi: 10.1016/j.vaccine.2017.02.059.

Launay O, Duval X, Fitoussi S, Jilg W, Kerdpanich A, Montellano M, Schwarz TF, Watanveerade V, Wenzel JJ, Zalcman G, Bambure V, Li P, Caplanusi A, Madan A, Gillard P, Vaughn DW. Extended antigen sparing potential of AS03-adjuvanted pandemic H1N1 vaccines in children, and immunologic equivalence of two formulations of AS03-adjuvanted H1N1 vaccines: results from two randomised trials. BMC Infect Dis 2013;13:435. doi: 10.1186/1471-2334-13-435.

Law B, Sturkenboom M. Safety platform for emergency vaccines (SPEAC). D2.3 Priority list of adverse events of special interest: COVID-19. Report v1.1. 2020 Mar 5.

Law B, Sturkenboom M. Safety platform for emergency vaccines (SPEAC). D2.3 Priority list of adverse events of special interest: COVID-19. Report v2.0. 2020a May 25.

Leroux-Roels I, Borkowski A, Vanwolleghem T, Drame M, Clement F, Hons E, Devaster J-M, Leroux-Roels G. Antigen sparing and cross-reactive immunity with an adjuvanted rH5N1 prototype pandemic influenza vaccine: a randomised controlled trial. Lancet 2007;370:580–9. doi: 10.1016/s0140-6736(07)61297-5.

Li H, Liu Z, Ge J. Scientific research progress of COVID-19/SARS-CoV-2 in the first five months. J Cell Mol Med. 2020 Jun;24(12):6558-6570. doi: 10.1111/jcmm.15364.

Liu J, Zheng X, Tong Q, Li W, Wang B, Sutter K, Trilling M, Lu M, Dittmer U, Yang D. Overlapping and discrete aspects of the pathology and pathogenesis of the emerging human pathogenic coronaviruses SARS-CoV, MERS-CoV, and 2019-nCoV. J Med Virol. 2020 May;92(5):491-494. doi: 10.1002/jmv.25709.

Liu K, Chen Y, Lin R, Han K. Clinical features of COVID-19 in elderly patients: A comparison with young and middle-aged patients. J Infect. 2020a Mar 27. pii: S0163-4453(20)30116-X. doi: 10.1016/j.jinf.2020.03.005.

Lu S. Timely development of vaccines against SARS-CoV-2. Emerg Microbes Infect. 2020 Mar 8;9(1):542-544. doi: 10.1080/22221751.2020.1737580.

Madan A, Collins H, Sheldon E, Frenette L, Chu L, Friel D, Drame M, Vaughn DW, Innis BL, Schuind A. Evaluation of a primary course of H9N2 vaccine with or without AS03 adjuvant in adults: a phase I/II randomized trial. Vaccine 2017;35:4621–8. doi: 10.1016/j.vaccine.2017.07.013.

Madan A, Ferguson M, Rheault P, Seiden D, Toma A, Friel D, Soni J, Li P, Innis BL, Schuind A. Immunogenicity and safety of an AS03-adjuvanted H7N1 vaccine in adults 65 years of age and older: a phase II, observer-blind, randomized, controlled trial. Vaccine 2017a;35:1865–72. doi: 10.1016/j.vaccine.2017.02.057.

Madan A, Ferguson M, Sheldon E, Segall N, Chu L, Toma A, Rheault P, Friel D, Soni J, Li P, Innis BL, Schuind A. Immunogenicity and safety of an AS03-adjuvanted H7N1 vaccine in healthy adults: a phase I/II, observer-blind, randomized, controlled trial. Vaccine 2017b;35:1431–9. doi: 10.1016/j.vaccine.2017.01.054.

McElhaney JE, Beran J, Devaster JM, Esen M, Launay O, Leroux-Roels G, Ruiz-Palacios GM, van Essen GA, Caplanusi A, Claeys C, Durand C, Duval X, Idrissi ME, Falsey AR,

Feldman G, Frey SE, Galtier F, Hwang S-J, Innis BL, Kovac M, Kremsner P, McNeil S, Nowakowski A, Richardus JH, Trofa A, Oostvogels L, Influence65 study group. AS03-adjuvanted versus non-adjuvanted inactivated trivalent influenza vaccine against seasonal influenza in elderly people: a phase 3 randomised trial. Lancet Infect Dis 2013;13:485-496. doi: 10.1016/S1473-3099(13)70046-X.

McElhaney JE, Coler RN, Baldwin SL. Immunologic correlates of protection and potential role for adjuvants to improve influenza vaccines in older adults. Expert Rev Vaccines. 2013a Jul;12(7):759-66.

Mehra MR, Desai SS, Kuy S, Henry TD, Patel AN. Cardiovascular Disease, Drug Therapy, and Mortality in Covid-19. N Engl J Med. 2020 Jun 18;382(25):e102. doi: 10.1056/NEJMoa2007621.

Memish ZA, Perlman S, Van Kerkhove MD, Zumla A. Middle East respiratory syndrome. Lancet. 2020 Mar 28;395(10229):1063-1077. doi: 10.1016/S0140-6736(19)33221-0.

Montplaisir J, Petit D, Quinn MJ, Ouakki M, Deceuninck G, Desautels A, Mignot E, De Wals P. Risk of narcolepsy associated with inactivated adjuvanted AS03A/H1N1 (2009) pandemic influenza vaccine in Québec. PLoS One 2014;9(9):e108489. doi: 10.1371/journal.pone.0108489.

Nath KD, Burel JG, Shankar V, Pritchard AL, Towers M, Looke D, Davies JM, Upham JW. Clinical factors associated with the humoral immune response to influenza vaccination in chronic obstructive pulmonary disease. Int J Chron Obstruct Pulmon Dis. 2014;9:51-6. doi: 10.2147/COPD.S53590.

Ng OW, Chia A, Tan AT, Jadi RS, Leong HN, Bertoletti A, Tan YJ. Memory T cell responses targeting the SARS coronavirus persist up to 11 years post-infection. Vaccine. 2016 Apr 12;34(17):2008-14. doi: 10.1016/j.vaccine.2016.02.063.

Nolan T, Izurieta P, Lee BW, Chan PC, Marshall H, Booy R, Drame M, Vaughn DW. Heterologous prime-boost vaccination using an AS03B-adjuvanted influenza A(H5N1) vaccine in infants and children < 3 years of age. J Infect Dis 2014;210(11):1800-10. doi: 10.1093/infdis/jiu359.

Ou X, Liu Y, Lei X, Li P, Mi D, Ren L, Guo L, Guo R, Chen T, Hu J, Xiang Z, Mu Z, Chen X, Chen J, Hu K, Jin Q, Wang J, Qian Z. Characterization of spike glycoprotein of SARS-CoV-2 on virus entry and its immune cross-reactivity with SARS-CoV. Nat Commun. 2020 Mar 27;11(1):1620. doi: 10.1038/s41467-020-15562-9.

Paules CI, Marston HD, Fauci AS. Coronavirus infections-more than just the common cold. JAMA. 2020 Jan 23. doi: 10.1001/jama.2020.0757.

Pinti M, Appay V, Campisi J, Frasca D, Fülöp T, Sauce D, Larbi A, Weinberger B, Cossarizza A. Aging of the immune system – focus on inflammation and vaccination. Eur J Immunol. 2016 Oct;46(10):2286-2301. doi: 10.1002/eji.201546178.Randolph HE, Barreiro LB. Herd Immunity: Understanding COVID-19. Immunity. 2020 May 19;52(5):737-741.

Rothman KJ, Greenland S, Lash TL. Modern Epidemiology. 3rd ed. Philadelphia: Lippincott Williams & Wilkins, 2008. Chapter 14.

Sanyaolu A, Okorie C, Marinkovic A, Patidar R, Younis K, Desai P, Hosein Z, Padda I, Mangat J, Altaf M. Comorbidity and its Impact on Patients with COVID-19. SN Compr. Clin. Med. 2020. doi.org/10.1007/s42399-020-00363-4.

Sasaki S, Sullivan M, Narvaez CF, Holmes TH, Furman D, Zheng NY, Nishtala M, Wrammert J, Smith K, James JA, Dekker CL, Davis MM, Wilson PC, Greenberg HB, He XS. Limited

efficacy of inactivated influenza vaccine in elderly individuals is associated with decreased production of vaccine-specific antibodies. J Clin Invest. 2011 Aug;121(8):3109-19. doi: 10.1172/JCI57834.

Sette A, Crotty S. Pre-existing immunity to SARS-CoV-2: the knowns and unknowns. Nat Rev Immunol. 2020 Aug;20(8):457-458.

Shahid Z, Kalayanamitra R, McClafferty B, Kepko D, Ramgobin D, Patel R, Aggarwal CS, Vunnam R, Sahu N, Bhatt D, Jones K, Golamari R, Jain R. COVID-19 and Older Adults: What We Know. J Am Geriatr Soc. 2020 May;68(5):926-929. doi: 10.1111/jgs.16472.

Shaw AC, Goldstein DR, Montgomery RR. Age-dependent dysregulation of innate immunity. Nat Rev Immunol. 2013 Dec;13(12):875-87. doi: 10.1038/nri3547.

Stebbing J, Phelan A, Griffin I, Tucker C, Oechsle O, Smith D, Richardson P. COVID-19: combining antiviral and anti-inflammatory treatments. Lancet Infect Dis. 2020 Apr;20(4):400-402. doi: 10.1016/S1473-3099(20)30132-8.

Stokes EK, Zambrano LD, Anderson KN, Marder EP, Raz KM, El Burai Felix S, Tie Y, Fullerton KE. Coronavirus Disease 2019 Case Surveillance — United States, January 22–May 30, 2020. MMWR Morb Mortal Wkly Rep. 2020 Jun 19;69(24):759-765. doi: 10.15585/mmwr.mm6924e2.

Su S, Wong G, Shi W, Liu J, Lai ACK, Zhou J, Liu W, Bi Y, Gao GF. Epidemiology, genetic recombination, and pathogenesis of coronaviruses. Trends Microbiol. 2016 Jun;24(6):490-502. doi: 10.1016/j.tim.2016.03.003.

Tang F, Quan Y, Xin ZT, Wrammert J, Ma MJ, Lv H, Wang TB, Yang H, Richardus JH, Liu W, Cao WC. Lack of peripheral memory B cell responses in recovered patients with severe acute respiratory syndrome: a six-year follow-up study. J Immunol. 2011 Jun 15;186(12):7264-8. doi: 10.4049/jimmunol.0903490.

Thanh Le T, Andreadakis Z, Kumar A, Gómez Román R, Tollefsen S, Saville M, Mayhew S. The COVID-19 vaccine development landscape. Nat Rev Drug Discov. 2020 Apr 9. doi: 10.1038/d41573-020-00073-5.

Vaughn DW, Seifert H, Hepburn A, Dewe W, Li P, Drame M, Cohet C, Innis BL, Fries LF. Safety of AS03-adjuvanted inactivated split virion A(H1N1)pdm09 and H5N1 influenza virus vaccines administered to adults: pooled analysis of 28 clinical trials. Hum Vaccin Immunother 2014;10(10):2942-57. doi: 10.4161/21645515.2014.972149.

Vardeny O, Moran JJ, Sweitzer NK, Johnson MR, Hayney MS. Decreased T-cell responses to influenza vaccination in patients with heart failure. Pharmacotherapy. 2010 Jan;30(1):10-6. doi: 10.1592/phco.30.1.10.

Verschoor CP, Lelic A, Parsons R, Evelegh C, Bramson JL, Johnstone J, Loeb MB, Bowdish DME. Serum C-Reactive Protein and Congestive Heart Failure as Significant Predictors of Herpes Zoster Vaccine Response in Elderly Nursing Home Residents. J Infect Dis. 2017 Jul 15;216(2):191-197. doi: 10.1093/infdis/jix257.

Waddington CS, Walker WT, Oeser C, Reiner A, John T, Wilkins S, Casey M, Eccleston PE, Allen RJ, Okike I, Ladhani S, Sheasby E, Hoschler K, Andrews N, Waight P, Collinson AC, Heath PT, Finn A, Faust SN, Snape MD, Miller E, Pollard AJ. Safety and immunogenicity of AS03B adjuvanted split virion versus non-adjuvanted whole virion H1N1 influenza vaccine in UK children aged 6 months-12 years: open label, randomised, parallel group, multicentre study. BMJ 2010;340:c2649. doi: 10.1136/bmj.c2649.

Wang B, Li R, Lu Z, Huang Y. Does comorbidity increase the risk of patients with COVID-19: evidence from meta-analysis. Aging (Albany NY). 2020 Apr 8;12(7):6049-6057. doi: 10.18632/aging.103000.

Wathuo M, Medley GF, Nokes DJ and Munywoki PK. Quantification and determinants of the amount of respiratory syncytial virus (RSV) shed using real time PCR data from a longitudinal household study [version 2; peer review: 3 approved, 1 approved with reservations]. Wellcome Open Res 2017, 1:27. (https://doi.org/10.12688/wellcomeopenres.10284.2).

Weinberg A, Pang L, Johnson MJ, Caldas Y, Cho A, Tovar-Salazar A, Canniff J, Schmader KE, Popmihajlov Z, Levin MJ. The Effect of Age on the Immunogenicity of the Live Attenuated Zoster Vaccine Is Predicted by Baseline Regulatory T Cells and Varicella-Zoster Virus-Specific T Cell Immunity. J Virol. 2019 Jul 17;93(15):e00305-19. doi: 10.1128/JVI.00305-19.

Wilkinson K, Wei Y, Szwajcer A, Rabbani R, Zarychanski R, Abou-Setta AM, Mahmud SM. Efficacy and safety of high-dose influenza vaccine in elderly adults: A systematic review and meta-analysis. Vaccine. 2017 May 15;35(21):2775-2780. doi: 10.1016/j.vaccine.2017.03.092.

World Health Organization. WHO Director-General’s opening remarks at the media briefing on COVID-19 – 11 March 2020. https://www.who.int/dg/speeches/detail/who-director-general-s-opening-remarks-at-the-media-briefing-on-covid-19---11-march-2020. Published 11 March 2020. Accessed April 23, 2020.

World Health Organization. Clinical management of COVID-19: interim guidance, 27 May 2020a. World Health Organization. https://apps.who.int/iris/handle/10665/332196.

Yang J, Zheng Y, Gou X, Pu K, Chen Z, Guo Q, Ji R, Wang H, Wang Y, Zhou Y. Prevalence of comorbidities in the novel Wuhan coronavirus (COVID-19) infection: a systematic review and meta-analysis. Int J Infect Dis. 2020 Mar 12. pii: S1201-9712(20)30136-3. doi: 10.1016/j.ijid.2020.03.017.

Yang WH, Dionne M, Kyle M, Aggarwal N, Li P, Madariaga M, Godeaux O, Vaughn DW. Long term immunogenicity of an AS03-adjuvanted influenza A(H1N1)pdm09 vaccine in young and elderly adults: an observer-blind, randomised trial. Vaccine 2013;31:4389-4397. doi: 10.1016/j.vaccine.2013.07.007.

Yang X, Yu Y, Xu J, Shu H, Xia J, Liu H, Wu Y, Zhang L, Yu Z, Fang M, Yu T, Wang Y, Pan S, Zou X, Yuan S, Shang Y. Clinical course and outcomes of critically ill patients with SARS-CoV-2 pneumonia in Wuhan, China: a single-centered, retrospective, observational study. Lancet Respir Med. 2020a May;8(5):475-481. doi: 10.1016/S2213-2600(20)30079-5.

Yin JK, Khandaker G, Rashid H, Heron L, Ridda I, Booy R. Immunogenicity and safety of pandemic influenza A (H1N1) 2009 vaccine: systematic review and meta-analysis. Influenza Other Respir Viruses 2011;5(5):299-305. doi: 10.1111/j.1750-2659.2011.00229.x.

Zhou F, Yu T, Du R, Fan G, Liu Y, Liu Z, Xiang J, Wang Y, Song B, Gu X, Guan L, Wei Y, Li H, Wu X, Xu J, Tu S, Zhang Y, Chen H, Cao B. Clinical course and risk factors for mortality of adult inpatients with COVID-19 in Wuhan, China: a retrospective cohort study. Lancet. 2020 Mar 28;395(10229):1054-1062. doi: 10.1016/S0140-6736(20)30566-3.

Zhu N, Zhang D, Wang W, Li X, Yang B, Song J, Zhao X, Huang B, Shi W, Lu R, Niu P, Zhan F, Ma X, Wang D, Xu W, Wu G, Gao GF, Tan W; China novel coronavirus investigating and research team. A novel coronavirus from patients with pneumonia in China, 2019. N Engl J Med. 2020 Feb 20;382(8):727-733. doi: 10.1056/NEJMoa2001017.

Zimmermann P, Curtis N. Factors That Influence the Immune Response to Vaccination. Clin Microbiol Rev. 2019 Mar 13;32(2):e00084-18. doi: 10.1128/CMR.00084-18.

Zimmermann P, Curtis N. COVID-19 in Children, Pregnancy and Neonates: A Review of Epidemiologic and Clinical Features. Pediatr Infect Dis J. 2020 Jun;39(6):469-477.

19 APPENDICES

The clinical documents included in the appendices are meant as examples only; the actual documents used during the trial may differ slightly.

19.1 Appendix 1 – Sample Ruler to Measure Local Adverse Events

Site Adverse Event Measuring Ruler

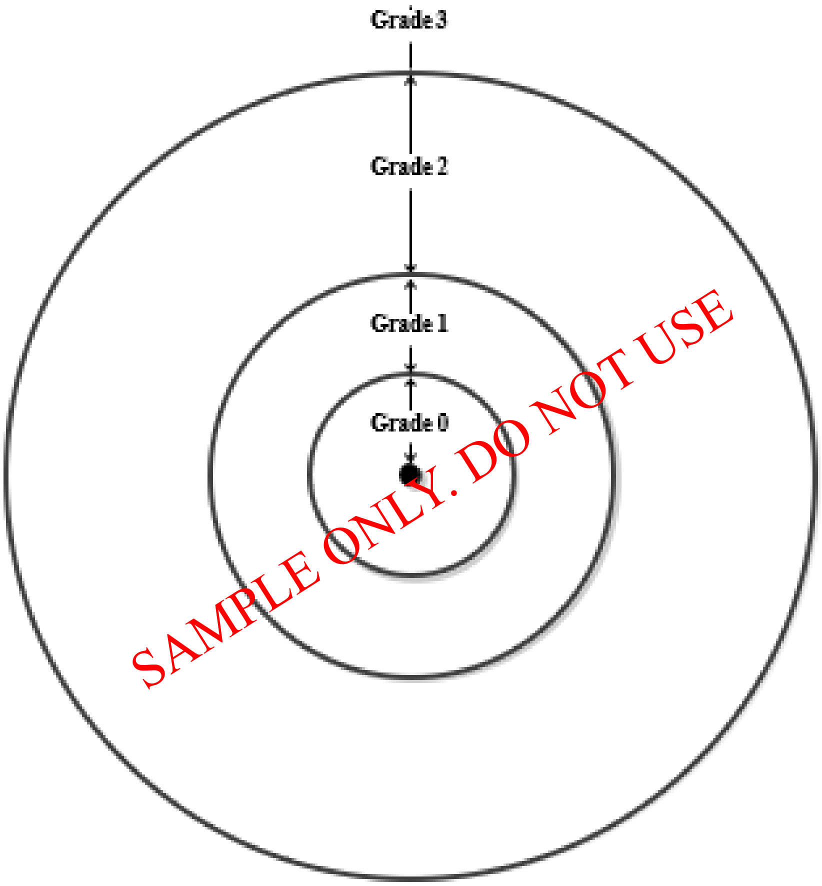

Grading for redness and swelling where the vaccine was given	
Grade 0	None or less than 25 mm
Grade 1	Between 25 mm and 50 mm
Grade 2	Between 51 and 100 mm
Grade 3	More than 100 mm
Grade 4	skin/tissue loss at the injection site (blister or ulcer formation) of any size

19.2 Appendix 2 – Subject Diary Sample Pages

DIARY (DAY 0 to DAY 7)	
**Study Name**	A Randomized, Observer-Blind, Placebo-Controlled, Phase 2/3 Study to Assess the Safety, Efficacy, and Immunogenicity of a Recombinant Coronavirus-Like Particle COVID-19 Vaccine in Adults 18 Years of Age or Older
**Protocol Number**	CP-PRO-CoVLP-021
**Sponsor**	Medicago R&D Inc.<br>1020, route de l'Église, bureau 600<br>Québec (Qc), Canada G1V 3V9
**Clinical site Address**	
**Principal Investigator Name**	
**Clinical Research Coordinator**	
**24-hour Emergency Number**	

SAMPLE ONLY. DO NOT USE

DIARY (DAY 0 to DAY 7) INSTRUCTIONS

- You should fill in your Diary at approximately the same time, preferably in the evening.
- You will need to refer to this Diary during the Telephone Calls (on Day 1 and Day 8).
- You need to bring this Diary with you at the Day 3 and Day 21 visits.

- Fill in the Day 0 to Day 7 tables EVERY DAY for the first 7 days after you receive your vaccine dose.
- Fill in each day's column by entering the WORST grade for each symptom that you had during the period.
- Grayed out sections are to be left blank and will be completed by the site personnel.
- To complete this document, use blue or black ink. No pencil or liquid paper is allowed.
- If you made a mistake, cross out the incorrect information, initial and date, and write the correct answer beside.
- Date should be recorded with 2 digits for day, 3 letters for month and 4 digits for year

January	JAN	April	APR	July	JUL	October	OCT
February	FEB	May	MAY	August	AUG	November	NOV
March	MAR	June	JUN	September	SEP	December	DEC

SAMPLE ONLY. DO NOT USE

TEMPERATURE:

- To record the temperature, use the thermometer you were given at the clinic. Do not drink, eat food or smoke prior to taking your temperature.
- You should take your temperature for the first 7 days following vaccination at approximately the same time each evening and at any other time if you feel feverish.
- You will need to record the <u>highest temperature of the day</u> on page 4.
- If your temperature is 38°C or 100.4°F or higher, you are allowed to take over-the-counter antipyretics (e.g., acetaminophen/paracetamol, aspirin, naproxen, or ibuprofen) and you should increase the frequency of temperature measurements to approximately every 4 hours, until you don't have a fever anymore.
- Medication intake needs to be documented on the Memory Aid and will be reviewed by the site personnel.

DIARY (DAY 0 to DAY 7) INSTRUCTIONS

SYMPTOMS:

- Complete the table referring to the following periods:
  - For the 1st column: the period between the vaccination and the 30 minutes post-vaccination;
  - For the 2nd column: the period between the last evaluation (30 minutes post-vaccination) and the evening;
  - For the remaining columns: the period since the previous evaluation of the symptoms.
- If the symptom is not present, indicate 0.
- If the symptom is present, a grade should be indicated.
- Evaluate the grade of all other symptoms according to the definitions:
  - **0= No symptom**
  - **1= Does not interfere with activity**
  - **2= Repeated use of non-narcotic pain reliever (e.g. Advil, Tylenol) >24 hours or interferes with activity but does not require medical intervention**
  - **3= Any use of narcotic pain reliever (e.g. codeine, morphine) or prevents daily activity and require medical intervention**
  - **4= Visit to Emergency room or hospitalization**
- List any other problems (not listed on pages 4 to 6) on the Memory Aid (Day 0 to Day 21) page “side effects (symptoms)”.
- Symptoms listed on pages 4 to 6 which persist longer than 7 days after study vaccine administration must also be listed on the Memory Aid (Day 0 to Day 21) page “side effects (symptoms)”.

You received the study vaccine on: |_|_|_|_|_|_|_|_|_| at: |_|_| : |_|_|
dd mmm yyyy hh mm

SAMPLE ONLY. DO NOT USE

DIARY (DAY 0 to DAY 7)

Day 0 to Day 7		30 min, Post-dose	Evening, day of dose	1st Day after dose	2nd Day after dose	3rd Day after dose	4th Day after dose	5th Day after dose	6th Day after dose	7th Day after dose
**Date (dd-mmm-yyyy)**										
Oral Temperature	°C / °F									
	Causality									
Redness where the injection was given	Grade									
	Causality									
	Use the measuring tool given to you at the site to estimate the size of the red area around the injection site and indicate the grade above.									
Swelling where the injection was given	Grade									
	Causality									
	Use the measuring tool given to you at the site to estimate the size of the swollen area around the injection site and indicate the grade above.									
Pain at vaccine injection site	Grade									
	Causality									
	**0**= No symptom; **1**= Does not interfere with activity; **2**= Repeated use of non-narcotic pain reliever >24 hours or interferes with activity; **3**= Any use of narcotic pain reliever or prevents daily activity; **4**= Visit to Emergency room or hospitalization.									

SAMPLE ONLY. DO NOT USE

Comments:

MD initials and date: |__|__|__|__|__|__|__|__|__|
dd mmm yyyy

<table>
<tr><th colspan="11">DIARY (DAY 0 to DAY 7)</th></tr>
<tr><th colspan="2">Day 0 to Day 7</th><th>30 min, Post-dose</th><th>Evening, day of dose</th><th>1st Day after dose</th><th>2nd Day after dose</th><th>3rd Day after dose</th><th>4th Day after dose</th><th>5th Day after dose</th><th>6th Day after dose</th><th>7th Day after dose</th></tr>
<tr><td rowspan="2">Headache</td><td>Grade</td><td></td><td></td><td></td><td></td><td></td><td></td><td></td><td></td><td></td></tr>
<tr><td>Causality</td><td></td><td></td><td></td><td></td><td></td><td></td><td></td><td></td><td></td></tr>
<tr><td>GRADES</td><td colspan="10">0= No symptom; 1= Does not interfere with activity; 2= Repeated use of non-narcotic pain reliever >24 hours or interferes with activity; 3= Any use of narcotic pain reliever or prevents daily activity; 4= Visit to Emergency room or hospitalization.</td></tr>
<tr><td rowspan="2">Muscle aches</td><td>Grade</td><td></td><td></td><td></td><td></td><td></td><td></td><td></td><td></td><td></td></tr>
<tr><td>Causality</td><td></td><td></td><td></td><td></td><td></td><td></td><td></td><td></td><td></td></tr>
<tr><td>GRADES</td><td colspan="10">0= No symptom; 1= Does not interfere with activity; 2= Interferes with activity; 3= Prevents daily activity; 4= Visit to Emergency room or hospitalization.</td></tr>
<tr><td rowspan="2">Fatigue</td><td>Grade</td><td></td><td></td><td></td><td></td><td></td><td></td><td></td><td></td><td></td></tr>
<tr><td>Causality</td><td></td><td></td><td></td><td></td><td></td><td></td><td></td><td></td><td></td></tr>
<tr><td>GRADES</td><td colspan="10">0= No symptom; 1= Does not interfere with activity; 2= Interferes with activity; 3= Prevents daily activity; 4= Visit to Emergency room or hospitalization.</td></tr>
<tr><td rowspan="2">Joint aches</td><td>Grade</td><td></td><td></td><td></td><td></td><td></td><td></td><td></td><td></td><td></td></tr>
<tr><td>Causality</td><td></td><td></td><td></td><td></td><td></td><td></td><td></td><td></td><td></td></tr>
<tr><td>GRADES</td><td colspan="10">0= No symptom; 1= Does not interfere with activity; 2= Interferes with activity but does not require medical intervention; 3= Prevents daily activity and requires medical intervention; 4= Visit to Emergency room or hospitalization.</td></tr>
</table>

SAMPLE ONLY. DO NOT USE

Comments:

MD initials and date: |_|_|_|_|_|_|_|_|_|
dd mmm yyyy

DIARY (DAY 0 to DAY 7)

**Day 0 to Day 7**		**30 min, Post-dose**	**Evening, day of dose**	**1st Day after dose**	**2nd Day after dose**	**3rd Day after dose**	**4th Day after dose**	**5th Day after dose**	**6th Day after dose**	**7th Day after dose**
Chills	Grade									
	Causality									
Feelings of general discomfort or uneasiness	Grade									
	Causality									
Feeling of swelling in the neck	Grade									
	Causality									
Feeling of swelling in the axilla (armpit)	Grade									
	Causality									

SAMPLE ONLY. DO NOT USE

GRADES **0**= No symptom; **1**= Does not interfere with activity; **2**= Interferes with activity but does not require medical intervention; **3**= Prevents daily activity and requires medical intervention; **4**= Visit to Emergency room or hospitalization.

Comments:

I confirm I have reviewed the 3 pages of solicited symptoms and evaluated the causalities when needed. I confirm I have reviewed all entries in the Comments section on this page.

MD initials and date: |_|_|_|_|_|_|_|_|_| dd mmm yyyy

19.3 Appendix 3 – Subject Memory Aid Sample Pages

MEMORY AID (DAY 0 to DAY 42)	
**Study Name**	A Randomized, Observer-Blind, Placebo-Controlled, Phase 2/3 Study to Assess the Safety, Efficacy, and Immunogenicity of a Recombinant Coronavirus-Like Particle COVID-19 Vaccine in Adults 18 Years of Age or Older
**Protocol Number**	CP-PRO-CoVLP-021
**Sponsor**	Medicago R&D Inc.<br>1020, route de l'Église, suite 600<br>Québec, QC, Canada G1V 3V9
**Clinical site Address**	
**Principal Investigator Name**	
**Clinical Research Coordinator**	
**24-hour Emergency Number**	

SAMPLE ONLY. DO NOT USE

MEMORY AID (DAY 0 to DAY 42) INSTRUCTIONS

- You should fill in your Memory aid at approximately the same time, preferably in the evening.
- You will need to refer to this Memory Aid during the Telephone Calls (on Day 1, Day 8, Day 22, and Day 29).
- You need to bring this Memory Aid with you at the Day 3, Day 21, Day 24, and Day 42 visits.

- Grayed out sections are to be left blank and will be completed by the site personnel.
- To complete this document, use blue or black ink. No pencil or liquid paper is allowed.
- If you made a mistake, cross out the incorrect information, initial and date, and write the correct answer beside.
- Date should be recorded with 2 digits for day, 3 letters for month and 4 digits for year

January	JAN	April	APR	July	JUL	October	OCT
February	FEB	May	MAY	August	AUG	November	NOV
March	MAR	June	JUN	September	SEP	December	DEC

SIDE EFFECTS (Symptoms) and MEDICATION:

- Use the space provided to record important information that you want to tell us about changes in your health. Note any health problems you experience, worsening of previous problems, or new medicines you are taking. Examples may include prescription drugs (including vaccines), over-the-counter drugs, herbal supplements and/or vitamins. Indicate each intake of medication you are usually taking as needed.
- It is especially important that you make a note of problems that required a visit to your doctor, a hospital stay, or a visit to an emergency room.
- It is also important that you make a note of changes in your medications.
- Each medication must be associated to a problem, but each problem does not require a medication associated with it.
- Indicate the start and stop date (when available) for each symptom and medication. However, if it is still ongoing at the Day 21 or Day 42 visits, you will be required to tick the ongoing checkbox.
- Please contact the study site if you experience any severe effects or if you have any concerns at any time by using the emergency contact number provided on the first page of this Memory Aid.

SAMPLE ONLY. DO NOT USE

You received the first study vaccine on: |__|__|__|__|__|__|__|__|__| at: |__|__| : |__|__|
dd mmm yyyy hh mm

You received the second study vaccine on: |__|__|__|__|__|__|__|__|__| at: |__|__| : |__|__|
dd mmm yyyy hh mm

COVID-19 INFORMATION

SYMPTOMS FOR COVID-19:

- The symptoms associated with COVID-19 are similar to the flu. These symptoms include:
  - Fever or chills;
  - Cough;
  - Shortness of breath or difficulty breathing;
  - Fatigue;
  - Muscle or body aches;
  - Headache;
  - New loss of taste or smell;
  - Sore throat;
  - Congestion or runny nose;
  - Nausea or vomiting;
  - Diarrhea.

- Individuals who have COVID-19 may show some symptoms or none at all.
- Symptoms for COVID-19 can appear up to 14 days after exposure to the virus.

SAMPLE ONLY. DO NOT USE

MEMORY AID (DAY 0 to DAY 21)						
**Day 0 to Day 21**					❑ Nothing to report	
#	**Side Effects (symptoms)**	**Grade** (See below)	**Date and time it started**	**Date and time it ended**	**Did you receive medical care?**	**Validated with subject**
				❑ Ongoing at Day 21	❑ Yes ❑ No	❑ Initial and date:
				❑ Ongoing at Day 21	❑ Yes ❑ No	❑ Initial and date:

SAMPLE ONLY. DO NOT USE

**GRADES**	**0**= No symptom; **1**= Does not interfere with activity; **2**= Repeated use of non-narcotic pain reliever >24 hours or interferes with activity but does not require medical intervention; **3**= Any use of narcotic pain reliever or prevents daily activity and does require medical intervention; **4**= Visit to Emergency room or hospitalization.

Comments:

MD initials and date:

MEMORY AID (DAY 0 to DAY 21)

**Day 0 to Day 21**				❑ Nothing to report	
#	**Medication** (Name, Dose, Route and Frequency)	**Start date**	**Stop date**	**Reason(s) why you are taking this medication?**	**Validated with subject**
			❑ Ongoing at Day 21		❑ Initial and date:
			❑ Ongoing at Day 21		❑ Initial and date:
		|_|_|_|_|_|_|_|_|_| dd mmm yyyy	|_|_|_|_|_|_|_|_|_| dd mmm yyyy ❑ Ongoing at Day 21		❑ Initial and date: |_|_|_|_|_|_|_|_|_| dd mmm yyyy

SAMPLE ONLY. DO NOT USE

**Dose**			**Route**			**Frequency**		
Capsule	Tablespoon	g	Oral	Intramuscular	Ophthalmic	Once a day	Every other day	Continuous
Tablet	Teaspoon	mcg	Topical	Nasal	Intra-articular	Twice a day	Every 3 days	Intermittent
Ring	Puff	mg	Inhalation	Intravenous	Rectal	Three times/day	Every 2 weeks	Once
Application	Drops	IU	Intrauterine	Transdermal	Sublingual	1x per week	Every 6 months	As needed
Lozenge	mL	Units	Vaginal	Subcutaneous	Unknown	2x per week	Every hour	Unknown
Syringe	%	Unknown				Once a month	Once a year	

Comments:

MD initials and date: |_|_|_|_|_|_|_|_|_| dd mmm yyyy

MEMORY AID (DAY 22 to DAY 42)

Day 22 to Day 42 ❑ Nothing to report

#	Side Effects (symptoms)	Grade (See below)	Date and time it started	Date and time it ended	Did you receive medical care?	Validated with subject
			dd mmm yyyy<br>hh : mm	dd mmm yyyy<br>hh : mm<br>❑ Ongoing at Day 42	❑ Yes<br>❑ No	❑ Initial and date:<br>dd mmm yyyy
			dd mmm yyyy<br>hh : mm	dd mmm yyyy<br>hh : mm<br>❑ Ongoing at Day 42	❑ Yes<br>❑ No	❑ Initial and date:<br>dd mmm yyyy

SAMPLE ONLY. DO NOT USE

GRADES **0**= No symptom; **1**= Does not interfere with activity; **2**= Repeated use of non-narcotic pain reliever >24 hours or interferes with activity but does not require medical intervention; **3**= Any use of narcotic pain reliever or prevents daily activity and does require medical intervention; **4**= Visit to Emergency room or hospitalization.

Comments:

MD initials and date: dd mmm yyyy

MEMORY AID (DAY 22 to DAY 42)

Day 22 to Day 42 ❑ Nothing to report

#	**Medication** (Name, Dose, Route and Frequency)	**Start date**	**Stop date**	**Reason(s) why you are taking this medication?**	**Validated with subject**
		dd mmm yyyy	dd mmm yyyy ❑ Ongoing at Day 42		❑ Initial and date: dd mmm yyyy
		dd mmm yyyy	dd mmm yyyy ❑ Ongoing at Day 42		❑ Initial and date: dd mmm yyyy
		dd mmm yyyy	dd mmm yyyy ❑ Ongoing at Day 42		❑ Initial and date: dd mmm yyyy

SAMPLE ONLY. DO NOT USE

**Dose**			**Route**			**Frequency**		
Capsule	Tablespoon	g	Oral	Intramuscular	Ophthalmic	Once a day	Every other day	Continuous
Tablet	Teaspoon	mcg	Topical	Nasal	Intra-articular	Twice a day	Every 3 days	Intermittent
Ring	Puff	mg	Inhalation	Intravenous	Rectal	Three times/day	Every 2 weeks	Once
Application	Drops	IU	Intrauterine	Transdermal	Sublingual	1x per week	Every 6 months	As needed
Lozenge	mL	Units	Vaginal	Subcutaneous	Unknown	2x per week	Every hour	Unknown
Syringe	%	Unknown				Once a month	Once a year	

Comments:

MD initials and date: dd mmm yyyy

medicago

19.4 Appendix 4 – List of Potential Immune-Mediated Diseases

Neuroinflammatory disorders	Musculoskeletal disorders	Skin disorders
• Cranial nerve neuropathy, including paralysis and paresis (eg, Bell's palsy).<br>• Optic neuritis.<br>• Multiple sclerosis.<br>• Transverse myelitis.<br>• Guillain-Barré syndrome, including Miller Fisher syndrome and other variants.<br>• Acute disseminated encephalomyelitis, including site specific variants, eg, noninfectious encephalitis, encephalomyelitis, myelitis, myeloradiculoneuritis.<br>• Myasthenia gravis, including Lambert-Eaton myasthenic syndrome.<br>• Demyelinating peripheral neuropathies and plexopathies including:<br>- Chronic inflammatory demyelinating polyneuropathy.<br>- Multifocal motor neuropathy.<br>- Polyneuropathies associated with monoclonal gammopathy.<br>• Narcolepsy.<br>• Tolosa Hunt syndrome.	• Systemic lupus erythematosus and associated conditions.<br>• Systemic scleroderma (systemic sclerosis), including:<br>- Diffuse scleroderma.<br>- CREST syndrome.<br>• Idiopathic inflammatory myopathies, including:<br>- Dermatomyositis.<br>- Polymyositis.<br>• Anti-synthetase syndrome.<br>• Rheumatoid arthritis and associated conditions including:<br>- Juvenile idiopathic arthritis.<br>- Still's disease.<br>• Polymyalgia rheumatica.<br>• Spondyloarthropathies, including:<br>- Ankylosing spondylitis.<br>- Reactive arthritis (Reiter's syndrome).<br>- Undifferentiated spondyloarthritis.<br>- Psoriatic arthritis.<br>- Enteropathic arthritis.<br>• Relapsing polychondritis.<br>• Mixed connective tissue disorder.<br>• Gout.	• Psoriasis.<br>• Vitiligo.<br>• Erythema nodosum.<br>• Autoimmune bullous skin diseases (including pemphigus, pemphigoid, and dermatitis herpetiformis).<br>• Lichen planus.<br>• Sweet's syndrome.<br>• Localized scleroderma (morphea).<br>• Cutaneous lupus erythematosus.<br>• Rosacea.

Vasculitis	Blood disorders	Others
• Large vessels vasculitis including:<br>- Giant cell arteritis (temporal arteritis).<br>- Takayasu's arteritis.<br>• Medium sized and/or small vessels vasculitis including:<br>- Polyarteritis nodosa.<br>- Kawasaki's disease.<br>- Microscopic polyangiitis.<br>- Wegener's granulomatosis (granulomatosis with polyangiitis).	• Autoimmune hemolytic anemia.<br>• Autoimmune thrombocytopenia.<br>• Antiphospholipid syndrome.<br>• Pernicious anemia.<br>• Autoimmune aplastic anemia.<br>• Autoimmune neutropenia.<br>• Autoimmune pancytopenia.	• Autoimmune glomerulonephritis including:<br>- IgA nephropathy.<br>- Glomerulonephritis rapidly progressive.<br>- Membranous glomerulonephritis.<br>- Membranoproliferative glomerulonephritis.<br>- Mesangioproliferative glomerulonephritis.<br>- Tubulointerstitial nephritis and uveitis syndrome.

Vasculitis (continued)	Blood disorders (continued)	Others (continued)
- Churg–Strauss syndrome (allergic granulomatous angiitis or eosinophilic granulomatosis with polyangiitis).<br>- Buerger's disease (thromboangiitis obliterans).<br>- Necrotizing vasculitis (cutaneous or systemic).<br>- Antineutrophil cytoplasmic antibody (ANCA) positive vasculitis (type unspecified).<br>- Henoch-Schonlein purpura (IgA vasculitis).<br>- Behcet's syndrome.<br>- Leukocytoclastic vasculitis.		• Ocular autoimmune diseases including:<br>- Autoimmune uveitis.<br>- Autoimmune retinitis.<br>• Autoimmune myocarditis/cardiomyopathy.<br>• Sarcoidosis.<br>• Stevens-Johnson syndrome.<br>• Sjögren's syndrome.<br>• Alopecia areata.<br>• Idiopathic pulmonary fibrosis.<br>• Goodpasture syndrome.<br>• Raynaud's phenomenon.
**Liver disorders**	**Gastrointestinal disorders**	**Endocrine disorders**
• Autoimmune hepatitis.<br>• Primary biliary cirrhosis.<br>• Primary sclerosing cholangitis.<br>• Autoimmune cholangitis.	• Inflammatory bowel disease, including:<br>- Crohn's disease.<br>- Ulcerative colitis.<br>- Microscopic colitis.<br>- Ulcerative proctitis.<br>• Celiac disease.<br>• Autoimmune pancreatitis.	• Autoimmune thyroiditis (including Hashimoto thyroiditis).<br>• Grave's or Basedow's disease.<br>• Diabetes mellitus type 1.<br>• Addison's disease.<br>• Polyglandular autoimmune syndrome.<br>• Autoimmune hypophysitis.

19.5 Appendix 5 – Symptoms, Risk Factors, and Disease Severity of COVID-19

Table 11 Symptoms and Risk Factors associated with COVID-19

**Clinical Presentation**	Presenting signs and symptoms of COVID-19 vary.
	Most persons experience fever (83–99%), cough (59–82%), fatigue (44–70%), anorexia (40–84%), shortness of breath (31–40%), myalgias (11–35%). Other non-specific symptoms, such as sore throat, nasal congestion, headache, diarrhoea, nausea and vomiting, have also been reported. Loss of smell (anosmia) or loss of taste (ageusia) preceding the onset of respiratory symptoms has also been reported.
	Older people and immunosuppressed patients in particular may present with atypical symptoms such as fatigue, reduced alertness, reduced mobility, diarrhoea, loss of appetite, delirium, and absence of fever.
	Symptoms such as dyspnoea, fever, gastrointestinal (GI) symptoms or fatigue due to physiologic adaptations in pregnant women, adverse pregnancy events, or other diseases such as malaria, may overlap with symptoms of COVID-19.
**Risk Factors for Severe Disease**	Age more than 60 years (increasing with age).
	Underlying noncommunicable diseases (NCDs): diabetes, hypertension, cardiac disease, chronic lung disease, cerebrovascular disease, chronic kidney disease, immunosuppression and cancer have been associated with higher mortality.
	Smoking.

Source: [WHO 2020a]

Table 12 COVID-19 Disease Severity

**Mild Disease**		Symptomatic patients (Table 11) meeting the case definition for COVID-19 without evidence of viral pneumonia or hypoxia.
**Moderate Disease**	**Pneumonia**	**Adult** with clinical signs of pneumonia (fever, cough, dyspnoea, fast breathing) but no signs of severe pneumonia, including $SpO_2 \geq 90\%$ on room air.<br>While the diagnosis can be made on clinical grounds; chest imaging (radiograph, CT scan, ultrasound) may assist in diagnosis and identify or exclude pulmonary complications.
**Severe Disease**	**Severe Pneumonia**	**Adult** with clinical signs of pneumonia (fever, cough, dyspnoea, fast breathing) plus one of the following: respiratory rate<br>> 30 breaths/min; severe respiratory distress; or SpO2 < 90% on room air.<br>While the diagnosis can be made on clinical grounds; chest imaging (radiograph, CT scan, ultrasound) may assist in diagnosis and identify or exclude pulmonary complications.
**Critical Disease**	**Acute Respiratory Distress Syndrome (ARDS)**	**Onset**: within 1 week of a known clinical insult (i.e. pneumonia) or new or worsening respiratory symptoms.<br>**Chest imaging**: (radiograph, CT scan, or lung ultrasound): bilateral opacities, not fully explained by volume overload, lobar or lung collapse, or nodules.<br>**Origin of pulmonary infiltrates**: respiratory failure not fully explained by cardiac failure or fluid overload. Need objective assessment (e.g. echocardiography) to exclude hydrostatic cause of infiltrates/oedema if no risk factor present.<br>**Oxygenation impairment in adults**:<br>• Mild ARDS: 200 mmHg < PaO_2/FiO_2[a] ≤ 300 mmHg (with PEEP or CPAP ≥ 5 cm H_2O).[b]<br>• Moderate ARDS: 100 mmHg < PaO_2/FiO_2 ≤ 200 mmHg (with PEEP ≥ 5 cmH_2O).[b]<br>• Severe ARDS: PaO_2/FiO_2 ≤ 100 mm Hg (with PEEP ≥ 5 cmH_2O).[b]
**Critical Disease**	**Sepsis**	**Adults**: acute life-threatening organ dysfunction caused by a dysregulated host response to suspected or proven infection. Signs of organ dysfunction include: altered mental status, difficult or fast breathing, low oxygen saturation, reduced urine output (3), fast heart rate, weak pulse, cold extremities or low blood pressure, skin mottling, laboratory evidence of coagulopathy, thrombocytopenia, acidosis, high lactate, or hyperbilirubinemia.
	**Septic Shock**	**Adults**: persistent hypotension despite volume resuscitation, requiring vasopressors to maintain MAP ≥ 65 mmHg and serum lactate level > 2 mmol/L.

Other complications that have been described in COVID-19 patients include acute, life-threatening conditions such as: acute pulmonary embolism, acute coronary syndrome, acute stroke and delirium. Clinical suspicion for these complications should be heightened when caring for COVID-19 patients, and appropriate diagnostic and treatment protocols available.

[a] If altitude is higher than 1000 m, then the correction factor should be calculated as follows: PaO_2/FiO_2 x barometric pressure/760.

[b] When PaO_2 is not available, $SpO_2/FiO_2 \leq 315$ suggests ARDS (including in non-ventilated patients).

Abbreviations: CPAP continuous positive airway pressure; CT computed tomography; FiO_2 fraction of inspired oxygen; MAP mean arterial pressure; OSI Oxygenation Index using SpO_2; PaO_2 partial pressure arterial oxygen; PEEP positive end-expiratory pressure; SD standard deviation;

Source: [WHO 2020a]

19.6 Appendix 6 – COVID-19 Diary Sample Pages

DIARY FOR COVID-19 SYMPTOMS	
**Study Name**	A Randomized, Observer-Blind, Placebo-Controlled, Phase 2/3 Study to Assess the Safety, Efficacy, and Immunogenicity of a Recombinant Coronavirus-Like Particle COVID-19 Vaccine in Adults 18 Years of Age or Older
**Protocol Number**	CP-PRO-CoVLP-021
**Sponsor**	Medicago R&D Inc.<br>1020, route de l'Église, suite 600<br>Québec, QC, Canada G1V 3V9
**Clinical site Address**	
**Principal Investigator Name**	
**Clinical Research Coordinator**	
**24-hour Emergency Number**	

SAMPLE ONLY. DO NOT USE

INSTRUCTIONS

The Diary for COVID-19 symptoms is to help you keep track of information needed during this study, when you are experiencing such symptoms. It should not be considered to replace data entry into your Memory Aid. This document will be collected by the site staff, and you should have it in hand for any contact with the study team, to provide verbally with information noted.

- Grayed out sections are to be left blank and will be completed by the site personnel.
- To complete this document, use blue or black ink. No pencil or liquid paper is allowed.
- If you made a mistake, cross out the incorrect information, initial and date, and write the correct answer beside.
- Date should be recorded with 2 digits for day, 3 letters for month and 4 digits for year

January	JAN	April	APR	July	JUL	October	OCT
February	FEB	May	MAY	August	AUG	November	NOV
March	MAR	June	JUN	September	SEP	December	DEC

SAMPLE ONLY. DO NOT USE

You will need to refer to this Diary for COVID-19 symptoms during the telephone contacts.

- Record below COVID-19 symptoms.
- Any medication taken for those symptoms should be recorded in your Memory Aid.

You received the second study vaccine on: |__|__|__|__|__|__|__|__|__| at: |__|__| : |__|__| **Initial and date:** |__|__|__|__|__|__|__|__|__|
dd mmm yyyy hh mm dd mmm yyyy

COVID-19 SYMPTOMS

From Day 0 after your first vaccination to the end of the study, record your symptoms in your Memory Aid when you experience one of the following. Call the site as soon as possible. After your visit to the site, you will have to record your COVID-19 symptoms (daily) and collect swabs (every other day) for 2 weeks.

Indicate the grade (refer to table presented on the following page) of each symptom you have.

Date	Fever*	Chills	Cough	Shortness of breath or difficulty breathing	Fatigue	Muscle or body aches	Headache	Congestion or runny nose	Sore throat	Nausea	Vomiting	Diarrhea	New loss of taste	New loss of smell	
Example<br>10-Nov-2020	100.6F	3	2	0	0	0	1	0	3	0	0	0	0	1	

SAMPLE ONLY. DO NOT USE

* Fever: indicate your oral temperature if ≥ 38.0 ºC or ≥ 100.4 °F

ASSESSMENT OF COMMON COVID-19-RELATED SYMPTOMS: GRADES	
What was the severity of your symptom at its worst over the last 24 hours?	Response options and scoring
Chills or shivering	None = 0<br>Mild = 1<br>Moderate = 2<br>Severe = 3<br><br>**0**= No symptom; **1**= Does not interfere with activity; **2**= Repeated use of non-narcotic pain reliever >24 hours or interferes with activity but does not require medical intervention; **3**= Any use of narcotic pain reliever or prevents daily activity and does require medical intervention.
Cough	
Shortness of breath (difficulty breathing)	
Fatigue	
Muscle or body ache	
Headache	
Congestion or runny nose	
Sore throat	
Nausea (feeling like you wanted to throw up)	
How many times did you vomit (throw up) in the last 24 hours?	I did not vomit at all = 0<br>1–2 times = 1<br>3–4 times = 2<br>5 or more times = 3
How many times did you have diarrhea (loose or watery stools) in the last 24 hours?	I did not have diarrhea at all = 0<br>1–2 times = 1<br>3–4 times = 2<br>5 or more times = 3
Rate your sense of smell in the last 24 hours:	My sense of smell is THE SAME AS usual = 0<br>My sense of smell is LESS THAN usual = 1<br>I have NO sense of smell = 2
Rate your sense of taste in the last 24 hours:	My sense of taste is THE SAME AS usual = 0<br>My sense of taste is LESS THAN usual = 1<br>I have NO sense of taste = 2

SAMPLE ONLY. DO NOT USE

Adapted from: https://www.fda.gov/regulatory-information/search-fda-guidance-documents/assessing-covid-19-related-symptoms-outpatient-adult-and-adolescent-subjects-clinical-trials-drugs

19.7 Appendix 7 – IDMC Meetings and Safety Data Review

The IDMC meetings and review of safety data for the Phase 2 and Phase 3 portions are outlined in Table 13 and Table 14, respectively. As each portion of the study progresses, the timing, content, and the number of meetings may change from that described in Table 13 and Table 14. The IDMC Charter should be referred to for the latest and up-to-date information regarding the IDMC meetings.

Table 13 Schedule of IDMC Meetings and Safety Data Review for Phase 2 Portion

Meeting #	Meeting context	Protocol Reference for meeting trigger	Raw Data Extract	Programmed Tables and Listings	Immunogenicity Data
1	Study population #2/ Healthy Elderly Adults<br>Vaccination 1: 7-day safety data for 10 sentinel subjects including lab data	Section 4.1 of the protocol and synopsis section titled Safety review	Open Session (Blinded listing)<br>A) Solicited listing<br>B) Unsolicited Listing<br>C) Clinically Significant abnormal (Lab, Vital Sign)<br><br>Closed Session (Unblinded listing)<br>A) Solicited listing<br>B) Unsolicited Listing<br>C) Clinically Significant abnormal (Lab, Vital Sign)<br>D) Covid-19 cases by treatment arm with severity provide by Investigator and unblinded Medical Monitor	Open (blinded) and closed Session (Unblinded) outputs for following AESIs<br><br>A) pIMD<br>B) VAED<br>C) Hypersensitivity	Not applicable

Meeting #	Meeting context	Protocol Reference for meeting trigger	Raw Data Extract	Programmed Tables and Listings	Immunogenicity Data
2	Study population #3/ Comorbid<br>Vaccination 1: 7-day safety data for 10 sentinel subjects including lab data	Section 4.1 of the protocol and synopsis section titled Safety review	Open Session (Blinded listing)<br>A) Solicited listing<br>B) Unsolicited Listing<br>C) Clinically Significant abnormal (Lab, Vital Sign)<br><br>Closed Session (Unblinded listing)<br>A) Solicited listing<br>B) Unsolicited Listing<br>C) Clinically Significant abnormal (Lab, Vital Sign)<br>D) Covid-19 cases by treatment arm with severity provide by Investigator and unblinded Medical Monitor	Open (blinded) and closed Session (Unblinded) outputs for following AESIs<br><br>A) pIMD<br>B) VAED<br>C) Hypersensitivity	Not applicable
3	Study population # 2/ Healthy Elderly Adults<br>Vaccination 2: day 28 for 10 sentinel subjects.<br>Vaccination 1: Any cumulative safety data for all subjects	Section 4.1 and synopsis section titled Safety review	Not Applicable	Open (blinded) and closed Session (Unblinded). Please see section 12.2 and 12.3 of the charter for detailed information on the outputs in shells<br><br>A) Open session (3 Tables and listings) per IDMC mock shells<br>B) Closed session (9 Listing and 5 Tables) per IDMC mock shells this includes solicited, unsolicited and Clinically Significant abnormal (Lab, Vital Sign)	Not applicable
4	Study population #3/ Comorbid<br>Vaccination 2: day 28 for 10 sentinel subjects.	Section 4.1 and synopsis section titled Safety review	Not Applicable	Open (blinded) and closed Session (Unblinded). Please see section 12.2 and 12.3 of the IDMC charter for detailed information on the outputs in shells	Not applicable

Meeting #	Meeting context	Protocol Reference for meeting trigger	Raw Data Extract	Programmed Tables and Listings	Immunogenicity Data
	Vaccination 1: Any cumulative safety data for all subjects			A) Open session (3 Tables and listings) per IDMC mock shells<br>B) Closed session (9 Listing and 5 Tables) per IDMC mock shells this includes solicited, unsolicited and Clinically Significant abnormal (Lab, Vital Sign)	
5	Study Population #1/ Healthy Adults<br>Vaccination 1 and 2: Day 28 safety<br><br>***Go/No go for this population for MHRA and FDA***	Synopsis section titled Safety review and section 12.4 of the protocol	Not Applicable	Open (blinded) and closed Session (Unblinded). Please see section 12.2 and 12.3 of the IDMC charter for detailed information on the outputs in shells<br><br>A) Open session (3 Tables and listings) per IDMC mock shells<br>B) Closed session (9 Listing and 5 Tables) per IDMC mock shells this includes solicited, unsolicited and Clinically Significant abnormal (Lab, Vital Sign)	Day 21 Nab immunogenicity data for population #2 (see section 12.3 of the protocol)
6	Study Population #1/ Healthy Adults<br>Vaccination 1 and 2: Day 28 safety<br><br>***Go/No go for this population for Health Canada***	Synopsis section titled Safety review and section 12.4 of the protocol	Not Applicable	Open (blinded) and closed Session (Unblinded). Please see section 12.2 and 12.3 of the IDMC charter for detailed information on the outputs in shells<br><br>A) Open session (3 Tables and listings) per IDMC mock shells<br>B) Closed session (9 Listing and 5 Tables) per IDMC mock shells this includes solicited, unsolicited and Clinically	Day 42 Nab immunogenicity data for population #1 (see section 12.3 of the protocol)

Meeting #	Meeting context	Protocol Reference for meeting trigger	Raw Data Extract	Programmed Tables and Listings	Immunogenicity Data
				Significant abnormal (Lab, Vital Sign)	
7	~100 subjects in Healthy Elderly Adults (Population # 2): Cumulative safety and Day 21 Immunogenicity<br><br>***Go/No go for this population for MHRA and FDA***	Synopsis section titled Safety review and section 12.4 of the protocol	Not Applicable	Open (blinded) and closed Session (Unblinded). Please see section 12.2 and 12.3 of the IDMC charter for detailed information on the outputs in shells<br><br>A) Open session (3 Tables and listings) per IDMC mock shells<br>B) Closed session (9 Listing and 5 Tables) per IDMC mock shells this includes solicited, unsolicited and Clinically Significant abnormal (Lab, Vital Sign)	Day 21 Nab immunogenicity data for population # 2<br>(see section 12.3 of the protocol)
8	~100 subjects in Healthy Elderly Adults (Population # 2): Day 28 Safety and Day 42 Immunogenicity<br><br>***Go/No go for this population for Health Canada***	Synopsis section titled Safety review and section 12.4 of the protocol		Open (blinded) and closed Session (Unblinded). Please see section 12.2 and 12.3 of the IDMC charter for detailed information on the outputs in shells<br><br>A) Open session (3 Tables and listings) per IDMC mock shells<br>B) Closed session (9 Listing and 5 Tables) per IDMC mock shells this includes solicited, unsolicited and Clinically	Day 42 Nab immunogenicity data for population # 2<br>(see section 12.3 of the protocol)

Meeting #	Meeting context	Protocol Reference for meeting trigger	Raw Data Extract	Programmed Tables and Listings	Immunogenicity Data
				Significant abnormal (Lab, Vital Sign)	
9	~100 subjects in Study Population # 3<br>Vaccination 1 and 2: Cumulative Safety data and Day 21 Immunogenicity data<br><br>***Go/No go for this population for MHRA and FDA***	Synopsis section titled Safety review and section 12.4 of the protocol	Not Applicable	Open (blinded) and closed Session (Unblinded). Please see section 12.2 and 12.3 of the IDMC charter for detailed information on the outputs in shells<br><br>A) Open session (3 Tables and listings) per IDMC mock shells<br>B) Closed session (9 Listing and 5 Tables) per IDMC mock shells this includes solicited, unsolicited and Clinically Significant abnormal (Lab, Vital Sign)	Day 21 Nab immunogenicity data for population # 3<br>(see section 12.3 of the protocol)
10	~100 subjects in Study Population #3/ Comorbid<br>Vaccination 1 and 2: Day-28 Safety data and Day 42 Immunogenicity data	Synopsis section titled Safety review and section 12.4 of the protocol	Not Applicable	Open (blinded) and closed Session (Unblinded). Please see section 12.2 and 12.3 of the IDMC charter for detailed information on the outputs in the shells<br><br>A) Open session (3 Tables and listings) per IDMC mock shells<br>B) Closed session (9 Listing and 5 Tables) per IDMC mock shells	Day 42 Nab immunogenicity data for population # 3 (see section 12.3 of the protocol)

Table 14 Schedule of IDMC Meetings and Safety Data Review for Phase 3 Portion

**Phase 3**			
**Meeting context**	**Protocol Reference for meeting trigger**	**Type of Data (Efficacy)**	**Type of Data (Safety)**
Final Analysis at 160 lab confirmed Covid-19 Cases for success or Futility<br><br>*when **160** laboratory-confirmed COVID-19 cases have been collected, the **unblinded statistical team** will perform a final analysis and produce final efficacy outputs*	Section 12.3 of the protocol	A) Vaccine Efficacy based on calculation in section 12.7.1 for primary Efficacy<br>B) Vaccine Efficacy based on calculation in section 12.7.2 for secondary Efficacy	A) Data for Occurrences of COVID-19-related symptoms and their severity summarized by treatment using descriptive statistics as described in section 12.7.2 of the protocol<br>B) Data on distribution of severe Covid-19 between the CoVLP and placebo groups during the Final analysis as described in section 12.7.2 of the protocol

Amendment Reference Document

Amendment No. 10 to the Study Protocol

Study Number: CP-PRO-CoVLP-021

A Randomized, Observer-Blind, Placebo-Controlled, Phase 2/3 Study to Assess the Safety, Efficacy, and Immunogenicity of a Recombinant Coronavirus-Like Particle COVID-19 Vaccine in Adults 18 Years of Age or Older

---

Previous Protocol Version:	7.1
Current Protocol Version:	7.2
Name of Sponsor:	Medicago R&D Inc.<br>1020 route de l'Église, bureau 600<br>Québec (QC), Canada G1V 3V9
Date:	25 January 2022

TABLE OF CONTENTS

TABLE OF CONTENTS 2
LIST OF TABLES 2
1 CONSEQUENCE OF CURRENT AMENDMENT 3
2 SIGNATURES 4
3 BACKGROUND FOR THE AMENDMENTS 5
4 ADMINISTRATIVE AND GCP REQUIREMENTS 6
5 SUMMARY OF KEY CHANGES 6
5.1 Current Amendment 7
5.2 Previous Amendments 21

LIST OF TABLES

Table 1: Summary of Changes from Protocol Version 7.1 to Version 7.2 (Effective Date 26 January 2022) 7
Table 2 Summary of Changes from Protocol Version 7.0 to Version 7.1 (Effective Date 28 October 2021) 8
Table 3 Summary of Changes from Protocol Version 6.0 to Version 7.0 (Effective Date 16 May 2021) 21
Table 4 Summary of Changes from Protocol Version 5.2 to Version 6.0 (Effective Date 26 April 2021) 68
Table 5 Summary of Changes from Protocol Version 5.1 to Version 5.2 (Effective Date 23 March 2021) 96
Table 6 Summary of Changes from Protocol Version 5.0 to Version 5.1 (Effective Date 11 March 2021) 98
Table 7 Summary of Changes from Protocol Version 4.0 to Version 5.0 (Effective Date 06 February 2021) 98
Table 8 Summary of Changes from Protocol Version 3.0 to Version 4.0 (Effective Date 17 December 2020) 153
Table 9 Summary of Changes from Protocol Version 2.0 to Version 3.0 (Effective Date 06 November 2020) 192
Table 10 Summary of Changes from Protocol Version 1.0 to Version 2.0 (Effective Date 06 October 2020) 241

1 CONSEQUENCE OF CURRENT AMENDMENT

	No	Yes
Applicable to all Investigators	X	
Involves only logistical or administrative aspects	X	
IEC /IRB vote expected		X
CRF needs modification	X	
Informed consent needs modification	X	
Regulatory notification required		X
Statistical analysis plan (SAP) needs modification		X

2 SIGNATURES

[Redacted] Date (YYYY-MM-DD)

[Redacted] Date (YYYY-MM-DD)

[Redacted] Date (YYYY-MM-DD)

[Redacted] Date (YYYY-MM-DD)

[Redacted] Date (YYYY-MM-DD)

3 BACKGROUND FOR THE AMENDMENTS

Amendment No. 10 has been generated to add precision to the protocol regarding the timing of the Phase 2 and Phase 3 study subject level treatment unblind. The subject level unblinding will be done only after the PVE analysis is completed, and may occur prior to the initiation of Period 2 (vaccination of placebo recipients) after first RA/A.

Amendment No. 09 has been generated to add precision to the current protocol (version 7.0) around the key elements of the type of regulatory submission for the CoVLP vaccine, subject eligibility for an approved or authorized COVID-19 vaccine, handling of subjects who received only a single dose of the CoVLP formulation, and contraindications to a second dose of the study treatments or two doses of the CoVLP formulation.

Amendment No. 08 was generated to add precision to the current protocol (version 6.0) around the key elements of study design such as removal of the cross-over design from the Phase 3 portion of the study and unblinding the Phase 2 and Phase 3 portions once the CoVLP vaccine obtains Interim Order Authorization (IOA) or Emergency Use Authorization (EUA). The study design updates also detailed how and when subjects in the placebo group will be offered the CoVLP vaccine after IOA/EUA is obtained.

Amendment No. 07 was generated to add precision to the current protocol (version 5.2) around the key elements of efficacy and immunogenicity assessments, unblinding requests from subjects who would like to receive an approved or authorized COVID-19 vaccine, and updates to the schedule of the Independent Data Monitoring Committee (IDMC) meetings and safety data reviews.

Amendment No. 06 was generated to add precision to the current protocol (version 5.1) around the key element of the enrollment of subjects in the Phase 2 portion of the study.

Amendment No. 05 was generated to add precision to the current protocol (version 5.0) around the key element of the description of the investigational product.

Amendment No. 04 was generated to add precision to the current protocol (version 4.0) around the key element of study design (addition of the cross-over design to the Phase 3 portion of the study), immunogenicity sample collection, and the evaluation of the efficacy endpoints. In addition, the schedule of the IDMC meetings and safety data reviews was updated.

Amendment No. 03 was generated to add precision to the current protocol (version 3.0) around the key element of Study Populations (enrollment of adults with comorbidities in the US), assessment of solicited adverse events (AEs), and the evaluation of endpoints after the second vaccination, based upon feedback received from Center for Biologics Evaluation and Research (CBER). In addition, details for the IDMC review meetings and study stopping rules were clarified.

Amendment No. 02 was generated to add precision to the current protocol (version 2.0) around the key element of the Day 21 and Day 42 immunogenicity data analysis as well as remove content about the vaccination of children and adolescents (Study Population #2), adults with comorbidities (Study Population #4), and adult females in the third trimester of their low-risk

pregnancy (Study Population #5) as these Study Populations will no longer be vaccinated in this study. In addition, the sample size of each Study Population in the Phase 2 portion was increased to vaccinate more subjects and an additional immunogenicity blood sample will be collected three months after the last vaccination.

Amendment No. 01 was generated to add precision to the previous protocol (version 1.0) around key elements of efficacy evaluation and assessments, immunogenicity assessments, safety evaluations, separate study population for pregnant women in the Phase 3 portion, and mitigation strategies for dose adjustment for some study populations after discussions with Biologic and Radiopharmaceutical Drugs Directorate (BRDD). In addition, a figure displaying the flow of subject enrollment in the study was included.

4 ADMINISTRATIVE AND GCP REQUIREMENTS

The study manager and monitors are responsible for the distribution of this amendment to the principal investigators of each participating center.

Each Principal Investigator must sign the amendment and is responsible for the distribution of the amendment and the consolidated protocol to all staff concerned in his/her center.

5 SUMMARY OF KEY CHANGES

All changes to the study protocol with their justification are summarized in Table 1 (Amendment No. 10), Table 2 (Amendment No. 09), Table 3 (Amendment No. 08), Table 4 (Amendment No. 07), Table 5 (Amendment No. 06), Table 6 (Amendment No. 05), Table 7 (Amendment No. 04), Table 8 (Amendment No. 03), Table 9 (Amendment No. 02), and Table 10 (Amendment No. 01).

Key changes are:

- Added precision to the protocol regarding the timing of the Phase 2 and Phase 3 study subject level treatment unblind.
- Add information clarifying that the subject level unblinding will be done only after the PVE analysis is completed, and may occur prior to the initiation of Period 2 (vaccination of placebo recipients) after first RA/A.

5.1 Current Amendment

Table 1: Summary of Changes from Protocol Version 7.1 to Version 7.2 (Effective Date 25 January 2022)

Section	Change (New text=red)	Rationale
Throughout document	Version changed to 7.2 from 7.1	New amendment
Throughout document	Date updated to 26 January 2022	New amendment
Synopsis-Study Rationale	~~the study will be unblinded~~ Period 2 (Table 3) may begin	New language regarding timing of the unblinding added
Synopsis-Statistical Methods-Phase 3 Portion	" ~~the study~~ Period 2 (Table 3) may begin ~~will be unblinded~~	New language regarding timing of the unblinding added
Section 1.5 Overall Rationale for the Study	"Upon obtaining the first RA/A in any country for the CoVLP formulation, Period 2 (Table 3) may begin ~~study will be unblinded~~	The main purpose of this is to allow the Sponsor to be unblinded at the subject treatment level in order to complete our responses to regulatory queries.
Table 3	"Time and Events Schedule for Period 2:"	Aligning Table 3 with changes
Section 4.1 Overview of Study Design	"in any country"	Additional clarification on the offering of CoVLP to placebo subjects after RA/A
Section 4.2.2 Blinding	~~"Once RA/A is obtained after the PVE~~ after the PVE analysis has been ~~fully~~ completed"	New language regarding timing of the unblinding added
Section 4.2.2 Blinding	"or free them from their study obligations to receive a deployed vaccine once RA/A is obtained in any country and Period 2 (Table 3) is initiated."	Additional language regarding subject decision making added
Section 6.2 Blinding	"~~RA/A~~ completion of the PVE analysis	New language regarding timing of the unblinding added
Section 6.2 Blinding	~~portions will be unblinded~~ studies may initiate Period 2 (Table 3). After the PVE analysis is completed and in preparation for Period 2, the study may be unblinded so that subjects may decide whether to participate in Period 2 after first RA/A.	Allows subjects to be unblinded so they can decide whether to participate in Period 2
Section 6.2 Blinding	completion of the PVE analysis ~~RA/A~~	New language regarding timing of the unblinding added
Section 6.2 Blinding	After the PVE analysis is completed and in preparation for Period 2, the Sponsor	Allows subjects to be unblinded so they can decide whether to participate in Period 2

	may be unblinded in order for Period 2 to be initiated immediately after first RA/A.	

Table 2 Summary of Changes from Protocol Version 7.0 to Version 7.1 (Effective Date 28 October 2021)

#	Section (page #)	Change	Rationale
1.	Throughout the protocol	The use of "Interim Order Authorization (IOA) or Emergency Use Authorization (EUA)" and the abbreviation "IOA/EUA" have been replaced with "Regulatory Authorization/Approval (RA/A)" and the abbreviation "RA/A" throughout the protocol.	The CoVLP submission categorization has changed from an Interim Order to NDS-CV and as a result the reference to the type of approval/authorization has been generalized.
2.	Synopsis (Study Rationale)<br>AND<br>Synopsis (Statistical Methods – Phase 3 Portion:	The sixth paragraph has been updated as follows:<br>Upon obtaining the **first** ~~IOA/EUA~~ **RA/A in any country** for the CoVLP formulation, the study will be unblinded to support the following:<br>1. Subjects in the placebo group will be offered the CoVLP formulation and will only be followed passively to identify symptomatic COVID-19 cases and for safety until the end of the study. A blood sample will be obtained immediately prior to the administration of the first dose of the CoVLP formulation for serologic analyses but no further blood samples will be collected in this group**. Placebo**	Clarified that this condition is based on the first regulatory approval/authorization that is received in any country. In addition, it was

#	Section (page #)	Change	Rationale
	Efficacy and Safety Analyses)<br>AND<br>Section 1.5 (Overall Rationale for the Study)<br>AND<br>Section 12.3 (Day 21 and Day 42 Immunogenicity Data Analysis and Efficacy and Safety Analyses)	**recipients who choose to receive an approved or authorized COVID-19 vaccine for which they are eligible instead of the CoVLP formulation will be withdrawn from the study**;<br>2. Subjects in the CoVLP formulation group will complete the study as planned with a blood draw at Day 201 to assess protection against serologically-confirmed COVID-19**, unless they decide to receive an approved or authorized COVID-19 vaccine for which they are eligible and thus will be withdrawn from the study**.	clarified that subjects have the option to be freed from the study so they can receive an approved or authorized COVID-19 vaccine.
3.	Synopsis (Study Design – Phase 2 Portion)<br>AND<br>Section 4.1 (Overview of Study Design)	The last two sentences of the last paragraph for the Phase 2 content have been updated as follows:<br>For active surveillance, subjects will be contacted once per week via **phone, text, or email** ~~the electronic diary~~ and asked about symptoms of COVID-19 disease. ~~Other methods of contact (phone, text, email) will only be used as a last resort to contact the subject.~~	Clarified the method of contact for active surveillance during the Phase 2 portion.
4.	Table 1 (Time and Events Schedule: General Information for the Phase 2 Portion)	The following updates have been made to the table:<br>1. The position of row “Randomization” has been moved and now placed below the row “Pregnancy test”.<br>2. The text for “Active Surveillance” in the row “Collection of COVID-19 symptoms through passive and active surveillance” has been updated as follows:<br>Active Surveillance: Between the day of the first vaccination (Day 0, post vaccination) and the end of the Phase 2 portion of the study, the subjects will be contacted once per week via **phone, text, or email** ~~the electronic diary~~ and asked	Updated the row entry to align with the study procedures. Also, clarified the method of contact for active surveillance

#	Section (page #)	Change	Rationale
		about symptoms of COVID-19. ~~Other methods of contact (phone, text, email) will only be used as a last resort to contact the subject.~~	during the Phase 2 portion.
5.	Table 3 (Time and Events Schedule: General Information for Placebo Subjects who will Receive the CoVLP Formulation Post-IOA/EUA in the Phase 2 and 3 Portions)	The following updates have been made to the table:<br>1. The title of the table has been updated to the following:<br>"Time and Events Schedule: General Information for Placebo Subjects who will Receive the CoVLP Formulation Post-~~IOA/EUA~~ **RA/A** in the Phase 2 and 3 Portions **and Subjects who Received a Single Dose of the CoVLP Formulation prior to RA/A**"<br>2. The column header "Post-IOA/EUA" has been updated to "Post-RA/A".<br>3. An "X" has been added to the cell for "Contraindications assessment" and "21 Days After First Vaccination (±2)".<br>4. Footnote 1 has been updated as follows: "~~This~~ **These** visit**s** will consist of assessing subjects for contraindications to the first **and second** CoVLP formulation vaccination**s**."<br>5. Footnote 6 has been updated as follows: "This blood sample will only be collected from ~~subjects~~ **placebo recipients who are going to receive two doses of the CoVLP formulation** in the Phase 3 portion**; this blood sample will not be collected from subjects who received a single dose of the CoVLP formulation prior to RA/A and are now going to receive a second dose of the CoVLP formulation post-RA/A**. If ~~IOA/EUA~~ **RA/A** occurs post-Day 201, then no blood sample will be collected since a blood sample to test for SARS-CoV-2 N antibodies will be collected on Day 201."<br>6. A footnote "**8**" has been added to the column "Post-RA/A" and the footnote text is as follows:<br>"**Subjects who received a single dose of the CoVLP formulation prior to RA/A will only proceed with the Post-RA/A visit for dosing and do not need to complete the 21 Days After First Vaccination visit. However, these subjects will proceed with the Monthly Calls and the Day 386 Telephone Contact.**"	Clarified that this schedule also applies to subjects who only receive a single dose of the CoVLP formulation prior to the regulatory authorization/approval of the CoVLP vaccine may be able to receive a second dose of the CoVLP formulation at the discretion of the Investigator. In addition, clarified when contraindications will be assessed.

#	Section (page #)	Change	Rationale
6.	Section 5.1 (Inclusion Criteria)	The inclusion criteria #3 has been updated as follows:<br>3. At Screening (Visit 1) and Vaccination (Visit 2), subject must have a body mass index (BMI) of:<br>For the Phase 2 portion of the study only:<br>• Study Populations #1 and #2: ≥ 18.5 and < 30 kg/m^2;<br>• **Study Population #3: no restrictions for BMI;**<br>For the Phase 3 portion of the study only:<br>• Study Populations #1 and #2: ≥ 18.5 and < 35 kg/m^2;<br>• **Study Population #3: no restrictions for BMI;**	Clarified that there are no specific limits for the BMI range for Study Population #3.
7.	Section 5.3 (Prior and Concomitant Therapy)	A new bullet has been added after the third bullet as follows:<br>• **Any concomitant medication used to treat COVID-19 symptoms;**	Clarified another condition when concomitant medication use should be documented.
8.	Section 6.2 (Blinding)	The eighth paragraph has been updated as follows:<br>As approved or authorized vaccines for COVID-19 become publicly available to subjects in some regions, subjects may request to become prematurely unblinded. In order to proceed with any such requests for unblinding in the Phase 2 or Phase 3 portion, the following two criteria must be met:<br>1. Subject is eligible for an approved or authorized COVID-19 vaccine in their local region, and<br>2. Subject has been approached or received notification by a healthcare provider**, or is eligible based on local rules and requirements,** to receive a publicly available approved or authorized COVID-19 vaccine. The notification must be shared with the Investigator.	Clarified that subject eligibility for an approved or authorized COVID-19 vaccine is not limited to being approached or notified by a healthcare provider.

#	Section (page #)	Change	Rationale
9.	Section 8.1 (Management of Samples)	The first and the second paragraphs have been updated as follows:<br>Blood samples for immunogenicity analysis will be collected from all subjects in the Phase 2 portion of the study and from a subset of subjects in the Phase 3 portion of the study. In the Phase 3 portion, additional immunogenicity samples will be collected at Day 0 and Day 42 from all subjects. ~~Should a subject be discontinued from the study, a sample will be drawn at the time of the final visit and sent to Medicago for analysis.~~ Bioanalysis of the immunogenicity blood samples will be conducted at central laboratories.<br>Between vaccination and the end of the surveillance period, nasal or NP swabs will be collected from any subject who reports any symptoms associated with COVID-19 after the start of active and passive surveillance at the discretion of the site Investigator; these swabs will be collected each time a subject reports such an event within the specified timeframe, so multiple collections may be required from some subjects. **The site Investigator's reason for not collecting swabs from such a subject must be clearly documented.** At least two swabs will be collected per subject per event (at least one from each nare). If the subject cannot visit the Investigator site, then the subject may receive a visit at home (or hospital, if applicable) to collect the nasal/NP swabs samples or be provided with self-administered nasal swabs for sample collection. Bioanalysis of the nasal or NP swabs will be conducted at the central laboratory as well as local laboratories, to mitigate the risk of long delays in shipping samples to the central virology laboratory (refer to Section 10.4.1 for more details).	Clarified that not all subjects who are discontinued from the study will have to provide a sample at the final visit since not all subjects will have a final visit as it could be a final telephone contact. In addition, clarified that when the Investigator believes that the collection of NP/nasal swabs is not needed, this decision must be documented.
10.	Section 8.2 (Collection of Samples)	The sixth and seventh paragraphs have been updated as follows:<br>In the Phase 2 portion of the study, pregnancy testing will be performed for all females of childbearing potential (in Study Populations #1 and #3). A serum pregnancy test will be performed during screening. A urine (dipstick or similar) pregnancy test will be performed at Day 0 prior to randomization and at Days 21 and 42. In Study Populations #1 and #3, females of childbearing potential must have negative serum pregnancy test result from screening and urine pregnancy test results from Day 0 **and Day 21** available prior to vaccination.<br>In the Phase 3 portion of the study, pregnancy testing will be performed for all females of childbearing potential (in Study Populations #1 and #3). A urine (dipstick or similar)	Clarified that urine pregnancy test results on Day 21 should also be available prior to vaccination.

#	Section (page #)	Change	Rationale
		pregnancy test will be performed at screening and at Day 0 prior to randomization and at Days 21 and 42. In Study Populations #1 and #3, females of childbearing potential must have negative urine pregnancy test results from screening and Day 0 **and Day 21** available prior to vaccination.	
11.	Section 10.1.6 (Surveillance for COVID-19 Cases)	The second bullet and the fifth and sixth sub-bullets of the third bullet have been updated and a new bullet has been added as follows:<br>• Passive surveillance: subjects will be instructed to contact the study site as soon as possible in case they experience symptoms of COVID-19, as defined in Section 10.4.1;<br>• Subjects will also be advised to inform the clinical site if they have tested positive for SARS-CoV-2 infection, with or without the presence of any symptoms associated with COVID-19, and will be asked to provide a documented test result. If/when the subject begins to experience symptoms associated with COVID-19, the process for the collection of symptoms and nasal or NP swabs (as noted below) will be followed;<br>• Active surveillance: **during the Phase 2 portion, subjects will be contacted once per week via phone, text, or email. During the Phase 3 portion,** the subjects will be contacted once per week via the electronic diary and asked about symptoms of COVID-19. Other methods of contact (phone, text, email) will only be used as a last resort to contact the subject;<br>• If a subject reports any symptom(s) of COVID-19:<br>• Collect information regarding COVID-19, including all applicable symptoms and the onset date:<br>• The onset date is defined as the first date when one or more of the symptoms occurred;<br>• Symptoms collected should also include the presence or absence, severity, and duration of concurrent symptoms (i.e. fever, feverishness [feeling of warmth], chills [shivering], tiredness [fatigue], headache, myalgia [muscle aches], nausea, vomiting, or diarrhea);<br>• Collect information on any associated concomitant medication use;	Clarified that subjects should be checking for any COVID-19 symptoms daily and recording this information in the memory aid until they visit the clinical site. In addition, clarified how subjects are contacted in Phase 2 vs Phase 3 in regards to active surveillance. Also clarified when swabs collection and daily reporting of symptoms should not occur.

#	Section (page #)	Change	Rationale
		• Schedule a time when the subject can return to the clinical site for the collection of nasal or NP swabs. If the subject cannot visit the Investigator site, then schedule a home (or hospital, if applicable) visit to collect the nasal/NP swabs samples or provide the subject with self-administering nasal swabs for sample collection. **Symptoms will be evaluated daily by the subject and reported in their memory aid until the subject visits the clinical site.** The Investigator will confirm whether nasal or NP swabs should be collected. The nasal or NP swabs are to be collected within 72 hours (preferably within 48 hours) after the reporting of a COVID-19 symptom;<br>Note: If swabs cannot be collected within 72 hours after reporting of the COVID-19-associated symptom, nasal or NP swabs should be collected at the next available opportunity for the subject to visit the Investigator site or at home (or hospital, if applicable) and date and time of collection recorded.<br>• At the same visit where the Investigator will confirm whether nasal or NP swabs should be collected, subjects will be trained on daily recording of their COVID-19-associated symptoms using a COVID-19 diary and will be asked to immediately start recording their symptoms in the COVID-19 diary. Subjects will collect any COVID-19-associated symptoms in their COVID-19 diary until a negative swab result for the subject is received from the central virology laboratory or until the subject has no more COVID-19-associated symptoms**. The Investigator's clinical judgement may be applicable to determine when COVID-19 disease has resolved or COVID-19-associated symptoms have resolved with some sequelae for a subject and when the collection of COVID-19-associated symptoms can be stopped**;<br>• COVID-19 cases that start on or after the day of the first vaccination (Day 0, post vaccination) will be followed up. This follow-up will be conducted via the planned active surveillance contacts. All follow-up information should be collected, regardless of whether nasal or NP swabs could be obtained. As part of this follow up, the following information will be needed (more details in Section 10.4.1):	

#	Section (page #)	Change	Rationale
		• Collect any new information regarding the evolution of the COVID-19 case, including symptoms and any associated concomitant medication use;<br>• Collect the results of the subject's serial (every other day) self-administered nasal swab testing results. The swab testing is to be continued for approximately 2 weeks. However, if the COVID-19-associated symptoms:<br>• Persist for more than 2 weeks, then the Investigator's clinical judgement is required to extend the collection of swabs beyond the 2 weeks;<br>• Have resolved in less than 2 weeks and the Investigator believes the subject has virologically recovered, then two consecutive negative results, obtained locally, are required prior to stopping the collection of the self-administered swabs;<br>• **If a subject has a positive COVID-19 case but does not present any COVID-19-associated symptoms, then there will be no collection of swabs from this subject nor the requirement to report daily COVID-19-associated symptoms. However, if this subject presents any COVID-19-associated symptoms, then the collection of swabs and reporting of symptoms can be started as described above;**<br>• Ensure subjects have the memory aid listing the symptoms of COVID-19 and contact information for the study site; ensure subjects use the diary or memory aid, as applicable, to record reportable information.	
12.	Section 10.1.15 (Placebo Subjects who Receive the CoVLP Formulation post-IOA/EUA for Phase 2 and Phase 3 portions)	The title and content of this section has been updates as follows:<br>Placebo Subjects who Receive the CoVLP Formulation post-**RA/A** ~~IOA/EUA~~ for Phase 2 and Phase 3 portions **and Subjects who Received a Single Dose of the CoVLP Formulation prior to RA/A**<br>For the Phase 2 and Phase 3 portions, placebo subjects who receive the CoVLP formulation post-**RA/A** ~~IOA/EUA~~ will stop following the planned study assessments (i.e. visits, contacts, surveillance) and will perform the following assessments instead.<br>**In addition, for subjects who only receive a single CoVLP formulation dose prior to RA/A, the Investigator will use his/her discretion to determine whether these**	Clarified how subjects who only receive a single dose of the CoVLP formulation prior to the regulatory authorization/approval of the CoVLP vaccine

#	Section (page #)	Change	Rationale
		**subjects should receive a second dose of the CoVLP formulation post-RA/A. Once a subject receives the second dose of the CoVLP formulation post-RA/A, then the subject will no longer follow the planned study assessments (i.e. visits, contacts, surveillance) and will perform the assessments detailed in Section 10.1.15.1, Section 10.1.15.1.1, Section 10.1.15.2, and Section 10.1.15.3 instead, with the following exceptions:**<br>• **Subjects will receive only one dose of CoVLP formulation;**<br>• **Subjects do not need to provide a blood sample prior to the administration of the CoVLP formulation.**	may be able to receive a second dose of the CoVLP formulation at the discretion of the Investigator and how these subjects will monitored in the study.
13.	Section 10.1.15.1 (Dose 1 and Dose 2 vaccinations with the CoVLP formulation)	A new paragraph has been added after the first paragraph as follows:<br>**Prior to the administration of dose 1 and dose 2 of the CoVLP formulation, subjects will be assessed for contraindications to the first and second CoVLP formulation vaccinations (refer to Section 11.3).**	The text has been updated to indicate that contraindications to vaccination will be assessed prior to each vaccination.
14.	Section 10.1.15.1.1 (Thirty Minutes Post-vaccination)	A new bullet point has been added to the list as follows:<br>The same procedures will be performed following each CoVLP formulation administration as will be performed following the administration of the study vaccine on Day 0 (see Section 10.1.4.3 for detailed procedures) for the Phase 2 and Phase 3 portions of the study, with the following exceptions:<br>• Non-immediate solicited local and systemic AEs (up to seven days after each vaccination) will not be collected;<br>• **Vital signs measurements will not be collected;**<br>• Provide appointments (date and time) for the monthly telephone calls and for the final study telephone call (Day 386);	Clarified that the collection of vital signs among placebo recipients who receive the CoVLP formulation is not required.

#	Section (page #)	Change	Rationale
		• The subject will be released from the clinical site once all post-vaccination procedures have been completed and the subject is in stable condition.	
15.	Section 11.3 (Contraindications for Subsequent Vaccination)	The content in this section has been updated as follows:<br>The following events constitute absolute contraindication to the further administration of the study treatments at Day 21 in the Phase 2 portion or Phase 3 portion~~, or to subjects in the placebo group who receive the CoVLP formulation post-IOA/EUA in the Phase 2 portion or Phase 3 portion~~:<br>• Pregnancy (see Section 10.2.2);<br>• Clinically-apparent hypersensitivity of any grade that is considered by the site Investigator to be related to the investigational product in response to first vaccine administration;<br>• Have a laboratory-confirmed SARS-CoV-2 infection**. However, a subject may proceed with the further administration of the study treatments at least one month after the resolutions of COVID-19 symptoms**;<br>• Acute disease within 48 hours prior to vaccination (acute disease is defined as presence of any moderate or severe acute illness with or without a fever)**. However, once the acute disease is resolved, then the subject may proceed with the further administration of the study treatments**;<br>• Diagnosis of pIMD in the context of an AESI and defined according to Section 13.1.3.2.1;<br>• In the Phase 2 portion of the study: Experience a possibly related, probably related, or definitely related SAE or Grade 3 or higher related AE that cannot be clearly attributed to another cause. The Investigator's opinion to administer the second dose of the vaccine will be presented to the IDMC for advice~~. Note: This event will not apply to subjects in the placebo group who receive their first dose of the CoVLP formulation~~;<br>• In the Phase 3 portion of the study: Experience a possibly related, probably related, or definitely related SAE that cannot be clearly attributed to another cause. ~~Note: This event will not apply to subjects in the placebo group who receive their first dose of the CoVLP formulation.~~	The contraindication section has been split so that one list addresses contraindications for the second dose of the study treatments to all subjects in Phase 2 and Phase 3. A second list of contraindications has been created to address contraindications for placebo recipients who will be receiving two doses of the CoVLP formulation.<br>In addition, it has been clarified that subjects who have a COVID-19 may still receive a second dose of the study

#	Section (page #)	Change	Rationale
		At the discretion of the Investigator, the following event constitutes contraindication to the further administration of the study treatments at Day 21 in the Phase 2 portion or Phase 3 portion, or to subjects in the placebo group who receive the CoVLP formulation post-**RA/A** ~~IOA/EUA~~ in the Phase 2 portion or Phase 3 portion:<br>• In the Phase 2 portion of the study: Discovery of any health condition which, in the Investigator's opinion, may place the subject at increased risk from receipt of further vaccination; or discovery of a change in the subject's status which may render him/her unable to comply with protocol-mandated safety follow-up. If an AE occurred prior to or at the time of the Day 21 scheduled visit, the subject may be discontinued at the discretion of the Investigator or the subject may be vaccinated at a later date, however, within the time window specified in the protocol (see Section **Error! Reference source not found.**). The subject must be followed until resolution of the event as with any AE (see Section **Error! Reference source not found.**).<br>• In the Phase 3 portion of the study: Discovery of any health condition which, in the Investigator's opinion, may place the subject at increased risk from receipt of further vaccination; or discovery of a change in the subject's status which may render him/her unable to comply with protocol-mandated safety follow-up (e.g. if a subject experienced possibly related, probably related, or definitely related Grade 3 AE after first vaccination that lasted more than 72 hours, Investigator should carefully evaluate the risk/benefit balance prior to administering the subsequent dose). If an AE occurred prior to or at the time of the Day 21 scheduled visit, the subject may be discontinued at the discretion of the Investigator or the subject may be vaccinated at a later date, however, within the time window specified in the protocol (see Section **Error! Reference source not found.**). The subject must be followed until resolution of the event as with any AE (see Section **Error! Reference source not found.**).<br>If any of the events listed above (both lists) occur during the study, the subject will not receive an additional dose of vaccine but will continue with the **Day 42 scheduled visit (see Section 10.1.11),** Day 128 scheduled visit (see Section 10.1.12; Phase 2 portion only), the Day 201 scheduled visit (see Section 10.1.13), and the Day 386 scheduled	treatment as long as their symptoms of COVID-19 are resolved. Similarly, subjects who have an acute disease but the condition is resolved may still receive a second dose of the study treatments.

#	Section (page #)	Change	Rationale
		visit/contact (see Section 10.1.14) of the respective portion at the discretion of the Investigator.<br>**The following events constitute absolute contraindication to the administration of the CoVLP formulation to subjects in the placebo group who might otherwise receive the CoVLP formulation post-RA/A in the Phase 2 portion or Phase 3 portion:**<br>• **Pregnancy (see Section 10.2.2);**<br>• **Clinically-apparent hypersensitivity of any grade that is considered by the site Investigator to be related to the investigational product in response to first CoVLP formulation administration;**<br>• **Have a laboratory-confirmed SARS-CoV-2 infection. However, a subject may proceed with the further administration of the study treatments at least one month after the resolutions of COVID-19 symptoms;**<br>• **Acute disease within 48 hours prior to vaccination (acute disease is defined as presence of any moderate or severe acute illness with or without a fever). However, once the acute disease is resolved, then the subject may proceed with the further administration of the CoVLP formulation;**<br>• **Diagnosis of pIMD in the context of an AESI and defined according to Section 13.1.3.2.1;**<br>• **In the Phase 2 portion of the study: Experience a possibly related, probably related, or definitely related SAE or Grade 3 or higher related AE that cannot be clearly attributed to another cause. The Investigator's opinion to administer the second dose of the CoVLP formulation will be presented to the IDMC for advice. Note: This event will not apply to placebo recipients (prior to RA/A) who receive their first dose of the CoVLP formulation post-RA/A;**<br>• **In the Phase 3 portion of the study: Experience a possibly related, probably related, or definitely related SAE that cannot be clearly attributed to another cause. Note: This event will not apply to placebo recipients (prior to RA/A) who receive their first dose of the CoVLP formulation post-RA/A.**	

#	Section (page #)	Change	Rationale
		**At the discretion of the Investigator, the following event constitutes contraindication to the administration of the CoVLP formulation to subjects in the placebo group who receive the CoVLP formulation post-RA/A in the Phase 2 portion or Phase 3 portion:**<br>• **In the Phase 2 portion of the study: Discovery of any health condition which, in the Investigator's opinion, may place the subject at increased risk from receipt of further vaccination; or discovery of a change in the subject's status which may render him/her unable to comply with protocol-mandated safety follow-up. If an AE occurred prior to or at the time of the first or second dose administration scheduled visits, the subject may be discontinued at the discretion of the Investigator or the subject may be vaccinated at a later date, however, within the time window specified in the protocol (see Section 10.1.15.1). The subject must be followed until resolution of the event as with any AE (see Section 13.1.2).**<br>• **In the Phase 3 portion of the study: Discovery of any health condition which, in the Investigator's opinion, may place the subject at increased risk from receipt of further vaccination; or discovery of a change in the subject's status which may render him/her unable to comply with protocol-mandated safety follow-up (e.g. if a subject experienced possibly related, probably related, or definitely related Grade 3 AE after first vaccination that lasted more than 72 hours, Investigator should carefully evaluate the risk/benefit balance prior to administering the subsequent dose). If an AE occurred prior to or at the time of the Day 21 scheduled visit, the subject may be discontinued at the discretion of the Investigator or the subject may be vaccinated at a later date, however, within the time window specified in the protocol (see Section 10.1.15.1). The subject must be followed until resolution of the event as with any AE (see Section 13.1.2).**<br>**If any of the events listed above (both lists) occur during the study, the subject may not receive the first and/or the second dose of the CoVLP formulation but will continue with the monthly calls (see Section 10.1.15.2) and the final telephone**	

#	Section (page #)	Change	Rationale
		**contact (see Section 10.1.15.3) of the respective portion at the discretion of the Investigator.**	
16.	Section 14.1.4 (Preparation, Handling, Storage, and Precautions for Use)	The first paragraph has been updated as follows:<br>The investigational products should be stored in an access-restricted area between 2 ºC and 8 ºC and protected from light; the vaccine should, however, be at room temperature before administration (i.e. the vaccine should not be administered cold and should be taken out of the refrigerator ~~at least five minutes~~ prior to administration **[refer to IPMM for details regarding time allowance]**).	The text has been updated since multi-dose vials are required to be removed from the refrigerator much sooner than 5 minutes.

5.2 Previous Amendments

Table 3 Summary of Changes from Protocol Version 6.0 to Version 7.0 (Effective Date 16 May 2021)

#	Section (page #)	Change	Rationale
1.	Synopsis (Study Rationale)<br>AND<br>Section 1.5 (Overall Rationale for the Study)	This section has been updated as follows:<br>This Phase 2/3 study is a multi-portion design to confirm that the chosen formulation and dosing regimen of CoVLP (i.e. two doses of 3.75 µg CoVLP adjuvanted with AS03 given 21 days apart) has an acceptable immunogenicity and safety profile in adults 18 years of age or older, including those with significant comorbidities (Phase 2 portion). The efficacy of this CoVLP formulation in these same Study Populations will be assessed in the Phase 3 portion.<br>The Phase 2 portion is a randomized, observer-blinded, placebo-controlled study to evaluate the CoVLP formulation, compared to placebo, in subjects from the three Study Populations:<br><table><tr><th>Study Population</th><th>Age and/or Health Category</th><th>Age Range (years)</th></tr><tr><td>1</td><td>Healthy adults</td><td>18-64</td></tr></table>	The cross-over will no longer be used in the Phase 3 portion. Instead, once the CoVLP vaccine obtains IOA/EUA, the CoVLP vaccine will be offered to all placebo subjects in the Phase 2 and Phase 3 portions.

<table>
<tr><th>#</th><th>Section (page #)</th><th>Change</th><th>Rationale</th></tr>
<tr><td></td><td></td><td>
<table>
<tr><td>2</td><td>Healthy elderly adults*</td><td>≥ 65</td></tr>
<tr><td>3</td><td>Adults with significant comorbidities*</td><td>≥ 18</td></tr>
</table>
*Subjects to be enrolled in country(ies) that has/have provided authorization to enroll these subjects in the study.

In each Study Population, 306 subjects will be randomized 5:1 to receive the CoVLP formulation:placebo. The total number of subjects in each Study Population may vary depending on when sufficient data has been accumulated to make a decision on when each Study Population can procced to the Phase 3 portion. Unless full enrollment (i.e., enrolled up to 306 subjects) has already been achieved, further enrollment into each Study Population will be closed once sufficient data has been accumulated to make the decision to proceed to Phase 3. All subjects will be followed for a period of 12 months after the last vaccination for durability of the immune responses, safety, and efficacy.

The Phase 3 portion is an event-driven, randomized, observer-blinded, placebo-controlled~~, 2-way cross-over~~ design that will evaluate the efficacy and safety of the CoVLP formulation, compared to placebo, in each of the Study Populations #1 - #3. Up to 30 000 subjects will be enrolled 1:1 to receive the CoVLP formulation or placebo.~~, according to the following treatment sequences presented in Table 6:~~

~~Table 6 Treatment Sequences in Phase 3 Portion~~
<table>
<tr><th>~~Treatment Sequence~~</th><th>~~Period 1~~</th><th>~~Period 2~~</th></tr>
<tr><td>~~1~~</td><td>~~CoVLP formulation~~</td><td>~~Placebo~~</td></tr>
<tr><td>~~2~~</td><td>~~Placebo~~</td><td>~~CoVLP formulation~~</td></tr>
</table>
~~In the 2-way cross-over design, subjects who receive the CoVLP formulation in Period 1 will receive the placebo in Period 2, while subjects who receive the placebo in Period 1 will receive the CoVLP formulation in Period 2. Initiation of the cross-over design for subjects enrolled in the study will be dependent on the following overall study and subject-specific criteria:~~

3. When Emergency Use Authorization or Approval for the CoVLP formulation is granted by any Regulatory Agency(ies); and
</td><td></td></tr>
</table>

medicago

#	Section (page #)	Change	Rationale
		4. When one or more authorized or approved COVID-19 vaccines become available for the subject in his/her country of recruitment; and<br>5. The subject has completed a minimum of 60 days in Period 1.<br>~~Emergency use authorization or approval will be pursued after the success criterion has been met for the primary efficacy endpoint of the Phase 3 portion. The primary efficacy endpoint will be evaluated once at least 160 laboratory-confirmed COVID-19 cases have been accumulated and the median safety follow-up duration of at least 2 months (post-administration of the second vaccination) of at least 3000 subjects in each of the CoVLP formulation and placebo groups has been achieved.~~<br>**The primary efficacy endpoint will be evaluated once at least 160 laboratory-confirmed COVID-19 cases (≥ 7 days post-second vaccination) have been accumulated and the median safety follow-up duration of at least 2 months (post-administration of the second vaccination) of at least 3000 subjects in each of the CoVLP formulation and placebo groups has been achieved. After the success criterion has been met for the primary efficacy endpoint of the Phase 3 portion, Interim Order Authorization (IOA) or Emergency Use Authorization (EUA) for the CoVLP formulation will be pursued.**<br>**Upon obtaining the IOA/EUA for the CoVLP formulation, the study will be unblinded to support the following:**<br>6. Subjects in the placebo group will be offered the CoVLP formulation and will only be followed passively to identify symptomatic COVID-19 cases and for safety until the end of the study. A blood sample will be obtained immediately prior to the administration of the first dose of the CoVLP formulation but no further blood samples will be collected in this group;<br>7. Subjects in the CoVLP formulation group will complete the study as planned with a blood draw at Day 201 to assess protection against serologically-confirmed COVID-19.<br>**Subjects in the placebo group who do not want to be administered the CoVLP formulation and still want to remain in the study will continue the study as planned with a blood draw at Day 201. Upon obtaining the IOA/EUA, the CoVLP formulation will be offered to placebo subjects in the Phase 2 portion of the study.**	

#	Section (page #)	Change	Rationale
		**Once the placebo subject receives the CoVLP formulation, the subject will only be followed for safety until the end of the study and no further blood draws will be collected. Subjects in the placebo group who do not wish to receive the CoVLP formulation or subjects who are in the CoVLP formulation group will continue the study as planned.**<br>~~All s~~Subjects **in the CoVLP formulation group** will be followed for efficacy **(i.e. active and passive surveillance)** and safety until **IOA/EUA and then for COVID-19 cases (i.e. passive surveillance only) and safety until the end of the study (Day 386)** ~~12 months after the last vaccination after the cross-over~~. **Subjects in the placebo group will be followed for efficacy (i.e. active and passive surveillance) and safety up until IOA/EUA and followed for safety and COVID-19 cases (i.e. passive surveillance only) until the end of the study.** The sero-status of all subjects will be assessed at the time of enrollment ~~and at the time of the cross-over~~. Serum samples will be obtained from all subjects at **Day 42** ~~21 days after the second CoVLP vaccination~~ so that a possible serologic correlate of protection can be evaluated once the primary efficacy outcome has been met and at later timepoints in the study. Only **the subjects who initially receive the CoVLP formulation or placebo subjects who do not want the CoVLP formulation among** the 288 subjects (96 subjects from each Study Population) in the immunogenicity subset will be assessed for the persistence of the vaccine-induced ~~humoral and~~ cellular immune responses until **Day 386** ~~12 months after the last study vaccination~~.	
2.	Synopsis (Planned Study Period)<br>AND<br>Section 4.2.5 (Study Duration)	The second paragraph has been updated as follows:<br>The **planned duration of th**e Phase 3 portion **is approximately 13 months, from screening procedures up to the end of the Phase 3 portion** ~~will consist of two periods, Period 1 and Period 2 (pre- and post-cross-over respectively), and the planned duration is up to approximately 26 months, from screening procedures up to the end of Period 2 in the Phase 3 portion~~.	The cross-over will no longer be used in the Phase 3 portion.
3.	Synopsis (Primary Objectives – Phase 2 Portion and Phase 3 Portion)	The primary efficacy objective of the Phase 3 portion has been updated as follows:<br>Efficacy:	The cross-over will no longer be used in the Phase 3 portion.

#	Section (page #)	Change	Rationale
	AND<br>Section 2.1 (Primary Objectives)	8. To evaluate the efficacy of the CoVLP formulation, compared to placebo, in the prevention of laboratory-confirmed symptomatic SARS-CoV-2 infection (virologic method) starting 7 days after the second vaccination at the time of the primary vaccine efficacy (PVE) analysis (i.e., once at least 160 COVID-19 cases [≥ 7 days post-second vaccination] have been collected and the median safety follow-up duration of at least 2 months (post-administration of the second vaccination) of at least 3000 subjects in each of the CoVLP formulation and placebo groups has been achieved) ~~and prior to the cross-over~~.	
4.	Synopsis (Secondary Objectives – Phase 2 Portion and Phase 3 Portion)<br>AND<br>Section 2.2 (Secondary Objectives)	The secondary immunogenicity and safety objectives of the Phase 2 portion and the efficacy, immunogenicity, and safety objectives of the Phase 3 portions have been updated as follows:<br>Phase 2 portion<br>Immunogenicity:<br>• To assess the relative neutralizing antibody response between the healthy adults (Study Population #1) and the healthy elderly populations (Study Population #2; each age strata) at 21 days after the second vaccination;<br>• To assess the relative neutralizing antibody response between the healthy adults (Study Population #1) and the healthy elderly adults (Study Population #2) combined and the adults with significant comorbidities (Study Population #3) at 21 days after the second vaccination;<br>• To assess the immunogenicity of the CoVLP formulation, compared to placebo**, when applicable,** in each of the Study Populations, as determined by the:<br>• Persistence of Nab titers induced by the vaccine against the SARS-CoV-2 virus at Days 128, 201, and Day 386;<br>• IgG (enzyme-linked immunosorbent assay [ELISA]) titers induced by the vaccine against the SARS-CoV-2 virus up to 21 days after the second vaccination and persistence of these antibodies at Day 128, Day 201, and Day 386;<br>• The ratio of Nab titers:IgG (ELISA) antibody titers at Day 21, Day 42, Day 128, Day 201, and Day 386;	The cross-over will no longer be used in the Phase 3 portion. Clarified the details of the timing of some of the analyses as well as the conditions for the analyses.

#	Section (page #)	Change	Rationale
		• IFN-γ ELISpot response induced by the vaccine against the SARS-CoV-2 virus at Day 201 and Day 386;<br>• Interleukin-4 (IL-4) ELISpot response induced by the vaccine against the SARS-CoV-2 virus up to 21 days after the second vaccination and at Day 201 and Day 386;<br>**Safety:**<br>• To assess the relative incidence of solicited local and systemic AEs up to 7 days after the first and second vaccinations between the healthy adults (Study Population #1) and each healthy elderly population (Study Population #2) **in the CoVLP formulation group prior to IOA/EUA only**;<br>• To assess the relative incidence of solicited local and systemic AEs up to 7 days after the first and second vaccinations between the healthy adults (Study Population #1) and the healthy elderly adults (Study Population #2) combined and the adults with significant comorbidities (Study Population #3) **in the CoVLP formulation group prior to IOA/EUA only**;<br>• To assess safety and tolerability of the CoVLP formulation, compared to placebo in each of the Study Populations , as determined by the occurrence(s) of:<br>• Medically attended adverse events (MAAEs), serious adverse events (SAEs), AEs leading to withdrawal, AEs of special interest (AESIs; including vaccine-**associated** enhanced disease [V**A**ED]), and deaths from 22 days after the second vaccination up to **IOA/EUA** ~~the end of the study~~;<br><br>Phase 3 portion<br>Efficacy:<br>9. To evaluate the efficacy of the CoVLP formulation~~, compared to placebo,~~ in the prevention of laboratory-confirmed asymptomatic SARS-CoV-2 infection at **Day 201** ~~cross-over and Day 386 in Period 1~~ in subjects who were seronegative at baseline (confirmed by serologic method);	

#	Section (page #)	Change	Rationale
		10. To evaluate the efficacy of the CoVLP formulation, compared to placebo, in the prevention of severe COVID-19 disease starting 7 days after the second vaccination at the time of the PVE analysis ~~and prior to the cross-over~~ (see definition of severe COVID-19 disease above);<br>11. To assess COVID-19-related symptoms in virologically-confirmed cases, and the severity of these symptoms up until resolution in subjects administered the CoVLP formulation ~~in Period 1~~ compared to subjects administered the placebo **at the time of the PVE analysis** ~~and compared to subjects administered CoVLP at the time of cross-over in Period 2~~;<br>12. To evaluate the efficacy of the CoVLP formulation, compared to placebo, in the prevention of laboratory-confirmed symptomatic SARS-CoV-2 infection (virologic method) starting after the first vaccination at the time of the PVE analysis ~~and prior to the cross-over~~;<br>13. To evaluate the efficacy of the CoVLP formulation, compared to placebo, in the prevention of laboratory-confirmed symptomatic SARS-CoV-2 infection (virologic method) after the first vaccination and prior to the second vaccination at the time of the PVE analysis~~, and prior to the cross-over~~;<br>14. To evaluate the efficacy of the CoVLP formulation, compared to placebo, in the prevention of laboratory-confirmed symptomatic SARS-CoV-2 infection (virologic method) after the second vaccination and prior to 7 days post-second vaccination at the time of the PVE analysis~~, and prior to the cross-over~~;<br>15. To assess the duration and intensity of viral shedding after virologically-confirmed SARS-CoV-2 infection in subjects administered the CoVLP formulation compared to subjects administered the placebo at the time of the PVE analysis ~~and prior to the cross-over~~;<br>16. To evaluate the efficacy of the CoVLP formulation, compared to placebo, in the prevention of laboratory-confirmed symptomatic SARS-CoV-2 infection (virologic method) by strain starting 7 days after the second vaccination at the time of the PVE analysis ~~and prior to the cross-over~~;<br>Immunogenicity:	

#	Section (page #)	Change	Rationale
		17. To assess the immunogenicity of the CoVLP formulation, compared to placebo **when applicable**, in the immunogenicity subset ~~prior to and post-cross-over~~, as determined by:<br>18. Nab titers induced by the vaccine against the SARS-CoV-2 virus at Day 0 (prior to vaccination; all subjects), Day 21, Day 42 (all subjects), Day 201 and Day 386;<br>19. IgG (ELISA) titers induced by the vaccine against the SARS-CoV-2 virus up to 21 days after the second vaccination and at Day 201 and Day 386;<br>20. The ratio of Nab titers:IgG (ELISA) antibody titers at Day 21, Day 42, Day 201 and Day 386;<br>21. IFN-γ ELISpot response induced by the vaccine against the SARS-CoV-2 virus up to 21 days after the second vaccination and at Day 201 and Day 386;<br>22. IL-4 ELISpot response induced by the vaccine against the SARS-CoV-2 virus up to 21 days after the second vaccination and at Day 201 and Day 386;<br>Safety:<br>23. To assess the safety and tolerability of the CoVLP formulation~~, compared to placebo,~~ up to the end of the study.	
5.	Synopsis (Exploratory Objectives – Phase 2 Portion and Phase 3 Portion)<br>AND<br>Section 2.3 (Exploratory Objectives)	The exploratory efficacy objectives of the Phase 3 portions have been updated as follows:<br>Phase 3 portion<br>Efficacy:<br>~~24. To evaluate the duration of protection (vaccine efficacy versus virologically-confirmed symptomatic disease as well as serologically-confirmed asymptomatic disease) of the CoVLP formulation in subjects initially randomized to the CoVLP group compared to the original placebo group for any strain and by strain, at the end of the study;~~<br>25. To evaluate the efficacy of the CoVLP formulation, compared to placebo, in the prevention of severe COVID-19 disease by strain starting 7 days after the second	The cross-over will no longer be used in the Phase 3 portion. Also, removed an efficacy objective that cannot be assessed since placebo subjects will be offered the CoVLP vaccine post-IOA/EUA.

<table>
<tr><th>#</th><th>Section (page #)</th><th>Change</th><th>Rationale</th></tr>
<tr><td></td><td></td><td>vaccination at the time of the PVE analysis ~~and prior to the cross-over~~ (see definition of severe COVID-19 disease above);

Immunogenicity:

26. To assess the immunogenicity of the CoVLP formulation, compared to placebo when applicable, in the immunogenicity subset ~~prior to and post-cross-over~~, as determined by:
27. Specific cell-mediated immune (CMI) response induced by the vaccine against the SARS-CoV-2 virus up to 21 days after the second vaccination and at Day 201 and Day 386, as measured by the percentage of CD4+ T cells expressing functional markers;
28. To further characterize the immune response of the CoVLP formulation, if it is likely to be informative;</td><td></td></tr>
<tr><td>6.</td><td>Synopsis (Dosage and Administration)
AND
Section 7 (Dosage and Administration)</td><td>The Phase 3 portion section has been updated as follows:

Each Study Population will receive the same dose of CoVLP adjuvanted with AS03 and injection volume established for that particular Study Population during the Phase 2 portion of the study.

~~In each period, s~~Subjects will receive two IM injections 21 days apart on Days 0 and 21, into the deltoid region of the alternating (if possible) arm (i.e., each arm will be injected once), of either the CoVLP formulation or the placebo.~~, according to the following treatment sequences:~~

<table><tr><th>~~Treatment Sequence~~</th><th>~~Period 1~~</th><th>~~Period 2~~</th></tr><tr><td>~~1~~</td><td>~~CoVLP formulation~~</td><td>~~Placebo~~</td></tr><tr><td>~~2~~</td><td>~~Placebo~~</td><td>~~CoVLP formulation~~</td></tr></table>
~~Subjects who receive the CoVLP formulation in Period 1 will receive the placebo in Period 2, while subjects who receive the placebo in Period 1 will receive the CoVLP formulation in Period 2.~~</td><td>The cross-over will no longer be used in the Phase 3 portion.</td></tr>
</table>

<table>
<tr><th>#</th><th>Section (page #)</th><th>Change</th><th>Rationale</th></tr>
<tr><td></td><td></td><td>Subjects in the placebo group will be offered the CoVLP formulation once IOA/EUA is obtained.</td><td></td></tr>
<tr><td>7.</td><td>Synopsis (Study Design)
AND
Section 4.1 (Overview of Study Design)</td><td>In the Phase 2 portion, the third and last paragraphs have been updated as follows:
All subjects will begin enrollment at the same time. To the greatest extent possible given local conditions (i.e. COVID-19 vaccine roll-out), e<s>E</s>nrollment of elderly adult subjects (Study Population #2) will be stratified by age in an approximately 2:1 ratio of 65 to 74 years of age and 75 years of age and older, respectively. Enrollment of subjects in Study Population #3 will include adults with co-existing conditions such as, but not limited to, obesity or being overweight, cardiovascular diseases, hyperlipidemia, chronic kidney disease or related disorders, chronic respiratory disease, pre-diabetes and diabetes (type 1 and type 2), and treatment-controlled HIV.

Active and passive surveillance will be conducted in the study from the day of the first vaccination (Day 0, post-vaccination) until 386 days. For passive surveillance, subjects will be instructed to report symptoms meeting the definition of new COVID-19 disease. For active surveillance, subjects will be contacted once per week via the electronic diary <s>subject's preferred method (e.g. phone, text, email, electronic diary),</s> and asked about symptoms of COVID-19 disease. Other methods of contact (phone, text, email) will only be used as a last resort to contact the subject.</td><td>Clarified the details regarding the enrollment of subjects and the method of contacting subjects during the surveillance period.</td></tr>
<tr><td>8.</td><td>Synopsis (Study Design)
AND
Section 4.1 (Overview of Study Design)</td><td>The Phase 3 portion has been updated as follows:
The Phase 3 portion is a randomized, observer-blinded, placebo-controlled, efficacy, safety, immunogenicity (in a subset of subjects), multicenter<s>, 2-way cross-over</s> design with male and female subjects. Similar to the Phase 2 portion, subjects will be enrolled from three Study Populations:
<table>
<tr><th>Study Population</th><th>Age and/or Health Category</th><th>Age Range (years)</th></tr>
<tr><td>1</td><td>Healthy adults</td><td>18-64</td></tr>
<tr><td>2</td><td>Healthy elderly adults*</td><td>≥ 65</td></tr>
<tr><td>3</td><td>Adults with significant comorbidities*</td><td>≥ 18</td></tr>
</table>
*Subjects to be enrolled in country(ies) that has/have provided authorization to enroll these subjects in the study.</td><td>The cross-over will no longer be used in the Phase 3 portion. Instead, once the CoVLP vaccine obtains IOA/EUA, the CoVLP vaccine will be offered to all placebo subjects in the Phase 2 and Phase</td></tr>
</table>

<table>
<tr><th>#</th><th>Section (page #)</th><th>Change</th><th>Rationale</th></tr>
<tr><td></td><td></td><td>Study Population #2 will be roughly stratified by age in the same manner to the greatest extent possible given local conditions (i.e. COVID-19 vaccine roll-out). Enrollment of subjects in Study Population #3 will include adults with co-existing conditions such as, but not limited to, obesity (moderate or greater), cardiovascular diseases, hyperlipidemia, chronic kidney disease or related disorders, chronic respiratory disease, pre-diabetes and diabetes (type 1 and type 2), and treatment-controlled HIV.
The overall goal of the Phase 3 portion of this study is to enroll approximately 30 000 subjects who are generally representative of the overall target population in terms of racial diversity with enrichment for those at highest risk for serious COVID-19 disease (i.e. the elderly and those with significant comorbidities).
In each Study Population, subjects will be randomized to receive either the CoVLP formulation or the placebo.~~, according to the following treatment sequences:~~
<table>
<tr><th>~~Treatment Sequence~~</th><th>~~Period 1~~</th><th>~~Period 2~~</th></tr>
<tr><td>~~1~~</td><td>~~CoVLP formulation~~</td><td>~~Placebo~~</td></tr>
<tr><td>~~2~~</td><td>~~Placebo~~</td><td>~~CoVLP formulation~~</td></tr>
</table>
~~Subjects who receive the CoVLP formulation in Period 1 will receive the placebo in Period 2, while subjects who receive the placebo in Period 1 will receive the CoVLP formulation in Period 2.~~
Subjects will be screened and will demonstrate a satisfactory baseline medical assessment by history and general physical examination prior to the first vaccine administration.<del> start of Period 1. Prior to starting Period 2, subjects will be assessed to ensure they do not meet any contraindications to the first vaccination in Period 2. The study visits and contacts described below are planned for both Period 1 and Period 2. During Period 1, subjects may not complete all study contacts prior to cross-over since cross-over for a subject can be initiated as early as Day 60; even after the cross-over has been initiated, subjects will still return for the Day 201 and Day 386 visits (which will be aligned with post-cross-over visits that are within close proximity of the planned date). During Period 2, study visits and contacts will be performed as planned (i.e., Day 21, Day 42, Day 201, and Day 386 post-cross-over).</del>
On Day 0 and Day 21, vaccine administration will occur. Subjects will be provided with a diary and memory aid to collect safety and concomitant medication data. A visit to the</td><td>3 portions. Also, clarified the details regarding the enrollment of subjects and the method of contacting subjects during the surveillance period.</td></tr>
</table>

#	Section (page #)	Change	Rationale
		Investigator site will occur on Day 21 (immunogenicity subset only) and Day 42 (all subjects) for immunogenicity assessments. Subjects **who initially receive the CoVLP formulation or subjects in the placebo group who do not want the CoVLP formulation** will return to the Investigator site on Day 201 and Day 386 (6-month and 12-month safety follow-ups and immunogenicity assessments [immunogenicity subset only] after the last vaccine administration).<br>Active **surveillance will be conducted in the study from the day of the first vaccination (Day 0, post-vaccination) until IOA/EUA** and passive surveillance will be conducted in the study from the day of the first vaccination (Day 0, post-vaccination) until the end of the study. For passive surveillance, subjects will be instructed to report symptoms meeting the definition of new COVID-19 disease. For active surveillance, subjects will be contacted once per week via the **electronic diary** ~~subject's preferred method (e.g. phone, text, email, electronic diary),~~ and asked about symptoms of COVID-19 disease. **Other methods of contact (phone, text, email) will only be used as a last resort to contact the subject.** In addition, to monitor asymptomatic infection, subjects **who initially receive the CoVLP formulation or subjects in the placebo group who do not want the CoVLP formulation** will perform a test for SARS-CoV-2 N antibodies on Day 0 and Day 201~~, Day 386 (in Period 1), and at cross-over~~. **Subjects in the placebo group who receive the CoVLP formulation post-IOA/EUA will perform the same test on Day 0 and just prior to receiving the first dose of the CoVLP formulation.**<br>**Once subjects in the placebo group receive the CoVLP formulation (post-IOA/EUA), they will no longer be followed for efficacy (no more active surveillance) or immunogenicity (those included in the immunogenicity subset). Passive surveillance for possible COVID-19 cases will continue however. Subjects will provide a blood draw immediately prior to receiving the first dose of the CoVLP formulation and then will be followed for safety with monthly phone contacts until Day 386, when the final study telephone contact will occur.**	
9.	Synopsis (Safety Evaluations)<br>AND	This section has been updated as follows:<br>Safety and tolerability endpoints will include immediate AEs (30 minutes after each vaccination), solicited local and systemic AEs (up to seven days after each vaccination), unsolicited AEs up to 21 days after each vaccination, SAEs, MAAEs, AEs leading to	The cross-over will no longer be used in the Phase 3 portion. Instead,

#	Section (page #)	Change	Rationale
	Section 10.2.1 (Safety Evaluations	withdrawal, AESIs (including VAED), and deaths up to the end of each phase of the study. In the Phase 3 portion **post-IOA/EUA** ~~after the cross-over~~, the same safety endpoints will be collected (including immediate AEs) **from subjects in the placebo group who receive the CoVLP formulation** with one exception: solicited local and systemic AEs (up to seven days after each vaccination) will not be collected **after the administration of the CoVLP formulation**.	once the CoVLP vaccine obtains IOA/EUA, the CoVLP vaccine will be offered to all placebo subjects in the Phase 2 and Phase 3 portions.
10.	Synopsis (Immunogenicity Evaluations)<br>AND<br>Section 10.3.1 (Immunogenicity Evaluations)	A new paragraph has been added at the end of the section:<br>**In the Phase 2 and Phase 3 portion, immunologic outcomes will be determined for subjects who originally receive the CoVLP formulation and placebo subjects who do not want the CoVLP formulation post-IOA/EUA until the end of the study. These outcomes will also be determined in placebo subjects before IOA/EUA if these subjects receive the CoVLP formulation post-IOA/EUA.**	The cross-over will no longer be used in the Phase 3 portion. Instead, once the CoVLP vaccine obtains IOA/EUA, the CoVLP vaccine will be offered to all placebo subjects in the Phase 2 and Phase 3 portions.
11.	Synopsis (Efficacy Evaluations)<br>AND<br>Section 10.4.1 (Efficacy Evaluations)	The first and sixth paragraphs have been updated and a new paragraph has been added after the sixth paragraph as follows:<br>Following the first vaccination, subjects will be asked to report symptoms associated with COVID-19 from the day of the first vaccination (Day 0, post vaccination) until the end of the Phase 2 portion or the Phase 3 portion of the study (through active or passive surveillance**; active surveillance in the Phase 3 portion will end once IOA/EUA is obtained**). COVID-19 will be defined as the presence of a laboratory-confirmed (virologic method) SARS-CoV-2 infection with the occurrence of a new onset of one or more of the following symptoms:	Clarified the method of contacting subjects during the surveillance period and the period of active surveillance. Also, added

#	Section (page #)	Change	Rationale
		All subjects with virologically-confirmed SARS-CoV-2 infection, based on results from the locally performed testing, will be asked to ~~keep a detailed daily record of their symptoms and~~ provide serial (every other day) self-administered nasal swabs (2 swabs every other day for the central virology laboratory) for approximately 2 weeks. In the event, the COVID-19-associated symptoms persist for more than 2 weeks, then the Investigator's clinical judgement is required to extend the collection of swabs beyond the 2 weeks from the subject. If the COVID-19-associated symptoms have resolved in less than 2 weeks and the Investigator believes it is likely the subject has virologically recovered, then two consecutive negative results, obtained locally, are required prior to stopping the collection of the self-administered swabs for the purpose of testing at the central laboratory.<br>**At the same visit where the Investigator will confirm whether nasal or NP swabs should be collected, subjects will be trained on daily recording of their COVID-19-associated symptoms using a COVID-19 diary and will be asked to immediately start recording their symptoms in the COVID-19 diary. Subjects will collect any COVID-19-associated symptoms in their COVID-19 diary until a negative swab result for the subject is received from the central virology laboratory or until the subject has no more COVID-19-associated symptoms.**	details of when and how the COVID-19 diary should be completed.
12.	Synopsis (Primary Endpoints - Efficacy)<br>AND<br>Section 10.4.2.1 (Primary Endpoint)	The following Efficacy primary endpoint has been updated as follows:<br>In the Phase 3 portion, the primary endpoint is:<br>First occurrence, in a subject, of laboratory-confirmed (virologic method) symptomatic SARS-CoV-2 infection (≥ 7 days post-second vaccination) at the time of the primary analysis (i.e., once at least 160 COVID-19 cases [≥ 7 days post-second vaccination] have been collected) ~~and prior to cross-over~~.	The cross-over will no longer be used in the Phase 3 portion.
13.	Synopsis (Secondary Endpoints – Efficacy)<br>AND	The following Efficacy secondary endpoints have been updated as follows:<br>• Occurrences of laboratory-confirmed asymptomatic SARS-CoV-2 infection at Day 201 ~~cross-over and Day 386 in Period 1~~ in subjects who were seronegative at baseline (confirmed by the ELISA method for the N protein);<br>• Occurrences of severe COVID-19 disease (≥ 7 days post-second vaccination) at the time of the PVE analysis ~~and prior to cross-over~~. Refer to the definition of severe COVID-19 disease in the Secondary Objectives section above;	The cross-over will no longer be used in the Phase 3 portion.

#	Section (page #)	Change	Rationale
	Section 10.4.2.2 (Secondary Endpoints)	• Occurrence and intensity of COVID-19-related symptoms in virologically-confirmed cases up until resolution of the symptoms ~~in Period 1~~ **in subjects administered the CoVLP formulation** compared to subjects administered the placebo **at the time of the PVE analysis** ~~and compared to subjects administered CoVLP at the time of cross-over in Period 2~~;<br>• First occurrence, in a subject, of laboratory-confirmed (virologic method) symptomatic SARS-CoV-2 infection (≥ first vaccination) at the time of the PVE analysis ~~and prior to cross-over~~;<br>• First occurrence, in a subject, of laboratory-confirmed (virologic method) symptomatic SARS-CoV-2 infection (≥ first vaccination and < second vaccination) at the time of the PVE analysis ~~and prior to cross-over~~;<br>• First occurrence, in a subject, of laboratory-confirmed (virologic method) symptomatic SARS-CoV-2 infection (≥ second vaccination and < 7 days post-second vaccination) at the time of the PVE analysis ~~and prior to cross-over~~;<br>• Duration and intensity of viral shedding after virologically-confirmed SARS-CoV-2 infection at the time of the PVE analysis ~~and prior to cross-over~~;<br>• First occurrence, in a subject, of laboratory-confirmed (virologic method) symptomatic SARS-CoV-2 infection (≥ 7 days post-second vaccination) at the time of the PVE analysis by strain ~~and prior to cross-over~~;	
14.	Synopsis (Exploratory Endpoints – Efficacy)<br>AND<br>Section 10.4.2.3 (Exploratory Endpoints)	The following Efficacy exploratory endpoint has been removed:<br>~~• Time to first occurrence of virologically-confirmed symptomatic disease as well as serologically-confirmed asymptomatic disease assessed for any strain and by strain at the time of the PVE analysisthroughout the study;~~	Removed an efficacy endpoint that cannot be assessed since placebo subjects will be offered the CoVLP vaccine post-IOA/EUA.
15.	Synopsis (Secondary	The following immunogenicity secondary endpoints have been updated as follows:<br>• In the immunogenicity subset ~~prior to cross-over and post-cross-over~~, Nab antibody response induced in each Study Population against the SARS-CoV-2	The cross-over will no longer be

medicago

#	Section (page #)	Change	Rationale
	Endpoints – Immunogenicity)<br>AND<br>Section 10.3.2.2 (Secondary Endpoints)	virus on Days 0 (all subjects), 21, 42 (all subjects), 201, and 386 analyzed, using the following parameters: GMT, SC rate, and GMFR;<br>• In the immunogenicity subset ~~prior to cross-over and post-cross-over~~, specific antibody response induced in each Study Population against the SARS-CoV-2 virus measured by the total IgG levels on Days 0, 21, 42, 201, and 386, and analyzed using the following parameters: GMT, SC rate, and GMFR;<br>• In the immunogenicity subset ~~prior to cross-over and post-cross-over~~, the ratio of neutralizing antibody titers:IgG (ELISA) antibody titers at Day 21, Day 42, Day 201, and Day 386;<br>• In the immunogenicity subset ~~prior to cross-over and post-cross-over~~, specific Th1 CMI response induced in each Study Population against the SARS-CoV-2 virus on Days 0, 21, 42, 201, and 386, as measured by IFN-γ ELISpot;<br>• In the immunogenicity subset ~~prior to cross-over and post-cross-over~~, specific Th2 CMI response induced in each Study Population against the SARS-CoV-2 virus on Days 0, 21, 42, 201, and 386, as measured by IL-4 ELISpot;	used in the Phase 3 portion.
16.	Synopsis (Exploratory Endpoints – Immunogenicity)<br>AND<br>Section 10.3.2.3 (Exploratory Endpoints)	The following Immunogenicity exploratory endpoint has been updated as follows:<br>• In the immunogenicity subset ~~prior to cross-over and post-cross-over~~, specific CMI response induced in each Study Population against the SARS-CoV-2 virus up to 21 days after the second vaccination and at Day 201 and Day 386, as measured by the percentage of CD4+ T cells expressing functional markers;<br>Further characterization of the immune response of the CoVLP formulation, if it is likely to be informative;	The cross-over will no longer be used in the Phase 3 portion.
17.	Synopsis (Secondary Endpoints – Safety)<br>AND	The following safety secondary endpoints have been updated as follows:	The cross-over will no longer be used in the Phase 3 portion. In addition, clarified that AESIs also

#	Section (page #)	Change	Rationale
	Section 10.2.2.2 (Secondary Endpoints)	• Occurrence, intensity, and relationship to vaccination of immediate AEs (30 minutes after each vaccine administration) ~~for both Period 1 and Period 2 vaccinations~~;<br>• Occurrence and intensity of solicited local and systemic AEs (for seven days following each vaccine administration) for **the initial set of** ~~Period 1~~ vaccinations;<br>• Occurrence, intensity, and relationship of unsolicited AEs for 21 days following each vaccine administration ~~for both Period 1 and Period 2 vaccinations~~;<br>• Occurrences of SAEs, MAAEs, AEs leading to withdrawal, AESIs (including VAED**, anaphylaxis, and severe allergic reactions**), and deaths up to 21 days following each vaccine administration ~~for both Period 1 and Period 2 vaccinations~~;<br>• Occurrences of SAEs, MAAEs, AEs leading to withdrawal, AESIs (including VAED**, anaphylaxis, and severe allergic reactions)**, and deaths from Day 43 to Day 201 ~~in both Period 1 and Period 2~~;<br>• Occurrences of SAEs, MAAEs, AEs leading to withdrawal, AESIs (including VAED**, anaphylaxis, and severe allergic reactions**), and deaths from Day 202 to Day 386 ~~in both Period 1 and Period 2~~.	includes anaphylaxis and severe allergic reactions.
18.	Synopsis (Statistical Methods – Phase 3 Portion Efficacy and Safety Analyses)<br>AND<br>Section 12.3 (Day 21 and Day 42 Immunogenicity Data Analysis and Efficacy and Safety Analyses)	The section has been updated as follows:<br>When at least 160 laboratory-confirmed COVID-19 cases (≥ 7 days post-second vaccination) have been collected and the median safety follow-up duration of at least 2 months (post-administration of the second vaccination) of at least 3000 subjects in each of the CoVLP formulation and placebo groups has been achieved, the unblinded statistical team will perform the final analysis and produce final efficacy outputs for the Phase 3 portion of the efficacy data in order to test for vaccine efficacy. If the primary efficacy success criterion is met during this analysis, **IOA/EUA** ~~emergency use authorization or approval~~ will be pursued**.** ~~and initiation of the cross-over design. Initiation of the cross-over design for subjects enrolled in the study will be dependent on the following overall study and subject-specific criteria:~~	The cross-over will no longer be used in the Phase 3 portion. Instead, once the CoVLP vaccine obtains IOA/EUA, the CoVLP vaccine will be offered to all placebo subjects in the Phase 2 and Phase 3 portions.

medicago

#	Section (page #)	Change	Rationale
		~~1. When Emergency Use Authorization or Approval for the CoVLP formulation is granted by any Regulatory Agency(ies); and~~<br>~~2. When one or more authorized or approved COVID-19 vaccines become available for the subject in his/her country of recruitment; and~~<br>~~3. The subject has completed a minimum of 60 days in Period 1.~~<br>**Upon obtaining the IOA/EUA for the CoVLP formulation, the study will be unblinded to support the following:**<br>**1. Subjects in the placebo group will be offered the CoVLP formulation and will only be followed passively to identify symptomatic COVID-19 cases and for safety until the end of the study. A blood sample will be obtained immediately prior to the administration of the first dose of the CoVLP formulation for serologic analyses but no further blood samples will be collected in this group;**<br>**2. Subjects in the CoVLP formulation group will complete the study as planned with a blood draw at Day 201 to assess protection against serologically-confirmed COVID-19.**	
19.	Synopsis (Statistical Methods – Publication/Release of Study Results for the Phase 2 and Phase 3 Portions)	The following new content has been added:<br>**After presenting to Regulatory Authority(ies), Medicago intends to release and/or publish the clinical data. Data that are released and/or published will only present group level results and will not involve any unblinding or the presentation of subject level data.**	Clarified that results generated in the study will be released and/or published as the results become available.
20.	Synopsis (Statistical Methods – Efficacy Analyses)	The second and last paragraphs have been updated as follows:<br>The primary assessment of VE will be evaluated ~~before cross-over~~ as the incidence rate ratio of CoVLP vaccinated versus placebo subjects to develop laboratory-confirmed symptomatic SARS-CoV-2 infection ≥ 7 days after the second vaccination, as per the following formula:<br>~~Any duration of protection assessments along with its two-sided 95 % CI in symptomatic subjects performed after cross-over will be evaluated using a Cox proportional hazard model with time-varying data and time-varying parameters for any strain and by strain.~~	The cross-over will no longer be used in the Phase 3 portion. Removed an efficacy analysis that cannot be performed since

#	Section (page #)	Change	Rationale
			placebo subjects will be offered the CoVLP vaccine post-IOA/EUA.
21.	Table 1 (Time and Events Schedule: General Information for the Phase 2 Portion)	The time and events schedule for the Phase 2 portion has been updated to include a column for an unscheduled contact for unblinding subjects and three additional footnotes have been added. In addition, the description for active surveillance has been updated.	Once the CoVLP vaccine obtains IOA/EUA, the CoVLP vaccine will be offered to all placebo subjects in the Phase 2 and Phase 3 portions.
22.	Table 2 (Time and Events Schedule: General Information for the Phase 3 Portion)	The time and events schedule table for the Phase 3 portion has been updated to remove references to Period 1 and remove non-applicable footnotes. A column for an unscheduled contact for unblinding subjects and three additional footnotes have been added. In addition, the description for active surveillance and testing for laboratory confirmation of SARS-CoV-2 infection have been updated.	The cross-over will no longer be used in the Phase 3 portion. Instead, once the CoVLP vaccine obtains IOA/EUA, the CoVLP vaccine will be offered to all placebo subjects in the Phase 2 and Phase 3 portions.
23.	Table 3 (Time and Events Schedule: General Information for the Phase 3 Portion)	This table has been updated to present the visits and contacts for placebo subjects who will receive the CoVLP formulation post-IOA/EAU in the Phase 2 and Phase 3 portions.	The cross-over will no longer be used in the Phase 3 portion. Instead, once the CoVLP

#	Section (page #)	Change	Rationale
			vaccine obtains IOA/EUA, the CoVLP vaccine will be offered to all placebo subjects in the Phase 2 and Phase 3 portions.
24.	Section 1.1 (Background)	The fifth paragraph has been updated as follows:<br>Vaccination is the most effective strategy for preventing infectious diseases since it appears to be more cost-effective than therapeutic drugs and reduces morbidity and mortality without causing long-lasting undesirable effects. Although three human coronaviruses emerged worldwide over the past two decades, causing considerable threat to global health, only **a few vaccines have been approved for the prevention of COVID-19 including the Pfizer-BioNTech COVID-19 vaccine (Canada and USA), the Moderna COVID-19 vaccine (Canada and USA), the AstraZeneca COVID-19 vaccine (Canada), and the Janssen COVID-19 vaccine (Canada and USA) with specific conditions, in a particular age range** ~~the Pfizer-BioNTech COVID-19 vaccine (16+ years of age) and the Moderna COVD-19 vaccine (18+ years of age) have been approved for the prevention of COVID-19 in Canada and the US~~. Research groups around the world are accelerating the development of COVID-19 vaccines using various approaches [Ahn 2020, Lu 2020].	Updated the list of COVID-19 vaccines approved in Canada and the USA to date.
25.	Section 4.2.2 (Blinding)	The first paragraph has been updated as follows:<br>Both portions of the study will be observer-blind to reduce potential bias during data collection and evaluation of the study endpoints. **However, once IOA/EUA is obtained, the Phase 2 and Phase 3 portions will be unblinded in order to offer subjects in the placebo group the CoVLP formulation.** Details of who will remain blinded during the Phase 2 and Phase 3 portions of the study are presented in Section 6.2.	The cross-over will no longer be used in the Phase 3 portion. Instead, once the CoVLP vaccine obtains IOA/EUA, the CoVLP vaccine will be offered to

#	Section (page #)	Change	Rationale
			all placebo subjects in the Phase 2 and Phase 3 portions.
26.	Section 4.2.3 (Dose Selection and Dosage Regimen)	The content in this section has been updated as follows:<br>In both the Phase 2 and Phase 3 portions, a single dose level of the CoVLP formulation (i.e. 3.75 µg CoVLP adjuvanted with AS03) will be tested in a two-dose regimen on Day 0 and Day 21. ~~In the Phase 3 portion, the use of the cross-over design will result in each subject receiving the CoVLP formulation and the placebo.~~	The cross-over will no longer be used in the Phase 3 portion.
27.	Section 4.2.6 (Cross-over Design)	This section has been removed:<br>~~The use of the blinded cross-over in the Phase 3 portion of the study will allow subjects who receive the placebo to get timely access to an authorized COVID-19 vaccine, in this case the CoVLP formulation upon its receiving emergency use authorization or approval in any jurisdiction.~~	The cross-over will no longer be used in the Phase 3 portion.
28.	Section 5.3 (Prior and Concomitant Therapy)	The second and third bullets have been updated as follows:<br>• From randomization to Day 42 (Phase 2 portion and ~~Period 1 of~~ Phase 3 portion) ~~or Day 0 to Day 42 (Period 2 of Phase 3 portion)~~, inclusive: any medication (including, but not limited to, over-the-counter medicines such as aspirin or antacids), vitamins, and mineral supplements;<br>• From Day 43 to the end of the study (Phase 2 portion **or Phase 3 portion**) ~~or of each period (Phase 3 portion)~~, inclusive: any concomitant medication(s) administered to treat an AESI, MAAE, SAE, or AE leading to withdrawal; any concomitant medication used to treat an AE that occurred before Day 42 and that is still being used afterwards (i.e. on-going use);	The cross-over will no longer be used in the Phase 3 portion.
29.	Section 5.4 (Prohibited Therapy)	Point #1 and point #3 have been updated as follows:<br>1. Administration of any vaccine (other than the study vaccine) up to Day 28 of the study (Phase 2 portion **or Phase 3 portion**) ~~or of each period (Phase 3 portion)~~ as well as administration of any investigational or approved coronavirus vaccine (other than the study vaccine) up to end of the study. Immunization on an emergency basis during the study will be evaluated on case-by-case basis by the Investigator;	The cross-over will no longer be used in the Phase 3 portion.

#	Section (page #)	Change	Rationale
		3. Study Populations #1 and #2: Administration of any medication or treatment that may alter the vaccine immune responses, such as:<br>• Systemic glucocorticoids;<br>• Cytotoxic, antineoplastic, or immunosuppressant drugs;<br>• Any immunoglobulin preparations or blood products, or blood transfusion.<br>Administration of such medications should be specifically avoided up to Day 42 of the study (Phase 2 portion **or Phase 3 portion**) ~~or of each period (Phase 3 portion)~~. Use of any medication or treatment that may alter the vaccine immune responses due to a medical need during the study will be evaluated on case-by-case basis by the Investigator;	
30.	Section 6.2 (Blinding)	The second, fourth, and eighth paragraphs have been updated and a new paragraph has been added after the fourth paragraph as follows:<br>**From the start of the Phase 2 and Phase 3 portions up to IOA/EUA** ~~During the entire duration of the study~~, the following individuals will not have access to treatment allocation (i.e. remain "blind"): the subjects, the Investigators and all personnel involved in the clinical conduct of the study (except the staff involved in the preparation and administration of the study vaccine, the quality assurance auditor, and quality control reviewers), Medicago clinical staff and medical staff involved in safety evaluations (e.g. causality assessments), and all personnel involved in sample analysis at the central and testing (Nab, ELISA, ELISpot, ICS [flow cytometry], serological, and virologic method SARS-CoV-2 diagnostics) laboratories. **Once IOA/EUA is obtained, the Phase 2 and Phase 3 portions will be unblinded.**<br>**From the start of the Phase 2 and Phase 3 portions up to IOA/EUA** ~~During the study~~, the Sponsor's Study Team will remain blinded. A small number of Medicago personnel (not included in Study Team) will be involved in the planned analyses during the Phase 2 and Phase 3 portions. During the PVE analyses in the Phase 3 portion, some Medicago personnel will be unblinded at the group level to support the review of the analyses however these individuals will NOT be unblinded at the treatment allocation level. The selected individuals and the process and level of unblinding will be documented.	The cross-over will no longer be used in the Phase 3 portion. Instead, once the CoVLP vaccine obtains IOA/EUA, the CoVLP vaccine will be offered to all placebo subjects in the Phase 2 and Phase 3 portions.

#	Section (page #)	Change	Rationale
		In order to efficiently manage IP inventory at sites in the Phase 3 portion, a Medicago Unblinded Clinical Supply Manager will have access to site inventory and may therefore be exposed to subject level treatment allocation. The unblinded role of this individual will be documented and the individual will be trained to maintain the study blind. The Unblinded Clinical Supply Manager will not have access to study outcomes such as COVID-19 test results.<br><br>If the subject fulfils requirements of the two criteria, then the date of **premature** unblinding will be captured in the IRT system. If the subject does not meet both criteria, he/she will not be eligible for **premature** unblinding. Details regarding the subject's status in the study are included in Section 11.6.	
31.	Section 8.2 (Collection of Samples)	The third and last paragraphs have been updated as well as the Table 8 as follows:<br><br>In the Phase 3 portion of the study, all subjects will have up to a total of ~~55~~ **30** mL of blood drawn. Subjects included in the immunogenicity subset will have up to an additional blood volume of up to approximately ~~500~~ **250** mL drawn up to the end of the study. In total, subjects will have up to ~~55~~ **30** to ~~555~~ **280** mL of blood drawn up to the end of the study (Table 8~~9~~).<br><br>**Once placebo subjects receive the CoVLP formulation post-IOA/EUA, they will no longer provide any blood draws with the exception of the following:**<br><br>• **Subjects will provide a 5 mL blood sample immediately prior to the administration of the first dose of the CoVLP formulation to test for SARS-CoV-2 N antibodies. The total blood drawn up to the end of the study in all subjects remains the same.**<br>• **If IOA/EUA occurs post-Day 201, then no blood sample will be collected as a blood sample to test for SARS-CoV-2 N antibodies will be collected on Day 201.**	The cross-over will no longer be used in the Phase 3 portion. Instead, once the CoVLP vaccine obtains IOA/EUA, the CoVLP vaccine will be offered to all placebo subjects in the Phase 2 and Phase 3 portions.

Table **8**~~9~~ Estimated Blood Volume Drawn: Phase 3 Portion

Type of Sample	Volume per Sample (mL)	Number of Samples per Subject						Total Volume of Blood per Subject ~~per Period~~ (mL)	~~Total Volume of Blood per Subject (mL)~~
		Visit 1 (Day 0)	Visit 2 (Day 21)	Visit 3 (Day 42)	Visit 4 (Day 201)~~[§]~~	Visit 5 (Day 386)~~[§]~~	Total		
Test for SARS-CoV-2 **N** antibodies	5	1~~[‡]~~			**1**[†]	~~1[‡]~~	2	10	~~15[†,‡]~~
Immunogenicity – serology (Nab assay only)	10	1		1			2	20	~~40~~
Immunogenicity-Serology (Nab assay, ELISA)*	10	1	1	1	1	1	5	50	~~100~~
Immunogenicity- CMI response (ELISpot and ICS)*	40	1	1	1	1	1	5	200	~~400~~
Total volume of blood per subject (mL)		15-65	50	10-60	**5**-55~~0~~	~~5-~~55**0**		30-280	~~55-555~~

* These samples will be collected for pre-defined subset of subjects.

† This blood sample will be collected from all subjects on Day 201. However, if IOA/EUA occurs prior to Day 201, then only in placebo subjects receive the CoVLP formulation post-IOA/EUA, this blood sample will be collected immediately prior to the administration of the first dose of the CoVLP formulation.

~~† The Day 0 sample will be collected prior to vaccination in Period 1 and Period 2. The sample collected prior to vaccination in Period 2 represents the sample collected "at cross-over" for the purposes of the efficacy evaluation.~~

~~‡ The Day 386 sample will only be collected during the visit for Day 386 of Period 1.~~

~~§ Based on the expected SARS-CoV-2 attack rate in unvaccinated subjects and the expected rate of subject enrollment in the Phase 3 portion, it is assumed that cross-over will occur prior to Day 201 (more than 45 days before Day 201), thus the Day 201 visit of Period 1 will occur at approximately the same time as the Day 42 visit of Period 2 (i.e., on the same day). If cross-over occurs within 45 days before the Day 201 visit, then the Day 201 visit of Period 1 will occur at approximately the same time as the Day 0 visit of Period 2 (i.e., on the same day). If cross-~~

#	Section (page #)	Change	Rationale
		~~over occurs prior to Day 386, thus the Day 386 visit of Period 1 will occur at approximately the same time as the Day 201 visit of Period 2 (i.e., on the same day).~~<br><br>In the Phase 3 portion of the study, pregnancy testing will be performed for all females of childbearing potential (in Study Populations #1 and #3) ~~during each period~~. A urine (dipstick or similar) pregnancy test will be performed at screening and at Day 0 prior to randomization and at Days 21 and 42. In Study Populations #1 and #3, females of child bearing potential must have negative urine pregnancy test results from screening and Day 0 available prior to vaccination ~~in each period~~.	
32.	Section 10.1.1 (Overview)	This section has been updated as follows:<br><br>During the Phase 2 and Phase 3 portions of the study, the Time and Events Schedules: General Information for each portion (see Table 1, Table 2, and Table 3) summarizes the frequency and timing of scheduled assessments applicable to each portion of the study.<br><br>**For the Phase 3 portion, once placebo subjects receive the CoVLP formulation post-IOA/EUA, these subjects will stop following the planned assessments (i.e. visits, contacts, surveillance) and will perform the following assessments in Section 10.1.15. Subjects in the placebo group who do not want the CoVLP formulation but want to remain in the study will continue the study as planned.**<br><br>~~For the Phase 3 portion of the study, the frequency and timing of scheduled assessments will be the same for Period 1 and Period 2, with the exception of the following:~~<br><br>~~29. Screening visit (Visit 1 for Phase 3 portion):~~<br>• ~~Prior to starting Period 1, screening assessments will be performed to determine initial eligibility of subjects in the study;~~<br>• ~~Prior to starting Period 2, subjects will be assessed to ensure they do not meet any contraindications to the first vaccination in Period 2;~~<br>~~30. The informed consent form will be signed and dated prior to the start of Period 1. Unless a new consent form has been approved for use and is required to be signed and dated by all the subjects, subjects will not be required to sign and date the same consent form prior to starting Period 2;~~<br>~~31. For the SARS-CoV-2 antibodies (Phase 3 portion) assessment:~~	The cross-over will no longer be used in the Phase 3 portion. Instead, once the CoVLP vaccine obtains IOA/EUA, the CoVLP vaccine will be offered to all placebo subjects in the Phase 2 and Phase 3 portions.

#	Section (page #)	Change	Rationale
		• ~~The Day 0 sample will be collected prior to vaccination in Period 1 and Period 2. The sample collected prior to vaccination in Period 2 represents the sample collected "at cross-over" for the purposes of the efficacy evaluation;~~<br>• ~~The Day 386 sample will only be collected during Period 1 and subjects should return for this visit even if cross-over has been initiated prior to Day 386 of Period 1;~~<br>~~32. Randomization will only occur prior to the start of Period 1. Treatment assignments for Period 2 will be done in the IRT system.~~<br><br>~~During Period 1, subjects may not complete all study contacts prior to cross-over since cross-over for a subject can be initiated as early as Day 60 once emergency use authorization or approval for the CoVLP formulation is granted in any jurisdiction and when one or more COVID-19 vaccines become available for the subject in his/her country of recruitment; even after the cross-over has been initiated, subjects will still return for the Day 201 and Day 386 visits (which will be aligned with post-cross-over visits that are within close proximity of planned date). Based on the expected SARS-CoV-2 attack rate in subjects who receive placebo and the expected rate of subject enrollment in the Phase 3 portion, it is assumed that cross-over will occur prior to the Day 201 visit (more than 45 days before Day 201), thus:~~<br><br>~~33. The Day 201 visit of Period 1 will occur at approximately the same time as the Day 42 visit of Period 2 (i.e., on the same day);~~<br>~~34. The Day 386 visit of Period 1 will occur at approximately the same time as the Day 201 visit of Period 2 (i.e., on the same day).~~<br><br>~~If cross-over occurs within 45 days before the Day 201 visit, then:~~<br><br>~~35. The Day 201 visit of Period 1 will occur at approximately the same time as the Day 0 visit of Period 2 (i.e., on the same day);~~<br>~~36. The Day 386 visit of Period 1 will occur at approximately the same time as the Day 201 visit of Period 2 (i.e., on the same day).~~<br><br>~~In the event that the cross-over occurs after the Day 201 visit and before the Day 386 visit, then:~~	

#	Section (page #)	Change	Rationale
		37. ~~The Day 386 visit of Period 1 will still occur at approximately the same time as the Day 201 visit of Period 2 (i.e., on the same day).~~<br>~~During Period 2, study visits and contacts will be performed as planned.~~	
33.	Section 10.1.6 (Surveillance for COVID-19 Cases)	The first paragraph and the second bullet have been updated and a new bullet has been added after the third sub-bullet of the third bullet as follows:<br>From the day of the first vaccination (Day 0, post vaccination), passive and active surveillance will be performed until the end of the Phase 2 or the Phase 3 portion of the study**, except active surveillance in the Phase 3 portion will end once IOA/EUA is obtained**:<br>• Active surveillance: the subjects will be contacted once per week via the **electronic diary** ~~method most likely to be successful (e.g. subject's preferred method [phone, text, email, electronic diary]),~~ and asked about symptoms of COVID-19**. Other methods of contact (phone, text, email) will only be used as a last resort to contact the subject**;<br>• **At the same visit where the Investigator will confirm whether nasal or NP swabs should be collected, subjects will be trained on daily recording of their COVID-19-associated symptoms using a COVID-19 diary and will be asked to immediately start recording their symptoms in the COVID-19 diary. Subjects will collect any COVID-19-associated symptoms in their COVID-19 diary until a negative swab result for the subject is received from the central virology laboratory or until the subject has no more COVID-19-associated symptoms;**	Clarified the method of contacting subjects during the surveillance period and the period of active surveillance. Also, added details of when and how the COVID-19 diary should be completed.
34.	Section 10.1.13 (Day 201)	A new bullet has been added after the third bullet as follows:<br>• **Phase 3 portion only: collect blood sample to test for SARS-CoV-2 antibodies;**	Clarified that the blood draw will be collected on Day 201.
35.	Section 10.1.14 (Day 386)	This section has been updated as follows:<br>In the Phase 2 or Phase 3 portion, subjects who are withdrawn/discontinued from the	The cross-over will no longer be

#	Section (page #)	Change	Rationale
		study due to receiving an approved or authorized COVID-19 vaccine **prior to IOA/EUA** will be asked to complete the procedures for the final telephone contact (refer to Section 10.1.14.2) at the time of **premature** unblinding and prior to receipt of the approved or authorized COVID-19 vaccine, if the subject agrees. If the subject unblinding occurs prior to the Day 42 visit, then the subject will also be asked about any AEs and recorded appropriately. For subjects who receive the approved or authorized COVID-19 vaccine and then inform the Investigator, the final telephone contact will focus only on the period prior to receiving the approved or authorized COVID-19 vaccine and the subject may be informed retroactively about their treatment allocation.<br><br>For all other subjects who withdraw consent from the Phase 2 portion **(subjects who initially receive the CoVLP formulation or subjects in the placebo group who do not want the CoVLP formulation post-IOA/EUA)** or ~~Period 1 (all subjects) or Period 2~~ **Phase 3 portion** (**subjects in the** immunogenicity subset **who initially receive the CoVLP formulation or receive placebo and do not want the CoVLP formulation post-IOA/EUA**) ~~of the Phase 3 portion of the study~~, they will be asked to undergo Day 386 visit (refer to Section 10.1.14.1) procedures within two weeks of withdrawal, if the subject agrees.<br><br>For all other subjects in ~~Period 2~~ **the Phase 2 portion (subjects in the placebo group who receive the CoVLP formulation post-IOA/EUA) or Phase 3 portion** (all subjects who are not included in the immunogenicity subset **and subjects in the placebo group who receive the CoVLP formulation post-IOA/EUA**) ~~of the Phase 3 portion of the study~~, they will be asked to participate in the final telephone contact (refer to Section 10.1.14.2) within two weeks of withdrawal, if the subject agrees.	used in the Phase 3 portion. Instead, once the CoVLP vaccine obtains IOA/EUA, the CoVLP vaccine will be offered to all placebo subjects in the Phase 2 and Phase 3 portions.
36.	10.1.14.1 (Visit 8 for Phase 2 portion and Visit 5 for Phase 3 portion)	The first paragraph and the fourth bullet have been updated as follows:<br><br>For the Phase 2 portion **(subjects who initially receive the CoVLP formulation or subjects in the placebo group who do not want the CoVLP formulation post-IOA/EUA)** and ~~Period 1 (all subjects) and Period 2~~ **Phase 3 portion** (**subjects in the** immunogenicity subset **who initially receive the CoVLP formulation or receive placebo and do not want the CoVLP formulation post-IOA/EUA**) ~~of the Phase 3 portion of the study~~: the post-vaccination final visit procedures will be performed by	The cross-over will no longer be used in the Phase 3 portion. Instead, once the CoVLP vaccine obtains IOA/EUA, the CoVLP vaccine

#	Section (page #)	Change	Rationale
		~~blinded~~ site staff members at the clinical site. For subjects in quarantine, these procedures will be performed by ~~blinded~~ trained medical staff and may be at the subject's home (or place of quarantine). The following procedures will be performed during the Day 386 visit (± 14 days):<br>• ~~Phase 3 portion only (Period 1 only): collect blood sample to test for SARS-CoV-2 antibodies;~~	will be offered to all placebo subjects in the Phase 2 and Phase 3 portions.
37.	Section 10.1.14.2 (Telephone Contact for Phase 3 Portion)	The section title and the first paragraph have been updated as follows:<br>Telephone Contact for Phase 3 portion ~~(Period 2 only)~~<br>For **the Phase 2 portion (subjects in the placebo group who receive the CoVLP formulation post-IOA/EUA) and** ~~Period 2 (all subjects who are not included in the immunogenicity subset) of~~ the Phase 3 portion **(all subjects who are not included in the immunogenicity subset and subjects in the placebo group who receive the CoVLP formulation post-IOA/EUA)** ~~of the study~~: the post-vaccination final telephone contact will be performed by ~~blinded~~ site staff members. The following procedures will be performed during the Day 386 phone contact (± 14 days):	The cross-over will no longer be used in the Phase 3 portion. Instead, once the CoVLP vaccine obtains IOA/EUA, the CoVLP vaccine will be offered to all placebo subjects in the Phase 2 and Phase 3 portions.
38.	10.1.15 (Placebo Subjects who Receive the CoVLP Formulation post-IOA/EUA for Phase 2 and Phase 3 portions)	This new section has been added:<br>**For the Phase 2 and Phase 3 portions, placebo subjects who receive the CoVLP formulation post-IOA/EUA will stop following the planned study assessments (i.e. visits, contacts, surveillance) and will perform the following assessments instead.**	The cross-over will no longer be used in the Phase 3 portion. Instead, once the CoVLP vaccine obtains IOA/EUA, the CoVLP vaccine will be offered to all placebo subjects in the

#	Section (page #)	Change	Rationale
			Phase 2 and Phase 3 portions.
39.	10.1.15.1 (Dose 1 and Dose 2 vaccinations with the CoVLP formulation)	This new section has been added:<br>**Phase 3 portion only: Prior to the administration of the first dose of the CoVLP formulation, a blood sample will be collected from subjects to test for SARS-CoV-2 antibodies. The collected blood sample will be prepared and stored until shipment to the analytical laboratory. The procedure to collect the blood sample will be performed by an unblinded site staff member.**<br>**Note: If IOA/EUA occurs post-Day 201, then no blood sample will be collected as a blood sample to test for SARS-CoV-2 antibodies will be collected on Day 201.**<br>**The first CoVLP formulation dose will be administered IM into the deltoid muscle of the non-dominant (if possible) arm, as described in the Investigational Product Management Manual. The arm used for vaccination will be documented in the source documents. For subjects with BMI < 30 kg/m², a 23 gauge needle of at least one inch or 2.5 cm in length can be used for vaccination. For subjects with BMI ≥ 30 kg/m², a needle of at least 1.5 inches or 3.5 cm in length must be used to ensure intramuscular delivery. The preparation and administration of the study vaccine will be performed by an unblinded site staff member.**<br>**The second CoVLP formulation dose will be administered 21 days after the first dose administration. The same procedures noted above will be followed. The second CoVLP formulation dose will be administered IM into the deltoid muscle of the alternate (if possible) arm (i.e. the arm not used for the previous dose administration). Record the administration, left or right arm, in the subject source documents.**	The cross-over will no longer be used in the Phase 3 portion. Instead, once the CoVLP vaccine obtains IOA/EUA, the CoVLP vaccine will be offered to all placebo subjects in the Phase 2 and Phase 3 portions.
40.	10.1.15.1.1 (Thirty Minutes Post-vaccination)	This new section has been added:<br>**The same procedures will be performed following each CoVLP formulation administration as will be performed following the administration of the study vaccine on Day 0 (see Section 10.1.4.3 for detailed procedures) for the Phase 2 and Phase 3 portions of the study, with the following exceptions:**	The cross-over will no longer be used in the Phase 3 portion. Instead, once the CoVLP

#	Section (page #)	Change	Rationale
		• **Non-immediate solicited local and systemic AEs (up to seven days after each vaccination) will not be collected;**<br>• **Provide appointments (date and time) for the monthly telephone calls and for the final study telephone call (Day 386);**<br>• **The subject will be released from the clinical site once all post-vaccination procedures have been completed and the subject is in stable condition.**	vaccine obtains IOA/EUA, the CoVLP vaccine will be offered to all placebo subjects in the Phase 2 and Phase 3 portions.
41.	10.1.15.2 (Monthly Calls Thereafter)	This new section has been added:<br>**Subjects should be contacted by telephone once every month (every 30 days ± 14 days; the second vaccination visit date as starting reference or last day of contact with the clinic site or Investigator if a second vaccination did not occur). The following procedures will be performed during the phone contacts:**<br>• **Ask the subjects about any change in health (AEs ongoing from 42 days after the first CoVLP formulation vaccination, SAEs, or AESIs), any visits to health care facilities, and/or medical practitioners and use of any concomitant medications. Record the information in the source documents;**<br>• **Advise the subjects to immediately contact the Investigator (or his/her designee), in the event of any AE which require a visit to the emergency and/or hospitalization;**<br>• **Remind the subjects of their next appointment (date and time) for the next telephone contact and/or the final telephone contact (Day 386);**<br>**In the event that a subject cannot be reached via telephone, he/she may be contacted by text message or via email (if these contacts are available). However, the telephone should be the initial and preferred means of communication. At least three attempts within allowed time window should be made in order to reach the subject.**	The cross-over will no longer be used in the Phase 3 portion. Instead, once the CoVLP vaccine obtains IOA/EUA, the CoVLP vaccine will be offered to all placebo subjects in the Phase 2 and Phase 3 portions.

#	Section (page #)	Change	Rationale
42.	10.1.15.3 (Final Telephone Contact)	This new section has been added:<br>**The final telephone contact will be performed according to the procedures outlined in Section 10.1.14.2.**	The cross-over will no longer be used in the Phase 3 portion. Instead, once the CoVLP vaccine obtains IOA/EUA, the CoVLP vaccine will be offered to all placebo subjects in the Phase 2 and Phase 3 portions.
43.	Section 10.2.1.1 (Solicited Local and System Adverse Events)	The first paragraph has been updated as follows:<br>Subjects will be monitored for both solicited local AEs (erythema, swelling, and pain at the injection site) and solicited systemic AEs (fever, headache, fatigue, muscle aches, joint aches, chills, a feeling of general discomfort, swelling in the axilla, and swelling in the neck) from the time of each vaccination through seven days. While the subjects remain in the clinic following vaccine administration (at least 30 minutes), staff will monitor them for solicited local and systemic AEs; after release from the clinic facility, from the evening of each vaccination to the evening of the seventh day after each vaccination, the subject will measure and record the subject's local and systemic AEs in the diary. The only exception for the monitoring of solicited AEs is: solicited local and systemic AEs (up to seven days after each vaccination) will not be collected from subjects **in the placebo group who receive the CoVLP formulation post-IOA/EUA** ~~after the cross-over in the Phase 3 portion~~, however the immediate AEs (30 minutes after each vaccination) will still be collected.	The cross-over will no longer be used in the Phase 3 portion. Instead, once the CoVLP vaccine obtains IOA/EUA, the CoVLP vaccine will be offered to all placebo subjects in the Phase 2 and Phase 3 portions.
44.	Section 10.2.1.4 (Clinical Laboratory Tests)	The third paragraph has been updated as follows:<br>Other clinical laboratory tests include a test to measure SARS-CoV-2 antibodies at screening for all subjects in all Study Populations in the Phase 2 portion and at Day 0, **Day 201 (in CoVLP formulation subjects and placebo subjects who do not want the**	The cross-over will no longer be used in the Phase 3 portion. Instead,

#	Section (page #)	Change	Rationale
		**CoVLP), and immediately prior to the administration of dose 1 of the CoVLP formulation in placebo subjects who receive the CoVLP formulation post-IOA/EUA** ~~Day 386 (in Period 1), and at cross-over~~ in the Phase 3 portion, and a serum (Phase 2 portion) or urine (Phase 3 portion; in each period) pregnancy test at screening and a urine (dipstick or similar) pregnancy test prior to randomization on Day 0 and on Days 21 and 42 for all female subjects of childbearing potential in Study Populations #1 and #3.	once the CoVLP vaccine obtains IOA/EUA, the CoVLP vaccine will be offered to all placebo subjects in the Phase 2 and Phase 3 portions.
45.	Section 10.2.1.5 (Vital Signs)	The first paragraph has been updated as follows:<br>Vital signs measurements (resting BP, HR, RR, and OT) will be performed as part of screening procedures, prior to vaccination (on Day 0 and Day 21), and after the post-vaccination 30-minute surveillance period (and repeated if deemed necessary by the Investigator) during the Phase 2 portion. In addition, vital signs measurements will be performed at the clinic site visits on Day 3, Day 24, Day 42, Day 128, Day 201, and Day 386 for the Phase 2 portion of the study. Vital signs measurements will only be performed prior to each vaccination during the Phase 3 portion **prior to IOA/EUA**.	Vital signs will not be collected from subjects post-IOA/EUA.
46.	Section 11.3 (Contraindications for Subsequent Vaccination)	The first paragraph and the sixth and seventh bullets and the second paragraph have been updated as follows:<br>The following events constitute absolute contraindication to the further administration of the study treatments at Day 21 in the Phase 2 portion or~~ in each period of the~~ Phase 3 portion, or **to subjects in the placebo group who receive the CoVLP formulation post-OA/EUA in** ~~Day 0 in Period 2 of~~ the Phase 3 portion:<br>• In the Phase 2 portion of the study: Experience a possibly related, probably related, or definitely related SAE or Grade 3 or higher related AE that cannot be clearly attributed to another cause. The Investigator's opinion to administer the second dose of the vaccine will be presented to the IDMC for advice. **Note: This event will not apply to subjects in the placebo group who receive their first dose of the CoVLP formulation**;<br>• In the Phase 3 portion of the study: Experience a possibly related, probably related, or definitely related SAE that cannot be clearly attributed to another cause. **Note:**	The cross-over will no longer be used in the Phase 3 portion. Instead, once the CoVLP vaccine obtains IOA/EUA, the CoVLP vaccine will be offered to all placebo subjects in the Phase 2 and Phase 3 portions.

#	Section (page #)	Change	Rationale
		**This event will not apply to subjects in the placebo group who receive their first dose of the CoVLP formulation**.<br>At the discretion of the Investigator, the following event constitutes contraindication to the further administration of the study treatments at Day 21 in the Phase 2 portion or ~~in each period of the~~ Phase 3 portion, or **to subjects in the placebo group who receive the CoVLP formulation post-IOA/EUA in the Phase 2 portion or Phase 3 portion** ~~at Day 0 in Period 2 of the Phase 3 portion~~:	
47.	Section 11.4 (Removal of Subjects from Treatment or Assessment)	The third paragraph has been updated as follows:<br>Withdrawal subjects will be those who leave the Phase 2 portion **or Phase 3 portion** of the study before Day 386 ~~or the Phase 3 portion of the study before Day 386 of Period 2~~, for whatever reason; withdrawal subjects will not be replaced. Any subject who withdraws consent from the study will be asked to visit the clinical site (Phase 2 portion or ~~Period 1 [all subjects] or Period 2~~ **Phase 3 portion** [immunogenicity subset] ~~of the Phase 3 portion~~) or participate in the final telephone contact (~~Period 2~~ **Phase 3 portion** [all subjects who are not included in the immunogenicity subset **or placebo subjects in the immunogenicity subset who receive the CoVLP formulation post-IOA/EUA**] ~~of the Phase 3 portion~~) within two weeks of withdrawal, if the subject agrees. The procedures performed for the final assessment or telephone contact will comprise of those for the Day 386 visit/telephone contact (as applicable), if permitted by the subject. In the Phase 2 or Phase 3 portion, subjects who are withdrawn from the study due to receiving an approved or authorized COVID-19 vaccine will be asked to complete the procedures for the final telephone contact at the time of unblinding and prior to receipt of the approved or authorized COVID-19 vaccine, if permitted by the subject.	The cross-over will no longer be used in the Phase 3 portion. Instead, once the CoVLP vaccine obtains IOA/EUA, the CoVLP vaccine will be offered to all placebo subjects in the Phase 2 and Phase 3 portions.
48.	Section 11.6 (Unblinding Due to Availability of Approved or Authorized COVID-19 Vaccines Prior to IOA/EUA	The section title and first, third, and fourth paragraphs and figure caption have been updated as follows:<br>**Premature** Unblinding Due to Availability of Approved or Authorized COVID-19 Vaccines **Prior to IOA/EUA**<br>As discussed in Section 6.2, some subjects may request to become **prematurely** unblinded in order to receive an approved or authorized vaccine for COVID-19 when it becomes publicly available to them in their region. If subjects meet the criteria outlined in Section 6.2, they will be **prematurely** unblinded to the treatment they have received. The subject's status in the study will be dependent on the treatment received and whether	The cross-over will no longer be used in the Phase 3 portion. Instead, once the CoVLP vaccine obtains IOA/EUA, the CoVLP vaccine will be offered to

#	Section (page #)	Change	Rationale
		the subject actually receives the approved or authorized COVID-19 vaccine (refer to Figure 3).<br>Figure 3 Decision Tree for Possible **Premature** Unblinding in Phase 2 and Phase 3 Portions **Prior to IOA/EUA**<br>In the Phase 3 portion, if a subject requests to be **prematurely** unblinded before the completion of their Day 21 visit (in Period 1) and does not receive the approved or authorized COVID-19 vaccine, then the unblinded subject will be eligible to receive their second study vaccination at the Day 21 visit (in Period 1). Subjects will still be assessed for any contraindications for the second study vaccinations (as per Section **Error! Reference source not found.**).<br>Subject who receives the approved or authorized COVID-19 vaccine**, prior to IOA/EUA,** will be asked to complete the final telephone contact at the time of unblinding and prior to receipt of the approved or authorized COVID-19 vaccine, if permitted by the subject.	all placebo subjects in the Phase 2 and Phase 3 portions.
49.	Section 12.1.1 (Safety Analysis Set)	This section has been updated as follows:<br>The SAS is defined as all subjects who received at least one dose of either the CoVLP formulation or placebo **prior to IOA/EUA**. All safety analyses will be performed using the SAS and according to actual vaccination received. ~~More particularly, for CoVLP formulation, s~~Safety data for subjects will be analyzed after each vaccination (Day 0-Day 21 period) according to the last vaccine they actually received, and after any vaccination (Day 0-Day 21 after last vaccination period/Day 0-End of study period) according to the actual vaccine received at the first dose.<br>**After IOA/EUA, safety data after each vaccination period for placebo subjects who receive the CoVLP formulation post-IOA/EUA will be analyzed according to the last vaccine actually received and after any vaccination (Day 0-Day 21 after last vaccination period/Day 0 -End of study period) according to the actual vaccine received at first dose post-IOA/EUA. Safety data collected after IOA/EUA for all other subjects (i.e., those who did not receive CoVLP formulation post-IOA/EUA and those receiving CoVLP formulation prior to IOA/EUA) will be analyzed as described in the previous paragraph.**	The cross-over will no longer be used in the Phase 3 portion. Instead, once the CoVLP vaccine obtains IOA/EUA, the CoVLP vaccine will be offered to all placebo subjects in the Phase 2 and Phase 3 portions.

<table>
<tr><th>#</th><th>Section (page #)</th><th>Change</th><th>Rationale</th></tr>
<tr><td></td><td></td><td>

However, for subjects who become **prematurely** unblinded because they requested to be unblinded for the purposes of receiving an approved or authorized COVID-19 vaccine, their safety data will be handled according to Figure 4.

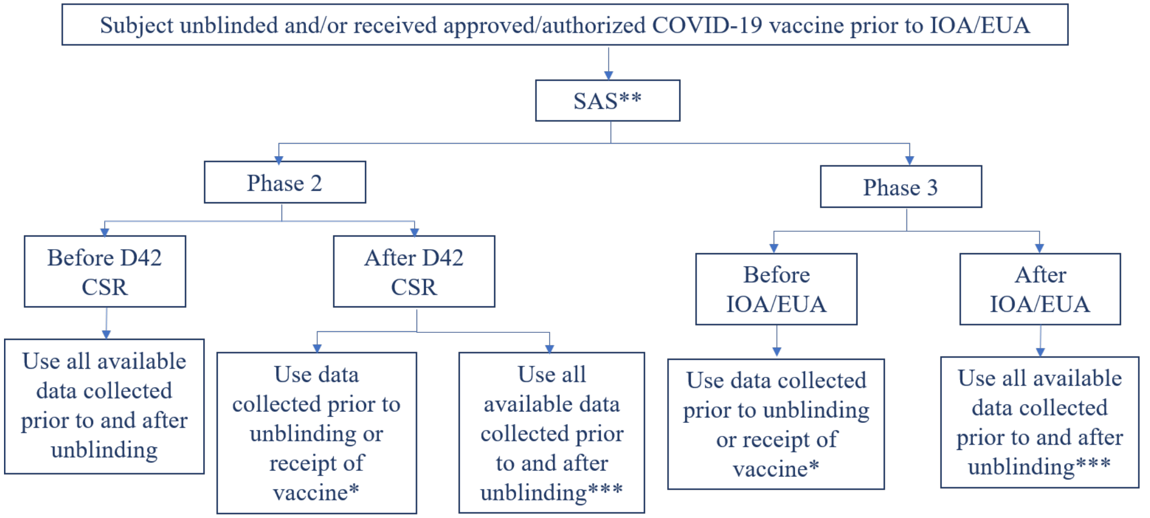

*whichever occurs first
**Subject who becomes unblinded or receives an approved or authorized COVID-19 vaccine will be included in the SAS however her/his data collected after unblinding or receipt of the vaccine, whichever comes first, will be excluded from some analyses, as noted in this flowchart.
***For subjects who receive the CoVLP formulation post-IOA/EUA, analyses will be presented separately for data collected prior to and after IOA/EUA

Figure 4 Decision Tree for Handling of Safety Data from Subjects Who Request to Become **Prematurely** Unblinded to Receive an Approved or Authorized COVID-19 Vaccine in Phase 2 and Phase 3 Portions **Prior to IOA/EUA**

For these particular subjects, the safety data collected will be analyzed in the following manner:

~~In the~~ Phase 2 portion:

- **Prior to Day 42 clinical study report (CSR):**
  - ~~the s~~Safety **analyses performed using safety** data collected from subjects who become unblinded because they requested to be unblinded for the

</td><td></td></tr>
</table>

#	Section (page #)	Change	Rationale
		purposes of receiving an approved or authorized COVID-19 vaccine will be analyzed using all available data collected prior and after unblinding;<br>• **After Day 42 CSR:**<br>o **Safety analyses performed using safety data collected prior to unblinding or receipt of an approved or authorized COVID-19 vaccine, which ever occurs first, from subjects in the SAS;**<br>o **In addition, safety analyses will also be performed using safety data collected from subjects who become unblinded because they requested to be unblinded for the purposes of receiving an approved or authorized COVID-19 vaccine will be analyzed using all available data collected prior and after unblinding. For subjects who receive the CoVLP formulation post-IOA/EUA, safety analyses will be presented separately for data collected prior to and after IOA/EUA.**<br><br>In the Phase 3 portion, the safety data collected from subjects who become unblinded because they requested to be unblinded for the purposes of receiving an approved or authorized COVID-19 vaccine will be analyzed in the following manner:<br><br>38. Prior to Emergency Use Authorization **IOA/EUA**:<br>• Safety analyses performed using safety data collected prior to unblinding or receipt of an approved or authorized COVID-19 vaccine, which ever occurs first, from subjects in the SAS;<br>39. After Emergency Use Authorization **IOA/EUA**:<br>• ~~Main safety analyses: safety analyses performed using safety data collected prior to unblinding or receipt of an approved or authorized COVID-19 vaccine, which ever occurs first, from subjects in the SAS;~~<br>• ~~Supplementary safety analyses: safety~~ **Safety** analyses performed using safety data collected prior to and after unblinding from subjects in the SAS;<br>• **For subjects who receive the CoVLP formulation post-IOA/EUA, safety analyses will be presented separately for data collected prior to and after IOA/EUA.**	

#	Section (page #)	Change	Rationale
50.	Section 12.1.3 (Per Protocol Set)	This section has been updated as follows:<br><br>The PP set will consist of a subset of the ITT set who participated in the study until the end of the surveillance period in the Phase 2 or Phase 3 portions of the study; had no major protocol deviations related to subject eligibility, the ability to develop a valid immune response, prohibited medication use, or the efficacy analyses; and who received both doses of either the CoVLP formulation or placebo. Major protocol deviations will be identified and documented during a blinded data review prior to database lock and confirmed at the time of database lock. The only exceptions for the PP set are the inclusion of subjects who become **prematurely** unblinded because they requested to be unblinded for the purposes of receiving an approved or authorized COVID-19 vaccine or subjects who received an approved or authorized COVID-19 vaccine (refer to Figure 5).<br>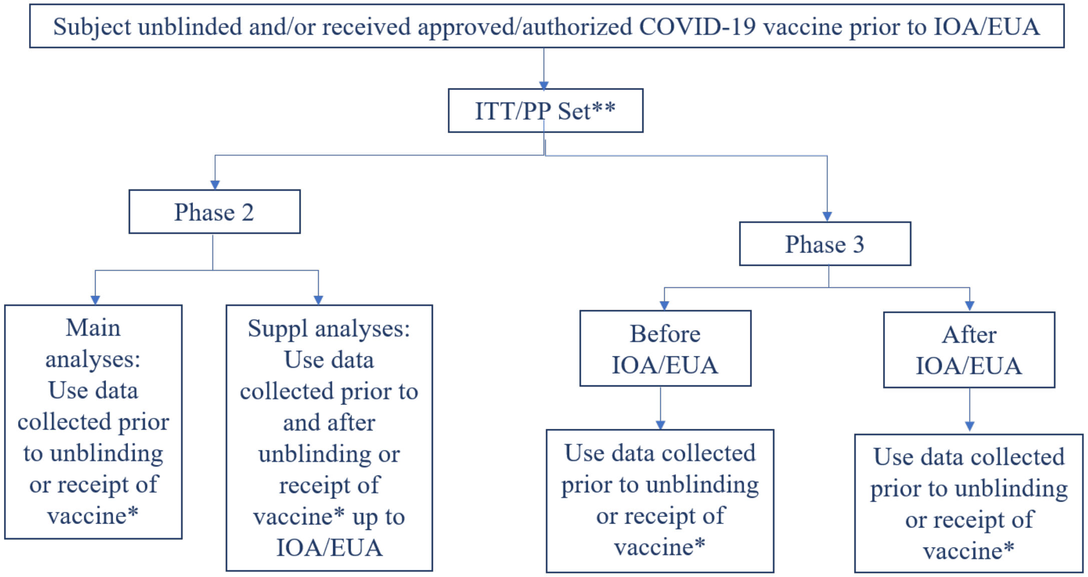<br><br>*whichever occurs first<br>**Subject who becomes unblinded or receives an approved or authorized COVID-19 vaccine will be included in the ITT/PP set however her/his data collected after unblinding or receipt of the vaccine, whichever comes first, will be excluded from some analyses, as noted in this flowchart.	The cross-over will no longer be used in the Phase 3 portion. Instead, once the CoVLP vaccine obtains IOA/EUA, the CoVLP vaccine will be offered to all placebo subjects in the Phase 2 and Phase 3 portions.

#	Section (page #)	Change	Rationale
		Figure 5 Decision Tree for Handling of Efficacy Data from Subjects Who Request to Become **Prematurely** Unblinded to Receive an Approved or Authorized COVID-19 Vaccine in Phase 2 and Phase 3 Portions **Prior to IOA/EUA**<br>For these particular subjects, the efficacy data collected will be analyzed in the following manner:<br>Phase 2 portion:<br>• Main efficacy analyses: efficacy analyses performed using efficacy data collected prior to unblinding or receipt of an approved or authorized COVID-19 vaccine, which ever occurs first, from subjects in the PP set and ITT set;<br>• Supplementary efficacy analyses: efficacy analyses performed using efficacy data collected prior to and after unblinding or receipt of an approved or authorized COVID-19 vaccine, which ever occurs first, from subjects in the PP set and ITT set **up to IOA/EUA**.<br>Phase 3 portion:<br>40. Prior to ~~Emergency Use Authorization~~ **IOA/EUA**:<br>• Efficacy analyses performed using efficacy data collected prior to unblinding or receipt of an approved or authorized COVID-19 vaccine, which ever occurs first, from subjects in the PP set and ITT set;<br>41. After ~~Emergency Use Authorization~~ **IOA/EUA**:<br>~~• Main efficacy analyses: efficacy analyses performed using efficacy data collected prior to unblinding or receipt of an approved or authorized COVID-19 vaccine, which ever occurs first, from subjects in the PP set and ITT set;~~<br>• Efficacy analyses based on asymptomatic cases performed using efficacy data collected prior to unblinding or receipt of an approved or authorized COVID-19 vaccine, which ever occurs first, from subjects in the PP set and ITT set;<br>~~• Supplementary efficacy analyses: efficacy analyses performed using efficacy data collected prior to and after unblinding or receipt of an approved or authorized COVID-19 vaccine, which ever occurs first, from subjects in the PP set and ITT set.~~	

#	Section (page #)	Change	Rationale
		Subjects who received the wrong treatment, but for whom the treatment received can be unequivocally confirmed, will be analyzed as treated, provided they have no other deviations that compromise their data.<br>In Phase 2, the analyses of all efficacy endpoints will be performed using the PP set as primary analysis and the ITT set as a secondary analysis population.<br>In Phase 3, the analyses of all efficacy endpoints will be performed using the ITT set as primary analysis and the PP set as a secondary analysis population. **In the Phase 3 portion, the PP set and the ITT set will be defined separately for each period.**	
51.	Section 12.1.4 (Immunogenicity Per Protocol Set)	This section has been updated as follows:<br>The IPP set will consist of a subset of the ITT set who participated in the immunogenicity portions of the Phase 2 or Phase 3 portions of the study and completed the study with no major deviations related to subject eligibility, the ability to develop a valid immune response, prohibited medication use, or the immunogenicity analyses; and who received either the CoVLP formulation or placebo. The only exceptions for the IPP set are the inclusion of subjects who become **prematurely** unblinded because they requested to be unblinded for the purposes of receiving an approved or authorized COVID-19 vaccine or subjects who received an approved or authorized COVID-19 vaccine (refer to Figure 6).	The cross-over will no longer be used in the Phase 3 portion. Instead, once the CoVLP vaccine obtains IOA/EUA, the CoVLP vaccine will be offered to all placebo subjects in the Phase 2 and Phase 3 portions.

#	Section (page #)	Change	Rationale
		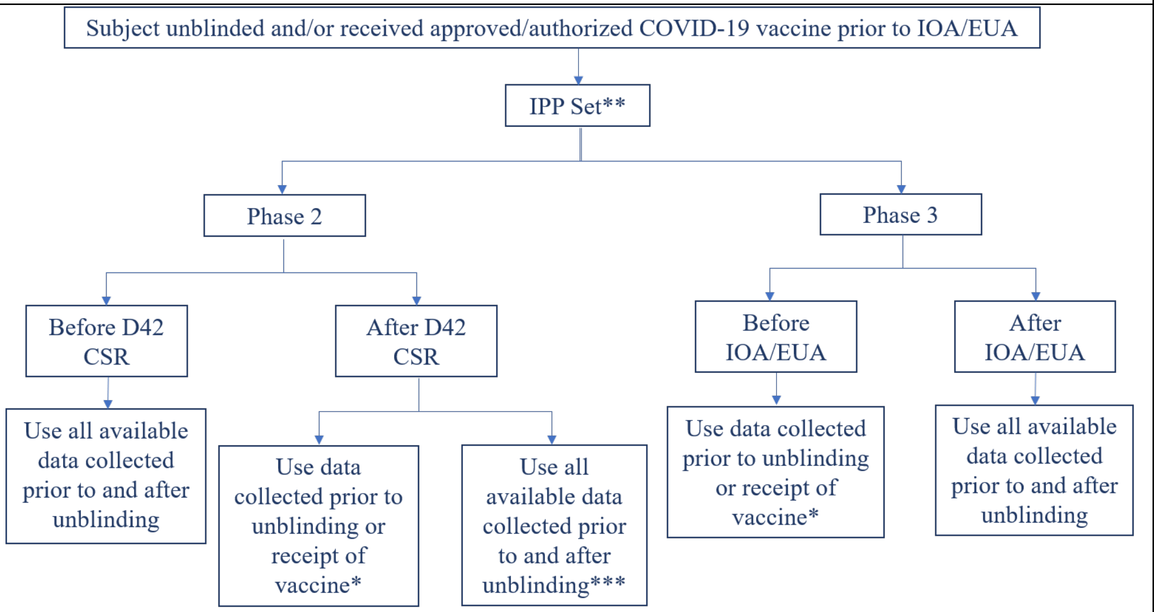<br><br>*whichever occurs first<br>**Subject who becomes unblinded or receives an approved or authorized COVID-19 vaccine will be included in the IPP set however her/his data collected after unblinding or receipt of the vaccine, whichever comes first, will be excluded from the analysis, as noted in this flowchart.<br>***For subjects who receive the CoVLP formulation post-IOA/EUA, analyses will be presented separately for data collected prior and after IOA/EUA.<br>Figure 6 Decision Tree for Handling of Immunogenicity Data from Subjects Who Request to Become **Prematurely** Unblinded to Receive an Approved or Authorized COVID-19 Vaccine in Phase 2 and Phase 3 Portions **Prior to IOA/EUA**<br>**Phase 2 portion:**<br>• **Prior to Day 42 CSR:**<br>○ **Immunogenicity analyses performed using immunogenicity data collected from subjects who become unblinded because they requested to be unblinded for the purposes of receiving an approved or authorized COVID-19 vaccine will be analyzed using all available data collected prior and after unblinding from subjects in the ITT;**<br>• **After Day 42 CSR:**	

#	Section (page #)	Change	Rationale
		○ **Immunogenicity analyses performed using immunogenicity data collected prior to unblinding or receipt of an approved or authorized COVID-19 vaccine, which ever occurs first, from subjects in the IPP/ITT;**<br>○ **In addition, immunogenicity analyses will also be performed using immunogenicity data collected from subjects who become unblinded because they requested to be unblinded for the purposes of receiving an approved or authorized COVID-19 vaccine will be analyzed using all available data collected prior and after unblinding, from subjects in the IPP/ITT. For subjects who receive the CoVLP formulation post-IOA/EUA, immunogenicity analyses will be presented separately for data collected prior to and after IOA/EUA.**<br><br>**Phase 3 portion:**<br>For these particular subjects, **the immunogenicity data collected will be analyzed in the following manner:** ~~all data collected before the time of unblinding or receipt of an approved or authorized COVID-19 vaccine, which ever occurs first, will be included in the immunogenicity analyses.~~<br>**42. Prior to IOA/EUA:**<br>• **Immunogenicity analyses performed using immunogenicity data collected prior to unblinding or receipt of an approved or authorized COVID-19 vaccine, which ever occurs first, from subjects in the IPP/ITT;**<br>**43. After IOA/EUA:**<br>• **Immunogenicity analyses performed using immunogenicity data collected prior to and after unblinding from subjects in the IPP/ITT.**<br><br>For the Day 21 analysis, this should include the subjects who received the first vaccine dose and had Day 0 and Day 21 immunogenicity sample collections. For the Day 42 analysis, this should include the subjects who received both vaccine doses and had Day 0 and Day 42 immunogenicity sample collections. For the Day 128 analysis, this should include the subjects who received both vaccine doses and had Day 0 and Day 128 immunogenicity sample collections. For the Day 201 analysis, this should include the	

#	Section (page #)	Change	Rationale
		subjects who received both vaccine doses and had Day 0 and Day 201 immunogenicity sample collections. For the Day 386 analysis, this should include the subjects who received both vaccine doses and had Day 0 and Day 386 immunogenicity sample collections. Subjects who had blood samples for immunogenicity taken outside of the time window for blood sample collection are to be excluded from the IPP set for the specific visit. Subjects who received the wrong treatment at the first vaccination, but for whom the treatment received can be unequivocally confirmed, will be analyzed as treated, provided they have no other deviations that compromise their data. Subjects who receive a treatment at the second vaccination that is not the same treatment received at the first vaccination will not be included in the IPP set at Day 42, Day 128, Day 201, and Day 386; the subjects will remain in the Day 21 analysis and analyzed as treated.<br>The analyses of all immunogenicity endpoints will be performed using the IPP set in both Phase 2 and Phase 3 portions, as the primary analysis population, and the ~~immunogenicity~~ ITT set, as a secondary analysis population. ~~In the Phase 3 portion, the IPP set and the immunogenicity ITT set will be defined separately for each period.~~	
52.	Section 12.5.2 (Analysis of Secondary Endpoints)	The fourth and fifth paragraphs have been updated as follows:<br>For the Phase 3 portion, immediate AEs (30 minutes after each vaccination) and solicited local and systemic AEs reported up to seven days after each vaccination **(for the initial set of vaccinations)** ~~(in Period 1 only)~~ will be summarized by treatment using descriptive statistics. In addition, all unsolicited AEs, SAEs, AEs leading to subject withdrawal, AESIs, MAAEs, and deaths reported up to 21 days after each vaccination will be summarized by treatment using descriptive statistics.<br>For the Phase 2 and Phase 3 portions, SAEs, AEs leading to subject withdrawal, AESIs, MAAEs, and deaths reported from Day 43 to the end of the study will be summarized by treatment**, when applicable,** using descriptive statistics.	The cross-over will no longer be used in the Phase 3 portion. Instead, once the CoVLP vaccine obtains IOA/EUA, the CoVLP vaccine will be offered to all placebo subjects in the Phase 2 and Phase 3 portions.
53.	Section 12.6.2 (Analysis of	The seventh, eighth, nineth, tenth, and eleventh paragraphs have been updated as follows:	Clarified the details of the timing of some of

#	Section (page #)	Change	Rationale
	Secondary Endpoints)	GMT will be compared between treatment groups**, when applicable,** using the analysis of variance (ANOVA) model. GMFR will be compared using the ANCOVA model. For SC rate, Fisher's exact tests will be used to compare between the treatment groups**, when applicable**.<br>The Nab/IgG ratio will be calculated as the antilog of the Nab/IgG ratio and 95 % CI of log transformed titer values. The ratio will be compared between treatment groups**, when applicable,** using ANOVA.<br>The specific Th1 CMI response induced on Day 201 and Day 386 will be measured by the number of T cells expressing IFN-γ, using ELISpot, for each treatment group. The specific Th2 CMI response induced on Day 201 and Day 386 will be measured by the number of T cells expressing IL-4, using ELISpot, for each treatment group. The responses will be compared between treatment groups**, when applicable,** and timepoints using appropriate non-parametric (Wilcoxon) models. Additional details will be provided in the SAP.<br>For the Phase 3 portion, the same immunogenicity analyses will be performed as described for the primary and secondary immunogenicity endpoints for the Phase 2 portion of the study (with the exception of the analysis on Day 128 **and the Nab GMT comparisons between study populations**). **The responses will be compared between treatment groups, only when applicable.**	the analyses as well as the conditions for the analyses.
54.	Section 12.6.3 (Analysis of Exploratory Endpoints)	The second paragraph has been updated as follows:<br>For the Phase 3 portion, the CMI response induced on Day 0, Day 21, Day 42, Day 201, and Day 386will be measured by the percentage of T cells expressing functional markers, using flow cytometry, for each treatment group. The responses will be compared between treatment groups**, when applicable,** and timepoints using appropriate non-parametric (Wilcoxon) models. Additional details will be provided in the SAP.	Clarified the details of the timing of some of the analyses as well as the conditions for the analyses.
55.	Section 12.7.1 (Analysis of Primary Endpoint)	The third and sixth paragraphs have been updated as follows:<br>VE will be evaluated for any strain ~~before cross-over~~ as the incidence rate ratio of CoVLP formulation vaccinated versus placebo subjects to develop laboratory-confirmed symptomatic SARS-CoV-2 infection ≥ 7 days after the second vaccination, as per the following formula:	The cross-over will no longer be used in the Phase 3 portion.

#	Section (page #)	Change	Rationale
		For both analysis sets, censoring will be performed when subjects ~~will~~ experience their first COVID-19 case, the date the database will be frozen for the primary analysis, ~~the subject's cross-over occurs,~~ the date subject is unblinded, the date **subject** receives an approved or authorized COVID-19 vaccine, or at the date of subject completion/withdrawal from the study, whichever occurs first. The VE success criterion for the primary efficacy endpoint, in the Phase 3 portion, is defined as a ≥ 50 % point estimate and a > 30 % lower limit of the 95 % CI.	
56.	Section 12.7.2 (Analysis of Secondary Endpoints)	The fourth, fifth, sixth, eighth paragraphs have been updated and a new paragraph has been added at the end as follows:<br>For the Phase 3 portion of the study, **secondary efficacy endpoints** VE will be evaluated **at the time of the PVE analysis** ~~prior to the cross-over for the secondary efficacy endpoints~~. VE will be evaluated by strain as the incidence rate ratio of CoVLP formulation vaccinated versus placebo subjects to develop laboratory-confirmed symptomatic SARS-CoV-2 infection after the second vaccination. VE will also be evaluated for any strain as the incidence rate ratio of CoVLP formulation vaccinated versus placebo subjects to develop severe COVID-19 disease ≥ 7 days after the second vaccination. In addition, VE will be evaluated for any strain as the incidence rate ratio of CoVLP formulation vaccinated versus placebo subjects to develop laboratory-confirmed symptomatic SARS-CoV-2 infection after the first vaccination, after first vaccination and prior to the second vaccination, and after the second vaccination and prior to 7 days post-second vaccination. **Post-IOA/EUA, the number of symptomatic cases collected from passive surveillance will be listed and will not be summarized by treatment.** ~~Similarly to the analysis of the primary efficacy endpoint, for the ITT set analysis, the number of cases included will depend on the endpoint, and the follow-up time will be calculated from the day of randomization through censoring time. For the PP set analysis the number of cases will be adjusted according to the endpoint as well as the follow-up time.~~<br>VE will be evaluated for any strain as the incidence rate ratio of CoVLP formulation vaccinated versus placebo subjects to develop laboratory-confirmed asymptomatic SARS-CoV-2 infection at Day 201 ~~386 in Period 1~~ in subjects who were seronegative at baseline (confirmed by the ELISA method for the N protein). **The number of cases in the CoVLP arm collected at Day 201 will be compared to the number of cases**	The cross-over will no longer be used in the Phase 3 portion. Instead, once the CoVLP vaccine obtains IOA/EUA, the CoVLP vaccine will be offered to all placebo subjects in the Phase 2 and Phase 3 portions.

#	Section (page #)	Change	Rationale
		**collected in the placebo arm prior to IOA/EUA.** Assuming the number of cases in each arm follows a Poisson distribution, then conditioning on the total number of cases and the ratio of person-time of follow up, exact 95 % CIs for the incidence rate ratio IRR, and hence for VE = 1-IRR, are obtained via binomial distribution calculations [Rothman 2008].<br>For the Phase 3 portion, **at the time of the PVE analysis,** the occurrences of COVID-19-related symptoms and their severity will be summarized by treatment using descriptive statistics. The occurrence of unusual manifestations of COVID-19 as well as the severity of COVID-19 symptoms will also be used to assess imbalance between the CoVLP and placebo groups as a possible indication of enhanced disease (full details provided in the Medical Management Plan and IDMC Charter).<br>**Similarly to the analysis of the primary efficacy endpoint, for the ITT set analysis, the number of cases included will depend on the endpoint, and the follow-up time will be calculated from the day of randomization through censoring time. For the PP set analysis the number of cases will be adjusted according to the endpoint as well as the follow-up time.**	
57.	Section 12.7.3 (Analysis of Exploratory Endpoints)	The third paragraph has been removed:<br>~~For the Phase 3 portion, the duration of protection and the corresponding 95 % CI in symptomatic subjects will be evaluated at the end of the study for any strain and by strain by assessing the coefficient for the product of the initial vaccine assignment and the time since vaccination using a Cox proportional hazard model parameterized with a linear decay on the log-hazard scale [Fintzi 2021]. A positive coefficient (θ_2 in Fintzi 2021) with significant p-value ($p < 0.05$) will indicate VE is waning through time. More details will be provided in the SAP.~~	Removed an efficacy analysis that cannot be performed since placebo subjects will be offered the CoVLP vaccine post-IOA/EUA.
58.	Section 13.1.3.1.2 (Hypersensitivity Reactions)	The section title and the last sentence of the paragraph have been updated as follows:<br>Hypersensitivity Reactions **(Anaphylaxis and Severe Allergic Reactions)**<br>To collect data on these events, Medicago will closely monitor and assess **anaphylaxis and severe** allergic reactions assessed as related to the investigational product as AESIs.	Clarified that AESIs also includes anaphylaxis and severe allergic reactions.

#	Section (page #)	Change	Rationale
59.	Section 14.1.3 (Preparation and Administration of Study Vaccine)	The third paragraph has been updated as follows:<br>During the Phase 2 portion and ~~each period of~~ the Phase 3 portion, the CoVLP formulation will be administered on study Day 0 and Day 21 as an IM injection of 0.5 mL, into the deltoid muscle. For subjects with BMI < 30 kg/m^2, a 23 gauge needle of at least one inch or 2.5 cm in length can be used for vaccination. For subjects with BMI ≥ 30 kg/m^2, a needle of at least 1.5 inches or 3.5 cm in length must be used to ensure intramuscular delivery. **In addition, subjects in the placebo group will be offered the CoVLP formulation once IOA/EUA for the CoVLP formulation is granted.**	The cross-over will no longer be used in the Phase 3 portion. Instead, once the CoVLP vaccine obtains IOA/EUA, the CoVLP vaccine will be offered to all placebo subjects in the Phase 2 and Phase 3 portions.
60.	Section 17.10.1 (Publication Release of Results)	This new section has been added with the following content:<br>**As clinical data becomes available in the Phase 2 and Phase 3 portions, Medicago's intent is to release and/or publish this data after the clinical data have been presented to Regulatory Authority(ies). There may be multiple clinical data releases and/or publications generated over the course of the Phase 2 and Phase 3 portions as the clinical data are presented to Regulatory Authority(ies). Moreover, the clinical data that are released and/or published will only present group level results and will not involve any unblinding or the presentation of subject level data.**	Clarified that study results may be released and/or published as they are available and after presenting the results to Regulatory Authority.
61.	Section 18 (References)	The following reference has been removed:<br>~~Fintzi, J. and Follman, D. 2021. Assessing Vaccine Durability in Randomized Trials Following Placebo Crossover: pp.1-28. arXiv:2101.01295 [stat.AP]~~	Removed a reference that is no longer relevant.
62.	Section 19.7 (Appendix 7)	The following statement has been removed from the cell for the last row and second column in Table 14:<br>~~Go/No go for this population for Health Canada~~	Clarified the context of the meeting.

Table 4 Summary of Changes from Protocol Version 5.2 to Version 6.0 (Effective Date 26 April 2021)

#	Section (page #)	Change	Rationale
1.	Synopsis (Study Rationale)<br>AND<br>Section 1.5 (Overall Rationale for the Study)	The sixth and last paragraphs in this section have been updated as follows:<br>Emergency use authorization or approval will be pursued after the success criterion has been met for the primary efficacy endpoint of the Phase 3 portion. The primary efficacy endpoint will be evaluated once at least 160 laboratory-confirmed COVID-19 cases have been accumulated **and the median safety follow-up duration of at least 2 months (post-administration of the second vaccination) of at least 3000 subjects in each of the CoVLP formulation and placebo groups has been achieved**.<br>~~In addition, a subset of 400 subject from the United Kingdom (UK; UK-CMI subset) will be included in the exploratory evaluation of cell-mediated immunity (CMI) in Period 1 of the Phase 3 portion of the study.~~	The evaluation of cell-mediated immunity in Period 1 of the Phase 3 portion in a subset of subjects from the UK will no longer be performed.
2.	Synopsis (Primary Objectives – Phase 2 Portion and Phase 3 Portion)<br>AND<br>Section 2.1 (Primary Objectives)	The primary efficacy objective of the Phase 3 portion has been updated as follows:<br>Efficacy:<br>44. To evaluate the efficacy of the CoVLP formulation, compared to placebo, in the prevention of laboratory-confirmed symptomatic SARS-CoV-2 infection (virologic method) starting 7 days after the second vaccination **at the time of the primary vaccine efficacy (PVE) analysis (i.e., once at least 160 COVID-19 cases [≥ 7 days post-second vaccination] have been collected and the median safety follow-up duration of at least 2 months (post-administration of the second vaccination) of at least 3000 subjects in each of the CoVLP formulation and placebo groups has been achieved)** ~~in Period 1~~ and prior to the cross-over.	Clarified when this objective will be assessed during the study.
3.	Synopsis (Secondary Objectives – Phase 2 Portion and Phase 3 Portion)<br>AND	The secondary efficacy and immunogenicity objective of the Phase 3 portion have been updated as follows:<br>Efficacy:<br>45. To evaluate the efficacy of the CoVLP formulation, compared to placebo, in the prevention of laboratory-confirmed asymptomatic SARS-CoV-2 infection **at cross-over and Day 386** ~~(serologic method) starting 7 days after the second~~	Clarified when these objectives will be assessed during the study. Also, the assessment of the CMI (CD+4)

#	Section (page #)	Change	Rationale
	Section 2.2 (Secondary Objectives)	~~vaccination~~ in Period 1 **in subjects who were seronegative at baseline (confirmed by serologic method)** ~~and prior to the cross-over~~;<br>46. To evaluate the efficacy of the CoVLP formulation, compared to placebo, in the prevention of severe COVID-19 disease starting 7 days after the second vaccination **at the time of the PVE analysis** ~~in Period 1~~ and prior to the cross-over (see definition of severe COVID-19 disease above);<br>47. To assess COVID-19-related symptoms in virologically-confirmed cases, and the severity of these symptoms up until resolution in subjects administered the CoVLP formulation in Period 1 compared to subjects administered the placebo and compared to subjects administered CoVLP at the time of cross-over in Period 2;<br>48. To evaluate the efficacy of the CoVLP formulation, compared to placebo, in the prevention of laboratory-confirmed symptomatic SARS-CoV-2 infection (virologic method) starting after the first vaccination **at the time of the PVE analysis** ~~in Period 1~~ and prior to the cross-over;<br>49. To evaluate the efficacy of the CoVLP formulation, compared to placebo, in the prevention of laboratory-confirmed symptomatic SARS-CoV-2 infection (virologic method) after the first vaccination and prior to the second vaccination **at the time of the PVE analysis** ~~in Period 1~~, and prior to the cross-over;<br>50. To evaluate the efficacy of the CoVLP formulation, compared to placebo, in the prevention of laboratory-confirmed symptomatic SARS-CoV-2 infection (virologic method) after the second vaccination and prior to 7 days post-second vaccination **at the time of the PVE analysis** ~~in Period 1~~, and prior to the cross-over;<br>~~51. To evaluate the efficacy of the CoVLP formulation, in subjects who only received a single dose compared to placebo, in the prevention of laboratory-confirmed asymptomatic SARS-CoV-2 infection (virologic and serologic methods) at starting 7 days after the vaccination in Period 1 and prior to the cross-over;~~	response in Phase 3 was moved from a secondary objective to an exploratory objective.

#	Section (page #)	Change	Rationale
		52. To assess the duration and intensity of viral shedding after virologically-confirmed SARS-CoV-2 infection in subjects administered the CoVLP formulation compared to subjects administered the placebo **at the time of the PVE analysis** ~~in Period 1~~ and prior to the cross-over;<br>53. To evaluate the efficacy of the CoVLP formulation, compared to placebo, in the prevention of laboratory-confirmed symptomatic SARS-CoV-2 infection (virologic method) **by strain** starting 7 days after the second vaccination **at the time of the PVE analysis** ~~in Period 1~~ ~~by strain~~ and prior to the cross-over;<br>Immunogenicity:<br>54. To assess the immunogenicity of the CoVLP formulation, compared to placebo, in the immunogenicity subset prior to and post-cross-over, as determined by:<br>55. **Nab** ~~Neutralizing antibody~~ titers induced by the vaccine against the SARS-CoV-2 virus at Day 0 (prior to vaccination; all subjects), Day 21, Day 42 (all subjects), Day 201 and Day 386;<br>56. IgG (ELISA) titers induced by the vaccine against the SARS-CoV-2 virus up to 21 days after the second vaccination and at Day 201 and Day 386;<br>57. The ratio of **Nab** ~~neutralizing antibody~~ titers:IgG (ELISA) antibody titers at Day 21, Day 42, Day 201 and Day 386;<br>58. IFN-γ ELISpot response induced by the vaccine against the SARS-CoV-2 virus up to 21 days after the second vaccination and at Day 201 and Day 386;<br>59. IL-4 ELISpot response induced by the vaccine against the SARS-CoV-2 virus up to 21 days after the second vaccination and at Day 201 and Day 386;<br>~~60. Specific cell-mediated immune (CMI) response induced by the vaccine against the SARS-CoV-2 virus up to 21 days after the second vaccination and at Day 201 and Day 386, as measured by the percentage of CD4+ T cells expressing functional markers;~~	

#	Section (page #)	Change	Rationale
4.	Synopsis (Exploratory Objectives – Phase 2 Portion and Phase 3 Portion)<br>AND<br>Section 2.3 (Exploratory Objectives)	The exploratory efficacy objectives of the Phase 3 portions have been updated as follows:<br>Phase 3 portion<br>Efficacy:<br>61. To evaluate the duration of protection (vaccine efficacy versus virologically-confirmed symptomatic disease as well as serologically-confirmed asymptomatic disease) of the CoVLP formulation in subjects initially randomized to the CoVLP group compared to the original placebo group for any strain and by strain, **at the end of the study** ~~after the cross-over~~;<br>62. To evaluate the efficacy of the CoVLP formulation, compared to placebo, in the prevention of severe COVID-19 disease by strain starting 7 days after the second vaccination **at the time of the PVE analysis** ~~in Period 1~~ and prior to the cross-over (see definition of severe COVID-19 disease above);<br>Immunogenicity:<br>**63. To assess the immunogenicity of the CoVLP formulation, compared to placebo, in the immunogenicity subset prior to and post-cross-over, as determined by:**<br>64. **Specific cell-mediated immune (CMI) response induced by the vaccine against the SARS-CoV-2 virus up to 21 days after the second vaccination and at Day 201 and Day 386, as measured by the percentage of CD4+ T cells expressing functional markers;**<br>65. To further characterize the immune response of the CoVLP formulation, if it is likely to be informative;	Clarified when these objectives will be assessed during the study. Also, the assessment of the CMI (CD+4) response in Phase 3 was moved from a secondary objective to an exploratory objective.
5.	Synopsis (Number of Planned Subjects)	The fourth and last paragraphs in this section have been updated as follows:<br>Only a subset of approximately 288 subjects (96 subjects from each Study Population) will be included in the immunogenicity evaluation. These subjects will be enrolled from selected sites~~ in Canada and/or the US that will be defined prior to the start of the Phase 3 portion~~. **Subjects that have consented to participate in this subset will be selected after the Investigational Product randomization has been performed in the randomization and trial supply management (RTSM) system.**	The evaluation of cell-mediated immunity in Period 1 of the Phase 3 portion in a subset of subjects from the

#	Section (page #)	Change	Rationale
		**Immunogenicity subset subjects will be randomized 5:1 CoVLP:placebo. Therefore, some subjects that opt-in to the subset may not be selected after the secondary randomization.**<br>~~In addition, a subset of approximately 400 subjects from the UK will be included in an exploratory evaluation of cell-mediated immunity. These subjects will be enrolled from selected sites in the UK that will be defined prior to the start of the Phase 3 portion in this country.~~	UK will no longer be performed. Also, provided additional details regarding the randomization of the subjects in the immunogenicity subset.
6.	Synopsis (Sample Size Determination)<br>AND<br>Section 12.2 (Sample Size Determination)	The last paragraph in this section has been removed:<br>~~A UK-CMI subset sample size of 400 subjects is deemed sufficient to provide meaningful statistical assessment of immunologic outcomes.~~	The evaluation of cell-mediated immunity in Period 1 of the Phase 3 portion in a subset of subjects from the UK will no longer be performed.
7.	Synopsis (Immunogenicity Evaluations)<br>AND<br>Section 10.3.1 (Immunogenicity Evaluations)	The last paragraph in this section has been removed:<br>~~In addition, during Period 1 of the Phase 3 portion, cell-mediated immunity will be evaluated in the UK-CMI subset of 400 subjects on Day 0 and Day 42 using standardized methods for ELISpot and flow cytometry (ICS).~~	The evaluation of cell-mediated immunity in Period 1 of the Phase 3 portion in a subset of subjects from the UK will no longer be performed.
8.	Synopsis (Efficacy Evaluations)<br>AND	The first two paragraphs in this section have been updated as follows:<br>Following the first vaccination, subjects will be asked to report symptoms associated with COVID-19 from the day of the first vaccination (Day 0, post vaccination) until the end of the Phase 2 portion or the Phase 3 portion of the study (through active or passive surveillance). COVID-19 will be defined as the presence of a laboratory-	Clarified that subjects who test positive for SARS-CoV-2 infection

#	Section (page #)	Change	Rationale
	Section 10.4.1 (Efficacy Evaluations)	confirmed (virologic method) SARS-CoV-2 infection with the occurrence of a new onset of one or more of the following symptoms ~~that persist(s) for or reoccur(s) after a period of at least 12 hours~~:<br>• Fever or chills;<br>• Cough;<br>• Shortness of breath or difficulty breathing;<br>• Fatigue;<br>• Muscle or body aches;<br>• Headache;<br>• New loss of taste or smell;<br>• Sore throat;<br>• Congestion or runny nose;<br>• Nausea or vomiting;<br>• Diarrhea.<br>**Subjects will also be advised to inform the clinical site if they have tested positive for SARS-CoV-2 infection, with or without the presence of any symptoms associated with COVID-19, and will be asked to provide a documented test result. If/when the subject begins to experience symptoms associated with COVID-19, the process for the collection of symptoms and nasal or nasopharyngeal (NP) swabs (as noted below) will be followed.**	should notify the clinical site.
9.	Synopsis (Primary Endpoints - Efficacy)<br>AND<br>Section 10.4.2.1 (Primary Endpoint)	The following Efficacy primary endpoint has been updated as follows:<br>In the Phase 3 portion, the primary endpoint is:<br>• First occurrence, in a subject, of laboratory-confirmed (virologic method) symptomatic SARS-CoV-2 infection (≥ 7 days post-second vaccination) **at the time of the primary analysis (i.e., once at least 160 COVID-19 cases [≥ 7 days post-second vaccination] have been collected)** ~~in Period 1~~ and prior to cross-over.	Clarified when this endpoint will be assessed during the study.

#	Section (page #)	Change	Rationale
10.	Synopsis (Secondary Endpoints – Efficacy)<br>AND<br>Section 10.4.2.2 (Secondary Endpoints)	The following Efficacy secondary endpoints have been updated as follows:<br>• Occurrences of laboratory-confirmed ~~(confirmed by the ELISA method for the N protein)~~ asymptomatic SARS-CoV-2 infection **at cross-over and Day 386** ~~starting 7 days after the second vaccination~~ in Period 1 **in subjects who were seronegative at baseline (confirmed by the ELISA method for the N protein)** ~~and prior to the cross-over~~;<br>• **Occurre**nces of severe COVID-19 disease (≥ 7 days post-second vaccination) **at the time of the PVE analysis** ~~in Period 1~~ and prior to cross-over. Refer to the definition of severe COVID-19 disease in the Secondary Objectives section above;<br>• Occurrence and intensity of COVID-19-related symptoms in virologically-confirmed cases up until resolution of the symptoms in Period 1 compared to subjects administered the placebo and compared to subjects administered CoVLP at the time of cross-over in Period 2;<br>• First occurrence, in a subject, of laboratory-confirmed (virologic method) symptomatic SARS-CoV-2 infection (≥ first vaccination) **at the time of the PVE analysis** ~~in Period 1~~ and prior to cross-over;<br>• First occurrence, in a subject, of laboratory-confirmed (virologic method) symptomatic SARS-CoV-2 infection (≥ first vaccination and < second vaccination) **at the time of the PVE analysis** ~~in Period 1~~ and prior to cross-over;<br>• First occurrence, in a subject, of laboratory-confirmed (virologic method) symptomatic SARS-CoV-2 infection (≥ second vaccination and < 7 days post-second vaccination) **at the time of the PVE analysis** ~~in Period 1~~ and prior to cross-over;<br>• ~~Occurrences of laboratory-confirmed (confirmed by the ELISA method for the N protein) asymptomatic SARS-CoV-2 infection starting 7 days after the vaccination in Period 1 and prior to the cross-over in subjects who only received a single vaccination;~~	Clarified when these endpoints will be assessed during the study.

#	Section (page #)	Change	Rationale
		• Duration and intensity of viral shedding after virologically-confirmed SARS-CoV-2 infection **at the time of the PVE analysis** ~~in Period 1~~ and prior to cross-over;<br>• First occurrence, in a subject, of laboratory-confirmed (virologic method) symptomatic SARS-CoV-2 infection (≥ 7 days post-second vaccination) **at the time of the PVE analysis** ~~in Period 1~~ by strain and prior to cross-over;	
11.	Synopsis (Secondary Endpoints – Immunogenicity)<br>AND<br>Section 10.3.2.2 (Secondary Endpoints)	The sixth Immunogenicity secondary endpoint has been removed:<br>**Immunogenicity:**<br>~~• In the immunogenicity subset prior to cross-over and post-cross-over, specific CMI response induced in each Study Population against the SARS-CoV-2 virus up to 21 days after the second vaccination and at Day 201 and Day 386, as measured by the percentage of CD4+ T cells expressing functional markers;~~	The assessment of the CMI (CD+4) response in Phase 3 was moved from a secondary objective to an exploratory objective.
12.	Synopsis (Exploratory Endpoints – Immunogenicity)<br>AND<br>Section 10.3.2.3 (Exploratory Endpoints)	The following Immunogenicity exploratory endpoints have been updated as follows:<br>**Immunogenicity:**<br>• **In the immunogenicity subset prior to cross-over and post-cross-over, specific CMI response induced in each Study Population against the SARS-CoV-2 virus up to 21 days after the second vaccination and at Day 201 and Day 386, as measured by the percentage of CD4+ T cells expressing functional markers;**<br>• Further characterization of the immune response of the CoVLP formulation, if it is likely to be informative;	The assessment of the CMI (CD+4) response in Phase 3 was moved from a secondary objective to an exploratory objective.
13.	Synopsis (Statistical Methods – Populations)	The following paragraph has been added at the end of this section:<br>**As approved or authorized vaccines for COVID-19 become publicly available to subjects in some regions, subjects may request to become prematurely unblinded. The criteria to become prematurely unblinded in this circumstance is discussed in Section 6.2 and subject status upon premature unblinding is detailed in Section 11.6. Details of how the data for prematurely unblinded subjects will be handled is described in Section 12.1.**	Included details in the protocol on how subjects who request to be unblinded to receive the approved or

#	Section (page #)	Change	Rationale
			authorized COVID-19 vaccine will be unblinded, their status in the study, and how their data will be handled.
14.	Synopsis (Statistical Methods – Phase 3 Portion Interim Efficacy Analyses)<br>AND<br>Section 12.3 (Day 21 and Day 43 Immunogenicity Data Analysis and Interim Efficacy Analyses)	The section header and first paragraph have been updated as follows:<br>Phase 3 portion: Efficacy **and Safety** Analys~~i~~es:<br>When at least 160 laboratory-confirmed COVID-19 cases (≥ 7 days post-second vaccination) have been collected **and the median safety follow-up duration of at least 2 months (post-administration of the second vaccination) of at least 3000 subjects in each of the CoVLP formulation and placebo groups has been achieved**, the unblinded statistical team will perform the final analysis and produce final efficacy outputs for the Phase 3 portion of the efficacy data in order to test for vaccine efficacy. If the primary efficacy success criterion is met during this analysis, emergency use authorization or approval will be pursued and initiation of the cross-over design. Initiation of the cross-over design for subjects enrolled in the study will be dependent on the following overall study and subject-specific criteria:	Based on BRDD feedback, clarified that the primary efficacy analysis will be performed once the required minimal safety data has also been collected.
15.	Synopsis (Statistical Methods – Phase 3 Portion Initiation)<br>AND<br>Section 12.4 (Initiation of the Phase 3 Portion)	The second bullet point of the first paragraph has been updated as follows:<br>The start of the Phase 3 portion of the study will be determined independently for each Study Population and will be based on the outcomes of the following two assessments during the Phase 2 portion of the study:<br>• Day 21 Data Analysis of the immunogenicity data in Study Population #1 (described above);<br>• **Day 21 and/or** Day 42 Data Analysis of the immunogenicity data in Study Population #2 and Study Population #3 (described above);	Updated information based on the latest information for Study Populations proceeding to the Phase 3 portion.

#	Section (page #)	Change	Rationale
		• Day 28 Safety Review in Study Population #1, Study Population #2, and Study Population #3 (described in the Safety Review section above).	
16.	Table 2 (Time and Events Schedule: General Information for the Phase 3 Portion)	The time and events schedule table for the Phase 3 portion has been split into two tables to separate the activities of Period 1 and Period 2. Each table for each period now represents period-specific events and in each table, the description for collecting nasal/NP swabs was further expanded to incorporate additional details (consistent with the content in the Section 10.4.1 Efficacy Evaluation).<br>In addition, the following row and footnote were removed from the table:<br>The following updates have been made to the table:<br>~~66. A new row, "Immunogenicity – UK CMI (ELISpot and ICS)" as been added with the "X[9,22]" placed in the "Day 0" column and the "X[22]" placed in the "Day 42" column;~~<br>~~67. Footnote 22: [22] Samples for the immunogenicity UK CMI (ELISpot and ICS) assessment will be collected at Day 0 and Day 42 from 400 subjects included in the UK-CMI subset in Period 1 only.~~	Separated the table into two tables and added additional details based on BRDD feedback. Also, the evaluation of cell-mediated immunity in Period 1 of the Phase 3 portion in a subset of subjects from the UK will no longer be performed.
17.	Section 5.1 (Inclusion Criteria)	The first paragraph of inclusion criterion #7 has been updated as follows:<br>7. <u>Study Populations #1 and #3</u>: Female subjects of childbearing potential must use an effective method of contraception for one month prior to vaccination (Visit 2 **for the Phase 2 portion and Visit 1 for the Phase 3 portion**) and agree to continue employing highly effective birth control measures for at least one month after the last study vaccination (or in the case of early termination, she must not plan to become pregnant for at least one month after her last study vaccination).	Clarified the visit for Phase 2 and Phase 3 portions.
18.	Section 5.2 (Exclusion Criteria)	The following exclusion criteria have been updated as follows:<br>5. <u>Study Populations #1 and #2</u>: Administration of any medication or treatment that may alter the vaccine immune responses, such as:<br>• Systemic glucocorticoids at a dose exceeding 10 mg of prednisone (or equivalent) per day for more than seven consecutive days or for 10 or more days in total, within one month prior to the Vaccination visit (Visit 2 **for the**	Clarified the visits for Phase 2 and Phase 3 portions.

#	Section (page #)	Change	Rationale
		**Phase 2 portion and Visit 1 for the Phase 3 portion**). Inhaled, nasal, ophthalmic, dermatological, and other topical glucocorticoids are permitted;<br>• Cytotoxic, antineoplastic, or immunosuppressant drugs - within 36 months prior to Vaccination (Visit 2 **for the Phase 2 portion and Visit 1 for the Phase 3 portion**);<br>• Any immunoglobulin preparations or blood products, blood transfusion – within 6 months prior to Vaccination (Visit 2 **for the Phase 2 portion and Visit 1 for the Phase 3 portion**);<br>7. Administration of any vaccine within 14 days prior to Vaccination (Visit 2 **for the Phase 2 portion and Visit 1 for the Phase 3 portion**); planned administration of any vaccine during the study (up to Day 28 of the study). Immunization on an emergency basis during the study will be evaluated on case-by-case basis by the Investigator;<br>10. Use of any investigational or non-registered product within 30 days or 5 half-lives, whichever is longer, prior to Vaccination (Visit 2 **for the Phase 2 portion and Visit 1 for the Phase 3 portion**) or planned use during the study period. Subjects who are in a prolonged post-administration observation period of another investigational or marketed drug clinical study, for which there is no ongoing exposure to the investigational or marketed product and all scheduled on-site visits are completed, will be allowed to take part in this study, if all other eligibility criteria are met;<br>12. Use of any prescription antiviral drugs with the intention of COVID-19 prophylaxis, including those that are thought to be effective for prevention of COVID-19 but have not been licensed for this indication, within one month prior to Vaccination (Visit 2 **for the Phase 2 portion and Visit 1 for the Phase 3 portion**);	
19.	Section 6.1 (Randomization)	The third paragraph in this section has been removed:<br>~~For the UK-CMI subset in the Phase 3 portion, subjects will be enrolled from select clinical sites in the UK. At the clinical sites, selection of the subjects to be included in this subset will be performed by the unblinded statistical team or the IRT system~~	The evaluation of cell-mediated immunity in Period 1 of the Phase 3 portion in

#	Section (page #)	Change	Rationale
		~~sequentially starting with the first subject of the day, based on age distribution (when applicable), and treatment received until the targeted sample size of the site is obtained.~~	a subset of subjects from the UK will no longer be performed.
20.	Section 6.2 (Blinding)	The third and fourth paragraphs have been updated and new content has been added after the sixth paragraph as follows:<br>During the study, the IDMC and unblinded team, compromised of the unblinded medical monitor, unblinded biostatistician, unblinded senior reviewer, and unblinded programmer involved in the preparation of the safety data summary for the IDMC reviews, will have access to or be aware of treatment allocation (i.e. be "unblinded"). The IDMC will review safety data and will make recommendations for the entire duration of the study. The same unblinded statistical team will be involved in the Day 21 and Day 42 (if needed) immunogenicity data analyses in the Phase 2 portion and in the ~~interim~~ **primary vaccine** efficacy **(PVE)** analyses in the Phase 3 portion.<br>During the study, the Sponsor's Study Team will remain blinded. A small number of Medicago personnel (not included in Study Team) will be involved in the planned analyses during the Phase 2 and Phase 3 portions. **During the PVE analyses in the Phase 3 portion, some Medicago personnel will be unblinded at the group level to support the review of the analyses however these individuals will NOT be unblinded at the treatment allocation level.** The selected individuals and the process and level of unblinding will be documented.<br>**As approved or authorized vaccines for COVID-19 become publicly available to subjects in some regions, subjects may request to become prematurely unblinded. In order to proceed with any such requests for unblinding in the Phase 2 or Phase 3 portion, the following two criteria must be met:**<br>**1. Subject is eligible for an approved or authorized COVID-19 vaccine in their local region, and**<br>**2. Subject has been approached or received notification by a healthcare provider to receive a publicly available approved or authorized COVID-19 vaccine. The notification must be shared with the Investigator.**	Included details in the protocol on how subjects who request to be unblinded to receive the approved or authorized COVID-19 vaccine will be unblinded, their status in the study, and how their data will be handled.

<table>
<tr><th>#</th><th>Section (page #)</th><th>Change</th><th>Rationale</th></tr>
<tr><td></td><td></td><td>If the subject fulfils requirements of the two criteria, then the date of unblinding will be captured in the IRT system. If the subject does not meet both criteria, he/she will not be eligible for unblinding. Details regarding the subject's status in the study are included in Section 11.6.
Serological testing performed/requested by study subjects or performed as part of any medical procedures (e.g. blood/plasma donation) that may result in treatment unblinding is not permitted.</td><td></td></tr>
<tr><td>21.</td><td>Section 8.2 (Collection of Samples)</td><td>The first and third paragraphs and the second table in this section have been updated as follows:
In the Phase 3 portion of the study, all subjects will have up to a total of 55 mL of blood drawn. Subjects included in the immunogenicity subset will have up to an additional blood volume of up to approximately 500 mL drawn up to the end of the study. ~~Subjects included in the UK-CMI subset will have up to an additional blood volume of up to approximately 60 mL drawn up to the end of Period 1.~~ In total, subjects will have up to 55 to 555 mL of blood drawn up to the end of the study (Table ~~8~~9).
Table 9~~8~~ Estimated Blood Volume Drawn: Phase 3 Portion
<table>
<tr><th rowspan="2">Type of Sample</th><th rowspan="2">Volume per Sample (mL)</th><th colspan="6">Number of Samples per Subject</th><th rowspan="2">Total Volume of Blood per Subject per Period (mL)</th><th rowspan="2">Total Volume of Blood per Subject (mL)</th></tr>
<tr><th>Visit 1 (Day 0)</th><th>Visit 2 (Day 21)</th><th>Visit 3 (Day 42)</th><th>Visit 4 (Day 201)[§]</th><th>Visit 5 (Day 386)[§]</th><th>Total</th></tr>
<tr><td>Test for SARS-CoV-2 antibodies</td><td>5</td><td>1[†]</td><td></td><td></td><td></td><td>1[‡]</td><td>2</td><td>10</td><td>15[†,‡]</td></tr>
<tr><td>Immunogenicity – serology (Nab assay only)</td><td>10</td><td>1</td><td></td><td>1</td><td></td><td></td><td>2</td><td>20</td><td>40</td></tr>
<tr><td>~~Immunogenicity – UK-CMI (ELISpot and ICS)*~~</td><td>~~30~~</td><td>~~1~~</td><td></td><td>~~1~~</td><td></td><td></td><td>~~2~~</td><td>~~60~~</td><td>~~60~~</td></tr>
<tr><td>Immunogenicity - serology (Nab assay, ELISA)*</td><td>10</td><td>1</td><td>1</td><td>1</td><td>1</td><td>1</td><td>5</td><td>50</td><td>100</td></tr>
</table></td><td>The evaluation of cell-mediated immunity in Period 1 of the Phase 3 portion in a subset of subjects from the UK will no longer be performed.</td></tr>
</table>

<table>
<tr><th>#</th><th>Section (page #)</th><th>Change</th><th>Rationale</th></tr>
<tr><td></td><td></td><td>
<table>
<tr><td>Immunogenicity - CMI response (ELISpot and ICS)*</td><td>40</td><td>1</td><td>1</td><td>1</td><td>1</td><td>1</td><td>5</td><td>200</td><td>400</td></tr>
<tr><td>Total volume of blood per subject (mL)</td><td></td><td>15-65</td><td>50</td><td>10-60</td><td>50</td><td>5-55</td><td></td><td>30-280</td><td>55-555</td></tr>
</table>
* These samples will be collected for ~~a~~ pre-defined subset~~s~~ of subjects.
† The Day 0 sample will be collected prior to vaccination in Period 1 and Period 2. The sample collected prior to vaccination in Period 2 represents the sample collected “at cross-over” for the purposes of the efficacy evaluation.
‡ The Day 386 sample will only be collected during the visit for Day 386 of Period 1.
§ Based on the expected SARS-CoV-2 attack rate in unvaccinated subjects and the expected rate of subject enrollment in the Phase 3 portion, it is assumed that cross-over will occur prior to Day 201 (more than 45 days before Day 201), thus the Day 201 visit of Period 1 will occur at approximately the same time as the Day 42 visit of Period 2 (i.e., on the same day). If cross-over occurs within 45 days before the Day 201 visit, then the Day 201 visit of Period 1 will occur at approximately the same time as the Day 0 visit of Period 2 (i.e., on the same day). If cross-over occurs prior to Day 386, thus the Day 386 visit of Period 1 will occur at approximately the same time as the Day 201 visit of Period 2 (i.e., on the same day).
</td><td></td></tr>
<tr><td>22.</td><td>Section 10.1.4.1 (Prior to Vaccination)</td><td>The third sub-bullet of the last bullet point in this section has been removed:
• ~~Immunogenicity (UK CMI [ELISpot and ICS]) assessment (only Period 1 of Phase 3 portion – UK-CMI subset);~~</td><td>The evaluation of cell-mediated immunity in Period 1 of the Phase 3 portion in a subset of subjects from the UK will no longer be performed.</td></tr>
<tr><td>23.</td><td>Section 10.1.4.2 (Vaccination)</td><td>The fifth sentence of the paragraph has been updated as follows:
For subjects ~~in Study Population #3~~ with BMI ≥ 30 kg/m^2, a needle of at least 1.5 inches or 3.5 cm in length must be used to ensure intramuscular delivery.</td><td>Clarified that the needle length requirements based no BMI are not specific to</td></tr>
</table>

#	Section (page #)	Change	Rationale
			Study Population #3.
24.	Section 10.1.4.3 (Thirty Minutes Post-vaccination)	The first bullet point has been updated as follows:<br>• Subjects will remain in the clinic for at least 30 minutes post-vaccination for observation. The observation period will include an assessment of immediate solicited local and systemic AEs. Solicited local and systemic AEs occurring within 30 minutes post-vaccination will be recorded in the ~~subject~~ **electronic** diary **(in Period 1) by the subjects** and corresponding eCRF. All unsolicited AEs occurring within 30 minutes post-vaccination will be recorded in the subject source and AE eCRF. Any unusual signs or symptoms reported during the initial 30 minutes post-vaccination will result in continued close monitoring. Based on their condition, subjects may be asked to remain in the clinic for their safety for more than 30 minutes after vaccination (the reason will be recorded in the source documents). All data (including the assessment of solicited local and systemic AEs) will be recorded in the source documents during and after the observation period. Refer to Section 10.2.1 for details regarding the assessment of AEs and/or solicited local and systemic AEs;	Clarified the collection of solicited AEs in the electronic diary in period 1 only.
25.	Section 10.1.6 (Surveillance for COVID-19 Cases)	The first and third bullet points and the last paragraph have been updated as follows:<br>• Passive surveillance: subjects will be instructed to contact the study site **as soon as possible** ~~within 72 hours~~ in case they experience symptoms of COVID-19, as defined in Section 10.4.1;<br>• **Subjects will also be advised to inform the clinical site if they have tested positive for SARS-CoV-2 infection, with or without the presence of any symptoms associated with COVID-19, and will be asked to provide a documented test result. If/when the subject begins to experience symptoms associated with COVID-19, the process for the collection of symptoms and nasal or NP swabs (as noted below) will be followed;**<br>• If a subject reports any symptom(s) of COVID-19:<br>• Collect information regarding COVID-19, including all applicable symptoms and the onset date:	Clarified that subjects who test positive for SARS-CoV-2 infection should notify the clinical site.

#	Section (page #)	Change	Rationale
		• The onset date is defined as the first date when one or more of the symptoms occurred ~~and persisted for at least 12 hours~~;<br>In the event that a subject cannot be reached via telephone, he/she may be contacted by text message or via email (if these contacts are available). However, the telephone should be the initial and preferred means of communication. At least three attempts within allowed time window should be made in order to reach the subject.	
26.	Section 10.1.8.1 (Pre-vaccination)	The last two bullet points have been updated as follows:<br>• Assess the subject for contraindications to the second vaccination according to Section 11.3. If the subject has no contraindications to the second vaccination, proceed to register the visit in the **subject's eCRF** ~~interactive response technology (IRT) system~~;<br>• Register the visit in the **subject's eCRF** ~~IRT system~~. The IRT system will assign the same treatment assignment at Day 21 as Day 0. If the subject has a contraindication for the second vaccination, record the status **in the subject's eCRF** ~~according to the IRT manual~~.	Since subjects will be provided with electronic diaries, the phone contacts on Days 1, 8, 22, and 29 are no longer needed in the Phase 3 portion.
27.	Section 10.1.11 (Day 42)	The second sub-bullet of the fourth bullet point in this section has been removed:<br>~~• Immunogenicity (UK CMI [ELISpot and ICS]) assessment (only Period 1 of Phase 3 portion – UK-CMI subset);~~	The evaluation of cell-mediated immunity in Period 1 of the Phase 3 portion in a subset of subjects from the UK will no longer be performed.
28.	Section 10.1.14 (Day 386)	The following content has been added to this section:<br>**In the Phase 2 or Phase 3 portion, subjects who are withdrawn/discontinued from the study due to receiving an approved or authorized COVID-19 vaccine will be asked to complete the procedures for the final telephone contact (refer to Section 10.1.14.2) at the time of unblinding and prior to receipt of the approved**	Included details in the protocol on how subjects who request to be unblinded to

#	Section (page #)	Change	Rationale
		**or authorized COVID-19 vaccine, if the subject agrees. If the subject unblinding occurs prior to the Day 42 visit, then the subject will also be asked about any AEs and recorded appropriately. For subjects who receive the approved or authorized COVID-19 vaccine and then inform the Investigator, the final telephone contact will focus only on the period prior to receiving the approved or authorized COVID-19 vaccine and the subject may be informed retroactively about their treatment allocation.**<br>**For all other subjects who withdraw consent from the Phase 2 portion or Period 1 (all subjects) or Period 2 (immunogenicity subset) of the Phase 3 portion of the study, they will be asked to undergo Day 386 visit (refer to Section 10.1.14.1) procedures within two weeks of withdrawal, if the subject agrees.**<br>**For all other subjects in Period 2 (all subjects who are not included in the immunogenicity subset) of the Phase 3 portion of the study, they will be asked to participate in the final telephone contact (refer to Section 10.1.14.2) within two weeks of withdrawal, if the subject agrees.**	receive the approved or authorized COVID-19 vaccine will be unblinded, their status in the study, and how their data will be handled.
29.	Section 10.1.14.1 (Visit 8 for Phase 2 portion and Visit 5 for Phase 3 portion)	The following paragraph at the end of the section has been removed:<br>~~Any subject who withdraws consent from the study will be asked to undergo Day 386 visit procedures within two weeks of withdrawal, if the subject agrees.~~	This statement has been moved to Section 10.1.14.
30.	Section 10.1.14.2 (Telephone Contact for Phase 3 portion)	The following paragraph at the end of the section has been removed:<br>~~Any subject who withdraws consent from the study will be asked to participate in the final telephone contact within two weeks of withdrawal, if the subject agrees.~~	This statement has been moved to Section 10.1.14
31.	Section 11.4 (Removal of Subjects from Treatment or Assessment)	The third paragraph has been updated as follows:<br>Withdrawal subjects will be those who leave the Phase 2 portion of the study before Day 386 or the Phase 3 portion of the study before Day 386 of Period 2, for whatever reason; withdrawal subjects will not be replaced. Any subject who withdraws consent from the study will be asked to visit the clinical site (Phase 2 portion or Period 1 [all subjects] or Period 2 [immunogenicity subset] of the Phase 3 portion) or participate	Included details in the protocol on how subjects who request to be unblinded to receive the

#	Section (page #)	Change	Rationale
		in the final telephone contact (Period 2 [all subjects who are not included in the immunogenicity subset] of the Phase 3 portion) within two weeks of withdrawal, if the subject agrees. The procedures performed for the final assessment or telephone contact will comprise of those for the Day 386 visit/telephone contact (as applicable), if permitted by the subject. **In the Phase 2 or Phase 3 portion, subjects who are withdrawn from the study due to receiving an approved or authorized COVID-19 vaccine will be asked to complete the procedures for the final telephone contact at the time of unblinding and prior to receipt of the approved or authorized COVID-19 vaccine, if permitted by the subject.**<br>All withdrawal subjects must be reported to Medicago (and/or its designee). The reason for withdrawal should be documented in the subjects' records and in the appropriate section of the eCRF. The eCRF must be completed up to and including the time of the drop-out/final assessment.	approved or authorized COVID-19 vaccine will be unblinded, their status in the study, and how their data will be handled.
32.	11.4.1 (Follow-up of Discontinuations)	The content in this section has been updated as follows:<br>All subjects who receive a study vaccine will be followed for safety until the end of the Phase 2 or Phase 3 portion of the study, if permitted by the subject. **However, subject who receives an approved or authorized COVID-19 vaccine will be asked to complete the final telephone contact at the time of unblinding and prior to receipt of the approved or authorized COVID-19 vaccine, if permitted by the subject.**	Included details in the protocol on how subjects who request to be unblinded to receive the approved or authorized COVID-19 vaccine will be unblinded, their status in the study, and how their data will be handled.
33.	11.6 (Unblinding Due to Availability of Approved or	This new section has been added with the following content:<br>**As discussed in Section 6.2, some subjects may request to become unblinded in order to receive an approved or authorized vaccine for COVID-19 when it becomes publicly available to them in their region. If subjects meet the criteria**	Included details in the protocol on how subjects who request to be

<table>
<tr><th>#</th><th>Section (page #)</th><th>Change</th><th>Rationale</th></tr>
<tr><td></td><td>Authorized COVID-19 Vaccines)</td><td>

outlined in Section 6.2, they will be unblinded to the treatment they have received. The subject’s status in the study will be dependent on the treatment received and whether the subject actually receives the approved or authorized COVID-19 vaccine (refer to Figure 1).

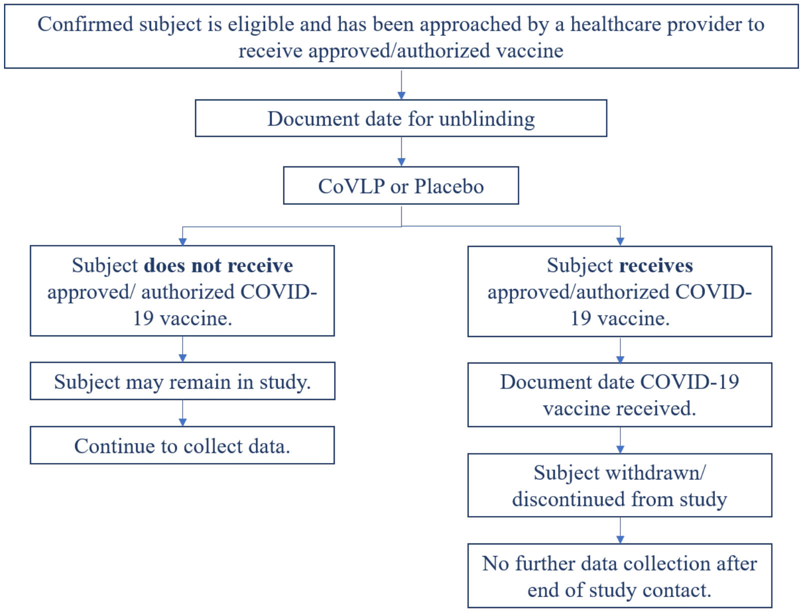

Figure 1 Decision Tree for Possible Unblinding in Phase 2 and Phase 3 Portions

If the subject received the CoVLP formulation and did not receive the approved or authorized COVID-19 vaccine, then the subject may remain in the study. If the subject received placebo or received the CoVLP formulation and the approved or authorized COVID-19 vaccine, then the subject will be withdrawn/discontinued from the study. Subjects will be asked to complete the final telephone contact within two weeks of withdrawal/discontinuation, if permitted by the subject, after they have received the approved or authorized COVID-19 vaccine. The date of when the approved or authorized COVID-19 vaccine was received by the subject must be documented in the EDC.

</td><td>unblinded to receive the approved or authorized COVID-19 vaccine will be unblinded, their status in the study, and how their data will be handled.</td></tr>
</table>

#	Section (page #)	Change	Rationale
		**In the Phase 3 portion, if a subject requests to be unblinded before the completion of their Day 21 visit (in Period 1) and does not receive the approved or authorized COVID-19 vaccine, then the unblinded subject will be eligible to receive their second study vaccination at the Day 21 visit (in Period 1). Subjects will still be assessed for any contraindications for the second study vaccinations (as per Section 11.3).**<br>**Subject who receives the approved or authorized COVID-19 vaccine will be asked to complete the final telephone contact at the time of unblinding and prior to receipt of the approved or authorized COVID-19 vaccine, if permitted by the subject.**	
34.	Section 12.1.1 (Safety Analysis Set)	The following content has been added to the end of the section:<br>**However, for subjects who become unblinded because they requested to be unblinded for the purposes of receiving an approved or authorized COVID-19 vaccine, their safety data will be handling according to Figure 4.**	Included details in the protocol on how subjects who request to be unblinded to receive the approved or authorized COVID-19 vaccine will be unblinded, their status in the study, and how their data will be handled.

#	Section (page #)	Change	Rationale
		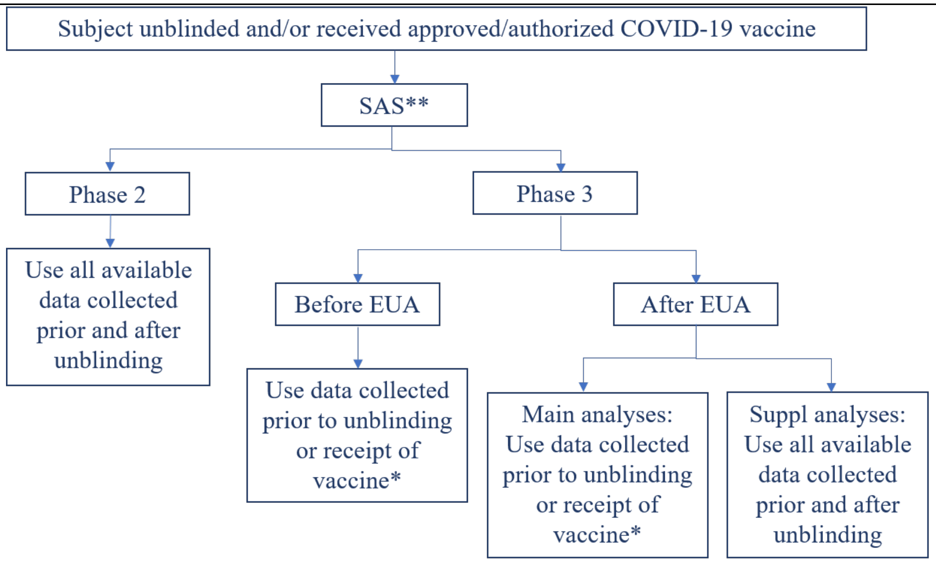<br>*whichever occurs first<br>**Subject who becomes unblinded or receives an approved or authorized COVID-19 vaccine will be included in the SAS however her/his data collected after unblinding or receipt of the vaccine, whichever comes first, will be excluded from some analyses, as noted in this flowchart.<br>**Figure 4 Decision Tree for Handling of Safety Data From Subjects Who Request to Become Unblinded to Receive an Approved or Authorized COVID-19 Vaccine in Phase 2 and Phase 3 Portions**<br>**In the Phase 2 portion, the safety data collected from subjects who become unblinded because they requested to be unblinded for the purposes of receiving an approved or authorized COVID-19 vaccine will be analyzed using all available data collected prior and after unblinding.**<br>**In the Phase 3 portion, the safety data collected from subjects who become unblinded because they requested to be unblinded for the purposes of receiving an approved or authorized COVID-19 vaccine will be analyzed in the following manner:**<br>• **Prior to Emergency Use Authorization:**	

#	Section (page #)	Change	Rationale
		• **Safety analyses performed using safety data collected prior to unblinding or receipt of an approved or authorized COVID-19 vaccine, which ever occurs first, from subjects in the SAS;**<br>• **After Emergency Use Authorization:**<br>• **Main safety analyses: safety analyses performed using safety data collected prior to unblinding or receipt of an approved or authorized COVID-19 vaccine, which ever occurs first, from subjects in the SAS;**<br>• **Supplementary safety analyses: safety analyses performed using safety data collected prior to and after unblinding from subjects in the SAS.**	
35.	12.1.3 (Per Protocol Set)	The content in this section has been updated as follows:<br>The PP set will consist of a subset of the ITT set who participated in the study until the end of the surveillance period in the Phase 2 or Phase 3 portions of the study; had no major protocol deviations related to subject eligibility, the ability to develop a valid immune response, prohibited medication use, or the efficacy analyses; and who received both doses of either the CoVLP formulation or placebo. Major protocol deviations will be identified and documented during a blinded data review prior to database lock and confirmed at the time of database lock. **The only exceptions for the PP set are the inclusion of subjects who become unblinded because they requested to be unblinded for the purposes of receiving an approved or authorized COVID-19 vaccine or subjects who received an approved or authorized COVID-19 vaccine (refer to Figure 5).**	Included details in the protocol on how subjects who request to be unblinded to receive the approved or authorized COVID-19 vaccine will be unblinded, their status in the study, and how their data will be handled.

#	Section (page #)	Change	Rationale
		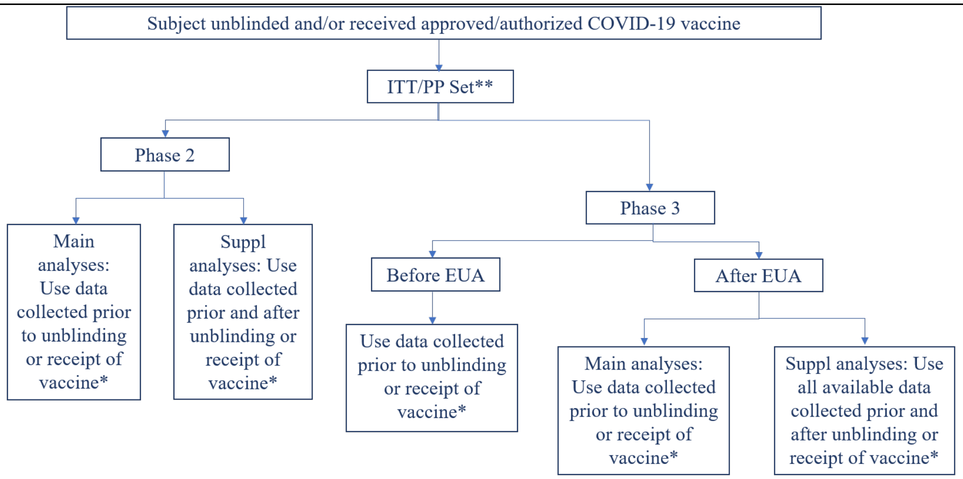<br><br>*whichever occurs first<br>**Subject who becomes unblinded or receives an approved or authorized COVID-19 vaccine will be included in the ITT/PP set however her/his data collected after unblinding or receipt of the vaccine, whichever comes first, will be excluded from some analyses, as noted in this flowchart.<br>**Figure 5 Decision Tree for Handling of Efficacy Data from Subjects Who Request to Become Unblinded to Receive an Approved or Authorized COVID-19 Vaccine in Phase 2 and Phase 3 Portions**<br>**For these particular subjects, the efficacy data collected will be analyzed in the following manner:**<br>**Phase 2 portion:**<br>• **Main efficacy analyses: efficacy analyses performed using efficacy data collected prior to unblinding or receipt of an approved or authorized COVID-19 vaccine, which ever occurs first, from subjects in the PP set and ITT set;**<br>• **Supplementary efficacy analyses: efficacy analyses performed using efficacy data collected prior to and after unblinding or receipt of an approved or authorized COVID-19 vaccine, which ever occurs first, from subjects in the PP set and ITT set.**	

#	Section (page #)	Change	Rationale
		**Phase 3 portion:**<br>• **Prior to Emergency Use Authorization:**<br>• **Efficacy analyses performed using efficacy data collected prior to unblinding or receipt of an approved or authorized COVID-19 vaccine, which ever occurs first, from subjects in the PP set and ITT set;**<br>• **After Emergency Use Authorization:**<br>• **Main efficacy analyses: efficacy analyses performed using efficacy data collected prior to unblinding or receipt of an approved or authorized COVID-19 vaccine, which ever occurs first, from subjects in the PP set and ITT set;**<br>• **Supplementary efficacy analyses: efficacy analyses performed using efficacy data collected prior to and after unblinding or receipt of an approved or authorized COVID-19 vaccine, which ever occurs first, from subjects in the PP set and ITT set.**<br>Subjects who received the wrong treatment, but for whom the treatment received can be unequivocally confirmed, will be analyzed as treated, provided they have no other deviations that compromise their data.<br>In Phase 2, the analyses of all efficacy endpoints will be performed using the PP set as primary analysis and the ITT set as a secondary analysis population.<br>In Phase 3, the analyses of all efficacy endpoints will be performed using the ITT set as primary analysis and the PP set as a secondary analysis population. **In the Phase 3 portion, the PP set and the ITT set will be defined separately for each period.**	
36.	12.1.4 (Immunogenicity Per Protocol Set)	The first and last paragraphs in this section have been updated as follows:<br>The IPP set will consist of a subset of the ITT set who participated in the immunogenicity portions of the Phase 2 or Phase 3 portion**s** of the study and completed the study with no major deviations related to subject eligibility, the ability to develop a valid immune response, prohibited medication use, or the	Included details in the protocol on how subjects who request to be unblinded to

#	Section (page #)	Change	Rationale
		immunogenicity analyses; and who received either the CoVLP formulation or placebo. **The only exceptions for the IPP set are the inclusion of subjects who become unblinded because they requested to be unblinded for the purposes of receiving an approved or authorized COVID-19 vaccine or subjects who received an approved or authorized COVID-19 vaccine (refer to Figure 6).**<br>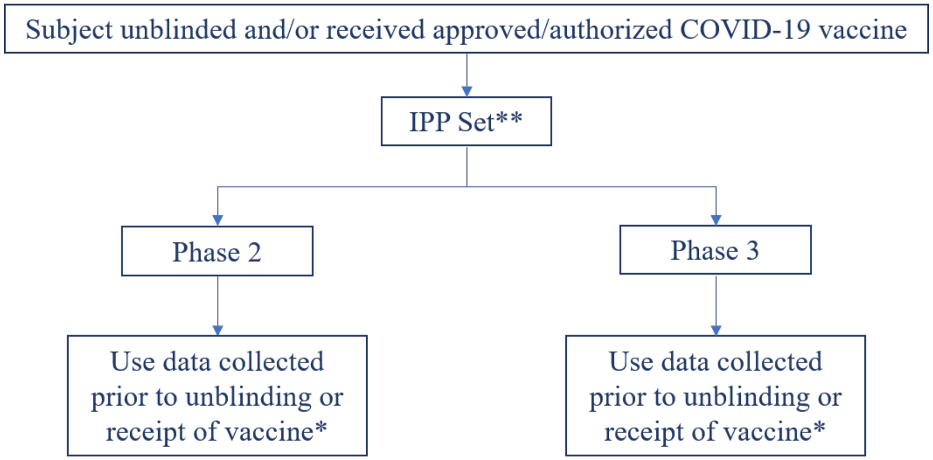<br><br>*whichever occurs first<br>**Subject who becomes unblinded or receives an approved or authorized COVID-19 vaccine will be included in the IPP set however her/his data collected after unblinding or receipt of the vaccine, whichever comes first, will be excluded from the analysis, as noted in this flowchart.<br>**Figure 6 Decision Tree for Handling of Immunogenicity Data from Subjects Who Request to Become Unblinded to Receive an Approved or Authorized COVID-19 Vaccine in Phase 2 and Phase 3 Portions**<br>**For these particular subjects, all data collected before the time of unblinding or receipt of an approved or authorized COVID-19 vaccine, which ever occurs first, will be included in the immunogenicity analyses.**<br>The analyses of all immunogenicity endpoints will be performed using the IPP set in both Phase 2 and Phase 3 portions, as the primary analysis population, and the	receive the approved or authorized COVID-19 vaccine will be unblinded, their status in the study, and how their data will be handled.

#	Section (page #)	Change	Rationale
		immunogenicity ITT set, as a secondary analysis population. **In the Phase 3 portion, the IPP set and the immunogenicity ITT set will be defined separately for each period.**	
37.	Section 12.3 (Day 21 and Day 42 Immunogenicity Data Analysis and Efficacy Analysis)	The following new paragraphs have been added after the third and fourth paragraphs as follows:<br>**The analyses performed up to Day 42 for available data for Study Populations #1, #2, and #3 will generate selected tables, listings, and figures (as applicable), by the unblinded statistical team. The results of these analyses will be used to prepare the clinical study report (CSR) and an addendum to the CSR (up to Day 42) for the purpose of reporting to regulatory agencies the safety and immune response profile of the CoVLP formulation up to 42 days after the second vaccination. The Phase 2 results not included in the Phase 2 Day 42 CSR and addendum will be presented in a subsequent addendum to the CSR. In addition, the results included the Phase 2 Day 42 CSR and addendum will be used for publication.**<br>**The final analysis of the efficacy data to test for vaccine efficacy will produce final efficacy outputs that will be used, in addition to safety outputs, to prepare an addendum to the CSR for the purposes of reporting to regulatory agencies the efficacy and safety profile of the CoVLP formulation. The Phase 3 results that are not included in this addendum to the CSR will be presented in a subsequent addendum to the CSR.**	Included details of the clinical study reports and addenda to generate for the Phase 2 and Phase 3 portions.
38.	Section 12.6.2 (Analysis of Secondary Endpoints)	The last paragraph in this section has been removed:<br>~~In addition, the CMI response induced on Day 0, Day 21, Day 42, Day 201, and Day 386 will be measured by the percentage of T cells expressing functional markers, using flow cytometry, for each treatment group. The responses will be compared between treatment groups and timepoints using appropriate non-parametric (Wilcoxon) models. Additional details will be provided in the SAP.~~	The assessment of the CMI (CD+4) response in Phase 3 has been moved from a secondary analysis to an exploratory analysis.

#	Section (page #)	Change	Rationale
39.	Section 12.6.3 (Analysis of Exploratory Endpoints)	A new paragraph has been added after the first paragraph as follows:<br>**For the Phase 3 portion, the CMI response induced on Day 0, Day 21, Day 42, Day 201, and Day 386 will be measured by the percentage of T cells expressing functional markers, using flow cytometry, for each treatment group. The responses will be compared between treatment groups and timepoints using appropriate non-parametric (Wilcoxon) models. Additional details will be provided in the SAP.**	The assessment of the CMI (CD+4) response in Phase 3 has been moved from a secondary analysis to an exploratory analysis.
40.	Section 12.7 (Efficacy Analyses)	The third paragraph has been updated as follows:<br>Efficacy summary tables will present data for the overall population and for each age stratum according to each Study Population, as applicable. **In addition, efficacy summary tables will also be presented by gender, race, ethnicity and sero-status at baseline.**	Clarified additional efficacy summary tables will be generated.
41.	Section 12.7.1 (Analysis of Primary Endpoint)	Two new paragraphs have been added after the third paragraph and the fourth paragraph has been updated as follows:<br>**For the ITT set analysis, the number of cases in the IRR calculation will include those occurring + 7 days after second vaccination per the definition of the primary endpoint. The follow-up time used in the calculation of person-years will be calculated from the day of randomization.**<br>**For the PP set analysis, the number of cases in the IRR calculation will also include those occurring + 7 days after second vaccination per the definition of the primary endpoint. The follow-up time used in the calculation of person-years will be calculated from the date of second vaccination + 7 days.**<br>**For both analysis sets,** ~~The follow-up time will be calculated from the date of second vaccination + 7 days and~~ censoring will be performed when subjects will experience their first COVID-19 case, the date the database will be ~~freeze~~ frozen for the primary analysis, the subject's cross-over occurs, **the date subject is unblinded,**	Clarified the efficacy analysis using the ITT set and PP set and incorporated details regarding the analysis of data for subjects who request to be unblinded to receive the approved or authorized COVID-19 vaccine.

#	Section (page #)	Change	Rationale
		**the date receives an approved or authorized COVID-19 vaccine,** or at the date of subject completion/withdrawal from the study, whichever occurs first.	
42.	Section 12.7.2 (Analysis of Secondary Endpoints)	A new paragraph has been added after the fourth paragraph and the fifth paragraph has been updated as follows:<br>**Similarly to the analysis of the primary efficacy endpoint, for the ITT set analysis, the number of cases included will depend on the endpoint, and the follow-up time will be calculated from the day of randomization through censoring time. For the PP set analysis the number of cases will be adjusted according to the endpoint as well as the follow-up time.**<br>VE will be evaluated for any strain as the incidence rate ratio of CoVLP formulation vaccinated versus ~~unvaccinated~~ subjects to develop laboratory-confirmed ~~(confirmed by the ELISA method for the N protein)~~ asymptomatic SARS-CoV-2 infection at Day 386 ~~(~~in Period 1~~) and at cross-over in 1) all~~ **in** subjects **who were seronegative at baseline (confirmed by the ELISA method for the N protein)** ~~and 2) in subjects who only received a single vaccination~~. Assuming the number of cases in each arm follows a Poisson distribution, then conditioning on the total number of cases and the ratio of person-time of follow up, exact 95 % CIs for the incidence rate ratio IRR, and hence for VE = 1-IRR, are obtained via binomial distribution calculations [Rothman 2008].	Clarified the efficacy analysis using the ITT set and PP set. Also, updated the efficacy analysis for asymptomatic SARS-CoV-2 infection.
43.	Section 14.1.3 (Preparation and Administration of Study Vaccine)	The third paragraph has been updated as follows:<br>During the Phase 2 portion and each period of the Phase 3 portion, the CoVLP formulation will be administered on study Day 0 and Day 21 as an IM injection of 0.5 mL, into the deltoid muscle. For subjects with BMI < 30 kg/m^2, a 23 gauge needle of at least one inch or 2.5 cm in length can be used for vaccination. For subjects ~~in Study Population #3~~ with BMI ≥ 30 kg/m^2, a needle of at least 1.5 inches or 3.5 cm in length must be used to ensure intramuscular delivery.	Clarified that the needle length requirements based no BMI are not specific to Study Population #3.

#	Section (page #)	Change	Rationale
44.	Section 19.7 (Appendix 7)	The table listing the meeting schedule for the IDMC reviews and the list of safety data to be reviewed has been updated with additional information for the Phase 2 portion of the study and the following text has been added prior to the table:<br>**The IDMC meetings and review of safety data for the Phase 2 and Phase 3 portions are outlined in Table 14 and Table 15, respectively. As each portion of the study progresses, the timing, content, and the number of meetings may change from that described in Table 14 and Table 15. The IDMC Charter should be referred to for the latest and up-to-date information regarding the IDMC meetings.**	The table presenting the Phase 2 information has been updated to include the latest information. A statement has been added prior to the table for clarification about where to find the most up-to-date information about the IDMC review meetings.

Table 5 Summary of Changes from Protocol Version 5.1 to Version 5.2 (Effective Date 23 March 2021)

#	Section (page #)	Change	Rationale
1.	Synopsis (Study Rationale)<br>AND<br>Section 1.5 (Overall Rationale for the Study)	The third paragraph has been updated as follows:<br>In each Study Population, 306 subjects will be randomized 5:1 to receive the CoVLP formulation:placebo. **The total number of subjects in each Study Population may vary depending on when sufficient data has been accumulated to make a decision on when each Study Population can procced to the Phase 3 portion. Unless full enrollment (i.e., enrolled up to 306 subjects) has already been achieved, further enrollment into each Study Population will be closed once sufficient data has been accumulated to make the decision to proceed to Phase 3.**	Provided details for closing further enrollment of subjects in the Phase 2 portion of the study. Full enrollment in Study Population

#	Section (page #)	Change	Rationale
		All subjects will be followed for a period of 12 months after the last vaccination for durability of the immune responses, safety, and efficacy.	#1 has been completed and further enrollment in Study Populations #2 and #3 is no longer needed since sufficient data has been accumulated for these two Study Populations to support proceeding into the Phase 3 portion of the study.
2.	Synopsis (Number of Planned Subjects)	The second paragraph has been updated as follows:<br>In the Phase 2 portion of the study, up to 306 subjects will be enrolled in a 5:1 ratio to receive CoVLP formulation:placebo in each Study Population (up to 918 subjects in total). **The total number of subjects in each Study Population may vary depending on when sufficient data has been accumulated to make a decision on when each Study Population can procced to the Phase 3 portion. Unless full enrollment (i.e., enrolled up to 306 subjects) has already been achieved, further enrollment into each Study Population will be closed once sufficient data has been accumulated to make the decision to proceed to Phase 3.**	
3.	Synopsis (Study Design)<br>AND<br>Section 4.1 (Overview of Study Design)	The second paragraph has been updated as follows:<br>In each of the three Study Populations, 306 subjects will be randomized 5:1 and will receive the same CoVLP formulation or placebo. **The total number of subjects in each Study Population may vary depending on when sufficient data has been accumulated to make a decision on when each Study Population can procced to the Phase 3 portion. Unless full enrollment (i.e., enrolled up to 306 subjects) has already been achieved, further enrollment into each Study Population will be closed once sufficient data has been accumulated to make the decision to proceed to Phase 3.** Subjects will receive two IM injections 21 days apart on Days 0 and 21, into the deltoid region of the alternating (if possible) arm (i.e., each arm will be injected once), of one of the following:<br>• CoVLP formulation;<br>• Placebo.	
4.	Table 2 (Time and Events Schedule: General	A typo in the following statement has been corrected as follows:<br>During the first 8 days after each vaccination in Period ~~2~~**1**, if data entry in the electronic diary is missed for 2 consecutive days by a subject then the subject will	Typo corrected.

#	Section (page #)	Change	Rationale
	Information for the Phase 3 Portion – Footnote 21)<br>AND<br>Section 17.3.1 (Diary and Memory Aid)	receive a follow-up contact (phone, text, or email) to remind him/her to record his/her data in the electronic diary.	

Table 6 Summary of Changes from Protocol Version 5.0 to Version 5.1 (Effective Date 11 March 2021)

#	Section (page #)	Change	Rationale
1.	Section 14.1.1 (Identity of Investigational Product)	The first paragraph has been updated as follows:<br>The CoVLP vaccine is a sterile transparent to opalescent, colorless to yellowish ~~or greenish~~ liquid suspension **that may contain a small number of visible white particulates**. The CoVLP vaccine will be supplied in 0.35 mL single dose vials in the Phase 2 portion and in 3.1 ml multidose vials (10 doses/vial) in the Phase 3 portion. The S glycoprotein concentration in the vaccine will be 15 µg/mL for a final dosage of 3.75 µg.	Provided an updated description of the appearance of the investigational product.

Table 7 Summary of Changes from Protocol Version 4.0 to Version 5.0 (Effective Date 06 February 2021)

#	Section (page #)	Change	Rationale
1.	Synopsis (Study Rationale)<br>AND<br>Section 1.5 (Overall	The content in this section has been updated as follows:<br>This Phase 2/3 study is a multi-portion design to confirm that the chosen formulation and dosing regimen of CoVLP (i.e. two doses of 3.75 µg CoVLP adjuvanted with AS03 [hereafter referred to as "CoVLP formulation"] given 21 days apart) has an acceptable immunogenicity and safety profile in adults 18 years of age or older, including those with significant comorbidities (Phase 2 portion). The efficacy of this	For Study Population #3, it has been clarified that subjects may be enrolled from countries where authorization to

<table>
<tr><th>#</th><th>Section (page #)</th><th>Change</th><th>Rationale</th></tr>
<tr><td></td><td>Rationale for the Study)</td><td>
CoVLP formulation in these same Study Populations will be assessed in the Phase 3 portion.

The Phase 2 portion is a randomized, observer-blinded, placebo-controlled study to evaluate the CoVLP formulation, compared to placebo, in subjects from the following three Study Populations:
<table>
<tr><td>Study Population</td><td>Age and/or Health Category</td><td>Age Range (years)</td></tr>
<tr><td>1</td><td>Healthy adults</td><td>18-64</td></tr>
<tr><td>2</td><td>Healthy elderly adults*</td><td>≥ 65</td></tr>
<tr><td>3</td><td>Adults with significant comorbidities*</td><td>≥ 18</td></tr>
</table>
*Subjects to be enrolled in **country(ies) that has/have provided authorization to enroll these subjects in the study** ~~the US only~~.

In each Study Population, 306 subjects will be randomized 5:1 to receive the CoVLP formulation:placebo. All subjects will be followed for a period of 12 months after the last vaccination for durability of the immune responses, safety, and efficacy.

The Phase 3 portion is an event-driven, randomized, observer-blinded, placebo-controlled**, 2-way cross-over** design that will evaluate the efficacy and safety of the CoVLP formulation, compared to placebo, in the same Study Populations #1 - #3 presented above. Up to 30 000 subjects will be enrolled in a 1:1 ratio to receive the CoVLP formulation **or** ~~:~~placebo**, according to the following treatment sequences:**~~.~~
<table>
<tr><th>Treatment Sequence</th><th>Period 1</th><th>Period 2</th></tr>
<tr><td>**1**</td><td>**CoVLP formulation**</td><td>**Placebo**</td></tr>
<tr><td>**2**</td><td>**Placebo**</td><td>**CoVLP formulation**</td></tr>
</table>
~~Immunogenicity will only be evaluated in a subset of 288 subjects.~~
</td><td>enroll such subjects has been received, rather than just specifying the US. In addition, the cross-over design has been incorporated into the Phase 3 portion of the study as a method to provide subjects who receive the placebo to receive the CoVLP formulation if/when emergency use authorization is granted. Also, a subset of subjects from the UK will be included in the evaluation of cell-mediated immunity in Period 1 of the Phase 3 portion.</td></tr>
</table>

#	Section (page #)	Change	Rationale
		**In the 2-way cross-over design, subjects who receive the CoVLP formulation in Period 1 will receive the placebo in Period 2, while subjects who receive the placebo in Period 1 will receive the CoVLP formulation in Period 2. Initiation of the cross-over design for subjects enrolled in the study will be dependent on the following overall study and subject-specific criteria:**<br>1. **When Emergency Use Authorization or Approval for the CoVLP formulation is granted by any Regulatory Agency(ies); and**<br>2. **When one or more authorized or approved COVID-19 vaccines become available for the subject in his/her country of recruitment; and**<br>3. **The subject has completed a minimum of 60 days in Period 1.**<br>**Emergency use authorization or approval will be pursued after the success criterion has been met for the primary efficacy endpoint of the Phase 3 portion will be evaluated. The primary efficacy endpoint will be evaluated o**~~O~~nce ~~approximately~~ **at least** 160 **laboratory-confirmed** COVID-19 cases have been accumulated~~, the primary efficacy endpoint of the Phase 3 portion will be evaluated~~.<br>All subjects will be followed for efficacy and safety **until** ~~for a period of~~ **until** 12 months after the last vaccination **after the cross-over**. **The sero-status of all subjects will be assessed at the time of enrollment and at the time of the cross-over. Serum samples will be obtained from all subjects at 21 days after the second CoVLP vaccination so that a possible serologic correlate of protection can be evaluated once the primary efficacy outcome has been met and at later timepoints in the study. Only the 288 subjects (96 subjects from each Study Population) in the immunogenicity subset will be assessed for the p**~~P~~ersistence of the vaccine-induced **humoral and cellular** immune responses **until** ~~up to~~ 12 months after the last **study** vaccination~~ will be assessed in a subset of subjects~~.<br>**In addition, a subset of 400 subject from the United Kingdom (UK; UK-CMI subset) will be included in the exploratory evaluation of cell-mediated immunity (CMI) in Period 1 of the Phase 3 portion of the study.**	

#	Section (page #)	Change	Rationale
2.	Synopsis (Planned Study Period)<br>AND<br>Section 4.2.5 (Study Duration)	The content in this section has been updated as follows:<br>The planned duration of the Phase 2 portion is approximately 14 to 16 months**, from screening procedures up to the end of the Phase 2 portion.** ~~and t~~The Phase 3 portion **will consist of two periods, Period 1 and Period 2, and the planned duration** is **up to** approximately **26**~~14~~ months, from screening procedures up to the end of **Period 2 in the Phase 3 portion**. ~~each portion.~~	Clarified the duration of each portion of the study and incorporated the details of the cross-over design in regards to duration.
3.	Synopsis (Primary Objectives – Phase 2 Portion and Phase 3 Portion)<br>AND<br>Section 2.1 (Primary Objectives)	The primary efficacy objective of the Phase 3 portion has been updated as follows:<br>Efficacy:<br>68. To evaluate the efficacy of the CoVLP formulation, compared to placebo, in the prevention of laboratory-confirmed symptomatic SARS-CoV-2 infection (virologic method) starting 7 days after the second vaccination **in Period 1 and prior to the cross-over**.	Clarified the efficacy objective to take into account the cross-over design.
4.	Synopsis (Secondary Objectives – Phase 2 Portion and Phase 3 Portion)<br>AND<br>Section 2.2 (Secondary Objectives)	The secondary efficacy and immunogenicity objective of the Phase 3 portion have been updated as follows:<br>Efficacy:<br>69. To evaluate the efficacy of the CoVLP formulation, compared to placebo, in the prevention of laboratory-confirmed asymptomatic SARS-CoV-2 infection (serologic method) ~~at Day 201 and Day 386~~ **starting 7 days after the second vaccination in Period 1 and prior to the cross-over**;<br>70. To evaluate the efficacy of the CoVLP formulation, compared to placebo, in the prevention of severe COVID-19 disease starting 7 days after the second vaccination **in Period 1 and prior to the cross-over** (see definition of severe COVID-19 disease above);<br>71. To assess COVID-19-related symptoms in **virologically-confirmed cases**, and the severity of these symptoms ~~as well as viral shedding,~~ up until resolution in subjects administered the CoVLP formulation **in Period 1** compared to subjects	Clarified the efficacy and immunogenicity objectives to take into account the cross-over design.

#	Section (page #)	Change	Rationale
		administered the placebo **and compared to subjects administered CoVLP at the time of cross-over in Period 2**;<br>72. To evaluate the efficacy of the CoVLP formulation, compared to placebo, in the prevention of laboratory-confirmed symptomatic SARS-CoV-2 infection (virologic method) starting after the first vaccination **in Period 1 and prior to the cross-over**;<br>73. To evaluate the efficacy of the CoVLP formulation, compared to placebo, in the prevention of laboratory-confirmed symptomatic SARS-CoV-2 infection (virologic method) after the first vaccination and prior to the second vaccination **in Period 1, and prior to the cross-over**;<br>74. To evaluate the efficacy of the CoVLP formulation, compared to placebo, in the prevention of laboratory-confirmed symptomatic SARS-CoV-2 infection (virologic method) after the second vaccination and prior to 7 days post-second vaccination **in Period 1, and prior to the cross-over**;<br>75. To evaluate the efficacy of the CoVLP formulation, in subjects who only received a single dose compared to placebo, in the prevention of laboratory-confirmed asymptomatic SARS-CoV-2 infection (virologic and serologic methods) at ~~Day 201 and Day 386~~ **starting 7 days after the vaccination in Period 1 and prior to the cross-over**;<br>76. To assess the duration and intensity of viral shedding after **virologically-confirmed** SARS-COV-2 infection in subjects administered the CoVLP formulation compared to subjects administered the placebo **in Period 1 and prior to the cross-over**;<br>**77. To evaluate the efficacy of the CoVLP formulation, compared to placebo, in the prevention of laboratory-confirmed symptomatic SARS-CoV-2 infection (virologic method) starting 7 days after the second vaccination in Period 1 by strain and prior to the cross-over;**<br>Immunogenicity:	

#	Section (page #)	Change	Rationale
		78. To assess the immunogenicity of the CoVLP formulation, compared to placebo, in ~~a~~ **the immunogenicity** subset ~~of subjects in each of the Study Populations~~ **prior to and post-cross-over**, as determined by:<br>79. Neutralizing antibody titers induced by the vaccine against the SARS-CoV-2 virus at Day 0 (prior to vaccination; all subjects), Day 21, Day 42 (all subjects), Day 201 and Day 386;<br>80. IgG (ELISA) titers induced by the vaccine against the SARS-CoV-2 virus up to 21 days after the second vaccination and at Day 201 and Day 386;<br>81. The ratio of neutralizing antibody titers:IgG (ELISA) antibody titers at Day 21, Day 42, Day 201 and Day 386;<br>82. IFN-γ ELISpot response induced by the vaccine against the SARS-CoV-2 virus up to 21 days after the second vaccination and at Day 201 and Day 386;<br>83. IL-4 ELISpot response induced by the vaccine against the SARS-CoV-2 virus up to 21 days after the second vaccination and at Day 201 and Day 386;<br>84. Specific cell-mediated immune (CMI) response induced by the vaccine against the SARS-CoV-2 virus up to 21 days after the second vaccination and at Day 201 and Day 386, as measured by the percentage of CD4+ T cells expressing functional markers;	
5.	Synopsis (Exploratory Objectives – Phase 2 Portion and Phase 3 Portion)<br>AND<br>Section 2.3 (Exploratory Objectives)	The exploratory efficacy objectives of the Phase 2 and Phase 3 portions have been updated as follows:<br>Phase 2 portion<br>Efficacy:<br>85. To assess COVID-19-related symptoms **in subjects with virologically-confirmed disease**, and the severity of these symptoms, up until resolution in subjects administered the CoVLP formulation compared to subjects administered the placebo;<br>Phase 3 portion<br>Efficacy:	Clarified the efficacy objective to take into account the cross-over design and specify subjects with virologically-confirmed disease.

#	Section (page #)	Change	Rationale
		86. To evaluate the duration of protection (vaccine efficacy **versus virologically-confirmed symptomatic disease as well as serologically-confirmed asymptomatic disease**) of the CoVLP formulation **in subjects initially randomized to the CoVLP group** compared to **the original** placebo **group for any strain and by strain after the cross-over**;<br>**87. To evaluate the efficacy of the CoVLP formulation, compared to placebo, in the prevention of severe COVID-19 disease by strain starting 7 days after the second vaccination in Period 1 and prior to the cross-over (see definition of severe COVID-19 disease above);**	
6.	Synopsis (Number of Planned Subjects)	A new paragraph has been added to the end of this section:<br>**In addition, a subset of approximately 400 subjects from the UK will be included in an exploratory evaluation of cell-mediated immunity. These subjects will be enrolled from selected sites in the UK that will be defined prior to the start of the Phase 3 portion in this country.**	A subset of subjects from the UK will be included in the evaluation of cell-mediated immunity in Period 1 of the Phase 3 portion.
7.	Synopsis (Sample Size Determination)<br>AND<br>Section 12.2 (Sample Size Determination)	The content in this section has been updated as follows:<br>Phase 3 portion:<br>The sample size of up to 30 000 subjects has been calculated using an exact conditional Poisson approach based on the following assumptions:<br>88. A 1:1 ratio for CoVLP formulation:placebo;<br>89. At least 90% statistical power to reject the null hypothesis of 1) a lower limit of the 95 % confidence interval (CI) for a vaccine efficacy of the CoVLP formulation compared to placebo ≤ 30% and 2) true efficacy < 50%, assuming a vaccine efficacy of 60%;<br>~~90. At least 90 % statistical power to obtain an observed vaccine efficacy of the CoVLP formulation, compared to placebo, of at least 50 % with an appropriately alpha-adjusted 95 % confidence interval lower limit greater than 30 %;~~	Updated the Phase 3 sample size calculation to take into account the addition of the cross-over design and removal of the interim efficacy analysis. Also, a subset of subjects from the UK will be included in the evaluation of cell-

#	Section (page #)	Change	Rationale
		91. A SARS-CoV-2 attack rate (i.e. symptomatic disease) in unvaccinated subjects of 0.8 % across all study populations;<br>**92.** An attrition of 5 %**.** ~~;~~<br>~~93. An interim analysis for an early efficacy assessment will be performed when 80 laboratory-confirmed symptomatic COVID-19 cases have been collected in the Phase 3 portion;~~<br>~~94. A statistical correction for the interim analysis will be applied that uses the O'Brien-Fleming alpha-spending function. With interim and final assessments at 80 and 160 cases, for example, the two-sided test p-value thresholds will be 0.0027 and 0.0478, respectively.~~<br>With 15 000 subjects receiving the CoVLP formulation, there will be at least 95 % probability of observing an adverse event that occurs as frequently as 1 in 5000 vaccinees.<br>An immunogenicity subset sample size of 288 subjects is deemed sufficient to provide meaningful statistical assessment of immunologic outcomes.<br>**A UK-CMI subset sample size of 400 subjects is deemed sufficient to provide meaningful statistical assessment of immunologic outcomes.**	mediated immunity in Period 1 of the Phase 3 portion.
8.	Synopsis (Dosage and Administration)<br>AND<br>Section 7 (Dosage and Administration)	The content in this section has been updated as follows:<br>The CoVLP formulation for the Phase 2 and Phase 3 portions of the study has been selected based on the Phase 1 study data, including acceptable safety and immunogenicity profiles.<br>**<u>Phase 2 portion</u>:**<br>Subjects will receive two intramuscular (IM) injections 21 days apart on Days 0 and 21, into the deltoid region of the alternating (if possible) arm (**i.e.,** each arm will be injected once), of one of the following:<br>95. CoVLP formulation;<br>96. Placebo.<br>The use of the same dose of CoVLP for all Study Populations may not be adequate. Subjects in Study Population #1 (healthy adults 18 to 64 years of age) will receive the	Separated the information for Phase 2 and Phase 3 portions and incorporated details of the cross-over design in regards to dosing.

<table>
<tr><th>#</th><th>Section (page #)</th><th>Change</th><th>Rationale</th></tr>
<tr><td></td><td></td><td>
standard dose of CoVLP (3.75 µg) adjuvanted with AS03 in an injection volume of 0.5 mL.
Study Populations #2 and #3 (elderly adults or adults with significant comorbidities, respectively) will receive the standard dose of CoVLP (3.75 µg) adjuvanted with AS03 in an injection volume of 0.5 mL. ~~In Phase 2, i~~**I**f an acceptable immune response is not achieved at either Day 21 or Day 42 in one or both populations, the IDMC may recommend a dose escalation to 7.5 µg CoVLP adjuvanted with AS03 in a volume of 0.5 mL, and the same number of subjects (i.e. 306 subjects per Study Population) in the particular Study Population will be recruited, using the same enrollment approach (i.e. use of sentinel subjects and IDMC reviews). If the higher dose of the CoVLP formulation achieves an acceptable immune response in the Phase 2 portion, then the same higher dose will be used in that particular Study Population during the Phase 3 portion of the study.
<u>Phase 3 portion</u>:
Each Study Population will receive the same dose of CoVLP adjuvanted with AS03 and injection volume established for that particular Study Population during the Phase 2 portion of the study.
In each period, subjects will receive two IM injections 21 days apart on Days 0 and 21, into the deltoid region of the alternating (if possible) arm (i.e., each arm will be injected once), of either the CoVLP formulation or the placebo, according to the following treatment sequences:
<table>
<tr><th>Treatment Sequence</th><th>Period 1</th><th>Period 2</th></tr>
<tr><td>1</td><td>CoVLP formulation</td><td>Placebo</td></tr>
<tr><td>2</td><td>Placebo</td><td>CoVLP formulation</td></tr>
</table>
</td><td></td></tr>
</table>

<table>
<tr><th>#</th><th>Section (page #)</th><th>Change</th><th>Rationale</th></tr>
<tr><td></td><td></td><td>Subjects who receive the CoVLP formulation in Period 1 will receive the placebo in Period 2, while subjects who receive the placebo in Period 1 will receive the CoVLP formulation in Period 2.</td><td></td></tr>
<tr><td>9.</td><td>Synopsis (Study Design)
AND
Section 4.1 (Overview of Study Design)</td><td>The table under the Phase 2 portion sub-header has been updated as follows:
The Phase 2 portion is a randomized, observer-blinded, placebo-controlled, design with male and female subjects. Subjects will be enrolled from three Study Populations:
<table>
<tr><td>Study Population</td><td>Age and/or Health Category</td><td>Age Range (years)</td></tr>
<tr><td>1</td><td>Healthy adults</td><td>18-64</td></tr>
<tr><td>2</td><td>Healthy elderly adults*</td><td>≥ 65</td></tr>
<tr><td>3</td><td>Adults with significant comorbidities*</td><td>≥ 18</td></tr>
</table>
*Subjects to be enrolled in country(ies) that has/have provided authorization to enroll these subjects in the study ~~the US only~~.</td><td>For Study Population #3, it has been clarified that subjects may be enrolled from countries where authorization to enroll such subjects has been received, rather than just specifying the US. A consistent footnote has been used for the same table.</td></tr>
<tr><td>10.</td><td>Synopsis (Study Design)
AND
Section 4.1 (Overview of Study Design)</td><td>The table and the first, third, fourth, fifth, and sixth paragraphs under the Phase 3 portion sub-header have been updated as follows:
The Phase 3 portion is a randomized, observer-blinded, placebo-controlled, efficacy, safety, immunogenicity (in a subset of subjects), multicenter, 2-way cross-over design with male and female subjects. Similar to the Phase 2 portion, subjects will be enrolled from three Study Populations:
<table>
<tr><th>Study Population</th><th>Age and/or Health Category</th><th>Age Range (years)</th></tr>
<tr><td>1</td><td>Healthy adults</td><td>18-64</td></tr>
<tr><td>2</td><td>Healthy elderly adults*</td><td>≥ 65</td></tr>
<tr><td>3</td><td>Adults with significant comorbidities*</td><td>≥ 18</td></tr>
</table></td><td>For Study Population #3, it has been clarified that subjects may be enrolled from countries where authorization to enroll such subjects has been received, rather than just specifying the US.</td></tr>
</table>

<table>
<tr><th>#</th><th>Section (page #)</th><th>Change</th><th>Rationale</th></tr>
<tr><td></td><td></td><td>
*Subjects to be enrolled in country(ies) that has/have provided authorization to enroll these subjects in the study <s>the US only</s>.

Study Population #2 will be roughly stratified by age in the same manner. Enrollment of subjects in Study Population #3 will include adults with co-existing conditions such as, but not limited to, obesity (moderate or greater), cardiovascular diseases, hyperlipidemia, chronic kidney disease or related disorders, chronic respiratory disease, pre-diabetes and diabetes (type 1 and type 2), and treatment-controlled HIV.

The overall goal of the Phase 3 portion of this study is to enroll approximately 30 000 subjects who are generally representative of the overall target population in terms of racial diversity with enrichment for those at highest risk for serious COVID-19 disease (i.e. the elderly and those with significant comorbidities).

In each Study Population, subjects will be randomized to receive either the CoVLP formulation or the placebo, according to the following treatment sequences: <s>into one of two treatment groups:</s>

• <s>CoVLP formulation;</s>
• <s>Placebo.</s>
<table>
<tr><th>Treatment Sequence</th><th>Period 1</th><th>Period 2</th></tr>
<tr><td>1</td><td>CoVLP formulation</td><td>Placebo</td></tr>
<tr><td>2</td><td>Placebo</td><td>CoVLP formulation</td></tr>
</table>
Subjects who receive the CoVLP formulation in Period 1 will receive the placebo in Period 2, while subjects who receive the placebo in Period 1 will receive the CoVLP formulation in Period 2.

Subjects will be screened and will demonstrate a satisfactory baseline medical assessment by history and general physical examination prior to the start of Period 1. Prior to starting Period 2, subjects will be assessed to ensure they do not meet any contraindications to the first vaccination in Period 2. The study visits and contacts described below are planned for both Period 1 and Period 2. During
</td><td>In addition, incorporation of details of the cross-over design in regards to study design.</td></tr>
</table>

#	Section (page #)	Change	Rationale
		**Period 1, subjects may not complete all study contacts prior to cross-over since cross-over for a subject can be initiated as early as Day 60; even after the cross-over has been initiated, subjects will still return for the Day 201 and Day 386 visits (which will be aligned with post-cross-over visits that are within close proximity of the planned date). During Period 2, study visits and contacts will be performed as planned (i.e., Day 21, Day 42, Day 201, and Day 386 post-cross-over).**<br><br>On Day 0 and Day 21, vaccine administration will occur. ~~Phone contacts will be made one day and eight days after each vaccine administration, specifically for review of the subject's~~ **Subjects will be provided with a diary and memory aid to collect** safety and concomitant medication data. A visit to the Investigator site will occur on Day 21 (immunogenicity subset only) and Day 42 (all subjects) for immunogenicity assessments. Subjects will return to the Investigator site on Day 201 and Day 386 (6-month and 12-month safety follow-ups and immunogenicity assessments [immunogenicity subset only] after the last vaccine administration).<br><br>Active and passive surveillance will be conducted in the study from the day of the first vaccination (Day 0, post-vaccination) until ~~386 days~~ **the end of the study**. For passive surveillance, subjects will be instructed to report symptoms meeting the definition of new COVID-19 disease. For active surveillance, subjects will be contacted once per week via the subject's preferred method (e.g. phone, text, email, electronic diary), and asked about symptoms of COVID-19 disease. In addition, to monitor asymptomatic infection, subjects will perform a test for SARS-CoV-2 N antibodies on Day 0, Day ~~201,~~ **386 (in Period 1),** and ~~Day 386~~ **at cross-over**.	
11.	Synopsis (Safety Evaluations)<br>AND<br>Section 10.2.1 (Safety Evaluations)	A sentence has been added to the end of the section as follows:<br>**In the Phase 3 portion after the cross-over, the same safety endpoints will be collected (including immediate AEs) with one exception: solicited local and systemic AEs (up to seven days after each vaccination) will not be collected.**	Clarified that post-cross-over, solicited AEs will not be collected after each vaccination. Subjects will still

#	Section (page #)	Change	Rationale
			be expected to report all AEs.
12.	Synopsis (Immunogenicity Evaluations)<br>AND<br>Section 10.3.1 (Immunogenicity Evaluations)	The content in this section has been updated as follows:<br>Immunogenicity will be evaluated in all subjects in the Phase 2 portion and in a subset of 288 subjects in the Phase 3 portion of the study across the Study Populations with age distribution similar to the Phase 2 portion. Immunologic outcomes will include the humoral immune response (~~neutralization~~ **Nab** assay and ~~IgG~~ ELISA) and the CMI response induced in subjects on Day 0, Day 21, Day 42, Day 128 (~~neutralization~~ **Nab** assay and ~~IgG~~ ELISA only for Phase 2 portion only), Day 201, and Day 386.<br>**In addition, during Period 1 of the Phase 3 portion, cell-mediated immunity will be evaluated in the UK-CMI subset of 400 subjects on Day 0 and Day 42 using standardized methods for ELISpot and flow cytometry (ICS).**	Simplified the text by using consistent terminology. Also, a subset of subjects from the UK will be included in the evaluation of cell-mediated immunity in Period 1 of the Phase 3 portion.
13.	Synopsis (Efficacy Evaluations)<br>AND<br>Section 10.4.1 (Efficacy Evaluations)	The content in this section has been updated as follows:<br>Following the first vaccination, subjects will be asked to report symptoms associated with COVID-19 from the day of the first vaccination (Day 0, post vaccination) until the end of the Phase 2 portion or the Phase 3 portion of the study (through active or passive surveillance). COVID-19 will be defined as the presence of a laboratory-confirmed (virologic method) SARS-CoV-2 infection with the occurrence of a new onset of one or more of the following symptoms that persist(s) for or reoccur(s) after a period of at least 12 hours:<br>97. Fever or chills;<br>98. Cough;<br>99. Shortness of breath or difficulty breathing;<br>100. Fatigue;<br>101. Muscle or body aches;<br>102. Headache;	Clarified the process of collecting swabs in subjects who have reported symptoms associated with COVID-19 and how the collected swabs will be tested (centrally vs locally).

#	Section (page #)	Change	Rationale
		103. New loss of taste or smell;<br>104. Sore throat;<br>105. Congestion or runny nose;<br>106. Nausea or vomiting;<br>**107.** Diarrhea.<br><br>Subjects who report any symptoms associated with COVID-19 after the start of active and passive surveillance will be requested to visit the Investigator site to provide nasal or nasopharyngeal (NP) swabs ~~(2 per subject per event)~~ to test for SARS-CoV-2 infection. During the visit, the Investigator will confirm whether nasal or NP swabs should be collected. If the subject cannot visit the Investigator site, then the subject may receive a visit at home **(or hospital, if applicable)** to collect the nasal/NP swabs samples or be provided with self-administered nasal swabs for sample collection. **During the 7 days following each vaccination, Investigator judgement will be used to determine whether swabs should be collected from subjects reporting symptoms similar to those associated with COVID-19 since such symptoms may also be due to the reactogenicity of the study treatment administered rather than SARS-CoV-2 infection.**<br><br>The swabs will be collected within 72 hours (preferably within 48 hours) of the reporting of a COVID-19-associated symptom. If swabs cannot be collected within 72 hours after reporting of the COVID-19-associated symptom, nasal or NP swabs should be collected at the next available opportunity for the subject to visit the Investigator site **or at home (or hospital, if applicable)**.<br><br>~~One of the~~ **Two** swabs **per event per subject** will be **collected and** submitted for analysis using a virologic method**, at the central virology laboratory** ~~while the another swab will be kept as a back-up~~. **To mitigate the risk of long delays in shipping samples to the central virology laboratory, an additional local virologic test will be performed. The purpose of this supplementary test is to provide a**	

#	Section (page #)	Change	Rationale
		**preliminary diagnosis and permit appropriate symptom-recording and sample collection as quickly as possible.**<br><br>All subjects with virologically-confirmed SARS-COV-2 infection**, based on results from the locally performed testing,** will be asked to keep a detailed daily record of their symptoms and provide serial (every other day) self-administered nasal swabs ~~for testing, using a virologic method, until symptoms resolution or until two consecutive negative results are obtained.~~ **(2 swabs every other day for the central virology laboratory) for approximately 2 weeks. In the event, the COVID-19-associated symptoms persist for more than 2 weeks, then the Investigator's clinical judgement is required to extend the collection of swabs beyond the 2 weeks from the subject. If the COVID-19-associated symptoms have resolved in less than 2 weeks and the Investigator believes it is likely the subject has virologically recovered, then two consecutive negative results, obtained locally, are required prior to stopping the collection of the self-administered swabs for the purpose of testing at the central laboratory.**<br>**The swab sample results from the central virology laboratory will be considered the primary results for vaccine efficacy evaluation. For subjects who are hospitalized with severe COVID-19 and cannot provide samples for testing at the central virology laboratory, SARS-CoV-2 testing results from the hospital may be considered for use in the evaluation of vaccine efficacy if documentation of the local testing procedures and results is available.**<br>~~To mitigate the risk of long delays in shipping samples to the central virology laboratory in the Netherlands, an additional rapid turn-around virologic test will be performed 'locally' using an approved NP, nasal or saliva assay (i.e., local laboratory or point-of-care, nucleic acid or antigen-detection). The purpose of this supplementary assay is to provide a preliminary diagnosis and permit appropriate symptom-recording and sample collection as quickly as possible.~~	
14.	Synopsis (Primary Endpoints - Efficacy)	The following Efficacy primary endpoint has been updated as follows:<br>In the Phase 3 portion, the primary endpoint is:	Clarified the efficacy endpoint to take into

#	Section (page #)	Change	Rationale
	AND<br>Section 10.4.2.1 (Primary Endpoint)	• First occurrence, in a subject, of laboratory-confirmed (virologic method) symptomatic SARS-CoV-2 infection (≥ 7 days post-second vaccination) **in Period 1 and prior to cross-over**.	account the cross-over design.
15.	Synopsis (Secondary Endpoints – Safety)<br>AND<br>Section 10.2.3.2 (Secondary Endpoints)	The following Safety secondary endpoints have been updated as follows:<br>Phase 2 portion<br>• Relative occurrence of solicited local and systemic AEs by intensity grades for seven days following each vaccine administration ~~between~~ **for** the **combination of** healthy adults (Study Population #1) and ~~the~~ healthy elderly adults (Study Population #2) ~~combined~~ **compared to adults** and **the elderly** adults with significant comorbidities (Study Population #3);<br><br>Phase 3 portion<br>• Occurrence, intensity, and relationship to vaccination of immediate AEs (30 minutes after each ~~vaccination);~~ **vaccine administration) for both Period 1 and Period 2 vaccinations;**<br>• Occurrence and intensity of solicited local and systemic AEs (for seven days following each vaccine administration) **for Period 1 vaccinations**;<br>• Occurrence, intensity, and relationship of unsolicited AEs for 21 days following each vaccine administration **for both Period 1 and Period 2 vaccinations**;<br>• Occurrences of SAEs, MAAEs, AEs leading to withdrawal, AESIs (including VED), and deaths up to 21 days following each vaccine administration **for both Period 1 and Period 2 vaccinations**;<br>• Occurrences of SAEs, MAAEs, AEs leading to withdrawal, AESIs (including VED, and deaths from Day 43 to Day 201 **in both Period 1 and Period 2**;<br>• Occurrences of SAEs, MAAEs, AEs leading to withdrawal, AESIs (including VED), and deaths from Day 202 to Day 386 **in both Period 1 and Period 2**.	Clarified the safety endpoints to take into account the cross-over design and clarified the text.

#	Section (page #)	Change	Rationale
16.	Synopsis (Secondary Endpoints – Immunogenicity)<br>AND<br>Section 10.3.2.2 (Secondary Endpoints)	The following Immunogenicity secondary endpoints have been updated as follows:<br>Phase 2 portion<br>• Relative neutralizing antibody response ~~between~~ **for** the **combination of** healthy adults (Study Population #1) and ~~the~~ healthy elderly adults (Study Population #2) ~~combined~~ **compared to adults** and **the elderly** adults with significant comorbidities (Study Population #3) induced at 21 days after the second vaccination will be analyzed using the following parameter: GMT;<br><br>Phase 3 portion<br>• In ~~a~~ **the immunogenicity** subset ~~of subjects~~ **prior to cross-over and post-cross-over**, Nab antibody response induced in each Study Population against the SARS-CoV-2 virus on Days 0 (all subjects), 21, 42 (all subjects), 201, and 386 analyzed, using the following parameters: GMT, SC rate, and GMFR;<br>• In ~~a~~ **the immunogenicity** subset ~~of subjects~~ **prior to cross-over and post-cross-over**, specific antibody response induced in each Study Population against the SARS-CoV-2 virus measured by the total IgG levels on Days 0, 21, 42, 201, and 386, and analyzed using the following parameters: GMT, SC rate, and GMFR;<br>• In ~~a~~ **the immunogenicity** subset ~~of subjects~~ **prior to cross-over and post-cross-over**, the ratio of neutralizing antibody titers:IgG (ELISA) antibody titers at Day 21, Day 42, Day 201, and Day 386;<br>• In ~~a~~ **the immunogenicity** subset ~~of subjects~~ **prior to cross-over and post-cross-over**, specific Th1 CMI response induced in each Study Population against the SARS-CoV-2 virus on Days 0, 21, 42, 201, and 386, as measured by IFN-γ ELISpot;<br>• In ~~a~~ **the immunogenicity** subset ~~of subjects~~ **prior to cross-over and post-cross-over**, specific Th2 CMI response induced in each Study Population against the SARS-CoV-2 virus on Days 0, 21, 42, 201, and 386, as measured by IL-4 ELISpot;	Clarified the immunogenicity endpoints to take into account the cross-over design and clarify the text.

#	Section (page #)	Change	Rationale
		• In ~~a~~ **the immunogenicity** subset ~~of subjects~~ **prior to cross-over and post-cross-over**, specific CMI response induced in each Study Population against the SARS-CoV-2 virus up to 21 days after the second vaccination and at Day 201 and Day 386, as measured by the percentage of CD4+ T cells expressing functional markers;	
17.	Synopsis (Secondary Endpoints – Efficacy)<br>AND<br>Section 10.4.2.2 (Secondary Endpoints)	The following Efficacy secondary endpoints have been updated as follows:<br>• Occurrences of laboratory-confirmed (confirmed by the ELISA method for the N protein) asymptomatic SARS-CoV-2 infection ~~at Day 201 and Day 386;~~ **starting 7 days after the second vaccination in Period 1 and prior to the cross-over;**<br>• Occurrences of severe COVID-19 disease (≥ 7 days post-second vaccination) **in Period 1 and prior to cross-over**. Refer to the definition of severe COVID-19 disease in the Secondary Objectives section above;<br>• Occurrence and intensity of COVID-19-related symptoms ~~up to~~ **in virologically-confirmed cases up until** resolution of the symptoms **in Period 1 compared to subjects administered the placebo and compared to subjects administered CoVLP at the time of cross-over in Period 2**;<br>• First occurrence, in a subject, of laboratory-confirmed (virologic method) symptomatic SARS-CoV-2 infection (≥ first vaccination) **in Period 1 and prior to cross-over**;<br>• First occurrence, in a subject, of laboratory-confirmed (virologic method) symptomatic SARS-CoV-2 infection (≥ first vaccination and < second vaccination) **in Period 1 and prior to cross-over**;<br>• First occurrence, in a subject, of laboratory-confirmed (virologic method) symptomatic SARS-CoV-2 infection (≥ second vaccination and < 7 days post-second vaccination) **in Period 1 and prior to cross-over**;<br>• Occurrences of laboratory-confirmed (confirmed by the ELISA method for the N protein) asymptomatic SARS-CoV-2 infection ~~at Day 201 and Day 386~~ **starting 7 days after the vaccination in Period 1 and prior to the cross-over** in subjects who only received a single vaccination;	Clarified the efficacy endpoints to take into account the cross-over design.

#	Section (page #)	Change	Rationale
		• Duration and intensity of viral shedding after **virologically-confirmed** SARS-CoV-2 infection **in Period 1 and prior to cross-over**;<br>• **First occurrence, in a subject, of laboratory-confirmed (virologic method) symptomatic SARS-CoV-2 infection (≥ 7 days post-second vaccination) in Period 1 by strain and prior to cross-over;**	
18.	Synopsis (Exploratory Endpoints – Efficacy)<br>AND<br>Section 10.4.2.3 (Secondary Endpoints)	The following Efficacy secondary endpoints have been updated as follows:<br>• ~~The slope estimate of the weighted Schoenfeld residuals against event time for the evaluation of vaccine efficacy determined from the~~ **Time to** first occurrence~~, in a subject,~~ of ~~laboratory~~ **virologically**-confirmed ~~(virologic method)~~symptomatic ~~SARS-CoV-2 infection~~ **disease as well as serologically-confirmed asymptomatic disease assessed for any strain and by strain throughout the study**;<br>• **Occurrences of severe COVID-19 disease** (≥ 7 days post-second vaccination) **by strain. Refer to the definition of severe COVID-19 disease in the Secondary Objectives section above;**	Clarified the efficacy endpoints to take into account the cross-over design.
19.	Synopsis (Statistical Methods – Phase 3 Portion Interim Efficacy Analyses)<br>AND<br>Section 12.3 (Day 21 and Day 43 Immunogenicity Data Analysis and Interim Efficacy Analyses)	The title and content under the sub-header for the Phase 3 portion Interim Efficacy Analyses have been updated as follows:<br>Phase 3 portion: ~~Interim~~ Efficacy Analys~~e~~**i**s:<br>When ~~80~~ **at least 160** laboratory-confirmed COVID-19 cases **(≥ 7 days post-second vaccination)** have been collected, the unblinded statistical team will perform ~~a first interim~~ **the final** analysis **and produce final efficacy outputs** for the Phase 3 portion of the efficacy data in order to test for ~~early~~ vaccine efficacy. If the primary efficacy success criterion is met during this ~~interim~~ analysis**, emergency use authorization or approval will be pursued and initiation of the cross-over design. Initiation of the cross-over design for subjects enrolled in the study will be dependent on the following overall study and subject-specific criteria:**<br>1. **When Emergency Use Authorization or Approval for the CoVLP formulation is granted by any Regulatory Agency(ies); and**	The cross-over design has been incorporated into the Phase 3 portion of the study as a method to provide subjects who receive the placebo to receive the CoVLP formulation if/when emergency use authorization is granted. In addition, the

#	Section (page #)	Change	Rationale
		2. **When one or more authorized or approved COVID-19 vaccines become available for the subject in his/her country of recruitment; and**<br>3. **The subject has completed a minimum of 60 days in Period 1.**<br>~~enrollment of subjects into the placebo treatment group will stop; subject enrollment into the CoVLP formulation treatment group will continue for the collection of safety data and for a descriptive assessment of the efficacy and immunogenicity endpoints. A second interim analysis for the Phase 3 portion will be performed as soon as 160 cases are collected in order to perform the final analysis and produce final efficacy outputs.~~	interim efficacy analysis has been removed.
20.	Synopsis (Statistical Methods – Efficacy Analyses)	The content in this section has been updated as follows:<br>Once ~~approximately~~ **at least** 160 COVID-19 cases **(≥ 7 days post-second vaccination)** have accumulated during the study, the primary efficacy endpoint of the Phase 3 portion of the study will be evaluated to determine if the vaccine efficacy success criterion has been met. For the efficacy endpoints, vaccine efficacy (VE) will be evaluated. ~~An interim VE analysis will be performed when 80 cases have been collected for an early efficacy assessment.~~<br>The **primary assessment of** VE will be evaluated **before cross-over** as the ~~relative risk~~ **incidence rate ratio** of CoVLP vaccinated versus unvaccinated subjects to develop laboratory-confirmed symptomatic SARS-CoV-2 infection ≥ 7 days after the second vaccination, as per the following formula:<br>$VE=(1-IRR)=(1-(\frac{\boldsymbol{a}}{\boldsymbol{N_1}} \div \frac{\boldsymbol{b}}{\boldsymbol{N_0}})$ ~~ARV/ARU~~ $)*100\ \%$<br>Where:<br>IRR= ~~relative risk~~ **incidence rate ratio**<br>~~ARV= attack rate in subjects vaccinated with CoVLP; and~~<br>~~ARU= attack rate in unvaccinated subjects.~~	The efficacy analysis has been updated to incorporate the cross-over design into the analysis. In addition, the interim efficacy analysis has been removed.

medicago

#	Section (page #)	Change	Rationale
		**a= number of subjects with a least one positive COVID-19 case; and**<br><br>**b= number of subjects with a least one positive COVID-19 cases; and**<br>**N_1= person-years for subjects vaccinated with CoVLP; and**<br>**N_0= person-years in unvaccinated subjects.**<br><br>**Assuming the number of cases in each arm follows a Poisson distribution, then conditioning on the total number of cases and the ratio of person-time of follow up, exact 95 % CIs for the incidence rate ratio IRR, and hence for VE = 1-IRR, are obtained via binomial distribution calculations.**<br><br>The VE success criterion for the primary efficacy endpoint, in the Phase 3 portion, is defined as a ≥ 50 % point estimate and a > 30 % lower limit of the ~~appropriately alpha-adjusted~~ **95 % CI** ~~confidence interval that maintains overall Type I error at 5 %. Statistical significance of the primary efficacy endpoint can be achieved at the interim analysis or at the primary analysis~~.<br><br>VE will also be calculated for the secondary and exploratory efficacy endpoints. The VE success criterion for the secondary efficacy endpoints, in the Phase 3 portion, is defined as > 0 % lower limit of the ~~alpha-adjusted confidence interval~~ 95 % CI ~~confidence interval~~.<br><br>~~The CI around RR will be calculated based on score confidence limits.~~<br><br>To assess the duration and intensity of viral shedding after SARS-COV-2 infection, point estimates and two-sided 95 % CI will be calculated. The area under the curve of the viral titer curve, based on serial quantitative RT-PCR results, will be compared between treatment groups using a linear mixed model with testing dates considered as repeated measures. The peak viral shedding assessed by the lowest CT value and the number of days to negative test will be compared between treatment groups using an ANOVA.<br><br>**Any duration of protection assessments along with its two-sided 95 % CI performed after cross-over will be evaluated using a Cox proportional hazard**	

#	Section (page #)	Change	Rationale
		**model with time-varying data and time-varying parameters for any strain and by strain.**	
21.	Table 1 (Time and Events Schedule: General Information for the Phase 2 Portion)	The following updates have been made to the table:<br>108. The row "History/symptom-directed physical examination" has been updated to "Physical Examination";<br>109. Footnote 2: A limited physical examination will occur at screening. History/symptom-directed physical examinations ~~will not~~ **may** be ~~routinely~~ performed at ~~any~~ other visits~~, unless~~ **if 1)** new complaints or concerns are raised by either the study subject or study staff~~,~~ and ~~if~~ **2)** deemed to be necessary by the Investigator;<br>110. Footnote 7: AEs will be collected up to Day 42; SAEs, MAAEs, AEs leading to withdrawal, and AESIs will be collected through to the end of the study. Investigators should report SAEs, ~~MAAEs,~~ AESIs, and pregnancy reports to Sponsor within 24 hours of the Investigator learning of the event. Specific contacts for the collection of information regarding all of these events will occur on Day 386 (during the Day 386 final visit) for SAEs, MAAEs, AEs leading to withdrawal, and AESI.;	Clarified details for performing the physical examination during the Phase 2 portion. Also clarified that MAAEs do not need to be reported to the Sponsor within 24 hours.
22.	Table 2 (Time and Events Schedule: General Information for the Phase 3 Portion)	The following updates have been made to the table:<br>111. Title: Table 2 Time and Events Schedule: General Information for **Period 1 and Period 2 of** the Phase 3 Portion<br>112. Cell "Visit Type[12]" now includes the footnote number "12";<br>113. Cell "Screening[11]/Vaccination" now includes the footnote number "11" on the word "Screening";<br>114. Four new rows have been added to provide identifiers for each study day/visit in Period 1 and Period 2;<br>115. Columns for study days "Day 1, Day 8, Day 22, and Day 29 have been removed;<br>116. The footnote number "17" has been added to column header "D201[17]";<br>117. The footnote number "18" has been added to column header "D386[18]";	Incorporated details regarding each Phase of the study and the cross-over design in the Phase 3 portion. In addition, clarified when additional immunogenicity samples will be collected. Also, since subjects will be provided with

#	Section (page #)	Change	Rationale
		118. The cell with visit number "5" has been updated as "5/Phone[19]"<br>119. The footnote number "13" has been added to cell "Informed consent[13]";<br>120. The cell "History/symptom-directed physical examination" has been updated to "Physical Examination";<br>121. The footnote number "11" has been added to cell "Inclusion/exclusion criteria[13]";<br>122. The footnote number "11" has been removed from cell "Test for SARS-CoV-2 N antibodies~~[11]~~";<br>123. A new row, "**Immunogenicity – Serology**" as been added with the "**X[9,10]**" placed in the "Day 0" column and the "**X[10]**" placed in the "Day 42" column;<br>124. A new row, "**Immunogenicity – UK CMI (ELISpot and ICS)**" as been added with the "**X[9,22]**" placed in the "Day 0" column and the "**X[22]**" placed in the "Day 42" column;<br>125. The footnote number "10" has been removed from cell "Immunogenicity – Serology (Nab assay and ELISA)[1,~~10~~]";<br>126. Cell "Randomization[16]" now includes the footnote number "16";<br>127. The footnote number "21" has been added to cell "Provide and collect diary and memory aid instructions[21]";<br>128. The footnote number "20" has been added to cell "Collection of solicited local/systemic adverse events[20]";<br>129. The content for the cell "Testing for laboratory confirmation of SARS-CoV-2 infection" has been updated as follows:<br>Subjects who report any symptoms associated with COVID-19 after the start of the active and passive surveillance will be requested to visit the Investigator site to provide nasal or NP swabs ~~(2 per subject per event)~~ to test for COVID-19. **During the visit, the Investigator will confirm whether nasal or NP swabs should be collected.** If the subject cannot visit the Investigator site, then the subject may receive a visit at home **(or hospital, if applicable)** to collect the nasal/NP swabs samples or be provided with self-administered nasal swabs for	electronic diaries, the phone contacts on Days 1, 8, 22, and 29 are no longer needed.

#	Section (page #)	Change	Rationale
		sample collection. **During the 7 days following each vaccination, Investigator judgement will be used to determine whether swabs should be collected from subjects reporting symptoms similar to those associated with COVID-19 since such symptoms may also be due to the reactogenicity of the study treatment administered rather than SARS-CoV-2 infection.**<br>The swabs will be collected within 72 hours (preferably within 48 hours) of the reporting of a COVID-19-associated symptom. If swabs cannot be collected within 72 hours after reporting of the COVID-19-associated symptom, nasal or NP swabs should be collected at the next available opportunity for the subject to visit the Investigator site **or at home (or hospital, if applicable)**. **Two swabs per event per subject will be collected and submitted for analysis using a virologic method, at the central virology laboratory. To mitigate the risk of long delays in shipping samples to the central virology laboratory, an additional local virologic test will be performed.**<br>130. Footnote 1: [1] These samples will only be collected from ~~a subset of subjects.~~ **the subjects included in the immunogenicity subset. The immunogenicity subset will include the same subjects in Period 1 and Period 2.**<br>131. Footnote 2: [2] **A limited physical examination will occur at screening.** History/symptom-directed physical examinations ~~will~~ **may** ~~not~~ be ~~routinely~~ performed at ~~any~~ other visits~~, unless~~ **if 1)** new complaints or concerns are raised by either the study subject or study staff~~,~~ and ~~if~~ **2)** deemed to be necessary by the Investigator.<br>132. Footnote 7: [7] AEs will be collected up to Day 42; SAEs, MAAEs, AEs leading to withdrawal, and AESIs will be collected through to the end of the study. Investigators should report SAEs~~, MAAEs,~~ AESIs, and pregnancy reports to Sponsor within 24 hours of the Investigator learning of the event. Specific contacts for the collection of information regarding all of these events will occur on Day 386 ~~(during the Day 386 final visit)~~ for SAEs, MAAEs, AEs leading to withdrawal, and AESI.<br>133. Footnote 8: [8] All efforts will be done to have subjects returning on planned date for Day 21 activities. If for any reason the visit is done before or after this	

#	Section (page #)	Change	Rationale
		planned date, subsequent visits/procedures (Days ~~22, 29,~~ 42, 201, and 386) will be adjusted accordingly.<br>134. Footnote 9: [9] Blood and urine **(pregnancy test)** samples are to be collected prior to vaccination on the respective day.<br>135. Footnote 10: [10] ~~The~~ **An** immunogenicity**-serology** sample at **Day 0 and** Day 42 will be collected from all subjects.<br>136. Footnote 11: [11] **In Period 1, this visit will consist of Screening activities prior to vaccination in Period 1. In Period 2, this visit will consist of assessing subjects for contraindications to the first vaccination in Period 2.** ~~The Day 0 sample collected, prior to vaccination, from subjects not included in the immunogenicity subset will also be used for the immunogenicity assessment (Nab assay and ELISA).~~<br>**137.** Footnote 12: **[12] The same study visits and contacts described are planned for both Period 1 and Period 2. During Period 1, subjects may not complete all planned study visits or contacts prior to cross-over since cross-over for a subject can be initiated as early as Day 60 once emergency use authorization or approval for the CoVLP formulation is granted in any jurisdiction and when one or more COVID-19 vaccines become available for the subject in his/her country of recruitment; even after the cross-over has been initiated, subjects will still return for the Day 201 and Day 386 visits (which will be aligned with post-cross-over visits that are within close proximity of the planned date). During Period 2, study visits/contacts will be performed as planned (i.e., Day 21, Day 42, Day 201, and Day 386 post-cross-over).**<br>138. Footnote 13: **[13] The informed consent form will be signed and dated prior to the start of Period 1. Unless a new consent form has been approved for use and is required to be signed and dated by all the subjects, subjects will not be required to sign and date the same consent form prior to starting Period 2.**<br>139. Footnote 14: **[14] The Day 0 sample will be collected prior to vaccination in Period 1 and Period 2. The sample collected prior to vaccination in Period 2**	

#	Section (page #)	Change	Rationale
		**represents the sample collected "at cross-over" for the purposes of the efficacy evaluation.**<br>140. Footnote 15: [15] **The Day 386 sample will only be collected during the visit for Day 386 of Period 1.**<br>141. Footnote 16: [16] **Randomization will only occur prior to the start of Period 1. Treatment assignments for Period 2 will be done in the interactive response technology (IRT) system.**<br>142. Footnote 17: [17] **Based on the expected SARS-CoV-2 attack rate in unvaccinated subjects and the expected rate of subject enrollment in the Phase 3 portion, it is assumed that cross-over will occur prior to Day 201 (more than 45 days before Day 201), thus the Day 201 visit of Period 1 will occur at approximately the same time as the Day 42 visit of Period 2 (i.e., on the same day). If cross-over occurs within 45 days before the Day 201 visit, then the Day 201 visit of Period 1 will occur at approximately the same time as the Day 0 visit of Period 2 (i.e., on the same day).**<br>143. Footnote 18: [18] **Based on the expected SARS-CoV-2 attack rate in unvaccinated subjects and the expected rate of subject enrollment in the Phase 3 portion, it is assumed that cross-over will occur prior to Day 386, thus the Day 386 visit of Period 1 will occur at approximately the same time as the Day 201 visit of Period 2 (i.e., on the same day).**<br>144. Footnote 19: [19] **In Period 2, all subjects in the immunogenicity subset will visit the clinical site to complete the Day 386 visit. For all other subjects, a final phone contact will be done.**<br>145. Footnote 20: [20] **In Period 1, immediate AEs (30 minutes after each vaccination) and solicited local and systemic AEs (up to seven days after each vaccination) will be collected from all subjects. In Period 2, immediate AEs (30 minutes after each vaccination) will still be collected however solicited local and systemic AEs (up to seven days after each vaccination) will not be collected from the subjects.**	

#	Section (page #)	Change	Rationale
		146. Footnote 21: [21] **During the first 8 days after each vaccination in Period 2, if data entry in the electronic diary is missed for 2 consecutive days by a subject then the subject will receive a follow-up contact (phone, text, or email) to remind him/her to record his/her data in the electronic diary.**<br>147. Footnote 22: [22] **Samples for the immunogenicity-UK CMI (ELISpot and ICS) assessment will be collected at Day 0 and Day 42 from 400 subjects included in the UK-CMI subset in Period 1 only.**	
23.	Table 3 (Time and Events Schedule: Onset of COVID-19)	The following updates have been made to the table:<br>148. Footnote 1: Nasal or NP swab collection is to be done within 72 hours after reporting of the COVID-19 symptoms (preferably within 48 hours), at the discretion of the site Investigator, and prior to the use of any antiviral treatment medication. If swab collection cannot be done within 72 hours after reporting of the COVID-19 symptoms, swabs should be collected at the next available opportunity for the subject to visit the Investigator site **or at home (or hospital, if applicable)** and date and time of collection recorded.<br>149. Footnote 3: Swab collection will be done for any subject who reports any COVID-19-associated symptom after the start of the active and passive surveillance, **at the discretion of the site Investigator**.	Added clarifying details about swab collection.
24.	Section 1.1 (Background)	The fifth paragraph has been updated as follows:<br>Vaccination is the most effective strategy for preventing infectious diseases since it appears to be more cost-effective than therapeutic drugs and reduces morbidity and mortality without causing long-lasting undesirable effects. Although three human coronaviruses emerged worldwide over the past two decades, causing considerable threat to global health, only the Pfizer-BioNTech COVID-19 vaccine (**16+ years of age** ~~Canada and the US~~) **and the Moderna COVD-19 vaccine (18+ years of age)** ha**ve**~~s~~ been approved for the prevention of COVID-19 in **Canada and the US** people who are 16 years of age and older. Research groups around the world are accelerating the development of COVID-19 vaccines using various approaches [Ahn 2020, Lu 2020].	Added updated information about COVID-19 vaccine approval in Canada and the US.

#	Section (page #)	Change	Rationale
25.	Section 4.1 (Overview of Study Design)	A new figure describing the cross-over design for the Phase 3 portion of the study has been added to the end of the section.	Incorporated a descriptive image of the cross-over design in the Phase 3 portion.
26.	Section 4.2.3 (Dose Selection and Dosage Regimen)	The content in this section has been updated as follows:<br>In both the Phase 2 and Phase 3 portions, a single dose level of the CoVLP formulation (i.e. 3.75 µg CoVLP adjuvanted with AS03) will be tested in a two-dose regimen on Day 0 and Day 21. **In the Phase 3 portion, the use of the cross-over design will result in each subject receiving the CoVLP formulation and the placebo.**	Added details of the cross-over design.
27.	Section 4.2.6 (Cross-over Design)	A new section with the following content has been added:<br>**The use of the blinded cross-over in the Phase 3 portion of the study will allow subjects who receive the placebo to get timely access to an authorized COVID-19 vaccine, in this case the CoVLP formulation upon its receiving emergency use authorization or approval in any jurisdiction.**	A rationale for the use of the cross-over design in the Phase 3 portion of the study has been included.
28.	Section 5.1 (Inclusion Criteria)	The inclusion criteria # and # have been updated as follows:<br>3. At Screening (Visit 1) and Vaccination (Visit 2), subject must have a body mass index (BMI) of:<br>**<u>For the Phase 2 portion of the study only</u>:**<br>• Study Populations #1 and #2: ≥ 18.5 and < 30 kg/m^2;<br>**<u>For the Phase 3 portion of the study only</u>:**<br>• **Study Populations #1 and #2: ≥ 18.5 and < 35 kg/m^2;**<br>9. <u>Study Population #3</u>: Subjects must have one or more co-morbid conditions that puts them at higher risk for severe COVID-19 disease. These comorbidities include but are not limited to obesity **(moderate or greater)**, hypertension, type 1 or type 2 diabetes, chronic obstructive pulmonary disease (COPD), cardiovascular diseases, chronic kidney diseases, or be immunocompromised persons (e.g., treatment-	Clarified the BMI range for Study Populations #1 and #2 and updated the obesity range for Study Population #3.

#	Section (page #)	Change	Rationale
		controlled HIV infection, organ transplant recipients, or patients receiving cancer chemotherapy). Investigator discretion will be permitted with this inclusion criterion.	
29.	Section 5.1 (Inclusion Criteria)	The last bullet point for inclusion criteria #7 has been updated as follows:<br>• Bilateral tubal ~~ligation~~ **occlusion**	Corrected a typo.
30.	Section 5.2 (Exclusion Criteria)	The following exclusion criteria have been updated as follows:<br>4. Study Populations #1 and #2: Current autoimmune disease (such as rheumatoid arthritis, systemic lupus erythematosus~~,~~ **or** multiple sclerosis~~ or narcolepsy~~). Investigator discretion is permitted with this exclusion criterion, and subjects may be eligible to participate with appropriate written justification in the source document (i.e. subjects with a history of autoimmune disease who are disease-free without treatment for three years or more, or on stable thyroid replacement therapy, mild psoriasis [i.e. a small number of minor plaques requiring no systemic treatment], etc.);<br>16. Personal or family **(first-degree relatives)** history of narcolepsy;	Clarified the exclusion criteria for narcolepsy.
31.	Section 5.3 (Prior and Concomitant Therapy)	The second and third bullet points of the second paragraph have been updated as follows:<br>• From randomization to Day 42 **(Phase 2 portion and Period 1 of Phase 3 portion) or Day 0 to Day 42 (Period 2 of Phase 3 portion)**, inclusive: any medication (including, but not limited to, over-the-counter medicines such as aspirin or antacids), vitamins, and mineral supplements;<br>• From Day 43 to the end of the study **(Phase 2 portion) or of each period (Phase 3 portion)**, inclusive: any concomitant medication(s) administered to treat an AESI, MAAE, SAE, or AE leading to withdrawal; any concomitant medication used to treat an AE that occurred before Day 42 and that is still being used afterwards (i.e. on-going use);	Incorporated details regarding each Phase of the study and the cross-over design in the Phase 3 portion.
32.	Section 5.4 (Prohibited Therapy)	The first and third points have been updated as follows:<br>1. Administration of any vaccine (other than the study vaccine) up to Day 28 of the study **(Phase 2 portion) or of each period (Phase 3 portion)** as well as administration of any investigational or approved coronavirus vaccine (other	Incorporated details regarding each Phase of the study and the cross-over

#	Section (page #)	Change	Rationale
		than the study vaccine) up to end of the study. Immunization on an emergency basis during the study will be evaluated on case-by-case basis by the Investigator;<br>3. Study Populations #1 and #2: Administration of any medication or treatment that may alter the vaccine immune responses, such as:<br>• Systemic glucocorticoids;<br>• Cytotoxic, antineoplastic, or immunosuppressant drugs;<br>• Any immunoglobulin preparations or blood products, or blood transfusion.<br>Administration of such medications should be specifically avoided up to Day 42 of the study **(Phase 2 portion) or of each period (Phase 3 portion)**. Use of any medication or treatment that may alter the vaccine immune responses due to a medical need during the study will be evaluated on case-by-case basis by the Investigator;	design in the Phase 3 portion.
33.	Section 6.1 (Randomization)	Two new paragraphs have been added after the first paragraph as follows:<br>**For the immunogenicity subset in the Phase 3 portion, subjects will be enrolled from select clinical sites based on the sites capability to perform either cryopreservation of peripheral blood mononuclear cell (PBMC) samples and / or the requirement to be located near the laboratory where whole blood samples could be delivered quickly for PBMC processing. At the clinical sites, selection of the subjects to be included in this subset will be performed by the unblinded statistical team or the IRT system sequentially starting with the first subject of the day, based on age distribution (when applicable), treatment received, and the site's daily capacity to process PBMC samples until the targeted sample size of the site is obtained. The same process will be followed for enrolling subjects into each of the three Study Populations.**<br>**For the UK-CMI subset in the Phase 3 portion, subjects will be enrolled from select clinical sites in the UK. At the clinical sites, selection of the subjects to be included in this subset will be performed by the unblinded statistical team or the IRT system sequentially starting with the first subject of the day, based on age**	Clarified how the immunogenicity subset will be enrolled in the Phase 3 portion of the study, based on feedback from CBER. Also added details of how the UK-CMI subset will be enrolled.

#	Section (page #)	Change	Rationale
		**distribution (when applicable), and treatment received until the targeted sample size of the site is obtained.**	
34.	Section 8.1 (Management of Samples)	The second, third, fourth, and fifth paragraphs have been updated as follows:<br>Blood samples for immunogenicity analysis will be collected from all subjects in the Phase 2 portion of the study and from ~~a~~ subset**s** of subjects in the Phase 3 portion of the study. **In the Phase 3 portion, additional immunogenicity samples will be collected at Day 0 and Day 42 from all subjects.** Should a subject be discontinued from the study, a sample will be drawn at the time of the final visit and sent to Medicago for analysis. Bioanalysis of the immunogenicity blood samples will be conducted at central laboratories.<br>Between vaccination and the end of the surveillance period, nasal or NP swabs will be collected from any subject who reports any symptoms associated with COVID-19 after the start of active and passive surveillance at the discretion of the site Investigator; these swabs will be collected each time a subject reports such an event within the specified timeframe, so multiple collections may be required from some subjects. **At least t**~~T~~wo swabs will be collected per subject per event (**at least** one from each nare). **If the subject cannot visit the Investigator site, then the subject may receive a visit at home (or hospital, if applicable) to collect the nasal/NP swabs samples or be provided with self-administered nasal swabs for sample collection.** Bioanalysis of the nasal or NP swabs will be conducted at **the** central laboratory **as well as local laboratories, to mitigate the risk of long delays in shipping samples to the central virology laboratory (refer to Section 10.4.1 for more details)**.<br>~~To mitigate the risk of long delays in shipping samples to the central virology laboratory in the Netherlands, an additional rapid turn-around virologic test will be performed 'locally' using an approved NP or nasal assay (i.e., local laboratory or point-of-care, nucleic acid or antigen-detection). The purpose of this supplementary assay is to provide a preliminary diagnosis and permit appropriate symptom-recording and sample collection as quickly as possible.~~<br>Complete information on the **supplies,** handling, storage, and shipment of all laboratory samples will be described in the study-specific documentation.	Clarified that an additional immunogenicity sample at Day 0 and Day 42 will be collected in all subjects; for subjects in the immunogenicity subset, they will provide two immunogenicity samples at Day 0 and Day 42. Also, clarified details regarding swab collection.

<table>
<tr><th>#</th><th>Section (page #)</th><th>Change</th><th>Rationale</th></tr>
<tr>
<td>35.</td>
<td>Section 8.2 (Collection of Samples)</td>
<td>
The third, sixth, seventh, and eighth paragraphs and the second table in this section have been updated as follows:

In the Phase 3 portion of the study, all subjects will have **up to a total of 55** ~~25~~ mL of blood drawn**.** ~~up to the end of the study (Day 386)~~**.** ~~and only a subset of subjects~~ **Subjects** included in the immunogenicity ~~analyses~~ **subset** will have **up to** an additional blood volume of up to approximately ~~250~~ **500** mL drawn up to the end of the study ~~(Day 386)~~**. Subjects included in the UK-CMI subset will have up to an additional blood volume of up to approximately 60 mL drawn up to the end of Period 1. In total, subjects will have up to 55 to 555 mL of blood drawn up to the end of the study** (Table 8).

Table 8 Estimated Blood Volume Drawn: Phase 3 Portion
<table>
<tr><th>Type of Sample</th><th>Volume per Sample (mL)</th><th colspan="7">Number of Samples per Subject</th><th>Total Volume of Blood per Subject **per Period** (mL)</th><th>**Total Volume of Blood per Subject (mL)**</th></tr>
<tr><th></th><th></th><th>Visit 1 (Day 0)</th><th>Visit 2 (Day 21)</th><th>Visit 3 (Day 42)</th><th></th><th>Visit 4 (Day 201)[§]</th><th>Visit 5 (Day 386)[§]</th><th>Total</th><th></th><th></th></tr>
<tr><td>Test for SARS-CoV-2 antibodies</td><td>5</td><td>1[†]</td><td></td><td></td><td></td><td>~~1~~</td><td>1[‡]</td><td>**2**~~3~~</td><td>~~15~~**10**</td><td>**15[†,‡]**</td></tr>
<tr><td>**Immunogenicity – serology (Nab assay only)**</td><td>**10**</td><td>**1**</td><td></td><td>**1**</td><td></td><td></td><td></td><td>**2**</td><td>**20**</td><td>**40**</td></tr>
<tr><td>**Immunogenicity – UK CMI (ELISpot and ICS)***</td><td>**30**</td><td>**1**</td><td></td><td>**1**</td><td></td><td></td><td></td><td>**2**</td><td>**60**</td><td>**60**</td></tr>
<tr><td>~~Serology for~~ ~~i~~**I**mmunogenicity **- serology** (Nab assay, ELISA)*</td><td>10</td><td>1</td><td>1</td><td>1**</td><td></td><td>1</td><td>1</td><td>5</td><td>50</td><td>**100**</td></tr>
<tr><td>**Immunogenicity -** CMI response (**ELISpot and ICS** ~~PBMC~~)*</td><td>40</td><td>1</td><td>1</td><td>1</td><td></td><td>1</td><td>1</td><td>5</td><td>200</td><td>**400**</td></tr>
</table>
</td>
<td>Clarified that additional immunogenicity samples will be collected at Day 0 and Day 42. In addition, incorporated details of the cross-over design in the Phase 3 portion.</td>
</tr>
</table>

<table>
<tr><th>#</th><th>Section (page #)</th><th>Change</th><th>Rationale</th></tr>
<tr><td></td><td></td><td>
<table>
<tr><td>Total volume of blood per subject (mL)</td><td></td><td>15-~~5~~65</td><td>50</td><td>10-~~5~~60</td><td></td><td>50~~5~~</td><td>5-55</td><td></td><td>30-~~265~~280</td><td>55-555</td></tr>
</table>
* These samples will be collected for ~~a~~ pre-defined subsets of subjects.
† The Day 0 sample will be collected prior to vaccination in Period 1 and Period 2. The sample collected prior to vaccination in Period 2 represents the sample collected “at cross-over” for the purposes of the efficacy evaluation.
‡ The Day 386 sample will only be collected during the visit for Day 386 of Period 1.
§ Based on the expected SARS-CoV-2 attack rate in unvaccinated subjects and the expected rate of subject enrollment in the Phase 3 portion, it is assumed that cross-over will occur prior to Day 201 (more than 45 days before Day 201), thus the Day 201 visit of Period 1 will occur at approximately the same time as the Day 42 visit of Period 2 (i.e., on the same day). If cross-over occurs within 45 days before the Day 201 visit, then the Day 201 visit of Period 1 will occur at approximately the same time as the Day 0 visit of Period 2 (i.e., on the same day). If cross-over occurs prior to Day 386, thus the Day 386 visit of Period 1 will occur at approximately the same time as the Day 201 visit of Period 2 (i.e., on the same day).
~~**This sample will be collected in a subjects.~~
In the Phase 3 portion of the study, pregnancy testing will be performed for all females of childbearing potential (in Study Populations #1 and #3) during each period. A urine (dipstick or similar) pregnancy test will be performed at screening and at Day 0 prior to randomization and at Days 21 and 42. In Study Populations #1 and #3, females of child bearing potential must have negative urine pregnancy test results from screening and Day 0 available prior to vaccination in each period.
~~Nasal or NP swabs will be collected from any subject who reports any symptoms associated with COVID-19 after the start of active and passive surveillance, at the discretion of the site Investigator. Two swabs will be collected per subject per event (one from each nare). The swabs will be collected within 72 hours (preferably within 48 hours) of the reporting of a COVID-19-associated symptom. If swabs cannot be collected within 72 hours after reporting of the COVID-19-associated symptom, nasal or NP swabs should be collected at the next available opportunity for the subject to visit the Investigator site.~~
~~Additional serial nasal swabs (self-collected) and blood sampling may be requested of subjects with virologically-confirmed COVID-19 infection.~~
</td><td></td></tr>
</table>

#	Section (page #)	Change	Rationale
36.	Section 10.1.1 (Overview)	A new paragraph has been added after the first paragraph as follows:<br>**For the Phase 3 portion of the study, the frequency and timing of scheduled assessments will be the same for Period 1 and Period 2, with the exception of the following:**<br>• **Screening visit (Visit 1 for Phase 3 portion):**<br>• **Prior to starting Period 1, screening assessments will be performed to determine initial eligibility of subjects in the study;**<br>• **Prior to starting Period 2, subjects will be assessed to ensure they do not meet any contraindications to the first vaccination in Period 2;**<br>• **The informed consent form will be signed and dated prior to the start of Period 1. Unless a new consent form has been approved for use and is required to be signed and dated by all the subjects, subjects will not be required to sign and date the same consent form prior to starting Period 2;**<br>• **For the SARS-CoV-2 antibodies (Phase 3 portion) assessment:**<br>• **The Day 0 sample will be collected prior to vaccination in Period 1 and Period 2. The sample collected prior to vaccination in Period 2 represents the sample collected "at cross-over" for the purposes of the efficacy evaluation;**<br>• **The Day 386 sample will only be collected during Period 1 and subjects should return for this visit even if cross-over has been initiated prior to Day 386 of Period 1;**<br>• **Randomization will only occur prior to the start of Period 1. Treatment assignments for Period 2 will be done in the IRT system.**<br>**During Period 1, subjects may not complete all study contacts prior to cross-over since cross-over for a subject can be initiated as early as Day 60 once emergency use authorization or approval for the CoVLP formulation is granted in any jurisdiction and when one or more COVID-19 vaccines become available for the subject in his/her country of recruitment; even after the cross-over has**	Provided additional details regarding the implementation of the cross-over design and clarified how Section 10.1 should be interpreted in regards to the study visits and contacts in the Phase 3 portion of the study.

#	Section (page #)	Change	Rationale
		**been initiated, subjects will still return for the Day 201 and Day 386 visits (which will be aligned with post-cross-over visits that are within close proximity of planned date). Based on the expected SARS-CoV-2 attack rate in unvaccinated subjects and the expected rate of subject enrollment in the Phase 3 portion, it is assumed that cross-over will occur prior to the Day 201 visit (more than 45 days before Day 201), thus:**<br>• **The Day 201 visit of Period 1 will occur at approximately the same time as the Day 42 visit of Period 2 (i.e., on the same day);**<br>• **The Day 386 visit of Period 1 will occur at approximately the same time as the Day 201 visit of Period 2 (i.e., on the same day).**<br>**If cross-over occurs within 45 days before the Day 201 visit, then:**<br>• **The Day 201 visit of Period 1 will occur at approximately the same time as the Day 0 visit of Period 2 (i.e., on the same day);**<br>• **The Day 386 visit of Period 1 will occur at approximately the same time as the Day 201 visit of Period 2 (i.e., on the same day).**<br>**In the event that the cross-over occurs after the Day 201 visit and before the Day 386 visit, then:**<br>• **The Day 386 visit of Period 1 will still occur at approximately the same time as the Day 201 visit of Period 2 (i.e., on the same day).**<br>**During Period 2, study visits and contacts will be performed as planned.**	
37.	Section 10.1.3 (Screening)	The sixth bullet point has been updated as follows:<br>• Perform a ~~history- or symptom-directed~~ **limited** physical examination. The physical examination will be performed by the Investigator or sub-Investigator;	Clarified details of the physical examination to be performed at screening.

#	Section (page #)	Change	Rationale
38.	Section 10.1.4.1 (Prior to Vaccination)	The last bullet point in this section has been updated as follows:<br>• After confirmation of eligibility and randomization, collect baseline blood samples to test for:<br>• SARS-CoV-2 antibodies (Phase 3 portion) **assessment;** ~~and~~<br>• ~~for i~~**I**mmunogenicity (serology [Nab assay and ELISA] and CMI) assessments (Phase 2 portion - all subjects; Phase 3 portion - immunogenicity subset);<br>• **Immunogenicity (UK CMI [ELISpot and ICS]) assessment (only Period 1 of Phase 3 portion – UK-CMI subset);**<br>• **Additional immunogenicity (serology) assessment (Phase 3 portion).**<br>~~p~~**P**repare and store these samples until shipment to the analytical laboratory.	Clarified that an additional immunogenicity sample at Day 0 will be collected in all subjects; for subjects in the immunogenicity subset, they will provide two immunogenicity samples at Day 0.
39.	Section 10.1.4.3 (Thirty Minutes Post-vaccination)	The fourth and sixth bullet points have been updated and a new bullet point has been added after the fourth bullet point as follows:<br>• **Phase 2 portion and Period 1 of Phase 3 portion only:** Instruct the subjects to perform measurements of local and systemic AEs at approximately the same time each day from Day 0 to Day 7 (preferably in the evening);<br>• **Period 2 of the Phase 3 portion only: Subjects will not be required to collect solicited local and systemic AEs from Day 0 to Day 7 post-vaccination. Subjects will continue to use the memory aid to record unsolicited AEs and any concomitant medication use;**<br>• Provide appointments (date and time) for the next planned visit to the clinical site (Day 3 for the Phase 2 portion or Day 21 for the Phase 3 portion) and for the Days 1 and 8 phone contacts **(Phase 2 portion only)**;	Since subjects will be provided with electronic diaries, the phone contacts on Days 1, 8, 22, and 29 are no longer needed. Also, post-cross-over, solicited AEs do not need to be collection.
40.	Section 10.1.5 (Day 1 and Day 8)	The title for this section has been updated as follows:<br>Day 1 and Day 8 (Telephone Contact **for Phase 2 portion only**)	Since subjects will be provided with electronic diaries, the phone contacts on Days 1, 8, 22,

#	Section (page #)	Change	Rationale
			and 29 are no longer needed in the Phase 3 portion.
41.	Section 10.1.7 (Day 21 Dose 2 Vaccination)	The content in this section has been updated as follows:<br>If for any reason the Day 21 visit is done before or after the planned date, then subsequent visits/procedures (Days 22 **(Phase 2 portion only)**, 24 (Phase 2 portion only), 29 **(Phase 2 portion only)**, 42, 128 **(Phase 2 portion only)**, 201, and 386) will be adjusted accordingly.	Since subjects will be provided with electronic diaries, the phone contacts on Days 1, 8, 22, and 29 are no longer needed in the Phase 3 portion.
42.	Section 10.1.7.3 (Post-Dose 2 Vaccination)	The first bullet point has been updated as follows:<br>• Provide appointments (date and time) for the next planned visit to the clinical site (Day 24 for the Phase 2 portion or Day 42 for the Phase 3 portion) and for Days 22 and 29 telephone contacts **(Phase 2 portion only)**;	Since subjects will be provided with electronic diaries, the phone contacts on Days 1, 8, 22, and 29 are no longer needed in the Phase 3 portion.
43.	Section 10.1.8 (Day 22 and Day 29)	The title for this section has been updated as follows:<br>Day 22 and Day 29 (Telephone Contact **for Phase 2 portion only**)	Since subjects will be provided with electronic diaries, the phone contacts on Days 1, 8, 22, and 29 are no longer needed in

#	Section (page #)	Change	Rationale
			the Phase 3 portion.
44.	Section 10.1.10 (Surveillance for COVID-19 Cases)	The content of Section 10.1.10 has been moved to Section 10.1.6, with the following changes made to the fifth bullet point and the sub-bullet points of the sixth bullet point:<br>• Schedule a time when the subject can return to the clinical site for the collection of nasal or NP swabs. If the subject cannot visit the Investigator site, then schedule a home **(or hospital, if applicable)** visit to collect the nasal/NP swabs samples or provide the subject with self-administering nasal swabs for sample collection. The Investigator will confirm whether nasal or NP swabs should be collected. The nasal or NP swabs are to be collected within 72 hours (preferably within 48 hours) after the reporting of a COVID-19 symptom;<br>Note: If swabs cannot be collected within 72 hours after reporting of the COVID-19-associated symptom, nasal or NP swabs should be collected at the next available opportunity for the subject to visit the Investigator site or at home **(or hospital, if applicable)** and date and time of collection recorded.<br>• COVID-19 cases that start on or after the day of the first vaccination (Day 0, post vaccination) will be followed up. This follow-up will be conducted via the planned active surveillance contacts. All follow-up information should be collected, regardless of whether nasal or NP swabs could be obtained. As part of this follow up, the following information will be needed (more details in Section **Error! Reference source not found.**):<br>• Collect any new information regarding the evolution of the COVID-19 case, including symptoms and any associated concomitant medication use;<br>• **Collect the results of the subject's serial (every other day) self-administered nasal swab testing results. The swab testing is to be continued for 2 weeks. However, if the COVID-19-associated symptoms:**	Added more details about the surveillance of COVID-19-assoicated symptoms and the collection of swab samples.

#	Section (page #)	Change	Rationale
		• **Persist for more than 2 weeks, then the Investigator's clinical judgement is required to extend the collection of swabs beyond the 2 weeks;**<br>• **Have resolved in less than 2 weeks and the Investigator believes the subject has virologically recovered, then two consecutive negative results, obtained locally, are required prior to stopping the collection of the self-administered swabs;**<br>• ~~Collect data regarding the end date (if resolved). The end date is defined as the date on which the last symptom resolved;~~	
45.	Section 10.1.11 (Day 42)	The fourth bullet point has been updated as follows:<br>• Collect blood samples for**:**<br>• ~~i~~**I**mmunogenicity (serology [Nab assay and ELISA~~; all subjects~~] and CMI**) assessments Phase 2 portion - all subjects; Phase 3 portion - immunogenicity subset** ~~[all subjects in Phase 2 and immunogenicity subset only in Phase 3]) assessments~~;<br>• **Immunogenicity (UK CMI [ELISpot and ICS]) assessment (only Period 1 of Phase 3 portion – UK-CMI subset);**<br>• **Additional immunogenicity (serology) assessment (Phase 3 portion).**<br>~~p~~**P**repare and store these samples until shipment to the analytical laboratory;	Clarified that additional immunogenicity samples at Day 42 will be collected.
46.	Section 10.1.13 (Day 201)	The fourth and seventh bullet points have been updated as follows:<br>• ~~Phase 3 portion only: collect blood sample to test for SARS-CoV-2 antibodies;~~<br>• Provide appointments (date and time) for the next telephone contacts **(including Day 386, if applicable)** and the next planned visit to the clinical site (Day 386)**, if applicable**.	Updated the visit details to remove a sample that will be collected at cross-over instead.
47.	Section 10.1.14 (Final Visit – Day 386)	The content in this section has been updated as follows:<br>10.1.14 ~~Final Visit –~~ Day 386 (Visit 8 for Phase 2 portion and Visit 5/**Telephone Contact** for Phase 3 portion)<br>**10.1.14.1 Visit 8 for Phase 2 portion and Visit 5 for Phase 3 portion**	Clarified the activities for Day 386 since not all subjects will need to visit the clinical

#	Section (page #)	Change	Rationale
		**For the Phase 2 portion and Period 1 (all subjects) and Period 2 (immunogenicity subset) of the Phase 3 portion of the study: t**~~T~~he post-vaccination final visit procedures will be performed by blinded site staff members at the clinical site. For subjects in quarantine, these procedures will be performed by blinded trained medical staff and may be at the subject's home (or place of quarantine). The following procedures will be performed during the Day 386 visit (± 14 days)~~for the Phase 2 and Phase 3 portions of the study;~~:<br>• Review the subject's safety data and ensure all updates on concomitant medication usage and any changes in the subject's health (AEs ongoing from Day 42, SAEs, AESIs, or MAAEs) are recorded appropriately. Collect the subject's memory aid;<br>• Confirm that the subject has continued to comply with protocol requirements (e.g. no use of prohibited concomitant medications, reporting of any COVID-19 symptoms);<br>• Phase 2 portion only: perform a vital signs measurement, including resting BP, HR, RR, and OT;<br>• Phase 3 portion only **(Period 1 only)**: collect blood sample to test for SARS-CoV-2 antibodies;<br>• Phase 2 portion (all subjects) and Phase 3 portion (immunogenicity subset): collect blood samples for immunogenicity (serology [Nab assay and ELISA] and CMI) assessments; prepare and store these samples until shipment to the analytical laboratories.<br>Any subject who withdraws consent from the study will be asked to undergo Day 386 visit procedures within two weeks of withdrawal, if the subject agrees.<br>**10.1.14.2 Telephone Contact for Phase 3 portion (Period 2 only)**<br>**For Period 2 (all subjects who are not included in the immunogenicity subset) of the Phase 3 portion of the study: the post-vaccination final telephone contact**	site and these subjects will receive a final telephone contact.

#	Section (page #)	Change	Rationale
		**will be performed by blinded site staff members. The following procedures will be performed during the Day 386 phone contact (± 14 days):**<br>• **Review the subject's safety data and ensure all updates on concomitant medication usage and any changes in the subject's health (AEs ongoing from Day 42, SAEs, AESIs, or MAAEs) are recorded appropriately. Confirm the subject has sent images of the memory aid to the clinical site (using the electronic diary);**<br>• **Confirm that the subject has continued to comply with protocol requirements (e.g. no use of prohibited concomitant medications, reporting of any COVID-19 symptoms).**<br>**Any subject who withdraws consent from the study will be asked to participate in the final telephone contact within two weeks of withdrawal, if the subject agrees.**	
48.	Section 10.2.1.1 (Solicited Local and Systemic Adverse Events)	A sentence has been added to the end of the first and second paragraphs as follows:<br>Subjects will be monitored for both solicited local AEs (erythema, swelling, and pain at the injection site) and solicited systemic AEs (fever, headache, fatigue, muscle aches, joint aches, chills, a feeling of general discomfort, swelling in the axilla, and swelling in the neck) from the time of each vaccination through seven days. While the subjects remain in the clinic following vaccine administration (at least 30 minutes), staff will monitor them for solicited local and systemic AEs; after release from the clinic facility, from the evening of each vaccination to the evening of the seventh day after each vaccination, the subject will measure and record the subject's local and systemic AEs in the diary. **The only exception for the monitoring of solicited AEs is: solicited local and systemic AEs (up to seven days after each vaccination) will not be collected from subjects after the cross-over in the Phase 3 portion, however the immediate AEs (30 minutes after each vaccination) will still be collected.**<br>The intensity of the solicited local and systemic AEs will be assessed by the subject, documented in the diary, and graded as: mild (1), moderate (2), severe (3) or	Added a statement to indicate that all solicited AEs are considered related as clarification for previous CBER feedback. Also noted here that solicited AEs will not be collected post-cross-over in the Phase 3 portion.

#	Section (page #)	Change	Rationale
		potentially life threatening (4) (please refer to Table 9). **The causal relationship of all solicited local and systemic AEs will be considered related.**	
49.	Section 10.2.1.2 (Adverse Events and SARS-CoV-2 Positive Reports)	This section has been split into two separate sections and the following updates have been made:<br>Section 10.2.1.2 **Unsolicited** Adverse Events ~~and SARS-CoV-2 Positive Reports~~<br><br>Section 10.2.1.3 **SARS-CoV-2 Positive Reports**<br>COVID-19 cases will be continuously monitored by an unblinded team, including an unblinded Medical Monitor, to identify an adverse imbalance of COVID-19 and/or severe COVID-19 cases between subjects who receive the vaccine or placebo. Such imbalance would trigger either the stopping or alert rules (see Section 13.1.11). **The unblinded team will review COVID-19 and/or severe COVID-19 cases on a ongoing basis to determine if the probability of observing the same or a more extreme case split is 5% or less when the true incidence of severe disease is the same for the CoVLP and placebo recipients. If this criterion is met, then the unblinded team will advise the IDMC members who will review the information along with other available information and determine if the study should continue, be modified, be halted, or be stopped. The monitoring of potential imbalance of severe COVID-19 cases and pausing rule will start at a CoVLP-to-placebo case split of 5:0 for severe disease. The assessment will be performed from Study Day 0 to trial completion using a one-sided binomial test, which will provide probabilities of having an adverse imbalance between the CoVLP and placebo arms given an expected ratio of 5:1 in the Phase 2 portion and a ratio of 1:1 in the Phase 3 portion. In addition, an alert rule will be put in place and will be triggered when the probability of having imbalance in severe cases in the CoVLP arm versus placebo arm is less than 15%.** The unblinded team will contact the IDMC in the event either the stopping or alert rule is met. ~~The IDMC will review the available study data and make a recommendation to the Sponsor.~~ Full details of	Details of the ongoing monitoring of COVID-19 and severe COVID-19 cases have been included.

#	Section (page #)	Change	Rationale
		the VED surveillance process can be found in the Medical Management Plan and IDMC Charter.<br><br>Potential COVID-19 cases will be reviewed by the unblinded medical monitor (part of the unblinded team) during the Phase 2 and Phase 3 portions of the study. Confirmed COVID-19 cases will not be recorded as AEs since these events will be collected for the planned efficacy evaluation. Also, confirmed COVID-19 cases will not be reported according to the process for reporting SAEs, even in the event such case may meet the definition of an SAE. However, any potential COVID-19 case that is not a confirmed event (i.e. does not meet the definition of the efficacy endpoints) and does meet the criteria for an SAE will be reported back to the clinical site of incidence. The unblinded medical monitor and the clinical site Investigator both must report the SAE to Medicago within 24 hours (see Section 13.1.5) of becoming or being made aware that the potential COVID-19 case was not a confirmed event. The Investigator's SAE awareness date will be the date on which the clinical site of incidence receives the SAE back from the unblinded medical monitor. Confirmed COVID-19 cases will be reported in the relevant screen (e.g. COVID-19 symptoms page) in the subject's eCRF within 1 day of reporting by the subject to the clinical site.	
50.	Section 10.2.1.3 (Clinical Laboratory Tests)	The third paragraph has been updated as follows:<br>Other clinical laboratory tests include a test to measure SARS-CoV-2 antibodies at screening for all subjects in all Study Populations in the Phase 2 portion and at Days ~~s~~ 0, ~~201, and~~ **Day** 386 **(in Period 1), and at cross-over** in the Phase 3 portion, and a serum (Phase 2 portion) or urine (Phase 3 portion**; in each period**) pregnancy test at screening and a urine (dipstick or similar) pregnancy test prior to randomization on Day 0 and on Days 21 and 42 for all female subjects of childbearing potential in Study Populations #1 and #3.	Incorporated details regarding the cross-over design in the Phase 3 portion.
51.	Section 10.2.1.5 (Physical Examinations)	The content in this section has been updated as follows:<br>A ~~history- or symptom-directed~~ **limited** physical examination will be performed by the Investigator (or sub-Investigator) as part of screening procedures. **History/symptom-directed physical examinations may be performed at any**	Clarified details regarding the physical

#	Section (page #)	Change	Rationale
		**other study visit if 1) new complaints or concerns are raised by either the study subject or study staff and 2) deemed to be necessary by the Investigator.**	examination to be performed.
52.	Section 10.4.1 (Efficacy Evaluations)	A new paragraph has been added to the end of the section:<br>**For the swabs sent to the central virology laboratory for testing using the virologic method, any swabs with a positive RT-PCR result (positive for the SARS-CoV-2 virus) will undergo additional testing. Direct sequencing will be used to identify the strain of the implicated virus. Information on processing and the method will be provided in the study-specific documentation.**	Added details regarding the method to identify the virus strain from positive swabs.
53.	Section 11.3 (Contraindications for Subsequent Vaccination)	The content in this section has been updated as follows:<br>The following events constitute absolute contraindication to the further administration of the study treatments at Day 21 **in the Phase 2 portion or in each period of the Phase 3 portion**~~; if any of these events occur during the study, the subject will not receive an additional dose of vaccine but will continue with the Day 128 scheduled visit (see Section 10.1.12), the Day 201 scheduled visit (see Section 10.1.13), and the Day 386 scheduled visit (see Section 10.1.14) at the discretion of the Investigator~~:<br>• Pregnancy (see Section 10.2.2);<br>• Clinically-apparent hypersensitivity of any grade that is considered by the site Investigator to be related to the investigational product in response to first vaccine administration;<br>• Have a laboratory-confirmed SARS-CoV-2 infection;<br>• Acute disease within 48 hours prior to vaccination (acute disease is defined as presence of any moderate or severe acute illness with or without a fever);<br>• ~~Suspected or confirmed~~ **Diagnosis of** pIMD **in the context of an AESI and defined according to Section 13.1.3.2.1**;<br>• **In the Phase 2 portion of the study:** Experience a possibly related, probably related, or definitely related SAE or Grade 3 or higher related AE that cannot be clearly attributed to another cause. The Investigator's opinion to administer the second dose of the vaccine will be presented to the IDMC for advice;	Incorporated details regarding each Phase of the study and the cross-over design in the Phase 3 portion. Also, revised the contraindication criteria for additional clarification.

#	Section (page #)	Change	Rationale
		• **In the Phase 3 portion of the study: Experience a possibly related, probably related, or definitely related SAE that cannot be clearly attributed to another cause.**<br><br>**At the discretion of the Investigator, the following event constitutes contraindication to the further administration of the study treatments at Day 21 in the Phase 2 portion or in each period of the Phase 3 portion, or at Day 0 in Period 2 of the Phase 3 portion:**<br><br>• **In the Phase 2 portion of the study:** Discovery of any health condition which, in the Investigator's opinion, may place the subject at increased risk from receipt of further vaccination; or discovery of a change in the subject's status which may render him/her unable to comply with protocol-mandated safety follow-up. If an AE occurred prior to or at the time of the Day 21 scheduled visit, the subject may be discontinued at the discretion of the Investigator or the subject may be vaccinated at a later date, however, within the time window specified in the protocol (see Section 10.1.8.1). The subject must be followed until resolution of the event as with any AE (see Section 13.1.2).<br>• **In the Phase 3 portion of the study: Discovery of any health condition which, in the Investigator's opinion, may place the subject at increased risk from receipt of further vaccination; or discovery of a change in the subject's status which may render him/her unable to comply with protocol-mandated safety follow-up (e.g. if a subject experienced possibly related, probably related, or definitely related Grade 3 AE after first vaccination that lasted more than 72 hours, Investigator should carefully evaluate the risk/benefit balance prior to administering the subsequent dose). If an AE occurred prior to or at the time of the Day 21 scheduled visit, the subject may be discontinued at the discretion of the Investigator or the subject may be vaccinated at a later date, however, within the time window specified in the protocol (see Section 10.1.8.1). The subject must be followed until resolution of the event as with any AE (see Section 13.1.2).**<br><br>**If any of the events listed above (both lists) occur during the study, the subject will not receive an additional dose of vaccine but will continue with the Day 128**	

#	Section (page #)	Change	Rationale
		**scheduled visit (see Section 10.1.12; Phase 2 portion only), the Day 201 scheduled visit (see Section 10.1.13), and the Day 386 scheduled visit/contact (see Section 10.1.14) of the respective portion or period at the discretion of the Investigator.**	
54.	Section 11.4 (Removal of Subjects from Treatment or Assessment)	The third paragraph has been updated as follows:<br>Withdrawal subjects will be those who leave the Phase 2 **portion of the study before Day 386** or **the** Phase 3 portion of the study before Day 386 **of Period 2**, for whatever reason; withdrawal subjects will not be replaced. Any subject who withdraws consent from the ~~Phase 2 or Phase 3 portion of the~~ study will be asked to visit the clinical site **(Phase 2 portion or Period 1 [all subjects] or Period 2 [immunogenicity subset] of the Phase 3 portion) or participate in the final telephone contact (Period 2 [all subjects who are not included in the immunogenicity subset] of the Phase 3 portion)** within two weeks of withdrawal, if the subject agrees. The procedures performed for the final assessment **or telephone contact** will comprise of those for the Day 386 visit**/telephone contact (as applicable),** if permitted by the subject. All withdrawal subjects must be reported to Medicago (and/or its designee). The reason for withdrawal should be documented in the subjects' records and in the appropriate section of the eCRF. The eCRF must be completed up to and including the time of the drop-out/final assessment.	Incorporated details regarding each Phase of the study and the cross-over design in the Phase 3 portion.
55.	Section 12.1.3 (Per Protocol Set)	The third sentence of the first paragraph has been removed as follows:<br>~~The PP set will be the primary analysis population for the efficacy endpoints.~~	This statement has been removed based on MHRA feedback.
56.	Section 12.7.1 (Analysis of Primary Endpoint)	The content in this section has been updated as follows:<br>The primary efficacy endpoint is defined in Section 10.4.2.1.<br>For the Phase 3 portion of the study, once ~~approximately~~ **at least** 160 COVID-19 cases **(≥ 7 days post-second vaccination)** have accumulated during the study, the primary efficacy endpoint of the Phase 3 portion of the study will be evaluated to determine if the vaccine efficacy success criterion has been met. ~~For the efficacy~~	The primary efficacy analysis has been updated to incorporate the cross-over design into the analysis. In addition, the interim efficacy

#	Section (page #)	Change	Rationale
		~~endpoints, VE will be evaluated. An interim analysis will be performed when 80 cases have been collected for an early efficacy assessment.~~<br><br>VE will be evaluated **for any strain before cross-over** as the ~~relative risk~~ **incidence rate ratio** of CoVLP formulation vaccinated versus unvaccinated subjects to develop laboratory-confirmed symptomatic SARS-CoV-2 infection ≥ 7 days after the second vaccination, as per the following formula:<br><br>$$\mathbf{VE=(1\text{-}IRR)=(1\text{-}(\frac{a}{N_1} \div \frac{b}{N_0}))*100\ \%}$$<br><br>**Where:**<br>**IRR= incidence rate ratio**<br>**a= number of subjects with a least one positive COVID-19 case; and**<br>**b= number of subjects with a least one positive COVID-19 case; and**<br>**N_1= person-years for subjects vaccinated with CoVLP; and**<br>**N_0= person-years in unvaccinated subjects.**<br><br>~~VE=(1-RR)=(1-ARV/ARU)*100 %~~<br><br>~~Where:~~<br>~~RR= relative risk~~<br>~~ARV = attack rate in subjects vaccinated with the CoVLP formulation; and~~<br>~~ARU = attack rate in unvaccinated subjects.~~<br><br>**The follow-up time will be calculated from the date of second vaccination + 7 days and censoring will be performed when subjects will experience their first COVID-19 case, the date the database will be freeze for the primary analysis,**	analysis has been removed.

#	Section (page #)	Change	Rationale
		**the subject's cross-over occurs, or at the date of subject completion/withdrawal from the study, whichever occurs first.**<br><br>The VE success criterion for the primary efficacy endpoint, in the Phase 3 portion, is defined as a ≥ 50 % point estimate and a > 30 % lower limit of the ~~appropriately alpha-adjusted confidence interval that maintains overall Type I error at 5%~~ **95 % CI** ~~confidence interval~~. ~~Statistical significance of the primary efficacy endpoint can be achieved at the interim analysis or at the primary analysis.~~<br><br>**Assuming the number of cases in each arm follows a Poisson distribution, then conditioning on the total number of cases and the ratio of person-time of follow up, exact 95 % CIs for the incidence rate ratio IRR, and hence for VE = 1-IRR, are obtained via binomial distribution calculations [Rothman 2008].**<br>~~The CI around RR will be calculated based on score confidence limits [Miettinen 1985, Farrington 1990].~~	
57.	Section 12.7.2 (Analysis of Secondary Endpoints)	The third, fourth, fifth, sixth, seventh, and ninth paragraphs have been updated as follows:<br><br>VE will be evaluated as the ~~relative risk~~ **incidence rate ratio** of CoVLP formulation vaccinated versus unvaccinated subjects to develop laboratory-confirmed symptomatic SARS-CoV-2 infection ≥ 7 days after the second vaccination, as per the formula used for the analysis of the primary efficacy endpoint. In addition, the same formula will be used to evaluate the relative risk of CoVLP formulation vaccinated versus unvaccinated to develop severe COVID-19 disease ≥ 7 days after the second vaccination.<br><br>For the Phase 3 portion of the study, ~~the~~ VE will ~~also~~ be evaluated **prior to the cross-over** for the secondary efficacy endpoints. VE will be evaluated **by strain** as the ~~relative risk of CoVLP formulation vaccinated versus unvaccinated subjects to develop severe COVID-19 disease ≥ 7 days after the second vaccination. In addition, VE will be evaluated as the relative risk~~ **incidence rate ratio** of CoVLP formulation vaccinated versus unvaccinated subjects to develop laboratory-confirmed	The secondary efficacy analysis has been updated to incorporate the cross-over design into the analysis.

#	Section (page #)	Change	Rationale
		symptomatic SARS-CoV-2 infection after **the second vaccination. VE will also be evaluated for any strain as the incidence rate ratio of CoVLP formulation vaccinated versus unvaccinated subjects to develop severe COVID-19 disease ≥ 7 days after the second vaccination. In addition, VE will be evaluated for any strain as the incidence rate ratio of CoVLP formulation vaccinated versus unvaccinated subjects to develop laboratory-confirmed symptomatic SARS-CoV-2 infection after the** first vaccination, after first vaccination and prior to the second vaccination, and after the second vaccination and prior to 7 days post-second vaccination.<br><br>**VE will be evaluated for any strain as the incidence rate ratio of CoVLP formulation vaccinated versus unvaccinated subjects to develop laboratory-confirmed (confirmed by the ELISA method for the N protein) asymptomatic SARS-CoV-2 infection at Day 386 (in Period 1) and at cross-over in 1) all subjects and 2) in subjects who only received a single vaccination. Assuming the number of cases in each arm follows a Poisson distribution, then conditioning on the total number of cases and the ratio of person-time of follow up, exact 95 % CIs for the incidence rate ratio IRR, and hence for VE = 1-IRR, are obtained via binomial distribution calculations [Rothman 2008].**<br><br>The VE success criterion for the secondary efficacy endpoints, in the Phase 3 portion, is defined as > 0 % lower limit of the ~~alpha-adjusted~~ **95 % CI** ~~confidence interval~~.<br><br>~~The CI around RR will be calculated based on score confidence limits.~~<br>The area under the curve of the viral titer curve, based on serial quantitative RT-PCR results, for each positive COVID-19 case will be calculated ~~at the laboratory.~~ **using the SARS-CoV-2 virus load (log 10 copies / mL) [Wathuo 2017].** The point estimates and the corresponding two-sided 95 % CI will be compared between treatment groups using a linear mixed model with testing dates considered as repeated measures.	
58.	Section 12.7.3 (Analysis of	The third paragraph has been updated as follows:	The exploratory efficacy analysis

medicago

#	Section (page #)	Change	Rationale
	Exploratory Endpoints)	For the Phase 3 portion, **the duration of protection and the corresponding 95 % CI will be evaluated at the end of the study for any strain and by strain by assessing the coefficient for the product of the initial vaccine assignment and the time since vaccination using a Cox proportional hazard model parameterized with a linear decay on the log-hazard scale [Fintzi, 2021]. A positive coefficient (θ_2 in Fintzi 2021) with significant p-value ($p < 0.05$) will indicate VE is waning through time. More details will be provided in the SAP.**<br>**VE will also be evaluated by strain as the incidence rate ratio of CoVLP formulation vaccinated versus unvaccinated to develop severe COVID-19 disease ≥ 7 days after the second vaccination.**<br>~~the presence of VE waning will be tested by the linear trend (i.e., the proportional hazards) in the scaled Schoenfeld residuals from the Cox proportional hazard model. Specifically, it will be analyzed as below:~~<br>~~Step 1: Fit a regular Cox proportional hazard model with the treatment group as the main effect, and placebo will be coded as the reference group in the model;~~<br>~~Step 2: Find the scaled Schoenfeld residual from the Cox proportional hazard model in step 1. The scaled Schoenfeld residual has value at all event-time for the main effect (treatment group);~~<br>~~Step 3: Fit a linear regression on the scaled Schoenfeld residuals against event time;~~<br>~~Step 4: The slope and p-value will be output. VE(t)=1-exp(beta(t)), VE(t) waning (smaller with time) is corresponding to the increasing beta(t). Thus, a positive slope with significant p-value (< 0.05) will indicate VE is waning though time.~~	has been updated to incorporate the cross-over design into the analysis.
59.	Section 13.1.2 (Adverse Events)	At the end of the third paragraph a note has been added as follows:<br>**Note: According to the FDA Guidance for Industry: Toxicity Grading Scale for Healthy Adult and Adolescent Volunteers Enrolled in Preventive Vaccine Clinical Trials [FDA 2007]: "*Nonetheless, we believe that categorization or grading of data as outlined in this document is supplementary to and should not replace full and complete data analysis. These guidelines for toxicity grading scales are primarily intended for healthy adult and adolescent volunteers.*" and "*Toxicity grading scales for laboratory abnormalities should consider the local laboratory reference values when the parameter limit values are defined. The characterization***	Incorporated additional information from the FDA guidance into the protocol.

#	Section (page #)	Change	Rationale
		of laboratory parameters among some populations of healthy adults and adolescents may require the exercise of clinical judgment, for example, consideration of the potential for ethnic differences in white blood cell (WBC) counts or gender differences in creatine phosphokinase (CPK) values.”	
60.	Section 13.1.5 (Initial SAE, AESI, and Pregnancy Reports Reporting by the Investigator)	This section has been updated as follows:<br>Details on safety reporting (refer to Section 10.2.1.2 **and 10.2.1.3**) are defined in study-specific documentation, including SAE processing, country-specific reporting requirements, regulatory unblinding, roles and responsibilities of different stake holders, and contact details of personnel responsible for safety reporting. A brief summary of salient information is presented below.<br>All post-vaccination SAEs, AESIs, and pregnancy reports will be reported from the time of receiving the study vaccine on Day 0 through to the end of the study (final scheduled visit). The Investigator (or designee) must report, by e-mail, all SAEs, AESIs **(refer to Section 13.1.3)**, and pregnancy reports whether considered related to the study vaccine or not to Medicago within 24 hours of the Investigator learning of the event. **For the Phase 2 portion of the study, t**~~T~~he Investigator must also complete, sign, and date the paper SAE/**AESI** report form **or pregnancy report form**, and send, via e-mail, a copy to the Medicago safety e-mail address **(listed below)** and the appropriate regional e-mail address within 24 hours of awareness of event:<br>**For the Phase 3 portion of the study, the Investigator must complete the electronic SAE/AESI form in the electronic data capture (EDC) database within 24 hours of awareness of the event following the Electronic Case Report Form Completion Guidelines (eCCG). An automatic email notification will be sent to the Medicago Clinical Safety mailbox after the Investigator submits the electronic SAE/AESI form. If the EDC database is not available, the Investigator must complete, sign, and date the paper SAE/AESI report form, and send, via e-mail, a copy to the Medicago safety e-mail address (listed below)**	Details for reporting SAEs, AESIs, and pregnancy have been further clarified.

#	Section (page #)	Change	Rationale
		**and the appropriate regional e-mail address within 24 hours of awareness of event. If the SAE/AESI paper reporting process is followed, the Investigator will need to enter the SAE /AESI information in the EDC system once available.**<br><br>**For the Phase 3 portion of the study, the Investigator must complete, sign, and date the paper pregnancy report form, and send, via e-mail, a copy to the Medicago clinical safety e-mail address (listed below) and the appropriate regional e-mail address within 24 hours of awareness of event.**<br><br>Medicago Safety e-mail address: Medicago-ClinicalSafety@medicago.com<br><br>Sponsor Safety Contact: [Redacted]<br>[Redacted]<br>[Redacted]<br><br>Serious AEs will be reported to the local (or contract) IRB by the Investigator according to the IRB's policy and procedures.	
61.	Section 13.1.8 (Causal Relationship)	The first paragraph has been updated as follows:<br><br>The Investigator must make the determination of relationship to the study vaccine for each **unsolicited** AE. **The causal relationship of all solicited local and systemic AEs will be considered related.** The Investigator should decide whether, in his/her medical judgment, there is a reasonable possibility that the event may have been caused by the investigational vaccine. If there is any valid reason, even if undetermined or untested, for suspecting a possible cause-and-effect relationship between the investigational vaccine and the occurrence of the AE, then the AE should be considered "definitely related", "probably related", or "possibly related". Otherwise, if no valid reason exists for suggesting a possible relationship, then the AE should be classified as "probably not related" or "definitely not related". The following guidance should be followed:	Added a statement to indicate that all solicited AEs are considered related as clarification for previous CBER feedback.

#	Section (page #)	Change	Rationale
62.	Section 13.1.11 (Safety Review and Stopping Rules)	The third and fifth paragraphs and the third bullet point of the fifth paragraph under the Stopping Rules sub-heading have been updated as follows:<br>If any of the following events occur to the sentinel subjects, the study enrollment and vaccinations **will** ~~may~~ be paused based on IDMC recommendations:<br>The study **will** ~~may~~ be paused based on IDMC recommendations if any of the following events occur:<br>• If an important imbalance in unusual manifestations of COVID-19 or severity of COVID-19 symptoms is observed between the CoVLP and placebo groups ~~(full details provided in the Medical Management Plan and IDMC Charter)~~. **The unblinded team will review COVID-19 and/or severe COVID-19 cases on a ongoing basis to determine if the probability of observing the same or a more extreme case split is 5 % or less when the true incidence of severe disease is the same for the CoVLP and placebo recipients. If this criterion is met, then the unblinded team will advise the IDMC members who will review the information along with other available information and determine if the study should continue, be modified, be halted, or be stopped. The monitoring of potential imbalance of severe COVID-19 cases and pausing rule will start at a CoVLP-to-placebo case split of 5:0 for severe disease. The assessment will be performed from Study Day 0 to trial completion using a one-sided binomial test, which will provide probabilities of having an adverse imbalance between the CoVLP and placebo arms given an expected ratio of 5:1 in the Phase 2 portion and a ratio of 1:1 in the Phase 3 portion. In addition, an alert rule will be put in place and will be triggered when the probability of having imbalance in severe cases in the CoVLP arm versus placebo arm is less than 15 %.**	The stopping rules have been clarified based on MHRA feedback. In addition, details of the ongoing monitoring of COVID-19 and severe COVID-19 cases have been included.
63.	Section 14.1.3 (Preparation and Administration of Study Vaccine)	The third paragraph has been updated as follows:<br>**During the Phase 2 portion and each period of the Phase 3 portion, t**~~T~~he CoVLP formulation will be administered on study Day 0 and Day 21 as an IM injection of 0.5 mL, into the deltoid muscle. For subjects with BMI < 30 kg/m^2, a 23 gauge needle of at least one inch or 2.5 cm in length can be used for vaccination. For	Incorporated details regarding each Phase of the study and the cross-over design

#	Section (page #)	Change	Rationale
		subjects in Study Population #3 with BMI ≥ 30 kg/m^2, a needle of at least 1.5 inches or 3.5 cm in length must be used to ensure intramuscular delivery.	in the Phase 3 portion.
64.	Section 16.1 (Study-Specific Design Considerations)	The third paragraph in this section has been removed:<br>~~In the event a safe and effective treatment or vaccine becomes available for COVID-19 during the Phase 3 portion of the study, consideration will be given to offering the treatment/vaccine to the subjects who were administered placebo after all the COVID-19 cases required for the study have been collected. The consideration will take into account the local regulatory stipulations for the availability of such treatment/vaccine to the local population.~~	Since the Phase 3 portion incorporates a cross-over design, all placebo subjects in the study will receive the CoVLP vaccine.
65.	Section 17.3.1 (Diary and Memory Aid)	The content in this section has been updated as follows:<br>Subjects will be provided with a paper or electronic diary or application in which to record solicited AEs, COVID-19 symptoms, and other safety information. For example, the electronic diary may be configured to transmit images of the memory aid to sites. The electronic diary may also be used for the active weekly surveillance for COVID-19 symptoms and daily symptoms for cases of COVID-19. Subject diaries are considered source documents. **During the first 8 days after each vaccination in Period 2 of the Phase 3 portions, if data entry in the electronic diary is missed for 2 consecutive days by a subject then the subject will receive a follow-up contact (phone, text, or email) to remind him/her to record his/her data in the electronic diary.**<br>Subjects will be provided with memory aids to record information on unsolicited AEs, SAEs, AEs leading to withdrawal, AESIs, and reportable concomitant medications from Day 0 to Day 42, Day 43 to Day 201, and Day 202 to the end of the study (Day 386) **in the Phase 2 portion and each period in the Phase 3 portion**. These memory aids will be collected by the site and are intended to be used by the subjects to help them in reporting this information to site staff during phone contacts and clinical site visits.	Incorporated details regarding each Phase of the study and the cross-over design in the Phase 3 portion. Since the phone contacts on Days 1, 8, 22, and 29 are no longer needed in the Phase 3 portion, subjects will be followed-up if they are not using their electronic diaries.

#	Section (page #)	Change	Rationale
66.	Section 18 (References)	The following updates have been made to the list of references:<br>~~Farrington CP, Manning G. "Test Statistics and Sample Size Formulae for Comparative Binomial Trials with Null Hypothesis of Non-zero Risk Difference or Non-unity Relative Risk." Statistics in Medicine. 1990;9:1447–1454.~~<br>**Fintzi, J. and Follman, D. 2021. Assessing Vaccine Durability in Randomized Trials Following Placebo Crossover: pp.1-28. arXiv:2101.01295 [stat.AP]**<br>~~Miettinen OS, Nurminen MM. "Comparative Analysis of Two Rates." Statistics in Medicine. 1985;4:213–226.~~<br>**Rothman KJ, Greenland S, Lash TL. Modern Epidemiology. 3rd ed. Philadelphia: Lippincott Williams & Wilkins, 2008. Chapter 14.**<br>**Wathuo M, Medley GF, Nokes DJ and Munywoki PK. Quantification and determinants of the amount of respiratory syncytial virus (RSV) shed using real time PCR data from a longitudinal household study [version 2; peer review: 3 approved, 1 approved with reservations]. Wellcome Open Res 2017, 1:27. (https://doi.org/10.12688/wellcomeopenres.10284.2).**	New references have been added to support the revised efficacy analysis.
67.	Section 19.4 (Appendix 4)	The list of potential immune-mediated diseases has been replaced with the previous list.	The potential immune-mediated diseases list has been reverted back to the previous list that presented categorical information.
68.	Section 19.6 (Appendix 6)	The first paragraph of the table for the sample COVID-19 Symptoms page has been updated as follows:<br>*From Day ~~14~~ **0** after your ~~second~~ **first** vaccination to the end of the study **or each period**, record your symptoms in your Memory Aid when you experience one of the following. Call the site as soon as possible. After your visit to the site, you will have to record your COVID-19 symptoms (daily) and collect swabs (every other day) ~~until symptoms resolution~~ **for 2 weeks**.*	The text has been updated for consistency with the protocol and in response to a non-hold clinical

#	Section (page #)	Change	Rationale
			comments from CBER.
69.	Section 19.7 (Appendix 7)	The table listing the meeting schedule for the IDMC reviews and the list of safety data to be reviewed has been updated with additional information for the Phase 2 portion of the study and a second table has been added that includes the details for the Phase 3 portion of the study.	The table presenting the Phase 2 information has been updated to include the latest information and a separate table for Phase 3 has been added.

Table 8 Summary of Changes from Protocol Version 3.0 to Version 4.0 (Effective Date 17 December 2020)

#	Section	Change	Rationale
1.	Synopsis (Study Rationale)<br>AND<br>Section 1.5 (Overall Rationale for the Study)	The first, second, and fourth paragraphs and table have been updated as follows:<br>This Phase 2/3 study is a multi-portion design to confirm that the chosen formulation and dosing regimen of CoVLP (i.e. two doses of 3.75 µg CoVLP adjuvanted with AS03 [hereafter referred to as "CoVLP formulation"] given 21 days apart) has an acceptable immunogenicity and safety profile in adults 18 years of age or older**, including those with significant comorbidities** (Phase 2 portion). The efficacy of this CoVLP formulation in these same Study Populations will be assessed in the Phase 3 portion.<br>The Phase 2 portion is a randomized, observer-blinded, placebo-controlled study to evaluate the CoVLP formulation, compared to placebo, in subjects from the following ~~two~~ **three** Study Populations:	Based on feedback from CBER, the study population of adults with comorbidities can be included in this study and these subjects will only be enrolled in the US. In addition, the sample size for the immunogenicity subset has been updated to incorporate this new study population.

<table>
<tr><th>#</th><th>Section</th><th>Change</th><th>Rationale</th></tr>
<tr><td></td><td></td><td>
<table>
<tr><td>Study Population</td><td>Age and/or Health Category</td><td>Age Range (years)</td></tr>
<tr><td>1</td><td>Healthy adults</td><td>18-64</td></tr>
<tr><td>2</td><td>Healthy elderly adults</td><td>≥ 65</td></tr>
<tr><td>3</td><td>Adults with significant comorbidities*</td><td>≥ 18</td></tr>
</table>
*Subjects to be enrolled in the US only.
The Phase 3 portion is an event-driven, randomized, observer-blinded, placebo-controlled design that will evaluate the efficacy and safety of the CoVLP formulation, compared to placebo, in the same Study Populations #1 - #~~2~~3 presented above. Up to 30 000 subjects will be enrolled in a 1:1 ratio to receive the CoVLP formulation:placebo. Immunogenicity will only be evaluated in a subset of ~~192~~ 288 subjects.
</td><td></td></tr>
<tr><td>2.</td><td>Synopsis (Primary Objectives – Phase 2 Portion and Phase 3 Portion)
AND
Section 2.1 (Primary Objectives)</td><td>
The primary objectives of the Phase 2 and Phase 3 portions have been updated as follows:
Phase 2 portion
The primary objectives of the Phase 2 portion of the study are:
Safety:
• To assess the safety and tolerability of the CoVLP formulation, compared to placebo in each of the Study Populations, up to 21 days after the ~~last~~ second vaccination;
Immunogenicity:
• To assess the immunogenicity of the CoVLP formulation, compared to placebo, up to 21 days after the ~~last~~ second vaccination in each of the Study Populations, as determined by:
  • Neutralizing antibody (Nab) titers induced by the vaccine against the SARS-CoV-2 virus;
  • IFN-γ enzyme-linked immuno spot assay (ELISpot) response induced by the vaccine against the SARS-CoV-2 virus to assess the T helper 1 (Th1) type response.
</td><td>Based on CBER feedback, the objectives have clarified to confirm the assessments will be performed after both vaccinations. In addition, the evaluation of the efficacy endpoint will occur 7 days after the second vaccination.</td></tr>
</table>

#	Section	Change	Rationale
		**Phase 3 portion**<br>The primary objective of the Phase 3 portion of the study is:<br>**Efficacy:**<br>• To evaluate the efficacy of the CoVLP formulation, compared to placebo, in the prevention of laboratory-confirmed symptomatic SARS-CoV-2 infection (virologic method) starting ~~14~~ **7** days after the ~~last~~ **second** vaccination.	
3.	Synopsis (Secondary Objectives – Phase 2 Portion and Phase 3 Portion – Immunogenicity and Safety)<br>AND<br>Section 2.2 (Secondary Objectives – Immunogenicity and Safety)	The following new secondary objectives have been added for Immunogenicity and Safety:<br>Immunogenicity:<br>• **To assess the relative neutralizing antibody response between the healthy adults (Study Population #1) and the healthy elderly adults (Study Population #2) combined and the adults with significant comorbidities (Study Population #3) at 21 days after the second vaccination;**<br>Safety:<br>• **To assess the relative incidence of solicited local and systemic AEs up to 7 days after the first and second vaccinations between the healthy adults (Study Population #1) and the healthy elderly adults (Study Population #2) combined and the adults with significant comorbidities (Study Population #3);**	Based on feedback from CBER, the study population of adults with comorbidities can be included in this study and these subjects will only be enrolled in the US.
4.	Synopsis (Secondary Objectives – Phase 2 Portion and Phase 3 Portion – Immunogenicity)<br>AND	The Immunogenicity secondary objectives for the Phase 2 and the Phase 3 portions have been updated as follows:<br>Phase 2 Portion<br>• To assess the relative neutralizing antibody response between the healthy adults (Study Population #1) and the healthy elderly populations	Based on CBER feedback, the objectives have clarified to confirm the assessments will be performed after both vaccinations. In

#	Section	Change	Rationale
	Section 2.2 (Secondary Objectives – Immunogenicity)	(Study Population #2; each age strata) at 21 days after the ~~last~~ **second** vaccination;<br>• To assess the immunogenicity of the CoVLP formulation, compared to placebo **in each of the Study Populations**, as determined by the:<br>• Persistence of neutralizing antibody titers induced by the vaccine against the SARS-CoV-2 virus at Days 128, 201~~,~~ and Day 386;<br>• IgG (enzyme-linked immunosorbent assay [ELISA]) titers induced by the vaccine against the SARS-CoV-2 virus up to 21 days after the ~~last~~ **second** vaccination and persistence of these antibodies at Day 128, Day 201, and Day 386;<br>• The ratio of neutralizing antibody titers:IgG (ELISA) antibody titers at Day 21, Day 42, Day 128, Day 201, and Day 386;<br>• Interleukin-4 (IL-4) ELISpot response induced by the vaccine against the SARS-CoV-2 virus up to 21 days after the ~~last~~ **second** vaccination and at Day 201 and Day 386;<br>Phase 3 Portion<br>• To assess the immunogenicity of the CoVLP formulation, compared to placebo, in a subset of subjects **in each of the Study Populations**, as determined by:<br>• Neutralizing antibody titers induced by the vaccine against the SARS-CoV-2 virus ~~up to 21 days after the last vaccination and~~ at ~~Day 128,~~ **Day 0 (prior to vaccination; all subjects), Day 21, Day 42 (all subjects),** Day 201, and Day 386;<br>• IgG (ELISA) titers induced by the vaccine against the SARS-CoV-2 virus up to 21 days after the ~~last~~ **second** vaccination and at ~~Day 128,~~ Day 201, and Day 386;<br>• The ratio of neutralizing antibody titers:IgG (ELISA) antibody titers at Day 21, Day 42, ~~Day 128,~~ Day 201 and Day 386;	addition, the Day 128 immunogenicity sample collection has been removed in the Phase 3 portion and instead, the same sample will be collected from all subjects on Day 42.

#	Section	Change	Rationale
		• IFN-γ ELISpot response induced by the vaccine against the SARS-CoV-2 virus up to 21 days after the ~~last~~ **second** vaccination and at Day 201 and Day 386;<br>• IL-4 ELISpot response induced by the vaccine against the SARS-CoV-2 virus up to 21 days after the ~~last~~ **second** vaccination and at Day 201 and Day 386;<br>• Specific cell-mediated immune (CMI) response induced by the vaccine against the SARS-CoV-2 virus up to 21 days after the ~~last~~ **second** vaccination and at Day 201 and Day 386, as measured by the percentage of CD4+ T cells expressing functional markers;	
5.	Synopsis (Secondary Objectives – Phase 2 Portion – Safety)<br>AND<br>Section 2.2 (Secondary Objectives – Safety)	The sub-bullet of the last Safety secondary objective for the Phase 2 portion has been updated as follows:<br>Phase 2 Portion<br>• To assess ~~the~~ safety and tolerability of the CoVLP formulation, compared to placebo **in each of the Study Populations**, as determined by the occurrence(s) of:<br>• Medically attended adverse events (MAAEs), serious adverse events (SAEs), AEs leading to withdrawal, AEs of special interest (AESIs; including vaccine-enhanced disease [VED]), and deaths from 22 days after the ~~last~~ **second** vaccination up to the end of the study;	Based on CBER feedback, the objectives have clarified to confirm the assessments will be performed after the second vaccination.
6.	Synopsis (Secondary Objectives – Phase 2 Portion – Efficacy)<br>AND<br>Section 2.2 (Secondary Objectives – Efficacy)	The first two bullet points for the Phase 2 portion and the three bullet points for the Phase 3 portion of the Efficacy secondary objectives have been updated as follows:<br>Phase 2 Portion<br>• To evaluate the efficacy of the CoVLP formulation, compared to placebo, in the prevention of laboratory-confirmed symptomatic SARS-CoV-2 infection (virologic method) starting ~~14~~ **7** days after the ~~last~~ **second** vaccination;<br>• To evaluate the efficacy of the CoVLP formulation, compared to placebo, in the prevention of severe COVID-19 disease starting ~~14~~ **7**	Based on CBER feedback, the objectives have clarified to confirm the assessments will be performed after the second vaccination. In addition, the evaluation of the efficacy endpoint will

medicago

#	Section	Change	Rationale
		days after the ~~last~~ **second** vaccination. Severe COVID-19 disease will be defined as virologically-confirmed SARS-CoV-2 infection with the occurrence of a new onset of any of the following:<br>Phase 3 Portion<br>• To evaluate the efficacy of the CoVLP formulation, compared to placebo, in the prevention of severe COVID-19 disease starting ~~14~~ **7** days after the ~~last~~ **second** vaccination (see definition of severe COVID-19 disease above);<br>• To assess COVID-19-related symptoms, and the severity of these symptoms **as well as viral shedding**, up until resolution ~~of~~ **in** subjects administered the CoVLP formulation compared to subjects administered the placebo;	occur 7 days after the second vaccination.
7.	Synopsis (Secondary Objectives – Phase 3 Portion – Efficacy)<br>AND<br>Section 2.2 (Secondary Objectives – Efficacy)	The following new secondary objectives have been added for Efficacy in the Phase 3 portion:<br>• **To evaluate the efficacy of the CoVLP formulation, compared to placebo, in the prevention of laboratory-confirmed symptomatic SARS-CoV-2 infection (virologic method) starting after the first vaccination;**<br>• **To evaluate the efficacy of the CoVLP formulation, compared to placebo, in the prevention of laboratory-confirmed symptomatic SARS-CoV-2 infection (virologic method) after the first vaccination and prior to the second vaccination;**<br>• **To evaluate the efficacy of the CoVLP formulation, compared to placebo, in the prevention of laboratory-confirmed symptomatic SARS-CoV-2 infection (virologic method) after the second vaccination and prior to 7 days post-second vaccination;**<br>• **To evaluate the efficacy of the CoVLP formulation, in subjects who only received a single dose compared to placebo, in the prevention of laboratory-confirmed asymptomatic SARS-CoV-2 infection (virologic and serologic methods) at Day 201 and Day 386;**	These objectives have been added to further assess the efficacy of the CoVLP vaccine.

#	Section	Change	Rationale
		• **To assess the duration and intensity of viral shedding after SARS-COV-2 infection in subjects administered the CoVLP formulation compared to subjects administered the placebo;**	
8.	Synopsis (Number of Planned Subjects)<br>AND<br>Section 12.2 (Sample Size Determination)	The first, second, and fourth paragraphs have been updated as follows:<br>Overall, up to a maximum of 30 **918** ~~612~~ subjects are planned for randomization in the Phase 2 and Phase 3 portions in total.<br>In the Phase 2 portion of the study, up to ~~612~~ **306** subjects will be enrolled in a 5:1 ratio to receive CoVLP formulation:placebo in each Study Population **(up to 918 subjects in total)**.<br>Only a subset of approximately ~~192~~ **288** subjects (96 subjects from each Study Population) will be included in the immunogenicity evaluation. These subjects will be enrolled from selected sites in Canada and/or the US that will be defined prior to the start of the Phase 3 portion.	Based on feedback from CBER, the study population of adults with comorbidities can be included in this study and these subjects will only be enrolled in the US. In addition, the sample sizes for the Phase 2 portion and the immunogenicity subset in the Phase 3 portion have been updated to incorporate this new study population.
9.	Synopsis (Sample Size Determination)<br>AND<br>Section 12.2 (Sample Size Determination)	The first and last paragraphs have been updated as follows:<br>The sample size of up to ~~612~~ **918** subjects will serve to confirm vaccine immunogenicity in the ~~two~~ **three** Study Populations and detect sizable differences in rates of adverse events. The sample size is not large enough to detect less frequent or rare adverse reactions. With ~~510~~ **765** subjects receiving the CoVLP formulation, there will be at least 95 % probability of observing an adverse event that occurs as frequently as 1 in ~~170~~ **255** vaccinees.<br>An immunogenicity subset sample size of ~~192~~ **288** subjects is deemed sufficient to provide meaningful statistical assessment of immunologic outcomes.	
10	Synopsis (Study Population)	The section has been updated as follows:<br>Male and female subjects aged 18 years of age and older from ethnically and racially diverse populations will be included in each of the ~~two~~ **three** Study Populations in the study.	Based on feedback from CBER, the study population of adults with comorbidities can be included in this study and these

#	Section	Change	Rationale
			subjects will only be enrolled in the US.
11	Synopsis (Dosage and Administration)<br>AND<br>Section 7 (Dosage and Administration)	The second sentence of the first paragraph and the second and third paragraphs have been updated as follows:<br>Subjects will receive two intramuscular (IM) injections 21 days apart on Days 0 and 21, into the deltoid region of the alternating **(if possible)** arm (each arm will be injected once), of one of the following:<br>• CoVLP formulation;<br>• Placebo.<br>The use of the same dose of ~~the~~ CoVLP ~~formulation~~ for all Study Populations may not be adequate. Subjects in Study Population #1 (healthy adults 18 to 64 years of age) will receive the ~~full~~ standard dose of CoVLP (3.75 µg) adjuvanted with AS03 in an injection volume of 0.5 mL.<br>Study Population**s** #2 **and #3** (elderly adults **or adults with significant comorbidities, respectively**) will receive the ~~full~~ standard dose of CoVLP (3.75 µg) adjuvanted with AS03 in an injection volume of 0.5 mL. In Phase 2, if an acceptable immune response is not achieved at either Day 21 or Day 42, the IDMC may recommend a dose escalation to 7.5 µg CoVLP adjuvanted with AS03 in a volume of 0.5 mL, and the same number of subjects (i.e. 306 subjects **per Study Population**) **in the particular Study Population** will be recruited, using the same enrollment approach (i.e. use of sentinel subjects and IDMC reviews). If the higher dose of the CoVLP formulation achieves an acceptable immune response in the Phase 2 portion, then the same higher dose will be used in **that particular** Study Population ~~#2~~ during the Phase 3 portion of the study.	Based on feedback from CBER, the study population of adults with comorbidities can be included in this study and these subjects will only be enrolled in the US. In addition, it has been clarified that a subject can be vaccinated in the same arm if the alternate arm cannot be used due to a tattoo, scar, or other abnormality in the deltoid region of the arm.
12	Synopsis (Study Design)<br>AND<br>Section 4.1 (Overview of Study Design)	The first four and last two paragraphs and table under the Phase 2 portion sub-header have been updated as follows:<br>The Phase 2 portion is a randomized, observer-blinded, placebo-controlled, design with male and female subjects. Subjects will be enrolled from ~~two~~ **three** Study Populations:	Based on feedback from CBER, the study population of adults with comorbidities can be included in this study and these

<table>
<tr><th>#</th><th>Section</th><th>Change</th><th>Rationale</th></tr>
<tr><td></td><td></td><td>
<table>
<tr><td>Study Population</td><td>Age and/or Health Category</td><td>Age Range (years)</td></tr>
<tr><td>1</td><td>Healthy adults</td><td>18-64</td></tr>
<tr><td>2</td><td>Healthy elderly adults</td><td>≥ 65</td></tr>
<tr><td>**3**</td><td>**Adults with significant comorbidities***</td><td>**≥ 18**</td></tr>
</table>
***Subjects to be enrolled in the US only.**
In each of the ~~two~~ **three** Study Populations, 306 subjects will be randomized 5:1 and will receive the same CoVLP formulation or placebo. Subjects will receive two IM injections 21 days apart on Days 0 and 21, into the deltoid region of the alternating **(if possible)** arm (each arm will be injected once), of one of the following:
• CoVLP formulation;
• Placebo.
All subjects will begin enrollment at the same time. Enrollment of elderly adult subjects (Study Population #2) will be stratified by age in an approximately 2:1 ratio of 65 to 74 years of age and 75 years of age and older, respectively. **Enrollment of subjects in Study Population #3 will include adults with co-existing conditions such as, but not limited to, obesity or being overweight, cardiovascular diseases, hyperlipidemia, chronic kidney disease or related disorders, chronic respiratory disease, pre-diabetes and diabetes, treatment-controlled HIV, and psychiatric disorders.**
The IDMC will review unblinded safety data **(i.e. solicited and unsolicited safety data including laboratory safety data collected up to 7 days after each vaccination)** for the first 10 sentinel subjects after each dose in **all Study Populations, with the exception of** Study Population #~~2~~**1**, according to the details outlined in the IDMC Charter. All sentinel subjects will be dosed at least 30 minutes apart.
Subjects will be screened up to 14 days in advance of the first vaccine administration and will demonstrate a satisfactory baseline medical assessment by history, general physical examination, haematological,
</td><td>subjects will only be enrolled in the US. In addition, it has been clarified that a subject can be vaccinated in the same arm if the alternate arm cannot be used due to a tattoo, scar, or other abnormality in the deltoid region of the arm. Also, the unblinded safety data to be reviewed by the IDMC was clarified as requested by CBER. Also, clarified that COVID-19 symptoms will be monitored after the first vaccination on Day 0 until the end of the study, as per feedback from CBER.</td></tr>
</table>

#	Section	Change	Rationale
		biochemical, and serological analysis. Although a test for SARS-CoV-2 antibodies will be performed at screening, both seronegative and seropositive subjects will be enrolled. On Day 0 and Day 21, vaccine administration will occur. Phone contacts will be made one day and eight days after each vaccine administration, specifically for review of the subject's safety and concomitant medication data. Visits to the Investigator site will occur 3 days after each vaccine administration for key safety assessments, and 21 days after each vaccine administration for key safety and immunogenicity assessments. Subjects will return to the Investigator site on Day 128, Day 201, and Day 386 (3-month, 6-month, and 12-month safety follow-ups and immunogenicity assessments after the last vaccine administration).<br>Active and passive surveillance will be conducted in the study from ~~14 days after the last vaccination~~ **the day of the first vaccination (Day 0, post-vaccination)** until 386 days. For passive surveillance, subjects will be instructed to report symptoms meeting the definition of new COVID-19 disease. For active surveillance, subjects will be contacted once per week via the subject's preferred method (e.g. phone, text, email, electronic diary), and asked about symptoms of COVID-19 disease.	
13	Synopsis (Study Design)<br>AND<br>Section 4.1 (Overview of Study Design)	The first two and last two paragraphs and table under the Phase 3 portion sub-header have been updated as follows:<br>The Phase 3 portion is a randomized, observer-blinded, placebo-controlled, efficacy, safety, immunogenicity (in a subset of subjects), multicenter design with male and female subjects. Similar to the Phase 2 portion, subjects will be enrolled from ~~two~~ **three** Study Populations:<br>Study Population / Age and/or Health Category / Age Range (years):<br>1 / Healthy adults / 18-64<br>2 / Healthy elderly adults / ≥ 65<br>**3 / Adults with significant comorbidities* / ≥ 18**<br>***Subjects to be enrolled in the US only.**	Based on feedback from CBER, the study population of adults with comorbidities can be included in this study and these subjects will only be enrolled in the US. In addition, it has been clarified that a subject can be vaccinated in the same arm if the alternate arm cannot be used due to a

#	Section	Change	Rationale
		Study Population~~s~~ #2 will be roughly stratified by age in the same manner. The overall goal of the Phase 3 portion of this study is to enroll approximately 30 000 subjects who are generally representative of the overall target population in terms of racial diversity with enrichment for those at highest risk for serious COVID-19 disease (i.e. the elderly **and those with significant comorbidities**).<br><br>Subjects will be screened and will demonstrate a satisfactory baseline medical assessment by history and general physical examination. On Day 0 and Day 21, vaccine administration will occur. Phone contacts will be made one day and eight days after each vaccine administration, specifically for review of the subject's safety and concomitant medication data. **A v**~~V~~isit to the Investigator site will occur on **Day 21 (immunogenicity subset only) and Day 42 (all subjects)** ~~21 days after each vaccine administration~~ for immunogenicity assessments ~~in the immunogenicity subset only~~. Subjects will return to the Investigator site on ~~Day 128 (immunogenicity subset only),~~ Day 201 and Day 386 (6-month and 12-month safety follow-ups and ~~3-month, 6-month, and 12-month~~ immunogenicity assessments [immunogenicity subset only] after the last vaccine administration).<br><br>Active and passive surveillance will be conducted in the study from ~~14 days after the last vaccination~~ **the day of the first vaccination (Day 0, post-vaccination)** until 386 days. For passive surveillance, subjects will be instructed to report symptoms meeting the definition of new COVID-19 disease. For active surveillance, subjects will be contacted once per week via the subject's preferred method (e.g. phone, text, email, electronic diary), and asked about symptoms of COVID-19 disease. In addition, to monitor asymptomatic infection, subjects will perform a test for SARS-CoV-2 N antibodies on Day 0, Day 201, and Day 386.	tattoo, scar, or other abnormality in the deltoid region of the arm. Also, clarified that COVID-19 symptoms will be monitored after the first vaccination on Day 0 until the end of the study, as per feedback from CBER. Also, the Day 128 immunogenicity sample was removed and replaced by the Day 42 immunogenicity sample to be collected in all subjects.
14	Synopsis (Immunogenicity Evaluations)	The content in this section has been updated as follows:<br>Immunogenicity will be evaluated in all subjects in the Phase 2 portion and in a subset of ~~192~~ **288** subjects in the Phase 3 portion of the study across the	Based on feedback from CBER, the study population of adults

#	Section	Change	Rationale
	AND<br>Section 10.3.1 (Immunogenicity Evaluations)	Study Populations with age distribution similar to the Phase 2 portion. Immunologic outcomes will include the humoral immune response (neutralization assay and IgG ELISA) and the CMI response induced in subjects on Day 0, Day 21, Day 42, Day 128 (neutralization assay and IgG ELISA only **for Phase 2 portion only**), Day 201, and Day 386.	with comorbidities can be included in this study and these subjects will only be enrolled in the US. In addition, the sample size for the immunogenicity subset has been updated to incorporate this new study population.
15	Synopsis (Efficacy Evaluations)<br>AND<br>Section 10.4.1 (Efficacy Evaluations)	The content in this section has been updated as follows:<br>Following the first vaccination, subjects will be asked to report symptoms associated with COVID-19 from ~~14 days after the last vaccination~~ **the day of the first vaccination (Day 0, post-vaccination)** until the end of the Phase 2 portion or the Phase 3 portion of the study (through active or passive surveillance). COVID-19 will be defined as the presence of a laboratory-confirmed (virologic method) SARS-CoV-2 infection with the occurrence of a new onset of one or more of the following symptoms that persist(s) for or reoccur(s) after a period of at least 12 hours:<br>• Fever or chills;<br>• Cough;<br>• Shortness of breath or difficulty breathing;<br>• Fatigue;<br>• Muscle or body aches;<br>• Headache;<br>• New loss of taste or smell;<br>• Sore throat;<br>• Congestion or runny nose;	Clarified the start of the surveillance period to begin right after the first vaccination. Included a mitigation strategy for swabs that need to the central laboratory and the receipt of results may take longer than expected. Also, included a mitigation strategy for swabs that need to the central laboratory and the receipt of results may take longer than expected.

#	Section	Change	Rationale
		• Nausea or vomiting;<br>• Diarrhea.<br>Subjects who report any symptoms associated with COVID-19 after the start of active and passive surveillance will be requested to visit the Investigator site to provide nasal or nasopharyngeal (NP) swabs (2 per subject per event) to test for ~~COVID-19~~ **SARS-CoV-2 infection**. During the visit, the Investigator will confirm whether nasal or NP swabs should be collected. If the subject cannot visit the Investigator site, then the subject may receive a visit at home to collect the nasal/NP swabs samples or be provided with self-administered nasal~~/NP~~ swabs for sample collection.<br>The swabs will be collected within 72 hours (preferably within 48 hours) of the reporting of a COVID-19-associated symptom. If swabs cannot be collected within 72 hours after reporting of the COVID-19-associated symptom, nasal or NP swabs should be collected at the next available opportunity for the subject to visit the Investigator site. One of the swabs will be submitted for analysis using a virologic method while the other swab will be kept as a back-up. All subjects with virologically-confirmed SARS-COV-2 infection will be asked to keep a detailed daily record of their symptoms and provide serial (every other day) self-administered nasal~~/NP/buccal~~ swabs for testing, using a virologic method, until symptoms resolution or **until two consecutive negative results are obtained**.<br>**To mitigate the risk of long delays in shipping samples to the central virology laboratory in the Netherlands, an additional rapid turn-around virologic test will be performed 'locally' using an approved NP, nasal or saliva assay (i.e., local laboratory or point-of-care, nucleic acid or antigen-detection). The purpose of this supplementary assay is to provide a preliminary diagnosis and permit appropriate symptom-recording and sample collection as quickly as possible.**	
16	Synopsis (Primary Endpoints - Safety)<br>AND	The following Safety primary endpoint has been updated as follows:<br>In the Phase 2 portion, the primary endpoints are:	Based on feedback from CBER, all

#	Section	Change	Rationale
	Section 10.2.3.1 (Primary Endpoints)	• Occurrence~~,~~ **and** intensity~~, and relationship to vaccination~~ of solicited local and systemic AEs (for seven days following each vaccine administration);	solicited AEs should be considered related.
17	Synopsis (Primary Endpoints - Efficacy)<br>AND<br>Section 10.4.2.1 (Primary Endpoint)	The following Efficacy primary endpoint has been updated as follows:<br>In the Phase 3 portion, the primary endpoint~~s~~ ~~are~~ **is**:<br>• First occurrence, in a subject, of laboratory-confirmed (virologic method) symptomatic SARS-CoV-2 infection (≥ ~~14~~ **7** days post~~-last~~ **second** vaccination).	Based on CBER feedback, the objectives have clarified to confirm the assessments will be performed after the second vaccination. In addition, the evaluation of the efficacy endpoint will occur 7 days after the second vaccination.
18	Synopsis (Secondary Endpoints – Safety)<br>AND<br>Section 10.2.3.2 (Secondary Endpoints)	The following new Safety secondary endpoint has been added in the Phase 2 portion:<br>In the Phase 2 portion, the secondary safety endpoints are:<br>• **Relative occurrence of solicited local and systemic AEs by intensity grades for seven days following each vaccine administration between the healthy adults (Study Population #1) and the healthy elderly adults (Study Population #2) combined and the adults with significant comorbidities (Study Population #3);**	Based on feedback from CBER, the study population of adults with comorbidities can be included in this study and these subjects will only be enrolled in the US.
19	Synopsis (Secondary Endpoints – Safety)<br>AND<br>Section 10.2.3.2 (Secondary Endpoints)	The second bullet point of the Safety secondary endpoints in the Phase 2 portion and the second bullet point of the Safety secondary endpoints in the Phase 3 portion have been updated as follows:<br>Phase 2 portion<br>• Relative occurrence of solicited local and systemic AEs **by intensity grades** for seven days following each vaccine administration between the healthy adults (Study Population #1) and each healthy elderly population (Study Population #2);	Based on feedback from CBER, all solicited AEs should be considered related.

#	Section	Change	Rationale
		Phase 3 portion<br>• Occurrence~~,~~ **and** intensity~~, and relationship to vaccination~~ of solicited local and systemic AEs (for seven days following each vaccine administration);	
20	Synopsis (Secondary Endpoints – Immunogenicity)<br>AND<br>Section 10.3.2.2 (Secondary Endpoints)	The Immunogenicity secondary endpoints in the Phase 2 portion and the Phase 3 portion have been updated as follows:<br>Phase 2 portion<br>• Relative neutralizing antibody response between the healthy adults (Study Population #1) and the healthy elderly populations (Study Population #2; each age strata) induced at 21 days after the ~~last~~ **second** vaccination will be analyzed using the following parameter: GMT;<br>Phase 3 portion<br>• In a subset of subjects, Nab antibody response induced in each Study Population against the SARS-CoV-2 virus on Days 0 **(all subjects)**, 21, 42 **(all subjects)**, ~~128~~, 201, and 386 analyzed, using the following parameters: GMT, SC rate, and GMFR;<br>• In a subset of subjects, specific antibody response induced in each Study Population against the SARS-CoV-2 virus measured by the total IgG levels on Days 0, 21, 42, ~~128,~~ 201, and 386, and analyzed using the following parameters: GMT, SC rate, and GMFR;<br>• In a subset of subjects, the ratio of neutralizing antibody titers:IgG (ELISA) antibody titers at Day 21, Day 42, ~~Day 128,~~ Day 201, and Day 386;<br>• In a subset of subjects, specific CMI response induced in each Study Population against the SARS-CoV-2 virus up to 21 days after the ~~last~~ **second** vaccination and at Day 201 and Day 386, as measured by the percentage of CD4+ T cells expressing functional markers;	Based on CBER feedback, the objectives have clarified to confirm the assessments will be performed after both vaccinations. In addition, the Day 128 immunogenicity sample collection has been removed in the Phase 3 portion and instead, the same sample will be collected from all subjects on Day 42.
21	Synopsis (Secondary Endpoints – Immunogenicity)<br>AND	The following new Immunogenicity secondary endpoint has been added in the Phase 2 portion:<br>In the Phase 2 portion, the secondary immunogenicity endpoints are:<br>• **Relative neutralizing antibody response between the healthy adults (Study Population #1) and the healthy elderly adults (Study Population**	Based on feedback from CBER, the study population of adults with comorbidities can be included in

#	Section	Change	Rationale
	Section 10.3.2.2 (Secondary Endpoints)	**#2) combined and the adults with significant comorbidities (Study Population #3) induced at 21 days after the second vaccination will be analyzed using the following parameter: GMT;**	this study and these subjects will only be enrolled in the US.
22	Synopsis (Secondary Endpoints – Efficacy)<br>AND<br>Section 10.4.2.2 (Secondary Endpoints)	The two bullet points of the Efficacy secondary endpoints in the Phase 2 portion and the second bullet point of the Efficacy secondary endpoints in the Phase 3 portion have been updated as follows:<br>Phase 2 portion<br>• First occurrence, in a subject, of laboratory-confirmed (virologic method) symptomatic SARS-CoV-2 infection (≥~~14~~ **7** days post-~~last~~ **second** vaccination);<br>• Occurrences of severe COVID-19 disease (≥~~14~~ **7** days post-~~last~~ **second** vaccination). Refer to the definition of severe COVID-19 disease in the Secondary Objectives section above.<br>Phase 3 portion<br>• Occurrences of severe COVID-19 disease (≥~~14~~ **7** days post-~~last~~ **second** vaccination). Refer to the definition of severe COVID-19 disease in the Secondary Objectives section above;	Based on CBER feedback, the objectives have clarified to confirm the assessments will be performed after the second vaccination. In addition, the evaluation of the efficacy endpoint will occur 7 days after the second vaccination.
23	Synopsis (Secondary Endpoints – Efficacy)<br>AND<br>Section 10.4.2.2 (Secondary Endpoints)	The following new Efficacy secondary endpoints have been added in the Phase 3 portion:<br>Phase 3 portion<br>• **First occurrence, in a subject, of laboratory-confirmed (virologic method) symptomatic SARS-CoV-2 infection (≥ first vaccination);**<br>• **First occurrence, in a subject, of laboratory-confirmed (virologic method) symptomatic SARS-CoV-2 infection (≥ first vaccination and < second vaccination);**<br>• **First occurrence, in a subject, of laboratory-confirmed (virologic method) symptomatic SARS-CoV-2 infection (≥ second vaccination and < 7 days post-second vaccination);**	These endpoints have been added to further assess the efficacy of the CoVLP vaccine.

#	Section	Change	Rationale
		• **Occurrences of laboratory-confirmed (confirmed by the ELISA method for the N protein) asymptomatic SARS-CoV-2 infection at Day 201 and Day 386 in subjects who only received a single vaccination;**<br>• **Duration and intensity of viral shedding after SARS-CoV-2 infection;**	
24	Synopsis (Exploratory Endpoint – Efficacy)<br>AND<br>Section 10.4.2.3 (Exploratory Endpoints)	The bullet point in the Efficacy exploratory endpoint for the Phase 3 portion has been updated as follows:<br>• The slope estimate of the weighted Schoenfeld residuals against event time for the evaluation of vaccine efficacy determined from the first occurrence, in a subject, of laboratory-confirmed (virologic method) symptomatic SARS-CoV-2 infection (≥~~14~~ **7** days post-~~last~~ **second** vaccination);	Based on CBER feedback, the objectives have clarified to confirm the assessments will be performed after the second vaccination. In addition, the evaluation of the efficacy endpoint will occur 7 days after the second vaccination.
25	Synopsis (Safety Review)<br>AND<br>Section 13.1.11 (Safety Review and Stopping Rules)	The two paragraphs have been updated as follows:<br>During the study, the IDMC will review safety data to ensure the ongoing safety of all the subjects in the study. During the Phase 2 portion, the safety data **(i.e. solicited and unsolicited safety data including laboratory safety data collected up to 7 days after each vaccination)** for the first 10 sentinel subjects after each dose in **all Study Populations, with the exception of** Study Population #~~2~~**1**, will be reviewed by the IDMC before permitting enrollment of the remaining subjects, according to the details outlined in the IDMC Charter. Additional information about the planned and ad hoc IDMC review meetings is also available in the IDMC ~~c~~**C**harter.<br>During the Phase 2 portion, the IDMC will review the safety data collected up to Day 28 from all the subjects in each Study Population to confirm that each Study Population can be included in the Phase 3 portion of the study. **These safety data, as well as the Day 21 and Day 42 immunogenicity data, will be provided to Regulatory Authority(ies) as required for review prior to**	Based on feedback from CBER, details regarding the unblinded safety data to be reviewed by the IDMC were clarified. In addition, a confirmatory statement that the Phase 2 results for each study population will be provided to Regulatory Authorities prior to enrolling subjects in

#	Section	Change	Rationale
		**enrolling subjects from their jurisdictions in each Study Population into the Phase 3 portion of the study.**	the Phase 3 portion was included as requested by CBER.
26	Synopsis (Statistical Methods – Populations)	The second paragraph in this section has been updated as follows:<br>In each of the Study Populations for the Phase 2 and Phase 3 portions:<br>• All safety analyses will be performed using the SAS;<br>• All efficacy analyses will be performed using both the PP set and the ITT set;<br>• All immunogenicity analyses will be performed using both the IPP set and the immunogenicity ITT set;<br>• In **the** Phase 2 **portion**, analyses using the PP **set** and IPP **set** will be considered the primary analyses for efficacy and immunogenicity objectives respectively.<br>• In **the** Phase 3 **portion**, analyses using the ITT **set** will be considered the primary analyses for efficacy objectives. Analyses using the IPP **set** will be considered the primary analyses for immunogenicity objectives.	Details of this section were clarified for use of consistent terminology.
27	Synopsis (Statistical Methods – Phase 2 Portion Day 21 and Day 42 Immunogenicity Data Analysis)<br><br>AND<br><br>Section 12.3 (Day 21 and Day 42 Immunogenicity Data	The first two paragraphs have been updated as follows:<br>The unblinded statistical team will analyze the immunogenicity data after Day 21 and Day 42 in each of the Study Populations. The data analysis will involve selected individuals from the unblinded statistical team and Medicago who will be unblinded (as described in Blinding section). The Day 21 analysis in Study Population #1 and the Day 42 analysis in Study Population**s** #2 **and #3** will confirm whether an acceptable immune response has been induced in each Study Population prior to the initiation of the Phase 3 portion of the study (refer to the Phase 3 Portion Initiation section below for details of the criteria that will be used), without having to wait until after the end of the 386 day follow-up period for study completion. **These immunogenicity data, as well as the Day 28 safety data, will be provided to Regulatory Authority(ies) as required for review prior to enrolling subjects from**	Based on feedback from CBER, the study population of adults with comorbidities can be included in this study and these subjects will only be enrolled in the US. In addition, a confirmatory statement that the Phase 2 results for each study population will be provided to

#	Section	Change	Rationale
	Analysis and Interim Efficacy Analyses)	**their jurisdictions in each Study Population into the Phase 3 portion of the study.**<br><br>If the Day 42 analysis in Study Population #2 **or #3** confirms that an acceptable response has been induced (refer to Section 12.4 for details of the criteria that will be used at Day 42), the **particular** Study Population ~~#2~~ will be permitted to proceed into the Phase 3 portion of the study. However, if at Day 42, the immune response for Study Population #2 **or #3** is still relatively weak compared to the healthy adult Day 42 responses, an ad hoc IDMC meeting will be convened to consider changes to the Protocol to permit alternate vaccination strategies in **the particular** Study Population ~~#2~~.	Regulatory Authorities prior to enrolling subjects in the Phase 3 portion was included as requested by CBER.
28	Synopsis (Statistical Methods – Phase 3 Portion Initiation)<br><br>AND<br><br>Section 12.4 (Initiation of the Phase 3 Portion)	The section has been updated as follows:<br><br>The start of the Phase 3 portion of the study will be determined independently for each Study Population and will be based on the outcomes of the following two assessments during the Phase 2 portion of the study:<br>• Day 21 Data Analysis of the immunogenicity data in Study Population #1 (described above);<br>• Day 42 Data Analysis of the immunogenicity data in Study Population #2 **and Study Population #3** (described above);<br>• Day 28 Safety Review in Study Population #1**,** ~~and~~ Study Population #2**, and Study Population #3** (described in the Safety Review section above).<br><br>Study Population #1: If the safety and immunogenicity profiles of the CoVLP formulation observed in the Phase 1 study are acceptable in the Phase 2 portion, this group will proceed into the Phase 3 portion. Another key outcome will be the absence of a significant safety signal as assessed by the IDMC.<br><br>Study Populations #2 **and #3**: If the safety and immunogenicity profiles of the CoVLP formulation in ~~this~~ **these** population**s** are acceptable, entry of Study Population #2 **or #3** into the Phase 3 portion will be based on both the absence	Based on feedback from CBER, the study population of adults with comorbidities can be included in this study and these subjects will only be enrolled in the US. In addition, the criteria for moving into the Phase 3 portion has been updated to include this study population.

#	Section	Change	Rationale
		of an important safety signal and demonstration of an acceptable immune response as assessed by the IDMC. The criteria for acceptability will be:<br>• No statistically significant difference in the Nab responses at Day 42 between Study Population #1 and Study Population #2;<br>• **No statistically significant difference in the Nab responses at Day 42 between Study Population #1 and Study Population #2 combined and Study Populations #3;**<br>• No significant higher incidence of solicited local and systemic AEs reported 7 days after each vaccine administration in Study Population #2 compared to Study Population #1;<br>• **No significant higher incidence of solicited local and systemic AEs reported 7 days after each vaccine administration in Study Population #3 compared to Study Population #1 and Study Population #2 combined.**<br>**For Study Population #2,** ~~T~~the same criteria will be applied to the comparisons of the two age strata for Study Population #2 versus Study Population #1. In the event of a significant difference between Study Population #1 and one of the age stratum for Study Population #2, the next highest dose level **(7.5 µg CoVLP)** that can be administered to this population will be assessed in the same number of subjects (as planned for the age stratum in the Phase 2 portion).	
29	Synopsis (Statistical Methods – Efficacy Analyses)<br>AND<br>Section 12.7.1 (Analysis of Primary Endpoint)	The second and fourth paragraph have been updated and a new paragraph has been added as follows:<br>VE will be evaluated as the relative risk of CoVLP vaccinated versus unvaccinated subjects to develop laboratory-confirmed symptomatic SARS-CoV-2 infection ≥ ~~14~~ **7** days after the ~~last~~ **second** vaccination, as per the following formula:<br>VE will also be calculated for the secondary and exploratory efficacy endpoints. The VE success criterion for the secondary efficacy endpoints, in	Based on CBER feedback, the objectives have clarified to confirm the assessments will be performed after the second vaccination. In addition, the analysis of the new efficacy

#	Section	Change	Rationale
		the Phase 3 portion, is defined as > 0 % lower limit of the **alpha-adjusted confidence interval** ~~95%~~ confidence interval.<br>**To assess the duration and intensity of viral shedding after SARS-COV-2 infection, point estimates and two-sided 95 % CI will be calculated. The area under the curve of the viral titer curve, based on serial quantitative RT-PCR results, will be compared between treatment groups using a linear mixed model with testing dates considered as repeated measures. The peak viral shedding assessed by the lowest CT value and the number of days to negative test will be compared between treatment groups using an ANOVA.**	endpoints has been added.
30	Table 1 and Table 2 (Time and Events Schedules: General Information for the Phase 2 Portion and the Phase 3 Portion)	The passive and active surveillance statements in the tables have been updated as follows:<br>Table 1<br>Passive Surveillance: Subjects will be instructed to contact the study site if they experience symptoms of COVID-19 from ~~14 days after the last vaccination~~ **the day of the first vaccination (Day 0, post-vaccination)** until the end of the Phase 2 portion of the study.<br>Active Surveillance: Between ~~14 days after the last vaccination~~ **the day of the first vaccination (Day 0, post-vaccination)** and the end of the Phase 2 portion of the study, the subjects will be contacted once per week via the method most likely to be successful (e.g. subject's preferred method [phone, text, email, electronic diary]), and asked about symptoms of COVID-19.<br>Table 2<br>Passive Surveillance: Subjects will be instructed to contact the study site if they experience symptoms of COVID-19 from ~~14 days after the last vaccination~~ **the day of the first vaccination (Day 0, post-vaccination)** until the end of the Phase 3 portion of the study.<br>Active Surveillance: Between ~~14 days after the last vaccination~~ **the day of the first vaccination (Day 0, post-vaccination)** and the end of the Phase 3 portion of the study, the subjects will be contacted once per week via the	Clarified that symptoms of COVID-19 should be reported after vaccination on Day 0 in phase 2 of the study.

#	Section	Change	Rationale
		method most likely to be successful (e.g. subject's preferred method [phone, text, email, electronic diary]), and asked about symptoms of COVID-19.	
31	Table 1 and Table 2 (Time and Events Schedules: General Information for the Phase 2 Portion and the Phase 3 Portion)	Footnote #4 has been updated as follows:<br>Phase 2 portion<br>[4] In all females of childbearing potential in Study Population #1 **and #3**; it will be tested in serum at screening and in urine at Days 0, 21, and 42.<br>Phase 3 portion<br>[4] In all females of childbearing potential in Study Populations #1 **and #3**; it will be tested in urine at Days 0, 21, and 42.	Based on feedback from CBER, the study population of adults with comorbidities can be included in this study and these subjects will only be enrolled in the US.
32	Table 2 (Time and Events Schedule: General Information for the Phase 3 Portion)	Table 2 has been updated as follows:<br>The column for Day 128 (± 14) has been removed and all occurrences of Day 128. In addition, Day 201 and Day 386 visits have been renumbered to visit 4 and visit 5.<br>Footnote 10 has been updated and added to the Day 42 visit and the row "Immunogenicity – Serology (Nab assay and ELISA):<br>[10] ~~This visit is only for subjects included in the immunogenicity subset~~ **The immunogenicity sample at Day 42 will be collected from all subjects**.<br>Footnote 11 has been added to the row "Test for SARS-CoV-2 N antibodies":<br>**[11] The Day 0 sample collected, prior to vaccination, from subjects not included in the immunogenicity subset will also be used for the immunogenicity assessment (Nab assay and ELISA).**	The Day 128 visit has been removed from the Phase 3 portion of the study.
33	Section 1.1 (Background)	The fourth and fifth paragraphs have been updated as follows:<br>~~Since there is currently no effective treatment available for coronavirus infections, s~~Significant efforts have been made to the development of vaccines and therapeutic drugs. **Currently, the therapy Remdesivir (Canada and the USA) and convalescent plasma (USA) have been approved to treat COVID-19, with specific conditions for use and for specific patients.** Most ~~of~~ therapeutic approaches that are currently being tested are based on repurposing the therapeutic agents previously designed for other applications.	Content has been updated with current information.

#	Section	Change	Rationale
		These agents can either directly target the virus replication cycle or aim boosting the innate antiviral immune responses or attenuating the damage induced by dysregulated inflammatory responses [Ahn 2020, Stebbing 2020].<br>Vaccination is the most effective strategy for preventing infectious diseases since it appears to be more cost-effective than therapeutic drugs and reduces morbidity and mortality without causing long-lasting undesirable effects. Although three human coronaviruses emerged worldwide over the past two decades, causing considerable threat to global health, **only the Pfizer-BioNTech COVID-19 (Canada and the US) vaccine has been approved for the prevention of COVID-19 in people who are 16 years of age and older** ~~there is still no approved vaccines for human coronaviruses~~. Research groups around the world are accelerating the development of COVID-19 vaccines using various approaches [Ahn 2020, Lu 2020].	
34	Section 1.1.2 (Adults with Comorbidities)	A new sub-section with information about adults with comorbidities has been added:<br>**People of any age with serious underlying medical conditions such as diabetes and cardiovascular or lung disease are at a greater risk of severe COVID-19. Those individuals with underlying uncontrolled medical conditions such as diabetes, hypertension, lung, liver, and kidney disease, cancer patients on chemotherapy, smokers, transplant recipients, and patients taking steroids represent a vulnerable population who are not only at a higher risk of developing severe illness but are also at an increased risk of death [Li 2020, Sanyaolu 2020]. Hypertension (odds ratio [OR]: 2.29), diabetes (OR: 2.47), chronic obstructive pulmonary disease (COPD; OR: 5.97), cardiovascular disease (OR: 2.93), and cerebrovascular disease (OR: 3.89) were independent risk factors associated with COVID-19 [Wang 2020]. A strong association of worse clinical outcomes in COVID-19 disease with obesity, even in the absence of any other comorbidity, has been reported [Korakas 2020]. The in-hospital death OR was found as high as 2.70 in patients with coronary artery disease, 2.48 in patients with congestive heart failure, 1.95 in those**	Based on feedback from CBER, the study population of adults with comorbidities can be included in this study and these subjects will only be enrolled in the US.

#	Section	Change	Rationale
		**with cardiac arrhythmias, 2.96 in patients with COPD, and 1.79 for current smokers [Mehra 2020]. Overall, the risk of severe COVID-19 requiring admission to intensive care unit, invasive ventilation, or leading to death was found to be 1.79 fold greater among patients with at least one comorbidity and 2.59 fold greater among patients with two or more comorbidities compared to generally healthy subjects [Guan 2020a].**<br>**The need for an effective COVID-19 vaccine for people with underlying health conditions is obvious; however, vaccination strategies for these individuals may require some adjustment to achieve the desirable immune responses and vaccine effectiveness. Many comorbidities can adversely impact the response to vaccination, either due to general deterioration of the immune reactivity or as the result of pharmaceutical management of the underlying disease [Kwetkat 2020, Zimmermann 2019]. For example, patients with COPD generally respond poorly to influenza vaccination with significantly lower antibody titers and seroconversion rates: 43% in COPD compared to 90 % of healthy participants [Nath 2014]. Congestive heart failure patients develop a significantly lower T cell immune response to live-attenuated varicella-zoster vaccine and inactivated influenza vaccine than those without a cardiac impairment [Vardeny 2010, Verschoor 2017]. The effectiveness of 23-valent polysaccharide pneumococcal vaccine (PPV23) against invasive pneumococcal disease in individuals aged ≥65 years falls from 45 % in the healthy elderly to 25 % in high-risk immunocompetent patients with chronic respiratory/heart/liver/renal disease; diabetes mellitus; cerebrospinal fluid leaks; cochlear implants and is further reduced to 13 % in patients with underlying health conditions thought to be associated with an immune impairment (asplenia/splenic dysfunction, malignancy or an immunosuppressive drug treatment) [Djennad 2019].**	
35	Section 1.1.3 (Risk:Benefit in the	The first paragraph has been updated as follows:<br>Although the focus of this protocol is on the administration of the CoVLP formulation in ~~healthy~~ **the** adult and elderly populations, **including those**	Based on feedback from CBER, the study population of adults with comorbidities

#	Section	Change	Rationale
	Different Study Populations)	**with comorbidities,** the intent of Medicago's **long-term** clinical development program is to develop a CoVLP formulation that is safe and effective for use in a much larger range of the population, including ~~adults with comorbidities,~~ children**,** ~~and~~ adolescents, and pregnant women.	can be included in this study and these subjects will only be enrolled in the US.
36	Section 4.1 (Overview of Study Design)	Figure 1 has been updated to include text referring to adults with comorbidities.	Based on feedback from CBER, the study population of adults with comorbidities can be included in this study and these subjects will only be enrolled in the US.
37	Section 5.1 (Inclusion Criteria)	The first paragraph has been updated as follows:<br>Subjects must meet all of the following inclusion criteria at the Screening (Visit 1) and/or Vaccination (Visit 2) visits **(these two visits are combined into one visit in the Phase 3 portion)** to be eligible for participation in this study; no protocol waivers are allowed. All Investigator assessment-based judgements must be carefully and fully documented in the source documents:	Clarified the visits for the Phase 2 and Phase 3 portions.
38	Section 5.1 (Inclusion Criteria)	The inclusion criterion #2 has been updated as follows:<br>At the Screening visit (Visit 1), male and female subjects must be**:**<br>• Study Populations #1: 18 to 64 (has not yet had his/her 65th birthday) years of age, inclusive;<br>• Study Population #2: 65 years of age or older;<br>• **Study Population #3: 18 years of age or older;**	Based on feedback from CBER, the study population of adults with comorbidities can be included in this study and these subjects will only be enrolled in the US.
39	Section 5.1 (Inclusion Criteria)	The inclusion criterion #3 has been updated as follows:<br>At Screening (Visit 1) and Vaccination (Visit 2), subject must have a body mass index (BMI) of:<br>• **Study Populations #1 and #2:** $\geq$ 18.5 and < 30 kg/m^2;	BMI requirements have been specified according to study population.

#	Section	Change	Rationale
40	Section 5.1 (Inclusion Criteria)	The inclusion criterion #5 has been updated as follows:<br>Study Population #1: Subjects must be in good general health prior to study participation, with no clinically relevant abnormalities that could jeopardize subject safety or interfere with study assessments, as determined by medical history, physical examination, and vital signs. Investigator discretion will be permitted with this inclusion criterion;<br>**All regions except Canada: Note: Subjects with a pre-existing chronic disease will be allowed to participate if the disease is stable and, according to the Investigator's judgment that must be documented in the source documents, the condition is unlikely to confound the results of the study or pose additional risk to the subject by participating in the study. Stable disease is generally defined as no new onset or exacerbation of pre-existing chronic disease three months prior to vaccination. Based on the Investigator's judgment, a subject with more recent stabilization of a disease could also be eligible.**	Based on feedback from CBER, the additional text regarding the Investigator discretion is acceptable.
41	Section 5.1 (Inclusion Criteria)	The first paragraph for inclusion criterion #6 has been updated as follows:<br>Study Populations #1 **and #3**: Female subjects of childbearing potential must have a negative serum pregnancy test result at Screening (Visit 1 **for the Phase 2 portion**) and/**or** a negative urine pregnancy test result at Vaccination (Visit 2 f**or the Phase 2 portion; Visit 1 for the Phase 3 portion**):	Based on feedback from CBER, the study population of adults with comorbidities can be included in this study and these subjects will only be enrolled in the US.
42	Section 5.1 (Inclusion Criteria)	The Study Populations have been updated and 2 new criteria have been added as follows for inclusion criteria #7:<br>Study Populations #1 **and #3**:<br>• **All regions except the US: Vasectomised partner, provided that this partner is the sole sexual partner of the study participant and that the vasectomised partner has received a medical assessment of the surgical success;**	Identified two more highly effective methods of contraception that can be considered in the study.

#	Section	Change	Rationale
		• **Bilateral tubal ligation.**	
43	Section 5.1 (Inclusion Criteria)	The inclusion criterion #8 has been updated as follows:<br>Study Population #2: Subjects must be non-institutionalized (e.g. not living in rehabilitation centres or old-age homes; living in an elderly community is acceptable) and have no acute or evolving medical problems prior to study participation and no clinically relevant abnormalities that could jeopardize subject safety or interfere with study assessments, as assessed by the Principal Investigator or sub-Investigator (thereafter referred as Investigator) and determined by medical history, physical examination, serology (only for the Phase 2 portion), clinical chemistry and haematology tests (only for the Phase 2 portion), urinalysis (only for the Phase 2 portion), and vital signs. Investigator discretion will be permitted with this inclusion criterion.<br>**All regions except Canada: Note: Subjects with a pre-existing chronic disease will be allowed to participate if the disease is stable and, according to the Investigator's judgment that must be documented in the source documents, the condition is unlikely to confound the results of the study or pose additional risk to the subject by participating in the study. Stable disease is generally defined as no new onset or exacerbation of pre-existing chronic disease three months prior to vaccination. Based on the Investigator's judgment and documented in source documentation, a subject with more recent stabilization of a disease could also be eligible;**	Based on feedback from CBER, the additional text regarding the Investigator discretion is acceptable.
44	Section 5.1 (Inclusion Criteria)	The inclusion criteria #9 has been added:<br>**Study Population #3: Subjects must have one or more co-morbid conditions that puts them at higher risk for severe COVID-19 disease. These comorbidities include but are not limited to obesity, hypertension, type 1 or type 2 diabetes, chronic obstructive pulmonary disease (COPD), cardiovascular diseases, chronic kidney diseases, or be immunocompromised persons (e.g., treatment-controlled HIV infection, organ transplant recipients, or patients receiving cancer chemotherapy). Investigator discretion will be permitted with this inclusion criterion.**	Based on feedback from CBER, the study population of adults with comorbidities can be included in this study and these subjects will only be enrolled in the US.

#	Section	Change	Rationale
45	Section 5.2 (Exclusion Criteria)	The first paragraph has been updated as follows:<br>Subjects who meet any of the following criteria at the Screening (Visit 1) and/or Vaccination (Visit 2) visits **(these two visits are combined into one visit in the Phase 3 portion)** will not be eligible for participation in this study; no protocol waivers are allowed. All Investigator assessment-based judgements must be thoroughly documented in the source documents:	Clarified that the screening visit and the vaccination visit are the same in the Phase 3 portion.
46	Section 5.2 (Exclusion Criteria)	The Study Populations have been added as follows to the exclusion criteria #1 to #5:<br>**Study Populations #1 and #2**:	Study populations for the exclusion criteria have been clarified.
47	Section 5.2 (Exclusion Criteria)	A new exclusion criterion has been added after exclusion criterion #5:<br>**Study Population #3: Acute disease defined as presence of any moderate or severe acute illness with or without a fever within 48 hours prior to the Screening (Visit 1) and/or Vaccination visit (Visit 2);**	Based on feedback from CBER, the study population of adults with comorbidities can be included in this study and these subjects will only be enrolled in the US.
48	Section 5.2 (Exclusion Criteria)	A new exclusion criterion has been added after exclusion criterion #8:<br>**History of virologically-confirmed COVID-19;**	Added the exclusion criterion to ensure subjects who have had COVID-19 are not included in the study.
49	Section 5.2 (Exclusion Criteria)	Exclusion criterion #14 has been updated as follows:<br>**Personal or f**~~F~~amily history of narcolepsy;	Expanded exclusion criterion to include personal history of the subjects.
50	Section 5.2 (Exclusion Criteria)	The Study Populations have been updated as follows for exclusion criteria #16:	Based on feedback from CBER, the study

#	Section	Change	Rationale
		Study Populations #1 **and #3**:	population of adults with comorbidities can be included in this study and these subjects will only be enrolled in the US.
51	Section 5.4 (Prohibited Therapy)	The Study Populations have been added as follows to the list item #3:<br>**Study Populations #1 and #2**:	Study populations for the prohibited therapy have been clarified.
52	Section 8.1 (Management of Samples)	A new paragraph has been added at the end of the section:<br>**To mitigate the risk of long delays in shipping samples to the central virology laboratory in the Netherlands, an additional rapid turn-around virologic test will be performed 'locally' using an approved NP or nasal assay (i.e., local laboratory or point-of-care, nucleic acid or antigen-detection). The purpose of this supplementary assay is to provide a preliminary diagnosis and permit appropriate symptom-recording and sample collection as quickly as possible.**	Included a mitigation strategy for swabs that need to the central laboratory and the receipt of results may take longer than expected.
53	Section 8.2 (Collection of Samples)	This section has been updated as follows:<br>In the Phase 3 portion of the study, all subjects will have **25** ~~15~~ mL of blood drawn up to the end of the study (Day 386) and only a subset of subjects included in the immunogenicity analyses will have an additional blood volume of up to approximately **250** ~~260~~ mL drawn up to the end of the study (Day 386) (Table 7).<br>Table 7 – has been updated to remove text related to Day 128<br>Urine samples will be collected at screening and Days 3, 21, and 24 during the Phase 2 portion of the study.<br>In the Phase 2 portion of the study, pregnancy testing will be performed for all females of childbearing potential (in Study Population**s** #1 **and #3**). A serum pregnancy test will be performed during screening. A urine (dipstick or similar) pregnancy test will be performed at Day 0 prior to randomization and	Based on feedback from CBER, the study population of adults with comorbidities can be included in this study and these subjects will only be enrolled in the US.<br>In addition, the Day 128 visit has been removed from the Phase 3 portion of the study.

#	Section	Change	Rationale
		at Days 21 and 42. In Study Populations #1 **and #3**, females of child bearing potential must have negative serum pregnancy test result from screening and urine pregnancy test result from Day 0 available prior to vaccination.<br>In the Phase 3 portion of the study, pregnancy testing will be performed for all females of childbearing potential (in Study Populations #1 **and #3**). A urine (dipstick or similar) pregnancy test will be performed at screening and at Day 0 prior to randomization and at Days 21 and 42. In Study Populations #1 **and #3**, females of child bearing potential must have negative urine pregnancy test results from screening and Day 0 available prior to vaccination.<br>Nasal or NP swabs will be collected from any subject who reports any symptoms associated with COVID-19 after the start of active and passive surveillance, at the discretion of the site Investigator. Two swabs will be collected per subject per event (one from each nare). The swabs will be collected within 72 hours (preferably within 48 hours) of the reporting of a COVID-19-associated symptom. If swabs cannot be collected within 72 hours after reporting of the COVID-19-associated symptom, nasal or NP swabs should be collected at the next available opportunity for the subject to visit the Investigator site.<br>Additional serial nasal~~/NP/buccal~~ swabs (self-collected) and blood sampling may be requested of subjects with virologically-confirmed COVID-19 infection.	
54	Section 10.1.3 (Visit 1 for Phase 2 Portion and Phase 3 Portion)	The Study Populations have been added for the secondary sub-bullet points of the fifth bullet point as follows:<br>• **Study Populations #1 and #2:**	Study populations have been clarified.
55	Section 10.1.4.2 (Vaccination)	A sentence has been added before the last sentence of the paragraph as follows:<br>**For subjects in Study Population #3 with BMI ≥ 30 kg/m², a needle of at least 1.5 inches or 3.5 cm in length must be used to ensure intramuscular delivery.**	Based on feedback from CBER, the study population of adults with comorbidities can be included in this study and these

#	Section	Change	Rationale
			subjects will only be enrolled in the US.
56	Section 10.1.7.3 (Post-Dose 2 Vaccination)	The bullet point has been updated as follows:<br>• The study vaccine should be administered IM into the deltoid muscle of the alternate **(if possible)** arm (i.e. the arm not used for the previous dose administration). Record the administration, left or right arm, in the subject source documents.	Clarified that a subject can be vaccinated in the same arm if the alternate arm cannot be used due to a tattoo, scar, or other abnormality in the deltoid region of the arm.
57	Section 10.1.10 (Surveillance for COVID-19 Cases)	The first paragraph and the fourth main bullet point have been updated as follows:<br>From **the day of the first vaccination (Day 0, post vaccination)** ~~14 days following the last vaccination~~, passive and active surveillance will be performed until the end of the Phase 2 or the Phase 3 portion of the study:<br>• COVID-19 cases that start on or after **the day of the first vaccination (Day 0, post vaccination)** ~~14 days after the last vaccination~~ will be followed up. This follow-up will be conducted via the planned active surveillance contacts. All follow-up information should be collected, regardless of whether nasal or NP swabs could be obtained. As part of this follow up, the following information will be needed:	Clarified that symptoms of COVID-19 should be reported after vaccination on Day 0 in each phase of the study.
58	Section 10.1.11 (Day 42)	A sentence has been added to the beginning of the first paragraph and the fourth and last bullet points have been updated as follows:<br>**For subjects in quarantine, these procedures will be performed by blinded trained medical staff and may be at the subject's home (or place of quarantine).**<br>~~Phase 2 portion (all subjects) and Phase 3 portion (immunogenicity subset):~~ ~~c~~**C**ollect blood samples for immunogenicity (serology [Nab assay and ELISA**;**	Clarified that the Day 42 procedures for subjects in quarantine do not need to be performed at the site.

#	Section	Change	Rationale
		**all subjects**] and CMI **[all subjects in Phase 2 and immunogenicity subset only in Phase 3]**) assessments; prepare and store these samples until shipment to the analytical laboratory;<br>Provide appointments (date and time) for the next telephone contacts and the next planned visit to the clinical site (Day 128 **for the Phase 2 portion and Day 201 for the Phase 3 portion**)	
59	Section 10.1.12 (Day 128)	The third and fourth bullet points have been updated as follows:<br>~~Phase 2 portion only: p~~**P**erform a vital signs measurement, including resting BP, HR, RR, and OT;<br>~~Phase 2 portion (all subjects) and Phase 3 portion (immunogenicity subset): c~~**C**ollect blood samples for immunogenicity (serology [Nab assay and ELISA]) assessments; prepare and store these samples until shipment to the analytical laboratories;	The Day 128 visit has been removed from the Phase 3 portion of the study.
60	Section 10.2.1.1 (Solicited Local and Systemic Adverse Events)	The second paragraph has been modified as follows:<br>The intensity of the solicited local and systemic AEs will be assessed by the subject, documented in the diary, and graded as: mild (1), moderate (2), severe (3) or potentially life threatening (4) (please refer to **Error! Reference source not found.**). ~~The causal relationship with the study vaccine of the solicited local and systemic AEs will be assessed by the Investigator (definitely not related, probably not related, possibly related, probably related or definitely related) and documented in the Investigator section of the diary; see Section~~ ~~**Error! Reference source not found.**~~ ~~for a definition of these causal relationships.~~	Based on feedback from CBER, all solicited AEs should be considered related.
61	Section 10.2.1.3 (Clinical Laboratory Tests)	The Study Population numbers have been updated as follows for the third sentence of the first paragraph and the second last paragraph:<br>In addition, serum or urine samples from all females of childbearing potential in Study Population**s** #1 **and #3** for pregnancy testing will be collected.<br>AND<br>Other clinical laboratory tests include a test to measure SARS-CoV-2 antibodies at screening for all subjects in all Study Populations in the Phase 2	Based on feedback from CBER, the study population of adults with comorbidities can be included in this study and these

#	Section	Change	Rationale
		portion and at Days 0, 201, and 386 in the Phase 3 portion, and a serum (Phase 2 portion) or urine (Phase 3 portion) pregnancy test at screening and a urine (dipstick or similar) pregnancy test prior to randomization on Day 0 and on Days 21 and 42 for all female subjects of childbearing potential in Study Population**s** #1 **and #3**.	subjects will only be enrolled in the US.
62	Section 11.3 (Contraindications for Subsequent Vaccination)	The second bullet point has been updated as follows:<br>• ~~Anaphylaxis~~ **Clinically-apparent hypersensitivity of any grade that is considered by the site Investigator to be related to the investigational product** in response to first vaccine administration;	Based on CBER feedback, this contraindication for subsequent vaccination was revised.
63	Section 12.1.3 (Per Protocol Set)	The first sentence of the first paragraph has been updated as follows:<br>The PP set will consist of a subset of the ITT set who participated in the study until the end of the surveillance period in the Phase 2 or Phase 3 portions of the study; had no major protocol deviations related to subject eligibility, the ability to develop a valid immune response, prohibited medication use, or the efficacy analyses; and who received **both doses of** either the CoVLP formulation or placebo.	Based on CBER feedback, the PP set for the efficacy analysis will include subjects who receive both vaccinations.
64	Section 12.5.2 (Analysis of Secondary Endpoints)	The second paragraph has been updated and a new paragraph has been added after the second paragraph as follows:<br>The incidence of solicited local and systemic AEs **by intensity grades** reported 7 days after each vaccine administration will be compared between Study Population #2 and Study Population #1 using **Chi-square** ~~Fisher's exact~~ tests. Each age strata in Study Population #2 will be compared separately to the Study Population #1.<br>**The incidence of solicited local and systemic AEs reported 7 days after each vaccine administration will be compared between Study Population #3 and Study Population #1 and Study Population #2 combined using Chi-square test.**	Based on feedback from CBER, the study population of adults with comorbidities can be included in this study and these subjects will only be enrolled in the US.

#	Section	Change	Rationale
65	Section 12.6.1 (Analysis of Primary Endpoints)	The second bullet point for the second paragraph has been updated as follows:<br>• SC rate (Day 0, Day 21, and Day 42): The point estimates and the corresponding two-sided 95 % CI for subjects achieving SC by treatment group will be calculated and reported. **The SC rate is defined as the proportion of subjects achieving SC in the analysis set:**<br>• **For subjects with detectable Nab titer at Day 0 (i.e. baseline Nab titer ≥10): a ≥ 4-fold increase in Nab titers between Day 0 and Day 21 and Day 42, respectively;**<br>• **For subjects with undetectable Nab titer at Day 0 (i.e. baseline Nab titer < 10): Nab titer of ≥ 40 on Day 21 and Day 42, respectively;**	Based on CBER feedback, the seroconversion rate was defined.
66	Section 12.6.2 (Analysis of Secondary Endpoints)	A new paragraph has been added after the fifth paragraph and the second last paragraph has been updated as follows:<br>**Nab GMT at Day 42 between Study Population #1 and Study Population #2 combined and Study Population #3 will be compared using the analysis of variance (ANOVA) model.**<br>For the Phase 3 portion, the same immunogenicity analyses will be performed as described for the primary and secondary immunogenicity endpoints for the Phase 2 portion of the study **(with the exception of the analysis on Day 128).**	Based on feedback from CBER, the study population of adults with comorbidities can be included in this study and these subjects will only be enrolled in the US.
67	Section 12.7.2 (Analysis of Secondary Endpoints)	The third and fourth paragraphs have been updated as follows:<br>VE will be evaluated as the relative risk of CoVLP formulation vaccinated versus unvaccinated subjects to develop laboratory-confirmed symptomatic SARS-CoV-2 infection ≥ ~~14~~ **7** days after the ~~last~~ **second** vaccination, as per the formula used for the analysis of the primary efficacy endpoint. In addition, the same formula will be used to evaluate the relative risk of CoVLP formulation vaccinated versus unvaccinated to develop severe COVID-19 disease ≥ ~~14~~ **7** days after the ~~last~~ **second** vaccination.<br>For the Phase 3 portion of the study, the VE will also be evaluated for the secondary efficacy endpoints. VE will be evaluated as the relative risk of CoVLP formulation vaccinated versus unvaccinated subjects to develop	Based on CBER feedback, the objectives have clarified to confirm the assessments will be performed after the second vaccination.<br>In addition, the analyses of the new efficacy endpoints have been included.

#	Section	Change	Rationale
		severe COVID-19 disease ≥ ~~14~~ **7** days after the ~~last~~ **second** vaccination. **In addition, VE will be evaluated as the relative risk of CoVLP formulation vaccinated versus unvaccinated subjects to develop laboratory-confirmed symptomatic SARS-CoV-2 infection after the first vaccination, after first vaccination and prior to the second vaccination, and after the second vaccination and prior to 7 days post-second vaccination.**<br>The VE success criterion for the secondary efficacy endpoints, in the Phase 3 portion, is defined as > 0 % lower limit of the **alpha-adjusted confidence interval** ~~95 %~~.<br>The ~~95%~~ CI around RR will be calculated based on score confidence limits.<br>For the Phase 3 portion, the occurrences of COVID-19-related symptoms and their severity will be summarized by treatment using descriptive statistics. The occurrence of unusual manifestations of COVID-19 as well as the severity of COVID-19 symptoms will also be used to assess imbalance between the CoVLP and placebo groups as a possible indication of enhanced disease (full details provided in the Medical ~~Monitoring~~ **Management** Plan and IDMC Charter).<br>**The area under the curve of the viral titer curve, based on serial quantitative RT-PCR results, for each positive COVID-19 case will be calculated at the laboratory. The point estimates and the corresponding two-sided 95 % CI will be compared between treatment groups using a linear mixed model with testing dates considered as repeated measures.**<br>**The peak viral shedding will be assessed by retrieving the lowest CT value observed at any time at the laboratory for each positive COVID-19 case. Lowest CT values and the corresponding two-sided 95 % CI will be compared between treatment groups using an ANOVA.**<br>**For each positive COVID-19 case, the number of days to negative test from the start of the symptoms and the first positive test and the corresponding two-sided 95 % CI will be compared between treatment groups using an ANOVA.**	

#	Section	Change	Rationale
68	Section 13.1.11 (Safety Review and Stopping Rules)	The second paragraph and second bullet points for the third and fourth paragraphs under the Stopping Rules heading have been updated as follows:<br>For sentinel subjects in Study Populations #2 **and #3** during the Phase 2 portion of the study:<br>If any of the following events occur to the sentinel subjects, the study enrollment and vaccinations may be paused based on IDMC recommendations:<br>• Any SAE in a subject for which causality cannot be attributed to another cause;<br>• Two (2) or more subjects who receive the CoVLP formulation experience ~~a~~ **the same or** similar AE ~~(i.e. the same Preferred Terms based on MedDRA] coding)~~:<br>◦ Experience the same **or similar** Grade 3 or higher solicited local AE or systemic AE **which began** within 7 days after administration of the vaccine;<br>◦ Experience the same **or similar** Grade 3 ~~AE~~ or higher unsolicited AE **(including symptoms, signs or laboratory safety AEs) that is judged anything but unrelated to the vaccine**~~, vital sign, and/or clinical laboratory abnormality, in the same Preferred terms based on the MedDRA coding, and is considered related to the vaccine~~.<br>After vaccination of all sentinel subjects in Phase 2, the stopping rules for the remainder of the Phase 2 and the Phase 3 portions of the study:<br>The study may be paused based on IDMC recommendations if any of the following events occur:<br>• Any subject experiences an SAE after administration of the vaccine that is considered related to vaccine;	Based on feedback from CBER, the study population of adults with comorbidities can be included in this study and these subjects will only be enrolled in the US. In addition, CBER requested specific changes to the stopping rules criteria.

#	Section	Change	Rationale
		• If 5 % or more subjects who receive the CoVLP formulation experience ~~a~~ **the same or** similar AE ~~(i.e. the same Preferred Terms based on MedDRA] coding)~~:<br>◦ Experience the same **or similar** Grade 3 or higher solicited local AE or systemic AE, **which began** within 7 days after administration of the vaccine;<br>◦ Experience the same **or similar** Grade 3 ~~AE~~ or higher unsolicited AE **(including symptoms, signs or laboratory safety AEs) that is judged anything but unrelated to the vaccine**~~, vital sign, and/or clinical laboratory abnormality, in the same Preferred terms based on the MedDRA coding, after administration of the vaccine and is considered related to the vaccine~~;<br>• If an important imbalance in unusual manifestations of COVID-19 or severity of COVID-19 symptoms is observed between the CoVLP and placebo groups (full details provided in the Medical **Management** ~~Monitoring~~ Plan and IDMC Charter).	
69	14.1.1 (Study Vaccine Composition)	The first and second paragraphs have been updated as follows:<br>The CoVLP vaccine is a sterile transparent to opalescent, colorless to yellowish or greenish liquid suspension. The CoVLP vaccine will be supplied in 0.35 mL single dose vials **in the Phase 2 portion and in 3.1 ml multidose vials (10 doses/vial) in the Phase 3 portion**. The S glycoprotein concentration in the vaccine will be 15 µg/mL for a final dosage of 3.75 µg.<br>AS03 is an established effective adjuvant licensed for Arepanrix™ H1N1 and Arepanrix™ H5N1 (in Canada). The adjuvant is a whitish to yellowish homogeneous milky liquid emulsion and it will be provided in the original sterile **3.0 ml multidose (10 doses/vial)** vial container received from the manufacturer (GlaxoSmithKline). A 0.25 mL dose of AS03 represents one human dose.	Clarified details about the investigational product.
70	14.1.2 (Placebo)	The first paragraph has been updated as follows:	Clarified details about the placebo.

#	Section	Change	Rationale
		The control product (placebo) will be composed of a phosphate-buffered saline (PBS) solution (100 mM $NaKPO_4$, 150 mM NaCl) and 0.01 % polysorbate 80 at pH 7.4. The control product will be provided in **6.2 mL multidose** vials.	
71	Section 14.1.3 (Preparation and Administration of Study Vaccine)	A sentence has been added to the end of the third paragraph as follows:<br>**For subjects in Study Population #3 with BMI ≥ 30 kg/m^2, a needle of at least 1.5 inches or 3.5 cm in length must be used to ensure intramuscular delivery.**	Based on feedback from CBER, the study population of adults with comorbidities can be included in this study and these subjects will only be enrolled in the US.
72	Section 18 (References)	The following references have been added to the protocol:<br>**Djennad A, Ramsay ME, Pebody R, Fry NK, Sheppard C, Ladhani SN, Andrews NJ. Effectiveness of 23-Valent Polysaccharide Pneumococcal Vaccine and Changes in Invasive Pneumococcal Disease Incidence from 2000 to 2017 in Those Aged 65 and Over in England and Wales. EClinicalMedicine. 2019 Jan 2;6:42-50. doi: 10.1016/j.eclinm.2018.12.007.**<br>**Guan WJ, Liang WH, Zhao Y, Liang HR, Chen ZS, Li YM, Liu XQ, Chen RC, Tang CL, Wang T, Ou CQ, Li L, Chen PY, Sang L, Wang W, Li JF, Li CC, Ou LM, Cheng B, Xiong S, Ni ZY, Xiang J, Hu Y, Liu L, Shan H, Lei CL, Peng YX, Wei L, Liu Y, Hu YH, Peng P, Wang JM, Liu JY, Chen Z, Li G, Zheng ZJ, Qiu SQ, Luo J, Ye CJ, Zhu SY, Cheng LL, Ye F, Li SY, Zheng JP, Zhang NF, Zhong NS, He JX; China Medical Treatment Expert Group for COVID-19. Comorbidity and its impact on 1590 patients with COVID-19 in China: a nationwide analysis. Eur Respir J. 2020a May 14;55(5):2000547. doi: 10.1183/13993003.00547-2020.**<br>**Korakas E, Ikonomidis I, Kousathana F, Balampanis K, Kountouri A, Raptis A, Palaiodimou L, Kokkinos A, Lambadiari V. Obesity and**	Based on feedback from CBER, the study population of adults with comorbidities can be included in this study and these subjects will only be enrolled in the US. The background information discussing this population has been added and these references are needed in the protocol.

#	Section	Change	Rationale
		**COVID-19: immune and metabolic derangement as a possible link to adverse clinical outcomes. Am J Physiol Endocrinol Metab. 2020 Jul 1;319(1):E105-E109.**<br>**Kwetkat A, Heppner HJ. Comorbidities in the Elderly and Their Possible Influence on Vaccine Response. Interdiscip Top Gerontol Geriatr. 2020;43:73-85. doi: 10.1159/000504491.**<br>**Mehra MR, Desai SS, Kuy S, Henry TD, Patel AN. Cardiovascular Disease, Drug Therapy, and Mortality in Covid-19. N Engl J Med. 2020 Jun 18;382(25):e102. doi: 10.1056/NEJMoa2007621.**<br>**Nath KD, Burel JG, Shankar V, Pritchard AL, Towers M, Looke D, Davies JM, Upham JW. Clinical factors associated with the humoral immune response to influenza vaccination in chronic obstructive pulmonary disease. Int J Chron Obstruct Pulmon Dis. 2014;9:51-6. doi: 10.2147/COPD.S53590.**<br>**Sanyaolu A, Okorie C, Marinkovic A, Patidar R, Younis K, Desai P, Hosein Z, Padda I, Mangat J, Altaf M. Comorbidity and its Impact on Patients with COVID-19. SN Compr. Clin. Med. 2020. doi.org/10.1007/s42399-020-00363-4.**<br>**Vardeny O, Moran JJ, Sweitzer NK, Johnson MR, Hayney MS. Decreased T-cell responses to influenza vaccination in patients with heart failure. Pharmacotherapy. 2010 Jan;30(1):10-6. doi: 10.1592/phco.30.1.10.**<br>**Verschoor CP, Lelic A, Parsons R, Evelegh C, Bramson JL, Johnstone J, Loeb MB, Bowdish DME. Serum C-Reactive Protein and Congestive Heart Failure as Significant Predictors of Herpes Zoster Vaccine Response in Elderly Nursing Home Residents. J Infect Dis. 2017 Jul 15;216(2):191-197. doi: 10.1093/infdis/jix257.**<br>**Wang B, Li R, Lu Z, Huang Y. Does comorbidity increase the risk of patients with COVID-19: evidence from meta-analysis. Aging (Albany NY). 2020 Apr 8;12(7):6049-6057. doi: 10.18632/aging.103000.**	

#	Section	Change	Rationale
		**Zimmermann P, Curtis N. Factors That Influence the Immune Response to Vaccination. Clin Microbiol Rev. 2019 Mar 13;32(2):e00084-18. doi: 10.1128/CMR.00084-18.**	
73	Section 19.4 (Appendix 4)	The list of potential immune-mediated diseases has been replaced with a new list.	The potential immune-mediated diseases list was updated with the latest information available.
74	Section 19.7 (Appendix 7)	A table listing the meeting schedule for the IDMC reviews and the list of safety data to be reviewed has been added to the protocol.	Based on feedback from CBER, details regarding when the IDMC will meet and the safety data to be reviewed by the IDMC have been provided.

Table 9 Summary of Changes from Protocol Version 2.0 to Version 3.0 (Effective Date 06 November 2020)

#	Section	Change	Rationale
1	Title Page<br>AND<br>Signatures<br>AND<br>Investigator Agreement<br>AND<br>Synopsis (Protocol Title)	The title of the protocol has been updated as follows:<br>A Randomized, Observer-Blind, Placebo-Controlled, Phase 2/3 Study to Assess the Safety, Efficacy, and Immunogenicity of a Recombinant Coronavirus-Like Particle COVID-19 Vaccine in Adults 18 Years of Age or Older ~~and Children and Adolescents 5-17 Years of Age~~	Based on feedback from BRDD, the study population of children and adolescents will no longer be included in this study.

#	Section	Change	Rationale
	AND<br>Section 19.2 (Appendix 2 – Subject Diary Sample Pages)<br>AND<br>Section 19.3 (Appendix 3 – Subject Memory Aid Sample Pages)<br>AND<br>Section 19.6 (Appendix 6 – COVID-19 Diary Sample Pages)		
2.	Synopsis (Study Rationale)<br>AND<br>Section 1.5 (Overall Rationale for the Study)	The first, second, third, fourth, and sixth paragraphs and table have been updated as follows:<br>This Phase 2/3 study is a multi-portion design to confirm that the chosen formulation and dosing regimen of CoVLP (i.e. two doses of 3.75 µg CoVLP adjuvanted with AS03 [hereafter referred to as "CoVLP formulation"] given 21 days apart) has an acceptable immunogenicity and safety profile in adults 18 years of age or older ~~and children and adolescents 5 to 17 years of age~~ (Phase 2 portion). The efficacy of this CoVLP formulation in these same Study Populations will be assessed in the Phase 3 portion.<br>The Phase 2 portion is a randomized, observer-blinded, placebo-controlled study to evaluate the CoVLP formulation, compared to placebo, in subjects from the following ~~four~~ **two** Study Populations:<br><table><tr><th>Study Population</th><th>Age and/or Health Category</th><th>Age Range (years)</th></tr><tr><td>1</td><td>Healthy adults</td><td>18-64</td></tr></table>	Based on feedback from BRDD, the study populations of children and adolescents, adults with comorbidities, and pregnant women will no longer be included in this study. In addition, the sample size for the healthy adults and elderly populations has been revised based on feedback from BRDD.

<table>
<tr><th>#</th><th>Section</th><th>Change</th><th>Rationale</th></tr>
<tr><td></td><td></td><td>
<table>
<tr><td>~~2~~</td><td>~~Healthy children and adolescents~~</td><td>~~5-17~~</td></tr>
<tr><td>**2**~~3~~</td><td>**Healthy** ~~E~~elderly adults</td><td>≥ 65</td></tr>
<tr><td>~~4~~</td><td>~~Adults with significant co-morbidities~~</td><td>~~≥ 18~~</td></tr>
</table>
In each Study Population, ~~120~~ **306** subjects will be randomized **5**:1 to receive the CoVLP formulation:placebo. All subjects will be followed for a period of 12 months after the last vaccination for durability of the immune responses, safety, and efficacy.
The Phase 3 portion is an event-driven, randomized, observer-blinded, placebo-controlled design that will evaluate the efficacy and safety of the CoVLP formulation, compared to placebo, in the **same** Study Populations #1 - #~~4~~**2** presented above ~~and a possible fifth Study Population of healthy female adults who are in the 3rd trimester of their low-risk pregnancy~~. Up to 30 000 subjects will be enrolled in a 1:1 ratio to receive the CoVLP formulation:placebo. Immunogenicity will only be evaluated in a subset of ~~480~~ **192** subjects.
~~Risk:benefit considerations are different for each of these five Study Populations and will influence how these populations are included in the Phase 2 portion of the study and their relative proportions included in the Phase 3 portion. Population-specific mitigation strategies will be incorporated into the transition between the Phase 2 and Phase 3 portions.~~</td><td></td></tr>
<tr><td>3.</td><td>Synopsis (Study Rationale)
AND
Section 1.5 (Overall Rationale for the Study)</td><td>The fifth paragraph has been updated as follows:
~~All subjects will be followed for efficacy until~~ **Once** approximately 160 COVID-19 cases have been accumulated**,** ~~in order to evaluate~~ the primary efficacy endpoint of the Phase 3 portion **will be evaluated**. **All** ~~S~~**s**ubjects will be followed for **efficacy and** safety for a period of 12 months after the last vaccination. Persistence of the vaccine-induced immune responses up to 12 months after the last vaccination will be assessed in a subset of subjects.</td><td>Clarified that subjects will be followed for efficacy until the end of the study in the Phase 3 portion.</td></tr>
<tr><td>4.</td><td>Synopsis (Secondary Objectives – Phase 2</td><td>The following new secondary objectives have been added for immunogenicity and safety:</td><td>Based on feedback from BRDD, these</td></tr>
</table>

#	Section	Change	Rationale
	Portion and Phase 3 Portion – Immunogenicity and Safety)<br>AND<br>Section 2.2 (Secondary Objectives – Immunogenicity and Safety)	Immunogenicity:<br>• **To assess the relative neutralizing antibody response between the healthy adults (Study Population #1) and the healthy elderly populations (Study Population #2; each age strata) at 21 days after the last vaccination;**<br>Safety:<br>• **To assess the relative incidence of solicited local and systemic AEs up to 7 days after the first and second vaccinations between the healthy adults (Study Population #1) and each healthy elderly population (Study Population #2);**	two new objectives have been added.
5.	Synopsis (Secondary Objectives – Phase 2 Portion and Phase 3 Portion – Immunogenicity)<br>AND<br>Section 2.2 (Secondary Objectives – Immunogenicity)	The first three sub-bullet points for the immunogenicity secondary endpoints for the Phase 2 and Phase 3 portion have been updated as follows:<br>Phase 2 Portion<br>• To assess the immunogenicity of the CoVLP formulation, compared to placebo, as determined by the:<br>• Persistence of neutralizing antibody titers induced by the vaccine against the SARS-CoV-2 virus at Days **128,** 201**,** and Day 386;<br>• IgG (enzyme-linked immunosorbent assay [ELISA]) titers induced by the vaccine against the SARS-CoV-2 virus up to 21 days after the last vaccination and persistence of these antibodies at **Day 128,** Day 201**,** and Day 386;<br>• The ratio of neutralizing antibody titers:IgG (ELISA) antibody titers at Day 21, Day 42, **Day 128,** Day 201, and Day 386;<br>Phase 3 Portion<br>• To assess the immunogenicity of the CoVLP formulation, compared to placebo, in a subset of subjects, as determined by:	Based on BRDD feedback, an immunogenicity (serology only) blood draw at 3 months post second vaccination has been included in each portion of the study.

#	Section	Change	Rationale
		• Neutralizing antibody titers induced by the vaccine against the SARS-CoV-2 virus up to 21 days after the last vaccination and at **Day 128,** Day 201**,** and Day 386;<br>• IgG (ELISA) titers induced by the vaccine against the SARS-CoV-2 virus up to 21 days after the last vaccination and at **Day 128,** Day 201**,** and Day 386;<br>• The ratio of neutralizing antibody titers:IgG (ELISA) antibody titers at Day 21, Day 42, **Day 128,** Day 201, and Day 386;	
6.	Synopsis (Secondary Objectives – Phase 2 Portion – Efficacy)<br>AND<br>Section 2.2 (Secondary Objectives – Efficacy)	For the second bullet point for the Phase 2 portion Efficacy secondary objectives, the following text has been removed:<br>The list above applies to the adult populations; for children and adolescents, the following list of occurrences is applicable:<br>• Severe pneumonia:<br>• Polypnea: ≥ 30 times/min (>5 years) (after ruling out the effects of fever and crying);<br>• Oxygen saturation < 92% under a resting state;<br>• Dyspnea: assisted breathing (moans, nasal flaring, and three concave sign), cyanosis, intermittent apnea;<br>• Disturbance of consciousness: somnolence, coma, or convulsion;<br>• Pulmonary high-resolution CT (HRCT) examination showing bilateral or multi-lobe infiltrates, rapid progression of disease in a short period or with pleural effusion;<br>• Critical cases: those who meet any of the following criteria and require hospitalization:<br>• Respiratory failure requiring mechanical ventilation;<br>• Shock;<br>• Combined with other organs failure;<br>• Death.	Based on feedback from BRDD, the study population of children and adolescents will no longer be included in this study.

#	Section	Change	Rationale
7.	Synopsis (Secondary Objectives – Phase 3 Portion – Efficacy)<br>AND<br>Section 2.2 (Secondary Objectives – Efficacy)	The first bullet point for the Phase 3 portion Efficacy secondary objectives has been moved to the Phase 3 portion Efficacy exploratory objectives section:<br>**Efficacy:**<br>To evaluate the duration of protection (vaccine efficacy) of the CoVLP formulation compared to placebo;	Based on feedback from BRDD, this efficacy endpoint was moved to the exploratory endpoint section.
8.	Synopsis (Number of Planned Subjects)<br>AND<br>Section 12.2 (Sample Size Determination)	The first, second, and fourth paragraphs have been updated as follows:<br>Overall, up to a maximum of 30 **612** ~~480~~ subjects are planned for randomization in the Phase 2 and Phase 3 portions in total.<br>In the Phase 2 portion of the study, up to ~~480~~ **612** subjects will be enrolled in a 4**5**:1 ratio to receive CoVLP formulation:placebo in each Study Population.<br>Only a subset of approximately ~~480~~ **192** subjects (96 subjects from each Study Population) will be included in the immunogenicity evaluation. These subjects will be enrolled from selected sites in Canada and/or the US that will be defined prior to the start of the Phase 3 portion.	Based on feedback from BRDD, the sample size for the healthy adults and elderly populations has been revised.
9.	Synopsis (Sample Size Determination)<br>AND<br>Section 12.2 (Sample Size Determination)	The first and last paragraphs have been updated as follows:<br>The sample size of up to ~~480~~ **612** subjects will serve to confirm vaccine immunogenicity in the two Study Populations and detect sizable differences in rates of adverse events. The sample size is not large enough to detect less frequent or rare adverse reactions. With ~~384~~ **510** subjects receiving the CoVLP formulation, there will be at least 95 % probability of observing an adverse event that occurs as frequently as 1 in ~~128~~ **170** vaccinees.<br>An immunogenicity subset sample size of ~~480~~ **192** subjects is deemed sufficient to provide meaningful statistical assessment of immunologic outcomes.	Based on feedback from BRDD, the sample size for the healthy adults and elderly populations has been revised.
10	Synopsis (Study Population)	The section has been updated as follows:<br>Male and female subjects aged ~~5~~ **18** years of age and older from ethnically and racially diverse populations will be included in each of the ~~four~~ **two** Study Populations in the study. ~~In addition, healthy female adults 18 years of age or~~	Based on feedback from BRDD, the study populations of children and

medicago

#	Section	Change	Rationale
		~~older who are in the 3rd trimester of their low-risk pregnancy will be included in the Phase 3 portion of the study.~~	adolescents, adults with comorbidities, and pregnant women will no longer be included in this study.
11	Synopsis (Dosage and Administration)<br>AND<br>Section 7 (Dosage and Administration)	The fourth, fifth, and sixth paragraphs have been updated as follows:<br>~~In Study Population #2, subjects who are 10 to 17 years of age will receive 3.75 µg CoVLP adjuvanted with AS03 in 0.5 mL. Subjects who are 5-9 years of age will receive half the dose of the CoVLP formulation (i.e. 0.25 mL containing 1.87 µg CoVLP adjuvanted with half the standard dose of AS03).~~<br><br>~~For the adolescents in Study Population #2, a dose de-escalation may occur to reduce the dose of CoVLP and AS03 by half (i.e. 0.25 mL of the full dose will be administered containing 1.87 µg CoVLP and half of the AS03 dose). In the event that the IDMC considers the safety profile to be acceptable but the immunogenicity profile is not acceptable, then this population will proceed to the second strategy of administering the standard antigen dose (i.e. 3.75 µg) with half the adjuvant dose in a 0.25 mL volume. If the safety profile is acceptable to the IDMC, then recruitment will continue until a total of 60 adolescents and 60 children receiving the same CoVLP formulation in each age cohort have been enrolled in Study Population #2. If the half dose of the CoVLP formulation is used in the Phase 2 portion for one or both age cohorts in Study Population #2, then the same formulation(s) will be used during the Phase 3 portion of the study.~~<br><br>Study Populations #**2**~~3 and #4~~ (elderly adults~~ or adults with significant comorbidities, respectively~~) will receive the full standard dose of CoVLP (3.75 µg) adjuvanted with AS03 in an injection volume of 0.5 mL. In Phase 2, if an acceptable immune response is not achieved at either Day 21 or Day 42, the IDMC may recommend a dose escalation to 7.5 µg CoVLP adjuvanted with AS03 in a volume of 0.5 mL, and the same number of subjects (i.e. ~~120~~ **306** subjects~~ per study population~~) ~~in the particular Study Population~~ will be recruited, using the same enrollment approach (i.e. use of sentinel subjects	Based on feedback from BRDD, the study populations of children and adolescents, adults with comorbidities, and pregnant women will no longer be included in this study. In addition, the sample size for the healthy adults and elderly populations has been revised based on feedback from BRDD.

<table>
<tr><th>#</th><th>Section</th><th>Change</th><th>Rationale</th></tr>
<tr><td></td><td></td><td>and IDMC reviews). If the higher dose of the CoVLP formulation achieves an acceptable immune response in the Phase 2 portion, then the same higher dose will be used in Study Population~~s~~ #2~~3 and/or #4~~ during the Phase 3 portion of the study.</td><td></td></tr>
<tr><td>12</td><td>Synopsis (Study Design)
AND
Section 4.1 (Overview of Study Design)</td><td>The content under the Phase 2 portion sub-header has been updated as follows:
The Phase 2 portion is a randomized, observer-blinded, placebo-controlled, design with male and female subjects. Subjects will be enrolled from ~~four~~ two Study Populations:
<table>
<tr><th>Study Population</th><th>Age and/or Health Category</th><th>Age Range (years)</th></tr>
<tr><td>1</td><td>Healthy adults</td><td>18-64</td></tr>
<tr><td>~~2~~</td><td>~~Healthy children and adolescents~~</td><td>~~5-17~~</td></tr>
<tr><td>2~~3~~</td><td>Healthy ~~E~~elderly adults</td><td>≥ 65</td></tr>
<tr><td>~~4~~</td><td>~~Adults with significant co-morbidities~~</td><td>~~≥ 18~~</td></tr>
</table>
In each of the ~~four~~ two Study Populations, ~~120~~ 306 subjects will be randomized 45:1 and will receive the same CoVLP formulation or placebo. Subjects will receive two IM injections 21 days apart on Days 0 and 21, into the deltoid region of the alternating arm (each arm will be injected once), of one of the following:
• CoVLP formulation;
• Placebo.
~~Adults 18 years of age and older (i.e. Study Populations #1, #3, and #4)~~ All subjects will begin enrollment at the same time. Enrollment of elderly adult subjects (Study Population #2) will be stratified by age in an approximately 2:1 ratio of 65 to 74 years of age and 75 years of age and older, respectively. ~~Enrollment of healthy children and adolescents aged 5 to 17 years (i.e. Study Population #2) will begin only after the safety data following the second vaccination for the Healthy Adult population (i.e. Day 28 for all subjects in Study Population #1) have been reviewed by the Independent Data Monitoring Committee (IDMC). Study Population #2 will be stratified by age in a 1:1 ratio~~</td><td>Based on feedback from BRDD, the study populations of children and adolescents, adults with comorbidities, and pregnant women will no longer be included in this study. In addition, the sample size for the healthy adults and elderly populations has been revised based on feedback from BRDD.</td></tr>
</table>

<table>
<tr><th>#</th><th>Section</th><th>Change</th><th>Rationale</th></tr>
<tr><td></td><td></td><td>of adolescents 10-17 years of age followed by children 5-9 years of age. Enrollment of children will only begin after the IDMC review of safety data following the second vaccination for all adolescent subjects by the IDMC (i.e. Day 28). Enrollment of subjects in Study Population #34 will include adults with co-existing conditions such as, but not limited to, obesity or being overweight, cardiovascular diseases, hyperlipidemia, chronic kidney disease or related disorders, chronic respiratory disease, pre-diabetes and diabetes, treatment-controlled HIV, and psychiatric disorders.
The IDMC will review unblinded safety data for the first 10 sentinel subjects after each dose in all Study Populations #2, with the exception of Study Population #1, according to the details outlined in the IDMC Charter. All sentinel subjects will be dosed at least 30 minutes apart. Further, the enrollment for Study Population #2 will begin with the adolescent subjects after all of the seven-day safety data following the second vaccination of the healthy adults (Study Population #1) have been reviewed by the IDMC and after the availability of the Day 21 immunogenicity data from all the healthy adults (Study Population #1).</td><td></td></tr>
<tr><td>13</td><td>Synopsis (Study Design)
AND
Section 4.1 (Overview of Study Design)</td><td>The content under the Phase 3 portion sub-header has been updated as follows:

The Phase 3 portion is a randomized, observer-blinded, placebo-controlled, efficacy, safety, immunogenicity (in a subset of subjects), multicenter design with male and female subjects. Similar to the Phase 2 portion, subjects will be enrolled from five two Study Populations:

<table><tr><th>Study Population</th><th>Age and/or Health Category</th><th>Age Range (years)</th></tr><tr><td>1</td><td>Healthy adults</td><td>18-64</td></tr><tr><td>2</td><td>Healthy children and adolescents</td><td>5-17</td></tr><tr><td>23</td><td>Healthy Eelderly adults</td><td>≥ 65</td></tr><tr><td>4</td><td>Adults with significant co-morbidities</td><td>≥ 18</td></tr></table></td><td>Based on feedback from BRDD, the study populations of children and adolescents, adults with comorbidities, and pregnant women will no longer be included in this study. In addition, the sample size for the healthy adults and elderly populations has been revised</td></tr>
</table>

<table>
<tr><th>#</th><th>Section</th><th>Change</th><th>Rationale</th></tr>
<tr><td></td><td></td><td>
<table>
<tr><td>~~5~~</td><td>~~Healthy female adults who are in the 3rd trimester of their low-risk pregnancy~~</td><td>~~≥ 18~~</td></tr>
</table>
Study Populations #2 ~~and #3~~ will be roughly stratified by age in the same manner. The overall goal of the Phase 3 portion of this study is to enroll approximately 30 000 subjects who are generally representative of the overall target population in terms of racial diversity with enrichment for those at highest risk for serious COVID-19 disease (i.e. the elderly ~~and those with co-morbidities~~) ~~and relatively smaller numbers of the subjects at lowest risk of serious COVID-19 (i.e. children and adolescents)~~. ~~In addition, Study Population #45 enrollment will only begin after the administration of the second vaccination of all subjects in Study Populations #1 to #4.~~

In each Study Population, subjects will be randomized into one of two treatment groups:
150. CoVLP formulation;
151. Placebo.

Active and passive surveillance will be conducted in the study from 14 days after the last vaccination until ~~160 COVID-19 cases have been accumulated~~ **386 days**. For passive surveillance, subjects will be instructed to report symptoms meeting the definition of new COVID-19 disease. For active surveillance, subjects will be contacted once per week via the subject's preferred method (e.g. phone, text, email, electronic diary), and asked about symptoms of COVID-19 disease. In addition, to monitor asymptomatic infection, subjects will perform a test for SARS-CoV-2 N antibodies on Day 0, Day 201, and Day 386.
</td><td>based on feedback from BRDD.</td></tr>
<tr><td>14</td><td>Synopsis (Study Design)
AND
Section 4.1 (Overview of Study Design)</td><td>The last sentence of the fifth paragraph under the Phase 2 portion sub-header and the last sentence of the fourth paragraph under the Phase 3 portion sub-header have been updated as follows:
Phase 2 Portion</td><td>Based on BRDD feedback, an immunogenicity (serology only) blood draw at 3 months post second vaccination</td></tr>
</table>

#	Section	Change	Rationale
		Subjects will return to the Investigator site on **Day 128,** Day 201**,** and Day 386 (**3-month,** 6-month**,** and 12-month safety follow-ups and immunogenicity assessments after the last vaccine administration).<br><br>Phase 3 Portion<br>Subjects will return to the Investigator site on **Day 128 (immunogenicity subset only),** Day 201**,** and Day 386 (6-month and 12-month safety follow-ups and **3-month, 6-month, and 12-month** immunogenicity assessments [immunogenicity subset only] after the last vaccine administration).	has been included in each portion of the study.
15	Synopsis (Immunogenicity Evaluations)<br>AND<br>Section 10.3.1 (Immunogenicity Evaluations)	The first sentence has been updated as follows:<br>Immunogenicity will be evaluated in all subjects in the Phase 2 portion and in a subset of ~~480~~ **192** subjects in the Phase 3 portion of the study across the Study Populations with age distribution similar to the Phase 2 portion. Immunologic outcomes will include the humoral immune response (neutralization assay and IgG ELISA) and the CMI response induced in subjects on Day 0, Day 21, Day 42, **Day 128 (neutralization assay and IgG ELISA),** Day 201, and Day 386.	Based on BRDD feedback, an immunogenicity (serology only) blood draw at 3 months post second vaccination has been included in each portion of the study.<br>In addition, the immunogenicity sample size was updated due to fewer study populations.
16	Synopsis (Efficacy Evaluations)<br>AND<br>Section 10.4.1 (Efficacy Evaluations)	The first paragraph has been modified as follows:<br>Following the first vaccination, subjects will be asked to report symptoms associated with COVID-19 from 14 days after the last vaccination until the end of the Phase 2 portion or ~~until the required number of COVID-19 cases have been collected in order to evaluate the primary efficacy endpoint in~~ the Phase 3 portion of the study (through active or passive surveillance). COVID-19 will be defined as the presence of a laboratory-confirmed (virologic method) SARS-CoV-2 infection with the occurrence of a new onset of one or	Clarified that subjects will be followed for efficacy until the end of the study in the Phase 3 portion.

#	Section	Change	Rationale
		more of the following symptoms that persist(s) for or reoccur(s) after a period of at least 12 hours:	
17	Synopsis (Efficacy Evaluations)<br>AND<br>Section 10.4.1 (Efficacy Evaluations)<br>AND<br>Section 19.3 (Appendix 3 – Subject Memory Aid Sample Pages)	The second paragraph has been removed:<br>The symptoms listed above apply to the adult populations, and will also apply to children and adolescents with the following additions:<br>Additional symptoms:<br>• Abdominal pain;<br>• Poor appetite.	Based on feedback from BRDD, the study population of children and adolescents will no longer be included in this study.
18	Synopsis (Secondary Endpoints)<br>AND<br>Section 10.2.3.2 (Secondary Endpoints)	The following new secondary safety endpoint has been added in the Phase 2 portion:<br>In the Phase 2 portion, the secondary safety endpoints are:<br>• **Relative occurrence of solicited local and systemic AEs for seven days following each vaccine administration between the healthy adults (Study Population #1) and each healthy elderly population (Study Population #2);**	Based on feedback from BRDD, a new safety secondary endpoint has been added.
19	Synopsis (Secondary Endpoints)<br>AND<br>Section 10.3.2.2 (Secondary Endpoints)	The following new secondary immunogenicity endpoint has been added in the Phase 2 portion:<br>In the Phase 2 portion, the secondary immunogenicity endpoints are:<br>• **Relative neutralizing antibody response between the healthy adults (Study Population #1) and the healthy elderly populations (Study Population #2; each age strata) induced at 21 days after the last vaccination will be analyzed using the following parameter: GMT;**	Based on feedback from BRDD, a new immunogenicity secondary endpoint has been added.
20	Synopsis (Secondary Endpoints)	The first three bullet points for the immunogenicity secondary endpoints for the Phase 2 and Phase 3 portions have been updated as follows:	Based on BRDD feedback, an

#	Section	Change	Rationale
	AND<br>Section 10.3.2.2 (Secondary Endpoints)	Phase 2 Portion<br>• Persistence of the Nab antibody response induced in each Study Population against the SARS-CoV-2 virus at **Day 128,** Day 201**,** and Day 386 will be analyzed using the following parameters: GMT, SC rate, and GMFR;<br>• Specific antibody response induced in each Study Population against the SARS-CoV-2 virus will be measured by the total IgG levels on Days 0, 21, and 42, and the persistence of these antibodies at Days **128,** 201**,** and 386 and analyzed using the following parameters: GMT, SC rate, and GMFR;<br>• The ratio of neutralizing antibody titers: IgG (ELISA) antibody titers at Day 21, Day 42, **Day 128,** Day 201, and Day 386;<br>Phase 3 Portion<br>• In a subset of subjects, Nab antibody response induced in each Study Population against the SARS-CoV-2 virus on Days 0, 21, 42, **128,** 201, and 386 analyzed, using the following parameters: GMT, SC rate, and GMFR;<br>• In a subset of subjects, specific antibody response induced in each Study Population against the SARS-CoV-2 virus measured by the total IgG levels on Days 0, 21, 42, **128,** 201, and 386, and analyzed using the following parameters: GMT, SC rate, and GMFR;<br>• In a subset of subjects, the ratio of neutralizing antibody titers:IgG (ELISA) antibody titers at Day 21, Day 42, **Day 128,** Day 201, and Day 386;	immunogenicity (serology only) blood draw at 3 months post second vaccination has been included in each portion of the study.
21	Synopsis (Secondary Endpoints)<br>AND<br>Section 10.4.2.2 (Secondary Endpoints)	The first bullet point in the Efficacy secondary endpoints for the Phase 3 portion has been moved to the Efficacy exploratory endpoints section for Phase:<br>**Efficacy:**<br>The slope estimate of the weighted Schoenfeld residuals against event time for the evaluation of vaccine efficacy determined from the first occurrence, in a	

#	Section	Change	Rationale
		subject, of laboratory-confirmed (virologic method) symptomatic SARS-CoV-2 infection (≥ 14 days post-last vaccination);	
22	Synopsis (Safety Review)<br>AND<br>Section 13.1.11 (Safety Review and Stopping Rules)	The second line of the first paragraph has been updated as follows:<br>During the Phase 2 portion, the safety data for the first 10 sentinel subjects after each dose in ~~all~~ Study Population~~s~~ **#2**~~, with the exception of Study Population #1,~~ will be reviewed by the IDMC before permitting enrollment of the remaining subjects, according to the details outlined in the IDMC Charter.	Based on feedback from BRDD, the study populations of children and adolescents, adults with comorbidities, and pregnant women will no longer be included in this study.
23	Synopsis (Statistical Methods – Populations)	The second paragraph in this section has been updated as follows:<br>In each of the Study Populations for the Phase 2 and Phase 3 portions:<br>• All safety analyses will be performed using the SAS;<br>• All efficacy analyses will be performed using both the PP set and the ITT set;<br>• All immunogenicity analyses will be performed using both the IPP set and the immunogenicity ITT set;<br>• **In Phase 2, analyses using the PP and IPP will be considered the primary analyses for efficacy and immunogenicity objectives respectively.**<br>• **In Phase 3, analyses using the ITT will be considered the primary analyses for efficacy objectives. Analyses using the IPP will be considered the primary analyses for immunogenicity objectives.**	Based on BRDD feedback, clarification has been added regarding the primary analyses populations for the efficacy and immunogenicity analyses.
24	Synopsis (Statistical Methods – Phase 2 Portion Day 21 and Possibly Day 42	The first two paragraphs have been updated as follows:<br>For the Phase 2 portion, the unblinded statistical team will **analyze the** ~~perform~~ immunogenicity data after Day 21 in each of the Study Populations. The data analysis will involve selected individuals from the unblinded statistical team and Medicago who will be unblinded (see Section 6.2). The~~is~~	To clarify the details regarding the Day 21 and Day 42 immunogenicity data analysis and to ensure

#	Section	Change	Rationale
	Immunogenicity Data Analysis)<br><br>AND<br><br>Section 12.3 (Day 21 and Possibly Day 42 Immunogenicity Data Analysis and Interim Efficacy Analyses)	**Day 21** analysis **in Study Population #1 and the Day 42 analysis in Study Population #2** will confirm **whether** ~~that~~ an acceptable immune response has been induced in each Study Population prior to the initiation of the Phase 3 portion of the study **(refer to Section 12.4 for details of the criteria that will be used)**, without having to wait until after the end of the 386 days follow-up period for study completion.<br><br>~~In the event that the Day 21 Nab response~~ **If the Day 42 analysis in Study Population #2 confirms that an acceptable response has been induced (refer to Section 12.4 for details of the criteria that will be used), the Study Population #2 will be permitted to proceed into the Phase 3 portion of the study.** ~~in any of the Study Populations is <50% of that achieved by healthy adults, then a similar data analysis will be performed after Day 42 for the Study Populations with the lower Nab response in order to initiate the Phase 3 portion of the study for the particular Study Population.~~ However, if at Day 42**,** the ~~Nab~~ **immune** response for ~~a~~ Study Population **#2** is still **relatively weak compared to the healthy adult Day 42 responses,** ~~<50 % of that achieved by healthy adults,~~ an ad hoc IDMC meeting will be convened to consider changes to the Protocol to permit alternate vaccination strategies in ~~the implicated~~ Study Population **#2**~~(s)~~.	consistency with the content of Section 12.4 of the protocol.
25	Synopsis (Statistical Methods – Phase 3 Portion Initiation)	The section has been updated as follows:<br><br>The start of the Phase 3 portion of the study will be determined independently for each Study Population and will be based on the outcomes of the following two assessments during the Phase 2 portion of the study:<br>• Day 21 Data Analysis of the immunogenicity data **in Study Population #1** (described above);<br>• **Day 42 Data Analysis of the immunogenicity data in Study Population #2 (described above);**<br>• Day 28 Safety Review **in Study Population #1 and Study Population #2** (described in the Safety Review section above).	Based on feedback from BRDD, the study populations of children and adolescents, adults with comorbidities, and pregnant women will no longer be included in this study. In addition, further clarification on the criteria for moving

		Study Population #1: If the safety and immunogenicity profiles of the CoVLP formulation observed in the Phase 1 study are acceptable in the Phase 2 portion, this group will proceed into the Phase 3 portion. Another key outcome will be the absence of a significant safety signal as assessed by the IDMC.<br><br>**Study Populations #2: If the safety and immunogenicity profiles of the CoVLP formulation in this population are acceptable, entry of Study Population #2 into the Phase 3 portion will be based on both the absence of an important safety signal and demonstration of an acceptable immune response as assessed by the IDMC. The criteria for acceptability will be:**<br>• **No statistically significant difference in the Nab responses at Day 42 between Study Population #1 and Study Population #2;**<br>• **No significant higher incidence of solicited local and systemic AEs reported 7 days after each vaccine administration in Study Population #2 compared to Study Population #1.**<br><br>**The same criteria will be applied to the comparisons of the two age strata for Study Population #2 versus Study Population #1. In the event of a significant difference between Study Population #1 and one of the age stratum for Study Population #2, the next highest dose level that can be administered to this population will be assessed in the same number of subjects (as planned for the age stratum in the Phase 2 portion).**<br><br>~~If the Day 21 immunogenicity data and Day 28 safety data results are favorable based upon pre-defined criteria and show acceptable safety and immune responses for Study Populations #**2**3 and #4, assuming the results in Study Population #1 will be favorable, then the Phase 3 portion of the study can start for all three both Study Populations. The decision to proceed into the Phase 3 portion of the study for these two Study Populations #**2** will be taken in close consultation with the IDMC.~~<br>~~As mentioned above, if the Day 21 immune response is not acceptable in one or both of Study Populations #**2**3 and #4, then the Day 42 immunogenicity data will be assessed in the applicable Study Population(s) for the purposes of~~	into the Phase 3 portion has been provided.

#	Section	Change	Rationale
		~~determining whether the Phase 3 portion may be started for that/those Study Population(s) #~~**~~2~~**~~.~~<br><br>~~For Study Population #2, the Day 21 immunogenicity data and Day 28 safety data results will also be assessed and if an acceptable immune response is observed and the IDMC has no concerns with the safety data up to Day 28, then the Phase 3 portion of the study can start for Study Population #2 with staggering of the age cohorts as outlined above.~~	
26	Synopsis (Statistical Methods – Immunogenicity Analyses)	The third sentence in the first paragraph has been updated as follows:<br>GMFR will be derived by using Analysis of Covariance (ANCOVA) to model the difference in the log of the titer values between Day 21/42/**128**/201/386 and Day 0, with treatment group as main effect and baseline titer as covariate.	Based on BRDD feedback, an immunogenicity (serology only) blood draw at 3 months post second vaccination has been included in each portion of the study.
27	Synopsis (Statistical Methods – Efficacy Analyses)	The second last paragraph has been updated as follows:<br><br>~~Absolute~~ VE will also be calculated for the secondary and exploratory efficacy endpoints. The ~~absolute~~ VE success criterion for the secondary efficacy endpoints, in the Phase 3 portion, is defined as > 0 % lower limit of the ~~appropriately alpha-adjusted~~ **95%** confidence interval ~~that maintains overall Type I error at 5 %~~.	Clarified the lower limit based on BRDD feedback.
28	Throughout the Protocol	The use of “absolute” in reference to vaccine efficacy has been removed from all applicable sections of the protocol.	Based on BRDD feedback, clarified that vaccine efficacy will not be referred to as “absolute vaccine efficacy”.

#	Section	Change	Rationale
29	Table 1 and thereafter in Protocol	All occurrences of the following text have been removed from the protocol:<br>“(and/or their parent/legally acceptable representative [LAR])”<br>And<br>“(and/or their parent/LAR)”<br>And<br>“their child”	Based on feedback from BRDD, the study populations of children and adolescents will no longer be included in this study.
30	Table 1 (Time and Events Schedule: General Information for the Phase 2 Portion)	Table 1 has been updated as follows:<br>A new column for Day 128 (± 14) has been added after the Day 42 column with the following changes:<br>• New visit number 7<br>• An “X” for Vital Signs<br>• An “X” for Immunogenicity – Serology (Nab assay and ELISA)<br>• An “X” for Provide and collect diary and memory aid instructions<br>• Day 201 is now visit number ~~7~~**8**<br>• Day 386 is now visit number ~~8~~**9**	Based on BRDD feedback, an immunogenicity (serology only) blood draw at 3 months post second vaccination has been included in each portion of the study.
31	Table 1 (Time and Events Schedule: General Information for the Phase 2 Portion)	The content under each sub-heading for the Collection of COVID-19 symptoms through passive and active surveillance row has been updated as follows:<br>Passive Surveillance: Subjects will be instructed to contact the study site if they experience symptoms of COVID-19 from 14 days after the last vaccination until the end of the Phase 2~~ or until 160 COVID-19 cases have been accumulated in the Phase 3~~ portion of the study.<br>Active Surveillance: Between 14 days after the last vaccination and the end of the Phase 2 ~~or until 160 COVID-19 cases have been accumulated in the Phase 3~~ portion of the study, the subjects will be contacted once per week via the method most likely to be successful (e.g. subject’s preferred method [phone, text, email, electronic diary]), and asked about symptoms of COVID-19.	Clarified that subjects will be followed for efficacy until the end of the study in the Phase 3 portion.
32	Table 1 (Time and Events Schedule:	Footnotes #4 and #8 have been updated as follows:	Based on feedback from BRDD, the

medicago

#	Section	Change	Rationale
	General Information for the Phase 2 Portion)	[4] In all females of childbearing potential in Study Population #1~~, #2, and #4~~; it will be tested in serum at screening and in urine at Days 0, 21, and 42.<br>[8] All efforts will be done to have subjects returning on planned date for Day 21 activities. If for any reason the visit is done before or after this planned date, subsequent visits/procedures (Days 22, 24, 29, 42, **128,** 201, and 386) will be adjusted accordingly.	study populations of children and adolescents, adults with comorbidities, and pregnant women will no longer be included in this study. In addition, an immunogenicity (serology only) blood draw at 3 months post second vaccination has been included in each portion of the study based on BRDD feedback.
33	Table 2 (Time and Events Schedule: General Information for the Phase 3 Portion)	Table 2 has been updated as follows:<br>A new column for Day 128 (± 14) has been added after the Day 42 column with the following changes:<br>• New visit number 4<br>• New footnote "10" has been added<br>• An "X" for Immunogenicity – Serology (Nab assay and ELISA)<br>• An "X" for Provide and collect diary and memory aid instructions<br>• Day 201 is now visit number ~~4~~**5**<br>• Day 386 is now visit number ~~5~~**6**	Based on BRDD feedback, an immunogenicity (serology only) blood draw at 3 months post second vaccination has been included in each portion of the study.
34	Table 2 (Time and Events Schedule: General Information for the Phase 3 Portion)	The content under each sub-heading for the Collection of COVID-19 symptoms through passive and active surveillance row has been updated as follows:<br><u>Passive Surveillance</u>: Subjects will be instructed to contact the study site if they experience symptoms of COVID-19 from 14 days after the last	Clarified that subjects will be followed for efficacy until the end of the study in the Phase 3 portion.

#	Section	Change	Rationale
		vaccination until the end of the ~~Phase 2 or until 160 COVID-19 cases have been accumulated in the~~ Phase 3 portion of the study.<br>Active Surveillance: Between 14 days after the last vaccination and the end of the ~~Phase 2 or until 160 COVID-19 cases have been accumulated in the~~ Phase 3 portion of the study, the subjects will be contacted once per week via the method most likely to be successful (e.g. subject's preferred method [phone, text, email, electronic diary]), and asked about symptoms of COVID-19.	
35	Table 2 (Time and Events Schedule: General Information for the Phase 3 Portion)	Footnotes #4 and #8 have been updated and a new footnote #10 has been added as follows:<br>[4] In all females of childbearing potential in Study Population #1~~, #2, #4, and #5~~; it will be tested in serum at screening and in urine at Days 0, 21, and 42. ~~Study Population #5: any female subject who is in the 1st or 2nd trimester of her pregnancy cannot participate in the study. However, any female subject who is in the 3rd trimester of her low-risk pregnancy can participate in the study.~~<br>[8] All efforts will be done to have subjects returning on planned date for Day 21 activities. If for any reason the visit is done before or after this planned date, subsequent visits/procedures (Days 22, 29, 42, **128,** 201, and 386) will be adjusted accordingly.<br>**[10] This visit is only for subjects included in the immunogenicity subset.**	Based on feedback from BRDD, the study populations of children and adolescents, adults with comorbidities, and pregnant women will no longer be included in this study. In addition, an immunogenicity (serology only) blood draw at 3 months post second vaccination has been included in each portion of the study based on BRDD feedback.
36	Section 1.1.2 (Adults with Comorbidities)	The content of this section has been removed:<br>People of any age with serious underlying medical conditions such as diabetes and cardiovascular or lung disease are at a greater risk of severe COVID-19. Those individuals with underlying uncontrolled medical conditions such as diabetes, hypertension, lung, liver, and kidney disease, cancer patients on chemotherapy, smokers, transplant recipients, and patients taking steroids	Based on feedback from BRDD, the study populations of children and adolescents, adults with comorbidities,

#	Section	Change	Rationale
		represent a vulnerable population who are not only at a higher risk of developing severe illness but are also at an increased risk of death [Li 2020, Sanyaolu 2020]. Hypertension (odds ratio [OR]: 2.29), diabetes (OR: 2.47), chronic obstructive pulmonary disease (COPD; OR: 5.97), cardiovascular disease (OR: 2.93), and cerebrovascular disease (OR: 3.89) were independent risk factors associated with COVID-19 [Wang 2020]. A strong association of worse clinical outcomes in COVID-19 disease with obesity, even in the absence of any other comorbidity, has been reported [Korakas 2020]. The in-hospital death OR was found as high as 2.70 in patients with coronary artery disease, 2.48 in patients with congestive heart failure, 1.95 in those with cardiac arrhythmias, 2.96 in patients with COPD, and 1.79 for current smokers [Mehra 2020]. Overall, the risk of severe COVID-19 requiring admission to intensive care unit, invasive ventilation, or leading to death was found to be 1.79 fold greater among patients with at least one comorbidity and 2.59 fold greater among patients with two or more comorbidities compared to generally healthy subjects [Guan 2020a].<br>The need for an effective COVID-19 vaccine for people with underlying health conditions is obvious; however, vaccination strategies for these individuals may require some adjustment to achieve the desirable immune responses and vaccine effectiveness. Many comorbidities can adversely impact the response to vaccination, either due to general deterioration of the immune reactivity or as the result of pharmaceutical management of the underlying disease [Kwetkat 2020, Zimmermann 2019]. For example, patients with COPD generally respond poorly to influenza vaccination with significantly lower antibody titers and seroconversion rates: 43% in COPD compared to 90 % of healthy participants [Nath 2014]. Congestive heart failure patients develop a significantly lower T cell immune response to live-attenuated varicella-zoster vaccine and inactivated influenza vaccine than those without a cardiac impairment [Vardeny 2010, Verschoor 2017]. The effectiveness of 23-valent polysaccharide pneumococcal vaccine (PPV23) against invasive pneumococcal disease in individuals aged ≥65 years falls from 45 % in the healthy elderly to 25 % in high-risk immunocompetent	and pregnant women will no longer be included in this study.

#	Section	Change	Rationale
		patients with chronic respiratory/heart/liver/renal disease; diabetes mellitus; cerebrospinal fluid leaks; cochlear implants and is further reduced to 13 % in patients with underlying health conditions thought to be associated with an immune impairment (asplenia/splenic dysfunction, malignancy or an immunosuppressive drug treatment) [Djennad 2019].	
37	Section 1.1.3 (Pediatric Population)	The content of this section has been removed:<br><br>People of all ages, including children, are at risk of contracting COVID-19 [Liu 2020b]. However, relatively fewer cases of symptomatic COVID-19 are observed among children compared to adult patients. In the US, 1.7 % of COVID-19 cases occurred in individuals aged <18 years, while this age group represents 22 % of the population [CDC 2020a]. Children typically have milder disease and a better prognosis comparted to adults, and very few deaths in children have been reported to date [Ludvigsson 2020]. Nevertheless, children who are in contact with severe COVID-19 cases, those with underlying diseases, such as congenital heart, lung and airway diseases, chronic heart and kidney diseases, malnutrition, tumors, diabetes, immunodeficiency, hereditary metabolic diseases, those taking immunosuppressive medications, and infants under 3 months of age are considered to be at high risk of developing severe or critical illness [Shen 2020]. A severe, potentially life-threatening health condition called multisystem inflammatory syndrome is thought to be associated with SARS-CoV-2 infection in children. Its clinical manifestations such as acute illness accompanied by a hyperinflammatory syndrome leading to multiorgan failure and shock are to some extend similar to those of Kawasaki disease and toxic shock syndrome [Feldstein 2020]. Children may also play a role in SARS-CoV-2 community transmission. They often have symptoms of acute upper respiratory tract infection rather than lower respiratory tract involvement [Dong 2020]. Pediatric subjects were found to have detectable viral RNA in fecal samples 18 to 30 days from the disease onset [Cai 2020], which raised a concern about fecal-oral transmission of the infection. Prolonged shedding of the virus through nasal secretions and stool may have substantial implications	Based on feedback from BRDD, the study populations of children and adolescents, adults with comorbidities, and pregnant women will no longer be included in this study.

#	Section	Change	Rationale
		for SARS-CoV-2 community spread at child-care facilities, schools and at home [Cruz 2020].<br><br>Children generally respond well to all vaccines including pandemic influenza vaccines and adjuvanted formulations, in terms of safety, immunogenicity and effectiveness [Dowling 2015, Garcia-Sicilia 2015, Manzoli 2012]. Antigen dose sparing has been achieved with adjuvanted vaccine formulations in children [Bravo 2020; Vesikari 2012].	
38	Section 1.1.4 (COVID-19 Infection in Pregnancy)	The content of this section has been removed:<br>Highly pathogenic coronavirus infections including SARS-COV-2 are associated with increased risk in pregnant women including severe disease [Buonsenso 2020, Galang 2020, Rasmunssen 2020] and fetal loss [Baud 2020, Hachem 2020, Mehan 2020, Zimmermann 2020]. Some have advocated strongly for the inclusion of pregnant women in trials for COVID therapies and vaccines [DiMascio 2020] and the FDA has encouraged vaccine manufacturers to include pregnant women and women of childbearing potential in their trials [FDA 2020].	Based on feedback from BRDD, the study populations of children and adolescents, adults with comorbidities, and pregnant women will no longer be included in this study.
39	Section 1.1.5 (Risk:Benefit in the Different Study Populations)	A new paragraph has been added before the first paragraph and the last paragraph has been updated as follows:<br>**Although the focus of this protocol is on the administration of the CoVLP formulation in healthy adult and elderly populations, the intent of Medicago's clinical development program is to develop a CoVLP formulation that is safe and effective for use in a much larger range of the population, including adults with comorbidities, children and adolescents, and pregnant women.**<br>At the current time, there is no reason to suspect the CoVLP formulation that will be used in the proposed study poses an elevated risk specific to any of the ~~Study Populations~~ **populations discussed above**. In particular, the adjuvant (AS03) has been used across the entire age range including pregnant women and WOCBP [Cohet 2015] (see Section 1.2.1). As a result, risk:benefit calculations for each of the~~se~~ ~~S~~study ~~P~~populations are driven primarily by the	Based on feedback from BRDD, the study populations of children and adolescents, adults with comorbidities, and pregnant women will no longer be included in this study.

#	Section	Change	Rationale
		benefit denominator. ~~Medicago considers that the direct and indirect benefits amply justify the inclusion of all five of the Study Populations in the current Phase 2/3 study with the safeguards currently proposed including the active role of the IDMC.~~	
40	Section 4.1 (Overview of Study Design)	The fourth paragraph has been updated as follows:<br>The IDMC will review unblinded safety data for the first 10 sentinel subjects after each dose in Study Populations #2~~, #3, and #4~~ prior to vaccinating the remaining subjects in each Study Population according to the details outlined in the IDMC Charter. All sentinel subjects will be dosed at least 30 minutes apart. ~~Further, the enrollment for Study Population #2 will begin with the adolescent subjects after all of the seven-day safety data following the second vaccination of the healthy adults (Study Population #1) have been reviewed by the IDMC and after the availability of the Day 21 immunogenicity data from all the healthy adults (Study Population #1).~~	Based on feedback from BRDD, the study populations of children and adolescents, adults with comorbidities, and pregnant women will no longer be included in this study.
41	Section 4.1 (Overview of Study Design)	Figure 1 has been updated to remove all text referring to children and adolescents as well as adults with comorbidities.	Based on feedback from BRDD, the study populations of children and adolescents, adults with comorbidities, and pregnant women will no longer be included in this study.
42	Section 5.1 (Inclusion Criteria)	The inclusion criteria #2 has been updated as follows:<br>At the Screening visit (Visit 1), male and female subjects must be**:**<br>• Study Populations #1~~ and #5~~: 18 to 64 (has not yet had his/her 65th birthday) years of age, inclusive;<br>~~• Study Population #2: 5 to 17 (has not yet had his/her 18th birthday) years of age, inclusive;~~<br>• Study Population #**2**~~3~~: 65 years of age or older;	Based on feedback from BRDD, the study populations of children and adolescents, adults with comorbidities, and pregnant women

#	Section	Change	Rationale
		• Study Population #4: 18 years of age or older;	will no longer be included in this study.
43	Section 5.1 (Inclusion Criteria)	The inclusion criteria #3 has been updated as follows:<br>At Screening (Visit 1) and Vaccination (Visit 2), subject must have a body mass index (BMI) of:<br>• Study Populations #1, #3, and #5: ≥ 18.5 and < 30 kg/m^2;<br>• Study Population #2: > 5th and < 85th percentile based on the CDC 'BMI for 'age' scale (https;//www.cdc.gov/healthyweight/bmi/calculator.html);	Based on feedback from BRDD, the study populations of children and adolescents, adults with comorbidities, and pregnant women will no longer be included in this study.
44	Section 5.1 (Inclusion Criteria)	The inclusion criteria #5 has been updated as follows:<br>Study Populations #1, #2, and #5: Subjects must be in good general health prior to study participation, with no clinically relevant abnormalities that could jeopardize subject safety or interfere with study assessments, as determined by medical history, physical examination, and vital signs. Investigator discretion will be permitted with this inclusion criterion;<br>Note: Subjects with a pre-existing chronic disease will be allowed to participate if the disease is stable and, according to the Investigator's judgment that must be documented in the source documents, the condition is unlikely to confound the results of the study or pose additional risk to the subject by participating in the study. Stable disease is generally defined as no new onset or exacerbation of pre-existing chronic disease three months prior to vaccination. Based on the Investigator's judgment, a subject with more recent stabilization of a disease could also be eligible.	Based on feedback from BRDD, the study populations of children and adolescents, adults with comorbidities, and pregnant women will no longer be included in this study.
45	Section 5.1 (Inclusion Criteria)	The Study Populations have been updated and the third bullet point has been removed for inclusion criterion #6:<br>• Study Populations #1, #2, and #4:<br>• Non-ovulating, pre-menstrual girls < 17 years of age;	Based on feedback from BRDD, the study populations of children and adolescents, adults with comorbidities,

#	Section	Change	Rationale
			and pregnant women will no longer be included in this study.
46	Section 5.1 (Inclusion Criteria)	The inclusion criterion #7 has been removed:<br>Study Population #5: Female subjects who are in the 3rd trimester of a low-risk pregnancy at Screening (Visit 1) can be enrolled in the study;	Based on feedback from BRDD, the study population pregnant women will no longer be included in this study.
47	Section 5.1 (Inclusion Criteria)	The Study Populations have been updated and the fourth bullet point and the last sentence of the fifth bullet point have been removed for inclusion criterion #8:<br>• Study Populations #1~~, #2, and #4~~:<br>• Vasectomised partner, provided that this partner is the sole sexual partner of the study participant and that the vasectomised partner has received a medical assessment of the surgical success;<br>• Ideally, the parent/LAR should not be present when a female subject < 18 years of age is reporting her sexual activity level and method of contraception	Based on feedback from BRDD, the study populations of children and adolescents, adults with comorbidities, and pregnant women will no longer be included in this study. In addition, the IRB recommended that the option of vasectomised partner be removed.
48	Section 5.1 (Inclusion Criteria)	The inclusion criteria #9 have been updated as follows:<br>Study Population #2~~3~~: Subjects must be non-institutionalized (e.g. not living in rehabilitation centres or old-age homes; living in an elderly community is acceptable) and have no acute or evolving medical problems prior to study participation and no clinically relevant abnormalities that could jeopardize subject safety or interfere with study assessments, as assessed by the Principal Investigator or sub-Investigator (thereafter referred as Investigator) and determined by medical history, physical examination,	Based on feedback from BRDD, the study populations of children and adolescents, adults with comorbidities, and pregnant women will no longer be

#	Section	Change	Rationale
		serology (only for the Phase 2 portion), clinical chemistry and haematology tests (only for the Phase 2 portion), urinalysis (only for the Phase 2 portion), and vital signs. Investigator discretion will be permitted with this inclusion criterion.<br>~~Note: Subjects with a pre-existing chronic disease will be allowed to participate if the disease is stable and, according to the Investigator's judgment that must be documented in the source documents, the condition is unlikely to confound the results of the study or pose additional risk to the subject by participating in the study. Stable disease is generally defined as no new onset or exacerbation of pre-existing chronic disease three months prior to vaccination. Based on the Investigator's judgment and documented in source documentation, a subject with more recent stabilization of a disease could also be eligible;~~	included in this study. Hence, the study population number was renumbered.
49	Section 5.1 (Inclusion Criteria)	The inclusion criterion #10 has been removed:<br>Study Population #4: Subjects must have one or more co-morbid conditions that puts them at higher risk for severe COVID-19 disease. These co-morbidities include but are not limited to obesity, documented hypertension, type-1 or type 2 diabetes, chronic obstructive lung diseases (COPD), cardiovascular disease, chronic kidney, or be immunocompromised persons (e.g., HIV-infected patients, organ transplant recipients, or patients receiving cancer chemotherapy). Investigator discretion will be permitted with this inclusion criterion.	Based on feedback from BRDD, the study population adults with comorbidities will no longer be included in this study.
50	Section 5.2 (Exclusion Criteria)	The Study Population assignment for all the exclusion criteria have been removed with the exception of exclusion criterion #15, for which the Study Populations have been updated as follows:<br>Study Populations #1~~, #2, and #4~~: Any female subject who has a positive or doubtful pregnancy test result prior to vaccination or who is lactating;	Based on feedback from BRDD, the study populations of children and adolescents, adults with comorbidities, and pregnant women will no longer be included in this study.

#	Section	Change	Rationale
51	Section 5.2 (Exclusion Criteria)	The second bullet point for exclusion criterion #1 has been updated as follows:<br>• Requiring any significant change in a chronic medication (i.e. drug, dose, frequency) during the three months prior to study vaccine administration due to uncontrolled symptoms or drug toxicity unless the innocuous nature of the medication change meets the criteria outlined in inclusion criterion no. 5 (Study Population #1) or no. **0**~~9~~ (Study Population #**2**~~3~~) and is appropriately justified and documented by the Investigator.	Based on feedback from BRDD, the study populations of children and adolescents, adults with comorbidities, and pregnant women will no longer be included in this study.
52	Section 5.2 (Exclusion Criteria)	A new exclusion criterion has been after exclusion criterion #1:<br>2. Any chronic medical condition associated with elevated risk of severe outcomes of COVID-19, including obesity, diabetes (type I/II), significant cardiovascular or respiratory disease including asthma, chronic renal failure, disorders of bleeding/coagulation, chronic inflammatory or autoimmune conditions, immunosuppressive conditions (including HIV), and hypertension;	Based on BRDD feedback, this exclusion criterion was added.
53	Section 5.2 (Exclusion Criteria)	The first sentence of the exclusion criterion #3 has been updated as follows:<br>~~Study Populations #1, #2, #3, and #45:~~ Current autoimmune disease (such as rheumatoid arthritis, systemic lupus erythematosus**,** ~~or~~ multiple sclerosis **or narcolepsy**).	Based on feedback from CBER for a different clinical protocol that includes AS03, this exclusion criterion was revised.
54	Section 5.2 (Exclusion Criteria)<br>AND<br>Section 5.4 (Prohibited Therapy)	The Study Populations and the first two bullets points for the exclusion criterion #4 have been updated as follows:<br>~~Study Populations #1, #2, #3 and #45:~~ Administration of any medication or treatment that may alter the vaccine immune responses, such as:<br>• ~~Study Populations #1, #3, and #5:~~ Systemic glucocorticoids at a dose exceeding 10 mg of prednisone (or equivalent) per day for more than seven consecutive days or for 10 or more days in total, within one month prior to the Vaccination visit (Visit 2). Inhaled, nasal,	Based on feedback from BRDD, the study populations of children and adolescents, adults with comorbidities, and pregnant women will no longer be included in this study.

#	Section	Change	Rationale
		ophthalmic, dermatological, and other topical glucocorticoids are permitted;<br>• Study Population #2: Systemic glucocorticoids at a dose exceeding 0.28 mg / kg of prednisone (or equivalent) per day for more than seven consecutive days or for ten or more days in total, within one month prior to the Vaccination visit (Visit 2). Inhaled, nasal, ophthalmic, dermatological, and other topical glucocorticoids are permitted;	
55	Section 5.2 (Exclusion Criteria)	The exclusion criterion #5 has been removed:<br>Study Population #4: Acute disease defined as presence of any moderate or severe acute illness with or without a fever within 48 hours prior to the Screening (Visit 1) and/or Vaccination visit (Visit 2);	Based on feedback from BRDD, the study populations of children and adolescents, adults with comorbidities, and pregnant women will no longer be included in this study.
56	Section 5.2 (Exclusion Criteria)<br>AND<br>Section 5.4 (Prohibited Therapy)	The text for exclusion criterion #6 has been updated as follows:<br>Administration of any vaccine within 30 **14** days prior to Vaccination (Visit 2); planned administration of any vaccine during the study (up to blood sampling on Day 42 **28** of the study). Immunization on an emergency basis during the study will be evaluated on case-by-case basis by the Investigator;	The restriction window was narrowed to support subject enrollment.
57	Section 5.2 (Exclusion Criteria)	A new exclusion criterion has been after exclusion criterion #13:<br>14. Family history of narcolepsy;	Based on feedback from CBER for a different clinical protocol that includes AS03, this exclusion criterion was added.
58	Section 5.2 (Exclusion Criteria)	The exclusion criterion #16 has been removed:	Based on feedback from BRDD, the study population of

#	Section	Change	Rationale
		Study Population #5: Any female subject who is in the 1st or 2nd trimester of her pregnancy or whose pregnancy is considered to be high-risk by her obstetrician;	pregnant women will no longer be included in this study.
59	Section 6.1 (Randomization)	The first paragraph has been updated as follows:<br>In the Phase 2 portion, subjects will be randomized **45**:1 to receive the CoVLP formulation and placebo in each Study Population. In the Phase 3 portion, subjects will be randomized 1:1 to receive the CoVLP formulation and placebo in each Study Population. Randomization of elderly adult subjects (Study Population #**2**~~3~~) will be stratified by age in a**n approximately** 2:1 ratio of 65 to 74 years of age and 75 years of age and older, respectively. ~~Randomization of healthy children and adolescents (Study Population #2) will be stratified by age in a 1:1 ratio of adolescents 10-17 years of age followed by children 5-9 years of age.~~ During Phase 2 and Phase 3 portions of the study, eligible subjects will be randomized at the Day 0 visit using the interactive response technology (IRT) system.	Based on feedback from BRDD, the study populations of children and adolescents, adults with comorbidities, and pregnant women will no longer be included in this study. In addition, the sample size for the healthy adults and elderly populations has been revised based on feedback from BRDD.
60	Section 8.2 (Collection of Samples)	The second and third paragraphs and Table 6 and Table 7 have been updated as follows:<br>All subjects in the Phase 2 portion of the study will have blood sampled. Subjects will have blood volumes drawn of up to approximately ~~287~~ **297** mL over a period of 386 days (Table 6).	Based on BRDD feedback, an immunogenicity (serology only) blood draw at 3 months post second vaccination has been included in each portion of the study.

Table 6 Estimated Blood Volume Drawn: Phase 2 Portion

Type of Sample	Volume per Sample (mL)	Number of Samples per Subject										Total Volume of Blood per Subject (mL)
		Visit 1 (Day -14 to 0)	Visit 2 (Day 0)	Visit 3 (Day 3)	Visit 4 (Day 21)	Visit 5 (Day 24)	Visit 6 (Day 42)	**Visit 7 (Day 128)**	Visit ~~7~~**8** (Day 201)	Visit ~~8~~**9** (Day 386)	Total	
Test for SARS-CoV-2 antibodies	5	1									1	5
Biochemistry, haematology, serology (HIV, hepatitis B & C, pregnancy test)*	8	1		1	1	1					4	32
Serology for immunogenicity (Nab assay, ELISA)	10		1		1		1	**1**	1	1	~~5~~**6**	~~50~~ **60**
CMI response (PBMC) ~~‡~~	~~20~~ 40		1		1		1		1	1	5	~~100~~ 200
Total volume of blood per subject (mL) ~~‡~~		13	~~30~~ 50	8	~~38~~ 58	8	~~30~~ 50	10	~~30~~ 50	~~30~~ 50		~~187~~ ~~287~~ **297**

* Samples collected during the screening visit should be performed under fasting conditions (approximately 12 hours) for cholesterol and triglyceride analysis. Note that cholesterol and triglyceride analysis will be performed only at screening.

~~‡Only a volume of 20 mL will be drawn at each applicable time-point from children < 12 years of age or weigh less than 40 kg (90 lbs).~~

In the Phase 3 portion of the study, all subjects will have 15 mL of blood drawn up to the end of the study (Day 386) and only a subset of subjects included in the immunogenicity analyses will have an additional blood volume of up to approximately ~~250~~ **260** mL drawn up to the end of the study (Day 386) (Table 7).

Table 7 Estimated Blood Volume Drawn: Phase 3 Portion

<table>
<tr><th>#</th><th>Section</th><th>Change</th><th>Rationale</th></tr>
<tr><td></td><td></td><td>
<table>
<tr><td rowspan="2">Type of Sample</td><td rowspan="2">Volume per Sample (mL)</td><td colspan="7">Number of Samples per Subject</td><td rowspan="2">Total Volume of Blood per Subject (mL)</td></tr>
<tr><td>Visit 1 (Day 0)</td><td>Visit 2 (Day 21)</td><td>Visit 3 (Day 42)</td><td>**Visit 4 (Day 128)**</td><td>Visit ~~4~~**5** (Day 201)</td><td>Visit ~~5~~**6** (Day 386)</td><td>Total</td></tr>
<tr><td>Test for SARS-CoV-2 antibodies</td><td>5</td><td>1</td><td></td><td></td><td></td><td>1</td><td>1</td><td>4</td><td>15</td></tr>
<tr><td>Serology for immunogenicity (Nab assay, ELISA)*</td><td>10</td><td>1</td><td>1</td><td>1</td><td>1</td><td>1</td><td>1</td><td>~~5~~**6**</td><td>~~50~~**60**</td></tr>
<tr><td>CMI response (PBMC)*‡</td><td>~~20~~ 40</td><td>1</td><td>1</td><td>1</td><td></td><td>1</td><td>1</td><td>5</td><td>~~100~~ 200</td></tr>
<tr><td>Total volume of blood per subject (mL)‡</td><td></td><td>~~30~~ 50</td><td>~~30~~ 50</td><td>~~30~~ 50</td><td>10</td><td>~~30~~ 50</td><td>~~30~~ 50</td><td></td><td>~~155~~ ~~265~~ **275**</td></tr>
</table>
* These samples will be collected for a pre-defined subset of subjects.
~~‡Only a volume of 20 mL will be drawn at each applicable time-point from children < 12 years of age or weigh less than 40 kg (90 lbs).~~
</td><td></td></tr>
<tr><td>61</td><td>Section 8.2 (Collection of Samples)</td><td>The fifth and sixth paragraphs have been updated as follows:
In the Phase 2 portion of the study, pregnancy testing will be performed for all females of childbearing potential (in Study Population~~s~~ #1~~, #2, and #4~~). A serum pregnancy test will be performed during screening. A urine (dipstick or similar) pregnancy test will be performed at Day 0 prior to randomization and at Days 21 and 42. In Study Population~~s~~ #1~~, #2, and #4~~, females of child bearing potential must have negative serum pregnancy test result from screening and urine pregnancy test result from Day 0 available prior to vaccination.
In the Phase 3 portion of the study, pregnancy testing will be performed for all females of childbearing potential (in Study Populations #1~~, #2, #4, and #5~~). A urine (dipstick or similar) pregnancy test will be performed at screening and at Day 0 prior to randomization and at Days 21 and 42. In Study Populations #1, ~~#2, and #4,~~ females of child bearing potential must have negative urine pregnancy test results from screening and Day 0 available prior to</td><td>Based on feedback from BRDD, the study populations of children and adolescents, adults with comorbidities, and pregnant women will no longer be included in this study. In addition, the sample size for the healthy adults and elderly populations has been revised</td></tr>
</table>

#	Section	Change	Rationale
		vaccination. ~~In Study Population #5, any female subject who is in the 1st or 2nd trimester of her pregnancy cannot participate in the study. However, any female subject who is in the 3rd trimester of a low-risk pregnancy can participate in the study.~~	based on feedback from BRDD.
62	Section 10.1.3 (Visit 1 for Phase 2 Portion and Phase 3 Portion)	The Study Populations listed for the secondary sub-bullet points of the fifth bullet point have been removed:<br>• Study Populations #1, #2, #3, and #5	Based on feedback from BRDD, the study populations of children and adolescents, adults with comorbidities, and pregnant women will no longer be included in this study. In addition, the sample size for the healthy adults and elderly populations has been revised based on feedback from BRDD.
63	Section 10.1.4.1 (Prior to Vaccination)	The fourth bullet point has been updated as follows:<br>• Collect a urine sample for female subjects of childbearing potential and perform a urine dipstick (or similar) pregnancy test. No study vaccine must be administered to a childbearing potential female until a negative result is obtained and documented~~, with the following exception:~~<br>• ~~Study Population #5: in the Phase 3 portion of the study, any female subject who is in the 1st or 2nd trimester of her pregnancy cannot participate in the study. However, any female subject who is in the 3rd trimester of her low-risk pregnancy can participate in the study~~;	Based on feedback from BRDD, the study population of pregnant women will no longer be included in this study.

#	Section	Change	Rationale
64	Section 10.1.4.2 (Vaccination)	The fourth sentence of the first paragraph has been updated as follows:<br>For ~~subjects < 18 years of age and adult~~ subjects with BMI < 30 kg/m^2, a 23 gauge needle of at least one inch or 2.5 cm in length can be used for vaccination.	Based on feedback from BRDD, the study populations of children and adolescents will no longer be included in this study.
65	Section 10.1.7 (Day 21 Dose 2 Vaccination)	The paragraph has been updated as follows:<br>If for any reason the Day 21 visit is done before or after the planned date, then subsequent visits/procedures (Days 22, 24 (Phase 2 portion only), 29, 42, **128,** 201, and 386) will be adjusted accordingly.	Based on BRDD feedback, an immunogenicity (serology only) blood draw at 3 months post second vaccination has been included in each portion of the study.
66	Section 10.1.7.1 (Pre-vaccination)	The second and third bullet points have been updated as follows:<br>• **Phase 2 portion only:** Perform urinalysis on all subjects;<br>• Perform urine (dipstick or similar) pregnancy testing for female subjects of childbearing potential ~~at screening~~. No study vaccine must be administered to a childbearing potential female until a negative result is obtained and documented~~, with the following exception:~~<br>• ~~Study Population #5: any female subject who is in the 1st or 2nd trimester of her pregnancy cannot participate in the study. However, any female subject who is in the 3rd trimester of her low-risk pregnancy can participate in the study~~;	To clarify the phase in which the test will be performed. In addition, the study population of pregnant women will no longer be included in this study based on feedback from BRDD.
67	Section 10.1.10 (Surveillance for COVID-19 Cases)	The first sentence of the first paragraph has been updated as follows:<br>From 14 days following the last vaccination, passive and active surveillance will be performed until the end of the Phase 2 or ~~until approximately 160 COVID-19 cases have been accumulated in~~ the Phase 3 portion of the study:	Clarified that subjects will be followed for efficacy until the end

#	Section	Change	Rationale
			of the study in the Phase 3 portion.
68	Section 10.1.11 (Day 42)	The last bullet point has been updated as follows:<br>• Provide appointments (date and time) for the next telephone contacts and the next planned visit to the clinical site (Day ~~201~~ **128**).	Based on BRDD feedback, an immunogenicity (serology only) blood draw at 3 months post second vaccination has been included in each portion of the study.
69	Section 10.1.12 (Day 201)	The content in Section 10.1.12 has been moved to Section 10.1.13 and the section title has been updated as follows:<br>Day 201 (Visit 7**8** for Phase 2 portion and Visit 4**5** for Phase 3 portion)<br>The following new content has been added to Section 10.1.12:<br>Day 128 (Visit 7 for Phase 2 portion and Visit 4 for Phase 3 portion)<br>The post-vaccination visit procedures will be performed by blinded site staff members at the clinical site. For subjects in quarantine, these procedures will be performed by blinded trained medical staff and may be at the subject's home (or place of quarantine). The following procedures will be performed during the Day 128 visit (± 14 days) for the Phase 2 and Phase 3 portions of the study:<br>• Review the memory aid content with the subject to ensure appropriate completion. Corrections must be made by the subject him/herself and all corrections must be initialed and dated;<br>• Review the subject's safety data and ensure all updates on concomitant medication usage and any changes in the subject's health (AEs ongoing from Day 42, SAEs, AESIs, or MAAEs) are recorded appropriately;	Based on BRDD feedback, an immunogenicity (serology only) blood draw at 3 months post second vaccination has been included in each portion of the study.

medicago

#	Section	Change	Rationale
		• Phase 2 portion only: perform a vital signs measurement, including resting BP, HR, RR, and OT;<br>• Phase 2 portion (all subjects) and Phase 3 portion (immunogenicity subset): collect blood samples for immunogenicity (serology [Nab assay and ELISA]) assessments; prepare and store these samples until shipment to the analytical laboratories;<br>• Remind each subject to immediately report to the clinical site any symptoms associated with COVID-19 or if he/she has tested positive for COVID-19, as defined in Section 10.4.1. Ensure that subjects have the memory aid listing the COVID-19 symptoms and contact information for the study site;<br>• Provide appointments (date and time) for the next telephone contacts and the next planned visit to the clinical site (Day 201).	
70	Section 10.1.13 (Final Visit – Day 386)	The content in Section 10.1.13 has moved to Section 10.1.14 and the section title has been updated as follows:<br>Day 128 (Visit ~~8~~**9** for Phase 2 portion and Visit ~~5~~**6** for Phase 3 portion)	Based on BRDD feedback, an immunogenicity (serology only) blood draw at 3 months post second vaccination has been included in each portion of the study.
71	Section 10.2.1.3 (Clinical Laboratory Tests)	The Study Population numbers have been updated as follows for the third sentence of the first paragraph and the last paragraph:<br>In addition, serum or urine samples from all females of childbearing potential in Study Population~~s~~ #1~~, #2, #4, and #5~~ for pregnancy testing will be collected.<br>AND<br>Other clinical laboratory tests include a test to measure SARS-CoV-2 antibodies at screening for all subjects in all Study Populations in the Phase 2	Based on feedback from BRDD, the study populations of children and adolescents, adults with comorbidities, and pregnant women

#	Section	Change	Rationale
		portion and at Days 0, 201, and 386 in the Phase 3 portion, and a serum (Phase 2 portion) or urine (Phase 3 portion) pregnancy test at screening and a urine (dipstick or similar) pregnancy test prior to randomization on Day 0 and on Days 21 and 42 for all female subjects of childbearing potential in Study Populations ~~s~~ #1~~, #2, #4, and #5~~.	will no longer be included in this study.
72	Section 10.2.1.4 (Vital Signs)	The last sentence of the first paragraph has been updated as follows:<br>In addition, vital signs measurements will be performed at the clinic site visits on Day 3, Day 24, Day 42, **Day 128,** Day 201, and Day 386 for the Phase 2 portion of the study. Vital signs measurements will only be performed prior to each vaccination during the Phase 3 portion.	Based on BRDD feedback, an immunogenicity (serology only) blood draw at 3 months post second vaccination has been included in each portion of the study.
73	Section 10.2.2 (Pregnancy)	The Study Population number has been updated as follows for the first sentence of the first paragraph and the last sentence of the second paragraph:<br>Female subjects who become pregnant during the Phase 2 or Phase 3 portions of the study ~~or are in the 3rd trimester of their low-risk pregnancy (Study Population #5 only) during the Phase 3 portion of the study~~ will be followed for safety.<br>AND<br>~~Information on pregnancies identified during the screening phase/prior to vaccine administration need to be collected for female subjects in the 3rd trimester of their pregnancy in Study Population #5 for the Phase 3 portion of the study.~~	Based on feedback from BRDD, the study population of pregnant women will no longer be included in this study.
74	Section 11.3 (Contraindications for Subsequent Vaccination)	The first paragraph and first bullet point have been updated as follows:<br>The following events constitute absolute contraindication to the further administration of the study treatments at Day 21; if any of these events occur during the study, the subject will not receive an additional dose of vaccine but will continue with the **Day 128 scheduled visit (see Section 10.1.12), the**	Based on BRDD feedback, an immunogenicity (serology only) blood draw at 3 months post second vaccination

#	Section	Change	Rationale
		Day 201 scheduled visit (see Section **10.1.13** ~~10.1.12~~)**,** and the Day 386 scheduled visit (see Section **10.1.14** ~~10.1.13~~) at the discretion of the Investigator:<br>• Pregnancy~~, with the exception of female subjects in the 3rd trimester of a low-risk pregnancy in Study Population #45 for the Phase 3 portion~~ (see Section 10.2.2);	has been included in each portion of the study.
75	Section 12.1.4 (Immunogenicity Per Protocol Set)	The first paragraph has been updated as follows:<br>The IPP set will consist of a subset of the ITT set who participated in the immunogenicity portion of the Phase 2 or Phase 3 portions of the study and completed the study with no major deviations related to subject eligibility, the ability to develop a valid immune response, prohibited medication use, or the immunogenicity analyses; and who received either the CoVLP formulation or placebo. For the Day 21 analysis, this should include the subjects who received the first vaccine dose and had Day 0 and Day 21 immunogenicity sample collections. For the Day 42 analysis, this should include the subjects who received both vaccine doses and had Day 0 and Day 42 immunogenicity sample collections. **For the Day 128 analysis, this should include the subjects who received both vaccine doses and had Day 0 and Day 128 immunogenicity sample collections.** For the Day 201 analysis, this should include the subjects who received both vaccine doses and had Day 0 and Day 201 immunogenicity sample collections. For the Day 386 analysis, this should include the subjects who received both vaccine doses and had Day 0 and Day 386 immunogenicity sample collections. Subjects who had blood samples for immunogenicity taken outside of the time window for blood sample collection are to be excluded from the IPP set for the specific visit. Subjects who received the wrong treatment at the first vaccination, but for whom the treatment received can be unequivocally confirmed, will be analyzed as treated, provided they have no other deviations that compromise their data. Subjects who receive a treatment at the second vaccination that is not the same treatment received at the first vaccination will not be included in the IPP	Based on BRDD feedback, an immunogenicity (serology only) blood draw at 3 months post second vaccination has been included in each portion of the study.

#	Section	Change	Rationale
		set at Day 42, **Day 128,** Day 201, and Day 386; the subjects will remain in the Day 21 analysis and analyzed as treated.	
76	Section 12.4 (Initiation of the Phase 3 Portion)	The third paragraph and the first sentence of the fourth paragraph have been updated as follows:<br>~~Study Population #2: If the safety and immunogenicity profiles of the CoVLP formulation in this population are acceptable, this group will also proceed into the Phase 3 portion. A key outcome will be the absence of a significant safety signal as assessed by the IDMC. As noted above, any important safety signal in this Study Population may trigger one or more mitigation strategies prior to entry into the Phase 3 portion and the younger and older cohorts within this Study Population (5-9 and 10-17 year old) may enter into the Phase 3 portion asynchronously (outlined in Figure 1).~~<br>Study Population~~s~~ #**2**~~3 and #4~~: If the safety and immunogenicity profiles of the CoVLP formulation in this population are acceptable, entry of ~~these two~~ Study Population~~s~~ **#2** into the Phase 3 portion will be based on both the absence of an important safety signal and demonstration of an acceptable immune response as assessed by the IDMC.	Based on feedback from BRDD, the study populations of children and adolescents, adults with comorbidities, and pregnant women will no longer be included in this study.
77	Section 12.6.2 (Analysis of Secondary Endpoints)	The second, third, and fourth paragraphs have been updated as follows:<br>For the Phase 2 portion: the following analyses for the NAb assay will be performed on the immunogenicity PP set:<br>• GMT (Days **128,** 201**,** and 386): The point estimates and the corresponding two-sided 95 % CI by treatment group will be calculated as the antilog of the mean and 95 % CI of log transformed titer values;<br>• SC rate (Days **128,** 201**,** and 386): The point estimates and the corresponding two-sided 95 % CI for subjects achieving SC by treatment group will be calculated and reported;<br>• GMFR: the geometric mean of the ratio of GMTs (**Day 128/Day 0,** Day 201/Day 0**,** and Day 386/Day 0).	Based on BRDD feedback, an immunogenicity (serology only) blood draw at 3 months post second vaccination has been included in each portion of the study.

#	Section	Change	Rationale
		For the analyses of the total IgG antibody response, the GMT and SC rate will be calculated on Day 0, Day 21, Day 42, **Day 128,** Day 201, and Day 386 as well as the GMFR (Day 21/Day 0, Day 42/Day 0, **Day 128/Day 0,** Day 201/Day 0, and Day 386/Day 0).<br>The GMFR will be derived by using ANCOVA to model the difference in the log of the titer values between Day 21 and Day 0, between Day 42 and Day 0, **Day 128 and Day 0,** Day 201 and Day 0, and Day 386 and Day 0 with treatment group as main effect and baseline titer as covariate.	
78	Section 13.1.3.1.1 (Vaccine Enhanced Disease)	The third paragraph has been removed:<br>~~A sub-optimal immune response after vaccination may increase the risk of VED in subjects who receive a vaccine formulation retrospectively considered to be "sub-optimal" (i.e. subjects who received a lower dose level/regimen and/or non-adjuvanted formulation compared to the final formulation determined to be safe and effective). These subjects (and/or their parent/LAR) will be informed that they/their child may be eligible to participate in a potential extension study of the CoVLP formulation and in this study, they/their child will be administered the final vaccine formulation.~~	To remove content that is not relevant to the study as the relevant mitigation has been included in Section 16.1 of the protocol.
79	Section 13.1.11 (Safety Review and Stopping Rules)	The second paragraph under the Stopping Rules heading has been updated as follows:<br>For sentinel subjects in Study Population~~s~~ #2~~, #3, and #4~~ during the Phase 2 portion of the study:	Based on feedback from BRDD, the study populations of children and adolescents, adults with comorbidities, and pregnant women will no longer be included in this study.
80	Section 14.1.3 (Preparation and Administration of Study Vaccine)	The second sentence of the third paragraph has been updated as follows:<br>For ~~subjects < 18 years of age and adult~~ subjects with BMI < 30 kg/m^2, a 23 gauge needle of at least one inch or 2.5 cm in length can be used for vaccination.	Based on feedback from BRDD, the study population of children and adolescents will no

#	Section	Change	Rationale
			longer be included in this study.
81	Section 18 (References)	The following references have been removed from the protocol:<br>Baud D., Greub G., Favre G. Second-trimester miscarriage in a pregnant woman with SARS-CoV-2 infection. JAMA. 2020;323(21):2198–2200.<br>Bravo LC, Carlos JC, Gatchalian SR, Montellano MEB, Tabora CFCB, Thierry-Carstensen B, Tingskov PN, Sørensen C, Wachmann H, Bandyopadhyay AS, Nielsen PI, Kusk MV. Immunogenicity and safety of an adjuvanted inactivated polio vaccine, IPV-Al, compared to standard IPV: A phase 3 observer-blinded, randomised, controlled trial in infants vaccinated at 6, 10, 14 weeks and 9 months of age. Vaccine. 2020 Jan 16;38(3):530-538. doi: 10.1016/j.vaccine.2019.10.064.<br>Buonsenso D, Costa S, Sanguinetti M, Cattani P, Posteraro B, Marchetti S, Carducci B, Lanzone A, Tamburrini E, Vento G, Valentini P. Neonatal Late Onset Infection with Severe Acute Respiratory Syndrome Coronavirus 2. Am J Perinatol. 2020 Jun;37(8):869-872. doi<br>Cai J, Xu J, Lin D, Yang Z, Xu L, Qu Z, Zhang Y, Zhang H, Jia R, Liu P, Wang X, Ge Y, Xia A, Tian H, Chang H, Wang C, Li J, Wang J, Zeng M. A Case Series of children with 2019 novel coronavirus infection: clinical and epidemiological features. Clin Infect Dis. 2020 Feb 28:ciaa198. doi: 10.1093/cid/ciaa198.<br>CDC COVID-19 Response Team. Coronavirus Disease 2019 in Children - United States, February 12-April 2, 2020. MMWR Morb Mortal Wkly Rep. 2020a Apr 10;69(14):422-426. doi: 10.15585/mmwr.mm6914e4.<br>Cruz AT, Zeichner SL. COVID-19 in Children: Initial Characterization of the Pediatric Disease. Pediatrics. 2020 Jun;145(6):e20200834. doi: 10.1542/peds.2020-0834.<br>Di Mascio D, Khalil A, Saccone G. Outcome of coronavirus spectrum infections (SARS, MERS, COVID 1-19) during pregnancy: a	Based on feedback from BRDD, the study populations of children and adolescents, adults with comorbidities, and pregnant women will no longer be included in this study. The background information discussing these populations has been removed and these references are no longer needed in the protocol.

#	Section	Change	Rationale
		systematic review and meta-analysis. Am J Obstet Gynecol MFM. 2020 doi: 10.1016/j.ajogmf.2020.100107. published online March 25.<br>Djennad A, Ramsay ME, Pebody R, Fry NK, Sheppard C, Ladhani SN, Andrews NJ. Effectiveness of 23-Valent Polysaccharide Pneumococcal Vaccine and Changes in Invasive Pneumococcal Disease Incidence from 2000 to 2017 in Those Aged 65 and Over in England and Wales. EClinicalMedicine. 2019 Jan 2;6:42-50. doi: 10.1016/j.eclinm.2018.12.007.<br>Dong Y, Mo X, Hu Y, Qi X, Jiang F, Jiang Z, Tong S. Epidemiology of COVID-19 Among Children in China. Pediatrics. 2020 Jun;145(6):e20200702. doi: 10.1542/peds.2020-0702.<br>Dowling DJ, Levy O. Pediatric Vaccine Adjuvants: Components of the Modern Vaccinologist's Toolbox. Pediatr Infect Dis J. 2015 Dec;34(12):1395-8. doi: 10.1097/INF.0000000000000893.<br>FDA. Guidance for Industry: Development and licensure of vaccines to prevent COVID-19. 2020.<br>Feldstein LR, Rose EB, Horwitz SM, Collins JP, Newhams MM, Son MBF, Newburger JW, Kleinman LC, Heidemann SM, Martin AA, Singh AR, Li S, Tarquinio KM, Jaggi P, Oster ME, Zackai SP, Gillen J, Ratner AJ, Walsh RF, Fitzgerald JC, Keenaghan MA, Alharash H, Doymaz S, Clouser KN, Giuliano JS Jr, Gupta A, Parker RM, Maddux AB, Havalad V, Ramsingh S, Bukulmez H, Bradford TT, Smith LS, Tenforde MW, Carroll CL, Riggs BJ, Gertz SJ, Daube A, Lansell A, Coronado Munoz A, Hobbs CV, Marohn KL, Halasa NB, Patel MM, Randolph AG; Overcoming COVID-19 Investigators and the CDC COVID-19 Response Team. Multisystem Inflammatory Syndrome in U.S. Children and Adolescents. N Engl J Med. 2020 Jul 23;383(4):334-346. doi: 10.1056/NEJMoa2021680.<br>Galang RR, Chang K, Strid P, Snead MC, Woodworth KR, House LD, Perez M, Barfield WD, Meaney-Delman D, Jamieson DJ, Shapiro-Mendoza CK, Ellington SR. Severe Coronavirus Infections in Pregnancy: A Systematic Review. Obstet Gynecol. 2020 Aug;136(2):262-272.	

#	Section	Change	Rationale
		Garcia-Sicilia J, Arístegui J, Omeñaca F, Carmona A, Tejedor JC, Merino JM, García-Corbeira P, Walravens K, Bambure V, Moris P, Caplanusi A, Gillard P, Dieussaert I. Safety and persistence of the humoral and cellular immune responses induced by 2 doses of an AS03-adjuvanted A(H1N1)pdm09 pandemic influenza vaccine administered to infants, children and adolescents: Two open, uncontrolled studies. Hum Vaccin Immunother. 2015;11(10):2359-69. doi: 10.1080/21645515.2015.1063754.<br>Guan WJ, Liang WH, Zhao Y, Liang HR, Chen ZS, Li YM, Liu XQ, Chen RC, Tang CL, Wang T, Ou CQ, Li L, Chen PY, Sang L, Wang W, Li JF, Li CC, Ou LM, Cheng B, Xiong S, Ni ZY, Xiang J, Hu Y, Liu L, Shan H, Lei CL, Peng YX, Wei L, Liu Y, Hu YH, Peng P, Wang JM, Liu JY, Chen Z, Li G, Zheng ZJ, Qiu SQ, Luo J, Ye CJ, Zhu SY, Cheng LL, Ye F, Li SY, Zheng JP, Zhang NF, Zhong NS, He JX; China Medical Treatment Expert Group for COVID-19. Comorbidity and its impact on 1590 patients with COVID-19 in China: a nationwide analysis. Eur Respir J. 2020a May 14;55(5):2000547. doi: 10.1183/13993003.00547-2020.<br>Hachem R, Markou GA, Veluppillai C, Poncelet C. Late miscarriage as a presenting manifestation of COVID-19. Eur J Obstet Gynecol Reprod Biol. 2020 Sep;252:614.<br>Korakas E, Ikonomidis I, Kousathana F, Balampanis K, Kountouri A, Raptis A, Palaiodimou L, Kokkinos A, Lambadiari V. Obesity and COVID-19: immune and metabolic derangement as a possible link to adverse clinical outcomes. Am J Physiol Endocrinol Metab. 2020 Jul 1;319(1):E105-E109.<br>Kwetkat A, Heppner HJ. Comorbidities in the Elderly and Their Possible Influence on Vaccine Response. Interdiscip Top Gerontol Geriatr. 2020;43:73-85. doi: 10.1159/000504491.<br>Liu W, Zhang Q, Chen J, Xiang R, Song H, Shu S, Chen L, Liang L, Zhou J, You L, Wu P, Zhang B, Lu Y, Xia L, Huang L, Yang Y, Liu F, Semple MG, Cowling BJ, Lan K, Sun Z, Yu H, Liu Y. Detection of	

#	Section	Change	Rationale
		Covid-19 in Children in Early January 2020 in Wuhan, China. N Engl J Med. 2020b Apr 2;382(14):1370-1371. doi: 10.1056/NEJMc2003717.<br>Ludvigsson JF. Systematic review of COVID-19 in children shows milder cases and a better prognosis than adults. Acta Paediatr. 2020 Jun;109(6):1088-1095. doi: 10.1111/apa.15270.<br>Manzoli L, Ioannidis JP, Flacco ME, De Vito C, Villari P. Effectiveness and harms of seasonal and pandemic influenza vaccines in children, adults and elderly: a critical review and re-analysis of 15 meta-analyses. Hum Vaccin Immunother. 2012 Jul;8(7):851-62. doi: 10.4161/hv.19917.<br>Mehan A, Venkatesh A, Girish M. COVID-19 in pregnancy: Risk of adverse neonatal outcomes. J Med Virol. 2020 Apr 30:10.1002/jmv.25959.<br>Mehra MR, Desai SS, Kuy S, Henry TD, Patel AN. Cardiovascular Disease, Drug Therapy, and Mortality in Covid-19. N Engl J Med. 2020 Jun 18;382(25):e102. doi: 10.1056/NEJMoa2007621.<br>Nath KD, Burel JG, Shankar V, Pritchard AL, Towers M, Looke D, Davies JM, Upham JW. Clinical factors associated with the humoral immune response to influenza vaccination in chronic obstructive pulmonary disease. Int J Chron Obstruct Pulmon Dis. 2014;9:51-6. doi: 10.2147/COPD.S53590.<br>Rasmussen SA, Smulian JC, Lednicky JA, Wen TS, Jamieson DJ. Coronavirus Disease 2019 (COVID-19) and pregnancy: what obstetricians need to know. Am J Obstet Gynecol. 2020 May;222(5):415-426.<br>Sanyaolu A, Okorie C, Marinkovic A, Patidar R, Younis K, Desai P, Hosein Z, Padda I, Mangat J, Altaf M. Comorbidity and its Impact on Patients with COVID-19. SN Compr. Clin. Med. 2020. doi.org/10.1007/s42399-020-00363-4<br>Shen KL, Yang YH, Jiang RM, Wang TY, Zhao DC, Jiang Y, Lu XX, Jin RM, Zheng YJ, Xu BP, Xie ZD, Liu ZS, Li XW, Lin LK, Shang YX, Shu SN, Bai Y, Lu M, Lu G, Deng JK, Luo WJ, Xiong LJ, Liu M, Cui YX, Ye LP, Li JF, Shao JB, Gao LW, Wang YY, Wang XF; China	

#	Section	Change	Rationale
		National Clinical Research Center for Respiratory Diseases; National Center for Children's Health, Beijing, China; Group of Respirology, Chinese Pediatric Society, Chinese Medical Association; Chinese Medical Doctor Association Committee on Respirology Pediatrics; China Medicine Education Association Committee on Pediatrics; Chinese Research Hospital Association Committee on Pediatrics; China Non-government Medical Institutions Association Committee on Pediatrics; China Association of Traditional Chinese Medicine, Committee on Children's Health and Medicine Research; China News of Drug Information Association, Committee on Children's Safety Medication; Global Pediatric Pulmonology Alliance. Updated diagnosis, treatment and prevention of COVID-19 in children: experts' consensus statement (condensed version of the second edition). World J Pediatr. 2020 Jun;16(3):232-239. doi: 10.1007/s12519-020-00362-4.<br>Vardeny O, Moran JJ, Sweitzer NK, Johnson MR, Hayney MS. Decreased T-cell responses to influenza vaccination in patients with heart failure. Pharmacotherapy. 2010 Jan;30(1):10-6. doi: 10.1592/phco.30.1.10.<br>Verschoor CP, Lelic A, Parsons R, Evelegh C, Bramson JL, Johnstone J, Loeb MB, Bowdish DME. Serum C-Reactive Protein and Congestive Heart Failure as Significant Predictors of Herpes Zoster Vaccine Response in Elderly Nursing Home Residents. J Infect Dis. 2017 Jul 15;216(2):191-197. doi: 10.1093/infdis/jix257.<br>Vesikari T, Pepin S, Kusters I, Hoffenbach A, Denis M. Assessment of squalene adjuvanted and non-adjuvanted vaccines against pandemic H1N1 influenza in children 6 months to 17 years of age. Hum Vaccin Immunother. 2012 Sep;8(9):1283-92. doi: 10.4161/hv.21265.<br>Wang B, Li R, Lu Z, Huang Y. Does comorbidity increase the risk of patients with COVID-19: evidence from meta-analysis. Aging (Albany NY). 2020 Apr 8;12(7):6049-6057. doi: 10.18632/aging.103000.<br>Zimmermann P, Curtis N. Factors That Influence the Immune Response to Vaccination. Clin Microbiol Rev. 2019 Mar 13;32(2):e00084-18. doi: 10.1128/CMR.00084-18.	

<table>
<tr><th>#</th><th>Section</th><th>Change</th><th>Rationale</th></tr>
<tr><td></td><td></td><td></td><td></td></tr>
<tr><td>82</td><td>Section 19.5 (Appendix 5 – Symptoms Risk Factors, and Disease Severity of COVID-19)</td><td>In Table 10, the following statement has been removed under the Clinical Presentation sub-heading:
Children might not have reported fever or cough as frequently as adults.</td><td>Based on feedback from BRDD, the study population of children and adolescents will no longer be included in this study. Content regarding this population has been removed.</td></tr>
<tr><td>83</td><td>Section 19.5 (Appendix 5 – Symptoms Risk Factors, and Disease Severity of COVID-19)</td><td>Table 11 has been updated as follows:
Table 11 COVID-19 Disease Severity
<table>
<tr><td>Mild Disease</td><td></td><td>Symptomatic patients (Table 10) meeting the case definition for COVID-19 without evidence of viral pneumonia or hypoxia.</td></tr>
<tr><td>Moderate Disease</td><td>Pneumonia</td><td>~~Adolescent or a~~**A**dult with clinical signs of pneumonia (fever, cough, dyspnoea, fast breathing) but no signs of severe pneumonia, including $SpO_2 \geq 90\%$ on room air.
~~Child with clinical signs of non-severe pneumonia (cough or difficulty breathing + fast breathing and/or chest indrawing) and no signs of severe pneumonia.~~
~~Fast breathing (in breaths/min): < 2 months: ≥ 60; 2–11 months: ≥ 0;~~
~~1–5 years: ≥40.~~
While the diagnosis can be made on clinical grounds; chest imaging (radiograph, CT scan, ultrasound) may assist in diagnosis and identify or exclude pulmonary complications.</td></tr>
<tr><td>Severe Disease</td><td>Severe Pneumonia</td><td>~~Adolescent or a~~**A**dult with clinical signs of pneumonia (fever, cough, dyspnoea, fast breathing) plus one of the following: respiratory rate</td></tr>
</table></td><td>Based on feedback from BRDD, the study population of children and adolescents will no longer be included in this study. Content regarding this population has been removed.</td></tr>
</table>

#	Section	Change			Rationale
				> 30 breaths/min; severe respiratory distress; or $SpO2 < 90\%$ on room air.<br>~~Child with clinical signs of pneumonia (cough or difficulty in breathing) + at least one of the following:~~<br>• ~~Central cyanosis or SpO2 < 90%; severe respiratory distress (e.g. fast breathing, grunting, very severe chest indrawing); general danger sign: inability to breastfeed or drink, lethargy or unconsciousness, or convulsions.~~<br>• ~~Fast breathing (in breaths/min): < 2 months: ≥60; 2–11 months: ≥ 50;~~<br>~~1–5 years: ≥40.~~<br>While the diagnosis can be made on clinical grounds; chest imaging (radiograph, CT scan, ultrasound) may assist in diagnosis and identify or exclude pulmonary complications.	
		Critical Disease	Acute Respiratory Distress Syndrome (ARDS)	Onset: within 1 week of a known clinical insult (i.e. pneumonia) or new or worsening respiratory symptoms.<br>Chest imaging: (radiograph, CT scan, or lung ultrasound): bilateral opacities, not fully explained by volume overload, lobar or lung collapse, or nodules.<br>Origin of pulmonary infiltrates: respiratory failure not fully explained by cardiac failure or fluid overload. Need objective assessment (e.g. echocardiography) to exclude hydrostatic cause of infiltrates/oedema if no risk factor present.<br>Oxygenation impairment in adults:<br>• Mild ARDS: 200 mmHg < PaO_2/FiO_2[a] ≤ 300 mmHg (with PEEP or<br>CPAP ≥ 5 cm H_2O).[b]<br>• Moderate ARDS: 100 mmHg < PaO_2/FiO_2 ≤ 200 mmHg (with<br>PEEP ≥ 5 cmH_2O).[b]<br>• Severe ARDS: PaO_2/FiO_2 ≤ 100 mm Hg (with PEEP ≥ 5 cmH_2O).[b]	

#	Section	Change			Rationale
				~~Oxygenation impairment in children: note OI and OSI.[e] Use OI when available. If PaO_2 not available, wean FiO_2 to maintain $SpO_2 \leq 97\%$ to calculate OSI or SpO_2/FiO_2 ratio:~~<br>• ~~Bilevel (NIV or CPAP) ≥ 5 cmH2O via full face mask: $PaO_2/FiO_2 \leq 300$ mmHg or $SpO_2/FiO_2 \leq 264$.~~<br>• ~~Mild ARDS (invasively ventilated): $4 \leq OI < 8$ or $5 \leq OSI < 7.5$.~~<br>• ~~Moderate ARDS (invasively ventilated): $8 \leq OI < 16$ or $7.5 \leq OSI < 12.3$.~~<br>• ~~Severe ARDS (invasively ventilated): $OI \geq 16$ or $OSI \geq 12.3$.~~	
		Critical Disease	Sepsis	Adults: acute life-threatening organ dysfunction caused by a dysregulated host response to suspected or proven infection. Signs of organ dysfunction include: altered mental status, difficult or fast breathing, low oxygen saturation, reduced urine output (3), fast heart rate, weak pulse, cold extremities or low blood pressure, skin mottling, laboratory evidence of coagulopathy, thrombocytopenia, acidosis, high lactate, or hyperbilirubinemia.<br>~~Children: suspected or proven infection and ≥2 age_-based systemic inflammatory response syndrome (SIRS) criteria[e] of which one must be abnormal temperature or white blood cell count.~~	
			Septic Shock	Adults: persistent hypotension despite volume resuscitation, requiring vasopressors to maintain MAP ≥ 65 mmHg and serum lactate level > 2 mmol/L.<br>~~Children: any hypotension (SBP < 5th centile or > 2 SD below normal for age) or two or three of the following: altered mental status; bradycardia or tachycardia (HR < 90 bpm or > 160 bpm in infants and heart rate < 70 bpm or >150 bpm in children); prolonged capillary refill (> 2 sec) or weak pulse; fast breathing; mottled or cool skin or petechial or purpuric rash; high lactate; reduced urine output; hyperthermia or hypothermia.~~	
		Other complications that have been described in COVID-19 patients include acute, life-threatening conditions such as: acute pulmonary embolism, acute coronary syndrome,			

medicago

#	Section	Change	Rationale
		acute stroke and delirium. Clinical suspicion for these complications should be heightened when caring for COVID-19 patients, and appropriate diagnostic and treatment protocols available.<br>[a] If altitude is higher than 1000 m, then the correction factor should be calculated as follows: PaO_2/FiO_2 x barometric pressure/760.<br>[b] When PaO_2 is not available, $SpO_2/FiO_2 \leq 315$ suggests ARDS (including in non-ventilated patients).<br>~~[c] Oxygenation Index (OI) is an invasive measurement of the severity of hypoxaemic respiratory failure and may be used to predict outcomes in paediatric patients. It is calculated as follows: percentage of fraction of inhaled oxygen multiplied by the mean airway pressure (in mmHg), divided by the partial pressure of arterial oxygen (in mmHg). Oxygen saturation index (OSI) is a non-invasive measurement and has been shown to be a reliable surrogate marker of OI in children and adults with respiratory failure. OSI replaces PaO_2 with oxygen saturation as measured by pulse oximetry (SpO_2) in the OI equation.~~<br>~~[d] The SOFA score ranges from 0 to 24 and includes points related to six organ systems: respiratory (hypoxaemia defined by low PaO_2/FiO_2); coagulation (low platelets); liver (high bilirubin); cardiovascular (hypotension); central nervous system (low level of consciousness defined by Glasgow Coma Scale); and renal (low urine output or high creatinine). Sepsis is defined by an increase in the sepsis-related SOFA score of ≥ 2 points. Assume the baseline score is 0 if data are not available.~~<br>~~[e] SIRS criteria: abnormal temperature (> 38.5 °C or < 36 °C); tachycardia for age or bradycardia for age if < 1 year; tachypnoea for age or need for mechanical ventilation; abnormal white blood cell count for age or > 10% bands.~~<br>Abbreviations: ~~BP blood pressure; bpm beats per minute;~~ CPAP continuous positive airway pressure; CT computed tomography; FiO_2 fraction of inspired oxygen; MAP mean arterial pressure; ~~NIV non-invasive ventilation;~~ ~~OI Oxygenation Index;~~ OSI Oxygenation Index using SpO_2; PaO_2 partial pressure arterial oxygen; PEEP positive end-expiratory pressure; ~~SBP systolic blood pressure;~~ SD standard deviation;<br>Source: [WHO 2020a]	
84	Section 19.6 (Appendix 6 – COVID-19 Diary Sample Pages)	The COVID-19 Symptoms table and assessment of common COVID-19-related symptoms page have been updated to remove reference to:<br>• For children and adolescents<br>• Abdominal pain<br>• Poor appetite	Based on feedback from BRDD, the study population of children and adolescents will no

medicago

#	Section	Change	Rationale
			longer be included in this study.

Table 10 Summary of Changes from Protocol Version 1.0 to Version 2.0 (Effective Date 06 October 2020)

#	Section	Change	Rationale
1.	Synopsis (Study Rationale)<br><br>AND<br><br>Section 1.5 (Overall Rationale for the Study)	The first sentence of the fourth paragraph in this section has been updated as follows:<br>The Phase 3 portion is an event-driven, randomized, observer-blinded, placebo-controlled design that will evaluate the efficacy and safety of the CoVLP formulation, compared to placebo, in the ~~four~~ Study Populations **#1 - #4** presented above **and a possible fifth Study Population of healthy female adults who are in the 3rd trimester of their low-risk pregnancy**.	Based on feedback from BRDD, pregnant females in the 3rd trimester may be included in a separate cohort during the Phase 3 portion of the study as Study Population #5.
2.	Synopsis (Study Rationale)<br>AND<br>Section 1.5 (Overall Rationale for the Study)<br>AND<br>Synopsis (Number of Planned Subjects)<br>AND<br>Synopsis (Sample Size Determination)<br>AND<br>Section 12.2 (Sample Size Determination)<br>AND	The immunogenicity subset sample size has been increased from 384 to 480 subjects in the following sections:<br><br>Immunogenicity will only be evaluated in a subset of ~~384~~ **480** subjects.<br><br>Only a subset of approximately ~~384~~ **480** subjects (96 subjects from each Study Population) will be included in the immunogenicity evaluation.<br><br>An immunogenicity subset sample size of ~~384~~ **480** subjects is deemed sufficient to provide meaningful statistical assessment of immunologic outcomes.	The subset sample increased due to the addition of Study Population #5 in the Phase 3 portion.

#	Section	Change	Rationale
	Synopsis (Immunogenicity Evaluations)	Immunogenicity will be evaluated in all subjects in the Phase 2 portion and in a subset of ~~384~~ **480** subjects in the Phase 3 portion of the study across the Study Populations with age distribution similar to the Phase 2 portion.	
3.	Synopsis (Study Rationale)<br>AND<br>Section 1.5 (Overall Rationale for the Study)	The following paragraph has been added after the fifth paragraph:<br>Risk:benefit considerations are different for each of these five study populations and will influence how these populations are included in the Phase 2 portion of the study and their relative proportions included in the Phase 3 portion. Population-specific mitigation strategies will be incorporated into the transition between the Phase 2 and Phase 3 portions.	Based on feedback from BRDD, risk:benefit considerations for each Study Population has been included.
4.	Synopsis (Secondary Objectives)<br>AND<br>Section 2.2 (Secondary Objectives)	The first two sub-bullets for the Phase 2 portion immunogenicity secondary objectives have been revised as follows:<br>• **Persistence of** ~~N~~**n**eutralizing antibody titers induced by the vaccine against the SARS-CoV-2 virus at Days 201 and Day 386;<br>• IgG (enzyme-linked immunosorbent assay [ELISA]) titers induced by the vaccine against the SARS-CoV-2 virus up to 21 days after the last vaccination and **persistence of these antibodies** at Day 201 and Day 386;	To further clarify the two secondary immunogenicity objectives.
5.	Synopsis (Secondary Objectives)<br>AND<br>Section 2.2 (Secondary Objectives)	The following two efficacy secondary objectives have been added to the Phase 3 portion:<br>• To evaluate the duration of protection (vaccine efficacy) of the CoVLP formulation compared to placebo;	Based on feedback from BRDD, two new efficacy objectives were added to the Phase 3 portion.

#	Section	Change	Rationale
		• To evaluate the absolute efficacy of the CoVLP formulation, compared to placebo, in the prevention of laboratory-confirmed asymptomatic SARS-CoV-2 infection (serologic method) at Day 201 and Day 386;	
6.	Synopsis (Secondary Objectives)<br>AND<br>Section 2.2 (Secondary Objectives)	The following immunogenicity exploratory objectives have been moved to secondary objectives for the Phase 3 portion and combined:<br>• In a subset of subjects, neutralizing antibody titers induced by the vaccine against the SARS-CoV-2 virus up to 21 days after the last vaccination and at Day 201 and Day 386;<br>• In a subset of subjects, IgG (ELISA) titers induced by the vaccine against the SARS-CoV-2 virus up to 21 days after the last vaccination and at Day 201 and Day 386;<br>• In a subset of subjects, the ratio of neutralizing antibody titers:IgG (ELISA) antibody titers at Day 21, Day 42, Day 201, and Day 386;<br>• In a subset of subjects, IFN-γ ELISpot response induced by the vaccine against the SARS-CoV-2 virus up to 21 days after the last vaccination and at Day 201 and Day 386;<br>• In a subset of subjects, IL-4 ELISpot response induced by the vaccine against the SARS-CoV-2 virus up to 21 days after the last vaccination and at Day 201 and Day 386;<br>• In a subset of subjects, specific cell-mediated immune (CMI) response induced by the vaccine against the SARS-CoV-2 virus up to 21 days after the last	For consistency with the Phase 2 portion, these immunogenicity objectives are now secondary objectives.

#	Section	Change	Rationale
		vaccination and at Day 201 and Day 386, as measured by the percentage of CD4+ T cells expressing functional markers;	
7.	Synopsis (Sample Size Determination)<br>AND<br>Section 12.2 (Sample Size Determination)	The third and fifth bullets below the Phase 3 portion header have been updated as following:<br>• A SARS-CoV-2 attack rate **(i.e. symptomatic disease) in unvaccinated subjects** of 0.8 % **across all study populations**;<br>• An interim analysis for an early efficacy assessment will be performed when 80 laboratory-confirmed **symptomatic** COVID-19 cases have been collected in the Phase 3 portion;	To further clarify the details for determining the sample size for the Phase 3 portion.
8.	Synopsis (Study Population)	The following statement has been added to the end of the paragraph:<br>In addition, healthy female adults 18 years of age or older who are in the 3rd trimester of their low-risk pregnancy will be included in the Phase 3 portion of the study.	Based on feedback from BRDD, pregnant females in the 3rd trimester may be included in a separate cohort during the Phase 3 portion of the study as Study Population #5.
9.	Synopsis (Dosage and Administration)<br>AND<br>Section	This section has been updated as follows:<br>**The CoVLP formulation selected for** ~~In~~ the Phase 2 and Phase 3 portions of the study~~, the CoVLP formulation~~ has been selected based on the Phase 1 study data, including acceptable safety and immunogenicity profiles.<br>~~Subjects in each of the four Study Populations will receive the same CoVLP formulation or placebo.~~<br>Subjects will receive two intramuscular (IM) injections 21 days apart on Days 0 and 21, into the deltoid region of the alternating arm (each arm will be injected once), of one of the following:<br>• CoVLP formulation;<br>• Placebo.	Based on feedback from GSK, clarified the dose of the treatment to be administered to children. In addition, based on feedback from BRDD, included dose mitigation strategies for children/adolescents population and the elderly/comorbidities population.

#	Section	Change	Rationale
		~~The volume of each injection will be 0.5 mL.~~<br>**The use of the same dose of the CoVLP formulation for all Study Populations may not be adequate. Subjects in Study Population #1 (healthy adults 18 to 64 years of age) will receive the full standard dose of CoVLP (3.75 µg) adjuvanted with AS03 in an injection volume of 0.5 mL.**<br>**In Study Population #2, subjects who are 10 to 17 years of age will receive 3.75 µg CoVLP adjuvanted with AS03 in 0.5 mL. Subjects who are 5-9 years of age will receive half the dose of the CoVLP formulation (i.e. 0.25 mL containing 1.87 µg CoVLP adjuvanted with half the standard dose of AS03).**<br>**For the adolescents in Study Population #2, a dose de-escalation may occur to reduce the dose of CoVLP and AS03 by half (i.e. 0.25 mL of the full dose will be administered containing 1.87 mg CoVLP and half of the AS03 dose). In the event that the IDMC considers the safety profile to be acceptable but the immunogenicity profile is not acceptable, then this population will proceed to the second strategy of administering the standard antigen dose (i.e. 3.75 µg) with half the adjuvant dose in a 0.25 mL volume. If the safety profile is acceptable to the IDMC, then recruitment will continue until a total of 60 adolescents and 60 children receiving the same CoVLP formulation in each age cohort have been enrolled in Study Population #2. If the half dose of the CoVLP formulation is used in the Phase 2 portion for one or both age cohorts in Study Population #2, then the same formulation(s) will be used during the Phase 3 portion of the study.**<br>**Study Populations #3 and #4 (elderly adults or adults with significant comorbidities, respectively) will receive the full standard dose of CoVLP (3.75 µg) adjuvanted with AS03 in**	

<table>
<tr><th>#</th><th>Section</th><th>Change</th><th>Rationale</th></tr>
<tr><td></td><td></td><td>an injection volume of 0.5 mL. In Phase 2, if an acceptable immune response is not achieved at either Day 21 or Day 42, the IDMC may recommend a dose escalation to 7.5 µg CoVLP adjuvanted with AS03 in a volume of 0.5 mL, and the same number of subjects (i.e. 120 subjects per study population) in the particular Study Population will be recruited, using the same enrollment approach (i.e. use of sentinel subjects and IDMC reviews). If the higher dose of the CoVLP formulation achieves an acceptable immune response in the Phase 2 portion, then the same higher dose will be used in Study Populations #3 and/or #4 during the Phase 3 portion of the study.</td><td></td></tr>
<tr><td>10.</td><td>Synopsis (Study Design)
AND
Section 7 (Dosage and Administration)</td><td>The following statement has been added to the end of the third paragraph under the Phase 2 portion header:
Enrollment of subjects in Study Population #4 will include adults with co-existing conditions such as, but not limited to, obesity or being overweight, cardiovascular diseases, hyperlipidemia, chronic kidney disease or related disorders, chronic respiratory disease, pre-diabetes and diabetes, treatment-controlled HIV, and psychiatric disorders.</td><td>Based on BRRD feedback, clarified the comorbidities for Study Population #4.</td></tr>
<tr><td>11.</td><td>Synopsis (Study Design)
AND</td><td>The following updates have been made to the first two paragraphs below the Phase 3 portion header:
The Phase 3 portion is a randomized, observer-blinded, placebo-controlled, efficacy, safety, immunogenicity (in a subset of subjects), multicenter design with male and female subjects. Similar to the Phase 2 portion, subjects will be enrolled from ~~four~~ five Study Populations:
<table>
<tr><th>Study Population</th><th>Age and/or Health Category</th><th>Age Range (years)</th></tr>
<tr><td>1</td><td>Healthy adults</td><td>18-64</td></tr>
</table></td><td>Based on feedback from BRDD, pregnant females in the 3rd trimester may be included in a separate cohort during the Phase 3 portion of the study as Study Population #5.</td></tr>
</table>

<table>
<tr><th>#</th><th>Section</th><th>Change</th><th>Rationale</th></tr>
<tr><td></td><td></td><td>
<table>
<tr><td>2</td><td>Healthy children and adolescents</td><td>5-17</td></tr>
<tr><td>3</td><td>Elderly adults</td><td>≥ 65</td></tr>
<tr><td>4</td><td>Adults with significant co-morbidities</td><td>≥ 18</td></tr>
<tr><td>5</td><td>Healthy female adults who are in the 3rd trimester of their low-risk pregnancy</td><td>≥ 18</td></tr>
</table>
~~(listed above in the Study Rationale section) and each~~ Study Populations #2 and #3 will be roughly stratified by age in the same manner. The overall goal of the Phase 3 portion of this study is to enroll approximately 30 000 subjects who are generally representative of the overall target population in terms of racial diversity with enrichment for those at highest risk for serious COVID-19 disease (i.e. the elderly and those with co-morbidities) and relatively smaller numbers of the subjects at lowest risk of serious COVID-19 (i.e. children and adolescents). In addition, Study Population #5 enrollment will only begin after the administration of the second vaccination of all subjects in Study Populations #1 to #4.
</td><td></td></tr>
<tr><td>12.</td><td>Synopsis (Study Design)
AND
Section 4.1 (Overview of Study Design)</td><td>The first sentence of the tenth paragraph has been modified as follows:
Subjects will be screened ~~up to 14 days in advance of the first vaccine administration~~ and will demonstrate a satisfactory baseline medical assessment by history and general physical examination.</td><td>Removed irrelevant text as the screening and dose 1 vaccination visits will occur at the same visit during the Phase 3 portion.</td></tr>
<tr><td>13.</td><td>Synopsis (Study Design)
AND</td><td>The following sentence has been removed from the tenth paragraph:</td><td>To clarify that this test will not be performed at screening in the Phase 3 portion.</td></tr>
</table>

#	Section	Change	Rationale
	Section 4.1 (Overview of Study Design)	Although a test for SARS-CoV-2 antibodies will be performed at screening, both seronegative and seropositive subjects will be enrolled.	
14.	Synopsis (Study Design)<br>AND<br>Section 4.1 (Overview of Study Design)<br>AND<br>Table 2 (Time and Events Schedule: General Information for the Phase 3 Portion)	The following statement has been added to the end of the eleventh paragraph:<br>In addition, to monitor asymptomatic infection, subjects will perform a test for SARS-CoV-2 N antibodies on Day 0, Day 201, and Day 386.<br>AND<br>An “X” has been added to the cells for Study Days 0, 201, and 386 for the “Test for SARS-CoV-2 N antibodies” row.	Based on BRDD feedback, this test has been added at specific timepoints to assess a new efficacy endpoint.
15.	Synopsis (Blinding)	This section has been removed from the Synopsis section only:<br>Both the Phase 2 and Phase 3 portions of this study will be observer-blind.<br>During the entire duration of the study, the following individuals will not have access to treatment allocation (i.e. remain “blind”): the subjects, the Investigators and all personnel involved in the clinical conduct of the study (except the staff involved in the preparation and administration of the study vaccine, the quality assurance auditor, and quality control reviewers), Medicago clinical staff and medical staff involved in safety evaluations (e.g. causality assessments), and all personnel involved in sample analysis at the central and testing (Nab, ELISA, ELISpot, ICS [flow cytometry], serological, and virologic method SARS-CoV-2 diagnostics) laboratories.	To simplify the Synopsis section.

#	Section	Change	Rationale
		During the study, the IDMC and an unblinded team, compromised of the unblinded medical monitor, unblinded biostatistician, unblinded senior reviewer, and unblinded programmer involved in the preparation of the safety data summary for the IDMC reviews, will have access to or be aware of treatment allocation (i.e. be "unblinded"). The IDMC will review safety data and will make recommendations for the entire duration of the study. The same unblinded statistical team will be involved in the Day 21 and Day 42 (if needed) immunogenicity data analyses in the Phase 2 portion and in the interim efficacy analyses in the Phase 3 portion.<br>During the study, the Sponsor's study team will remain blinded.	
16.	Synopsis (Efficacy Evaluations)<br>AND<br>Table 1 and Table 2 (Row: Testing for laboratory confirmation of SARS-CoV-2 infection)<br>AND<br>Section 10.1.10 (Surveillance for COVID-19 Cases)<br>AND<br>Section 10.4.1 (Efficacy Evaluations)	The following content has been added to the fourth paragraph:<br>Subjects who report any symptoms associated with COVID-19 after the start of active and passive surveillance will be requested to visit the Investigator site to provide nasal or nasopharyngeal (NP) swabs (2 per subject per event) to test for COVID-19. **During the visit, the Investigator will confirm whether nasal or NP swabs should be collected. If the subject cannot visit the Investigator site, then the subject may receive a visit at home to collect the nasal/NP swabs samples or be provided with self-administered nasal/NP swabs for sample collection.**	To clarify that the collection of swabs during the surveillance period will involve input from the Investigator.
17.	Synopsis (Secondary Endpoints)<br>AND	The first two bullets for the Phase 2 portion immunogenicity secondary endpoints have been revised as follows:	To further clarify the two secondary immunogenicity endpoints.

#	Section	Change	Rationale
	Section 10.3.2.2 (Secondary Endpoints)	• **Persistence of the** Nab antibody response induced in each Study Population against the SARS-CoV-2 virus ~~on~~ **at** Day 201 and Day 386 will be analyzed using the following parameters: GMT, SC rate, and GMFR;<br>• Specific antibody response induced in each Study Population against the SARS-CoV-2 virus will be measured by the total IgG levels on Days 0, 21, **and** 42, **and the persistence of these antibodies at Days** 201~~,~~ and 386 and analyzed using the following parameters: GMT, SC rate, and GMFR;	
18.	Synopsis (Secondary Endpoints)<br>AND<br>Section 10.4.2.2 (Secondary Endpoints)	The following two efficacy secondary endpoints have been added to the Phase 3 portion:<br>• The slope estimate of the weighted Schoenfeld residuals against event time for the evaluation of absolute vaccine efficacy determined from the first occurrence, in a subject, of laboratory-confirmed (virologic method) symptomatic SARS-CoV-2 infection (≥ 14 days post-last vaccination);<br>• Occurrences of laboratory-confirmed (confirmed by the ELISA method for the N protein) asymptomatic SARS-CoV-2 infection at Day 201 and Day 386;	Based on feedback from BRDD, two new efficacy endpoints were added to the Phase 3 portion.
19.	Synopsis (Secondary Endpoints)<br>AND<br>Section 10.3.2.2 (Secondary Endpoints)	The following immunogenicity exploratory endpoints have been moved to secondary endpoints for the Phase 3 portion and combined:<br>• In a subset of subjects, Nab antibody response induced in each Study Population against the SARS-CoV-2 virus on Days 0, 21, 42, 201, and 386 analyzed, using the following parameters: GMT, SC rate, and GMFR;<br>• In a subset of subjects, specific antibody response induced in each Study Population against the SARS-CoV-2 virus measured by the total IgG levels on Days	For consistency with the Phase 2 portion, these immunogenicity endpoints are now secondary endpoints.

#	Section	Change	Rationale
		0, 21, 42, 201, and 386, and analyzed using the following parameters: GMT, SC rate, and GMFR;<br>• In as subset of subjects, the ratio of neutralizing antibody titers:IgG (ELISA) antibody titers at Day 21, Day 42, Day 201, and Day 386;<br>• In a subset of subjects, specific Th1 CMI response induced in each Study Population against the SARS-CoV-2 virus on Days 0, 21, 42, 201, and 386, as measured by IFN-γ ELISpot;<br>• In a subset of subjects, specific Th2 CMI response induced in each Study Population against the SARS-CoV-2 virus on Days 0, 21, 42, 201, and 386, as measured by IL-4 ELISpot;<br>• In a subset of subjects, specific CMI response induced in each Study Population against the SARS-CoV-2 virus up to 21 days after the last vaccination and at Day 201 and Day 386, as measured by the percentage of CD4+ T cells expressing functional markers;	
20.	Synopsis (Safety Review)	The following statement has been added to the first paragraph as follows:<br>During the study, the IDMC will review safety data to ensure the ongoing safety of all the subjects in the study. **During the Phase 2 portion, the safety data for the first 10 sentinel subjects after each dose in all Study Populations, with the exception of Study Population #1, will be reviewed by the IDMC before permitting enrollment of the remaining subjects, according to the details outlined in the IDMC Charter. Additional** ~~I~~information about the planned and ad hoc IDMC review meetings is **also** available in the IDMC charter.	Based on BRDD feedback, included more details about subject enrollment and the IDMC reviews.

#	Section	Change	Rationale
21.	Synopsis (Safety Review)<br>AND<br>Section 13.1.11 (Safety Review and Stopping Rules)<br>AND<br>Synopsis (Statistical Methods – Phase 2 Portion Day 21 and Possibly Day 42 Immunogenicity Data Analysis)<br>AND<br>Section 12.3 (Phase 2 Portion Day 21 and Possibly Day 42 Immunogenicity Data Analysis and Interim Efficacy Analyses)	The use of "(last subject)" has been removed from the following statements:<br>During the Phase 2 portion, the IDMC will review the safety data collected up to Day 28 ~~(last subject)~~ from all the subjects in each Study Population to confirm that each Study Population can be included in the Phase 3 portion of the study.<br><br>The unblinded statistical team will perform immunogenicity data analysis after Day 21 ~~(last subject)~~ in each of the Study Populations.<br><br>In the event that the Day 21 NAb response in any of the Study Populations is <50 % of that achieved by healthy adults, then a similar data analysis will be performed after Day 42 ~~(last subject)~~ for the Study Population(s) with the lower Nab response in order to initiate the Phase 3 portion of the study for the particular Study Population.	To simplify the collection of data up to particular days.
22.	Synopsis (Statistical Methods – Phase 3 Portion Interim Efficacy Analysis)<br>AND<br>Section 12.3 (Phase 2 Portion Day 21 and	The following statement has been added after the first sentence:<br>When 80 laboratory-confirmed COVID-19 cases have been collected, the unblinded statistical team will perform a first interim analysis for the Phase 3 portion of the efficacy data in order to test for early vaccine efficacy. **If the primary efficacy success criterion is met during this interim analysis, enrollment of subjects into the placebo treatment group will**	Based on BRDD feedback, clarified when enrollment into the placebo group will stop during the Phase 3 portion of the study.

#	Section	Change	Rationale
	Possibly Day 42 Immunogenicity Data Analysis and Interim Efficacy Analyses)	**stop; subject enrollment into the CoVLP formulation treatment group will continue for the collection of safety data and for a descriptive assessment of the efficacy and immunogenicity endpoints.** A second interim analysis for the Phase 3 portion will be performed as soon as 160 cases are collected in order to perform the final analysis and produce final efficacy outputs.	
23.	Synopsis (Statistical Methods – Phase 3 Portion Initiation)	The second paragraph has been modified and the fourth paragraph has been added as follows:<br>If the Day 21 immunogenicity data and Day 28 safety data results are favorable **based upon pre-defined criteria** and show acceptable safety and immune responses for Study Populations #3 and #4, assuming the results in Study Population #1 will be favorable, then the Phase 3 portion of the study can start for all three Study Populations. **The decision to proceed into the Phase 3 portion of the study for these two Study Populations will be taken in close consultation with the IDMC.**<br>As mentioned above, if the Day 21 immune response is not acceptable in one or both of Study Populations #3 and #4, then the Day 42 immunogenicity data will be assessed in the applicable Study Population(s) for the purposes of determining whether the Phase 3 portion may be started for that/those Study Population(s).<br>**For Study Population #2, the Day 21 immunogenicity data and Day 28 safety data results will also be assessed and if an acceptable immune response is observed and the IDMC has no concerns with the safety data up to Day 28, then the Phase 3 portion of the study can start for Study Population #2 with staggering of the age cohorts as outlined above.**	To clarify how the Phase 2 portion will transition to the Phase 3 portion.

#	Section	Change	Rationale
24.	Table 2 (Time and Events Schedule: General Information for the Phase 3 Portion)	For the Phase 3 portion, the Screening visit and Day 0 visit have been combined and procedures for the two visits were combined into a single column (without duplicating any procedures) and the relevant footnotes have also been updated:<br>The Visits have been renumbered from 1-6 to 1-5 in the sub-header.<br>An "X" was placed in the cell for Informed Consent and Demographics on Day 0.<br>The footnote "1" was removed from the "X" for Medical history/prior medication on Day 0.<br>The footnote "2" and "X" have been added to History/symptom-directed physical examination on Day 0.<br>The content for footnote "1" has been replaced with the content for footnote "10".<br>The footnote "3" content has been revised as follows: Record prior to study vaccine, ~~after the observation period~~ and as deemed necessary.<br>The footnote "4" explanation has been updated as follows:<br>In all females of childbearing potential in Study Populations #1, #2, ~~and~~ #4**, and #5**; it will be tested in urine at screening and at Days 0, 21, and 42. Study Population #**5**~~1~~: ~~in the Phase 3 portion of the study,~~ any female subject who is in the 1st or 2nd trimester of her pregnancy cannot participate in the study. However, any female subject who is in the 3rd trimester of her low-risk pregnancy can participate in the study.	Table has been updated to incorporate changes to tests and procedures.
25.	Table 3 (Time and Events Schedule: Onset of COVID-19)	The following statement has been added to the first cell of the third row in the Table:<br>Swab collection will be done at the discretion of the site Investigator.	

#	Section	Change	Rationale
26.	Table 3 (Time and Events Schedule: Onset of COVID-19)	The first sentence of footnote "1" has been updated as follows:<br>Nasal or NP swab collection is to be done within 72 hours after reporting of the COVID-19 symptoms (preferably within 48 hours)**, at the discretion of the site Investigator,** and prior to the use of any antiviral treatment medication.	To clarify when specific tests will be performed and that the collection of swabs during the surveillance period will involve input from the Investigator.
27.	Section 1.1.4 (COVID-19 Infection in Pregnancy)	A new sub-section has been added with the following content:<br>Highly pathogenic coronavirus infections including SARS-COV-2 are associated with increased risk in pregnant women including severe disease [Buonsenso 2020, Galang 2020, Rasmunssen 2020] and fetal loss [Baud 2020,Hachem 2020, Mehan 2020, Zimmerman n2020]. Some have advocated strongly for the inclusion of pregnant women in trials for COVID therapies and vaccines [DiMascio 2020] and the FDA has encouraged vaccine manufacturers to include pregnant women and women of childbearing potential in their trials [FDA 2020].	Based on feedback from BRDD, pregnant females in the 3rd trimester may be included in a separate cohort during the Phase 3 portion of the study as Study Population #5.
28.	Section 1.1.5 (Risk:Benefit in the Different Study Populations)	A new sub-section has been added with the following content:<br>As outlined above, while most people appear to be equally susceptible to SARS-COV-2 infection, the risks from COVID-19 disease are not evenly distributed across all ages and all populations. Although the role of children in chains of transmission is still a matter of some debate [Li 2020, Zimmermann 2020], it is becoming clear that any infected individual including those who as pre-symptomatic, asymptomatic and symptomatic can contribute to transmissi [Furukawa 2020]. Although the emergence of SARS-COV-2 has caused massive disruption, the proportion the world's population that has been infected remains relatively small despite its expanding global circulation for the past [illegible] months [Chen 2020c]. As a result, most authorities consider	Based on feedback from BRDD, risk:benefit considerations for each Study Population has been included.

#	Section	Change	Rationale
		rapid immunization of the entire population will be needed to achieve herd immunity in a reasonable time-frame [Randolph 2020].<br><br>Because the risks of COVID-19 fall most heavily on the elderly and those with co-morbid conditions, the benefits in vaccinating young, healthy adults and children are primarily societal and indirect (i.e. public health, enhancing herd immunity, societal re-opening) while individuals in the higher risk groups also derive a clear and direct personal health benefit. The case of pregnant women at different stages of gestation as well as women of child-bearing potential (WOCBP) not actively avoiding pregnancy introduces unique risks at the same time as providing unique benefits for both the mother and the unborn child/neonate.<br><br>At the current time, there is no reason to suspect the CoVLP formulation that will be used in the proposed study poses an elevated risk specific to any of the Study Populations. In particular, the adjuvant (AS03) has been used across the entire age range including pregnant women and WOCBP [Cohet 2015](see Section 1.2.1). As a result, risk:benefit calculations for each of these Study Populations are driven primarily by the benefit denominator. Medicago considers that the direct and indirect benefits amply justify the inclusion of all five of the Study Populations in the current Phase 2/3 study with the safeguards currently proposed including the active role of the IDMC.	
29.	Section 1.5 (Overall Rationale for the Study)	Table 4 Study Populations has been updated to include a fifth row for Study Population 5:	Based on feedback from BRDD, pregnant females in the 3rd trimester may be included in a separate cohort during the Phase 3

#	Section	Change	Rationale
		5* | Healthy female adults who are in the 3rd trimester of their low-risk pregnancy | ≥ 18<br>*This study population is only for the Phase 3 portion of the study.	portion of the study as Study Population #5.
30.	Section 4.1 (Overview of Study Design)	A reference to a new figure, “Figure 1”, has been added to the first sentence of the third paragraph and the new figure has been added after the sixth paragraph in this section.	Based on BRDD feedback, included more details about subject enrollment and the IDMC reviews.
31.	Section 4.1 (Overview of Study Design)	The fourth paragraph has been updated as follows:<br>The IDMC will review unblinded safety data for the first 10 sentinel subjects after each dose in ~~all~~ Study Populations **#2, #3, and #4 prior to vaccinating the remaining subjects in each Study Population**, ~~with the exception of Study Population #1,~~ according to the details outlined in the IDMC Charter. All sentinel subjects will be dosed at least 30 minutes apart. **Further, the enrollment for Study Population #2 will begin with the adolescent subjects after all of the seven-day safety data following the second vaccination of the healthy adults (Study Population #1) have been reviewed by the IDMC and after the availability of the Day 21 immunogenicity data from all the healthy adults (Study Population #1).**	Based on BRDD feedback, included more details about subject enrollment and the IDMC reviews.
32.	Section 4.1 (Overview of Study Design)	The following content has been added after the seventh paragraph:<br>Transition of each of the Study Populations from the Phase 2 to the Phase 3 portion of the study will be determined by the safety and immunogenicity profile unique to that Study Population in the Phase 2 portion (refer to Section 12.4 for details).	To clarify how the Phase 2 portion will transition to the Phase 3 portion.

#	Section	Change	Rationale
33.	Section 5.1 (Inclusion Criteria)	Study Population #5 has been added to the following inclusion criteria:<br>2. At the Screening visit (Visit 1), male and female subjects must be<br>• Study Populations #1 **and #5**: 18 to 64 (has not yet had his/her 65th birthday) years of age, inclusive;<br>3. At Screening (Visit 1) and Vaccination (Visit 2), subject must have a body mass index (BMI) of:<br>• Study Populations #1, ~~and~~ #3, **and #5**: ≥ 18.5 and < 30 kg/m^2;<br>5. Study Populations #1, ~~and~~ #2, **and #5**: Subjects must be in good general health prior to study participation, with no clinically relevant abnormalities that could jeopardize subject safety or interfere with study assessments, as determined by medical history, physical examination, and vital signs. Investigator discretion will be permitted with this inclusion criterion;	Based on feedback from BRDD, pregnant females in the 3rd trimester may be included in a separate cohort during the Phase 3 portion of the study as Study Population #5.
34.	Section 5.1 (Inclusion Criteria)	The following inclusion criteria have been updated and a new criterion has been included:<br>152. Study Populations #1, #2, and #4: Female subjects of childbearing potential must have a negative serum pregnancy test result at Screening (Visit 1) and a negative urine pregnancy test result at Vaccination (Visit 2)~~, with the following exception~~:<br>~~• Study Population #1 (for the Phase 3 portion of the study only): Female subjects who are in the 3rd trimester of a low-risk pregnancy at Screening (Visit 1) can be enrolled in the study;~~<br>Non-childbearing females are defined as:	Based on feedback from BRDD, pregnant females in the 3rd trimester may be included in a separate cohort during the Phase 3 portion of the study as Study Population #5.

#	Section	Change	Rationale
		• Surgically-sterile (defined as bilateral tubal ligation, hysterectomy or bilateral oophorectomy performed more than one month prior to the first study vaccination); or<br>• Post-menopausal (absence of menses for 12 consecutive months and age consistent with natural cessation of ovulation);<br>• Non-ovulating, pre-menstrual girls < 17 years of age;<br>**153. Study Population #5: Female subjects who are in the 3rd trimester of a low-risk pregnancy at Screening (Visit 1) can be enrolled in the study;**<br>154. Study Populations #1, #2, and #4: ~~With the exception of low-risk pregnant women in their third trimester noted above in inclusion criterion no. 6 for the Phase 3 study only, f~~**F**emale subjects of childbearing potential must use an effective method of contraception for one month prior to vaccination (Visit 2) and agree to continue employing highly effective birth control measures for at least one month after the last study vaccination (or in the case of early termination, she must not plan to become pregnant for at least one month after her last study vaccination), with the exception of the following subjects:	
35.	Section 5.2 (Exclusion Criteria)	Study Population #5 has been added to or removed from the following exclusion criteria:<br>1. Study Populations #1, #2, ~~and~~ #3, **and #5**: According to the Investigator's opinion, significant acute or chronic, uncontrolled medical or neuropsychiatric illness.<br>2. Study Populations #1, #2, ~~and~~ #3, **and #5**: Any confirmed or suspected current immunosuppressive condition or immunodeficiency, including cancer, human immunodeficiency virus (HIV), hepatitis B or C	Based on feedback from BRDD, pregnant females in the 3rd trimester may be included in a separate cohort during the Phase 3 portion of the study as Study Population #5.

#	Section	Change	Rationale
		infection (subjects with a history of cured hepatitis B or C infection without any signs of immunodeficiency at present time are allowed). Investigator discretion is permitted with this exclusion criterion;<br>3. Study Populations #1, #2, ~~and~~ #3, **and #5**: Current autoimmune disease (such as rheumatoid arthritis, systemic lupus erythematosus or multiple sclerosis). Investigator discretion is permitted with this exclusion criterion, and subjects may be eligible to participate with appropriate written justification in the source document (i.e. subjects with a history of autoimmune disease who are disease-free without treatment for three years or more, or on stable thyroid replacement therapy, mild psoriasis [i.e. a small number of minor plaques requiring no systemic treatment], etc.);<br>4. Study Populations #1, #2, ~~and~~ #3, **and #5**: Administration of any medication or treatment that may alter the vaccine immune responses, such as:<br>• Study Populations #1, ~~and~~ #3, **and #5**: Systemic glucocorticoids at a dose exceeding 10 mg of prednisone (or equivalent) per day for more than seven consecutive days or for 10 or more days in total, within one month prior to the Vaccination visit (Visit 2). Inhaled, nasal, ophthalmic, dermatological, and other topical glucocorticoids are permitted;<br>15. Study Populations #1 ~~(for the Phase 2 portion of the study)~~, #2, and #4: Any female subject who has a positive or doubtful pregnancy test result prior to vaccination or who is lactating;	

#	Section	Change	Rationale
		16. **Study Population #5**: Any female subject who is in the 1st or 2nd trimester of her pregnancy or whose pregnancy is considered to be high-risk by her obstetrician;	
36.	Section 5.4 (Prohibited Therapy)	Study Population #5 has been added to the following criteria:<br>3. Study Populations #1, #2, ~~and~~ #3, **and #5**: Administration of any medication or treatment that may alter the vaccine immune responses, such as:	
37.	Section 8.1 (Management of Samples)	The first and third paragraphs have been revised as follows:<br>Blood samples for biochemistry, haematology, and serology (for HIV, Hepatitis B, and Hepatitis C) and urine samples for urinalysis will be collected at screening and/or during the study for the Phase 2 portion of the study. Serum (Phase 2 portion only) and urine (dipstick or similar) pregnancy testing will be performed for females of childbearing potential. Blood samples will also be collected for the testing of SARS-CoV-2 antibodies at screening **for the Phase 2 portion and during the Phase 3 portion of the study**.<br>Between vaccination and the end of the surveillance period, nasal or NP swabs will be collected from any subject who reports any symptoms associated with COVID-19 after the start of active and passive surveillance **at the discretion of the site Investigator**; these swabs will be collected each time a subject reports such an event within the specified timeframe, so multiple collections may be required from some subjects. Two swabs will be collected per subject per event (one from each nare). Bioanalysis of the nasal or NP swabs will be conducted at central laboratories.	To clarify when specific tests will be performed and that the collection of swabs during the surveillance period will involve input from the Investigator.
38.	Section 8.2 Collection of Samples	The third, sixth, and seventh paragraphs and Table 7 have been revised as follows:	To update the blood draws and volume to be consistent with Table 1 for the Phase 3 portion.

#	Section	Change	Rationale
		In the Phase 3 portion of the study, all subjects will have **1**5 mL of blood drawn **up to the end of the study (Day 386)** ~~at screening~~ and only a subset of subjects included in the immunogenicity analyses will have an additional blood volume of up to approximately 250 mL drawn up to the end of the study (Day 386) (Table 7).<br>In the Phase 3 portion of the study, pregnancy testing will be performed for all females of childbearing potential (in Study Populations #1, #2, ~~and~~ #4, **and #5**). A urine (dipstick or similar) pregnancy test will be performed at screening and at Day 0 prior to randomization and at Days 21 and 42. In Study Populations #1, #2, and #4, females of child bearing potential must have negative urine pregnancy test results from screening and Day 0 available prior to vaccination. In Study Population **#5**~~1~~, any female subject who is in the 1st, or 2nd, trimester of her pregnancy cannot participate in the study. However, any female subject who is in the 3rd trimester of a low-risk pregnancy can participate in the study.<br>Nasal or NP swabs will be collected from any subject who reports any symptoms associated with COVID-19 after the start of active and passive surveillance, **at the discretion of the site Investigator**. Two swabs will be collected per subject per event (one from each nare). The swabs will be collected within 72 hours (preferably within 48 hours) of the reporting of a COVID-19-associated symptom. If swabs cannot be collected within 72 hours after reporting of the COVID-19-associated symptom, nasal or NP swabs should be collected at the next available opportunity for the subject to visit the Investigator site.<br>Table 7<br>Visit 1 has been removed and the remaining visits have been renumbered.	

#	Section	Change	Rationale
		Blood collection of 5 mL for Test for SARS-CoV-2 antibodies had been added to Day 0, Day 201, and Day 386 visits.<br>The total volume of blood per subject to be collected has been updated to 15-265 mL.<br>The table footnote has been updated to remove “384”.	
39.	Section 10.1 (Study Procedures)	The following sub-headers have been updated:<br>10.1.3 Screening (Visit~~s~~ 1 **for Phase 2 Portion and Phase 3 Portion**)<br>10.1.4 Dose 1 Vaccination (Day 0) (Visit 2 **for the Phase 2 Portion and Continuation of Visit 1 for Phase 3 Portion**)<br>10.1.11 Day 42 (Visit 6 for Phase 2 portion and Visit ~~4~~**3** for Phase 3 portion)<br>10.1.12 Day 201 (Visit 7 for Phase 2 portion and Visit ~~5~~**4** for Phase 3 portion)	For the Phase 3 portion, screening and dose 1 vaccination will occur on the same day and clarification for the visit numbers has been provided.
40.	Section 10.1.3 (Screening)	The following bullet point has been added to this section:<br>• For the Phase 3 portion only: proceed to the procedures for Dose 1 Vaccination (see Section 10.1.4) and perform only the procedures in Section 10.1.4.1 that have not been performed.	For the Phase 3 portion, screening and dose 1 vaccination will occur on the same day and clarification around procedures has been provided.
41.	Section 10.1.4.1 (Prior to Vaccination)	The first paragraph and the fifth and seventh bullets have been revised as follows:<br>The following procedures will be performed on Day 0 (Visit 2) prior to vaccination for the Phase 2 ~~and Phase 3~~ portion~~s~~ of the study. **For the Phase 3 portion, the screening and vaccination visit will occur on the same day and the following procedures will be performed (as long as they have not been completed as per the Phase 3 specific procedures listed in Section 10.1.3)**:	Based on feedback from BRDD, to clarify the testing for pregnancy in Study Population #5 and incorporate the blood sample collection for the SARS-CoV-2 testing in the Phase 3 portion.

#	Section	Change	Rationale
		• Study Population #**5**~~1~~: in the Phase 3 portion of the study, any female subject who is in the 1st or 2nd trimester of her pregnancy cannot participate in the study. However, any female subject who is in the 3rd trimester of her low-risk pregnancy can participate in the study;<br>• After confirmation of eligibility and randomization, collect baseline blood samples **to test for SARS-CoV-2 antibodies (Phase 3 portion) and** for immunogenicity (serology [Nab assay and ELISA] and CMI) assessments (Phase 2 portion - all subjects; Phase 3 portion - immunogenicity subset); prepare and store these samples until shipment to the analytical laboratory.	
42.	Section 10.1.4.3 (Thirty Minutes Post-vaccination)	The bullet point has been revised as follows:<br>• **Phase 2 portion only: a**~~A~~fter the 30-minute observation period (allowed window of + 15 minutes) is completed, measure vital signs (BP, HR, RR, and OT) as described in Section 10.2.1.4. Any out-of-range measurements will be assessed by the Investigator (or his/her designee) and any further action will be determined upon his/her medical decision;	To clarify that these procedures will only be performed in the Phase 2 portion.
43.	Section 10.1.7.1 (Pre-vaccination)	The bullet point has been revised as follows:<br>• Study Population #~~1~~**5**: ~~in the Phase 3 portion of the study,~~ any female subject who is in the 1st or 2nd trimester of her pregnancy cannot participate in the study. However, any female subject who is in the 3rd trimester of her **low-risk** pregnancy can participate in the study;	Based on feedback from BRDD, pregnant females in the 3rd trimester may be included in a separate cohort during the Phase 3 portion of the study as Study Population #5.
44.	Section 10.1.12 (Day 201)	The following bullet point has been added:	To clarify a blood sample collection will occur for the

#	Section	Change	Rationale
	AND<br>Section 10.1.13 (Day 386)	• Phase 3 portion only: collect blood sample to test for SARS-CoV-2 antibodies;	SARS-CoV-2 testing in the Phase 3 portion.
45.	Section 10.2.1.2 (Adverse Events and SARS-CoV-2 Positive Reports)	The following paragraph has been added to the end of the section:<br>Potential COVID-19 cases will be reviewed by the unblinded medical monitor (part of the unblinded team) during the Phase 2 and Phase 3 portions of the study. Confirmed COVID-19 cases will not be recorded as AEs since these events will be collected for the planned efficacy evaluation. Also, confirmed COVID-19 cases will not be reported according to the process for reporting SAEs, even in the event such case may meet the definition of an SAE. However, any potential COVID-19 case that is not a confirmed event (i.e. does not meet the definition of the efficacy endpoints) and does meet the criteria for an SAE will be reported back to the clinical site of incidence. The unblinded medical monitor and the clinical site Investigator both must report the SAE to Medicago within 24 hours (see Section 13.1.5) of becoming or being made aware that the potential COVID-19 case was not a confirmed event. The Investigator's SAE awareness date will be the date on which the clinical site of incidence receives the SAE back from the unblinded medical monitor. Confirmed COVID-19 cases will be reported in the relevant screen (e.g. COVID-19 symptoms page) in the subject's eCRF within 1 day of reporting by the subject to the clinical site.	To clarify the assessment and communication of potential COVID-19 cases.
46.	Section 10.2.1.3 (Clinical Laboratory Tests)	The first and third paragraphs have been revised as follows:<br>Blood samples for biochemistry and hematology, HIV, Hepatitis B and Hepatitis C markers in serum and urine samples for urinalysis will be collected according to the Time and Events Schedule (see Table 1) for the Phase 2 portion of the study. Blood samples for testing for SARS-CoV-2 antibodies will be	To clarify the testing for pregnancy in Study Population #5 and incorporate the blood sample collection for the SARS-CoV-2 testing in the Phase 3 portion.

#	Section	Change	Rationale
		collected according to the Time and Events Schedules (see Table 2) for the Phase 2 and Phase 3 portions of the study. In addition, serum or urine samples from all females of childbearing potential in Study Populations #1, #2, ~~and~~ #4, **and #5** for pregnancy testing will be collected.<br>Other clinical laboratory tests include a test to measure SARS-CoV-2 antibodies at screening for all subjects in all Study Populations **in the Phase 2 portion and at Days 0, 201, and 386 in the Phase 3 portion**, and a serum (Phase 2 portion) or urine (Phase 3 portion) pregnancy test at screening and a urine (dipstick or similar) pregnancy test prior to randomization on Day 0 and on Days 21 and 42 for all female subjects of childbearing potential in Study Populations #1, #2, ~~and~~ #4, **and #5**.	
47.	Section 10.2.1.4 (Vital Signs)	The first paragraph has been updated as follows:<br>Vital signs measurements (resting BP, HR, RR, and OT) will be performed as part of screening procedures, prior to vaccination (on Day 0 and Day 21), and after the post-vaccination 30-minute surveillance period (and repeated if deemed necessary by the Investigator) **during the Phase 2 portion**. In addition, vital signs measurements will be performed at the clinic site visits on Day 3 ~~(Phase 2 portion only)~~, Day 24 ~~(Phase 2 portions only)~~, Day 42, Day 201, and Day 386 for the Phase 2 ~~and Phase 3~~ portion~~s~~ of the study. **Vital signs measurements will only be performed prior to each vaccination during the Phase 3 portion.**	To clarify that these procedures will only be performed in the Phase 2 portion.
48.	Section 10.2.2 (Pregnancy)	The first sentence of the first paragraph and the last sentence of the second paragraph have been updated as follows:<br>Female subjects who become pregnant during the Phase 2 or Phase 3 portions of the study or are in the 3rd trimester of their	Based on feedback from BRDD, pregnant females in the 3rd trimester may be included in a separate cohort during the Phase 3

#	Section	Change	Rationale
		**low-risk** pregnancy (Study Population #**5**~~1~~ only) during the Phase 3 portion of the study will be followed for safety.<br>Information on pregnancies identified during the screening phase/prior to vaccine administration need to be collected for female subjects in the 3rd trimester of their pregnancy in Study Population #**5**~~1~~ for the Phase 3 portion of the study.	portion of the study as Study Population #5.
49.	Section 11.3 (Contraindications for Subsequent Vaccination)	The first bullet point has been revised as follows:<br>• Pregnancy, with the exception of female subjects in the 3rd trimester of a low-risk pregnancy in Study Population #**5**~~1~~ for the Phase 3 portion (see Section 10.2.2);	Based on feedback from BRDD, pregnant females in the 3rd trimester may be included in a separate cohort during the Phase 3 portion of the study as Study Population #5.
50.	Section 12.4 (Initiation of the Phase 3 Portion)	The third and fourth paragraphs have been replaced with the following content:<br>~~If the Day 21 immunogenicity data and Day 28 safety data results are favorable and show acceptable safety and immune responses for Study Populations #3 and #4, assuming the results in Study Population #1 will be favorable, then the Phase 3 portion of the study can start for all three Study Populations.~~<br>~~As mentioned above, if the Day 21 immune response is not acceptable in one or both of Study Populations #3 and #4, then the Day 42 immunogenicity data will be assessed in the applicable Study Population(s) for the purposes of determining whether the Phase 3 portion may be started for that/those Study Population(s).~~<br>**Study Population #1: If the safety and immunogenicity profiles of the chosen CoVLP formulation observed in the Phase 1 study are acceptable in the Phase 2 portion, this group will proceed into the Phase 3 portion. Another key**	To clarify how the Phase 2 portion will transition to the Phase 3 portion, including the criteria to be applied to each Study Population.

#	Section	Change	Rationale
		**outcome will be the absence of a significant safety signal as assessed by the IDMC.**<br><br>**Study Population #2: If the safety and immunogenicity profiles of the CoVLP formulation in this population are acceptable, this group will also proceed into the Phase 3 portion. A key outcome will be the absence of a significant safety signal as assessed by the IDMC. As noted above, any important safety signal in this Study Population may trigger one or more mitigation strategies prior to entry into the Phase 3 portion and the younger and older cohorts within this Study Population (5-9 and 10-17 year old) may enter into the Phase 3 portion asynchronously (outlined in Figure 1).**<br><br>**Study Populations #3 and #4: If the safety and immunogenicity profiles of the CoVLP formulation in this population are acceptable, entry of these two Study Populations into the Phase 3 portion will be based on both the absence of an important safety signal and demonstration of an acceptable immune response as assessed by the IDMC. The criteria for acceptability will be based on NAb and IFN-γ ELISpot results after either the first or second immunizations. If the 1st dose immunogenicity criteria (below) are not met at Day 21, the 2nd dose criteria (below) will be applied. This two-step approach is justified by the observation of a strong correlation between the responses elicited by the first and second doses of the CoVLP formulation. Because cellular immune responses are thought to be important for protection against highly pathogenic coronaviruses [Channappanavar 2014] including SARS-COV-2 [Sette 2020] and because cellular responses to some viral pathogens are better maintained than antibody**	

#	Section	Change	Rationale
		**responses in older subjects [McElhaney 2013a] the criteria below incorporate both Nab (A) and IFN-γ ELISpot (B) results in a cumulative A + B formula to determine acceptability. Our Phase 1 data further support this approach since occasional individuals were identified who made little-to-no antibody response but mounted IFN-γ ELISpot responses.**<br>**<u>First Dose Criteria</u>**<br>**Proportion of subjects with NtAb GMT ≥ 2-fold increase over baseline will be considered responders = A**<br>**Proportion of subjects with ≥ 2-fold increase over baseline in IFN-γ ELISpot will be considered responders = B**<br>**Acceptable to advance to Phase 3 portion if A + B ≥ 50% responders.**<br>**<u>Second Dose Criteria</u>**<br>**Proportion of subjects with NtAb GMT ≥ 4-fold increase over baseline = A**<br>**Proportion of subjects who with ≥ 2-fold increase over baseline in IFN-γ ELISpot = B**<br>**Acceptable to advance to Phase 3 portion if A + B ≥ 60% of the responders.**	
51.	Section 12.6.2 (Analysis of Secondary Endpoints)	The last paragraph has been modified and a new paragraph has been added right after:<br>For the Phase 3 portion, the same immunogenicity analyses will be performed as described for the primary **and secondary** immunogenicity endpoints for the Phase 2 portion of the study.<br>**In addition, the CMI response induced on Day 0, Day 21, Day 42, Day 201, and Day 386 will be measured by the**	In the Phase 3 portion, the immunogenicity exploratory endpoints have been updated to secondary endpoints. Thus, the analysis of these endpoints has moved to a new section.

#	Section	Change	Rationale
		**percentage of T cells expressing functional markers, using flow cytometry, for each treatment group. The responses will be compared between treatment groups and timepoints using appropriate non-parametric (Wilcoxon) models. Additional details will be provided in the SAP.**	
52.	Section 12.6.3 (Analysis of Exploratory Endpoints)	The section has been updated as follows:<br>The exploratory immunogenicity endpoints are defined in Section 10.3.2.3.<br>For the Phase 2 **and Phase 3** portions, if deemed necessary, further characterization of the immune response of the CoVLP formulation will be done and the results presented.<br>~~For the Phase 3 portion, the same immunogenicity analyses will be performed as described for the secondary immunogenicity endpoints for the Phase 2 portion of the study.~~<br>~~In addition, the CMI response induced on Day 0, Day 21, Day 42, Day 201, and Day 386 will be measured by the percentage of T cells expressing functional markers, using flow cytometry, for each treatment group. The responses will be compared between treatment groups and timepoints using appropriate non-parametric (Wilcoxon) models. Additional details will be provided in the SAP. If deemed necessary, further characterization of the immune response of the CoVLP formulation will be done and the results presented.~~	In the Phase 3 portion, the immunogenicity exploratory endpoints have been updated to secondary endpoints. Thus, the analysis of these endpoints has moved to a new section.
53.	Section 12.7.2 (Analysis of Secondary Endpoints)	The following paragraphs have been added above the last paragraph:<br>The presence of absolute VE waning will be tested by the linear trend (i.e., the proportional hazards) in the scaled Schoenfeld residuals from the Cox proportional hazard model. Specifically, it will be analyzed as below:	Based on feedback from BRDD, the analysis of a new efficacy endpoint has been added to the Phase 3 portion.

#	Section	Change	Rationale
		Step 1: Fit a regular Cox proportional hazard model with the treatment group as the main effect, and placebo will be coded as the reference group in the model;<br>Step 2: Find the scaled Schoenfeld residual from the Cox proportional hazard model in step 1. The scaled Schoenfeld residual has value at all event-time for the main effect (treatment group);<br>Step 3: Fit a linear regression on the scaled Schoenfeld residuals against event time;<br>Step 4: The slope and p-value will be output. VE(t)=1-exp(beta(t)), VE(t) waning (smaller with time) is corresponding to the increasing beta(t). Thus, a positive slope with significant p-value (< 0.05) will indicate VE is waning though time.	
54.	Section 13.1.3.1.1	In the first sentence of the first paragraph, the acronym "NEC" has been removed:<br>Safety signal of VED after exposure to the CoVLP formulation will be closely monitored and assessed by retrieving data for this AESI as follows: AEs within the system organ class (SOC): immune system disorders and high level group term (HLGT): lower respiratory tract disorders (excluding obstruction and infection), cardiac disorders, signs and symptoms not elsewhere classified~~ (NEC)~~, vascular disorders, heart failures~~ NEC~~, arteriosclerosis, stenosis, vascular insufficiency and necrosis, cardiac arrhythmias, myocardial disorders, and vascular hemorrhagic disorders.	To correct an administrative error.
55.	Section 13.1.3.1.2	The last sentence of the paragraph has been revised as follows:<br>To collect data on these events, Medicago will closely monitor and assess allergic ~~or allergic-like~~ reactions **assessed as related to the investigational product** as AESIs.	To specify that allergic reactions and not allergic-like reactions that are assessed as related to the Investigational product by the site Investigators will be collected.

#	Section	Change	Rationale
56.	Section 13.1.10	The first sentence of the first paragraph has been revised as follows:<br>The IDMC will consist of a multidisciplinary group of **two** ~~three~~ clinicians and/or physicians and one biostatistician who will, collectively, have extensive experience in the use of vaccines for patient treatment and in the conduct and safety monitoring of randomized clinical trials.	To clarify that there will only be three member of the IDMC.
57.	Section 14.1.3 (Preparation and Administration of Study Vaccine)	The third paragraph has been revised as follows:<br>The CoVLP formulation will be administered on study Day 0 and Day 21 as an IM injection of 0.5 mL **or 0.25 mL (as applicable)**, into the deltoid muscle. For subjects < 18 years of age and adult subjects with BMI < 30 kg/m^2, a 23 gauge needle of at least one inch or 2.5 cm in length can be used for vaccination. For subjects with BMI ≥ 30 kg/m^2, a needle of at least 1.5 inches or 3.5 cm in length must be used to ensure intramuscular delivery.	To clarify that a volume of 0.5 mL and 0.25 mL will be injected during the study.
58.	Section 19.5 (Appendix 5)<br>AND<br>Section 10.4.1 (Efficacy Evaluation)	Two tables listing the symptoms and risk factors associated with COVID-19 and COVID-19 disease severity have been added to the protocol.<br>A reference to this section has been added to the last sentence of the third paragraph as follows:<br>At the visit, subjects will be trained on daily recording of their symptoms **(refer to Section 19.5)** using a COVID-19 diary (refer to Section 19.6) and collecting self-administered nasal/NP/buccal swabs for testing using a virologic method.	To include additional information about COVID-19 symptoms and disease severity in the protocol.
59.	Section 19.6 (Appendix 6)<br>AND<br>Section 10.4.1 (Efficacy Evaluation)	Sample pages of the diary to collect COVID-19 symptoms have been added to the protocol.<br>A reference to this section has been added to the last sentence of the third paragraph as follows:	The COVID-19 diary will be used by subjects to document their COVID-19 symptoms during the surveillance period of the study.

#	Section	Change	Rationale
		At the visit, subjects will be trained on daily recording of their symptoms (refer to Section 19.5) using a COVID-19 diary **(refer to Section 19.6)** and collecting self-administered nasal/NP/buccal swabs for testing using a virologic method.	

Statistical Analysis Plan for Interventional Studies

Sponsor Name: Medicago R&D Inc.

Protocol Number: CP-PRO-CoVLP-021
Protocol Title: A Randomized, Observer-Blind, Placebo-Controlled, Phase 2/3 Study to Assess the Safety, Efficacy, and Immunogenicity of a Recombinant Coronavirus-Like Particle COVID-19 Vaccine in Adults 18 Years of Age or Older and Children and Adolescents 5-17 Years of Age

Protocol Version and Date: Final Version 2.0 (06-Oct-2020)

Syneos Health Project Code: [Redacted]

Authors: [Redacted]

Notice of Confidential and Proprietary Information:

The information contained in this document is confidential belonging to Medicago R&D Inc.. Acceptance of this document constitutes agreement by the recipient that no information contained herein will be published or disclosed without prior written authorization from an official of Medicago R&D Inc. However, this document may be disclosed to appropriate Institutional Review Board and Ethics Committees or duly authorized representatives of a national regulatory authority under the condition that they are requested to keep it confidential. In the event of an actual or suspected breach of this obligation, Syneos Health should be notified promptly.

Revision History

Version #	Date (DD-Mmm-YYYY)	Document Owner	Revision Summary
0.1	20-Oct-2020	[Redacted]	Initial Release Version
1.0	29-Oct-2020	[Redacted]	Final Version

I confirm that I have reviewed this document and agree with the content.

**Approvals**		
**Syneos Health Approval**<br><br>[Redacted]		
Name, Title<br>Author Biostatistician	Signature	Date (DD-Mmm-YYYY)
[Redacted]		
Name, Title<br>Senior Reviewing Biostatistician	Signature	Date (DD-Mmm-YYYY)
**Medicago Approval**<br><br>[Redacted]		
Name, Title<br>Sponsor Contact	Signature	Date (DD-Mmm-YYYY)

Table of Contents

Revision History 2
Approvals....................3
1. Purpose 12
  1.1. Responsibilities....................12
  1.2. Timings of Analyses....................12
2. Study Objectives....................16
  2.1. Primary Objectives....................16
  2.2. Secondary Objectives 16
  2.3. Exploratory Objectives 19
3. Study Details/Design....................21
  3.1. Brief Description....................21
  3.2. Subject Selection....................22
    3.2.1. Inclusion Criteria....................22
    3.2.2. Exclusion Criteria....................26
  3.3. Determination of Sample Size....................29
  3.4. Treatment Assignment and Blinding....................30
  3.5. Administration of Study Medication....................30
  3.6. Study Procedures and Flowchart 32
4. Endpoints 38
  4.1. Efficacy Endpoints 38
    4.1.1. Primary Endpoints....................38
    4.1.2. Secondary Endpoints 38
    4.1.3. Exploratory Endpoints 38
  4.2. Immunogenicity Endpoints....................39
    4.2.1. Primary Endpoints....................39
    4.2.2. Secondary Endpoints 39

This document is confidential.

4.2.3. Exploratory Endpoints ... 40
4.3. Safety Endpoints ... 40
4.3.1. Primary Endpoints ... 40
4.3.2. Secondary Endpoints ... 41
4.3.3. Exploratory Endpoints ... 41
5. Analysis Sets ... 42
5.1. Safety Analysis Set ... 42
5.2. Intention-To-Treat Set ... 42
5.3. Per Protocol Set ... 42
5.4. Immunogenicity Per Protocol Set ... 42
6. General Aspects for Statistical Analysis ... 44
6.1. General Methods ... 44
6.1.1. Geometric Mean Titer (GMT) ... 44
6.1.2. Geometric Mean Fold Rise (GMFR) ... 44
6.1.3. Seroconversion (SC) Rate ... 44
6.2. Key Definitions ... 45
6.2.1. Baseline ... 45
6.2.2. Change from Baseline ... 45
6.2.3. Onset Day ... 45
6.3. Missing Data ... 45
6.3.1. Safety Data ... 45
6.3.2. Immunogenicity ... 47
6.4. Visit Windows ... 47
6.5. Pooling of Centers ... 47
6.6. Subgroups ... 47
7. Demographic, Other Baseline Characteristics and Medication ... 49
7.1. Subject Disposition and Withdrawals ... 49

This document is confidential.

7.2. Demographic and Baseline Characteristics 49
7.3. Medical History and Concomitant Diseases 50
7.4. Medication 50
7.4.1. Prior Medication 50
7.4.2. Concomitant Medication 50
7.5. Treatment Compliance 51
8. Efficacy 52
8.1. Primary Efficacy Endpoint(s) and Analysis 52
8.2. Secondary Efficacy Endpoint(s) and Analyses 53
8.2.1. First occurrence of laboratory-confirmed (virologic method) symptomatic SARS-CoV-2 infection (≥ 14 days post-last vaccination) in a subject 53
8.2.2. Occurrences of severe COVID-19 disease (≥ 14 days post-last vaccination) 53
8.2.3. The slope estimate of the weighted Schoenfeld residuals against event time for the evaluation of absolute vaccine efficacy determined from the first occurrence of laboratory-confirmed (virologic method) symptomatic SARS-CoV-2 infection (≥ 14 days post-last vaccination) in a subject 53
8.2.4. Occurrences of laboratory-confirmed (confirmed by the ELISA method for the N protein) asymptomatic SARS-CoV-2 infection at Day 201 and Day 386 54
8.2.5. Occurrences of severe COVID-19 disease (≥ 14 days post-last vaccination) 54
8.2.6. Occurrence and intensity of COVID-19-related symptoms up to resolution of the symptoms 54
8.3. Exploratory Efficacy Endpoint(s) and Analyses 54
8.3.1. Occurrence and intensity of COVID-19-related symptoms up to resolution of the symptoms 54
9. Immunogenicity 56
9.1. Primary Immunogenicity Endpoint(s) and Analysis 56
9.1.1. Nab response induced in each Study Population against the SARS-CoV-2 virus on Days 0, 21, and 42 using GMT, SC rate, and GMFR 56
9.1.2. Specific Th1 CMI response induced in each Study Population against the SARS-CoV-2 virus on Days 0, 21, and 42 measured by IFN-γ ELISpot 57

9.2. Secondary Immunogenicity Endpoint(s) and Analysis 57

9.2.1. Persistence of the Nab antibody response induced in each Study Population against the SARS-CoV-2 virus on Day 201 and Day 386 using GMT, SC rate, and GMFR 57

9.2.2. Specific antibody response induced in each Study Population against the SARS-CoV-2 virus by the total IgG levels on Days 0, 21, and 42, and the persistence of these antibodies on Days 201 and 386 using GMT, SC rate, and GMFR 58

9.2.3. The ratio of neutralizing antibody titers:IgG ELISA antibody titers at Day 21, Day 42, Day 201, and Day 386....58

9.2.4. Specific Th1 CMI response induced in each Study Population against the SARS-CoV-2 virus on Day 201 and Day 386 measured by IFN-γ ELISpot....58

9.2.5. Specific Th2 CMI response induced in each Study Population against the SARS-CoV-2 virus on Days 0, 21, 42, 201, and 386 measured by IL-4 (ELISpot)....59

9.2.6. Nab antibody response induced in each Study Population against the SARS-CoV-2 virus on Days 0, 21, 42, 201, and 386 using GMT, SC rate, and GMFR....59

9.2.7. Specific antibody response induced in each Study Population against the SARS-CoV-2 virus by the total IgG levels on Days 0, 21, 42, 201, and 386 using GMT, SC rate, and GMFR 59

9.2.8. The ratio of neutralizing antibody titers:IgG ELISA antibody titers at Day 21, Day 42, Day 201, and Day 386....59

9.2.9. Specific Th1 CMI response induced in each Study Population against the SARS-CoV-2 virus on Days 0, 21, 42, 201, and 386 measured by IFN-γ ELISpot....59

9.2.10. Specific Th2 CMI response induced in each Study Population against the SARS-CoV-2 virus on Days 0, 21, 42, 201, and 386 measured by IL-4 (ELISpot)....59

9.2.11. Specific CMI response induced in each Study Population against the SARS-CoV-2 virus up to 21 days after the last vaccination and at Day 21, Day 42, Day 201 and Day 386 measured by the percentage of CD4+ T cells expressing functional markers....60

9.3. Exploratory Immunogenicity Endpoint(s) and Analysis 60

9.3.1. Further characterization of the immune response of the CoVLP formulation....60

10. Safety....61

10.1. Extent of Exposure....62

10.2. Adverse Events....62

10.2.1. Solicited Local and Systemic Adverse Events 63
10.2.2. Unsolicited Adverse Events 63
10.2.3. Serious Adverse Events (SAE) 63
10.2.4. Medically Attended Adverse Events (MAAE) 63
10.2.5. Adverse Events of Special Interest (AESI) 64
10.3. Laboratory Evaluations 64
10.4. Vital Signs 66
10.5. Primary Safety Endpoint(s) and Analysis 66
10.5.1. Occurrence, intensity, and relationship to vaccination of immediate AEs (30 minutes after each vaccination) 66
10.5.2. Occurrence, intensity, and relationship to vaccination of solicited local and systemic AEs (for seven days following each vaccine administration) 66
10.5.3. Occurrence, intensity, and relationship of unsolicited AEs for 21 days following each vaccine administration 67
10.5.4. Number and percentage of subjects with normal and abnormal clinically significant urine, haematological and biochemical values prior to and three days following each vaccination 67
10.5.5. Occurrences of SAEs, MAAEs, AEs leading to withdrawal, AESIs (including VED), and deaths up to 21 days following each vaccine administration 67
10.6. Secondary Safety Endpoint(s) and Analysis 67
10.6.1. Occurrences of SAEs, MAAEs, AEs leading to withdrawal, AESIs (including VED), and deaths from Day 43 to Day 201 68
10.6.2. Occurrences of SAEs, MAAEs, AEs leading to withdrawal, AESIs (including VED), and deaths from Day 202 to Day 386 68
10.6.3. Occurrence, intensity, and relationship to vaccination of immediate AEs (30 minutes after each vaccination) 68
10.6.4. Occurrence, intensity, and relationship to vaccination of solicited local and systemic AEs (for seven days following each vaccine administration) 68
10.6.5. Occurrence, intensity, and relationship of unsolicited AEs for 21 days following each vaccine administration 68

10.6.6. Occurrences of SAEs, MAAEs, AEs leading to withdrawal, AESIs (including VED), and deaths up to 21 days following each vaccine administration 68
10.6.7. Occurrences of SAEs, MAAEs, AEs leading to withdrawal, AESIs (including VED, and deaths from Day 43 to Day 201 68
10.6.8. Occurrences of SAEs, MAAEs, AEs leading to withdrawal, AESIs (including VED), and deaths from Day 202 to Day 386 68
10.7. Exploratory Safety Endpoint(s) and Analysis 69
10.7.1. Further characterization of the safety profile of the CoVLP formulation 69
10.8. Pregnancy 69
10.9. Physical Examination 69
11. Interim Analyses 70
12. Changes from Analysis Planned in Protocol 73
13. Reference List 74
14. Programming Considerations 75
14.1. General Considerations 75
14.2. Table, Figure, and Listing Format 75
14.2.1. General 75
14.2.2. Headers 76
14.2.3. Display Titles 76
14.2.4. Column Headers 76
14.2.5. Body of the Data Display 77
14.2.6. Footnotes 80
15. Quality Control 81
16. Index of Tables 82
17. Index of Figures 95
18. Index of Listings 96
19. Appendices 98

Glossary of Abbreviations

Abbreviation	Description
AE	adverse event
AESI	adverse event of special interest
ANOVA	analysis of variance
ANCOVA	analysis of covariance
BMI	body mass index
BP	blood pressure
CBER	Center for Biologics Evaluation and Research
CI	confidence interval
CMI	cell-mediated immune (response)
eCRF	electronic case report form
ELISA	enzyme-linked immunosorbent assay
ELISpot	enzyme-linked immuno spot assay
FDA	Food and Drug Administration
GCP	good clinical practice
GMFR	geometric mean fold rise or seroconversion factor
GMT	geometric mean titer
HR	heart rate
IB	Investigator's Brochure
ICF	informed consent form
ICH	International Council for Harmonisation
IDMC	Independent Data Monitoring Committee
IEC	independent ethics committee
IFN-γ	interferon gamma
IM	intramuscular
IRB	institutional review board
IRT	interactive response technology

ITT	intention-to-treat
LAR	Legally Authorized Representative
MedDRA	Medical Dictionary for Regulatory Activities
Nab	neutralizing antibody
OR	odds ratio
OT	oral temperature
PBMC	peripheral blood mononuclear cell
pIMD	potential immune-mediated disease
PP	per protocol
RR	respiratory rate
SAE	serious adverse event
SAP	statistical analysis plan
SAS	safety analysis set
SAS®	Statistical Analysis System®
SC	seroconversion
VE	vaccine efficacy
VED	vaccine-enhanced disease
VLP	virus-like particle
WHO	World Health Organization
US	United States

1. Purpose

The purpose of this statistical analysis plan (SAP) is to ensure that the data listings, summary tables and figures which will be produced, and the statistical methodologies which will be used, are complete and appropriate to allow valid conclusions regarding the study objectives.

1.1. Responsibilities

Syneos Health will perform the statistical analyses and is responsible for the production and quality control of all tables, figures and listings.

1.2. Timings of Analyses

Primary analysis for Phase 2 is up to 21 days after last vaccination. For Phase 3, the primary analysis for efficacy will occur when 160 cases will be collected.

The IDMC will consist of a multidisciplinary group of two clinicians and/or physicians and one biostatistician who will, collectively, have extensive experience in the use of vaccines for patient treatment and in the conduct and safety monitoring of randomized clinical trials. The IDMC will monitor the study conduct and review blinded and unblinded safety data at pre-defined times during the study for the purpose of providing recommendations to the Sponsor about whether:

- subject vaccination in different Study Populations will occur;
- subject vaccination in each Study Population will continue;
- the study portion will continue in the event of a pre-defined safety or efficacy signal.

Table 1 Timing of Analyses

Timing of Analyses	Population	Purposes	Requirements
**Phase 2**			
IDMC 1	3, 4	• Reviewing solicited Day 7 safety data for 10 sentinel subjects	• See IDMC Charter for more details (safety)
IDMC 2	3, 4	• Reviewing Day 28 safety data for 10 sentinel subjects;<br>• Reviewing Day 21 safety and immunogenicity data for all subjects	• See IDMC Charter for more details (safety)<br>• Day 21 Nab and INF ELIspot data<br>• Nab % of responders

		• Assessing if dose escalation is required<br>• Evaluating if these populations can be enrolled in Phase 3	• INF ELIspot % of responders
IDMC 3	1	• Reviewing Day 28 safety data<br>• Reviewing Day 21 immunogenicity data<br>• Evaluating if this population can be enrolled in Phase 3	• See IDMC Charter for more details (safety)<br>• Day 21 Nab and INF ELIspot data
IDMC 4	2/Adolescent	• Reviewing solicited Day 7 safety data for 10 sentinel subjects<br>• Assessing if dose de-escalation is required	• See IDMC Charter for more details (safety)
IDMC 5	2/Adolescent	• Reviewing Day 28 safety data for 10 sentinel subjects<br>• Assessing if dose de-escalation is required	• See IDMC Charter for more details (safety)<br>•
IDMC 6	2/Adolescent	• Reviewing Day 28 safety data for all subjects<br>• Reviewing day 21 immunogenicity data for all subjects<br>• Evaluating if this population can be enrolled in Phase 3	• See IDMC Charter for more details (safety)<br>• Day 21 Nab and INF ELIspot data
IDMC 7	2/Children	• Reviewing 7-day safety data for 10 sentinel subjects after first vaccine dose	• See IDMC Charter for more details (safety)
IDMC 8	2/Children	• Reviewing Day 21 safety and immunogenicity data<br>• Reviewing Day 28 safety data for 10 sentinel subjects<br>• Evaluating if this population can be enrolled in Phase 3	• See IDMC Charter for more details (safety)<br>• Day 21 Nab and INF ELIspot data
Ad-hoc IDMC (if needed)	3, 4	• Reviewing Day 28 safety data for all subjects<br>• Reviewing Day 42 immunogenicity data<br>• If the Day 21 immunogenicity data are not conclusive: Using Day 42 immunogenicity data to evaluate if these populations can be enrolled in Phase 3 and	• See IDMC Charter for more details (safety)<br>• Day 42 Nab and INF ELIspot data<br>• Day 42 Nab % of responders<br>• Day 42 INF ELISpot % of responders

This document is confidential.

		assess if dose escalation is required	
Day 42 Timepoint	All	• To deliver to identified unblinded people at Medicago all TFLs summarizing data up to Day 42 visit	• All TLFs presenting data up to Day 42 visit
Ad-hoc IDMC (if needed)	2	• Reviewing Day 28 safety data for all subjects<br>• Reviewing Day 42 immunogenicity data<br>• If the Day 21 immunogenicity data are not conclusive: Using Day 42 immunogenicity data to evaluate if this populations can be enrolled in Phase 3	• See IDMC Charter for more details (safety)<br>• Day 42 Nab and INF ELIspot data<br>• All TLFs up to Day 42
Day 201 Timepoint		• To deliver to identified unblinded people at Medicago all TFLs summarizing data up to Day 42 visit	• All TLFs presenting data up to Day 201 visit
End of Study	All	• Preparation of the CSR	• All TLFs specified in SAP mock shells
**Phase 3**			
Ad-hoc IDMC	All	• Assessing early efficacy once 80 positive COVID-19 cases will be collected<br>• Assessing futility	• Efficacy assessment for the primary endpoint<br>• Occurrences of COVID-19-related symptoms and their severity summarized by treatment using descriptive<br>• Distribution of severe COVID-19 cases between the CoVLP and placebo groups
Ad-hoc IDMC	All	• Performing primary efficacy analysis once 160 positive COVID-19 cases will be collected	• Efficacy assessment for the primary and secondary endpoint<br>• Occurrences of COVID-19-related symptoms and their severity summarized by treatment using descriptive

			• Distribution of severe COVID-19 cases between the CoVLP and placebo groups
End of Study	All	• Preparation of the CSR	• All TLFs specified in SAP mock shells

An interim analysis for an early efficacy assessment will be performed when 80 laboratory-confirmed symptomatic COVID-19 cases have been collected in the Phase 3 portion.

2. Study Objectives

2.1. Primary Objectives

The primary objectives of the Phase 2 portion of the study are:

Safety:

- To assess the safety and tolerability of the CoVLP formulation, compared to placebo, up to 21 days after the last vaccination;

Immunogenicity:

- To assess the immunogenicity of the CoVLP formulation, compared to placebo, up to 21 days after the last vaccination, as determined by:
    - Neutralizing antibody titers induced by the vaccine against the SARS-CoV-2 virus;
    - IFN-γ ELISpot response induced by the vaccine against the SARS-CoV-2 virus to assess the Th1 type response.

The primary objective of the Phase 3 portion of the study is:

Efficacy:

- To evaluate the absolute efficacy of the CoVLP formulation, compared to placebo, in the prevention of laboratory-confirmed symptomatic SARS-CoV-2 infection (virologic method) starting 14 days after the last vaccination.

2.2. Secondary Objectives

The secondary objectives of the Phase 2 portion of the study are:

Immunogenicity:

- To assess the immunogenicity of the CoVLP formulation, compared to placebo, as determined by the:
    - Persistence of neutralizing antibody titers induced by the vaccine against the SARS-CoV-2 virus at Days 201 and Day 386;
    - IgG (ELISA) titers induced by the vaccine against the SARS-CoV-2 virus up to 21 days after the last vaccination and at persistence of these antibodies Day 201 and Day 386;

This document is confidential.

  - The ratio of neutralizing antibody titers:IgG (ELISA) antibody titers at Day 21, Day 42, Day 201, and Day 386;
  - IFN-γ ELISpot response induced by the vaccine against the SARS-CoV-2 virus at Day 201 and Day 386;
  - Interleukin-4 (IL-4) ELISpot response induced by the vaccine against the SARS-CoV-2 virus up to 21 days after the last vaccination and at Day 201 and Day 386;

Safety:

- To assess the safety and tolerability of the CoVLP formulation, compared to placebo, as determined by the occurrence(s) of:
  - Medically attended adverse events (MAAEs), serious adverse events (SAEs), AEs leading to withdrawal, AEs of special interest (AESIs; including vaccine-enhanced disease [VED]), and deaths from 22 days after the last vaccination up to the end of the study;

Efficacy:

- To evaluate the absolute efficacy of the CoVLP formulation, compared to placebo, in the prevention of laboratory-confirmed symptomatic SARS-CoV-2 infection (virologic method) starting 14 days after the last vaccination;
- To evaluate the absolute efficacy of the CoVLP formulation, compared to placebo, in the prevention of severe COVID-19 disease starting 14 days after the last vaccination. Severe COVID-19 disease will be defined as virologically-confirmed SARS-CoV-2 infection with the occurrence of a new onset of any of the following:
  - Clinical signs at rest indicative of severe systemic illness (respiratory rate ≥ 30 per minute, heart rate ≥ 125 per minute, SpO2 ≤ 93% on room air at sea level or PaO2/FiO2 < 300 mmHg);
  - Respiratory failure (defined as needing high-flow oxygen, non-invasive ventilation, mechanical ventilation or ECMO);
  - Evidence of shock (SBP < 90 mmHg, DBP < 60 mmHg, or requiring vasopressors);
  - Significant acute renal, hepatic, or neurologic dysfunction;
  - Hospitalization;
  - Death.

  The list above applies to the adult populations; for children and adolescents, the following list of occurrences is applicable:

- Severe pneumonia:
  - Polypnea: ≥ 30 times/min (>5 years) (after ruling out the effects of fever and crying);
  - Oxygen saturation < 92% under a resting state;
  - Dyspnea: assisted breathing (moans, nasal flaring, and three concave sign), cyanosis, intermittent apnea;
  - Disturbance of consciousness: somnolence, coma, or convulsion;
  - Pulmonary high-resolution CT (HRCT) examination showing bilateral or multi-lobe infiltrates, rapid progression of disease in a short period or with pleural effusion.
- Critical cases: those who meet any of the following criteria and require hospitalization:
  - Respiratory failure requiring mechanical ventilation;
  - Shock;
  - Combined with other organs failure;
- Death.

The secondary objectives of the Phase 3 portion of the study are:

Efficacy:

- To evaluate the duration of protection (vaccine efficacy) of the CoVLP formulation compared to placebo;
- To evaluate the absolute efficacy of the CoVLP, compared to placebo, in the prevention of laboratory-confirmed asymptomatic SARS-CoV-2 infection (serologic method) at Day 201 and Day 386;
- To evaluate the absolute efficacy of the CoVLP formulation, compared to placebo, in the prevention of severe COVID-19 disease starting 14 days after the last vaccination (see definition for severe COVID-19 disease above);
- To assess COVID-19-related symptoms, and the severity of these symptoms, up until resolution of subjects administered the CoVLP formulation compared to subjects administered the placebo;

Immunogenicity:

- To assess the immunogenicity of the CoVLP formulation, compared to placebo, in a subset of subjects, as determined by:

- Neutralizing antibody titers induced by the vaccine against the SARS-CoV-2 virus up to 21 days after the last vaccination and at Day 201 and Day 386;
- IgG (ELISA) titers induced by the vaccine against the SARS-CoV-2 virus up to 21 days after the last vaccination and at Day 201 and Day 386;
- The ratio of neutralizing antibody titers:IgG (ELISA) antibody titers at Day 21, Day 42, Day 201, and Day 386;
- IFN-γ ELISpot response induced by the vaccine against the SARS-CoV-2 virus up to 21 days after the last vaccination and at Day 201 and Day 386;
- IL-4 ELISpot response induced by the vaccine against the SARS-CoV-2 virus up to 21 days after the last vaccination and at Day 201 and Day 386;
- Specific cell-mediated immune (CMI) response induced by the vaccine against the SARS-CoV-2 virus up to 21 days after the last vaccination and at Day 201 and Day 386, as measured by the percentage of CD4+ T cells expressing functional markers;

Safety:

- To assess the safety and tolerability of the CoVLP formulation, compared to placebo, up to the end of the study.

2.3. Exploratory Objectives

The exploratory objectives of the Phase 2 portion of the study are:

Efficacy:

- To assess COVID-19-related symptoms, and the severity of these symptoms, up until resolution of subjects administered the CoVLP formulation compared to subjects administered the placebo;

Immunogenicity:

- To further characterize the immune response of the CoVLP formulation, if it is likely to be informative;

Safety:

- To further characterize the safety profile of the CoVLP formulation, if it is likely to be informative.

This document is confidential.

The exploratory objectives of the Phase 3 portion of the study are:

Immunogenicity:

- To further characterize the immune response of the CoVLP formulation, if it is likely to be informative;

Safety:

- To further characterize the safety profile of the CoVLP formulation, if it is likely to be informative.

This document is confidential.

3. Study Details/Design

3.1. Brief Description

The Phase 2 portion is a randomized, observer-blinded, placebo-controlled study with male and female subjects. Subjects will be enrolled from four Study Populations (refer to Table 1).

In each of the four Study Populations, 120 subjects will be randomized 4:1 and will receive the same CoVLP formulation or placebo. Subjects will receive two IM injections 21 days apart on Days 0 and 21, into the deltoid region of the alternating arm (each arm will be injected once), of one of the following:

- CoVLP formulation;
- Placebo.

Adults 18 years of age and older (i.e. Study Populations #1, #3, and #4) will begin enrollment at the same time. Enrollment of elderly adult subjects (Study Population #3) will be stratified by age in a 2:1 ratio of 65 to 74 years of age and 75 years of age and older, respectively. Enrollment of healthy children and adolescents aged 5 to 17 years (i.e. Study Population #2) will begin only after the safety data following the second vaccination for the Healthy Adult population (i.e. Day 28 for all subjects in Study Population #1) have been reviewed by the Independent Data Monitoring Committee (IDMC). Study Population #2 will be stratified by age in a 1:1 ratio of adolescents 10-17 years of age followed by children 5-9 years of age. Enrollment of subjects in Study Population #4 will include adults with co-existing conditions such as, but not limited to, obesity or being overweight, documented hypertension, cardiovascular diseases, hyperlipidemia, chronic kidney disease or related disorders, chronic respiratory disease, pre-diabetes and diabetes (type 1 and type 2), treatment-controlled HIV, and psychiatric disorders.

The IDMC will review unblinded safety data for the first 10 sentinel subjects after each dose in Study Populations #2, #3, and #4 prior to vaccinating the remaining subjects in each Study Population according to the details outlined in the IDMC Charter. These IDMC reviews will detect any early negative trends in the safety data from a subset of subjects vaccinated within each Study Population and may necessitate a decision to not administer the study vaccine to the remaining study subjects. During the Phase 2 portion, the IDMC will review the safety data collected up to Day 28 from all the subjects in each Study Population to confirm that each Study Population can be included in the Phase 3 portion of the study.

The Phase 3 portion is a randomized, observer-blinded, placebo-controlled, efficacy, safety, immunogenicity (in a subset of subjects), multicenter design with male and female subjects.

Similar to the Phase 2 portion, subjects will be enrolled from five Study Populations (refer to Table 1). Study Populations #2 and #3 will be stratified by age in the same manner. The overall goal of the Phase 3 portion of this study is to enroll approximately 30 000 subjects who are generally representative of the overall target population in terms of racial diversity with some degree of enrichment for those at highest risk for serious COVID-19 disease (i.e. the elderly and those with co-morbidities) and relatively smaller numbers of the subjects at lowest risk of serious COVID-19 (i.e. children and adolescents). In addition, Study Population #5 enrollment will only begin after the administration of the second vaccination of all subjects in Study Populations #1 to #4.

In each Study Population, subjects will be randomized into one of two treatment groups:

- CoVLP formulation;
- Placebo.

Table 2 Study Populations

Study Population	Age and/or Health Category	Age Range (years)
1	Healthy adults	18-64
2	Healthy children and adolescents	5-17
3	Elderly adults	≥65
4	Adults with significant co-morbidities	≥18
5*	Healthy female adults who are in the 3rd trimester of their low-risk pregnancy	≥18

*This study population is only for the Phase 3 portion of the study.

3.2. Subject Selection

3.2.1. Inclusion Criteria

Subjects must meet all of the following inclusion criteria at the Screening (Visit 1) and/or Vaccination (Visit 2) visits to be eligible for participation in this study; no protocol waivers are allowed. All Investigator assessment-based judgements must be carefully and fully documented in the source documents:

1. Subjects (and/or their parent/LAR) must have read, understood, and signed the informed consent form (ICF) prior to participating in the study; subjects must also complete study-related procedures and the subjects (and/or their parent/LAR) must communicate with the study staff at visits and by phone during the study;

2. At the Screening visit (Visit 1), male and female subjects must be
    - Study Populations #1 and #5: 18 to 64 (has not yet had his/her 65th birthday) years of age, inclusive;
    - Study Population #2: 5 to 17 (has not yet had his/her 18th birthday) years of age, inclusive;
    - Study Population #3: 65 years of age or older;
    - Study Population #4: 18 years of age or older;
3. At Screening (Visit 1) and Vaccination (Visit 2), subject must have a body mass index (BMI) of:
    - Study Populations #1, #3, and #5: ≥ 18.5 and < 30 kg/m^2;
    - Study Population #2: > 5th and < 85th percentile based on the CDC 'BMI for 'age' scale (https;//www.cdc.gov/healthyweight/bmi/calculator.html);
4. Subjects (and/or their parent/LAR) are considered by the Investigator to be reliable and likely to cooperate with the assessment procedures and be available for the duration of the study;
5. Study Populations #1, #2, and #5: Subjects must be in good general health prior to study participation, with no clinically relevant abnormalities that could jeopardize subject safety or interfere with study assessments, as determined by medical history, physical examination, and vital signs. Investigator discretion will be permitted with this inclusion criterion;

   Note: Subjects with a pre-existing chronic disease will be allowed to participate if the disease is stable and, according to the Investigator's judgment that must be documented in the source documents, the condition is unlikely to confound the results of the study or pose additional risk to the subject by participating in the study. Stable disease is generally defined as no new onset or exacerbation of pre-existing chronic disease three months prior to vaccination. Based on the Investigator's judgment, a subject with more recent stabilization of a disease could also be eligible.

6. Study Populations #1, #2, and #4: Female subjects of childbearing potential must have a negative serum pregnancy test result at Screening (Visit 1) and a negative urine pregnancy test result at Vaccination (Visit 2):

   Non-childbearing females are defined as:

   - Surgically-sterile (defined as bilateral tubal ligation, hysterectomy or bilateral oophorectomy performed more than one month prior to the first study vaccination); or
   - Post-menopausal (absence of menses for 12 consecutive months and age consistent with natural cessation of ovulation);
   - Non-ovulating, pre-menstrual girls < 17 years of age;

7. Study Population #5: Female subjects who are in the 3rd trimester of a low-risk pregnancy at Screening (Visit 1) can be enrolled in the study;

8. Study Populations #1, #2, and #4: Female subjects of childbearing potential must use an effective method of contraception for one month prior to vaccination (Visit 2) and agree to continue employing highly effective birth control measures for at least one month after the last study vaccination (or in the case of early termination, she must not plan to become pregnant for at least one month after her last study vaccination), with the exception of the following subjects:

   The following relationship or methods of contraception are considered to be highly effective:

   - Combined (estrogen and progestogen containing) hormonal contraception associated with inhibition of ovulation:
     - Oral;
     - Intravaginal;
     - Transdermal;
   - Progestogen-only hormonal contraception associated with inhibition of ovulation:

  - Oral;
  - Injectable;
  - Implantable;
- Intra-uterine device with or without hormonal release;
- Vasectomised partner, provided that this partner is the sole sexual partner of the study participant and that the vasectomised partner has received a medical assessment of the surgical success;
- Credible self-reported history of heterosexual vaginal intercourse abstinence prior to and for at least one month after the last study vaccination. Abstinent subjects who are ovulating should be asked what method(s) they would use should their circumstances change, and subjects without a well-defined plan should be excluded. Ideally, the parent/LAR should not be present when a female subject < 18 years of age is reporting her sexual activity level and method of contraception;
- Female partner.

9. Study Population #3: Subjects must be non-institutionalized (e.g. not living in rehabilitation centres or old-age homes; living in an elderly community is acceptable) and have no acute or evolving medical problems prior to study participation and no clinically relevant abnormalities that could jeopardize subject safety or interfere with study assessments, as assessed by the Principal Investigator or sub-Investigator (thereafter referred as Investigator) and determined by medical history, physical examination, serology (only for the Phase 2 portion), clinical chemistry and haematology tests (only for the Phase 2 portion), urinalysis (only for the Phase 2 portion), and vital signs. Investigator discretion will be permitted with this inclusion criterion.

   Note: Subjects with a pre-existing chronic disease will be allowed to participate if the disease is stable and, according to the Investigator's judgment that must be documented in the source documents, the condition is unlikely to confound the results of the study or pose additional risk to the subject by participating in the study. Stable disease is generally defined as no new onset or exacerbation of pre-existing chronic disease three months prior to vaccination. Based on the Investigator's judgment and documented in source documentation, a subject with more recent stabilization of a disease could also be

eligible;

10. Study Population #4: Subjects must have one or more co-morbid conditions that puts them at higher risk for severe COVID-19 disease. These co-morbidities include but are not limited to obesity, documented hypertension, type-1 or type 2 diabetes, chronic obstructive lung diseases (COPD), cardiovascular disease, chronic kidney, or be immunocompromised persons (e.g., HIV-infected patients, organ transplant recipients, or patients receiving cancer chemotherapy). Investigator discretion will be permitted with this inclusion criterion.

3.2.2. Exclusion Criteria

Subjects who meet any of the following criteria at the Screening (Visit 1) and/or Vaccination (Visit 2) visits will not be eligible for participation in this study; no protocol waivers are allowed. All Investigator assessment-based judgements must be thoroughly documented in the source documents:

1. Study Populations #1, #2, #3, and #5: According to the Investigator's opinion, significant acute or chronic, uncontrolled medical or neuropsychiatric illness.

   Acute disease is defined as presence of any moderate or severe acute illness with or without a fever within 48 hours prior to the Screening (Visit 1) and/or Vaccination visit (Visit 2). 'Uncontrolled' is defined as:

   - Requiring a new medical or surgical treatment during the three months prior to study vaccine administration;
   - Requiring any significant change in a chronic medication (i.e. drug, dose, frequency) during the three months prior to study vaccine administration due to uncontrolled symptoms or drug toxicity unless the innocuous nature of the medication change meets the criteria outlined in inclusion criterion no. 5 (Study Population #1) or no. 9 (Study Population #3) and is appropriately justified and documented by the Investigator.

   Investigator discretion is permitted with this exclusion criterion and must be carefully and fully documented in the source documents;

2. Study Populations #1, #2, #3, and #5: Any confirmed or suspected current immunosuppressive condition or immunodeficiency, including cancer, human

immunodeficiency virus (HIV), hepatitis B or C infection (subjects with a history of cured hepatitis B or C infection without any signs of immunodeficiency at present time are allowed). Investigator discretion is permitted with this exclusion criterion;

3. Study Populations #1, #2, #3, and #5: Current autoimmune disease (such as rheumatoid arthritis, systemic lupus erythematosus or multiple sclerosis). Investigator discretion is permitted with this exclusion criterion, and subjects may be eligible to participate with appropriate written justification in the source document (i.e. subjects with a history of autoimmune disease who are disease-free without treatment for three years or more, or on stable thyroid replacement therapy, mild psoriasis [i.e. a small number of minor plaques requiring no systemic treatment], etc.);

4. Study Populations #1, #2, #3, and #5: Administration of any medication or treatment that may alter the vaccine immune responses, such as:

    - Study Populations #1, #3, and #5: Systemic glucocorticoids at a dose exceeding 10 mg of prednisone (or equivalent) per day for more than seven consecutive days or for 10 or more days in total, within one month prior to the Vaccination visit (Visit 2). Inhaled, nasal, ophthalmic, dermatological, and other topical glucocorticoids are permitted;

    - Study Population #2: Systemic glucocorticoids at a dose exceeding 0.28 mg / kg of prednisone (or equivalent) per day for more than seven consecutive days or for ten or more days in total, within one month prior to the Vaccination visit (Visit 2). Inhaled, nasal, ophthalmic, dermatological, and other topical glucocorticoids are permitted;

    - Cytotoxic, antineoplastic, or immunosuppressant drugs - within 36 months prior to Vaccination (Visit 2);

    - Any immunoglobulin preparations or blood products, blood transfusion – within 6 months prior to Vaccination (Visit 2);

5. Study Population #4: Acute disease defined as presence of any moderate or severe acute illness with or without a fever within 48 hours prior to the Screening (Visit 1) and/or Vaccination visit (Visit 2);

6. Administration of any vaccine within 30 days prior to Vaccination (Visit 2); planned administration of any vaccine during the study (up to blood sampling on Day 42 of the

study). Immunization on an emergency basis during the study will be evaluated on case-by-case basis by the Investigator;

7. Administration of any other SARS-CoV-2 / COVID-19, or other experimental coronavirus vaccine at any time prior to or during the study;

8. Use of any investigational or non-registered product within 30 days or 5 half-lives, whichever is longer, prior to Vaccination (Visit 2) or planned use during the study period. Subjects who are in a prolonged post-administration observation period of another investigational or marketed drug clinical study, for which there is no ongoing exposure to the investigational or marketed product and all scheduled on-site visits are completed, will be allowed to take part in this study, if all other eligibility criteria are met;

9. Have a rash, dermatological condition, tattoos, muscle mass, or any other abnormalities at injection site that may interfere with injection site reaction rating. Investigator discretion will be permitted with this exclusion criterion;

10. Use of any prescription antiviral drugs with the intention of COVID-19 prophylaxis, including those that are thought to be effective for prevention of COVID-19 but have not been licensed for this indication, within one month prior to Vaccination (Visit 2);

11. For the Phase 2 portion of the study only: Use of prophylactic medications (e.g. antihistamines [H1 receptor antagonists], nonsteroidal anti-inflammatory drugs [NSAIDs], systemic and topical glucocorticoids, non-opioid and opioid analgesics) within 24 hours prior to the Vaccination (Visit 2) to prevent or pre-empt symptoms due to vaccination;

12. History of a serious allergic response to any of the constituents of CoVLP including AS03;

13. History of a documented anaphylactic reactions to plants or plant components (including tobacco, fruits and nuts);

14. Subjects with a history of Guillain-Barré Syndrome;

15. Study Populations #1, #2, and #4: Any female subject who has a positive or doubtful pregnancy test result prior to vaccination or who is lactating;

This document is confidential.

16. Study Population #5: Any female subject who is in the 1st or 2nd trimester of her pregnancy or whose pregnancy is considered to be high-risk by her obstetrician;

17. Subjects identified as an Investigator or employee of the Investigator or clinical site with direct involvement in the proposed study, or identified as an immediate family member (i.e. parent, spouse, natural or adopted child) of the Investigator or employee with direct involvement in the proposed study, or any employees of Medicago.

3.3. Determination of Sample Size

Phase 2 portion:

The sample size of up to 480 subjects will serve to confirm vaccine immunogenicity in the four Study Populations and detect sizable differences in rates of adverse events. The sample size is not large enough to detect less frequent or rare adverse reactions. With 384 subjects receiving the CoVLP formulation, there will be at least 95 % probability of observing an adverse event that occurs as frequently as 1 in 128 vaccinees.

Phase 3 portion:

The sample size of up to 30 000 subjects has been calculated using an exact conditional Poisson approach based on the following assumptions:

- A 1:1 ratio for CoVLP formulation:placebo;
- At least 90% statistical power to reject the null hypothesis of 1) a lower limit of the alpha-adjusted confidence interval for an absolute vaccine efficacy of the CoVLP formulation compared to placebo ≤ 30% and 2) true efficacy < 50%, assuming a vaccine efficacy of 60%;
- A SARS-CoV-2 attack rate in unvaccinated subjects of 0.8 %;
- An attrition of 5 %;
- An interim analysis for an early efficacy assessment will be performed when 80 laboratory-confirmed COVID-19 cases have been collected in the Phase 3 portion;
- A statistical correction for the interim analysis will be applied that uses the O'Brien-Fleming alpha-spending function. With interim and final assessments at 80 and 160 cases, for example, the two-sided test p-value thresholds will be 0.0027 and 0.0478, respectively.

With 15 000 subjects receiving the CoVLP formulation, there will be at least 95 % probability of observing an adverse event that occurs as frequently as 1 in 5000 vaccinees.

This document is confidential.

An immunogenicity subset sample size of 480 subjects is deemed sufficient to provide meaningful statistical assessment of immunologic outcomes.

3.4. Treatment Assignment and Blinding

Both portions of the study will be observer-blinded.

During the entire duration of the study, the following individuals will not have access to treatment allocation (i.e. remain "blind"): the subjects (and/or their parent/LAR), the Investigators and all personnel involved in the clinical conduct of the study (except the staff involved in the preparation and administration of the study vaccine, the quality assurance auditor, and quality control reviewers), Medicago clinical staff and medical staff involved in safety evaluations (e.g. causality assessments), and all personnel involved in sample analysis at the central and testing (Nab, ELISA, ELISpot, ICS [flow cytometry], serological, and virologic method SARS-CoV-2 diagnostics) laboratories.

During the study, the IDMC and unblinded team, compromised of the unblinded medical monitor, unblinded biostatistician, unblinded senior reviewer, and unblinded programmer involved in the preparation of the safety data summary for the IDMC reviews, will have access to or be aware of treatment allocation (i.e. be "unblinded"). The IDMC will review safety data and will make recommendations for the entire duration of the study. The same unblinded statistical team will be involved in the Day 21 and Day 42 (if needed) immunogenicity data analyses in the Phase 2 portion and in the interim efficacy analyses in the Phase 3 portion.

During the study, the Sponsor's Study Team will remain blinded. A small number of Medicago personnel (not included in Study Team) will be involved in the planned analyses during the Phase 2 and Phase 3 portions. The selected individuals and the process and level of unblinding will be documented.

3.5. Administration of Study Medication

The CoVLP formulation for the Phase 2 portion of the study has been selected based on the Phase 1 study data, including acceptable safety and immunogenicity profiles. This Phase 2 portion is confirmatory for the dose to be used in each population for Phase 3.

Subjects will receive two intramuscular (IM) injections 21 days apart on Days 0 and 21, into the deltoid region of the alternating arm (each arm will be injected once), of one of the following:

- CoVLP formulation;

- Placebo.

The use of the same dose of the CoVLP formulation for all Study Populations may not be adequate. Subjects in Study Population #1 (healthy adults 18 to 64 years of age) will receive the full standard dose of CoVLP (3.75 µg) adjuvanted with AS03 in an injection volume of 0.5 mL.

In Study Population #2, subjects who are 10 to 17 years of age will receive 3.75 µg CoVLP adjuvanted with AS03 in 0.5 mL. Subjects who are 5-9 years of age will receive half the dose of the CoVLP formulation (i.e. 0.25 mL containing 1.87 µg CoVLP adjuvanted with half the standard dose of AS03). For the adolescents in Study Population #2, a dose de-escalation may occur to reduce the dose of CoVLP and AS03 by half (i.e. 0.25 mL of the full dose will be administered containing 1.87 µg CoVLP and half of the AS03 dose). Because reactogenicity in this population is likely to be attributable to the adjuvant, an alternate approach for these subjects may be to administer the standard antigen dose (i.e. 3.75 µg) with half the adjuvant dose in a 0.25 mL volume. If the safety profile is acceptable to the IDMC, then recruitment will continue until a total of 60 adolescents and 60 children receiving the same CoVLP formulation in each age cohort have been enrolled in Study Population #2. If the half dose of the CoVLP formulation is used in the Phase 2 portion for one or both age cohorts in Study Population #2, then the same formulation(s) will be used during the Phase 3 portion of the study.

Study Populations #3 and #4 (elderly adults or adults with significant comorbidities, respectively) will receive the full standard dose of CoVLP (3.75 µg) adjuvanted with AS03 in an injection volume of 0.5 mL. In the Phase 2 portion, if an acceptable immune response is not achieved at either Day 21 or Day 42, the IDMC may recommend a dose escalation to 7.5 µg CoVLP adjuvanted with AS03 in a volume of 0.5 mL, and the same number of subjects (i.e. 120 subjects per study population) in the particular study population will be recruited, using the same enrollment approach (i.e. use of sentinel subjects and IDMC reviews). If the higher dose of the CoVLP formulation achieves an acceptable immune response in the Phase 2 portion, then the same higher dose will be used in Study Populations #3 and/or #4 during the Phase 3 portion of the study.

The start of the Phase 3 portion of the study will be determined independently for each Study Population and will be based on the outcomes of the following two assessments during the Phase 2 portion of the study:

- Day 21 Data Analysis of the immunogenicity data;
- Day 28 Safety Review.

3.6. Study Procedures and Flowchart

Table 3 Time and Events Schedule: General Information for the Phase 2 Portion

Visit Type	Screening	Vaccination	Post-vaccination Visits/Contacts									
**Study Day**	**Day -14 to 0**	**Day 0**	**Day 1 (+ 1)**	**Day 3 (± 1)**	**Day 8 (± 1)**	**Day 21 (± 2)[8]**	**Day 22 (+ 1)**	**Day 24 (± 1)**	**Day 29 (± 1)**	**Day 42 (± 2)**	**Day 201 (± 14)**	**Day 386 (± 14)**
**Visit Number**	**1**	**2**	**Phone**	**3**	**Phone**	**4**	**Phone**	**5**	**Phone**	**6**	**7**	**8**
Informed consent	X											
Demographics	X											
Height, weight, and BMI	X	X										
Medical history/prior medication	X	X[1]										
History/symptom-directed physical examination	X[2]											
Vital Signs	X	X[3]		X		X[3]		X		X	X	X
Inclusion/exclusion criteria	X	X										
Urinalysis	X			X		X[9]		X				
Pregnancy test[4]	X	X[9]				X[9]				X		
Test for SARS-CoV-2 antibodies	X											
Serology tests for HIV, Hepatitis B, and Hepatitis C	X											
Blood chemistry And Haematology	X			X		X[9]		X				
Immunogenicity -Serology (Nab		X[9]				X[9]				X	X	X

**Visit Type**	**Screening**	**Vaccination**	**Post-vaccination Visits/Contacts**									
**Study Day**	**Day -14 to 0**	**Day 0**	**Day 1 (+ 1)**	**Day 3 (± 1)**	**Day 8 (± 1)**	**Day 21 (± 2)** [8]	**Day 22 (+ 1)**	**Day 24 (± 1)**	**Day 29 (± 1)**	**Day 42 (± 2)**	**Day 201 (± 14)**	**Day 386 (± 14)**
**Visit Number**	**1**	**2**	**Phone**	**3**	**Phone**	**4**	**Phone**	**5**	**Phone**	**6**	**7**	**8**
assay and ELISA)												
Immunogenicity – CMI Response (ELISpot and ICS)		X[9]				X[9]				X	X	X
Randomization		X										
Vaccine admin		X				X						
Immediate surveillance (30 minutes)		X				X						
Provide and collect diary and memory aid instructions		X		X		X		X		X	X	X
Oral digital thermometer and instructions on reactions[5]		X				X						
Collection of solicited local/ systemic adverse events		X	X	X	X	X	X	X	X			
Concomitant medications[6]		At any time during the study period										
Collection of COVID-19 symptoms through passive and active surveillance		Passive Surveillance: Subjects (and/or their parent/legally acceptable representative [LAR]) will be instructed to contact the study site if they experience symptoms of COVID-19 from 14 days after the last vaccination until the end of the Phase 2 or until 160 COVID-19 cases have been accumulated in the Phase 3 portion of the study.<br>Active Surveillance: Between 14 days after the last vaccination and the end of the Phase 2 or until 160 COVID-19 cases have been accumulated in the Phase 3 portion of the study, the subjects will be contacted once per week via the method most likely to be successful (e.g. subject's (and/or their parent/LAR's) preferred method [phone, text, email, electronic diary]), and asked about symptoms of COVID-19.										

**Visit Type**	**Screening**	**Vaccination**	**Post-vaccination Visits/Contacts**									
**Study Day**	**Day -14 to 0**	**Day 0**	**Day 1 (+ 1)**	**Day 3 (± 1)**	**Day 8 (± 1)**	**Day 21 (± 2)** [8]	**Day 22 (+ 1)**	**Day 24 (± 1)**	**Day 29 (± 1)**	**Day 42 (± 2)**	**Day 201 (± 14)**	**Day 386 (± 14)**
**Visit Number**	**1**	**2**	**Phone**	**3**	**Phone**	**4**	**Phone**	**5**	**Phone**	**6**	**7**	**8**
Testing for laboratory confirmation of SARS-CoV-2 infection		Subjects who report any symptoms associated with COVID-19 after the start of the active and passive surveillance will be requested to visit the Investigator site to provide nasal or NP swabs (2 per subject per event) to test for COVID-19. If the subject cannot visit the Investigator site, then the subject may receive a visit at home to collect the nasal/NP swabs samples or be provided with self-administered nasal/NP swabs for sample collection. The swabs will be collected within 72 hours (preferably within 48 hours) of the reporting of a COVID-19-associated symptom. If swabs cannot be collected within 72 hours after reporting of the COVID-19-associated symptom, nasal or NP swabs should be collected at the next available opportunity for the subject to visit the Investigator site.										
AEs, SAEs, MAAEs, and AESIs[7]		At any time during the study period.										
Termination record												X

[1] Record any changes in medical history and medications and confirmation that the subject continues to meet all inclusion and no exclusion criteria since screening.

[2] History/symptom-directed physical examinations will not be routinely performed at any other visits, unless new complaints or concerns are raised by either the study subject or study staff, and if deemed to be necessary by the Investigator.

[3] Record prior to study vaccine, after the observation period and as deemed necessary.

[4] In all females of childbearing potential in Study Populations #1, #2, and #4; it will be tested in serum at screening and in urine at Days 0, 21, and 42.

[5] After vaccination, subjects (and/or their parent/LAR) will be instructed on the diary and memory aid provided for their use for recording reactions, AEs, and concomitant medication use.

[6] After the Day 42 visit, concomitant medication collection will be limited to those used to treat an SAE, MAAE, AE leading to withdrawal, AESIs, or an AE that occurred before Day 42; any vaccine not foreseen in the study protocol; and prohibited medications.

[7] AEs will be collected up to Day 42; SAEs, MAAEs, AEs leading to withdrawal, and AESIs will be collected through to the end of the study. Investigators should report SAEs, MAAEs, AESIs, and pregnancy reports to Sponsor within 24 hours of the Investigator learning of the event. Specific contacts for the collection of information regarding all of these events will occur on Day 386 (during the Day 386 final visit) for SAEs, MAAEs, AEs leading to withdrawal, and AESI.

[8] All efforts will be done to have subjects returning on planned date for Day 21 activities. If for any reason the visit is done before or after this planned date, subsequent visits/procedures (Days 22, 24, 29, 42, 201, and 386) will be adjusted accordingly.
[9] Blood and urine samples are to be collected prior to vaccination on the respective day.

Table 4 Time and Events Schedule: General Information for the Phase 3 Portion

**Visit Type**	**Screening/Vaccination**	**Post-vaccination Visits/Contacts**							
**Study Day**	**Day 0**	**Day 1 (+ 1)**	**Day 8 (± 1)**	**Day 21 (± 2)** [8]	**Day 22 (+ 1)**	**Day 29 (± 1)**	**Day 42 (± 2)**	**Day 201 (± 14)**	**Day 386 (± 14)**
**Visit Number**	**1**	**Phone**	**Phone**	**2**	**Phone**	**Phone**	**3**	**4**	**5**
Informed consent	X								
Demographics	X								
Height, weight, and BMI	X								
Medical history/prior medication	X								
History/symptom-directed physical examination	X[2]								
Vital Signs	X[3]			X[3]					
Inclusion/exclusion criteria	X								
Pregnancy test [4]	X[9]			X[9]			X		
Test for SARS-CoV-2 N antibodies	X[9]							X	X
Immunogenicity - Serology (Nab assay and ELISA)[1]	X[9]			X[9]			X	X	X
Immunogenicity – CMI Response (ELISpot and ICS)[1]	X[9]			X[9]			X	X	X
Randomization	X								
Vaccine admin	X			X					

**Visit Type**	**Screening/Vaccination**	**Post-vaccination Visits/Contacts**							
**Study Day**	**Day 0**	**Day 1 (+ 1)**	**Day 8 (± 1)**	**Day 21 (± 2)** [8]	**Day 22 (+ 1)**	**Day 29 (± 1)**	**Day 42 (± 2)**	**Day 201 (± 14)**	**Day 386 (± 14)**
**Visit Number**	**1**	**Phone**	**Phone**	**2**	**Phone**	**Phone**	**3**	**4**	**5**
Immediate surveillance (30 minutes)	X			X					
Provide and collect diary and memory aid instructions	X			X			X	X	X
Oral digital thermometer and instructions on reactions[5]	X			X					
Collection of solicited local/ systemic adverse events	X	X	X	X	X	X			
Concomitant medications[6]	At any time during the study period								
Collection of COVID-19 symptoms through passive and active surveillance	Passive Surveillance: Subjects (and/or their parent/LAR) will be instructed to contact the study site if they experience symptoms of COVID-19 from 14 days after the last vaccination until the end of the Phase 2 or until 160 COVID-19 cases have been accumulated in the Phase 3 portion of the study.<br>Active Surveillance: Between 14 days after the last vaccination and the end of the Phase 2 or until 160 COVID-19 cases have been accumulated in the Phase 3 portion of the study, the subjects will be contacted once per week via the method most likely to be successful (e.g. subject's (and/or their parent/LAR's)preferred method [phone, text, email, electronic diary]), and asked about symptoms of COVID-19.								
Testing for laboratory confirmation of SARS-CoV-2 infection	Subjects who report any symptoms associated with COVID-19 after the start of the active and passive surveillance will be requested to visit the Investigator site to provide nasal or NP swabs (2 per subject per event) to test for COVID-19. If the subject cannot visit the Investigator site, then the subject may receive a visit at home to collect the nasal/NP swabs samples or be provided with self-administered nasal/NP swabs for sample collection. The swabs will be collected within 72 hours (preferably within 48 hours) of the reporting of a COVID-19-associated symptom. If swabs cannot be collected within 72 hours after reporting of the COVID-19-associated symptom, nasal or NP swabs should be collected at the next available opportunity for the subject to visit the Investigator site.								

This document is confidential.

**Visit Type**	**Screening/Vaccination**	**Post-vaccination Visits/Contacts**							
**Study Day**	**Day 0**	**Day 1 (+ 1)**	**Day 8 (± 1)**	**Day 21 (± 2)** [8]	**Day 22 (+ 1)**	**Day 29 (± 1)**	**Day 42 (± 2)**	**Day 201 (± 14)**	**Day 386 (± 14)**
**Visit Number**	**1**	**Phone**	**Phone**	**2**	**Phone**	**Phone**	**3**	**4**	**5**
AEs, SAEs, MAAEs, and AESIs[7]	At any time during the study period.								
Termination record									X

4. Endpoints

4.1. Efficacy Endpoints

4.1.1. Primary Endpoints

In the Phase 3 portion, the primary efficacy endpoint is:

- First occurrence, in a subject, of laboratory-confirmed (virologic method) symptomatic SARS-CoV-2 infection (≥ 14 days post-last vaccination).

4.1.2. Secondary Endpoints

In the Phase 2 portion, the secondary efficacy endpoints are:

- First occurrence, in a subject, of laboratory-confirmed (virologic method) symptomatic SARS-CoV-2 infection (≥ 14 days post-last vaccination);
- Occurrences of severe COVID-19 disease (≥ 14 days post-last vaccination).

In the Phase 3 portion, the secondary efficacy endpoints are:

- The slope estimate of the weighted Schoenfeld residuals against event time for the evaluation of absolute vaccine efficacy determined from the first occurrence, in a subject, of laboratory-confirmed (virologic method) symptomatic SARS-CoV-2 infection (≥ 14 days post-last vaccination);
- Occurrences of laboratory-confirmed (confirmed by the ELISA method for the N protein) asymptomatic SARS-CoV-2 infection at Day 201 and Day 386;
- Occurrences of severe COVID-19 disease (≥ 14 days post-last vaccination).
- Occurrence and intensity of COVID-19-related symptoms up to resolution of the symptoms.

4.1.3. Exploratory Endpoints

In the Phase 2 portion, the exploratory efficacy endpoint is:

- Occurrence and intensity of COVID-19-related symptoms up to resolution of the symptoms.

4.2. Immunogenicity Endpoints

Point estimates and 95 % CI will be calculated for all immunogenicity endpoints and responses for the treatment groups will be compared using descriptive statistics.

4.2.1. Primary Endpoints

In the Phase 2 portion, the primary immunogenicity endpoints are:

- Nab response induced in each Study Population against the SARS-CoV-2 virus on Days 0, 21, and 42 will be analyzed using the following parameter: GMT, SC rate, and GMFR;
- Specific Th1 CMI response induced in each Study Population against the SARS-CoV-2 virus on Days 0, 21, and 42, as measured by IFN-γ ELISpot.

4.2.2. Secondary Endpoints

In the Phase 2 portion, the secondary immunogenicity endpoints are:

- Persistence of the Nab antibody response induced in each Study Population against the SARS-CoV-2 virus on Day 201 and Day 386 will be analyzed using the following parameters: GMT, SC rate, and GMFR;
- Specific antibody response induced in each Study Population against the SARS-CoV-2 virus will be measured by the total IgG levels on Days 0, 21, and 42, and the persistence of these antibodies on Days 201 and 386 and analyzed using the following parameters: GMT, SC rate, and GMFR;
- The ratio of neutralizing antibody titers:IgG ELISA antibody titers at Day 21, Day 42, Day 201, and Day 386;
- Specific Th1 CMI response induced in each Study Population against the SARS-CoV-2 virus on Day 201 and Day 386, as measured by IFN-γ ELISpot;
- Specific Th2 CMI response induced in each Study Population against the SARS-CoV-2 virus on Days 0, 21, 42, 201, and 386, as measured by IL-4 (ELISpot).

In the Phase 3 portion, the secondary immunogenicity endpoints are:

- In a subset of subjects, Nab antibody response induced in each Study Population against the SARS-CoV-2 virus on Days 0, 21, 42, 201, and 386 analyzed, using the following parameters: GMT, SC rate, and GMFR;
- In a subset of subjects, specific antibody response induced in each Study Population against the SARS-CoV-2 virus measured by the total IgG levels on Days 0, 21, 42, 201, and 386, and analyzed using the following parameters: GMT, SC rate, and GMFR;

- In a subset of subjects, the ratio of neutralizing antibody titers:IgG ELISA antibody titers at Day 21, Day 42, Day 201, and Day 386;
- In a subset of subjects, specific Th1 CMI response induced in each Study Population against the SARS-CoV-2 virus on Days 0, 21, 42, 201, and 386, as measured by IFN-γ ELISpot;
- In a subset of subjects, specific Th2 CMI response induced in each Study Population against the SARS-CoV-2 virus on Days 0, 21, 42, 201, and 386, as measured by IL-4 (ELISpot);
- In a subset of subjects, specific CMI response induced in each Study Population against the SARS-CoV-2 virus up to 21 days after the last vaccination and at Day 201 and Day 386, as measured by the percentage of CD4+ T cells expressing functional markers.

4.2.3. Exploratory Endpoints

In the Phase 2 and Phase 3 portions, the exploratory immunogenicity endpoint is:

- Further characterization of the immune response of the CoVLP formulation, if it is likely to be informative.

4.3. Safety Endpoints

4.3.1. Primary Endpoints

In the Phase 2 portion, the primary safety endpoints are:

- Occurrence, intensity, and relationship to vaccination of immediate AEs (30 minutes after each vaccination);
- Occurrence, intensity, and relationship to vaccination of solicited local and systemic AEs (for seven days following each vaccine administration);
- Occurrence, intensity, and relationship of unsolicited AEs for 21 days following each vaccine administration;
- Number and percentage of subjects with normal and abnormal clinically significant urine, haematological and biochemical values prior to and three days following each vaccination;
- Occurrences of SAEs, MAAEs, AEs leading to withdrawal, AESIs (including VED), and deaths up to 21 days following each vaccine administration.

4.3.2. Secondary Endpoints

In the Phase 2 portion, the secondary safety endpoints are:

- Occurrences of SAEs, MAAEs, AEs leading to withdrawal, AESIs (including VED), and deaths from Day 43 to Day 201;
- Occurrences of SAEs, MAAEs, AEs leading to withdrawal, AESIs (including VED), and deaths from Day 202 to Day 386.

In the Phase 3 portion, the secondary safety endpoints are:

- Occurrence, intensity, and relationship to vaccination of immediate AEs (30 minutes after each vaccination);
- Occurrence, intensity, and relationship to vaccination of solicited local and systemic AEs (for seven days following each vaccine administration);
- Occurrence, intensity, and relationship of unsolicited AEs for 21 days following each vaccine administration;
- Occurrences of SAEs, MAAEs, AEs leading to withdrawal, AESIs (including VED), and deaths up to 21 days following each vaccine administration;
- Occurrences of SAEs, MAAEs, AEs leading to withdrawal, AESIs (including VED, and deaths from Day 43 to Day 201;
- Occurrences of SAEs, MAAEs, AEs leading to withdrawal, AESIs (including VED), and deaths from Day 202 to Day 386.

4.3.3. Exploratory Endpoints

In the Phase 2 and Phase 3 portions, the exploratory safety endpoint is:

- Further characterization of the safety profile of the CoVLP formulation, if it is likely to be informative.

This document is confidential.

5. Analysis Sets

5.1. Safety Analysis Set

The SAS is defined as all subjects who received at least one dose of either the CoVLP formulation or placebo. All safety analyses will be performed using the SAS and according to actual vaccination received. More particularly, safety data for subjects will be analyzed after each vaccination (Day 0-Day 21 period) according to the last vaccine they actually received, and after any vaccination (Day 0-Day 21 after last vaccination period/Day 0-End of study period) according to the actual vaccine received at the first dose.

5.2. Intention-To-Treat Set

The ITT set will consist of all subjects who were randomized in the study. Subjects who received the wrong treatment will be analyzed as randomized.

5.3. Per Protocol Set

The PP set will consist of a subset of the ITT set who participated in the study until the end of the surveillance period in the Phase 2 or Phase 3 portions of the study; had no major protocol deviations related to subject eligibility, the ability to develop a valid immune response, prohibited medication use, or the efficacy analyses; and who received either the CoVLP formulation or placebo. Major protocol deviations will be identified and documented during a blinded data review prior to database lock and confirmed at the time of database lock. The PP set will be the primary analysis population for the efficacy endpoints. Subjects who received the wrong treatment, but for whom the treatment received can be unequivocally confirmed, will be analyzed as treated, provided they have no other deviations that compromise their data.

The analyses of all efficacy endpoints will be performed using the PP set and the ITT set.

5.4. Immunogenicity Per Protocol Set

The IPP set will consist of a subset of the ITT set who participated in the immunogenicity portion of the Phase 2 or Phase 3 portions of the study and completed the study with no major deviations related to subject eligibility, the ability to develop a valid immune response, prohibited medication use, or the immunogenicity analyses; and who received either the CoVLP formulation or placebo. For the Day 21 analysis, this should include the subjects who received the first vaccine dose and had Day 0 and Day 21 immunogenicity sample collections. For the Day 42 analysis, this should include the subjects who received both vaccine doses and had Day 0 and Day 42 immunogenicity sample collections. For the Day 201 analysis, this should include

This document is confidential.

the subjects who received both vaccine doses and had Day 0 and Day 201 immunogenicity sample collections. For the Day 386 analysis, this should include the subjects who received both vaccine doses and had Day 0 and Day 386 immunogenicity sample collections. Subjects who had blood samples for immunogenicity taken outside of the time window for blood sample collection are to be excluded from the IPP set for the specific visit. Subjects who received the wrong treatment at the first vaccination, but for whom the treatment received can be unequivocally confirmed, will be analyzed as treated, provided they have no other deviations that compromise their data. Subjects who receive a treatment at the second vaccination that is not the same treatment received at the first vaccination will not be included in the IPP set at Day 42, Day 201, and Day 386; the subjects will remain in the Day 21 analysis and analyzed as treated.

The analyses of all immunogenicity endpoints will be performed using the IPP set, as the primary analysis population, and the immunogenicity ITT set, as a secondary analysis population.

6. General Aspects for Statistical Analysis

6.1. General Methods

- All statistical analyses will be conducted with the SAS® software package version 9.4 or higher.
- Unless otherwise specified, summaries will be presented for each treatment and overall
- Continuous variables will be summarized using the number of observations (n), mean, standard deviation (SD), median, minimum, and maximum. Categorical variables will be summarized using number of observations (n), frequency and percentages of subjects
- All relevant subject data will be included in listings. All subjects entered into the database will be included in subject data listings
- Unless otherwise specified, confidence intervals (CI) will be based on two-sided 95% confidence.
- If multiple assessments occur at a given post-baseline time point, the latest value within that time point will be used.

6.1.1. Geometric Mean Titer (GMT)

Continuous immunogenicity endpoints will be logarithmically transformed with 10 as base for analysis. GMT point estimates and the corresponding two-sided 95% CI by treatment group will be calculated as the antilog of the mean and 95% CI of log transformed titer values for Day 0, Day 21, Day 42, Day 201, and Day 386.

6.1.2. Geometric Mean Fold Rise (GMFR)

GMFR will be defined as the geometric mean of the ratio of GMTs (Day 21/Day 0,Day 42/Day 0, Day 201/Day 0 and Day 386/Day 0). GMFR will be derived by using ANCOVA to model the difference in the log of the titer values between Day 21/42/201/386, with treatment group as main effect and log-transformed baseline titer as covariate.

6.1.3. Seroconversion (SC) Rate

The point estimates and the corresponding two-sided 95% CI for subjects achieving SC by treatment group will be calculated and reported. Fisher's exact tests will be used to compare between the treatment groups.

6.2. Key Definitions

6.2.1. Baseline

Unless otherwise specified, Baseline is defined as the last available value prior to the first administration of COVID-19 vaccine (or placebo) and includes assessments taken on Day 0.

6.2.2. Change from Baseline

The change from baseline will be calculated for each post-baseline assessment as:

Change from Baseline = Post-baseline value – Baseline value

6.2.3. Onset Day

The Onset Day is defined as the day of start and calculated as day of vaccination + 1.

6.3. Missing Data

6.3.1. Safety Data

Missing date imputation will be applied to concomitant medications and adverse events. There is no imputation for completely missing dates.

Missing or partial concomitant medication start date:

- If only DAY is missing, use the first day of the month.
- If DAY and Month are both missing, use the first day of the year.

Missing or partial concomitant medication stop date:

- If only DAY is missing, use the last day of the month.
- If DAY and Month are both missing, use the last day of the year.
- If DAY, Month and year are all missing, assign 'continuing' status to stop date.

Missing or partial AE date:
No imputation of AE partial/missing start/end dates or times will be performed. In case of missing or incomplete AE onset date/time, the AE will be included in the analysis unless the incomplete AE onset date/time information unequivocally indicates that the AE started prior to Day 0 (see below for further details).

All analyses described in this section will be based on AE with an onset date on or after the date and time of the first vaccination (Day 0) if not otherwise specified. In case of missing or

This document is confidential.

incomplete AE onset date/time, the AE will be included in the analysis unless the incomplete AE onset date/time information unequivocally indicates that the AE started prior to Day 0, e.g. if a subject is vaccinated on the 15JUL2020 and the AE onset date is XXJUN2020, then the AE will not be included in the analysis. If the AE onset date is JUL2020, then the AE will be included in the analysis.

Adverse events analyses will be presented for the following reporting period, as applicable:

- Day 0-21 period after vaccination 1: Any AEs meeting the following criteria will be allocated to the first vaccination (vaccination 1) and included in the Day 0-21 analysis for first vaccination:
    - Any AEs with:
        - onset date/time < date and time of second vaccine administration, if second vaccination is done,
        - else if vaccination 2 not performed, any AEs with onset date ≤ date of vaccination 1+ 21
- Day 0-21 period after vaccination 2: Any AEs meeting the following criteria will be allocated to second vaccination (vaccination 2) and included in the Day 0-21 analysis for second vaccination:
    - Any AEs with onset date/time ≥ Date/time of vaccination 2 and with onset date ≤ date of vaccination 2 + 21.
- Day 0-21 period after last vaccination: Any AEs meeting the following criteria will be included in the Day 0-21 period after last vaccination:
    - Any AEs with onset date ≤ date of last vaccination + 21 i.e.:
        - Any AEs with onset date ≤ date of vaccination 2 + 21, if vaccination 2 is performed
        - Any AEs with onset date ≤ date of vaccination 1 + 21, if vaccination 2 is not performed
- Day 0 to Day 201: Any AEs with onset date ≤ Day 201 visit date (or vaccination 1 + 201 if Day 201 visit not performed).
- Day 0 to End of Study (EOS): Any AEs recorded in the database
- Day 22 after last vaccination to Day 201: Any AEs recorded in the database at time of analysis and meeting the following criteria will be included in the Day 22 after last vaccination – Day 201 analysis:
    - Any AEs with date of last vaccination + 21 < onset date ≤ Day 201 visit date (or date of vaccination 1 + 201 if Day 201 visit is missing) i.e.:

  - Any AEs with date of vaccination 2 + 21 < onset date ≤ Day 201 visit date (or date of vaccination 1 + 201 if Day 201 visit is missing), if vaccination 2 is performed
  - Any AEs with date of vaccination 1 + 21 < onset date ≤ Day 201 visit date (or date of vaccination 1 + 201 if Day 201 visit is missing), if vaccination 2 is not performed

- Day 201 to EOS: Any AEs recorded in the database at time of analysis with onset date > Day 201 visit date (or date of vaccination 1 + 201 if Day 201 visit is missing)

Note: If an AE allocation to a reporting cannot be unequivocally ascertained due to partial/missing date, the AE will be allocated to all relevant period by. E.g. if a subject is vaccinated on the 01AUG2020 and then on the 23AUG2020 and has an AE with onset date on XXAUG2020, the AE will be allocated to Day 0-21 period after vaccination 1 and Day 0-21 period after vaccination 2.

6.3.2. Immunogenicity

Missing immunogenicity data will be retained as missing. Antibody titers and concentrations below the cutoff will be set to 0.5*cutoff before performing GMT calculations.

6.4. Visit Windows

There will be no derivation for visit windows in terms of summary of assessments. Nominal visits will be used for listings and tables.

6.5. Pooling of Centers

Data from all sites will be summarized together for analyses.

6.6. Subgroups

The following subgroup analyses will be performed for identified safety data:

- Gender (male and female);
- Race (Caucasian or White; Black or African American; Asian).

Identified safety data include:

- Immediate solicited and unsolicited AEs up to 30 minutes after each vaccination;
- Solicited local and systemic AEs within first seven days after each vaccination;
- Most frequent unsolicited AEs within 21 days after vaccination;
- AESIs;
- SAEs;
- MAAEs;
- AEs leading to death;
- AEs leading to withdrawal.

7. Demographic, Other Baseline Characteristics and Medication

7.1. Subject Disposition and Withdrawals

Subject disposition will be presented for all the subjects, which will include the following:

- The number of subjects enrolled
- The number of subjects resulting in screen failure
- The number of subjects randomized

Among the randomized subjects, the following will be summarized by treatment groups:

- The number (%) of subjects in the Safety Analysis Set
- The number (%) of subjects in the Intention-To-Treat Set
- The number (%) of subjects in the Per Protocol Set
- The number (%) of subjects in the Immunogenicity Per Protocol Set (Day 0, Day 21, Day 42, Day 201, and Day 386)
- The number (%) of subjects who completed the study (Day 21, Day 42, Day 201 and Day 386)
- The number (%) of subjects who prematurely discontinued from the study at Day 21, Day 42 and Day 386 and the associated reasons

All protocol deviations will also be listed.

7.2. Demographic and Baseline Characteristics

Demographic data will be presented in listings and summarized by treatment. Continuous variables including age, weight, height, and BMI will be summarized with mean, median, standard deviation, minimum, and maximum. Count and percentage of subjects will be presented for categorical variables such as sex at birth, race, ethnicity, and baseline SARS-CoV-2 testing results. Summary tables will be provided by study population and by age strata within each population when applicable.

Age at Study day 1 = Age at time of consent

Height (in cm) = height (in inches) * 2.54

Weight (in kg) = weight (in lbs) * 0.4536

BMI (kg/m^2) = Weight(kg)/[Height$(m)^2$]

7.3. Medical History and Concomitant Diseases

A summary table of the number and percentage of subjects by medical history using system organ class (SOC) will be produced for patients in the Safety Analysis Set. Medical history will be sorted by serial code for international SOC sort order using the Medical Dictionary for Regulatory Activities (MedDRA) coding dictionary, version 23.1 or higher. Within SOC, medial history will be sorted by preferred term in descending order of total number. For the summary tables, a subject may appear more than once if he/she has more than one medical history coded under different SOC categories. However, the subject will be counted only once in the overall category. Medical History will be summarized by treatment group. A separate by-subject listing for medical history data will also be provided.

7.4. Medication

Prior and Concomitant medications will be summarized by ATC level 2 and preferred drug name for the Safety Analysis Set by treatment group.

7.4.1. Prior Medication

Prior medication is any medication that was started prior to study vaccine date and ended before study vaccine date.

7.4.2. Concomitant Medication

Concomitant medication is a medication that starts on or after vaccination date/time at Day 0 or start before vaccination and was still on-going at the time of the study vaccine.

Concomitant medications must be reported (reason for use, dates of administration, dosage, and route) if the use meets the following conditions:

- Within 30 days preceding vaccination: any treatments and/or medications specifically contraindicated (e.g. vaccines, any immunoglobulins or other blood products, or any immune modifying drugs, etc.);

This document is confidential.

- From randomization to Day 42, inclusive: any medication (including, but not limited to, over-the-counter medicines such as aspirin or antacids), vitamins, and mineral supplements;
- From Day 43 to the end of the study, inclusive: any concomitant medication(s) administered to treat an AESI, MAAE, SAE, or AE leading to withdrawal; any concomitant medication used to treat an AE that occurred before Day 42 and that is still being used afterwards (i.e. on-going use);
- Any concomitant medication used to treat conditions reported as medical history;
- Any investigational medication or vaccine; any vaccine not foreseen in the study protocol.

7.5. Treatment Compliance

Study treatments will be prepared and administered by trained unblinded site staff via IM injection to each subject at each Investigator site. In addition, the Investigator or designated study center personnel will maintain a log of all study treatments dispensed and administered to the subject. The number, percentage and the reason of subjects refusal of vaccination post-randomization will be summarized.

8. Efficacy

Efficacy summary tables will present data for the overall population and for each age stratum according to each Study Population, as applicable. Summary tables will include subjects in the PP set and ITT set.

8.1. Primary Efficacy Endpoint(s) and Analysis

In the Phase 3 portion:

The primary efficacy endpoint for Phase 3 portion is the first occurrence, in a subject, of laboratory-confirmed (virologic method) symptomatic SARS-CoV-2 infection (≥ 14 days post-last vaccination).

For the Phase 3 portion of the study, once approximately 160 COVID-19 cases have accumulated during the study, the primary efficacy endpoint of the Phase 3 portion of the study will be evaluated to determine if the vaccine efficacy (VE) success criterion has been met. For the efficacy endpoints, absolute VE will be evaluated.

Absolute VE will be evaluated as the relative risk of CoVLP formulation vaccinated versus unvaccinated subjects to develop laboratory-confirmed symptomatic SARS-CoV-2 infection ≥ 14 days after the last vaccination, as per the following formula:

Absolute VE=(1-RR)=(1-ARV/ARU)*100 %

Where:

RR= relative risk

ARV = attack rate in subjects vaccinated with the CoVLP formulation; and

ARU = attack rate in unvaccinated subjects.

The absolute VE success criterion for the primary efficacy endpoint, in the Phase 3 portion, is defined as a ≥ 50 % point estimate and a > 30 % lower limit of the appropriately alpha-adjusted confidence interval that maintains overall Type I error at 5%. The alpha-adjusted CI around RR will be calculated based on score confidence limits [Miettinen 1985, Farrington 1990].

Attack rate in subjects vaccinated with the CoVLP formulation and attack rate in unvaccinated subjects will be summarized. Absolute VE will also be summarized along with the alpha-

This document is confidential.

adjusted CI of RR. An interim analysis for an early efficacy assessment will be performed when 80 laboratory-confirmed symptomatic COVID-19 cases have been collected in the Phase 3 portion and a statistical correction for the interim analysis will be applied that uses the O'Brien-Fleming alpha-spending function. With interim and final assessments at 80 and 160 cases, the two-sided test p-value thresholds will be 0.0027 and 0.0478, respectively. This means that the alpha-adjusted CI will be 99.73% at the interim and 95.22% at the primary analysis.

8.2. Secondary Efficacy Endpoint(s) and Analyses

Summary tables will be provided using PP set and ITT set.

In the Phase 2 portion:

8.2.1. First occurrence of laboratory-confirmed (virologic method) symptomatic SARS-CoV-2 infection (≥ 14 days post-last vaccination) in a subject

The absolute VE will be evaluated and this endpoint will be calculated and summarized using the same method described for the primary efficacy endpoint (Section 8.1), except for 95% CI will be used for VE in the Phase 2 portion.

8.2.2. Occurrences of severe COVID-19 disease (≥ 14 days post-last vaccination)

The same formula mentioned in Section 8.1 will be used to evaluate the relative risk of CoVLP formulation of vaccinated subjects versus unvaccinated subjects who develop severe COVID-19 disease ≥ 14 days after the last vaccination. 95% CI will be used for VE in the Phase 2 portion.

In the Phase 3 portion:

8.2.3. The slope estimate of the weighted Schoenfeld residuals against event time for the evaluation of absolute vaccine efficacy determined from the first occurrence of laboratory-confirmed (virologic method) symptomatic SARS-CoV-2 infection (≥ 14 days post-last vaccination) in a subject

The presence of absolute VE waning will be tested by the linear trend (i.e., the proportional hazards) in the scaled Schoenfeld residuals from the Cox proportional hazard model. Specifically, it will be analyzed as below:

Step 1: Fit a regular Cox proportional hazard model with the treatment group as the main effect, and placebo will be coded as the reference group in the model;

Step 2: Find the scaled Schoenfeld residual from the Cox proportional hazard model in step 1. The scaled Schoenfeld residual has value at all event-time for the main effect (treatment group);

Step 3: Fit a linear regression on the scaled Schoenfeld residuals against event time;

Step 4: The slope and p-value will be output. VE(t)=1-exp(beta(t)), VE(t) waning (smaller with time) is corresponding to the increasing beta(t). Thus, a positive slope with significant p-value (< 0.05) will indicate VE is waning though time.

8.2.4. Occurrences of laboratory-confirmed (confirmed by the ELISA method for the N protein) asymptomatic SARS-CoV-2 infection at Day 201 and Day 386

The absolute VE will be evaluated and this endpoint will be calculated and summarized using the same method described for the primary efficacy endpoint (Section 8.1).

8.2.5. Occurrences of severe COVID-19 disease (≥ 14 days post-last vaccination)

The same formula mentioned in Section 8.1 will be used to evaluate the relative risk of CoVLP formulation of vaccinated subjects versus unvaccinated subjects who develop severe COVID-19 disease ≥ 14 days after the last vaccination.

8.2.6. Occurrence and intensity of COVID-19-related symptoms up to resolution of the symptoms

The occurrences of COVID-19-related symptoms and their severity will be summarized by treatment using descriptive statistics. The occurrence of unusual manifestations of COVID-19 as well as the severity of COVID-19 symptoms will also be used to assess imbalance between the CoVLP and placebo groups as a possible indication of enhanced disease (full details provided in the Medical Monitoring Plan and IDMC Charter).

8.3. Exploratory Efficacy Endpoint(s) and Analyses

In the Phase 2 portion:

8.3.1. Occurrence and intensity of COVID-19-related symptoms up to resolution of the symptoms

The occurrences of COVID-19-related symptoms and their severity will be summarized by treatment using descriptive statistics. The occurrence of unusual manifestations of COVID-19 as well as the severity of COVID-19 symptoms will also be used to assess imbalance between the

This document is confidential.

CoVLP and placebo groups as a possible indication of enhanced disease (full details provided in the Medical Monitoring Plan and IDMC Charter).

9. Immunogenicity

Immunogenicity summary tables will present data for:

- All subjects according to Study Population;
- Subjects who are seropositive at baseline for SARS-CoV-2 antibodies according to Study Population;
- Subjects who are seronegative at baseline for SARS-CoV-2 antibodies according to Study Population;
- Each of the different age strata according to Study Population, as applicable.

Point estimates and 95% CI will be calculated for all immunogenicity endpoints and responses for the treatment groups will be compared using descriptive statistics.

9.1. Primary Immunogenicity Endpoint(s) and Analysis

In the Phase 2 portion:

9.1.1. Nab response induced in each Study Population against the SARS-CoV-2 virus on Days 0, 21, and 42 using GMT, SC rate, and GMFR

The following analyses for the NAb assay will be performed on the Immunofenicity Per Protocol (IPP) set:

- GMT (Day 0, Day 21, and Day 42): The point estimates and the corresponding two-sided 95 % CI by treatment group will be calculated as the antilog of the mean and 95 % CI of log transformed titer values;
- SC rate (Day 0, Day 21, and Day 42): The point estimates and the corresponding two-sided 95 % CI for subjects achieving SC by treatment group will be calculated and reported;
- GMFR: the geometric mean of the ratio of GMTs (Day 21/Day 0 and Day 42/Day 0).

GMT will be compared between treatment groups using the analysis of variance (ANOVA) model. GMFR will be compared using the ANCOVA model. For SC rate, Fisher's exact tests will be used to compare between the treatment groups, along with exact Clopper-Pearson 95% CI. Pairwise comparisons for SC rate will be performed without any adjustment method. The GMFR will be derived by using ANCOVA to model the difference in the log of the titer values between Day 21 and Day 0 and between Day 42 and Day 0, with treatment group as main effect and log-transformed baseline titer as covariate.

9.1.2. Specific Th1 CMI response induced in each Study Population against the SARS-CoV-2 virus on Days 0, 21, and 42 measured by IFN-γ ELISpot

The specific Th1 CMI response induced on Day 0, Day 21, and Day 42 will be measured by the number of T cells expressing IFN-γ, using ELISpot, for each treatment group. The response will be compared between treatment groups and timepoints using appropriate non-parametric (Wilcoxon) models. The Wilcoxon Rank Sum Test, also known as the Mann Whitney Wilcoxon Test, will be used to estimate the difference in response between treatment groups, along with the corresponding 95% CI for the median and p-value. The test statistic for the Mann Whitney U Test is denoted U and is the smaller of U1 and U2, defined below.

$$U_1 = n_1 n_2 + \frac{n_1(n_1+1)}{2} - R_1$$

$$U_2 = n_1 n_2 + \frac{n_2(n_2+1)}{2} - R_2$$

where R1 = sum of the ranks for group 1, R2 = sum of the ranks for group 2, n1 = sample size for group 1 and n2 = sample size for group 2.

Since the response between timepoints are paired data, the Wilcoxon Signed Rank Test will be used to estimate the difference in response between timepoints, along with the corresponding 95% CI for the median and p-value.

9.2. Secondary Immunogenicity Endpoint(s) and Analysis

In the Phase 2 portion:

9.2.1. Persistence of the Nab antibody response induced in each Study Population against the SARS-CoV-2 virus on Day 201 and Day 386 using GMT, SC rate, and GMFR

The following analyses for the NAb assay will be performed on the Immunogenicity Per Protocol (IPP) set:

- GMT (Days 201 and 386): The point estimates and the corresponding two-sided 95 % CI by treatment group will be calculated as the antilog of the mean and 95 % CI of log transformed titer values;

- SC rate (Days 201 and 386): The point estimates and the corresponding two-sided 95 % CI for subjects achieving SC by treatment group will be calculated and reported;
- GMFR: the geometric mean of the ratio of GMTs (Day 201/Day 0 and Day 386/Day 0).

GMT will be compared between treatment groups using the analysis of variance (ANOVA) model. GMFR will be compared using the ANCOVA model. For SC rate, Fisher's exact tests will be used to compare between the treatment groups, along with exact Clopper-Pearson 95% CI . The GMFR will be derived by using ANCOVA to model the difference in the log of the titer values between Day 21 and Day 0, between Day 42 and Day 0, Day 201 and Day 0, and Day 386 and Day 0 with treatment group as main effect and baseline titer as covariate.

9.2.2. Specific antibody response induced in each Study Population against the SARS-CoV-2 virus by the total IgG levels on Days 0, 21, and 42, and the persistence of these antibodies on Days 201 and 386 using GMT, SC rate, and GMFR

The GMT and SC rate will be calculated on Day 0, Day 21, Day 42, Day 201, and Day 386 as well as the GMFR (Day 21/Day 0, Day 42/Day 0, Day 201/Day 0, and Day 386/Day 0).

GMT will be compared between treatment groups using the analysis of variance (ANOVA) model. GMFR will be compared using the ANCOVA model. For SC rate, Fisher's exact tests will be used to compare between the treatment groups. The GMFR will be derived by using ANCOVA to model the difference in the log of the titer values between Day 21 and Day 0, between Day 42 and Day 0, Day 201 and Day 0, and Day 386 and Day 0 with treatment group as main effect and log-transformed baseline titer as covariate.

9.2.3. The ratio of neutralizing antibody titers:IgG ELISA antibody titers at Day 21, Day 42, Day 201, and Day 386

The Nab/IgG ratio will be calculated as the antilog of the Nab/IgG ratio and 95 % CI of log transformed titer values. The ratio will be compared between treatment groups using ANOVA.

9.2.4. Specific Th1 CMI response induced in each Study Population against the SARS-CoV-2 virus on Day 201 and Day 386 measured by IFN-γ ELISpot

The specific Th1 CMI response induced on Day 201 and Day 386 will be measured by the number of T cells expressing IFN-γ, using ELISpot, for each treatment group. The response will be compared between treatment groups and timepoints using appropriate non-parametric (Wilcoxon) models. The Wilcoxon Rank Sum Test will be used to estimate the difference in response between treatment groups, along with the corresponding 95% CI for the median and p-

value. The Wilcoxon Signed Rank Test will be used to estimate the difference in response between timepoints, along with the corresponding 95% CI for the median and p-value.

9.2.5. Specific Th2 CMI response induced in each Study Population against the SARS-CoV-2 virus on Days 0, 21, 42, 201, and 386 measured by IL-4 (ELISpot)

The specific Th2 CMI response induced on Day 201 and Day 386 will be measured by the number of T cells expressing IL-4, using ELISpot, for each treatment group. The response will be compared between treatment groups and timepoints using appropriate non-parametric (Wilcoxon) models. The Wilcoxon Rank Sum Test will be used to estimate the difference in response between treatment groups, along with the corresponding 95% CI for the median and p-value. The Wilcoxon Signed Rank Test will be used to estimate the difference in response between timepoints, along with the corresponding 95% CI for the median and p-value.

In the Phase 3 portion:

9.2.6. Nab antibody response induced in each Study Population against the SARS-CoV-2 virus on Days 0, 21, 42, 201, and 386 using GMT, SC rate, and GMFR

The same immunogenicity analyses will be performed as described in Section 9.2.1.

9.2.7. Specific antibody response induced in each Study Population against the SARS-CoV-2 virus by the total IgG levels on Days 0, 21, 42, 201, and 386 using GMT, SC rate, and GMFR

The same immunogenicity analyses will be performed as described in Section 9.2.2.

9.2.8. The ratio of neutralizing antibody titers:IgG ELISA antibody titers at Day 21, Day 42, Day 201, and Day 386

The same immunogenicity analyses will be performed as described in Section 9.2.3.

9.2.9. Specific Th1 CMI response induced in each Study Population against the SARS-CoV-2 virus on Days 0, 21, 42, 201, and 386 measured by IFN-γ ELISpot

The same immunogenicity analyses will be performed as described in Section 9.2.4.

9.2.10. Specific Th2 CMI response induced in each Study Population against the SARS-CoV-2 virus on Days 0, 21, 42, 201, and 386 measured by IL-4 (ELISpot)

The same immunogenicity analyses will be performed as described in Section 9.2.5.

9.2.11. Specific CMI response induced in each Study Population against the SARS-CoV-2 virus up to 21 days after the last vaccination and at Day 21, Day 42, Day 201 and Day 386 measured by the percentage of CD4+ T cells expressing functional markers

The CMI response induced on Day 0, Day 21, Day 42, Day 201, and Day 386 will be measured by the percentage of T cells expressing functional markers, using flow cytometry, for each treatment group. The responses will be compared between treatment groups and timepoints using appropriate non-parametric (Wilcoxon) models. The Wilcoxon Rank Sum Test will be used to estimate the difference in response between treatment groups, along with the corresponding 95% CI for the median and p-value. The Wilcoxon Signed Rank Test will be used to estimate the difference in response between timepoints, along with the corresponding 95% CI for the median and p-value.

This analysis could be repeated for all available data under different stimulating conditions.

9.3. Exploratory Immunogenicity Endpoint(s) and Analysis

In the Phase 2 and Phase 3 portion:

9.3.1. Further characterization of the immune response of the CoVLP formulation

If deemed necessary, further characterization of the immune response of the CoVLP formulation will be done and the results will be presented.

This document is confidential.

10. Safety

Safety and tolerability will be evaluated by solicited local and systemic adverse events (immediate solicited adverse events within 30 minutes post-vaccination and solicited adverse events up to seven days after each vaccination), unsolicited AEs within 30 minutes post-vaccination and up to 21 days after each vaccination, SAEs, AESIs, MAAEs, and AEs leading to withdrawal up to the end of the study. In addition, events will be monitored for possible VED, hypersensitivity components, and potential immune-mediated diseases, from all reported events during the study (collected AEs, SAEs, AESIs, MAAEs, and AEs leading to withdrawal). Clinical safety methods will include repeated urinalysis, blood chemistry, and haematology testing.

Safety monitoring of safety signals will be performed for each of the Study Populations throughout the Phase 2 and Phase 3 portions of the study. Stopping rules or conditions for stopping each portion of the study would occur if there was clear evidence of harm or harmful effects such as SAEs related to the treatment (study vaccine). An SAE which is assessed as unrelated to the study vaccine would not warrant stopping the trial.

For sentinel subjects in Study Populations #2, #3, and #4 during the Phase 2 portion of the study:

If any of the following events occur to the sentinel subjects, the study enrollment and vaccinations may be paused based on IDMC recommendations:

- Any SAE in a subject for which causality cannot be attributed to another cause;
- Two (2) or more subjects who receive the CoVLP formulation experience a similar AE (i.e. the same Preferred Terms based on MedDRA] coding):
  - Experience the same Grade 3 or higher solicited local AE or systemic AE within 7 days after administration of the vaccine;
  - Experience the same Grade 3 AE or higher unsolicited AE, vital sign, and/or clinical laboratory abnormality, in the same Preferred terms based on the MedDRA coding, and is considered related to the vaccine.

After vaccination of all sentinel subjects in Phase 2, the stopping rules for the remainder of the Phase 2 and the Phase 3 portions of the study:

The study may be paused based on IDMC recommendations if any of the following events occur:

- Any subject experiences an SAE after administration of the vaccine that is considered related to vaccine;

- If 5 % or more subjects who receive the CoVLP formulation experience a similar AE (i.e. the same Preferred Terms based on MedDRA coding):
    - Experience the same Grade 3 or higher solicited local AE or systemic AE, within 7 days after administration of the vaccine;
    - Experience the same Grade 3 AE or higher unsolicited AE, vital sign, and/or clinical laboratory abnormality, in the same Preferred terms based on the MedDRA coding, after administration of the vaccine and is considered related to the vaccine;

If an important imbalance in unusual manifestations of COVID-19 or severity of COVID-19 symptoms is observed between the CoVLP and placebo groups (full details provided in the Medical Monitoring Plan and IDMC Charter).Safety summary tables will present data for the overall population and for each age stratum according to each Study Population, as applicable.

10.1. Extent of Exposure

Treatment exposure will be summarized using the number of vaccination for each subjects.

10.2. Adverse Events

An AE or adverse experience is defined as any untoward medical occurrence in a subject or clinical investigation subject who was administered a pharmaceutical product, with or without a causal relationship with the treatment. Information such as the date and time of onset and resolution (duration), intensity (defined below), seriousness, any required treatment or action taken, outcome, relationship to the investigational vaccine, and whether the AE caused withdrawal from the study will be collected.

The intensity of all AEs will be graded as mild (1), moderate (2) severe (3), or potentially life threatening (4). If a subject has the same AE but with different grades over time, the highest grade will be reported. The causal relationship with the study vaccine of the solicited local and systemic and unsolicited AEs will be assessed by the Investigator, and will be classified as "definitely not related", "probably not related", "possibly related", "probably related", or "definitely related". If there is any valid reason, even if undetermined or untested, for suspecting a possible cause-and-effect relationship between the investigational vaccine and the occurrence of the AE, then the AE should be considered "definitely related", "probably related", or "possibly related". Otherwise, if no valid reason exists for suggesting a possible relationship, then the AE should be classified as "probably not related" or "definitely not related". AE outcomes will be classified as recovered/resolved, not recovered/not resolved, recovered/resolved with sequelae, recovering/resolving, or death.

10.2.1. Solicited Local and Systemic Adverse Events

Subjects will be monitored for both solicited local AEs (erythema, swelling, and pain at the injection site) and solicited systemic AEs (fever, headache, fatigue, muscle aches, joint aches, chills, a feeling of general discomfort, swelling in the axilla, and swelling in the neck) from the time of each vaccination through seven days. While the subjects remain in the clinic following vaccine administration (at least 30 minutes), staff will monitor them for solicited local and systemic AEs; after release from the clinic facility, from the evening of each vaccination to the evening of the seventh day after each vaccination, the subject (and/or their parent/LAR) will measure and record the subject's local and systemic AEs in the diary. Solicited local and systemic AEs will be summarized through primary and secondary safety endpoints.

10.2.2. Unsolicited Adverse Events

All spontaneous unsolicited AEs occurring on vaccination or within 21 days after each vaccination will be reported and classified by system organ class (SOC) and preferred term (PT). Frequency count and the number of unique subjects with a AE will be tabulated by treatment(s) received. For the number of unique subjects reporting, if a subject reported more than one AE that was coded to the same SOC or PT, the subject will be counted only once for that specific SOC or PT.

10.2.3. Serious Adverse Events (SAE)

An SAE is any untoward medical occurrence (whether or not considered to be related to the study vaccine) that, at any dose:

- Results in death;
- Is life-threatening (at the time of the event);
- Requires inpatient hospitalization (≥ 24 hours) or prolongation of existing hospitalization (elective hospitalizations/procedures for pre-existing conditions that have not worsened are excluded);
- Results in persistent or significant disability/incapacity;
- Is a congenital abnormality/birth defect;
- Is another medically important event.

10.2.4. Medically Attended Adverse Events (MAAE)

Medically attended adverse events are defined as symptoms or illnesses requiring hospitalization, or emergency room visit, or visit to/by a health care provider.

This document is confidential.

10.2.5. Adverse Events of Special Interest (AESI)

10.2.5.1. Vaccine Enhanced Disease (VED)

Safety signal of VED after exposure to the CoVLP formulation will be closely monitored and assessed by retrieving data for this AESI as follows: AEs within the system organ class (SOC): immune system disorders and high level group term (HLGT): lower respiratory tract disorders (excluding obstruction and infection), cardiac disorders, signs and symptoms not elsewhere classified, vascular disorders, heart failures, arteriosclerosis, stenosis, vascular insufficiency and necrosis, cardiac arrhythmias, myocardial disorders, and vascular hemorrhagic disorders. High level term (HLT): renal failure and impairment and preferred term (PT): pericarditis, coagulopathy, deep vein thrombosis, pulmonary embolism, cerebrovascular accidents, peripheral ischemia, liver injury, Guillain-Barre syndrome, anosmia, ageusia, encephalitis, chilblains, vasculitis, erythema multiforme that require inpatient hospitalization (≥ 24 hours) and have laboratory confirmed SARS-Cov-2 infection will be monitored for assessment of any potential case of VED. As this list can be updated during the study, any further changes to the AESI terms will be updated in the Signal Management Plan (SMP).

10.2.5.2. Hypersensitivity Reactions

All reported events will also be monitored for related hypersensitivity reactions after exposure to the CoVLP formulation. Hypersensitivity will be identified uing SMQ broad and narrow terms.

10.2.5.3. Potential Immune-Mediated Diseases (pIMD)

Potential immune-mediated diseases (pIMDs) are a subset of AEs associated to the adjuvant that include autoimmune diseases and other inflammatory and/or neurologic disorders of interest which may or may not have an autoimmune aetiology. pIMDs will be identified using SMQ terms (Appendix 1).

10.3. Laboratory Evaluations

Blood samples for biochemistry and hematology, HIV, Hepatitis B and Hepatitis C markers in serum and urine samples for urinalysis will be collected according to the Time and Events Schedule (see Table 2) for the Phase 2 portion of the study. Blood samples for testing for SARS-CoV-2 antibodies will be collected according to the Time and Events Schedules (see Table 3) for the Phase 2 and Phase 3 portions of the study. In addition, serum or urine samples from all females of childbearing potential in Study Populations #1, #2, #4, and #5 for pregnancy testing will be collected. Any laboratory result outside of the testing laboratory's normal range will be classified as 'clinically significant' (CS) or 'not clinically significant' (NCS) by the site

This document is confidential.

Investigator, with appropriate documentation. Any laboratory test performed at Screening with a value outside the normal range may be repeated (prior to vaccination) at the discretion of the Investigator. Repeated tests will be considered the Baseline results. Baseline for Day 24 assessment will be Day 21, as subject will receive a second vaccination at Day 21.

The tests to be performed by the laboratory are presented in Table 5.

Table 5 Clinical Laboratory Tests for the Phase 2 Portion of the Study

**Biochemistry (serum):**	
Sodium<br>Potassium<br>Urea	Alkaline phosphatase<br>Alaninetransferase (ALT)<br>Aspartatetransferase (AST)
Creatinine<br>Glucose<br>Bilirubin (total)<br>Albumin<br>Total protein<br>Phosphorus	Gamma glutamyltransferase (GGT)<br>Cholesterol (total, HDL, LDL)<br>Triglyceride<br>Chloride<br>Calcium
**Haematology:**	
Haemoglobin<br>Hematocrit<br>Red blood cells<br>Platelets<br>Mean platelet volume (MPV)<br>White cell count (total, WBC)<br>Neutrophils	Mean cell haemoglobin (MCH)<br>Mean cell concentration (MCHC)<br>Mean cell volume (MCV)<br>Lymphocytes<br>Monocytes<br>Eosinophils<br>Basophils
**Serology:**	
HIV<br>Hepatitis C	Hepatitis B
**Urinalysis:**	
Macroscopic examination (color, aspect)<br>pH<br>Specific gravity	Glucose<br>Protein<br>Blood

Other clinical laboratory tests include a test to measure SARS-CoV-2 antibodies at screening for all subjects in all Study Populations in the Phase 2 portion and at Days 0, 201, and 386 in the Phase 3 portion, and a serum (Phase 2 portion) or urine (Phase 3 portion) pregnancy test at screening and a urine (dipstick or similar) pregnancy test prior to randomization on Day 0 and on Days 21 and 42 for all female subjects of childbearing potential in Study Populations #1, #2, #4, and #5.

Laboratory results will be displayed using the conventional units for all summaries and listings. Clinical laboratory test results (hematology and chemistry) and their changes from baseline will be summarized by visit for the safety analysis set using descriptive statistics.

10.4. Vital Signs

Vital signs measurements (resting BP, HR, RR, and OT) will be performed as part of screening procedures, prior to vaccination (on Day 0 and Day 21), and after the post-vaccination 30-minute surveillance period (and repeated if deemed necessary by the Investigator) during the Phase 2 portion. In addition, vital signs measurements will be performed at the clinic site visits on Day 3, Day 24, Day 42, Day 201, and Day 386 for the Phase 2 portion of the study. Vital signs measurements will only be performed prior to each vaccination during the Phase 3 portion. Baseline for Day 24 assessment will be Day 21, as subject will receive a second vaccination at Day 21.

A summary table will include the subjects in the safety analysis set with descriptive statistics for baseline, actual value and change from baseline by treatment group and overall.

All vital signs will be listed in by-subject listings and will be sorted by treatment group, subject ID, and date/time assessment.

10.5. Primary Safety Endpoint(s) and Analysis

All safety analyses will be performed using the Safety Analysis set and according to actual vaccination received.

In the Phase 2 portion:

10.5.1. Occurrence, intensity, and relationship to vaccination of immediate AEs (30 minutes after each vaccination)

Occurrence, intensity, and relationship to vaccination of immediate AEs (30 minutes after each vaccination) will be summarized by treatment using descriptive statistics.

10.5.2. Occurrence, intensity, and relationship to vaccination of solicited local and systemic AEs (for seven days following each vaccine administration)

Occurrence, intensity, and relationship to vaccination of solicited local and systemic AEs reported up to seven days after each vaccination will be summarized by treatment using descriptive statistics.

10.5.3. Occurrence, intensity, and relationship of unsolicited AEs for 21 days following each vaccine administration

Occurrence, intensity, and relationship to vaccination of unsolicited AEs will be summarized by treatment using descriptive statistics.

10.5.4. Number and percentage of subjects with normal and abnormal clinically significant urine, haematological and biochemical values prior to and three days following each vaccination

Laboratory (biochemical, haematological, and urine) data will be summarized by type of laboratory test. Reference ranges and markedly abnormal results will be used in the summary of laboratory data. Descriptive statistics will be calculated for each laboratory test at baseline and at each scheduled timepoint (Day 3, Day 21, and Day 24). Changes from Baseline results will be presented in pre- versus post-treatment cross tabulations (with classes for below, within, and above normal ranges).

10.5.5. Occurrences of SAEs, MAAEs, AEs leading to withdrawal, AESIs (including VED), and deaths up to 21 days following each vaccine administration

Occurrences of SAEs, AEs leading to subject withdrawal, AESIs, MAAEs, and deaths reported up to 21 days after each vaccination will be summarized by treatment using descriptive statistics.

10.6. Secondary Safety Endpoint(s) and Analysis

For the following categories, AE information will be presented by gender (male and female) and race (Caucasian or White; Black or African American; Asian):

- Immediate solicited and unsolicited AEs up to 30 minutes after each vaccination;
- Solicited local and systemic AEs within first seven days after each vaccination;
- Most frequent unsolicited AEs within 21 days after vaccination;
- AESIs;
- SAEs;
- MAAEs;
- AEs leading to death;
- AEs leading to withdrawal.

In the Phase 2 portion:

10.6.1. Occurrences of SAEs, MAAEs, AEs leading to withdrawal, AESIs (including VED), and deaths from Day 43 to Day 201

Occurrences of SAEs, MAAEs, AEs leading to subject withdrawal, AESIs, and deaths reported from Day 43 to the Day 201 will be summarized by treatment using descriptive statistics.

10.6.2. Occurrences of SAEs, MAAEs, AEs leading to withdrawal, AESIs (including VED), and deaths from Day 202 to Day 386

Occurrences of SAEs, MAAEs, AEs leading to subject withdrawal, AESIs, and deaths reported from Day 202 to the Day 386 will be summarized by treatment using descriptive statistics.

In the Phase 3 portion:

10.6.3. Occurrence, intensity, and relationship to vaccination of immediate AEs (30 minutes after each vaccination)

The same safety analyses will be performed as described in Section 10.5.1.

10.6.4. Occurrence, intensity, and relationship to vaccination of solicited local and systemic AEs (for seven days following each vaccine administration)

The same safety analyses will be performed as described in Section 10.5.2.

10.6.5. Occurrence, intensity, and relationship of unsolicited AEs for 21 days following each vaccine administration

The same safety analyses will be performed as described in Section 10.5.3.

10.6.6. Occurrences of SAEs, MAAEs, AEs leading to withdrawal, AESIs (including VED), and deaths up to 21 days following each vaccine administration

The same safety analyses will be performed as described in Section 10.5.5.

10.6.7. Occurrences of SAEs, MAAEs, AEs leading to withdrawal, AESIs (including VED, and deaths from Day 43 to Day 201

The same safety analyses will be performed as described in Section 10.6.1.

10.6.8. Occurrences of SAEs, MAAEs, AEs leading to withdrawal, AESIs (including VED), and deaths from Day 202 to Day 386

The same safety analyses will be performed as described in Section 10.6.2.

10.7. Exploratory Safety Endpoint(s) and Analysis

In the Phase 2 and Phase 3 portion:

10.7.1. Further characterization of the safety profile of the CoVLP formulation

If deemed necessary, further characterization of the safety profile of the CoVLP formulation will be done and the results will be presented.

10.8. Pregnancy

Pregnancy information will be provided in a listing.

10.9. Physical Examination

A history- or symptom-directed physical examination will be performed by the Investigator (or sub-Investigator) as part of screening procedures.

A summary tables will include the subjects in the safety analysis set with number and percentage of each physical examination finding.

The physical examination data will be listed with findings.

11. Interim Analyses

In an attempt to identify an adverse imbalance of COVID-19 and/or severe COVID-19 cases between the vaccine and placebo recipients, which is indicative of possible vaccine-enhanced disease (VED), an unblinded team will review COVID-19 and/or severe COVID-19 cases on a ongoing basis to determine if the probability of observing the same or a more extreme case split is 5% or less when the true incidence of severe disease is the same for vaccine and placebo recipients. If this criterion is met, then the unblinded team will advise the IDMC members who will review the information along with other available information and determine if the study should continue, be modified, be halted or be stopped. The monitoring of potential imbalance of severe COVID-19 cases will start once 8 cases have been accumulated. The assessment will be performed using a one-sided binomial test, which will provide probabilities of having an adverse imbalance between the vaccine and placebo arms given an expected ratio of 4:1 in phase 2 and a ratio of 1:1 in phase 3. In addition, an alert rule will be put in place and will be triggered when the probability of having imbalance in severe cases in the vaccine arm versus placebo arm is less than 15%.

The start of the Phase 3 portion of the study will be determined independently for each Study Population and will be based on the outcomes of the following two assessments during the Phase 2 portion of the study:

- Day 21 Data Analysis of the immunogenicity data;
- Day 28 Safety Review.

Study Population #1: If the safety and immunogenicity profiles of the chosen CoVLP formulation observed in the Phase 1 study are acceptable in the Phase 2 portion, this group will proceed into the Phase 3 portion. Another key outcome will be the absence of a significant safety signal as assessed by the IDMC.

Study Population #2: If the safety and immunogenicity profiles of the CoVLP formulation in this population are acceptable, this group will also proceed into the Phase 3 portion. A key outcome will be the absence of a significant safety signal as assessed by the IDMC. As noted above, any important safety signal in this Study Population may trigger one or more mitigation strategies prior to entry into the Phase 3 portion and the younger and older cohorts within this Study Population (5-9 and 10-17 year old) may enter into the Phase 3 portion.

Study Populations #3 and #4: If the safety and immunogenicity profiles of the CoVLP formulation in this population are acceptable, entry of these two Study Populations into the Phase 3 portion will be based on both the absence of an important safety signal and demonstration of an acceptable immune response as assessed by the IDMC. The criteria for acceptability will be based on NAb and IFN-γ ELISpot results after either the first or second immunizations. If the 1st dose immunogenicity criteria (below) are not met at Day 21, the 2nd dose criteria (below) will be applied. This two-step approach is justified by the observation of a strong correlation between the responses elicited by the first and second doses of the CoVLP formulation. Because cellular immune responses are thought to be important for protection against highly pathogenic coronaviruses [Channappanavar 2014] including SARS-COV-2 [Sette 2020] and because cellular responses to some viral pathogens are better maintained than antibody responses in older subjects [McElhaney 2013a] the criteria below incorporate both Nab (A) and IFN-γ ELISpot (B) results in a cumulative A + B formula to determine acceptability. Our Phase 1 data further support this approach since occasional individuals were identified who made little-to-no antibody response but mounted IFN-γ ELISpot responses.

First Dose Criteria

Proportion of subjects with NtAb GMT ≥ 2-fold increase over baseline will be considered responders = A

Proportion of subjects with ≥ 2-fold increase over baseline in IFN-γ ELISpot will be considered responders = B

Acceptable to advance to Phase 3 portion if A + B ≥ 50% responders.

Second Dose Criteria

Proportion of subjects with NtAb GMT ≥ 4-fold increase over baseline = A

Proportion of subjects who with ≥ 2-fold increase over baseline in IFN-γ ELISpot = B

Acceptable to advance to Phase 3 portion if A + B ≥ 60% of the responders.

For Phase 3, an interim analysis for an early efficacy assessment will be performed when 80 laboratory-confirmed symptomatic COVID-19 cases have been collected in the Phase 3 portion. When 80 laboratory-confirmed COVID-19 cases have been collected, the unblinded statistical team will perform a first interim analysis for the Phase 3 portion of the efficacy data in order to

test for early vaccine efficacy. If the primary efficacy success criterion is met during this interim analysis, enrollment of subjects into the placebo treatment group will stop; subject enrollment into the CoVLP formulation treatment group will continue for the collection of safety data and for a descriptive assessment of the efficacy and immunogenicity endpoints. The IDMC will also monitor the study for non-efficacy at the interim analysis once 80 cases will be collected. The boundary for non-efficacy is non-binding and defined as the upper limit of the 95% CI for VE being < 20%. A second interim analysis for the Phase 3 portion will be performed as soon as 160 cases are collected in order to perform the primary analysis and produce primary efficacy outputs.

12. Changes from Analysis Planned in Protocol

NA

13. Reference List

Protocol: A Randomized, Observer-Blind, Placebo-Controlled, Phase 2/3 Study to Assess the Safety, Efficacy, and Immunogenicity of a Recombinant Coronavirus-Like Particle COVID-19 Vaccine in Adults 18 Years of Age or Older and Children and Adolescents 5-17 Years of Age. CP-PRO-CoVLP-021, Final Version 2.0.

Miettinen OS, Nurminen MM. "Comparative Analysis of Two Rates." Statistics in Medicine. 1985;4:213–226.

Farrington CP, Manning G. "Test Statistics and Sample Size Formulae for Comparative Binomial Trials with Null Hypothesis of Non-zero Risk Difference or Non-unity Relative Risk." Statistics in Medicine. 1990;9:1447–1454.

Channappanavar R, Zhao J, Perlman S. T cell-mediated immune response to respiratory coronaviruses. Immunol Res. 2014 Aug;59(1-3):118-28. doi: 10.1007/s12026-014-8534-z.

Sette A, Crotty S. Pre-existing immunity to SARS-CoV-2: the knowns and unknowns. Nat Rev Immunol. 2020 Aug;20(8):457-458.

McElhaney JE, Coler RN, Baldwin SL. Immunologic correlates of protection and potential role for adjuvants to improve influenza vaccines in older adults. Expert Rev Vaccines. 2013a Jul;12(7):759-66.

This document is confidential.

14. Programming Considerations

14.1. General Considerations

- One SAS program can create several outputs, or a separate SAS program will be created for each output
- One output file can contain several outputs.or each output will be stored in a separate file
- Output files will be delivered in Word format or portable document format pdf
- Numbering of TFLs will follow ICH E3 guidance

14.2. Table, Figure, and Listing Format

14.2.1. General

- All TFLs will be produced in landscape format on American letter size, unless otherwise specified
- All TFLs will be produced using the Courier New font, size 8 which is the smallest acceptable point size for the Regulatory Authorities
- The data displays for TFLs will have a minimum blank 1-inch margin on all 4 sides
- Headers and footers for figures will be in Courier New font, size 8 which is the smallest acceptable point size for the Regulatory Authorities
- Legends will be used for all figures with more than one variable, group, or item displayed
- TFLs will be in black and white (no color), unless otherwise specified
- Specialized text styles, such as bolding, italics, borders, shading, and superscripted and subscripted text, will not be used in the TFLs, unless otherwise specified. On some occasions, superscripts 1, 2, or 3 may be used (see below)
- Only standard keyboard characters will be used in the TFLs. Special characters, such as non-printable control characters, printer-specific, or font-specific characters, will not be used. Hexadecimal-derived characters will be used, where possible, if they are appropriate to help display math symbols (e.g., µ). Certain subscripts and superscripts (e.g., cm2, Cmax) will be employed on a case-by-case basis

- Mixed case will be used for all titles, footnotes, column headers, and programmer-supplied formats, as appropriate.

14.2.2. Headers

- All output will have the following header at the top left of each page:
- Medicago Protocol CP-PRO-CoVLP-021 (Syneos Health study number [Redacted])
- Draft/Final Run <*date*>
- All output will have Page n of N at the top or bottom right corner of each page. TFLs are internally paginated in relation to the total length (i.e., the page number will appear sequentially as page n of N, where N is the total number of pages in the table)
- The date the output was generated will appear along with the program name as a footer on each page

14.2.3. Display Titles

- Each TFL will be identified by the designation and a numeral. (i.e., Table 14.1.1). A decimal system (x.y and x.y.z) are used to identify TFLs with related contents. The title will be centered. The analysis set will be identified on the line immediately following the title and will be enclosed in parenthesis. The title and table designation will be single spaced. A solid line spanning the margins will separate the display titles from the Column headers. There will be one blank line between the last title and the solid line.

Table x.y.z
First Line of Title
Second Line of Title if Needed
(ITT Analysis Set)

14.2.4. Column Headers

- Column headings will be displayed immediately below the solid line described above in initial upper-case characters
- In the case of efficacy tables, the variable (or characteristic) column is on the far left followed by the treatment group columns and total column (if applicable). P-values may be

presented under the total column or in separate p-value column (if applicable). Within-treatment comparisons may have p-values presented in a row beneath the summary statistics for that treatment

- For numeric variables, include ‘unit’ in column or row heading when appropriate
- Analysis set sizes will be presented for each treatment group in the column heading as (N=xx) (or in the row headings, if applicable). This is distinct from the ‘n’ used for the descriptive statistics representing the number of subjects in the analysis set
- The order of treatments in the tables and listings will be Placebo first in the case of placebo controlled studies, followed by a total column

14.2.5. Body of the Data Display

14.2.5.1. *General Conventions*

Data in columns of a table or listing are formatted as follows:

- Alphanumeric values will be left-justified;
- Whole numbers (e.g., counts) will be right-justified; and
- Numbers containing fractional portions will be decimal aligned

14.2.5.2. *Table Conventions*

- Units will be included where available
- For categorical parameters,all categories will be presented in the table, even if n=0 for all treatment groups in a given category. For example, the frequency distribution for symptom severity would appear as:

Severity Rating	N
severe	0
moderate	8
mild	3

Where percentages are presented in these tables, zero percentages will not be presented and so counts of 0 will be presented as 0 and not as 0 (0%).

- An Unknown or Missing category will be added to each parameter for which information is not available for 1 or more subjects
- Unless otherwise specified, the estimated mean and median for a set of values will be printed out to 1 more significant digit than the original values, and standard deviations will be printed out to 2 more significant digits than the original values. The minimum and maximum will report the same significant digits as the original values. For example, systolic blood pressure will be presented as follows:

N	XX
Mean	XXX.X
Std Dev	X.XX
Median	XXX.X
Minimum	XXX
Maximum	XXX

- P-values will be output in the format: '0.xxx', where xxx is the value rounded to 3 decimal places. Every p-value less than 0.001 will be presented as <0.001. If the p-value are less than 0.0001, then present as <0.0001. If the p-value is returned as >0.999, then present as >0.999
- Percentage values will be printed to one decimal place, in parentheses with no spaces, one space after the count (e.g., 7 (12.8%), 13 (5.4%)). Unless otherwise noted, for all percentages, the number of subjects in the analysis set for the treatment group who have an observation will be the denominator. Percentages after zero counts will not be displayed and percentages equating to 100% will be presented as 100%, without decimal places. If percentages are smaller than 0.1%, then it will be specified xx (<0.1)
- Tabular display of data for medical history, prior/concomitant medications, and all tabular displays of adverse event data will be presented by the body system, treatment class, or SOC, sorted by serial code for international SOC sort order, with the highest occurrence in the active treatment group in decreasing order, assuming all terms are coded. Within the body system, drug class and SOC, medical history (by preferred term), drugs (by ATC1 code), and adverse events (by preferred term) will be displayed in decreasing order. If incidence for more than 1 term is identical, they will then be sorted alphabetically.

- The percentage of subjects will normally be calculated as a proportion of the number of subjects assessed in the relevant treatment group (or overall) for the analysis set presented. However, careful consideration is required in many instances due to the complicated nature of selecting the denominator, usually the appropriate number of subjects exposed. Details will be described in footnotes or programming notes, as necessary

- For categorical summaries (number and percentage of subjects) where a subject can be included in more than one category, a footnote or programming note will be added describing whether the subject is included in the summary statistics for all relevant categories or just 1 category as well as the selection criteria

- Where a category with a subheading (such as system organ class) has to be split over more than one page, output the subheading followed by '(cont)' at the top of each subsequent page. The overall summary statistics for the subheading should only be output on the first relevant page

14.2.5.3. *Listing Conventions*

- Listings will be sorted for presentation in order of treatment groups as above, subject number, visit/collection day, and visit/collection time

- Missing data will be represented on subject listings as either a hyphen ('-') with a corresponding footnote ('- = unknown or not evaluated'), or as 'N/A', with the footnote 'N/A = not applicable', whichever is appropriate

- Dates will be printed in SAS DATE9.format ('DD_MMM_YYYY': 01JUL2000). Missing portions of dates will be represented on subject listings as dashes (--JUL2000). Dates that are missing because they are not applicable for the subject will be output as 'N/A', unless otherwise specified

- All observed time values will be presented using a 24-hour clock HH:MM or HH:MM:SS format (e.g., 11:26:45, or 11:26). Time will only be reported if it was measured as part of the study

- Units will be included where available

This document is confidential.

14.2.5.4. *Figure Conventions*

- Unless otherwise specified, for all figures, study visits will be displayed on the X-axis and endpoint (e.g., treatment mean change from Baseline) values will be displayed on the Y-axis

14.2.6. Footnotes

- A solid line spanning the margins will separate the body of the data display from the footnotes.
- All footnotes will be left justified with single-line spacing immediately below the solid line underneath the data display
- Footnotes will always begin with 'Note:' if an informational footnote, or 1, 2, 3, etc. if a reference footnote. Each new footnote will start on a new line, where possible
- Subject specific footnotes are avoided, where possible
- Footnotes will be used sparingly and add value to the TFL. If more than six lines of footnotes are planned, then a cover page is strongly recommended to be used to display footnotes, and only those essential to comprehension of the data will be repeated on each page
- The last line of the footnote section will be a standard source line that indicates the name of the program used to produce the data display, the date the program was run, and the listing source (i.e., 'Program : myprogram.sas Listing source: 16.x.y.z')
- Sources and/or cross-references in footnotes will use the keyword prefix (in singular form) for each reference and will be separated by a comma when mutiple cross-references are displayed
  <u>Example</u>
  Listing source: Listing 16.2.4.1.1, Listing 16.2.4.1.2, Listing 16.2.4.2.1

This document is confidential.

15. Quality Control

SAS programs are developed to produce output such as analysis data sets, summary tables, data listings, figures or statistical analyses. An overview of the development of programs is detailed in Syneos Health Developing Statistical Programs [Redacted].

Syneos Health Developing Statistical Programs [Redacted], Conducting the Transfer of Biostatistical Deliverables [Redacted] and the SAS Programming and Vaidation Plan describes the quality control procedures that are performed for all SAS programs and output. Quality control is defined here as the operational techniques and activities undertaken to verify that the SAS programs produce the output by checking for their logic, efficiency and commenting and by review of the produced output.

16. Index of Tables

Table Number	Name	Analysis Set
14.1.1.1	Subject Disposition	Randomized Subjects
14.1.2.1	Major Protocol Deviations	SAS
14.1.3.1	Demographic and Baseline Characteristics	ITT Set
14.1.3.2	Demographic and Baseline Characteristics	PP Set
14.1.3.3	Demographic and Baseline Characteristics	SAS
14.1.3.4	Demographic and Baseline Characteristics	IPP Set
14.1.3.3	Medical History	SAS
14.1.4.1	Prior Medications	SAS
14.1.4.2	Concomitant Medications	SAS
14.1.5.1	Extent of Exposure for Phase 2	SAS
14.1.5.2	Extent of Exposure for Phase 3	SAS
14.2.1.1	Sumary of Vaccine Efficacy of Symptomatic SARS-CoV-2 Infection, Severe COVID-19 Disease, and Asymptomatic SARS-CoV-2 Infection for Phase 2	PP Set and ITT Set
14.2.1.2	Sumary of Vaccine Efficacy of Symptomatic SARS-CoV-2 Infection, Severe COVID-19 Disease, and Asymptomatic SARS-CoV-2 Infection for Phase 3	PP Set and ITT Set
14.2.2.1	Slope estimate of the weighted Schoenfeld residuals against event time for the evaluation of absolute vaccine efficacy in a subject for Phase 3	PP Set and ITT Set
14.2.2.2	Occurrence and intensity of COVID-19-related symptoms up to resolution of the symptoms for Phase 3	PP Set and ITT Set
14.2.3.1	Occurrence and intensity of COVID-19-related symptoms up to resolution of the symptoms for Phase 2	PP Set and ITT Set
14.2.4.1	Nab response induced in each Study Population against the SARS-CoV-2 virus on Days 0, 21, 42, 201 and 386 using GMT, SC rate, and GMFR for Phase 2	IPP Set and Immunogenicity ITT Set

Table Number	Name	Analysis Set
14.2.4.2	Specific Th1 CMI response induced in each Study Population against the SARS-CoV-2 virus on Days 0, 21, 42, 201 and 386 measured by IFN-γ ELISpot for Phase 2	IPP Set and Immunogenicity ITT Set
14.2.5.1	Specific antibody response induced in each Study Population against the SARS-CoV-2 virus by the total IgG levels on Days 0, 21, and 42, and the persistence of these antibodies on Days 201 and 386 using GMT, SC rate, and GMFR for Phase 2	IPP Set and Immunogenicity ITT Set
14.2.5.2	The ratio of neutralizing antibody titers:IgG ELISA antibody titers at Day 21, Day 42, Day 201, and Day 386 for Phase 2	IPP Set and Immunogenicity ITT Set
14.2.5.3	Specific Th2 CMI response induced in each Study Population against the SARS-CoV-2 virus on Days 0, 21, 42, 201, and 386 measured by IL-4 (ELISpot) for Phase 2	IPP Set and Immunogenicity ITT Set
14.2.5.4	Nab antibody response induced in each Study Population against the SARS-CoV-2 virus on Days 0, 21, 42, 201, and 386 using GMT, SC rate, and GMFR for Phase 3	IPP Set and Immunogenicity ITT Set
14.2.5.5	Specific antibody response induced in each Study Population against the SARS-CoV-2 virus by the total IgG levels on Days 0, 21, 42, 201, and 386 using GMT, SC rate, and GMFR for Phase 3	IPP Set and Immunogenicity ITT Set
14.2.5.6	The ratio of neutralizing antibody titers:IgG ELISA antibody titers at Day 21, Day 42, Day 201, and Day 386 for Phase 3	IPP Set and Immunogenicity ITT Set
14.2.5.7	Specific Th1 CMI response induced in each Study Population against the SARS-CoV-2 virus on Days 0, 21, 42, 201, and 386 measured by IFN-γ ELISpot for Phase 3	IPP Set and Immunogenicity ITT Set

Table Number	Name	Analysis Set
14.2.5.8	Specific Th2 CMI response induced in each Study Population against the SARS-CoV-2 virus on Days 0, 21, 42, 201, and 386 measured by IL-4 (ELISpot) for Phase 3	IPP Set and Immunogenicity ITT Set
14.2.5.9	Specific CMI response induced in each Study Population against the SARS-CoV-2 virus up to 21 days after the last vaccination and at Day 201 and Day 386 measured by the percentage of CD4+ T cells expressing functional markers for Phase 3	IPP Set and Immunogenicity ITT Set
14.3.1.1.1	Adverse Events – Overall Summary for Phase 2	SAS
14.3.1.1.2	Adverse Events – Overall Summary for Phase 3	SAS
14.3.1.2.1	Adverse Events by System Organ Class and Preferred Term for Phase 2	SAS
14.3.1.2.2	Adverse Events by System Organ Class and Preferred Term for Phase 3	SAS
14.3.1.3.1	Summary of Occurrence of Immediate Adverse Events for Phase 2	SAS
14.3.1.3.1.1	Summary of Occurrence of Immediate Adverse Events for Phase 2 by Gender	SAS
14.3.1.3.1.2	Summary of Occurrence of Immediate Adverse Events for Phase 2 by Race	SAS
14.3.1.3.2	Summary of Intensity of Immediate Adverse Events for Phase 2	SAS
14.3.1.3.3	Summary of Related Cases of Adverse Events by Intensity for Phase 2	SAS
14.3.1.4.1	Summary of Occurrence of Solicited Local and Systemic Adverse Events for Phase 2	SAS
14.3.1.4.1.1	Summary of Occurrence of Solicited Local and Systemic Adverse Events for Phase 2 by Gender	SAS

This document is confidential.

Table Number	Name	Analysis Set
14.3.1.4.1.2	Summary of Occurrence of Solicited Local and Systemic Adverse Events for Phase 2 by Race	SAS
14.3.1.4.2	Summary of Intensity of Solicited Local and Systemic Adverse Events for Phase 2	SAS
14.3.1.4.3	Summary of Related Cases of Solicited Local and Systemic Adverse Events by Intensity for Phase 2	SAS
14.3.1.5.1	Summary of Occurrence of Unsolicited Adverse Events for Phase 2	SAS
14.3.1.5.1.1	Summary of Occurrence of Unsolicited Adverse Events for Phase 2 by Gender	SAS
14.3.1.5.1.2	Summary of Occurrence of Unsolicited Adverse Events for Phase 2 by Race	SAS
14.3.1.5.2	Summary of Intensity of Unsolicited Adverse Events for Phase 2	SAS
14.3.1.5.3	Summary of Related Cases of Adverse Events by Intensity for Phase 2	SAS
14.3.1.6.1	Summary of Occurrences of Serious Adverse Events up to 21 Days Following each Vaccine Administration for Phase 2	SAS
14.3.1.6.1.1	Summary of Occurrences of Serious Adverse Events up to 21 Days Following each Vaccine Administration for Phase 2 by Gender	SAS
14.3.1.6.1.2	Summary of Occurrences of Serious Adverse Events up to 21 Days Following each Vaccine Administration for Phase 2 by Race	SAS
14.3.1.6.2	Summary of Occurrences of Medically Attended Adverse Events up to 21 Days Following each Vaccine Administration for Phase 2	SAS
14.3.1.6.2.1	Summary of Occurrences of Medically Attended Adverse Events up to 21 Days Following each Vaccine Administration for Phase 2 by Gender	SAS

This document is confidential.

Table Number	Name	Analysis Set
14.3.1.6.2.2	Summary of Occurrences of Medically Attended Adverse Events up to 21 Days Following each Vaccine Administration for Phase 2 by Race	SAS
14.3.1.6.3	Summary of Occurrences of Adverse Events Leading to Withdrawl up to 21 Days Following each Vaccine Administration for Phase 2	SAS
14.3.1.6.3.1	Summary of Occurrences of Adverse Events Leading to Withdrawl up to 21 Days Following each Vaccine Administration for Phase 2 by Gender	SAS
14.3.1.6.3.2	Summary of Occurrences of Adverse Events Leading to Withdrawl up to 21 Days Following each Vaccine Administration for Phase 2 by Race	SAS
14.3.1.6.4	Summary of Occurrences of Adverse Events of Special Interest (Including VED) up to 21 Days Following each Vaccine Administration for Phase 2	SAS
14.3.1.6.4.1	Summary of Occurrences of Adverse Events of Special Interest (Including VED) up to 21 Days Following each Vaccine Administration for Phase 2 by Gender	SAS
14.3.1.6.4.2	Summary of Occurrences of Adverse Events of Special Interest (Including VED) up to 21 Days Following each Vaccine Administration for Phase 2 by Race	SAS
14.3.1.6.5	Summary of Occurrences of Deaths up to 21 Days Following each Vaccine Administration for Phase 2	SAS
14.3.1.6.5.1	Summary of Occurrences of Deaths up to 21 Days Following each Vaccine Administration for Phase 2 by Gender	SAS

This document is confidential.

Table Number	Name	Analysis Set
14.3.1.6.5.2	Summary of Occurrences of Deaths up to 21 Days Following each Vaccine Administration for Phase 2 by Race	SAS
14.3.1.7.1	Occurrences of Serious Adverse Events from Day 43 to Day 201 for Phase 2	SAS
14.3.1.7.1.1	Occurrences of Serious Adverse Events from Day 43 to Day 201 for Phase 2 by Gender	SAS
14.3.1.7.1.2	Occurrences of Serious Adverse Events from Day 43 to Day 201 for Phase 2 by Race	SAS
14.3.1.7.2	Summary of Occurrences of Medically Attended Adverse Events from Day 43 to Day 201 for Phase 2	SAS
14.3.1.7.2.1	Summary of Occurrences of Medically Attended Adverse Events from Day 43 to Day 201 for Phase 2 by Gender	SAS
14.3.1.7.2.2	Summary of Occurrences of Medically Attended Adverse Events from Day 43 to Day 201 for Phase 2 by Race	SAS
14.3.1.7.3	Summary of Occurrences of Adverse Events Leading to Withdrawl from Day 43 to Day 201 for Phase 2	SAS
14.3.1.7.3.1	Summary of Occurrences of Adverse Events Leading to Withdrawl from Day 43 to Day 201 for Phase 2 by Gender	SAS
14.3.1.7.3.2	Summary of Occurrences of Adverse Events Leading to Withdrawl from Day 43 to Day 201 for Phase 2 by Race	SAS
14.3.1.7.4	Summary of Occurrences of Adverse Events of Special Interest (Including VED) from Day 43 to Day 201 for Phase 2	SAS
14.3.1.7.4.1	Summary of Occurrences of Adverse Events of Special Interest (Including VED) from Day 43 to Day 201 for Phase 2 by Gender	SAS

This document is confidential.

Table Number	Name	Analysis Set
14.3.1.7.4.2	Summary of Occurrences of Adverse Events of Special Interest (Including VED) from Day 43 to Day 201 for Phase 2 by Race	SAS
14.3.1.7.5	Summary of Occurrences of Deaths from Day 43 to Day 201 for Phase 2	SAS
14.3.1.7.5.1	Summary of Occurrences of Deaths from Day 43 to Day 201 for Phase 2 by Gender	SAS
14.3.1.7.5.2	Summary of Occurrences of Deaths from Day 43 to Day 201 for Phase 2 by Race	SAS
14.3.1.8.1	Summary of Occurrences of Serious Adverse Events from Day 202 to Day 386 for Phase 2	SAS
14.3.1.8.1.1	Summary of Occurrences of Serious Adverse Events from Day 202 to Day 386 for Phase 2 by Gender	SAS
14.3.1.8.1.2	Summary of Occurrences of Serious Adverse Events from Day 202 to Day 386 for Phase 2 by Race	SAS
14.3.1.8.2	Summary of Occurrences of Medically Attended Adverse Events from Day 202 to Day 386 for Phase 2	SAS
14.3.1.8.2.1	Summary of Occurrences of Medically Attended Adverse Events from Day 202 to Day 386 for Phase 2 by Gender	SAS
14.3.1.8.2.2	Summary of Occurrences of Medically Attended Adverse Events from Day 202 to Day 386 for Phase 2 by Race	SAS
14.3.1.8.3	Summary of Occurrences of Adverse Events Leading to Withdrawl from Day 202 to Day 386 for Phase 2	SAS
14.3.1.8.3.1	Summary of Occurrences of Adverse Events Leading to Withdrawl from Day 202 to Day 386 for Phase 2 by Gender	SAS
14.3.1.8.3.2	Summary of Occurrences of Adverse Events Leading to Withdrawl from Day 202 to Day 386 for Phase 2 by Race	SAS

This document is confidential.

Table Number	Name	Analysis Set
14.3.1.8.4	Summary of Occurrences of Adverse Events of Special Interest (Including VED) from Day 202 to Day 386 for Phase 2	SAS
14.3.1.8.4.1	Summary of Occurrences of Adverse Events of Special Interest (Including VED) from Day 202 to Day 386 for Phase 2 by Gender	SAS
14.3.1.8.4.2	Summary of Occurrences of Adverse Events of Special Interest (Including VED) from Day 202 to Day 386 for Phase 2 by Race	SAS
14.3.1.8.5	Summary of Occurrences of Deaths from Day 202 to Day 386 for Phase 2	SAS
14.3.1.8.5.1	Summary of Occurrences of Deaths from Day 202 to Day 386 for Phase 2 by Gender	SAS
14.3.1.8.5.2	Summary of Occurrences of Deaths from Day 202 to Day 386 for Phase 2 by Race	SAS
14.3.1.9.1	Summary of Occurrence of Immediate Adverse Events for Phase 3	SAS
14.3.1.9.1.1	Summary of Occurrence of Immediate Adverse Events for Phase 3 by Gender	SAS
14.3.1.9.1.2	Summary of Occurrence of Immediate Adverse Events for Phase 3 by Race	SAS
14.3.1.9.2	Summary of Intensity of Immediate Adverse Events for Phase 3	SAS
14.3.1.9.3	Summary of Related Cases of Adverse Events by Intensity for Phase 3	SAS
14.3.1.10.1	Summary of Occurrence of Solicited Local and Systemic Adverse Events for Phase 3	SAS
14.3.1.10.1.1	Summary of Occurrence of Solicited Local and Systemic Adverse Events for Phase 3 by Gender	SAS
14.3.1.10.1.2	Summary of Occurrence of Solicited Local and Systemic Adverse Events for Phase 3 by Race	SAS
14.3.1.10.2	Summary of Intensity of Solicited Local and Systemic Adverse Events for Phase 3	SAS

Table Number	Name	Analysis Set
14.3.1.10.3	Summary of Related Cases of Solicited Local and Systemic Adverse Events by Intensity for Phase 3	SAS
14.3.1.11.1	Summary of Occurrence of Unsolicited Adverse Events for Phase 3	SAS
14.3.1.11.1.1	Summary of Occurrence of Unsolicited Adverse Events for Phase 3 by Gender	SAS
14.3.1.11.1.2	Summary of Occurrence of Unsolicited Adverse Events for Phase 3 by Race	SAS
14.3.1.11.2	Summary of Intensity of Unsolicited Adverse Events for Phase 3	SAS
14.3.1.11.3	Summary of Related Cases of Adverse Events by Intensity for Phase 3	SAS
14.3.1.12.1	Summary of Occurrences of Serious Adverse Events up to 21 Days Following each Vaccine Administration for Phase 3	SAS
14.3.1.12.1.1	Summary of Occurrences of Serious Adverse Events up to 21 Days Following each Vaccine Administration for Phase 3 by Gender	SAS
14.3.1.12.1.2	Summary of Occurrences of Serious Adverse Events up to 21 Days Following each Vaccine Administration for Phase 3 by Race	SAS
14.3.1.12.2	Summary of Occurrences of Medically Attended Adverse Events up to 21 Days Following each Vaccine Administration for Phase 3	SAS
14.3.1.12.2.1	Summary of Occurrences of Medically Attended Adverse Events up to 21 Days Following each Vaccine Administration for Phase 3 by Gender	SAS
14.3.1.12.2.2	Summary of Occurrences of Medically Attended Adverse Events up to 21 Days Following each Vaccine Administration for Phase 3 by Race	SAS

Table Number	Name	Analysis Set
14.3.1.12.3	Summary of Occurrences of Adverse Events Leading to Withdrawl up to 21 Days Following each Vaccine Administration for Phase 3	SAS
14.3.1.12.3.1	Summary of Occurrences of Adverse Events Leading to Withdrawl up to 21 Days Following each Vaccine Administration for Phase 3 by Gender	SAS
14.3.1.12.3.2	Summary of Occurrences of Adverse Events Leading to Withdrawl up to 21 Days Following each Vaccine Administration for Phase 3 by Race	SAS
14.3.1.12.4	Summary of Occurrences of Adverse Events of Special Interest (Including VED) up to 21 Days Following each Vaccine Administration for Phase 3	SAS
14.3.1.12.4.1	Summary of Occurrences of Adverse Events of Special Interest (Including VED) up to 21 Days Following each Vaccine Administration for Phase 3 by Gender	SAS
14.3.1.12.4.2	Summary of Occurrences of Adverse Events of Special Interest (Including VED) up to 21 Days Following each Vaccine Administration for Phase 3 by Race	SAS
14.3.1.12.5	Summary of Occurrences of Deaths up to 21 Days Following each Vaccine Administration for Phase 3	SAS
14.3.1.12.5.1	Summary of Occurrences of Deaths up to 21 Days Following each Vaccine Administration for Phase 3 by Gender	SAS
14.3.1.12.5.2	Summary of Occurrences of Deaths up to 21 Days Following each Vaccine Administration for Phase 3 by Race	SAS
14.3.1.13.1	Occurrences of Serious Adverse Events from Day 43 to Day 201 for Phase 3	SAS

Table Number	Name	Analysis Set
14.3.1.13.1.1	Occurrences of Serious Adverse Events from Day 43 to Day 201 for Phase 3 by Gender	SAS
14.3.1.13.1.2	Occurrences of Serious Adverse Events from Day 43 to Day 201 for Phase 3 by Race	SAS
14.3.1.13.2	Summary of Occurrences of Medically Attended Adverse Events from Day 43 to Day 201 for Phase 3	SAS
14.3.1.13.2.1	Summary of Occurrences of Medically Attended Adverse Events from Day 43 to Day 201 for Phase 3 by Gender	SAS
14.3.1.13.2.2	Summary of Occurrences of Medically Attended Adverse Events from Day 43 to Day 201 for Phase 3 by Race	SAS
14.3.1.13.3	Summary of Occurrences of Adverse Events Leading to Withdrawl from Day 43 to Day 201 for Phase 3	SAS
14.3.1.13.3.1	Summary of Occurrences of Adverse Events Leading to Withdrawl from Day 43 to Day 201 for Phase 3 by Gender	SAS
14.3.1.13.3.2	Summary of Occurrences of Adverse Events Leading to Withdrawl from Day 43 to Day 201 for Phase 3 by Race	SAS
14.3.1.13.4	Summary of Occurrences of Adverse Events of Special Interest (Including VED) from Day 43 to Day 201 for Phase 3	SAS
14.3.1.13.4.1	Summary of Occurrences of Adverse Events of Special Interest (Including VED) from Day 43 to Day 201 for Phase 3 by Gender	SAS
14.3.1.13.4.2	Summary of Occurrences of Adverse Events of Special Interest (Including VED) from Day 43 to Day 201 for Phase 3 by Race	SAS
14.3.1.13.5	Summary of Occurrences of Deaths from Day 43 to Day 201 for Phase 3	SAS
14.3.1.13.5.1	Summary of Occurrences of Deaths from Day 43 to Day 201 for Phase 3 by Gender	SAS

This document is confidential.

Table Number	Name	Analysis Set
14.3.1.13.5.2	Summary of Occurrences of Deaths from Day 43 to Day 201 for Phase 3 by Race	SAS
14.3.1.14.1	Summary of Occurrences of Serious Adverse Events from Day 202 to Day 386 for Phase 3	SAS
14.3.1.14.1.1	Summary of Occurrences of Serious Adverse Events from Day 202 to Day 386 for Phase 3 by Gender	SAS
14.3.1.14.1.2	Summary of Occurrences of Serious Adverse Events from Day 202 to Day 386 for Phase 3 by Race	SAS
14.3.1.14.2	Summary of Occurrences of Medically Attended Adverse Events from Day 202 to Day 386 for Phase 3	SAS
14.3.1.14.2.1	Summary of Occurrences of Medically Attended Adverse Events from Day 202 to Day 386 for Phase 3 by Gender	SAS
14.3.1.14.2.2	Summary of Occurrences of Medically Attended Adverse Events from Day 202 to Day 386 for Phase 3 by Race	SAS
14.3.1.14.3	Summary of Occurrences of Adverse Events Leading to Withdrawl from Day 202 to Day 386 for Phase 3	SAS
14.3.1.14.3.1	Summary of Occurrences of Adverse Events Leading to Withdrawl from Day 202 to Day 386 for Phase 3 by Gender	SAS
14.3.1.14.3.2	Summary of Occurrences of Adverse Events Leading to Withdrawl from Day 202 to Day 386 for Phase 3 by Race	SAS
14.3.1.14.4	Summary of Occurrences of Adverse Events of Special Interest (Including VED) from Day 202 to Day 386 for Phase 3	SAS
14.3.1.14.4.1	Summary of Occurrences of Adverse Events of Special Interest (Including VED) from Day 202 to Day 386 for Phase 3 by Gender	SAS

Table Number	Name	Analysis Set
14.3.1.14.4.2	Summary of Occurrences of Adverse Events of Special Interest (Including VED) from Day 202 to Day 386 for Phase 3 by Race	SAS
14.3.1.14.5	Summary of Occurrences of Deaths from Day 202 to Day 386 for Phase 3	SAS
14.3.1.14.5.1	Summary of Occurrences of Deaths from Day 202 to Day 386 for Phase 3 by Gender	SAS
14.3.1.14.5.2	Summary of Occurrences of Deaths from Day 202 to Day 386 for Phase 3 by Race	SAS
14.3.4.1.1.1	Hematology: Summary by Visit and Change from Baseline	SAS
14.3.4.1.1.2	Hematology: Proportion of Abnormal Results by Study Visit	SAS
14.3.4.1.2.1	Blood Chemistry: Summary by Visit and Change from Baseline	SAS
14.3.4.1.2.2	Blood Chemistry: Proportion of Abnormal Results by Study Visit	SAS
14.3.4.1.3.1	Urinalysis: Proportion of Abnormal Results by Study Visit	SAS
14.3.4.2.1	Summary of Vital Signs by Visit and Change from Baseline	SAS
14.3.4.3.1	Summary of Physical Examination	SAS

17. Index of Figures

Figure Number	Name	Analysis Set
14.2.4.1.1	Reverse Cumulative Distribution Curves (RCDC) for Specific Antibody Response by Nab	PP Set
14.2.5.1.1	Reverse Cumulative Distribution Curves (RCDC) for Specific Antibody Response by IgG	PP Set
14.2.4.2.1	Boxplot of Th1 CMI Response by Timepont for Phase 2	PP Set
14.2.4.2.2	Boxplot of Th1 CMI Response by Timepont for Phase 3	PP Set
14.2.5.3.1	Boxplot of Th2 CMI Response by Timepont for Phase 2	PP Set
14.2.5.3.2	Boxplot of Th2 CMI Response by Timepont for Phase 3	PP Set
14.2.5.9.1	Boxplot of CMI Response Measured by the Percentage of CD4+T Cells Expressing Functional Markers by Timepont for Phase 3	PP Set

18. Index of Listings

Listing Number	Name	Analysis Set
16.2.1	Subject Disposition	Randomized Subjects
16.2.2	Protocol Deviations	SAS
16.2.3.1	Subject Withdrawl/Discontinued	All Subjects
16.2.3.2	Subjects Not Randomized	All Subjects
16.2.3.3	Screen Failures	All Subjects
16.2.3.3	Exclusions from Analysis Sets	SAS
16.2.4.1	Demographics	SAS
16.2.4.2	Medical History	SAS
16.2.4.3	Baseline Characteristics	SAS
16.2.4.4	Prior and Concomitant Medications	SAS
16.2.5	Extent of Exposure	SAS
16.2.6.1	First occurrence of laboratory-confirmed (virologic method) symptomatic SARS-CoV-2 infection (≥ 14 days post-last vaccination) in a subject	ITT Set
16.2.6.2	Occurrences of severe COVID-19 disease (≥ 14 days post-last vaccination)	ITT Set
16.2.6.3	Occurrences of laboratory-confirmed (confirmed by the ELISA method for the N protein) asymptomatic SARS-CoV-2 infection	ITT Set
16.2.6.4	Occurrence and intensity of COVID-19-related symptoms up to resolution of the symptoms	ITT Set
16.2.6.5	Nab response, specific antibody response by total IgG level and the ratio of Nab:IgG induced in each Study Population against the SARS-CoV-2 virus	ITT Set
16.2.6.6	Specific Th1 CMI response induced in each Study Population against the SARS-CoV-2 virus	ITT Set
16.2.6.7	Specific Th2 CMI response induced in each Study Population against the SARS-CoV-2 virus	ITT Set

This document is confidential.

Listing Number	Name	Analysis Set
16.2.6.8	Specific CMI response measured by the percentage of CD4+ T cells expressing functional markers induced in each Study Population against the SARS-CoV-2 virus	ITT Set
16.2.7.1	Unsolicited Adverse Events	SAS
16.2.7.2	Solicited Adverse Events	SAS
16.2.7.3	Serious Adverse Events	SAS
16.2.7.4	Adverse Events of Special Interest	SAS
16.2.7.5	Death	SAS
16.2.7.6	Adverse Events Leading to Permanent Discontinuation of Treatment	SAS
16.2.8.1.1	Hematology	SAS
16.2.8.1.2	Blood Chemistry	SAS
16.2.8.1.3	Urinalysis	SAS
16.2.8.2.1	Vital Signs	SAS
16.2.8.2.2	Physical Examination	SAS
16.2.8.2.3	Pregnancy	SAS

19. Appendices

Appendix 1 – List of Potential Immune-Mediated Diseases

Neuroinflammatory disorders	Musculoskeletal disorders	Skin disorders
• Cranial nerve neuropathy, including paralysis and paresis (eg, Bell's palsy).<br>• Optic neuritis.<br>• Multiple sclerosis.<br>• Transverse myelitis.<br>• Guillain-Barré syndrome, including Miller Fisher syndrome and other variants.<br>• Acute disseminated encephalomyelitis, including site specific variants, eg, noninfectious encephalitis, encephalomyelitis, myelitis, myeloradiculoneuritis.<br>• Myasthenia gravis, including Lambert-Eaton myasthenic syndrome.<br>• Demyelinating peripheral neuropathies and plexopathies including:<br>- Chronic inflammatory demyelinating polyneuropathy.<br>- Multifocal motor neuropathy.<br>- Polyneuropathies associated with monoclonal gammopathy.<br>• Narcolepsy.<br>• Tolosa Hunt syndrome.	• Systemic lupus erythematosus and associated conditions.<br>• Systemic scleroderma (systemic sclerosis), including:<br>- Diffuse scleroderma.<br>- CREST syndrome.<br>• Idiopathic inflammatory myopathies, including:<br>- Dermatomyositis.<br>- Polymyositis.<br>• Anti-synthetase syndrome.<br>• Rheumatoid arthritis and associated conditions including:<br>- Juvenile idiopathic arthritis.<br>- Still's disease.<br>• Polymyalgia rheumatica.<br>• Spondyloarthropathies, including:<br>- Ankylosing spondylitis.<br>- Reactive arthritis (Reiter's syndrome).<br>- Undifferentiated spondyloarthritis.<br>- Psoriatic arthritis.<br>- Enteropathic arthritis.<br>• Relapsing polychondritis.<br>• Mixed connective tissue disorder.<br>• Gout.	• Psoriasis.<br>• Vitiligo.<br>• Erythema nodosum.<br>• Autoimmune bullous skin diseases (including pemphigus, pemphigoid, and dermatitis herpetiformis).<br>• Lichen planus.<br>• Sweet's syndrome.<br>• Localized scleroderma (morphea).<br>• Cutaneous lupus erythematosus.<br>• Rosacea.
**Vasculitis**	**Blood disorders**	**Others**
• Large vessels vasculitis including:<br>- Giant cell arteritis (temporal arteritis).<br>- Takayasu's arteritis.<br>• Medium sized and/or small vessels vasculitis including:<br>- Polyarteritis nodosa.	• Autoimmune hemolytic anemia.<br>• Autoimmune thrombocytopenia.<br>• Antiphospholipid syndrome.<br>• Pernicious anemia.<br>• Autoimmune aplastic anemia.<br>• Autoimmune neutropenia.<br>• Autoimmune pancytopenia.	• Autoimmune glomerulonephritis including:<br>- IgA nephropathy.<br>- Glomerulonephritis rapidly progressive.<br>- Membranous glomerulonephritis.

This document is confidential.

- Kawasaki's disease.<br>- Microscopic polyangiitis.<br>- Wegener's granulomatosis (granulomatosis with polyangiitis).<br>- Churg–Strauss syndrome (allergic granulomatous angiitis or eosinophilic granulomatosis with polyangiitis).<br>- Buerger's disease (thromboangiitis obliterans).<br>- Necrotizing vasculitis (cutaneous or systemic).<br>- Antineutrophil cytoplasmic antibody (ANCA) positive vasculitis (type unspecified).<br>- Henoch-Schonlein purpura (IgA vasculitis).<br>- Behcet's syndrome.<br>- Leukocytoclastic vasculitis.		- Membranoproliferative glomerulonephritis.<br>- Mesangioproliferative glomerulonephritis.<br>- Tubulointerstitial nephritis and uveitis syndrome.<br>• Ocular autoimmune diseases including:<br>- Autoimmune uveitis.<br>- Autoimmune retinitis.<br>• Autoimmune myocarditis/cardiomyopathy.<br>• Sarcoidosis.<br>• Stevens-Johnson syndrome.<br>• Sjögren's syndrome.<br>• Alopecia areata.<br>• Idiopathic pulmonary fibrosis.<br>• Goodpasture syndrome.<br>• Raynaud's phenomenon.
**Liver disorders**	**Gastrointestinal disorders**	**Endocrine disorders**
• Autoimmune hepatitis.<br>• Primary biliary cirrhosis.<br>• Primary sclerosing cholangitis.<br>• Autoimmune cholangitis	• Inflammatory bowel disease, including:<br>- Crohn's disease.<br>- Ulcerative colitis.<br>- Microscopic colitis.<br>- Ulcerative proctitis.<br>• Celiac disease.<br>• Autoimmune pancreatitis.	• Autoimmune thyroiditis (including Hashimoto thyroiditis).<br>• Grave's or Basedow's disease.<br>• Diabetes mellitus type 1.<br>• Addison's disease.<br>• Polyglandular autoimmune syndrome.<br>• Autoimmune hypophysitis.

Statistical Analysis Plan for Interventional Studies

Sponsor Name: Medicago R&D Inc.

Protocol Number: CP-PRO-CoVLP-021

Protocol Title: A Randomized, Observer-Blind, Placebo-Controlled, Phase 2/3 Study to Assess the Safety, Efficacy, and Immunogenicity of a Recombinant Coronavirus-Like article COVID-19 Vaccine in Adults 18 Years of Age or Older

Protocol Version and Date: Final Version 7.0 (16-May-2021)

Syneos Health Project Code: [Redacted]

Authors: [Redacted]

Notice of Confidential and Proprietary Information:

The information contained in this document is confidential belonging to Medicago R&D Inc.. Acceptance of this document constitutes agreement by the recipient that no information contained herein will be published or disclosed without prior written authorization from an official of Medicago R&D Inc. However, this document may be disclosed to appropriate Institutional Review Board and Ethics Committees or duly authorized representatives of a national regulatory authority under the condition that they are requested to keep it confidential. In the event of an actual or suspected breach of this obligation, Syneos Health should be notified promptly.

Revision History

Version #	Date (DD-Mmm-YYYY)	Document Owner	Revision Summary
0.1	20-Oct-2020	[Redacted]	Initial Release Version
1.0	29-Oct-2020	[Redacted]	Final Version
1.1	18-Nov-2020	[Redacted]	Protocol Amendment
1.2	10-Dec-2020	[Redacted]	Protocol Amendment
2.0	08-Jan-2021	[Redacted]	Protocol Amendment
3.0	16-Mar-2021	[Redacted]	Protocol Amendment
4.0	21-Sep-2021	[Redacted]	Updated SAP based on comments from Medicago
5.0	18-Oct-2021	[Redacted]	Updated SAP based on comments from Medicago

I confirm that I have reviewed this document and agree with the content.

**Approvals**		
**Syneos Health Approval**<br><br>[Redacted]		
Name, Title<br>Author Biostatistician	Signature	Date (DD-Mmm-YYYY)
[Redacted]		
Name, Title<br>Senior Reviewing Biostatistician	Signature	Date (DD-Mmm-YYYY)
**Medicago Approval**<br><br>[Redacted]		
Name, Title<br>Sponsor Contact	Signature	Date (DD-Mmm-YYYY)
[Redacted]		
Name, Title<br>Sponsor Contact	Signature	Date (DD-Mmm-YYYY)

This document is confidential.

Table of Contents

Revision History 2
Approvals 3
Glossary of Abbreviations 11
1. Purpose 14
1.1. Responsibilities 14
1.2. Timings of Analyses 14
2. Study Objectives 21
2.1. Primary Objectives 21
2.2. Secondary Objectives 21
2.3. Exploratory Objectives 24
3. Study Details/Design 25
3.1. Brief Description 25
3.2. Subject Selection 27
3.2.1. Inclusion Criteria 27
3.2.2. Exclusion Criteria 30
3.3. Determination of Sample Size 33
3.4. Treatment Assignment and Blinding 33
3.4.1. Premature Unblinding Due to Availability of Approved or Authorized COVID-19 Vaccines Prior to IOA/EUA 35
3.5. Administration of Study Medication 36
3.6. Study Procedures and Flowchart 39
4. Endpoints 50
4.1. Efficacy Endpoints 50
4.1.1. Primary Endpoints 50
4.1.2. Secondary Endpoints 51
4.1.3. Exploratory Endpoints 52
4.2. Immunogenicity Endpoints 52
4.2.1. Primary Endpoints 52
4.2.2. Secondary Endpoints 52
4.2.3. Exploratory Endpoints 53
4.3. Safety Endpoints 53

4.3.1. Primary Endpoints 53
4.3.2. Secondary Endpoints 54
4.3.3. Exploratory Endpoint 55
5. Analysis Sets 56
5.1. Safety Analysis Set 56
5.1.1. Solicited Safety Analysis Subset 58
5.2. Intention-To-Treat (ITT) Set 58
5.3. Per Protocol (PP) Set 58
5.4. Immunogenicity ITT and Per Protocol (IPP) Sets 60
6. General Aspects for Statistical Analysis 63
6.1. General Methods 63
6.1.1. Geometric Mean Titer (GMT) 64
6.1.2. Geometric Mean Fold Rise (GMFR) 64
6.1.3. Seroconversion (SC) Rate 64
6.2. Key Definitions 64
6.2.1. Baseline 64
6.2.2. Change from Baseline 64
6.2.3. Onset Day 64
6.2.4. COVID-19 Case 65
6.2.5. Severe COVID-19 Case 65
6.2.6. Date of Unblinding 66
6.3. Missing Data 66
6.3.1. Safety Data 66
6.3.2. Immunogenicity 68
6.4. Solicited Safety Data 68
6.5. Visit Windows 68
6.6. Pooling of Centers 68
6.7. Subgroups 68
7. Demographic, Other Baseline Characteristics and Medication 70
7.1. Subject Disposition and Withdrawals 70
7.2. Demographic and Baseline Characteristics 71

7.3. Medical History and Concomitant Diseases 72
7.4. Medication 72
7.4.1. Prior Medication 72
7.4.2. Concomitant Medication 72
7.5. Treatment Exposure and Compliance 73
8. Efficacy 74
8.1. Primary Efficacy Endpoint(s) and Analysis 74
8.2. Secondary Efficacy Endpoint(s) and Analyses 75
8.2.1. First occurrence of laboratory-confirmed (virologic method) symptomatic SARS-CoV-2 infection (≥ 7 days post-second vaccination) 75
8.2.2. Occurrences of severe COVID-19 disease (≥ 7 days post-second vaccination) 75
8.2.3. Occurrences of laboratory-confirmed asymptomatic SARS-CoV-2 infection at Day 201 in subjects who were seronegative at baseline (confirmed by the ELISA method for the N protein); 76
8.2.4. Occurrences of severe COVID-19 disease (≥ 7 days post-second vaccination) at the time of the PVE analysis 76
8.2.5. Occurrence and intensity of COVID-19-related symptoms in virologically-confirmed cases up until resolution of the symptoms in subjects administered the CoVLP formulation compared to subjects administered the placebo at the time of the PVE analysis 76
8.2.6. First occurrence, in a subject, of laboratory-confirmed (virologic method) symptomatic SARS-CoV-2 infection (≥ first vaccination) at the time of PVE analysis 76
8.2.7. First occurrence, in a subject, of laboratory-confirmed (virologic method) symptomatic SARS-CoV-2 infection (≥ first vaccination and < second vaccination) at the time of PVE analysis 77
8.2.8. First occurrence, in a subject, of laboratory-confirmed (virologic method) symptomatic SARS-CoV-2 infection (≥ second vaccination and < 7 days post-second vaccination) at the time of PVE analysis 77
8.2.9. Duration and intensity of viral shedding after SARS-CoV-2 infection at the time of PVE analysis 77
8.2.10. First occurrence, in a subject, of laboratory-confirmed (virologic method) symptomatic SARS-CoV-2 infection (≥ 7 days post-second vaccination) by strain at the time of the PVE analysis 78
8.3. Exploratory Efficacy Endpoint(s) and Analyses 78
8.3.1. Occurrence and intensity of COVID-19-related symptoms up to resolution of the symptoms 78

8.3.2. Occurrences of severe COVID-19 disease (≥ 7 days post-second vaccination) by strain.78

9. Immunogenicity 79

9.1. Primary Immunogenicity Endpoint(s) and Analysis 79

9.1.1. Nab response induced in each Study Population against the SARS-CoV-2 virus on Days 0, 21, and 42 using GMT, SC rate, and GMFR 79

9.1.2. Specific Th1 CMI response induced in each Study Population against the SARS-CoV-2 virus on Days 0, 21, and 42 measured by IFN-γ ELISpot 80

9.2. Secondary Immunogenicity Endpoint(s) and Analysis 80

9.2.1. Relative neutralizing antibody response between the healthy adults (Study Population #1) and the healthy elderly populations (Study Population #2; each age strata) induced at 21 days after the second vaccination will be analyzed using the following parameter: GMT 80

9.2.2. Relative neutralizing antibody response for the combination of healthy adults (Study Population #1) and the healthy elderly adults (Study Population #2) compared to adults and the elderly adults with significant comorbidities (Study Population #3) induced at 21 days after the second vaccination will be analyzed using the following parameter: GMT 80

9.2.3. Persistence of the Nab antibody response induced in each Study Population against the SARS-CoV-2 virus on Day 128, Day 201, and Day 386 using GMT, SC rate, and GMFR 81

9.2.4. Specific antibody response induced in each Study Population against the SARS-CoV-2 virus by the total IgG levels on Days 0, 21, and 42, and the persistence of these antibodies on Days 128, 201, and 386 using GMT, SC rate, and GMFR 81

9.2.5. The ratio of neutralizing antibody titers:IgG ELISA antibody titers at Day 21, Day 42, Day 128, Day 201, and Day 386....................................82

9.2.6. Specific Th1 CMI response induced in each Study Population against the SARS-CoV-2 virus on Day 201 and Day 386 measured by IFN-γ ELISpot 82

9.2.7. Specific Th2 CMI response induced in each Study Population against the SARS-CoV-2 virus on Days 0, 21, 42, 201, and 386 measured by IL-4 (ELISpot) 83

9.2.8. In the immunogenicity subset, Nab antibody response induced in each Study Population against the SARS-CoV-2 virus on Days 0, 21, 42, 201, and 386 using GMT, SC rate, and GMFR....................................83

9.2.9. In the immunogenicity subset, specific antibody response induced in each Study Population against the SARS-CoV-2 virus by the total IgG levels on Days 0, 21, 42, 201, and 386 using GMT, SC rate, and GMFR 83

9.2.10. In the immunogenicity subset, the ratio of neutralizing antibody titers:IgG ELISA antibody titers at Day 21, Day 42, Day 201, and Day 386....................................83

9.2.11. In the immunogenicity subset, specific Th1 CMI response induced in each Study Population against the SARS-CoV-2 virus on Days 0, 21, 42, 201, and 386 measured by IFN-γ ELISpot 83

9.2.12. In the immunogenicity subset, specific Th2 CMI response induced in each Study Population against the SARS-CoV-2 virus on Days 0, 21, 42, 201, and 386 measured by IL-4 (ELISpot) 83

9.3. Exploratory Immunogenicity Endpoint(s) and Analysis 83

9.3.1. In the immunogenicity subset, specific CMI response induced in each Study Population against the SARS-CoV-2 virus up to 21 days after the second vaccination and at Day 201 and Day 386, as measured by the percentage of CD4+ T cells expressing functional markers 84

9.3.2. Further characterization of the immune response of the CoVLP formulation 84

10. Safety 85

10.1. Adverse Events 86

10.1.1. Solicited Local and Systemic Adverse Events 86

10.1.2. Unsolicited Adverse Events 88

10.1.3. Serious Adverse Events (SAE) 88

10.1.4. Medically Attended Adverse Events (MAAE) 89

10.1.5. Adverse Events of Special Interest (AESI) 89

10.2. Laboratory Evaluations 90

10.3. Vital Signs 92

10.4. Primary Safety Endpoint(s) and Analysis 92

10.4.1. Occurrence, intensity, and relationship (relationship to be assessed for unsolicited AEs only) to vaccination of immediate AEs (30 minutes after each vaccination) 92

10.4.2. Occurrence and intensity of solicited local and systemic AEs (for seven days following each vaccine administration) 92

10.4.3. Occurrence, intensity, and relationship of unsolicited AEs for 21 days following each vaccine administration 92

10.4.4. Number and percentage of subjects with normal and abnormal clinically significant urine, haematological and biochemical values prior to and three days following each vaccination 93

10.4.5. Occurrences of SAEs, MAAEs, AEs leading to withdrawal, AESIs (including VAED), and deaths up to 21 days following each vaccine administration 93

10.5. Secondary Safety Endpoint(s) and Analysis 93

10.5.1. Relative occurrence of solicited local and systemic AEs by intensity grades for seven days following each vaccine administration between the healthy adults (Study Population #1) and the healthy elderly population (Study Population #2, each age strata) 93

10.5.2. Relative occurrence of solicited local and systemic AEs by intensity grades for seven days following each vaccine administration between the healthy adults (Study Population #1) and the healthy elderly adults (Study Population #2) combined and the adults with significant comorbidities (Study Population #3) 93

10.5.3. Occurrences of SAEs, MAAEs, AEs leading to withdrawal, AESIs (including VAED), and deaths from Day 43 to Day 201 94

10.5.4. Occurrences of SAEs, MAAEs, AEs leading to withdrawal, AESIs (including VAED), and deaths from Day 202 to Day 386 94

10.5.5. Occurrence, intensity, and relationship (relationship to be assessed for unsolicited AEs only) to vaccination of immediate AEs (30 minutes after each vaccine administration)..94

10.5.6. Occurrence and intensity of solicited local and systemic AEs (for seven days following each vaccine administration) for the initial set of vaccinations 94

10.5.7. Occurrence, intensity, and relationship of unsolicited AEs for 21 days following each vaccine administration 94

10.5.8. Occurrences of SAEs, MAAEs, AEs leading to withdrawal, AESIs (including VAED, anaphylaxis, and severe allergic reactions), and deaths up to 21 days following each vaccine administration 94

10.5.9. Occurrences of SAEs, MAAEs, AEs leading to withdrawal, AESIs (including VED, anaphylaxis, and severe allergic reactions), and deaths from Day 43 to Day 201 The same safety analyses will be performed as described in Section 10.6.3. 95

10.5.10. Occurrences of SAEs, MAAEs, AEs leading to withdrawal, AESIs (including VAED, anaphylaxis, and severe allergic reactions), and deaths from Day 202 to Day 386..........95

10.6. Exploratory Safety Endpoint(s) and Analysis 95

10.6.1. Further characterization of the safety profile of the CoVLP formulation........................95

10.7. Pregnancy 95

10.8. Physical Examination 95

11. Day 21 and Day 42 Immunogenicity Data Analysis and Efficacy Analysis 96

12. Changes from Analysis Planned in Protocol 98

13. Reference List 101

14. Programming Considerations 102

14.1. General Considerations 102

14.2. Table, Figure, and Listing Format 102

14.2.1. General 102

14.2.2. Headers 102
14.2.3. Display Titles 103
14.2.4. Column Headers 103
14.2.5. Body of the Data Display 104
14.2.6. Footnotes 106
15. Quality Control 107
16. Appendices 108

List of Tables

Table 1 Timing of Analyses 14
Table 2 Study Populations 27
Table 3 Time and Events Schedule: General Information for the Phase 2 Portion 39
Table 4 Time and Events Schedule: General Information for the Phase 3 Portion 43
Table 5 Time and Events Schedule: Onset of COVID-19 48
Table 6 Severity Grades for Solicited Local and Systemic Adverse Events 87
Table 7 Clinical Laboratory Tests for the Phase 2 Portion of the Study 91

List of Figures

Figure 1 Decision Tree for Possible Premature Unblinding in Phase 2 and Phase 3 Portions Prior to IOA/EUA 36
Figure 2 Decision Tree for Handling of Safety Data from Subjects Who Request to Become Prematurely Unblinded to Receive an Approved or Authorized COVID-19 Vaccine in Phase 2 and Phase 3 Portions Prior to IOA/EUA 57
Figure 3 Decision Tree for Handling of Efficacy Data from Subjects Who Request to Become Prematurely Unblinded to Receive an Approved or Authorized COVID-19 Vaccine in Phase 2 and Phase 3 Portions Prior to IOA/EUA 59
Figure 4 Decision Tree for Handling of Immunogenicity Data from Subjects Who Request to Become Prematurely Unblinded to Receive an Approved or Authorized COVID-19 Vaccine in Phase 2 and Phase 3 Portions Prior to IOA/EUA 62

Glossary of Abbreviations

Abbreviation	Description
AE	adverse event
AESI	adverse event of special interest
ANOVA	analysis of variance
ANCOVA	analysis of covariance
BMI	body mass index
BP	blood pressure
CBER	Center for Biologics Evaluation and Research
CI	confidence interval
CMI	cell-mediated immune (response)
CSR	clinical study report
DBP	diastolic blood pressure
eCRF	electronic case report form
ECMO	extracorporeal membrane oxygenation
ELISA	enzyme-linked immunosorbent assay
ELISpot	enzyme-linked immuno spot assay
EUA	Emergency Use Authorization
FDA	Food and Drug Administration
FiO2	fraction of inspired oxygen
GCP	good clinical practice
GMFR	geometric mean fold rise
GMT	geometric mean titer
HIV	human immunodeficiency virus
HR	heart rate
IB	Investigator's Brochure
IOA	Interim Order Authorization
ICF	informed consent form
ICH	International Council for Harmonisation
IDMC	Independent Data Monitoring Committee
IEC	independent ethics committee
IFN-γ	interferon gamma
IgG	Immunoglobulin G

IL-4	Interleukin 4
IM	intramuscular
IPP	Immunogenicity per protocol
IRB	institutional review board
IRT	interactive response technology
ITT	intention-to-treat
LOD	Limit of Detection
MAAE	medically attended adverse events
MedDRA	Medical Dictionary for Regulatory Activities
Nab	neutralizing antibody
OR	odds ratio
OT	oral temperature
PaO2	partial pressure of oxygen
PBMC	peripheral blood mononuclear cell
pIMD	potential immune-mediated disease
PP	per protocol
PVE	primary vaccine efficacy
RR	respiratory rate
RTSM	randomization and trial supply management system
SAE	serious adverse event
SAP	statistical analysis plan
SAS	safety analysis set
SAS®	Statistical Analysis System®
SBP	systolic blood pressure
SC	seroconversion
SpO2	oxygen saturation as measured by pulse oximetry
Th1	T helper 1
TFL	tables, figures and listings
ULOQ	Upper Limit of Quantification
VE	vaccine efficacy
VED	vaccine-enhanced disease
VLP	virus-like particle
WHO	World Health Organization

UK	United Kingdom
US	United States

1. Purpose

The purpose of this statistical analysis plan (SAP) is to ensure that the data listings, summary tables and figures which will be produced, and the statistical methodologies which will be used, are complete and appropriate to allow valid conclusions regarding the study objectives.

1.1. Responsibilities

Syneos Health will perform the statistical analyses and is responsible for the production and quality control of all tables, figures and listings.

1.2. Timings of Analyses

Primary analysis for Phase 2 is up to 21 days after second vaccination. For Phase 3, the primary analysis for efficacy will occur when at least 160 subjects have at least one laboratory-confirmed symptomatic COVID-19 cases (≥ 7 days post-second vaccination) and the median blinded safety follow-up duration of at least 2 months (post-administration of the second vaccination) for at least 3000 subjects in each of the CoVLP formulation and placebo groups has been achieved.

The Independent Data Monitoring Committee (IDMC) will consist of a multidisciplinary group of two clinicians and/or physicians and one biostatistician who will, collectively, have extensive experience in the use of vaccines for patient treatment and in the conduct and safety monitoring of randomized clinical trials. The IDMC will monitor the study conduct and review blinded and unblinded safety data at pre-defined times during the study for the purpose of providing recommendations to the Sponsor about whether:

- subject vaccination in different Study Populations will occur;
- subject vaccination in each Study Population will continue;
- the study portion will continue in the event of a pre-defined safety or efficacy signal.

A subcommittee of the IDMC will review all symptomatic COVID-19 cases potentially meeting the criteria of the primary and secondary efficacy endpoints.

Table 1 Timing of Analyses

Timing of Analyses	Population	Purposes	Requirements
**Phase 2**			
IDMC 1	2	• Reviewing solicited, unsolicited, and adverse events of special interest (AESI)<br>• Day 7 safety data for 10 sentinel subjects	• See IDMC Charter for more details (safety)

Timing of Analyses	Population	Purposes	Requirements
IDMC 2	3	• Reviewing solicited, unsolicited, and AESI. Day 7 safety data for 10 sentinel subjects	• See IDMC Charter for more details (safety)
IDMC 3	2	• Reviewing Day 28 safety data for 10 sentinel subjects.	• See IDMC Charter for more details (safety)
IDMC 4	3	• Reviewing Day 28 safety data for 10 sentinel subjects.	• See IDMC Charter for more details (safety)
Phase 2 to 3 package No.1 to be delivered to Medicago unblinded individuals	1	• Assessing at Medicago if Population #1 can proceed into the Phase 3 portion based on Day 28 safety and Day 21 immunogenicity data.	• Days 0, 21 Neutralizing antibodies (Nab) tables[1]<br>• Days 0, 21 ELISpot IFN-g and IL-4 tables[1]<br>• Demographics table<br>• Safety summary tables[2]<br>• Immediate AE tables per intensity (solicited and unsolicited)[2]<br>• Solicited AE tables per intensity[2]<br>• Unsolicited AE table per intensity[2]<br>• SAE table[2]<br>• MAAE table[2]<br>• AESI tables[2]<br>• (Output could be delivered in batches as outputs become available)
IDMC 5	1	• Confirming if Population #1 can be enrolled in Phase 3 based on Day 28 safety and Day 21 immunogenicity data.	• Phase 2 to 3 package No.1
Phase 2 to 3 package No.2 to be delivered to Medicago	1	• Assessing at Medicago if Population #1 can proceed into the Phase 3 portion based on Day 42 safety and immunogenicity data.	• Days 0, 21, 42 Nab tables[1]<br>• Days 0, 21, 42 ELISpot IFN-g and IL-4 tables[1]<br>• Safety summary tables[3]<br>• Immediate AE tables per intensity (solicited and unsolicited)[3]<br>• Solicited AE tables per intensity[3]<br>• Unsolicited AE tables per intensity[3]<br>• SAE tables[3]<br>• MAAE tables[3]

Timing of Analyses	Population	Purposes	Requirements
			• AESI tables[3]<br>• (Output could be delivered in batches as outputs become available)
IDMC 6	1	• Confirming if Population #1 can be enrolled in Phase 3 Day 42 safety and immunogenicity data.	• Phase 2 to 3 package No.2
Phase 2 to 3 package No.3 to be delivered to Medicago unblinded individuals	2	• Assessing at Medicago if Population #2 can proceed into the Phase 3 portion based on Day 28 safety and Day 21 immunogenicity data.	• Days 0, 21 Nab tables[1]<br>• Days 0, 21 ELISpot IFN-g and IL-4 tables[1]<br>• Days 0, 21 Nab comparative tables between Population #1 and each age strata included in Population #2[1]<br>• Comparative Solicited AE tables between Population #1 and each age strata included in Population #2 presented by intensity<br>• Demographics table<br>• Safety summary tables[2]<br>• Immediate AE tables per intensity (solicited and unsolicited)[2]<br>• Solicited AE tables per intensity[2]<br>• Unsolicited AE tables per intensity[2]<br>• SAE tables[2]<br>• MAAE tables[2]<br>• AESI tables[2]<br>• (Output could be delivered in batches as outputs become available)
Phase 2 to 3 package No.4 to be delivered to Medicago unblinded individuals	2	• Assessing at Medicago if Population #2 can proceed into the Phase 3 portion based on Day 42 safety and immunogenicity data.	• Days 0, 21, 42 Nab tables[1]<br>• Days 0, 21, 42 ELISpot IFN-g and IL-4 tables[1]<br>• Days 0, 21, 42 Nab comparative tables between Population #1

Timing of Analyses	Population	Purposes	Requirements
			and each age strata included in Population #2[1]<br>• Comparative Solicited AE tables between Population #1 and each age strata included in Population #2 presented by intensity<br>• Safety summary tables[3]<br>• Immediate AE tables per intensity (solicited and unsolicited)[3]<br>• Solicited AE tables per intensity[3]<br>• Unsolicited AE tables per intensity[3]<br>• SAE tables[3]<br>• MAAE tables[3]<br>• AESI tables[3]<br>• (Output could be delivered in batches as outputs become available)
IDMC 7	2	• Evaluating if this population can be enrolled in Phase 3 and assess if dose escalation is required	• Phase 2 to 3 package No.2
Phase 2 to 3 package No.5 to be delivered to Medicago unblinded individuals	3	• Assessing at Medicago if Population #3 can proceed into the Phase 3 portion based on Day 28 safety and Day 21 immunogenicity data.	• Days 0, 21 Nab tables[1]<br>• Days 0, 21 ELISpot IFN-g and IL-4 tables[1]<br>• Days 0, 21 Nab comparative tables between Population #1 and Population #3, Population #2 and Population #3, and Populations #1 + #2 combined and Population #3[1]<br>• Comparative Solicited AE tables between Population #1 and Population #3, Population #2 and Population #3, and Populations #1 + #2 combined and

Timing of Analyses	Population	Purposes	Requirements
			Population #3 presented by intensity<br>• Demographics table<br>• Safety summary tables[2]<br>• Immediate AE tables per intensity (solicited and unsolicited)[2]<br>• Solicited AE tables per intensity[2]<br>• Unsolicited AE tables per intensity[2]<br>• SAE tables[2]<br>• MAAE tables[2]<br>• AESI tables[2]<br>• (Output could be delivered in batched as outputs become available)
Phase 2 to 3 package No.6 to be delivered to Medicago unblinded individuals	3	• Assessing at Medicago if Population #3 can proceed into the Phase 3 portion based on Day 42 safety and immunogenicity data.	• Days 0, 21, 42 Nab tables[1]<br>• Days 0, 21, 42 ELISpot IFN-g and IL-4 tables[1]<br>• Days 0, 21, 42 Nab comparative tables between Population #1 and Population #3, Population #2 and Population #3, and Populations #1 + #2 combined and Population #3[1]<br>• Comparative Solicited AE tables between Population #1 and Population #3, Population #2 and Population #3, and Populations #1 + #2 combined and Population #3 presented by intensity<br>• Safety summary tables[3]<br>• Immediate AE tables per intensity (solicited and unsolicited)[3]<br>• Solicited AE tables per intensity[3]

Timing of Analyses	Population	Purposes	Requirements
			• Unsolicited AE tables per intensity[3]<br>• SAE tables[3]<br>• MAAE tables[3]<br>• AESI tables[3]<br>• (Output could be delivered in batches as outputs become available)
IDMC 8	3	• Evaluating if this population can be enrolled in Phase 3 and assess if dose escalation is required	• Phase 2 to 3 package No.3
Day 201	All	• Preparation of the Day 201 CSR	• All TLFs specified in SAP mock shells
End of Study	All	• Preparation of the final CSR	• All TLFs specified in SAP mock shells
IDMC 9	3	• Population 3 Day 28 Safety and Immunogenicity tables and listings were delivered.	• Safety tables/listings<br>• Immunogenicity tables/listings
IDMC 10	3	• Population 3 Day 42 Safety and Immunogenicity tables and listings were delivered.	• Safety tables/listings<br>• Immunogenicity (Nab/ELISpot) tables/listings
**Phase 3**			
IOA/EUA<br>Ad-hoc IDMC	All	• Performing primary efficacy analysis once 160 subjects with positive COVID-19 symptomatic cases (≥ 7 days post-second vaccination) have been collected and the median blinded safety follow-up duration of at least 2 months (post-administration of the second vaccination) for at least 3000 subjects in each of the CoVLP formulation and placebo groups has been achieved	• Assessment for the primary efficacy endpoint<br>• Distribution of severe COVID-19 cases between the CoVLP and placebo groups
End of Study	All	• Preparation of the CSR	• All TLFs specified in SAP mock shells

[1] To be presented separately for i) All subjects, ii) Seronegative subjects at baseline for SARS-CoV-2 and iii) Seropositive subjects at baseline for SARS-CoV-2.

Timing of Analyses	Population	Purposes	Requirements

[2] Solicited AEs to be presented up to 7-day after each vaccination. Unsolicited AEs, SAE, MAAE, AESI to be presented up to 21 days after first vaccination and up to 7 days after the second vaccination.
[3] Solicited AEs to be presented up to 7-day after each vaccination. Unsolicited AEs, SAE, MAAE, AESI to be presented up to 21 days after each vaccination.
[4] Timing of analysis may change if the enrollment rate is different from the initial plan.

2. Study Objectives

2.1. Primary Objectives

The primary objectives of the Phase 2 portion of the study are:

Safety:

- To assess the safety and tolerability of the CoVLP formulation, compared to placebo in each of the Study Populations, up to 21 days after the second vaccination.

Immunogenicity:

- To assess the immunogenicity of the CoVLP formulation, compared to placebo, up to 21 days after the second vaccination in each of the Study Populations, as determined by:
- Neutralizing antibody (Nab) titers induced by the vaccine against the SARS-CoV-2 virus;
- IFN-γ ELISpot response induced by the vaccine against the SARS-CoV-2 virus to assess the T helper 1 (Th1) type response.

The primary objective of the Phase 3 portion of the study is:

Efficacy:

- To evaluate the efficacy of the CoVLP formulation, compared to placebo, in the prevention of laboratory-confirmed symptomatic SARS-CoV-2 infection (virologic method) starting 7 days after the second vaccination at the time of the primary vaccine efficacy (PVE) analysis (i.e., once at least 160 COVID-19 cases [≥ 7 days post-second vaccination] have been collected and the median blinded safety follow-up duration of at least 2 months (post-administration of the second vaccination) for at least 3000 blinded subjects in each of the CoVLP formulation and placebo groups has been achieved).

2.2. Secondary Objectives

The secondary objectives of the Phase 2 portion of the study are:

Immunogenicity:

- To assess the relative Nab response between the healthy adults (Study Population #1) and the healthy elderly populations (Study Population #2; each age strata) at 21 days after the second vaccination;
- To assess the relative Nab response between the healthy adults (Study Population #1) and the healthy elderly adults (Study Population #2) combined and the adults with significant comorbidities (Study Population #3) at 21 days after the second vaccination;
- To assess the immunogenicity of the CoVLP formulation, compared to placebo, when applicable, in each of the Study Populations, as determined by the:
- Persistence of Nab titers induced by the vaccine against the SARS-CoV-2 virus at Days 128, 201, and Day 386;

- Immunoglobulin G (IgG) (ELISA) titers induced by the vaccine against the SARS-CoV-2 virus up to 21 days after the second vaccination and at persistence of these antibodies Day 128, 201, and Day 386;
- The ratio of Nab titers:IgG (ELISA) antibody titers at Day 21, Day 42, Day 128, Day 201, and Day 386;
- IFN-γ ELISpot response induced by the vaccine against the SARS-CoV-2 virus at Day 201 and Day 386;
- Interleukin-4 (IL-4) ELISpot response induced by the vaccine against the SARS-CoV-2 virus up to 21 days after the second vaccination and at Day 201 and Day 386.

Safety:

- To assess the relative incidence of solicited local and systemic adverse events (AEs) up to 7 days after the first and second vaccinations between the healthy adults (Study Population #1) and each healthy elderly population (Study Population #2) in the CoVLP formulation group prior to Interim Order Authorization (IOA)/Emergency Use Authorization (EUA) only;
- To assess the relative incidence of solicited local and systemic AEs up to 7 days after the first and second vaccinations between the healthy adults (Study Population #1) and the healthy elderly adults (Study Population #2) combined and the adults with significant comorbidities (Study Population #3) in the CoVLP formulation group prior to IOA/EUA only;
- To assess the safety and tolerability of the CoVLP formulation, compared to placebo in each of the Study Populations, as determined by the occurrence(s) of:
    - Medically attended adverse events (MAAEs), serious adverse events (SAEs), AEs leading to withdrawal, AEs of special interest (AESIs; including vaccine-enhanced disease [VAED]), and deaths from 22 days after the second vaccination up to IOA/EUA.

Efficacy:

- To evaluate the efficacy of the CoVLP formulation, compared to placebo, in the prevention of laboratory-confirmed symptomatic SARS-CoV-2 infection (virologic method) starting 7 days after the second vaccination;
- To evaluate the efficacy of the CoVLP formulation, compared to placebo, in the prevention of severe COVID-19 disease starting 7 days after the second vaccination.

The secondary objectives of the Phase 3 portion of the study are:

Efficacy:

- To evaluate the efficacy of the CoVLP formulation, in the prevention of laboratory-confirmed asymptomatic SARS-CoV-2 infection at Day 201 in subjects who were seronegative at baseline (confirmed by serologic method);

- To evaluate the efficacy of the CoVLP formulation, compared to placebo, in the prevention of severe COVID-19 disease starting 7 days after the second vaccination at the time of the PVE analysis;
- To assess COVID-19-related symptoms in virologically-confirmed cases, and the severity of these symptoms up until resolution in subjects administered the CoVLP formulation compared to subjects administered the placebo at the time of the PVE analysis;
- To evaluate the efficacy of the CoVLP formulation, compared to placebo, in the prevention of laboratory-confirmed symptomatic SARS-CoV-2 infection (virologic method) starting after the first vaccination at the time of the PVE analysis;
- To evaluate the efficacy of the CoVLP formulation, compared to placebo, in the prevention of laboratory-confirmed symptomatic SARS-CoV-2 infection (virologic method) after the first vaccination and prior to the second vaccination at the time of the PVE analysis;
- To evaluate the efficacy of the CoVLP formulation, compared to placebo, in the prevention of laboratory-confirmed symptomatic SARS-CoV-2 infection (virologic method) after the second vaccination and prior to 7 days post-second vaccination at the time of the PVE analysis;
- To assess the duration and intensity of viral shedding after virologically-confirmed SARS-CoV-2 infection in subjects administered the CoVLP formulation compared to subjects administered the placebo at the time of the PVE analysis;
- To evaluate the efficacy of the CoVLP formulation, compared to placebo, in the prevention of laboratory-confirmed symptomatic SARS-CoV-2 infection (virologic method) by strain starting 7 days after the second vaccination at the time of the PVE analysis.

Immunogenicity:

- To assess the immunogenicity of the CoVLP formulation, compared to placebo when applicable, in the immunogenicity subset, as determined by:
    - Nab titers induced by the vaccine against the SARS-CoV-2 virus at Day 0 (prior to vaccination; all subjects), Day 21, Day 42 (all subjects), Day 201, and Day 386;
    - IgG (ELISA) titers induced by the vaccine against the SARS-CoV-2 virus up to 21 days after the second vaccination and at Day 201 and Day 386;
    - The ratio of Nab titers:IgG (ELISA) antibody titers at Day 21, Day 42, Day 201, and Day 386;
    - IFN-γ ELISpot response induced by the vaccine against the SARS-CoV-2 virus up to 21 days after the second vaccination and at Day 201 and Day 386;
    - IL-4 ELISpot response induced by the vaccine against the SARS-CoV-2 virus up to 21 days after the second vaccination and at Day 201 and Day 386.

Safety:

- To assess the safety and tolerability of the CoVLP formulation, up to the end of the study.

2.3. Exploratory Objectives

The exploratory objectives of the Phase 2 portion of the study are:

Efficacy:

- To assess COVID-19-related symptoms in subjects with virologically-confirmed disease, and the severity of these symptoms, up until resolution in subjects administered the CoVLP formulation compared to subjects administered the placebo.

Immunogenicity:

- To further characterize the immune response of the CoVLP formulation, if it is likely to be informative.

Safety:

- To further characterize the safety profile of the CoVLP formulation, if it is likely to be informative.

The exploratory objectives of the Phase 3 portion of the study are:

Efficacy:

- To evaluate the efficacy of the CoVLP formulation, compared to placebo, in the prevention of severe COVID-19 disease by strain starting 7 days after the second vaccination at the time of the PVE analysis.

Immunogenicity:

- To assess the immunogenicity of the CoVLP formulation, compared to placebo when applicable, in the immunogenicity subset, as determined by:
    - Specific cell-mediated immune (CMI) response induced by the vaccine against the SARS-CoV-2 virus up to 21 days after the second vaccination and at Day 201 and Day 386, as measured by the percentage of CD4+ T cells expressing functional markers;
- To further characterize the immune response of the CoVLP formulation, if it is likely to be informative.

Safety:

- To further characterize the safety profile of the CoVLP formulation, if it is likely to be informative.

3. Study Details/Design

3.1. Brief Description

The Phase 2 portion is a randomized, observer-blinded, placebo-controlled study with male and female subjects. Subjects will be enrolled from three Study Populations (refer to Table 2).

In each of the three Study Populations, up to 306 subjects will be randomized 5:1 and will receive the same CoVLP formulation or placebo. The total number of subjects in each Study Population may vary depending on when sufficient data has been accumulated to make a decision on when each Study Population can procced to the Phase 3 portion. Unless full enrollment (i.e., enrolled up to 306 subjects) has already been achieved, further enrollment into each Study Population will be closed once sufficient data has been accumulated to make the decision to proceed to Phase 3. Subjects will receive two intramuscular (IM) injections 21 days apart on Days 0 and 21, into the deltoid region of the alternating arm (i.e., each arm will be injected once), of one of the following:

- CoVLP formulation;
- Placebo.

All subjects will begin enrollment at the same time. To the greatest extent possible given local conditions (i.e., COVID-19 vaccine roll-out), enrollment of elderly adult subjects (Study Population #2) will be stratified by age in an approximately 2:1 ratio of 65 to 74 years of age and 75 years of age and older, respectively. Enrollment of subjects in Study Population #3 will include adults with significant comorbidities such as, but not limited to, obesity or being overweight, documented hypertension, cardiovascular diseases, hyperlipidemia, chronic kidney disease or related disorders, chronic respiratory disease, pre-diabetes and diabetes (type 1 and type 2), and treatment-controlled human immunodeficiency virus (HIV).

The IDMC will review unblinded safety data (i.e., solicited and unsolicited safety data including laboratory safety data collected up to 7 days after each vaccination) for the first 10 sentinel subjects after each dose in all Study Populations, with the exception of Study Populations #1, according to the details outlined in the IDMC Charter. These IDMC reviews will detect any early negative trends in the safety data from a subset of subjects vaccinated within the Study Population and may necessitate a decision to not administer the study vaccine to the remaining study subjects. During the Phase 2 portion, the IDMC will review the safety data collected up to Day 28 from all the subjects in each Study Population to confirm that each Study Population can be included in the Phase 3 portion of the study.

The Phase 3 portion is a randomized, observer-blinded, placebo-controlled, efficacy, safety, immunogenicity (in a subset of subjects), multicenter design with male and female subjects. Similar to the Phase 2 portion, subjects will be enrolled from three Study Populations (refer to Table 2). Study Population #2 will be roughly stratified by age in the same manner to the greatest extent possible given local conditions (i.e., COVID-19 vaccine roll-out). Enrollment of subjects in Study Population #3 will include adults with significant comorbidities such as, but not limited

to, obesity (moderate or greater), cardiovascular diseases, hyperlipidemia, chronic kidney disease or related disorders, chronic respiratory disease, pre-diabetes and diabetes, and treatment-controlled HIV.

The overall goal of the Phase 3 portion of this study is to enroll approximately 30 000 subjects who are generally representative of the overall target population in terms of racial diversity with some degree of enrichment for those at highest risk for serious COVID-19 disease (i.e., the elderly and adults with significant comorbidities).

In an attempt to identify an adverse imbalance of COVID-19 and/or severe COVID-19 cases between the vaccine and placebo recipients, which is indicative of possible vaccine-enhanced disease (VED), an unblinded team will review COVID-19 and/or severe COVID-19 cases on an ongoing basis to determine if the probability of observing the same or a more extreme case split is 5% or less when the true incidence of severe disease is the same for vaccine and placebo recipients. If this criterion is met, then the unblinded team will advise the IDMC members who will review the information along with other available information and determine if the study should continue, be modified, be halted or be stopped. The monitoring of potential imbalance of severe COVID-19 cases will start once 8 cases have been accumulated. The assessment will be performed using a one-sided binomial test, which will provide probabilities of having an adverse imbalance between the vaccine and placebo arms given an expected ratio of 5:1 in phase 2 and a ratio of 1:1 in phase 3. In addition, an alert rule will be put in place and will be triggered when the probability of having imbalance in severe cases in the vaccine arm versus placebo arm is less than 15%.

In each Study Population, subjects will be randomized to receive either the CoVLP formulation or the placebo. Subjects in the placebo group will be offered the CoVLP formulation once IOA/EUA is obtained.

Subjects will be screened and will demonstrate a satisfactory baseline medical assessment by history and general physical examination prior to the first vaccine administration. On Day 0 and Day 21, vaccine administration will occur. Subjects will be provided with a diary and memory aid to collect safety and concomitant medication data. A visit to the Investigator site will occur on Day 21 (immunogenicity subset only) and Day 42 (all subjects) for immunogenicity assessments. Subjects who initially receive the CoVLP formulation and subjects in the placebo group who do not want the CoVLP formulation will return to the Investigator site on Day 201, and Day 386 (6-month and 12-month safety follow-ups and immunogenicity assessments [immunogenicity subset only] after the last vaccine administration).

Active surveillance will be conducted in the study from the day of the first vaccination (Day 0, post-vaccination) until IOA/EUA and passive surveillance will be conducted in the study from the day of the first vaccination (Day 0, post vaccination) until the end of the study. For passive surveillance, subjects will be instructed to report symptoms meeting the definition of new COVID-19 disease. For active surveillance, subjects will be contacted once per week via the electronic diary and asked about symptoms of COVID-19 disease. Other methods of contact

This document is confidential.

(phone, text, email) will only be used as a last resort to contact the subject. In addition, to monitor asymptomatic infection, subjects who initially receive the CoVLP formulation or subjects in the placebo group who do not want the CoVLP formulation will perform a test for SARS-CoV-2 N antibodies on Day 0 and Day 201. Subjects in the placebo group who receive the CoVLP formulation post-IOA/EUA will perform the same test on Day 0 and just prior to receiving the first dose of the CoVLP formulation.

Once subjects in the placebo group receive the CoVLP formulation (post-IOA/EUA), they will no longer be followed for efficacy (no more active surveillance) or immunogenicity (those included in the immunogenicity subset). Passive surveillance for possible COVID-19 cases will continue however. Subjects will provide a blood draw immediately prior to receiving the first dose of the CoVLP formulation and then will be followed for safety with monthly phone contacts until Day 386, when the final study telephone contact will occur.

Table 2 Study Populations

Study Population	Age and/or Health Category	Age Range (years)
1	Healthy adults	18-64
2	Healthy elderly adults*	≥ 65
3	Adults with significant co-morbidities*	≥ 18

*Subjects to be enrolled in country(ies) that has/have provided authorization to enroll these subjects in the study.

3.2. Subject Selection

3.2.1. Inclusion Criteria

Subjects must meet all of the following inclusion criteria at the Screening (Visit 1) and/or Vaccination (Visit 2) visits (these two visits are combined into one visit in the Phase 3 portion [Visit 1]) to be eligible for participation in this study; no protocol waivers are allowed. All Investigator assessment-based judgements must be carefully and fully documented in the source documents:

1. Subjects must have read, understood, and signed the informed consent form (ICF) prior to participating in the study; subjects must also complete study-related procedures and the subjects must communicate with the study staff at visits and by phone during the study;

2. At the Screening visit (Visit 1), male and female subjects must be

    - Study Populations #1: 18 to 64 (has not yet had his/her 65th birthday) years of age, inclusive;

    - Study Population #2: 65 years of age or older;

    - Study Population #3: 18 years of age or older;

3. At Screening (Visit 1) and Vaccination (Visit 2), subject must have a body mass index (BMI) of:

   <u>For the Phase 2 portion of the study only:</u>

   - Study Populations #1 and #2: ≥ 18.5 and < 30 kg/m^2;

   <u>For the Phase 3 portion of the study only:</u>

   - Study Populations #1 and #2: ≥ 18.5 and < 35 kg/m^2;

4. Subjects are considered by the Investigator to be reliable and likely to cooperate with the assessment procedures and be available for the duration of the study;

5. <u>Study Population #1</u>: Subjects must be in good general health prior to study participation, with no clinically relevant abnormalities that could jeopardize subject safety or interfere with study assessments, as determined by medical history, physical examination, and vital signs. Investigator discretion will be permitted with this inclusion criterion;

   All regions except Canada: Note: Subjects with a pre-existing chronic disease will be allowed to participate if the disease is stable and, according to the Investigator's judgment that must be documented in the source documents, the condition is unlikely to confound the results of the study or pose additional risk to the subject by participating in the study. Stable disease is generally defined as no new onset or exacerbation of pre-existing chronic disease three months prior to vaccination. Based on the Investigator's judgment, a subject with more recent stabilization of a disease could also be eligible.

6. <u>Study Population #1 and #3</u>: Female subjects of childbearing potential must have a negative serum pregnancy test result at Screening (Visit 1 for the Phase 2 portion) and/or a negative urine pregnancy test result at Vaccination (Visit 2 for the Phase 2 portion; Visit 1 for the Phase 3 portion):

   Non-childbearing females are defined as:

   - Surgically-sterile (defined as bilateral tubal ligation, hysterectomy or bilateral oophorectomy performed more than one month prior to the first study vaccination); or

   - Post-menopausal (absence of menses for 12 consecutive months and age consistent with natural cessation of ovulation);

7. <u>Study Population #1 and #3</u>: Female subjects of childbearing potential must use an effective method of contraception for one month prior to vaccination (Visit 2 for the Phase 2 portion and Visit 1 for the Phase 3 portion) and agree to continue employing

highly effective birth control measures for at least one month after the last study vaccination (or in the case of early termination, she must not plan to become pregnant for at least one month after her last study vaccination.

The following relationship or methods of contraception are considered to be highly effective:

- Combined (estrogen and progestogen containing) hormonal contraception associated with inhibition of ovulation:
    - Oral;
    - Intravaginal;
    - Transdermal;
- Progestogen-only hormonal contraception associated with inhibition of ovulation:
    - Oral;
    - Injectable;
    - Implantable;
- Intra-uterine device with or without hormonal release;
- Credible self-reported history of heterosexual vaginal intercourse abstinence prior to and for at least one month after the last study vaccination. Abstinent subjects who are ovulating should be asked what method(s) they would use should their circumstances change, and subjects without a well-defined plan should be excluded.
- Female partner;
- All regions except the US: Vasectomised partner, provided that this partner is the sole sexual partner of the study participant and that the vasectomised partner has received a medical assessment of the surgical success;
- Bilateral tubal occlusion.

8. <u>Study Population #2</u>: Subjects must be non-institutionalized (e.g. not living in rehabilitation centres or old-age homes; living in an elderly community is acceptable) and have no acute or evolving medical problems prior to study participation and no clinically relevant abnormalities that could jeopardize subject safety or interfere with study assessments, as assessed by the Principal Investigator or sub-Investigator (thereafter

referred as Investigator) and determined by medical history, physical examination, serology (only for the Phase 2 portion), clinical chemistry and haematology tests (only for the Phase 2 portion), urinalysis (only for the Phase 2 portion), and vital signs. Investigator discretion will be permitted with this inclusion criterion.

All regions except Canada: Note: Subjects with a pre-existing chronic disease will be allowed to participate if the disease is stable and, according to the Investigator's judgment that must be documented in the source documents, the condition is unlikely to confound the results of the study or pose additional risk to the subject by participating in the study. Stable disease is generally defined as no new onset or exacerbation of pre-existing chronic disease three months prior to vaccination. Based on the Investigator's judgment and documented in source documentation, a subject with more recent stabilization of a disease could also be eligible;

9. Study Population #3: Subjects must have one or more co-morbid conditions that puts them at higher risk for severe COVID-19 disease. These comorbidities include but are not limited to obesity (moderate or greater), hypertension, type 1 or type 2 diabetes, chronic obstructive pulmonary disease (COPD), cardiovascular diseases, chronic kidney diseases, or be immunocompromised persons (e.g., treatment-controlled HIV infection, organ transplant recipients, or patients receiving cancer chemotherapy). Investigator discretion will be permitted with this inclusion criterion.

3.2.2. Exclusion Criteria

Subjects who meet any of the following criteria at the Screening (Visit 1) and/or Vaccination (Visit 2) visits (these two visits are combined into one visit in the Phase 3 portion [Visit 1]) will not be eligible for participation in this study; no protocol waivers are allowed. All Investigator assessment-based judgements must be thoroughly documented in the source documents:

1. Study Populations #1 and #2: According to the Investigator's opinion, significant acute or chronic, uncontrolled medical or neuropsychiatric illness.

   Acute disease is defined as presence of any moderate or severe acute illness with or without a fever within 48 hours prior to the Screening (Visit 1) and/or Vaccination visit (Visit 2). 'Uncontrolled' is defined as:

   - Requiring a new medical or surgical treatment during the three months prior to study vaccine administration;
   - Requiring any significant change in a chronic medication (i.e., drug, dose, frequency) during the three months prior to study vaccine administration due to uncontrolled symptoms or drug toxicity unless the innocuous nature of the medication change meets the criteria outlined in inclusion criterion no. 5 (Study Population #1) or no. 8 (Study Population #2) and is appropriately justified and documented by the Investigator.

Investigator discretion is permitted with this exclusion criterion and must be carefully and fully documented in the source documents;

2. Study Populations #1 and #2: Any chronic medical condition associated with elevated risk of severe outcomes of COVID-19, including obesity, diabetes (type I/II), significant cardiovascular or respiratory disease including asthma, chronic renal failure, disorders of bleeding/coagulation, chronic inflammatory or autoimmune conditions, immunosuppressive conditions (including HIV), and hypertension;

3. Study Populations #1 and #2: Any confirmed or suspected current immunosuppressive condition or immunodeficiency, including cancer, human immunodeficiency virus (HIV), hepatitis B or C infection (subjects with a history of cured hepatitis B or C infection without any signs of immunodeficiency at present time are allowed). Investigator discretion is permitted with this exclusion criterion;

4. Study Populations #1 and #2: Current autoimmune disease (such as rheumatoid arthritis, systemic lupus erythematosus or multiple sclerosis). Investigator discretion is permitted with this exclusion criterion, and subjects may be eligible to participate with appropriate written justification in the source document (i.e., subjects with a history of autoimmune disease who are disease-free without treatment for three years or more, or on stable thyroid replacement therapy, mild psoriasis [i.e., a small number of minor plaques requiring no systemic treatment], etc.);

5. Study Populations #1 and #2: Administration of any medication or treatment that may alter the vaccine immune responses, such as:

    - Systemic glucocorticoids at a dose exceeding 10 mg of prednisone (or equivalent) per day for more than seven consecutive days or for 10 or more days in total, within one month prior to the Vaccination visit (Visit 2 for the Phase 2 portion and Visit 1 for the Phase 3 portion). Inhaled, nasal, ophthalmic, dermatological, and other topical glucocorticoids are permitted;

    - Cytotoxic, antineoplastic, or immunosuppressant drugs - within 36 months prior to Vaccination (Visit 2 for the Phase 2 portion and Visit 1 for the Phase 3 portion);

    - Any immunoglobulin preparations or blood products, blood transfusion – within 6 months prior to Vaccination (Visit 2 for the Phase 2 portion and Visit 1 for the Phase 3 portion);

6. Study Population #3: Acute disease defined as presence of any moderate or severe acute illness with or without a fever within 48 hours prior to the Screening (Visit 1) and/or Vaccination visit (Visit 2);

7. Administration of any vaccine within 14 days prior to Vaccination (Visit 2 for the Phase 2 portion and Visit 1 for the Phase 3 portion); planned administration of any vaccine during the study (up to Day 28 of the study). Immunization on an emergency basis during the study will be evaluated on case-by-case basis by the Investigator;

8. Administration of any other SARS-CoV-2 / COVID-19, or other experimental coronavirus vaccine at any time prior to or during the study;

9. History of virologically-confirmed COVID-19;

10. Use of any investigational or non-registered product within 30 days or 5 half-lives, whichever is longer, prior to Vaccination (Visit 2 for the Phase 2 portion and Visit 1 for the Phase 3 portion) or planned use during the study period. Subjects who are in a prolonged post-administration observation period of another investigational or marketed drug clinical study, for which there is no ongoing exposure to the investigational or marketed product and all scheduled on-site visits are completed, will be allowed to take part in this study, if all other eligibility criteria are met;

11. Have a rash, dermatological condition, tattoos, muscle mass, or any other abnormalities at injection site that may interfere with injection site reaction rating. Investigator discretion will be permitted with this exclusion criterion;

12. Use of any prescription antiviral drugs with the intention of COVID-19 prophylaxis, including those that are thought to be effective for prevention of COVID-19 but have not been licensed for this indication, within one month prior to Vaccination (Visit 2 for the Phase 2 portion and Visit 1 for the Phase 3 portion);

13. For the Phase 2 portion of the study only: Use of prophylactic medications (e.g., antihistamines [H1 receptor antagonists], nonsteroidal anti-inflammatory drugs [NSAIDs], systemic and topical glucocorticoids, non-opioid and opioid analgesics) within 24 hours prior to the Vaccination (Visit 2) to prevent or pre-empt symptoms due to vaccination;

14. History of a serious allergic response to any of the constituents of CoVLP including AS03;

15. History of a documented anaphylactic reactions to plants or plant components (including tobacco, fruits and nuts);

16. Personal or family (first-degree relatives) history of narcolepsy;

17. Subjects with a history of Guillain-Barré Syndrome;

18. Study Population #1 and #3: Any female subject who has a positive or doubtful pregnancy test result prior to vaccination or who is lactating;

This document is confidential.

19. Subjects identified as an Investigator or employee of the Investigator or clinical site with direct involvement in the proposed study, or identified as an immediate family member (i.e., parent, spouse, natural or adopted child) of the Investigator or employee with direct involvement in the proposed study, or any employees of Medicago.

3.3. Determination of Sample Size

Phase 2 portion:

The sample size of up to 918 subjects will serve to confirm vaccine immunogenicity in the two Study Populations and detect sizable differences in rates of adverse events. The sample size is not large enough to detect less frequent or rare adverse reactions. With 765 subjects receiving the CoVLP formulation, there will be at least 95 % probability of observing an adverse event that occurs as frequently as 1 in 255 vaccinees.

Phase 3 portion:

The sample size of up to 30 000 subjects has been calculated using an exact conditional Poisson approach based on the following assumptions:

- A 1:1 ratio for CoVLP formulation:placebo;
- Assumed vaccine efficacy of the CoVLP formulation, compared to placebo, of 60 %;
- At least 90% statistical power to reject the null hypothesis of 1) a lower limit of the 95% confidence interval for a vaccine efficacy of the CoVLP formulation compared to placebo ≤ 30% and 2) true efficacy < 50%, assuming a vaccine efficacy of 60%;
- A SARS-CoV-2 attack rate (i.e., symptomatic disease) in subjects who receive placebo of 0.8 % across all study populations;
- An attrition of 5 %;

With 15 000 subjects receiving the CoVLP formulation, there will be at least 95 % probability of observing an adverse event that occurs as frequently as 1 in 5000 vaccinees.

3.4. Treatment Assignment and Blinding

In the Phase 2 portion, subjects will be randomized 5:1 to receive the CoVLP formulation and placebo in each Study Population. In the Phase 3 portion, subjects will be randomized 1:1 to receive the CoVLP formulation and placebo in each Study Population. Randomization of elderly adult subjects (Study Population #2) will be stratified by age in an approximately 2:1 ratio of 65 to 74 years of age and 75 years of age and older, respectively. During Phase 2 and Phase 3 portions of the study, eligible subjects will be randomized at the Day 0 visit using the interactive response technology (IRT) system.

Both Phase 2 and Phase 3 portions of the study will be observer-blinded.

From the start of the Phase 2 and Phase 3 portions up to IOA/EUA, the following individuals will not have access to treatment allocation (i.e. remain “blind”): the subjects, the Investigators

and all personnel involved in the clinical conduct of the study (except the staff involved in the preparation and administration of the study vaccine, the quality assurance auditor, and quality control reviewers), Medicago clinical staff and medical staff involved in safety evaluations (e.g. causality assessments), and all personnel involved in sample analysis at the central and testing (Nab, ELISA, ELISpot, ICS [flow cytometry], serological, and central lab virologic method SARS-CoV-2 diagnostics) laboratories. Once IOA/EUA is obtained, the Phase 2 and Phase 3 portions will be unblinded.

During the study, the IDMC and unblinded team, compromised of the unblinded medical monitor, unblinded biostatistician, unblinded senior reviewer, and unblinded programmer involved in the preparation of the safety data summary for the IDMC reviews, will have access to or be aware of treatment allocation (i.e., be "unblinded"). The IDMC will review safety data and will make recommendations for the entire duration of the study. The same unblinded statistical team will be involved in the Day 21 and Day 42 (if needed) immunogenicity data analyses in the Phase 2 portion and in the PVE analyses in the Phase 3 portion.

From the start of the Phase 2 and Phase 3 portions up to IOA/EUA, the Sponsor's Study Team will remain blinded. A small number of Medicago personnel (not included in Study Team) will be involved in the planned analyses during the Phase 2 and Phase 3 portions. During the PVE analyses in the Phase 3 portion, some Medicago personnel will be unblinded at the group level to support the review of the analyses however these individuals will NOT be unblinded at the treatment allocation level. The selected individuals and the process and level of unblinding will be documented.

In order to efficiently manage IP inventory at sites in the Phase 3 portion, a Medicago Unblinded Clinical Supply Manager will have access to site inventory and may therefore be exposed to subject level treatment allocation. The unblinded role of this individual will be documented and the individual will be trained to maintain the study blind. The Unblinded Clinical Supply Manager will not have access to study outcomes such as COVID-19 test results.

Since the CoVLP formulation will have a different physical appearance and viscosity from the placebo, the site staff involved in the preparation and administration of the treatments will not be involved in any activity that could introduce a bias, such as the evaluations of reactogenicity or AEs experienced by the subjects following vaccination.

Any code break will be documented and reported to Medicago (or its designee) in a timely manner. In a medical emergency, the investigator may unblind the treatment for that subject without prior consultation with the Sponsor. In such an event, the Investigator will need to contact the responsible Medical Monitor as soon as possible after the unblinding to discuss the case. The treatment allocation should not be disclosed by the Investigator to the responsible Medical Monitor.

As approved or authorized vaccines for COVID-19 become publicly available to subjects in some regions, subjects may request to become prematurely unblinded. In order to proceed with

any such requests for unblinding in the Phase 2 or Phase 3 portion, the following two criteria must be met:

1. Subject is eligible for an approved or authorized COVID-19 vaccine in their local region, and

2. Subject has been approached or received notification by a healthcare provider to receive a publicly available approved or authorized COVID-19 vaccine. The notification must be shared with the Investigator.

If the subject fulfils the requirements of these two criteria, then the date of premature unblinding will be captured in the IRT system. If the subject does not meet both criteria, he/she will not be eligible for premature unblinding. Details regarding the subject's status in the study are included in Section 3.4.1.

Serological testing performed/requested by study subjects or performed as part of any medical procedures (e.g., blood/plasma donation) that may result in treatment unblinding is not permitted.

3.4.1. Premature Unblinding Due to Availability of Approved or Authorized COVID-19 Vaccines Prior to IOA/EUA

As discussed in Section 3.4, some subjects may request to become prematurely unblinded in order to receive an approved or authorized vaccine for COVID-19 when it becomes publicly available to them in their region. If subjects meet the criteria outlined in Protocol Section 6.2, they will be prematurely unblinded to the treatment they have received. The subject's status in the study will be dependent on the treatment received and whether the subject actually receives the approved or authorized COVID-19 vaccine (refer to Figure 1).

If the subject received the CoVLP formulation and did not receive the approved or authorized COVID-19 vaccine, then the subject may remain in the study. If the subject received placebo or received the CoVLP formulation and the approved or authorized COVID-19 vaccine, then the subject will be withdrawn/discontinued from the study. Subjects will be asked to complete the final telephone contact within two weeks of withdrawal/discontinuation, if permitted by the subject, after they have received the approved or authorized COVID-19 vaccine. The date the approved or authorized COVID-19 vaccine was received by the subject must be documented in the EDC.

In the Phase 3 portion, if a subject requests to be prematurely unblinded before the completion of their Day 21 visit and does not receive the approved or authorized COVID-19 vaccine, then the unblinded subject will be eligible to receive their second study vaccination at the Day 21. Subjects will still be assessed for any contraindications for the second study vaccinations.

Subjects who receive the approved or authorized COVID-19 vaccine prior to IOA/EUA will be asked to complete the final telephone contact at the time of unblinding and prior to receipt of the approved or authorized COVID-19 vaccine, if permitted by the subject.

Figure 1 Decision Tree for Possible Premature Unblinding in Phase 2 and Phase 3 Portions Prior to IOA/EUA

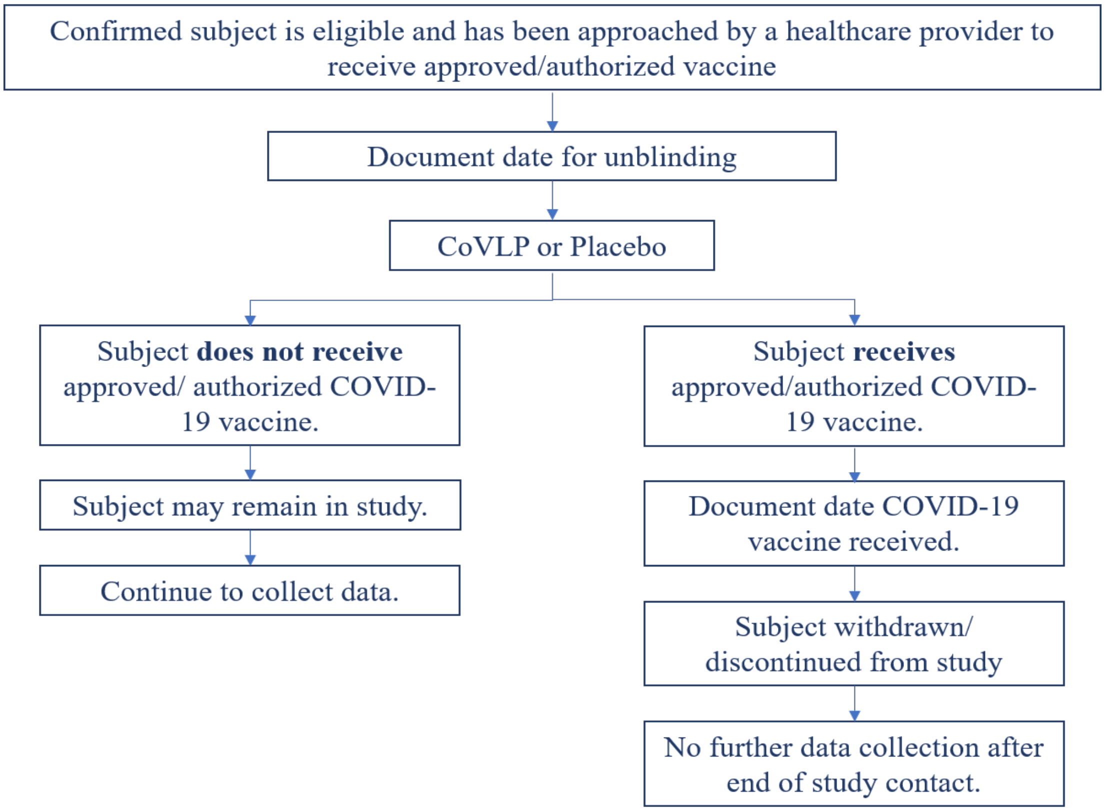

3.5. Administration of Study Medication

The CoVLP formulation for the Phase 2 and Phase 3 portion of the study has been selected based on the Phase 1 study data, including acceptable safety and immunogenicity profiles. This Phase 2 portion is confirmatory for the dose to be used in each population for Phase 3.

In the Phase 2 proportion, subjects will receive two IM injections 21 days apart on Days 0 and 21, into the deltoid region of the alternating (if possible) arm (i.e., each arm will be injected once), of one of the following:

- CoVLP formulation;
- Placebo.

The use of the same dose of CoVLP for all Study Populations may not be adequate. Subjects in Study Population #1 (healthy adults 18 to 64 years of age) will receive the standard dose of CoVLP (3.75 µg) adjuvanted with AS03 in an injection volume of 0.5 mL.

Study Populations #2 and #3 (elderly adults or adults with significant comorbidities, respectively) will receive the standard dose of CoVLP (3.75 µg) adjuvanted with AS03 in an

injection volume of 0.5 mL. If an acceptable immune response is not achieved at either Day 21 or Day 42, the IDMC may recommend a dose escalation to 7.5 µg CoVLP adjuvanted with AS03 in a volume of 0.5 mL, and the same number of subjects (i.e., up to 306 subjects per Study Population) in the particular Study Population will be recruited, using the same enrollment approach (i.e., use of sentinel subjects and IDMC reviews). If the higher dose of the CoVLP formulation achieves an acceptable immune response in the Phase 2 portion, then the same higher dose will be used in that particular Study Population during the Phase 3 portion of the study.

The start of the Phase 3 portion of the study will be determined independently for the Study Population and will be based on the outcomes of the following assessments during the Phase 2 portion of the study:

- Day 21 Data Analysis of the immunogenicity data in Study Population #1;
- Day 42 Data Analysis of the immunogenicity data in Study Populations #2 and Study Population #3;
- Day 28 Safety Review in Study Population #1, Study Population #2 and Study Population #3.

In the Phase 3 portion, each Study Population will receive the same dose of CoVLP adjuvanted with AS03 and injection volume established for that particular Study Population during the Phase 2 portion of the study.

Subjects will receive two IM injections 21 days apart on Days 0 and 21, into the deltoid region of the alternating (if possible) arm (i.e., each arm will be injected once), of either the CoVLP formulation or the placebo. Subjects in the placebo group will be offered the CoVLP formulation once IOA/EUA is obtained.

Upon obtaining the IOA/EUA for the CoVLP formulation, the study will be unblinded to support the following:

1. Subjects in the placebo group will be offered the CoVLP formulation and will only be followed passively to identify symptomatic COVID-19 cases and for safety until the end of the study. A blood sample will be obtained immediately prior to the administration of the first dose of the CoVLP formulation but no further blood samples will be collected in this group;

2. Subjects in the CoVLP formulation group will complete the study as planned with a blood draw at Day 201 to assess protection against serologically-confirmed COVID-19.

Subjects in the placebo group who do not want to be administered the CoVLP formulation and still want to remain in the study will continue the study as planned with a blood draw at Day 201. Upon obtaining the IOA/EUA, the CoVLP formulation will be offered to placebo subjects in the Phase 2 portion of the study. Once the placebo subject receives the CoVLP formulation, the subject will only be followed for safety until the end of the study and no further blood draws will

be collected. Subjects in the placebo group who do not wish to receive the CoVLP formulation or subjects who are in the CoVLP formulation group will continue the study as planned.

Subjects in the CoVLP formulation group will be followed for efficacy (i.e., active and passive surveillance) and safety until IOA/EUA and then for COVID-19 cases (i.e., passive surveillance only) and safety until the end of the study (Day 386). Subjects in the placebo group will be followed for efficacy (i.e., active and passive surveillance) and safety up until IOA/EUA and followed for safety and COVID-19 cases (i.e., passive surveillance only) until the end of the study. The sero-status of all subjects will be assessed at the time of enrollment. Serum samples will be obtained from all subjects at Day 42 so that a possible serologic correlate of protection can be evaluated once the primary efficacy outcome has been met and at later timepoints in the study. Only the subjects who initially receive the CoVLP formulation or placebo subjects who do not want the CoVLP formulation among the 288 subjects (96 subjects from each Study Population) in the immunogenicity subset will be assessed for the persistence of the vaccine-induced cellular immune responses until Day 386.

3.6. Study Procedures and Flowchart

Table 3 Time and Events Schedule: General Information for the Phase 2 Portion

**Visit Type**	**Screening**	**Vaccination**	**Post-vaccination Visits/Contacts**											
**Study Day**	**Day -14 to 0**	**Day 0**	**Day 1 (+ 1)**	**Day 3 (± 1)**	**Day 8 (± 1)**	**Day 21 (± 2)[8]**	**Day 22 (+ 1)**	**Day 24 (± 1)**	**Day 29 (± 1)**	**Day 42 (± 2)**	**Day 128 (± 14)**	**Day 201 (± 14)[10]**	**Day 386 (± 14)[11]**	**Unscheduled Contact for Unblinding[12]**
**Visit Number**	**1**	**2**	**Phone**	**3**	**Phone**	**4**	**Phone**	**5**	**Phone**	**6**	**7**	**8**	**9**	**Phone**
Informed consent	X													
Demographics	X													
Height, weight, and BMI	X	X												
Medical history/prior medication	X	X[1]												
Physical examination	X[2]													
Vital Signs	X	X[3]		X		X[3]		X		X	X	X	X	
Inclusion/exclusion criteria	X	X												
Urinalysis	X			X		X[9]		X						
Pregnancy test [4]	X	X[9]				X[9]				X				
Test for SARS-CoV-2 antibodies	X													
Serology tests for HIV, Hepatitis B, and Hepatitis C	X													
Blood chemistry And Haematology	X			X		X[9]		X						
Immunogenicity -Serology (Nab assay and ELISA)		X[9]				X[9]				X	X	X	X	

**Visit Type**	**Screening**	**Vaccination**	**Post-vaccination Visits/Contacts**											
**Study Day**	**Day -14 to 0**	**Day 0**	**Day 1 (+ 1)**	**Day 3 (± 1)**	**Day 8 (± 1)**	**Day 21 (± 2)[8]**	**Day 22 (+ 1)**	**Day 24 (± 1)**	**Day 29 (± 1)**	**Day 42 (± 2)**	**Day 128 (± 14)**	**Day 201 (± 14)[10]**	**Day 386 (± 14)[11]**	**Unscheduled Contact for Unblinding[12]**
**Visit Number**	**1**	**2**	**Phone**	**3**	**Phone**	**4**	**Phone**	**5**	**Phone**	**6**	**7**	**8**	**9**	**Phone**
Immunogenicity – CMI Response (ELISpot and ICS)		X[9]				X[9]				X		X	X	
Randomization		X												
Vaccine admin		X				X								
Immediate surveillance (30 minutes)		X				X								
Provide and collect diary and memory aid instructions		X		X		X		X		X	X	X	X	
Oral digital thermometer and instructions on reactions[5]		X				X								
Collection of solicited local/ systemic adverse events		X	X	X	X	X	X	X	X					
Concomitant medications[6]			At any time during the study period											
Collection of COVID-19 symptoms through passive and active surveillance			Passive Surveillance: Subjects will be instructed to contact the study site if they experience symptoms of COVID-19 from the day of the first vaccination (Day 0, post vaccination) until the end of the Phase 2 portion of the study. Active Surveillance: Between the day of the first vaccination (Day 0, post vaccination) and the end of the Phase 2 portion of the study, the subjects will be contacted once per week via the electronic diary and asked about symptoms of COVID-19. Other methods of contact (phone, text, email) will only be used as a last resort to contact the subject.											
Testing for laboratory confirmation			Subjects who report any symptoms associated with COVID-19 after the start of the active and passive surveillance will be requested to visit the Investigator site to provide nasal or NP swabs (2 per subject per event) to test											

Visit Type	Screening	Vaccination	Post-vaccination Visits/Contacts											
**Study Day**	**Day -14 to 0**	**Day 0**	**Day 1 (+ 1)**	**Day 3 (± 1)**	**Day 8 (± 1)**	**Day 21 (± 2)[8]**	**Day 22 (+ 1)**	**Day 24 (± 1)**	**Day 29 (± 1)**	**Day 42 (± 2)**	**Day 128 (± 14)**	**Day 201 (± 14)[10]**	**Day 386 (± 14)[11]**	**Unscheduled Contact for Unblinding[12]**
**Visit Number**	**1**	**2**	**Phone**	**3**	**Phone**	**4**	**Phone**	**5**	**Phone**	**6**	**7**	**8**	**9**	**Phone**
of SARS-CoV-2 infection			for COVID-19. If the subject cannot visit the Investigator site, then the subject may receive a visit at home to collect the nasal swabs samples or be provided with self-administered nasal/NP swabs for sample collection. The swabs will be collected within 72 hours (preferably within 48 hours) of the reporting of a COVID-19-associated symptom. If swabs cannot be collected within 72 hours after reporting of the COVID-19-associated symptom, nasal or NP swabs should be collected at the next available opportunity for the subject to visit the Investigator site.											
AEs, SAEs, MAAEs, and AESIs[7]			At any time during the study period.											
Termination record													X	

[1] Record any changes in medical history and medications and confirmation that the subject continues to meet all inclusion and no exclusion criteria since screening.

[2] A limited physical examination will occur at screening. History/symptom-directed physical examinations may be performed at other visits if 1) new complaints or concerns are raised by either the study subject or study staff and 2) deemed to be necessary by the Investigator.

[3] Record prior to study vaccine, after the observation period and as deemed necessary.

[4] In all females of childbearing potential in Study Population #1 and #3; it will be tested in serum at screening and in urine at Days 0, 21, and 42.

[5] After vaccination, subjects will be instructed on the diary and memory aid provided for their use for recording reactions, AEs, and concomitant medication use.

[6] After the Day 42 visit, concomitant medication collection will be limited to those used to treat an SAE, MAAE, AE leading to withdrawal, AESIs, or an AE that occurred before Day 42; any vaccine not foreseen in the study protocol; and prohibited medications.

[7] AEs will be collected up to Day 42; SAEs, MAAEs, AEs leading to withdrawal, and AESIs will be collected through to the end of the study. Investigators should report SAEs, AESIs, and pregnancy reports to Sponsor within 24 hours of the Investigator learning of the event. Specific contacts for the collection of information regarding all of these events will occur on Day 386 (during the Day 386 final visit) for SAEs, MAAEs, AEs leading to withdrawal, and AESI.

[8] All efforts will be done to have subjects returning on planned date for Day 21 activities. If for any reason the visit is done before or after this planned date, subsequent visits/procedures (Days 22, 24, 29, 42, 128, 201, and 386) will be adjusted accordingly.

[9] Blood and urine samples are to be collected prior to vaccination on the respective day.

[10] If IOA/EUA occurs prior to Day 201, blood samples on Day 201 will not be collected from subjects in the placebo group who receive the CoVLP formulation post-IOA/EUA. These subjects will follow the Table 3: Time and Events Schedule once they agree to receive the CoVLP formulation post-IOA/EUA.

This document is confidential.

[11] If IOA/EUA occurs prior to Day 386, blood samples on Day 386 will not be collected from subjects in the placebo group who receive the CoVLP formulation post-IOA/EUA. These subjects will follow the Table 3: Time and Events Schedule once they agree to receive the CoVLP formulation post-IOA/EUA.
[12] Once IOA/EUA is obtained, subjects in the study will be contacted by phone (or any other method of communication such as the electronic diary, text, or email) and informed of the treatment they received in the study. Subjects in the placebo group will be offered the CoVLP formulation. Subjects in the placebo group who receive the CoVLP formulation (post-IOA/EUA) will follow the Table 3: Time and Events Schedule. Subjects in the placebo group who do not want the CoVLP formulation and remain in the study will continue to follow the Table 1: Time and Events Schedule.

Table 4 Time and Events Schedule: General Information for the Phase 3 Portion

**Visit Type[12]**	**Screening / Vaccination**	**Post-vaccination Visits/Contacts**				
**Study Day**	**Day 0**	**Day 21 (± 2)[8]**	**Day 42 (± 2)[10]**	**Day 201 (± 14)[12]**	**Day 386 (± 14)[13]**	**Unschedule Contact for Unblinding[14]**
**Visit Number**	**1**	**2**	**3**	**4**	**5/Phone**	**Phone**
Informed consent	X					
Demographics	X					
Height, weight, and BMI	X					
Medical history/prior medication	X					
Physical examination	X[2]					
Vital Signs	X[3]	X[3]				
Inclusion/exclusion criteria	X					
Pregnancy test[4]	X[9]	X[9]	X			
Randomization	X					
Test for SARS-CoV-2 N antibodies	X[9]			X		
Immunogenicity - Serology (Nab assay only)	X[9,10]		X[10]			
Immunogenicity -Serology (Nab assay and ELISA)[1]	X[9]	X[9]	X	X	X	
Immunogenicity – CMI Response (ELISpot and ICS)[1]	X[9]	X[9]	X	X	X	
Vaccine admin	X	X				
Immediate surveillance (30 minutes)	X	X				
Provide and collect diary and memory aid instructions[13]	X	X	X	X	X	
Oral digital thermometer and instructions on reactions[5]	X	X				
Collection of solicited local/ systemic adverse events	X	X				
Concomitant medications[6]	At any time during the study.					
Collection of COVID-19 symptoms through passive and active surveillance	Passive Surveillance: Subjects will be instructed to contact the study site if they experience symptoms of COVID-19 from the day of the first vaccination (Day 0, post vaccination) until the end of the Phase 3 portion of the study.<br>Subjects will also be advised to inform the clinical site if they have tested positive for SARS-CoV-2 infection, with or without the					

**Visit Type[12]**	**Screening / Vaccination**	**Post-vaccination Visits/Contacts**				
**Study Day**	**Day 0**	**Day 21 (± 2)[8]**	**Day 42 (± 2)[10]**	**Day 201 (± 14)[12]**	**Day 386 (± 14)[13]**	**Unschedule Contact for Unblinding[14]**
**Visit Number**	**1**	**2**	**3**	**4**	**5/Phone**	**Phone**
	presence of any symptoms associated with COVID-19, and will be asked to provide a documented test result.<br>Active Surveillance: Between the day of the first vaccination (Day 0, post vaccination) and when IOA/EUA is granted for the CoVLP formulation, the subjects will be contacted once per week via the electronic diary and asked about symptoms of COVID-19. Other methods of contact (phone, text, email) will only be used as a last resort to contact the subject.					
Testing for laboratory confirmation of SARS-CoV-2 infection	Subjects who report any symptoms associated with COVID-19 after the start of the active and passive surveillance will be requested to visit the Investigator site to provide nasal or NP swabs to test for COVID-19. During the visit, the Investigator will confirm whether nasal or NP swabs should be collected. If the subject cannot visit the Investigator site, then the subject may receive a visit at home (or hospital, if applicable) to collect the nasal/NP swabs samples or be provided with self-administered nasal swabs for sample collection. During the 7 days following each vaccination, Investigator judgement will be used to determine whether swabs should be collected from subjects reporting symptoms similar to those associated with COVID-19 since such symptoms may also be due to the reactogenicity of the study treatment administered rather than SARS-CoV-2 infection.<br>The swabs will be collected within 72 hours (preferably within 48 hours) of the reporting of a COVID-19-associated symptom. If swabs cannot be collected within 72 hours after reporting of the COVID-19-associated symptom, nasal or NP swabs should be collected at the next available opportunity for the subject to visit the Investigator site or at home (or hospital, if applicable). Two swabs per event per subject will be collected and submitted for analysis using a virologic method, at the central virology laboratory. To mitigate the risk of long delays in shipping samples to the central virology laboratory, an additional local virologic test will be performed.<br>The purpose of this supplementary test is to provide a preliminary diagnosis and permit appropriate symptom-recording and sample collection as quickly as possible.<br>All subjects with virologically-confirmed SARS-CoV-2 infection, based on results from the locally performed testing, will be asked to provide serial (every other day) self-administered nasal swabs (2 swabs every other day for the central virology laboratory) for approximately 2 weeks. In the event, the COVID-19-associated symptoms persist for more than 2 weeks, then the Investigator's clinical judgement is required to extend the collection of swabs beyond the 2 weeks from the subject. If the COVID-19-associated symptoms have resolved in less than 2 weeks and the Investigator believes it is likely the subject has virologically recovered, then two					

**Visit Type[12]**	**Screening / Vaccination**	**Post-vaccination Visits/Contacts**				
**Study Day**	**Day 0**	**Day 21 (± 2)[8]**	**Day 42 (± 2)[10]**	**Day 201 (± 14)[12]**	**Day 386 (± 14)[13]**	**Unschedule Contact for Unblinding[14]**
**Visit Number**	**1**	**2**	**3**	**4**	**5/Phone**	**Phone**
	consecutive negative results, obtained locally, are required prior to stopping the collection of the self-administered swabs for the purpose of testing at the central laboratory.<br>At the same visit where the Investigator will confirm whether nasal or NP swabs should be collected, subjects will be trained on daily recording of their COVID-19-associated symptoms using a COVID-19 diary and will be asked to immediately start recording their symptoms in the COVID-19 diary. Subjects will collect any COVID-19-associated symptoms in their COVID-19 diary until a negative swab result for the subject is received from the central virology laboratory or until the subject has no more COVID-19-associated symptoms.					
AEs, SAEs, MAAEs, and AESIs[7]	At any time during the study.					
Termination record					X	

[1] These samples will only be collected from the subjects included in the immunogenicity subset.

[2] A limited physical examination will occur at screening. History/symptom-directed physical examinations may be performed at other visits if 1) new complaints or concerns are raised by either the study subject or study staff and 2) deemed to be necessary by the Investigator.

[3] Record prior to study vaccine, and as deemed necessary.

[4] In all females of childbearing potential in Study Populations #1 and #3; it will be tested in urine at Days 0, 21, and 42.

[5] After vaccination, subjects will be instructed on the diary and memory aid provided for their use for recording reactions, AEs, and concomitant medication use.

[6] After the Day 42 visit, concomitant medication collection will be limited to those used to treat an SAE, MAAE, AE leading to withdrawal, AESIs, or an AE that occurred before Day 42; any vaccine not foreseen in the study protocol; and prohibited medications.

[7] AEs will be collected up to Day 42; SAEs, MAAEs, AEs leading to withdrawal, and AESIs will be collected through to the end of the study. Investigators should report SAEs, AESIs, and pregnancy reports to Sponsor within 24 hours of the Investigator learning of the event. Specific contacts for the collection of information regarding all of these events will occur on Day 386 for SAEs, MAAEs, AEs leading to withdrawal, and AESI.

[8] All efforts will be done to have subjects returning on planned date for Day 21 activities. If for any reason the visit is done before or after this planned date, subsequent visits/contacts (Days 42, 201, and 386) will be adjusted accordingly.

[9] Blood and urine (pregnancy test) samples are to be collected prior to vaccination on the respective day.

[10] An immunogenicity-serology (Nab assay only) sample at Day 0 and Day 42 will be collected from all subjects.

[11] During the first 8 days after each vaccination (prior to IOA/EUA), if data entry in the electronic diary is missed for 2 consecutive days by a subject then the subject will receive a follow-up contact (phone, text, or email) to remind him/her to record his/her data in the electronic diary.

[12] If IOA/EUA occurs prior to Day 201, blood samples on Day 201 will not be collected from subjects in the placebo group who receive the CoVLP formulation post-IOA/EUA. These subjects will follow the Table 3: Time and Events

Schedule once they agree to receive the CoVLP formulation post-IOA/EUA.[13] If IOA/EUA occurs prior to Day 386, blood samples on Day 386 will not be collected from subjects in the placebo group who receive the CoVLP formulation post-IOA/EUA. These subjects will follow the Table 3: Time and Events Schedule once they agree to receive the CoVLP formulation post-IOA/EUA.

[14] Once IOA/EUA is obtained, subjects in the study will be contacted by phone (or any other method of communication such as the electronic diary, text, or email) and informed of the treatment they received in the study. Subjects in the placebo group will be offered the CoVLP formulation. Subjects in the placebo group who receive the CoVLP formulation (post-IOA/EUA) will follow the Table 3: Time and Events Schedule. Subjects in the placebo group who do not want the CoVLP formulation and remain in the study will continue to follow the Table 2: Time and Events Schedule.

Table 4.1 Time and Events Schedule: General Information for Placebo Subjects who will Receive the CoVLP Formulation post-IOA/EUA in the Phase 2 and 3 Portions

**Visit Type**	**Vaccination**		**Post-vaccination Contacts**	
**Study Day**	**Post-IOA/EUA**	**21 Days After First Vaccination (± 2)**	**Monthly Calls (± 14)[5]**	**Day 386 (± 14)**
**Visit Number**	**1**	**2**	**Phone**	**Phone**
Contraindications assessment[1]	X			
Urine Pregnancy test[2]	X	X		
Test for SARS-CoV-2 N antibodies[6]	X[7]			
Vaccine admin	X	X		
Immediate surveillance (30 minutes)	X	X		
Provide and collect memory aid instructions	X	X		X
Concomitant medications[3]	At any time during the study period			
Collection of COVID-19 symptoms through passive	Passive Surveillance: Subjects will be instructed to contact the study site if they experience symptoms of COVID-19 from the day of the first vaccination of the CoVLP formulation (post vaccination) until the end of the Phase 3 portion of the study.<br>Subjects will also be advised to inform the clinical site if they have tested positive for SARS-CoV-2 infection, with or without the presence of any symptoms associated with COVID-19, and will be asked to provide a documented test result.			
Testing for laboratory confirmation of SARS-CoV-2 infection	Subjects who report any symptoms associated with COVID-19 after the start of the active and passive surveillance will be requested to visit the Investigator site to provide nasal or NP swabs to test for COVID-19. During the visit, the Investigator will confirm whether nasal or NP swabs should be collected. If the subject cannot visit the Investigator site, then the subject may receive a visit at home (or hospital, if applicable) to collect the nasal/NP swabs samples or be provided with self-administered nasal swabs for sample collection. During the 7 days following each vaccination, Investigator judgement will be used to determine whether swabs should be collected from subjects reporting symptoms similar to those associated with COVID-19 since such symptoms may also be due to the reactogenicity of the study treatment administered rather than SARS-CoV-2 infection.<br>The swabs will be collected within 72 hours (preferably within 48 hours) of the reporting of a COVID-19-associated symptom. If swabs cannot be collected within 72 hours after reporting of the COVID-19-associated symptom, nasal or NP swabs should be collected at the next available opportunity for the subject to visit the Investigator site or at home (or hospital, if applicable). Two swabs per event per subject will be collected and submitted for analysis using a virologic method, at the central virology laboratory. To mitigate the risk of long delays in shipping samples to the central virology laboratory, an additional local virologic test will be performed. The purpose of this supplementary test is to provide a preliminary diagnosis and permit appropriate symptom-recording and sample collection as quickly as possible.<br>All subjects with virologically-confirmed SARS-CoV-2 infection, based on results from the locally performed testing, will be asked to provide serial (every other day) self-administered nasal swabs (2 swabs every other day for the central virology laboratory) for approximately 2 weeks. In the event, the COVID-19-associated symptoms persist for more than 2 weeks, then the Investigator's clinical judgement is required to extend the collection of swabs beyond the 2 weeks from the subject. If the COVID-19-associated symptoms have resolved in less than 2 weeks and the Investigator believes it is likely the subject has virologically recovered, then two consecutive negative results, obtained locally, are required prior to stopping the collection of the self-administered swabs for the purpose of testing at the central laboratory.			

This document is confidential.

**Visit Type**	**Vaccination**		**Post-vaccination Contacts**	
**Study Day**	**Post-IOA/EUA**	**21 Days After First Vaccination (± 2)**	**Monthly Calls (± 14)[5]**	**Day 386 (± 14)**
**Visit Number**	**1**	**2**	**Phone**	**Phone**
	At the same visit where the Investigator will confirm whether nasal or NP swabs should be collected, subjects will be trained on daily recording of their COVID-19-associated symptoms using a COVID-19 diary and will be asked to immediately start recording their symptoms in the COVID-19 diary. Subjects will collect any COVID-19-associated symptoms in their COVID-19 diary until a negative swab result for the subject is received from the central virology laboratory or until the subject has no more COVID-19-associated symptoms.			
AEs, SAEs, MAAEs, and AESIs[4]	At any time during the study			
Termination record				X

[1] This visit will consist of assessing subjects for contraindications to the first CoVLP formulation vaccination.

[2] In all females of childbearing potential in Study Populations #1 and #3. Urine (pregnancy test) samples are to be collected prior to vaccination on the respective day.

[3] Forty-two (42) days after the first CoVLP formulation vaccination, concomitant medication collection will be limited to those used to treat an SAE, MAAE, AE leading to withdrawal, AESIs, or an AE that occurred before 42 days post-vaccination; any vaccine not foreseen in the study protocol; and prohibited medications.

[4] AEs will be collected up to 42 days after the first CoVLP formulation vaccination; SAEs, MAAEs, AEs leading to withdrawal, and AESIs will be collected through to the end of the study. Investigators should report SAEs, AESIs, and pregnancy reports to Sponsor within 24 hours of the Investigator learning of the event. Specific contacts for the collection of information regarding all of these events will occur on Day 386 for SAEs, MAAEs, AEs leading to withdrawal, and AESI.

[5] Subjects should be reached once a month with no more than 45 days between phone contacts (use the second vaccination date as starting reference or last day of contact with the clinic site or Investigator if a second vaccination did not occur).

[6] This blood sample will only be collected from subjects in the Phase 3 portion. If IOA/EUA occurs post-Day 201, then no blood sample will be collected since a blood sample to test for SARS-CoV-2 N antibodies will be collected on Day 201.

[7] Blood sample is to be collected prior to vaccination.

Table 5 Time and Events Schedule: Onset of COVID-19

**Days after Reporting of COVID-19[3]:**	**0-3 Days**	**0-3 Days[1]**	**Monitoring[2]**
**Contact Type**	**Phone**	**Visit**	**Phone / Text / Email Messaging / Electronic Diary**
Verify information on COVID-19 and confirm swab collection within 72 hours (preferably within 48 hours) of the reporting of a COVID-19 symptom. Swab collection will be done at the discretion of the site Investigator.	X		
Remind subject to continue to record data and in a timely manner	X		
Collection of information on COVID-19 symptoms	X	X	X

[1] Nasal or NP swab collection is to be done within 72 hours after reporting of the COVID-19 symptoms (preferably within 48 hours), at the discretion of the site Investigator, and prior to the use of any antiviral treatment medication. If swab collection cannot be done within 72 hours after reporting of the COVID-19 symptoms, swabs should be collected at the next available opportunity for the subject to visit the Investigator site or at home (or hospital, if applicable) and date and time of collection recorded.
[2] After swab collection is done, the planned active surveillance (phone, text, email, electronic diary) will continue and will include questions on the progression of COVID-19.
[3] Swab collection will be done for any subject who reports any COVID-19-associated symptom after the start of the active and passive surveillance, at the discretion of the site Investigator.

4. Endpoints

4.1. Efficacy Endpoints

To mitigate the risk of long delays in shipping samples to the central virology laboratory, an additional local virologic test will be performed. The purpose of this supplementary test is to provide a preliminary diagnosis and permit appropriate symptom-recording and sample collection as quickly as possible. The virologic confirmation mentioned below refers to central laboratory testing of samples for SARS-CoV-2.

Following the first vaccination, each subject will be asked to report symptoms associated with COVID-19 from the day of the first vaccination (Day 0, post vaccination) until the end of the Phase 2 portion or the Phase 3 portion of the study (through active or passive surveillance). COVID-19 will be defined as the presence of a laboratory-confirmed (virologic method) SARS-CoV-2 infection with the occurrence of a new onset of one or more of the following symptoms:

- Fever or chills;
- Cough;
- Shortness of breath or difficulty breathing;
- Fatigue;
- Muscle or body aches;
- Headache;
- New loss of taste or smell;
- Sore throat;
- Congestion or runny nose;
- Nausea or vomiting;
- Diarrhea.

4.1.1. Primary Endpoints

In the Phase 3 portion, the primary efficacy endpoint is:

- First occurrence (as defined by date of symptom start), in a subject, of laboratory-confirmed (virologic method) symptomatic SARS-CoV-2 infection (≥ 7 days post-second vaccination) at the time of the PVE analysis.

This document is confidential.

4.1.2. Secondary Endpoints

In the Phase 2 portion, the secondary efficacy endpoints are:

- First occurrence, in a subject, of laboratory-confirmed (virologic method) symptomatic SARS-CoV-2 infection (≥ 7 days post-second vaccination);
- Occurrences of severe COVID-19 disease (≥ 7 days post-second vaccination).

In the Phase 3 portion, the secondary efficacy endpoints are:

- Occurrences of laboratory-confirmed asymptomatic SARS-CoV-2 infection at Day 201 in subjects who were seronegative at baseline (confirmed by the ELISA method for the N protein);
- Occurrences of severe COVID-19 disease (≥ 7 days post-second vaccination) at the time of the PVE analysis;
- Occurrence and intensity of COVID-19-related symptoms in virologically-confirmed cases up until resolution of the symptoms in subjects administered the CoVLP formulation compared to subjects administered the placebo at the time of the PVE analysis;
- First occurrence, in a subject, of laboratory-confirmed (virologic method) symptomatic SARS-CoV-2 infection (≥ first vaccination) at the time of the PVE analysis;
- First occurrence, in a subject, of laboratory-confirmed (virologic method) symptomatic SARS-CoV-2 infection (≥ first vaccination and < second vaccination) at the time of the PVE analysis;
- First occurrence, in a subject, of laboratory-confirmed (virologic method) symptomatic SARS-CoV-2 infection (≥ second vaccination and < 7 days post-second vaccination) at the time of the PVE analysis;
- Duration and intensity of viral shedding after virologically-confirmed SARS-CoV-2 infection at the time of the PVE analysis;
- First occurrence, in a subject, of laboratory-confirmed (virologic method) symptomatic SARS-CoV-2 infection (≥ 7 days post-second vaccination) by strain at the time of the PVE analysis.

4.1.3. Exploratory Endpoints

In the Phase 2 portion, the exploratory efficacy endpoint is:

- Occurrence and intensity of COVID-19-related symptoms up to resolution of the symptoms.

In the Phase 3 portion, the exploratory efficacy endpoints is:

- Occurrences of severe COVID-19 disease (≥ 7 days post-second vaccination) by strain.

4.2. Immunogenicity Endpoints

Point estimates and 95 % CI will be calculated for all immunogenicity endpoints and responses for the treatment groups will be compared using descriptive statistics.

4.2.1. Primary Endpoints

In the Phase 2 portion, the primary immunogenicity endpoints are:

- Nab response induced in each Study Population against the SARS-CoV-2 virus on Days 0, 21, and 42 will be analyzed using the following parameter: GMT, seroconversion (SC) rate, and GMFR;
- Specific Th1 CMI response induced in each Study Population against the SARS-CoV-2 virus on Days 0, 21, and 42, as measured by IFN-γ ELISpot.

4.2.2. Secondary Endpoints

In the Phase 2 portion, the secondary immunogenicity endpoints are:

- Relative neutralizing antibody response between the healthy adults (Study Population #1) and the healthy elderly population (Study Population #2; each age strata) induced at 21 days after the second vaccination will be analyzed using the following parameter: GMT;
- Relative neutralizing antibody response for the combination of healthy adults (Study Population #1) and the healthy elderly adults (Study Population #2) compared to adults and the elderly adults with significant comorbidities (Study Population #3) induced at 21 days after the second vaccination will be analyzed using the following parameter: GMT;
- Persistence of the Nab antibody response induced in each Study Population against the SARS-CoV-2 virus on Day 128, Day 201, and Day 386 will be analyzed using the following parameters: GMT, SC rate (percentage of subjects achieving seroconversion), and GMFR;
- Specific antibody response induced in each Study Population against the SARS-CoV-2 virus will be measured by the total IgG levels on Days 0, 21, and 42, and the persistence of these antibodies on Days 128, 201, and 386 and analyzed using the following parameters: GMT, SC rate, and GMFR;

- The ratio of neutralizing antibody titers:IgG ELISA antibody titers at Day 21, Day 42, Day 128, Day 201, and Day 386;
- Specific Th1 CMI response induced in each Study Population against the SARS-CoV-2 virus on Day 201 and Day 386, as measured by IFN-γ ELISpot;
- Specific Th2 CMI response induced in each Study Population against the SARS-CoV-2 virus on Days 0, 21, 42, 201, and 386, as measured by IL-4 (ELISpot).

In the Phase 3 portion, the secondary immunogenicity endpoints are:

- In the immunogenicity subset, Nab antibody response induced in each Study Population against the SARS-CoV-2 virus on Days 0, 21, 42, 201, and 386 analyzed, using the following parameters: GMT, SC rate, and GMFR;
- In the immunogenicity subset, specific antibody response induced in each Study Population against the SARS-CoV-2 virus measured by the total IgG levels on Days 0, 21, 42, 201, and 386, and analyzed using the following parameters: GMT, SC rate, and GMFR;
- In the immunogenicity subset, the ratio of neutralizing antibody titers:IgG ELISA antibody titers at Day 21, Day 42, Day 201, and Day 386;
- In the immunogenicity subset, specific Th1 CMI response induced in each Study Population against the SARS-CoV-2 virus on Days 0, 21, 42, 201, and 386, as measured by IFN-γ ELISpot;
- In the immunogenicity subset, specific Th2 CMI response induced in each Study Population against the SARS-CoV-2 virus on Days 0, 21, 42, 201, and 386, as measured by IL-4 (ELISpot).

4.2.3. Exploratory Endpoints

In the Phase 2 portion, the exploratory immunogenicity endpoint is:

Further characterization of the immune response of the CoVLP formulation, if it is likely to be informative.

In the Phase 3 portion only, the exploratory immunogenicity endpoints are:

- In the immunogenicity subset, specific CMI response induced in each Study Population against the SARS-CoV-2 virus up to 21 days after the second vaccination and at Day 201 and Day 386, as measured by the percentage of CD4+ T cells expressing functional markers;
- Further characterization of the immune response of the CoVLP formulation, if it is likely to be informative.

4.3. Safety Endpoints

4.3.1. Primary Endpoints

In the Phase 2 portion, the primary safety endpoints are:

- Occurrence, intensity, and relationship (relationship to be assessed for unsolicited AEs only) to vaccination of immediate AEs (30 minutes after each vaccination);
- Occurrence and intensity of solicited local and systemic AEs (for seven days following each vaccine administration);
- Occurrence, intensity, and relationship of unsolicited AEs for 21 days following each vaccine administration;
- Number and percentage of subjects with normal and abnormal clinically significant urine, haematological and biochemical values prior to and three days following each vaccination;
- Occurrences of SAEs, MAAEs, AEs leading to withdrawal, AESIs (including VAED), and deaths up to 21 days following each vaccine administration.

4.3.2. Secondary Endpoints

In the Phase 2 portion, the secondary safety endpoints are:

- Relative occurrence of solicited local and systemic AEs by intensity grades for seven days following each vaccine administration between the healthy adults (Study Population #1) and the healthy elderly population (Study Population #2, each age strata);
- Relative occurrence of solicited local and systemic AEs by intensity grades for seven days following each vaccine administration for the combination of healthy adults (Study Population #1) and the healthy elderly adults (Study Population #2) compared to adults and the elderly adults with significant comorbidities (Study Population #3);
- Occurrences of SAEs, MAAEs, AEs leading to withdrawal, AESIs (including VAED), and deaths from Day 43 to Day 201;
- Occurrences of SAEs, MAAEs, AEs leading to withdrawal, AESIs (including VAED), and deaths from Day 202 to Day 386.

In the Phase 3 portion, the secondary safety endpoints are:

- Occurrence, intensity, and relationship (relationship to be assessed for unsolicited AEs only) to vaccination of immediate AEs (30 minutes after each vaccine administration);
- Occurrence and intensity of solicited local and systemic AEs (for seven days following each vaccine administration) for the initial set of vaccinations;
- Occurrence, intensity, and relationship of unsolicited AEs for 21 days following each vaccine administration;

- Occurrences of SAEs, MAAEs, AEs leading to withdrawal, AESIs (including VAED anaphylaxis, and severe allergic reactions), and deaths up to 21 days following each vaccine administration;
- Occurrences of SAEs, MAAEs, AEs leading to withdrawal, AESIs (including VAED, anaphylaxis, and severe allergic reactions), and deaths from Day 43 to Day 201;
- Occurrences of SAEs, MAAEs, AEs leading to withdrawal, AESIs (including VAED, anaphylaxis, and severe allergic reactions), and deaths from Day 202 to Day 386.

4.3.3. Exploratory Endpoint

In the Phase 2 and Phase 3 portions, the exploratory safety endpoint is:

- Further characterization of the safety profile of the CoVLP formulation, if it is likely to be informative.

5. Analysis Sets

5.1. Safety Analysis Set

The SAS is defined as all subjects who received at least one dose of either the CoVLP formulation or placebo prior to IOA/EUA. All safety analyses will be performed using the SAS and according to actual vaccination received. Solicited safety data in the 7 day period after each vaccination will be analyzed according to actual vaccine received at last dose. Unsolicited safety events (i.e., non-serious AEs, SAEs, AESIs, MAAEs, etc.) occurring in the period between Day 0 and Day 21 (before second dose), and in the period between Day 21 (on or after second dose) and Day 42 will be analyzed according to actual last vaccination received. All other summaries of safety events (e.g., for period between Day 43 and Day 201) will be summarized according to actual vaccination received at first dose.

Same logic will be applied for analysis of safety data collected after unblinding after IOA/EUA and receipt of CoVLP formulation. Unsolicited safety events (i.e., non-serious AEs, SAEs, AESIs, MAAEs, etc.) occurring in the period between first dose and 21 days after first dose, and in the period from second dose to 21 days after will be analyzed according to actual last vaccination received. All other summaries of safety events (e.g., for the period starting 21 days after second dose) will be summarized according to actual vaccination received at first dose after IOA/EUA. Safety data collected after IOA/EUA for all other subjects (i.e., those who did not receive CoVLP formulation post-IOA/EUA and those receiving CoVLP formulation prior to IOA/EUA) will be analyzed as described in the previous paragraph.

However, for subjects who become prematurely unblinded because they requested to be unblinded for the purposes of receiving an approved or authorized COVID-19 vaccine, their safety data will be handled according to Figure 2.

Figure 2 Decision Tree for Handling of Safety Data from Subjects Who Request to Become Prematurely Unblinded to Receive an Approved or Authorized COVID-19 Vaccine in Phase 2 and Phase 3 Portions Prior to IOA/EUA

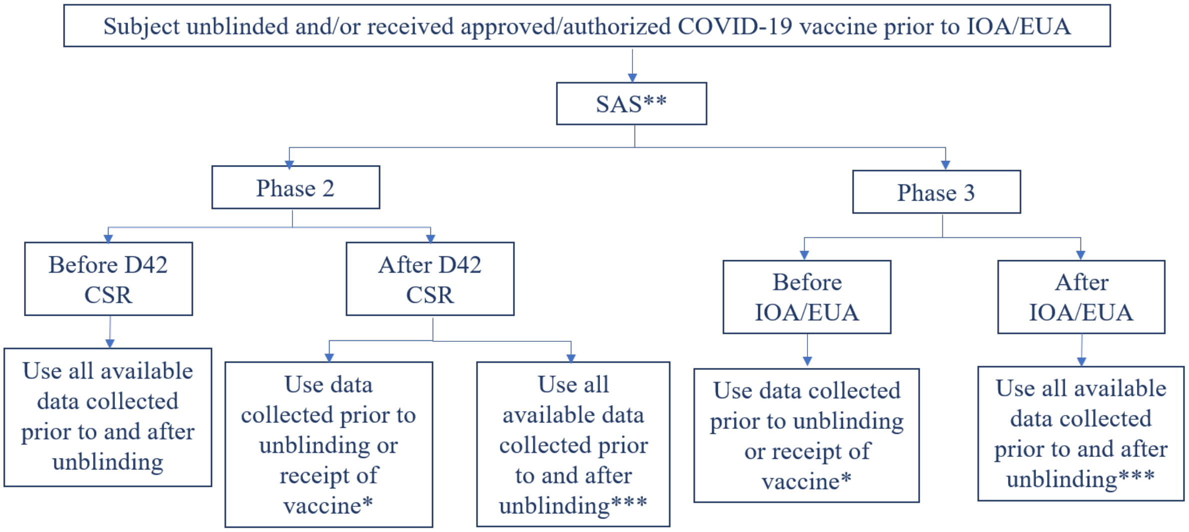

*whichever occurs first
**Subject who becomes unblinded or receives an approved or authorized COVID-19 vaccine will be included in the SAS however her/his data collected after unblinding or receipt of the vaccine, whichever comes first, will be excluded from some analyses, as noted in this flowchart.
***For subjects who receive the CoVLP formulation post-IOA/EUA, analyses will be presented separately for data collected prior to and after IOA/EUA.

For these particular subjects, the safety data collected will be analyzed in the following manner:

Phase 2 portion:

• Prior to Day 42 clinical study report (CSR):

o Safety analyses performed using safety data collected from subjects who become unblinded because they requested to be unblinded for the purposes of receiving an approved or authorized COVID-19 vaccine will be analyzed using all available data collected prior to and after unblinding.

• After Day 42 CSR:

o Safety analyses will be performed using only safety data collected prior to unblinding or receipt of an approved or authorized COVID-19 vaccine, whichever occurs first, from subjects in the SAS;

o Safety data collected from subjects who become unblinded because they requested to be unblinded for the purposes of receiving an approved or authorized COVID-19 vaccine

will be analyzed using all available data collected prior and after unblinding. For subjects who receive the CoVLP formulation post-IOA/EUA, safety analyses will be presented separately for data collected prior to and after IOA/EUA.

In the Phase 3 portion, the safety data collected from subjects who become unblinded because they requested to be unblinded for the purposes of receiving an approved or authorized COVID-19 vaccine will be analyzed in the following manner:

- Prior to IOA/EUA:

  • Safety analyses performed using safety data collected prior to unblinding or receipt of an approved or authorized COVID-19 vaccine, which ever occurs first, from subjects in the SAS;

- After IOA/EUA:

  • Safety analyses performed using safety data collected prior to and after unblinding from subjects in the SAS.

  • For subjects who receive the CoVLP formulation post-IOA/EUA, safety analyses will be presented separately for data collected prior to and after IOA/EUA.

5.1.1. Solicited Safety Analysis Subset

All subjects will be required to complete an e-diary or paper diary (backup) of solicited adverse events, from day of vaccination through 7 days after vaccination. The data to be collected includes solicited local and systemic adverse events, and daily body temperature for assessment of fever. A subset of at least 6000 subjects (with at least 3000 subjects in each treatment group as defined by randomization) who meet the dosing regimen, i.e., two vaccinations within protocol-stated window and correct needle use for administration will be selected from subjects who have at least two months of follow-up after second vaccination. Summaries of solicited adverse events will be produced for this group of subjects at time of initial submission of PVE and summaries will be updated with all data available at end of study.

5.2. Intention-To-Treat (ITT) Set

The ITT set will consist of all subjects who were randomized in the study. Analysis will be performed according to randomized treatment assigned regardless of treatment received.

5.3. Per Protocol (PP) Set

The PP set will consist of a subset of the ITT set who participated in the study until the end of the surveillance period in the Phase 2 or Phase 3 portions of the study; had no major protocol deviations related to subject eligibility, the ability to develop a valid immune response, prohibited medication use, or the efficacy analyses; and who received both doses of either the

CoVLP formulation or placebo. Major protocol deviations will be identified and documented during a blinded data review prior to database lock and confirmed at the time of database lock.

The only exceptions for the PP set are the inclusion of subjects who become prematurely unblinded because they requested to be unblinded for the purposes of receiving an approved or authorized COVID-19 vaccine or subjects who received an approved or authorized COVID-19 vaccine (refer to Figure 3).

Figure 3 Decision Tree for Handling of Efficacy Data from Subjects Who Request to Become Prematurely Unblinded to Receive an Approved or Authorized COVID-19 Vaccine in Phase 2 and Phase 3 Portions Prior to IOA/EUA

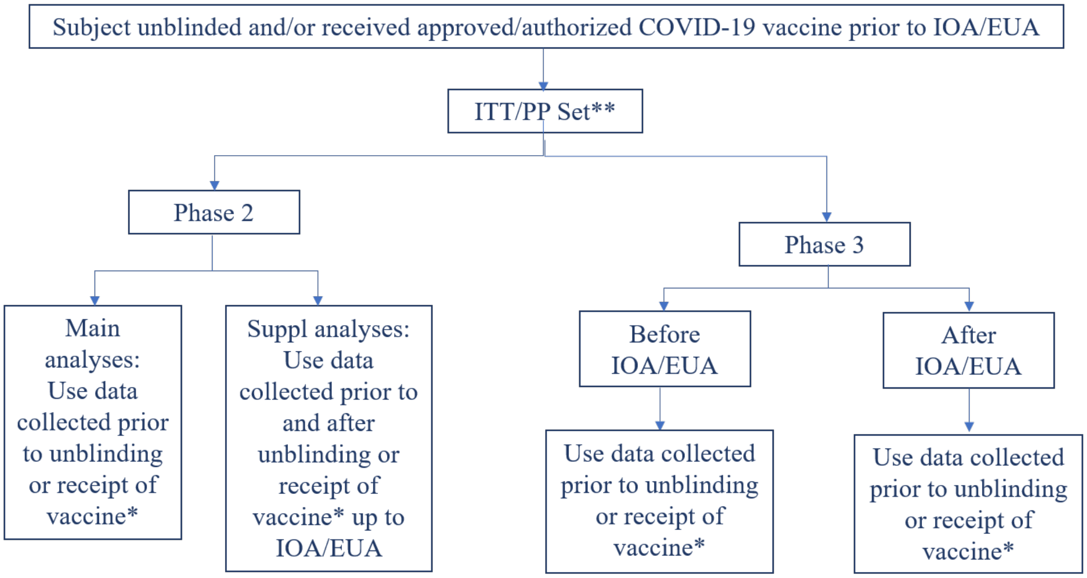

*whichever occurs first
**Subject who becomes unblinded or receives an approved or authorized COVID-19 vaccine will be included in the ITT/PP set however her/his data collected after unblinding or receipt of the vaccine, whichever comes first, will be excluded from some analyses, as noted in this flowchart.

For these particular subjects, the efficacy data collected will be analyzed in the following manner:

Phase 2 portion:

• Main efficacy analyses: efficacy analyses performed using efficacy data collected prior to unblinding or receipt of an approved or authorized COVID-19 vaccine, which ever occurs first, from subjects in the PP set and ITT set;

• Supplementary efficacy analyses: efficacy analyses performed using efficacy data collected prior to and after unblinding or receipt of an approved or authorized COVID-19 vaccine, which ever occurs first, from subjects in the PP set and ITT set up to IOA/EUA.

Phase 3 portion:

• Prior to IOA/EUA:
    • Efficacy analyses performed using efficacy data collected prior to unblinding or receipt of an approved or authorized COVID-19 vaccine, whichever occurs first, from subjects in the PP set and ITT set;

• After IOA/EUA:
    • Efficacy analyses based on asymptomatic cases performed using efficacy data collected prior to unblinding or receipt of an approved or authorized COVID-19 vaccine, whichever occurs first, from subjects in the PP set and ITT set.

Subjects who received the wrong treatment, but for whom the treatment received can be unequivocally confirmed, will be analyzed as treated, according to the first actual vaccination received, provided they have no other deviations that compromise their data.

In Phase 2, the analyses of all efficacy endpoints will be performed using the PP set as primary analysis and the ITT set as a secondary analysis population.

In Phase 3, the analyses of all efficacy endpoints will be performed using the ITT set as primary analysis and the PP set as a secondary analysis population.

5.4. Immunogenicity ITT and Per Protocol (IPP) Sets

The immunogenicity ITT set will consist of all subjects in the ITT who were also part of the immunogenicity subset of the Phase 2 and Phase 3 portions of the study. The IPP set will consist of a subset of the immunogenicity ITT set who completed the study with no major deviations related to subject eligibility, the ability to develop a valid immune response, prohibited medication use, or the immunogenicity analyses; and who received either the CoVLP formulation or placebo. The only exceptions for the IPP set are the inclusion of subjects who become unblinded prematurely because they requested to be unblinded for the purposes of receiving an approved or authorized COVID-19 vaccine or subjects who received an approved or authorized COVID-19 vaccine (refer to Figure 4). For these particular subjects, all data collected before the time of unblinding or receipt of an approved or authorized COVID-19 vaccine, whichever occurs first, will be included in the immunogenicity analyses.

Phase 2 portion:

• Prior to Day 42 CSR:
    • Immunogenicity analyses performed using immunogenicity data collected from subjects who become unblinded because they requested to be unblinded for the purposes of receiving

an approved or authorized COVID-19 vaccine will be analyzed using all available data collected prior to and after unblinding from subjects in the immunogenicity ITT set;

• After Day 42 CSR:

• Immunogenicity analyses performed using immunogenicity data collected prior to unblinding or receipt of an approved or authorized COVID-19 vaccine, whichever occurs first, from subjects in the IPP/ITT;

• In addition, immunogenicity analyses performed using immunogenicity data collected from subjects who become unblinded because they requested to be unblinded for the purposes of receiving an approved or authorized COVID-19 vaccine will be analyzed using all available data collected prior and after unblinding, from subjects in the IPP/immunogenicity ITT sets.

For subjects who receive the CoVLP formulation post-IOA/EUA, immunogenicity analyses will be presented separately for data collected prior to and after IOA/EUA.

Phase 3 portion:

For these particular subjects, the immunogenicity data collected will be analyzed in the following manner.

• Prior to IOA/EUA:

• Immunogenicity analyses performed using immunogenicity data collected prior to unblinding or receipt of an approved or authorized COVID-19 vaccine, whichever occurs first, from subjects in the IPP/immunogenicity ITT sets;

• After IOA/EUA:

• Immunogenicity analyses performed using immunogenicity data collected prior to and after unblinding from subjects in the IPP/immunogenicity ITT sets.

For the Day 21 analysis, this should include the subjects who received the first vaccine dose and had Day 0 and Day 21 immunogenicity sample collections. For the Day 42 analysis, this should include the subjects who received both vaccine doses and had Day 0 and Day 42 immunogenicity sample collections. For the Day 128 analysis, this should include the subjects who received both vaccine doses and had Day 0 and Day 128 immunogenicity sample collections. For the Day 201 analysis, this should include the subjects who received both vaccine doses and had Day 0 and Day 201 immunogenicity sample collections. For the Day 386 analysis, this should include the subjects who received both vaccine doses and had Day 0 and Day 386 immunogenicity sample collections. The timepoints above refer to both Phase 2 and Phase 3 portions except for Day 128. Subjects who had blood samples for immunogenicity taken outside of the time window for blood sample collection are to be excluded from the IPP set for the specific visit. Subjects who received the wrong treatment at the first vaccination, but for whom the treatment received can be unequivocally confirmed, will be analyzed as treated, provided

they have no other deviations that compromise their data. Subjects who receive a treatment at the second vaccination that is not the same treatment received at the first vaccination will not be included in the IPP set at Day 42, Day 128, Day 201, and Day 386; the subjects will remain in the Day 21 analysis and analyzed as treated.

The analyses of all immunogenicity endpoints will be performed using the IPP set in both Phase 2 and Phase 3 portions, as the primary analysis population, and the immunogenicity ITT set, as a secondary analysis population.

Figure 4 Decision Tree for Handling of Immunogenicity Data from Subjects Who Request to Become Prematurely Unblinded to Receive an Approved or Authorized COVID-19 Vaccine in Phase 2 and Phase 3 Portions Prior to IOA/EUA

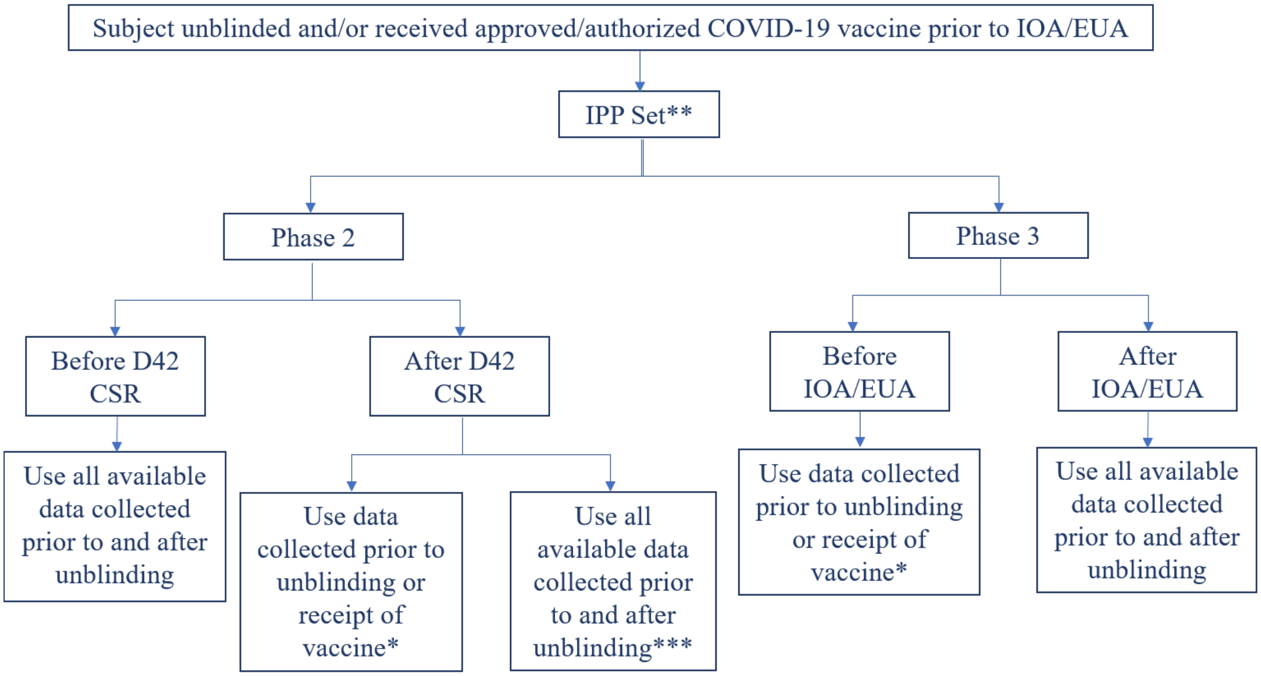

*whichever occurs first
**Subject who becomes unblinded or receives an approved or authorized COVID-19 vaccine will be included in the IPP set however her/his data collected after unblinding or receipt of the vaccine, whichever comes first, will be excluded from the analysis, as noted in this flowchart.
***For subjects who receive the CoVLP formulation post-IOA/EUA, analyses will be presented separately for data collected prior and after IOA/EUA.

6. General Aspects for Statistical Analysis

6.1. General Methods

- All statistical analyses will be conducted with the SAS® software package version 9.4 or higher.
- Unless otherwise specified, summaries will be presented for each treatment and overall.
- Continuous variables will be summarized using the number of observations (n), mean, standard deviation (SD), median, minimum, and maximum. Categorical variables will be summarized using number of observations (n), frequency and percentages of subjects.
- All relevant subject data will be included in listings. All subjects entered into the database will be included in subject data listings.
- Unless otherwise specified, confidence intervals (CI) will be based on two-sided 95% confidence.
- If multiple assessments occur at a given post-baseline time point, the latest value within that time point will be used.
- “Below LOD” in IFN-γ and IL-4 data should be considered as “0”.
- “NR” in IFN-γ and IL-4 data should be considered as “not reportable” results.
- Value of “< 100” and “IR” in Nab data due to hemolyzed samples should be considered as “not reportable” results.
- “>ULOQ” in Nab data are results above ULOQ at the maximal dilution and the “>ULOQ” value should be assigned as “64000”.
- All the summaries will be provided by phase (Phase 2 and Phase 3) and by enrollment population (Study Population #1, #2, or #3) and by age category if applicable for each study population. For the Phase 3 portion of the study, Study Population as defined at randomization will be utilized for analysis except for the following situations:
    - If a subject has a BMI value at screening < 18.5 kg/m^2 or ≥ 35 kg/m^2, the subject will be analyzed under Study Population #3
    - If a subject has a BMI ≥ 18.5 and < 35 kg/m^2 and no other comorbidities, the subject will be analyzed under Study Population #1 or #2 according to relevant age value at screening.

6.1.1. Geometric Mean Titer (GMT)

Continuous immunogenicity endpoints will be logarithmically transformed with 10 as base for analysis. GMT point estimates and the corresponding two-sided 95% CI by treatment group will be calculated as the antilog of the mean and 95% CI of log transformed titer values for Day 0, Day 21, Day 42, Day 128, Day 201, and Day 386.

6.1.2. Geometric Mean Fold Rise (GMFR)

GMFR will be defined as the geometric mean of the ratio of GMTs (Day 21/Day 0, Day 42/Day 0, Day 128/Day 0, Day 201/Day 0 and Day 386/Day 0). GMFR will be derived by using ANCOVA to model the difference in the log of the titer values between Day 21/42/128/201/386, with treatment group as main effect and log-transformed baseline titer as covariate.

6.1.3. Seroconversion (SC) Rate

The point estimates and the corresponding two-sided 95% CI for subjects achieving SC by treatment group will be calculated and reported. Fisher's exact tests will be used to compare between the treatment groups.

SC rate is defined as the proportion of subjects achieving SC in the analysis set i.e., subjects with:

- For subjects with detectable Nab titer at Day 0 (i.e., baseline Nab titer ≥10): a ≥ 4-fold increase in Nab titers between Day 0 and Day 21/42/128/201/386, respectively.
- For subjects with undetectable Nab titer at Day 0 (i.e., baseline Nab titer < 10): Nab titer of ≥ 40 on Day 21/42/128/201/386, respectively.

6.2. Key Definitions

6.2.1. Baseline

Unless otherwise specified, Baseline is defined as the last available value prior to the first administration of COVID-19 vaccine (or placebo) and includes assessments taken on Day 0.

6.2.2. Change from Baseline

The change from baseline will be calculated for each post-baseline assessment as:

Change from Baseline = Post-baseline value – Baseline value

If either value is missing, change from baseline will not be calculated.

6.2.3. Onset Day

The Onset Day is defined as the day of start and calculated as day of vaccination + 1.

6.2.4. COVID-19 Case

COVID-19 case is one with at least one symptom reported by the subject, a virologically-confirmed test result, and confirmation by the IDMC.

More specifically, COVID-19 will be defined as the presence of a laboratory-confirmed (virologic method) SARS-CoV-2 infection with the occurrence of a new onset of one or more of the following symptoms:

- Fever or chills;
- Cough;
- Shortness of breath or difficulty breathing;
- Fatigue;
- Muscle or body aches;
- Headache;
- New loss of taste or smell;
- Sore throat;
- Congestion or runny nose;
- Nausea or vomiting;
- Diarrhea.

The swab sample results from the central virology laboratory will be considered the primary results for vaccine efficacy evaluation. For subjects who are hospitalized with severe COVID-19 and cannot provide samples for testing at the central virology laboratory, SARS-CoV-2 testing results from the hospital may be considered for use in the evaluation of vaccine efficacy if documentation of the local testing procedures and results is available.

6.2.5. Severe COVID-19 Case

Severe COVID-19 disease will be defined as virologically-confirmed SARS-CoV-2 infection with the occurrence of a new onset of any of the following:

- Clinical signs at rest indicative of severe systemic illness (respiratory rate $\geq$ 30 per minute, heart rate $\geq$ 125 per minute, SpO2 $\leq$ 93% on room air at sea level or PaO2/FiO2 < 300 mmHg);
- Respiratory failure (defined as needing high-flow oxygen, non-invasive ventilation, mechanical ventilation or ECMO);
- Evidence of shock (SBP < 90 mmHg, DBP < 60 mmHg, or requiring vasopressors);
- Significant acute renal, hepatic, or neurologic dysfunction;
- Hospitalization;
- Death.

This document is confidential.

For the Phase 2 part of the study, the severity of disease will be ascertained by the site. For the Phase 3 part of the study, a subcommittee of the IDMC will assess severity of COVID-19 cases using the definition described above with the exception that ICU hospitalization is expected instead of any hospitalization.

6.2.6. Date of Unblinding

If unblinding (access to treatment code for a subject) has occurred more than once at different dates or times, the earliest of those dates and times will considered the unblinding date and time.

6.3. Missing Data

6.3.1. Safety Data

Missing date imputation will be applied to concomitant medications and adverse events. There is no imputation for completely missing dates.

Missing or partial concomitant medication start date:

- If only DAY is missing, use the first day of the month.
- If DAY and Month are both missing, use the first day of the year.

Missing or partial concomitant medication stop date:

- If only DAY is missing, use the last day of the month.
- If DAY and Month are both missing, use the last day of the year.
- If DAY, Month and year are all missing, assign 'continuing' status to stop date.

Missing or partial AE date:
No imputation of AE partial/missing start/end dates or times will be performed. In case of missing or incomplete AE onset date/time, the AE will be included in the analysis unless the incomplete AE onset date/time information unequivocally indicates that the AE started prior to Day 0 (see below for further details).

All analyses described in this section will be based on AE with an onset date on or after the date and time of the first vaccination (Day 0) if not otherwise specified. In case of missing or incomplete AE onset date/time, the AE will be included in the analysis unless the incomplete AE onset date/time information unequivocally indicates that the AE started prior to Day 0, e.g. if a subject is vaccinated on the 15JUL2020 and the AE onset date is XXJUN2020, then the AE will not be included in the analysis. If the AE onset date is JUL2020, then the AE will be included in the analysis.

Adverse events analyses will be presented for the following reporting period, as applicable:

This document is confidential.

- Day 0-21 period after vaccination 1: Any AEs meeting the following criteria will be allocated to the first vaccination (vaccination 1) and included in the Day 0-21 analysis for first vaccination:
    - Any AEs with:
        - onset date/time < date and time of second vaccine administration, if second vaccination is done,
        - else if vaccination 2 not performed, any AEs with onset date ≤ date of vaccination 1+ 21
- Day 0-21 period after vaccination 2: Any AEs meeting the following criteria will be allocated to second vaccination (vaccination 2) and included in the Day 0-21 analysis for second vaccination:
    - Any AEs with onset date/time ≥ Date/time of vaccination 2 and with onset date ≤ date of vaccination 2 + 21.
- Day 0-21 period after last vaccination: Any AEs meeting the following criteria will be included in the Day 0-21 period after last vaccination:
    - Any AEs with onset date ≤ date of last vaccination + 21 i.e.:
        - Any AEs with onset date ≤ date of vaccination 2 + 21, if vaccination 2 is performed
        - Any AEs with onset date ≤ date of vaccination 1 + 21, if vaccination 2 is not performed
- Day 0 to Day 201: Any AEs with onset date ≤ Day 201 visit date (or vaccination 1 + 201 if Day 201 visit not performed).
- Day 0 to End of Study (EOS): Any AEs recorded in the database
- Day 22 after last vaccination to Day 201: Any AEs recorded in the database at time of analysis and meeting the following criteria will be included in the Day 22 after last vaccination – Day 201 analysis:
    - Any AEs with date of last vaccination + 21 < onset date ≤ Day 201 visit date (or date of vaccination 1 + 201 if Day 201 visit is missing) i.e.:
        - Any AEs with date of vaccination 2 + 21 < onset date ≤ Day 201 visit date (or date of vaccination 1 + 201 if Day 201 visit is missing), if vaccination 2 is performed
        - Any AEs with date of vaccination 1 + 21 < onset date ≤ Day 201 visit date (or date of vaccination 1 + 201 if Day 201 visit is missing), if vaccination 2 is not performed
- Day 201 to EOS: Any AEs recorded in the database at time of analysis with onset date > Day 201 visit date (or date of vaccination 1 + 201 if Day 201 visit is missing)

Note: If an AE allocation to a reporting cannot be unequivocally ascertained due to partial/missing date, the AE will be allocated to all relevant periods,. e.g., if a subject is vaccinated on 01AUG2020 and then on 23AUG2020 and has an AE with onset date on

XXAUG2020, the AE will be allocated to Day 0-21 period after vaccination 1 and the Day 0-21 period after vaccination 2.

Adverse events analyses will be presented separately for placebo subjects who receive CoVLP after IOA/EUA in a similar pattern to that described above.

6.3.2. Immunogenicity

Missing immunogenicity data will be retained as missing. If antibody titers and concentrations are below the cutoff, i.e., the result is of the form '<xx', the value will be set to 0.5*'xx' before performing GMT calculations.

For analysis of counts, e.g., for the calculation of fold change (ratio of post-baseline count/baseline count) of IFN-γ (ELISpot) assay results, a value of '5' will be imputed for values of '0' at baseline.

6.4. Solicited Safety Data

The PI assessment of a solicited adverse event will always be used when available.

In the event that both an e-diary and a paper diary were completed by the subject for the same solicited AE and the same timepoint, the record with the PI assessment will be used for analysis; otherwise, the higher grade will be used in the analysis.

Scheduled day and timepoint, if applicable, will be used as record identifier(s) in solicited AE summaries.

6.5. Visit Windows

For the Day 21 and Day 42 visits, a window of +/-2 days will be applied to sample collection data. Subjects who have blood samples for immunogenicity taken outside of the time window for blood sample collection are to be excluded from the IPP set for the specific visit.

6.6. Pooling of Centers

Data from all sites will be summarized together for analyses.

6.7. Subgroups

The following subgroup analyses will be performed for identified safety data:

- Gender (male and female);
- Race (Caucasian or White; Black or African American; Asian).
- Ethnicity (Hispanic or Latino; Not Hispanic or Latino)

Efficacy analyses will be presented by sex, race and ethnicity.

Identified safety data include:

- Immediate solicited and unsolicited AEs up to 30 minutes after each vaccination;
- Solicited local and systemic AEs within first seven days after each vaccination;
- Most frequent unsolicited AEs within 21 days after vaccination;
- AESIs;
- SAEs;
- MAAEs;
- AEs leading to death;
- AEs leading to withdrawal.

7. Demographic, Other Baseline Characteristics and Medication

7.1. Subject Disposition and Withdrawals

Subject disposition will be presented for all the subjects, which will include the following for each study phase:

- The number of subjects screened
- The number of subjects resulting in screen failure
- The number of subjects randomized
- The number (%) of placebo subjects who go on to receive the CoVLP vaccine
- The number (%) of subjects in each arm who were unblinded as per the pre-specified criteria
- The number (%) of subjects in each arm who were unblinded as per the pre-specified criteria but did not receive the approved COVID-19 vaccine and remained on the study with data collection continuing
- The number (%) of subjects in each arm who were unblinded as per the pre-specified criteria and received an approved or authorized COVID-19 vaccine and are withdrawn/discontinued from the study
- In Phase 3, the number (%) of subjects in each arm who requested to be unblinded before the completion of their Day 21 visit but did not receive the approved or authorized COVID-19 vaccine and were therefore eligible to receive their second study vaccination at the Day 21 visit.
- In Phase 3, the analyses of all efficacy endpoints will be performed using the ITT set as primary analysis and the PP set as a secondary analysis population. For both analysis sets, the number (%) of subjects in each arm who are censored at the primary analysis of the primary endpoint due to the various reasons listed below.
    - censoring will be performed when subjects will experience their first COVID-19 case, the date the database will be frozen for the primary analysis, the date subject is unblinded, the date subject receives an approved or authorized COVID-19 vaccine, or at the date of subject completion/withdrawal from the study, whichever occurs first.

Among the randomized subjects, the following will be summarized by treatment group:

This document is confidential.

- The number (%) of subjects in the SAS
- The number (%) of subjects with a one-month safety follow-up after the second dose
- The number (%) of subjects with a two-month safety follow-up after the second dose
- The number (%) of subjects in the ITT Set
- The number (%) of subjects in the PP Set
- The number (%) of subjects in the IPP Set (Day 0, Day 21, Day 42, Day 128, Day 201, and Day 386)
- The number (%) of subjects who completed the study (Day 21, Day 42, Day 128, Day 201, and Day 386)

The number (%) of subjects who prematurely discontinued from the study at Day 21, Day 42, Day 128, Day 201, and Day 386 and the associated reasons Day 128 visit is only applicable for Phase 2 summaries.

All protocol deviations will be listed and major protocol deviations will be summarized for Phase 2 and Phase 3 separately.

7.2. Demographic and Baseline Characteristics

Demographics summary will be presented for a) all subjects, b) subjects with data collected prior to unblinding and included in the PVE analysis and c) subjects who are unblinded and withdrew from the study before the PVE analysis was performed separately for each of the following analysis sets:

- ITT Set for Phase 2 and Phase 3 separately
- PP Set for Phase 2 and Phase 3 separately
- SAS for Phase 2 and Phase 3 separately
- IPP Set for Phase 2 and Phase 3 separately

Demographic data will be presented in listings and summarized by treatment. Continuous variables including age, weight, height, and BMI will be summarized with mean, median, standard deviation, minimum, and maximum. Count and percentage of subjects will be presented for categorical variables such as sex at birth, race, ethnicity, and baseline SARS-CoV-2 testing results. Summary tables will be provided by study population and by age strata within each population when applicable.

Age at Study day 1 = Age at time of consent

Height (in cm) = height (in inches) * 2.54

Weight (in kg) = weight (in lbs) * 0.4536

BMI (kg/m^2) = Weight(kg)/[Height$(m)^2$]

7.3. Medical History and Concomitant Diseases

Medical History will be presented for each of the following analysis sets:

- Safety Analysis Set for Phase 2 and Phase 3 separately

A summary table of the number and percentage of subjects by medical history using system organ class (SOC) will be produced for patients in the Safety Analysis Set. Medical history will be sorted by serial code for international SOC sort order using the Medical Dictionary for Regulatory Activities (MedDRA) coding dictionary, version 23.1 or higher. Within SOC, medical history will be sorted by preferred term in descending order of total number. For the summary tables, a subject may appear more than once if he/she has more than one medical history coded under different SOC categories. However, the subject will be counted only once in the overall category. Medical history will be summarized by treatment group. A separate by-subject listing for medical history data will also be provided.

7.4. Medication

Prior and Concomitant medications will be summarized by ATC level 2 and preferred drug name for the Safety Analysis Set by treatment group.

7.4.1. Prior Medication

Prior medication is any medication that was started prior to study vaccine date and ended before study vaccine date.

7.4.2. Concomitant Medication

Concomitant medication is a medication that starts on or after vaccination date/time at Day 0 or starts before vaccination and is still on-going at the time of the study vaccine.

A separate summary will be provided for concomitant medications started on or after day of first vaccination received after IOA/EUA.

Concomitant medications must be reported (reason for use, dates of administration, dosage, and route) if the use meets the following conditions:

- Within 30 days preceding vaccination: any treatments and/or medications specifically contraindicated (e.g., vaccines, any immunoglobulins or other blood products, or any immune modifying drugs, etc.);
- From randomization to Day 42 (Phase 2 and Phase 3 portions), inclusive: any medication

(including, but not limited to, over-the-counter medicines such as aspirin or antacids), vitamins, and mineral supplements;

- From Day 43 to the end of the study (Phase 2 and Phase 3 portions), inclusive: any concomitant medication(s) administered to treat an AESI, MAAE, SAE, or AE leading to withdrawal; any concomitant medication used to treat an AE that occurred before Day 42 and that is still being used afterwards (i.e., ongoing use);
- Any concomitant medication used to treat conditions reported as medical history;
- Any investigational medication or vaccine; any vaccine not foreseen in the study protocol.

7.5. Treatment Exposure and Compliance

Study treatments will be prepared and administered by trained unblinded site staff via IM injection to each subject at each Investigator site. In addition, the Investigator or designated study center personnel will maintain a log of all study treatments dispensed and administered to the subject. The number, percentage and the reason of subject's refusal of vaccination post-randomization will be summarized.

Summary of extent of treatment exposure will be provided based on the Safety Analysis Set for Phase 2 and Phase 3 separately, and for before IOA/EUA and after IOA/EUA separately.

8. Efficacy

Efficacy summary tables will present data for the overall population and for each age stratum according to each Phase 3 Study Population, as applicable. In addition, efficacy summary tables will also be presented by gender, race, ethnicity and sero-status at baseline. Summary tables will include subjects in the PP set and ITT set.

8.1. Primary Efficacy Endpoint(s) and Analysis

In the Phase 3 portion:

The primary efficacy endpoint for Phase 3 portion is the first occurrence, in a subject, of laboratory-confirmed (virologic method) symptomatic SARS-CoV-2 infection (≥ 7 days post-second vaccination) at the time of PVE analysis.

For the Phase 3 portion of the study, once at least 160 subjects have at least one symptomatic COVID-19 case episode (≥ 7 days post-second vaccination) and the median blinded safety follow-up duration of at least 2 months (post-administration of the second vaccination) for at least 3000 subjects in each of the CoVLP formulation and placebo groups has been achieved during the study, the primary efficacy endpoint of the Phase 3 portion of the study will be evaluated to determine if the vaccine efficacy (VE) success criterion has been met.

Vaccine efficacy will be evaluated for any strain as the incidence rate ratio of CoVLP formulation vaccinated versus placebo subjects to develop laboratory-confirmed symptomatic SARS-CoV-2 infection ≥ 7 days after the second vaccination, as per the following formula:

$$VE=(1-IRR)=(1-(\frac{a}{N_1} \div \frac{b}{N_0}))*100\ \%$$

Where:

IRR = incidence rate ratio

a = number of subjects with at least one positive COVID-19 case for subjects vaccinated with CoVLP; and

b = number of subjects with at least one positive COVID-19 case for subjects who receive placebo; and

N_1 = person-years for subjects vaccinated with CoVLP; and

N_0 = person-years in subjects who receive placebo.

For the ITT set analysis, the number of cases in the IRR calculation will include those occurring >= 7 days after second vaccination per the definition of the primary endpoint. The follow-up time used in the calculation of person-years will be calculated from the day of randomization.

This document is confidential.

For the PP set analysis, the number of cases in the IRR calculation will also include those occurring >= 7 days after second vaccination per the definition of the primary endpoint. The follow-up time used in the calculation of person-years will be calculated from the date of second vaccination + 7 days.

For both analysis sets, censoring will be performed when subject experiences their first virologically-confirmed COVID-19 case (date of first symptoms), the date of database freeze for the primary analysis, the date subject is unblinded, the date subject receives an approved or authorized COVID-19 vaccine, or at the date of subject completion/withdrawal from the study, whichever occurs first.

The VE success criterion for the primary efficacy endpoint, in the Phase 3 portion, is defined as a ≥ 50 % point estimate and a > 30 % lower limit of the 95 % CI.

Assuming the number of cases in each arm follows a Poisson distribution, then conditioning on the total number of cases and the ratio of person-time of follow up, the exact 95 % CIs for the incidence rate ratio IRR, and hence for VE = 1-IRR, are obtained via binomial distribution calculations [Rothman 2008].

For Phase 3, the primary efficacy endpoint will be summarized for subjects with data collected prior to unblinding or receipt of vaccine before and after IOA/EUA.

8.2. Secondary Efficacy Endpoint(s) and Analyses

Summary tables will be provided using PP set and ITT sets.

In the Phase 2 portion:

8.2.1. First occurrence of laboratory-confirmed (virologic method) symptomatic SARS-CoV-2 infection (≥ 7 days post-second vaccination)

VE will be evaluated as the incidence rate ratio of CoVLP formulation vaccinated versus placebo subjects to develop laboratory-confirmed symptomatic SARS-CoV-2 infection ≥ 7 days after the second vaccination, as per the formula used for the analysis of the primary efficacy endpoint (Section 8.1). The 95% CI will be used for VE in the Phase 2 portion.

8.2.2. Occurrences of severe COVID-19 disease (≥ 7 days post-second vaccination)

The same formula mentioned in Section 8.1 will be used to evaluate the incidence rate ratio of CoVLP formulation of vaccinated subjects versus placebo subjects who develop severe COVID-19 disease ≥ 7 days after the second vaccination. 95% CI will be used for VE in the Phase 2 portion.

In the Phase 3 portion:

For Phase 3, all the secondary efficacy endpoints will be summarized for subjects with data collected prior to unblinding or receipt of vaccine.

8.2.3. Occurrences of laboratory-confirmed asymptomatic SARS-CoV-2 infection at Day 201 in subjects who were seronegative at baseline (confirmed by the ELISA method for the N protein);

The same formula mentioned in Section 8.1 will be used to evaluate the incidence rate ratio of CoVLP formulation of vaccinated subjects versus placebo subjects to develop laboratory-confirmed asymptomatic SARS-CoV-2 infection at Day 201 in subjects who were seronegative at baseline (confirmed by the ELISA method for the N protein) except for VE success criterion as > 0 % lower limit of the 95 % CI will be used in the Phase 3 portion. The number of cases in the CoVLP arm collected at Day 201 will be compared to the number of cases collected in the placebo arm prior to IOA/EUA.

8.2.4. Occurrences of severe COVID-19 disease (≥ 7 days post-second vaccination) at the time of the PVE analysis

The same formula mentioned in Section 8.1 will be used to evaluate the incidence rate ratio of CoVLP formulation of vaccinated subjects versus placebo subjects who develop severe COVID-19 disease ≥ 7 days after the second vaccination.

8.2.5. Occurrence and intensity of COVID-19-related symptoms in virologically-confirmed cases up until resolution of the symptoms in subjects administered the CoVLP formulation compared to subjects administered the placebo at the time of the PVE analysis

The occurrences of COVID-19-related symptoms and their severity will be summarized by treatment using descriptive statistics. The occurrence of unusual manifestations of COVID-19 as well as the severity of COVID-19 symptoms will also be used to assess imbalance between the CoVLP and placebo groups as a possible indication of enhanced disease (full details provided in the Medical Management Plan and IDMC Charter).

8.2.6. First occurrence, in a subject, of laboratory-confirmed (virologic method) symptomatic SARS-CoV-2 infection (≥ first vaccination) at the time of PVE analysis

VE will be evaluated for any strain and this endpoint will be calculated and summarized using the same method described for the primary efficacy endpoint (Section 8.1), except for VE success criterion as > 0 % lower limit of the 95 % CI will be used in the Phase 3 portion.

8.2.7. First occurrence, in a subject, of laboratory-confirmed (virologic method) symptomatic SARS-CoV-2 infection (≥ first vaccination and < second vaccination) at the time of PVE analysis

VE will be evaluated for any strain and this endpoint will be calculated and summarized using the same method described for the primary efficacy endpoint (Section 8.1), except for VE success criterion as > 0 % lower limit of the 95 % CI will be used in the Phase 3 portion.

8.2.8. First occurrence, in a subject, of laboratory-confirmed (virologic method) symptomatic SARS-CoV-2 infection (≥ second vaccination and < 7 days post-second vaccination) at the time of PVE analysis

VE will be evaluated for any strain and this endpoint will be calculated and summarized using the same method described for the primary efficacy endpoint (Section 8.1), except for VE success criterion as > 0 % lower limit of the 95 % CI will be used in the Phase 3 portion.

8.2.9. Duration and intensity of viral shedding after SARS-CoV-2 infection at the time of PVE analysis

The maximum area under the curve (AUC) of the viral titer curve, based on serial quantitative RT-PCR results, for each positive COVID-19 case will be calculated using the SARS-CoV-2 virus load (log 10 copies / mL) [Wathuo 2017]. Theoretically, to calculate the maximum AUC, individuals would be assumed to have begun shedding immediately after the last negative sample before the start of an episode, and stopped shedding immediately before the first negative sample after the episode. For this study, since samples are collected only at the potential start of symptoms, and subjects are expected to have only one incidence of COVID-19, individuals will be assumed to have begun shedding at the time of collection of the first RT-PCR positive test performed by the central laboratory (D_A) and stopped shedding immediately after the last swab was collected that was positive per RT-PCR assay (D_B). A subject with a single positive RT-PCR result from central laboratory will be assumed to have shed the virus for 24 hours. The point estimates of mean AUC and the corresponding two-sided 95 % CI will be compared between treatment groups using an ANCOVA with fixed factors for treatment, age and sex. Age and sex will be used as covariates in the model.

To calculate the AUC for an episode with one positive observation:

$$AUC = 0.5 * (D_B - D_A) * Y_1,$$

And to calculate the AUC for an episode with three positive observations:

$$AUC = (0.5 * Y_1 * (D_1 - D_A)) + (0.5 * (D_2 - D_1) * (Y_1 + Y_2)) + (0.5 * (D_3 - D_2) * (Y_2 + Y_3)) + (0.5 * Y_3 * (D_B - D_3))$$

Y_i – Log viral density values; D_A – Day of last negative sample before start of episode; D_B – Day of first negative sample after end of episode; D_1 – Day of first positive sample; D_2 – Day of second positive sample; D_3 – Day of third positive sample.

The peak viral shedding will be assessed by retrieving the lowest CT value observed at any time at the laboratory for each positive COVID-19 case. Lowest CT values and the corresponding two-sided 95 % CI will be compared between treatment groups using an ANCOVA with fixed factors for treatment, age and sex.

For each positive COVID-19 case, the number of days to negative test from the start of the symptoms and from the first positive swab collected and the corresponding two-sided 95 % CI will be compared between treatment groups using an ANCOVA with fixed factors for treatment, age and sex.

8.2.10. First occurrence, in a subject, of laboratory-confirmed (virologic method) symptomatic SARS-CoV-2 infection (≥ 7 days post-second vaccination) by strain at the time of the PVE analysis

VE will be evaluated by strain and this endpoint will be calculated and summarized using the same method described for the primary efficacy endpoint (Section 8.1), except for VE success criterion as > 0 % lower limit of the 95 % CI will be used in the Phase 3 portion.

8.3. Exploratory Efficacy Endpoint(s) and Analyses

In the Phase 2 portion:

8.3.1. Occurrence and intensity of COVID-19-related symptoms up to resolution of the symptoms

The occurrences of COVID-19-related symptoms and their severity will be summarized by treatment using descriptive statistics. The occurrence of unusual manifestations of COVID-19 as well as the severity of COVID-19 symptoms will also be used to assess imbalance between the CoVLP and placebo groups as a possible indication of enhanced disease (full details provided in the Medical Management Plan and IDMC Charter). Symptoms are counted at the maximum severity experienced.

In the Phase 3 portion:

For Phase 3, all the exploratory efficacy endpoints will be summarized for subjects with data collected prior to unblinding or receipt of vaccine before and after IOA/EUA.

8.3.2. Occurrences of severe COVID-19 disease (≥ 7 days post-second vaccination) by strain.

The same formula mentioned in Section 8.1 will be used to evaluate the incidence rate ratio of CoVLP formulation of vaccinated subjects versus placebo subjects who develop severe COVID-19 disease ≥ 7 days after the second vaccination by strain.

9. Immunogenicity

Immunogenicity summary tables will present data for:

- All subjects according to Study Population;
- Subjects who are seropositive at baseline for SARS-CoV-2 antibodies according to Study Population;
- Subjects who are seronegative at baseline for SARS-CoV-2 antibodies according to Study Population;
- Each of the different age strata according to Study Population, as applicable.

Point estimates and 95% CI will be calculated for all immunogenicity endpoints and responses for the treatment groups will be compared using descriptive statistics.

9.1. Primary Immunogenicity Endpoint(s) and Analysis

In the Phase 2 portion:

9.1.1. Nab response induced in each Study Population against the SARS-CoV-2 virus on Days 0, 21, and 42 using GMT, SC rate, and GMFR

The following analyses for the Nab assay will be performed on the Immunogenicity Per Protocol (IPP) set:

- GMT (Day 0, Day 21, and Day 42): The point estimates and the corresponding two-sided 95 % CI by treatment group will be calculated as the antilog of the mean and 95 % CI of log transformed titer values;
- SC rate (Day 0, Day 21, and Day 42): The point estimates and the corresponding two-sided 95 % CI for subjects achieving SC by treatment group will be calculated and reported;
- GMFR: the geometric mean of the ratio of GMTs (Day 21/Day 0 and Day 42/Day 0).

GMT will be compared between treatment groups using the analysis of variance (ANOVA) model.

SC rate will be defined as the proportion of subjects achieving SC in the analysis set i.e., subjects with:

- For subjects with detectable Nab titer at Day 0 (i.e., baseline Nab titer ≥10): a ≥ 4-fold increase in Nab titers between Day 0 and Day 21/42/128/201/386, respectively.
- For subjects with undetectable Nab titer at Day 0 (i.e., baseline Nab titer < 10): Nab titer of ≥ 40 on Day 21/42/128/201/386, respectively.

For SC rate, Fisher's exact tests will be used to compare between the treatment groups, along with exact Clopper-Pearson 95% CI.

GMFR will be compared using the ANCOVA model. The GMFR will be derived by using ANCOVA to model the difference in the log of the titer values between Day 21 and Day 0 and between Day 42 and Day 0, with treatment group as main effect and log-transformed baseline titer as covariate.

9.1.2. Specific Th1 CMI response induced in each Study Population against the SARS-CoV-2 virus on Days 0, 21, and 42 measured by IFN-γ ELISpot

The specific Th1 CMI response induced on Day 0, Day 21, and Day 42 will be measured by the number of T cells expressing IFN-γ, using ELISpot, for each treatment group. The response at each timepoint and the change from baseline (Day 0) at each post-baseline timepoint will be compared between treatment groups using appropriate non-parametric (Wilcoxon) models. The Wilcoxon Rank Sum Test, also known as the Mann-Whitney Wilcoxon Test, will be used to estimate the difference in response between treatment groups, along with the corresponding 95% CI for the median and p-value. Since the response between timepoints are paired data, the Wilcoxon Signed Rank Test will be used to estimate the difference in response between timepoints, along with the corresponding 95% CI for the median and p-value.

In addition, the median of the ratio of CMI response (Day 21/Day 0, Day 42/Day 0) will be compared between treatment groups. The Wilcoxon Rank Sum Test, also known as the Mann-Whitney Wilcoxon Test, will be used to estimate the difference in response between treatment groups, along with the corresponding 95% CI for the median and p-value.

9.2. Secondary Immunogenicity Endpoint(s) and Analysis

In the Phase 2 portion:

9.2.1. Relative neutralizing antibody response between the healthy adults (Study Population #1) and the healthy elderly populations (Study Population #2; each age strata) induced at 21 days after the second vaccination will be analyzed using the following parameter: GMT

Nab GMT at Day 21 and Day 42 between Study Population #1 and Study Population #2 and #3 will be compared using the analysis of variance (ANOVA) model. Tukey's test will be performed to show p-values for the pairwise comparisons between Study Population #1 and each age strata in Study Population #2.

9.2.2. Relative neutralizing antibody response for the combination of healthy adults (Study Population #1) and the healthy elderly adults (Study Population #2) compared to adults and the elderly adults with significant comorbidities (Study Population #3) induced at 21 days after the second vaccination will be analyzed using the following parameter: GMT

Nab GMT at Day 0, Day 21 and Day 42 between Study Population #1 and Study Population #2 combined and #3 will be compared using the analysis of variance (ANOVA) model. Tukey's test

will be performed to show p-values for the pairwise comparisons between Study Population #1 and Study Population #3, Study Population #2 and Study Population #3, and Study Population #1 and Study Population #2 combined and Study Population #3.

9.2.3. Persistence of the Nab antibody response induced in each Study Population against the SARS-CoV-2 virus on Day 128, Day 201, and Day 386 using GMT, SC rate, and GMFR

The following analyses for the Nab assay will be performed on the Immunogenicity Per Protocol (IPP) set:

- GMT (Days 128, 201, and 386): The point estimates and the corresponding two-sided 95 % CI by treatment group will be calculated as the antilog of the mean and 95 % CI of log transformed titer values;
- SC rate (Days 128, 201, and 386): The point estimates and the corresponding two-sided 95 % CI for subjects achieving SC by treatment group will be calculated and reported;
- GMFR: the geometric mean of the ratio of GMTs (Day 201/Day 0 and Day 386/Day 0).

GMT will be compared between treatment groups using the analysis of variance (ANOVA) model.

SC rate will be defined as the proportion of subjects achieving SC in the analysis set i.e., subjects with:

- For subjects with detectable Nab titer at Day 0 (i.e., baseline Nab titer ≥10): a ≥ 4-fold increase in Nab titers between Day 0 and Day 21/42/128/201/386, respectively.
- For subjects with undetectable Nab titer at Day 0 (i.e., baseline Nab titer < 10): Nab titer of ≥ 40 on Day 21/42/128/201/386, respectively.

For SC rate, Fisher's exact tests will be used to compare between the treatment groups, along with exact Clopper-Pearson 95% CI.

GMFR will be compared using the ANCOVA model. The GMFR will be derived by using ANCOVA to model the difference in the log of the titer values between Day 21 and Day 0, between Day 42 and Day 0, Day 201 and Day 0, and Day 386 and Day 0 with treatment group as main effect and baseline titer as covariate.

9.2.4. Specific antibody response induced in each Study Population against the SARS-CoV-2 virus by the total IgG levels on Days 0, 21, and 42, and the persistence of these antibodies on Days 128, 201, and 386 using GMT, SC rate, and GMFR

The GMT and SC rate will be calculated on Day 0, Day 21, Day 42, Day 128, Day 201, and Day 386 as well as the GMFR (Day 21/Day 0, Day 42/Day 0, Day 128/Day 0, Day 201/Day 0, and Day 386/Day 0).

GMT will be compared between treatment groups using the analysis of variance (ANOVA) model.

SC rate will be defined as the proportion of subjects achieving SC in the analysis set i.e., subjects with:

- For subjects with detectable IgG titer at Day 0 (i.e., baseline IgG titer ≥100): a ≥ 4-fold increase in IgG titers between Day 0 and Day 21/42/128/201/386, respectively.
- For subjects with undetectable IgG titer at Day 0 (i.e., baseline IgG titer < 100): IgG titer of ≥ 400 on Day 21/42/128/201/386, respectively.

For SC rate, Fisher's exact tests will be used to compare between the treatment groups, along with exact Clopper-Pearson 95% CI.

GMFR will be compared using the ANCOVA model. The GMFR will be derived by using ANCOVA to model the difference in the log of the titer values between Day 21 and Day 0, between Day 42 and Day 0, Day 128 and Day 0, Day 201 and Day 0, and Day 386 and Day 0 with treatment group as main effect and log-transformed baseline titer as covariate.

9.2.5. The ratio of neutralizing antibody titers:IgG ELISA antibody titers at Day 21, Day 42, Day 128, Day 201, and Day 386

The Nab/IgG ratio will be calculated as the antilog of the Nab/IgG ratio and 95 % CI of log transformed titer values. The ratio will be compared between treatment groups using ANOVA.

9.2.6. Specific Th1 CMI response induced in each Study Population against the SARS-CoV-2 virus on Day 201 and Day 386 measured by IFN-γ ELISpot

The specific Th1 CMI response induced on Day 201 and Day 386 will be measured by the number of T cells expressing IFN-γ, using ELISpot, for each treatment group. The response at each timepoint and the change from baseline (Day 0) at each post-baseline timepoint will be compared between treatment groups using appropriate non-parametric (Wilcoxon) models. The Wilcoxon Rank Sum Test will be used to estimate the difference in response between treatment groups, along with the corresponding 95% CI for the median and p-value. The Wilcoxon Signed Rank Test will be used to estimate the difference in response between timepoints, along with the corresponding 95% CI for the median and p-value. The difference in response will be assessed only for subjects who had samples collected at both Day 0 and Day x. For the assessment at specific timepoint (e.g., Day 0, Day 21 etc.), we need to include all available sample data.

In addition, the median of the ratio of CMI response (Day 201/Day 0, Day 386/Day 0) will be compared between treatment groups. The Wilcoxon Rank Sum Test, also known as the Mann-Whitney Wilcoxon Test, will be used to estimate the difference in response between treatment groups, along with the corresponding 95% CI for the median and p-value.

9.2.7. Specific Th2 CMI response induced in each Study Population against the SARS-CoV-2 virus on Days 0, 21, 42, 201, and 386 measured by IL-4 (ELISpot)

The specific Th2 CMI response induced on Day 201 and Day 386 will be measured by the number of T cells expressing IL-4, using ELISpot, for each treatment group. The response will be compared between treatment groups and timepoints using appropriate non-parametric (Wilcoxon) models. The Wilcoxon Rank Sum Test will be used to estimate the difference in response between treatment groups, along with the corresponding 95% CI for the median and p-value. The Wilcoxon Signed Rank Test will be used to estimate the difference in response between timepoints, along with the corresponding 95% CI for the median and p-value.

In the Phase 3 portion:

For Phase 3, all the immunogenicity endpoints will be summarized as described below.

- Before IOA/EUA, use data for subjects collected prior to unblinding or receipt of vaccine
- After IOA/EUA, use all available data for subjects collected prior to and after unblinding.

9.2.8. In the immunogenicity subset, Nab antibody response induced in each Study Population against the SARS-CoV-2 virus on Days 0, 21, 42, 201, and 386 using GMT, SC rate, and GMFR

The same immunogenicity analyses will be performed as described in Section 9.2.3.

9.2.9. In the immunogenicity subset, specific antibody response induced in each Study Population against the SARS-CoV-2 virus by the total IgG levels on Days 0, 21, 42, 201, and 386 using GMT, SC rate, and GMFR

The same immunogenicity analyses will be performed as described in Section 9.2.4.

9.2.10. In the immunogenicity subset, the ratio of neutralizing antibody titers:IgG ELISA antibody titers at Day 21, Day 42, Day 201, and Day 386

The same immunogenicity analyses will be performed as described in Section 9.2.5.

9.2.11. In the immunogenicity subset, specific Th1 CMI response induced in each Study Population against the SARS-CoV-2 virus on Days 0, 21, 42, 201, and 386 measured by IFN-γ ELISpot

The same immunogenicity analyses will be performed as described in Section 9.2.6.

9.2.12. In the immunogenicity subset, specific Th2 CMI response induced in each Study Population against the SARS-CoV-2 virus on Days 0, 21, 42, 201, and 386 measured by IL-4 (ELISpot)

The same immunogenicity analyses will be performed as described in Section 9.2.7.

9.3. Exploratory Immunogenicity Endpoint(s) and Analysis

In the Phase 2 and Phase 3 portions:

9.3.1. In the immunogenicity subset, specific CMI response induced in each Study Population against the SARS-CoV-2 virus up to 21 days after the second vaccination and at Day 201 and Day 386, as measured by the percentage of CD4+ T cells expressing functional markers

The CMI response induced on Day 0, Day 21, Day 42, Day 201, and Day 386 will be measured by the percentage of T cells expressing functional markers, using flow cytometry, for each treatment group. The responses will be compared between treatment groups and timepoints using appropriate non-parametric (Wilcoxon) models. The Wilcoxon Rank Sum Test will be used to estimate the difference in response between treatment groups, along with the corresponding 95% CI for the median and p-value. The Wilcoxon Signed Rank Test will be used to estimate the difference in response between timepoints, along with the corresponding 95% CI for the median and p-value.

This analysis could be repeated for all available data under different stimulating conditions.

9.3.2. Further characterization of the immune response of the CoVLP formulation

If deemed necessary, further characterization of the immune response of the CoVLP formulation will be done and the results will be presented.

10. Safety

Safety and tolerability will be evaluated by solicited local and systemic adverse events (immediate solicited adverse events within 30 minutes post-vaccination and solicited adverse events up to seven days after each vaccination), unsolicited AEs within 30 minutes post-vaccination and up to 21 days after each vaccination, SAEs, AESIs, MAAEs, and AEs leading to withdrawal up to the end of the study. In addition, events will be monitored for possible VED, hypersensitivity components, and potential immune-mediated diseases, from all reported events during the study (collected AEs, SAEs, AESIs, MAAEs, and AEs leading to withdrawal). In the Phase 3 portion, the same safety endpoints will be collected (including immediate AEs) with one exception: solicited local and systemic AEs (up to seven days after each vaccination) will not be collected. Clinical safety methods will include repeated urinalysis, blood chemistry, and haematology testing.

Safety monitoring of safety signals will be performed for each of the Study Populations throughout the Phase 2 and Phase 3 portions of the study. Stopping rules or conditions for stopping each portion of the study would occur if there was clear evidence of harm or harmful effects such as SAEs related to the treatment (study vaccine). An SAE which is assessed as unrelated to the study vaccine would not warrant stopping the trial.

If any of the following events occur to the sentinel subjects, the study enrollment and vaccinations may be paused based on IDMC recommendations:

- Any SAE in a subject for which causality cannot be attributed to another cause;
- Two (2) or more subjects who receive the CoVLP formulation experience the same or similar AE:
    - Experience the same Grade 3 or higher solicited local AE or systemic AE which began within 7 days after administration of the vaccine;
    - Experience the same or similar Grade 3 or higher unsolicited AE (including symptoms, signs or laboratory safety AEs) that is judged anything but unrelated to the vaccine.

After vaccination of all sentinel subjects, the stopping rules for the remainder of the study:

The study may be paused based on IDMC recommendations if any of the following events occur:

- Any subject experiences an SAE after administration of the vaccine that is considered related to vaccine;
- If 5 % or more subjects who receive the CoVLP formulation experience the same or similar AE:
    - Experience the same or similar Grade 3 or higher solicited local AE or systemic AE, which began within 7 days after administration of the vaccine;
    - Experience the same or similar Grade 3 or higher unsolicited AE (including symptoms, signs or laboratory safety AEs) that is judged anything but unrelated to the vaccine;

- If an important imbalance in unusual manifestations of COVID-19 or severity of COVID-19 symptoms is observed between the CoVLP and placebo groups (full details provided in the Medical Management Plan and IDMC Charter).

Safety summary tables will present data for the overall population and for each age stratum according to each Study Population, as applicable.

10.1. Adverse Events

An AE or adverse experience is defined as any untoward medical occurrence in a subject or clinical investigation subject who was administered a pharmaceutical product, with or without a causal relationship with the treatment. Information such as the date and time of onset and resolution (duration), intensity (defined below), seriousness, any required treatment or action taken, outcome, relationship to the investigational vaccine, and whether the AE caused withdrawal from the study will be collected.

The intensity of all AEs will be graded as mild (1), moderate (2) severe (3), or potentially life threatening (4). If a subject has the same AE but with different grades over time, the highest grade will be reported. The causal relationship with the study vaccine of the unsolicited AEs will be assessed by the Investigator, and will be classified as "definitely not related", "probably not related", "possibly related", "probably related", or "definitely related". If there is any valid reason, even if undetermined or untested, for suspecting a possible cause-and-effect relationship between the investigational vaccine and the occurrence of the AE, then the AE should be considered "definitely related", "probably related", or "possibly related". Otherwise, if no valid reason exists for suggesting a possible relationship, then the AE should be classified as "probably not related" or "definitely not related". The causal relationship of all solicited local and systemic AEs will be considered related. Missing relatedness and intensity of AEs will not be imputed and will be presented as "Missing". AE outcomes will be classified as recovered/resolved, not recovered/not resolved, recovered/resolved with sequelae, recovering/resolving, or death. Most frequent AEs are defined as those that occur > 5% of either of the treatment groups and will only be summarized by PT.

An overall summary table for AE by vaccination 1 vs 2 will be provided for both Phase 2 and Phase 3 portions.

10.1.1. Solicited Local and Systemic Adverse Events

Subjects will be monitored for both solicited local AEs (erythema, swelling, and pain at the injection site) and solicited systemic AEs (fever, headache, fatigue, muscle aches, joint aches, chills, a feeling of general discomfort, swelling in the axilla, and swelling in the neck) from the time of each vaccination through seven days. While the subjects remain in the clinic following vaccine administration (at least 30 minutes), staff will monitor them for solicited local and systemic AEs; after release from the clinic facility, from the evening of each vaccination to the evening of the seventh day after each vaccination, the subject will measure and record the subject's local and systemic AEs in the diary. The only exception for the monitoring of solicited AEs is: solicited local and systemic AEs (up to seven days after each vaccination) will not be

collected from subjects in the placebo group who receive the CoVLP formulation post-IOA/EUA, however the immediate AEs (30 minutes after each vaccination) will still be collected. Solicited local and systemic AEs will be summarized through primary and secondary safety endpoints. Solicited AEs collected during the Phase 3 portion of the study will be summarized in the solicited safety analysis subset (Section 5.1.1) at the time of the PVE analysis, and on all subjects at study termination. The intensity of the solicited local and systemic AEs will be assessed by the subject, documented in the diary, and graded as: mild (1), moderate (2), severe (3) or potentially life threatening (4). The causal relationship of all solicited local and systemic AEs will be considered related. For each of these solicited events, occurrence and intensity (grading) will be summarized overall and by treatment group for each of these symptoms listed in Table 6 below. If a subject has the same AE but with different grades over time, the highest grade will be reported. Medically implausible fever temperature values (> 46.0 ºC) are excluded from tables but presented in the listings. Solicited adverse events with severity grade of 'None' are not collected.

Table 6 Severity Grades for Solicited Local and Systemic Adverse Events

**Symptoms**	**Severity**				
	**None**	**Grade 1 (Mild)**	**Grade 2 (Moderate)**	**Grade 3 (Severe)**	**Grade 4 (Potentially life-threatening)**
**Injection Site Adverse Events (Local Adverse Events)**					
**Erythema (redness)**	< 25 mm	25 - 50 mm	51 - 100 mm	> 100 mm	Necrosis or exfoliative dermatitis
**Swelling**	< 25 mm	25 - 50 mm and does not interfere with activity	51 – 100 mm or interferes with activity	> 100 mm or prevents daily activity	Necrosis
**Pain**	None	Does not interfere with activity	Repeated use of non-narcotic pain reliever for more than 24 hours or interferes with activity	Any use of narcotic pain reliever or prevents daily activity	Results in a visit to emergency room (ER) or hospitalization
**Solicited Systemic Adverse Events**					
**Fever (ºC or ºF)**	< 38.0 ºC<br>< 100.4 ºF	38.0 - 38.4 ºC<br>100.4 - 101.1 ºF	38.5 - 38.9 ºC<br>101.2 - 102.0 ºF	39.0 - 40.0 ºC<br>102.1 - 104.0 ºF	> 40.0 ºC<br>> 104.0 ºF
**Headache**	None	No interference with activity	Repeated use of non-narcotic pain reliever for more than 24 hours or some interference with activity	Significant; any use of narcotic pain reliever or prevents daily activity	Results in a visit to emergency room (ER) or hospitalization

Symptoms	Severity				
	**None**	**Grade 1 (Mild)**	**Grade 2 (Moderate)**	**Grade 3 (Severe)**	**Grade 4 (Potentially life-threatening)**
**Fatigue**	None	No interference with activity	Some interference with activity	Significant; prevents daily activity	Results in a visit to emergency room (ER) or hospitalization
**Muscle aches**	None	No interference with activity	Some interference with activity	Significant; prevents daily activity	Results in a visit to emergency room (ER) or hospitalization
**Joint aches, chills, feeling of general discomfort or uneasiness (malaise), swelling in the axilla, swelling in the neck**	None	No interference with activity	Some interference with activity not requiring medical intervention	Prevents daily activity and requires medical intervention	Results in a visit to emergency room (ER) or hospitalization

10.1.2. Unsolicited Adverse Events

All spontaneous unsolicited AEs occurring on vaccination or within 21 days after each vaccination will be reported and classified by system organ class (SOC) and preferred term (PT). Frequency count and the number of unique subjects with an AE will be tabulated by treatment(s) received. For the number of unique subjects reporting, if a subject reported more than one AE that was coded to the same SOC or PT, the subject will be counted only once for that specific SOC or PT.

The number and percentage of subjects with the following unsolicited events will be summarized by vaccination number (1 or 2) and overall by SOC and PT for each of the following for overall and by gender and race separately:

- Immediate adverse events
- Immediate related adverse events and
- All adverse events

10.1.3. Serious Adverse Events (SAE)

An SAE is any untoward medical occurrence (whether or not considered to be related to the study vaccine) that, at any dose:

- Results in death;
- Is life-threatening (at the time of the event);
- Requires inpatient hospitalization ($\geq$ 24 hours) or prolongation of existing hospitalization (elective hospitalizations/procedures for pre-existing conditions that have not worsened are excluded);

- Results in persistent or significant disability/incapacity;
- Is a congenital abnormality/birth defect;
- Is another medically important event.

The number and percentage of subjects for SAE with the following events will be summarized by vaccination number (1 or 2) and overall by SOC and PT for each of the following for overall and by gender and race separately:

- Serious adverse events (up to 21 days following each vaccine administration, from Day 43 to Day 201, and from Day 202 to Day 386)
- Adverse events Leading to Study Withdrawal (up to 21 days following each vaccine administration, from Day 43 to Day 201, and from Day 202 to Day 386)
- Adverse events Leading to Drug Withdrawal (up to 21 days following each vaccine administration, from Day 43 to Day 201, and from Day 202 to Day 386)
- Adverse events Leading to Death (up to 21 days following each vaccine administration, from Day 43 to Day 201, and from Day 202 to Day 386)

10.1.4. Medically Attended Adverse Events (MAAE)

Medically attended adverse events are defined as symptoms or illnesses requiring hospitalization, or emergency room visit, or visit to/by a health care provider.

The number and percentage of subjects with the following events will be summarized by vaccination number (1 or 2) and overall by SOC and PT for each of the following for overall and by gender and race separately:

- Medically Attended adverse events (up to 21 days following each vaccine administration, from Day 43 to Day 201, and from Day 202 to Day 386)

10.1.5. Adverse Events of Special Interest (AESI)

10.1.5.1. Vaccine Associated Enhanced Disease (VAED)

Safety signal of VED after exposure to the CoVLP formulation will be closely monitored and assessed by retrieving data for this AESI as follows: AEs within the system organ class (SOC): immune system disorders and high level group term (HLGT): lower respiratory tract disorders (excluding obstruction and infection), cardiac disorders, signs and symptoms not elsewhere classified, vascular disorders, heart failures, arteriosclerosis, stenosis, vascular insufficiency and necrosis, cardiac arrhythmias, myocardial disorders, and vascular hemorrhagic disorders. High level term (HLT): renal failure and impairment and preferred term (PT): pericarditis, coagulopathy, deep vein thrombosis, pulmonary embolism, cerebrovascular accidents, peripheral ischemia, liver injury, Guillain-Barre syndrome, anosmia, ageusia, encephalitis, chilblains, vasculitis, erythema multiforme that require inpatient hospitalization (≥ 24 hours) and have laboratory confirmed SARS-Cov-2 infection will be monitored for assessment of any potential

case of VAED. As this list can be updated during the study, any further changes to the AESI terms will be updated in the Signal Management Plan (SMP).

The number and percentage of subjects with the following events will be summarized by vaccination number (1 or 2) and overall by SOC and PT for each of the following for overall and by gender and race separately:

- Adverse events of Special Interest (Potential VAED) (up to 21 days following each vaccine administration, from Day 43 to Day 201, and from Day 202 to Day 386)

10.1.5.2. Hypersensitivity Reactions (Anaphylaxis and Severe Allergic Reactions)

All reported events will also be monitored for related hypersensitivity reactions after exposure to the CoVLP formulation. Hypersensitivity will be identified using SMQ broad and narrow terms.

The number and percentage of subjects with the following events will be summarized by vaccination number (1 or 2) and overall by SOC and PT for each of the following for overall and by gender and race separately:

- Adverse events of Special Interest (Related Hypersensitivity Reactions) (up to 21 days following each vaccine administration, from Day 43 to Day 201, and from Day 202 to Day 386)

10.1.5.3. Potential Immune-Mediated Diseases (pIMD)

Potential immune-mediated diseases (pIMDs) are a subset of AEs associated to the adjuvant that include autoimmune diseases and other inflammatory and/or neurologic disorders of interest which may or may not have an autoimmune aetiology. pIMDs will be identified using SMQ terms.

The number and percentage of subjects with the following events will be summarized by vaccination number (1 or 2) and overall by SOC and PT for each of the following for overall and by gender and race separately:

- Adverse events of Special Interest (pMIDs) (up to 21 days following each vaccine administration, from Day 43 to Day 201, and from Day 202 to Day 386)

10.2. Laboratory Evaluations

Blood samples for biochemistry and hematology, HIV, Hepatitis B and Hepatitis C markers in serum and urine samples for urinalysis will be collected according to the Time and Events Schedule (see Table 4) for the Phase 2 portion of the study. Blood samples for testing for SARS-CoV-2 antibodies will be collected according to the Time and Events Schedules (see Table 4 and 5) for the Phase 2 and Phase 3 portions of the study. In addition, serum or urine samples from all females of childbearing potential in Study Populations #1, #2, and #3 for pregnancy testing will

be collected. Any laboratory result outside of the testing laboratory's normal range will be classified as 'clinically significant' (CS) or 'not clinically significant' (NCS) by the site Investigator, with appropriate documentation. Any laboratory test performed at Screening with a value outside the normal range may be repeated (prior to vaccination) at the discretion of the Investigator. Repeated tests will be considered the Baseline results. Baseline for Day 24 assessment will be Day 21, as subject will receive a second vaccination at Day 21.

The tests to be performed by the laboratory are presented in Table 7.

Table 7 Clinical Laboratory Tests for the Phase 2 Portion of the Study

**Biochemistry (serum):**	
Sodium<br>Potassium<br>Urea<br>Creatinine<br>Glucose<br>Bilirubin (total)<br>Albumin<br>Total protein<br>Phosphorus	Alkaline phosphatase<br>Alaninetransferase (ALT)<br>Aspartatetransferase (AST)<br>Gamma glutamyltransferase (GGT)<br>Cholesterol (total, HDL, LDL)<br>Triglyceride<br>Chloride<br>Calcium
**Haematology:**	
Haemoglobin<br>Hematocrit<br>Red blood cells<br>Platelets<br>Mean platelet volume (MPV)<br>White cell count (total, WBC)<br>Neutrophils	Mean cell haemoglobin (MCH)<br>Mean cell concentration (MCHC)<br>Mean cell volume (MCV)<br>Lymphocytes<br>Monocytes<br>Eosinophils<br>Basophils
**Serology:**	
HIV<br>Hepatitis C	Hepatitis B
**Urinalysis:**	
Macroscopic examination (color, aspect)<br>pH<br>Specific gravity	Glucose<br>Protein<br>Blood

Other clinical laboratory tests include a test to measure SARS-CoV-2 antibodies at screening for all subjects in all Study Populations in the Phase 2 portion and at Day 0, Day 386 , and a serum (Phase 2 portion) or urine (Phase 3 portion) pregnancy test at screening and a urine (dipstick or similar) pregnancy test prior to randomization on Day 0 and on Days 21 and 42 for all female subjects of childbearing potential in Study Populations #1, #2, and #3.

Laboratory results will be displayed using the conventional units for all summaries and listings. Clinical laboratory test results (hematology and chemistry) and their changes from baseline will be summarized by visit for the safety analysis set using descriptive statistics.

This document is confidential.

10.3. Vital Signs

Vital signs measurements (resting BP, HR, RR, and OT) will be performed as part of screening procedures, prior to vaccination (on Day 0 and Day 21), and after the post-vaccination 30-minute surveillance period (and repeated if deemed necessary by the Investigator) during the Phase 2 portion. In addition, vital signs measurements will be performed at the clinic site visits on Day 3, Day 24, Day 42, Day 128, Day 201, and Day 386 for the Phase 2 portion of the study. Vital signs measurements will only be performed prior to each vaccination during the Phase 3 portion. Baseline for Day 24 assessment will be Day 21, as subject will receive a second vaccination at Day 21.

A summary table will include the subjects in the safety analysis set with descriptive statistics for baseline, actual value and change from baseline by treatment group and overall.

All vital signs will be listed in by-subject listings and will be sorted by treatment group, subject ID, and date/time assessment.

10.4. Primary Safety Endpoint(s) and Analysis

All safety analyses will be performed using the Safety Analysis set and according to actual vaccination received. Safety data will be summarized by treatment.

For Phase 3, all the safety endpoints will be summarized separately for subjects with data collected prior to unblinding or receipt of vaccine and for subjects with data collected prior to and after unblinding or receipt of vaccine.

In the Phase 2 portion:

10.4.1. Occurrence, intensity, and relationship (relationship to be assessed for unsolicited AEs only) to vaccination of immediate AEs (30 minutes after each vaccination)

Occurrence and intensity of immediate AEs (30 minutes after each vaccination) will be summarized by treatment using descriptive statistics.

10.4.2. Occurrence and intensity of solicited local and systemic AEs (for seven days following each vaccine administration)

Occurrence and intensity of solicited local and systemic AEs reported up to seven days after each vaccination will be summarized by treatment using descriptive statistics.

10.4.3. Occurrence, intensity, and relationship of unsolicited AEs for 21 days following each vaccine administration

Occurrence, intensity, and relationship to vaccination of unsolicited AEs will be summarized by treatment using descriptive statistics.

10.4.4. Number and percentage of subjects with normal and abnormal clinically significant urine, haematological and biochemical values prior to and three days following each vaccination

Laboratory (biochemical, haematological, and urine) data will be summarized by type of laboratory test. Reference ranges and markedly abnormal results will be used in the summary of laboratory data. Descriptive statistics will be calculated for each laboratory test at baseline and at each scheduled timepoint (Day 3, Day 21, and Day 24). Changes from Baseline results will be presented in pre- versus post-treatment cross tabulations (with classes for below, within, and above normal ranges).

10.4.5. Occurrences of SAEs, MAAEs, AEs leading to withdrawal, AESIs (including VAED), and deaths up to 21 days following each vaccine administration

Occurrences of SAEs, AEs leading to subject withdrawal, AESIs, MAAEs, and deaths reported up to 21 days after each vaccination will be summarized by treatment using descriptive statistics.

10.5. Secondary Safety Endpoint(s) and Analysis

For the following categories, AE information will be presented by gender (male and female) and race (Caucasian or White; Black or African American; Asian):

- Immediate solicited and unsolicited AEs up to 30 minutes after each vaccination;
- Solicited local and systemic AEs within first seven days after each vaccination;
- Most frequent unsolicited AEs within 21 days after vaccination;
- AESIs;
- SAEs;
- MAAEs;
- AEs leading to death;
- AEs leading to withdrawal.

In the Phase 2 portion:

10.5.1. Relative occurrence of solicited local and systemic AEs by intensity grades for seven days following each vaccine administration between the healthy adults (Study Population #1) and the healthy elderly population (Study Population #2, each age strata)

The incidence of solicited local and systemic AEs by intensity grades reported for 7 days after each vaccine administration will be compared between Study Population #2 and Study Population #1 using Chi-square tests. Each age strata in Study Population #2 will be compared separately to the Study Population #1.

10.5.2. Relative occurrence of solicited local and systemic AEs by intensity grades for seven days following each vaccine administration between the healthy adults (Study

Population #1) and the healthy elderly adults (Study Population #2) combined and the adults with significant comorbidities (Study Population #3)

The incidence of solicited local and systemic AEs by intensity grades reported for 7 days after each vaccine administration will be compared between Study Population #1 and Study Population #2 combined and Study Population #3 using Chi-square tests.

10.5.3. Occurrences of SAEs, MAAEs, AEs leading to withdrawal, AESIs (including VAED), and deaths from Day 43 to Day 201

Occurrences of SAEs, MAAEs, AEs leading to subject withdrawal, AESIs, and deaths reported from Day 43 to the Day 201 will be summarized by treatment using descriptive statistics.

10.5.4. Occurrences of SAEs, MAAEs, AEs leading to withdrawal, AESIs (including VAED), and deaths from Day 202 to Day 386

Occurrences of SAEs, MAAEs, AEs leading to subject withdrawal, AESIs, and deaths reported from Day 202 to the Day 386 will be summarized by treatment using descriptive statistics.

In the Phase 3 portion:

Solicited AEs collected during the Phase 3 portion of the study will initially be summarized in the solicited safety analysis subset (Section 5.1.1). All solicited AEs will be summarized at end of study as well.

10.5.5. Occurrence, intensity, and relationship (relationship to be assessed for unsolicited AEs only) to vaccination of immediate AEs (30 minutes after each vaccine administration)

The same safety analyses will be performed as described in Section 10.5.1.

10.5.6. Occurrence and intensity of solicited local and systemic AEs (for seven days following each vaccine administration) for the initial set of vaccinations

The same safety analyses will be performed as described in Section 10.5.2.

10.5.7. Occurrence, intensity, and relationship of unsolicited AEs for 21 days following each vaccine administration

The same safety analyses will be performed as described in Section 10.5.3.

10.5.8. Occurrences of SAEs, MAAEs, AEs leading to withdrawal, AESIs (including VAED, anaphylaxis, and severe allergic reactions), and deaths up to 21 days following each vaccine administration

The same safety analyses will be performed as described in Section 10.5.5.

This document is confidential.

10.5.9. Occurrences of SAEs, MAAEs, AEs leading to withdrawal, AESIs (including VED, anaphylaxis, and severe allergic reactions), and deaths from Day 43 to Day 201 The same safety analyses will be performed as described in Section 10.6.3.

10.5.10. Occurrences of SAEs, MAAEs, AEs leading to withdrawal, AESIs (including VAED, anaphylaxis, and severe allergic reactions), and deaths from Day 202 to Day 386

The same safety analyses will be performed as described in Section 10.6.4.

10.6. Exploratory Safety Endpoint(s) and Analysis

In the Phase 2 and Phase 3 portion:

10.6.1. Further characterization of the safety profile of the CoVLP formulation

If deemed necessary, further characterization of the safety profile of the CoVLP formulation will be done and the results will be presented.

10.7. Pregnancy

Pregnancy information will be provided in a listing.

10.8. Physical Examination

A limited physical examination will be performed by the Investigator (or sub-Investigator) as part of screening procedures. History/symptom-directed physical examinations may be performed at any other study visit if 1) new complaints or concerns are raised by either the study subject or study staff and 2) deemed to be necessary by the Investigator.

A summary table will include the subjects in the safety analysis set with number and percentage of each physical examination finding.

The physical examination data will be listed with findings.

11. Day 21 and Day 42 Immunogenicity Data Analysis and Efficacy Analysis

For the Phase 2 portion, the unblinded statistical team will analyze the immunogenicity data after Day 21 and Day 42 in each of the Study Populations. The data analysis will involve selected individuals from the unblinded statistical team and Medicago who will be unblinded. The Day 21 analysis in Study Population #1 and the Day 21 and/or Day 42 analysis in Study Populations #2 and #3 will confirm whether an acceptable immune response has been induced in each Study Population prior to the initiation of the subsequent Phase 3 study, without having to wait until after the end of the 386 days follow-up period for study completion. These immunogenicity data, as well as the Day 28 safety data, will be provided to Regulatory Authority(ies) as required for review prior to enrolling subjects from their jurisdictions in each Study Population into the Phase 3 portion of the study.

If the Day 42 analysis in Study Population #2 and #3 confirms that an acceptable response has been induced (refer to Section 3.5 for details of the criteria that will be used at Day 42), the particular Study Population will be permitted to proceed into the Phase 3 portion of the study. However, if at Day 42, the immune response for Study Population #2 and #3 is still relatively weak compared to the healthy adult Day 42 responses, an ad hoc IDMC meeting will be convened to consider changes to the Protocol to permit alternate vaccination strategies in the particular Study Population.

The analyses performed up to Day 42 for available data for Study Populations #1, #2, and #3 will generate selected tables, listings, and figures (as applicable), by the unblinded statistical team. The results of these analyses will be used to prepare the clinical study report (CSR) and an addendum to the CSR (up to Day 42) for the purpose of reporting to regulatory agencies the safety and immune response profile of the CoVLP formulation up to 42 days after the second vaccination. The Phase 2 results not included in the Phase 2 Day 42 CSR and addendum will be presented in a subsequent addendum to the CSR. In addition, the results included the Phase 2 Day 42 CSR and addendum will be used for publication.

Study Population #1: If the safety and immunogenicity profiles of the chosen CoVLP formulation observed in the Phase 1 study are acceptable in the Phase 2 portion, this group will proceed into the Phase 3 portion. Another key outcome will be the absence of a significant safety signal as assessed by the IDMC.

Study Population #2 and #3: If the safety and immunogenicity profiles of the CoVLP formulation in these populations are acceptable, entry of Study Population #2 or #3 into the Phase 3 portion will be:

- No statistically significant difference in the Nab responses at Day 42 between Study Population #1 and Study Population #2;
- No statistically significant difference in the Nab responses at Day 42 between Study Population #1 and Study Population #2 combined and Study Population #3;

- No significant higher incidence of solicited local and systemic AEs reported 7 days after each vaccine administration in Study Population #2 compared to Study Population #1;
- No significant higher incidence of solicited local and systemic AEs reported 7 days after each vaccine administration in Study Population #3 compared to Study Population #1 and #2 combined.

For Study Population #2, the same criteria will be applied to the comparisons of the two age strata for Study Population #2 versus Study Population #1. In the event of a significant difference between Study Population #1 and one of the age stratum for Study Population #2, the next highest dose level (7.5 µg CoVLP) that can be administered to this population will be assessed in the same number of subjects (as planned for the age stratum in the Phase 2 portion).

For the Phase 3 portion, when at least 160 symptomatic laboratory-confirmed COVID-19 cases (≥ 7 days post-second vaccination) have been collected and the median blinded safety follow-up duration of at least 2 months (post-administration of the second vaccination) for at least 3000 subjects in each of the CoVLP formulation and placebo groups has been achieved, the unblinded statistical team will perform the final analysis and produce final efficacy outputs of the efficacy data in order to test for vaccine efficacy. If the primary efficacy success criterion is met during this analysis, emergency use authorization or approval will be pursued.

The final analysis of the efficacy data to test for vaccine efficacy will produce final efficacy outputs that will be used, in addition to safety outputs, to prepare an addendum to the CSR for the purposes of reporting to regulatory agencies. The Phase 3 results that are not included in this addendum to the CSR will be presented in a subsequent addendum to the CSR.

12. Changes from Analysis Planned in Protocol

The definition of 'severe COVID-19' for the Phase 3 part of the study used for the review of cases by the IDMC is described below.

The 'severe COVID-19' definition in the protocol, section 2.2 Secondary Objectives, Safety:

Severe COVID-19 disease will be defined as virologically-confirmed SARS-CoV-2 infection with the occurrence of a new onset of any of the following:

- Clinical signs at rest indicative of severe systemic illness (respiratory rate ≥ 30 per minute, heart rate ≥ 125 per minute, SpO2 ≤ 93% on room air at sea level or PaO2/FiO2 < 300 mmHg);
- Respiratory failure (defined as needing high-flow oxygen, non-invasive ventilation, mechanical ventilation or ECMO);
- Evidence of shock (SBP < 90 mmHg, DBP < 60 mmHg, or requiring vasopressors);
- Significant acute renal, hepatic, or neurologic dysfunction;
- **Hospitalization;**
- Death.

The definition of 'severe COVID-19' used by the IDMC:

Severe COVID-19 disease will be defined as virologically-confirmed SARS-CoV-2 infection with the occurrence of a new onset of any of the following:

- Clinical signs at rest indicative of severe systemic illness (respiratory rate ≥ 30 per minute, heart rate ≥ 125 per minute, SpO2 ≤ 93% on room air at sea level or PaO2/FiO2 < 300 mmHg);
- Respiratory failure (defined as needing high-flow oxygen, non-invasive ventilation, mechanical ventilation or ECMO);
- Evidence of shock (SBP < 90 mmHg, DBP < 60 mmHg, or requiring vasopressors);
- Significant acute renal, hepatic, or neurologic dysfunction;
- **Intensive Care Unit (ICU) admission;**
- Death.

The criterion modification was made in response to a request by the IDMC.

The criteria defined in the FDA Guidance for Industry 'COVID-19: Developing Drugs and Biological Products for Treatment or Prevention' (2020) (below) are generally followed for the severity assessments of COVID-19 cases by the IDMC.

<u>SARS-CoV-2 infection without symptoms</u>

- Positive testing by standard reverse transcription polymerase chain reaction (RT-PCR) assay or equivalent test
- No symptoms

<u>Mild COVID-19</u>

- Positive testing by standard RT-PCR assay or equivalent test
- Symptoms of mild illness with COVID-19 that could include fever, cough, sore throat, malaise, headache, muscle pain, gastrointestinal symptoms, without shortness of breath or dyspnea
- No clinical signs indicative of Moderate, Severe, or Critical Severity

<u>Moderate COVID-19</u>

- Positive testing by standard RT-PCR assay or equivalent testing
- Symptoms of moderate illness with COVID-19, which could include any symptom of mild illness or shortness of breath with exertion
- Clinical signs suggestive of moderate illness with COVID-19, such as respiratory rate $\geq$ 20 breaths per minute, saturation of oxygen (SpO2) > 93% on room air at sea level, heart rate $\geq$ 90 beats per minute
- No clinical signs indicative of Severe or Critical Illness Severity

<u>Severe COVID-19</u>

- Positive testing by standard RT-PCR assay or an equivalent test
- Symptoms suggestive of severe systemic illness with COVID-19, which could include any symptom of moderate illness or shortness of breath at rest, or respiratory distress
- Clinical signs indicative of severe systemic illness with COVID-19, such as respiratory rate $\geq$ 30 per minute, heart rate $\geq$ 125 per minute, SpO2 $\leq$ 93% on room air at sea level or PaO2/FiO2 < 300
- No criteria for Critical Severity

<u>Critical COVID-19</u>

- Positive testing by standard RT-PCR assay or equivalent test
- Evidence of critical illness, defined by at least one of the following:
    - Respiratory failure defined based on resource utilization requiring at least one of the following:

- Endotracheal intubation and mechanical ventilation, oxygen delivered by high-flow nasal cannula (heated, humidified, oxygen delivered via reinforced nasal cannula at flow rates > 20 L/min with fraction of delivered oxygen ≥ 0.5), noninvasive positive pressure ventilation, ECMO, or clinical diagnosis of respiratory failure (i.e., clinical need for one of the preceding therapies, but preceding therapies not able to be administered in setting of resource limitation)
- Shock (defined by systolic blood pressure < 90 mm Hg, or diastolic blood pressure < 60 mm Hg or requiring vasopressors)
- Multi-organ dysfunction/failure

NOTE: A clinical diagnosis of respiratory failure (in the setting of resource limitation) in which the management deviates from standard of care should be recorded as part of formal data collection.

13. Reference List

Channappanavar R, Zhao J, Perlman S. T cell-mediated immune response to respiratory coronaviruses. Immunol Res. 2014 Aug;59(1-3):118-28. doi: 10.1007/s12026-014-8534-z.

FDA. Guidance for Industry: Development and Licensure of Vaccines to Prevent COVID-19. June 2020. https://www.fda.gov/media/139638/download

FDA. Guidance for Industry: Developing Drugs and Biological Products for Treatment or Prevention. May 2020. https://www.fda.gov/media/137926/download

McElhaney JE, Coler RN, Baldwin SL. Immunologic correlates of protection and potential role for adjuvants to improve influenza vaccines in older adults. Expert Rev Vaccines. 2013a Jul;12(7):759-66.

Protocol: A Randomized, Observer-Blind, Placebo-Controlled, Phase 2/3 Study to Assess the Safety, Efficacy, and Immunogenicity of a Recombinant Coronavirus-Like Particle COVID-19 Vaccine in Adults 18 Years of Age or Older and Children and Adolescents 5-17 Years of Age. CP-PRO-CoVLP-021, Final Version 2.0.

Rothman KJ, Greenland S, Lash TL. Modern Epidemiology. 3rd ed. Philadelphia: Lippincott Williams & Wilkins, 2008. Chapter 14.

Sette A, Crotty S. Pre-existing immunity to SARS-CoV-2: the knowns and unknowns. Nat Rev Immunol. 2020 Aug;20(8):457-458.

Wathuo M, Medley GF, Nokes DJ and Munywoki PK. Quantification and determinants of the amount of respiratory syncytial virus (RSV) shed using real time PCR data from a longitudinal household study [version 2; peer review: 3 approved, 1 approved with reservations]. Wellcome Open Res 2017, 1:27. (https://doi.org/10.12688/wellcomeopenres.10284.2).

This document is confidential.

14. Programming Considerations

14.1. General Considerations

- One SAS program can create several outputs
- Each output will be stored in a separate file
- Output files will be delivered in RTF format or portable document format (PDF)
- Numbering of TFLs will follow ICH E3 guidance

14.2. Table, Figure, and Listing Format

14.2.1. General

- All TFLs will be produced in landscape format on American letter size, unless otherwise specified
- All TFLs will be produced using the Courier New, size 10
- The data displays for TFLs will have a minimum blank 1-inch margin on all 4 sides
- Headers and footers for figures will be in Courier New font, size 8
- Legends will be used for all figures with more than one variable, group, or item displayed
- TFLs will be in black and white (no color), unless otherwise specified
- Specialized text styles, such as bolding, italics, borders, shading, and superscripted and subscripted text, will not be used in the TFLs, unless otherwise specified. On some occasions, superscripts 1, 2, or 3 may be used (see below)
- Only standard keyboard characters will be used in the TFLs. Special characters, such as non-printable control characters, printer-specific, or font-specific characters, will not be used. Hexadecimal-derived characters will be used, where possible, if they are appropriate to help display math symbols (e.g., µ). Certain subscripts and superscripts (e.g., cm2, Cmax) will be employed on a case-by-case basis
- Mixed case will be used for all titles, footnotes, column headers, and programmer-supplied formats, as appropriate.

14.2.2. Headers

- All output will have the following header at the top left of each page:

  Medicago R&D Inc.

CP-PRO-CoVLP-021

Data Extract: DDMMMYYYY

- All output will have Page n of N at the top or bottom right corner of each page. TFLs are internally paginated in relation to the total length (i.e., the page number will appear sequentially as page n of N, where N is the total number of pages in the table)

- The date the output was generated will appear along with the program name as a footer on each page

14.2.3. Display Titles

- Each TFL will be identified by the designation and a numeral. (i.e., Table 14.1.1). A decimal system (x.y and x.y.z) are used to identify TFLs with related contents. The title will be centered. The analysis set will be identified on the line immediately following the title and will be enclosed in parenthesis. The title and table designation will be single spaced. A solid line spanning the margins will separate the display titles from the Column headers. There will be one blank line between the last title and the solid line.

Table x.y.z
First Line of Title
Second Line of Title if Needed
(ITT Analysis Set)

14.2.4. Column Headers

- Column headings will be displayed immediately below the solid line described above in initial upper-case characters

- In the case of efficacy tables, the variable (or characteristic) column is on the far left followed by the treatment group columns and total column (if applicable). P-values may be presented under the total column or in separate p-value column (if applicable). Within-treatment comparisons may have p-values presented in a row beneath the summary statistics for that treatment

- For numeric variables, include ‘unit’ in column or row heading when appropriate

- Analysis set sizes will be presented for each treatment group in the column heading as (N=xx) (or in the row headings, if applicable). This is distinct from the ‘n’ used for the descriptive statistics representing the number of subjects in the analysis set

- The order of treatments in the tables and listings will be 3.75 µg CoVLP + AS03 first, followed by Placebo and then a total column (when applicable)

14.2.5. Body of the Data Display

14.2.5.1. *General Conventions*

Data in columns of a table or listing are formatted as follows:

- Alphanumeric values will be left-justified;
- Whole numbers (e.g., counts) will be right-justified; and
- Numbers containing fractional portions will be decimal aligned

14.2.5.2. *Table Conventions*

- Units will be included where available
- For categorical parameters, all categories will be presented in the table, even if n=0 for all treatment groups in a given category. For example, the frequency distribution for symptom severity would appear as:

Severity Rating	N
potentially life-threatening	2
severe	0
moderate	8
mild	3

Where percentages are presented in these tables, zero percentages will not be presented and so counts of 0 will be presented as 0 and not as 0 (0%).

- An Unknown or Missing category will be added to each parameter for which information is not available for 1 or more subjects
- Unless otherwise specified, the estimated mean and median for a set of values will be printed out to 1 more significant digit than the original values, and standard deviations will be printed out to 2 more significant digits than the original values. The minimum and maximum will report the same significant digits as the original values. For example, systolic blood pressure will be presented as follows:

N	XX
Mean	XXX.X
Std Dev	X.XX
Median	XXX.X
Minimum	XXX
Maximum	XXX

- P-values will be output in the format: '0.xxx', where xxx is the value rounded to 3 decimal places. Every p-value less than 0.001 will be presented as <0.001. If the p-value are less than 0.0001, then present as <0.0001. If the p-value is returned as >0.999, then present as >0.999

- Percentage values will be printed to one decimal place, in parentheses with no spaces, one space after the count (e.g., 7 (12.8%), 13 (5.4%)). Unless otherwise noted, for all percentages, the number of subjects in the analysis set for the treatment group who have an observation will be the denominator. Percentages after zero counts will not be displayed and percentages equating to 100% will be presented as 100%, without decimal places. If percentages are smaller than 0.1%, then it will be specified xx (<0.1)

- Tabular display of data for medical history, prior/concomitant medications, and all tabular displays of adverse event data will be presented by the body system, treatment class, or SOC, sorted by serial code for international SOC sort order, with the highest occurrence in the active treatment group in decreasing order, assuming all terms are coded. Within the body system, drug class and SOC, medical history (by preferred term), drugs (by ATC1 code), and adverse events (by preferred term) will be displayed in decreasing order. If incidence for more than 1 term is identical, they will then be sorted alphabetically.

- The percentage of subjects will normally be calculated as a proportion of the number of subjects assessed in the relevant treatment group (or overall) for the analysis set presented. However, careful consideration is required in many instances due to the complicated nature of selecting the denominator, usually the appropriate number of subjects exposed. Details will be described in footnotes or programming notes, as necessary

- For categorical summaries (number and percentage of subjects) where a subject can be included in more than one category, a footnote or programming note will be added describing whether the subject is included in the summary statistics for all relevant categories or just 1 category as well as the selection criteria

- Where a category with a subheading (such as system organ class) has to be split over more than one page, output the subheading followed by '(cont)' at the top of each subsequent page. The overall summary statistics for the subheading should only be output on the first relevant page

14.2.5.3. *Listing Conventions*

- Listings will be sorted for presentation in order of treatment groups as above, subject number, visit/collection day, and visit/collection time

- Missing data will be represented on subject listings as either a hyphen ('-') with a corresponding footnote ('- = unknown or not evaluated'), or as 'N/A', with the footnote 'N/A = not applicable', whichever is appropriate

- Dates will be printed in SAS DATE9.format ('DD_MMM_YYYY': 01JUL2000). Missing portions of dates will be represented on subject listings as dashes (--JUL2000). Dates that are missing because they are not applicable for the subject will be output as 'N/A', unless otherwise specified

- All observed time values will be presented using a 24-hour clock HH:MM or HH:MM:SS format (e.g., 11:26:45, or 11:26). Time will only be reported if it was measured as part of the study

- Units will be included where available

14.2.5.4. *Figure Conventions*

- Unless otherwise specified, for all figures, study visits will be displayed on the X-axis and endpoint (e.g., treatment mean change from Baseline) values will be displayed on the Y-axis

14.2.6. Footnotes

- A solid line spanning the margins will separate the body of the data display from the footnotes.

- All footnotes will be left justified with single-line spacing immediately below the solid line underneath the data display

- Footnotes will always begin with 'Note:' if an informational footnote, or 1, 2, 3, etc. if a reference footnote. Each new footnote will start on a new line, where possible

- Subject specific footnotes are avoided, where possible

- Footnotes will be used sparingly and add value to the TFL. If more than six lines of footnotes are planned, then a cover page is strongly recommended to be used to display footnotes, and only those essential to comprehension of the data will be repeated on each page

- The last line of the footnote section will be a standard source line that indicates the name of the program used to produce the data display, the date the program was run, and the listing source (i.e., 'Program: myprogram.sas Listing source: 16.x.y.z')

- Sources and/or cross-references in footnotes will use the keyword prefix (in singular form) for each reference and will be separated by a comma when multiple cross-references are displayed
  <u>Example</u>
  Listing source: Listing 16.2.4.1.1, Listing 16.2.4.1.2, Listing 16.2.4.2.1

15. Quality Control

SAS programs are developed to produce output such as analysis data sets, summary tables, data listings, figures or statistical analyses. An overview of the development of programs is detailed in Syneos Health Developing Statistical Programs [Redacted].

Syneos Health Developing Statistical Programs [Redacted], Conducting the Transfer of Biostatistical Deliverables [Redacted] and the SAS Programming and Validation Plan describes the quality control procedures that are performed for all SAS programs and output. Quality control is defined here as the operational techniques and activities undertaken to verify that the SAS programs produce the output by checking for their logic, efficiency and commenting and by review of the produced output.

16. Appendices

Appendix 1 – List of Potential Immune-Mediated Diseases

**Neuroinflammatory disorders**	**Musculoskeletal disorders**	**Skin disorders**
• Cranial nerve neuropathy, including paralysis and paresis (eg, Bell's palsy).<br>• Optic neuritis.<br>• Multiple sclerosis.<br>• Transverse myelitis.<br>• Guillain-Barré syndrome, including Miller Fisher syndrome and other variants.<br>• Acute disseminated encephalomyelitis, including site specific variants, eg, noninfectious encephalitis, encephalomyelitis, myelitis, myeloradiculoneuritis.<br>• Myasthenia gravis, including Lambert-Eaton myasthenic syndrome.<br>• Demyelinating peripheral neuropathies and plexopathies including:<br>- Chronic inflammatory demyelinating polyneuropathy.<br>- Multifocal motor neuropathy.<br>- Polyneuropathies associated with monoclonal gammopathy.<br>• Narcolepsy.<br>• Tolosa Hunt syndrome.	• Systemic lupus erythematosus and associated conditions.<br>• Systemic scleroderma (systemic sclerosis), including:<br>- Diffuse scleroderma.<br>- CREST syndrome.<br>• Idiopathic inflammatory myopathies, including:<br>- Dermatomyositis.<br>- Polymyositis.<br>• Anti-synthetase syndrome.<br>• Rheumatoid arthritis and associated conditions including:<br>- Juvenile idiopathic arthritis.<br>- Still's disease.<br>• Polymyalgia rheumatica.<br>• Spondyloarthropathies, including:<br>- Ankylosing spondylitis.<br>- Reactive arthritis (Reiter's syndrome).<br>- Undifferentiated spondyloarthritis.<br>- Psoriatic arthritis.<br>- Enteropathic arthritis.<br>• Relapsing polychondritis.<br>• Mixed connective tissue disorder.<br>• Gout.	• Psoriasis.<br>• Vitiligo.<br>• Erythema nodosum.<br>• Autoimmune bullous skin diseases (including pemphigus, pemphigoid, and dermatitis herpetiformis).<br>• Lichen planus.<br>• Sweet's syndrome.<br>• Localized scleroderma (morphea).<br>• Cutaneous lupus erythematosus.<br>• Rosacea.
**Vasculitis**	**Blood disorders**	**Others**
• Large vessels vasculitis including:<br>- Giant cell arteritis (temporal arteritis).<br>- Takayasu's arteritis.<br>• Medium sized and/or small vessels vasculitis including:<br>- Polyarteritis nodosa.	• Autoimmune hemolytic anemia.<br>• Autoimmune thrombocytopenia.<br>• Antiphospholipid syndrome.<br>• Pernicious anemia.<br>• Autoimmune aplastic anemia.<br>• Autoimmune neutropenia.	• Autoimmune glomerulonephritis including:<br>- IgA nephropathy.<br>- Glomerulonephritis rapidly progressive.<br>• Membranous glomerulonephritis.

This document is confidential.

- Kawasaki's disease.<br>- Microscopic polyangiitis.<br>- Wegener's granulomatosis (granulomatosis with polyangiitis).	• Autoimmune pancytopenia.	• Membranoproliferative glomerulonephritis.<br>• Mesangioproliferative glomerulonephritis.<br>• Tubulointerstitial nephritis and uveitis syndrome.
**Vasculitis (continued)**	**Blood disorders (continued)**	**Others (continued)**
• Churg–Strauss syndrome (allergic granulomatous angiitis or eosinophilic granulomatosis with polyangiitis).<br>• Buerger's disease (thromboangiitis obliterans).<br>• Necrotizing vasculitis (cutaneous or systemic).<br>• Antineutrophil cytoplasmic antibody (ANCA) positive vasculitis (type unspecified).<br>• Henoch-Schonlein purpura (IgA vasculitis).<br>• Behcet's syndrome.<br>• Leukocytoclastic vasculitis.		• Ocular autoimmune diseases including:<br>- Autoimmune uveitis.<br>- Autoimmune retinitis.<br>• Autoimmune myocarditis/cardiomyopathy.<br>• Sarcoidosis.<br>• Stevens-Johnson syndrome.<br>• Sjögren's syndrome.<br>• Alopecia areata.<br>• Idiopathic pulmonary fibrosis.<br>• Goodpasture syndrome.<br>• Raynaud's phenomenon.
**Liver disorders**	**Gastrointestinal disorders**	**Endocrine disorders**
• Autoimmune hepatitis.<br>• Primary biliary cirrhosis.<br>• Primary sclerosing cholangitis.<br>• Autoimmune cholangitis.	• Inflammatory bowel disease, including:<br>- Crohn's disease.<br>- Ulcerative colitis.<br>- Microscopic colitis.<br>- Ulcerative proctitis.<br>• Celiac disease.<br>• Autoimmune pancreatitis.	• Autoimmune thyroiditis (including Hashimoto thyroiditis).<br>• Grave's or Basedow's disease.<br>• Diabetes mellitus type 1.<br>• Addison's disease.<br>• Polyglandular autoimmune syndrome.<br>• Autoimmune hypophysitis.
