# Supplementary material for: Safety and immunogenicity of an AS03-adjuvanted plant-based SARS-CoV-2 vaccine in Adults with and without Comorbidities
Source: NPJ Vaccines. 2022 Nov 9;7:142. doi: 10.1038/s41541-022-00561-2 (PMC9646261; doi:10.1038/s41541-022-00561-2)
Supplement: Supplementary file 2 — REPORTING SUMMARY [file 41541_2022_561_MOESM2_ESM.pdf]

Corresponding author(s): Brian J Ward
Last updated by author(s): Aug 8, 2022

# Reporting Summary

Nature Portfolio wishes to improve the reproducibility of the work that we publish. This form provides structure for consistency and transparency in reporting. For further information on Nature Portfolio policies, see our Editorial Policies and the Editorial Policy Checklist.

## Statistics

For all statistical analyses, confirm that the following items are present in the figure legend, table legend, main text, or Methods section.

| n/a | Confirmed | |
|---|---|---|
| ☐ | ☒ | The exact sample size (*n*) for each experimental group/condition, given as a discrete number and unit of measurement |
| ☐ | ☒ | A statement on whether measurements were taken from distinct samples or whether the same sample was measured repeatedly |
| ☐ | ☒ | The statistical test(s) used AND whether they are one- or two-sided<br>*Only common tests should be described solely by name; describe more complex techniques in the Methods section.* |
| ☐ | ☒ | A description of all covariates tested |
| ☐ | ☒ | A description of any assumptions or corrections, such as tests of normality and adjustment for multiple comparisons |
| ☐ | ☒ | A full description of the statistical parameters including central tendency (e.g. means) or other basic estimates (e.g. regression coefficient) AND variation (e.g. standard deviation) or associated estimates of uncertainty (e.g. confidence intervals) |
| ☐ | ☒ | For null hypothesis testing, the test statistic (e.g. *F*, *t*, *r*) with confidence intervals, effect sizes, degrees of freedom and *P* value noted<br>*Give P values as exact values whenever suitable.* |
| ☒ | ☐ | For Bayesian analysis, information on the choice of priors and Markov chain Monte Carlo settings |
| ☒ | ☐ | For hierarchical and complex designs, identification of the appropriate level for tests and full reporting of outcomes |
| ☒ | ☐ | Estimates of effect sizes (e.g. Cohen's *d*, Pearson's *r*), indicating how they were calculated |

*Our web collection on statistics for biologists contains articles on many of the points above.*

## Software and code

Policy information about availability of computer code

| | |
|---|---|
| Data collection | N/A |
| Data analysis | N/A |

For manuscripts utilizing custom algorithms or software that are central to the research but not yet described in published literature, software must be made available to editors and reviewers. We strongly encourage code deposition in a community repository (e.g. GitHub). See the Nature Portfolio guidelines for submitting code & software for further information.

## Data

Policy information about availability of data

All manuscripts must include a data availability statement. This statement should provide the following information, where applicable:

- Accession codes, unique identifiers, or web links for publicly available datasets
- A description of any restrictions on data availability
- For clinical datasets or third party data, please ensure that the statement adheres to our policy

Medicago Inc. is committed to providing access to anonymized data collected during the trial that underlie the results reported in this article, at the end of the clinical trial, which is currently scheduled to be 1 year after the last participant is enrolled, unless granted an extension. Medicago Inc. will collaborate with its partners (GSK, Wavre, Belgium) on such requests before disclosure. Proposals should be directed to wardb@medicago.com or daoustma@medicago.com. To gain

access, data requestors will need to sign a data access agreement and access will be granted for non-commercial research purposes only. The following publicly-available databases were accessed to complete this work: GISAID database (https://www.gisaid.org/) and Genbank (https://www.ncbi.nlm.nih.gov/genbank/).

## Human research participants

Policy information about studies involving human research participants and Sex and Gender in Research.

| | |
|---|---|
| Reporting on sex and gender | See Protocol attached as Supplementary Material |
| Population characteristics | See Protocol attached as Supplementary Material |
| Recruitment | See Protocol attached as Supplementary Material |
| Ethics oversight | The study was approved by a central Institutional Review Board/ Research Ethics Review Board as well as the Health Products and Food Branch of Health Canada and was carried out in accordance with the Declaration of Helsinki and the principles of Good Clinical Practices. Written informed consent was obtained from all study participants prior to any study procedure. Subjects were offered modest compensation for their participation in this study (ie: time off work, displacement costs). |

Note that full information on the approval of the study protocol must also be provided in the manuscript.

# Field-specific reporting

Please select the one below that is the best fit for your research. If you are not sure, read the appropriate sections before making your selection.

☒ Life sciences ☐ Behavioural & social sciences ☐ Ecological, evolutionary & environmental sciences

For a reference copy of the document with all sections, see nature.com/documents/nr-reporting-summary-flat.pdf

# Life sciences study design

All studies must disclose on these points even when the disclosure is negative.

| | |
|---|---|
| Sample size | The number of participants selected provided insight into immunogenicity with appropriate power to confirm phase 1 dosing. |
| Data exclusions | Data was only excluded from the day 42 immunogenicity analysis if the subject failed to recieve their second (day 21) vaccine dose. |
| Replication | All immunogenicity analysis was performed twice and the mean of the repeats reported for each data point. |
| Randomization | Randomization was performed 5:1 vaccine to placebo. Randomization was used to minimize bias in the assignment of subjects to treatment groups, within each Study Population, to increase the likelihood that known and unknown subject attributes (e.g. demographic and baseline characteristics) are evenly balanced across treatment groups and to enhance the validity of statistical comparisons across treatment groups within each Study Population. |
| Blinding | Investigators and all personnel involved in the clinical conduct of the study (except the staff involved in the preparation and administration of study vaccine, quality assurance auditor and quality control reviews) remained blinded during the course of the study. Selected individuals involved in the IDMC review and/or interim analysis were unblinded to allow safety review and recommendations as well as day 21 and 42 immunogenicity analysis. |

# Reporting for specific materials, systems and methods

We require information from authors about some types of materials, experimental systems and methods used in many studies. Here, indicate whether each material, system or method listed is relevant to your study. If you are not sure if a list item applies to your research, read the appropriate section before selecting a response.

### Materials & experimental systems

| n/a | Involved in the study | |
|---|---|---|
| ☐ | ☒ | Antibodies |
| ☐ | ☒ | Eukaryotic cell lines |
| ☒ | ☐ | Palaeontology and archaeology |
| ☒ | ☐ | Animals and other organisms |
| ☐ | ☒ | Clinical data |
| ☒ | ☐ | Dual use research of concern |

### Methods

| n/a | Involved in the study | |
|---|---|---|
| ☒ | ☐ | ChIP-seq |
| ☒ | ☐ | Flow cytometry |
| ☒ | ☐ | MRI-based neuroimaging |

## Antibodies

| | |
|---|---|
| Antibodies used | Cellular Technology Limited (CTL) antibodies used in the ELISpot asays included: human IFNg cat # hIFNgp-2M/10, lot #I0708JMM, and IL-4 cat#hIL4p-2M/10, Lot#40721JEW |
| Validation | CTL antibodies are validated specifically for use in the kits they are provided with. |

## Eukaryotic cell lines

Policy information about cell lines and Sex and Gender in Research

| | |
|---|---|
| Cell line source(s) | Nexelis uses ACE-2 expressing VERO cells in their pseudovirion assay. |
| Authentication | The assays are authenticated.  this includes consistent expression of ACE-2 |
| Mycoplasma contamination | certified mycoplasma free |
| Commonly misidentified lines (See ICLAC register) | na- cells express ACE-2 and expression is validated so they are unlikely to be mixed with parental VERO cells. |

## Clinical data

Policy information about clinical studies

All manuscripts should comply with the ICMJE guidelines for publication of clinical research and a completed CONSORT checklist must be included with all submissions.

| | |
|---|---|
| Clinical trial registration | NCT04636697 |
| Study protocol | Attached as Supplementary Material |
| Data collection | See Protocol attached as Supplementary Material |
| Outcomes | See Protocol attached as Supplementary Material |
